# Virus-specific editing identification approach reveals the landscape of A-to-I editing and its impacts on SARS-CoV-2 characteristics and evolution

**DOI:** 10.1093/nar/gkac120

**Published:** 2022-03-02

**Authors:** Yulong Song, Xiuju He, Wenbing Yang, Yaoxing Wu, Jun Cui, Tian Tang, Rui Zhang

**Affiliations:** MOE Key Laboratory of Gene Function and Regulation, Guangdong Province Key Laboratory of Pharmaceutical Functional Genes, State Key Laboratory of Biocontrol, School of Life Sciences, Sun Yat-Sen University, Guangzhou510275, PR China; MOE Key Laboratory of Gene Function and Regulation, Guangdong Province Key Laboratory of Pharmaceutical Functional Genes, State Key Laboratory of Biocontrol, School of Life Sciences, Sun Yat-Sen University, Guangzhou510275, PR China; MOE Key Laboratory of Gene Function and Regulation, Guangdong Province Key Laboratory of Pharmaceutical Functional Genes, State Key Laboratory of Biocontrol, School of Life Sciences, Sun Yat-Sen University, Guangzhou510275, PR China; MOE Key Laboratory of Gene Function and Regulation, Guangdong Province Key Laboratory of Pharmaceutical Functional Genes, State Key Laboratory of Biocontrol, School of Life Sciences, Sun Yat-Sen University, Guangzhou510275, PR China; MOE Key Laboratory of Gene Function and Regulation, Guangdong Province Key Laboratory of Pharmaceutical Functional Genes, State Key Laboratory of Biocontrol, School of Life Sciences, Sun Yat-Sen University, Guangzhou510275, PR China; MOE Key Laboratory of Gene Function and Regulation, Guangdong Province Key Laboratory of Pharmaceutical Functional Genes, State Key Laboratory of Biocontrol, School of Life Sciences, Sun Yat-Sen University, Guangzhou510275, PR China; MOE Key Laboratory of Gene Function and Regulation, Guangdong Province Key Laboratory of Pharmaceutical Functional Genes, State Key Laboratory of Biocontrol, School of Life Sciences, Sun Yat-Sen University, Guangzhou510275, PR China

## Abstract

Upon SARS-CoV-2 infection, viral intermediates specifically activate the IFN response through MDA5-mediated sensing and accordingly induce ADAR1 p150 expression, which might lead to viral A-to-I RNA editing. Here, we developed an RNA virus-specific editing identification pipeline, surveyed 7622 RNA-seq data from diverse types of samples infected with SARS-CoV-2, and constructed an atlas of A-to-I RNA editing sites in SARS-CoV-2. We found that A-to-I editing was dynamically regulated, varied between tissue and cell types, and was correlated with the intensity of innate immune response. On average, 91 editing events were deposited at viral dsRNA intermediates per sample. Moreover, editing hotspots were observed, including recoding sites in the spike gene that affect viral infectivity and antigenicity. Finally, we provided evidence that RNA editing accelerated SARS-CoV-2 evolution in humans during the epidemic. Our study highlights the ability of SARS-CoV-2 to hijack components of the host antiviral machinery to edit its genome and fuel its evolution, and also provides a framework and resource for studying viral RNA editing.

## INTRODUCTION

SARS-CoV-2, a positive-sense single-stranded RNA ((+)ssRNA) virus, emerged in late 2019 and expanded globally, resulting in over 82 million confirmed cases by the end of 2020 (1,2). Given the continuing spread of SARS-CoV-2 and the rise of new variants of concern (3–6), it is of vital importance to understand the sources of mutations and the principles of their accumulation in SARS-CoV-2. SARS-CoV-2 has complex processes of replication and transcription facilitated by the replication-transcription complex with RNA-dependent RNA polymerase (RdRp) activity (7). Negative-strand RNAs are synthesized by RdRp starting from the 3′ end of positive genomic RNAs ((+)gRNAs), from which continuous synthesis generates full-length (-)gRNAs, whereas discontinuous jumping produces negative subgenomic RNAs ((−)sgRNAs). (+)gRNA and (+)sgRNA progenies are then synthesized using these negative-strand RNA intermediates as templates. During these processes, viral substitutions are produced from two types of sources. The first type is substitutions introduced by replication error. SARS-CoV-2 acquires such substitutions slowly as the result of a proofreading RdRp (8), and the rate is estimated to be ∼3 × 10^–6^ per infection cycle (9). The second type is substitutions introduced by host immune response factors, such as cytidine to uridine (C-to-U) and adenosine to inosine (A-to-I) RNA editing mediated by APOBEC and ADAR deaminases that target ssRNA and dsRNA substrates, respectively.

In this study, we focused on ADAR-mediated A-to-I RNA editing (10,11) and aimed to investigate its occurrence and impacts on SARS-CoV-2 characteristics and evolution. ADARs are a family of dsRNA binding enzymes present in animals that deaminate A-to-I in dsRNA. There are three mammalian ADAR proteins: ADAR1, -2 and -3, and only ADAR1 and ADAR2 demonstrate editing activity. ADAR1 is widely expressed and is present as a predominantly nuclear, constitutive ADAR1 p110 isoform expressed in all tissues. It also has an additional IFN-inducible ADAR1 p150 isoform that is found in both the nucleus and the cytoplasm (12). ADAR1 p150 isoform has a key role in suppressing IFN signaling and plays an important role during viral infections (13–15). ADAR2 is most highly expressed in the brain and is localized exclusively in the nucleus (16). Recent studies found that MDA5 is the major sensor involved in sensing SARS-CoV-2 infection, and viral intermediates specifically activate the IFN response through MDA5-mediated sensing (17–19). The IFN response may further induce cytoplasmic expression of ADAR1 p150 isoform (12), which leads to A-to-I RNA editing of SARS-CoV-2.

To identify A-to-I RNA editing sites genome-wide, numerous approaches were developed recently built upon bioinformatic analyses and high-throughput sequencing methods. Editing sites show up when a sequence is mapped to the genome: while the unedited sequence reads A, sequencing identifies the inosine in the edited site as guanosine (G), thus A-to-G variants are indicative of A-to-I editing sites. Although signatures of ADAR activity have been previously inferred in the consensus genome sequences of various RNA viruses (20), including SARS-CoV-2 (21), such inferences were indirect and unconvincing. To date, viral A-to-I editing has not been directly detected within the infected individuals by high-throughput sequencing, due to technical difficulty. A-to-I RNA editing of RNA viruses may regulate viral biology in two ways. First, viral editing within the infected individual may affect viral characteristics and virus-host interaction. In this case, we would expect that editing events need to achieve a certain level. For example, at least a 5% level, for proper function. Second, viral editing as a possible source of mutations can fuel viral evolution during transmission. In this case, it is similar to the process in which newly emerged variants introduced by replication error are fixed at the point of transmission and fuel the evolution of RNA viruses (22,23). Even the sites with extremely low-level editing might contribute to viral evolution during viral spread.

## MATERIALS AND METHODS

### The SARS-CoV-2 reference genome sequence

We used the complete genome sequence SARS-CoV-2 Wuhan-Hu-1 strain (Accession NC_045512, Version NC_045512.2) as the reference genome.

### RNA-seq data collection

RNA-seq data were downloaded from NIH SARS-CoV-2 resource website (https://www.ncbi.nlm.nih.gov/sars-cov-2/). A list of sample IDs is shown in Supplementary Table S1. We only collected data generated using Illumina, Ion Torrent, and BGI platform.

### Mapping of RNA-seq reads

We first trimmed adapters and low-quality bases using cutadapt (24). Next, reads were trimmed to 60–80 nt, and reads with a length <60 nt were discarded. The cleaned reads were mapped to the reference sequence using BWA (25) aln (bwa aln -t 20) and mem (bwa mem -M -t 20 -k 50). The unmapped reads were extracted for editing site calling.

### Calling of putative RNA editing sites

To realign reads with a cluster of mismatches caused by A-to-G editing, we transformed every A to G in both the unmapped reads and the viral reference genome. We aligned the transformed reads to the transformed genome, again using BWA aln (bwa aln -t 5 -n 2 -o 0). The original (four-letter) sequences of the reads that aligned (after the transformation) were recovered, and RNA variants between the reads and the reference genome were examined. For multiple mapped reads, only the best hits were retained. Next, we grouped RNA variants into three categories: (1) A-to-G/T-to-C sites with quality ≥ 30 as edited sites (labeled as‘*’), (2) A-to-G/T-to-C sites with quality <30 as low-quality sites (labeled as ‘−’), (3) non-A-to-G/T-to-C sites as mismatch sites (labeled as ‘x’). To identify edited reads, we specifically required that the reads had a minimum number of edited sites ≥4 (one-step read, with either A-to-G or T-to-C sites in a read) or 5 (two-step read, with both A-to-G and T-to-C sites in a read). We also excluded putative misaligned reads matching any of the criteria below: a, ≥3 */x/- sites at the end (5 nt); b, read end in a homopolymer runs of ≥3 nt and with ≥ 2 */x/- sites; c, ≥ 3 continuously edited sites; d, **/x/-/ sites within the flanking 2-nt sequences of an edited site; e, ≥3 edited sites in dinucleotide repeat sequences (≥3); f, * site in homopolymer runs of ≥5 bp. Last, we required that the reads had no more than 2, 1 or 0 mismatch and low-quality sites. To achieve high accuracy in calling A-to-G editing sites without a substantial reduction in sensitivity, we chose a cutoff (from 2 to 0) that satisfies the edited sample requirement (edited read count ≥ 5, A-to-G/T-to-C percentage ≥ 80% and edited site number ≥ 10). For individual editing site calling, we removed sites with editing level ≥0.95, which were unlikely introduced by an RNA editing mechanism.

To determine the specificity of our pipeline, we repeated it when searching for other types of variant sites (e.g. A-to-C, G-to-A, and so on), which involved modifying the transformation and the definition of the editing mismatches accordingly, but was otherwise identical to the A-to-G screen. There are 12 possible single-nucleotide mismatches. However, since the RNA-seq reads could be either sense or antisense, we could not distinguish between a given mismatch and its complementary one. We therefore reported results for six categories of variant types.

### RNA variant calling via the conventional editing identification pipeline

We used a widely used pipeline for conventional RNA variant calling (26). In brief, we mapped cleaned reads to the reference genome via BWA as above. Next, we removed identical reads (PCR duplicates). Then, we inspected all positions that showed variation in the RNA using samtools (Version: 1.2) mpileup. We only took variant positions in the RNA into consideration if they conformed to our requirements for the number and quality of bases that vary from the reference genome. We specifically required that each variant was supported by three or more variant bases having a base quality score ≥30. To avoid false positives at the 5′ read ends due to random-hexamer priming, we truncated the first 6 bases of each read. We also removed RNA editing candidates if they were located in regions of high similarity to other parts of the genome.

### Editing level quantification

We first merged the bam file containing clustered read mapping results with the bam file containing the mapped reads. Next, we inspected all editing positions using samtools for editing level quantification. We required that the minimal base quality was 30, and only sites covered by at least 30 reads were retained.

### Editing index analysis

617 nasopharyngeal swab samples from the New York City metropolitan area during the COVID-19 outbreak in spring 2020 (27) were used for analysis. For each sample, the editing index was defined as the number of edited viral reads divided by the total number of viral reads. Only reads with the number of mismatch and low-quality A-to-G/T-to-C sites ≤2 were considered as edited reads.

### Gene expression level quantification

TPM (transcripts per million) was used to represent ADAR1 and MDA5 (*IFIH1* gene) expression. Reads were mapped to the human reference genome GRCh38 using Hisat2 (Version 2.0.4) (28) with default parameters. Then, StringTie (Version v1.3.0) (29) was used to calculate the TPM values with default parameters.

### Spike protein recoding site analysis

The effects of recoding sites in spike protein on RBD expression and ACE2 binding were measured based on deep mutational scanning data (30). The effects of recoding sites in spike protein on viral infectivity and antigenicity were measured based on the high-throughput pseudovirus assay (31).

To examine the overlaps between recoding editing sites in spike protein and variants of concern, we downloaded variant watch lists using the GISAID ‘Emerging Variants’ portal.

### Cloning and transfection of SARS-CoV-2 spike gene variants

We selected seven recoding editing hotspots located in the NTD and generated mutants with individual editing sites. As negative and positive controls, we generated wild type spike gene and a deletion mutant that is known to affect the binding of a neutralizing antibody targeting the NTD (32). Vero E6 cells were plated on polylysine-treated coverslips in six-well plates. After 24 h, cells were transiently transfected with 2 μg plasmids individually using Lipofectamine 3000 (Thermo Fisher Scientific, cat. no. L3000015) following the manufacturer's protocol.

### Indirect immunofluorescence assay

Indirect immunofluorescence was performed as previously reported (32). In brief, 36 h after transfection, coverslips were washed three times with PBS, fixed with 4% (w/v) paraformaldehyde in PBS for 10 min at room temperature, rinsed three times with PBS, and permeabilized with 0.1% (v/v) Triton-X100 (Sigma) in PBS for 15 min at room temperature. Next, 1 ml 1% BSA/PBS was added to the coverslips for 30 min at room temperature to block unspecific binding of antibodies. Primary antibodies (rabbit anti-SARS-CoV-2 spike protein monoclonal antibody, 40150-R007, Sino Biological, 1/100 dilution; human anti-SARS-CoV-2 4A8 monoclonal antibody, CPC514A, Cell Sciences, 1/100 dilution in PBS with 1% BSA) were added and incubated at 4°C overnight. Coverslips were washed with PBS three times, and secondary antibodies (Goat anti-Rabbit Alexa Fluor Plus 555 Invitrogen, and Goat anti-Human Alexa Fluor 488, Invitrogen, 1/400 dilution in PBS with 1% BSA) were added and incubated at room temperature for 1 h. Coverslips were washed with PBS three times and counterstained with DAPI (Invitrogen). Immunofluorescence images were acquired with a Leica SP8 X Confocal Microscope (Lecia) using LAS X software.

### RNA structure analysis

The in vivo structure of SARS-CoV-2 was obtained from a previous study (33). For each nucleotide of the genome, an icSHAPE reactivity score between 0 and 1 for each nucleotide was obtained, with a higher score indicating that a nucleotide is more likely single-stranded.

### Protein structure illustration

The spike protein sequence diagram was created by IBS 1.0.3 (34). Spike protein crystallization data were from Zhou *et al.* (35) (PDB: 7KNE). Structure visualization and variant annotation were performed with PyMOL (http://www.pymol.org/).

### Motif discovery

For each RNA variant, the flanking sequence of each site was extracted from the reference genome. Motif logos were plotted with WebLogo v.3.7 (36).

### GISAID sequence analysis

Sequences were obtained from the GISAID database (37). Our dataset was composed of 827074 SARS-CoV-2 sequences collected and deposited between 1 December 2019 and 22 March 2021. All GISAID sequences were fist aligned to NC_045512.2 using blast (blastn -strand plus -num_threads 20 -line_length 300000 -perc_identity 80). Since RNA editing sites present in the (+)gRNAs were most likely fixed during transmission, we only used A-to-G editing sites identified in human tissue samples for analysis. For analysis in Figure 5A, in all editing positions, we scanned A-to-G or A-to-C mutation clusters (from two sites to six sites) using a 60-, 80- or 100-nt sliding window. Next, the enrichment was defined as the number of clusters with A-to-G mutations divided by that with A-to-C mutations. For analysis in Figure 5B, we randomly selected the same number of uneditable A positions with GAN or CAN motif that were unfavored by ADAR and scanned A-to-G mutation clusters as we did for editable A positions. We repeated the random sampling processes 1000 times and calculated the mean number of clusters with A-to-G mutations. Finally, the enrichment was defined as the number of clusters with A-to-G mutations in editable A positions divided by that in uneditable A positions. For analysis in Figure 5C, the frequencies of A-to-G mutations were calculated based on the aligned data. Editing sites and flanking 200 bp regions were used for analysis. To exclude the variants that may be under positive selection, only sites with frequency ≤ 0.0002 were used for analysis. For analysis in Figure 5D and E, to have a fair comparison of tree lengths between editable and uneditable As, we first randomly extracted the same number of uneditable As as editable As. Next, we randomly extracted 200 000 GISAID sequences. For each sequence, the editable and uneditable positions were joined from 5′ to 3′, and the non-A/G mutations were masked as As. ML trees were then constructed using FastTree (38) (Version 2.1.11) with the default parameters. The analysis was repeated 1000 times and the tree lengths were compared.

## RESULTS

### The computational pipeline for A-to-I RNA editing discovery in RNA viruses

Many tools have been recently developed to identify A-to-I RNA editing sites from RNA-seq data (summarized in (39,40)), but detection typically requires matched genomic sequences from the same sample to discriminate RNA editing events from other types of variants. As RNA viruses do not possess an unedited DNA form, conventional pipelines, in principle, will not work for viral editing identification. Indeed, an initial attempt (41) to identify A-to-I editing sites in SARS-CoV-2 using a conventional pipeline obtained putative variants with motifs inconsistent with the common view of ADAR-mediated RNA editing, suggesting that it identifies a large number of false-positive sites.

**Figure 1.**
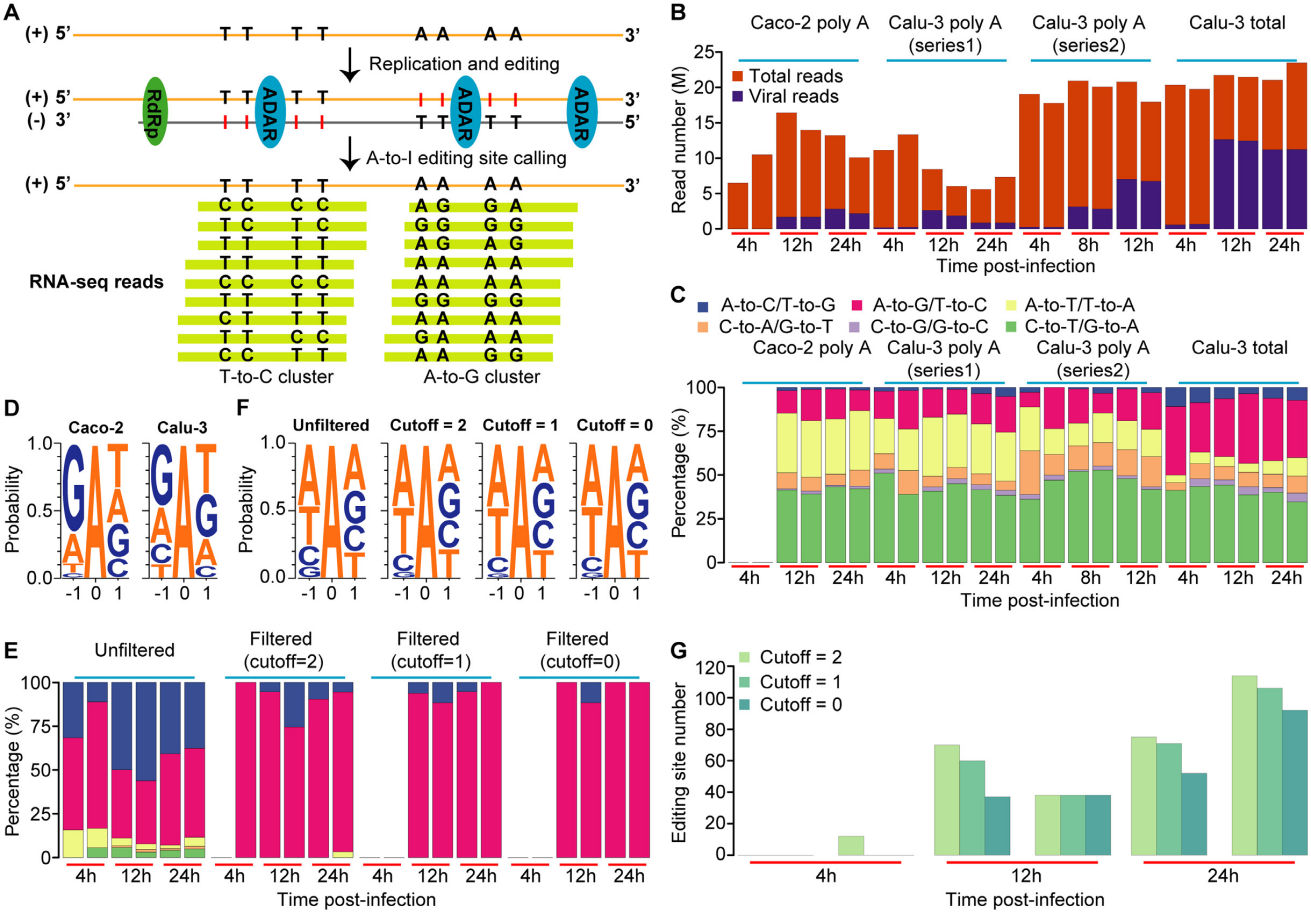
Development and verification of our computational pipeline. (**A**) Schematic diagram of variants that occur in each RNA and are indicative of A-to-I editing. The presence of A-to-I editing sites in the different viral species (+ strand genome, - strand RdRp products) will appear as A-to-G and T-to-C mismatches during variant calling. (**B**) The viral load in each sample. In this dataset, Caco-2 and Calu-3 cells were infected with SARS-CoV-2, and poly A RNA-seq and/or total RNA-seq were performed for samples collected from different time points after infection. For each infection time point, two replicates were performed. Data were from PRJNA625518. (**C**) Percentage of all 12 mismatch types called using the conventional pipeline. No RNA variants were identified in Caco-2 sample collected 4h after SARS-CoV-2 infection because of the low viral load. Different mismatch types are indicated by different colors. (**D**) The nucleotides neighboring the detected A-to-G/T-to-C RNA variant sites called using the conventional pipeline. The reverse complements of the triplets of T-to-C variants were combined with the triplets of A-to-G sites for analysis. For the two types of cells in panel C, sites identified at different time points were combined for analysis. (**E**) Percentage of all 12 mismatch types called using our RNA virus-specific pipeline. Total RNA samples from Calu-3 cells were used for analysis because they had the highest viral load. The editing sites called before and after the edited read filtering step were analyzed, respectively. The color codes for different mismatch types are the same as in C. (**F**) The nucleotides neighboring the detected A-to-G/T-to-C RNA variant sites called using the RNA virus-specific pipeline before and after the edited read filtering step. The reverse complements of the triplets of T-to-C variants were combined with the triplets of A-to-G sites for analysis. For each mismatch and low-quality site cutoff, sites identified at different time points were combined for analysis. (**G**) The number of editing sites identified with different infection times. Sites identified with three mismatch and low-quality site cutoffs were plotted separately.

One hallmark of ADAR-mediated editing is that editing sites are clustered together, and such a feature has been successfully used to develop a pipeline to call hyper-editing sites (42). Moreover, in (+)ssRNA viruses, the presence of A-to-I editing sites in the different viral species (i.e. + strand genomic RNA, - strand RdRp products) will appear as distinct nucleotide changes (Figure 1A), which are informative for A-to-I editing identification. Based on these features, we developed a computational pipeline that is specific for editing identification of RNA viruses (Supplementary Figure S1, see details in Materials and methods). The characteristic features that distinguish our approach are: (i) the careful design of read preprocessing and viral genome optimized mapping steps; (ii) the addition of a two-step editing calling step specifically for (+)ssRNA viruses, which may introduce both A-to-G and T-to-C variants in (+)gRNA and (+)sgRNA sequences during the production of (+)gRNAs and (+)sgRNAs (Supplementary Figure S2); (iii) the incorporation of multiple additional filters to remove misaligned and low-quality reads; (iv) the editing site number filter to remove false-positives due to the overamplification issue of small-genome viruses and (v) the editing level filter to remove variants that are unlikely introduced by an RNA editing mechanism.

To verify our pipeline, we applied it to RNA-seq data from a cell culture model of SARS-CoV-2 infection (43). In brief, Caco-2 and Calu-3 cells were infected with SARS-CoV-2 (patient isolate BetaCoV/Munich/BavPat1/2020|EPI_ISL_406862) at an MOI of 0.33 and sampled at different time points after infection. Next, poly A or total RNA-seq sequencing was performed for each sample. We first confirmed that the conventional pipeline identified different types of RNA variants (Figure 1B and C) and the putative editing events had no ADAR motif preference (Figure 1D). Note that in our analysis, all reads were mapped to the + strand genomic RNA, thus A-to-I editing events that occurred in the + strand were identified as A-to-G variants, and those that occurred in - strand RNA were identified as T-to-C variants. In sharp contrast, the vast majority of the variants identified with the RNA virus-specific pipeline were A-to-G/T-to-C types, indicative of A-to-I editing (Figure 1E). Most importantly, the nucleotides neighboring the A-to-G/T-to-C variants showed a pattern consistent with known ADAR preference (44), i.e., the underrepresentation of G upstream of the editing site (Figure 1F). Notably, the proportions of A-to-G/T-to-C sites and the motif preference were greatly increased with the edited read filtering steps of our pipeline (Figure 1E and F), supporting the efficacy of our filters. As expected, the proportion of viral reads and the number of editing sites increased with the increase of infection time (Figure 1B and G). Together, these data highlight the accuracy of our approach.

### Global characterization of A-to-I RNA editing sites in SARS-CoV-2

**Figure 2.**
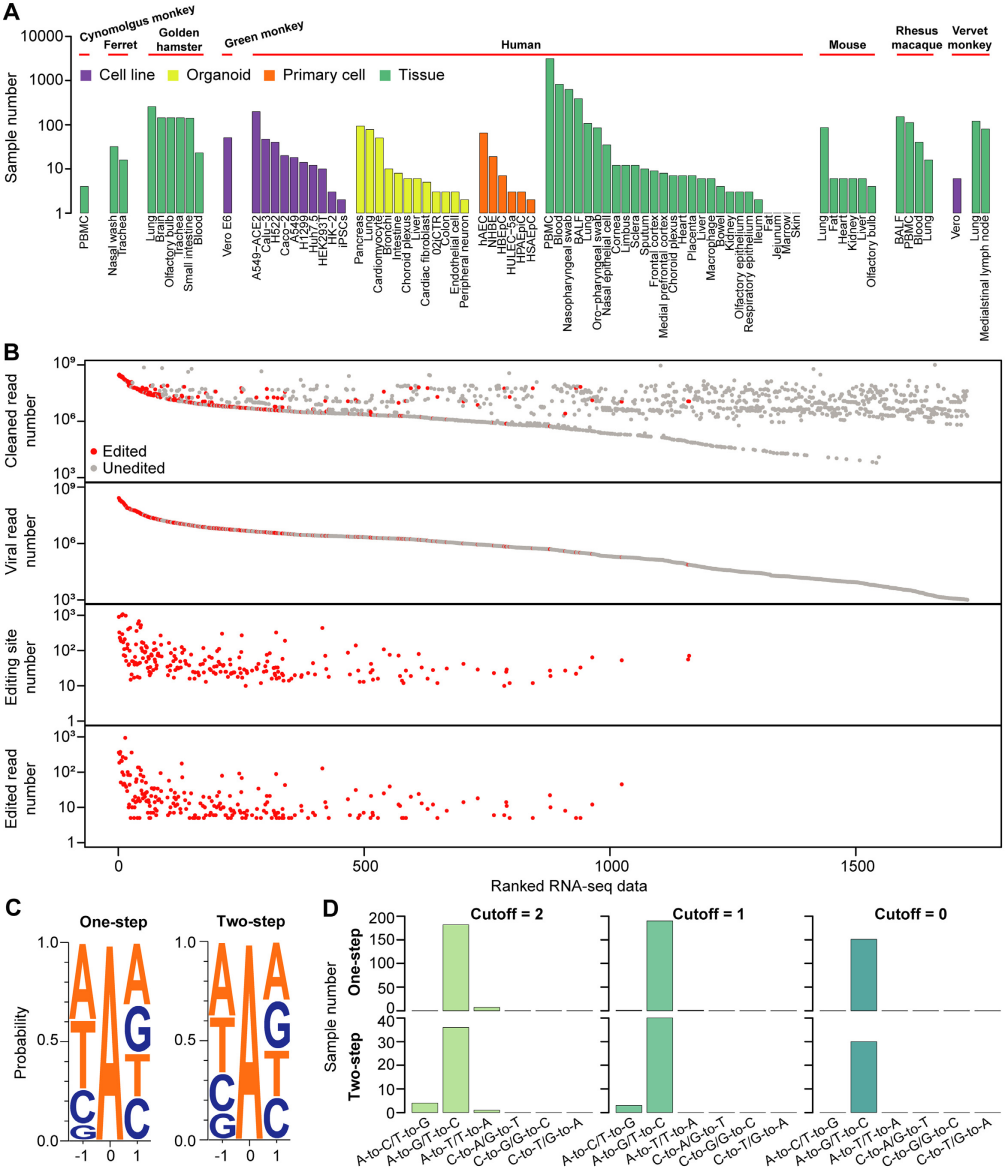
RNA virus-specific pipeline identifies authentic A-to-I RNA editing sites in SARS-CoV-2. (**A**) Summary of the RNA-seq data analyzed. These data were generated from nasopharyngeal swabs of COVID-19 patients, autopsy tissue samples of donors who died of COVID-19, and organoids and cell models infected with SARS-CoV-2. PBMC, peripheral blood mononuclear cells; 02iCTR, a hiPSC line generated from peripheral blood mononuclear cells; hAEC, human aortic endothelial cells; NHBE, normal human bronchial epithelial cells; BALF, bronchoalveolar lavage fluid; HBEpC, primary human bronchial epithelial cells; HPAEpiC, human pulmonary alveolar epithelial cells; HSAEpC, primary human small airway epithelial cells. (**B**) The numbers of total cleaned reads, viral reads, editing sites, and edited reads in RNA-seq data that had viral reads >1000. Samples were ranked by viral read number. (**C**) Nucleotides neighboring editing sites called using either one- or two-step editing pipelines. A total of 8590 and 1415 sites identified from 227 samples with diverse types of origins were used for analysis. (**D**) Numbers of samples with different variant types identified using our method with different mismatch and low-quality site cutoffs (see Materials and methods and Supplementary Figure S1).

Having established the method for virus-specific editing calling, we analyzed RNA-seq data from diverse types of samples for a global characterization of A-to-I RNA editing events in SARS-CoV-2. A total of 7622 published RNA-seq samples, including nasopharyngeal swabs of COVID-19 patients, autopsy tissue samples of donors who died of COVID-19, and organoids and cell models infected with SARS-CoV-2, were collected (Figure 2A and Supplementary Table S1). 1727 (23%) samples were found to have >1000 reads mapped to the SARS-CoV-2 genome (Figure 2B), of which 227 samples had viral editing events (Figure 2B and Supplementary Table S2). Using RNA-seq data from diverse types of samples, we further confirmed that the additional filtering steps we developed could remove false positives (Supplementary Figure S3). On average, approximately 91 editing events were identified per sample. There was a clear positive correlation between the viral load and the detection of RNA editing (Figure 2B). For example, of the samples with viral reads > 5 million, 56% had editing events detected. The overall low percentage of edited reads could be explained by the possibility that A-to-I editing is effective in restricting viral propagation, thus reducing the number of viruses that show evidence of these changes. In total, we identified 8590 and 1415 A-to-G/T-to-C sites from the one-step and two-step editing calling pipelines (Supplementary Table S2), respectively, and a substantial fraction of sites called from the two pipelines were overlapped (Supplementary Figure S4A). As expected, editing sites from both pipelines showed a pattern consistent with known ADAR preference (Figure 2C). As a control, we called other types of variants using the same criteria (see Materials and methods), and very few samples had other types of RNA variants detected (Figure 2D), further supporting the validity of our editing identification pipeline. Note that the use of the previously developed hyper-editing pipeline led to a large number of samples with other types of variants (e.g., A-to-T/T-to-A and A-to-C/T-to-G) identified (Supplementary Figure S4B). Moreover, the A-to-G/T-to-C percentages of sites called with our pipeline were significantly higher than those called with the previously developed hyper-editing pipeline (Supplementary Figure S4C). These analyses highlight the necessity of virus-specific filtering steps in our pipeline.

### Dynamic landscape and properties of A-to-I RNA editing in SARS-CoV-2

**Figure 3.**
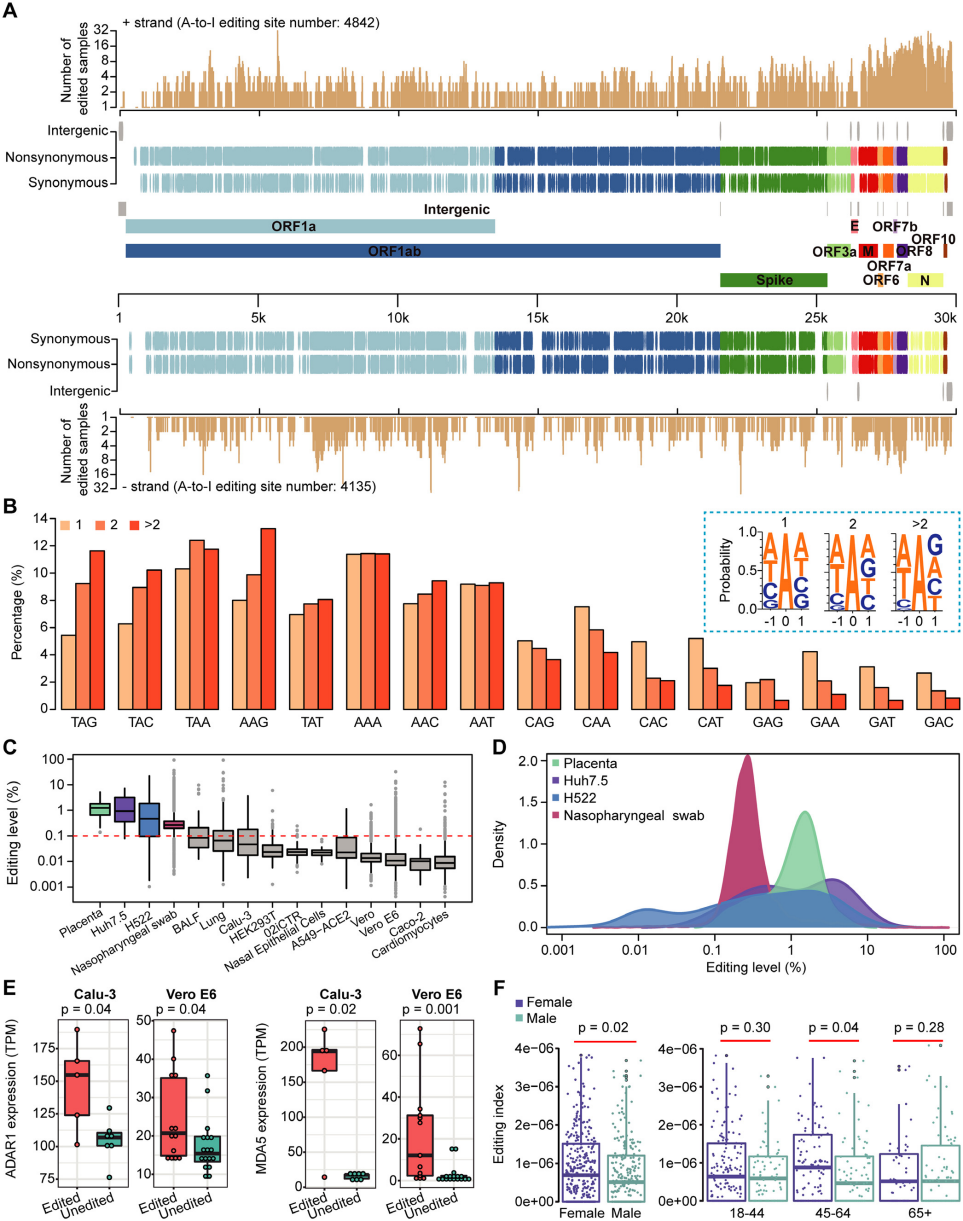
The landscape of A-to-I RNA editing events in the SARS-CoV-2 genome. (**A**) Genic location and annotation of editing sites SARS-CoV-2. A-to-I editing sites in + and − strands were plotted separately. The number of edited samples at each editing site is indicated. (**B**) Comparison of triplet preferences for editing sites identified in one, two, or more than two samples. The triplets were ranked from preferred to disfavored triplets of ADAR1 based on a previous study (44). (**C**) Boxplot showing the editing levels of sites among samples. Only editing sites with coverage ≥30 were used for analysis. Note that for the lowly edited sites (e.g. with editing level < 0.1%), our calculations overestimated the editing level because G reads caused by sequencing errors were also counted as edited reads (see Materials and methods). Red line, sequencing error rate based on our quality cutoff (Q30). (**D**) Density plot showing the distribution of editing level in selected human samples. The four types of samples with the highest median editing levels in C were shown. (**E**) The relationship between editing levels and ADAR1 or MDA5 expression levels. Cell models infected with SARS-CoV-2 from two independent studies (Calu-3: PRJNA625518, Vero E6: PRJNA667051) were used for analysis. Samples were grouped based on their viral editing status, and then ADAR1 and MDA5 expression levels were compared. *P*-values were calculated using one-sided Mann−Whitney *U*-test. (**F**) Comparison of editing indexes between males and females. All individuals (left) or individuals of different ages (right) were analyzed. The editing index was calculated as described in the Materials and methods. *P*-values were calculated using one-sided Mann−Whitney *U*-test.

Next, we investigated the landscape of A-to-I RNA editing in SARS-CoV-2. Similar numbers of editing sites in + and - strands were identified, and both were relatively evenly distributed across the genome (Figure 3A). This result suggests that RNA editing mainly occurs in viral dsRNA intermediates, which can be produced during gRNA replication and sgRNA transcription processes (7,45). In line with this, edited As did not tend to be located in the more structured regions as compared with the unedited As (Supplementary Figure S5). There were a total of 3314 and 2018 non-synonymous editing sites in + and - strands identified, which may potentially affect virus characteristics (Figure 3A and Supplementary Table S2). Moreover, 1417 and 2068 synonymous editing sites in + and − strands were found (Figure 3A and Supplementary Table S2), some of which might play additional regulatory roles in the viral genome. Note that some of the negative-strand RNAs with A-to-I editing (i.e. T-to-C variants) may not be further used as a template for (+)gRNA or (+)sgRNA synthesis and thus did not recode or regulate the viral genome.

To ask whether editing hotspots were present in the viral genome, we examined the number of edited samples at each editing position. We found 2271 sites present in multiple samples, ranging from 3 to 41 (Figure 3A). As a group, the triplet motifs of editing hotspots were biased towards the ones most favored by ADAR1 (e.g., TAG) and biased against those unfavored by ADAR1 (e.g. GAN) (Figure 3B), which underlies the basis of their high editability (44).

To understand the dynamics of RNA editing, we performed three analyses. First, we compared the overall editing levels between samples and found that they varied (Figure 3C), which was probably due to the different levels of MDA5 associated response. Moreover, editing levels among sites varied within a sample (Figure 3D). Second, we examined the association between MDA5/ADAR1 expression and editing status using cell models infected with SARS-CoV-2. Higher expression of MDA5/ADAR1 was observed in edited samples (Figure 3E and Supplementary Figure S6), suggesting that MDA5-mediated innate response may promote viral RNA editing. When quantifying the expression of ADAR1 p110 and ADAR1 p150 isoforms separately, we found that only ADAR1 p150 had an increased expression in edited samples (Supplementary Figure S6), consistent with the previous observation (17). Third, we analyzed the viral editing status of 617 nasopharyngeal swab samples from the New York City metropolitan area during the COVID-19 outbreak in spring 2020 (27). We found that females had a higher viral editing index than males, particularly for those aged between 45 and 64 (Figure 3F), which is consistent with previous observations that adult females mount stronger innate immune responses than males in general (46). Altogether, these data suggest that A-to-I RNA editing in SARS-CoV-2 was dynamically regulated.

### Impacts of A-to-I RNA editing on the spike protein functions

**Figure 4.**
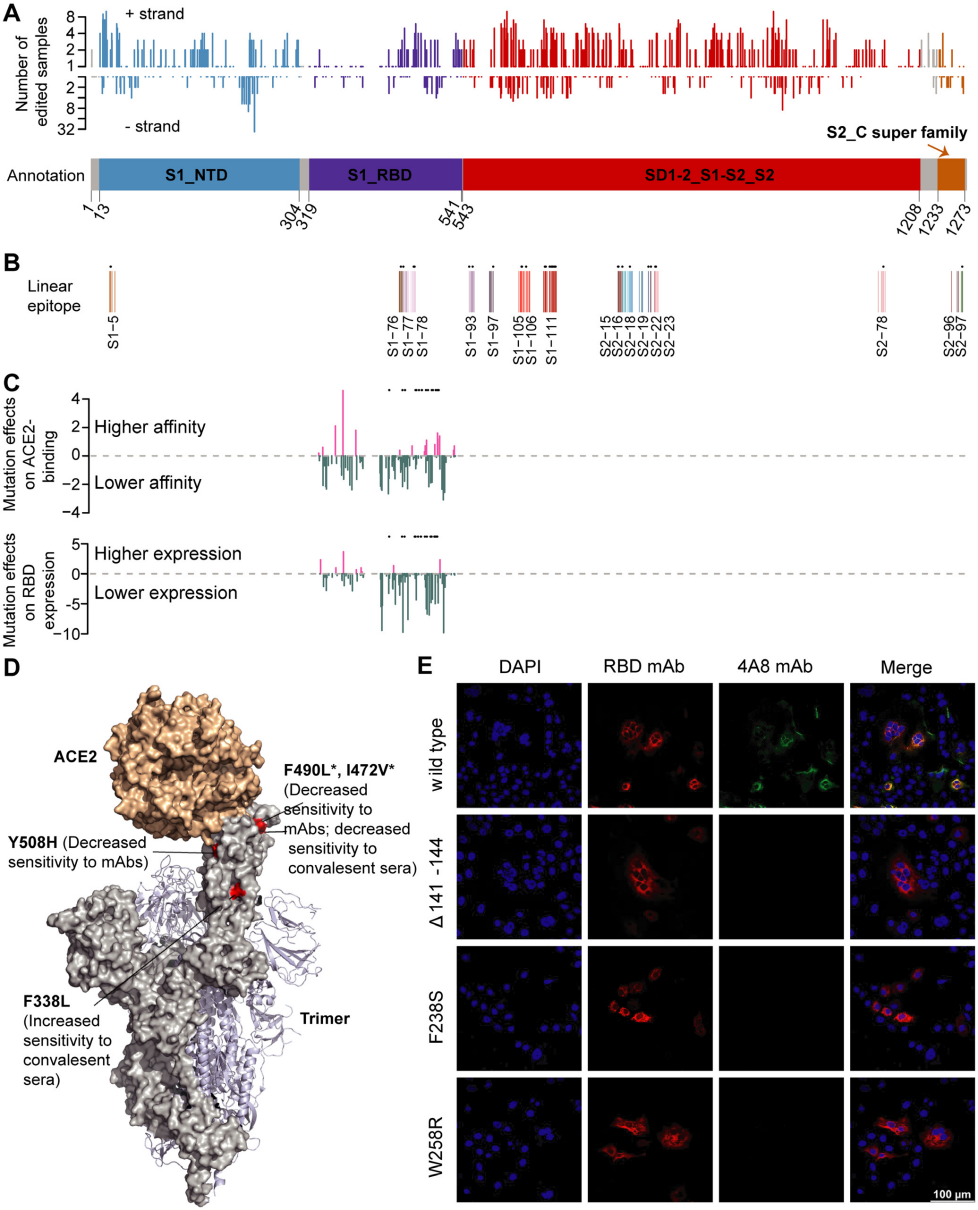
Impacts of RNA editing on spike protein functions. (**A**) The distribution of recoding editing sites in the spike protein. The number of edited samples at each editing site is indicated. Spike protein annotation information was from NCBI SARS-CoV-2 Protein Domains resource, and different domains are shown by different colors. S1_NTD, N-terminal domain of the S1 subunit; S1_RBD, receptor-binding domain of the S1 subunit; SD1-2_S1-S2_S2, SD-1 and SD-2 subdomains, the S1/S2 cleavage region, and the S2 fusion subunit; S2_C super family, S2 subunit intravirion. (**B**) The recoding sites that were located at the linear epitope regions of the spike protein. Linear epitope regions were mapped by Li *et al.* (50). The editing sites detected in more than two samples are labeled as ‘*’. (**C**) The effects of recoding editing on ACE2 binding (top) and RBD expression (bottom). Data were from Starr *et al.* (30). Positive value, higher affinity; negative value, lower affinity. The values were normalized to the median values of all positive or negative mutation effects. The editing sites detected in more than two samples are labeled as ‘*’. (**D**) Structure of spike protein bound to ACE2. Four recoding sites found to affect viral infectivity and antigenicity are highlighted in red. Data were from Li *et al.* (31). Note that I472V recoding site in the D614G background can further lead to increased infectivity and decreased sensitivity to neutralizing mAb and convalescent sera. The editing sites detected in more than two samples are labeled as ‘*’. (**E**) Spike protein distribution in Vero E6 cells at 36 h after transfection with the editing mutants or wild type gene, visualized by indirect immunofluorescence. Mutation of Δ141–144, which completely abolished the binding of antibody 4A8 (32), was used as the positive control. F238S and W258R were two editing mutants of the spike gene. RBD mAb (red) was a neutralizing antibody targeting the spike RBD; 4A8 mAb (green) was a neutralizing antibody targeting spike NTD. Scale bar, 100 μm.

The trimeric spike protein decorates the surface of coronavirus and plays a key role in the receptor recognition and cell membrane fusion process (47). The spike protein is composed of two subunits, S1 and S2 (Figure 4A). The S1 subunit contains a receptor-binding domain (RBD) that recognizes and binds to the host receptor angiotensin-converting enzyme 2 (ACE2), while the S2 subunit mediates viral cell membrane fusion by forming a six-helical bundle via the two-heptad repeat domain. The spike protein is the major antigen inducing protective immune responses and the major target for vaccine development (48,49). Therefore, the editing-introduced mutations in the spike protein may provide a new source for its evolution and adaptation in humans. We found a total of 751 A-to-G/T-to-C recoding editing sites in the spike protein (Figure 4A and Supplementary Table S3). An integrated analysis of these recoding sites with three published functional assay data revealed that these sites may affect virus characteristics in a variety of ways. First, a linear epitope landscape of the spike protein was generated by analyzing the serum immunoglobulin G (IgG) response of 1051 COVID-19 patients with a peptide microarray (50). We found that 151 recoding sites were located at the linear epitope regions of the spike protein, which may alter the immunogenicity of the epitope and thus affect the host immunogenic response (Figure 4B). Second, a quantitative deep mutational scanning approach was applied to experimentally measure how all possible SARS-CoV-2 RBD amino acid mutations affect ACE2- binding affinity and protein expression (30). Base on this data, we found that a few recoding sites might enhance RBD expression and ACE2 affinity (Figure 4C). Because the RBD is a major determinant of viral infectivity, pathogenesis and host range, these recoding events in RBD may impact these processes. Third, over 100 spike protein mutants were generated, and their infectivity and reactivity to neutralizing antibodies were analyzed using the high-throughput pseudotyped virus system (31). Based on this data, we found that four recoding sites altered the ACE2 binding affinity to the spike protein and affected viral infectivity and antigenicity (Figure 4D).

To experimentally examine the impact of RNA editing on altering antigenicity, we selected seven recoding editing spots located in the N-terminal domain (NTD) of the spike protein. We generated spike mutants with individual sites and assessed the impact of RNA editing on antibody binding. We also included a deletion mutant that is known to affect the binding of 4A8 mAb, a neutralizing antibody targeting the NTD (32). Cells were transfected with plasmids expressing these mutants, and indirect immunofluorescence was used to determine whether editing modulated the binding of 4A8 mAb as previously described (32). Of the 7 editing sites, two sites completely abolished binding of 4A8 while still allowing recognition by a neutralizing antibody targeting the RBD (Figure 4E and Supplementary Figure S7), indicating that some of the editing mutants can confer resistance to neutralizing antibodies. Altogether, these results suggest that RNA editing provides a new source to regulate virus-host interactions.

### A-to-I RNA editing fuels SARS-CoV-2 evolution during the epidemic

**Figure 5.**
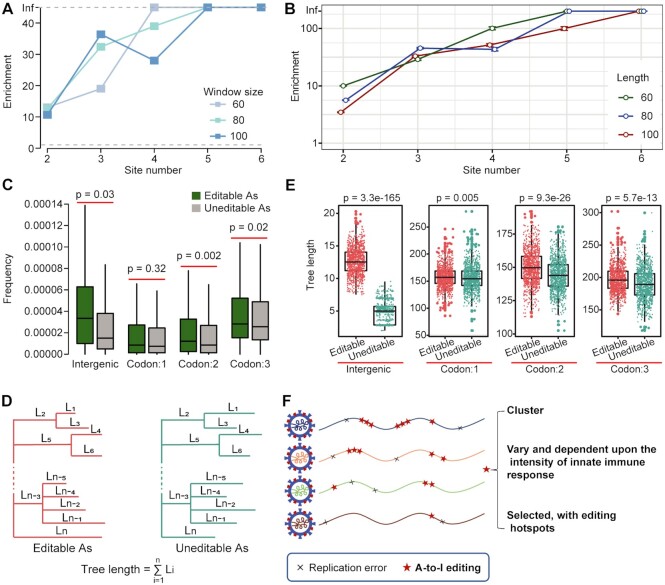
A-to-I RNA editing fuels SARS-CoV-2 evolution in humans. (**A**, **B**) Enrichment of the clustered A-to-G sites in the global samples of SARS-CoV-2. We used two different controls for enrichment calculation. In panel A, enrichment was defined as the number of clusters with A-to-G mutations in editable A positions divided by that with A-to-C mutations in editable A positions (see Materials and methods). Window size, A-to-G or A-to-C mutation clusters (from 2 sites to 6 sites) scanned using a 60-, 80- or 100-nt sliding window. In panel B, enrichment was defined as the number of clusters with A-to-G mutations within defined lengths (60-, 80- or 100-nt) in editable A positions divided by that with A-to-G mutations within the same lengths in the randomly sampled uneditable A positions (see Materials and methods). Control sites were sampled 1000 times and 95% confidence interval is shown. In both A and B, ‘Inf’ means no GISAID sequences in the control sets were identified. (**C**) Comparison of the GISAID A-to-G variant frequencies between editable and uneditable As. Sites located in intergenic regions and three codon positions were analyzed separately. *P*-values were calculated using one-sided Mann-Whitney U-test. (**D**) Illustration of phylogenetic tree construction and tree length calculation using editable and uneditable A positions of genome sequences deposited in GISAID (see Materials and methods). (**E**) Comparison of tree lengths between editable and uneditable As. *P*-values were calculated using paired Mann−Whitney *U*-test. (**F**) Summary of the characteristic of A-to-I editing on SARS-CoV-2. Compared with replication error-introduced substitutions, A-to-I editing sites tends to be clustered. Moreover, editing levels vary among tissue types and between sexes. Finally, since ADAR has a motif preference, the editing sites are selected, and editing hotspots are presented.

The RNA editing events may occur in both strands of sgRNAs and gRNAs. Those in sgRNAs may only affect the characteristics of the viruses within the infected individual; while those in gRNAs might transmit, thus fueling viral evolution in humans. Given the overall low-level editing in SARS-CoV-2, we expect that A-to-I editing may have a low or moderate effect on the infected individual. However, RNA editing introduced substitutions might provide a new source of mutation and contribute to SARS-CoV-2 evolution in human populations. Extensive global sampling and sequencing of SARS-CoV-2 have enabled us to examine the impact of A-to-I RNA editing on its evolution. Because A-to-I editing tended to be clustered, we reasoned that if A-to-I editing contributes to virus evolution, such cluster signature could be found in viral sequences. Thus, we searched the GISAID sequence database (37) for sequences containing clustered A-to-G mutations in the editing site positions (see Materials and methods). GISAID is a global science initiative and primary source that provides open access to genomic data of viruses, including SARS-CoV-2 (37). Several million SARS-CoV-2 genome sequences have been shared on GISAID, which is helpful for us to track and analyze the spread of viral variants. As a control, we searched clustered A-to-C mutations in the same positions, because experimental data suggest that SARS-CoV-2 had comparable rates of replication error-introduced A-to-G and A-to-C mutations (9). We found 11–39-fold enrichment of sequences containing clustered A-to-G mutations (Figure 5A), supporting our hypothesis. We also used randomly sampled A positions without editing events observed (in short, uneditable As) as a control, and similar enrichment was observed (Figure 5B).

If A-to-I RNA editing accelerates SARS-CoV-2 evolution in humans, we would expect that the A positions with editing events observed (in short, editable As) may be more likely fixed as Gs during the epidemic. We thus compared the frequencies of A-to-G mutations inferred using sequences in GISAID between editable and uneditable As in the genome. We found that editable As tended to have higher frequencies, particularly for the sites in the intergenic regions (Figure 5C), which are expected to be under less selective constraints. We also compared the relative rates of evolution between editable and uneditable As in a phylogenetic context. We extracted the editable and uneditable As from sequences in GISAID, respectively, constructed two phylogenetic trees, and summed up and compared the branch lengths of two trees (Figure 5D, see Materials and methods). We found that the total branch lengths of editable A positions were longer than those of uneditable A positions (Figure 5E), suggesting an editing-dependent accelerated evolution of editable A positions of SARS-CoV-2 in humans. Together, these analyses imply that RNA editing as a possible source of mutations can fuel viral evolution.

## DISCUSSION

Knowledge of the host immune response factors that introduce mutations is important for the simple reason that RNA viruses, particularly (+)ssRNA viruses, are major pathogens of humans. In this study, we developed an approach to robustly identify A-to-I RNA editing in RNA viruses. By applying our approach to SARS-CoV-2, we characterized the landscape of RNA editing in the SARS-CoV-2 genome and the association between viral editing and intensity of host innate immune response. The comprehensive lists of SARS-CoV-2 A-to-I editing sites and hotspots identified here present a resource for the study of SARS-CoV-2 biology, evolution and spread. Moreover, when applying our approach to four other human (+)ss RNA viruses (SARS-CoV, MERS-CoV, Zika virus and Dengue virus), we found the presence of A-to-I RNA editing in all four viruses investigated (Supplementary Figure S8 and Supplementary Table S4), suggesting that our approach is readily applicable to a wide range of RNA viruses.

**Figure 6.**
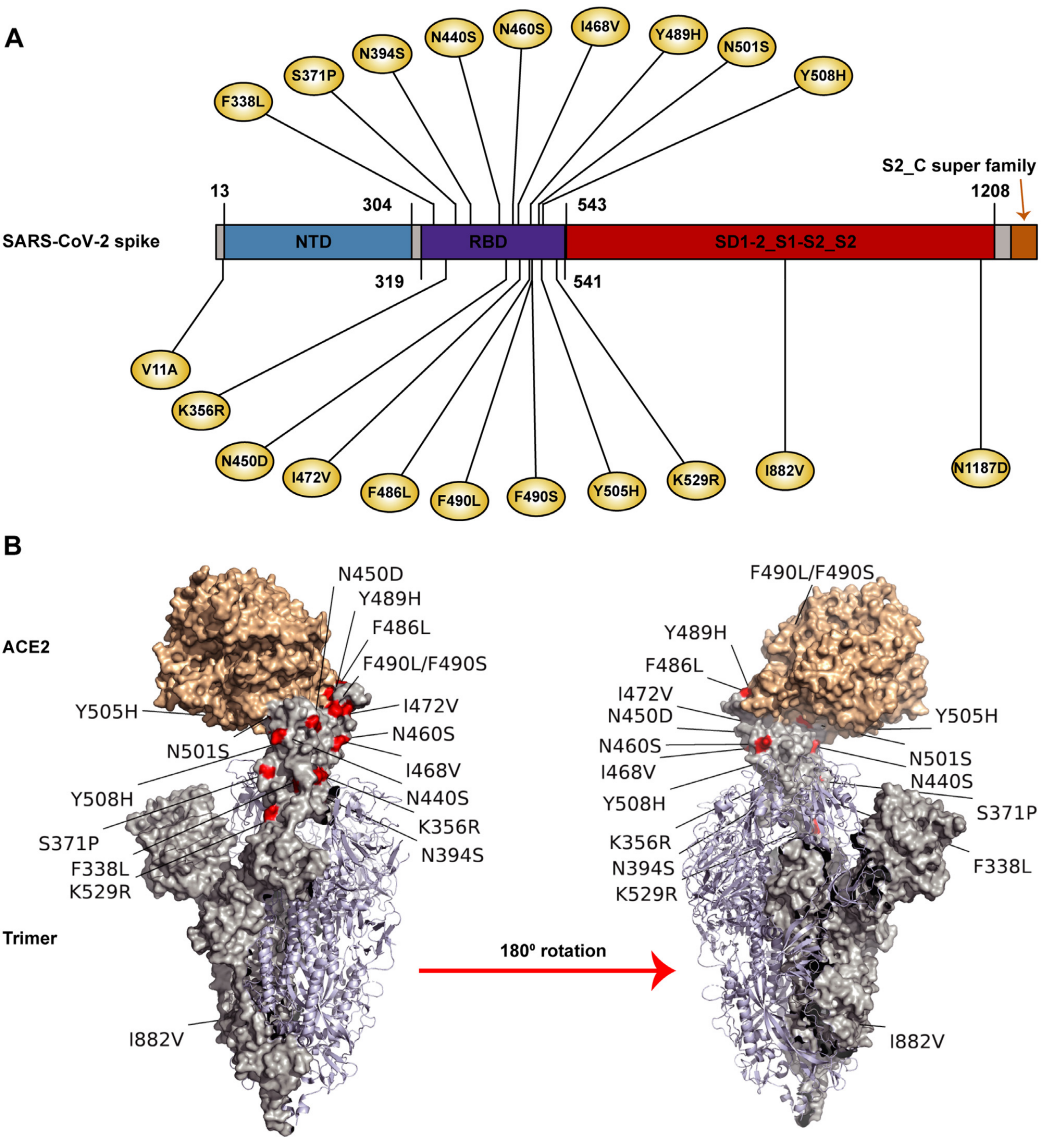
Overlaps between editing sites and watch list variants in spike protein. (**A**) Recoding editing sites that were overlapped with watch list variants in the spike protein. Domains were annotated as in Figure 4A. Variant watch lists were downloaded from GISAID database on June-26–2021. (**B**) Structure of spike protein bound to ACE2. Recoding editing sites that were overlapped with watch list variants are highlighted in red.

In combination with an integrative analysis of GISAID sequence database (Supplementary Table S5) and our full list of viral editing sites, we revealed that A-to-I editing provides a new source of substitutions to fuel the evolution of SARS-CoV-2 in humans during the epidemic. A further comparison between our editing list and watch lists of variants of concern in the world revealed 20 overlapping sites (Figure 6), suggesting that host RNA editing machinery does have the ability to generate variants of concern.

Compared with replication error-introduced substitutions, A-to-I editing-introduced substitutions have distinct features (Figure 5F). First, RNA editing tends to be clustered, and such substitutions may lead to stronger changes in a small region, which provides a unique mode of substitutions for virus evolution. Second, editing levels vary among tissue types and between sexes, dependent upon the intensity of the innate immune response; thus, caution may be needed for individuals who have a stronger innate immune response and likely generate a high-level of editing-introduced A-to-G substitutions. Third, since ADAR has a motif preference, the editing sites are selected, and editing hotspots are presented. We propose that editing hotspots in the SARS-CoV-2 spike protein that enhance ACE2 affinity may be integrated into mRNA vaccine design in advance to defend against possible new variants.

## NOTE ADDED IN PROOF

While this paper was being revised, Picardi et al. (51) published a study to examine whether and to what extent A-to-I RNA editing is present in SARS-CoV-2 in infected cell model using a previously developed pipeline (42). Although both studies focus on A-to-I RNA editing of SARS-CoV-2, our study aim to develop an RNA virus-specific editing identification pipeline, examine the viral editing status from diverse types of samples infected with SARS-CoV-2, construct an atlas of A-to-I RNA editing sites, and uncover the editing landscape and hotspots in the viral genome. Thus, the focuses and aims of the two studies are largely different.

## DATA AVAILABILITY

All raw sequencing data were downloaded from NIH SARS-CoV-2 resource website (Supplementary Table S1). All other sequences are available via the GISAID database (www.gsaid.org; see Supplementary Table S5 for a full list of acknowledgments). All relevant code and data processing pipelines have been deposited in GitHub (https://github.com/SYSU-zhanglab/RNA-virus-editing).

## Supplementary Material

gkac120_Supplemental_Files
