# Supplementary material for: Virus-specific editing identification approach reveals the landscape of A-to-I editing and its impacts on SARS-CoV-2 characteristics and evolution
Source: Nucleic Acids Res. 2022 Mar 2;50(5):2509–21. doi: 10.1093/nar/gkac120 (PMC8934641; doi:10.1093/nar/gkac120)
Supplement: gkac120_Supplemental_Files [file gkac120_supplemental_files.zip › SUPPLEMENTARY_DATA_revison-.pdf]

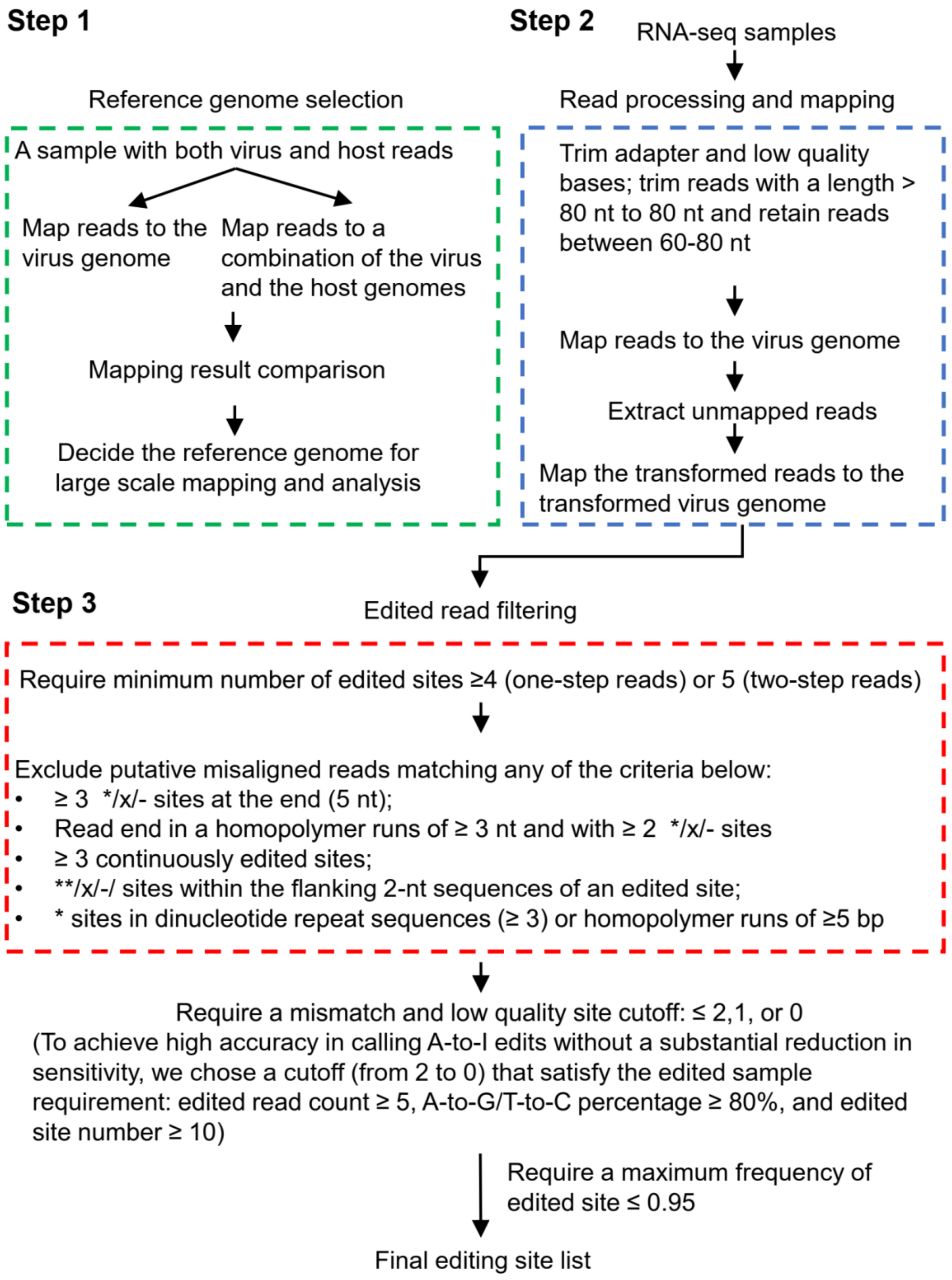

Figure S1. Our computational pipeline development.

Schematic diagram of the RNA virus-specific RNA editing analysis pipeline. In step 1, using test samples, all viral reads were found to be unambiguously mapped to the virus genome but not host genome. Therefore, in step 2, for large-scale data analysis, we mapped RNA-seq data to the virus genome directly. We considered T-to-C variants

as ADAR mediated RNA editing events of the negative-strand RNA of SARS-CoV-2. “*” represents edited sites with base quality ≥ 30, “-” represents edited sites with base quality < 30, “x” represents non-A-to-G/T-to-C sites.

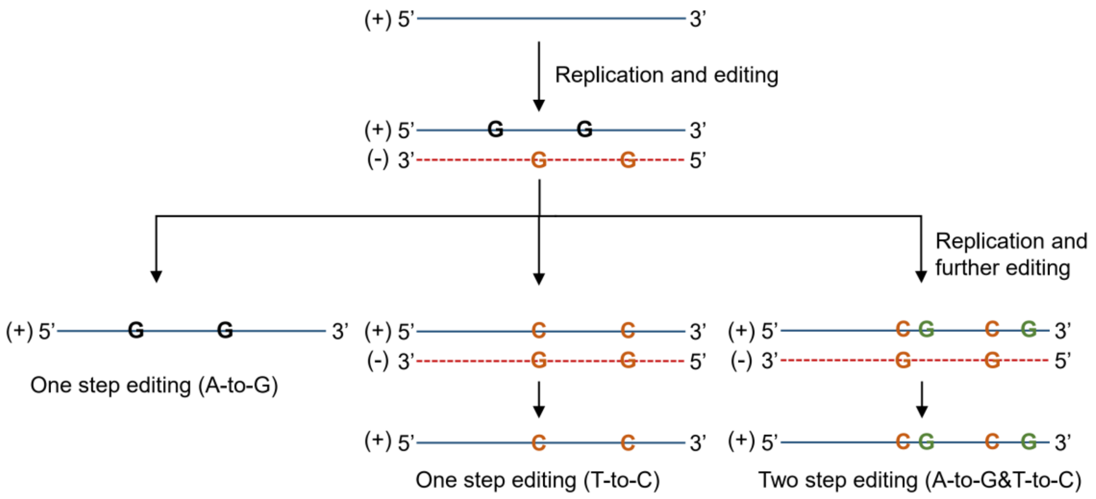

Figure S2. Illustration of the one- and two-step editing generation of (+)ss RNA virus.

In the one-step editing procedure, ADAR1 bound to the dsRNA and edited both strands. Those occurring in the sense strand were detected as A-to-G variants, and those occurring in the negative-strand were detected as T-to-C variants. In the two-step editing procedure, the edited negative-strand may be further used for (+)gRNA or (+)sgRNA generation and form dsRNAs. These dsRNAs were edited again in the sense strand and then both A-to-G and T-to-C variants were simultaneously detected in a read.

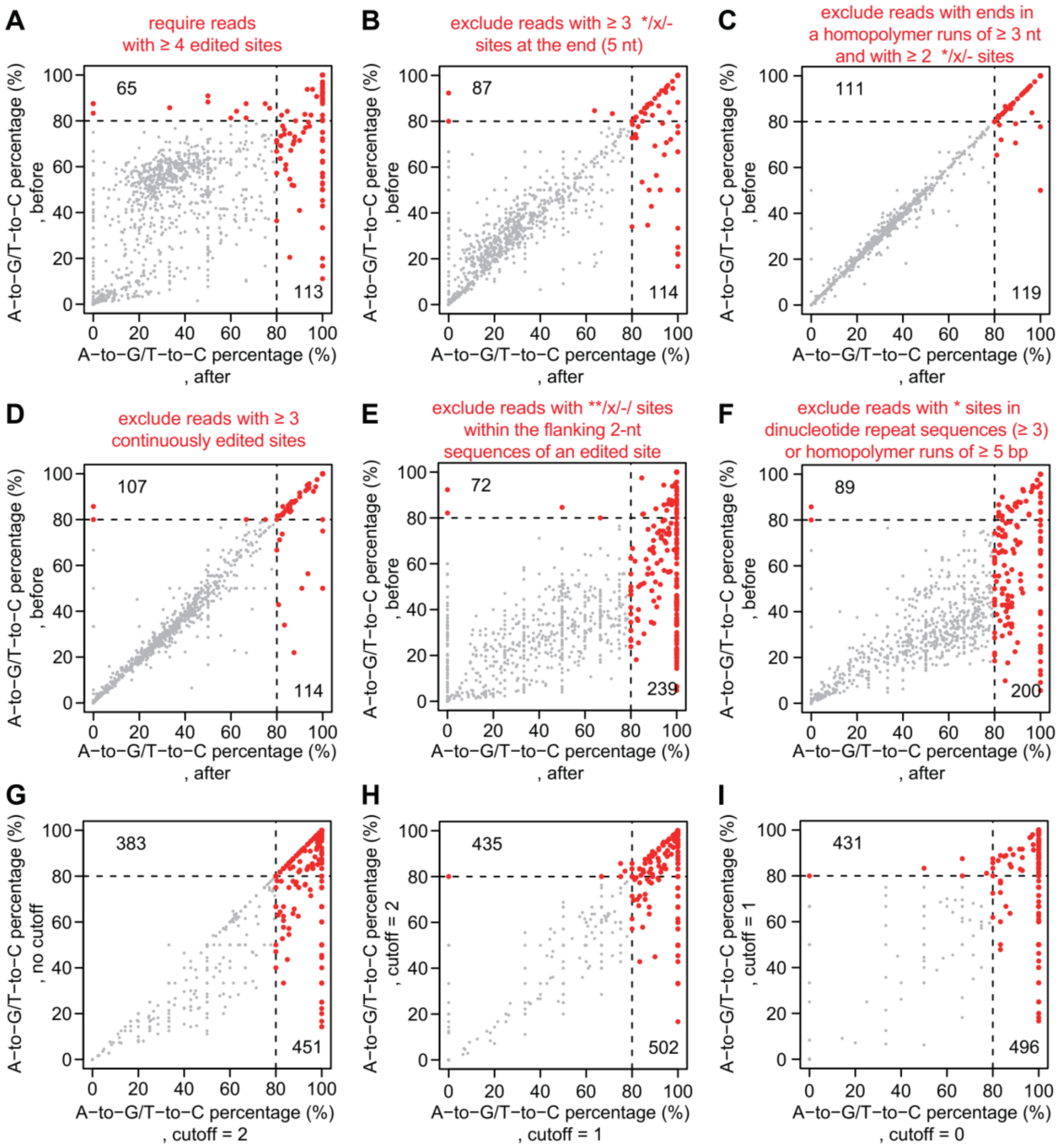

Figure S3. The impacts of each filtering procedure applied in our computational pipeline.

(**A**) The numbers of samples that were with an A-to-G/T-to-C percentage ≥ 80% before and after the first filtering step (require that the reads had a minimum number of edited sites ≥ 4). The numbers of samples that were with an A-to-G/T-to-C percentage ≥ 80% before and after the filtering step are indicated at the top-left and bottom-right, respectively.

(**B-F**) The numbers of samples that were with an A-to-G/T-to-C percentage ≥ 80% before and after the additional filtering step (require that the reads had a minimum

number of edited sites ≥ 4). For this analysis, the reads that were passed the first filtering step were used.

(**G-I**) The numbers of samples that were with an A-to-G/T-to-C percentage ≥ 80% before and after the last cutoff filter (require that the reads had no more than 2, 1, or 0 mismatch and low-quality sites). For this analysis, the reads that were passed the first and all additional filtering steps were used.

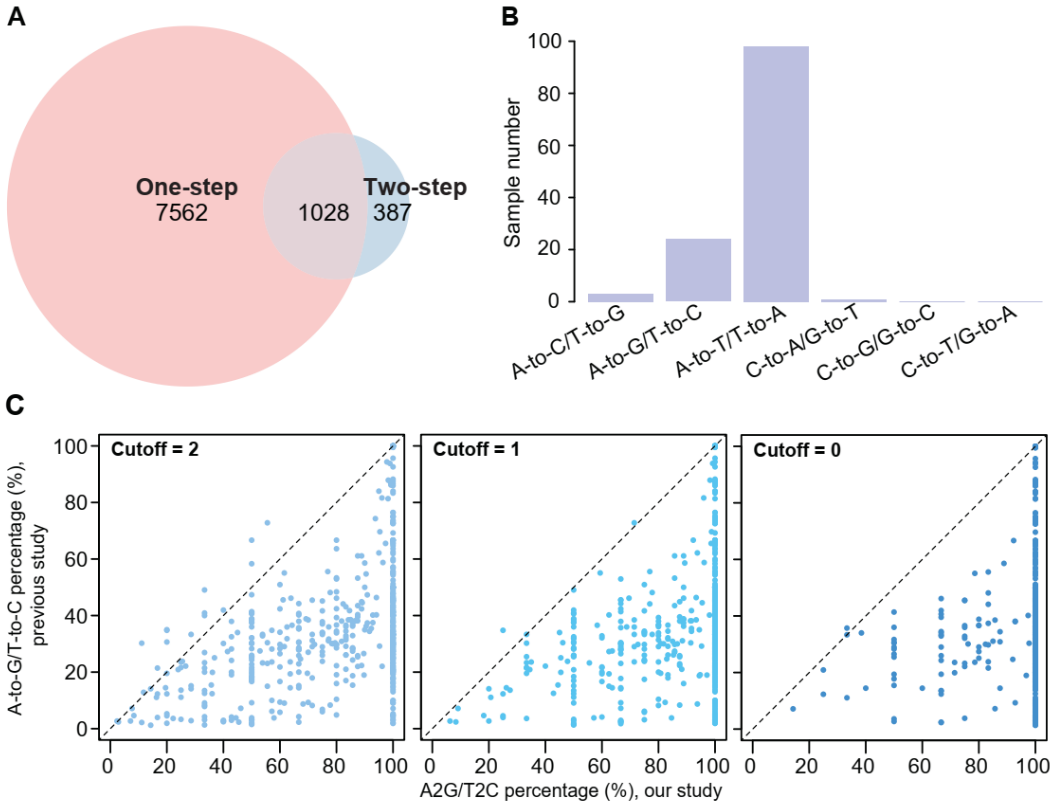

Figure S4. Identification of A-to-I RNA editing sites in SARS-CoV-2.

(**A**) Overlaps of editing sites identified by one- and two-step editing call pipelines.

(**B**) The performance of the previous hyper-editing pipeline. The numbers of samples with different types of variants are shown.

(**C**) Comparison of A-to-G/T-to-C percentages of variants called between our pipeline and previous hyper-editing pipeline.

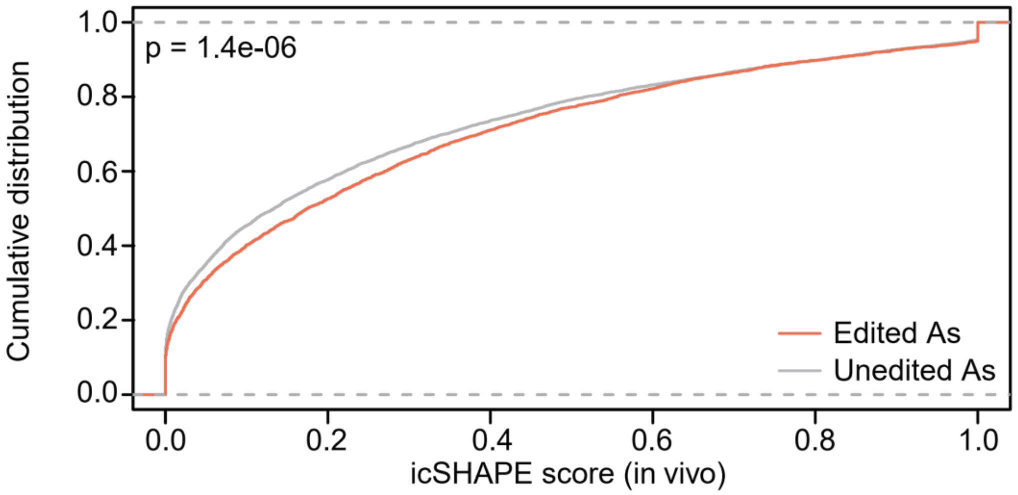

Figure S5. Comparison of the icSHAPE scores between edited and unedited As in the (+)gRNA.

A higher score indicates that a nucleotide is more likely single-stranded. P-value was calculated using Kolmogorov-Smirnov test.

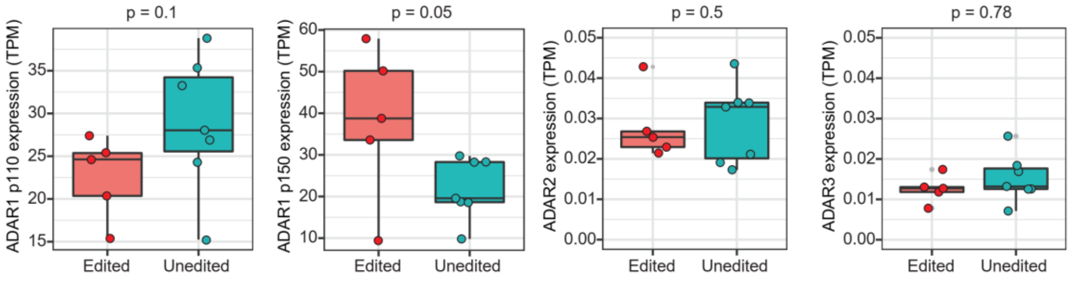

Figure S6. Comparison of gene expression levels of ADAR1 p110, ADAR1 p150, ADAR2, and ADAR3 in human Calu-3 cell line samples with edited and unedited viruses. Samples were grouped based on their viral editing status, and then gene expression levels were compared. Edited sample means the sample with A-to-G/T-to-C percentage ≥ 80%, editing site number ≥ 10, and edited read number ≥ 5. P-values were calculated using one-sided Mann-Whitney U-test.

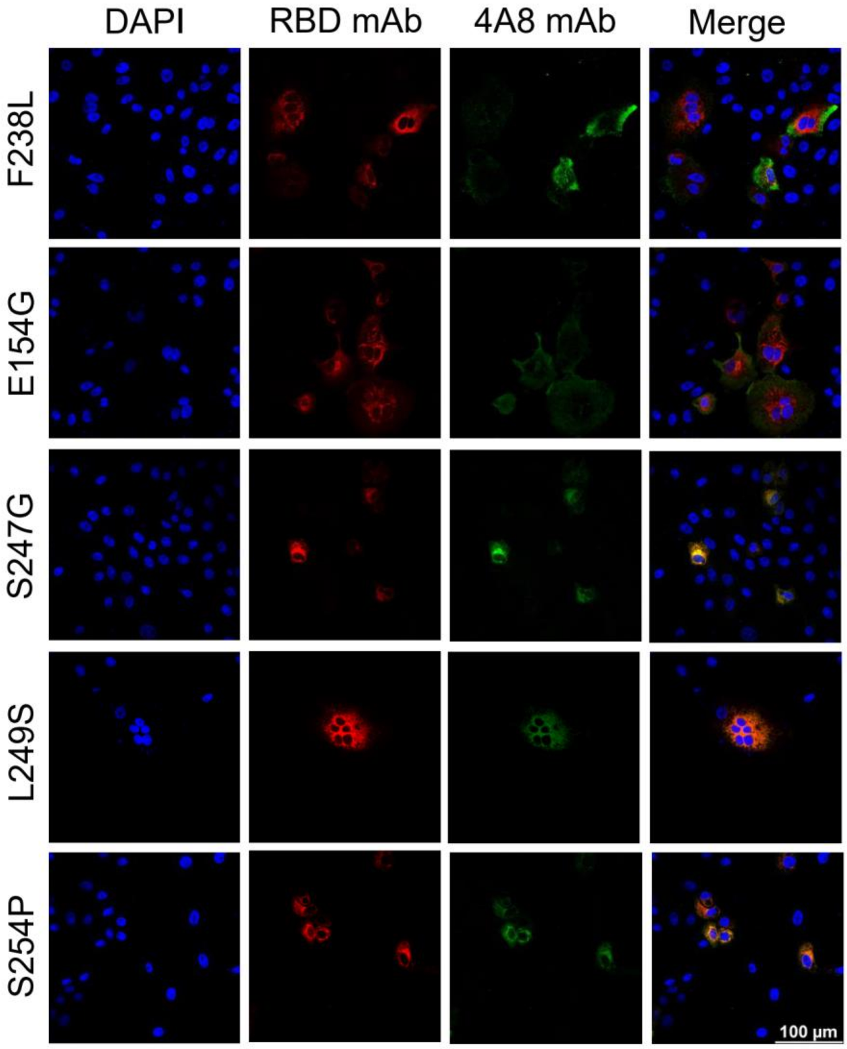

Figure S7. The immunofluorescence staining of different spike editing mutants. F238L, E154G, S247G, L249S, and S254P were mutants with editing sites in the NTD. RBD mAb (red) is a monoclonal antibody to the RBD; 4A8 mAb (green) is a monoclonal antibody to the NTD. Scale bar, 100 μm.

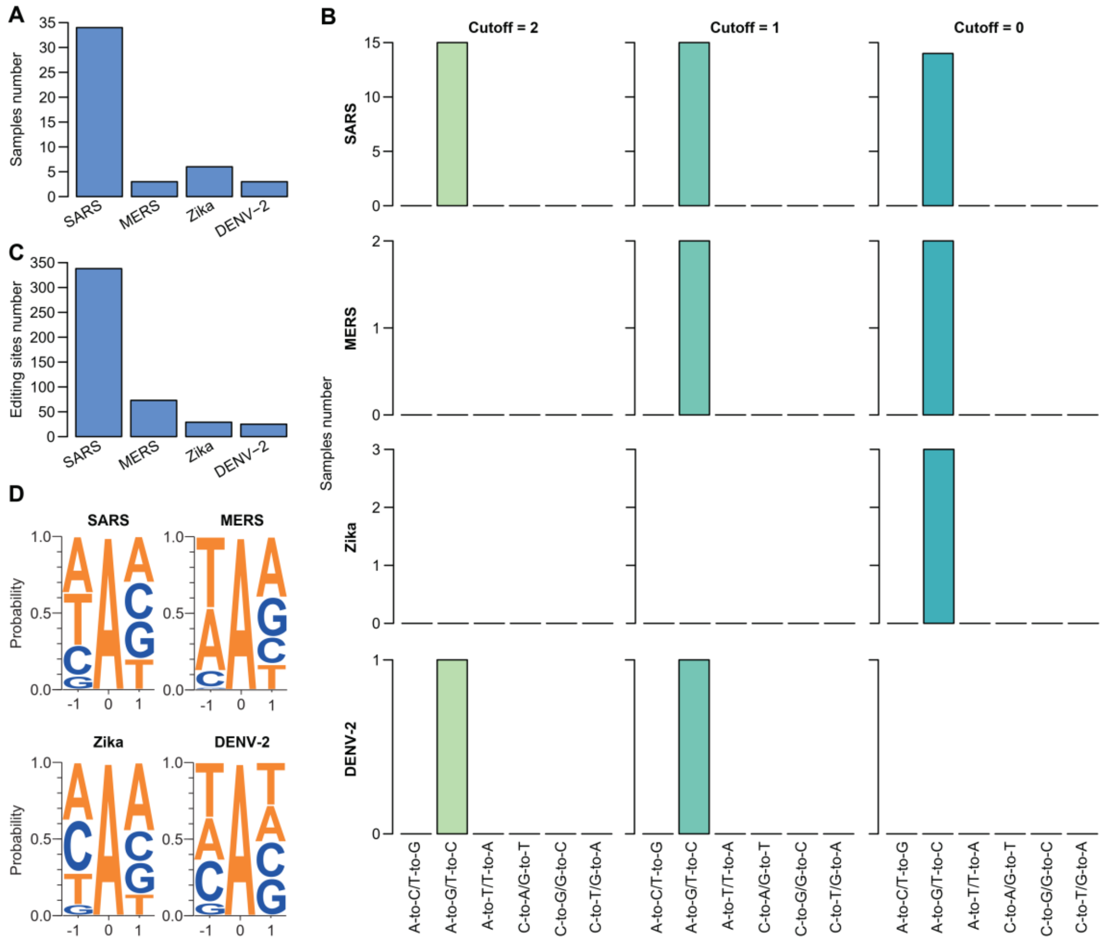

Figure S8. RNA virus-specific pipeline identifies A-to-I RNA editing sites in other RNA viruses as well.

(**A**) Summary of the RNA-seq data analyzed. A total of 52 RNA-seq data from four types of RNA viruses, including SARS-CoV, MERS-CoV, Zika virus and Dengue virus, were collected.

(**B**) Numbers of samples with different variant types identified using our method with different mismatch and low-quality site cutoffs.

(**C**) The numbers of editing sites in different RNA viruses. For each virus type, all sites identified were combined.

(D) Nucleotides neighboring editing sites in each RNA virus.

Legend for table S5: A full list of GISAID sequence data used in this study
