# Supplementary material for: Virus-specific editing identification approach reveals the landscape of A-to-I editing and its impacts on SARS-CoV-2 characteristics and evolution
Source: Nucleic Acids Res. 2022 Mar 2;50(5):2509–21. doi: 10.1093/nar/gkac120 (PMC8934641; doi:10.1093/nar/gkac120)
Supplement: gkac120_Supplemental_Files [file gkac120_supplemental_files.zip › Table S5.pdf]

We gratefully acknowledge the following Authors from the Originating laboratories responsible for obtaining the specimens, as well as the Submitting laboratories where the genome data were generated and shared via GISAID, on which this research is based.

All Submitters of data may be contacted directly via www.gisaid.org

Authors are sorted alphabetically.

Accession ID	Originating Laboratory	Submitting Laboratory	Authors
EPI_ISL_875674	"1.AO Universitaria 'S. Giovanni di Dio e Ruggi D'Aragona, Scuola Medica Salernitana' Hospital / 2.UOC di Virologia e Microbiologia, Università della Campania 'L. Vanvitelli' / 3.AO Universitaria 'Federico II' Napoli Hospital / 4.AORN 'San Giuseppe Moscati' Avellino Hospital / 5.AO 'San Pio - presidio G. Rummo' Benevento Hospital / 6.AO 'Sant'Anna e San Sebastiano' Caserta Hospital / 7.PO 'Maria Santissima Addolorata' Eboli Hospital / 8.Biogem Istituto di Ricerche Genetiche"	"1. Genome Research Center for Health (CRGS) / 2. Laboratory of Molecular Medicine and Genomics(LMMGe) / 3. Center for Research in Pure and Applied Mathematics (CRMPA)"	"Giorgio Giurato (Corresponding Author); Alessandro Weisz (Corresponding Author); Alessia Cossu; Aniello Gentile; Annamaria Salvati; Antonello Saccomanno; Arnolfo Petruzziello; Assunta Sellitto; Carlo Ferravante; Domenico Memoli; Domenico Palumbo; Elena Alexandrova; Emilia Vaccaro; Francesca Marciano; Francesca Rizzo (Corresponding Author); Gianluigi Franci; Giovanni Nassa; Giovanni Pecoraro; Giuseppe Fenza; Giuseppe Portella; Gregorio Goffredi; Ilaria Terenzi; Jessica Lamberti; Maddalena Schioppa; Maria Grazia Foti; Maria Landi; Marianna Scrima"; Mariarosaria Ingino; Massimiliano Galdiero; Maurizio Fumi; Michele Caraglia; Michele Cennamo; Oriana Strianese; Pasquale Pagliano; Rita Greco; Roberta Tarallo; Sonia Amabile; Teresa Rocco; Valeria Mirici Cappa; Vincenzo Rocco; Viola Melone; Vittoria Letizia; Ylenia D'Agostino
EPI_ISL_1225874 to 1225880, EPI_ISL_1226177 to 1226191	"AK State Public Health Lab, State Health Department"	Genomics and Discovery, Respiratory Viruses Branch, Division of Viral Diseases, Centers for Disease Control and Prevention	Anna Montmayeur; Anna Uehara; Ben L. Rambo-Martin; Clinton R. Paden; Dhwani Batra; Haibin Wang; Jasmine Padilla; Jing Zhang; Justin Lee; Katie Dillon; Krista Queen; Kristen Knipe; Kristine Lacek; Lori Rowe; Mark Burroughs; Matthew Schmerer; Mili Sheth; Peter W. Cook; Rachel Marine; Sam Shepard; Sarah Nobles; Shoshona Le; Suxiang Tong; Yan Li; Ying Tao
EPI_ISL_1226381, EPI_ISL_1226407	"AZ SPHL, Arizona Department of Health Services"	Genomics and Discovery, Respiratory Viruses Branch, Division of Viral Diseases, Centers for Disease Control and Prevention	Anna Montmayeur; Anna Uehara; Ben L. Rambo-Martin; Clinton R. Paden; Dhwani Batra; Haibin Wang; Jasmine Padilla; Jing Zhang; Justin Lee; Katie Dillon; Krista Queen; Kristen Knipe; Kristine Lacek; Lori Rowe; Mark Burroughs; Matthew Schmerer; Mili Sheth; Peter W. Cook; Rachel Marine; Sam Shepard; Sarah Nobles; Shoshona Le; Suxiang Tong; Yan Li; Ying Tao
EPI_ISL_1225958 to 1225974, EPI_ISL_1226237 to 1226275	"CDPH, Viral and Rickettsial Disease Laboratory"	Genomics and Discovery, Respiratory Viruses Branch, Division of Viral Diseases, Centers for Disease Control and Prevention	Anna Montmayeur; Anna Uehara; Ben L. Rambo-Martin; Clinton R. Paden; Dhwani Batra; Haibin Wang; Jasmine Padilla; Jing Zhang; Justin Lee; Katie Dillon; Krista Queen; Kristen Knipe; Kristine Lacek; Lori Rowe; Mark Burroughs; Matthew Schmerer; Mili Sheth; Peter W. Cook; Rachel Marine; Sam Shepard; Sarah Nobles; Shoshona Le; Suxiang Tong; Yan Li; Ying Tao
EPI_ISL_1225975 to 1225986, EPI_ISL_1226192 to 1226211	"CO Dept. of Public Health and Environment, Lab Services Division"	Genomics and Discovery, Respiratory Viruses Branch, Division of Viral Diseases, Centers for Disease Control and Prevention	Anna Montmayeur; Anna Uehara; Ben L. Rambo-Martin; Clinton R. Paden; Dhwani Batra; Haibin Wang; Jasmine Padilla; Jing Zhang; Justin Lee; Katie Dillon; Krista Queen; Kristen Knipe; Kristine Lacek; Lori Rowe; Mark Burroughs; Matthew Schmerer; Mili Sheth; Peter W. Cook; Rachel Marine; Sam Shepard; Sarah Nobles; Shoshona Le; Suxiang Tong; Yan Li; Ying Tao
EPI_ISL_1251018	"DIP. PREV. AVEZZANO SERVIZIO DI IGIENE EPIDEMIOLOGIA E SANITA' PUBBLICA	Istituto Zooprofilattico Sperimentale dell'Abruzzo e Molise "G. Caporale"	Ancora M; Calistri P; Cammà C; Curini V; Di Domenico M; Di Pasquale A; Lorusso A; Mangone I; Marcacci M; Puglia I; Rinaldi A; Savini G; Scialabba S
EPI_ISL_1167022 to 1167087, EPI_ISL_1191610	"Dr. Andrija Stampar" Teaching Institute of Public Health, Department of Clinical Microbiology	Istituto di Genomica Applicata	Davide Scaglione; Eleonora Paparelli; Fedrica Cattonaro; Gabriele Magris; Irena Jurman; Jasmina Vranes; Michele Morgante; Slobodanka Radovic; Vera Vendramin
EPI_ISL_1109549 to 1109552, EPI_ISL_1109555 to 1109557, EPI_ISL_1168769, EPI_ISL_1180208	"Dr. Andrija Stampar" Teaching Institute of Public Health	Croatian Institute of Public Health	Irena Tabain; Ivana Ferenak
EPI_ISL_985399 to 985400, EPI_ISL_985402, EPI_ISL_985405 to 985407, EPI_ISL_985410, EPI_ISL_985433, EPI_ISL_988352, EPI_ISL_989241, EPI_ISL_990198, EPI_ISL_991178, EPI_ISL_993056, EPI_ISL_993930, EPI_ISL_994654 to 994667, EPI_ISL_994739 to 994740, EPI_ISL_996002, EPI_ISL_996004, EPI_ISL_996028			
see above	"Dr. Andrija Stampar" Teaching Institute of Public Health, Department of Clinical Microbiology	Institute of Applied Genomics	Federica Cattonaro; Jasmina Vranes; Michele MOrgante; Michele Morgante
EPI_ISL_1302448, EPI_ISL_1302460, EPI_ISL_1302464, EPI_ISL_1302475 to 1302492, EPI_ISL_1302659 to 1302665	"InMedica"	Lithuanian University of Health Sciences Hospital, Department of Genetics and Molecular Medicine	Astra Vitkauskiene; Darius Cereskevicius; Inga Nasvytiene; Mantas Sarauskas; Marius Sukys; Rasa Ugenskiene; Renaldas Jurkevicius; Zivile Zemeckiene
EPI_ISL_1226035 to 1226038, EPI_ISL_1226341 to 1226343	"MN PHL Division, Minnesota Department of Health"	Genomics and Discovery, Respiratory Viruses Branch, Division of Viral Diseases, Centers for Disease Control and Prevention	Anna Montmayeur; Anna Uehara; Ben L. Rambo-Martin; Clinton R. Paden; Dhwani Batra; Haibin Wang; Jasmine Padilla; Jing Zhang; Justin Lee; Katie Dillon; Krista Queen; Kristen Knipe; Kristine Lacek; Lori Rowe; Mark Burroughs; Matthew Schmerer; Mili Sheth; Peter W. Cook; Rachel Marine; Sam Shepard; Sarah Nobles; Shoshona Le; Suxiang Tong; Yan Li; Ying Tao
EPI_ISL_1226281 to 1226287	"NM Dept. Health, Scientific Laboratory Division "	Genomics and Discovery, Respiratory Viruses Branch, Division of Viral Diseases, Centers for Disease Control and Prevention	Anna Montmayeur; Anna Uehara; Ben L. Rambo-Martin; Clinton R. Paden; Dhwani Batra; Haibin Wang; Jasmine Padilla; Jing Zhang; Justin Lee; Katie Dillon; Krista Queen; Kristen Knipe; Kristine Lacek; Lori Rowe; Mark Burroughs; Matthew Schmerer; Mili Sheth; Peter W. Cook; Rachel Marine; Sam Shepard; Sarah Nobles; Shoshona Le; Suxiang Tong; Yan Li; Ying Tao
EPI_ISL_1226003 to 1226006, EPI_ISL_1226048 to 1226081	"NYSDOH Wadsworth Center, Virology Lab"	Genomics and Discovery, Respiratory Viruses Branch, Division of Viral Diseases, Centers for Disease Control and Prevention	Anna Montmayeur; Anna Uehara; Ben L. Rambo-Martin; Clinton R. Paden; Dhwani Batra; Haibin Wang; Jasmine Padilla; Jing Zhang; Justin Lee; Katie Dillon; Krista Queen; Kristen Knipe; Kristine Lacek; Lori Rowe; Mark Burroughs; Matthew Schmerer; Mili Sheth; Peter W. Cook; Rachel Marine; Sam Shepard; Sarah Nobles; Shoshona Le; Suxiang Tong; Yan Li; Ying Tao
EPI_ISL_1226326 to 1226340	"OK Public Health Laboratory, Oklahoma State DOH"	Genomics and Discovery, Respiratory Viruses Branch, Division of Viral Diseases, Centers for Disease Control and Prevention	Anna Montmayeur; Anna Uehara; Ben L. Rambo-Martin; Clinton R. Paden; Dhwani Batra; Haibin Wang; Jasmine Padilla; Jing Zhang; Justin Lee; Katie Dillon; Krista Queen; Kristen Knipe; Kristine Lacek; Lori Rowe; Mark Burroughs; Matthew Schmerer; Mili Sheth; Peter W. Cook; Rachel Marine; Sam Shepard; Sarah Nobles; Shoshona Le; Suxiang Tong; Yan Li; Ying Tao
EPI_ISL_1167936 to 1167937, EPI_ISL_1167999 to 1168000, EPI_ISL_1226016 to 1226034, EPI_ISL_1226382 to 1226384	"PA Department of Health, Bureau of Laboratories"	Genomics and Discovery, Respiratory Viruses Branch, Division of Viral Diseases, Centers for Disease Control and Prevention	; Anna Montmayeur; Anna Uehara; Ben L. Rambo-Martin; Brian Lynch; Clinton R. Paden; Dhwani Batra; Haibin Wang; Jasmine Padilla; Jing Zhang; Justin Lee; Katie Dillon; Krista Queen; Kristen Knipe; Kristine Lacek; Lori Rowe; Mark Burroughs; Matthew Schmerer; Mili Sheth; Peter W. Cook; Rachel Marine; Sam Shepard; Sarah Nobles; Shoshona Le; Suxiang Tong; Yan Li; Ying Tao
EPI_ISL_833052 to 833054, EPI_ISL_1251078	"Presidio Ospedaliero "San Liberatore" Atri	Istituto Zooprofilattico Sperimentale dell'Abruzzo e Molise "G. Caporale"	Ancora M; Calistri P; Cammà C; Curini V; Delli Compagni E; Di Domenico M; Di Pasquale A; Lorusso A; Mangone I; Marcacci M; Puglia I; Rinaldi A; Savini G; Scialabba S
EPI_ISL_833055 to 833056	"SIESP DIPARTIMENTO DI PREVENZIONE CHIETi	Istituto Zooprofilattico Sperimentale dell'Abruzzo e Molise "G. Caporale"	Ancora M; Calistri P; Cammà C; Curini V; Delli Compagni E; Di Domenico M; Di Pasquale A; Lorusso A; Mangone I; Marcacci M; Puglia I; Rinaldi A; Savini G
EPI_ISL_1208949, EPI_ISL_1208951 to 1208952	"Scientific and Practical Center for Sanitary and Epidemiological Expertise and Monitoring" branch of the RSE on the REU "National Center for Public Health" of the Ministry of Health RK	Kazakh National Agrarian University (KazNAU); TreeGene LLP Genetic Laboratory; National Scientific Center for Especially Dangerous Infections (NSCEDI)	Beloussov Vyacheslav; Granica Joanna; Sandybayev Nurlan; Strochkov Vitaliy; Tabynov Kairat; Tabynov Kaissar; Turebekov Nurkeldy; Yerubayev Toktassyn; Yespolov Tlektes
EPI_ISL_1081954, EPI_ISL_1081958 to 1081961, EPI_ISL_1082252, EPI_ISL_1082269, EPI_ISL_1082290 to 1082291, EPI_ISL_1082294, EPI_ISL_1251274 to 1251277, EPI_ISL_1260862, EPI_ISL_1260871, EPI_ISL_1265368, EPI_ISL_1265371 to 1265373			
see above	"Stefan S. Nicolau"Institute of Virology	"Stefan S. Nicolau"Institute of Virology	Adriana Plesa; Alina Nastasie; Ana Iulia Neagu; Anca Botezatu; Camelia Sultana; Carmen Cristina Diaconu; Carmen Cristina Diaconu.; Coralia Bleotu; Cristina Mambet; Denisa Dragu; Gabriela Anton; Ioana Pitica; Iulia Virginia Iancu; Laura Grecu; Laura Necula; Lilia Matei; Marinela Bostan; Mihaela Economescu; Mirela Mihaila; Saviana Nedeianu; Simona Ruta
EPI_ISL_1081962, EPI_ISL_1082296	"Stefan S. Nicolau"Institute of Virology	285 Mihai Bravu Ave, Bucharest, Romania	Adriana Plesa; Alina Nastasie; Ana Iulia Neagu; Anca Botezatu; Camelia Sultana; Carmen Cristina Diaconu; Coralia Bleotu; Cristina Mambet; Denisa Dragu; Gabriela Anton; Ioana Pitica; Iulia Virginia Iancu; Laura Grecu; Laura Necula; Lilia Matei; Marinela Bostan; Mihaela Economescu; Mirela Mihaila;

			Saviana Nedeianu; Simona Ruta
EPI_ISL_1225931 to 1225935	"TX DSHS, Lab Services Section MC 1947"	Genomics and Discovery, Respiratory Viruses Branch, Division of Viral Diseases, Centers for Disease Control and Prevention	Anna Montmayeur; Anna Uehara; Ben L. Rambo-Martin; Clinton R. Paden; Dhwani Batra; Haibin Wang; Jasmine Padilla; Jing Zhang; Justin Lee; Katie Dillon; Krista Queen; Kristen Knipe; Kristine Lacek; Lori Rowe; Mark Burroughs; Matthew Schmerer; Mili Sheth; Peter W. Cook; Rachel Marine; Sam Shepard; Sarah Nobles; Shoshona Le; Suxiang Tong; Yan Li; Ying Tao
EPI_ISL_632310	1-Laboratory of Microbiology, National Reference Lab, Charles Nicolle Hospital; 2-University of Tunis ElManar, Faculty of Medicine of Tunis, LR99ES09, Tunis, Tunisia	1-Clinical and Experimental Pharmacology Lab, LR16SP02, National Center of Pharmacovigilance, University of Tunis El Manar, Tunis, Tunisia. 2-Neurodegenerative diseases and psychiatric troubles, LR18SP03, Razi Hospital, University of Tunis El Manar, Tunis, Tunisia. 3- Ministry of Health, National Observatory of New and Emerging Diseases, 1006, Tunis, Tunisia	Alia Ben Kahla; Gaies Emna; Ilhem Boutiba-Ben Boubaker; Imen Kacem; Imen Mkada; Jalila Ben Khelil; Maher Kharrat; Mouna Ben Sassi; Mouna Safer; Nissaf Ben Alaya; Riadh Daghfous; Riadh Gouider.; Salma Abid; Sameh Trabelsi; Sana Ferjani; Soumaya Rammeh
EPI_ISL_634977, EPI_ISL_635059 to 635062, EPI_ISL_683329, EPI_ISL_699655 to 699657, EPI_ISL_707697 to 707700, EPI_ISL_707791 to 707793, EPI_ISL_733499 to 733500, EPI_ISL_763065, EPI_ISL_763067, EPI_ISL_794735 to 794738			
see above	1-Laboratory of Microbiology, National Reference Lab, Charles Nicolle Hospital; 2-University of Tunis ElManar, Faculty of Medicine of Tunis, LR99ES09, Tunis, Tunisia	1-Clinical and Experimental Pharmacology Lab, LR16SP02, National Center of Pharmacovigilance, University of Tunis El Manar, Tunis, Tunisia. 2-Neurodegenerative diseases and psychiatric troubles, LR18SP03, Razi Hospital, University of Tunis El Manar, Tunis, Tunisia. 3- Ministry of Health, National Observatory of New and Emerging Diseases, 1006, Tunis, Tunisia	Alia Ben Kahla; Alia BenKahla; Asma Ferjani; Awatef El MOussi; Awatef El Moussi; Gaies Emna; Guedi Ali Barreh; Guedi Berrabeh; Habiba Ben Romdhane; Hanen El Jebari; Hanen ElJebari; Ilhem Boutiba-Ben Boubaker; Imen Kacem; Imen Mkada; Ines Mdini; Jalila Ben Khelil; Maher Kharrat; Mouna Ben Sassi; Mouna Safer; Nissaf Ben Alaya; Riadh Daghfous; Riadh Gouider.; Rouaa Ben Othman; Salma Abid; Salwa Mrabet; Sameh Trabelsi; Sana Ferjani; Sarra Chamman; Souissi Amira; Soumaya Rammeh; Zaineb Hamzaoui
EPI_ISL_451971 to 451987	1. ViroGenetics - BSL3 Laboratory of Virology, Maopolska Centre of Biotechnology, Jagiellonian University; 2. II Department of Internal Medicine, Faculty of Medicine, Jagiellonian University Medical College; 3. DIAGNOSTYKA Ltd.	1. ViroGenetics - BSL3 Laboratory of Virology, Maopolska Centre of Biotechnology, Jagiellonian University; 2. II Department of Internal Medicine, Faculty of Medicine, Jagiellonian University Medical College.	Jakub Swadba; Krzysztof Pyr; Marcin Surmiak; Marek Sanak; Marta Rogalska-Kupiec; Monika Gsecka-Czapla; Pawe P abaj; Wojciech Branicki
EPI_ISL_455440 to 455453, EPI_ISL_492066 to 492073	1. ViroGenetics - BSL3 Laboratory of Virology, Maopolska Centre of Biotechnology, Jagiellonian University; 2. II Department of Internal Medicine, Faculty of Medicine, Jagiellonian University Medical College; 3. Narodowy Instytut Zdrowia Publicznego - Pastwowy Zakad Higieny (NIZP-PZH)	1. ViroGenetics - BSL3 Laboratory of Virology, Maopolska Centre of Biotechnology, Jagiellonian University; 2. II Department of Internal Medicine, Faculty of Medicine, Jagiellonian University Medical College; 3. Narodowy Instytut Zdrowia Publicznego - Pastwowy Zakad Higieny (NIZP-PZH).	Agnieszka Koakowska-Kulesza; Aleksandra A. Zasada; Aleksandra Milewska; Ewelina Hallman-Szeliska; Katarzyna Owczarek; Katarzyna Pancer; Katarzyna Zacharczuk; Krzysztof Pyr; Magdalena Rzeczkowska; Marek Sanak; Natalia Wolaniuk; Pawe P abaj; Tomasz Wokowicz; Wojciech Branicki
EPI_ISL_1265393 to 1265440	1. Wojewódzka Stacja Sanitarno - Epidemiologiczna w Katowicach (WSSE Katowice); 2. Wojewódzka Stacja Sanitarno - Epidemiologiczna w Kielcach (WSSE Kielce); 3. Wojewódzka Stacja Sanitarno - Epidemiologiczna w Szczecinie (WSSE Szczecin); 4. Wojewódzka Stacja Sanitarno - Epidemiologiczna w Lublinie (WSSE Lublin); 5. Wojewódzka Stacja Sanitarno - Epidemiologiczna w Gorzowie Wielkopolskim (WSSE Gorzów Wielkopolski); 6. Wojewódzka Stacja Sanitarno-Epidemiologiczna w Gdasku (WSSE Gdask); 7. Wojewódzka Stacja Sanitarno-Epidemiologiczna w Poznaniu (WSSE Pozna); 8. Wojewódzka Stacja Sanitarno-Epidemiologiczna w Opolu (WSSE Opole); 9. Wojewódzka Stacja Sanitarno-Epidemiologiczna we Wrocawiu (WSSE Wrocaw); 10. Wojewódzka Stacja Sanitarno-Epidemiologiczna w Krakowie (WSSE Kraków); 11. Wojewódzka Stacja Sanitarno-Epidemiologiczna w Olsztynie (WSSE Olsztyn); 12. Wojewódzka Stacja Sanitarno-Epidemiologiczna w Bydgoszczy (WSSE Bydgoszcz); 13. Wojewódzka Stacja Sanitarno-Epidemiologiczna w Rzeszowie (WSSE Rzeszów); 14. Wojewódzka Stacja Sanitarno-Epidemiologiczna w Biaymstoku (WSSE Biaystok);	1. ViroGenetics - BSL3 Laboratory of Virology, Maopolska Centre of Biotechnology, Jagiellonian University; 2. Human Genome Variation Research Group, Malopolska Centre of Biotechnology, Jagiellonian University;	Branicki, W.; Gromowski, T.; Klajmon, A.; Kowalski, M.; Labaj; Marszalek, K.; Mazur-Panasiuk, N.; P.P.; Pyrc, K.; Szulc, P.
EPI_ISL_876042 to 876043, EPI_ISL_876969 to 877034, EPI_ISL_877036, EPI_ISL_882674 to 882768, EPI_ISL_883499 to 883500, EPI_ISL_887506 to 887507, EPI_ISL_888679 to 888682, EPI_ISL_894218, EPI_ISL_918128, EPI_ISL_918412 to 918422, EPI_ISL_918457 to 918460, EPI_ISL_918462 to 918482, EPI_ISL_925093 to 925136, EPI_ISL_925497 to 925501, EPI_ISL_934419 to 934423, EPI_ISL_935103 to 935154, EPI_ISL_954183 to 954225, EPI_ISL_1013523 to 1013582			
see above	1.AO Universitaria 'S. Giovanni di Dio e Ruggi D'Aragona, Scuola Medica Salernitana' Hospital / 2.UOC di Virologia e Microbiologia, Università della Campania 'L. Vanvitelli' / 3.AO Universitaria 'Federico II' Napoli Hospital / 4.AORN 'San Giuseppe Moscati' Avellino Hospital / 5.AO 'San Pio - presidio G. Rummo' Benevento Hospital / 6.AO 'Sant'Anna e San Sebastiano' Caserta Hospital / 7.PO 'Maria Santissima Addolorata' Eboli Hospital / 8.Biogem Istituto di Ricerche Genetiche	1. Genome Research Center for Health (CRGS) / 2. Laboratory of Molecular Medicine and Genomics(LMMGe) / 3. Center for Research in Pure and Applied Mathematics (CRMPA)	Alessandro Weisz; Alessandro Weisz (Corresponding Author); Alessia Cossu; Andreina Baj; Aniello Gentile; Annamaria Salvati; Antonello Saccomanno; Arnolfo Petruzziello; Assunta Sellitto; Carlo Ferravante; Domenico Memoli; Domenico Palumbo; Edmondo Adorisio; Elena Alexandrova; Emilia Vaccaro; Fausto Sessa.; Francesca Marciano; Francesca Rizzo; Francesca Rizzo (Corresponding Author); Francesco Curcio; Gianluigi Franci; Giorgio Dirani; Giorgio Giurato; Giorgio Giurato (Corresponding Author); Giovanni Nassa; Giovanni Pecoraro; Giuseppe Fenza; Giuseppe Portella; Gregorio Goffredi; Ilaria Terenzi; Jessica Lamberti; Maddalena Schioppa; Maria Grazia Foti; Maria Landi; Marianna Scrima; Mariarosaria Ingino; Massimiliano Galdiero; Maurizio Fumi; Michela Iacobellis; Michele Caraglia; Michele Cennamo; Morena D'Avenia; Oriana Strianese; Pasquale Pagliano; Rita Greco; Roberta Tarallo; Rosanna Piluscio; Silvia Zanoli; Simona Semprini; Sonia Amabile; Stefania Marzinotto; Teresa Rocco; Valeria Mirici Cappa; Vincenzo Rocco; Viola Melone; Vittoria Letizia; Vittorio Sambri; Ylenia D'Agostino
EPI_ISL_640056, EPI_ISL_640078, EPI_ISL_640089, EPI_ISL_640107, EPI_ISL_640115, EPI_ISL_640117, EPI_ISL_640127, EPI_ISL_700446, EPI_ISL_700555, EPI_ISL_1040736 to 1040737, EPI_ISL_1040741, EPI_ISL_1040743 to 1040744, EPI_ISL_1040769 to 1040773, EPI_ISL_1040790 to 1040792			
see above	2 Military Hospital wc MAA	NHLS/UCT	Arash Iranzadeh; Bruna Galvao; Carolyn Williamson; Deelan Doolabh; Diana Hardie; Innocent Mudau; Kruger Marais; Lynn Tyers; Marvin Hsiao; Stephen Korsman
EPI_ISL_960130, EPI_ISL_960151	2 Military Hospital wc MAA	National Health Laboratory Service/UCT	Arash Iranzadeh; Bruna Galvao; Carolyn Williamson; Deelan Doolabh; Diana Hardie; Innocent Mudau; Kruger Marais; Lynn Tyers; Marvin Hsiao; Stephen Korsman
EPI_ISL_450444	20 Dongda Street, Fengtai District, Beijing, Beijing 100071, China	Dept. OPA, Beijing Institute of Microbiology and Epidemiology	Fan, H.; Fang; Gong; J.H.; L.Q. and Liu, W.; Qi; R.Z.; X.A.; Zhang; Zheng, K.; Zheng, W.
EPI_ISL_1135044	21625 HJ PATHO OSS RHUMATO CLIMA PAV F	CNR Virus des Infections Respiratoires - France SUD	Antonin Bal; Bruno Lina; Gregory Destras; Gwendolyne Burfin; Hadrien Regue; Laurence Josset; Martine Valette; Quentin Semanas
EPI_ISL_1165901, EPI_ISL_1165903, EPI_ISL_1165908 to 1165910, EPI_ISL_1165914 to 1165918, EPI_ISL_1165933, EPI_ISL_1165946 to 1165949, EPI_ISL_1165954, EPI_ISL_1165968 to 1166009, EPI_ISL_1166054 to 1166081			
see above	24023 PLATEFORME MGI COVID	CNR Virus des Infections Respiratoires - France SUD	Antonin Bal; Bruno Lina; Gregory Destras; Gwendolyne Burfin; Hadrien Regue; Laurence Josset; Martine Valette; Quentin Semanas
EPI_ISL_1118064, EPI_ISL_1209365 to 1209368	3. Medizinische Abteilung, Hanusch Krankenhaus	Bergthaler laboratory, CeMM Research Center for Molecular Medicine of the Austrian Academy of Sciences	Andreas Bergthaler; Anna Schedl; Bekir Erguner; Benedikt Agerer; Christoph Bock; Fabian Amman; Jan Laine; Lukas Endler; Maelle Le Moing; Martin Senekowitsch; Michael Schuster; Thomas Penz
EPI_ISL_513348 to 513349, EPI_ISL_513402, EPI_ISL_513413, EPI_ISL_526186, EPI_ISL_593646 to 593648, EPI_ISL_767931, EPI_ISL_806723, EPI_ISL_806726 to 806727			
see above	4Cyte Pathology	NSW Health Pathology - Institute of Clinical Pathology and Medical Research; Westmead Hospital; University of Sydney	CIDM-PH et al.
EPI_ISL_729337 to 729345, EPI_ISL_729347, EPI_ISL_729349 to 729352, EPI_ISL_729354, EPI_ISL_729358 to 729359, EPI_ISL_729361 to 729364, EPI_ISL_729367 to 729369, EPI_ISL_729371 to 729376, EPI_ISL_729379 to 729381, EPI_ISL_729383 to 729386, EPI_ISL_729389 to 729397, EPI_ISL_729399, EPI_ISL_729401,			

EPI_ISL_729403, EPI_ISL_729407, EPI_ISL_729411, EPI_ISL_729414, EPI_ISL_729416 to 729467, EPI_ISL_729469 to 729472, EPI_ISL_729498, EPI_ISL_729500 to 729513, EPI_ISL_729515 to 729528, EPI_ISL_729535 to 729547, EPI_ISL_729549 to 729552, EPI_ISL_729562 to 729595, EPI_ISL_729608 to 729682, EPI_ISL_729684 to 729734, EPI_ISL_824742 to 824786, EPI_ISL_824864, EPI_ISL_849794 to 849866, EPI_ISL_869082, EPI_ISL_869084, EPI_ISL_869086 to 869117, EPI_ISL_869120 to 869123, EPI_ISL_869137 to 869142, EPI_ISL_909745 to 909746, EPI_ISL_909748 to 909749, EPI_ISL_909964 to 910010			
see above	A. Krumbholz, Labor Dr. Krause und Kollegen MVZ GmbH, Kiel	Charité Universitätsmedizin Berlin, Institut für Virologie	Barbara Mühlemann; Christian Drosten; Cornelia Schlee; Julia Schneider; Julia Tesch; Jörn Beheim-Schwarzbach; Talitha Veith; Terry Jones; Tobias Bleicker; Tomasz Zemojtel; Victor M Corman
EPI_ISL_1064693 to 1064694, EPI_ISL_1168051	A05	The Public Health Agency of Sweden	Anna Risberg; Anna-Malin Linde; Carlo Berg; Karin Tegmark-Wisell; Maria Lind Karlberg; Mattias Haukland; Mia Brytting; Noura Walai; Oskar Karlsson Lindsjo; Petra Edquist; Petra Holmstrom; Reza Advani; Samuel Ohman; Sofia Stamouli
EPI_ISL_766695, EPI_ISL_766698 to 766699, EPI_ISL_766708 to 766709, EPI_ISL_766715 to 766723	A05 Biomedicum	The Public Health Agency of Sweden	Department of Microbiology; The Public Health Agency of Sweden
EPI_ISL_1008401 to 1008411	A05 Diagnostics	The Public Health Agency of Sweden	Anna Risberg; Anna-Malin Linde; Carlo Berg; Karin Tegmark-Wisell; Maria Lind Karlberg; Mattias Haukland; Mia Brytting; Noura Walai; Oskar Karlsson Lindsjo; Petra Edquist; Petra Holmstrom; Reza Advani; Sofia Stamouli
EPI_ISL_1008534 to 1008539, EPI_ISL_1008601, EPI_ISL_1168099 to 1168106, EPI_ISL_1231917 to 1231918, EPI_ISL_1231944, EPI_ISL_1231957, EPI_ISL_1231969, EPI_ISL_1231978 to 1231980, EPI_ISL_1231991 to 1231993			
see above	A05 diagnostics	The Public Health Agency of Sweden	Anna Risberg; Anna-Malin Linde; Carlo Berg; Karin Tegmark-Wisell; Maria Lind Karlberg; Mattias Haukland; Mia Brytting; Noura Walai; Oskar Karlsson Lindsjo; Petra Edquist; Petra Holmstrom; Reza Advani; Sofia Stamouli
EPI_ISL_775533 to 775541, EPI_ISL_775550 to 775571, EPI_ISL_775575 to 775582, EPI_ISL_788991 to 788998, EPI_ISL_789000 to 789011, EPI_ISL_789014 to 789016, EPI_ISL_789018 to 789028, EPI_ISL_831944, EPI_ISL_831947 to 831952, EPI_ISL_831959, EPI_ISL_831982 to 831985			
see above	ABC	The Public Health Agency of Sweden	Department of Microbiology; The Public Health Agency of Sweden
EPI_ISL_913282, EPI_ISL_913287 to 913293, EPI_ISL_913316, EPI_ISL_913486	ABC Labs	The Public Health Agency of Sweden	Anna Risberg; Anna-Malin Linde; Carlo Berg; Karin Tegmark-Wisell; Maria Lind Karlberg; Mattias Haukland; Mia Brytting; Noura Walai; Oskar Karlsson Lindsjo; Petra Edquist; Petra Holmstrom; Reza Advani; Sofia Stamouli
EPI_ISL_1008586 to 1008588, EPI_ISL_1168069, EPI_ISL_1168136	ABC labs	The Public Health Agency of Sweden	Anna Risberg; Anna-Malin Linde; Carlo Berg; Karin Tegmark-Wisell; Maria Lind Karlberg; Mattias Haukland; Mia Brytting; Noura Walai; Oskar Karlsson Lindsjo; Petra Edquist; Petra Holmstrom; Reza Advani; Sofia Stamouli
EPI_ISL_455042 to 455043, EPI_ISL_455050, EPI_ISL_455074	ACT Pathology	NSW Health Pathology - Institute of Clinical Pathology and Medical Research; Westmead Hospital; University of Sydney	CIDM-PH et al.
EPI_ISL_498468 to 498548, EPI_ISL_735503, EPI_ISL_1170946 to 1170947, EPI_ISL_1208400 to 1208402	ACT Pathology	Schwessinger Lab	Ashley Jones; Benjamin Schwessinger; Craig Kennedy; Karina Kennedy; Kevin Murray; Megan McDonald; Ming-Dao Chia; Robert Lanfear; Robyn N Hall
EPI_ISL_427711, EPI_ISL_427717 to 427719, EPI_ISL_427721	ACT Pathology, The Canberra Hospital	NSW Health Pathology - Institute of Clinical Pathology and Medical Research; Westmead Hospital; University of Sydney	Arnott A; Bachmann N; Basile K; Byun R; Carter I; Chang S; Chen SC; Draper J; Dwyer DE for the 2019-nCoV Study Group; Eden JS; Gall M; Gray K; Holmes EC; Kok J; Lam C; Maddocks S; O'Sullivan MV; Propenko M; Rockett R; Sadsad R; Sim E; Sintchenko V; Sorrell T; Timms V
EPI_ISL_451540, EPI_ISL_451588, EPI_ISL_451590 to 451593, EPI_ISL_451595 to 451596, EPI_ISL_451598, EPI_ISL_451612	ACT pathology	NSW Health Pathology - Institute of Clinical Pathology and Medical Research; Westmead Hospital; University of Sydney	CIDM-PH et al.
EPI_ISL_830724 to 830725, EPI_ISL_853676 to 853682	ACUTIS DIAGNOSTICS	Wadsworth Center, New York State Department of Health	Alexis Russel; Daryl M. Lamson; Erasmus Schneider; Erica Lasek-Nesselquist; John Kelly; Jonathan Plitnick; Kirsten St. George; Matthew Shudt; Melissa A Leisner; Navjot Singh
EPI_ISL_861135 to 861143, EPI_ISL_883436 to 883444, EPI_ISL_883473, EPI_ISL_936046 to 936059, EPI_ISL_982499 to 982504, EPI_ISL_1227555, EPI_ISL_1228292			
see above	ADIRONDACK MEDICAL CENTER	Wadsworth Center, New York State Department of Health	Alexis Russel; Daryl M. Lamson; Erasmus Schneider; Erica Lasek-Nesselquist; John Kelly; Jonathan Plitnick; Kirsten St. George; Matthew Shudt; Melissa A Leisner; Navjot Singh
EPI_ISL_1007605 to 1007610, EPI_ISL_1120875	ADMED	Laboratory of genomics and metagenomics, Institute of Microbiology, University Hospital Centre and University of Lausanne, Switzerland	Claire Bertelli; Damien Jacot; Gilbert Greub; Sébastien Aeby; Trestan Pillonel
EPI_ISL_934405, EPI_ISL_934978 to 935011, EPI_ISL_1302788 to 1302819, EPI_ISL_1302838 to 1303030	ADMED Microbiologie	Genomics and Transcriptomics, Philip Morris International	David Bornand; Emmanuel Guedj; Manuel Peitsch; Marie-Lise Tritten; Maxime Berthouzoz; Mehdi Auberson; Nicolas Sierro; Nikolai Ivanov; Reto Lienhard; Rémi Dulize
EPI_ISL_602622 to 602631, EPI_ISL_605784	AHRI-Sigal	KRISP, KZN Research Innovation and Sequencing Platform	Cele S; Gazy I; Giandhari J; Karim F; Pillay S; Sigal A; Sigl A; Sigla; Tegally H; Wilkinson E; de Oliveira T
EPI_ISL_1229367 to 1229368	AHRI-Sigal	KRISP, KZn Research Innovation and Sequencing Platform	Cele S; Gazy I; Giandhari J; Karim F; Pillay S; Sigal A; Tegally H; Wilkinson E; de Oliveira T
EPI_ISL_1200655	AIDS Vaccine Research Laboratories	AIDS Vaccine Research Laboratories	AVRL et al.; Gage Moreno; Katarina Braun
EPI_ISL_763362 to 763364, EPI_ISL_768752 to 768797, EPI_ISL_768833	AIID	Irish Coronavirus Sequencing Consortium - National Virus Reference Laboratory	Alejandro Abner Garcia Leon; Gabriel Gonzalez; Michael Carr; Patrick Mallon
EPI_ISL_778819 to 778841, EPI_ISL_871793 to 871814, EPI_ISL_918434 to 918456, EPI_ISL_960986 to 961008	AIID	Irish Coronavirus Sequencing Consortium-Teagasc Grange	Aljandro Abner Garcia Leon; Calum Walsh; Fiona Crispie; Gabriel Gonzalez; John Kenny; Matthew McCabe; Michael Carr; Patrick Mallon; Paul Cotter
EPI_ISL_1272681 to 1272688	AK State Public Health Lab, State Health Department	Centers for Disease Control and Prevention Division of Viral Diseases, Pathogen Discovery	Anna Montmayeur; Anna Uehara; Ben L. Rambo-Martin; Clinton R. Paden; Dhwani Batra; Haibin Wang; Jasmine Padilla; Jing Zhang; Justin Lee; Katie Dillon; Krista Queen; Kristen Knipe; Kristine Lacek; Lori Rowe; Mark Burroughs; Matthew Schmerer; Mili Sheth; Peter W. Cook; Rachel Marine; Sam Shepard; Sarah Nobles; Shoshona Le; Suxiang Tong; Yan Li; Ying Tao
EPI_ISL_751592 to 751593, EPI_ISL_751597 to 751598, EPI_ISL_751694, EPI_ISL_751793	AK State Public Health Lab, State Health Department	Genomics and Discovery, Respiratory Viruses Branch, Division of Viral Diseases, Centers for Disease Control and Prevention	Anna Montmayeur; Anna Uehara; Clinton R. Paden; Haibin Wang; Jing Zhang; Justin Lee; Krista Queen; Mili Sheth; Peter W. Cook; Rachel Marine; Suxiang Tong; Yan Li; Ying Tao
EPI_ISL_1094238, EPI_ISL_1094272, EPI_ISL_1094319, EPI_ISL_1094353, EPI_ISL_1094366, EPI_ISL_1094380, EPI_ISL_1094707 to 1094719, EPI_ISL_1095227 to 1095232			
see above	AK State Public Health Lab, State Health Department	Respiratory Viruses Branch, Division of Viral Diseases, Centers for Disease Control and Prevention	Anna Montmayeur; Anna Uehara; Ben L. Rambo-Martin; Clinton R. Paden; Dhwani Batra; Haibin Wang; Jasmine Padilla; Jing Zhang; Justin Lee; Krista Queen; Lori Rowe; Mark Burroughs; Mili Sheth; Peter W. Cook; Rachel Marine; Sarah Nobles; Suxiang Tong; Yan Li; Ying Tao
EPI_ISL_1303431	AKH Wien, Klinik für Notfallmedizin, Notfallambulanz	Dept. of Laboratory Medicine	Harald Esterbauer; Oswald Wagner; Petra Jurkowitsch; Robert Strassl
EPI_ISL_751647, EPI_ISL_751668, EPI_ISL_751753, EPI_ISL_751755 to 751756, EPI_ISL_751791 to 751792, EPI_ISL_1168016	AL Dept. of Public Health Bureau of Clinical Laboratories	Genomics and Discovery, Respiratory Viruses Branch, Division of Viral Diseases, Centers for Disease Control and Prevention	; Anna Montmayeur; Anna Uehara; Brian Lynch; Clinton R. Paden; Haibin Wang; Jing Zhang; Justin Lee; Krista Queen; Mili Sheth; Peter W. Cook; Rachel Marine; Suxiang Tong; Yan Li; Ying Tao
EPI_ISL_1094888 to 1094898, EPI_ISL_1095262	AL Dept. of Public Health Bureau of Clinical Laboratories	Respiratory Viruses Branch, Division of Viral Diseases, Centers for Disease Control and Prevention	Anna Montmayeur; Anna Uehara; Ben L. Rambo-Martin; Clinton R. Paden; Dhwani Batra; Haibin Wang; Jasmine Padilla; Jing Zhang; Justin Lee; Krista Queen; Lori Rowe; Mark Burroughs; Mili Sheth; Peter W. Cook; Rachel Marine; Sarah Nobles; Suxiang Tong; Yan Li; Ying Tao
EPI_ISL_1156537, EPI_ISL_1156646, EPI_ISL_1156715, EPI_ISL_1211182, EPI_ISL_1216495, EPI_ISL_1216619, EPI_ISL_1216701			
see above	ALB FILS KLINIKEN GmbH - Institut für Laboratoriumsmedizin Klinik am Eichert Göppingen	Robert Koch Institute	
EPI_ISL_936180 to 936181, EPI_ISL_1226485, EPI_ISL_1226511, EPI_ISL_1226563, EPI_ISL_1226704 to 1226706, EPI_ISL_1226819, EPI_ISL_1227029, EPI_ISL_1227031 to 1227032, EPI_ISL_1227208 to 1227209, EPI_ISL_1227232 to 1227233, EPI_ISL_1227235, EPI_ISL_1227249 to 1227253, EPI_ISL_1227256, EPI_ISL_1227263 to 1227271, EPI_ISL_1227458 to 1227462, EPI_ISL_1227556, EPI_ISL_1227558, EPI_ISL_1227564 to 1227565, EPI_ISL_1227572 to 1227573, EPI_ISL_1227790, EPI_ISL_1228218 to 1228221, EPI_ISL_1228270, EPI_ISL_1228275 to 1228277, EPI_ISL_1228279, EPI_ISL_1228282 to 1228283, EPI_ISL_1228430, EPI_ISL_1228477 to 1228479			

see above	ALBANY MEDICAL CENTER	Wadsworth Center, New York State Department of Health	Alexis Russel; Daryl M. Lamson; Erasmus Schneider; Erica Lasek-Nesselquist; John Kelly; Jonathan Plitnick; Kirsten St. George; Matthew Shudt; Melissa A Leisner; Navjot Singh
EPI_ISL_830683 to 830719, EPI_ISL_853684 to 853687, EPI_ISL_853690 to 853704, EPI_ISL_853709 to 853711, EPI_ISL_884008 to 884012, EPI_ISL_884017 to 884031, EPI_ISL_884042 to 884046, EPI_ISL_884048 to 884053, EPI_ISL_884059 to 884079, EPI_ISL_884081 to 884085, EPI_ISL_1016417, EPI_ISL_1016468 to 1016475			
see above	ALBANY MEDICAL CENTER HOSPITAL CLINICAL LABORATORIES	Wadsworth Center, New York State Department of Health	Alexis Russel; Daryl M. Lamson; Erasmus Schneider; Erica Lasek-Nesselquist; John Kelly; Jonathan Plitnick; Kirsten St. George; Matthew Shudt; Melissa A Leisner; Navjot Singh
EPI_ISL_918407	ALEA Dr Kandic	Alea Genetic Centre	Dino Pecar; Lana Salihefendic; Rijad Konjhodzic
EPI_ISL_910329, EPI_ISL_910334	ALEA dr Kandic	Alea Genetic Centre	Dino Pecar; Lana Salihefendic; Rijad Konjhodzic
EPI_ISL_1073008, EPI_ISL_1073029	ALGEMEEN MEDISCH LABO	UAntwerp, Laboratory of Medical Microbiology	Basil Britto Xavier; Christine Lammens; Herman Goossens; Jasmine Coppens; Marie Le Mercier; Veerle Matheeussen
EPI_ISL_1112340	ALGEMEEN MEDISCH LABO	UAntwerp, Laboratory of Medical Microbiology,	Basil Britto Xavier; Christine Lammens; Herman Goossens; Jasmine Coppens; Marie Le Mercier; Veerle Matheeussen
EPI_ISL_471546	AMA DR Jose Soares Hungria	Instituto Adolfo Lutz, Interdiciplinary Procedures Center, Strategic Laboratory	Claudia Regina Gonçalves; Claudio Tavares Sacchi; Erica Valessa Ramos Gomes
EPI_ISL_861651	AMA Jardim Brasil	Instituto Adolfo Lutz, Interdiciplinary Procedures Center, Strategic Laboratory	Claudia Regina Gonçalves; Claudio Tavares Sacchi; Erica Valessa Ramos Gomes; Karoline Rodrigues Campos
EPI_ISL_523989	AMA Jardim Joamar	Instituto Adolfo Lutz, Interdiciplinary Procedures Center, Strategic Laboratory	Claudia Regina Gonçalves; Claudio Tavares Sacchi; Erica Valessa Ramos Gomes
EPI_ISL_523990	AMA Jardim Peri	Instituto Adolfo Lutz, Interdiciplinary Procedures Center, Strategic Laboratory	Claudia Regina Gonçalves; Claudio Tavares Sacchi; Erica Valessa Ramos Gomes
EPI_ISL_861652	AMA Wamberto Dias da Costa	Instituto Adolfo Lutz, Interdiciplinary Procedures Center, Strategic Laboratory	Claudia Regina Gonçalves; Claudio Tavares Sacchi; Erica Valessa Ramos Gomes; Karoline Rodrigues Campos
EPI_ISL_467432 to 467435, EPI_ISL_467449 to 467474	AMPATH-DBN	KRISP, KZN Research Innovation and Sequencing Platform	Chimukangara B; Giandhari J; Khan S; Lessells R; Mdlalose K; Pillay S; Tegally H; Wilkinson E; York D; de Oliveira T
EPI_ISL_1220051	ANALICEMOS LABORATORIO CLINICO ESPECIALIZADO	Instituto Nacional de Salud- Dirección de Investigación en Salud Pública	Carlos Franco-Muñoz; Diego A. Álvarez-Díaz; Diego Andrés Prada; Gerardo Santamaría; Hector Alejandro Ruiz-Moreno; Jhonnatan Reales-González; Julian Naizaque; Katherine Laiton-Donato; Magdalena Wiesner; Marcela Mercado-Reyes.; Maria T. Herrera-Sepúlveda; Martha Lucia Ospina Martínez; Sheryll Corchuelo
EPI_ISL_1220052	ANALICEMOS LABORATORIO ESPECIALIZADO	Instituto Nacional de Salud- Dirección de Investigación en Salud Pública	Carlos Franco-Muñoz; Diego A. Álvarez-Díaz; Diego Andrés Prada; Gerardo Santamaría; Hector Alejandro Ruiz-Moreno; Jhonnatan Reales-González; Julian Naizaque; Katherine Laiton-Donato; Magdalena Wiesner; Marcela Mercado-Reyes.; Maria T. Herrera-Sepúlveda; Martha Lucia Ospina Martínez; Sheryll Corchuelo
EPI_ISL_1048244 to 1048245, EPI_ISL_1048249, EPI_ISL_1048251, EPI_ISL_1048256 to 1048257, EPI_ISL_1048261, EPI_ISL_1048272 to 1048273, EPI_ISL_1048280, EPI_ISL_1048289, EPI_ISL_1048297, EPI_ISL_1048321 to 1048323			
see above	ANICON Labor, Landkreis Cloppenburg , Gesundheitsamt	Robert Koch Institute, Influenza and respiratory viruses FG17 & Bioinformatics MF1, Berlin, Germany	Aleksandar Radonic; J. Haneke; Marianne Wedde; Oliver Drechsel; R. Dürrwald; Ralf Duerrwald; Rene Kmiecinski; Stefan Kroeger; Stephan Fuchs; Thorsten Wolff
EPI_ISL_458150	ANOUAL	ANOUAL	Azami Nawfel; Benhida Rachid; Chenaoui Mohamed; El Aliani Aissam; El Ansari Fatima Zahra; Fekkak Jamal; Jouali Farah; Kasmi Yassine; Kitane Driss Lahlou; Loukman Salma; Marchoudi Nabila
EPI_ISL_1009028 to 1009031	AOU Policlinico Umberto I; Sapienza Università di Roma	INMI Lazzaro Spallanzani IRCCS	A Di Caro; B Bartolini; C.E.M Gruber; CEM Gruber; E Giombini; F Messina; G Antonelli; M Rueca; M. Rueca; MR Capobianchi; O Butera; O Turriziani
EPI_ISL_1272856 to 1272868	AR Dept. of Health-PHL, Molecular Diagnostics	Centers for Disease Control and Prevention Division of Viral Diseases, Pathogen Discovery	Anna Montmayeur; Anna Uehara; Ben L. Rambo-Martin; Clinton R. Paden; Dhwani Batra; Haibin Wang; Jasmine Padilla; Jing Zhang; Justin Lee; Katie Dillon; Krista Queen; Kristen Knipe; Kristine Lacek; Lori Rowe; Mark Burroughs; Matthew Schmerer; Mili Sheth; Peter W. Cook; Rachel Marine; Sam Shepard; Sarah Nobles; Shoshona Le; Suxiang Tong; Yan Li; Ying Tao
EPI_ISL_955272, EPI_ISL_955313, EPI_ISL_955315, EPI_ISL_955331 to 955333	AR Dept. of Health-PHL, Molecular Diagnostics	Pathogen Discovery, Respiratory Viruses Branch, Division of Viral Diseases, Centers for Disease Control and Prevention	Anna Uehara; Clinton R. Paden; Haibin Wang; Jing Zhang; Krista Queen; Peter Cook; Suxiang Tong; Yan Li; Ying Tao
EPI_ISL_751574, EPI_ISL_751652, EPI_ISL_751697 to 751699, EPI_ISL_751723, EPI_ISL_751731, EPI_ISL_751760, EPI_ISL_751764 to 751765, EPI_ISL_903565, EPI_ISL_903697			
see above	AR Dept. of Health-Public Health Lab	Genomics and Discovery, Respiratory Viruses Branch, Division of Viral Diseases, Centers for Disease Control and Prevention	Anna Montmayeur; Anna Uehara; Ben L. Rambo-Martin; Clinton R. Paden; Dhwani Batra; Haibin Wang; Jasmine Padilla; Jing Zhang; Justin Lee; Krista Queen; Lori Rowe; Mark Burroughs; Mili Sheth; Peter W. Cook; Rachel Marine; Sarah Nobles; Suxiang Tong; Yan Li; Ying Tao
EPI_ISL_450800, EPI_ISL_509663 to 509668, EPI_ISL_509670 to 509685, EPI_ISL_527632 to 527657, EPI_ISL_535650 to 535661, EPI_ISL_576196 to 576197	AR Dept. of Health-Public Health Lab	Pathogen Discovery, Respiratory Viruses Branch, Division of Viral Diseases, Centers for Disease Control and Prevention	Anna Montmayeur; Anna Uehara; Bettina Bankamp; Brian Lynch; Clinton Paden; Clinton R. Paden; Haibin Wang; Jing Zhang; Krista Queen; Peter Cook; Rachel Marine; Suxiang Tong; Yan Li; Ying Tao; Zachary Weiner
EPI_ISL_1094208, EPI_ISL_1094308, EPI_ISL_1094318, EPI_ISL_1094814 to 1094818, EPI_ISL_1094821, EPI_ISL_1094824, EPI_ISL_1094826, EPI_ISL_1094830, EPI_ISL_1095249 to 1095253			
see above	AR Dept. of Health-Public Health Lab	Respiratory Viruses Branch, Division of Viral Diseases, Centers for Disease Control and Prevention	Anna Montmayeur; Anna Uehara; Ben L. Rambo-Martin; Clinton R. Paden; Dhwani Batra; Haibin Wang; Jasmine Padilla; Jing Zhang; Justin Lee; Krista Queen; Lori Rowe; Mark Burroughs; Mili Sheth; Peter W. Cook; Rachel Marine; Sarah Nobles; Suxiang Tong; Yan Li; Ying Tao
EPI_ISL_1120855	ARC LEMANIQUE SA	Laboratory of genomics and metagenomics, Institute of Microbiology, University Hospital Centre and University of Lausanne, Switzerland	Claire Bertelli; Damien Jacot; Gilbert Greub; Sébastien Aeby; Trestan Pillonel
EPI_ISL_682235, EPI_ISL_682258 to 682259, EPI_ISL_1067609	AREA DE SALUD ALAJUELA NORTE - CLINICA DR. MARCIAL RODRIGUEZ	Inciensa, Instituto Costarricense de Investigación y Enseñanza en Nutrición y Salud	Adriana Godinez; Adriana Godinez & Melany Calderon; Adriana Godínez; Claudio Soto-Garita; Estela Cordero; Francisco Duarte; Hebleen Porras; Melany Calderon & Mariel López; Melany Calderón & Mariel López
EPI_ISL_914828	AREA DE SALUD ALAJUELA SUR	Inciensa, Instituto Costarricense de Investigación y Enseñanza en Nutrición y Salud	Adriana Godínez; Claudio Soto-Garita; Estela Cordero; Francisco Duarte; Hebleen Porras; Melany Calderón & Mariel López
EPI_ISL_1067606	AREA DE SALUD ALAJUELITA	Inciensa, Instituto Costarricense de Investigación y Enseñanza en Nutrición y Salud	Adriana Godínez; Claudio Soto-Garita; Estela Cordero; Francisco Duarte; Hebleen Porras; Melany Calderón & Melissa Carazo-Gutiérrez
EPI_ISL_914794, EPI_ISL_1067588, EPI_ISL_1067610	AREA DE SALUD BUENOS AIRES	Inciensa, Instituto Costarricense de Investigación y Enseñanza en Nutrición y Salud	Adriana Godínez; Claudio Soto-Garita; Estela Cordero; Francisco Duarte; Hebleen Porras; Melany Calderón & Mariel López; Melany Calderón & Mónica Charpentier-Artavia
EPI_ISL_914830 to 914831	AREA DE SALUD CARRILLO	Inciensa, Instituto Costarricense de Investigación y Enseñanza en Nutrición y Salud	Adriana Godínez; Claudio Soto-Garita; Estela Cordero; Francisco Duarte; Hebleen Porras; Melany Calderón & Adriana Bermúdez-Espinoza
EPI_ISL_914798, EPI_ISL_914805	AREA DE SALUD CARTAGO	Inciensa, Instituto Costarricense de Investigación y Enseñanza en Nutrición y Salud	Adriana Godínez; Claudio Soto-Garita; Estela Cordero; Francisco Duarte; Hebleen Porras; Melany Calderón & Mariel López; Melany Calderón & Mónica Charpentier-Artavia
EPI_ISL_682260, EPI_ISL_914802, EPI_ISL_1067587, EPI_ISL_1067598	AREA DE SALUD CATEDRAL NORESTE	Inciensa, Instituto Costarricense de Investigación y Enseñanza en Nutrición y Salud	Adriana Godinez; Adriana Godínez; Claudio Soto-Garita; Estela Cordero; Francisco Duarte; Hebleen Porras; Melany Calderon & Mariel López; Melany Calderón & Mariel López
EPI_ISL_682247 to 682248	AREA DE SALUD CIUDAD QUESADA	Inciensa, Instituto Costarricense de Investigación y Enseñanza en Nutrición y Salud	Adriana Godinez; Adriana Godinez & Melany Calderon; Claudio Soto-Garita; Estela Cordero; Francisco Duarte; Hebleen Porras; Melany Calderon & Mariel López
EPI_ISL_914806	AREA DE SALUD CORONADO	Inciensa, Instituto Costarricense de Investigación y	Adriana Godínez & Melany Calderón; Claudio Soto-Garita; Estela Cordero; Francisco Duarte; Hebleen Porras

		Enseñanza en Nutrición y Salud	
EPI_ISL_1067618, EPI_ISL_1196438	AREA DE SALUD CORRALILLO	Inciensa, Instituto Costarricense de Investigación y Enseñanza en Nutrición y Salud	Adriana Godínez; Caterina Guzmán; Claudio Soto-Garita; Estela Cordero; Francisco Duarte; Hebleen Porras; Melany Calderón; Melany Calderón & Mónica Charpentier-Artavia; Nazareth Ruiz & Mónica Charpentier-Artavia
EPI_ISL_682237, EPI_ISL_682256, EPI_ISL_1196421 to 1196422	AREA DE SALUD CORREDORES	Inciensa, Instituto Costarricense de Investigación y Enseñanza en Nutrición y Salud	Adriana Godinez; Adriana Godinez & Melany Calderon; Adriana Godínez; Claudio Soto-Garita; Estela Cordero; Francisco Duarte; Hebleen Porras; Melany Calderon & Mariel López; Melany Calderón & Mariel López
EPI_ISL_1201437, EPI_ISL_1201440	AREA DE SALUD COTO BRUS	Inciensa, Instituto Costarricense de Investigación y Enseñanza en Nutrición y Salud	Adriana Godínez; Claudio Soto-Garita; Estela Cordero; Francisco Duarte; Hebleen Porras; Melany Calderón & Mariel López; Melany Calderón & Mónica Charpentier-Artavia
EPI_ISL_1067597, EPI_ISL_1196424	AREA DE SALUD DESAMPARADOS 1 - CLINICA DR. MARCIAL FALLAS	Inciensa, Instituto Costarricense de Investigación y Enseñanza en Nutrición y Salud	Adriana Godínez; Claudio Soto-Garita; Estela Cordero; Francisco Duarte; Hebleen Porras; Melany Calderón & Daniel Cascante-Serrano; Melany Calderón & Mariel López
EPI_ISL_682250 to 682252	AREA DE SALUD ESCAZU (COOPESANA)	Inciensa, Instituto Costarricense de Investigación y Enseñanza en Nutrición y Salud	Adriana Godinez & Melany Calderon; Claudio Soto-Garita; Estela Cordero; Francisco Duarte; Hebleen Porras
EPI_ISL_682240	AREA DE SALUD GOICOCHEA 1	Inciensa, Instituto Costarricense de Investigación y Enseñanza en Nutrición y Salud	Adriana Godinez & Melany Calderon; Claudio Soto-Garita; Estela Cordero; Francisco Duarte; Hebleen Porras
EPI_ISL_914811 to 914812, EPI_ISL_1067591, EPI_ISL_1067603, EPI_ISL_1067616 to 1067617	AREA DE SALUD GOICOECHEA 2 - CLINICA DR. JIMENEZ NUÑEZ	Inciensa, Instituto Costarricense de Investigación y Enseñanza en Nutrición y Salud	Adriana Godínez; Claudio Soto-Garita; Estela Cordero; Francisco Duarte; Hebleen Porras; Melany Calderón & Mariel López
EPI_ISL_1067605	AREA DE SALUD HATILLO - CLINICA DR. SOLON NUÑEZ	Inciensa, Instituto Costarricense de Investigación y Enseñanza en Nutrición y Salud	Adriana Godínez; Claudio Soto-Garita; Estela Cordero; Francisco Duarte; Hebleen Porras; Melany Calderón & Daniel Cascante-Serrano
EPI_ISL_682238, EPI_ISL_682244, EPI_ISL_1067621	AREA DE SALUD LA CRUZ	Inciensa, Instituto Costarricense de Investigación y Enseñanza en Nutrición y Salud	Adriana Godinez; Adriana Godinez & Melany Calderon; Adriana Godínez; Caterina Guzmán; Claudio Soto-Garita; Estela Cordero; Francisco Duarte; Hebleen Porras; Melany Calderon & Mariel López; Melany Calderón; Nazareth Ruiz & Ivanna Krize-Morún
EPI_ISL_682249, EPI_ISL_914803	AREA DE SALUD LA UNION	Inciensa, Instituto Costarricense de Investigación y Enseñanza en Nutrición y Salud	Adriana Godinez; Adriana Godínez; Claudio Soto-Garita; Estela Cordero; Francisco Duarte; Hebleen Porras; Melany Calderon & Mariel López; Melany Calderón & Mónica Charpentier-Artavia
EPI_ISL_682236	AREA DE SALUD LOS CHILES	Inciensa, Instituto Costarricense de Investigación y Enseñanza en Nutrición y Salud	Adriana Godinez; Claudio Soto-Garita; Estela Cordero; Francisco Duarte; Hebleen Porras; Melany Calderon & Mariel López
EPI_ISL_1196423	AREA DE SALUD MATA REDONDA-HOSPITAL - CLINICA DR. MORENO CAÑAS	Inciensa, Instituto Costarricense de Investigación y Enseñanza en Nutrición y Salud	Adriana Godínez; Claudio Soto-Garita; Estela Cordero; Francisco Duarte; Hebleen Porras; Melany Calderón & Daniel Cascante-Serrano
EPI_ISL_1067619	AREA DE SALUD MATA REDONDA-HOSPITAL - CLINICA DR. MORENO CAÑAS [SAN JOSE/SAN JOSE]	Inciensa, Instituto Costarricense de Investigación y Enseñanza en Nutrición y Salud	Adriana Godínez; Caterina Guzmán; Claudio Soto-Garita; Estela Cordero; Francisco Duarte; Hebleen Porras; Melany Calderón; Nazareth Ruiz & Nicole Vargas-Víquez
EPI_ISL_914825	AREA DE SALUD MORAVIA	Inciensa, Instituto Costarricense de Investigación y Enseñanza en Nutrición y Salud	Adriana Godínez; Claudio Soto-Garita; Estela Cordero; Francisco Duarte; Hebleen Porras; Melany Calderón & Mariel López
EPI_ISL_914809	AREA DE SALUD OREAMUNO-PACAYAS-TIERRA BLANCA	Inciensa, Instituto Costarricense de Investigación y Enseñanza en Nutrición y Salud	Adriana Godínez; Claudio Soto-Garita; Estela Cordero; Francisco Duarte; Hebleen Porras; Melany Calderón & Mariel López
EPI_ISL_914835	AREA DE SALUD PARAISO-CERVANTES	Inciensa, Instituto Costarricense de Investigación y Enseñanza en Nutrición y Salud	Adriana Godínez; Claudio Soto-Garita; Estela Cordero; Francisco Duarte; Hebleen Porras; Melany Calderón & Mariel López
EPI_ISL_1067622, EPI_ISL_1201439	AREA DE SALUD PAVAS (COOPESALUD)	Inciensa, Instituto Costarricense de Investigación y Enseñanza en Nutrición y Salud	Adriana Godínez; Caterina Guzmán; Claudio Soto-Garita; Estela Cordero; Francisco Duarte; Hebleen Porras; Melany Calderón; Melany Calderón & Melissa Carazo-Gutiérrez; Nazareth Ruiz & Melissa Carazo-Gutiérrez
EPI_ISL_914799 to 914800	AREA DE SALUD SAN FRANCISCO-SAN ANTONIO (COOPESANA)	Inciensa, Instituto Costarricense de Investigación y Enseñanza en Nutrición y Salud	Adriana Godínez; Claudio Soto-Garita; Estela Cordero; Francisco Duarte; Hebleen Porras; Melany Calderón & Mariel López
EPI_ISL_914810, EPI_ISL_914820, EPI_ISL_1196425	AREA DE SALUD SAN JUAN-SAN DIEGO-CONCEPCION 2	Inciensa, Instituto Costarricense de Investigación y Enseñanza en Nutrición y Salud	Adriana Godínez; Adriana Godínez & Melany Calderón; Claudio Soto-Garita; Estela Cordero; Francisco Duarte; Hebleen Porras; Melany Calderón & Mariel López
EPI_ISL_914804, EPI_ISL_1067599, EPI_ISL_1196429	AREA DE SALUD TURRIALBA-JIMENEZ	Inciensa, Instituto Costarricense de Investigación y Enseñanza en Nutrición y Salud	Adriana Godínez; Claudio Soto-Garita; Estela Cordero; Francisco Duarte; Hebleen Porras; Melany Calderón & Mónica Charpentier-Artavia
EPI_ISL_1067607, EPI_ISL_1067612	AREA DE SALUD ZAPOTE-CATEDRAL - CLINICA DR. CARLOS DURAN	Inciensa, Instituto Costarricense de Investigación y Enseñanza en Nutrición y Salud	Adriana Godínez; Caterina Guzmán; Claudio Soto-Garita; Estela Cordero; Francisco Duarte; Hebleen Porras; Melany Calderón; Nazareth Ruiz & Melissa Carazo-Gutiérrez; Nazareth Ruiz & Nicole Vargas-Víquez
EPI_ISL_418000 to 418001, EPI_ISL_418004	ARS Algarve - Laboratório Laura Ayres	Instituto Nacional de Saude (INSA)	Guiomar et al
EPI_ISL_911548	ARUP laboratories	ARUP Laboratories	Barker AP; Hillyard DR; Hymas W; Mallory MA; Pyne MT; Shakir SM; Simmon KE; Slechta ES
EPI_ISL_445326, EPI_ISL_445363, EPI_ISL_445367	ASISTENCIA PUBLICA DR.ALEJANDRO DEL RIO	Instituto de Salud Publica de Chile	Alejandra Acevedo; Andrés E Castillo; Bárbara Parra; Carolina Tambley; Gabriel Leal; Jaime Lagos; Jorge Fernandez; Loredana Arata; Patricia Bustos; Paz Tapia; Rodrigo Fasce; Winston Andrade
EPI_ISL_1036211	ASL AVEZZANO-SULMONA-L'AQUILA	Istituto Zooprofilattico Sperimentale dell'Abruzzo e Molise "G. Caporale"	Ancora M; Calistri P; Cammà C; Curini V; Di Domenico M; Di Pasquale A; Lorusso A; Mangone I; Marcacci M; Puglia I; Rinaldi A; Savini G; Scialabba S
EPI_ISL_1036198	ASL AVEZZANO-SULMONA-L'AQUILA DIP PREV SERV IGIENE E SAN PUBBLICA	Istituto Zooprofilattico Sperimentale dell'Abruzzo e Molise "G. Caporale"	Ancora M; Calistri P; Cammà C; Curini V; Di Domenico M; Di Pasquale A; Lorusso A; Mangone I; Marcacci M; Puglia I; Rinaldi A; Savini G; Scialabba S
EPI_ISL_983322	ASL LATINA PRESIDIO OSPEDALIERO NORD	INMI Lazzaro Spallanzani IRCCS	A Di Caro; A Lucci; B Bartolini; C.E.M Gruber; E Giombini; F Messina; M. Rueca; MR Capobianchi; O Butera; S Pignalosa
EPI_ISL_1073034, EPI_ISL_1074622 to 1074623, EPI_ISL_1080605 to 1080679, EPI_ISL_1080681 to 1080745, EPI_ISL_1080748, EPI_ISL_1082454 to 1082525, EPI_ISL_1085025 to 1085030, EPI_ISL_1085032 to 1085090, EPI_ISL_1085096 to 1085160, EPI_ISL_1085176 to 1085229, EPI_ISL_1087353 to 1087381, EPI_ISL_1087383 to 1087413, EPI_ISL_1088008 to 1088076, EPI_ISL_1096349 to 1096981, EPI_ISL_1157894 to 1158030, EPI_ISL_1166221 to 1166223, EPI_ISL_1166225 to 1166229, EPI_ISL_1166231 to 1166233, EPI_ISL_1166238 to 1166242, EPI_ISL_1166245, EPI_ISL_1166247, EPI_ISL_1166249, EPI_ISL_1166251 to 1166253, EPI_ISL_1166255 to 1166256, EPI_ISL_1166261, EPI_ISL_1166263 to 1166265, EPI_ISL_1166267 to 1166269, EPI_ISL_1166271 to 1166273, EPI_ISL_1166275 to 1166277, EPI_ISL_1166279 to 1166287, EPI_ISL_1166289 to 1166291, EPI_ISL_1166293 to 1166294, EPI_ISL_1166296 to 1166308, EPI_ISL_1166311 to 1166314, EPI_ISL_1166316, EPI_ISL_1166318 to 1166320, EPI_ISL_1166322 to 1166336, EPI_ISL_1166338 to 1166351, EPI_ISL_1166353 to 1166354, EPI_ISL_1166356, EPI_ISL_1166358 to 1166359, EPI_ISL_1166361 to 1166364, EPI_ISL_1166366 to 1166368, EPI_ISL_1166371, EPI_ISL_1166373 to 1166377, EPI_ISL_1166379 to 1166380, EPI_ISL_1166382 to 1166384, EPI_ISL_1166386, EPI_ISL_1166389 to 1166391, EPI_ISL_1166394, EPI_ISL_1166397 to 1166413, EPI_ISL_1166415 to 1166438, EPI_ISL_1166440, EPI_ISL_1166443 to 1166444, EPI_ISL_1166446 to 1166447, EPI_ISL_1166449 to 1166451, EPI_ISL_1166453 to 1166472, EPI_ISL_1166474 to 1166507, EPI_ISL_1166509 to 1166533, EPI_ISL_1167308 to 1167546, EPI_ISL_1169061 to 1169482, EPI_ISL_1219040 to 1219131, EPI_ISL_1229179 to 1229366, EPI_ISL_1229535 to 1229938, EPI_ISL_1298493 to 1299194			
see above	ASL Napoli 1 Centro	AMES Centro Polidiagnostico Strumentale S.r.l.	"Giovanni Savarese; Antonella Di Carlo; Antonio Fico; Antonio Fico"; Eloisa Evangelista; Giovanni Savarese; Luigi D'Amore; Luisa Circelli; Maurizio D'Amora; Monica Ianniello; Nadia Petrillo; Raffaella Ruggiero; Roberto Sirica
EPI_ISL_542098 to 542277, EPI_ISL_542400 to 542443	ASST GOM Niguarda	Dep. Of Oncology and Hemato-Oncology University of Milan	Antonio Piralla; Carlo Federico Perno; Chiara Vismara; Claudia Alteri; Elisa Matarazzo; Fausto Baldanti; Federica Giardina; Federica Novazzi; Luna Colagrossi; Maria Antonello; Massimo Puoti; Monica Tallarita; Oscar Massimiliano Epis; Roberto Fumagalli; Silvia Renica; Stefano Gaiarsa; Valentino Costabile; Valeria Cento
EPI_ISL_1312836 to 1312837, EPI_ISL_1312881 to 1312882, EPI_ISL_1312891 to 1312893, EPI_ISL_1312897 to 1312899	ASTRALAB	CNR Virus des Infections Respiratoires - France SUD	Antonin Bal; Bruno Lina; Gregory Destras; Gwendolyne Burfin; Hadrien Regue; Laurence Josset; Martine Valette; Quentin Semanas
EPI_ISL_1229105 to 1229113	ASTRALAB LIMOGES	CNR Virus des Infections Respiratoires - France SUD	Antonin Bal; Bruno Lina; Gregory Destras; Gwendolyne Burfin; Hadrien Regue; Laurence Josset; Martine Valette; Quentin Semanas
EPI_ISL_900506, EPI_ISL_1135057, EPI_ISL_1135060, EPI_ISL_1155709, EPI_ISL_1155711, EPI_ISL_1155713, EPI_ISL_1190786, EPI_ISL_1313121 to 1313122, EPI_ISL_1314037			
see above	AX BIO OCEAN	CNR Virus des Infections Respiratoires - France SUD	Antonin Bal; Bruno Lina; Gregory Destras; Gwendolyne Burfin; Hadrien Regue; Hadrien Règue; Laurence Josset; Martine Valette; Quentin Semanas

EPI_ISL_1265683, EPI_ISL_1290839 to 1290840	AXBIO	CNR Virus des Infections Respiratoires - France SUD	Antonin Bal; Bruno Lina; Gregory Destras; Gwendolyne Burfin; Hadrien Regue; Laurence Josset; Martine Valette; Quentin Semanas
EPI_ISL_891249 to 891250, EPI_ISL_909953 to 909954, EPI_ISL_909963, EPI_ISL_910011 to 910013, EPI_ISL_910015	AZ Delta	AZ Delta	Brigitte Maes; Dieter De Smet; Geert Marten; Geert Martens
EPI_ISL_420784	AZ Department of Health Services	Pathogen Discovery, Respiratory Viruses Branch, Division of Viral Diseases, Centers for Disease Control and Prevention	Alison S. Laufer Halpin; Anne Uehara; Christopher A. Elkins; Clinton R. Paden; Haibin Wang; Jasmine Padilla; Jing Zhang; Justin Lee; Krista Queen; Mary S. Keckler; Rachel Marine; Suxiang Tong; Yan Li; Ying Tao
EPI_ISL_981006, EPI_ISL_981370 to 981371, EPI_ISL_981373, EPI_ISL_981376, EPI_ISL_1018110, EPI_ISL_1018112, EPI_ISL_1018114, EPI_ISL_1018116, EPI_ISL_1020018 to 1020020, EPI_ISL_1020127, EPI_ISL_1020129 to 1020130, EPI_ISL_1020133, EPI_ISL_1020136 to 1020137, EPI_ISL_1020139, EPI_ISL_1020142, EPI_ISL_1020144, EPI_ISL_1020147 to 1020150, EPI_ISL_1020152 to 1020153, EPI_ISL_1020155 to 1020159, EPI_ISL_1020171 to 1020174, EPI_ISL_1020207, EPI_ISL_1020285 to 1020287, EPI_ISL_1020289 to 1020293, EPI_ISL_1103561, EPI_ISL_1103563, EPI_ISL_1103573, EPI_ISL_1103575, EPI_ISL_1103577 to 1103578, EPI_ISL_1103580 to 1103581, EPI_ISL_1103583, EPI_ISL_1105178, EPI_ISL_1108017, EPI_ISL_1108255, EPI_ISL_1108503, EPI_ISL_1108770, EPI_ISL_1109036, EPI_ISL_1109283, EPI_ISL_1109392 to 1109393, EPI_ISL_1109483, EPI_ISL_1109631, EPI_ISL_1176301 to 1176302, EPI_ISL_1176304, EPI_ISL_1176306, EPI_ISL_1176308 to 1176310, EPI_ISL_1176312, EPI_ISL_1176314 to 1176315, EPI_ISL_1176317, EPI_ISL_1176319, EPI_ISL_1176321 to 1176332			
see above	AZ Klina	AZ Klina	Dr. C. Vael; Dr.C. Vael
EPI_ISL_1299454 to 1299455	AZ Rivierenland, Campus Rumst	UAntwerp, Laboratory of Medical Microbiology	Basil Britto Xavier; Christine Lammens; Herman Goossens; Jasmine Coppens; Marie Le Mercier; Veerle Matheeussen
EPI_ISL_424848 to 424849, EPI_ISL_452118	AZ SPHL, Arizona Department of Health Services	Pathogen Discovery, Respiratory Viruses Branch, Division of Viral Diseases, Centers for Disease Control and Prevention	Alison S. Laufer Halpin; Anna Montmayeur; Anna Uehara; Christopher A. Elkins; Clinton R. Paden; Haibin Wang; Jing Zhang; Krista Queen; Mary S. Keckler; Rachel Marine; Suxiang Tong; Yan Li; Ying Tao; Zachary Weiner
EPI_ISL_1094327, EPI_ISL_1094346, EPI_ISL_1094984 to 1094992, EPI_ISL_1095270 to 1095275	AZ SPHL, Arizona Department of Health Services	Respiratory Viruses Branch, Division of Viral Diseases, Centers for Disease Control and Prevention	Anna Montmayeur; Anna Uehara; Ben L. Rambo-Martin; Clinton R. Paden; Dhwani Batra; Haibin Wang; Jasmine Padilla; Jing Zhang; Justin Lee; Krista Queen; Lori Rowe; Mark Burroughs; Mili Sheth; Peter W. Cook; Rachel Marine; Sarah Nobles; Suxiang Tong; Yan Li; Ying Tao
EPI_ISL_426483, EPI_ISL_426485, EPI_ISL_426512 to 426519, EPI_ISL_426527 to 426531, EPI_ISL_426537 to 426555, EPI_ISL_426558 to 426569, EPI_ISL_427271 to 427272, EPI_ISL_694049, EPI_ISL_694053 to 694138, EPI_ISL_694148 to 694218, EPI_ISL_694248 to 694315, EPI_ISL_694405 to 694427, EPI_ISL_694458 to 694600, EPI_ISL_694724 to 694796, EPI_ISL_694799 to 694844, EPI_ISL_694885 to 694950, EPI_ISL_695103 to 695228, EPI_ISL_695316 to 695371, EPI_ISL_695393 to 695520, EPI_ISL_695563 to 695613, EPI_ISL_695642 to 695655, EPI_ISL_695665 to 695701, EPI_ISL_695712 to 695752, EPI_ISL_695830 to 695839			
see above	AZ SPHL, Arizona Department of Health Services	TGen North	Ashlyn Pfeiffer; Chris French; Darrin Lemmer; Dave Engelthaler; Hayley Yaglom; Jolene Bowers; Megan Folkerts; The Arizona COVID Genomics Union (ACGU)
EPI_ISL_1265144 to 1265148, EPI_ISL_1265152 to 1265155, EPI_ISL_1265157 to 1265160	AZ Sint-Jan Brugge-Oostende AV	AZ SINT-JAN BRUGGE	Jorn Hellemans; Laurien Hoornaert; Marijke Reynders; Patrick Descheemaeker; Thomas Van Landschoot
EPI_ISL_1252485, EPI_ISL_1265170, EPI_ISL_1265172	AZ Sint-Maarten	Imelda hospital Bonheiden	Dagmar Obbels; Hanne Valgaeren; Johan Frans
EPI_ISL_1191626	AZ St.Jan	AZ SINT-JAN BRUGGE	Jorn Hellemans; Laurien Hoornaert; Marijke Reynders; Patrick Descheemaeker; Thomas Van Landschoot
EPI_ISL_1191080, EPI_ISL_1191088 to 1191091, EPI_ISL_1191624	AZ St.Jan Brugge	AZ SINT-JAN BRUGGE	Jorn Hellemans; Laurien Hoornaert; Marijke Reynders; Patrick Descheemaeker; Thomas Van Landschoot
EPI_ISL_1201480 to 1201481, EPI_ISL_1201483 to 1201484	AZ St.Jan Brugge-Oostende	AZ SINT-JAN BRUGGE	Jorn Hellemans; Laurien Hoornaert; Marijke Reynders; Patrick Descheemaeker; Thomas Van Landschoot
EPI_ISL_1191625	AZ St.Jan, campus Oostende	AZ SINT-JAN BRUGGE	Jorn Hellemans; Laurien Hoornaert; Marijke Reynders; Patrick Descheemaeker; Thomas Van Landschoot
EPI_ISL_1194703, EPI_ISL_1194706 to 1194708, EPI_ISL_1194711 to 1194714	AZ St.jan Brugge	AZ SINT-JAN BRUGGE	Jorn Hellemans; Laurien Hoornaert; Marijke Reynders; Patrick Descheemaeker; Thomas Van Landschoot
EPI_ISL_872065 to 872068	AZ Vesale (Tongres)	GIGA Medical Genomics	Bouchra Boujemla; Cécile Meex; Keith Durkin; Maria Artesi; Marie-Pierre Hayette; Pierrette Melin; Raphaël Boreux; Sébastien Bontems; Vincent Bours
EPI_ISL_1191627 to 1191629, EPI_ISL_1194705, EPI_ISL_1194710, EPI_ISL_1201482, EPI_ISL_1201485, EPI_ISL_1265143, EPI_ISL_1265149 to 1265151, EPI_ISL_1265156			
see above	AZ Zeno	AZ SINT-JAN BRUGGE	Jorn Hellemans; Laurien Hoornaert; Marijke Reynders; Patrick Descheemaeker; Thomas Van Landschoot
EPI_ISL_911386 to 911401, EPI_ISL_911430 to 911442, EPI_ISL_913496 to 913513, EPI_ISL_913594 to 913605, EPI_ISL_954755 to 954761, EPI_ISL_954763 to 954774, EPI_ISL_954814 to 954817, EPI_ISL_961181 to 961203, EPI_ISL_978788 to 978810, EPI_ISL_978812 to 978866, EPI_ISL_978868 to 978879, EPI_ISL_1058079 to 1058095, EPI_ISL_1058097 to 1058150, EPI_ISL_1060520 to 1060542, EPI_ISL_1109911 to 1109943, EPI_ISL_1181866 to 1181880, EPI_ISL_1222722 to 1222756, EPI_ISL_1295943 to 1295977, EPI_ISL_1299457 to 1299489			
see above	AZDelta	AZDelta	Dieter De Smet; Geert Martens
EPI_ISL_940573 to 940575, EPI_ISL_949079 to 949081	AZT	Jessa	Jessa_cmdLab
EPI_ISL_949091, EPI_ISL_1138888	AZV	Jessa	Cruys et al. on behalf of the Jessa_cmdLab; Jessa_cmdLab
EPI_ISL_735501 to 735502, EPI_ISL_1123293 to 1123296, EPI_ISL_1133119, EPI_ISL_1133203	Abdul Malek Ukil Medical College, Noakhali	Central Biological Research Laboratory and Department of Biochemistry and Molecular Biology	H. M. Abdullah Al Masud; Imam Hossen; Md. Arif Hossain; Md. Imranul Hoq; Md. Khondakar Raziur Rahman; Md. Omer Faruq; Mohammad Omar Faruque; Robiul Hasan Bhuiyan; Sajib Rudra; Shanta Paul
EPI_ISL_896109	Abklärungs- und _Teststation BL	University Hospital Basel, Clinical Bacteriology	Adrian Egli; Alfredo Mari; Hans Hirsch; Helena MB Seth-Smith; Juerg Sommer; Julia Bielicki; Karoline Leuzinger; Madlen Stange; Manuel Battegay; Samuel Erny; Thomas Goetz; Tim Roloff
EPI_ISL_1137613, EPI_ISL_1168289 to 1168303, EPI_ISL_1168306, EPI_ISL_1168308 to 1168347	Academic Center for Pathomorphological and Genetic-Molecular Diagnostics ltd, Bialystok, Poland	Academic Center for Pathomorphological and Genetic-Molecular Diagnostics ltd, Bialystok, Poland	Jacek Nikliski; Joanna Resze; Przemysaw Biecek; Radoslaw Charkiewicz
EPI_ISL_517612 to 517662, EPI_ISL_518799 to 518818	Academic Hospital Paramaribo	Erasmus Medical Center	Bas Oude Munnink; Dion Gajadin; Ed Ijzerman; Emmanuelle Munger; Gary Gummels; Ingrid Krishnadath; Lycke Woittiez; Marion Koopmans; Mireille Van de Veer; Princes Wongsowidjojo; Radjesh Ori; Rohma Banwari; Stephen Vreden
EPI_ISL_890185 to 890187, EPI_ISL_891151, EPI_ISL_905731, EPI_ISL_911707, EPI_ISL_911709	Academic Hospital of Gadjah Mada University (RSA UGM)	Genetics Working Group (Pokja Genetik) Faculty of Medicine, Public Health and Nursing Universitas Gadjah Mada (FK-KMK UGM); Disease Investigation Center Wates Ministry of Agriculture Indonesia; Department of Microbiology FK-KMK UGM; Laboratorium Diagnostik Yayasan Tahija World Mosquito Program (WMP) Yogyakarta Center for Tropical Medicine FK-KMK UGM; Integrated Research center FK-KMK UGM; Department of Computer Science and Electronics FMIPA UGM	Aditya Rifqi Fauzi; Afiahayati; Alvin Santoso Kalim; Audric Kenny Tedja; Dwi AA Nugrahaningsih; Dwiki afandy; Dyah Ayu Puspitarani; Edwin W. Daniwijaya; Eggi Arguni; Endah Supriyati; Fadil Fahri; Gunadi; Hendra Wibawa; Kemala Athollah; Kristy Iskandar; Ludhang P. Rizki; Marcellus; Maria Patricia Inggriani; Mohamad S. Hakim; Nungki Anggorowati; Siswanto; Susan Simanjaya; Titik Nuryastuti; Tri Wibawa; Untung Riawan; William Widitjiarso
EPI_ISL_877129	Academic Hospital of Gajdah Mada University (RSA UGM)	Genetics Working Group (Pokja Genetik) Faculty of Medicine, Public Health and Nursing Universitas Gadjah Mada (FK-KMK UGM); Disease Investigation Center Wates Ministry of Agriculture Indonesia; Department of Microbiology FK-KMK UGM; Laboratorium Diagnostik Yayasan Tahija World Mosquito Program (WMP) Yogyakarta Center for Tropical Medicine FK-KMK UGM; Integrated Research Center FK-KMK UGM; Department of Computer Science and Electronics FMIPA UGM	Afiahayati; Dwi AA Nugrahaningsih; Dwiki afandy; Dyah Ayu Puspitarani; Edwin W. Daniwijaya; Eggi Arguni; Endah Supriyati; Gunadi; Hendra Wibawa; Kristy Iskandar; Ludhang P. Rizki; Marcellus; Mohamad S. Hakim; Nungki Anggorowati; Siswanto; Titik Nuryastuti; Tri Wibawa

EPI_ISL_877126 to 877128, EPI_ISL_877130 to 877131	Academic Hospital of Gajdah Mada University (RSA UGM)	Genetics Working Group (Pokja Genetik) Faculty of Medicine, Public Health and Nursing Universitas Gadjah Mada (FK-KMK UGM); Disease Investigation Center Wates Ministry of Agriculture Indonesia; Department of Microbiology FK-KMK UGM; Laboratorium Diagnostik Yayasan Tahija World Mosquito Program (WMP) Yogyakarta Center for Tropical Medicine FK-KMK UGM; Integrated Research center FK-KMK UGM; Department of Computer Science and Electronics FMIPA UGM	Aditya Rifqi Fauzi; Afiahayati; Alvin Santoso Kalim; Audric Kenny Tedja; Desyifa Mursalin; Dwi AA Nugrahaningsih; Edwin W. Daniwijaya; Eggi Arguni; Endah Supriyati; Fadil Fahri; Gunadi; Hendra Wibawa; Kemala Athollah; Kristy Iskandar; Ludhang P. Rizki; Marcellus; Maria Patricia Inggriani; Mohamad S. Hakim; Nungki Anggorowati; Siswanto; Susan Simanjaya; Titik Nuryastuti; Tri Wibawa; Untung Riawan; William Widitjiarso
EPI_ISL_476135	Achima Care Fristadens VC	The Public Health Agency of Sweden	Anna Risberg; Anna-Malin Linde; Karin Tegmark-Wisell; Maria Lind Karlberg; Mattias Haukland; Mia Brytting; Olov Svartstrom; Oskar Karlsson Lindsjo; Petra Edquist; Reza Advani; Sandra Broddesson
EPI_ISL_509412 to 509417	Acibadem Labcell Cellular Therapy Laboratory	Acibadem Mehmet Ali Aydinlar University School of Medicine, Medical Genetics Department	Bulut Yurtsever; Cihan Tastan; Derya Dilek Kancagi; Ercument Ovali; Gozde Sir Karakus; Gunseli Bayram Akcapinar; Ilayda Sahin; Ozden Hatirnaz Ng; Ozkan Ozdemir; Sezer Akyoney; Ugur Ozbek
EPI_ISL_1159382, EPI_ISL_1159387	Adi Husada Kapasari Hospital	Institute of Tropical Disease, Universitas Airlangga	Aldise M Nastri; Gatot Soegiarto; Hermanto Wijaya; Jezzy R Dewantari; Kazufumi Shimizu; Krisnoadi Rahardjo; Laksmi Wulandari; Maria I Lusida; Resti Yudhawati; Rima R Prasetya; Soetjipto; Yasuko Mori
EPI_ISL_458083, EPI_ISL_956271	Adi Husada Undaan Hospital	Institute of Tropical Disease, Universitas Airlangga	Aldise M Nastri; Gatot Soegiarto; Irawati Marga; Jezzy R Dewantari; Kazufumi Shimizu; Krisnoadi Rahardjo; Laksmi Wulandari; Maria I Lusida; Mitsuhiro Nishimura; Resti Yudhawati; Retno A Setyoningrum; Rima R Prasetya; Soetjipto; Yasuko Mori; Yohko K Shimizu
EPI_ISL_1182095 to 1182096, EPI_ISL_1182098 to 1182104	Adolfo Lutz Institute Santos Regional Center Brazil	Retrovirus Laboratory Adolfo Lutz Institute	Audrey Cilli; Cintia Ahagon; Gabriela Bastos Cabral; Giselle I S Lopez-Lopes; Igor Mohamed Hussein; Luis Brigido; Paula Morena Guimaraes
EPI_ISL_514253	Advanced Biotechnology Laboratory	Genomic Research Lab, BCSIR	Abu Sayeed Mohammad Mahmud; Barna Goswami; Eshrar Osman; Eunus Ali; Hossain Uddin Shekhar; Iffat Jahan; M. Aftab Uddin; Md. Ahashan Habib; Md. Bayejid Hosen; Md. Murshed Hasan Sarkar; Md. Saddam Hossain; Md. Salim Khan; Mohammad Samir Uzzaman; Salek Ahmed Sajib; Shahina Akter; Tanjina Akhter Banu; Utpal Chandra Ray
EPI_ISL_1201884 to 1201888, EPI_ISL_1219028 to 1219036	Aeroporto Internacional de Guarulhos	Instituto Adolfo Lutz, Interdiciplinary Procedures Center, Strategic Laboratory	Caio Vinicius Dias Lopes; Claudia Regina Gonçalves; Claudio Tavares Sacchi; Erica Valessa Ramos Gomes; Karoline Rodrigues Campos
EPI_ISL_528934 to 528949	Agenzia di Tutela della Salute di Bergamo	Istitituto Zooprofilattico Sperimentale dell'Abruzzo e Molise "G.Caporale"	Ancora M; Cammà C; Curini V; Di Domenico M; Di Pasquale A; Lorusso A; Mangone I; Marcacci M; Puglia I; Rinaldi A; Savini G.
EPI_ISL_445244	Akbiomed lab	Tejgaon College bmb lab	Md.Abdul kaium; Md.Easin Arafat
EPI_ISL_420134, EPI_ISL_420136, EPI_ISL_420139, EPI_ISL_420311 to 420312, EPI_ISL_500779 to 500783, EPI_ISL_500793 to 500796, EPI_ISL_549083 to 549084, EPI_ISL_549089 to 549091, EPI_ISL_549169 to 549171, EPI_ISL_590889 to 590892, EPI_ISL_590951 to 590952, EPI_ISL_635102, EPI_ISL_635194, EPI_ISL_668391, EPI_ISL_668393, EPI_ISL_775269 to 775270, EPI_ISL_775275, EPI_ISL_775344, EPI_ISL_775387 to 775390, EPI_ISL_775479, EPI_ISL_775528 to 775530, EPI_ISL_796649, EPI_ISL_796665 to 796666, EPI_ISL_796699, EPI_ISL_796712 to 796713, EPI_ISL_813975, EPI_ISL_860210, EPI_ISL_860212, EPI_ISL_860219, EPI_ISL_860223, EPI_ISL_860232, EPI_ISL_860259 to 860260, EPI_ISL_860269, EPI_ISL_860277, EPI_ISL_906817 to 906820, EPI_ISL_906826 to 906829, EPI_ISL_962895, EPI_ISL_962897, EPI_ISL_962911 to 962913, EPI_ISL_964254, EPI_ISL_964257, EPI_ISL_964259, EPI_ISL_964284, EPI_ISL_964287, EPI_ISL_964289, EPI_ISL_964297, EPI_ISL_964300, EPI_ISL_964303, EPI_ISL_964305, EPI_ISL_964308, EPI_ISL_964953 to 964954, EPI_ISL_964958, EPI_ISL_964963 to 964965, EPI_ISL_964975 to 964976, EPI_ISL_964992 to 964994, EPI_ISL_1013470 to 1013473, EPI_ISL_1013478 to 1013481, EPI_ISL_1013495, EPI_ISL_1013499, EPI_ISL_1013508 to 1013516, EPI_ISL_1034307 to 1034308, EPI_ISL_1034311 to 1034312, EPI_ISL_1034314 to 1034315, EPI_ISL_1034317, EPI_ISL_1034325, EPI_ISL_1034364, EPI_ISL_1034366 to 1034367, EPI_ISL_1040147 to 1040148, EPI_ISL_1073916, EPI_ISL_1073919, EPI_ISL_1073925 to 1073926, EPI_ISL_1073930, EPI_ISL_1073933, EPI_ISL_1073943, EPI_ISL_1073949 to 1073951, EPI_ISL_1110514, EPI_ISL_1110516 to 1110520, EPI_ISL_1110524 to 1110533, EPI_ISL_1110543 to 1110545, EPI_ISL_1110548 to 1110552, EPI_ISL_1118293, EPI_ISL_1118304 to 1118305, EPI_ISL_1118310, EPI_ISL_1118320, EPI_ISL_1118326, EPI_ISL_1118329 to 1118330, EPI_ISL_1118333, EPI_ISL_1118335, EPI_ISL_1118340, EPI_ISL_1118350, EPI_ISL_1118393 to 1118394, EPI_ISL_1118396, EPI_ISL_1118398 to 1118399, EPI_ISL_1118402 to 1118407, EPI_ISL_1118410 to 1118412, EPI_ISL_1118439, EPI_ISL_1118466 to 1118467, EPI_ISL_1118478, EPI_ISL_1118490, EPI_ISL_1118498 to 1118499, EPI_ISL_1118513, EPI_ISL_1118533, EPI_ISL_1118535, EPI_ISL_1118538, EPI_ISL_1118547 to 1118548, EPI_ISL_1118552, EPI_ISL_1118560 to 1118561, EPI_ISL_1118563, EPI_ISL_1118566 to 1118567, EPI_ISL_1118574, EPI_ISL_1118578 to 1118580, EPI_ISL_1118582, EPI_ISL_1118584, EPI_ISL_1118593, EPI_ISL_1118601, EPI_ISL_1118607, EPI_ISL_1118660 to 1118663, EPI_ISL_1118665, EPI_ISL_1118667 to 1118668, EPI_ISL_1118674, EPI_ISL_1118676 to 1118677, EPI_ISL_1118679 to 1118681, EPI_ISL_1118684, EPI_ISL_1118688 to 1118689, EPI_ISL_1186136 to 1186137, EPI_ISL_1186139, EPI_ISL_1186143 to 1186144, EPI_ISL_1186146, EPI_ISL_1186192 to 1186198, EPI_ISL_1192165, EPI_ISL_1192171 to 1192172, EPI_ISL_1192177, EPI_ISL_1192198, EPI_ISL_1192210, EPI_ISL_1192214, EPI_ISL_1192219 to 1192225, EPI_ISL_1192311, EPI_ISL_1192313, EPI_ISL_1192315 to 1192316, EPI_ISL_1192319, EPI_ISL_1192335, EPI_ISL_1251304, EPI_ISL_1251322 to 1251325, EPI_ISL_1251441 to 1251442, EPI_ISL_1251445 to 1251446, EPI_ISL_1251449, EPI_ISL_1251452 to 1251457, EPI_ISL_1251468 to 1251469, EPI_ISL_1251509 to 1251512, EPI_ISL_1251529, EPI_ISL_1251568 to 1251574, EPI_ISL_1251576, EPI_ISL_1251594 to 1251598, EPI_ISL_1251647 to 1251661, EPI_ISL_1251671 to 1251673, EPI_ISL_1251675, EPI_ISL_1251682 to 1251685, EPI_ISL_1251715, EPI_ISL_1251718 to 1251719, EPI_ISL_1251722 to 1251723, EPI_ISL_1251789 to 1251795, EPI_ISL_1251813 to 1251823, EPI_ISL_1251832 to 1251844, EPI_ISL_1251848 to 1251892, EPI_ISL_1251907, EPI_ISL_1251909, EPI_ISL_1251971 to 1251972, EPI_ISL_1251984, EPI_ISL_1251986, EPI_ISL_1252009 to 1252011, EPI_ISL_1252020, EPI_ISL_1252028, EPI_ISL_1252040 to 1252041, EPI_ISL_1252044, EPI_ISL_1252487, EPI_ISL_1252497 to 1252499, EPI_ISL_1252512 to 1252517, EPI_ISL_1252520 to 1252521, EPI_ISL_1252523, EPI_ISL_1252525 to 1252528, EPI_ISL_1254533 to 1254534, EPI_ISL_1254539, EPI_ISL_1254543, EPI_ISL_1254547, EPI_ISL_1254549, EPI_ISL_1254556 to 1254557, EPI_ISL_1254562, EPI_ISL_1254564, EPI_ISL_1254568, EPI_ISL_1254570, EPI_ISL_1254574 to 1254578, EPI_ISL_1254584 to 1254585, EPI_ISL_1254590 to 1254592, EPI_ISL_1254595 to 1254596, EPI_ISL_1254603, EPI_ISL_1254606, EPI_ISL_1254612 to 1254614, EPI_ISL_1254616 to 1254618, EPI_ISL_1254624, EPI_ISL_1254627, EPI_ISL_1254635 to 1254638, EPI_ISL_1314414 to 1314434, EPI_ISL_1314525 to 1314526, EPI_ISL_1314530 to 1314533			
see above	Akershus University Hospital, Department for Microbiology and Infectious Disease Control	Norwegian Institute of Public Health, Department of Virology	Atiya R Ali; Debech Nadia; Engebretsen Serina Beate; Engebretsen Serina Beate Atiya R Ali; Garcia Llorente Ignacio; Hilde Elshaug; Hilde Synnøve Vollan; Hilde Vollan; Ignacio Garcia Llorente; Jon Bråte; Kamilla Heddeland Instefjord; Karoline Bragstad; Kathrine Stene-Johansen; Marie Paulsen Madsen; Olav Hungnes; Pedersen Benedikte Nevjen; Rasmus Riis Kopperud; Serina B Engebretsen
EPI_ISL_480204	Akita City Public Health Center	Pathogen Genomics Center, National Institute of Infectious Diseases	Hajime Kamiya; Kentaro Itokawa; Koichi Ito; Makoto Kuroda; Masanori Hashino; Motoi Suzuki; Rina Tanaka; Tsuyoshi Sekizuka
EPI_ISL_747240	Al Islam Hospital	West Java Health Laboratory; School of Life Sciences and Technology, Institut Teknologi Bandung	Azzania Fibriani; Cut Nur Cinthia Alamanda; Ema Rahmawati; Isak Solihin; Karimatu Khoirunnisa; Miftahul Faridl; Rifky Waluyajati Rachman; Rini Robiani; Ryan Bayusantika Ristandi
EPI_ISL_739659 to 739661	Al-Quds Nutrition and Health Research Institute, Al-Quds University	Al-Quds Nutrition and Health Research Institute, Al-Quds University	Al-Jawabreh, A.; Ereqat, S.; Nasereddin, A.; Rishmawi, C.
EPI_ISL_649153, EPI_ISL_661272	Al-Quds Nutrition and Health Research Institute, Al-Quds University	Al-Quds Nutrition and Health Research Institute, Al-Quds University	A. and Al-Jawabreh, A.; Ereqat; Ereqat, S.; Nasereddin; Nasereddin, A.; S. and Al-Jawabreh, A.
EPI_ISL_509688	Alabama Department of Public Health Bureau of Clinical Laboratories	Pathogen Discovery, Respiratory Viruses Branch, Division of Viral Diseases, Centers for Disease Control and Prevention	Anna Uehara; Clinton Paden; Haibin Wang; Jing Zhang; Krista Queen; Suxiang Tong; Yan Li; Ying Tao
EPI_ISL_1093546 to 1093601, EPI_ISL_1196775 to 1196791, EPI_ISL_1205471 to 1205484	Alameda County Public Health Department	Alameda County Public Health Department	Kevin Libuit; Kristna Hsieh; Maria Elma
EPI_ISL_454607 to 454613, EPI_ISL_468357 to 468387, EPI_ISL_625500 to 625546, EPI_ISL_625611 to 625622, EPI_ISL_672014, EPI_ISL_672023 to 672024, EPI_ISL_672029, EPI_ISL_672032, EPI_ISL_672036, EPI_ISL_672038 to 672040, EPI_ISL_672045, EPI_ISL_672050, EPI_ISL_672053 to 672054, EPI_ISL_672060, EPI_ISL_672062, EPI_ISL_672064, EPI_ISL_672066, EPI_ISL_672076, EPI_ISL_672078, EPI_ISL_672082, EPI_ISL_672086, EPI_ISL_672091 to 672092, EPI_ISL_672095, EPI_ISL_672097, EPI_ISL_672113, EPI_ISL_672305 to 672353, EPI_ISL_672439 to 672476, EPI_ISL_738509 to 738510, EPI_ISL_738513 to 738514, EPI_ISL_738519, EPI_ISL_738522 to 738524, EPI_ISL_738527 to 738528, EPI_ISL_738531, EPI_ISL_738535, EPI_ISL_738537 to 738538, EPI_ISL_738540, EPI_ISL_738545, EPI_ISL_738547, EPI_ISL_738553 to 738554, EPI_ISL_738561 to 738562, EPI_ISL_738564 to 738565, EPI_ISL_738572 to 738573, EPI_ISL_738577, EPI_ISL_738580, EPI_ISL_738584 to 738585, EPI_ISL_738587, EPI_ISL_738592, EPI_ISL_738604 to 738607, EPI_ISL_738609, EPI_ISL_738613 to 738616, EPI_ISL_738619, EPI_ISL_738622 to 738623, EPI_ISL_738625 to 738627, EPI_ISL_738629, EPI_ISL_738633, EPI_ISL_738642, EPI_ISL_738648, EPI_ISL_738654, EPI_ISL_738656, EPI_ISL_738659, EPI_ISL_738665, EPI_ISL_738668, EPI_ISL_738671, EPI_ISL_738674 to 738680, EPI_ISL_738686 to 738687, EPI_ISL_738692, EPI_ISL_738699, EPI_ISL_738711, EPI_ISL_738715, EPI_ISL_738721, EPI_ISL_738724, EPI_ISL_738743 to 738745, EPI_ISL_738748 to 738749, EPI_ISL_738751, EPI_ISL_738753, EPI_ISL_738760, EPI_ISL_738766 to 738768, EPI_ISL_738773 to 738776, EPI_ISL_738781, EPI_ISL_738783, EPI_ISL_738788, EPI_ISL_738792 to 738793, EPI_ISL_738796 to 738797, EPI_ISL_738800, EPI_ISL_738802, EPI_ISL_738804, EPI_ISL_738813 to 738814, EPI_ISL_738817, EPI_ISL_738819, EPI_ISL_738821 to 738822, EPI_ISL_738825, EPI_ISL_738828, EPI_ISL_738831, EPI_ISL_738833, EPI_ISL_738835, EPI_ISL_738837 to 738838, EPI_ISL_738843, EPI_ISL_738846 to 738848, EPI_ISL_738858, EPI_ISL_738868, EPI_ISL_738876, EPI_ISL_738881, EPI_ISL_738886, EPI_ISL_738891 to 738892, EPI_ISL_738895 to 738896, EPI_ISL_738904, EPI_ISL_738914, EPI_ISL_738919, EPI_ISL_738925, EPI_ISL_738932, EPI_ISL_738934, EPI_ISL_738940, EPI_ISL_738945, EPI_ISL_738950, EPI_ISL_738957, EPI_ISL_738963, EPI_ISL_738974, EPI_ISL_738976, EPI_ISL_738982 to 738983, EPI_ISL_738986, EPI_ISL_738992, EPI_ISL_739003, EPI_ISL_739018, EPI_ISL_739023, EPI_ISL_739029, EPI_ISL_739033, EPI_ISL_739035, EPI_ISL_739046 to 739047, EPI_ISL_739049, EPI_ISL_739054, EPI_ISL_739056 to 739057, EPI_ISL_739059, EPI_ISL_739062, EPI_ISL_739064, EPI_ISL_739073, EPI_ISL_739075, EPI_ISL_739077, EPI_ISL_739082 to 739084, EPI_ISL_739088, EPI_ISL_739090, EPI_ISL_739093, EPI_ISL_739096, EPI_ISL_739100, EPI_ISL_739109 to 739111, EPI_ISL_739113, EPI_ISL_739115, EPI_ISL_739118, EPI_ISL_739120, EPI_ISL_739124, EPI_ISL_739127, EPI_ISL_739134 to 739137, EPI_ISL_739140, EPI_ISL_739144, EPI_ISL_739149 to 739150, EPI_ISL_739158, EPI_ISL_739160 to 739161, EPI_ISL_739163 to 739164, EPI_ISL_739171, EPI_ISL_739173 to 739174, EPI_ISL_739178 to 739179, EPI_ISL_739189 to 739190, EPI_ISL_739192, EPI_ISL_739196, EPI_ISL_739200 to 739202, EPI_ISL_739206 to 739209, EPI_ISL_739212 to 739213, EPI_ISL_739215, EPI_ISL_739217 to 739218, EPI_ISL_739220, EPI_ISL_739226, EPI_ISL_739229, EPI_ISL_739231, EPI_ISL_739233, EPI_ISL_739236, EPI_ISL_739241, EPI_ISL_739243 to 739244, EPI_ISL_739257, EPI_ISL_739261 to 739262, EPI_ISL_739268, EPI_ISL_739270, EPI_ISL_739280, EPI_ISL_739283, EPI_ISL_739285 to 739286, EPI_ISL_739288, EPI_ISL_739290, EPI_ISL_739294, EPI_ISL_739300, EPI_ISL_739302 to 739303, EPI_ISL_739306 to 739309, EPI_ISL_739312, EPI_ISL_739323 to 739324, EPI_ISL_739326, EPI_ISL_739328, EPI_ISL_739333, EPI_ISL_739337 to 739338, EPI_ISL_739340, EPI_ISL_739349, EPI_ISL_739351, EPI_ISL_739356, EPI_ISL_739358, EPI_ISL_739361, EPI_ISL_739368, EPI_ISL_739378, EPI_ISL_739383, EPI_ISL_739385, EPI_ISL_739387, EPI_ISL_739391, EPI_ISL_739395, EPI_ISL_739406 to 739408, EPI_ISL_739422, EPI_ISL_739424, EPI_ISL_739426 to 739427, EPI_ISL_739434 to 739435, EPI_ISL_739439, EPI_ISL_739444, EPI_ISL_739446, EPI_ISL_739448 to 739449, EPI_ISL_739454, EPI_ISL_739459, EPI_ISL_739463, EPI_ISL_739468, EPI_ISL_739470, EPI_ISL_739472 to 739473, EPI_ISL_739483 to 739484, EPI_ISL_739495, EPI_ISL_739503, EPI_ISL_739506 to 739507, EPI_ISL_739511, EPI_ISL_739515 to 739516, EPI_ISL_739518 to 739519, EPI_ISL_739522 to 739523, EPI_ISL_739526, EPI_ISL_739528, EPI_ISL_739535, EPI_ISL_739538, EPI_ISL_739541, EPI_ISL_739544, EPI_ISL_739551 to 739552, EPI_ISL_739555, EPI_ISL_739563, EPI_ISL_739565, EPI_ISL_739569, EPI_ISL_739572, EPI_ISL_739575 to 739576, EPI_ISL_739578, EPI_ISL_739583, EPI_ISL_739587, EPI_ISL_739590, EPI_ISL_739598, EPI_ISL_739600 to 739601, EPI_ISL_739604, EPI_ISL_739606, EPI_ISL_739610, EPI_ISL_739616, EPI_ISL_739619, EPI_ISL_739622 to 739623, EPI_ISL_739631, EPI_ISL_739634, EPI_ISL_739638, EPI_ISL_739640, EPI_ISL_739643 to 739644, EPI_ISL_739648, EPI_ISL_739652, EPI_ISL_739656, EPI_ISL_955386 to 955426, EPI_ISL_1027296, EPI_ISL_1027303, EPI_ISL_1027307 to 1027309, EPI_ISL_1027323 to 1027324, EPI_ISL_1027330, EPI_ISL_1027333, EPI_ISL_1027336, EPI_ISL_1027341, EPI_ISL_1027345, EPI_ISL_1027348, EPI_ISL_1027361, EPI_ISL_1027363, EPI_ISL_1027365, EPI_ISL_1027445 to 1027473, EPI_ISL_1027479, EPI_ISL_1027543, EPI_ISL_1027560, EPI_ISL_1027568, EPI_ISL_1027578, EPI_ISL_1027582, EPI_ISL_1137082 to 1137169, EPI_ISL_1184624 to 1184680, EPI_ISL_1184861 to 1184966,			

EPI_ISL_1185027 to 1185028, EPI_ISL_1185031, EPI_ISL_1185033 to 1185038, EPI_ISL_1185041, EPI_ISL_1185047, EPI_ISL_1185054, EPI_ISL_1185056 to 1185057, EPI_ISL_1185066, EPI_ISL_1185071, EPI_ISL_1185073, EPI_ISL_1185082, EPI_ISL_1185085, EPI_ISL_1185087, EPI_ISL_1185094 to 1185095, EPI_ISL_1185100, EPI_ISL_1185102, EPI_ISL_1185105, EPI_ISL_1185109, EPI_ISL_1185117, EPI_ISL_1185121, EPI_ISL_1185124, EPI_ISL_1185126, EPI_ISL_1185128, EPI_ISL_1185130, EPI_ISL_1185136, EPI_ISL_1185142, EPI_ISL_1185157, EPI_ISL_1185161 to 1185162, EPI_ISL_1185164, EPI_ISL_1185172, EPI_ISL_1185176, EPI_ISL_1185179, EPI_ISL_1185187, EPI_ISL_1185198 to 1185199, EPI_ISL_1185204, EPI_ISL_1185207, EPI_ISL_1185215, EPI_ISL_1185222, EPI_ISL_1185229, EPI_ISL_1185232, EPI_ISL_1185243, EPI_ISL_1185250, EPI_ISL_1185258 to 1185259, EPI_ISL_1185262, EPI_ISL_1185271, EPI_ISL_1185281, EPI_ISL_1185283, EPI_ISL_1185286, EPI_ISL_1185305, EPI_ISL_1185313, EPI_ISL_1185315, EPI_ISL_1185322, EPI_ISL_1185351, EPI_ISL_1185356 to 1185358, EPI_ISL_1185362 to 1185364, EPI_ISL_1185367 to 1185368, EPI_ISL_1185372, EPI_ISL_1185376, EPI_ISL_1185383, EPI_ISL_1185388, EPI_ISL_1185393, EPI_ISL_1185401, EPI_ISL_1185414, EPI_ISL_1185422, EPI_ISL_1185427, EPI_ISL_1185430 to 1185431, EPI_ISL_1185447, EPI_ISL_1185449, EPI_ISL_1185452, EPI_ISL_1185456 to 1185457, EPI_ISL_1185459, EPI_ISL_1185461, EPI_ISL_1185466, EPI_ISL_1185475, EPI_ISL_1185480, EPI_ISL_1185491, EPI_ISL_1185493, EPI_ISL_1185496, EPI_ISL_1185499, EPI_ISL_1185501 to 1185503, EPI_ISL_1185517, EPI_ISL_1185529, EPI_ISL_1185533 to 1185534, EPI_ISL_1234542 to 1234543, EPI_ISL_1234562, EPI_ISL_1234564 to 1234568, EPI_ISL_1234574, EPI_ISL_1234597, EPI_ISL_1234600, EPI_ISL_1234603 to 1234604, EPI_ISL_1234611 to 1234612, EPI_ISL_1234617, EPI_ISL_1234621, EPI_ISL_1234637, EPI_ISL_1234640, EPI_ISL_1234646, EPI_ISL_1234648, EPI_ISL_1234661, EPI_ISL_1234665 to 1234666, EPI_ISL_1234670, EPI_ISL_1234672, EPI_ISL_1234676 to 1234677, EPI_ISL_1234679, EPI_ISL_1234683, EPI_ISL_1234698, EPI_ISL_1234701, EPI_ISL_1234716, EPI_ISL_1234718, EPI_ISL_1234720, EPI_ISL_1234723, EPI_ISL_1234732, EPI_ISL_1234739, EPI_ISL_1234742 to 1234743, EPI_ISL_1234751, EPI_ISL_1234755, EPI_ISL_1234758, EPI_ISL_1234762, EPI_ISL_1234765, EPI_ISL_1234767, EPI_ISL_1234773, EPI_ISL_1234776, EPI_ISL_1234779, EPI_ISL_1234782, EPI_ISL_1234785, EPI_ISL_1234790, EPI_ISL_1234795, EPI_ISL_1234802, EPI_ISL_1234808 to 1234809, EPI_ISL_1234815, EPI_ISL_1234819 to 1234820, EPI_ISL_1234827, EPI_ISL_1234829 to 1234830, EPI_ISL_1234832, EPI_ISL_1234834 to 1234835, EPI_ISL_1234839, EPI_ISL_1234844, EPI_ISL_1234848, EPI_ISL_1234858, EPI_ISL_1234860, EPI_ISL_1234862, EPI_ISL_1234870, EPI_ISL_1234872 to 1234873, EPI_ISL_1234877, EPI_ISL_1234892, EPI_ISL_1234894 to 1234895, EPI_ISL_1234900 to 1234901, EPI_ISL_1234904, EPI_ISL_1234907, EPI_ISL_1234920 to 1234921, EPI_ISL_1234924, EPI_ISL_1234932 to 1234933, EPI_ISL_1234939, EPI_ISL_1234941, EPI_ISL_1234950, EPI_ISL_1234952 to 1234953, EPI_ISL_1234956, EPI_ISL_1234959, EPI_ISL_1234968 to 1234969, EPI_ISL_1234979, EPI_ISL_1234982, EPI_ISL_1234986 to 1234987, EPI_ISL_1234990, EPI_ISL_1234995, EPI_ISL_1235006, EPI_ISL_1235013, EPI_ISL_1235021 to 1235022, EPI_ISL_1235025, EPI_ISL_1235028, EPI_ISL_1235032, EPI_ISL_1235042, EPI_ISL_1235048, EPI_ISL_1235054, EPI_ISL_1235059, EPI_ISL_1235075, EPI_ISL_1235077, EPI_ISL_1235083, EPI_ISL_1235091, EPI_ISL_1235093 to 1235094, EPI_ISL_1235098 to 1235099, EPI_ISL_1235102, EPI_ISL_1235111, EPI_ISL_1235115 to 1235116, EPI_ISL_1235122, EPI_ISL_1235124, EPI_ISL_1235141 to 1235142, EPI_ISL_1235147, EPI_ISL_1235149, EPI_ISL_1235158, EPI_ISL_1235165, EPI_ISL_1235171, EPI_ISL_1235175, EPI_ISL_1235178, EPI_ISL_1235181, EPI_ISL_1235183, EPI_ISL_1235185, EPI_ISL_1235188, EPI_ISL_1235194, EPI_ISL_1235198, EPI_ISL_1235210, EPI_ISL_1235213, EPI_ISL_1235217, EPI_ISL_1235219, EPI_ISL_1235223, EPI_ISL_1235230, EPI_ISL_1235235, EPI_ISL_1235241, EPI_ISL_1235243 to 1235244, EPI_ISL_1235249, EPI_ISL_1235255 to 1235256, EPI_ISL_1235263, EPI_ISL_1235270, EPI_ISL_1235276, EPI_ISL_1235279, EPI_ISL_1235281, EPI_ISL_1235289, EPI_ISL_1235292, EPI_ISL_1235294, EPI_ISL_1235299, EPI_ISL_1235303, EPI_ISL_1235308 to 1235309, EPI_ISL_1235312 to 1235314, EPI_ISL_1235318, EPI_ISL_1235335, EPI_ISL_1235338 to 1235339, EPI_ISL_1235359, EPI_ISL_1235361, EPI_ISL_1235363 to 1235366, EPI_ISL_1235368 to 1235369, EPI_ISL_1235374, EPI_ISL_1235376, EPI_ISL_1235380, EPI_ISL_1235385 to 1235393, EPI_ISL_1235397 to 1235399, EPI_ISL_1235402, EPI_ISL_1235404 to 1235407, EPI_ISL_1235414, EPI_ISL_1235421, EPI_ISL_1235423, EPI_ISL_1235425 to 1235426, EPI_ISL_1235432 to 1235433, EPI_ISL_1235437, EPI_ISL_1235440, EPI_ISL_1235443, EPI_ISL_1235446 to 1235448, EPI_ISL_1235456 to 1235460, EPI_ISL_1235462 to 1235465, EPI_ISL_1235471, EPI_ISL_1235475 to 1235477, EPI_ISL_1235480 to 1235482, EPI_ISL_1235485 to 1235486, EPI_ISL_1235489, EPI_ISL_1235492 to 1235495, EPI_ISL_1235497, EPI_ISL_1235499 to 1235500, EPI_ISL_1235503 to 1235505, EPI_ISL_1235510, EPI_ISL_1235512 to 1235514, EPI_ISL_1235516 to 1235517, EPI_ISL_1235520, EPI_ISL_1235523 to 1235524, EPI_ISL_1235527, EPI_ISL_1235529, EPI_ISL_1235532, EPI_ISL_1235537 to 1235538, EPI_ISL_1235543, EPI_ISL_1235548, EPI_ISL_1235550, EPI_ISL_1235552 to 1235555, EPI_ISL_1235557, EPI_ISL_1235559, EPI_ISL_1235561 to 1235562, EPI_ISL_1235564 to 1235569, EPI_ISL_1235571, EPI_ISL_1235573 to 1235577, EPI_ISL_1235579 to 1235580, EPI_ISL_1235584 to 1235587, EPI_ISL_1235589 to 1235590, EPI_ISL_1235592, EPI_ISL_1235594 to 1235595, EPI_ISL_1235597 to 1235599, EPI_ISL_1235603, EPI_ISL_1235605, EPI_ISL_1235609 to 1235611			
see above	Alameda County Public Health Lab	Chan-Zuckerberg Biohub	CZB Cliahub Consortium
EPI_ISL_872349	Alaska State Virology Lab	Alaska State Virology Lab	Jack Chen; Lisa Smith; Ph.D.; Stephanie DeRonde
EPI_ISL_420303 to 420306, EPI_ISL_424346 to 424347, EPI_ISL_427619 to 427622, EPI_ISL_431013 to 431019, EPI_ISL_435441 to 435444, EPI_ISL_436464, EPI_ISL_437437, EPI_ISL_437513 to 437518, EPI_ISL_437873, EPI_ISL_476898 to 476899, EPI_ISL_492049 to 492063, EPI_ISL_492087, EPI_ISL_512135 to 512157, EPI_ISL_522396 to 522405, EPI_ISL_525758 to 525760, EPI_ISL_528485 to 528537, EPI_ISL_560554 to 560566, EPI_ISL_576111 to 576112, EPI_ISL_586243 to 586266, EPI_ISL_602559 to 602561, EPI_ISL_806810 to 806852, EPI_ISL_884214 to 884221, EPI_ISL_911676 to 911703, EPI_ISL_1039712 to 1039736, EPI_ISL_1049483 to 1049512, EPI_ISL_1061279 to 1061308, EPI_ISL_1068293 to 1068314, EPI_ISL_1182693 to 1182811, EPI_ISL_1195910 to 1195945, EPI_ISL_1195964 to 1195999			
see above	Alaska State Virology Laboratory	Alaska State Virology Laboratory	Bortz E; Chen; Chen J et al with Pathogenomics group Dagdag R; Chen, J.; DeRonde, S.; Deuling, H.; Devin M. Drown; Drown DM; Elva House; George W; J; Jack Chen; Kovalenko A; Lisa Smith; Milton E; Ph.D.; PhD.; PhD. Jack Chen; Redlinger M; Stephanie DeRonde
EPI_ISL_769916 to 769985	Albany Medical Center Hospital Clinical Laboratories	Wadsworth Center, New York State Department.of Health	Alexis Russel; Daryl M. Lamson; Erasmus Schneider; Erica Lasek-Nesselquist; John Kelly; Jonathan Plitnick; Kirsten St. George; Matthew Shudt; Melissa A Leisner; Navjot Singh; Sara Griesemer
EPI_ISL_804985 to 806525, EPI_ISL_854461 to 854593	Alberta Precision Labs (APL)	Alberta Precision Labs (APL)	Berenger B; Bernier F; Chui L; Croxen M; Gordon P; Kellner J; Lam LG; Li V; Ma R; Melin A; Pabbaraju K; Tipples G; Wong A; Zelyas N
EPI_ISL_1287284 to 1287319	Alexianer DaKS GmbH (eigenes IT-Tochterunternehmen der Alexianer Krankenhäuser)	Robert Koch Institute	
EPI_ISL_1014940	Algemeen Klinisch Labo Lier	KU Leuven, Rega Institute, Clinical and Epidemiological Virology	Bert Vanmechelen; Joan Marti-Carerras; Piet Maes; Tony Wawina-Bokalanga
EPI_ISL_984288, EPI_ISL_984290 to 984296, EPI_ISL_984616 to 984617, EPI_ISL_985040, EPI_ISL_1036727, EPI_ISL_1103562, EPI_ISL_1103564			
see above	Algemeen Medisch Laboratorium	UAntwerp, Laboratory of Medical Microbiology	Basil Britto Xavier; Christine Lammens; Herman Goossens; Jasmine Coppens; Marie Le Mercier; Veerle Matheeussen
EPI_ISL_1039162, EPI_ISL_1039207, EPI_ISL_1039209 to 1039211, EPI_ISL_1039218, EPI_ISL_1039221, EPI_ISL_1039242 to 1039247, EPI_ISL_1164849, EPI_ISL_1171465 to 1171473, EPI_ISL_1190852, EPI_ISL_1259237, EPI_ISL_1259240, EPI_ISL_1279939			
see above	Algemeen Medisch Laboratorium (AML)	UAntwerp, Laboratory of Medical Microbiology	Basil Britto Xavier; Christine Lammens; Herman Goossens; Jasmine Coppens; Marie Le Mercier; Veerle Matheeussen
EPI_ISL_523854	Alice Ho Miu Ling Nethersole Hospital	Hong Kong Department of Health	Chan Rickjason C.W.; Lam Edman T.K.; Mak Gannon C.K.; Tsang Dominic N.C.
EPI_ISL_1173159 to 1173161, EPI_ISL_1173163 to 1173174	All India Institute of Medical Science, Raipur	Indian Council of Medical Research-National Institute of Virology, Microbial Containment Complex	Anudita Bhargava; Pragya D. Yadav
EPI_ISL_1231419 to 1231429, EPI_ISL_1231435, EPI_ISL_1251265	All India Institute of Medical Sciences,Bhopal	Indian Council of Medical Research-National Institute of Virology, Microbial Containment Complex	Debasis Biswas; Pragya D. Yadav
EPI_ISL_508156 to 508206	All india institute of Medical Sciences Rishikesh	National Institute of Biomedical Genomics	Amit Mangla; Arindam Maitra; Deepjyoti Kalita; Ravi Kant; Saumitra Das
EPI_ISL_511908 to 511922	All india institute of Medical Sciences Rishikesh	National Institute of Biomedical Genomics - DBT's PAN-INDIA 1000 SARS--CoV-2 RNA Genome Sequencing Consortium	Amit Mangla; Arindam Maitra; Deepjyoti Kalita; Ravi Kant; Saumitra Das
EPI_ISL_514647, EPI_ISL_514649, EPI_ISL_514651, EPI_ISL_644941, EPI_ISL_1132813	Allina Health Laboratory	Minnesota Department of Health, Public Health Laboratory	Alexandra Lorentz; Jacob Garfin; Matt Plumb; and Xiong Wang
EPI_ISL_700475 to 700476, EPI_ISL_700521, EPI_ISL_700548, EPI_ISL_1040749	Alma CDC wc AHC	NHLS/UCT	Arash Iranzadeh; Bruna Galvao; Carolyn Williamson; Deelan Doolabh; Diana Hardie; Houriiyah Tegally; Innocent Mudau; Kruger Marais; Lynn Tyers; Marvin Hsiao; Stephen Korsman
EPI_ISL_528538	Alsafar	Alsafar	Andreas Henschel; Ernesto Damiani; Gihan Elsir Ahmed Daw Elbait; Guan Tay; Habiba Alsafar; Rifat; Samuel Feng
EPI_ISL_528686 to 528721	Alsafar - Khalifa University Abu Dhabi	Alsafar - Khalifa University Abu Dhabi	Andreas Henschel; Ernesto Damiani; Gihan Daw Elbait; Guan Tay; Habiba Alsafar; Rifat Hamoudi; Samuel Feng
EPI_ISL_900491 to 900501, EPI_ISL_1018220 to 1018228, EPI_ISL_1109636 to 1109637, EPI_ISL_1109639 to 1109641, EPI_ISL_1109643, EPI_ISL_1180210 to 1180228	Althaia. Xarxa Assistencial Universitària de Manresa	IrsiCaixa - Can Ruti CovidSeq	2a planta; Badalona Gloria Trujillo; Bonaventura Clotet; Bonaventura Clotet Gloria Trujillo; Carolina Gonzalez Fernandez; Eulalia Grau; Francesc Catala-Moll; Fundació irsiCaixa. Hospital Universitari Germans Trias i Pujol(HUGTiP); Jaume Trape Pujol; Marc Noguera-Julian; Maria Casadellà; Mariona Parera; Pilar Armengol; Rafel Perez Vidal; Roger Paredes; maternal Ctra Canyet s/n
EPI_ISL_1222616 to 1222694, EPI_ISL_1229174 to 1229175	Altius Institute	Seattle Flu Study	Alex Nguyen; Amanda Adler; Andrew Meuser; Barry R. Lutz; Benjamin Pelle; Caitlin R. Wolf; Chris D. Frazar; Clem Green; Daniel Bates; Deborah A. Nickerson; Elisabeth Brandstetter; Erica Ryke; Hannah Petersen; Helen Y. Chu; Jacob Rodriguez; Janet A. Englund; Jay Shendure; Jessica Halow; John Stamatoyannopoulos; Joshua Richards; Jover Lee; Julia Wald; Kairsten Fay; Kirsten Lacombe; Kneshay Harper; Lea M. Starita; Mark J. Rieder; Matt Hartman; Matthew Richardson; Matthew Thompson; Melissa Truong; Michael Boeckh; Michael Famulare; Misja Ilcisin; Muhammad Halimun; Olivia Waltner; Peter D. Han; Rebecca Bruders; Ryan Alexander; Sadie Patraw; Sofia Olsson; Stephanie DeBaun; Thomas R. Sibley; Tobias Ragoczy; Trevor Bedford; Truong Nguyen
EPI_ISL_891094 to 891135, EPI_ISL_911972 to 912034, EPI_ISL_912109 to 912159, EPI_ISL_962305 to 962367, EPI_ISL_1015757 to 1015876, EPI_ISL_1015887 to 1016003, EPI_ISL_1049666 to 1049691, EPI_ISL_1049693 to 1049711, EPI_ISL_1049713 to 1049842, EPI_ISL_1114291 to 1114351			
see above	Altius Institute for Biomedical Sciences	Seattle Flu Study	Alex Nguyen; Amanda Adler; Andrew Meuser; Barry R. Lutz; Benjamin Pelle; Caitlin R. Wolf; Chris D. Frazar; Clem Green; Daniel Bates; Deborah A. Nickerson; Elisabeth Brandstetter; Erica Ryke; Hannah Petersen; Helen Y. Chu; Jacob Rodriguez; Janet A. Englund; Jay Shendure; Jessica Halow; John Stamatoyannopoulos; Joshua Richards; Jover Lee; Julia Wald; Kairsten Fay; Kirsten Lacombe; Kneshay Harper; Lea M. Starita; Mark J. Rieder; Matt Hartman; Matthew Richardson; Matthew Thompson; Melissa Truong; Michael Boeckh; Michael Famulare; Misja Ilcisin; Muhammad Halimun; Olivia Waltner; Peter D. Han; Rebecca Bruders; Ryan Alexander; Sadie Patraw; Sofia Olsson; Stephanie DeBaun; Thomas R. Sibley; Tobias Ragoczy; Trevor Bedford; Truong Nguyen
EPI_ISL_515544, EPI_ISL_523984, EPI_ISL_523986	Ama Dr Jose Soares Hungria	Instituto Adolfo Lutz, Interdiciplinary Procedures Center, Strategic Laboratory	Claudia Regina Gonçalves; Claudio Tavares Sacchi; Erica Valessa Ramos Gomes

EPI_ISL_410302	Amalea Dulcene Nicolasora Research Institute for Tropical Medicine, Molecular Biology Laboratory	Amalea Dulcene Nicolasora Research Institute for Tropical Medicine, Molecular Biology Laboratory	A.D.; E.S.; F.M.; I.P.; J.G.; K.M.; M.A.; Manalo; Medado; Mercado; Nicolasora; O.T. and Cruz; Onza; Polotan; Tujan
EPI_ISL_569864 to 569886	Amedeo di savoia	Crosetto lab, Karolinska Institutet, SciLifeLab	Anna Sapino; Luuk Harbers; Maria Grazia Milia; Michele Simonetti; Nicola Crosetto; Ning Zhang; Valeria Ghisetti
EPI_ISL_1009161 to 1009212	American Esoteric Laboratories	Colleen B. Jonsson	Colleen B. Jonsson; Jyothi Parvathareddy; Mariah K. Taylor; Walter Reichard
EPI_ISL_955271, EPI_ISL_955273 to 955274, EPI_ISL_955285, EPI_ISL_955314, EPI_ISL_955322 to 955327	American Esoteric Laboratory	Pathogen Discovery, Respiratory Viruses Branch, Division of Viral Diseases, Centers for Disease Control and Prevention	Anna Uehara; Clinton R. Paden; Haibin Wang; Jing Zhang; Krista Queen; Peter Cook; Suxiang Tong; Yan Li; Ying Tao
EPI_ISL_456656	American Type Culture Collection Inc. (ATCC)	American Type Culture Collection Inc. (ATCC)	A.M.; Benton, B.; Chu; D.K.W.; Flores, B.; Frank; J.S.M.; King; M. and Rashid, S.; M.A.; N.P.; Parker; Peiris; Puthuveetil; Riojas; S.P.
EPI_ISL_660121 to 660129	Ampath	National Health Laboratory Service (NHLS), Tygerberg	Bronwyn Kleinhans; Davis M-A; Draper C; Eduan Wilkindon; Gert van Zyl; Houriiyah Tegally; Hsiao M; Kayla Delaney; Siegfried N; Susan Engelbrecht; Tulio de Oliveira; Williamson C; Wolfgang Preiser
EPI_ISL_860553, EPI_ISL_860631 to 860632	Ampath-Netcare	KRISP, KZn Research Innovation and Sequencing Platform	Giandhari J; Khan S; Lessells R; Mdlalose K; Pillay S; Tegally H; Wilkinson E; York D; de Oliveira T
EPI_ISL_1138745	Analysis laboratory	National Reference Center for Viruses of Respiratory Infections, Institut Pasteur, Paris	Angela Brisebarre; Camille Capel; Etienne Simon-Lorière; J. Besson; Marion Barbet; Maud Vanpeene; Méline Bizard; Sylvie Behillil; Sylvie van der Werf; Vincent Enouf
EPI_ISL_766587, EPI_ISL_812257	Analytica Medizinische Laboratorien AG	Institute of Medical Virology, University of Zurich	Alexandra Trkola; Annette Audigé; Cyril Shah; Guido Bloemberg; Jon Huder; Jürg Böni; Kevin Steiner; Maria Grünberg; Maryam Zaheri; Michael Huber; Riccarda Capaul; Stefan Schmutz; Verena Kufner
EPI_ISL_414648, EPI_ISL_416457	Andersen Lab, The Scripps Research Institute	Andersen Lab, The Scripps Research Institute	Catie Anderson; Emily Spender; Karthik Gangavarapu; Kristian Andersen; Laura Nicholson; Mark Zeller; Raphaelle Klitting; Refugio Robles-Sikisaka; Sarah Topol
EPI_ISL_429991, EPI_ISL_430016	Andersen lab at Scripps Research	Andersen lab at Scripps Research	SEARCH Alliance San Diego
EPI_ISL_450818	Aneby VC	The Public Health Agency of Sweden	Anna Risberg; Anna-Malin Linde; Karin Tegmark-Wisell; Ken Granath; Maria Lind Karlberg; Mia Brytting; Olov Svartstrom; Oskar Karlsson Lindsjo; Theresa Enkirch
EPI_ISL_450427	Anhui Provincial Center for Disease Control	Anhui Provincial CDC, Acute Infectious Disease Prevention & Ctrl	Chen, Q.; Ge, Y.; Gong, L.; He, J.; He, L.; Hou, S.; Jiang, L.; Li, W.; Liu, J.; Liu, Z.; Lu, S.; Sa, N.; Shi, Y.; Sun, Y.; Wu, J.; Yu, J.; Yuan, Y.; Zhang, Z.
EPI_ISL_1216046, EPI_ISL_1216113, EPI_ISL_1216135, EPI_ISL_1216213, EPI_ISL_1216430	AniCon Labor GmbH	Robert Koch Institute	
EPI_ISL_649145 to 649148	Animal health and puplic health, Escola de Medicina Veterinaria	Animal health and puplic health, Escola de Medicina Veterinaria	Carvalho; I.G.C.; J.C.; M.O.; R.F.; Ribeiro Junior; Ribeiro, J.; da Silva; dos Santos
EPI_ISL_1252705, EPI_ISL_1252728 to 1252731, EPI_ISL_1252837 to 1252843	Anteja laboratorija (UAB Diagnostikos laboratorija)	Vilnius University Hospital Santaros Klinikos, Center of Laboratory Medicine	Daniel Naumovas; Dovile Ezerskyte; Gytis Dudas; Ingrida Olendraite; Laimonas Griskevicius; Ligita Raugaite; Mindaugas Stoskus; Monika Katenaite; Rimvydas Norvilas
EPI_ISL_582127 to 582132	Antwerp University Hospital	Institute of Tropical Medicine	Colin Anthony; Philippe Selhorst
EPI_ISL_877458, EPI_ISL_1165068, EPI_ISL_1165071	Anwar Medika General Hospital	Institute of Tropical Disease, Universitas Airlangga	Aldise M Nastri; Gatot Soegiarto; Jezzy R Dewantari; Kazufumi Shimizu; Krisnoadi Rahardjo; Laksmi Wulandari; Maria I Lusida; Nungky Taniasari; Resti Yudhawati; Rima R Prasetya; Soetjipto; Yasuko Mori
EPI_ISL_909955 to 909962	Apollo Hospitals	CSIR-Centre for Cellular and Molecular Biology	Divya Tej Sowpati; Irawathy Goud; Karthik Bharadwaj Tallapaka; Lamuk Zaveri; Onkar Kulkarni; Payel Mukherjee; Sofia Banu; Suneetha Narreddy
EPI_ISL_1297827 to 1297829	Arcispedale Santa Maria Nuova, Autoimmunità, Allergologia e Biotecnologie Innovative	Istituto Zooprofilattico Sperimentale della Lombardia e dell'Emilia Romagna (IZSLER), Risk Analysis and Genomic Epidemiology Unit	Alessandro Zerbini; Erika Scaltriti; Ilaria Menozzi; Lucia Belloni; Marina Morganti; Stefania Croci; Stefano Pongolini
EPI_ISL_1120120, EPI_ISL_1154361, EPI_ISL_1154890	Area Biologia Molecolare - Istituto Zooprofilattico Sperimentale della Sicilia	Area Biologia Molecolare - Istituto Zooprofilattico Sperimentale della Sicilia	BRUNO Federica; BRUNO Gabriella; CASTELLI Germano; DI NARO Daniela; MAIDA Carmelo Massimo; MAZZUCCO Walter; PIAZZA Angela; RANDAZZO Giulia; REALE Stefano; SCIBETTA Silvia; TRAMUTO Fabio; VITALE Fabrizio; VITALE Francesco
EPI_ISL_512663 to 512664, EPI_ISL_512669, EPI_ISL_527740, EPI_ISL_527749	Area De Salud Alajuela Norte - Clinica Dr. Marcial Rodriguez	Inciensa, Instituto Costarricense de Investigación y Enseñanza en Nutrición y Salud	Adriana Godinez & Melany Calderon; Claudio Soto-Garita; Estela Cordero; Francisco Duarte; Hebleen Porras
EPI_ISL_527756	Area De Salud Aserri	Inciensa, Instituto Costarricense de Investigación y Enseñanza en Nutrición y Salud	Adriana Godinez & Melany Calderon; Claudio Soto-Garita; Estela Cordero; Francisco Duarte; Hebleen Porras
EPI_ISL_770030	Area De Salud Buenos Aires	Inciensa, Instituto Costarricense de Investigación y Enseñanza en Nutrición y Salud	Adriana Godínez; Claudio Soto-Garita; Estela Cordero; Francisco Duarte; Hebleen Porras; Melany Calderón & Mariel López
EPI_ISL_769986, EPI_ISL_769994, EPI_ISL_769997 to 769998, EPI_ISL_770021, EPI_ISL_770026	Area De Salud Catedral Noreste	Inciensa, Instituto Costarricense de Investigación y Enseñanza en Nutrición y Salud	Adriana Godínez; Claudio Soto-Garita; Estela Cordero; Francisco Duarte; Hebleen Porras; Melany Calderón & Mariel López
EPI_ISL_770011 to 770012	Area De Salud Coronado	Inciensa, Instituto Costarricense de Investigación y Enseñanza en Nutrición y Salud	Adriana Godínez; Claudio Soto-Garita; Estela Cordero; Francisco Duarte; Hebleen Porras; Melany Calderón & Mariel López
EPI_ISL_512668, EPI_ISL_527745, EPI_ISL_527748, EPI_ISL_527751, EPI_ISL_770008, EPI_ISL_770029	Area De Salud Corredores	Inciensa, Instituto Costarricense de Investigación y Enseñanza en Nutrición y Salud	Adriana Godinez & Melany Calderon; Adriana Godínez; Claudio Soto-Garita; Estela Cordero; Francisco Duarte; Hebleen Porras; Melany Calderón & Mariel López
EPI_ISL_770014	Area De Salud Curridabat 2	Inciensa, Instituto Costarricense de Investigación y Enseñanza en Nutrición y Salud	Adriana Godínez; Claudio Soto-Garita; Estela Cordero; Francisco Duarte; Hebleen Porras; Melany Calderón & Mariel López
EPI_ISL_512653	Area De Salud Desamparados 1 - Clinica Dr. Marcial Fallas [Grifo Alto/Desampara	Inciensa, Instituto Costarricense de Investigación y Enseñanza en Nutrición y Salud	Adriana Godinez & Melany Calderon; Claudio Soto-Garita; Estela Cordero; Francisco Duarte; Hebleen Porras
EPI_ISL_769993	Area De Salud El Guarco	Inciensa, Instituto Costarricense de Investigación y Enseñanza en Nutrición y Salud	Adriana Godínez; Claudio Soto-Garita; Estela Cordero; Francisco Duarte; Hebleen Porras; Melany Calderón & Mariel López
EPI_ISL_770009 to 770010	Area De Salud Escazu (Coopesana)	Inciensa, Instituto Costarricense de Investigación y Enseñanza en Nutrición y Salud	Adriana Godínez; Adriana Godínez & Melany Calderón; Claudio Soto-Garita; Estela Cordero; Francisco Duarte; Hebleen Porras; Melany Calderón & Mariel López
EPI_ISL_512659	Area De Salud Fortuna	Inciensa, Instituto Costarricense de Investigación y Enseñanza en Nutrición y Salud	Adriana Godinez & Melany Calderon; Claudio Soto-Garita; Estela Cordero; Francisco Duarte; Hebleen Porras
EPI_ISL_527757	Area De Salud Goicoechea 1	Inciensa, Instituto Costarricense de Investigación y Enseñanza en Nutrición y Salud	Adriana Godinez & Melany Calderon; Claudio Soto-Garita; Estela Cordero; Francisco Duarte; Hebleen Porras
EPI_ISL_770016, EPI_ISL_770020	Area De Salud Goicoechea 2 - Clinica Dr. Jimenez Nuñez	Inciensa, Instituto Costarricense de Investigación y Enseñanza en Nutrición y Salud	Adriana Godínez; Claudio Soto-Garita; Estela Cordero; Francisco Duarte; Hebleen Porras; Melany Calderón & Mariel López
EPI_ISL_512662, EPI_ISL_512666 to 512667, EPI_ISL_512671, EPI_ISL_527746 to 527747, EPI_ISL_527760, EPI_ISL_770000 to 770003, EPI_ISL_770027			
see above	Area De Salud La Cruz	Inciensa, Instituto Costarricense de Investigación y Enseñanza en Nutrición y Salud	Adriana Godinez & Melany Calderon; Adriana Godínez; Claudio Soto-Garita; Estela Cordero; Francisco Duarte; Hebleen Porras; Melany Calderón & Mariel López
EPI_ISL_512660 to 512661	Area De Salud Los Chiles	Inciensa, Instituto Costarricense de Investigación y	Adriana Godinez & Melany Calderon; Claudio Soto-Garita; Estela Cordero; Francisco Duarte; Hebleen Porras

		Enseñanza en Nutrición y Salud	
EPI_ISL_770005	Area De Salud Moravia	Inciensa, Instituto Costarricense de Investigación y Enseñanza en Nutrición y Salud	Adriana Godínez; Claudio Soto-Garita; Estela Cordero; Francisco Duarte; Hebleen Porras; Melany Calderón & Mariel López
EPI_ISL_512658	Area De Salud Orotina-San Mateo [Orotina/Alajuela]	Inciensa, Instituto Costarricense de Investigación y Enseñanza en Nutrición y Salud	Adriana Godinez & Melany Calderon; Claudio Soto-Garita; Estela Cordero; Francisco Duarte; Hebleen Porras
EPI_ISL_769999	Area De Salud Paraiso-Cervantes	Inciensa, Instituto Costarricense de Investigación y Enseñanza en Nutrición y Salud	Adriana Godínez; Claudio Soto-Garita; Estela Cordero; Francisco Duarte; Hebleen Porras; Melany Calderón & Mariel López
EPI_ISL_512656	Area De Salud Pavas (Coopesalud) [Pavas/San Jose]	Inciensa, Instituto Costarricense de Investigación y Enseñanza en Nutrición y Salud	Adriana Godinez & Melany Calderon; Claudio Soto-Garita; Estela Cordero; Francisco Duarte; Hebleen Porras
EPI_ISL_770017, EPI_ISL_770022	Area De Salud Perez Zeledon	Inciensa, Instituto Costarricense de Investigación y Enseñanza en Nutrición y Salud	Adriana Godínez; Claudio Soto-Garita; Estela Cordero; Francisco Duarte; Hebleen Porras; Melany Calderón & Mariel López
EPI_ISL_769988 to 769989, EPI_ISL_770015, EPI_ISL_770023	Area De Salud San Francisco-San Antonio (Coopesana)	Inciensa, Instituto Costarricense de Investigación y Enseñanza en Nutrición y Salud	Adriana Godínez; Claudio Soto-Garita; Estela Cordero; Francisco Duarte; Hebleen Porras; Melany Calderón & Mariel López
EPI_ISL_769996, EPI_ISL_770025	Area De Salud San Juan-San Diego-Concepcion 2	Inciensa, Instituto Costarricense de Investigación y Enseñanza en Nutrición y Salud	Adriana Godínez; Claudio Soto-Garita; Estela Cordero; Francisco Duarte; Hebleen Porras; Melany Calderón & Mariel López
EPI_ISL_770007	Area De Salud San Rafael	Inciensa, Instituto Costarricense de Investigación y Enseñanza en Nutrición y Salud	Adriana Godínez; Claudio Soto-Garita; Estela Cordero; Francisco Duarte; Hebleen Porras; Melany Calderón & Mariel López
EPI_ISL_512657	Area De Salud Tibas-Uruca-Merced - Clinica Dr. Clorito Picado [Tibas/San Jose]	Inciensa, Instituto Costarricense de Investigación y Enseñanza en Nutrición y Salud	Adriana Godinez & Melany Calderon; Claudio Soto-Garita; Estela Cordero; Francisco Duarte; Hebleen Porras
EPI_ISL_491446	Area de Salud Alajuela Central	Inciensa, Instituto Costarricense de Investigación y Enseñanza en Nutrición y Salud	Adriana Godinez & Melany Calderon; Claudio Soto-Garita; Estela Cordero; Francisco Duarte; Hebleen Brenes
EPI_ISL_434533, EPI_ISL_434535, EPI_ISL_491449	Area de Salud Alajuela Sur	Inciensa, Instituto Costarricense de Investigación y Enseñanza en Nutrición y Salud	Adriana Godinez & Melany Calderon; Claudio Soto-Garita; Estela Cordero; Francisco Duarte; Hebleen Brenes; Hebleen Porras
EPI_ISL_491437, EPI_ISL_491444	Area de Salud Escazu (Coopesana)	Inciensa, Instituto Costarricense de Investigación y Enseñanza en Nutrición y Salud	Adriana Godinez & Melany Calderon; Claudio Soto-Garita; Estela Cordero; Francisco Duarte; Hebleen Brenes
EPI_ISL_491457	Area de Salud Los Santos	Inciensa, Instituto Costarricense de Investigación y Enseñanza en Nutrición y Salud	Adriana Godinez & Melany Calderon; Claudio Soto-Garita; Estela Cordero; Francisco Duarte; Hebleen Brenes
EPI_ISL_491445	Area de Salud Mata Redonda	Inciensa, Instituto Costarricense de Investigación y Enseñanza en Nutrición y Salud	Adriana Godinez & Melany Calderon; Claudio Soto-Garita; Estela Cordero; Francisco Duarte; Hebleen Brenes
EPI_ISL_434539	Area de Salud Orotina	Inciensa, Instituto Costarricense de Investigación y Enseñanza en Nutrición y Salud	Adriana Godinez & Melany Calderon; Claudio Soto-Garita; Estela Cordero; Francisco Duarte; Hebleen Porras
EPI_ISL_500596 to 500706, EPI_ISL_500717, EPI_ISL_509492 to 509523, EPI_ISL_527008 to 527064, EPI_ISL_527179 to 527180, EPI_ISL_527400, EPI_ISL_530334 to 530340, EPI_ISL_537286 to 537287, EPI_ISL_539327, EPI_ISL_569858, EPI_ISL_577597, EPI_ISL_577604, EPI_ISL_586571, EPI_ISL_710513, EPI_ISL_738137, EPI_ISL_745029 to 745031, EPI_ISL_775242, EPI_ISL_775245, EPI_ISL_794671, EPI_ISL_1205815, EPI_ISL_1240991 to 1240992, EPI_ISL_1255309 to 1255310			
see above	Area of Virology, Serology and Virology Division (SAViD), New South Wales Health Pathology Randwick	Area of Virology, Serology and Virology Division (SAViD), New South Wales Health Pathology Randwick	Au, J.; Bull, R.; Deveson, I.; Foster, C.; Rawlinson; Rawlinson, W.; Ruiz Silva, M.; Van Haal, S.; Van Hal, S.; W
EPI_ISL_1010705 to 1010706	Area of Virology, Serology and Virology Division (SAViD), New South Wales Health Pathology Randwick	Microbiology RPAH	Au, J.; Bull, R.; Deveson, I.; Foster, C.; Rawlinson, W.; Ruiz Silva, M.; Van Hal, S.
EPI_ISL_678292 to 678386, EPI_ISL_707891 to 707902, EPI_ISL_717701 to 717713, EPI_ISL_812347, EPI_ISL_812359, EPI_ISL_1005536 to 1005540, EPI_ISL_1069080, EPI_ISL_1098798 to 1098799, EPI_ISL_1121031, EPI_ISL_1121974 to 1121976, EPI_ISL_1184504 to 1184506, EPI_ISL_1293047 to 1293050			
see above	Area of Virology, Serology and Virology Division (SAViD), New South Wales Health Pathology Randwick	Virology Research Laboratory; Area of Virology, Serology and Virology Division (SAViD), New South Wales Health Pathology Randwick	Au, J.; Bull, R.; Deveson, I.; Foster, C.; Rawlinson, W.; Ruiz Silva, M.; Van Hal, S.
EPI_ISL_406223	Arizona Department of Health Services	Pathogen Discovery, Respiratory Viruses Branch, Division of Viral Diseases, Centers for Disease Control and Prevention	Anna Uehara; Brett L. Whitaker; Brian Lynch; Clinton R. Paden; Janna' R. Murray; Jing Zhang; Krista Queen; Lijuan Wang; Senthil Kumar K. Sakthivel; Shifaq Kamili; Stephen Lindstrom; Susan I. Gerber; Suxiang Tong; Xiaoyan Lu; Yan Li; Ying Tao
EPI_ISL_1113293 to 1113328, EPI_ISL_1113387 to 1113391, EPI_ISL_1113496 to 1113565, EPI_ISL_1183834 to 1183870	Arizona Department of Health Services	TGen North	"Jolene Bowers; Ashlyn Pfeiffer; Chris French; Darrin Lemmer; Dave Engelthaler; Hayley Yaglom; Heather Centner; Jolene Bowers; Megan Folkerts; The Arizona COVID Genomics Union (ACGU); The Arizona COVID Genomics Union (ACGU)"
EPI_ISL_824922 to 824941, EPI_ISL_824987 to 825012, EPI_ISL_925509 to 925523, EPI_ISL_925525 to 925534, EPI_ISL_925824 to 925845, EPI_ISL_930635 to 930664, EPI_ISL_933540 to 933563, EPI_ISL_933580 to 933601, EPI_ISL_967882 to 967884, EPI_ISL_968189 to 968211, EPI_ISL_978353 to 978395, EPI_ISL_978397 to 978487, EPI_ISL_1014993 to 1014995, EPI_ISL_1015713 to 1015732, EPI_ISL_1016887 to 1016895, EPI_ISL_1016897 to 1016912, EPI_ISL_1016914 to 1016946, EPI_ISL_1064172 to 1064196, EPI_ISL_1066758, EPI_ISL_1066760, EPI_ISL_1066762, EPI_ISL_1066764 to 1066765, EPI_ISL_1066767, EPI_ISL_1066769, EPI_ISL_1066771, EPI_ISL_1066773, EPI_ISL_1066775, EPI_ISL_1066777 to 1066778, EPI_ISL_1066780, EPI_ISL_1066783 to 1066784, EPI_ISL_1066786, EPI_ISL_1066788, EPI_ISL_1066790, EPI_ISL_1066792, EPI_ISL_1066794, EPI_ISL_1066796, EPI_ISL_1067696 to 1067716, EPI_ISL_1132643 to 1132667, EPI_ISL_1133209 to 1133222, EPI_ISL_1135078 to 1135112, EPI_ISL_1184559 to 1184581, EPI_ISL_1196391 to 1196412, EPI_ISL_1201441 to 1201460, EPI_ISL_1205356 to 1205380, EPI_ISL_1239048 to 1239108, EPI_ISL_1270202, EPI_ISL_1270204, EPI_ISL_1270206, EPI_ISL_1270208, EPI_ISL_1270210, EPI_ISL_1270212, EPI_ISL_1270214, EPI_ISL_1270216, EPI_ISL_1270218, EPI_ISL_1270220, EPI_ISL_1270222, EPI_ISL_1270224, EPI_ISL_1270227, EPI_ISL_1270229, EPI_ISL_1270231, EPI_ISL_1270233, EPI_ISL_1270235, EPI_ISL_1270237, EPI_ISL_1270239, EPI_ISL_1270241, EPI_ISL_1270243, EPI_ISL_1270245, EPI_ISL_1270247, EPI_ISL_1270250, EPI_ISL_1270254, EPI_ISL_1270797 to 1270821, EPI_ISL_1271968 to 1271986, EPI_ISL_1272077 to 1272094, EPI_ISL_1272276 to 1272299, EPI_ISL_1306093 to 1306120			
see above	Arizona State Public Health Laboratory	Arizona State Public Health Laboratory	Jessica Escobar; Katherine Fullerton; Linda Getsinger; Nobuko Fukushima; Stacy White; Trung Huynh; Victor Waddell
EPI_ISL_812114, EPI_ISL_903361 to 903363, EPI_ISL_943569, EPI_ISL_1048801, EPI_ISL_1048804, EPI_ISL_1050502, EPI_ISL_1050703, EPI_ISL_1050950, EPI_ISL_1051176, EPI_ISL_1091576, EPI_ISL_1132729, EPI_ISL_1167185, EPI_ISL_1184079 to 1184093, EPI_ISL_1291657 to 1291740, EPI_ISL_1292809 to 1292811, EPI_ISL_1299316 to 1299324, EPI_ISL_1300536 to 1300539			
see above	Arizona State University	Arizona State University	Efrem S. Lim; Emily A. Kaelin; Joshua LaBaer; Joy M. Blain; Kristina Buss; LaRinda A. Holland; Neal W Woodbury; Nicholas J. Mellor; Peter T. Skidmore; Rabia Maqsood; Valerie Harris; Vel Murugan
EPI_ISL_424668 to 424669, EPI_ISL_424671, EPI_ISL_467372 to 467373	Arizona State University Health Services	Arizona State University	Arvind Varsani; Bereket Estifanos; Brenda G. Hogue; Efrem S. Lim; Emily A. Kaelin; Jason Steel; LaRinda A. Holland; Lily I. Wu; Matthew Scotch; Nicholas J. Mellor; Peter T. Skidmore; Rabia Maqsood; Rolf U. Halden
EPI_ISL_1255065, EPI_ISL_1255079	Arlon	Plateforme de testing Namuroise	; Degosserie Jonathan; Denis Olivier; Mullier François; Otto Gaetan
EPI_ISL_496518 to 496520, EPI_ISL_496529, EPI_ISL_496533, EPI_ISL_496537 to 496545, EPI_ISL_496602, EPI_ISL_497880 to 497887, EPI_ISL_721629 to 721645, EPI_ISL_722187 to 722200			
see above	Armed Forces Medical College	National Centre For Cell Science	Arvind Sahu; DBT's PAN-INDIA 1000 SARS-CoV2 RNA genome sequencing consortium; Dhiraj Paul; Girdhari Lal; Janesh Kumar; Kavita Bala Anand; Kunal Jani; Maharashtra COVID-19 Study Group; Manoj Kumar Bhat; Murlidhar Tambe; Radha Chauhan; Rajesh Karyakarte; Rajiv Mohan Gupta; Santosh Karade; Shelinder Pal Singh Shergill; Sourav Sen; Suvarna Joshi; Vasudevan Seshadri; Yogesh S Shouche
EPI_ISL_1315065	Armed Forces Medical Research Laboratories and Blood Bank, Egypt	Department of Microbial Biotechnology, Genetic Engineering Division, National Research Centre	Ahmed Elsayed; Ahmed Taha; Ayman Farghaly; Mohamed Khalifa; Mohamed Shemis; Reham Dawood
EPI_ISL_1315064	Armed Forces Medical Research Laboratories and Blood Bank, Egypt	Department of Microbial Biotechnology, Genetic Engineering Division, National Research Centre,	Ahmed Elsayed; Ahmed Taha; Ayman Farghaly; Mohamed Khalifa; Mohamed Shemis; Reham Dawood
EPI_ISL_1110879 to 1110880, EPI_ISL_1110917, EPI_ISL_1111047, EPI_ISL_1111049, EPI_ISL_1168203 to 1168204, EPI_ISL_1168212 to 1168217, EPI_ISL_1201537 to 1201538, EPI_ISL_1262747 to 1262750, EPI_ISL_1301829, EPI_ISL_1301860, EPI_ISL_1301877 to 1301881			
see above	Armies	National Reference Center for Viruses of Respiratory	Angela Brisebarre; Camille Capel; Desroches Marine; Etienne Simon-Lorière; Foissaud Vincent; Louise Lefrançois; Marion Barbet; Maud Vanpeene;

		Infections, Institut Pasteur, Paris	Méline Bizard; Rousset Dominique; Sylvie Behillil; Sylvie van der Werf; Vincent Enouf
EPI_ISL_1118900	Army	National Reference Center for Viruses of Respiratory Infections, Institut Pasteur, Paris	Angela Brisebarre; Camille Capel; Desroches Marine; Etienne Simon-Lorière; Marion Barbet; Maud Vanpeene; Méline Bizard; Sylvie Behillil; Sylvie van der Werf; Vincent Enouf
EPI_ISL_457826	Army Medical Center - Scientific Department	Army Medical and Veterinary Research Center	Anna Anselmo; Antonella Fortunato; Florigio Lista; Francesco Giordani; Giovanni Faggioni; Nino D'Amore; Riccardo De Sanctis; Silvia Fillo; Vanessa Vera Fain
EPI_ISL_717978	Army Medical Center, Scientific Department, Virology Laboratory	Army Medical Center, Scientific Department, Virology Laboratory	Alessandra Amoroso; Andrea Ciammaruconi; Anella Monte; Anna Anselmo; Annalisa Pelo; Antonella Fortunato; Federica Galeano; Florigio Lista.; Francesco Giordani; Giovanni Faggioni; Giulia Campoli; Margherita De Santis; Marzia Cavalli; Nino D'Amore; Riccardo De Santis; Roberta Sorrentino; Rossella Tirelli; Silvia Fillo; Stella Lia; Vanessa Vera Fain
EPI_ISL_457825	Army Medical Research Center - Scientific Department	Army Medical and Veterinary Research Center	Anna Anselmo; Antonella Fortunato; Florigio Lista; Francesco Giordani; Giovanni Faggioni; Nino D'Amore; Riccardo De Sanctis; Silvia Fillo; Vanessa Vera Fain
EPI_ISL_539809 to 539812, EPI_ISL_539821	Asiaworld Expo Command Post	Hong Kong Department of Health	Alan K.L. Tsang; Dominic N.C. Tsang; Edman T.K. Lam; Peter C.W. Yip; Rickjason C.W. Chan
EPI_ISL_900525	Astralab	CNR Virus des Infections Respiratoires - France SUD	Antonin Bal; Bruno Lina; Gregory Destras; Gwendolyne Burfin; Hadrien Règue; Laurence Josset; Martine Valette; Quentin Semanas
EPI_ISL_1159701 to 1159728, EPI_ISL_1255416 to 1255423, EPI_ISL_1273379 to 1273381	Atlas Genomics	Atlas Genomics	MD; Michael Kalnoski
EPI_ISL_1049603	Atlas Genomics	Seattle Flu Study	Amanda Adler; Barry R. Lutz; Benjamin Pelle; Brian Hiatt; Caitlin R. Wolf; Chris D. Frazar; Deborah A. Nickerson; Elisabeth Brandstetter; Erica Ryke; Geoff Melly; Helen Y. Chu; Janet A. Englund; Jay Shendure; Jover Lee; Kairsten Fay; Kirsten Lacombe; Lea M. Starita; Mark J. Rieder; Matthew Richardson; Matthew Thompson; Melissa Truong; Michael Boeckh; Michael Famulare; Misja Ilcisin; Peter D. Han; Philip Dykema; Romesh Gautom; Scott Lindquist; Thomas R. Sibley; Trevor Bedford
EPI_ISL_902969	Atlas Genomics - UW Virology Lab	UW Virology Lab	Alexander Greninger; Hong Xie; Keith R Jerome; Lasata Shrestha; Meei-Li Huang; Michelle Lin; Pavitra Roychoudhury
EPI_ISL_977660, EPI_ISL_977665, EPI_ISL_1009666, EPI_ISL_1009668, EPI_ISL_1009670	Auburn Sports Medicine	Synergy Laboratories	Megan Cornwell
EPI_ISL_413490, EPI_ISL_416519, EPI_ISL_416526	Auckland Hospital	Institute of Environmental Science and Research (ESR)	Erasmus Smit; Gary McAuliffe; Joep de Ligt; Lauren Jelly; Matt Storey; Matthew Blakiston; Sally Roberts; Xiaoyun Ren
EPI_ISL_569616	Aurora County Clinic	South Dakota Public Health Laboratory	Jacob Garfin; Matt Plumb; Xiong Wang; and Chris Carlson
EPI_ISL_513340, EPI_ISL_526166 to 526169, EPI_ISL_593649	Austech Medical Laboratories	NSW Health Pathology - Institute of Clinical Pathology and Medical Research; Westmead Hospital; University of Sydney	CIDM-PH et al.
EPI_ISL_745091 to 745092	Australian Clinical Labs	CIDM-PH, Westmead Hospital	CIDM-PH et al.
EPI_ISL_451486, EPI_ISL_451574, EPI_ISL_451599, EPI_ISL_513331, EPI_ISL_544966 to 544967, EPI_ISL_544988, EPI_ISL_545024, EPI_ISL_547597, EPI_ISL_591484, EPI_ISL_767896, EPI_ISL_767902, EPI_ISL_767916 to 767918, EPI_ISL_872585			
see above	Australian Clinical Labs	NSW Health Pathology - Institute of Clinical Pathology and Medical Research; Westmead Hospital; University of Sydney	CIDM-PH et al.
EPI_ISL_526212, EPI_ISL_767884	Australian Clinical Labs (formerly Healthscope Pathology)	NSW Health Pathology - Institute of Clinical Pathology and Medical Research; Westmead Hospital; University of Sydney	CIDM-PH et al.
EPI_ISL_1827951	Australian Infectious Disease Research Centre, School of Chemistry and Molecular Biosciences, The University of Queensland	Australian Infectious Disease Research Centre, School of Chemistry and Molecular Biosciences, The University of Queensland	Parry; R.H. and Khromykh, A.
EPI_ISL_475830 to 475886, EPI_ISL_583573 to 583578, EPI_ISL_583630 to 583690, EPI_ISL_583883 to 583892, EPI_ISL_853721, EPI_ISL_853729 to 853731, EPI_ISL_853733 to 853738, EPI_ISL_853741, EPI_ISL_853746, EPI_ISL_853749, EPI_ISL_853753, EPI_ISL_853757 to 853758, EPI_ISL_853764, EPI_ISL_853766, EPI_ISL_853768, EPI_ISL_853775 to 853782, EPI_ISL_853789 to 853791, EPI_ISL_853793 to 853795, EPI_ISL_853797, EPI_ISL_853800 to 853801, EPI_ISL_853803 to 853804, EPI_ISL_853807 to 853813, EPI_ISL_853815, EPI_ISL_853817 to 853818, EPI_ISL_853962 to 854216, EPI_ISL_854223, EPI_ISL_854228, EPI_ISL_854231, EPI_ISL_854235, EPI_ISL_854242 to 854252, EPI_ISL_854254, EPI_ISL_854261 to 854294, EPI_ISL_854296 to 854298, EPI_ISL_854301, EPI_ISL_854304, EPI_ISL_934434 to 934539, EPI_ISL_934593 to 934632, EPI_ISL_1008019 to 1008081, EPI_ISL_1008083 to 1008191, EPI_ISL_1117776, EPI_ISL_1117798 to 1117799, EPI_ISL_1117804, EPI_ISL_1117824 to 1117830, EPI_ISL_1117838 to 1117839, EPI_ISL_1117854 to 1117869, EPI_ISL_1117887, EPI_ISL_1117907, EPI_ISL_1117914, EPI_ISL_1117916, EPI_ISL_1117923, EPI_ISL_1117927, EPI_ISL_1117937 to 1117948, EPI_ISL_1118030 to 1118038, EPI_ISL_1118044 to 1118045, EPI_ISL_1118049, EPI_ISL_1118073 to 1118078, EPI_ISL_1180752 to 1180830, EPI_ISL_1180932 to 1180934			
see above	Austrian Agency for Health and Food Safety (AGES)	Bergthaler laboratory, CeMM Research Center for Molecular Medicine of the Austrian Academy of Sciences	Adi Steinrigl; Alexander Lercher; Alexandra Popa; Andreas Bergthaler; Anna Schedl; Bekir Erguner; Benedikt Agerer; Christian Paar; Christoph Bock; Christoph Bock; Daniela Schmid; Dorothee von Laer; Elisabeth Puchhammer-Stoeckl; Fabian Amman; Franz Allerberger; Gernot Walder; Gregor Hörmann; Guenter Weiss; Gunther Vogl; Henrique Colaco; Jakob-Wendelin Genger; Jan Laine; Judith Aberle; Kinga Rigler-Hohenwarter; Lukas Endler; Maelle Le Moing; Manfred Nairz; Mark Smyth; Martin Senekowitsch; Martin Senekowitsch; Michael Schuster; Michael Schuster; Peter Hufnagl; Peter Obrist; Rainer Gattringer; Sabine Sussitz-Rack; Stephan Aberle; Thomas Penz; Wegene Borena
EPI_ISL_569610, EPI_ISL_569624	Avera McKennan Hospital	South Dakota Public Health Laboratory	Jacob Garfin; Matt Plumb; Xiong Wang; and Chris Carlson
EPI_ISL_507963 to 507972, EPI_ISL_514645	Avera Mckennan Laboratory	Minnesota Department of Health, Public Health Laboratory	Jacob Garfin; Matt Plumb; and Xiong Wang
EPI_ISL_845618 to 845619	Ayudas diagnósticas SURA	Instituto Nacional de Salud - Dirección de Investigación en Salud Pública	Carlos Franco-Muñoz; Diego A. Álvarez-Díaz; Diego Andrés Prada; Gerardo Santamaría; Jonathan Reales; Julian Naizaque; Katherine Laiton-Donato; Magdalena Wiesner; Marcela Mercado-Reyes; Maria T. Herrera-Sepúlveda; Martha Lucia Ospina Martinez; Mauricio Pacheco-Montealegre; Paola Muñoz-Laiton; Sheryll Corchuelo
EPI_ISL_882637 to 882644	Azerbaijan National Hematology Center Division of Medical Genetics	Azerbaijan National Hematology Center Division of Medical Genetics	Aghayev Agha Rza; Bayraml Ramin
EPI_ISL_902753, EPI_ISL_965133, EPI_ISL_983328, EPI_ISL_1009019 to 1009020, EPI_ISL_1039784, EPI_ISL_1079362 to 1079363, EPI_ISL_1079994, EPI_ISL_1091280 to 1091286, EPI_ISL_1091797			
see above	Azienda Ospedaliera San Camillo Forlanini	INMI Lazzaro Spallanzani IRCCS	A D'Agostino; A Di Caro; B Bartolini; C.E.M Gruber; CEM Gruber; D Gallone; D.Gallone; E Giombini; F Basile; F Messina; G Meoni; G Parisi; M Irno Consalvo; M Rueca; M. Rueca; MI Consalvo; ML Guarino; MR Capobianchi; O Butera
EPI_ISL_882937 to 882939, EPI_ISL_965131 to 965132	Azienda Ospedaliera San Giovanni Addolorata	INMI Lazzaro Spallanzani IRCCS	A Di Caro; B Bartolini; C.E.M Gruber; CEM Gruber; E Giombini; F Messina; M Gaudio; M Rueca; MR Capobianchi; O Butera; PM Placanica
EPI_ISL_1296263 to 1296267, EPI_ISL_1296269 to 1296278, EPI_ISL_1296280 to 1296287, EPI_ISL_1299501 to 1299520, EPI_ISL_1299522 to 1299526, EPI_ISL_1299563, EPI_ISL_1299570			
see above	Azienda Ospedaliera Terni	Istituto Zooprofilattico Sperimentale dell'Abruzzo e Molise "G. Caporale"	; Ancora M; Calistri P; Cammà C; Curini V; Di Domenico M; Di Pasquale A; Lorusso A; Mangone I; Marcacci M; Palumbo M; Palumbo m; Puglia I; Rinaldi A; Savini G; Scaccetti A; ScaccettiA; Scialabba S
EPI_ISL_1259009 to 1259018	Azienda Ospedaliero - Universitaria di Bologna IRCCS Policlinico di Sant'Orsola - Unità di Microbiologia	Istituto Zooprofilattico Sperimentale della Lombardia e dell'Emilia Romagna (IZSLER), Risk Analysis and Genomic Epidemiology Unit	Erika Scaltriti; Giada Rossini; Giuliano Furlini; Ilaria Menozzi; Marina Morganti; Stefano Pongolini; Tiziana Lazzarotto
EPI_ISL_1181933 to 1181945	Azienda Ospedaliero - Universitaria di Modena Policlinico - Virologia e Microbiologia Molecolare	Istituto Zooprofilattico Sperimentale della Lombardia e dell'Emilia Romagna (IZSLER), Risk Analysis and Genomic Epidemiology Unit	Erika Scaltriti; Giulia Fregni Serpini; Ilaria Menozzi; Marina Morganti; Monica Pecorari; Stefano Pongolini; William Gennari
EPI_ISL_1132668	Azienda Ospedaliero - Universitaria di Modena Policlinico - Virologia e Microbiologia Molecolare	Zooprofilattico Sperimentale dell'Emilia Romagna e della Lombardia (IZSLER), Risk Analysis and Genomic Epidemiology Unit	Erika Scaltriti; Giulia Fregni Serpini; Ilaria Menozzi; Marina Morganti; Monica Pecorari; Stefano Pongolini; William Gennari

EPI_ISL_1069204	Azienda Sanitaria Locale Napoli 1	Centro Polidiagnostico strumentale AMES	Sirica Roberto
EPI_ISL_965136, EPI_ISL_1295615	Azienda Sanitaria Locale Roma 5	INMI Lazzaro Spallanzani IRCCS	A Di Caro; B Bartolini; CEM Gruber; D Cerini; D Di Fusco; E Giombini; F Messina; F Santini; G Bonfiglio; M Rueca; MR Capobianchi; O Butera
EPI_ISL_1063404 to 1063481, EPI_ISL_1063491 to 1063492, EPI_ISL_1110295 to 1110388, EPI_ISL_1185777 to 1185827, EPI_ISL_1185829 to 1185846, EPI_ISL_1222783 to 1222785, EPI_ISL_1222787 to 1222797, EPI_ISL_1222799 to 1222820, EPI_ISL_1222822 to 1222829, EPI_ISL_1222831 to 1222843, EPI_ISL_1222845 to 1222861, EPI_ISL_1222863 to 1222866, EPI_ISL_1222868 to 1222869, EPI_ISL_1222871 to 1222886, EPI_ISL_1222888 to 1222891, EPI_ISL_1222893 to 1222914, EPI_ISL_1222916 to 1222922, EPI_ISL_1222924 to 1222926			
see above	Azienda Sanitaria dell'Alto Adige Laboratorio Aziendale di Microbiologia e Virologia	Istituto di Genomica Applicata	Davide Scaglione; Eleonora Paparelli; Elisa Masi; Elisabetta Giacobazzi; Elisabetta Pagani; Gabriele Magris; Irena Jurman; Irene Bianconi; Michele Morgante; Stefanie Wieser; Vera Vendramin
EPI_ISL_1013095	Azienda Sanitaria dell'Alto Adige Laboratorio Aziendale di Microbiologia e Virologia	Istituto di Genomica Applicata	Davide Scaglione; Eleonora Paparelli; Elisa Masi; Elisabetta Giacobazzi; Elisabetta Pagani; Irena Jurman; Irene Bianconi; Michele Morgante; Stefanie Wieser; Vera Vendramin
EPI_ISL_1015515 to 1015521, EPI_ISL_1015532 to 1015560, EPI_ISL_1048822 to 1048824, EPI_ISL_1048826 to 1048828	Azienda Sanitaria dell'Alto Adige Laboratorio Aziendale di Microbiologia e Virologia	Istituto di Genomica Applicata	Davide Scaglione; Eleonora Paparelli; Elisa Masi; Elisabetta Giacobazzi; Elisabetta Pagani; Gabriele Magris; Irena Jurman; Irene Bianconi; Michele Morgante; Stefanie Wieser; Vera Vendramin
EPI_ISL_1163796 to 1163797, EPI_ISL_1164754 to 1164756, EPI_ISL_1299500, EPI_ISL_1299521, EPI_ISL_1299532, EPI_ISL_1299543, EPI_ISL_1299554			
see above	Azienda USL Umbria 2	Istituto Zooprofilattico Sperimentale dell'Abruzzo e Molise "G. Caporale"	Ancora M; Calistri P; Cammà C; Curini V; Di Domenico M; Di Pasquale A; Lorusso A; Mangone I; Marcacci M; Pistoni E; Pistoni E.; Proietti A; Proietti A.; Puglia I; Rinaldi A; Savini G; Scialabba S
EPI_ISL_496521 to 496523, EPI_ISL_496527 to 496528, EPI_ISL_496530, EPI_ISL_496534, EPI_ISL_496546 to 496554, EPI_ISL_497888 to 497891, EPI_ISL_728254 to 728271, EPI_ISL_728325 to 728330			
see above	B.J. Govt. Medical College	National Centre For Cell Science	Arvind Sahu; DBT's PAN-INDIA 1000 SARS-CoV2 RNA genome sequencing consortium; Dhiraj Paul; Girdhari Lal; Janesh Kumar; Kavita Bala Anand; Kunal Jani; Maharashtra COVID-19 Study Group; Manoj Kumar Bhat; Murlidhar Tambe; Radha Chauhan; Rajesh Karyakarte; Rajiv Mohan Gupta; Santosh Karade; Shelinder Pal Singh Shergill; Sourav Sen; Suvarna Joshi; Vasudevan Seshadri; Yogesh S Shouche
EPI_ISL_435049 to 435054, EPI_ISL_437445 to 437454, EPI_ISL_444456 to 444481, EPI_ISL_447030 to 447046, EPI_ISL_458086 to 458102, EPI_ISL_461483 to 461506, EPI_ISL_467041 to 467054, EPI_ISL_469024 to 469028, EPI_ISL_495014 to 495019			
see above	B.J. Medical College and Civil hospital	Gujarat Biotechnology Research Centre	; A M Kadri; Afzal Ansari; Akanksha Verma; Amit Kanani; Anjali Rajwar; Ankit Hinsu; Apurvasinh Puvar; Armi Chaudhari; Bhavesh Modi; Bhavya Jindal; Binita Aring; Camellia Chakraborty; Chaitanya Joshi; Dhaval Vaghela; Dinesh Kumar; Dipa Kinariwala; Dipeshwari Shewale; Disha Patel; Fenil Patel; Gaurishankar Shrimali; Geeta Vaghela; Harsh Bakshi; Janvi Raval; Kairavi Joshi; Kamlesh J Upadhyay; Komal Patel; Labdhi Pandya; Madhvi Joshi; Maharshi Pandya; Monika Gandhi; Neelam Nathani; Neeta Khandelwal; Neha Rajpara; Nidhi Patel; Nidhi Sood; Nikha Trivedi; Nirav Mungalpara; Nitin Savaliya; Pinal Trivedi; Pooja P Doshi; Pragya Sharma; Pranay Shah; Pritesh Sabara; Priti Pandita; Priyanka P Vatsa; R D Dixit; Raghawendra Kumar; Ramesh Pandit; Ramesh Patel; Sanjay Kapadia; Sharmistha Majumdar; Siddhant Kumar; Snehal Bagatharia; Sonia Barve; Tejas Shah; Umang Mishra; Vasudha Sharma; Zarna Patel; Zuber Saiyed
EPI_ISL_1164932 to 1164933, EPI_ISL_1164935 to 1164939, EPI_ISL_1164943 to 1164945	B.J. Medical College and Civil hospital	Indian Council of Medical Research-National Institute of Virology, Microbial Containment Complex	Disha Patel; Pragya D. Yadav
EPI_ISL_512058 to 512065, EPI_ISL_514581 to 514609, EPI_ISL_524713 to 524729, EPI_ISL_525421 to 525422, EPI_ISL_586513 to 586530, EPI_ISL_590689, EPI_ISL_825034 to 825042			
see above	B.J. Medical College and Civil hospital, Ahmedabad	Gujarat Biotechnology Research Centre	A M Kadri; Afzal Ansari; Apurvasinh Puvar; Chaitanya Joshi; Dinesh Kumar; Dipa Kinariwala; Harsh Bakshi; Janvi Raval; Kamlesh J Upadhyay; Komal Patel; Labdhi Pandya; Madhvi Joshi; Maharshi Pandya; Monika Gandhi; Nidhi Patel; Nikha Trivedi; Nilima Shah; Nitin Savaliya; Pinal Trivedi; Pranay Shah; R D Dixit; Raghawendra Kumar; Ramesh Pandit; Sanjay Kapadia; Zarna Patel; Zuber Saiyed
EPI_ISL_995696, EPI_ISL_995708 to 995712, EPI_ISL_995724, EPI_ISL_995748, EPI_ISL_995763	BANGALORE MEDICAL COLLEGE AND RESEARCH INSTITUTE	Department of Neurovirology, National Institute of Mental Health and Neurosciences (NIMHANS)	Anita S Desai; Anson Kunjumon George; Chitra Pattabiraman; Darshan Sreenivas; Harsha.P.K; Nakka Vijay Kiran Reddy; Pramada Prasad; Risha Rasheed; V Ravi
EPI_ISL_747244, EPI_ISL_803901 to 803902, EPI_ISL_803904, EPI_ISL_803908, EPI_ISL_803911 to 803912, EPI_ISL_807158, EPI_ISL_825141 to 825143, EPI_ISL_825148, EPI_ISL_845874 to 845876, EPI_ISL_845878 to 845880, EPI_ISL_845883 to 845884, EPI_ISL_845887 to 845890, EPI_ISL_860189, EPI_ISL_860194 to 860195, EPI_ISL_860197, EPI_ISL_860199 to 860201, EPI_ISL_995173 to 995184, EPI_ISL_995704 to 995705, EPI_ISL_995715 to 995719, EPI_ISL_995725, EPI_ISL_995728 to 995729, EPI_ISL_995732, EPI_ISL_995735 to 995736, EPI_ISL_995739 to 995741, EPI_ISL_995746, EPI_ISL_995750 to 995752, EPI_ISL_995754 to 995757, EPI_ISL_1007660 to 1007661, EPI_ISL_1250853 to 1250858			
see above	BBMP Urban PHC	Department of Neurovirology, National Institute of Mental Health and Neurosciences (NIMHANS)	Anita S Desai; Anson Kunjumon George; Chitra Pattabiraman; Darshan Sreenivas; Harsha.P.K; Nakka Vijay Kiran Reddy; Pramada Prasad; Risha Rasheed; V Ravi
EPI_ISL_1257851 to 1257854	BBTKLPP Banjarbaru	National Institute of Health Research and Development	Arie Ardiansyah Nugraha; Dewi Hermawati; Hana Apsari Pawestri; Hartanti Dian Ikawati; Kartika Dewi Puspa; Krisna Pangesti; Mutia Raudah; Nelly Puspandari; Subangkit; Vivi Setiawaty
EPI_ISL_1169048	BBTKLPP Banjarbaru, Kalimantan Selatan	National Institute of Health Research and Development	Arie Ardiansyah Nugraha; Hana Apsari Pawestri; Hartanti Dian Ikawati; Kartika Dewi Puspa; Nelly Puspandari; Subangkit; Vivi Setiawaty
EPI_ISL_1257856 to 1257858	BBTKLPP Yogyakarta	National Institute of Health Research and Development	Arie Ardiansyah Nugraha; Hana Apsari Pawestri; Hartanti Dian Ikawati; Havid Setyawan; Indaryati; Irene; Kartika Dewi Puspa; Krisna Pangesti; Nelly Puspandari; Subangkit; Vivi Setiawaty
EPI_ISL_412965, EPI_ISL_415577 to 415590, EPI_ISL_418816 to 418859, EPI_ISL_460606 to 460616, EPI_ISL_462754 to 462844, EPI_ISL_463186 to 463276, EPI_ISL_466696 to 466838, EPI_ISL_467303, EPI_ISL_467305 to 467343, EPI_ISL_467423 to 467429, EPI_ISL_468657 to 468700, EPI_ISL_469209 to 469223, EPI_ISL_477017 to 477038, EPI_ISL_477064, EPI_ISL_477076 to 477119, EPI_ISL_740548 to 740862, EPI_ISL_968250 to 968251, EPI_ISL_968253 to 968663, EPI_ISL_968665, EPI_ISL_968667, EPI_ISL_968669, EPI_ISL_968671 to 968725, EPI_ISL_968727 to 968771, EPI_ISL_968774 to 968806, EPI_ISL_968809, EPI_ISL_968811 to 968812, EPI_ISL_968814, EPI_ISL_968818, EPI_ISL_968823 to 968824, EPI_ISL_968830, EPI_ISL_968833, EPI_ISL_968836, EPI_ISL_968840, EPI_ISL_968845, EPI_ISL_968848, EPI_ISL_968852, EPI_ISL_968859, EPI_ISL_968862, EPI_ISL_968866, EPI_ISL_968870, EPI_ISL_968872, EPI_ISL_968875 to 968876, EPI_ISL_968880, EPI_ISL_968886, EPI_ISL_968888, EPI_ISL_968893, EPI_ISL_968895, EPI_ISL_968900, EPI_ISL_968906, EPI_ISL_968912, EPI_ISL_968915, EPI_ISL_968918, EPI_ISL_968921, EPI_ISL_968923, EPI_ISL_968928, EPI_ISL_968931 to 968940, EPI_ISL_968944 to 968954, EPI_ISL_968960, EPI_ISL_968962 to 968971, EPI_ISL_968973 to 968983, EPI_ISL_968991, EPI_ISL_969000, EPI_ISL_969006, EPI_ISL_969015, EPI_ISL_969022, EPI_ISL_969032 to 969033, EPI_ISL_969035 to 969040, EPI_ISL_969045, EPI_ISL_969055, EPI_ISL_969062, EPI_ISL_969070, EPI_ISL_969075 to 969130, EPI_ISL_969132 to 969217, EPI_ISL_969219 to 969226, EPI_ISL_969228 to 969244, EPI_ISL_969246 to 969257, EPI_ISL_969259 to 969289, EPI_ISL_969291 to 969296, EPI_ISL_969298 to 969331, EPI_ISL_969333 to 969364, EPI_ISL_969366 to 969396, EPI_ISL_969398 to 969453, EPI_ISL_969470, EPI_ISL_969499, EPI_ISL_969547, EPI_ISL_969590, EPI_ISL_969601, EPI_ISL_969648, EPI_ISL_969676, EPI_ISL_969678, EPI_ISL_969680 to 969681, EPI_ISL_969684, EPI_ISL_969686 to 969687, EPI_ISL_969689, EPI_ISL_969692, EPI_ISL_969694, EPI_ISL_969696, EPI_ISL_969698, EPI_ISL_969700, EPI_ISL_969703, EPI_ISL_969705, EPI_ISL_969707, EPI_ISL_969709, EPI_ISL_969711, EPI_ISL_969713, EPI_ISL_969715, EPI_ISL_969717, EPI_ISL_969719, EPI_ISL_969721, EPI_ISL_969723, EPI_ISL_969725, EPI_ISL_969727, EPI_ISL_969729, EPI_ISL_969731, EPI_ISL_969733, EPI_ISL_969735, EPI_ISL_969737 to 969738, EPI_ISL_969741, EPI_ISL_969743, EPI_ISL_969745, EPI_ISL_969747, EPI_ISL_969749, EPI_ISL_969751, EPI_ISL_969753, EPI_ISL_969755, EPI_ISL_969757 to 969758, EPI_ISL_969761, EPI_ISL_969763, EPI_ISL_969765, EPI_ISL_969767, EPI_ISL_969770, EPI_ISL_969772 to 969773, EPI_ISL_969775, EPI_ISL_969777 to 969778, EPI_ISL_969781, EPI_ISL_969783 to 969784, EPI_ISL_969787 to 969788, EPI_ISL_969790, EPI_ISL_969792, EPI_ISL_969794, EPI_ISL_969796, EPI_ISL_969798, EPI_ISL_969800, EPI_ISL_969803, EPI_ISL_969805, EPI_ISL_969807, EPI_ISL_969810 to 969811, EPI_ISL_969814, EPI_ISL_969816, EPI_ISL_969818, EPI_ISL_969820, EPI_ISL_969822, EPI_ISL_969825, EPI_ISL_969827, EPI_ISL_969829 to 969830, EPI_ISL_969832, EPI_ISL_969835, EPI_ISL_969837, EPI_ISL_969839, EPI_ISL_969842, EPI_ISL_969844 to 969845, EPI_ISL_969847, EPI_ISL_969850 to 969851, EPI_ISL_969854 to 969855, EPI_ISL_969857, EPI_ISL_969860, EPI_ISL_969862, EPI_ISL_969864, EPI_ISL_969866, EPI_ISL_969868, EPI_ISL_969870, EPI_ISL_969872, EPI_ISL_969874, EPI_ISL_969876, EPI_ISL_969878, EPI_ISL_969880, EPI_ISL_969882, EPI_ISL_969885, EPI_ISL_969888, EPI_ISL_969890, EPI_ISL_969892, EPI_ISL_969894, EPI_ISL_969896, EPI_ISL_969898, EPI_ISL_969900, EPI_ISL_969902, EPI_ISL_969904 to 969905, EPI_ISL_969908, EPI_ISL_969910, EPI_ISL_969912 to 969913, EPI_ISL_969915, EPI_ISL_969918, EPI_ISL_969920, EPI_ISL_969922, EPI_ISL_969924, EPI_ISL_969927, EPI_ISL_969929, EPI_ISL_969931, EPI_ISL_969933, EPI_ISL_969935, EPI_ISL_969938, EPI_ISL_969941 to 969942, EPI_ISL_969944, EPI_ISL_969946, EPI_ISL_969949 to 969950, EPI_ISL_969953 to 969954, EPI_ISL_969957, EPI_ISL_969959 to 969960, EPI_ISL_969962, EPI_ISL_969964, EPI_ISL_969966, EPI_ISL_969968, EPI_ISL_969970, EPI_ISL_969972 to 969973, EPI_ISL_969976, EPI_ISL_969978, EPI_ISL_969980 to 969981, EPI_ISL_969984 to 969985, EPI_ISL_969987, EPI_ISL_969989, EPI_ISL_969991, EPI_ISL_969993, EPI_ISL_969995, EPI_ISL_969997, EPI_ISL_969999, EPI_ISL_970001, EPI_ISL_970003, EPI_ISL_970006, EPI_ISL_970008, EPI_ISL_970010, EPI_ISL_970012, EPI_ISL_970014, EPI_ISL_970016, EPI_ISL_970018, EPI_ISL_970020 to 970021, EPI_ISL_970023, EPI_ISL_970025, EPI_ISL_970027, EPI_ISL_970029, EPI_ISL_970031, EPI_ISL_970034, EPI_ISL_970036, EPI_ISL_970038, EPI_ISL_970040, EPI_ISL_970042, EPI_ISL_970044, EPI_ISL_970046, EPI_ISL_970048, EPI_ISL_970051, EPI_ISL_970053 to 970054, EPI_ISL_970056, EPI_ISL_970058, EPI_ISL_970060, EPI_ISL_970062, EPI_ISL_970065, EPI_ISL_970067, EPI_ISL_970069, EPI_ISL_970071 to 970072, EPI_ISL_970074, EPI_ISL_970077 to 970078, EPI_ISL_970081, EPI_ISL_970083, EPI_ISL_970085, EPI_ISL_970088, EPI_ISL_970090, EPI_ISL_970093, EPI_ISL_970095, EPI_ISL_970097, EPI_ISL_970100, EPI_ISL_970102, EPI_ISL_970104, EPI_ISL_970106, EPI_ISL_970108, EPI_ISL_970110, EPI_ISL_970112, EPI_ISL_970114, EPI_ISL_970116, EPI_ISL_970118, EPI_ISL_970121, EPI_ISL_970123, EPI_ISL_970125, EPI_ISL_970127, EPI_ISL_970129, EPI_ISL_970131, EPI_ISL_970133, EPI_ISL_970135, EPI_ISL_970137, EPI_ISL_970139, EPI_ISL_970141, EPI_ISL_970143 to 970144, EPI_ISL_970146, EPI_ISL_970149, EPI_ISL_970151, EPI_ISL_970154, EPI_ISL_970156, EPI_ISL_970158 to 970159, EPI_ISL_970162, EPI_ISL_970164, EPI_ISL_970167, EPI_ISL_970169, EPI_ISL_970171, EPI_ISL_970174, EPI_ISL_970176, EPI_ISL_970178, EPI_ISL_970180, EPI_ISL_970182, EPI_ISL_970185, EPI_ISL_970187, EPI_ISL_970189, EPI_ISL_970191, EPI_ISL_970193 to 970194, EPI_ISL_970196, EPI_ISL_970199, EPI_ISL_970201, EPI_ISL_970203 to 970204, EPI_ISL_970207 to 970208, EPI_ISL_970210, EPI_ISL_970212, EPI_ISL_970214, EPI_ISL_970216, EPI_ISL_970218, EPI_ISL_970220, EPI_ISL_970222 to 970223, EPI_ISL_970226, EPI_ISL_970228, EPI_ISL_970230, EPI_ISL_970232, EPI_ISL_970234, EPI_ISL_970236, EPI_ISL_970238, EPI_ISL_970240 to 970241, EPI_ISL_970243 to 970246, EPI_ISL_970248, EPI_ISL_970250, EPI_ISL_970253, EPI_ISL_970255, EPI_ISL_970257, EPI_ISL_970259, EPI_ISL_970261, EPI_ISL_970263, EPI_ISL_970265, EPI_ISL_970267, EPI_ISL_970269, EPI_ISL_970271, EPI_ISL_970273, EPI_ISL_970276 to 970277, EPI_ISL_970280, EPI_ISL_970282, EPI_ISL_970284, EPI_ISL_970286, EPI_ISL_970289 to 970290, EPI_ISL_970293 to 970294, EPI_ISL_970296, EPI_ISL_970298, EPI_ISL_970300, EPI_ISL_970302, EPI_ISL_970304 to 970305, EPI_ISL_970308, EPI_ISL_970310 to 970311, EPI_ISL_970313, EPI_ISL_970315, EPI_ISL_970317 to 970318, EPI_ISL_970320, EPI_ISL_970322, EPI_ISL_970325, EPI_ISL_970327, EPI_ISL_970329, EPI_ISL_970331, EPI_ISL_970333, EPI_ISL_970335 to 970336, EPI_ISL_970339, EPI_ISL_970341, EPI_ISL_970343, EPI_ISL_970345, EPI_ISL_970347, EPI_ISL_970349, EPI_ISL_970351, EPI_ISL_970353, EPI_ISL_970355, EPI_ISL_970357, EPI_ISL_970359, EPI_ISL_970361, EPI_ISL_970363, EPI_ISL_970366 to 970367, EPI_ISL_970369, EPI_ISL_970372, EPI_ISL_970374 to 970375, EPI_ISL_970378 to 970379, EPI_ISL_970381, EPI_ISL_970384 to 970385, EPI_ISL_970388 to 970389, EPI_ISL_970391, EPI_ISL_970393, EPI_ISL_970395, EPI_ISL_970397, EPI_ISL_970399, EPI_ISL_970401, EPI_ISL_970403, EPI_ISL_970405, EPI_ISL_970407 to 970408, EPI_ISL_970410, EPI_ISL_970413 to 970414, EPI_ISL_970417 to 970418, EPI_ISL_970420, EPI_ISL_970423, EPI_ISL_970425, EPI_ISL_970427, EPI_ISL_970429, EPI_ISL_970431, EPI_ISL_970433 to 970434, EPI_ISL_970437 to 970438, EPI_ISL_970440, EPI_ISL_970442, EPI_ISL_970445, EPI_ISL_970447, EPI_ISL_970449, EPI_ISL_970451, EPI_ISL_970453, EPI_ISL_970455 to 970456, EPI_ISL_970458 to 970459, EPI_ISL_970461, EPI_ISL_970463, EPI_ISL_970465, EPI_ISL_970467, EPI_ISL_970471 to 970472, EPI_ISL_970474, EPI_ISL_970476, EPI_ISL_970479 to 970480, EPI_ISL_970482, EPI_ISL_970484 to 970485, EPI_ISL_970487, EPI_ISL_970489, EPI_ISL_970491, EPI_ISL_970494, EPI_ISL_970496, EPI_ISL_970498, EPI_ISL_970501, EPI_ISL_970503, EPI_ISL_970505, EPI_ISL_970507, EPI_ISL_970509, EPI_ISL_970511, EPI_ISL_970514, EPI_ISL_970516, EPI_ISL_970518, EPI_ISL_970520, EPI_ISL_970522, EPI_ISL_970524, EPI_ISL_970526, EPI_ISL_970529, EPI_ISL_970531 to 970532, EPI_ISL_970534, EPI_ISL_970536, EPI_ISL_970538 to 970539, EPI_ISL_970542, EPI_ISL_970544 to 970545, EPI_ISL_970547, EPI_ISL_970549 to 970550, EPI_ISL_970552, EPI_ISL_970554, EPI_ISL_970556 to 970557, EPI_ISL_970559, EPI_ISL_970561, EPI_ISL_970563, EPI_ISL_970565, EPI_ISL_970567, EPI_ISL_970570, EPI_ISL_970572, EPI_ISL_970574, EPI_ISL_970576, EPI_ISL_970578, EPI_ISL_970580, EPI_ISL_970582, EPI_ISL_970584, EPI_ISL_970586, EPI_ISL_970588, EPI_ISL_970590,			

EPI_ISL_970592, EPI_ISL_970594, EPI_ISL_970596, EPI_ISL_970598, EPI_ISL_970600, EPI_ISL_970603 to 970604, EPI_ISL_970606, EPI_ISL_970608, EPI_ISL_970610 to 970611, EPI_ISL_970613, EPI_ISL_970615, EPI_ISL_970617, EPI_ISL_970620, EPI_ISL_970622, EPI_ISL_970624, EPI_ISL_970626 to 970627, EPI_ISL_970629, EPI_ISL_970631, EPI_ISL_970634, EPI_ISL_970636, EPI_ISL_970638, EPI_ISL_970640, EPI_ISL_970642, EPI_ISL_970644, EPI_ISL_970646, EPI_ISL_970649, EPI_ISL_970651, EPI_ISL_970653, EPI_ISL_970655, EPI_ISL_970657, EPI_ISL_970659, EPI_ISL_970662 to 970663, EPI_ISL_970665, EPI_ISL_970667, EPI_ISL_970669, EPI_ISL_970671 to 970672, EPI_ISL_970674, EPI_ISL_970676, EPI_ISL_970678 to 970679, EPI_ISL_970682, EPI_ISL_970684, EPI_ISL_970686, EPI_ISL_970688, EPI_ISL_970691, EPI_ISL_970693, EPI_ISL_970695 to 970696, EPI_ISL_970698, EPI_ISL_970700, EPI_ISL_970702, EPI_ISL_970705, EPI_ISL_970707, EPI_ISL_970710, EPI_ISL_970712, EPI_ISL_970714, EPI_ISL_970716, EPI_ISL_970718 to 970719, EPI_ISL_970721, EPI_ISL_970723, EPI_ISL_970725, EPI_ISL_970728, EPI_ISL_970730 to 970731, EPI_ISL_970733, EPI_ISL_970735, EPI_ISL_970737, EPI_ISL_970740, EPI_ISL_970742 to 970743, EPI_ISL_970745, EPI_ISL_970748, EPI_ISL_970750, EPI_ISL_970752, EPI_ISL_970754, EPI_ISL_970756, EPI_ISL_970759, EPI_ISL_970761, EPI_ISL_970763, EPI_ISL_970765, EPI_ISL_970767, EPI_ISL_970769, EPI_ISL_970771, EPI_ISL_970773, EPI_ISL_970776, EPI_ISL_970778, EPI_ISL_970780, EPI_ISL_970783, EPI_ISL_970785, EPI_ISL_970787, EPI_ISL_970789, EPI_ISL_970791, EPI_ISL_970793, EPI_ISL_970795, EPI_ISL_970798 to 970799, EPI_ISL_970801, EPI_ISL_970804, EPI_ISL_970806, EPI_ISL_970809, EPI_ISL_970818, EPI_ISL_970840, EPI_ISL_970856, EPI_ISL_970878, EPI_ISL_970902, EPI_ISL_970931, EPI_ISL_970947, EPI_ISL_970965, EPI_ISL_970995, EPI_ISL_971012, EPI_ISL_971036, EPI_ISL_971040, EPI_ISL_971043, EPI_ISL_971046, EPI_ISL_971049, EPI_ISL_971052, EPI_ISL_971054, EPI_ISL_971057, EPI_ISL_971061, EPI_ISL_971064, EPI_ISL_971068, EPI_ISL_971072, EPI_ISL_971075, EPI_ISL_971077, EPI_ISL_971080, EPI_ISL_971084, EPI_ISL_971086, EPI_ISL_971089, EPI_ISL_971091, EPI_ISL_971094, EPI_ISL_971097, EPI_ISL_971101, EPI_ISL_971104, EPI_ISL_971107, EPI_ISL_971111, EPI_ISL_971114, EPI_ISL_971116, EPI_ISL_971120, EPI_ISL_971122, EPI_ISL_971126, EPI_ISL_971129, EPI_ISL_971132, EPI_ISL_971137, EPI_ISL_971139, EPI_ISL_971141, EPI_ISL_971144, EPI_ISL_971147, EPI_ISL_971150, EPI_ISL_971154, EPI_ISL_971157, EPI_ISL_971160, EPI_ISL_971162, EPI_ISL_971167, EPI_ISL_971170, EPI_ISL_971173, EPI_ISL_971177, EPI_ISL_971179, EPI_ISL_971182, EPI_ISL_971187, EPI_ISL_971189, EPI_ISL_971192, EPI_ISL_971196, EPI_ISL_971199, EPI_ISL_971202, EPI_ISL_971205, EPI_ISL_971208, EPI_ISL_971212, EPI_ISL_971216, EPI_ISL_971219, EPI_ISL_971222, EPI_ISL_971225, EPI_ISL_971227, EPI_ISL_971230, EPI_ISL_971233, EPI_ISL_971235, EPI_ISL_971238, EPI_ISL_971240, EPI_ISL_971243, EPI_ISL_971245, EPI_ISL_971249, EPI_ISL_971253, EPI_ISL_971256, EPI_ISL_971259, EPI_ISL_971262, EPI_ISL_971265, EPI_ISL_971268, EPI_ISL_971271, EPI_ISL_971274, EPI_ISL_971276, EPI_ISL_971278, EPI_ISL_971282, EPI_ISL_971285, EPI_ISL_971288, EPI_ISL_971291, EPI_ISL_971294, EPI_ISL_971298, EPI_ISL_971300, EPI_ISL_971303, EPI_ISL_971305, EPI_ISL_971308, EPI_ISL_971311, EPI_ISL_971314, EPI_ISL_971317, EPI_ISL_971321, EPI_ISL_971325, EPI_ISL_971328, EPI_ISL_971331, EPI_ISL_971334, EPI_ISL_971337, EPI_ISL_971340, EPI_ISL_971342, EPI_ISL_971344, EPI_ISL_971348, EPI_ISL_971351, EPI_ISL_971354, EPI_ISL_971356, EPI_ISL_971359, EPI_ISL_971362, EPI_ISL_971367, EPI_ISL_971369, EPI_ISL_971372, EPI_ISL_971374, EPI_ISL_971377, EPI_ISL_971380, EPI_ISL_971383, EPI_ISL_971386, EPI_ISL_971390, EPI_ISL_971393, EPI_ISL_971396, EPI_ISL_971399, EPI_ISL_971403, EPI_ISL_971406, EPI_ISL_971409, EPI_ISL_971412, EPI_ISL_971414, EPI_ISL_971416, EPI_ISL_971421, EPI_ISL_971424, EPI_ISL_971427, EPI_ISL_971430, EPI_ISL_971432 to 971433, EPI_ISL_971435, EPI_ISL_971438, EPI_ISL_971440, EPI_ISL_971442, EPI_ISL_971444, EPI_ISL_971446, EPI_ISL_971448 to 971449, EPI_ISL_971453 to 971454, EPI_ISL_971457, EPI_ISL_971459, EPI_ISL_973477, EPI_ISL_973479, EPI_ISL_973481, EPI_ISL_973483, EPI_ISL_973485, EPI_ISL_973488, EPI_ISL_973490 to 973491, EPI_ISL_973494, EPI_ISL_973496, EPI_ISL_973498, EPI_ISL_973500, EPI_ISL_973502, EPI_ISL_973504, EPI_ISL_973506 to 973507, EPI_ISL_973509, EPI_ISL_973511, EPI_ISL_973513, EPI_ISL_973515, EPI_ISL_973517, EPI_ISL_973520, EPI_ISL_973522, EPI_ISL_973524, EPI_ISL_973526, EPI_ISL_973528, EPI_ISL_973530 to 973531, EPI_ISL_973534, EPI_ISL_973536 to 973537, EPI_ISL_973539, EPI_ISL_973541, EPI_ISL_973543, EPI_ISL_973545, EPI_ISL_973547, EPI_ISL_973549, EPI_ISL_973551 to 973552, EPI_ISL_973554, EPI_ISL_973556, EPI_ISL_973558, EPI_ISL_973560, EPI_ISL_973562, EPI_ISL_973564 to 973565, EPI_ISL_973567, EPI_ISL_973569, EPI_ISL_973571, EPI_ISL_973573, EPI_ISL_973575, EPI_ISL_973577, EPI_ISL_973579, EPI_ISL_973581, EPI_ISL_973583, EPI_ISL_973585, EPI_ISL_973587 to 973588, EPI_ISL_973590, EPI_ISL_973592, EPI_ISL_973594, EPI_ISL_973596, EPI_ISL_973598, EPI_ISL_973600, EPI_ISL_973602, EPI_ISL_973604, EPI_ISL_973606, EPI_ISL_973608, EPI_ISL_973610, EPI_ISL_973612 to 973613, EPI_ISL_973615, EPI_ISL_973617, EPI_ISL_973619 to 973620, EPI_ISL_973622, EPI_ISL_973625, EPI_ISL_973627, EPI_ISL_973630, EPI_ISL_973632, EPI_ISL_973634, EPI_ISL_973637, EPI_ISL_973639, EPI_ISL_973642 to 973643, EPI_ISL_973646, EPI_ISL_973649 to 973650, EPI_ISL_973653, EPI_ISL_973655, EPI_ISL_973657, EPI_ISL_973659, EPI_ISL_973661, EPI_ISL_973663, EPI_ISL_973665, EPI_ISL_973667, EPI_ISL_973669, EPI_ISL_973671, EPI_ISL_973673, EPI_ISL_973675, EPI_ISL_973678 to 973679, EPI_ISL_973681, EPI_ISL_973683, EPI_ISL_973685, EPI_ISL_973687, EPI_ISL_973690, EPI_ISL_973692, EPI_ISL_973695, EPI_ISL_973697, EPI_ISL_973700, EPI_ISL_973702, EPI_ISL_973705, EPI_ISL_973707, EPI_ISL_973709, EPI_ISL_973712, EPI_ISL_973714, EPI_ISL_973716, EPI_ISL_973718 to 973719, EPI_ISL_973721 to 973725, EPI_ISL_973727, EPI_ISL_973729, EPI_ISL_973731, EPI_ISL_973733, EPI_ISL_973736 to 973737, EPI_ISL_973740, EPI_ISL_973742, EPI_ISL_973745, EPI_ISL_973747, EPI_ISL_973749, EPI_ISL_973751, EPI_ISL_973753, EPI_ISL_973755, EPI_ISL_973758, EPI_ISL_973760, EPI_ISL_973764, EPI_ISL_973767, EPI_ISL_973769, EPI_ISL_973771, EPI_ISL_973775 to 973776, EPI_ISL_973779, EPI_ISL_973781, EPI_ISL_973783, EPI_ISL_973785, EPI_ISL_973788, EPI_ISL_973791, EPI_ISL_973794 to 973795, EPI_ISL_973797, EPI_ISL_973800, EPI_ISL_973802, EPI_ISL_973805, EPI_ISL_973807, EPI_ISL_973810, EPI_ISL_973813, EPI_ISL_973815, EPI_ISL_973818, EPI_ISL_973821, EPI_ISL_973823, EPI_ISL_973826, EPI_ISL_973828, EPI_ISL_973830, EPI_ISL_973832, EPI_ISL_973834, EPI_ISL_973837 to 974149, EPI_ISL_974151 to 974744, EPI_ISL_974746 to 974852, EPI_ISL_974854 to 975774, EPI_ISL_975776 to 976304, EPI_ISL_976306 to 976815, EPI_ISL_976817 to 976892, EPI_ISL_982299			
see above	BCCDC Public Health Laboratory	BCCDC Public Health Laboratory	Ana Pacagnella; Choi; Corrinne Ng; Dan Fornika; Gilmour; Harrigan; Hoang; Hope Lapointe; Inna Sekirov; Jinny Choi; John Tyson; Kamelian; Kim Macdonald; Kimia Kamelian; Krajden; Lapointe; Lee; Levett; Li; Linda Hoang; Loman; Loretta Janz; Mel Krajden; Natalie Prystajecky; Paul Levett; Prystajecky; Prystajecky Natalie; Quick; Richard Harrigan; Robert Azana; Robert Azana Terry Snutch; Sekirov; Shannon Russell; Snutch; Terry Snutch; Tyson
EPI_ISL_1008876 to 1008892, EPI_ISL_1041959 to 1041968, EPI_ISL_1095457	BCN-PEP-COV-2 Group - Fundació Lluita contra la SIDA i les malalties infeccioses	SeqCOVID-SPAIN consortium/IBV(CSIC)	Andrea Alemany Ortiz MD; Barbara Baro Sastre BsC MsC PhD; Camila González Beiras BsC MsC PhD; Marc Corbacho Monné MD; Martí Vall Mayans MD PhD and SeqCOVID-SPAIN consortium; María Ubals Cazorla MD; Oriol Mitjà i Villar MD PhD; Pau Rodó de Yebra BsC MsC
EPI_ISL_955248	BCO SANGRE CM LA RAZA	BIOBANCO / COCTI	Arias C; Borja-Aburto VH; Grajales-Muñiz C; Grande R; Isa P; López S; Muñoz-Medina JE; Ochoa Carrera LA; Rojas-Mendoza T; Santacruz Tinoco CE; Sánchez A; Taboada B
EPI_ISL_1015370, EPI_ISL_1015372, EPI_ISL_1015391	BCRM Cherbourg	IRBA, 2MI	EVRARD A.; GORGE O.; NOLENT F.; SARILAR V.
EPI_ISL_850945	BCSIR	Genomic Research Lab, BCSIR	Abu Sayeed Mohammad Mahmud; Barna Goswami; Eshrar Osman; Iffat Jahan; Md. Ahashan Habib; Md. Murshed Hasan Sarkar; Md. Saddam Hossain; Md. Salim Khan.; Mohammad Samir Uzzaman; Shahina Akter; Tanjina Akhtar Banu
EPI_ISL_486395, EPI_ISL_515933, EPI_ISL_515937, EPI_ISL_516075	BIMS	Department of Neurovirology, National Institute of Mental Health and Neuroscience (NIMHANS)	Anita Desai; Chitra Pattabiraman; Harsha PK; Manjunatha Venkataswamy; Pramada Prasad; Ravi Vasanthapuram; Risha Rasheed; Shafeeq S Hameed; Vijayalakshmi Reddy
EPI_ISL_804981 to 804984, EPI_ISL_830720 to 830723, EPI_ISL_854350 to 854367, EPI_ISL_854369, EPI_ISL_854372, EPI_ISL_861175 to 861182, EPI_ISL_861185, EPI_ISL_861187, EPI_ISL_861210 to 861232, EPI_ISL_896297, EPI_ISL_896300, EPI_ISL_1226551, EPI_ISL_1226556, EPI_ISL_1226571 to 1226572, EPI_ISL_1226574, EPI_ISL_1226577, EPI_ISL_1226583, EPI_ISL_1226622, EPI_ISL_1226645 to 1226646, EPI_ISL_1226658, EPI_ISL_1226669, EPI_ISL_1226932, EPI_ISL_1226985			
see above	BIO-REFERENCE LABORATORIES	Wadsworth Center, New York State Department of Health	Alexis Russel; Daryl M. Lamson; Erasmus Schneider; Erica Lasek-Nesselquist; John Kelly; Jonathan Plitnick; Kirsten St. George; Matthew Shudt; Melissa A Leisner; Navjot Singh
EPI_ISL_767648, EPI_ISL_767650 to 767653, EPI_ISL_767665 to 767680, EPI_ISL_802430, EPI_ISL_802445 to 802447, EPI_ISL_802790 to 802793, EPI_ISL_804946			
see above	BIO-REFERENCE LABORATORIES	Wadsworth Center, New York State Department.of Health	Alexis Russel; Daryl M. Lamson; Erasmus Schneider; Erica Lasek-Nesselquist; John Kelly; Jonathan Plitnick; Kirsten St. George; Matthew Shudt; Melissa A Leisner; Navjot Singh; Sara Griesemer
EPI_ISL_1017704	BIO-TEST	Instituto Nacional de Salud- Dirección de Investigación en Salud Pública	Carlos Franco-Muñoz; Diego A. Álvarez-Díaz; Diego Andrés Prada; Gerardo Santamaría; Hector Alejandro Ruiz-Moreno; Jhonnatan Reales-González; Julian Naizaque; Katherine Laiton-Donato; Magdalena Wiesner; Marcela Mercado-Reyes; Maria T. Herrera-Sepúlveda; Martha Lucia Ospina Martinez; Sheryll Corchuelo
EPI_ISL_1190805 to 1190810	BIOAXIOME SITE SAINT GILLES	CNR Virus des Infections Respiratoires - France SUD	Antonin Bal; Bruno Lina; Gregory Destras; Gwendolyne Burfin; Hadrien Règue; Laurence Josset; Martine Valette; Quentin Semanas
EPI_ISL_1165934 to 1165935, EPI_ISL_1165950, EPI_ISL_1166082, EPI_ISL_1166084 to 1166093, EPI_ISL_1220097 to 1220181, EPI_ISL_1253560	BIOGROUP	CNR Virus des Infections Respiratoires - France SUD	Antonin Bal; Bruno Lina; Gregory Destras; Gwendolyne Burfin; Hadrien Regue; Laurence Josset; Martine Valette; Quentin Semanas
EPI_ISL_1163914 to 1163930, EPI_ISL_1163932 to 1163942, EPI_ISL_1163944, EPI_ISL_1163946 to 1163947, EPI_ISL_1163949 to 1163952, EPI_ISL_1163954 to 1163958, EPI_ISL_1163960 to 1163961, EPI_ISL_1163963 to 1163965, EPI_ISL_1163967 to 1163984			
see above	BIOGROUP BIOLITTORAL	CNR Virus des Infections Respiratoires - France SUD	Antonin Bal; Bruno Lina; Gregory Destras; Gwendolyne Burfin; Hadrien Regue; Laurence Josset; Martine Valette; Quentin Semanas
EPI_ISL_1166051 to 1166052	BIOLAB 33	CNR Virus des Infections Respiratoires - France SUD	Antonin Bal; Bruno Lina; Gregory Destras; Gwendolyne Burfin; Hadrien Regue; Laurence Josset; Martine Valette; Quentin Semanas
EPI_ISL_1190840	BIOLITTORAL BIOGROUP PLATEAU TECH	CNR Virus des Infections Respiratoires - France SUD	Antonin Bal; Bruno Lina; Gregory Destras; Gwendolyne Burfin; Hadrien Règue; Laurence Josset; Martine Valette; Quentin Semanas
EPI_ISL_1134907, EPI_ISL_1134915 to 1134957, EPI_ISL_1134959 to 1134960, EPI_ISL_1134962 to 1134964, EPI_ISL_1134966, EPI_ISL_1134970, EPI_ISL_1134972, EPI_ISL_1134975 to 1134977, EPI_ISL_1134979 to 1134984, EPI_ISL_1134986 to 1135026, EPI_ISL_1135029 to 1135037, EPI_ISL_1155702, EPI_ISL_1155704, EPI_ISL_1155706, EPI_ISL_1155708, EPI_ISL_1191635, EPI_ISL_1191639, EPI_ISL_1191644, EPI_ISL_1191647, EPI_ISL_1191649, EPI_ISL_1191651 to 1191652, EPI_ISL_1191659, EPI_ISL_1191661, EPI_ISL_1191663 to 1191666, EPI_ISL_1191684, EPI_ISL_1191686 to 1191687, EPI_ISL_1191694, EPI_ISL_1191700, EPI_ISL_1191702, EPI_ISL_1191707 to 1191708, EPI_ISL_1200657 to 1200659, EPI_ISL_1200667, EPI_ISL_1200669, EPI_ISL_1200677, EPI_ISL_1200691, EPI_ISL_1200698, EPI_ISL_1200702, EPI_ISL_1200705, EPI_ISL_1200711 to 1200713, EPI_ISL_1200717 to 1200718, EPI_ISL_1200721 to 1200723, EPI_ISL_1200725 to 1200726, EPI_ISL_1200731, EPI_ISL_1200733 to 1200734, EPI_ISL_1200736, EPI_ISL_1200740, EPI_ISL_1200744 to 1200748, EPI_ISL_1200750, EPI_ISL_1200753, EPI_ISL_1200755, EPI_ISL_1200761, EPI_ISL_1200779 to 1200781, EPI_ISL_1200784, EPI_ISL_1200786 to 1200789, EPI_ISL_1200792 to 1200793, EPI_ISL_1200795 to 1200796, EPI_ISL_1200798 to 1200799, EPI_ISL_1200801 to 1200802, EPI_ISL_1200804, EPI_ISL_1200806, EPI_ISL_1200808 to 1200810, EPI_ISL_1200812 to 1200814, EPI_ISL_1200816 to 1200820, EPI_ISL_1200822, EPI_ISL_1200824 to 1200825, EPI_ISL_1200827, EPI_ISL_1200829 to 1200831, EPI_ISL_1200833, EPI_ISL_1200847, EPI_ISL_1200849 to 1200850, EPI_ISL_1200852, EPI_ISL_1200854, EPI_ISL_1201787, EPI_ISL_1201789, EPI_ISL_1201791, EPI_ISL_1201815 to 1201819, EPI_ISL_1201823, EPI_ISL_1201825, EPI_ISL_1201828, EPI_ISL_1201831 to 1201842, EPI_ISL_1220200 to 1220303, EPI_ISL_1239445, EPI_ISL_1239449 to 1239450, EPI_ISL_1241866, EPI_ISL_1241880, EPI_ISL_1241891 to 1241895, EPI_ISL_1241897, EPI_ISL_1241901, EPI_ISL_1241903, EPI_ISL_1241906, EPI_ISL_1241909, EPI_ISL_1241912, EPI_ISL_1241915, EPI_ISL_1241919, EPI_ISL_1241923, EPI_ISL_1241941 to 1241942, EPI_ISL_1241944 to 1241945, EPI_ISL_1241947 to 1241949, EPI_ISL_1253538 to 1253542, EPI_ISL_1253544 to 1253547, EPI_ISL_1253549 to 1253551, EPI_ISL_1253558 to 1253559, EPI_ISL_1265475, EPI_ISL_1265478, EPI_ISL_1265480, EPI_ISL_1265484, EPI_ISL_1265492, EPI_ISL_1265495, EPI_ISL_1265503 to 1265505, EPI_ISL_1265508, EPI_ISL_1265518 to 1265545, EPI_ISL_1265547, EPI_ISL_1265557 to 1265569, EPI_ISL_1265572 to 1265577, EPI_ISL_1265587 to 1265590, EPI_ISL_1265607, EPI_ISL_1265715, EPI_ISL_1290837, EPI_ISL_1290841 to 1290846, EPI_ISL_1290849 to 1290854, EPI_ISL_1297204, EPI_ISL_1297206, EPI_ISL_1297215, EPI_ISL_1297217, EPI_ISL_1297228, EPI_ISL_1297319 to 1297320, EPI_ISL_1297322 to 1297334, EPI_ISL_1297338, EPI_ISL_1297340 to 1297343, EPI_ISL_1297345 to 1297383, EPI_ISL_1297423 to 1297434, EPI_ISL_1297439, EPI_ISL_1297441 to 1297453, EPI_ISL_1297460 to 1297471, EPI_ISL_1297480 to 1297486, EPI_ISL_1312377 to 1312381, EPI_ISL_1313052 to 1313079, EPI_ISL_1313085 to 1313090, EPI_ISL_1313093 to 1313113, EPI_ISL_1313118 to 1313119, EPI_ISL_1313157 to 1313159, EPI_ISL_1313161 to 1313162			
see above	BIOMNIS LYON	CNR Virus des Infections Respiratoires - France SUD	Antonin Bal; Bruno Lina; Gregory Destras; Gwendolyne Burfin; Hadrien Regue; Laurence Josset; Martine Valette; Quentin Semanas
EPI_ISL_1134908 to 1134914, EPI_ISL_1191680, EPI_ISL_1297205, EPI_ISL_1297207 to 1297209, EPI_ISL_1297211 to 1297214, EPI_ISL_1297218 to 1297227, EPI_ISL_1297229 to 1297306, EPI_ISL_1297308 to 1297318, EPI_ISL_1297321, EPI_ISL_1297385 to 1297420, EPI_ISL_1297456 to 1297459, EPI_ISL_1297472 to 1297479, EPI_ISL_1297487 to 1297488, EPI_ISL_1312367 to 1312376, EPI_ISL_1313080 to 1313081			
see above	BIOMNIS PARIS	CNR Virus des Infections Respiratoires - France SUD	Antonin Bal; Bruno Lina; Gregory Destras; Gwendolyne Burfin; Hadrien Regue; Laurence Josset; Martine Valette; Quentin Semanas
EPI_ISL_1219159 to 1219193, EPI_ISL_1219288 to 1219295, EPI_ISL_1219297 to 1219301, EPI_ISL_1219308 to 1219309, EPI_ISL_1219314, EPI_ISL_1219317, EPI_ISL_1219352 to 1219392, EPI_ISL_1219412 to 1219457, EPI_ISL_1219472 to 1219476, EPI_ISL_1219478 to 1219483, EPI_ISL_1219489, EPI_ISL_1219493 to 1219502, EPI_ISL_1219609 to 1219657, EPI_ISL_1312730 to 1312771			

see above	BIOR	Latvian Biomedical Research and Study Centre	Daina Pule; Davids Fridmanis; Guntars Zarins; Irena Meistere; Ivars Silamikelis; Janis Klovins; Janis Pjalkovskis; Jurijs Perevoscikovs; Kaspars Megnis; Laila Silamikele; Lauma Freimane; Laura Ansone; Liga Birzniece; Monta Ustinova; Nikita Zrelovs; Uga Dumpis; Una Krumina; Vita Rovite
EPI_ISL_1123259, EPI_ISL_1123262	BKH Schwaz	Institute of Virology Department of Hygiene, Microbiology and Public Health at Innsbruck Medical University	Andre Volland; Barbara Falkensammer; David Bante; Dorothee von Laer; Heribert Stoiber; Janine Kimpel; Lisa Pipperger; Lydia Riepler; Wegene Borena
EPI_ISL_830214, EPI_ISL_854446, EPI_ISL_883425, EPI_ISL_883429 to 883430, EPI_ISL_1226540	BOSTON HEART DIAGNOSTICS CORP	Wadsworth Center, New York State Department of Health	Alexis Russel; Daryl M. Lamson; Erasmus Schneider; Erica Lasek-Nesselquist; John Kelly; Jonathan Plitnick; Kirsten St. George; Matthew Shudt; Melissa A Leisner; Navjot Singh
EPI_ISL_1241972 to 1241973, EPI_ISL_1241990 to 1241991, EPI_ISL_1253568 to 1253573, EPI_ISL_1253577 to 1253578, EPI_ISL_1265482, EPI_ISL_1265585 to 1265586	BOURG LES VALENCE	CNR Virus des Infections Respiratoires - France SUD	Antonin Bal; Bruno Lina; Gregory Destras; Gwendolyne Burfin; Hadrien Regue; Laurence Josset; Martine Valette; Quentin Semanas
EPI_ISL_1257855	BPOM Mamuju	National Institute of Health Research and Development	Arie Ardiansyah Nugraha; Hana Apsari Pawestri; Hartanti Dian Ikawati; Kartika Dewi Puspa; Krisna Pangesti; Nelly Puspandari; Subangkit; Vivi Setiawaty
EPI_ISL_1231328 to 1231339, EPI_ISL_1231351 to 1231364	BPS GOVT. Medical college for women,Khanapur Kalan,Sonepat	Indian Council of Medical Research-National Institute of Virology, Microbial Containment Complex	Pragya D. Yadav; Sarita Yadav
EPI_ISL_1239672, EPI_ISL_1273838	BRUSS	1. National Institute of Public Health - National Institute of Hygiene; 2. Eurofins Genomics Europe Sequencing GmbH	ECDC COVID-19 WGS support team; Eurofins Genomics Europe Sequencing Team; Gierczyki Rafa; Sadkowska-Todys Magorzata; Wokowicz Tomasz; Zacharczuk Katarzyna
EPI_ISL_1259400, EPI_ISL_1260834 to 1260836	BSL-3 Lab, National Institute for Viral Disease Control and Prevention, Chinese Center for Disease Control and Prevention	BSL-3 Lab, National Institute for Viral Disease Control and Prevention, Chinese Center for Disease Control and Prevention	Dayan Wang; George F. Gao; Guizhen Wu; Jing Zhang; Jun Han; Peipei Liu; Shumei Zou; Xiang Zhao; Yuchao Wu; Zhixiao Chen
EPI_ISL_500716	BSL3 Lab Pendik Veterinary Control Institute	Department of Medicinal Genetics, Bursa Uluda University, Faculty of medicine By Sehime Gülsün Temel, Adem Alemdar, Kadir Yeilba	Ahmet SAIT; Cumhur ADIAY; Fahriye SARAC; Hakan ENUL; Kadir YESILBAG; Mustafa HASOKSUZ; Oguz KARABEY; Orbay SAYI; Osman ERGANIS; Serdar UZAR
EPI_ISL_491476	BSL3 Lab, Pendik Veterinary Control Enstitue	Genomic Laboratory (GLAB), Istanbul Technical University	Ahmet SAIT; Cumhur ADIAY; Fahriye SARAC; Hakan ENUL; Kadir YESILBAG; Mustafa HASOKSUZ; Oguz KARABEY; Orbay SAYI; Osman ERGANIS; Serdar UZAR
EPI_ISL_1020186, EPI_ISL_1137600	BSL3 Laboratory, Bogor, West Java	Biosafety Level-3 Laboratory, Indonesian Institute of Sciences (LIPI)	Anggia Prasetyoputri; Eko Wahyu Putro; Gita Syahputra; Isa Nuryana; Ratih Asmana Ningrum; Sri Swasthikawati; Sugiyono Saputra; Syam Budi Iryanto
EPI_ISL_918496	BSWMC-Marble Falls	BSWMC-Temple Molecular	Ari Rao; Kimberly Walker; Linden Morales; Shelby Hendrickson
EPI_ISL_859561 to 860091	BTC, Khalifa University	BTC, Khalifa University	Al Safar et al
EPI_ISL_1257832, EPI_ISL_1257864	BTKL-PP Ambon	National Institute of Health Research and Development	Arie Ardiansyah Nugraha; Hana Apsari Pawestri; Hartanti Dian Ikawati; Kartika Dewi Puspa; Krisna Pangesti; Nelly Puspandari; Subangkit; Vivi Setiawaty
EPI_ISL_1263459	BTKLPP Ambon	National Institute of Health Research and Development	Arie Ardiansyah Nugraha; Hana Apsari Pawestri; Hartanti Dian Ikawati; Kartika Dewi Puspa; Krisna Pangesti; Nelly Puspandari; Subangkit; Vivi Setiawaty
EPI_ISL_1265441 to 1265462	BTKLPP Kelas I Makassar	Eijkman Institute for Molecular Biology, Ministry of Research and Technology/National Agency for Research and Innovation; National Institute of Health Research and Development	Amin Soebandrio; Edison Johar; Frilasita A Yudhaputri; Hana Apsari Pawestri; Hidayat Trimarsanto; Iskandar Adnan; Khin Saw Myint; Lydia V. Panggalo; Safarina G Malik; Slamet; Sukma Oktavianthi; Vivi Setiawaty; Willy Agustine
EPI_ISL_574619 to 574623	BTKLPP Kelas I Manado	Eijkman Institute for Molecular Biology, Ministry of Research and Technology/National Agency for Research and Innovation	Amin Soebandrio; David H Muljono; Edison Johar; Frilasita A Yudhaputri; Herawati Sudoyo; Hidayat Trimarsanto; Iskandar A Adnan; Khin Saw Myint; Safarina G Malik; Willy Agustine
EPI_ISL_445362	BUPA SERVICIOS CLINICOS S.A	Instituto de Salud Publica de Chile	Alejandra Acevedo; Andrés E Castillo; Bárbara Parra; Carolina Tambley; Gabriel Leal; Jaime Lagos; Jorge Fernandez; Loredana Arata; Patricia Bustos; Paz Tapia; Rodrigo Fasce; Winston Andrade
EPI_ISL_884857 to 884863	Bacteriology, Georgia Public Health Laboratory (GPHL)	Bacteriology, Georgia Public Health Laboratory (GPHL)	Dixey, C.; Edwards, J.; Parrott, T.; Reeves, S.
EPI_ISL_1081797 to 1081809, EPI_ISL_1081823 to 1081826	Baguio General Hospital and Medical Center	Philippine Genome Center	Alethea R. de Guzman; Anna Ong-Lim; Arianne A. Zamora; Asia Louisa U. Chong; Benedict A. Maralit; Candice Francheska B. Tambaoan; Carlo M. Lapid; Celia Carlos; Cynthia P. Saloma; Devon Ray Pacial; Edsel Maurice Salvaña; El King D. Morado; Eva Maria Cutiongco-de la Paz; Francis A. Tablizo; Irish Coleen A. Asin; Jaime C. Montoya; Jan Michael C. Yap; Jo-Hannah S. Llames; John Q. Wong; Joshua Gregor A. Dizon; Juan Antonio R. Magalang; Karol Sophia Agape R. Padilla; Kenneth M. Kim; Kris P. Punayan; Marc Edsel C. Ayes; Marc Jerrone R. Castro; Maria Sofia L. Yangzon; Marissa Alejandria; Razel Nikka M. Hao; Rianna Patricia S. Cruz; Sheila Mae M. Araiza; and Maria Rosario Singh-Vergeire
EPI_ISL_1159375	Bahman Hospital	Laboratory of Molecular Biology and Cancer Immunology,Lebanese University Public Health England	Fadi Abdel Sater; Steven Pullan
EPI_ISL_791988	Balai Besar POM Semarang	National Institute of Health Research and Development	AA; Aryanti; HA; HD; Ikawati; KD; KNA; N; Nugraha; Pangesti; Pawestri; Puspa; Puspandari; Setiawaty; Soekarso; Subangkit; T; V
EPI_ISL_1020199	Balai Besar Rehabilitasi (BNN)	Biosafety Level-3 Laboratory, Indonesian Institute of Sciences (LIPI)	Alfi T. Fathurahman; Idris; Linda Sukmarini; Masrukhin; Ratih Asmana Ningrum; Ruby Setiawan; Sri Swasthikawati; Sugiyono Saputra
EPI_ISL_775599	Balai Besar Veteriner Maros	National Institute of Health Research and Development	AA; HA; HD; Ikawati; KD; KNA; Mangidi; Muflihanah; N; Nugraha; Pangesti; Pawestri; Puspa; Puspandari; R; Setiawaty; Soekarso; Subangkit; T; V
EPI_ISL_753698	Balai Besar Veteriner Maros, Sulawesi Selatan	National Institute of Health Research and Development	AA; HA; HD; Ikawati; KD; KNA; Mangidi; Muflihanah; N; Nugraha; Pangesti; Pawestri; Puspa; Puspandari; R; Setiawaty; Soekarso; Subangkit; T; V
EPI_ISL_791978	Balai Labkes Lampung	National Institute of Health Research and Development	AA; HA; HD; Ikawati; KD; KNA; L; N; Nugraha; Pangesti; Pawestri; Puspa; Puspandari; Setiawaty; Soekarso; Subangkit; T; V; Yurina
EPI_ISL_791981	Balai Litbang Aceh	National Institute of Health Research and Development	AA; E; F; Fitria; HA; HD; Ichwansyah; Ikawati; KD; KNA; N; Nugraha; Pangesti; Pawestri; Puspa; Puspandari; Setiawaty; Soekarso; Subangkit; T; V
EPI_ISL_1257818 to 1257822	Balai Litbangkes Papua	National Institute of Health Research and Development	Antonius Octavian; Arie Ardiansyah Nugraha; Hana Apsari Pawestri; Hartanti Dian Ikawati; Hotma Sitompul; Kartika Dewi Puspa; Krisna Pangesti; Nelly Puspandari; Subangkit; Vivi Setiawaty
EPI_ISL_1257830 to 1257831	Balai Litbangkes Tanah Bumbu	National Institute of Health Research and Development	Arie Ardiansyah Nugraha; Hana Apsari Pawestri; Hartanti Dian Ikawati; Juhairiyah; Kartika Dewi Puspa; Krisna Pangesti; Nelly Puspandari; Subangkit; Syarif Hidayat; Vivi Setiawaty
EPI_ISL_538506 to 538507, EPI_ISL_538512, EPI_ISL_1048406	Balai Penelitian dan Pengembangan Biomedis Papua	National Institute of Health Research and Development	A; AA; Anton Oktavianus; Arie Ardiansyah Nugraha; HA; HD; HML; Hana Apsari Pawestri; Hartanti Dian Ikawati; Hotma Sitompul; Hutapea; Ikawati; KD; KNA; Kartika Dewi Puspa; Krisna Nur Andriana Pangesti; M; Nelly Puspandari; Nugraha; Oktavian; Paisal; Pangesti; Pasaribu; Pawestri; Puspa; Setiawaty, V.; Soekarso; Subangkit; T; Vivi Setiawaty.
EPI_ISL_775594	Balitvet Lampung	National Institute of Health Research and Development	AA; E; EA; HA; HD; Ikawati; KD; KNA; N; Nugraha; Pangesti; Pawestri; Puspa; Puspandari; Saswiyanti; Setiawaty; Soekarso; Srihanto; Subangkit; T; V
EPI_ISL_1302465 to 1302472, EPI_ISL_1302653 to 1302658	Baltic Medics	Lithuanian University of Health Sciences Hospital, Department of Genetics and Molecular Medicine	Astra Vitkauskiene; Darius Cereskevicius; Inga Nasvytiene; Mantas Sarauskas; Marius Sukys; Rasa Ugenskiene; Renaldas Jurkevicius; Zivile Zemeckiene
EPI_ISL_403962 to 403963	Bamrasnaradura Hospital	1. Department of Medical Sciences, Ministry of Public Health, Thailand 2. Thai Red Cross Emerging Infectious Diseases - Health Science Centre 3. Department of Disease Control, Ministry of Public Health, Thailand	Buathong; Chittaganpitch; Malinee; Mekha; Nanthawan; Okada; Parnmen; Phuygun; Pilailuk; Rome; Siripaporn; Sittiporn; Sunthareeya; Supaporn; Thanadachakul; Thanutsapa; Wacharapluesadee; Waicharoen; Warawan; Wongboot
EPI_ISL_635199	Bamrasnaradura Infectious Disease Institute	Molecular HIV and Emerging Infectious Diseases Laboratory	Sumonmal UTTAYAMAKUL
EPI_ISL_434692 to 434694, EPI_ISL_434696,	Bamrasnaradura hospital	National Institute of Health. Department of medical Sciences,	Chittaganpitch; Malinee; Okada; Parnmen; Phuygun; Pilailuk; Siripaporn; Sittiporn; Sunthareeya; Thanadachakul; Thanutsapa; Waicharoen; Warawan;

EPI_ISL_515469		Ministry of Public Health, Thailand	Wongboot
EPI_ISL_469048, EPI_ISL_475026 to 475029, EPI_ISL_476867 to 476868	Banas Medical College and Research Institute	Gujarat Biotechnology Research Centre	A M Kadri; Afzal Ansari; Ankit Hinsu; Apurvasinh Puvar; Chaitanya Joshi; Dinesh Kumar; Fenil Patel; Harsh Bakshi; Janvi Raval; Komal Patel; Labdhi Pandya; Madhvi Joshi; Maharshi Pandya; Monika Gandhi; Neelam Nathani; Neha Rajpara; Nidhi Patel; Nikha Trivedi; Nitin Savaliya; Pinal Trivedi; Pritesh Sabara; Priti Pandita; R D Dixit; Radhika Khara; Raghawendra Kumar; Snehal Bagatharia; Sunil R Joshi; Tejas Shah; Viren s Doshi; Zarna Patel; Zuber Saiyed
EPI_ISL_825144, EPI_ISL_860182 to 860186	Bangalore Medical College and Research Institute	Department of Neurovirology, National Institute of Mental Health and Neurosciences (NIMHANS)	Anita S Desai; Anson Kunjumon George; Chitra Pattabiraman; Darshan Sreenivas; Nakka Vijay Kiran Reddy; Pramada Prasad; Risha Rasheed; V Ravi
EPI_ISL_450339 to 450345	Bangladesh Institute of Tropical & Infectious Diseases, COVID-19 Testing Laboratory	Basic and Applied Research on Jute Project	A S M Anwarul Huq; AMAM Zonaed Siddiki; Eaftekhar Ahmed Rana; Emdadul Mannan Emdad; Goutam Buddha Das; M A Hassan Chowdhury; Md. Monjurul Alam; Md. Nazmul Haq Rony; Md. Sabbir Hossain; Md. Samiul Haque; Md. Shahidul Islam; Md. Shakeel Ahmed; Md. Sharifur Rahman; Paritous Kumar Biswas; Rasel Ahmed; Shah Md Tamim Kabir
EPI_ISL_699484, EPI_ISL_708721 to 708722, EPI_ISL_708736	Bangpakok 9 international hospital	National Institute of Health, Department of Medical Sciences, Ministry of Public Health, Thailand	Malinee Chittaganpitch; Pilailuk Okada; Siripaporn Phuygun; Sittiporn Parnmen; Sunthareeya Waicharoen; Thanutsapa Thanadachakul; Warawan Wongboot
EPI_ISL_912162	Baptist Medical Center	Grubaugh Lab - Yale School of Public Health	Anderson Brito; Anne Wyllie; Annie Watkins; Chaney Kalinich; Chantal Vogels; Isabel Ott; Joseph Fauver; Mallery Breban; Mary Petrone; Nathan Grubaugh; Tara Alpert
EPI_ISL_539886, EPI_ISL_710613, EPI_ISL_732657, EPI_ISL_766645	Barnakuten	The Public Health Agency of Sweden	Anna Risberg; Anna-Malin Linde; Department of Microbiology; Karin Tegmark-Wisell; Maria Lind Karlberg; Mattias Haukland; Mia Brytting; Olov Svartstrom; Oskar Karlsson Lindsjo; Reza Advani; Sandra Broddesson; The Public Health Agency of Sweden; Theresa Enkirch
EPI_ISL_855549, EPI_ISL_1265369	Barts Health NHS Trust	Barts Health NHS Trust	BROAD; Beatrix; CUTINO-MOGUEL; Claire; David; Dola; Esin; HARRINGTON; KARAA; KELE; KULASEGARAN-SHYLINI; Maria-Teresa; OWOYEMI; Raghavendran
EPI_ISL_842793 to 843150, EPI_ISL_865488 to 865583, EPI_ISL_919779 to 919855, EPI_ISL_949748 to 949784, EPI_ISL_997615 to 997658, EPI_ISL_997662 to 997667, EPI_ISL_997669 to 997676, EPI_ISL_1048053 to 1048060, EPI_ISL_1048062 to 1048064, EPI_ISL_1048066 to 1048067, EPI_ISL_1048069 to 1048072, EPI_ISL_1048074, EPI_ISL_1048076 to 1048084, EPI_ISL_1048088 to 1048102, EPI_ISL_1048104 to 1048135, EPI_ISL_1050356, EPI_ISL_1177989 to 1177990, EPI_ISL_1177994 to 1178008, EPI_ISL_1247947, EPI_ISL_1308949 to 1308950			
see above	Barts Health NHS Trust	COVID-19 Genomics UK (COG-UK) Consortium	BROAD; Beatrix; CUTINO-MOGUEL; Claire; David; Dola; HARRINGTON; KELE; KULASEGARAN-SHYLINI; Maria-Teresa; OWOYEMI; Raghavendran; SHYLINI
EPI_ISL_541752, EPI_ISL_541754 to 541755, EPI_ISL_541757 to 541766, EPI_ISL_541768 to 541772, EPI_ISL_541775 to 541783	Barts Health NHS Trust	Wellcome Sanger Institute for the COVID-19 Genomics UK (COG-UK) consortium	Beatrix Kele; Cordelia Langford; David Harrington and Alex Alderton; David K. Jackson; Dominic Kwiatkowski; Ewan Harrison; Ian Johnston; John Sillitoe on behalf of the Wellcome Sanger Institute COVID-19 Surveillance Team; Mark Hopkins; Roberto Amato; Sonia Goncalves; Teresa Cutino-Moguel
EPI_ISL_833333 to 833334, EPI_ISL_833340 to 833341	Batangas City Health Office	Research Institute for Tropical Medicine	Catalino Demetria; Daria Manalo; Edelwisa Mercado; Francisco Gerardo Polotan; Hannah Leah Morito; Inez Andrea Medado; John Leonard Chan; Kirstyn Brunker; Ma Angelica Tujan; Othoniel Jan Onza
EPI_ISL_1069187	Batangas Provincial Health Office	Research Institute for Tropical Medicine	Catalino Demetria; Daria Manalo; Edelwisa Mercado; Francisco Gerardo Polotan; Hannah Leah Morito; Inez Andrea Medado; John Leonard Chan; Joseph Hughes; Kirstyn Brunker; Ma Angelica Tujan; Othoniel Jan Onza
EPI_ISL_1143696 to 1143704, EPI_ISL_1143706 to 1143745, EPI_ISL_1143747 to 1143812, EPI_ISL_1143814 to 1143815, EPI_ISL_1143821, EPI_ISL_1143829 to 1143832, EPI_ISL_1143834 to 1143846, EPI_ISL_1143945 to 1143963, EPI_ISL_1143965 to 1143979, EPI_ISL_1143981 to 1143994, EPI_ISL_1143996 to 1143998, EPI_ISL_1144000 to 1144010, EPI_ISL_1144012 to 1144018, EPI_ISL_1144112 to 1144118, EPI_ISL_1148059 to 1148060, EPI_ISL_1148075 to 1148077, EPI_ISL_1148079, EPI_ISL_1148081 to 1148084, EPI_ISL_1148086 to 1148087, EPI_ISL_1148089 to 1148092, EPI_ISL_1148094 to 1148104, EPI_ISL_1148106 to 1148107, EPI_ISL_1148109, EPI_ISL_1148111, EPI_ISL_1148113 to 1148114, EPI_ISL_1148116 to 1148123, EPI_ISL_1148125 to 1148128, EPI_ISL_1148130 to 1148135, EPI_ISL_1148137 to 1148139, EPI_ISL_1148141 to 1148148, EPI_ISL_1148150, EPI_ISL_1148152 to 1148156, EPI_ISL_1148158, EPI_ISL_1148160, EPI_ISL_1148162, EPI_ISL_1151810 to 1151815, EPI_ISL_1151817 to 1151821, EPI_ISL_1151823 to 1151829, EPI_ISL_1151831, EPI_ISL_1151833 to 1151840, EPI_ISL_1151842, EPI_ISL_1151844 to 1151858, EPI_ISL_1151860 to 1151864, EPI_ISL_1151866, EPI_ISL_1151868 to 1151870, EPI_ISL_1151872 to 1151885, EPI_ISL_1151889, EPI_ISL_1157277, EPI_ISL_1157279, EPI_ISL_1157281, EPI_ISL_1157283, EPI_ISL_1157285, EPI_ISL_1157287 to 1157288, EPI_ISL_1157291 to 1157295, EPI_ISL_1157297 to 1157299, EPI_ISL_1157302 to 1157304, EPI_ISL_1157306 to 1157307, EPI_ISL_1157309 to 1157310, EPI_ISL_1157312 to 1157314, EPI_ISL_1157316 to 1157327, EPI_ISL_1157329 to 1157333, EPI_ISL_1157336 to 1157338, EPI_ISL_1157340 to 1157361, EPI_ISL_1280604 to 1280605, EPI_ISL_1280608 to 1280609, EPI_ISL_1280611, EPI_ISL_1280615 to 1280617, EPI_ISL_1280621, EPI_ISL_1280623 to 1280625, EPI_ISL_1280627 to 1280630, EPI_ISL_1280634 to 1280636, EPI_ISL_1280638, EPI_ISL_1280640, EPI_ISL_1280644, EPI_ISL_1280646 to 1280647, EPI_ISL_1280650, EPI_ISL_1280656 to 1280660			
see above	Bayerisches Landesamt für Gesundheit und Lebensmittelsicherheit (LGL)	Robert Koch Institute	
EPI_ISL_444022, EPI_ISL_445078 to 445084, EPI_ISL_501167 to 501174, EPI_ISL_513294	Baylor College of Medicine	Baylor College of Medicine: HGSC	David Henke; Donna Muzny; Erin Nicholson; George Weissenberger; Ginger Metcalf; Harsha Doddapaneni; Hsu Chao; Hua Shen; Joseph F. Petrosino; Kavya Kottapalli; Kristi L. Hoffman; Matthew C. Ross; Matthew Wong; Pedro Piedra; Qingchang Meng; Richard Sucgang; Sara J.J. Cregeen; Sejal Salvi; Tulin Ayvaz; Vasanthi Avadhanula; Vipin Menon; Yimiti Meiheerguli; Zeineen Momin
EPI_ISL_1112947 to 1113041	Baylor College of Medicine/ GCID	Baylor College of Medicine/ GCID	David Henke; Donna Muzny; Erin Nicholson; George Weissenberger; Ginger Metcalf; Harsha Doddapaneni; Hsu Chao; Hua Shen; Joseph F. Petrosino; Kavya Kottapalli; Kristi L. Hoffman; Matthew C. Ross; Matthew Wong; Pedro Piedra; Qingchang Meng; Richard Sucgang; Sara J.J. Cregeen; Sejal Salvi; Tulin Ayvaz; Vasanthi Avadhanula; Vipin Menon; Yimiti Meiheerguli; Zeineen Momin
EPI_ISL_1109635, EPI_ISL_1182692, EPI_ISL_1191094, EPI_ISL_1195294, EPI_ISL_1231436, EPI_ISL_1307717, EPI_ISL_1311848			
see above	Baylor Esoteric + Molecular Lab	Baylor Esoteric + Molecular Lab	Dawn Richards; Elizabeth Forrester; Elizabeth Forrester and Dawn Richards
EPI_ISL_966285	Baylor Scott & White - Irving	Baylor Scott & White - Temple	Ari Rao; Kimberly Walker; Linden Morales; Marcus Volz; Shelby Hendrickson
EPI_ISL_1058066	Baylor Scott & White - Irving	Baylor Scott & White-Temple	Ari Rao; Kimberly Walker; Linden Morales; Marcus Volz; Shelby Hendrickson
EPI_ISL_1058061	Baylor Scott & White - Southlake Family Medicine	Baylor Scott & White-Temple	Ari Rao; Kimberly Walker; Linden Morales; Marcus Volz; Shelby Hendrickson
EPI_ISL_966310, EPI_ISL_966313	Baylor Scott & White - Temple	Baylor Scott & White - Temple	Ari Rao; Kimberly Walker; Linden Morales; Marcus Volz; Shelby Hendrickson
EPI_ISL_1109990, EPI_ISL_1110024, EPI_ISL_1110026, EPI_ISL_1172020, EPI_ISL_1235680, EPI_ISL_1292089	Baylor Scott & White-Irving	Baylor Scott & White-Temple	Ari Rao; Caitlin Maloney; Kimberly Walker; Linden Morales; Marcus Volz; Shelby Hendrickson
EPI_ISL_925208, EPI_ISL_943550, EPI_ISL_1080853, EPI_ISL_1080855, EPI_ISL_1080857, EPI_ISL_1081136, EPI_ISL_1081149 to 1081151, EPI_ISL_1081171 to 1081191, EPI_ISL_1097018, EPI_ISL_1110290 to 1110292, EPI_ISL_1110294, EPI_ISL_1171707, EPI_ISL_1171710 to 1171711, EPI_ISL_1171770, EPI_ISL_1172018 to 1172019, EPI_ISL_1172021, EPI_ISL_1180229, EPI_ISL_1180231, EPI_ISL_1180235, EPI_ISL_1180237 to 1180238, EPI_ISL_1180255 to 1180256, EPI_ISL_1180258, EPI_ISL_1180279, EPI_ISL_1180669 to 1180670, EPI_ISL_1180958, EPI_ISL_1235678 to 1235679, EPI_ISL_1235681 to 1235685			
see above	Baylor Scott & White-Temple	Baylor Scott & White-Temple	Ari Rao; Caitlin Maloney; Kimberly Walker; Linden Morales; Marcus Volz; Shelby Hendrickson; ri Rao
EPI_ISL_700553	Beaufort West CDC wc BWC	NHLS/UCT	Arash Iranzadeh; Bruna Galvao; Carolyn Williamson; Deelan Doolabh; Diana Hardie; Innocent Mudau; Kruger Marais; Lynn Tyers; Marvin Hsiao; Stephen Korsman
EPI_ISL_640031, EPI_ISL_640047, EPI_ISL_700453	Beaufort West Hospital wc BWH	NHLS/UCT	Arash Iranzadeh; Bruna Galvao; Carolyn Williamson; Deelan Doolabh; Diana Hardie; Innocent Mudau; Kruger Marais; Lynn Tyers; Marvin Hsiao; Stephen Korsman
EPI_ISL_960141	Beaufort West Hospital wc BWH	National Health Laboratory Service/UCT	Arash Iranzadeh; Bruna Galvao; Carolyn Williamson; Deelan Doolabh; Diana Hardie; Innocent Mudau; Kruger Marais; Lynn Tyers; Marvin Hsiao; Stephen Korsman
EPI_ISL_1117376 to 1117378, EPI_ISL_1168770 to 1168771	Beijing Center for Disease Prevention and Control	Beijing Center for Disease Prevention and Control	Bing Lyu; Daitao Zhang; Fu Li; Lijuan Chen; Quanyi Wang; Shujuan Cui; Yang Pan; Zhaomin Feng; Zhichao Liang
EPI_ISL_412386	Beijing Ditan Hospital, Capital Medical University	National Institute for Communicable Disease Control and Prevention, Chinese Center for Disease Control and Prevention	Biao Kan; Chuansheng Li; Fangfang Jin; Haijian Zhou; Haofeng Xiong; Hebing Guo; Huizhu Wang; Jianbo Tan; Jie Gong; Jingjing Hao; Jingyuan Liu; Lili Gao; Lin Pu; Ming Zhang; Pan Xiang; Ruihong Li; Xiaoping Chen; Xin Lu; Xinmin Xu; Yajie Wang; Yanwen Xiong; Yao Sun; Yufeng Liu
EPI_ISL_410538 to 410544, EPI_ISL_424355 to 424360, EPI_ISL_529213 to 529217	Beijing Institute of Microbiology and Epidemiology	Beijing Institute of Microbiology and Epidemiology	B. and Cui, Y.; Bao-Gui Jiang; Cui, Y.; Fan; Fan, H.; Guo, Y.; Hang; Hou, J.; Jia-Fu Jiang; Li; Li, B.; Mi, Z.; Mu, J.; Na Jia; Qin, E.; Song; Song, Y.; Teng; Teng, Y.; Tommy Tsan-Yuk Lam; Wu, Y.; Wu-Chun Cao; Xu, Z.; Ya-Wei Zhang; Yajun.; Yang, R.; Yong, Y.; Yue; Zhang, X.
EPI_ISL_509711 to 509714	Belize Ministry of Health	Pathogen Discovery, Respiratory Viruses Branch, Division of Viral Diseases, Centers for Disease Control and Prevention	Anna Uehara; Clinton Paden; Haibin Wang; Jing Zhang; Krista Queen; Suxiang Tong; Yan Li; Ying Tao

EPI_ISL_631580, EPI_ISL_631765, EPI_ISL_631804, EPI_ISL_631838, EPI_ISL_632068 to 632069, EPI_ISL_632098, EPI_ISL_632121, EPI_ISL_632136			
see above	Bellevue Hospital Center	New York City Public Health Laboratory	Jade Wang; et al.
EPI_ISL_845891 to 845892	Benaroya Research Institute	UW Virology Lab	Alexander Greninger; Hong Xie; Keith R Jerome; Lasata Shrestha; Meei-Li Huang; Michelle Lin; Pavitra Roychoudhury
EPI_ISL_569609	Bennet County Hospital	South Dakota Public Health Laboratory	Jacob Garfin; Matt Plumb; Xiong Wang; and Chris Carlson
EPI_ISL_780382 to 780410, EPI_ISL_783850	Bermuda Government Molecular Diagnostics Laboratory (MDL)	Respiratory Virus Unit, National Infection Service, Public Health England	Dr Ayoola Oyinloye (Bermuda); Dr Carika Weldon (Bermuda); PHE Covid Sequencing Team
EPI_ISL_1085362, EPI_ISL_1085413, EPI_ISL_1085934 to 1085935	Besançon Laboratoire CBM25 Terre Rouge	Department of Virology, Henri Mondor University Hospital, Assistance Publique Hôpitaux de Paris, Université Paris-Est Créteil, INSERM U955	Alexandre Soulier; Christophe Rodriguez; Elisabeth Trawinski; Guillaume Gricourt; Jean-Michel Pawlotsky; Melissa N'Debi; Slim Fourati; Vanessa Demontant
EPI_ISL_430843	Bethany Hospital	Research Institute for Tropical Medicine	Bautista; Brunker, K.; C.S.; C.T.; D.L.; Demetria; E.S.; F.G.M.; I.A.P.; Manalo; Medado; Mercado; O.J.T.; Onza; Polotan
EPI_ISL_569618 to 569619	Bethel Lutheran Home	South Dakota Public Health Laboratory	Jacob Garfin; Matt Plumb; Xiong Wang; and Chris Carlson
EPI_ISL_833405 to 833412	Beverly Hospital	Los Angeles County PHL	P. Hemarajata et al.
EPI_ISL_457824	Bezmialem Vakif University, Dept Microbiology, Medical School, Fatih, Istanbul, Turkey	Bezmialem Vakif University, Medical School & Beykoz Institute of Life Sciences & Biotechnology	Bilge Sumbul; Elif Karaaslan; Filiz Guney; Mehmet Z. Doymaz; Merve Kalkan; Nesibe Cetin
EPI_ISL_735495	Bhashabir M A Wadud RT-PCR Lab, Chandpur	Central Biological Research Laboratory and Department of Biochemistry and Molecular Biology	H. M. Abdullah Al Masud; Imam Hossen; Md. Arif Hossain; Md. Imranul Hoq; Md. Khondakar Raziur Rahman; Md. Omer Faruq; Mohammad Omar Faruque; Robiul Hasan Bhuiyan; Sajib Rudra; Shanta Paul
EPI_ISL_1072964	Bhayangkara Hospital Medan	Institute of Tropical Disease, Universitas Airlangga; Faculty of Medicine, Universitas Sumatera Utara	Aldise M Nastri; Franciscus Ginting; Inke N D Lubis; Irbah R Nainggolan; Jezzy R Dewantari; Kazufumi Shimizu; Krisnoadi Rahardjo; Maria I Lusida; Meliani; Mirzan Hasibuan; Muhammad Ichwan; R Andika D Cahyadi; R Lia Kusumawati; Ramadhan Bestari; Rima R Prasetya; Soetjipto; Yasuko Mori
EPI_ISL_1069153	Binjai Health Office	Institute of Tropical Disease, Universitas Airlangga; Fakultas Kedokteran, Universitas Sumatra Utara	Aldise M Nastri; Franciscus Ginting; Inke N D Lubis; Irbah R Nainggolan; Jezzy R Dewantari; Kazufumi Shimizu; Krisnoadi Rahardjo; Maria I Lusida; Meliani; Mirzan Hasibuan; Muhammad Ichwan; R Andika D Cahyadi; R Lia Kusumawati; Ramadhan Bestari; Rima R Prasetya; Soetjipto; Yasuko Mori
EPI_ISL_955231 to 955232, EPI_ISL_955258 to 955260	BioReference Lab	NJ Public Health and Environmental Laboratories	Byeong Jeong; Dana Woell; Lindsey Bodnar; Shiv Verma
EPI_ISL_861865 to 861866, EPI_ISL_896095	Bioanalytika AG	University Hospital Basel, Clinical Bacteriology	Adrian Egli; Adrian Härri; Alfredo Mari; Hans Hirsch; Helena MB Seth-Smith; Julia Bielicki; Karoline Leuzinger; Madlen Stange; Manuel Battegay; Tim Roloff
EPI_ISL_1273084 to 1273096	Biochemistry and Molecular Biology Department-Faculty of Medicine, Al-Quds University	Biochemistry and Molecular Biology Department-Faculty of Medicine, Al-Quds University	Al-Jawabreh, A.; Al-Jawabreh, H.; Dumaidi, K.; Ereqat, S.; Nasereddin, A.
EPI_ISL_900515 to 900520, EPI_ISL_900529	Bioesterel	CNR Virus des Infections Respiratoires - France SUD	Antonin Bal; Bruno Lina; Gregory Destras; Gwendolyne Burfin; Géraldine Gonfrier; Hadrien Règue; Laurence Josset; Martine Valette; Quentin Semanas; Sylvie Larrat; Valérie Giordanengo
EPI_ISL_411959 to 411967	Bioinfo, Vision Medicals, Lianhe	Bioinfo, Vision Medicals, Lianhe	W.H; Zhang
EPI_ISL_411958	Bioinfo, Vision Medicals, Lianhe,	Bioinfo, Vision Medicals, Lianhe,	W.H; Zhang
EPI_ISL_814287 to 814290, EPI_ISL_814292, EPI_ISL_814351, EPI_ISL_814357 to 814361, EPI_ISL_814417 to 814421, EPI_ISL_814424 to 814438, EPI_ISL_814517 to 814522, EPI_ISL_814524 to 814525, EPI_ISL_814530, EPI_ISL_814532, EPI_ISL_814538 to 814539, EPI_ISL_814543 to 814544, EPI_ISL_814548, EPI_ISL_814610 to 814619, EPI_ISL_816657 to 817122, EPI_ISL_842355 to 842445, EPI_ISL_842447 to 842470, EPI_ISL_842472 to 842494, EPI_ISL_842496 to 842511, EPI_ISL_842513 to 842596, EPI_ISL_842598 to 842608, EPI_ISL_868716 to 868769, EPI_ISL_868771 to 868785, EPI_ISL_868787 to 868808, EPI_ISL_868810 to 868817, EPI_ISL_868819 to 868830, EPI_ISL_868832 to 868888, EPI_ISL_868890 to 868948, EPI_ISL_868950 to 869025, EPI_ISL_869027 to 869078, EPI_ISL_924430 to 924441, EPI_ISL_924443 to 924495, EPI_ISL_924497 to 924498, EPI_ISL_924500 to 924535, EPI_ISL_924537 to 924540, EPI_ISL_924542, EPI_ISL_924544 to 924545, EPI_ISL_924547 to 924623, EPI_ISL_924625 to 924642, EPI_ISL_924644 to 924670, EPI_ISL_924672 to 924673, EPI_ISL_924676 to 924750, EPI_ISL_924752 to 924766, EPI_ISL_924768 to 924786, EPI_ISL_924788 to 924798, EPI_ISL_924800 to 924852, EPI_ISL_924854 to 924875, EPI_ISL_924877 to 924897, EPI_ISL_924899 to 924902, EPI_ISL_924904 to 924919, EPI_ISL_924921 to 924931, EPI_ISL_924933, EPI_ISL_924935 to 924950, EPI_ISL_924952 to 924954, EPI_ISL_924956 to 924986, EPI_ISL_924988 to 925030, EPI_ISL_953024 to 953083, EPI_ISL_953085 to 953109, EPI_ISL_953111, EPI_ISL_953113 to 953133, EPI_ISL_953135 to 953140, EPI_ISL_953142 to 953154, EPI_ISL_953156 to 953167, EPI_ISL_953169 to 953244, EPI_ISL_953246 to 953247, EPI_ISL_953249 to 953252, EPI_ISL_953254 to 953265, EPI_ISL_953267, EPI_ISL_953269 to 953274, EPI_ISL_953278, EPI_ISL_953281 to 953282, EPI_ISL_953286 to 953375, EPI_ISL_1000795 to 1000841, EPI_ISL_1000843 to 1000845, EPI_ISL_1000847 to 1000851, EPI_ISL_1000853, EPI_ISL_1000855, EPI_ISL_1000857 to 1000863, EPI_ISL_1000865 to 1000881, EPI_ISL_1000883 to 1000909, EPI_ISL_1000911 to 1000922, EPI_ISL_1000924 to 1000925, EPI_ISL_1000927 to 1000933, EPI_ISL_1000935 to 1000939, EPI_ISL_1000941 to 1000959, EPI_ISL_1000961 to 1000966, EPI_ISL_1054710 to 1054755, EPI_ISL_1054758, EPI_ISL_1054760, EPI_ISL_1054763 to 1054764, EPI_ISL_1054766, EPI_ISL_1054768 to 1054769, EPI_ISL_1054771, EPI_ISL_1054776 to 1054794, EPI_ISL_1054796 to 1054851, EPI_ISL_1054853 to 1054860, EPI_ISL_1180105 to 1180140, EPI_ISL_1250310 to 1250317			
see above	Bioinformatics and Biostatistics Lab, Advanced Sequencing Facility	COVID-19 Genomics UK (COG-UK) Consortium	Aengus Stewart; Chelsea Sawyer; Harshil Patel; Jerome Nicod; Laura Cubitt; Margaret Crawford
EPI_ISL_759866 to 759867	Bioinformatics, National institute of traditional medicine	Bioinformatics, National institute of traditional medicine	Chattopadhyay, D.; D.R.; Harish; Ishwar, S.; Umashankar, V.
EPI_ISL_429992 to 430009, EPI_ISL_430011 to 430015, EPI_ISL_434516, EPI_ISL_450186 to 450189, EPI_ISL_635777 to 635782, EPI_ISL_636267 to 636458, EPI_ISL_730229 to 730290, EPI_ISL_730372 to 730563, EPI_ISL_755118, EPI_ISL_755120 to 755129, EPI_ISL_755131, EPI_ISL_755141 to 755145, EPI_ISL_755210 to 755214, EPI_ISL_755232 to 755268, EPI_ISL_878440, EPI_ISL_878443, EPI_ISL_878445, EPI_ISL_878448, EPI_ISL_878450, EPI_ISL_878452, EPI_ISL_878455, EPI_ISL_878457, EPI_ISL_878460, EPI_ISL_878463, EPI_ISL_878465, EPI_ISL_878468, EPI_ISL_878470, EPI_ISL_878472, EPI_ISL_878474, EPI_ISL_878477, EPI_ISL_878480, EPI_ISL_878483, EPI_ISL_878485, EPI_ISL_878488, EPI_ISL_878491 to 878492, EPI_ISL_878495, EPI_ISL_878498, EPI_ISL_878500, EPI_ISL_878503, EPI_ISL_878506, EPI_ISL_878509, EPI_ISL_878511, EPI_ISL_878514, EPI_ISL_878516, EPI_ISL_878519, EPI_ISL_878522, EPI_ISL_878524, EPI_ISL_878526, EPI_ISL_878571, EPI_ISL_878574, EPI_ISL_878576, EPI_ISL_878579, EPI_ISL_878581			
see above	Biolab Diagnostic Laboratories	Andersen lab at Scripps Research	Ahmad Tibi; Amid Abdelnour with SEARCH Alliance San Diego; Issa Abu-Dayyeh; Lama Hussein; Lina Mohammad; Zein Naber
EPI_ISL_977537, EPI_ISL_977600, EPI_ISL_977602, EPI_ISL_1089783, EPI_ISL_1116758	Biolab Diagnostic Laboratories	Biolab Diagnostic Laboratories	Ahmad Tibi; Amid Abdelnour; Badia Saddedin; Eiad Atwa; Issa Abu-Dayyeh; Lama Hussein; Shayma Ali
EPI_ISL_935026, EPI_ISL_935038 to 935039, EPI_ISL_935041	Biolab Diagnostic Laboratories	Princess Haya Biotechnology Center, Jordan University of Science and Technology	Amid Abdelnour; Areej Alquran; Hazem Haddad; Issa Abu-Dayyeh; Maha Karam; Moh''D Al-Zghoul; Mohammad Alboom; Mustafa Ababneh; Saied Jaradat; Shereen Issa; Suha Hasan
EPI_ISL_935043, EPI_ISL_935046	Biolab Diagnostic Laboratories	Princess Haya Biotechnology Center/ Jordan University of Science & Technology	Amid Abdelnour; Areej Alquran; Hazem Haddad; Issa Abu-Dayyeh; Maha Karam; Moh''D Al-Zghoul; Mohammad Alboom; Mustafa Ababneh; Saied Jaradat; Shereen Issa; Suha Hasan
EPI_ISL_900514	Biolittoral	CNR Virus des Infections Respiratoires - France SUD	Antonin Bal; Bruno Lina; Gregory Destras; Gwendolyne Burfin; Hadrien Règue; Laurence Josset; Martine Valette; Quentin Semanas
EPI_ISL_1040927	Biologia molecular de enfermedades emergentes y EPOC, Instituto Nacional de Enfermedades Respiratorias	Biologia molecular de enfermedades emergentes y EPOC, Instituto Nacional de Enfermedades Respiratorias	Hernández-Teran Alejandra; Mejía-Nepomuceno Fidencio; Perez-Padilla Rogelio; Ramirez-Gonzalez Ernesto.; Rodríguez-Maldonado Abril; Salas-Hernández Jorge; Serna Ricardo; Vazquez-Perez Joel Armando; Wong-Arambula Claudia
EPI_ISL_527007	Biological Prevention, Army	Biological Prevention, Army	A.E.; A.F.; A.M.; Ageez; B.E.; Elhoseiny; Gad; Harty; M.D.; M.F.; M.G.; Seadawy; Shabaan; Shamel
EPI_ISL_526975 to 527006	Biological prevention, army	Biological prevention, army	A.E.; A.F.; A.M.; Ageez; B.E.; Elhoseiny; Elhosieny; Gad; Harty; M.D.; M.F.; M.G.; Seadawy; Shabaan; Shamel
EPI_ISL_510526, EPI_ISL_510532	Biological prevention, army	Biological prevention, army	A.A.; A.F.; A.M. and Soliman; Ali; Amer; B.E.; B.S.; ELnabrawy; ElGohary; Elhoseiny; Elhoseny; Elnagdy; Elnakeeb; Gad; H.A.; Harty; Hassan; Kandeil; Karam; M.A.; M.D.; M.F.; M.G.; M.M. and Gad; Raouf; Seadawy; Shamel; T.A.; W.A.; Y.A.; k.E.
EPI_ISL_1263090 to 1263102, EPI_ISL_1263192 to 1263206	Biology Department, University of Babylon	Biology Department, University of Babylon	A.A.; A.H.; Al-Ghrairi; Hussein; M.A.; Mohammed; Salman
EPI_ISL_582029 to 582030	Biology Department, College of Science, Al-Muthanna University	International Centre for Genetic Engineering and Biotechnology (ICGEB) and ARGO Open Lab Platform	Alessandro Marcello; Danilo Licastro; Nihad Al-Rashedi; Simeone Dal Monego; Sreejith Rajasekharan
EPI_ISL_505003	Biology Dpt	Microbiology and Infections Diseases	Annabelle Garnier; Audrey Ferrier-Rembert; Clarisse Vigne; Emilie Tessier; Emmanuelle Billon-Denis; Flora Nolent; Isabelle Drouet; Jean-Nicolas Tournier; Jessica Denis; Laurence Cheutin; Noémie Verguet; Olivier Ferraris; Olivier Gorgé
EPI_ISL_506041	Biology Dpt, HIA Percy	Microbiology and Infectious Diseases Dpt	Annabelle Garnier; Audrey Ferrier-Rembert; Clarisse Vigne; Emilie Tessier; Emmanuelle Billon-Denis; Flora Nolent; Isabelle Drouet; Jean-Nicolas Tournier; Jessica Denis; Laurence Cheutin; Noémie Verguet; Olivier Ferraris; Olivier Gorgé
EPI_ISL_1015380, EPI_ISL_1081098,	Biology Lab, HIA BEGIN	IRBA, 2MI	CHAPUS C.; DEPEILLE A.; GORGE O.; MERENS-GONTIER A.; NOLENT F.; SARILAR V.

EPI_ISL_1081100 to 1081101, EPI_ISL_1081103 to 1081104, EPI_ISL_1081106 to 1081107			
EPI_ISL_1015382	Biology Lab, HIA CLERMONT-TONNERRE	IRBA, 2MI	DESIDERI C.; GORGE O.; NOLENT F.; SARILAR V.
EPI_ISL_1015369, EPI_ISL_1015376, EPI_ISL_1015378, EPI_ISL_1015383 to 1015386, EPI_ISL_1015390, EPI_ISL_1015408 to 1015413, EPI_ISL_1081108 to 1081109, EPI_ISL_1081114 to 1081130			
see above	Biology Lab, HIA PERCY	IRBA, 2MI	CHAPUS C.; DEPEILLE A.; FOISSAUD V.; GORGE O.; JARJAVAL F.; NOLENT F.; SARILAR V.; VERGUET N.
EPI_ISL_1015379, EPI_ISL_1081131 to 1081134	Biology Lab, HIA STE-ANNE	IRBA, 2MI	GORGE O.; JANVIER F.; NOLENT F.; OTTO M.-P.; SARILAR V.; VERGUET N.
EPI_ISL_907076 to 907085	Biology, MCL	Biology, MCL	A.F.; A.N.; Elhoseiny; Gad; M.D.; M.F.; M.G.; Seadawy; Shamel; Zekri
EPI_ISL_738081 to 738083	Biology, College of Education	Biology, College of Education	Al-Jaf; Mahmood; Niranji; S.M.; S.S.; Z.H.
EPI_ISL_794634	Biology, MCL	Biology, MCL	A.F.; A.S.; B.S. and EL-Safty; EL-harty; EL-hosieny; Gad; M.D.; M.F.; M.G.; Seadawy; Shamel
EPI_ISL_620406	Biomedical Science and Technology, Konkuk University, Laboratory of Cytokine Immunology (Room 601-1) IBST,Konkuk University	Biomedical Science and Technology, Konkuk University, Laboratory of Cytokine Immunology (Room 601-1) IBST,Konkuk University	Kim, S.
EPI_ISL_516079 to 516088	Biomedical Sciences and Public Health, Polytechnic University of Marche	Biomedical Sciences and Public Health, Polytechnic University of Marche	Alessandrini, F.; Bagnarelli, P.; Caucci, S.; Di Sante, L.; Melchionda, F.; Menzo, S.; Onofri, V.; Tagliabracci, A.; Turchi, C.
EPI_ISL_693249	Biomedical Sciences for Health, University of Milan	Biomedical Sciences for Health, University of Milan	Amendola, A.; Bianchi, S.; Borghi, E.; Canuti, M.; Colzani, D.; G.V. and Tanzi, E.; Gori, M.; Raviglione, M.; Zuccotti
EPI_ISL_889328 to 889330	Bioptická laborato s.r.o.	Institute of Applied Biotechnologies a.s.	Kateina Kvapilová; Martin Kašný; Ondej Brzo; Petr Klempt; Petr Kvapil
EPI_ISL_985058 to 985129, EPI_ISL_985237 to 985238	Biorepository and Clinical Virology Laboratory	Ozer Lab	Adeola A. Fowotade; Babafemi O. Taiwo; Egon A. Ozer; Ewean C. Omoruyi; Johnson A. Adeniji; Judd F. Hultquist; Lacy M. Simons; Olubusuyi M. Adewumi; Ramon Lorenzo-Redondo
EPI_ISL_458287	Biosafety Department PCL3	Biosafety Department PCL3	A. and El Kabbaj, S.; Lemriss, S.; Souiri
EPI_ISL_1039212	Biosafety Level 3 (BSL-3) core facility at Huazhong Agricultural University	State Key Laboratory of Agriculture Microbiology, Huazhong Agric Laboratory of Animal Virology, College of Veterinary Medicine	Yufei Zhang Kun HuangZhong Zou Meilin Jin
EPI_ISL_1140552 to 1140568, EPI_ISL_1140570 to 1140596, EPI_ISL_1140598 to 1140636, EPI_ISL_1140638 to 1140645, EPI_ISL_1140647 to 1140655, EPI_ISL_1140657 to 1140666, EPI_ISL_1140668 to 1140682, EPI_ISL_1140684 to 1140723, EPI_ISL_1140725 to 1140750, EPI_ISL_1140752 to 1140753, EPI_ISL_1140755 to 1140758, EPI_ISL_1140760 to 1140787, EPI_ISL_1140789, EPI_ISL_1140791 to 1140794, EPI_ISL_1140796 to 1140837, EPI_ISL_1141211 to 1141222, EPI_ISL_1141224 to 1141238, EPI_ISL_1141240 to 1141242, EPI_ISL_1141244 to 1141250, EPI_ISL_1141252 to 1141262, EPI_ISL_1141264 to 1141276, EPI_ISL_1141278 to 1141283, EPI_ISL_1141285 to 1141292, EPI_ISL_1141294 to 1141307, EPI_ISL_1141309, EPI_ISL_1141311 to 1141312, EPI_ISL_1141314, EPI_ISL_1141316 to 1141317, EPI_ISL_1141319, EPI_ISL_1141321 to 1141325, EPI_ISL_1141327 to 1141334, EPI_ISL_1141336 to 1141339, EPI_ISL_1141341, EPI_ISL_1141344 to 1141353, EPI_ISL_1141355, EPI_ISL_1141357 to 1141360, EPI_ISL_1141362 to 1141367, EPI_ISL_1141369 to 1141372, EPI_ISL_1141374 to 1141378, EPI_ISL_1141380 to 1141382, EPI_ISL_1141384 to 1141388, EPI_ISL_1141390 to 1141391, EPI_ISL_1141394 to 1141397, EPI_ISL_1141399 to 1141403, EPI_ISL_1141405 to 1141425, EPI_ISL_1141427, EPI_ISL_1141429, EPI_ISL_1141431 to 1141439, EPI_ISL_1141442 to 1141446, EPI_ISL_1141448 to 1141453, EPI_ISL_1141455 to 1141457, EPI_ISL_1141462 to 1141463, EPI_ISL_1141465 to 1141476, EPI_ISL_1141479 to 1141481, EPI_ISL_1141484 to 1141485, EPI_ISL_1141487, EPI_ISL_1141490 to 1141491, EPI_ISL_1141493 to 1141494, EPI_ISL_1141496 to 1141503, EPI_ISL_1141505 to 1141506, EPI_ISL_1141509 to 1141522, EPI_ISL_1141526 to 1141549, EPI_ISL_1141551 to 1141556, EPI_ISL_1141558 to 1141572, EPI_ISL_1141574 to 1141594, EPI_ISL_1141596 to 1141611, EPI_ISL_1142278 to 1142279, EPI_ISL_1142282, EPI_ISL_1142295, EPI_ISL_1142297, EPI_ISL_1142304, EPI_ISL_1142306, EPI_ISL_1142324 to 1142325, EPI_ISL_1142335 to 1142337, EPI_ISL_1142339, EPI_ISL_1142344, EPI_ISL_1142348, EPI_ISL_1142354, EPI_ISL_1142357, EPI_ISL_1142360, EPI_ISL_1142364, EPI_ISL_1142366, EPI_ISL_1142370, EPI_ISL_1142379, EPI_ISL_1142386 to 1142388, EPI_ISL_1142398, EPI_ISL_1142405, EPI_ISL_1142407, EPI_ISL_1142411, EPI_ISL_1142436 to 1142438, EPI_ISL_1142440 to 1142441, EPI_ISL_1142445, EPI_ISL_1142448, EPI_ISL_1142451, EPI_ISL_1142454, EPI_ISL_1142459, EPI_ISL_1142461, EPI_ISL_1142469, EPI_ISL_1142471, EPI_ISL_1142474, EPI_ISL_1142476, EPI_ISL_1142485, EPI_ISL_1142489, EPI_ISL_1142491, EPI_ISL_1142494, EPI_ISL_1142496, EPI_ISL_1142500, EPI_ISL_1142505, EPI_ISL_1142507 to 1142509, EPI_ISL_1142722 to 1142723, EPI_ISL_1142725 to 1142727, EPI_ISL_1142737 to 1142738, EPI_ISL_1142744, EPI_ISL_1142747, EPI_ISL_1142759, EPI_ISL_1142762, EPI_ISL_1142766 to 1142767, EPI_ISL_1142770, EPI_ISL_1142773 to 1142775, EPI_ISL_1142777, EPI_ISL_1142783, EPI_ISL_1142791, EPI_ISL_1142794, EPI_ISL_1142797, EPI_ISL_1142816, EPI_ISL_1142824, EPI_ISL_1142831, EPI_ISL_1142835, EPI_ISL_1142843, EPI_ISL_1142857, EPI_ISL_1142863, EPI_ISL_1142866, EPI_ISL_1142872 to 1142873, EPI_ISL_1142879, EPI_ISL_1142881 to 1142882, EPI_ISL_1142894 to 1142895, EPI_ISL_1142901 to 1142902, EPI_ISL_1142904 to 1142905, EPI_ISL_1142914, EPI_ISL_1142923, EPI_ISL_1142929, EPI_ISL_1142934, EPI_ISL_1142936, EPI_ISL_1142938, EPI_ISL_1142943, EPI_ISL_1142949, EPI_ISL_1142961, EPI_ISL_1142972, EPI_ISL_1142982, EPI_ISL_1142984, EPI_ISL_1142999, EPI_ISL_1143005, EPI_ISL_1143010, EPI_ISL_1143015 to 1143017, EPI_ISL_1143019, EPI_ISL_1143037, EPI_ISL_1143039, EPI_ISL_1143042, EPI_ISL_1143046, EPI_ISL_1143048, EPI_ISL_1143050, EPI_ISL_1143054, EPI_ISL_1143057, EPI_ISL_1143059, EPI_ISL_1143061, EPI_ISL_1143063 to 1143065, EPI_ISL_1143067 to 1143071, EPI_ISL_1143073 to 1143076, EPI_ISL_1143078, EPI_ISL_1143080, EPI_ISL_1143082, EPI_ISL_1143086, EPI_ISL_1143095 to 1143096, EPI_ISL_1143098, EPI_ISL_1143103 to 1143104, EPI_ISL_1143108 to 1143110, EPI_ISL_1143112, EPI_ISL_1143114 to 1143115, EPI_ISL_1143120, EPI_ISL_1143128, EPI_ISL_1143130, EPI_ISL_1143134, EPI_ISL_1143137, EPI_ISL_1143139 to 1143143, EPI_ISL_1143147 to 1143151, EPI_ISL_1143156, EPI_ISL_1143158, EPI_ISL_1143160, EPI_ISL_1143170 to 1143171, EPI_ISL_1143174 to 1143175, EPI_ISL_1143180 to 1143182, EPI_ISL_1143190, EPI_ISL_1143192, EPI_ISL_1143196 to 1143197, EPI_ISL_1143199, EPI_ISL_1143201, EPI_ISL_1143203, EPI_ISL_1143205, EPI_ISL_1143207, EPI_ISL_1143212 to 1143213, EPI_ISL_1143216 to 1143217, EPI_ISL_1143220, EPI_ISL_1143222, EPI_ISL_1143225, EPI_ISL_1143229, EPI_ISL_1143231, EPI_ISL_1143233, EPI_ISL_1143236, EPI_ISL_1143239, EPI_ISL_1143246, EPI_ISL_1143249, EPI_ISL_1143252 to 1143253, EPI_ISL_1143256, EPI_ISL_1143259, EPI_ISL_1143261, EPI_ISL_1143263, EPI_ISL_1143265, EPI_ISL_1143271, EPI_ISL_1143275, EPI_ISL_1143278 to 1143279, EPI_ISL_1143291 to 1143293, EPI_ISL_1143295, EPI_ISL_1143303, EPI_ISL_1144992 to 1144993, EPI_ISL_1144995, EPI_ISL_1144997, EPI_ISL_1144999, EPI_ISL_1145004, EPI_ISL_1145006 to 1145007, EPI_ISL_1145009, EPI_ISL_1145012 to 1145014, EPI_ISL_1145016, EPI_ISL_1145019 to 1145020, EPI_ISL_1145027, EPI_ISL_1145029, EPI_ISL_1145032, EPI_ISL_1145034 to 1145035, EPI_ISL_1145038 to 1145040, EPI_ISL_1145042, EPI_ISL_1145044 to 1145047, EPI_ISL_1145049, EPI_ISL_1145051 to 1145054, EPI_ISL_1145057 to 1145058, EPI_ISL_1145060, EPI_ISL_1145062, EPI_ISL_1145065 to 1145066, EPI_ISL_1145069 to 1145071, EPI_ISL_1145073, EPI_ISL_1145075 to 1145080, EPI_ISL_1145082 to 1145085, EPI_ISL_1145087, EPI_ISL_1145089 to 1145090, EPI_ISL_1145092, EPI_ISL_1145094 to 1145105, EPI_ISL_1145108 to 1145110, EPI_ISL_1145112, EPI_ISL_1145116, EPI_ISL_1145121 to 1145122, EPI_ISL_1145124, EPI_ISL_1145126 to 1145127, EPI_ISL_1145130 to 1145136, EPI_ISL_1145138, EPI_ISL_1145140, EPI_ISL_1145142 to 1145147, EPI_ISL_1145149, EPI_ISL_1145156 to 1145157, EPI_ISL_1145159 to 1145161, EPI_ISL_1145163, EPI_ISL_1145165, EPI_ISL_1145167 to 1145169, EPI_ISL_1145171 to 1145173, EPI_ISL_1145175, EPI_ISL_1145179, EPI_ISL_1145181, EPI_ISL_1145185 to 1145186, EPI_ISL_1145191, EPI_ISL_1145194 to 1145196, EPI_ISL_1145198, EPI_ISL_1145200 to 1145202, EPI_ISL_1145945, EPI_ISL_1146023, EPI_ISL_1146065, EPI_ISL_1146077, EPI_ISL_1146141, EPI_ISL_1146143, EPI_ISL_1148167, EPI_ISL_1148171, EPI_ISL_1148175, EPI_ISL_1148178 to 1148179, EPI_ISL_1148181, EPI_ISL_1148183, EPI_ISL_1148185 to 1148186, EPI_ISL_1148188, EPI_ISL_1148199 to 1148200, EPI_ISL_1148204, EPI_ISL_1148206 to 1148208, EPI_ISL_1148212, EPI_ISL_1148219, EPI_ISL_1148227, EPI_ISL_1148231, EPI_ISL_1148248 to 1148249, EPI_ISL_1148257 to 1148260, EPI_ISL_1148262, EPI_ISL_1148268 to 1148269, EPI_ISL_1148271, EPI_ISL_1148279, EPI_ISL_1148281, EPI_ISL_1148286, EPI_ISL_1148290, EPI_ISL_1148294, EPI_ISL_1148300, EPI_ISL_1148302, EPI_ISL_1148307, EPI_ISL_1148309, EPI_ISL_1148313, EPI_ISL_1148315, EPI_ISL_1148317 to 1148318, EPI_ISL_1148321, EPI_ISL_1148326, EPI_ISL_1148328, EPI_ISL_1148332 to 1148333, EPI_ISL_1148336, EPI_ISL_1148339, EPI_ISL_1148341, EPI_ISL_1148349, EPI_ISL_1148353, EPI_ISL_1148357 to 1148358, EPI_ISL_1148361, EPI_ISL_1148368, EPI_ISL_1148375, EPI_ISL_1148378, EPI_ISL_1148380, EPI_ISL_1148382, EPI_ISL_1148386 to 1148390, EPI_ISL_1148392, EPI_ISL_1148400, EPI_ISL_1148409, EPI_ISL_1148412 to 1148414, EPI_ISL_1148416 to 1148417, EPI_ISL_1148425, EPI_ISL_1148427, EPI_ISL_1148432, EPI_ISL_1148435, EPI_ISL_1148437, EPI_ISL_1148439, EPI_ISL_1148441 to 1148442, EPI_ISL_1148445, EPI_ISL_1148447, EPI_ISL_1150021, EPI_ISL_1150023, EPI_ISL_1150027 to 1150028, EPI_ISL_1150030 to 1150031, EPI_ISL_1150033 to 1150038, EPI_ISL_1150040 to 1150044, EPI_ISL_1150048, EPI_ISL_1150050 to 1150051, EPI_ISL_1150053, EPI_ISL_1150055, EPI_ISL_1150057, EPI_ISL_1150059 to 1150060, EPI_ISL_1150062, EPI_ISL_1150064, EPI_ISL_1150071 to 1150072, EPI_ISL_1150074, EPI_ISL_1150076 to 1150078, EPI_ISL_1150085 to 1150090, EPI_ISL_1150093 to 1150094, EPI_ISL_1150096 to 1150097, EPI_ISL_1150100 to 1150101, EPI_ISL_1150103 to 1150106, EPI_ISL_1150108, EPI_ISL_1150110 to 1150111, EPI_ISL_1150113, EPI_ISL_1150115, EPI_ISL_1150118 to 1150124, EPI_ISL_1150127, EPI_ISL_1150129 to 1150130, EPI_ISL_1150132 to 1150135, EPI_ISL_1150138, EPI_ISL_1150142, EPI_ISL_1150144 to 1150145, EPI_ISL_1150147, EPI_ISL_1150149, EPI_ISL_1150154 to 1150155, EPI_ISL_1150157, EPI_ISL_1150160 to 1150161, EPI_ISL_1150164 to 1150166, EPI_ISL_1150170, EPI_ISL_1150172, EPI_ISL_1150174, EPI_ISL_1150177, EPI_ISL_1150180 to 1150181, EPI_ISL_1150183, EPI_ISL_1150185 to 1150186, EPI_ISL_1150188 to 1150190, EPI_ISL_1150193, EPI_ISL_1150198, EPI_ISL_1150201 to 1150202, EPI_ISL_1150205, EPI_ISL_1150209, EPI_ISL_1150211 to 1150212, EPI_ISL_1150214 to 1150215, EPI_ISL_1150217, EPI_ISL_1150219, EPI_ISL_1150226, EPI_ISL_1150228, EPI_ISL_1150231 to 1150232, EPI_ISL_1150235, EPI_ISL_1150237, EPI_ISL_1150241 to 1150242, EPI_ISL_1150244, EPI_ISL_1150249, EPI_ISL_1150251, EPI_ISL_1150253 to 1150254, EPI_ISL_1150256, EPI_ISL_1150261 to 1150263, EPI_ISL_1150265, EPI_ISL_1150268 to 1150270, EPI_ISL_1150272, EPI_ISL_1150274, EPI_ISL_1150276 to 1150278, EPI_ISL_1150285 to 1150287, EPI_ISL_1150289 to 1150290, EPI_ISL_1150292 to 1150293, EPI_ISL_1150297 to 1150298, EPI_ISL_1150300 to 1150301, EPI_ISL_1150303 to 1150307, EPI_ISL_1150310, EPI_ISL_1150312, EPI_ISL_1150317 to 1150318, EPI_ISL_1150320, EPI_ISL_1150322, EPI_ISL_1150324 to 1150326, EPI_ISL_1150328, EPI_ISL_1150331 to 1150335, EPI_ISL_1150338 to 1150341, EPI_ISL_1150344, EPI_ISL_1150348, EPI_ISL_1150351, EPI_ISL_1150356 to 1150357, EPI_ISL_1150359 to 1150362, EPI_ISL_1150366, EPI_ISL_1150369, EPI_ISL_1150372 to 1150375, EPI_ISL_1150379 to 1150383, EPI_ISL_1150385 to 1150386, EPI_ISL_1153266, EPI_ISL_1154448, EPI_ISL_1154461, EPI_ISL_1154467, EPI_ISL_1154486 to 1154487, EPI_ISL_1154496, EPI_ISL_1154498, EPI_ISL_1154502, EPI_ISL_1154518, EPI_ISL_1154522, EPI_ISL_1154524, EPI_ISL_1154533, EPI_ISL_1154547, EPI_ISL_1154568, EPI_ISL_1154572, EPI_ISL_1154574, EPI_ISL_1154592, EPI_ISL_1154597, EPI_ISL_1154602, EPI_ISL_1154607, EPI_ISL_1154616, EPI_ISL_1154625, EPI_ISL_1154632, EPI_ISL_1154653, EPI_ISL_1154657, EPI_ISL_1154659, EPI_ISL_1154663 to 1154664, EPI_ISL_1154668, EPI_ISL_1154675, EPI_ISL_1154699, EPI_ISL_1154708, EPI_ISL_1154716, EPI_ISL_1154719, EPI_ISL_1154730, EPI_ISL_1154753, EPI_ISL_1154759, EPI_ISL_1154771, EPI_ISL_1154774 to 1154775, EPI_ISL_1154777, EPI_ISL_1154779, EPI_ISL_1154782 to 1154786, EPI_ISL_1154788, EPI_ISL_1154790, EPI_ISL_1154795 to 1154796, EPI_ISL_1154798, EPI_ISL_1154802 to 1154803, EPI_ISL_1154805, EPI_ISL_1154810, EPI_ISL_1154812, EPI_ISL_1154815, EPI_ISL_1154817 to 1154818, EPI_ISL_1154827, EPI_ISL_1154831, EPI_ISL_1154836, EPI_ISL_1154838, EPI_ISL_1154840, EPI_ISL_1154845, EPI_ISL_1154858 to 1154864, EPI_ISL_1154867, EPI_ISL_1154873 to 1154877, EPI_ISL_1154881, EPI_ISL_1154887 to 1154888, EPI_ISL_1154891, EPI_ISL_1154893, EPI_ISL_1154899 to 1154901, EPI_ISL_1154903 to 1154904, EPI_ISL_1154909, EPI_ISL_1154914, EPI_ISL_1154918, EPI_ISL_1154921, EPI_ISL_1154926, EPI_ISL_1154929 to 1154930, EPI_ISL_1154936, EPI_ISL_1154939, EPI_ISL_1154945, EPI_ISL_1154950, EPI_ISL_1154953, EPI_ISL_1154957, EPI_ISL_1154959, EPI_ISL_1154962, EPI_ISL_1154965 to 1154966, EPI_ISL_1154969, EPI_ISL_1154972 to 1154974, EPI_ISL_1154976, EPI_ISL_1154978 to 1154979, EPI_ISL_1154982 to 1154983, EPI_ISL_1154985 to 1154986, EPI_ISL_1154989, EPI_ISL_1154992 to 1154993, EPI_ISL_1154997, EPI_ISL_1155001, EPI_ISL_1155005, EPI_ISL_1155011 to 1155012, EPI_ISL_1155014 to 1155015, EPI_ISL_1155035 to 1155036, EPI_ISL_1155038, EPI_ISL_1155042, EPI_ISL_1155045 to 1155046, EPI_ISL_1155048, EPI_ISL_1155052, EPI_ISL_1155054, EPI_ISL_1155058 to 1155059, EPI_ISL_1155065, EPI_ISL_1155067, EPI_ISL_1155072, EPI_ISL_1155078, EPI_ISL_1155081 to 1155082, EPI_ISL_1155085 to 1155086, EPI_ISL_1155088 to 1155089, EPI_ISL_1155094, EPI_ISL_1155103, EPI_ISL_1155106, EPI_ISL_1155109, EPI_ISL_1155111, EPI_ISL_1155117, EPI_ISL_1155119, EPI_ISL_1155121, EPI_ISL_1155125, EPI_ISL_1155128, EPI_ISL_1155130 to 1155133, EPI_ISL_1155135 to 1155136, EPI_ISL_1156460 to 1156461, EPI_ISL_1156478, EPI_ISL_1156486, EPI_ISL_1156495, EPI_ISL_1156518, EPI_ISL_1156527, EPI_ISL_1156544, EPI_ISL_1156554 to 1156555, EPI_ISL_1156563, EPI_ISL_1156574, EPI_ISL_1156577, EPI_ISL_1156586, EPI_ISL_1156590, EPI_ISL_1156594 to 1156595, EPI_ISL_1156602, EPI_ISL_1156607, EPI_ISL_1156618, EPI_ISL_1156622, EPI_ISL_1156626, EPI_ISL_1156639, EPI_ISL_1156649, EPI_ISL_1156653, EPI_ISL_1156657, EPI_ISL_1156662, EPI_ISL_1156673, EPI_ISL_1156676, EPI_ISL_1156678 to 1156679, EPI_ISL_1156691 to 1156692, EPI_ISL_1156695 to 1156696, EPI_ISL_1156699, EPI_ISL_1156707, EPI_ISL_1156709, EPI_ISL_1156711, EPI_ISL_1156722, EPI_ISL_1156728, EPI_ISL_1156730, EPI_ISL_1156732, EPI_ISL_1156737, EPI_ISL_1156744, EPI_ISL_1156747, EPI_ISL_1156774, EPI_ISL_1156776, EPI_ISL_1211070, EPI_ISL_1211072 to 1211073, EPI_ISL_1211077 to 1211079, EPI_ISL_1211081, EPI_ISL_1211083, EPI_ISL_1211087, EPI_ISL_1211089 to 1211091, EPI_ISL_1211094, EPI_ISL_1211096 to 1211097, EPI_ISL_1211100, EPI_ISL_1211103, EPI_ISL_1211107, EPI_ISL_1211110, EPI_ISL_1211112, EPI_ISL_1211115, EPI_ISL_1211117 to 1211118, EPI_ISL_1211122 to 1211123, EPI_ISL_1211127 to 1211128, EPI_ISL_1211131, EPI_ISL_1211135, EPI_ISL_1211144 to 1211146, EPI_ISL_1211148, EPI_ISL_1211158, EPI_ISL_1211162 to 1211163, EPI_ISL_1211170 to 1211171, EPI_ISL_1211176 to 1211177, EPI_ISL_1211180, EPI_ISL_1211184, EPI_ISL_1211186, EPI_ISL_1211188, EPI_ISL_1211190 to 1211191, EPI_ISL_1211194, EPI_ISL_1211196, EPI_ISL_1211199 to 1211203, EPI_ISL_1211207, EPI_ISL_1211211, EPI_ISL_1211213 to 1211214, EPI_ISL_1211219 to 1211221, EPI_ISL_1211224 to 1211226, EPI_ISL_1211228, EPI_ISL_1211230, EPI_ISL_1211234 to 1211235, EPI_ISL_1211237, EPI_ISL_1211245 to 1211246, EPI_ISL_1211248, EPI_ISL_1211251, EPI_ISL_1211255, EPI_ISL_1211264, EPI_ISL_1211266, EPI_ISL_1211268, EPI_ISL_1211270 to 1211271, EPI_ISL_1211273, EPI_ISL_1211275, EPI_ISL_1211277 to 1211283, EPI_ISL_1211286, EPI_ISL_1211289 to 1211290, EPI_ISL_1211292 to 1211296, EPI_ISL_1211300, EPI_ISL_1211305, EPI_ISL_1211307, EPI_ISL_1211311 to 1211313, EPI_ISL_1211317, EPI_ISL_1211319 to 1211320, EPI_ISL_1211323 to 1211324, EPI_ISL_1211327 to 1211328, EPI_ISL_1211330, EPI_ISL_1211332, EPI_ISL_1211337, EPI_ISL_1211339 to 1211340, EPI_ISL_1211342 to 1211343, EPI_ISL_1211347 to 1211349, EPI_ISL_1211353, EPI_ISL_1211355 to 1211356, EPI_ISL_1211358, EPI_ISL_1211361 to 1211362, EPI_ISL_1211364 to 1211365, EPI_ISL_1211370 to 1211371, EPI_ISL_1211373, EPI_ISL_1211376 to 1211377, EPI_ISL_1211381, EPI_ISL_1211383 to 1211388, EPI_ISL_1211390, EPI_ISL_1211399 to 1211401, EPI_ISL_1211406, EPI_ISL_1211410 to 1211414, EPI_ISL_1211416 to 1211419, EPI_ISL_1211421 to 1211423, EPI_ISL_1211425, EPI_ISL_1212502, EPI_ISL_1212505, EPI_ISL_1212513, EPI_ISL_1212519, EPI_ISL_1212522, EPI_ISL_1212526 to 1212527, EPI_ISL_1212530 to 1212531, EPI_ISL_1212533, EPI_ISL_1212535 to 1212536, EPI_ISL_1212541, EPI_ISL_1212543 to 1212544, EPI_ISL_1212546, EPI_ISL_1212552 to 1212554, EPI_ISL_1212561, EPI_ISL_1212564, EPI_ISL_1212568 to 1212569, EPI_ISL_1212571, EPI_ISL_1212578 to 1212579, EPI_ISL_1212586, EPI_ISL_1212591, EPI_ISL_1212595, EPI_ISL_1212599, EPI_ISL_1212602 to 1212603, EPI_ISL_1212608 to 1212610, EPI_ISL_1212625, EPI_ISL_1212627, EPI_ISL_1212631, EPI_ISL_1212633, EPI_ISL_1212637, EPI_ISL_1212658, EPI_ISL_1212660, EPI_ISL_1212663, EPI_ISL_1212665, EPI_ISL_1212682, EPI_ISL_1212687, EPI_ISL_1212692, EPI_ISL_1212695, EPI_ISL_1212699, EPI_ISL_1212704, EPI_ISL_1212707, EPI_ISL_1212709, EPI_ISL_1212713, EPI_ISL_1212716 to 1212720, EPI_ISL_1212723 to 1212725, EPI_ISL_1212727 to 1212728, EPI_ISL_1212730, EPI_ISL_1212732, EPI_ISL_1212736 to 1212740, EPI_ISL_1212742 to 1212743, EPI_ISL_1212745 to 1212748, EPI_ISL_1212752, EPI_ISL_1212756, EPI_ISL_1212762, EPI_ISL_1212764 to 1212765, EPI_ISL_1212771 to 1212772, EPI_ISL_1212780 to 1212782, EPI_ISL_1212789, EPI_ISL_1212791 to 1212792, EPI_ISL_1212794 to 1212796, EPI_ISL_1212799, EPI_ISL_1212802 to 1212803, EPI_ISL_1212806 to 1212809, EPI_ISL_1212811, EPI_ISL_1212815, EPI_ISL_1212824, EPI_ISL_1212829, EPI_ISL_1212834, EPI_ISL_1212839, EPI_ISL_1212841, EPI_ISL_1212845, EPI_ISL_1212847, EPI_ISL_1212849,			

EPI_ISL_1212860, EPI_ISL_1212864 to 1212865, EPI_ISL_1216043 to 1216044, EPI_ISL_1216059, EPI_ISL_1216064, EPI_ISL_1216069, EPI_ISL_1216080, EPI_ISL_1216096, EPI_ISL_1216098, EPI_ISL_1216111, EPI_ISL_1216125, EPI_ISL_1216136, EPI_ISL_1216139, EPI_ISL_1216141, EPI_ISL_1216147, EPI_ISL_1216152 to 1216153, EPI_ISL_1216160, EPI_ISL_1216164, EPI_ISL_1216170, EPI_ISL_1216175, EPI_ISL_1216180, EPI_ISL_1216186, EPI_ISL_1216200, EPI_ISL_1216208, EPI_ISL_1216217, EPI_ISL_1216219, EPI_ISL_1216237, EPI_ISL_1216242, EPI_ISL_1216244, EPI_ISL_1216248, EPI_ISL_1216253, EPI_ISL_1216293, EPI_ISL_1216298, EPI_ISL_1216302, EPI_ISL_1216327, EPI_ISL_1216335, EPI_ISL_1216337, EPI_ISL_1216346, EPI_ISL_1216357, EPI_ISL_1216360, EPI_ISL_1216364, EPI_ISL_1216377 to 1216378, EPI_ISL_1216380, EPI_ISL_1216386 to 1216387, EPI_ISL_1216389, EPI_ISL_1216391 to 1216392, EPI_ISL_1216395 to 1216397, EPI_ISL_1216400 to 1216401, EPI_ISL_1216410 to 1216412, EPI_ISL_1216414, EPI_ISL_1216417, EPI_ISL_1216420, EPI_ISL_1216433, EPI_ISL_1216435 to 1216436, EPI_ISL_1216441, EPI_ISL_1216443 to 1216445, EPI_ISL_1216450 to 1216451, EPI_ISL_1216453 to 1216454, EPI_ISL_1216456 to 1216460, EPI_ISL_1216462, EPI_ISL_1216465 to 1216467, EPI_ISL_1216470 to 1216472, EPI_ISL_1216474, EPI_ISL_1216479, EPI_ISL_1216486, EPI_ISL_1216488, EPI_ISL_1216490, EPI_ISL_1216492, EPI_ISL_1216494, EPI_ISL_1216496 to 1216497, EPI_ISL_1216503 to 1216506, EPI_ISL_1216513 to 1216515, EPI_ISL_1216517, EPI_ISL_1216519, EPI_ISL_1216522, EPI_ISL_1216525, EPI_ISL_1216527 to 1216529, EPI_ISL_1216532, EPI_ISL_1216536, EPI_ISL_1216541 to 1216543, EPI_ISL_1216545 to 1216546, EPI_ISL_1216551, EPI_ISL_1216553 to 1216554, EPI_ISL_1216558 to 1216559, EPI_ISL_1216562 to 1216565, EPI_ISL_1216567, EPI_ISL_1216569, EPI_ISL_1216571, EPI_ISL_1216573, EPI_ISL_1216575, EPI_ISL_1216578, EPI_ISL_1216583, EPI_ISL_1216587, EPI_ISL_1216589, EPI_ISL_1216591, EPI_ISL_1216595, EPI_ISL_1216603 to 1216604, EPI_ISL_1216606, EPI_ISL_1216612 to 1216615, EPI_ISL_1216618, EPI_ISL_1216620 to 1216622, EPI_ISL_1216624, EPI_ISL_1216626 to 1216629, EPI_ISL_1216633 to 1216636, EPI_ISL_1216640, EPI_ISL_1216642 to 1216646, EPI_ISL_1216648, EPI_ISL_1216650, EPI_ISL_1216653, EPI_ISL_1216665, EPI_ISL_1216670, EPI_ISL_1216672, EPI_ISL_1216674, EPI_ISL_1216676, EPI_ISL_1216678, EPI_ISL_1216682, EPI_ISL_1216685 to 1216687, EPI_ISL_1216692 to 1216693, EPI_ISL_1216699 to 1216700, EPI_ISL_1216702, EPI_ISL_1216707, EPI_ISL_1216712 to 1216714, EPI_ISL_1216719, EPI_ISL_1216721, EPI_ISL_1216727 to 1216729, EPI_ISL_1216733, EPI_ISL_1216736 to 1216737, EPI_ISL_1216739, EPI_ISL_1216741, EPI_ISL_1216743 to 1216745, EPI_ISL_1216747, EPI_ISL_1216752 to 1216753, EPI_ISL_1216757 to 1216760, EPI_ISL_1216763, EPI_ISL_1216771, EPI_ISL_1216773 to 1216776, EPI_ISL_1216778, EPI_ISL_1216780, EPI_ISL_1281996, EPI_ISL_1282001, EPI_ISL_1282004 to 1282005, EPI_ISL_1282007 to 1282008, EPI_ISL_1282018 to 1282019, EPI_ISL_1282021, EPI_ISL_1282023, EPI_ISL_1282030, EPI_ISL_1282036 to 1282037, EPI_ISL_1282044 to 1282046, EPI_ISL_1282050 to 1282051, EPI_ISL_1282060, EPI_ISL_1282062, EPI_ISL_1282071 to 1282072, EPI_ISL_1282077, EPI_ISL_1282081, EPI_ISL_1282091 to 1282092, EPI_ISL_1282098, EPI_ISL_1282101, EPI_ISL_1282103, EPI_ISL_1282105, EPI_ISL_1282110, EPI_ISL_1282113 to 1282115, EPI_ISL_1282118, EPI_ISL_1282123, EPI_ISL_1282125, EPI_ISL_1282133 to 1282134, EPI_ISL_1282137, EPI_ISL_1282162, EPI_ISL_1282166 to 1282167, EPI_ISL_1282170, EPI_ISL_1282180, EPI_ISL_1282182, EPI_ISL_1282191, EPI_ISL_1282193, EPI_ISL_1282197, EPI_ISL_1282200 to 1282201, EPI_ISL_1282203, EPI_ISL_1282220 to 1282223, EPI_ISL_1282225 to 1282226, EPI_ISL_1282232, EPI_ISL_1282237, EPI_ISL_1282239 to 1282240, EPI_ISL_1282247, EPI_ISL_1282249 to 1282251, EPI_ISL_1282254, EPI_ISL_1282259 to 1282260, EPI_ISL_1282266 to 1282268, EPI_ISL_1282274, EPI_ISL_1282278, EPI_ISL_1282292, EPI_ISL_1282295, EPI_ISL_1282300, EPI_ISL_1282309, EPI_ISL_1284554 to 1284556, EPI_ISL_1284559 to 1284560, EPI_ISL_1284565 to 1284566, EPI_ISL_1284572 to 1284573, EPI_ISL_1284576 to 1284577, EPI_ISL_1284579, EPI_ISL_1284583, EPI_ISL_1284585, EPI_ISL_1284587, EPI_ISL_1284589, EPI_ISL_1284592 to 1284593, EPI_ISL_1284598, EPI_ISL_1284600 to 1284602, EPI_ISL_1284607 to 1284612, EPI_ISL_1284629, EPI_ISL_1284636, EPI_ISL_1284638, EPI_ISL_1284642, EPI_ISL_1284646 to 1284647, EPI_ISL_1284650 to 1284652, EPI_ISL_1284656 to 1284658, EPI_ISL_1284662, EPI_ISL_1284665, EPI_ISL_1284667 to 1284668, EPI_ISL_1284671, EPI_ISL_1284675, EPI_ISL_1284679, EPI_ISL_1284684, EPI_ISL_1284689, EPI_ISL_1284692 to 1284693, EPI_ISL_1284695, EPI_ISL_1284702, EPI_ISL_1284710, EPI_ISL_1284713 to 1284714, EPI_ISL_1284717, EPI_ISL_1284722 to 1284723, EPI_ISL_1284725 to 1284726, EPI_ISL_1284728 to 1284730, EPI_ISL_1284734, EPI_ISL_1284738, EPI_ISL_1284743, EPI_ISL_1284749 to 1284750, EPI_ISL_1284752, EPI_ISL_1284754 to 1284755, EPI_ISL_1284757 to 1284761, EPI_ISL_1284765, EPI_ISL_1284767, EPI_ISL_1284770, EPI_ISL_1284772 to 1284776, EPI_ISL_1284778, EPI_ISL_1284780 to 1284785, EPI_ISL_1284787, EPI_ISL_1284792 to 1284793, EPI_ISL_1284796, EPI_ISL_1284802 to 1284803, EPI_ISL_1284805, EPI_ISL_1284810, EPI_ISL_1284817 to 1284818, EPI_ISL_1284820, EPI_ISL_1284825 to 1284826, EPI_ISL_1284829 to 1284832, EPI_ISL_1284834, EPI_ISL_1284838 to 1284839, EPI_ISL_1284841, EPI_ISL_1284845 to 1284846, EPI_ISL_1284854, EPI_ISL_1284856 to 1284857, EPI_ISL_1284861, EPI_ISL_1284863 to 1284864, EPI_ISL_1284870, EPI_ISL_1284878 to 1284879, EPI_ISL_1285446, EPI_ISL_1285485, EPI_ISL_1285534, EPI_ISL_1285552, EPI_ISL_1285556 to 1285557, EPI_ISL_1285565, EPI_ISL_1285590, EPI_ISL_1285612, EPI_ISL_1285623, EPI_ISL_1285632, EPI_ISL_1285674			
see above	Bioscientia Labor Wermsdorf	Robert Koch Institute	
EPI_ISL_1142292, EPI_ISL_1142299, EPI_ISL_1142307 to 1142308, EPI_ISL_1142319, EPI_ISL_1142329, EPI_ISL_1142355, EPI_ISL_1142369, EPI_ISL_1142399, EPI_ISL_1142418, EPI_ISL_1142428, EPI_ISL_1142452, EPI_ISL_1142512, EPI_ISL_1142514, EPI_ISL_1142521, EPI_ISL_1142523, EPI_ISL_1143045, EPI_ISL_1143055, EPI_ISL_1143060, EPI_ISL_1143091, EPI_ISL_1143124 to 1143125, EPI_ISL_1143152, EPI_ISL_1143179, EPI_ISL_1143185 to 1143186, EPI_ISL_1143194, EPI_ISL_1143230, EPI_ISL_1145183, EPI_ISL_1145192, EPI_ISL_1145992, EPI_ISL_1146124, EPI_ISL_1148176, EPI_ISL_1148250, EPI_ISL_1148347, EPI_ISL_1148364, EPI_ISL_1148430, EPI_ISL_1150208, EPI_ISL_1150216, EPI_ISL_1154620, EPI_ISL_1154648, EPI_ISL_1154688, EPI_ISL_1154714, EPI_ISL_1154781, EPI_ISL_1154906, EPI_ISL_1155030 to 1155031, EPI_ISL_1155060, EPI_ISL_1155079, EPI_ISL_1155107, EPI_ISL_1155129, EPI_ISL_1156536, EPI_ISL_1156717, EPI_ISL_1211075, EPI_ISL_1211105, EPI_ISL_1211109, EPI_ISL_1211142, EPI_ISL_1211151, EPI_ISL_1211155 to 1211156, EPI_ISL_1211159, EPI_ISL_1211166, EPI_ISL_1211209, EPI_ISL_1211229, EPI_ISL_1211239 to 1211240, EPI_ISL_1211249, EPI_ISL_1211254, EPI_ISL_1211276, EPI_ISL_1211299, EPI_ISL_1211303, EPI_ISL_1211331, EPI_ISL_1211336, EPI_ISL_1211341, EPI_ISL_1211346, EPI_ISL_1211350, EPI_ISL_1211374, EPI_ISL_1211403, EPI_ISL_1211407, EPI_ISL_1212516, EPI_ISL_1212614, EPI_ISL_1212618, EPI_ISL_1212694, EPI_ISL_1212710, EPI_ISL_1212729, EPI_ISL_1212812, EPI_ISL_1216066, EPI_ISL_1216144, EPI_ISL_1216155, EPI_ISL_1216162, EPI_ISL_1216173, EPI_ISL_1216195, EPI_ISL_1216199, EPI_ISL_1216210, EPI_ISL_1216259, EPI_ISL_1216273, EPI_ISL_1216275, EPI_ISL_1216284, EPI_ISL_1216289, EPI_ISL_1216332, EPI_ISL_1216366, EPI_ISL_1216373 to 1216375, EPI_ISL_1216383, EPI_ISL_1216641, EPI_ISL_1216690, EPI_ISL_1216779, EPI_ISL_1281997, EPI_ISL_1282000, EPI_ISL_1282017, EPI_ISL_1282102, EPI_ISL_1282141, EPI_ISL_1282158, EPI_ISL_1282228, EPI_ISL_1282244, EPI_ISL_1282253, EPI_ISL_1282258, EPI_ISL_1282279, EPI_ISL_1282299, EPI_ISL_1284558, EPI_ISL_1284562, EPI_ISL_1284569, EPI_ISL_1284578, EPI_ISL_1284614, EPI_ISL_1284620, EPI_ISL_1284623 to 1284624, EPI_ISL_1284635, EPI_ISL_1284663, EPI_ISL_1284670, EPI_ISL_1284674, EPI_ISL_1284698, EPI_ISL_1284708, EPI_ISL_1284711 to 1284712, EPI_ISL_1284719, EPI_ISL_1284740, EPI_ISL_1284763, EPI_ISL_1284788, EPI_ISL_1284816, EPI_ISL_1284822 to 1284823, EPI_ISL_1284837, EPI_ISL_1284849			
see above	Bioscientia MVZ Labor Karlsruhe GmbH	Robert Koch Institute	
EPI_ISL_437498 to 437499	Biotechnology Center for Advanced Technologies	Biotechnology Center for Advanced Technologies	Abdullaev, A.; Abdurakhimov, A.; Bozorov, S.; Charishnikova, O.; D. and Turdikulova, S.; Dalimova; Dalimova, D.; Muminov, M.; Nuriddinov, S.; Tsay, E.; Tsoy, V.
EPI_ISL_414515	Biotechnology, National Centre for Disease Control	Biotechnology, National Centre for Disease Control	Bala, M.; Dhar, M.; Kumar, P.; Lall, H.; S.K. and Rakshit, P.; Saini, N.; Sharma, U.; Singh; Singh, P.; Singh, S.; Vashistha, H.
EPI_ISL_961288 to 961321, EPI_ISL_961323 to 961351, EPI_ISL_1168728 to 1168735	Biotia	Biotia	Agnes Berki; Christopher Mason; Colleen Jonsson; Courteny Hager; Dorottya Nagy-Szakal; Heather Wells; Joseph Barrows; Kristin Butcher; Mara Couto-Rodriguez; Mariah Taylor; Marilyne Debieu; Niamh O'Hara; Robert Boorstein; Siyuan Chen
EPI_ISL_745118, EPI_ISL_745134, EPI_ISL_745158 to 745159, EPI_ISL_745168 to 745169	Bishop Lavis CDC wc BLP	National Health Laboratory Service (NHLS), Tygerberg	Bronwyn Kleinhans; Eduan Wilkindon; Gert van Zyl; Houriiyah Tegally; Kayla Delaney; Susan Engelbrecht; Tulio de Oliveira; Wolfgang Preiser
EPI_ISL_450813, EPI_ISL_475540	Bla Kustens halsocentral	The Public Health Agency of Sweden	Anna Risberg; Anna-Malin Linde; Karin Tegmark-Wisell; Maria Lind Karlberg; Mattias Haukland; Mia Brytting; Olof Norrby; Olov Svartstrom; Oskar Karlsson Lindsjo; Reza Advani; Sandra Broddesson; Theresa Enkirch
EPI_ISL_876588 to 876589, EPI_ISL_1049207 to 1049208, EPI_ISL_1049210 to 1049211, EPI_ISL_1049220	Blackhawk Genomics	Pathogen Discovery, Respiratory Viruses Branch, Division of Viral Diseases, Centers for Disease Control and Prevention	Anna Uehara; Brian Lynch; Clinton R. Paden; Haibin Wang; Han Jia Ng; Jing Zhang; Krista Queen; Peter Cook; Suxiang Tong; Yan Li; Ying Tao
EPI_ISL_747238	Bogor Public Health	West Java Health Laboratory; School of Life Sciences and Technology, Institut Teknologi Bandung	Azzania Fibriani; Cut Nur Cinthia Alamanda; Ema Rahmawati; Isak Solihin; Karimatu Khoirunnisa; Miftahul Faridl; Rifky Waluyajati Rachman; Rini Robiani; Ryan Bayusantika Ristandi
EPI_ISL_1298483, EPI_ISL_1298488	Bogorodchany CRH	The Institute of Molecular Biology and Genetics of NASU	M.Tukalo et al.
EPI_ISL_1213504, EPI_ISL_1213506, EPI_ISL_1213508 to 1213509, EPI_ISL_1213511, EPI_ISL_1213513 to 1213514, EPI_ISL_1213516, EPI_ISL_1213518 to 1213519, EPI_ISL_1213521, EPI_ISL_1213523 to 1213524, EPI_ISL_1213526, EPI_ISL_1213528, EPI_ISL_1213530 to 1213531			
see above	Bohol Containerized PCR Laboratory	Philippine Genome Center	Alethea R. de Guzman; Anna Ong-Lim; Arianne A. Zamora; Asia Louisa U. Chong; Benedict A. Maralit; Candice Francheska B. Tambaoan; Carlo M. Lapid; Celia Carlos; Devon Ray Pacial; Edsel Maurice Salvaña; El King D. Morado; Eva Maria Cutiongco-de la Paz; Francis A. Tablizo; Irish Coleen A. Asin; Jaime C. Montoya; Jan Michael C. Yap; Jo-Hannah S. Llames; John Q. Wong; Joshua Gregor A. Dizon; Juan Antonio R. Magalang; Karol Sophia Agape R. Padilla; Kenneth M. Kim; Kris P. Punayan; Marc Edsel C. Ayes; Marc Jerrone R. Castro; Maria Rosario Singh-Vergeire and Cynthia P. Saloma; Maria Sofia L. Yangzon; Marissa Alejandria; Razel Nikka M. Hao; Rianna Patricia S. Cruz; Sheila Mae M. Araiza
EPI_ISL_700589	Bongolethu Clinic wc BLC	NHLS/UCT	Arash Iranzadeh; Bruna Galvao; Carolyn Williamson; Deelan Doolabh; Diana Hardie; Houriiyah Tegally; Innocent Mudau; Kruger Marais; Lynn Tyers; Marvin Hsiao; Stephen Korsman
EPI_ISL_596453	Booali laboratory, Qom, Iran. Department of Virology, School of Public Health, Tehran University of Medical Sciences, Tehran, Iran.	Genetics Research Center, University of Social Welfare and Rehabilitation Sciences	Ali Jafarpour; Azam Ghaziasadi; Hossein Najmbadi; Khadijeh Jalalvand; Kimia Kahrizi; Marzieh Mohseni; Mohammad Khazeni; Seyed Amir Momeni; Seyed Mohammad Jazayeri; Seyedeh elham Mortazavi; Zohreh Fattahi
EPI_ISL_718167, EPI_ISL_718174, EPI_ISL_718176, EPI_ISL_718178, EPI_ISL_718182, EPI_ISL_718185, EPI_ISL_718191 to 718192, EPI_ISL_718197 to 718198, EPI_ISL_718203 to 718205, EPI_ISL_718216, EPI_ISL_718218, EPI_ISL_718222			
see above	Borneo Medical Centre	Institute of Health and Community Medicine	Chan Chia Jui; Chua Hock Hin; David Perera; Ooi Mong How; Tonnii Sia Loong Loong; Wong Jyn Shan; Wong Kiing Aik
EPI_ISL_640075	Bothasig CDC wc BLD	NHLS/UCT	Arash Iranzadeh; Bruna Galvao; Carolyn Williamson; Deelan Doolabh; Diana Hardie; Innocent Mudau; Kruger Marais; Lynn Tyers; Marvin Hsiao; Stephen Korsman
EPI_ISL_965182	Botswana Harvard AIDS Institute Partnership	Botswana Harvard AIDS Institute Partnership	Boitumelo Zuze; Botshelo Radibe; David Lawrence; Dorcas Maruapula; Joseph Makhema; Mosepele Mosepele; Roger Shapiro; Shahin Lockman; Sikhulile Moyo; Simani Gaseitsiwe; Wonderful T. Choga
EPI_ISL_965178	Botswana Harvard HIV Reference Laboratory	Botswana Harvard AIDS Institute Partnership	Boitumelo Zuze; Botshelo Radibe; David Lawrence; Dorcas Maruapula; Joseph Makhema; Mosepele Mosepele; Roger Shapiro; Shahin Lockman; Sikhulile Moyo; Simani Gaseitsiwe; Wonderful T. Choga
EPI_ISL_871872, EPI_ISL_933724, EPI_ISL_935040, EPI_ISL_935042, EPI_ISL_935045, EPI_ISL_940762, EPI_ISL_940765, EPI_ISL_940768, EPI_ISL_940776, EPI_ISL_940844, EPI_ISL_943992, EPI_ISL_944590, EPI_ISL_944756 to 944757, EPI_ISL_944760 to 944761, EPI_ISL_944767, EPI_ISL_944782, EPI_ISL_944790, EPI_ISL_949068, EPI_ISL_955102, EPI_ISL_960226, EPI_ISL_960303, EPI_ISL_960312, EPI_ISL_962880, EPI_ISL_962882, EPI_ISL_962886, EPI_ISL_965181, EPI_ISL_965264, EPI_ISL_965277, EPI_ISL_965909, EPI_ISL_968214, EPI_ISL_968234, EPI_ISL_968252, EPI_ISL_968857, EPI_ISL_969034, EPI_ISL_977499, EPI_ISL_981384, EPI_ISL_981386, EPI_ISL_981548, EPI_ISL_982860, EPI_ISL_1287349, EPI_ISL_1287737, EPI_ISL_1287760, EPI_ISL_1293046, EPI_ISL_1296316, EPI_ISL_1307686, EPI_ISL_1307690, EPI_ISL_1307719			
see above	Botswana Harvard HIV Reference Laboratory	Botswana Harvard HIV Reference Laboratory	Boitumelo Zuze; Botshelo Radibe; David Lawrence; Dorcas Maruapula; Joseph; Joseph Makhema; Legodile Kooepile; Legodile T. Kooepile David Lawrence; Makhema; Mosepele Mosepele; Roger L. Shapiro; Roger Shapiro; Shahin Lockman; Sikhulile Moyo; Simani Gaseitsiwe; Thongbotho Mphoyakgosi; Wonderful Choga; Wonderful T. Choga
EPI_ISL_1135077	Bourg-en-Bresse	CNR Virus des Infections Respiratoires - France SUD	Antonin Bal; Bruno Lina; Gregory Destras; Gwendolyne Burfin; Hadrien Regue; Laurence Josset; Martine Valette; Quentin Semanas
EPI_ISL_437433, EPI_ISL_900691 to 900741	Bozeman Health Deaconess Hospital	Wiedenheft lab, Montana State University	Anna Nemudraia; Artem Nemudryi; Blake Wiedenheft; Calvin Cicha; Deann T. Snyder; Diane Bimczok; Helen Lee; Jodi F. Hedges; Joseph Nichols; Karl K. Vanderwood; Kevin Surya; Mark A. Jutila and Blake Wiedenheft; Murat Buyukyoruk; Royce Wilkinson; Tanner Wiegand

EPI_ISL_903339 to 903346, EPI_ISL_1081312 to 1081323	Bozeman Health Deaconess Hospital	Wiedenheft lab, Montana State University	Anna Nemudraia; Artem Nemudryi; Calvin Cicha; Deann T. Snyder; Diane Bimczok; Helen Lee; Jodi F. Hedges; Joseph Nichols; Karl K. Vanderwood; Mark A. Jutila and Blake Wiedenheft; Tanner Wiegand
EPI_ISL_745170, EPI_ISL_745190	Brackengate Field Hospital COVID-19 wc BRG	National Health Laboratory Service (NHLS), Tygerberg	Bronwyn Kleinhans; Eduan Wilkindon; Gert van Zyl; Houriiyah Tegally; Kayla Delaney; Susan Engelbrecht; Tulio de Oliveira; Wolfgang Preiser
EPI_ISL_1040762	Bredasdorp clinic	NHLS/UCT	Arash Iranzadeh; Bruna Galvao; Carolyn Williamson; Deelan Doolabh; Diana Hardie; Innocent Mudau; Kruger Marais; Lynn Tyers; Marvin Hsiao; Stephen Korsman
EPI_ISL_479777 to 479789	Breuer Lab, UCL	Breuer Lab, UCL	Breuer Lab
EPI_ISL_420799 to 420801	Brian D. Allgood Army Community Hospital	Pathogen Discovery, Respiratory Viruses Branch, Division of Viral Diseases, Centers for Disease Control and Prevention	Alison S. Laufer Halpin; Anne Uehara; Christopher A. Elkins; Clinton R. Paden; Haibin Wang; Jasmine Padilla; Jing Zhang; Justin Lee; Krista Queen; Mary S. Keckler; Rachel Marine; Suxiang Tong; Yan Li; Ying Tao
EPI_ISL_640036	Bridgeton CDC wc BTC	NHLS/UCT	Arash Iranzadeh; Bruna Galvao; Carolyn Williamson; Deelan Doolabh; Diana Hardie; Innocent Mudau; Kruger Marais; Lynn Tyers; Marvin Hsiao; Stephen Korsman
EPI_ISL_593478 to 593480, EPI_ISL_593553 to 593558	Brigham and Women's Hospital	Jonathan Li Laboratory	James Regan; Jonathan Z. Li; Manish C. Choudhary
EPI_ISL_632288 to 632309, EPI_ISL_648265 to 648270	Brigham and Women's Hospital	Jonathan Li laboratory	Choudhary MC; Douglas S Kwon; Esmaeilzadeh E; Etemad B; George Eng; Jonathan Z. Li; Li JZ; Manish C. Choudhary; Mohammadi A; Regan J; Upasana D. Adhikari
EPI_ISL_765583 to 765612	Brigham and Womens Hospital	Infectious Disease Program, Broad Institute of Harvard and MIT	A.E.; Adams, G.; Anahtar, M.; B.L.; B.W.; Bauer, M.; Birren; Branda, J.; Carter, A.; Cerrato, F.; Chaluvadi, S.; Chapman; Cusick, C.; D.J.; DeRuff, K.; E. and Sabeti; Flowers, K.; Gallagher, G.; Gladden-Young, A.; Gnirke, A.; Harris, J.; J.E.; K.J.; LaRocque, R.; Lagerborg, K.; Lemieux; Lin; Loreth, C.; MacInnis; Neumann, A.; Normandin, E.; P.C.; Park; Pierce, V.; Reilly, S.; Rosenberg; Rudy, M.; Ryan, E.; S.B.; Shaw, B.; Siddle; Slater, D.; Smole, S.; Tomkins-Tinch, C.; Turbett, S.
EPI_ISL_976941 to 976980, EPI_ISL_976982 to 977023, EPI_ISL_1011678 to 1011705, EPI_ISL_1067739 to 1067817, EPI_ISL_1121633 to 1121702, EPI_ISL_1121879 to 1121886, EPI_ISL_1121963 to 1121968, EPI_ISL_1121973, EPI_ISL_1253719 to 1253884			
see above	Broad Institute Clinical Research Sequencing Platform	Infectious Disease Program, Broad Institute of Harvard and MIT	Adams, G.; B.L.; B.W.; Bauer, M.; Birren; Carter, A.; Chaluvadi, S.; D.J.; DeRuff, K.; Gallagher, G.; Gladden-Young, A.; J.E.; K.J.; Lagerborg, K.; Lemieux; Loreth, C.; MacInnis; Normandin, E.; P.C.; Park; Reilly, S.; Rudy, M.; Siddle; Smole, S.; Tomkins-Tinch, C.; and Sabeti
EPI_ISL_631552 to 631553, EPI_ISL_631562, EPI_ISL_631565, EPI_ISL_631573, EPI_ISL_631800, EPI_ISL_631803, EPI_ISL_631805 to 631807, EPI_ISL_631819, EPI_ISL_631829, EPI_ISL_631864, EPI_ISL_631884, EPI_ISL_631976, EPI_ISL_631978, EPI_ISL_631981, EPI_ISL_631984, EPI_ISL_632077 to 632078, EPI_ISL_632089, EPI_ISL_632100, EPI_ISL_632114, EPI_ISL_632123, EPI_ISL_632142, EPI_ISL_632155 to 632156			
see above	Brookdale University Hospital Medical Center	New York City Public Health Laboratory	Jade Wang; et al.
EPI_ISL_631508	Brooklyn Hospital Center	New York City Public Health Laboratory	Jade Wang; et al.
EPI_ISL_603248, EPI_ISL_605791	Brotman Baty Institute for Precision Medicine	Brotman Baty Institute for Precision Medicine	Benjamin Pelle; Caitlin R. Wolf; Chris D. Frazar; Christina M. Lockwood; Deborah A. Nickerson; Erica Ryke; Helen Y. Chu; Jay Shendure; Jennifer K. Logue; Jover Lee; Lea M. Starita; Mark J. Rieder; Peter D. Han; Trevor Bedford
EPI_ISL_1201681 to 1201694, EPI_ISL_1239549, EPI_ISL_1239591, EPI_ISL_1239673, EPI_ISL_1273822	Bruss	1. National Institute of Public Health - National Institute of Hygiene; 2. Eurofins Genomics Europe Sequencing GmbH	ECDC COVID-19 WGS support team; Eurofins Genomics Europe Sequencing Team; Gierczyki Rafa; Sadkowska-Todys Magorzata; Wokowicz Tomasz; Zacharczuk Katarzyna
EPI_ISL_833343	Buenavista Rural Health Unit	Research Institute for Tropical Medicine	Catalino Demetria; Daria Manalo; Edelwisa Mercado; Francisco Gerardo Polotan; Hannah Leah Morito; Inez Andrea Medado; John Leonard Chan; Kirstyn Brunker; Ma Angelica Tujan; Othoniel Jan Onza
EPI_ISL_414520 to 414521, EPI_ISL_732524 to 732564, EPI_ISL_732658, EPI_ISL_755639, EPI_ISL_915422, EPI_ISL_915424 to 915427, EPI_ISL_915429, EPI_ISL_1000998 to 1001004, EPI_ISL_1034755 to 1034760, EPI_ISL_1046791 to 1046793			
see above	Bundeswehr Institute of Microbiology	Bundeswehr Institute of Microbiology	Alexandra Rehn; Christina Bugert; Elham Khatamzas; Enrico Georgi; Joachim Bugert; Malena Bestehorn-Willmann; Markus Antwerpen; Markus H Antwerpen and Roman Wölfel; Mathias C Walter; Mathias Walter; Michael von Bergwelt-Baildon; Mike Pillukat; Roman Wölfel; Sabine Zange
EPI_ISL_918431 to 918433, EPI_ISL_996415	Bundeswehrkrankenhaus Berlin	Bundeswehr Institute of Microbiology	Alexandra Rehn; Enrico Georgi; Malena Bestehorn-Willmann; Markus Antwerpen; Martin Müller; Mathias Walter; Mike Pillukat; Roman Wölfel; Sabine Zange
EPI_ISL_996417	Bundeswehrkrankenhaus Hamburg	Bundeswehr Institute of Microbiology	Alexandra Rehn; Enrico Georgi; Malena Bestehorn-Willmann; Markus Antwerpen; Mathias Walter; Mike Pillukat; Roman Wölfel; Sabine Zange
EPI_ISL_915430 to 915433, EPI_ISL_918430	Bundeswehrkrankenhaus Westerstede	Bundeswehr Institute of Microbiology	Alexandra Rehn; Enrico Georgi; Klaus Peter Ebert; Malena Bestehorn-Willmann; Markus Antwerpen; Mathias Walter; Roman Wölfel; Sabine Zange
EPI_ISL_918424 to 918425	Bundeswehrzentralkrankenhaus Koblenz	Bundeswehr Institute of Microbiology	Alexandra Rehn; Enrico Georgi; Ingo Fengler; Malena Bestehorn-Willmann; Markus Antwerpen; Mathias Walter; Ralf Hagen; Roman Wölfel; Sabine Zange
EPI_ISL_424981 to 424982, EPI_ISL_424984 to 424986, EPI_ISL_424988 to 424990, EPI_ISL_424992	Bureau of Laboratories, Michigan Department of Health and Human Services	Michigan Department of Health and Human Services	Blankenship HM; Riner D; Soehnlen MK
EPI_ISL_906884 to 906919	Bureau of Public Health Laboratories, Florida Department of Health (BPHL, FLDOH)	Bureau of Public Health Laboratories, Florida Department of Health (BPHL, FLDOH)	Blanton, J.; Schmedes, S.
EPI_ISL_833336	Bureau of Quarantine	Research Institute for Tropical Medicine	Catalino Demetria; Daria Manalo; Edelwisa Mercado; Francisco Gerardo Polotan; Hannah Leah Morito; Inez Andrea Medado; John Leonard Chan; Kirstyn Brunker; Ma Angelica Tujan; Othoniel Jan Onza
EPI_ISL_1298474	Burshtyn CCH	The Institute of Molecular Biology and Genetics of NASU	M.Tukalo et al.
EPI_ISL_450832	Byjorden vardcentral	The Public Health Agency of Sweden	Anna Risberg; Anna-Malin Linde; Karin Tegmark-Wisell; Maria Lind Karlberg; Mia Brytting; Olov Svartstrom; Oskar Karlsson Lindsjo; Pernilla Brunman; Theresa Enkirch
EPI_ISL_524476	Bülach Hospital	Institute of Medical Virology, University of Zurich	Alexandra Trkola; Andrea Zbinden; Fiona Steiner; Gabriela Ziltener; Jon Huder; Jürg Böni; Maryam Zaheri; Michael Huber; Patrick Redli; Riccarda Capaul; Stefan Schmutz; Verena Kufner
EPI_ISL_445320	C.C.SALUD FAMILIAR PADRE FELIX DONOSO G.	Instituto de Salud Publica de Chile	Alejandra Acevedo; Andrés E Castillo; Bárbara Parra; Carolina Tambley; Gabriel Leal; Jaime Lagos; Jorge Fernandez; Loredana Arata; Patricia Bustos; Paz Tapia; Rodrigo Fasce; Winston Andrade
EPI_ISL_445318	C.DE SALUD FAMILIAR PABLO NERUDA	Instituto de Salud Publica de Chile	Alejandra Acevedo; Andrés E Castillo; Bárbara Parra; Carolina Tambley; Gabriel Leal; Jaime Lagos; Jorge Fernandez; Loredana Arata; Patricia Bustos; Paz Tapia; Rodrigo Fasce; Winston Andrade
EPI_ISL_1241704	C.H Peltzer - la Tourelle	GIGA Medical Genomics	Bouchra Boujemla; Cécile Meex; Keith Durkin; Maria Artesi; Marie-Pierre Hayette; Nathalie Renotte; Pierrette Melin; Raphaël Boreux; Sébastien Bontems; Vincent Bours
EPI_ISL_1085408	C.H du Cotentin	Department of Virology, Henri Mondor University Hospital, Assistance Publique Hôpitaux de Paris, Université Paris-Est Créteil, INSERM U955	Alexandre Soulier; Christophe Rodriguez; Elisabeth Trawinski; Guillaume Gricourt; Jean-Michel Pawlotsky; Melissa N'Debi; Slim Fourati; Vanessa Demontant
EPI_ISL_1076435	C.H.Bois de l'Abbaye	GIGA Medical Genomics	Bouchra Boujemla; Cécile Meex; Keith Durkin; Maria Artesi; Marie-Pierre Hayette; Nathalie Renotte; Pierrette Melin; Raphaël Boreux; Sébastien Bontems; Vincent Bours
EPI_ISL_1058579 to 1058580, EPI_ISL_1058602, EPI_ISL_1241692	C.H.C MontLégia	GIGA Medical Genomics	Bouchra Boujemla; Cécile Meex; Keith Durkin; Maria Artesi; Marie-Pierre Hayette; Nathalie Renotte; Pierrette Melin; Raphaël Boreux; Sébastien Bontems; Vincent Bours
EPI_ISL_1085319	C.H.I. ELBEUF/LOUVIERS	Department of Virology, Henri Mondor University Hospital, Assistance Publique Hôpitaux de Paris, Université Paris-Est Créteil, INSERM U955	Alexandre Soulier; Christophe Rodriguez; Elisabeth Trawinski; Guillaume Gricourt; Jean-Michel Pawlotsky; Melissa N'Debi; Slim Fourati; Vanessa Demontant
EPI_ISL_912554 to 912556, EPI_ISL_912573	C.H.R. d'Orléans - Hôpital de la Source	National Reference Center for Viruses of Respiratory	Angela Brisebarre; Camille Capel; Etienne Simon-Lorière; Guinard Jérome; Marion Barbet; Maud Vanpeene; Méline Bizard; Sylvie Behillil; Sylvie van der

		Infections, Institut Pasteur, Paris	Werf; Vincent Enouf
EPI_ISL_1135028	C.H.R.U. MONTPIED	CNR Virus des Infections Respiratoires - France SUD	Antonin Bal; Bruno Lina; Gregory Destras; Gwendolyne Burfin; Hadrien Regue; Laurence Josset; Martine Valette; Quentin Semanas
EPI_ISL_539531	C.H.U Nuestra Señora de Candelaria	Instituto de Salud Carlos III	A. Monzón; F. Casas; I; I. Jiménez; Iglesias-Caballero; M. Camarero; M. Cuesta; M. González-Esguevillas; M. Molinero Calamita; M. Zaballos; O. Díez; P. Jiménez; S. Juliá; S. Pozo; S. Varona
EPI_ISL_884866 to 884870, EPI_ISL_1184809 to 1184824, EPI_ISL_1185016 to 1185025, EPI_ISL_1185030, EPI_ISL_1185042, EPI_ISL_1185050, EPI_ISL_1185052, EPI_ISL_1185059 to 1185061, EPI_ISL_1185063 to 1185064, EPI_ISL_1185067 to 1185069, EPI_ISL_1185077 to 1185080, EPI_ISL_1185084, EPI_ISL_1185088 to 1185090, EPI_ISL_1185096, EPI_ISL_1185099, EPI_ISL_1185103, EPI_ISL_1185119, EPI_ISL_1185122, EPI_ISL_1185132 to 1185133, EPI_ISL_1185135, EPI_ISL_1185138 to 1185139, EPI_ISL_1185144, EPI_ISL_1185147, EPI_ISL_1185150, EPI_ISL_1185153, EPI_ISL_1185155 to 1185156, EPI_ISL_1185158, EPI_ISL_1185166, EPI_ISL_1185168 to 1185169, EPI_ISL_1185173, EPI_ISL_1185177 to 1185178, EPI_ISL_1185182, EPI_ISL_1185184, EPI_ISL_1185186, EPI_ISL_1185188, EPI_ISL_1185190, EPI_ISL_1185192 to 1185193, EPI_ISL_1185195 to 1185196, EPI_ISL_1185202 to 1185203, EPI_ISL_1185205, EPI_ISL_1185208, EPI_ISL_1185210 to 1185211, EPI_ISL_1185213, EPI_ISL_1185220, EPI_ISL_1185223, EPI_ISL_1185226, EPI_ISL_1185230, EPI_ISL_1185244, EPI_ISL_1185246 to 1185249, EPI_ISL_1185253 to 1185254, EPI_ISL_1185256, EPI_ISL_1185260, EPI_ISL_1185264 to 1185265, EPI_ISL_1185268, EPI_ISL_1185270, EPI_ISL_1185275 to 1185276, EPI_ISL_1185279, EPI_ISL_1185282, EPI_ISL_1185289 to 1185290, EPI_ISL_1185293, EPI_ISL_1185295 to 1185297, EPI_ISL_1185299, EPI_ISL_1185303, EPI_ISL_1185307, EPI_ISL_1185310 to 1185311, EPI_ISL_1185314, EPI_ISL_1185316 to 1185317, EPI_ISL_1185320 to 1185321, EPI_ISL_1185327, EPI_ISL_1185330 to 1185339, EPI_ISL_1185341 to 1185344, EPI_ISL_1185347 to 1185350, EPI_ISL_1185354, EPI_ISL_1185366, EPI_ISL_1185370 to 1185371, EPI_ISL_1185373 to 1185375, EPI_ISL_1185377 to 1185381, EPI_ISL_1185384, EPI_ISL_1185386, EPI_ISL_1185389 to 1185392, EPI_ISL_1185396 to 1185398, EPI_ISL_1185400, EPI_ISL_1185402 to 1185406, EPI_ISL_1185411, EPI_ISL_1185415 to 1185416, EPI_ISL_1185418 to 1185421, EPI_ISL_1185423, EPI_ISL_1185426, EPI_ISL_1185429, EPI_ISL_1185433 to 1185435, EPI_ISL_1185437, EPI_ISL_1185441 to 1185442, EPI_ISL_1185444 to 1185446, EPI_ISL_1185450 to 1185451, EPI_ISL_1185453, EPI_ISL_1185458, EPI_ISL_1185462, EPI_ISL_1185464 to 1185465, EPI_ISL_1185467, EPI_ISL_1185470, EPI_ISL_1185472 to 1185473, EPI_ISL_1185477 to 1185478, EPI_ISL_1185482 to 1185485, EPI_ISL_1185490, EPI_ISL_1185492, EPI_ISL_1185494 to 1185495, EPI_ISL_1185500, EPI_ISL_1185506, EPI_ISL_1185508, EPI_ISL_1185510 to 1185515, EPI_ISL_1185520, EPI_ISL_1185522 to 1185525, EPI_ISL_1185527, EPI_ISL_1185530 to 1185532, EPI_ISL_1234544 to 1234545, EPI_ISL_1234550 to 1234551, EPI_ISL_1234554, EPI_ISL_1234557, EPI_ISL_1234571, EPI_ISL_1234575 to 1234576, EPI_ISL_1234581 to 1234582, EPI_ISL_1234589, EPI_ISL_1234591 to 1234595, EPI_ISL_1234599, EPI_ISL_1234601, EPI_ISL_1234605, EPI_ISL_1234608, EPI_ISL_1234619 to 1234620, EPI_ISL_1234623 to 1234625, EPI_ISL_1234627, EPI_ISL_1234633 to 1234635, EPI_ISL_1234638 to 1234639, EPI_ISL_1234644, EPI_ISL_1234649 to 1234652, EPI_ISL_1234656, EPI_ISL_1234658, EPI_ISL_1234663, EPI_ISL_1234668 to 1234669, EPI_ISL_1234673, EPI_ISL_1234681, EPI_ISL_1234695, EPI_ISL_1234699 to 1234700, EPI_ISL_1234703 to 1234704, EPI_ISL_1234712 to 1234715, EPI_ISL_1234717, EPI_ISL_1234719, EPI_ISL_1234722, EPI_ISL_1234730 to 1234731, EPI_ISL_1234733 to 1234735, EPI_ISL_1234740, EPI_ISL_1234747 to 1234748, EPI_ISL_1234750, EPI_ISL_1234752 to 1234754, EPI_ISL_1234761, EPI_ISL_1234766, EPI_ISL_1234771, EPI_ISL_1234774, EPI_ISL_1234778, EPI_ISL_1234780 to 1234781, EPI_ISL_1234784, EPI_ISL_1235060, EPI_ISL_1235063, EPI_ISL_1235068, EPI_ISL_1235070, EPI_ISL_1235074, EPI_ISL_1235079, EPI_ISL_1235084, EPI_ISL_1235092, EPI_ISL_1235095, EPI_ISL_1235097, EPI_ISL_1235104, EPI_ISL_1235107, EPI_ISL_1235112, EPI_ISL_1235118 to 1235119, EPI_ISL_1235121, EPI_ISL_1235123, EPI_ISL_1235125 to 1235128, EPI_ISL_1235132 to 1235133, EPI_ISL_1235135 to 1235136, EPI_ISL_1235138, EPI_ISL_1235140, EPI_ISL_1235144, EPI_ISL_1235148, EPI_ISL_1235150, EPI_ISL_1235152, EPI_ISL_1235162, EPI_ISL_1235176, EPI_ISL_1235182, EPI_ISL_1235186, EPI_ISL_1235190, EPI_ISL_1235192 to 1235193, EPI_ISL_1235195, EPI_ISL_1235197, EPI_ISL_1235199, EPI_ISL_1235203, EPI_ISL_1235206, EPI_ISL_1235212, EPI_ISL_1235220, EPI_ISL_1235222, EPI_ISL_1235226, EPI_ISL_1235229, EPI_ISL_1235232, EPI_ISL_1235234, EPI_ISL_1235236, EPI_ISL_1235238, EPI_ISL_1235242, EPI_ISL_1235248, EPI_ISL_1235251 to 1235252, EPI_ISL_1235254, EPI_ISL_1235269, EPI_ISL_1235272 to 1235274, EPI_ISL_1235282, EPI_ISL_1235285, EPI_ISL_1235287, EPI_ISL_1235290 to 1235291, EPI_ISL_1235295, EPI_ISL_1235298, EPI_ISL_1235307, EPI_ISL_1235310, EPI_ISL_1235317, EPI_ISL_1235322, EPI_ISL_1235346, EPI_ISL_1235351, EPI_ISL_1235354, EPI_ISL_1235360, EPI_ISL_1235367, EPI_ISL_1235372 to 1235373, EPI_ISL_1235375, EPI_ISL_1235377 to 1235378, EPI_ISL_1235381, EPI_ISL_1235383 to 1235384, EPI_ISL_1235400 to 1235401, EPI_ISL_1235403, EPI_ISL_1235410, EPI_ISL_1235416 to 1235419, EPI_ISL_1235424, EPI_ISL_1235427, EPI_ISL_1235434 to 1235435, EPI_ISL_1235438, EPI_ISL_1235441 to 1235442, EPI_ISL_1235450 to 1235451, EPI_ISL_1235455, EPI_ISL_1235468 to 1235469, EPI_ISL_1235472 to 1235473, EPI_ISL_1235478, EPI_ISL_1235488, EPI_ISL_1235490, EPI_ISL_1235496, EPI_ISL_1235498, EPI_ISL_1235501 to 1235502, EPI_ISL_1235506 to 1235507, EPI_ISL_1235509, EPI_ISL_1235511, EPI_ISL_1235515, EPI_ISL_1235521 to 1235522, EPI_ISL_1235525 to 1235526, EPI_ISL_1235528, EPI_ISL_1235539 to 1235540, EPI_ISL_1235544 to 1235547, EPI_ISL_1235549, EPI_ISL_1235551, EPI_ISL_1235558, EPI_ISL_1235570, EPI_ISL_1235572, EPI_ISL_1235578, EPI_ISL_1235582, EPI_ISL_1235588, EPI_ISL_1235591, EPI_ISL_1235596, EPI_ISL_1235600, EPI_ISL_1235602, EPI_ISL_1235604, EPI_ISL_1235606, EPI_ISL_1235608			
see above	CA DPH Viral and Rickettsial Disease Laboratory	Chan-Zuckerberg Biohub	CZB Cliahub Consortium
EPI_ISL_576177	CA, CDPH, Viral and Rickettsial Disease Laboratory	Pathogen Discovery, Respiratory Viruses Branch, Division of Viral Diseases, Centers for Disease Control and Prevention	Anna Uehara; Brian Lynch; Clinton R. Paden; Haibin Wang; Jing Zhang; Krista Queen; Peter Cook; Suxiang Tong; Yan Li; Ying Tao
EPI_ISL_1272873 to 1272895	CA-Los Angeles County Public Health Laboratory	Centers for Disease Control and Prevention Division of Viral Diseases, Pathogen Discovery	Anna Montmayeur; Anna Uehara; Ben L. Rambo-Martin; Clinton R. Paden; Dhwani Batra; Haibin Wang; Jasmine Padilla; Jing Zhang; Justin Lee; Katie Dillon; Krista Queen; Kristen Knipe; Kristine Lacek; Lori Rowe; Mark Burroughs; Matthew Schmerer; Mili Sheth; Peter W. Cook; Rachel Marine; Sam Shepard; Sarah Nobles; Shoshona Le; Suxiang Tong; Yan Li; Ying Tao
EPI_ISL_1225949 to 1225957, EPI_ISL_1226169 to 1226176	CA-Los Angeles County Public Health Laboratory	Genomics and Discovery, Respiratory Viruses Branch, Division of Viral Diseases, Centers for Disease Control and Prevention	Anna Montmayeur; Anna Uehara; Ben L. Rambo-Martin; Clinton R. Paden; Dhwani Batra; Haibin Wang; Jasmine Padilla; Jing Zhang; Justin Lee; Katie Dillon; Krista Queen; Kristen Knipe; Kristine Lacek; Lori Rowe; Mark Burroughs; Matthew Schmerer; Mili Sheth; Peter W. Cook; Rachel Marine; Sam Shepard; Sarah Nobles; Shoshona Le; Suxiang Tong; Yan Li; Ying Tao
EPI_ISL_1094325, EPI_ISL_1094361 to 1094363, EPI_ISL_1094559 to 1094571	CA-Los Angeles County Public Health Laboratory	Respiratory Viruses Branch, Division of Viral Diseases, Centers for Disease Control and Prevention	Anna Montmayeur; Anna Uehara; Ben L. Rambo-Martin; Clinton R. Paden; Dhwani Batra; Haibin Wang; Jasmine Padilla; Jing Zhang; Justin Lee; Krista Queen; Lori Rowe; Mark Burroughs; Mili Sheth; Peter W. Cook; Rachel Marine; Sarah Nobles; Suxiang Tong; Yan Li; Ying Tao
EPI_ISL_467516	CAPRISA	KRISP, KZN Research Innovation and Sequencing Platform	Chimukangara B; Giandhari J; Khan S; Lessells R; Mdlalose K; Pillay S; Tegally H; Wilkinson E; York D; de Oliveira T
EPI_ISL_1297307	CBL AVICENNE	CNR Virus des Infections Respiratoires - France SUD	Antonin Bal; Bruno Lina; Gregory Destras; Gwendolyne Burfin; Hadrien Regue; Laurence Josset; Martine Valette; Quentin Semanas
EPI_ISL_1190811 to 1190812	CBL NARBONNE	CNR Virus des Infections Respiratoires - France SUD	Antonin Bal; Bruno Lina; Gregory Destras; Gwendolyne Burfin; Hadrien Règue; Laurence Josset; Martine Valette; Quentin Semanas
EPI_ISL_1147933	CBT - Centrum für Blutgerinnungsstörungen und Transfusionsmedizin	Robert Koch Institute	
EPI_ISL_586549 to 586550, EPI_ISL_586555 to 586561, EPI_ISL_590690	CCC,Veraval	Gujarat Biotechnology Research Centre	A M Kadri; Afzal Ansari; Apurvasinh Puvar; Chaitanya Joshi; Dinesh Kumar; Harsh Bakshi; Janvi Raval; Jignesh Parmar; Jitendra Bamrotia; Komal Patel; Labdhi Pandya; Madhvi Joshi; Maharshi Pandya; Monika Gandhi; Nidhi Patel; Nikha Trivedi; Nitin Savaliya; Pinal Trivedi; R D Dixit; Raghawendra Kumar; Zarna Patel; Zuber Saiyed
EPI_ISL_1060877 to 1060883, EPI_ISL_1060885, EPI_ISL_1060888 to 1060890, EPI_ISL_1060892 to 1060899, EPI_ISL_1060901, EPI_ISL_1060903, EPI_ISL_1060905 to 1060909, EPI_ISL_1060911 to 1060913, EPI_ISL_1060915 to 1060917, EPI_ISL_1060919, EPI_ISL_1060921 to 1060922, EPI_ISL_1060929 to 1060942, EPI_ISL_1060944 to 1060946, EPI_ISL_1060948, EPI_ISL_1060950 to 1060953, EPI_ISL_1060955 to 1060962, EPI_ISL_1060964 to 1060966, EPI_ISL_1060968 to 1060970, EPI_ISL_1060973, EPI_ISL_1060975 to 1060977, EPI_ISL_1060979 to 1060980, EPI_ISL_1060982 to 1060989, EPI_ISL_1060991 to 1060993, EPI_ISL_1060996, EPI_ISL_1060998 to 1061005, EPI_ISL_1061007 to 1061024, EPI_ISL_1061026, EPI_ISL_1061028, EPI_ISL_1061030 to 1061032, EPI_ISL_1064736 to 1064750			
see above	CDL Laboratorio Santos e Vidal LTDA.	Instituto de Medicina Tropical de Sao Paulo	Brazil-UK Centre for Arbovirus Discovery Diagnosis Genomics and Epidemiology (CADDE) Genomic Network - Instituto de Medicina Tropical
EPI_ISL_1242005, EPI_ISL_1265511, EPI_ISL_1265672, EPI_ISL_1265688, EPI_ISL_1287759	CDP HUSSEL VIENNE	CNR Virus des Infections Respiratoires - France SUD	Antonin Bal; Bruno Lina; Gregory Destras; Gwendolyne Burfin; Hadrien Regue; Laurence Josset; Martine Valette; Quentin Semanas
EPI_ISL_1220348, EPI_ISL_1265859 to 1265861, EPI_ISL_1313024 to 1313025, EPI_ISL_1313030 to 1313033, EPI_ISL_1313133 to 1313134, EPI_ISL_1313137 to 1313139, EPI_ISL_1313146, EPI_ISL_1313156, EPI_ISL_1313671			
see above	CDP PIERRE OUDOT	CNR Virus des Infections Respiratoires - France SUD	Antonin Bal; Bruno Lina; Gregory Destras; Gwendolyne Burfin; Hadrien Regue; Laurence Josset; Martine Valette; Quentin Semanas
EPI_ISL_535662 to 535715	CDPH, Microbial Diseases Laboratory	Pathogen Discovery, Respiratory Viruses Branch, Division of Viral Diseases, Centers for Disease Control and Prevention	Anna Uehara; Brian Lynch; Clinton R. Paden; Haibin Wang; Jing Zhang; Krista Queen; Rachel Marine; Suxiang Tong; Yan Li; Ying Tao
EPI_ISL_1094536	CDPH, Microbial Diseases Laboratory	Respiratory Viruses Branch, Division of Viral Diseases, Centers for Disease Control and Prevention	Anna Montmayeur; Anna Uehara; Ben L. Rambo-Martin; Clinton R. Paden; Dhwani Batra; Haibin Wang; Jasmine Padilla; Jing Zhang; Justin Lee; Krista Queen; Lori Rowe; Mark Burroughs; Mili Sheth; Peter W. Cook; Rachel Marine; Sarah Nobles; Suxiang Tong; Yan Li; Ying Tao
EPI_ISL_1094216, EPI_ISL_1094224, EPI_ISL_1094233, EPI_ISL_1094268, EPI_ISL_1094348, EPI_ISL_1094577, EPI_ISL_1094579 to 1094581, EPI_ISL_1094583 to 1094589, EPI_ISL_1095190 to 1095201, EPI_ISL_1095203			
see above	CDPH, Viral and Rickettsial Disease Laboratory	Respiratory Viruses Branch, Division of Viral Diseases, Centers for Disease Control and Prevention	Anna Montmayeur; Anna Uehara; Ben L. Rambo-Martin; Clinton R. Paden; Dhwani Batra; Haibin Wang; Jasmine Padilla; Jing Zhang; Justin Lee; Krista Queen; Lori Rowe; Mark Burroughs; Mili Sheth; Peter W. Cook; Rachel Marine; Sarah Nobles; Suxiang Tong; Yan Li; Ying Tao
EPI_ISL_486852	CDRI/SGPGI	CSIR-CDRI/SGPGI	Dharam Veer Singh; Rahul Vishvkarma; Rajender Singh; Ravishankar Ramachandran; Saumya Sarkar; Tapas Kumar Kundu; Uday Ghoshal; Ujjala Ghoshal
EPI_ISL_605780 to 605782	CEIRS Data Processing and Coordinating Center, St. Jude Center of Excellence for Influenza Research and Surveillance (CEIRS)	CEIRS Data Processing and Coordinating Center, St. Jude Center of Excellence for Influenza Research and Surveillance (CEIRS)	A.E.; Ali; El-Guindy; El-Sayes, M.; El-Shesheny, R.; El-Taweel, A.; Gomaa, M.; Kamel; Kandeil, A.; Kayali, G.; Kayed; Kutkat, O.; M.A.; M.N.; Mahmoud; Mahrous, N.; Moatasim, Y.; Mostafa, A.; N.M.; Naguib, A.; Roshdy; S.H.; Shehata, M.; Showky, S.; W.H.; Webby, R.
EPI_ISL_1303032	CEIRS Data Processing and Coordinating Center, Center for Research on Influenza Pathogenesis (CRIP)	CEIRS Data Processing and Coordinating Center, Center for Research on Influenza Pathogenesis (CRIP)	Adolfo Garcia-Sastre; Adriana van De Guchte; Ajay Obla; Ana S. Gonzalez-Reiche; Bremy Alburquerque; Edward C. Holmes; Eileen Serrano; Erick Salinas; Hala Alshammary; Harm van Bakel; Jayeeta Dutta; Jorge Levican; Juan Soto; Leonardo I. Almonacid; Matthew M. Hernandez; Melissa Smith; Rafael A. Medina; Robert Sebra; Shwetha Hara Sridhar; Tamara García-Salum; Viviana Simon; Ying-Chih Wang; Zenab Khan
EPI_ISL_1190813 to 1190814	CENTRE BIOLOGIE BEZIERS	CNR Virus des Infections Respiratoires - France SUD	Antonin Bal; Bruno Lina; Gregory Destras; Gwendolyne Burfin; Hadrien Règue; Laurence Josset; Martine Valette; Quentin Semanas
EPI_ISL_1191709, EPI_ISL_1191726	CENTRE HOSPITALIER	CNR Virus des Infections Respiratoires - France SUD	Antonin Bal; Bruno Lina; Gregory Destras; Gwendolyne Burfin; Hadrien Regue; Laurence Josset; Martine Valette; Quentin Semanas
EPI_ISL_1265515 to 1265516, EPI_ISL_1312945 to 1312946, EPI_ISL_1313565, EPI_ISL_1313568, EPI_ISL_1313574 to 1313575, EPI_ISL_1313589, EPI_ISL_1313596, EPI_ISL_1313897 to 1313898, EPI_ISL_1313902, EPI_ISL_1313998, EPI_ISL_1314010, EPI_ISL_1314015			
see above	CENTRE HOSPITALIER DU HAUT BUGEY	CNR Virus des Infections Respiratoires - France SUD	Antonin Bal; Bruno Lina; Gregory Destras; Gwendolyne Burfin; Hadrien Regue; Laurence Josset; Martine Valette; Quentin Semanas
EPI_ISL_1265887	CENTRE HOSPITALIER AGEN	CNR Virus des Infections Respiratoires - France SUD	Antonin Bal; Bruno Lina; Gregory Destras; Gwendolyne Burfin; Hadrien Regue; Laurence Josset; Martine Valette; Quentin Semanas

EPI_ISL_1190776	CENTRE HOSPITALIER ANNECY GENEVOIS	CNR Virus des Infections Respiratoires - France SUD	Antonin Bal; Bruno Lina; Gregory Destras; Gwendolyne Burfin; Hadrien Règue; Laurence Josset; Martine Valette; Quentin Semanas
EPI_ISL_1165953, EPI_ISL_1165960	CENTRE HOSPITALIER ARDECHE NORD	CNR Virus des Infections Respiratoires - France SUD	Antonin Bal; Bruno Lina; Gregory Destras; Gwendolyne Burfin; Hadrien Regue; Laurence Josset; Martine Valette; Quentin Semanas
EPI_ISL_1313082	CENTRE HOSPITALIER CAREMEAU	CNR Virus des Infections Respiratoires - France SUD	Antonin Bal; Bruno Lina; Gregory Destras; Gwendolyne Burfin; Hadrien Regue; Laurence Josset; Martine Valette; Quentin Semanas
EPI_ISL_1166050	CENTRE HOSPITALIER DAX	CNR Virus des Infections Respiratoires - France SUD	Antonin Bal; Bruno Lina; Gregory Destras; Gwendolyne Burfin; Hadrien Regue; Laurence Josset; Martine Valette; Quentin Semanas
EPI_ISL_1220309, EPI_ISL_1220312 to 1220314, EPI_ISL_1265717, EPI_ISL_1265773, EPI_ISL_1265853 to 1265857, EPI_ISL_1297436, EPI_ISL_1312943, EPI_ISL_1312992, EPI_ISL_1312999, EPI_ISL_1313570, EPI_ISL_1313584, EPI_ISL_1313699			
see above	CENTRE HOSPITALIER DE BOURG EN BRESSE	CNR Virus des Infections Respiratoires - France SUD	Antonin Bal; Bruno Lina; Gregory Destras; Gwendolyne Burfin; Hadrien Regue; Laurence Josset; Martine Valette; Quentin Semanas
EPI_ISL_1190837, EPI_ISL_1312948, EPI_ISL_1313558, EPI_ISL_1313707	CENTRE HOSPITALIER DE CANNES	CNR Virus des Infections Respiratoires - France SUD	Antonin Bal; Bruno Lina; Gregory Destras; Gwendolyne Burfin; Hadrien Regue; Hadrien Règue; Laurence Josset; Martine Valette; Quentin Semanas
EPI_ISL_1190787 to 1190797, EPI_ISL_1314033	CENTRE HOSPITALIER DE LA COTE BASQUE	CNR Virus des Infections Respiratoires - France SUD	Antonin Bal; Bruno Lina; Gregory Destras; Gwendolyne Burfin; Hadrien Regue; Hadrien Règue; Laurence Josset; Martine Valette; Quentin Semanas
EPI_ISL_1265786, EPI_ISL_1297435	CENTRE HOSPITALIER DE MACON	CNR Virus des Infections Respiratoires - France SUD	Antonin Bal; Bruno Lina; Gregory Destras; Gwendolyne Burfin; Hadrien Regue; Laurence Josset; Martine Valette; Quentin Semanas
EPI_ISL_1134969	CENTRE HOSPITALIER DE VOIRON	CNR Virus des Infections Respiratoires - France SUD	Antonin Bal; Bruno Lina; Gregory Destras; Gwendolyne Burfin; Hadrien Regue; Laurence Josset; Martine Valette; Quentin Semanas
EPI_ISL_1312982	CENTRE HOSPITALIER DRAGUIGNAN	CNR Virus des Infections Respiratoires - France SUD	Antonin Bal; Bruno Lina; Gregory Destras; Gwendolyne Burfin; Hadrien Regue; Laurence Josset; Martine Valette; Quentin Semanas
EPI_ISL_1220310	CENTRE HOSPITALIER DU HAUT BUGEY	CNR Virus des Infections Respiratoires - France SUD	Antonin Bal; Bruno Lina; Gregory Destras; Gwendolyne Burfin; Hadrien Regue; Laurence Josset; Martine Valette; Quentin Semanas
EPI_ISL_1241883	CENTRE HOSPITALIER EDMOND GARCIN	CNR Virus des Infections Respiratoires - France SUD	Antonin Bal; Bruno Lina; Gregory Destras; Gwendolyne Burfin; Hadrien Regue; Laurence Josset; Martine Valette; Quentin Semanas
EPI_ISL_1135027	CENTRE HOSPITALIER INTERCOMMUNAL	CNR Virus des Infections Respiratoires - France SUD	Antonin Bal; Bruno Lina; Gregory Destras; Gwendolyne Burfin; Hadrien Regue; Laurence Josset; Martine Valette; Quentin Semanas
EPI_ISL_912614	CENTRE HOSPITALIER JACQUES LACARIN LABORATOIRE	National Reference Center for Viruses of Respiratory Infections, Institut Pasteur, Paris	Angela Brisebarre; Camille Capel; Etienne Simon-Lorière; Marion Barbet; Maud Vanpeene; Méline Bizard; Sylvie Behillil; Sylvie van der Werf; Vincent Enouf
EPI_ISL_912539, EPI_ISL_912580 to 912583	CENTRE HOSPITALIER LEON BINET	National Reference Center for Viruses of Respiratory Infections, Institut Pasteur, Paris	Angela Brisebarre; Camille Capel; Etienne Simon-Lorière; Marion Barbet; Maud Vanpeene; Meziane Ilham; Méline Bizard; Sylvie Behillil; Sylvie van der Werf; Vincent Enouf
EPI_ISL_1135053, EPI_ISL_1201872, EPI_ISL_1241986, EPI_ISL_1265483, EPI_ISL_1265510, EPI_ISL_1265720, EPI_ISL_1265726, EPI_ISL_1265758, EPI_ISL_1265884 to 1265886, EPI_ISL_1297216, EPI_ISL_1312827, EPI_ISL_1312928, EPI_ISL_1312932, EPI_ISL_1312958, EPI_ISL_1312984, EPI_ISL_1313018, EPI_ISL_1313021, EPI_ISL_1313652, EPI_ISL_1313674, EPI_ISL_1313678 to 1313679, EPI_ISL_1313687, EPI_ISL_1313705 to 1313706, EPI_ISL_1313907, EPI_ISL_1313917, EPI_ISL_1313994			
see above	CENTRE HOSPITALIER LUCIEN HUSSEL	CNR Virus des Infections Respiratoires - France SUD	Antonin Bal; Bruno Lina; Gregory Destras; Gwendolyne Burfin; Hadrien Regue; Laurence Josset; Martine Valette; Quentin Semanas
EPI_ISL_1190784	CENTRE HOSPITALIER PARIS ST JOSEPH	CNR Virus des Infections Respiratoires - France SUD	Antonin Bal; Bruno Lina; Gregory Destras; Gwendolyne Burfin; Hadrien Règue; Laurence Josset; Martine Valette; Quentin Semanas
EPI_ISL_1134904 to 1134906, EPI_ISL_1241962 to 1241963, EPI_ISL_1241967, EPI_ISL_1241977 to 1241978, EPI_ISL_1241980, EPI_ISL_1241982, EPI_ISL_1241992 to 1241993, EPI_ISL_1265465, EPI_ISL_1265488, EPI_ISL_1265501, EPI_ISL_1265517, EPI_ISL_1265553, EPI_ISL_1265555, EPI_ISL_1265591, EPI_ISL_1265594 to 1265596, EPI_ISL_1265696, EPI_ISL_1265718, EPI_ISL_1265728 to 1265729, EPI_ISL_1265732, EPI_ISL_1265844, EPI_ISL_1265858, EPI_ISL_1312822 to 1312823, EPI_ISL_1312927, EPI_ISL_1312957, EPI_ISL_1312962, EPI_ISL_1312968, EPI_ISL_1312988, EPI_ISL_1312993, EPI_ISL_1312998, EPI_ISL_1313559, EPI_ISL_1313578, EPI_ISL_1313603, EPI_ISL_1313615, EPI_ISL_1313627, EPI_ISL_1313637, EPI_ISL_1313648, EPI_ISL_1313656 to 1313657, EPI_ISL_1313684, EPI_ISL_1313688 to 1313689, EPI_ISL_1313696, EPI_ISL_1313698, EPI_ISL_1313704, EPI_ISL_1313913, EPI_ISL_1313920, EPI_ISL_1313960, EPI_ISL_1313979, EPI_ISL_1314002, EPI_ISL_1314006, EPI_ISL_1314019 to 1314020, EPI_ISL_1314029, EPI_ISL_1314031, EPI_ISL_1314036			
see above	CENTRE HOSPITALIER PIERRE OUDOT	CNR Virus des Infections Respiratoires - France SUD	Antonin Bal; Bruno Lina; Gregory Destras; Gwendolyne Burfin; Hadrien Regue; Laurence Josset; Martine Valette; Quentin Semanas
EPI_ISL_1190798 to 1190800, EPI_ISL_1287755	CENTRE HOSPITALIER POITIERS	CNR Virus des Infections Respiratoires - France SUD	Antonin Bal; Bruno Lina; Gregory Destras; Gwendolyne Burfin; Hadrien Regue; Hadrien Règue; Laurence Josset; Martine Valette; Quentin Semanas
EPI_ISL_1241958, EPI_ISL_1265551, EPI_ISL_1265710, EPI_ISL_1265807, EPI_ISL_1265877, EPI_ISL_1312829, EPI_ISL_1312959, EPI_ISL_1312990 to 1312991, EPI_ISL_1313561, EPI_ISL_1313646 to 1313647, EPI_ISL_1313666, EPI_ISL_1313677, EPI_ISL_1313908, EPI_ISL_1313921, EPI_ISL_1313942 to 1313943, EPI_ISL_1313959, EPI_ISL_1313980, EPI_ISL_1314012, EPI_ISL_1314032			
see above	CENTRE HOSPITALIER ST JOSEPH ST LUC	CNR Virus des Infections Respiratoires - France SUD	Antonin Bal; Bruno Lina; Gregory Destras; Gwendolyne Burfin; Hadrien Regue; Laurence Josset; Martine Valette; Quentin Semanas
EPI_ISL_1191685	CENTRE LEON BERARD	CNR Virus des Infections Respiratoires - France SUD	Antonin Bal; Bruno Lina; Gregory Destras; Gwendolyne Burfin; Hadrien Regue; Laurence Josset; Martine Valette; Quentin Semanas
EPI_ISL_1195275 to 1195276, EPI_ISL_1195279	CENTRO DE REFERENCIA EM SINDROMES GRIPAIS	Epiclin	Ana Paula Muterle; Carolina Comerlato; Eliana Márcia Da Ros Wendland; Fernando Hayashi Sant'Anna; Janira Prichula; Juliana Comerlato
EPI_ISL_1235688	CENTRO MEDICO COLSANITAS LA CALLEJA	Laboratorio de salud pública (Bogotá) and Gencore (Universidad de los Andes)	Alejandro Gómez; David González; Johana Hernandez Gabriela Delgado; Johanna Vargas; Luisa Sacristán; Marcela Guevara-Suarez; Silvia Restrepo
EPI_ISL_445266 to 445267	CENTRO ONCOLOGICO DEL NORTE	Instituto de Salud Publica de Chile	Alejandra Acevedo; Andrés E Castillo; Bárbara Parra; Carolina Tambley; Gabriel Leal; Jaime Lagos; Jorge Fernandez; Loredana Arata; Patricia Bustos; Paz Tapia; Rodrigo Fasce; Winston Andrade
EPI_ISL_750175	CENUR Este-Sede Rocha-UdelaR	Institut Pasteur de Montevideo	Ana Carolina Mendonça; Andres Lizasoain; Camila Simoes; Cecilia Alonso; Cecilia Salazar; Daiana Mir; Fernando Lopez-Tort; Fernando Motta; Gonzalo Bello; Ighor Arantes; Ignacio Ferrés; Jose Sotelo; Leticia Maya; Letícia Garay Martins; Luciana Appolinario; Lucia Spangenberg; Mailen Arleo; Mariana Brandes; Marilda Mendonça Siqueira; Marilda Tereza Mar da Rosa; Maria Jose Benitez-Galeano; Martín Graña; Matias Castells; Matias Victoria; Matías Salvo; Natalia Rego; Natalia Reyes; Pablo Smircich; Paola Cristina Resende; Rodney Colina; Tamara Fernandez-Calero; Tania Possi; Tatiana Schäffer Gregianini; Veronica Noya; Yasser Vega
EPI_ISL_751184 to 751190	CENUR Litoral Norte - UdelaR, Salto, Uruguay	Institut Pasteur de Montevideo	Ana Carolina Mendonça; Andres Lizasoain; Camila Simoes; Cecilia Alonso; Cecilia Salazar; Daiana Mir; Fernando Lopez-Tort; Fernando Motta; Gonzalo Bello; Ighor Arantes; Ignacio Ferrés; Jose Sotelo; Leticia Maya; Letícia Garay Martins; Luciana Appolinario; Lucia Spangenberg; Mailen Arleo; Mariana Brandes; Marilda Mendonça Siqueira; Marilda Tereza Mar da Rosa; Maria Jose Benitez-Galeano; Martín Graña; Matias Castells; Matias Victoria; Matías Salvo; Natalia Rego; Natalia Reyes; Pablo Smircich; Paola Cristina Resende; Rodney Colina; Tamara Fernandez-Calero; Tania Possi; Tatiana Schäffer Gregianini; Veronica Noya; Yasser Vega
EPI_ISL_750165	CENUR Litoral Norte - UdelaR, Salto, Uruguay.	Institut Pasteur de Montevideo	Ana Carolina Mendonça; Andres Lizasoain; Camila Simoes; Cecilia Alonso; Cecilia Salazar; Daiana Mir; Fernando Lopez-Tort; Fernando Motta; Gonzalo Bello; Ighor Arantes; Ignacio Ferrés; Jose Sotelo; Leticia Maya; Letícia Garay Martins; Luciana Appolinario; Lucia Spangenberg; Mailen Arleo; Mariana Brandes; Marilda Mendonça Siqueira; Marilda Tereza Mar da Rosa; Maria Jose Benitez-Galeano; Martín Graña; Matias Castells; Matias Victoria; Matías Salvo; Natalia Rego; Natalia Reyes; Pablo Smircich; Paola Cristina Resende; Rodney Colina; Tamara Fernandez-Calero; Tania Possi; Tatiana Schäffer Gregianini; Veronica Noya; Yasser Vega
EPI_ISL_644229 to 644230, EPI_ISL_644250 to 644254, EPI_ISL_644256, EPI_ISL_644258	CEPHR / Mater Hospital	Irish Coronavirus Sequencing Consortium - National Virus Reference Laboratory	Alejandro Abner Garcia Leon; Gabriel Gonzalez; Michael Carr; Patrick Mallon
EPI_ISL_644209 to 644228, EPI_ISL_644231 to 644249, EPI_ISL_644255, EPI_ISL_644257, EPI_ISL_644259 to 644345	CEPHR / Vincent's Hospital	Irish Coronavirus Sequencing Consortium - National Virus Reference Laboratory	Alejandro Abner Garcia Leon; Gabriel Gonzalez; Michael Carr; Patrick Mallon
EPI_ISL_861715 to 861716, EPI_ISL_861727 to 861728, EPI_ISL_862042 to 862045	CERBA	CERBA LAB	Costa JM; Haïm-Boukobza S.; Hedbaut E; Lecorche E; Malek Ramdane; Olivi M; Roquebert B; Trombert S; Verdurme L
EPI_ISL_1265673, EPI_ISL_1265681, EPI_ISL_1265695, EPI_ISL_1265708 to 1265709, EPI_ISL_1265712, EPI_ISL_1265716, EPI_ISL_1265733, EPI_ISL_1265737 to 1265738			
see above	CERBA	CNR Virus des Infections Respiratoires - France SUD	Antonin Bal; Bruno Lina; Gregory Destras; Gwendolyne Burfin; Hadrien Regue; Laurence Josset; Martine Valette; Quentin Semanas
EPI_ISL_780418	CERBA LAB	CERBA LAB	Costa JM; Haïm-Boukobza S; Hedbaut E; Lecorche E; Malek Ramdane; Olivi M; Roquebert B; Trombert S; Verdurme L
EPI_ISL_1191632, EPI_ISL_1191676, EPI_ISL_1191720 to 1191721, EPI_ISL_1239452	CERBALLIANCE	CNR Virus des Infections Respiratoires - France SUD	Antonin Bal; Bruno Lina; Gregory Destras; Gwendolyne Burfin; Hadrien Regue; Laurence Josset; Martine Valette; Quentin Semanas

EPI_ISL_1201867 to 1201871	CERBALLIANCE CHARENTES	CNR Virus des Infections Respiratoires - France SUD	Antonin Bal; Bruno Lina; Gregory Destras; Gwendolyne Burfin; Hadrien Regue; Laurence Josset; Martine Valette; Quentin Semanas
EPI_ISL_1166053	CERBALLIANCE CHARENTES PONS	CNR Virus des Infections Respiratoires - France SUD	Antonin Bal; Bruno Lina; Gregory Destras; Gwendolyne Burfin; Hadrien Regue; Laurence Josset; Martine Valette; Quentin Semanas
EPI_ISL_1313006	CERBALLIANCE CHARENTES SAINTES	CNR Virus des Infections Respiratoires - France SUD	Antonin Bal; Bruno Lina; Gregory Destras; Gwendolyne Burfin; Hadrien Regue; Laurence Josset; Martine Valette; Quentin Semanas
EPI_ISL_1265833, EPI_ISL_1265847 to 1265848, EPI_ISL_1287752 to 1287753	CERBALLIANCE PACA	CNR Virus des Infections Respiratoires - France SUD	Antonin Bal; Bruno Lina; Gregory Destras; Gwendolyne Burfin; Hadrien Regue; Laurence Josset; Martine Valette; Quentin Semanas
EPI_ISL_1313012 to 1313014	CERBALLIANCE RHONE ALPES	CNR Virus des Infections Respiratoires - France SUD	Antonin Bal; Bruno Lina; Gregory Destras; Gwendolyne Burfin; Hadrien Regue; Laurence Josset; Martine Valette; Quentin Semanas
EPI_ISL_445316	CESFAM BALMACEDA DE RENCA	Instituto de Salud Publica de Chile	Alejandra Acevedo; Andrés E Castillo; Bárbara Parra; Carolina Tambley; Gabriel Leal; Jaime Lagos; Jorge Fernandez; Loredana Arata; Patricia Bustos; Paz Tapia; Rodrigo Fasce; Winston Andrade
EPI_ISL_1312914	CH AGEN NERAC	CNR Virus des Infections Respiratoires - France SUD	Antonin Bal; Bruno Lina; Gregory Destras; Gwendolyne Burfin; Hadrien Regue; Laurence Josset; Martine Valette; Quentin Semanas
EPI_ISL_1265792 to 1265794, EPI_ISL_1287740, EPI_ISL_1312797 to 1312799, EPI_ISL_1312877 to 1312879	CH ALBI	CNR Virus des Infections Respiratoires - France SUD	Antonin Bal; Bruno Lina; Gregory Destras; Gwendolyne Burfin; Hadrien Regue; Laurence Josset; Martine Valette; Quentin Semanas
EPI_ISL_1190816 to 1190835, EPI_ISL_1190841, EPI_ISL_1201873 to 1201874, EPI_ISL_1228964, EPI_ISL_1313004, EPI_ISL_1313007, EPI_ISL_1313123 to 1313127			
see above	CH ANTIBES - JUAN LES PINS	CNR Virus des Infections Respiratoires - France SUD	Antonin Bal; Bruno Lina; Gregory Destras; Gwendolyne Burfin; Hadrien Regue; Hadrien Règue; Laurence Josset; Martine Valette; Quentin Semanas
EPI_ISL_1312832 to 1312834	CH ARDECHE NORD	CNR Virus des Infections Respiratoires - France SUD	Antonin Bal; Bruno Lina; Gregory Destras; Gwendolyne Burfin; Hadrien Regue; Laurence Josset; Martine Valette; Quentin Semanas
EPI_ISL_1209401 to 1209402	CH ARMENTIERES	CHU Lille - Laboratoire de Virologie	AIT YAHYA Emilie; ALIDJINOU Enagnon Kazali; BOCKET Laurence; CREPIN Michel; DEMAY Christophe; ENGELMANN Ilka; GEFFROY Sandrine; GUIGON Aurélie; LAZREK Mouna; NOBILLIAUX Florian; PREVOST Brigitte; THUILLIER Caroline; TINEZ Claire
EPI_ISL_1209373	CH ARRAS	CHU Lille - Laboratoire de Virologie	AIT YAHYA Emilie; ALIDJINOU Enagnon Kazali; BOCKET Laurence; CREPIN Michel; DEMAY Christophe; ENGELMANN Ilka; GEFFROY Sandrine; GUIGON Aurélie; LAZREK Mouna; NOBILLIAUX Florian; PREVOST Brigitte; THUILLIER Caroline; TINEZ Claire
EPI_ISL_1085254, EPI_ISL_1085409	CH AVRANCHES	Department of Virology, Henri Mondor University Hospital, Assistance Publique Hôpitaux de Paris, Université Paris-Est Créteil, INSERM U955	Alexandre Soulier; Christophe Rodriguez; Elisabeth Trawinski; Guillaume Gricourt; Jean-Michel Pawlotsky; Melissa N'Debi; Slim Fourati; Vanessa Demontant
EPI_ISL_1265787 to 1265789, EPI_ISL_1312800 to 1312801	CH BASTIA	CNR Virus des Infections Respiratoires - France SUD	Antonin Bal; Bruno Lina; Gregory Destras; Gwendolyne Burfin; Hadrien Regue; Laurence Josset; Martine Valette; Quentin Semanas
EPI_ISL_1253557	CH BAYONNE	CNR Virus des Infections Respiratoires - France SUD	Antonin Bal; Bruno Lina; Gregory Destras; Gwendolyne Burfin; Hadrien Regue; Laurence Josset; Martine Valette; Quentin Semanas
EPI_ISL_1209370, EPI_ISL_1209390, EPI_ISL_1209403, EPI_ISL_1209405	CH BETHUNE	CHU Lille - Laboratoire de Virologie	AIT YAHYA Emilie; ALIDJINOU Enagnon Kazali; BOCKET Laurence; CREPIN Michel; DEMAY Christophe; ENGELMANN Ilka; GEFFROY Sandrine; GUIGON Aurélie; LAZREK Mouna; NOBILLIAUX Florian; PREVOST Brigitte; THUILLIER Caroline; TINEZ Claire
EPI_ISL_1165961 to 1165964	CH BOURG EN BRESSE	CNR Virus des Infections Respiratoires - France SUD	Antonin Bal; Bruno Lina; Gregory Destras; Gwendolyne Burfin; Hadrien Regue; Laurence Josset; Martine Valette; Quentin Semanas
EPI_ISL_1241960, EPI_ISL_1253566, EPI_ISL_1265476, EPI_ISL_1265509, EPI_ISL_1265570, EPI_ISL_1265727	CH BOURG-EN-BRESSE	CNR Virus des Infections Respiratoires - France SUD	Antonin Bal; Bruno Lina; Gregory Destras; Gwendolyne Burfin; Hadrien Regue; Laurence Josset; Martine Valette; Quentin Semanas
EPI_ISL_421455, EPI_ISL_421479 to 421480, EPI_ISL_421485 to 421486	CH Barreiro Montijo	Instituto Nacional de Saude (INSA)	Guiomar et al
EPI_ISL_1220322, EPI_ISL_1220324 to 1220326	CH CAHORS	CNR Virus des Infections Respiratoires - France SUD	Antonin Bal; Bruno Lina; Gregory Destras; Gwendolyne Burfin; Hadrien Regue; Laurence Josset; Martine Valette; Quentin Semanas
EPI_ISL_1209383	CH CALAIS	CHU Lille - Laboratoire de Virologie	AIT YAHYA Emilie; ALIDJINOU Enagnon Kazali; BOCKET Laurence; CREPIN Michel; DEMAY Christophe; ENGELMANN Ilka; GEFFROY Sandrine; GUIGON Aurélie; LAZREK Mouna; NOBILLIAUX Florian; PREVOST Brigitte; THUILLIER Caroline; TINEZ Claire
EPI_ISL_1228933	CH COTE BASQUE	CNR Virus des Infections Respiratoires - France SUD	Antonin Bal; Bruno Lina; Gregory Destras; Gwendolyne Burfin; Hadrien Regue; Laurence Josset; Martine Valette; Quentin Semanas
EPI_ISL_1228930 to 1228931	CH Cahors	CNR Virus des Infections Respiratoires - France SUD	Antonin Bal; Bruno Lina; Gregory Destras; Gwendolyne Burfin; Hadrien Regue; Laurence Josset; Martine Valette; Quentin Semanas
EPI_ISL_420041, EPI_ISL_420049 to 420050, EPI_ISL_420056 to 420057, EPI_ISL_421500, EPI_ISL_421509 to 421511, EPI_ISL_428353, EPI_ISL_428359 to 428360, EPI_ISL_443309, EPI_ISL_443316			
see above	CH Compiègne Laboratoire de Biologie	National Reference Center for Viruses of Respiratory Infections, Institut Pasteur, Paris	Angela Brisebarre; Etienne Simon-Lorière; Flora Donati; Marion Barbet; Maud Vanpeene; Mélanie Albert; Méline Bizard; Olivia Raulin; Raulin Olivia; Sylvie Behillil; Sylvie van der Werf; Vincent Enouf
EPI_ISL_1209371 to 1209372, EPI_ISL_1209396	CH DOUAI	CHU Lille - Laboratoire de Virologie	AIT YAHYA Emilie; ALIDJINOU Enagnon Kazali; BOCKET Laurence; CREPIN Michel; DEMAY Christophe; ENGELMANN Ilka; GEFFROY Sandrine; GUIGON Aurélie; LAZREK Mouna; NOBILLIAUX Florian; PREVOST Brigitte; THUILLIER Caroline; TINEZ Claire
EPI_ISL_860848	CH Dunkerque Laboratoire de Biologie	National Reference Center for Viruses of Respiratory Infections, Institut Pasteur, Paris	Angela Brisebarre; Camille Capel; Etienne Simon-Lorière; Joly Isabelle; Marion Barbet; Maud Vanpeene; Méline Bizard; Sylvie Behillil; Sylvie van der Werf; Vincent Enouf
EPI_ISL_1312941, EPI_ISL_1313595, EPI_ISL_1313599 to 1313600, EPI_ISL_1313639, EPI_ISL_1313691, EPI_ISL_1313915 to 1313916	CH GIVORS	CNR Virus des Infections Respiratoires - France SUD	Antonin Bal; Bruno Lina; Gregory Destras; Gwendolyne Burfin; Hadrien Regue; Laurence Josset; Martine Valette; Quentin Semanas
EPI_ISL_1297422	CH Givors	CNR Virus des Infections Respiratoires - France SUD	Antonin Bal; Bruno Lina; Gregory Destras; Gwendolyne Burfin; Hadrien Regue; Laurence Josset; Martine Valette; Quentin Semanas
EPI_ISL_416493, EPI_ISL_420044, EPI_ISL_420053, EPI_ISL_428350	CH Jean de Navarre Laboratoire de Biologie	National Reference Center for Viruses of Respiratory Infections, Institut Pasteur, Paris	Angela Brisebarre; Etienne Simon-Lorière; Flora Donati; Marion Barbet; Maud Vanpeene; Mélanie Albert; Méline Bizard; Mélnie Albert; Sylvie Behillil; Sylvie van der Werf; Vincent Enouf
EPI_ISL_428358, EPI_ISL_428366	CH Jeanne de Navarre Laboratoire de Biologie	National Reference Center for Viruses of Respiratory Infections, Institut Pasteur, Paris	Angela Brisebarre; Etienne Simon-Lorière; Flora Donati; Marion Barbet; Maud Vanpeene; Mélanie Albert; Méline Bizard; Sylvie Behillil; Sylvie van der Werf; Vincent Enouf
EPI_ISL_1209381, EPI_ISL_1209388	CH LENS	CHU Lille - Laboratoire de Virologie	AIT YAHYA Emilie; ALIDJINOU Enagnon Kazali; BOCKET Laurence; CREPIN Michel; DEMAY Christophe; ENGELMANN Ilka; GEFFROY Sandrine; GUIGON Aurélie; LAZREK Mouna; NOBILLIAUX Florian; PREVOST Brigitte; THUILLIER Caroline; TINEZ Claire
EPI_ISL_1085294, EPI_ISL_1085412	CH Louis Jaillon (Saint-Claude) Laboratoire	Department of Virology, Henri Mondor University Hospital, Assistance Publique Hôpitaux de Paris, Université Paris-Est Créteil, INSERM U955	Alexandre Soulier; Christophe Rodriguez; Elisabeth Trawinski; Guillaume Gricourt; Jean-Michel Pawlotsky; Melissa N'Debi; Slim Fourati; Vanessa Demontant
EPI_ISL_1165912, EPI_ISL_1165959, EPI_ISL_1166047 to 1166048	CH MACON	CNR Virus des Infections Respiratoires - France SUD	Antonin Bal; Bruno Lina; Gregory Destras; Gwendolyne Burfin; Hadrien Regue; Laurence Josset; Martine Valette; Quentin Semanas
EPI_ISL_1209391, EPI_ISL_1209404	CH MAUBEUGE	CHU Lille - Laboratoire de Virologie	AIT YAHYA Emilie; ALIDJINOU Enagnon Kazali; BOCKET Laurence; CREPIN Michel; DEMAY Christophe; ENGELMANN Ilka; GEFFROY Sandrine; GUIGON Aurélie; LAZREK Mouna; NOBILLIAUX Florian; PREVOST Brigitte; THUILLIER Caroline; TINEZ Claire
EPI_ISL_1190780, EPI_ISL_1229103 to 1229104	CH METROPOLE SAVOIE	CNR Virus des Infections Respiratoires - France SUD	Antonin Bal; Bruno Lina; Gregory Destras; Gwendolyne Burfin; Hadrien Regue; Hadrien Règue; Laurence Josset; Martine Valette; Quentin Semanas
EPI_ISL_1220330 to 1220333	CH MONTAUBAN	CNR Virus des Infections Respiratoires - France SUD	Antonin Bal; Bruno Lina; Gregory Destras; Gwendolyne Burfin; Hadrien Regue; Laurence Josset; Martine Valette; Quentin Semanas
EPI_ISL_1265879	CH MONTLUCON	CNR Virus des Infections Respiratoires - France SUD	Antonin Bal; Bruno Lina; Gregory Destras; Gwendolyne Burfin; Hadrien Regue; Laurence Josset; Martine Valette; Quentin Semanas

EPI_ISL_1085376	CH Mémorial F.E.U Laboratoire	Department of Virology, Henri Mondor University Hospital, Assistance Publique Hôpitaux de Paris, Université Paris-Est Créteil, INSERM U955	Alexandre Soulier; Christophe Rodriguez; Elisabeth Trawinski; Guillaume Gricourt; Jean-Michel Pawlotsky; Melissa N'Debi; Slim Fourati; Vanessa Demontant
EPI_ISL_1220343, EPI_ISL_1312804, EPI_ISL_1312835	CH NIORT	CNR Virus des Infections Respiratoires - France SUD	Antonin Bal; Bruno Lina; Gregory Destras; Gwendolyne Burfin; Hadrien Regue; Laurence Josset; Martine Valette; Quentin Semanas
EPI_ISL_1209382, EPI_ISL_1209397 to 1209398	CH ROUBAIX	CHU Lille - Laboratoire de Virologie	AIT YAHYA Emilie; ALIDJINOU Enagnon Kazali; BOCKET Laurence; CREPIN Michel; DEMAY Christophe; ENGELMANN Ilka; GEFFROY Sandrine; GUIGON Aurélie; LAZREK Mouna; NOBILLIAUX Florian; PREVOST Brigitte; THUILLIER Caroline; TINEZ Claire
EPI_ISL_1228975 to 1228976, EPI_ISL_1228980 to 1228981, EPI_ISL_1228983 to 1228984, EPI_ISL_1228988, EPI_ISL_1228990 to 1228991, EPI_ISL_1228993 to 1228996, EPI_ISL_1228999			
see above	CH ROYAN	CNR Virus des Infections Respiratoires - France SUD	Antonin Bal; Bruno Lina; Gregory Destras; Gwendolyne Burfin; Hadrien Regue; Laurence Josset; Martine Valette; Quentin Semanas
EPI_ISL_1209384, EPI_ISL_1209393	CH SAINT-OMER	CHU Lille - Laboratoire de Virologie	AIT YAHYA Emilie; ALIDJINOU Enagnon Kazali; BOCKET Laurence; CREPIN Michel; DEMAY Christophe; ENGELMANN Ilka; GEFFROY Sandrine; GUIGON Aurélie; LAZREK Mouna; NOBILLIAUX Florian; PREVOST Brigitte; THUILLIER Caroline; TINEZ Claire
EPI_ISL_1220334, EPI_ISL_1220337 to 1220340	CH SAINTONGE	CNR Virus des Infections Respiratoires - France SUD	Antonin Bal; Bruno Lina; Gregory Destras; Gwendolyne Burfin; Hadrien Regue; Laurence Josset; Martine Valette; Quentin Semanas
EPI_ISL_1209374, EPI_ISL_1209399	CH SECLIN	CHU Lille - Laboratoire de Virologie	AIT YAHYA Emilie; ALIDJINOU Enagnon Kazali; BOCKET Laurence; CREPIN Michel; DEMAY Christophe; ENGELMANN Ilka; GEFFROY Sandrine; GUIGON Aurélie; LAZREK Mouna; NOBILLIAUX Florian; PREVOST Brigitte; THUILLIER Caroline; TINEZ Claire
EPI_ISL_1163945, EPI_ISL_1163959, EPI_ISL_1163962, EPI_ISL_1163985	CH SUD GIRONDE	CNR Virus des Infections Respiratoires - France SUD	Antonin Bal; Bruno Lina; Gregory Destras; Gwendolyne Burfin; Hadrien Regue; Laurence Josset; Martine Valette; Quentin Semanas
EPI_ISL_1220336, EPI_ISL_1220344 to 1220347, EPI_ISL_1253556	CH TOULOUSE	CNR Virus des Infections Respiratoires - France SUD	Antonin Bal; Bruno Lina; Gregory Destras; Gwendolyne Burfin; Hadrien Regue; Laurence Josset; Martine Valette; Quentin Semanas
EPI_ISL_1166041 to 1166046, EPI_ISL_1220335, EPI_ISL_1220341 to 1220342	CH VALENCE	CNR Virus des Infections Respiratoires - France SUD	Antonin Bal; Bruno Lina; Gregory Destras; Gwendolyne Burfin; Hadrien Regue; Laurence Josset; Martine Valette; Quentin Semanas
EPI_ISL_1209375, EPI_ISL_1209389	CH VALENCIENNES	CHU Lille - Laboratoire de Virologie	AIT YAHYA Emilie; ALIDJINOU Enagnon Kazali; BOCKET Laurence; CREPIN Michel; DEMAY Christophe; ENGELMANN Ilka; GEFFROY Sandrine; GUIGON Aurélie; LAZREK Mouna; NOBILLIAUX Florian; PREVOST Brigitte; THUILLIER Caroline; TINEZ Claire
EPI_ISL_1265766, EPI_ISL_1265883, EPI_ISL_1312906	CH VICHY	CNR Virus des Infections Respiratoires - France SUD	Antonin Bal; Bruno Lina; Gregory Destras; Gwendolyne Burfin; Hadrien Regue; Laurence Josset; Martine Valette; Quentin Semanas
EPI_ISL_900534	CH VILLEFRANCHE	CNR Virus des Infections Respiratoires - France SUD	Antonin Bal; Bruno Lina; Gregory Destras; Gwendolyne Burfin; Hadrien Règue; Laurence Josset; Martine Valette; Quentin Semanas
EPI_ISL_421482 to 421484	CH VN Gaia - Espinho	Instituto Nacional de Saude (INSA)	Guiomar et al
EPI_ISL_792008 to 792015	CH de Bethune - Laboratoire de Biologie Médicale	National Reference Center for Viruses of Respiratory Infections, Institut Pasteur, Paris	Angela Brisebarre; Camille Capel; Etienne Simon-Lorière; Léa Pilorgé; Marion Barbet; Maud Vanpeene; Méline Bizard; Sylvie Behillil; Sylvie van der Werf; Vincent Enouf
EPI_ISL_1273842	CH de Lens	CHU Lille - Laboratoire de Virologie	AIT YAHYA Emilie; ALIDJINOU Enagnon Kazali; BOCKET Laurence; CREPIN Michel; DEMAY Christophe; ENGELMANN Ilka; GEFFROY Sandrine; GUIGON Aurélie; LAZREK Mouna; NOBILLIAUX Florian; PREVOST Brigitte; THUILLIER Caroline; TINEZ Claire
EPI_ISL_1013099 to 1013209, EPI_ISL_1013224 to 1013234, EPI_ISL_1013237 to 1013240, EPI_ISL_1013242 to 1013407	CH de Mayotte	National Reference Center for Viruses of Respiratory Infections, Institut Pasteur, Paris	Angela Brisebarre; Camille Capel; Combe Patrice; Etienne Simon-Lorière; Marion Barbet; Maud Vanpeene; Méline Bizard; Sylvie Behillil; Sylvie van der Werf; Vincent Enouf
EPI_ISL_894220 to 894222, EPI_ISL_894224 to 894231, EPI_ISL_909674, EPI_ISL_909687 to 909701, EPI_ISL_909708 to 909723, EPI_ISL_912402 to 912403	CH de Mayotte - Laboratoire de Biologie	National Reference Center for Viruses of Respiratory Infections, Institut Pasteur, Paris	Angela Brisebarre; Camille Capel; Combe Patrice; Etienne Simon-Lorière; Marion Barbet; Maud Vanpeene; Méline Bizard; Sylvie Behillil; Sylvie van der Werf; Vincent Enouf
EPI_ISL_1118928	CH de ROUBAIX	CHU Lille - Laboratoire de Virologie	AIT YAHYA Emilie; ALIDJINOU Enagnon Kazali; BOCKET Laurence; CREPIN Michel; DEMAY Christophe; ENGELMANN Ilka; GEFFROY Sandrine; GUIGON Aurélie; LAZREK Mouna; NOBILLIAUX Florian; PREVOST Brigitte; THUILLIER Caroline; TINEZ Claire
EPI_ISL_860900 to 860904, EPI_ISL_860910	CH du MANS - Lab. Bio. Moléculaire	National Reference Center for Viruses of Respiratory Infections, Institut Pasteur, Paris	Angela Brisebarre; Camille Capel; Etienne Simon-Lorière; Marion Barbet; Maud Vanpeene; Méline Bizard; Ramanantsoa CéLine; Sylvie Behillil; Sylvie van der Werf; Vincent Enouf
EPI_ISL_1085298	CH. ALENCON-MAMERS	Department of Virology, Henri Mondor University Hospital, Assistance Publique Hôpitaux de Paris, Université Paris-Est Créteil, INSERM U955	Alexandre Soulier; Christophe Rodriguez; Elisabeth Trawinski; Guillaume Gricourt; Jean-Michel Pawlotsky; Melissa N'Debi; Slim Fourati; Vanessa Demontant
EPI_ISL_1085380, EPI_ISL_1085418	CH. BEGIN	Department of Virology, Henri Mondor University Hospital, Assistance Publique Hôpitaux de Paris, Université Paris-Est Créteil, INSERM U955	Alexandre Soulier; Christophe Rodriguez; Elisabeth Trawinski; Guillaume Gricourt; Jean-Michel Pawlotsky; Melissa N'Debi; Slim Fourati; Vanessa Demontant
EPI_ISL_1085910, EPI_ISL_1085938 to 1085940, EPI_ISL_1110189	CH. CHU DE REIMS	Department of Virology, Henri Mondor University Hospital, Assistance Publique Hôpitaux de Paris, Université Paris-Est Créteil, INSERM U955	Alexandre Soulier; Christophe Rodriguez; Elisabeth Trawinski; Guillaume Gricourt; Jean-Michel Pawlotsky; Melissa N'Debi; Slim Fourati; Vanessa Demontant
EPI_ISL_1085260, EPI_ISL_1085379, EPI_ISL_1085410, EPI_ISL_1085415	CH. E. MULLER	Department of Virology, Henri Mondor University Hospital, Assistance Publique Hôpitaux de Paris, Université Paris-Est Créteil, INSERM U955	Alexandre Soulier; Christophe Rodriguez; Elisabeth Trawinski; Guillaume Gricourt; Jean-Michel Pawlotsky; Melissa N'Debi; Slim Fourati; Vanessa Demontant
EPI_ISL_1085406, EPI_ISL_1110193	CH. FLERS Jacques MONOD	Department of Virology, Henri Mondor University Hospital, Assistance Publique Hôpitaux de Paris, Université Paris-Est Créteil, INSERM U955	Alexandre Soulier; Christophe Rodriguez; Elisabeth Trawinski; Guillaume Gricourt; Jean-Michel Pawlotsky; Melissa N'Debi; Slim Fourati; Vanessa Demontant
EPI_ISL_1085259, EPI_ISL_1085320 to 1085321, EPI_ISL_1085330 to 1085331, EPI_ISL_1085373, EPI_ISL_1110206	CH. HOPITAL CIVIL	Department of Virology, Henri Mondor University Hospital, Assistance Publique Hôpitaux de Paris, Université Paris-Est Créteil, INSERM U955	Alexandre Soulier; Christophe Rodriguez; Elisabeth Trawinski; Guillaume Gricourt; Jean-Michel Pawlotsky; Melissa N'Debi; Slim Fourati; Vanessa Demontant
EPI_ISL_1085374, EPI_ISL_1085378	CH. ROBERT BISSON	Department of Virology, Henri Mondor University Hospital, Assistance Publique Hôpitaux de Paris, Université Paris-Est Créteil, INSERM U955	Alexandre Soulier; Christophe Rodriguez; Elisabeth Trawinski; Guillaume Gricourt; Jean-Michel Pawlotsky; Melissa N'Debi; Slim Fourati; Vanessa Demontant
EPI_ISL_1085424	CH. ST DENIS	Department of Virology, Henri Mondor University Hospital, Assistance Publique Hôpitaux de Paris, Université Paris-Est Créteil, INSERM U955	Alexandre Soulier; Christophe Rodriguez; Elisabeth Trawinski; Guillaume Gricourt; Jean-Michel Pawlotsky; Melissa N'Debi; Slim Fourati; Vanessa Demontant
EPI_ISL_912681, EPI_ISL_912687, EPI_ISL_912781 to 912785, EPI_ISL_912891, EPI_ISL_912907, EPI_ISL_912961, EPI_ISL_981392, EPI_ISL_981408, EPI_ISL_981462 to 981465, EPI_ISL_981486, EPI_ISL_981488, EPI_ISL_981538 to 981546, EPI_ISL_982118, EPI_ISL_982147, EPI_ISL_982181, EPI_ISL_982226 to 982227, EPI_ISL_982303, EPI_ISL_982305 to 982306, EPI_ISL_982312 to 982320, EPI_ISL_1085232, EPI_ISL_1085235, EPI_ISL_1085245, EPI_ISL_1085251, EPI_ISL_1085253, EPI_ISL_1085261 to 1085262, EPI_ISL_1085265 to 1085266, EPI_ISL_1085286, EPI_ISL_1085295, EPI_ISL_1085316, EPI_ISL_1085344, EPI_ISL_1085358, EPI_ISL_1085363 to 1085369, EPI_ISL_1085377, EPI_ISL_1085383, EPI_ISL_1085392 to 1085395, EPI_ISL_1085443, EPI_ISL_1085461, EPI_ISL_1085509, EPI_ISL_1085512, EPI_ISL_1085529, EPI_ISL_1085599, EPI_ISL_1085609, EPI_ISL_1085614, EPI_ISL_1085629, EPI_ISL_1085642 to 1085643,			

EPI_ISL_1085645, EPI_ISL_1085652, EPI_ISL_1085768 to 1085769, EPI_ISL_1085771, EPI_ISL_1085790, EPI_ISL_1085794, EPI_ISL_1085799, EPI_ISL_1085803 to 1085806, EPI_ISL_1085816, EPI_ISL_1085818, EPI_ISL_1085822, EPI_ISL_1085826, EPI_ISL_1085828 to 1085830, EPI_ISL_1085834, EPI_ISL_1085839, EPI_ISL_1085842, EPI_ISL_1085857, EPI_ISL_1085869 to 1085872, EPI_ISL_1085876, EPI_ISL_1085887, EPI_ISL_1085897, EPI_ISL_1085899, EPI_ISL_1085906, EPI_ISL_1085911 to 1085915, EPI_ISL_1088551, EPI_ISL_1088557, EPI_ISL_1088576, EPI_ISL_1088579, EPI_ISL_1110180, EPI_ISL_1110199, EPI_ISL_1110201 to 1110202, EPI_ISL_1110205, EPI_ISL_1110208 to 1110209			
see above	CH.INTERCOMMUNAL DE CRETEIL	Department of Virology, Henri Mondor University Hospital, Assistance Publique Hôpitaux de Paris, Université Paris-Est Créteil, INSERM U955	Alexandre Soulier; Christophe Rodriguez; Elisabeth Trawinski; Guillaume Gricourt; Jean-Michel Pawlotsky; Melissa N'Debi; Slim Fourati; Vanessa Demontant
EPI_ISL_1085838, EPI_ISL_1085883, EPI_ISL_1085932	CH.de LONS LE SAUNIER	Department of Virology, Henri Mondor University Hospital, Assistance Publique Hôpitaux de Paris, Université Paris-Est Créteil, INSERM U955	Alexandre Soulier; Christophe Rodriguez; Elisabeth Trawinski; Guillaume Gricourt; Jean-Michel Pawlotsky; Melissa N'Debi; Slim Fourati; Vanessa Demontant
EPI_ISL_733569	CHAI WAN FAMILIES CLINIC	Hong Kong Department of Health	Alan K.L. Tsang; Dominic N.C. Tsang; Edman T.K. Lam; Peter C.W. Yip; Rickjason C.W. Chan
EPI_ISL_418006	CHBarreiro Montijo	Instituto Nacional de Saude (INSA)	Guiomar et al
EPI_ISL_872051, EPI_ISL_872110 to 872111, EPI_ISL_872146 to 872147, EPI_ISL_930580 to 930584, EPI_ISL_930604, EPI_ISL_930623	CHC	GIGA Medical Genomics	Bouchra Boujemla; Cécile Meex; Keith Durkin; Maria Artesi; Marie-Pierre Hayette; Pierrette Melin; Raphaël Boreux; Sébastien Bontems; Vincent Bours
EPI_ISL_959551 to 959552	CHC Liège	GIGA Medical Genomics	Bouchra Boujemla; Cécile Meex; Keith Durkin; Maria Artesi; Marie-Pierre Hayette; Pierrette Melin; Raphaël Boreux; Sébastien Bontems; Vincent Bours
EPI_ISL_1009673	CHI Andre Gregoire	Cerba	Benadza M; Fremont-Goudot G; Haïm-Boukobza S; Lecorche E; Olivi M; Penot P; Roquebert B; Trombert-Paolantoni S
EPI_ISL_1250609, EPI_ISL_1250635	CHI Elbeuf	Centre Hospitalier Universitaire de Rouen Laboratoire de Virologie	Alice Moisan; Fabienne De Oliveira; Marie Leoz
EPI_ISL_1265817, EPI_ISL_1265896 to 1265898, EPI_ISL_1287748	CHI TOULON LA SEYNE	CNR Virus des Infections Respiratoires - France SUD	Antonin Bal; Bruno Lina; Gregory Destras; Gwendolyne Burfin; Hadrien Regue; Laurence Josset; Martine Valette; Quentin Semanas
EPI_ISL_981460 to 981461, EPI_ISL_981466 to 981481, EPI_ISL_981483, EPI_ISL_982158, EPI_ISL_982179, EPI_ISL_1085241, EPI_ISL_1085337, EPI_ISL_1085342, EPI_ISL_1085414, EPI_ISL_1085420, EPI_ISL_1085428, EPI_ISL_1085441, EPI_ISL_1085472, EPI_ISL_1085476, EPI_ISL_1085505, EPI_ISL_1085511, EPI_ISL_1085525, EPI_ISL_1085535 to 1085539, EPI_ISL_1085546 to 1085547, EPI_ISL_1085561, EPI_ISL_1085563 to 1085564, EPI_ISL_1085570, EPI_ISL_1085573, EPI_ISL_1085581 to 1085582, EPI_ISL_1085584, EPI_ISL_1085596 to 1085598, EPI_ISL_1085601, EPI_ISL_1085638, EPI_ISL_1085644, EPI_ISL_1085783, EPI_ISL_1085791, EPI_ISL_1085793, EPI_ISL_1085797 to 1085798, EPI_ISL_1085807, EPI_ISL_1085879 to 1085880, EPI_ISL_1085896, EPI_ISL_1085902, EPI_ISL_1085904, EPI_ISL_1088572 to 1088574, EPI_ISL_1110138, EPI_ISL_1110155			
see above	CHI VILLENEUVE ST GEORGES	Department of Virology, Henri Mondor University Hospital, Assistance Publique Hôpitaux de Paris, Université Paris-Est Créteil, INSERM U955	Alexandre Soulier; Christophe Rodriguez; Elisabeth Trawinski; Guillaume Gricourt; Jean-Michel Pawlotsky; Melissa N'Debi; Slim Fourati; Vanessa Demontant
EPI_ISL_1190836	CHIAP LABORATOIRE MULTISITES	CNR Virus des Infections Respiratoires - France SUD	Antonin Bal; Bruno Lina; Gregory Destras; Gwendolyne Burfin; Hadrien Règue; Laurence Josset; Martine Valette; Quentin Semanas
EPI_ISL_1265735, EPI_ISL_1265799, EPI_ISL_1265862 to 1265863	CHMS CHAMBERY	CNR Virus des Infections Respiratoires - France SUD	Antonin Bal; Bruno Lina; Gregory Destras; Gwendolyne Burfin; Hadrien Regue; Laurence Josset; Martine Valette; Quentin Semanas
EPI_ISL_418017	CHMT	Instituto Nacional de Saude (INSA)	Guiomar et al
EPI_ISL_872052 to 872056, EPI_ISL_872117 to 872121, EPI_ISL_872123 to 872126, EPI_ISL_872134, EPI_ISL_872150 to 872152	CHR Citadelle	GIGA Medical Genomics	Bouchra Boujemla; Cécile Meex; Keith Durkin; Maria Artesi; Marie-Pierre Hayette; Pierrette Melin; Raphaël Boreux; Sébastien Bontems; Vincent Bours
EPI_ISL_1190785	CHR LA REUNION FELIX GUYON	CNR Virus des Infections Respiratoires - France SUD	Antonin Bal; Bruno Lina; Gregory Destras; Gwendolyne Burfin; Hadrien Règue; Laurence Josset; Martine Valette; Quentin Semanas
EPI_ISL_1085255, EPI_ISL_1085370, EPI_ISL_1085918 to 1085919, EPI_ISL_1085950	CHR METZ THIONVILLE Hôpital de Mercy	Department of Virology, Henri Mondor University Hospital, Assistance Publique Hôpitaux de Paris, Université Paris-Est Créteil, INSERM U955	Alexandre Soulier; Christophe Rodriguez; Elisabeth Trawinski; Guillaume Gricourt; Jean-Michel Pawlotsky; Melissa N'Debi; Slim Fourati; Vanessa Demontant
EPI_ISL_959553	CHR Namur	GIGA Medical Genomics	Bouchra Boujemla; Cécile Meex; Keith Durkin; Maria Artesi; Marie-Pierre Hayette; Pierrette Melin; Raphaël Boreux; Sébastien Bontems; Vincent Bours
EPI_ISL_1058457, EPI_ISL_1058459 to 1058461, EPI_ISL_1058534 to 1058557, EPI_ISL_1058604 to 1058614, EPI_ISL_1059070, EPI_ISL_1076397 to 1076398, EPI_ISL_1076409, EPI_ISL_1076446, EPI_ISL_1113084 to 1113110, EPI_ISL_1171512, EPI_ISL_1241688			
see above	CHR de La Citadelle	GIGA Medical Genomics	Bouchra Boujemla; Cécile Meex; Keith Durkin; Maria Artesi; Marie-Pierre Hayette; Nathalie Renotte; Pierrette Melin; Raphaël Boreux; Sébastien Bontems; Vincent Bours
EPI_ISL_935674 to 935695, EPI_ISL_959600 to 959602, EPI_ISL_965414 to 965415, EPI_ISL_965421 to 965430, EPI_ISL_965432, EPI_ISL_965437 to 965450	CHR de la Citadelle	GIGA Medical Genomics	Bouchra Boujemla; Cécile Meex; Keith Durkin; Maria Artesi; Marie-Pierre Hayette; Pierrette Melin; Raphaël Boreux; Sébastien Bontems; Vincent Bours
EPI_ISL_418222	CHRU Bretonneau - Serv. Bacterio-Virol.	National Reference Center for Viruses of Respiratory Infections, Institut Pasteur, Paris	Angela Brisebarre; Etienne Simon-Lorière; Fabiana Gambaro; Flora Donati; Julien Marlet; Marion Barbet; Maud Vanpeene; Mélanie Albert; Méline Bizard; Sylvie Behillil; Sylvie van der Werf; Vincent Enouf
EPI_ISL_1085263, EPI_ISL_1085299 to 1085300, EPI_ISL_1085361, EPI_ISL_1085407	CHRU HOPITAUX de BRABOIS	Department of Virology, Henri Mondor University Hospital, Assistance Publique Hôpitaux de Paris, Université Paris-Est Créteil, INSERM U955	Alexandre Soulier; Christophe Rodriguez; Elisabeth Trawinski; Guillaume Gricourt; Jean-Michel Pawlotsky; Melissa N'Debi; Slim Fourati; Vanessa Demontant
EPI_ISL_416502 to 416513, EPI_ISL_443289 to 443294, EPI_ISL_734167 to 734169	CHRU Pontchaillou - Laboratoire de Virologie	National Reference Center for Viruses of Respiratory Infections, Institut Pasteur, Paris	Angela Brisebarre; Camille Capel; Etienne Simon-Lorière; Flora Donati; Gisèle Lagathu; Marion Barbet; Maud Vanpeene; Mélanie Albert; Méline Bizard; Mélnie Albert; Sylvie Behillil; Sylvie van der Werf; Vincent Enouf
EPI_ISL_613545 to 613559, EPI_ISL_614281	CHRU Pontchaillou - Laboratoire de Virologie 2, rue Henri Le Guilloux	National Reference Center for Viruses of Respiratory Infections, Institut Pasteur, Paris	Angela Brisebarre; Camille Capel; Etienne Simon-Lorière; Gisèle Lagathu; Marion Barbet; Maud Vanpeene; Méline Bizard; Sylvie Behillil; Sylvie van der Werf; Vincent Enouf
EPI_ISL_418027, EPI_ISL_421453, EPI_ISL_421464 to 421465	CHTMAD	Instituto Nacional de Saude (INSA)	Guiomar et al
EPI_ISL_791991 to 791999, EPI_ISL_1013408 to 1013414	CHU - Hôpital Cavale Blanche	National Reference Center for Viruses of Respiratory Infections, Institut Pasteur, Paris	Angela Brisebarre; Camille Capel; Etienne Simon-Lorière; Léa Pilorgé; Marion Barbet; Maud Vanpeene; Méline Bizard; Pilorge LéA; Sylvie Behillil; Sylvie van der Werf; Vincent Enouf
EPI_ISL_418219, EPI_ISL_443265 to 443283, EPI_ISL_754853 to 754856	CHU - Hôpital Cavale Blanche - Labo. de Virologie	National Reference Center for Viruses of Respiratory Infections, Institut Pasteur, Paris	Angela Brisebarre; Camille Capel; Etienne Simon-Lorière; Fabiana Gambaro; Flora Donati; Léa Pilorge; Marion Barbet; Maud Vanpeene; Mélanie Albert; Méline Bizard; Pilorge léa; Sylvie Behillil; Sylvie van der Werf; Vincent Enouf
EPI_ISL_754842	CHU Amiens - Labo virologie	National Reference Center for Viruses of Respiratory Infections, Institut Pasteur, Paris	Angela Brisebarre; Camille Capel; Castelain Sandrine; Etienne Simon-Lorière; François Catherine; Marion Barbet; Maud Vanpeene; Méline Bizard; Sylvie Behillil; Sylvie van der Werf; Vincent Enouf
EPI_ISL_860905 to 860906, EPI_ISL_860911 to 860912, EPI_ISL_861236	CHU Angers - Dpt des Agents Infectieux	National Reference Center for Viruses of Respiratory Infections, Institut Pasteur, Paris	Angela Brisebarre; Camille Capel; Ducancelle Alexandra; Etienne Simon-Lorière; Marion Barbet; Maud Vanpeene; Méline Bizard; Sylvie Behillil; Sylvie van der Werf; Vincent Enouf
EPI_ISL_645169 to 645172, EPI_ISL_649954 to 649971, EPI_ISL_660372, EPI_ISL_700326, EPI_ISL_768823, EPI_ISL_779786, EPI_ISL_779837 to 779844			
see above	CHU Bordeaux	CNR Virus des Infections Respiratoires - France SUD	Antonin Bal; Bruno Lina; Camille Ciccone; Gregory Destras; Gwendolyne Burfin; Hadrien Règue; Isabelle Garrigue; Laurence Josset; Marie-Edith Lafon; Martine Valette; Pantxika Bellecave; Pascale Trimoulet; Quentin Semanas
EPI_ISL_1190778 to 1190779, EPI_ISL_1265777, EPI_ISL_1265830, EPI_ISL_1265866 to 1265867, EPI_ISL_1312880, EPI_ISL_1312883 to 1312885, EPI_ISL_1312900 to 1312902, EPI_ISL_1312905			
see above	CHU CLERMONT FERRAND	CNR Virus des Infections Respiratoires - France SUD	Antonin Bal; Bruno Lina; Gregory Destras; Gwendolyne Burfin; Hadrien Regue; Hadrien Règue; Laurence Josset; Martine Valette; Quentin Semanas
EPI_ISL_641546 to 641550, EPI_ISL_645188 to	CHU Clermont-Ferrand	CNR Virus des Infections Respiratoires - France SUD	Amélie Brebion; Antonin Bal; Audrey Mirand; Bruno Lina; Christel Regagnon; Christine Archimbaud; Cécile Henquell; Gregory Destras; Gwendolyne

645196, EPI_ISL_660353 to 660371, EPI_ISL_745312 to 745315, EPI_ISL_745335 to 745336, EPI_ISL_900528			Burfin; Hadrien Règue; Hélène Chabrolles; Laurence Josset; Martine Chambon; Martine Valette; Maxime Bisseux; Patricia Combes; Quentin Semanas
EPI_ISL_418002	CHU Coimbra	Instituto Nacional de Saude (INSA)	Guiomar et al
EPI_ISL_418005	CHU Coimbra - Pediátrico	Instituto Nacional de Saude (INSA)	Guiomar et al
EPI_ISL_1163943, EPI_ISL_1163948, EPI_ISL_1163953, EPI_ISL_1163966, EPI_ISL_1181959 to 1181966, EPI_ISL_1190777, EPI_ISL_1228938 to 1228942, EPI_ISL_1228945, EPI_ISL_1228947, EPI_ISL_1228949, EPI_ISL_1228952			
see above	CHU GRENOBLE	CNR Virus des Infections Respiratoires - France SUD	Antonin Bal; Bruno Lina; Gregory Destras; Gwendolyne Burfin; Hadrien Regue; Hadrien Règue; Laurence Josset; Martine Valette; Quentin Semanas
EPI_ISL_416751 to 416752	CHU Gabriel Montpied	CNR Virus des Infections Respiratoires - France SUD	Alexandre; Antonin; Bal; Bouscambert-Duchamp; Brengel-Pesce; Bruno.; Cheynet; Destras; Florence; Gaymard; Gregory; Josset; Karen; Laurence; Lina; Martine; Maude; Morfin-Sherpa; Valette; Valérie
EPI_ISL_1209377, EPI_ISL_1209386 to 1209387, EPI_ISL_1209392, EPI_ISL_1209395, EPI_ISL_1209400	CHU LILLE	CHU Lille - Laboratoire de Virologie	AIT YAHYA Emilie; ALIDJINOU Enagnon Kazali; BOCKET Laurence; CREPIN Michel; DEMAY Christophe; ENGELMANN Ilka; GEFFROY Sandrine; GUIGON Aurélie; LAZREK Mouna; NOBILLIAUX Florian; PREVOST Brigitte; THUILLIER Caroline; TINEZ Claire
EPI_ISL_1229064 to 1229067, EPI_ISL_1265768, EPI_ISL_1265770, EPI_ISL_1265888 to 1265894	CHU LIMOGES	CNR Virus des Infections Respiratoires - France SUD	Antonin Bal; Bruno Lina; Gregory Destras; Gwendolyne Burfin; Hadrien Regue; Laurence Josset; Martine Valette; Quentin Semanas
EPI_ISL_1229004 to 1229005, EPI_ISL_1229010, EPI_ISL_1229015	CHU MONTAUBAN	CNR Virus des Infections Respiratoires - France SUD	Antonin Bal; Bruno Lina; Gregory Destras; Gwendolyne Burfin; Hadrien Regue; Laurence Josset; Martine Valette; Quentin Semanas
EPI_ISL_641556, EPI_ISL_644681 to 644696, EPI_ISL_660672 to 660709	CHU Montpellier	CNR Virus des Infections Respiratoires - France SUD	Antonin Bal; Bruno Lina; Gregory Destras; Gwendolyne Burfin; Hadrien Règue; Laurence Josset; Martine Valette; Michel Segondy; Quentin Semanas; Vincent Foulongne
EPI_ISL_1312949, EPI_ISL_1312979	CHU NIMES	CNR Virus des Infections Respiratoires - France SUD	Antonin Bal; Bruno Lina; Gregory Destras; Gwendolyne Burfin; Hadrien Regue; Laurence Josset; Martine Valette; Quentin Semanas
EPI_ISL_645217 to 645218, EPI_ISL_649173 to 649187, EPI_ISL_663249 to 663284, EPI_ISL_666721 to 666724, EPI_ISL_700371	CHU Nantes	CNR Virus des Infections Respiratoires - France SUD	Antonin Bal; Bruno Lina; Celine Bressollette; Gregory Destras; Gwendolyne Burfin; Hadrien Règue; Laurence Josset; Louise Castain; Martine Valette; Quentin Semanas; Virginie Ferré
EPI_ISL_644697 to 644702, EPI_ISL_645197 to 645209, EPI_ISL_645215, EPI_ISL_660710 to 660731, EPI_ISL_663243 to 663248	CHU Nîmes	CNR Virus des Infections Respiratoires - France SUD	Antonin Bal; Bruno Lina; Gregory Destras; Gwendolyne Burfin; Hadrien Règue; Jean-Philippe Lavigne; Laurence Josset; Marie-Josée Carles; Martine Valette; Maxence Lotellier; Quentin Semanas; Stephan Robin
EPI_ISL_645216, EPI_ISL_663227 to 663242, EPI_ISL_707781 to 707782, EPI_ISL_768822, EPI_ISL_779785, EPI_ISL_900531	CHU Poitiers	CNR Virus des Infections Respiratoires - France SUD	Agnès Beby-Defaux; Antonin Bal; Bruno Lina; Clément Jousselin; Gregory Destras; Gwendolyne Burfin; Hadrien Règue; Laurence Josset; Magali Garcia; Martine Valette; Nicolas Lévêque; Quentin Semanas
EPI_ISL_591099, EPI_ISL_591541 to 591547, EPI_ISL_593855 to 593901, EPI_ISL_603216 to 603220, EPI_ISL_671941 to 671972, EPI_ISL_671975 to 671977, EPI_ISL_751448 to 751499, EPI_ISL_754136 to 754139, EPI_ISL_804370 to 804378, EPI_ISL_833457 to 833480, EPI_ISL_848063 to 848068, EPI_ISL_852814 to 852818, EPI_ISL_864581 to 864593, EPI_ISL_875666 to 875673, EPI_ISL_913102 to 913115, EPI_ISL_913592 to 913593			
see above	CHU Purpan - Laboratoire de Virologie - Institut Fédératif de Biologie	CHU Purpan - Laboratoire de Virologie - Institut Fédératif de Biologie	Boyer P.; Carcenac R.; Dubois M.; Harter A.; Izopet J.; Latour J.; Ranger N.; Tremeaux P.
EPI_ISL_424993, EPI_ISL_434616 to 434635, EPI_ISL_482879 to 482889	CHU Purpan - Laboratoire de Virologie - Institut Fédératif de Biologie	Laboratoire de virologie - École Nationale Vétérinaire de Toulouse	Croville, G.; Guerin; Guillaume Croville; J.-L. and Izopet, J.; Jacques Izopet; Jean-Luc Guérin
EPI_ISL_1265825 to 1265826, EPI_ISL_1265834, EPI_ISL_1265839 to 1265841, EPI_ISL_1265851 to 1265852	CHU REUNION	CNR Virus des Infections Respiratoires - France SUD	Antonin Bal; Bruno Lina; Gregory Destras; Gwendolyne Burfin; Hadrien Regue; Laurence Josset; Martine Valette; Quentin Semanas
EPI_ISL_1241885 to 1241890, EPI_ISL_1290836	CHU Rennes	CNR Virus des Infections Respiratoires - France SUD	Antonin Bal; Bruno Lina; Gregory Destras; Gwendolyne Burfin; Hadrien Regue; Laurence Josset; Martine Valette; Quentin Semanas
EPI_ISL_1228910 to 1228924	CHU SAINT-ETIENNE	CNR Virus des Infections Respiratoires - France SUD	Antonin Bal; Bruno Lina; Gregory Destras; Gwendolyne Burfin; Hadrien Regue; Laurence Josset; Martine Valette; Quentin Semanas
EPI_ISL_1163931, EPI_ISL_1191701, EPI_ISL_1191711 to 1191712	CHU ST ETIENNE	CNR Virus des Infections Respiratoires - France SUD	Antonin Bal; Bruno Lina; Gregory Destras; Gwendolyne Burfin; Hadrien Regue; Laurence Josset; Martine Valette; Quentin Semanas
EPI_ISL_1313709	CHU ST ETIENNE HOPITAL NORD	CNR Virus des Infections Respiratoires - France SUD	Antonin Bal; Bruno Lina; Gregory Destras; Gwendolyne Burfin; Hadrien Regue; Laurence Josset; Martine Valette; Quentin Semanas
EPI_ISL_1265713	CHU STRASBOURG	CNR Virus des Infections Respiratoires - France SUD	Antonin Bal; Bruno Lina; Gregory Destras; Gwendolyne Burfin; Hadrien Regue; Laurence Josset; Martine Valette; Quentin Semanas
EPI_ISL_982128, EPI_ISL_982142 to 982144, EPI_ISL_982146, EPI_ISL_982154, EPI_ISL_982165, EPI_ISL_982174 to 982176	CHU SUD AMIENS	Department of Virology, Henri Mondor University Hospital, Assistance Publique Hôpitaux de Paris, Université Paris-Est Créteil, INSERM U955	Alexandre Soulier; Christophe Rodriguez; Elisabeth Trawinski; Guillaume Gricourt; Jean-Michel Pawlotsky; Melissa N'Debi; Slim Fourati; Vanessa Demontant
EPI_ISL_1239444	CHU Saint-Étienne	CNR Virus des Infections Respiratoires - France SUD	Antonin Bal; Bruno Lina; Gregory Destras; Gwendolyne Burfin; Hadrien Regue; Laurence Josset; Martine Valette; Quentin Semanas
EPI_ISL_535732, EPI_ISL_535801	CHU Sainte-Justine	Laboratoire de santé publique du Québec	Guillaume Bourque; Ioannis Ragoussis; Jesse Shapiro; Mark Lathrop and Michel Roger; Mark Lathrop and Michel Roger on behalf of the CoVSeQ research group; Sandrine Moreira
EPI_ISL_1312803, EPI_ISL_1312842, EPI_ISL_1312844, EPI_ISL_1312846 to 1312847, EPI_ISL_1312849, EPI_ISL_1312852 to 1312853, EPI_ISL_1312855, EPI_ISL_1312857 to 1312858, EPI_ISL_1312861			
see above	CHU TOULOUSE	CNR Virus des Infections Respiratoires - France SUD	Antonin Bal; Bruno Lina; Gregory Destras; Gwendolyne Burfin; Hadrien Regue; Laurence Josset; Martine Valette; Quentin Semanas
EPI_ISL_641551 to 641555, EPI_ISL_660373 to 660377, EPI_ISL_660665 to 660671	CHU Toulouse	CNR Virus des Infections Respiratoires - France SUD	Antonin Bal; Bruno Lina; Gregory Destras; Gwendolyne Burfin; Hadrien Règue; Jean Michel Mansuy; Laurence Josset; Martine Valette; Quentin Semanas
EPI_ISL_735391	CHU Tours	CNR Virus des Infections Respiratoires - France SUD	Antonin Bal; Bruno Lina; Catherine Gaudy-Graffin; Claudia Gonzalez; Florence Morfin; Gregory Destras; Gwendolyne Burfin; Hadrien Regue; Julien Marlet; Karl Stefic; Laurence Josset; Martine Valette; Maude Bouscambert; Quentin Semanas; Thibault Guinoiseau; Yahia Mekki
EPI_ISL_860820, EPI_ISL_860822 to 860823, EPI_ISL_860825, EPI_ISL_860827, EPI_ISL_860832, EPI_ISL_860867, EPI_ISL_860891 to 860897, EPI_ISL_860907 to 860909, EPI_ISL_861235, EPI_ISL_861238			
see above	CHU Tours - Virologie	National Reference Center for Viruses of Respiratory Infections, Institut Pasteur, Paris	Angela Brisebarre; Camille Capel; Etienne Simon-Lorière; Gaudy Graffin Catherine; Marion Barbet; Maud Vanpeene; Méline Bizard; Sylvie Behillil; Sylvie van der Werf; Vincent Enouf
EPI_ISL_443261 to 443264	CHU de Dijon - Laboratoire de Virologie	National Reference Center for Viruses of Respiratory Infections, Institut Pasteur, Paris	Angela Brisebarre; Etienne Simon-Lorière; Flora Donati; Jean-Baptiste Bour; Marion Barbet; Maud Vanpeene; Mélanie Albert; Méline Bizard; Sylvie Behillil; Sylvie van der Werf; Vincent Enouf
EPI_ISL_644673 to 644680, EPI_ISL_645174 to 645183, EPI_ISL_663206 to 663226, EPI_ISL_666711 to 666720	CHU de Limoges	CNR Virus des Infections Respiratoires - France SUD	Antonin Bal; Bruno Lina; Gregory Destras; Gwendolyne Burfin; Hadrien Règue; Laurence Josset; Martine Valette; Quentin Semanas; Sylvie Rogez
EPI_ISL_860821, EPI_ISL_860828, EPI_ISL_860863, EPI_ISL_860865, EPI_ISL_860868, EPI_ISL_860890, EPI_ISL_860913 to 860930			
see above	CHU de Nantes - Hôtel Dieu - Labo. Virologie	National Reference Center for Viruses of Respiratory Infections, Institut Pasteur, Paris	Andre-Garnier Elisabeth; Angela Brisebarre; Camille Capel; Etienne Simon-Lorière; Marion Barbet; Maud Vanpeene; Méline Bizard; Sylvie Behillil; Sylvie van der Werf; Vincent Enouf
EPI_ISL_909702	CHU de Nantes - Hôtel Dieu. Laboratoire de Virologie	National Reference Center for Viruses of Respiratory Infections, Institut Pasteur, Paris	Angela Brisebarre; Bressollette CéLine; Camille Capel; Etienne Simon-Lorière; Marion Barbet; Maud Vanpeene; Méline Bizard; Sylvie Behillil; Sylvie van der Werf; Vincent Enouf

EPI_ISL_645173	CHU de Nice	CNR Virus des Infections Respiratoires - France SUD	Antonin Bal; Bruno Lina; Gregory Destras; Gwendolyne Burfin; Géraldine Gonfrier; Hadrien Règue; Laurence Josset; Martine Valette; Quentin Semanas; Valérie Giordanengo
EPI_ISL_641528	CHU de Nice - Hôpital Archet 10	CNR Virus des Infections Respiratoires - France SUD	Antonin Bal; Bruno Lina; Gregory Destras; Gwendolyne Burfin; Géraldine Gonfrier; Hadrien Règue; Laurence Josset; Martine Valette; Quentin Semanas; Valérie Giordanengo
EPI_ISL_641529	CHU de Nice - Hôpital Archet 11	CNR Virus des Infections Respiratoires - France SUD	Antonin Bal; Bruno Lina; Gregory Destras; Gwendolyne Burfin; Géraldine Gonfrier; Hadrien Règue; Laurence Josset; Martine Valette; Quentin Semanas; Valérie Giordanengo
EPI_ISL_641530	CHU de Nice - Hôpital Archet 12	CNR Virus des Infections Respiratoires - France SUD	Antonin Bal; Bruno Lina; Gregory Destras; Gwendolyne Burfin; Géraldine Gonfrier; Hadrien Règue; Laurence Josset; Martine Valette; Quentin Semanas; Valérie Giordanengo
EPI_ISL_641531	CHU de Nice - Hôpital Archet 13	CNR Virus des Infections Respiratoires - France SUD	Antonin Bal; Bruno Lina; Gregory Destras; Gwendolyne Burfin; Géraldine Gonfrier; Hadrien Règue; Laurence Josset; Martine Valette; Quentin Semanas; Valérie Giordanengo
EPI_ISL_641532	CHU de Nice - Hôpital Archet 14	CNR Virus des Infections Respiratoires - France SUD	Antonin Bal; Bruno Lina; Gregory Destras; Gwendolyne Burfin; Géraldine Gonfrier; Hadrien Règue; Laurence Josset; Martine Valette; Quentin Semanas; Valérie Giordanengo
EPI_ISL_641533	CHU de Nice - Hôpital Archet 15	CNR Virus des Infections Respiratoires - France SUD	Antonin Bal; Bruno Lina; Gregory Destras; Gwendolyne Burfin; Géraldine Gonfrier; Hadrien Règue; Laurence Josset; Martine Valette; Quentin Semanas; Valérie Giordanengo
EPI_ISL_641534	CHU de Nice - Hôpital Archet 16	CNR Virus des Infections Respiratoires - France SUD	Antonin Bal; Bruno Lina; Gregory Destras; Gwendolyne Burfin; Géraldine Gonfrier; Hadrien Règue; Laurence Josset; Martine Valette; Quentin Semanas; Valérie Giordanengo
EPI_ISL_641520, EPI_ISL_693387 to 693390, EPI_ISL_779846	CHU de Nice - Hôpital Archet 2	CNR Virus des Infections Respiratoires - France SUD	Antonin Bal; Bruno Lina; Gregory Destras; Gwendolyne Burfin; Géraldine Gonfrier; Hadrien Règue; Laurence Josset; Martine Valette; Quentin Semanas; Valérie Giordanengo
EPI_ISL_641522	CHU de Nice - Hôpital Archet 3	CNR Virus des Infections Respiratoires - France SUD	Antonin Bal; Bruno Lina; Gregory Destras; Gwendolyne Burfin; Géraldine Gonfrier; Hadrien Règue; Laurence Josset; Martine Valette; Quentin Semanas; Valérie Giordanengo
EPI_ISL_641523	CHU de Nice - Hôpital Archet 4	CNR Virus des Infections Respiratoires - France SUD	Antonin Bal; Bruno Lina; Gregory Destras; Gwendolyne Burfin; Géraldine Gonfrier; Hadrien Règue; Laurence Josset; Martine Valette; Quentin Semanas; Valérie Giordanengo
EPI_ISL_641524	CHU de Nice - Hôpital Archet 5	CNR Virus des Infections Respiratoires - France SUD	Antonin Bal; Bruno Lina; Gregory Destras; Gwendolyne Burfin; Géraldine Gonfrier; Hadrien Règue; Laurence Josset; Martine Valette; Quentin Semanas; Valérie Giordanengo
EPI_ISL_641525	CHU de Nice - Hôpital Archet 6	CNR Virus des Infections Respiratoires - France SUD	Antonin Bal; Bruno Lina; Gregory Destras; Gwendolyne Burfin; Géraldine Gonfrier; Hadrien Règue; Laurence Josset; Martine Valette; Quentin Semanas; Valérie Giordanengo
EPI_ISL_641526	CHU de Nice - Hôpital Archet 7	CNR Virus des Infections Respiratoires - France SUD	Antonin Bal; Bruno Lina; Gregory Destras; Gwendolyne Burfin; Géraldine Gonfrier; Hadrien Règue; Laurence Josset; Martine Valette; Quentin Semanas; Valérie Giordanengo
EPI_ISL_641521	CHU de Nice - Hôpital Archet 8	CNR Virus des Infections Respiratoires - France SUD	Antonin Bal; Bruno Lina; Gregory Destras; Gwendolyne Burfin; Géraldine Gonfrier; Hadrien Règue; Laurence Josset; Martine Valette; Quentin Semanas; Valérie Giordanengo
EPI_ISL_641527	CHU de Nice - Hôpital Archet 9	CNR Virus des Infections Respiratoires - France SUD	Antonin Bal; Bruno Lina; Gregory Destras; Gwendolyne Burfin; Géraldine Gonfrier; Hadrien Règue; Laurence Josset; Martine Valette; Quentin Semanas; Valérie Giordanengo
EPI_ISL_641519, EPI_ISL_641535 to 641545, EPI_ISL_645184 to 645187, EPI_ISL_649942 to 649953, EPI_ISL_660326 to 660352, EPI_ISL_660432	CHU de Saint-Étienne Hôpital Nord	CNR Virus des Infections Respiratoires - France SUD	Antonin Bal; Bruno Lina; Bruno Pozzetto; Gregory Destras; Gwendolyne Burfin; Hadrien Règue; Issam Bechri; Laurence Josset; Manon Vogrig; Marine Delorme; Martine Valette; Quentin Semanas; Sylvie Gonzalo; Sylvie Pillet; Thomas Bourlet
EPI_ISL_1085411, EPI_ISL_1085881, EPI_ISL_1110185, EPI_ISL_1110200	CHU. COTE DE NACRE	Department of Virology, Henri Mondor University Hospital, Assistance Publique Hôpitaux de Paris, Université Paris-Est Créteil, INSERM U955	Alexandre Soulier; Christophe Rodriguez; Elisabeth Trawinski; Guillaume Gricourt; Jean-Michel Pawlotsky; Melissa N'Debi; Slim Fourati; Vanessa Demontant
EPI_ISL_418024	CHUA - Faro	Instituto Nacional de Saude (INSA)	Guiomar et al
EPI_ISL_1265610 to 1265624, EPI_ISL_1265630 to 1265669	CHUGA-IBP-laboratoire de Virologie	IBP-laboratoire de virologie	Anne Signori -Schmuck; Anne-Karen Faure; Aurélie Truffot; Benjamin Nemoz; Julien Andréani; Julien Lupo; Léa Ponderand; Pascal Poignard; Patrice Morand; Raphaël Germi; Sylvie Larrat
EPI_ISL_535736 to 535737, EPI_ISL_535741, EPI_ISL_535772 to 535775, EPI_ISL_535781 to 535782, EPI_ISL_535784 to 535785, EPI_ISL_535822 to 535825, EPI_ISL_535828			
see above	CHUL-LABO MULTI / MICRO	Laboratoire de santé publique du Québec	Guillaume Bourque; Ioannis Ragoussis; Jesse Shapiro; Mark Lathrop and Michel Roger; Mark Lathrop and Michel Roger on behalf of the CoVSeQ research group; Sandrine Moreira
EPI_ISL_417988, EPI_ISL_417990 to 417991, EPI_ISL_417994 to 417996, EPI_ISL_418010 to 418016	CHULC - H Curry Cabral	Instituto Nacional de Saude (INSA)	Guiomar et al
EPI_ISL_417992 to 417993	CHULC - H D Estefania	Instituto Nacional de Saude (INSA)	Guiomar et al
EPI_ISL_535726, EPI_ISL_535729 to 535730, EPI_ISL_535735, EPI_ISL_535740, EPI_ISL_535753 to 535754, EPI_ISL_535760, EPI_ISL_535783, EPI_ISL_535789, EPI_ISL_535802, EPI_ISL_535806, EPI_ISL_535814, EPI_ISL_535826 to 535827, EPI_ISL_535830, EPI_ISL_535832			
see above	CHUM - Microbiologie - Hôpital Saint-Luc	Laboratoire de santé publique du Québec	Guillaume Bourque; Ioannis Ragoussis; Jesse Shapiro; Mark Lathrop and Michel Roger; Mark Lathrop and Michel Roger on behalf of the CoVSeQ research group; Sandrine Moreira
EPI_ISL_745260	CHUM-Site Glen-LAB Microbiologie	Laboratoire de santé publique du Québec	Guillaume Bourque; Ioannis Ragoussis; Jesse Shapiro; Mark Lathrop and Michel Roger on behalf of the CoVSeQ research group (http://covseq.ca/researchgroup); Sandrine Moreira
EPI_ISL_1007586 to 1007587, EPI_ISL_1007591 to 1007595, EPI_ISL_1007599 to 1007601, EPI_ISL_1007603, EPI_ISL_1007611, EPI_ISL_1007614 to 1007615, EPI_ISL_1007620 to 1007623, EPI_ISL_1007637, EPI_ISL_1007639, EPI_ISL_1120824, EPI_ISL_1120831 to 1120835, EPI_ISL_1120874, EPI_ISL_1120876 to 1120877			
see above	CHUV	Laboratory of genomics and metagenomics, Institute of Microbiology, University Hospital Centre and University of Lausanne, Switzerland	Claire Bertelli; Damien Jacot; Gilbert Greub; Sébastien Aeby; Trestan Pillonel
EPI_ISL_1299240 to 1299248, EPI_ISL_1299251 to 1299253, EPI_ISL_1299255 to 1299256, EPI_ISL_1311966 to 1311972	CHWAPI - SITE NOTRE DAME	Institut de Pathologie et Genetique (IPG)	Jérémie Gras; Pascale Hilbert
EPI_ISL_1191728 to 1191735, EPI_ISL_1201554 to 1201557, EPI_ISL_1219011 to 1219020, EPI_ISL_1229169 to 1229170	CHWAPI - SITE NOTRE DAME	Institut de Pathologie et Génétique (IPG)	Jérémie Gras; Pascale Hilbert
EPI_ISL_683835	CICM	Malaria Research and Training Center (MRTC-Parasito)	Abdoulaye Djimde; Antoine Dara
EPI_ISL_487446 to 487466	CICM-Mali	Bundeswehr Institut of Microbiology	Antwerpen; Bestehorn-Willmann; Dürr; Heitzer; Kouriba; Maiga; Quedraogo; Rehn; Sangaré; Sogodogo; Traoré; Walter; Wölfel; Zimmermann
EPI_ISL_1219714	CICSaB/UASLP/LESP SLP	Instituto de Diagnostico y Referencia Epidemiologicos (INDRE)	Abril Rodriguez-Maldonado; Andreu Comas-Garcia; Ariadna Medina-Benitez; Claudia Wong-Arambula; David Fragoso-Fonseca; Ernesto Ramirez-Gonzalez.; Gisela Barrera-Badillo; Irma Lopez-Martinez; Joaquin Quiroz-Mercado; Lucia Hernandez-Rivas; Natividad Cruz-Ortiz; Sergio

			Rangel-Guerrero; Tatiana Nunez-Garcia; Vanessa Rivero-Arredondo
EPI_ISL_445245, EPI_ISL_445248, EPI_ISL_445250, EPI_ISL_445253 to 445255, EPI_ISL_445258, EPI_ISL_445260	CLINICA ALEMANA DE SANTIAGO S.A.	Instituto de Salud Publica de Chile	Alejandra Acevedo; Andrés E Castillo; Bárbara Parra; Carolina Tambley; Gabriel Leal; Jaime Lagos; Jorge Fernandez; Loredana Arata; Patricia Bustos; Paz Tapia; Rodrigo Fasce; Winston Andrade
EPI_ISL_1196428	CLINICA BIBLICA	Inciensa, Instituto Costarricense de Investigación y Enseñanza en Nutrición y Salud	Adriana Godínez; Claudio Soto-Garita; Estela Cordero; Francisco Duarte; Hebleen Porras; Melany Calderón & Karla Gutiérrez-González
EPI_ISL_445272, EPI_ISL_445285, EPI_ISL_445287	CLINICA CIUDAD DEL MAR	Instituto de Salud Publica de Chile	Alejandra Acevedo; Andrés E Castillo; Bárbara Parra; Carolina Tambley; Gabriel Leal; Jaime Lagos; Jorge Fernandez; Loredana Arata; Patricia Bustos; Paz Tapia; Rodrigo Fasce; Winston Andrade
EPI_ISL_1303374	CLINICA COLSANITAS CENTRAL DE REFERENCIA	Instituto Nacional de Salud- Dirección de Investigación en Salud Pública	Carlos Franco-Muñoz; Carmen Osorio; Diana Malo; Diego A. Álvarez-Díaz; Diego Andrés Prada; Gerardo Santamaría; Hector Alejandro Ruiz-Moreno; Jhonnatan Reales-González; Juan Camilo Martinez; Julian Naizaque; Katherine Laiton-Donato; Lisseth Pardo; Magdalena Wiesner; Marcela Mercado-Reyes; Maria T. Herrera-Sepúlveda; Marta Lopez Blanco; Martha Lucia Ospina Martinez; Sergio Gomez; Sheryll Corchuelo; Ángela Alarcon Cruz
EPI_ISL_906540, EPI_ISL_906544	CLINICA DE OCCIDENTE	Instituto Nacional de Salud- Dirección de Investigación en Salud Pública, Universidad de los Andes- Applied genomics research group, Vicerrectoria de Investigación y Creación, Universidad de los Andes- Systems and Computing Engineering Department	Carlos Franco-Muñoz; Diego A. Álvarez-Díaz; Diego Andrés Prada; Gerardo Santamaría Jorge Duitama; Héctor Alejandro Ruiz-Moreno; Jhonnatan Reales-González; Jorge Ivan Diaz; Julian Naizaque; Katherine Laiton-Donato; Laura Natalia Gonzalez; Magdalena Wiesner; Marcela Mercado-Reyes; Maria T. Herrera-Sepúlveda; Martha Lucia Ospina Martinez; Mauricio Pacheco-Montealegre; Sheryll Corchuelo; Silvia Restrepo-Restrepo
EPI_ISL_445297	CLINICA INTEGRAL S.A.	Instituto de Salud Publica de Chile	Alejandra Acevedo; Andrés E Castillo; Bárbara Parra; Carolina Tambley; Gabriel Leal; Jaime Lagos; Jorge Fernandez; Loredana Arata; Patricia Bustos; Paz Tapia; Rodrigo Fasce; Winston Andrade
EPI_ISL_445256, EPI_ISL_445259	CLINICA LAS CONDES S.A.	Instituto de Salud Publica de Chile	Alejandra Acevedo; Andrés E Castillo; Bárbara Parra; Carolina Tambley; Gabriel Leal; Jaime Lagos; Jorge Fernandez; Loredana Arata; Patricia Bustos; Paz Tapia; Rodrigo Fasce; Winston Andrade
EPI_ISL_445282 to 445283, EPI_ISL_445290 to 445292, EPI_ISL_445299	CLINICA MAGALLANES S.A.	Instituto de Salud Publica de Chile	Alejandra Acevedo; Andrés E Castillo; Bárbara Parra; Carolina Tambley; Gabriel Leal; Jaime Lagos; Jorge Fernandez; Loredana Arata; Patricia Bustos; Paz Tapia; Rodrigo Fasce; Winston Andrade
EPI_ISL_906536	CLINICA OCCIDENTE	Instituto Nacional de Salud- Dirección de Investigación en Salud Pública, Universidad de los Andes- Applied genomics research group, Vicerrectoria de Investigación y Creación, Universidad de los Andes- Systems and Computing Engineering Department	Carlos Franco-Muñoz; Diego A. Álvarez-Díaz; Diego Andrés Prada; Gerardo Santamaría Jorge Duitama; Héctor Alejandro Ruiz-Moreno; Jhonnatan Reales-González; Jorge Ivan Diaz; Julian Naizaque; Katherine Laiton-Donato; Laura Natalia Gonzalez; Magdalena Wiesner; Marcela Mercado-Reyes; Maria T. Herrera-Sepúlveda; Martha Lucia Ospina Martinez; Mauricio Pacheco-Montealegre; Sheryll Corchuelo; Silvia Restrepo-Restrepo
EPI_ISL_445264	CLINICA REDSALUD VITACURA.	Instituto de Salud Publica de Chile	Alejandra Acevedo; Andrés E Castillo; Bárbara Parra; Carolina Tambley; Gabriel Leal; Jaime Lagos; Jorge Fernandez; Loredana Arata; Patricia Bustos; Paz Tapia; Rodrigo Fasce; Winston Andrade
EPI_ISL_445249	CLINICA SANTA MARIA S.A.	Instituto de Salud Publica de Chile	Alejandra Acevedo; Andrés E Castillo; Bárbara Parra; Carolina Tambley; Gabriel Leal; Jaime Lagos; Jorge Fernandez; Loredana Arata; Patricia Bustos; Paz Tapia; Rodrigo Fasce; Winston Andrade
EPI_ISL_445257, EPI_ISL_445262	CLINICA TABANCURA	Instituto de Salud Publica de Chile	Alejandra Acevedo; Andrés E Castillo; Bárbara Parra; Carolina Tambley; Gabriel Leal; Jaime Lagos; Jorge Fernandez; Loredana Arata; Patricia Bustos; Paz Tapia; Rodrigo Fasce; Winston Andrade
EPI_ISL_445306, EPI_ISL_445312, EPI_ISL_445315, EPI_ISL_445361	CLINICA UC SAN CARLOS DE APOQUINDO	Instituto de Salud Publica de Chile	Alejandra Acevedo; Andrés E Castillo; Bárbara Parra; Carolina Tambley; Gabriel Leal; Jaime Lagos; Jorge Fernandez; Loredana Arata; Patricia Bustos; Paz Tapia; Rodrigo Fasce; Winston Andrade
EPI_ISL_445334	CLINICA UNIVERSITARIA DE PUERTO MONTT S.A.	Instituto de Salud Publica de Chile	Alejandra Acevedo; Andrés E Castillo; Bárbara Parra; Carolina Tambley; Gabriel Leal; Jaime Lagos; Jorge Fernandez; Loredana Arata; Patricia Bustos; Paz Tapia; Rodrigo Fasce; Winston Andrade
EPI_ISL_445330	CLINICA VESPUCIO S. A.	Instituto de Salud Publica de Chile	Alejandra Acevedo; Andrés E Castillo; Bárbara Parra; Carolina Tambley; Gabriel Leal; Jaime Lagos; Jorge Fernandez; Loredana Arata; Patricia Bustos; Paz Tapia; Rodrigo Fasce; Winston Andrade
EPI_ISL_536310	CLSC et Centre d'Hébergement la Petite-Nation	Laboratoire de santé publique du Québec	Guillaume Bourque; Ioannis Ragoussis; Jesse Shapiro; Mark Lathrop and Michel Roger on behalf of the CoVSeQ research group; Sandrine Moreira
EPI_ISL_1299497 to 1299498	CMA Dano	Centre Muraz	Abdoul-Salam Ouedraogo; Ange Badjo; Armel Poda; Arsène Somé; Arsène Zongo; Essia Belarbi; Fabian Leendertz; Firmin Kaboré; Grit Schubert; Jasmin Schlotterbeck; Soumeya Ouangraoua; Thérèse Kagone; Yacouba Sawadogo
EPI_ISL_420043, EPI_ISL_420061	CMIP	National Reference Center for Viruses of Respiratory Infections, Institut Pasteur, Paris	Angela Brisebarre; Etienne Simon-Lorière; Flora Donati; Marion Barbet; Maud Vanpeene; Mélanie Albert; Méline Bizard; Sylvie Behillil; Sylvie van der Werf; Vincent Enouf
EPI_ISL_539492, EPI_ISL_547584, EPI_ISL_560320 to 560321	CMS, Roorkee	CSIR-Institute of Microbial Technology	Amandeep Kaur; Anu Singh; Ashwani Kumar; Debarghya Ghose; Dipak Dutta; Harsh Goar; Kanika Bansal; Navin Baid; Poushali Chakraborty; Prabhu B. Patil; Rajesh Kumar Mishra; Sanjeet Kumar; Sanjeev Khosla
EPI_ISL_410486, EPI_ISL_416745 to 416746, EPI_ISL_508912 to 508930, EPI_ISL_508933, EPI_ISL_508937, EPI_ISL_508939 to 508940, EPI_ISL_508942, EPI_ISL_508945, EPI_ISL_508948, EPI_ISL_508951, EPI_ISL_508953 to 508957, EPI_ISL_508961 to 508965, EPI_ISL_508967, EPI_ISL_508969 to 508974, EPI_ISL_508976 to 508977, EPI_ISL_508979 to 508997, EPI_ISL_508999 to 509002, EPI_ISL_525536 to 525538, EPI_ISL_525540 to 525543, EPI_ISL_578176 to 578177, EPI_ISL_582110 to 582120, EPI_ISL_582122, EPI_ISL_582508, EPI_ISL_623098 to 623102, EPI_ISL_636476 to 636477, EPI_ISL_636479, EPI_ISL_636484 to 636485, EPI_ISL_636487, EPI_ISL_636489 to 636490, EPI_ISL_639974 to 639977, EPI_ISL_639979 to 639983, EPI_ISL_639985 to 640014, EPI_ISL_676542 to 676573, EPI_ISL_678494 to 678508, EPI_ISL_678534 to 678547, EPI_ISL_681261 to 681263, EPI_ISL_683334 to 683402, EPI_ISL_684107, EPI_ISL_692733 to 692768, EPI_ISL_693488 to 693514, EPI_ISL_700327, EPI_ISL_728304 to 728320, EPI_ISL_728516 to 728551, EPI_ISL_730640 to 730651, EPI_ISL_732677 to 732703, EPI_ISL_745307 to 745311, EPI_ISL_745316 to 745321, EPI_ISL_745323 to 745324, EPI_ISL_745326 to 745333, EPI_ISL_745337 to 745391, EPI_ISL_745393 to 745397, EPI_ISL_768827 to 768828, EPI_ISL_779780, EPI_ISL_779783 to 779784, EPI_ISL_779787 to 779813, EPI_ISL_779845, EPI_ISL_900526, EPI_ISL_900532 to 900533, EPI_ISL_900544 to 900567, EPI_ISL_1135054 to 1135055, EPI_ISL_1135062, EPI_ISL_1135064 to 1135065, EPI_ISL_1135067, EPI_ISL_1135074 to 1135075, EPI_ISL_1165906, EPI_ISL_1190769 to 1190775, EPI_ISL_1190783, EPI_ISL_1191630 to 1191631, EPI_ISL_1191633, EPI_ISL_1191638, EPI_ISL_1191642 to 1191643, EPI_ISL_1191645 to 1191646, EPI_ISL_1191673 to 1191674, EPI_ISL_1191677 to 1191679, EPI_ISL_1191710, EPI_ISL_1191713 to 1191715, EPI_ISL_1201854 to 1201856, EPI_ISL_1201875 to 1201879, EPI_ISL_1220096, EPI_ISL_1220188 to 1220199, EPI_ISL_1220304 to 1220308, EPI_ISL_1220311, EPI_ISL_1220315, EPI_ISL_1220349, EPI_ISL_1239443, EPI_ISL_1239446, EPI_ISL_1241864 to 1241865, EPI_ISL_1241955 to 1241957, EPI_ISL_1241959, EPI_ISL_1241961, EPI_ISL_1241964 to 1241966, EPI_ISL_1241968 to 1241971, EPI_ISL_1241974 to 1241975, EPI_ISL_1241984 to 1241985, EPI_ISL_1241987 to 1241989, EPI_ISL_1241994 to 1242004, EPI_ISL_1242006, EPI_ISL_1253548, EPI_ISL_1253555, EPI_ISL_1253567, EPI_ISL_1253579, EPI_ISL_1265463, EPI_ISL_1265466, EPI_ISL_1265468 to 1265469, EPI_ISL_1265481, EPI_ISL_1265485, EPI_ISL_1265489, EPI_ISL_1265491, EPI_ISL_1265497 to 1265499, EPI_ISL_1265506 to 1265507, EPI_ISL_1265512 to 1265514, EPI_ISL_1265548 to 1265550, EPI_ISL_1265552, EPI_ISL_1265554, EPI_ISL_1265571, EPI_ISL_1265578 to 1265584, EPI_ISL_1265592 to 1265593, EPI_ISL_1265598 to 1265606, EPI_ISL_1265674 to 1265680, EPI_ISL_1265682, EPI_ISL_1265684, EPI_ISL_1265686 to 1265687, EPI_ISL_1265689 to 1265692, EPI_ISL_1265697 to 1265700, EPI_ISL_1265702 to 1265706, EPI_ISL_1265711, EPI_ISL_1265714, EPI_ISL_1265721 to 1265725, EPI_ISL_1265730 to 1265731, EPI_ISL_1265752, EPI_ISL_1265754 to 1265756, EPI_ISL_1265759, EPI_ISL_1265761, EPI_ISL_1265765, EPI_ISL_1265767, EPI_ISL_1265769, EPI_ISL_1265771 to 1265772, EPI_ISL_1265776, EPI_ISL_1265778 to 1265784, EPI_ISL_1265797 to 1265798, EPI_ISL_1265800 to 1265806, EPI_ISL_1265808 to 1265815, EPI_ISL_1265818 to 1265819, EPI_ISL_1265828 to 1265829, EPI_ISL_1265831 to 1265832, EPI_ISL_1265835, EPI_ISL_1265864 to 1265865, EPI_ISL_1265868 to 1265875, EPI_ISL_1265878, EPI_ISL_1287756 to 1287758, EPI_ISL_1297384, EPI_ISL_1297421, EPI_ISL_1297454 to 1297455, EPI_ISL_1312806 to 1312821, EPI_ISL_1312824 to 1312826, EPI_ISL_1312828, EPI_ISL_1312915 to 1312922, EPI_ISL_1312924 to 1312926, EPI_ISL_1312929 to 1312931, EPI_ISL_1312933 to 1312940, EPI_ISL_1312942, EPI_ISL_1312944, EPI_ISL_1312952 to 1312956, EPI_ISL_1312960 to 1312961, EPI_ISL_1312963 to 1312967, EPI_ISL_1312969 to 1312978, EPI_ISL_1312980 to 1312981, EPI_ISL_1312985 to 1312987, EPI_ISL_1312995 to 1312997, EPI_ISL_1313002, EPI_ISL_1313009 to 1313011, EPI_ISL_1313019 to 1313020, EPI_ISL_1313022 to 1313023, EPI_ISL_1313026 to 1313029, EPI_ISL_1313034 to 1313038, EPI_ISL_1313047 to 1313048, EPI_ISL_1313084, EPI_ISL_1313114 to 1313115, EPI_ISL_1313117, EPI_ISL_1313120, EPI_ISL_1313128 to 1313132, EPI_ISL_1313135 to 1313136, EPI_ISL_1313141 to 1313145, EPI_ISL_1313147, EPI_ISL_1313151, EPI_ISL_1313153 to 1313155, EPI_ISL_1313160, EPI_ISL_1313163, EPI_ISL_1313165 to 1313167, EPI_ISL_1313171 to 1313177, EPI_ISL_1313560, EPI_ISL_1313562 to 1313564, EPI_ISL_1313566 to 1313567, EPI_ISL_1313569, EPI_ISL_1313571 to 1313573, EPI_ISL_1313576 to 1313577, EPI_ISL_1313579 to 1313583, EPI_ISL_1313585 to 1313588, EPI_ISL_1313590 to 1313594, EPI_ISL_1313597 to 1313598, EPI_ISL_1313601 to 1313602, EPI_ISL_1313604 to 1313614, EPI_ISL_1313616 to 1313626, EPI_ISL_1313628 to 1313636, EPI_ISL_1313638, EPI_ISL_1313640 to 1313645, EPI_ISL_1313649 to 1313651, EPI_ISL_1313653 to 1313655, EPI_ISL_1313658 to 1313665, EPI_ISL_1313667 to 1313670, EPI_ISL_1313672 to 1313673, EPI_ISL_1313675, EPI_ISL_1313680 to 1313683, EPI_ISL_1313685 to 1313686, EPI_ISL_1313690, EPI_ISL_1313692 to 1313695, EPI_ISL_1313697, EPI_ISL_1313700 to 1313703, EPI_ISL_1313711, EPI_ISL_1313886 to 1313896, EPI_ISL_1313899 to 1313901, EPI_ISL_1313903 to 1313906, EPI_ISL_1313909 to 1313912, EPI_ISL_1313914, EPI_ISL_1313918 to 1313919, EPI_ISL_1313922, EPI_ISL_1313924 to 1313941, EPI_ISL_1313944 to 1313958, EPI_ISL_1313961 to 1313978, EPI_ISL_1313981 to 1313993, EPI_ISL_1313995 to 1313997, EPI_ISL_1313999 to 1314001, EPI_ISL_1314003 to 1314005, EPI_ISL_1314007 to 1314009, EPI_ISL_1314011, EPI_ISL_1314013 to 1314014, EPI_ISL_1314016 to 1314017, EPI_ISL_1314021 to 1314028, EPI_ISL_1314030, EPI_ISL_1314042, EPI_ISL_1314052 to 1314053			
see above	CNR Virus des Infections Respiratoires - France SUD	CNR Virus des Infections Respiratoires - France SUD	Alexandre; Alexandre Gaymard; Antonin; Antonin Bal; Bal; Bouscambert-Duchamp; Brengel-Pesce; Bruno Lina; Bruno.; Carine Moustaud; Cheynet; Claudia Gonzalez; Destras; Emilie Frobert; Florence; Florence Morfin-Sherpa; Gaymard; Gregory; Gregory Destras; Gregory Queromes; Gwendolyne Burfin; Hadrien Regue; Hadrien Règue; Josset; Karen; Laurence; Laurence Josset; Lina; Martine; Martine Valette; Maude; Maude Bouscambert-Duchamp; Morfin-Sherpa; Quentin Semanas; Raphaelle Lamy; Solenne Brun; Valette; Valérie
EPI_ISL_452127 to 452130	CO Department of Public Health and Environment	Pathogen Discovery, Respiratory Viruses Branch, Division of Viral Diseases, Centers for Disease Control and Prevention	Alison S. Laufer Halpin; Anna Montmayeur; Anna Uehara; Christopher A. Elkins; Clinton R. Paden; Haibin Wang; Jing Zhang; Krista Queen; Mary S. Keckler; Rachel Marine; Suxiang Tong; Yan Li; Ying Tao; Zachary Weiner
EPI_ISL_751575, EPI_ISL_751630, EPI_ISL_751701, EPI_ISL_751704 to 751705, EPI_ISL_751728, EPI_ISL_751763, EPI_ISL_751766, EPI_ISL_903648, EPI_ISL_903781, EPI_ISL_903798, EPI_ISL_903936			
see above	CO Dept. of Public Health and Environment, Lab Services	Genomics and Discovery, Respiratory Viruses Branch,	Anna Montmayeur; Anna Uehara; Ben L. Rambo-Martin; Clinton R. Paden; Dhwani Batra; Haibin Wang; Jasmine Padilla; Jing Zhang; Justin Lee; Krista

	Division	Division of Viral Diseases, Centers for Disease Control and Prevention	Queen; Lori Rowe; Mark Burroughs; Mili Sheth; Peter W. Cook; Rachel Marine; Sarah Nobles; Suxiang Tong; Yan Li; Ying Tao
EPI_ISL_1094231, EPI_ISL_1094234, EPI_ISL_1094261, EPI_ISL_1094511 to 1094516, EPI_ISL_1094899 to 1094906	CO Dept. of Public Health and Environment, Lab Services Division	Respiratory Viruses Branch, Division of Viral Diseases, Centers for Disease Control and Prevention	Anna Montmayeur; Anna Uehara; Ben L. Rambo-Martin; Clinton R. Paden; Dhwani Batra; Haibin Wang; Jasmine Padilla; Jing Zhang; Justin Lee; Krista Queen; Lori Rowe; Mark Burroughs; Mili Sheth; Peter W. Cook; Rachel Marine; Sarah Nobles; Suxiang Tong; Yan Li; Ying Tao
EPI_ISL_416994, EPI_ISL_418248 to 418249	COMPLEJO ASISTENCIAL UNIVERSITARIO DE BURGOS	Instituto de Salud Carlos III	A. Monzón; F. Casas; G. Hospital: ------; I. Jiménez; I. Megias-Lobon G.; Iglesias-Caballero; M. Camarero; M. Camarero S. Pozo F. Casas I. Jiménez P. Jiménez M. Zaballos A. Monzón; M. Cuesta; M. González-Esguevillas; M. Molinero Calamita; M. Zaballos; P. Jiménez; S. Juliá; S. Juliá M. Cuesta I. Megias Lobón; S. Pozo; S. Varona
EPI_ISL_1159194 to 1159197, EPI_ISL_1261458, EPI_ISL_1261479, EPI_ISL_1261495, EPI_ISL_1261508 to 1261509, EPI_ISL_1261518, EPI_ISL_1261538 to 1261539, EPI_ISL_1261680, EPI_ISL_1261682			
see above	COMPLEJO HOSPITALARIO DE NAVARRA	Instituto de Salud Carlos III	A; A. Monzón; ANA; F. Casas; I. Jiménez; I.NAVASCUES ORTEGA; I.Navascues; Iglesias-Caballero; M. Sandonís; P. Zaballos; S. Camarero; S. Cuesta; S. Iglesias-Caballero; S. Pozo; S. Varona; V. Camarero; V. Vázquez-Morón; Vázquez-Morón
EPI_ISL_1059924 to 1059926, EPI_ISL_1159136, EPI_ISL_1261480	COMPLEJO HOSPITALARIO UNIVERSITARIO LA CORUÑA	Instituto de Salud Carlos III	A. Monzón; F; F. Casas; FERNANDA; I. Jiménez; I. Peña, F.; I.PEÑA RODRIGUEZ; I.Peña; Iglesias-Caballero; M. Sandonís; P. Zaballos; S. Camarero; S. Cuesta; S. Iglesias-Caballero; S. Pozo; S. Varona; V. Camarero; V. Vázquez-Morón; Vázquez-Morón
EPI_ISL_954780	COMPLEJO HOSPITALARIO XERAL-CALDE	Instituto de Salud Carlos III	A. Monzón; F. Casas; I. Alonso, P.; I. Jiménez; Iglesias-Caballero; M. Camarero; P. Zaballos; S. Cuesta; S. Pozo; S. Sandonís; S. Varona; V. Vázquez
EPI_ISL_1059984, EPI_ISL_1159226, EPI_ISL_1261502 to 1261503, EPI_ISL_1261507, EPI_ISL_1261516, EPI_ISL_1261533, EPI_ISL_1262079, EPI_ISL_1262081 to 1262082, EPI_ISL_1262084 to 1262085			
see above	CONSEJERIA DE SANIDAD Y ASUNTOS SOCIALES	Instituto de Salud Carlos III	A. Monzón; F. Casas; G; GONZALO; I. Gutiérrez, G.; I. Jiménez; I.GUTIERREZ AVILA; I.Gutiérrez; Iglesias-Caballero; M. Camarero; M. Sandonís; P. Zaballos; S. Camarero; S. Cuesta; S. Iglesias-Caballero; S. Pozo; S. Varona; Sandonís; V. Camarero; V. Vázquez-Morón; Vázquez-Morón
EPI_ISL_434538	COOPESAIN	Inciensa, Instituto Costarricense de Investigación y Enseñanza en Nutrición y Salud	Adriana Godinez & Melany Calderon; Claudio Soto-Garita; Estela Cordero; Francisco Duarte; Hebleen Porras
EPI_ISL_735408	COVID 19 Centro de Combate ao Coronavirus CCC Jandira	Instituto Adolfo Lutz, Interdiciplinary Procedures Center, Strategic Laboratory	Claudia Regina Gonçalves; Claudio Tavares Sacchi; Erica Valessa Ramos Gomes; Karoline Rodrigues Campos
EPI_ISL_891259	COVID lab, Department of Microbiology	Department of Pathology, Bangladesh Agricultural University and Department of Microbiology, Mymensingh Medical College	Afrin; S. K. Parvin, R.; S.Z. Paul
EPI_ISL_882614, EPI_ISL_882619, EPI_ISL_882622, EPI_ISL_882626, EPI_ISL_882630, EPI_ISL_882633 to 882634	COVID lab, Mymensingh Medical College	Department of Pathology, Bangladesh Agricultural University & Department of Microbiology, Mymensingh Medical College	Afrin; S. K. Parvin, R.; S. Z. Paul
EPI_ISL_891258, EPI_ISL_891260, EPI_ISL_891262, EPI_ISL_892245	COVID lab, Mymensingh Medical College	Department of Pathology, Bangladesh Agricultural University and Department of Microbiology, Mymensingh Medical College	Afrin; S. K. Parvin, R.; S. Z. Paul; S. k. Parvin, R.
EPI_ISL_891257	COVID lab, Mymensingh Medical College	Department of Pathology, Bangladesh Agricultural University and Department of Microbiology, Mymensingh Medical College,	Afrin; S. K. Parvin, R.; S. Z. Paul
EPI_ISL_891261, EPI_ISL_892247	COVID lab, Mymensingh Medical College	Department of Pathology, Bangladesh Agricultural University and Department of Microbiology, Mymensingh Medical College	Afrin; S. K. Parvin, R.; S. K. Paul; S. Z. Paul
EPI_ISL_450841	COVID-19 Laboratory	DNA Solution Ltd	ABM Khademul Islam; AHM Nurun Nabi; Abu Sufian; Gazi Nurun Nahar; Habibul Bari Shozib; Haseena Khan; Imran Khan; Latiful Bari; M Anwar Hossain.; MA Malek; Mamun Ahmed; Md Imdadul Hoque; Md Ismail Hosen; Md Mizanur Rahman; Mohammad Riazul Islam; Nazmul Ahsan; Richard Malo; Sabita Rezwana Rahman; Sabrina Moriom Elius; Shahryar Nabi; Sharif Akhteruzzaman; Zeba Islam Seraj
EPI_ISL_450842 to 450843	COVID-19 Laboratory	DNA Solution Ltd.	ABM Khademul Islam; AHM Nurun Nabi; Abu Sufian; Gazi Nurun Nahar; Habibul Bari Shozib; Haseena Khan; Imran Khan; Latiful Bari; M Anwar Hossain.; MA Malek; Mamun Ahmed; Md Imdadul Hoque; Md Ismail Hosen; Md Mizanur Rahman; Mohammad Riazul Islam; Nazmul Ahsan; Richard Malo; Sabita Rezwana Rahman; Sabrina Moriom Elius; Shahryar Nabi; Sharif Akhteruzzaman; Zeba Islam Seraj
EPI_ISL_450840	COVID-19 Laboratory	DNA Solution Ltd. L-5	ABM Khademul Islam; AHM Nurun Nabi; Abu Sufian; Gazi Nurun Nahar; Habibul Bari Shozib; Haseena Khan; Imran Khan; Latiful Bari; M Anwar Hossain.; MA Malek; Mamun Ahmed; Md Imdadul Hoque; Md Ismail Hosen; Md Mizanur Rahman; Mohammad Riazul Islam; Nazmul Ahsan; Richard Malo; Sabita Rezwana Rahman; Sabrina Moriom Elius; Shahryar Nabi; Sharif Akhteruzzaman; Zeba Islam Seraj
EPI_ISL_450839	COVID-19 Laboratory Centre for Advanced Research in Sciences (CARS), University of Dhaka, Dhaka-1000, Bangladesh	DNA Solution Ltd	ABM Khademul Islam; AHM Nurun Nabi; Abu Sufian; Gazi Nurun Nahar; Habibul Bari Shozib; Haseena Khan; Imran Khan; Latiful Bari; M Anwar Hossain.; MA Malek; Mamun Ahmed; Md Imdadul Hoque; Md Ismail Hosen; Md Mizanur Rahman; Mohammad Riazul Islam; Nazmul Ahsan; Richard Malo; Sabita Rezwana Rahman; Sabrina Moriom Elius; Shahryar Nabi; Sharif Akhteruzzaman; Zeba Islam Seraj
EPI_ISL_847826 to 847827	COVID-19 National Reference Laboratory	COVID-19 National Reference Laboratory	Ahmad Ghasemi; Amitis Ramezani; Farideh Niknam; Hessam Nemati; Kayhan Azadmanesh; Mahsa Tavakoli; Maryam Rostamtabar; Marzieh Sadjadi; Mohamad Sadegh Shams Nosrati; Mohammad Hassan Pouriayevali; Mohammad Mehdi Mortazavipour; Mostafa Salehi-Vaziri; Parastoo Yekta; Sahar Khakifirouz; Sana Eybpoosh; Sanam Azad-Manjiri; Sepideh Gerdooei; Setareh Kashanian; Tahereh Mohammadi; Tahmineh Jalali; Zabihollah Shoja; Zahra Ahmadi; Zahra Fereydouni; Zeynab VeisiZadeh
EPI_ISL_803107	COVID-19 National Reference Laboratory, Pasteur Institute of Iran	COVID-19 National Reference Laboratory, Pasteur Institute of Iran	Ahmadi, Z.; Azadmanjiri, S.; Azarm, A.; Fereydouni, Z.; Gouya; Hassanpour, G.; Hassanzadeh, M.; Heidari; Jalali, T.; Karami, C.; Khayatzadeh, S.; M.H.; M.M.; Mohebali, M.; Parsaei, M.; Pouriayevali; Sadjadi, M.; Sarafraz, N.; Sasani, F.; Shoja, Z.; TavakoliRad, M.; Z. and Salehi-Vaziri, M.; Zainali, M.; Zarei, Z.
EPI_ISL_877764	COVID-19 lab, MMC Department of Microbiology, Mymensingh Medical college	Department of Pathology, Bangladesh Agricultural University & Department of Microbiology, Mymensingh Medical College	Afrin; S. K. Parvin, R.; S. Z. Paul
EPI_ISL_412981	CR&WISCO GENERAL HOSPITAL	Hubei Provincial Center for Disease Control and Prevention	Bin Fang; Bo Yang; Bo Yu; Faxian Zhan; Guojun Ye; Jing Li; Junqiang Xu; Kun Cai; Linlin Liu; Xiang Li; Xiao Yu; Xixiang Huo; Yongzhong Jiang.
EPI_ISL_1190749 to 1190768	CREMER(Centre de Rechercherches sur les Maladies Emergentes et Ré-émergentes)	TransVIHMI(Recherches Translationnelles sur le VIH et les Maladies Infectieuses)	Ahidjo Ayouba; Celestin Godwe; Christelle Butel; Dowbiss Meta Djomsi; Eitel Mpoudi Ngole; Eric Delaporte; Esemu Livo; Laetitia Serrano; Marcel Tongo; Marie Amougou; Martin Maidadi Foudi; Martine Peeters; Nicole Vidal; Rodrigue Kamga
EPI_ISL_1085292 to 1085293, EPI_ISL_1085333 to 1085334, EPI_ISL_1085405	CRICQUEBOEUF Cerballiance Normandie	Department of Virology, Henri Mondor University Hospital, Assistance Publique Hôpitaux de Paris, Université Paris-Est Créteil, INSERM U955	Alexandre Soulier; Christophe Rodriguez; Elisabeth Trawinski; Guillaume Gricourt; Jean-Michel Pawlotsky; Melissa N'Debi; Slim Fourati; Vanessa Demontant
EPI_ISL_861662	CS I Tacito Leite de Carvalho e Silva	Instituto Adolfo Lutz, Interdiciplinary Procedures Center, Strategic Laboratory	Claudia Regina Gonçalves; Claudio Tavares Sacchi; Erica Valessa Ramos Gomes; Karoline Rodrigues Campos
EPI_ISL_574593, EPI_ISL_574596, EPI_ISL_583494	CS II Dr. Antonio Vicoso Moreira de Rezende Sumare	Instituto Adolfo Lutz, Interdiciplinary Procedures Center, Strategic Laboratory	Claudia Regina Gonçalves; Claudio Tavares Sacchi; Erica Valessa Ramos Gomes; Karoline Rodrigues Campos
EPI_ISL_636980	CS Xai Xai	KRISP, KZN Research Innovation and Sequencing Platform	Giandhari J; Ismael N; Nadia Sitoe; Nedio Mabunda; Paulo Arnaldo; Pillay S; Tegally H; Wilkinson E; de Oliveira T
EPI_ISL_486853	CSIR-CDRI/SGPGI	CSIR-CDRI/SGPGI	Dharam Veer Singh; Rahul Vishvkarma; Rajender Singh; Ravishankar Ramachandran; Saumya Sarkar; Tapas Kumar Kundu; Uday Ghoshal; Ujjala Ghoshal
EPI_ISL_490013	CSIR-CDRI/SGPGI, Lucknow	CSIR-CDRI, Lucknow	Dharam Veer Singh; Rahul Vishvkarma; Rajender Singh; Ravishankar Ramachandran; Saumya Sarkar; Tapas Kumar Kundu; Uday Ghoshal; Ujjala Ghoshal

EPI_ISL_489995, EPI_ISL_490104, EPI_ISL_490106, EPI_ISL_491096, EPI_ISL_491113 to 491114, EPI_ISL_491477 to 491480, EPI_ISL_497762 to 497763, EPI_ISL_497765, EPI_ISL_497767			
see above	CSIR-CDRI/SGPGI, Lucknow	CSIR-CDRI/SGPGI, Lucknow	Dharam Veer Singh; Rahul Vishvkarma; Rajender Singh; Ravishankar Ramachandran; Saumya Sarkar; Tapas Kumar Kundu; Uday Ghoshal; Ujjala Ghoshal
EPI_ISL_497766	CSIR-CDRI/SGPGI, Lucknow	CSIR-CDRi/SGPGI, Lucknow	Dharam Veer Singh; Rahul Vishvkarma; Rajender Singh; Ravishankar Ramachandran; Saumya Sarkar; Tapas Kumar Kundu; Uday Ghoshal; Ujjala Ghoshal
EPI_ISL_497758, EPI_ISL_497760	CSIR-CDRI/SGPGI, Lucknow	CSIr-CDRI/SGPGI, Lucknow	Dharam Veer Singh; Rahul Vishvkarma; Rajender Singh; Ravishankar Ramachandran; Saumya Sarkar; Tapas Kumar Kundu; Uday Ghoshal; Ujjala Ghoshal
EPI_ISL_497764	CSIR-CDRi/SGPGI, Lucknow	CSIR-CDRI/SGPGI, Lucknow	Dharam Veer Singh; Rahul Vishvkarma; Rajender Singh; Ravishankar Ramachandran; Saumya Sarkar; Tapas Kumar Kundu; Uday Ghoshal; Ujjala Ghoshal
EPI_ISL_447556 to 447583, EPI_ISL_447847 to 447866, EPI_ISL_450326 to 450332, EPI_ISL_458045 to 458065, EPI_ISL_458070 to 458077, EPI_ISL_458080, EPI_ISL_458298, EPI_ISL_471585 to 471646, EPI_ISL_495161 to 495273, EPI_ISL_528823 to 528869, EPI_ISL_539616 to 539775, EPI_ISL_910031 to 910221, EPI_ISL_910223 to 910323			
see above	CSIR-Centre for Cellular and Molecular Biology	CSIR-Centre for Cellular and Molecular Biology	Ajay Sarawagi; Amrutha H C; Ananga Ghosh; Annapoorna P Karthyayani; Archana Bharadwaj Siva; B Himasri; Blessy B John; Debabrata Jana; Debrya Saha; Deepak Kumar; Devi Prasad Vijayashankar; Devi Prasad Vijayashankara; Dhiviya Vedagiri; Disha Nanda; Divya Das; Divya Gupta; Divya Tej Sowpati; G. Aditya Kumar; Gangumala Srinivas Reddy; Gokulan C G; Gunjan Purohit; Hanuman Tulashiram Kale; Jotin Gogoi; Kakade Aishwarya Arun; Karthik Bharadwaj Tallapaka; Kezia J Ann; Koushick Sivakumar; Krishnan Harinivas Harshan; Lamuk Zaveri; M Soujanya Reddy; M Soujanya Reddy Rakesh K Mishra; Manish Bhattacharjee; Namami Gaur; Nikhil Hajirnis; Onkar Kulkarni; Pankaj Kumar; Payel Mukherjee; Peddapuvala Sai Uday Kiran; Peddapuvala Sai Uday Kiran Rakesh K Mishra; Pooja Ramesh Gupta; Prachand Issarapu; Pratheusa Maccha; Preethi Jampala; Preethi Jampala Rakesh K Mishra; Priya Singh; Priyanka Pant; Purushotham Vodnala; Radhika Khandelwal; Rajan Kumar Jha; Rajkanwar Nathawat; Rakesh K Mishra; Ravi Prasad Mukku; Renu Sudhakar; Roshan Maku Venkata; Sakshi Shambhavi; Santosh Kumar Kuncha; Shagufta Khan; Sharada Ravi Iyer; Shemin Mansuri; Shraddha Vijay Lahoti; Sofia Banu; Somesh Gorde; Sonu Uday; Sudipta Mondal; Sujoy Deb; Sulagana Mukherjee; Swati Bayyana; Swetha Sundar; Tulasi Nagabandi; Umesh Kumar; Unis Ahmad Bhat; Vishal Sah; Viswagithe S L; Zeba Rizvi; Zuberwasim Sayyad
EPI_ISL_911532 to 911544, EPI_ISL_911546 to 911547	CSIR-IGIB	CSIR-IGIB	Akshay Kanakan; Anil Kumar; Aparna S Murali; Azka Khan; Janani SV; Nisha Rawat; Partha Chattopadhyay; Priti Devi; Priyanka Mehta; Rajesh Pandey; Ranjeet Maurya; Shweta Sahni
EPI_ISL_569859 to 569863, EPI_ISL_578080 to 578081, EPI_ISL_578168, EPI_ISL_578175, EPI_ISL_578178 to 578184, EPI_ISL_581449 to 581451, EPI_ISL_581494 to 581497, EPI_ISL_581499 to 581504, EPI_ISL_582028, EPI_ISL_661304 to 661311, EPI_ISL_944762 to 944763, EPI_ISL_944765, EPI_ISL_944768 to 944769, EPI_ISL_944771 to 944773, EPI_ISL_944775 to 944776, EPI_ISL_944781, EPI_ISL_944783 to 944784, EPI_ISL_944788 to 944789, EPI_ISL_944791			
see above	CSIR-Indian Institute of Chemical Biology, MEDICA Supercpecialty Hospital Kolkata	CSIR-Indian Institute of Chemical Biology, MEDICA Supercpecialty Hospital Kolkata	Abhishake Lahiri; Debaleena Bhowmik; Dr. Partha Chakrabarti; Dr. Arpita Ghosh Mitra; Dr. Aviral Roy; Dr. Aviral Roy; Dr. AviralRoy; Dr. Partha Chakrabarti; Dr. Rajesh Pandey; Dr. Saikat Chakrabarti; Dr. Sandip Paul; Dr. Soumen Saha; Dr. Soumen Saha; Dr.Partha Chakrabarti; Priyanka Mallick; Sujay Krishna Maity
EPI_ISL_450306, EPI_ISL_465685, EPI_ISL_465687, EPI_ISL_465692, EPI_ISL_535799 to 535800, EPI_ISL_535813, EPI_ISL_535815, EPI_ISL_535841, EPI_ISL_535846, EPI_ISL_535864, EPI_ISL_535876 to 535877, EPI_ISL_535884, EPI_ISL_535902, EPI_ISL_535910, EPI_ISL_535925, EPI_ISL_535928, EPI_ISL_535946, EPI_ISL_535967, EPI_ISL_535984, EPI_ISL_536027 to 536028, EPI_ISL_536040, EPI_ISL_536042, EPI_ISL_536044, EPI_ISL_536110, EPI_ISL_536150, EPI_ISL_536158, EPI_ISL_536160 to 536161, EPI_ISL_536164, EPI_ISL_536166, EPI_ISL_536172, EPI_ISL_536181, EPI_ISL_536197, EPI_ISL_536213, EPI_ISL_536215 to 536217, EPI_ISL_536235, EPI_ISL_536237, EPI_ISL_536243, EPI_ISL_536256, EPI_ISL_536258, EPI_ISL_536272, EPI_ISL_536278, EPI_ISL_536286, EPI_ISL_536299, EPI_ISL_536301 to 536302, EPI_ISL_536304, EPI_ISL_536321, EPI_ISL_536344 to 536345, EPI_ISL_536361, EPI_ISL_536382 to 536383			
see above	CSSS Haut-Richelieu/Rouville (Hôpital)	Laboratoire de santé publique du Québec	Guillaume Bourque; Ioannis Ragoussis; Jesse Shapiro; Mark Lathrop and Michel Roger; Mark Lathrop and Michel Roger on behalf of the CoVSeQ research group; Sandrine Moreira
EPI_ISL_536109	CSSS de Port-Cartier	Laboratoire de santé publique du Québec	Guillaume Bourque; Ioannis Ragoussis; Jesse Shapiro; Mark Lathrop and Michel Roger on behalf of the CoVSeQ research group; Sandrine Moreira
EPI_ISL_535970, EPI_ISL_536206, EPI_ISL_536260	CSSS de la Minganie	Laboratoire de santé publique du Québec	Guillaume Bourque; Ioannis Ragoussis; Jesse Shapiro; Mark Lathrop and Michel Roger; Mark Lathrop and Michel Roger on behalf of the CoVSeQ research group; Sandrine Moreira
EPI_ISL_1272720 to 1272726	CT-Dr. Katherine A. Kelley State Public Health Lab	Centers for Disease Control and Prevention Division of Viral Diseases, Pathogen Discovery	Anna Montmayeur; Anna Uehara; Ben L. Rambo-Martin; Clinton R. Paden; Dhwani Batra; Haibin Wang; Jasmine Padilla; Jing Zhang; Justin Lee; Katie Dillon; Krista Queen; Kristen Knipe; Kristine Lacek; Lori Rowe; Mark Burroughs; Matthew Schmerer; Mili Sheth; Peter W. Cook; Rachel Marine; Sam Shepard; Sarah Nobles; Shoshona Le; Suxiang Tong; Yan Li; Ying Tao
EPI_ISL_751554, EPI_ISL_751624, EPI_ISL_751649 to 751650, EPI_ISL_751706, EPI_ISL_751729, EPI_ISL_751759, EPI_ISL_751762, EPI_ISL_903851, EPI_ISL_903921, EPI_ISL_1226212 to 1226213			
see above	CT-Dr. Katherine A. Kelley State Public Health Lab	Genomics and Discovery, Respiratory Viruses Branch, Division of Viral Diseases, Centers for Disease Control and Prevention	Anna Montmayeur; Anna Uehara; Ben L. Rambo-Martin; Clinton R. Paden; Dhwani Batra; Haibin Wang; Jasmine Padilla; Jing Zhang; Justin Lee; Katie Dillon; Krista Queen; Kristen Knipe; Kristine Lacek; Lori Rowe; Mark Burroughs; Matthew Schmerer; Mili Sheth; Peter W. Cook; Rachel Marine; Sam Shepard; Sarah Nobles; Shoshona Le; Suxiang Tong; Yan Li; Ying Tao
EPI_ISL_426416, EPI_ISL_454642, EPI_ISL_454645, EPI_ISL_812141, EPI_ISL_914036 to 914038, EPI_ISL_1008916	CT-Dr. Katherine A. Kelley State Public Health Lab	Pathogen Discovery, Respiratory Viruses Branch, Division of Viral Diseases, Centers for Disease Control and Prevention	Alison S. Laufer Halpin; Anna Montmayeur; Anna Uehara; Bettina Bankamp; Brian Lynch; Christopher A. Elkins; Clinton R. Paden; Haibin Wang; Jing Zhang; Krista Queen; Mary S. Keckler; Peter Cook; Rachel Marine; Suxiang Tong; Yan Li; Ying Tao; Zachary Weiner
EPI_ISL_468314, EPI_ISL_583503	CTA Centro de Testagem e Aconselhamento	Instituto Adolfo Lutz, Interdiciplinary Procedures Center, Strategic Laboratory	Claudia Regina Gonçalves; Claudio Tavares Sacchi; Erica Valessa Ramos Gomes; Karoline Rodrigues Campos
EPI_ISL_445303	CTRO.DE SALUD FAMILIAR DR. RAUL YAZIGI	Instituto de Salud Publica de Chile	Alejandra Acevedo; Andrés E Castillo; Bárbara Parra; Carolina Tambley; Gabriel Leal; Jaime Lagos; Jorge Fernandez; Loredana Arata; Patricia Bustos; Paz Tapia; Rodrigo Fasce; Winston Andrade
EPI_ISL_451935, EPI_ISL_452140, EPI_ISL_452142, EPI_ISL_452148 to 452152	CUB Hopital Erasme Laboratoire d'Anatomie Pathologique	CUB Hopital Erasme Laboratoire d'Anatomie Pathologique	Dr Nicky D'Haene; Dr. Nicky D'Haene; Dr.Nikcy D'Haene; Isabelle Salmon; Nicky D'Haene; Nikcy D'Haene; Prof. Isabelle Salmon
EPI_ISL_756369 to 756374	CUMC - CHI Bergan Mercy	Creighton University School of Medicine, Departments of Medical Microbiology and Pharmacology and Neuroscience	Anne V. Cheng; Christopher J. Destache; Holly A. Stessman; Jacob A. Siedlik; Michael Belshan; Morgan A. Raine; Richard V. Goering
EPI_ISL_909946 to 909952, EPI_ISL_918489 to 918490	CUSL/UCLouvain COVID testing federal platform	UCLouvain/IREC/MBLG	Benoit Kabamba Mukadi; Jean Ruelle; Lysa Pinsmaye
EPI_ISL_535749, EPI_ISL_535761, EPI_ISL_535764 to 535765, EPI_ISL_535811, EPI_ISL_536337, EPI_ISL_536365 to 536366	CUSM-Site Glen-LAB Microbiologie	Laboratoire de santé publique du Québec	Guillaume Bourque; Ioannis Ragoussis; Jesse Shapiro; Mark Lathrop and Michel Roger; Mark Lathrop and Michel Roger on behalf of the CoVSeQ research group; Sandrine Moreira
EPI_ISL_515938 to 515942	CV RAMAN HOSPITAL	Department of Neurovirology, National Institute of Mental Health and Neuroscience (NIMHANS)	Anita Desai; Chitra Pattabiraman; Harsha PK; Manjunatha Venkataswamy; Pramada Prasad; Ravi Vasanthapuram; Risha Rasheed; Shafeeq S Hameed; Vijayalakshmi Reddy
EPI_ISL_486383, EPI_ISL_486881	CV Raman Hospital	Department of Neurovirology, National Institute of Mental Health and Neuroscience (NIMHANS)	Anita Desai; Chitra Pattabiraman; Harsha PK; Manjunatha Venkataswamy; Ravi Vasanthapuram; Risha Rasheed; Shafeeq S Hameed; Vijayalakshmi Reddy
EPI_ISL_428364, EPI_ISL_428367, EPI_ISL_443300 to 443302, EPI_ISL_443306, EPI_ISL_443311 to 443313, EPI_ISL_443317	Cabinet Médical	National Reference Center for Viruses of Respiratory Infections, Institut Pasteur, Paris	Angela Brisebarre; Etienne Simon-Lorière; Flora Donati; Marion Barbet; Maud Vanpeene; Mélanie Albert; Méline Bizard; Sylvie Behillil; Sylvie van der Werf; Vincent Enouf
EPI_ISL_1299249 to 1299250, EPI_ISL_1299254	Cabinet Privé	Institut de Pathologie et Genetique (IPG)	Jérémie Gras; Pascale Hilbert
EPI_ISL_1085287, EPI_ISL_1085289, EPI_ISL_1085403, EPI_ISL_1110192	Cabinet médical	Department of Virology, Henri Mondor University Hospital, Assistance Publique Hôpitaux de Paris, Université Paris-Est Créteil, INSERM U955	Alexandre Soulier; Christophe Rodriguez; Elisabeth Trawinski; Guillaume Gricourt; Jean-Michel Pawlotsky; Melissa N'Debi; Slim Fourati; Vanessa Demontant
EPI_ISL_418235	Cabinet médical	National Reference Center for Viruses of Respiratory Infections, Institut Pasteur, Paris	Angela Brisebarre; Etienne Simon-Lorière; Flora Donati; Marion Barbet; Maud Vanpeene; Mélanie Albert; Méline Bizard; Sylvie Behillil; Sylvie van der Werf; Vincent Enouf

EPI_ISL_418812 to 418813, EPI_ISL_429807, EPI_ISL_429817 to 429820, EPI_ISL_482474 to 482475, EPI_ISL_482478, EPI_ISL_482480 to 482484	Cadham Provincial Laboratory	National Microbiology Laboratory	; Anna Majer; David Alexander; Elsie Grudeski; Gary Van Domselaar; Grace Seo; Jared Bullard; Jennifer Tanner; Kerry Dust; Kristyn Burak; Matthew Gilmour; Morag Graham; Natalie Knox; Nathalie Bastien; Paul Van Caeseele; Philip Mabon; Rhiannon Huzarewich; Russell Mandes; Russell Mandez; Shari Tyson; Timothy Booth; Yan Li
EPI_ISL_582242 to 582249, EPI_ISL_582251 to 582286, EPI_ISL_582288 to 582306, EPI_ISL_582308, EPI_ISL_582311 to 582343, EPI_ISL_582345 to 582347, EPI_ISL_582349 to 582397, EPI_ISL_582399 to 582414, EPI_ISL_582416 to 582420, EPI_ISL_582422 to 582443, EPI_ISL_582445 to 582507			
see above	Cadham Provincial Laboratory	National Microbiology Laboratory (NML)	Anna Majer; Anneliese Landgraff; CanCOGeN's metadata curation team; Darian Hole; David Alexander; Elsie Grudeski; Gary Van Domselaar; Grace Seo; Jared Bullard; Jennifer Tanner; Kerry Dust; Madison Chapel; Morag Graham; Natalie Knox; Nathalie Bastien; Paul Van Caeseele; Philip Mabon; Public Health Agency of Canada CanCOGeN team; Rhiannon Huzarewich; Russell Mandes; Shari Tyson; Timothy Booth; Yan Li
EPI_ISL_632909 to 632933	Cadham Provincial laboratory	Cadham Provincial laboratory	Anna Majer; Anneliese Landgraff; CanCOGeN's metadata curation team; Darian Hole; David Alexander; Elsie Grudeski; Gary Van Domselaar; Grace Seo; Jared Bullard; Jennifer Tanner; Kerry Dust; Madison Chapel; Morag Graham; Natalie Knox; Nathalie Bastien; Paul Van Caeseele; Philip Mabon; Public Health Agency of Canada CanCOGeN team; Rhiannon Huzarewich; Russell Mandes; Shari Tyson; Timothy Booth; Yan Li
EPI_ISL_935753 to 935970, EPI_ISL_937514, EPI_ISL_979271 to 979326	Cadham Provincial laboratory	National Microbiology Laboratory (NML)	Anna Majer; Anneliese Landgraff; CanCOGeN's metadata curation team; Darian Hole; David Alexander; Elsie Grudeski; Gary Van Domselaar; Grace Seo; Jared Bullard; Jennifer Tanner; Kerry Dust; Kirsten Biggar; Madison Chapel; Morag Graham; Natalie Knox; Nathalie Bastien; Paul Van Caeseele; Philip Mabon; Public Health Agency of Canada CanCOGeN team; Rhiannon Huzarewich; Russell Mandes; Shari Tyson; Timothy Booth; Yan Li
EPI_ISL_444794	Cairns Hospital	Public Health Virology Laboratory	Alyssa Pyke; Amanda De Jong; Andrew Van Den Hurk; Bixing Huang; Carmel Taylor; David Warrilow; Doris Genge; Elisabeth Gamez; Glen Hewitson; Ian Maxwell Mackay; Inga Sultana; Jamie McMahon; Jean Barcelon; Judy Northill; Mitchell Finger; Natalie Simpson; Neelima Nair; Peter Burtonclay; Peter Moore; Sarah Wheatley; Sean Moody; Sonja Hall-Mendelin; Timothy Gardam; and Frederick Moore
EPI_ISL_1069176	Calamba Doctors Hospital	Research Institute for Tropical Medicine	Catalino Demetria; Daria Manalo; Edelwisa Mercado; Francisco Gerardo Polotan; Hannah Leah Morito; Inez Andrea Medado; John Leonard Chan; Joseph Hughes; Kirstyn Brunker; Ma Angelica Tujan; Othoniel Jan Onza
EPI_ISL_408009 to 408010	California Department of Health	Pathogen Discovery, Respiratory Viruses Branch, Division of Viral Diseases, Centers for Dieases Control and Prevention	Anna Uehara; Brett L. Whitaker; Brian Lynch; Clinton Paden; Janna' R. Murray; Jing Zhang; Krista Queen; Lijuan Wang; Senthil Kumar K. Sakthivel; Shifaq Kamili; Stephen Lindstrom; Susan I. Gerber; Suxiang Tong; Xiaoyan Lu; Yan Li; Ying Tao
EPI_ISL_408008	California Department of Health	Pathogen Discovery, Respiratory Viruses Branch, Division of Viral Diseases, Centers for Disease Control and Prevention	Anna Uehara; Brett L. Whitaker; Brian Lynch; Clinton Paden; Janna' R. Murray; Jing Zhang; Krista Queen; Lijuan Wang; Senthil Kumar K. Sakthivel; Shifaq Kamili; Stephen Lindstrom; Susan I. Gerber; Suxiang Tong; Xiaoyan Lu; Yan Li; Ying Tao
EPI_ISL_515894 to 515929, EPI_ISL_754914 to 755114, EPI_ISL_824390 to 824406	California Department of Public Health	California Department of Public Health	CDPH IDLB COVIDNet
EPI_ISL_413930	California Department of Public Health	Chiu Laboratory UCSF-Abbott Viral Diagnostics and Discovery Center University of California, San Francisco	Alicia Sotomayor-Gonzalez; Allan Gopez; Chao-Yang Pan; Debra A. Wadford; Guixia Yu; Hugo Guevara; Scot Federman; Steve Miller; Wei Gu; Xianding Deng; and Charles Y. Chiu
EPI_ISL_413557 to 413559, EPI_ISL_413561, EPI_ISL_413922, EPI_ISL_413924 to 413926, EPI_ISL_413928, EPI_ISL_413931, EPI_ISL_417322 to 417329, EPI_ISL_429875 to 429876, EPI_ISL_429881, EPI_ISL_755940 to 755942, EPI_ISL_847516, EPI_ISL_847518 to 847535, EPI_ISL_847537 to 847539, EPI_ISL_847541 to 847544, EPI_ISL_847547 to 847552, EPI_ISL_847554 to 847562, EPI_ISL_847564 to 847578, EPI_ISL_847580 to 847618, EPI_ISL_847623 to 847639, EPI_ISL_847645 to 847715, EPI_ISL_847717 to 847719, EPI_ISL_847721 to 847729, EPI_ISL_847732 to 847735, EPI_ISL_847737, EPI_ISL_847739 to 847740, EPI_ISL_847745, EPI_ISL_847748 to 847781, EPI_ISL_847783 to 847818, EPI_ISL_855389 to 855398, EPI_ISL_977721 to 977742, EPI_ISL_977745 to 977755, EPI_ISL_977757 to 977851, EPI_ISL_977864 to 977867, EPI_ISL_977869, EPI_ISL_977878, EPI_ISL_978976 to 978983, EPI_ISL_984318 to 984362, EPI_ISL_984364 to 984613			
see above	California Department of Public Health	Chiu Laboratory, University of California, San Francisco	Brian Bushnell; Candace Wang; Chao-Yang Pan; Charles Chiu; Debra A. Wadford; Debra Wadford; Hugo Guevara; Jill Hacker; Scot Federman; Venice Servellita; Wei Gu; Xianding (Wayne) Deng; Xianding Deng; and Charles Y. Chiu
EPI_ISL_406034, EPI_ISL_406036, EPI_ISL_410044, EPI_ISL_411954 to 411955	California Department of Public Health	Pathogen Discovery, Respiratory Viruses Branch, Division of Viral Diseases, Centers for Dieases Control and Prevention	Anna Uehara; Brett L. Whitaker; Brian Lynch; Clinton R. Paden; Haibin Wang; Janna' R. Murray; Jing Zhang; Krista Queen; Lijuan Wang; Senthil Kumar K. Sakthivel; Shifaq Kamili; Stephen Lindstrom; Susan I. Gerber; Suxiang Tong; Xiaoyan Lu; Yan Li; Ying Tao
EPI_ISL_412862, EPI_ISL_419554, EPI_ISL_594458	California Department of Public Health	Pathogen Discovery, Respiratory Viruses Branch, Division of Viral Diseases, Centers for Disease Control and Prevention	Anna Uehara; Brett L. Whitaker; Brian Lynch; Clinton Paden; Clinton R. Paden; Haibin Wang; Janna' R. Murray; Jasmine Padilla; Jing Zhang; Julu Bhatnagar; Justin Lee; Krista Queen; Lijuan Wang; Senthil Kumar K. Sakthivel; Shifaq Kamili; Stephen Lindstrom; Susan I. Gerber; Suxiang Tong; Xiaoyan Lu; Yan Li; Ying Tao
EPI_ISL_418865	California Department of Public Health	University of California, San Francisco	Chao-Yang Pan; Debra A. Wadford; Hugo Guevara; Scot Federman; Wei Gu; Xianding Deng; and Charles Y. Chiu
EPI_ISL_885128 to 885138, EPI_ISL_912187 to 912194, EPI_ISL_1234813, EPI_ISL_1234845, EPI_ISL_1234867, EPI_ISL_1234885, EPI_ISL_1234891, EPI_ISL_1234915, EPI_ISL_1234947, EPI_ISL_1234951, EPI_ISL_1234964, EPI_ISL_1234967, EPI_ISL_1234978, EPI_ISL_1234992, EPI_ISL_1234994, EPI_ISL_1235014, EPI_ISL_1235026 to 1235027, EPI_ISL_1235035 to 1235036, EPI_ISL_1235055 to 1235056			
see above	California Institute of Technology	Chan-Zuckerberg Biohub	CZB Cliahub Consortium
EPI_ISL_447594	Caloundra Hospital	Public Health Virology Laboratory	Alyssa Pyke; Amanda De Jong; Andrew Van Den Hurk; Bixing Huang; Carmel Taylor; David Warrilow; Doris Genge; Elisabeth Gamez; Glen Hewitson; Ian Maxwell Mackay; Inga Sultana; Jamie McMahon; Jean Barcelon; Judy Northill; Mitchell Finger; Natalie Simpson; Neelima Nair; Peter Burtonclay; Peter Moore; Sarah Wheatley; Sean Moody; Sonja Hall-Mendelin; Timothy Gardam; and Frederick Moore
EPI_ISL_745121	Calvinia Hospital	National Health Laboratory Service (NHLS), Tygerberg	Bronwyn Kleinhans; Eduan Wilkindon; Gert van Zyl; Houriiyah Tegally; Kayla Delaney; Susan Engelbrecht; Tulio de Oliveira; Wolfgang Preiser
EPI_ISL_1096139 to 1096140, EPI_ISL_1096347 to 1096348, EPI_ISL_1098602 to 1098605	Cambodian National Public Health Laboratory, National Institute of Public Health	Virology Unit, Institut Pasteur du Cambodge	Chau Darapheak; Chin Savuth; Erik A Karlsson; Kraing Sidonn; Ly Sovann; Sokhoun Yann; Veasna Duong; Yi Sengdoeurn
EPI_ISL_576371 to 576373, EPI_ISL_857315 to 857347, EPI_ISL_862783 to 862813, EPI_ISL_890195 to 890231, EPI_ISL_907086 to 907118	Cancer Biology Department, National Cancer Institute	Cancer Biology Department, National Cancer Institute	A.A.; A.E.; A.N.; Abouelhoda, M.; Ahmed; Bahnassy; Elhosieny; F.W.; Gad; H.K.; Hafez; Hamdy; M.G.; M.M.; M.S.; O.S.; Sedawy; Soliman; Soliman, L.; Zekri
EPI_ISL_1040916	Cancer Biology Department, National Cancer Institute	Cancer Biology Department, National Cancer Institute	A.A.; A.E.; A.N.; Ahmed; Bahnassy; Elhosieny; F.W.; Gad; H.K.; Hafez; Hamdy; L. and Abouelhoda, M.; M.G.; M.M.; M.S.; O.S.; Sedawy; Soliman; Zekri
EPI_ISL_447054	Cantacuzino National Military-Medical Institute for Research and Development	Cantacuzino Institute	A.Cretu; L.Ustea; M.Lazar
EPI_ISL_456321 to 456322, EPI_ISL_456348 to 456375, EPI_ISL_536792, EPI_ISL_547980 to 547985, EPI_ISL_548116 to 548118, EPI_ISL_548129, EPI_ISL_548134, EPI_ISL_548138 to 548140, EPI_ISL_579060 to 579092, EPI_ISL_579220 to 579227, EPI_ISL_579408 to 579414, EPI_ISL_579426 to 579471, EPI_ISL_579473 to 579506, EPI_ISL_622768 to 622771, EPI_ISL_622775, EPI_ISL_622780 to 622782, EPI_ISL_622784, EPI_ISL_622786, EPI_ISL_622788 to 622791, EPI_ISL_622794 to 622801, EPI_ISL_622808 to 622813, EPI_ISL_622824 to 622830, EPI_ISL_661260, EPI_ISL_682283 to 682286, EPI_ISL_682295 to 682296, EPI_ISL_732963, EPI_ISL_755619 to 755623, EPI_ISL_755638, EPI_ISL_794627, EPI_ISL_843194 to 843195, EPI_ISL_843197 to 843200, EPI_ISL_877219 to 877220, EPI_ISL_1016881 to 1016882, EPI_ISL_1082255			
see above	Canterbury Health Laboratories	Institute of Environmental Science and Research (ESR)	Anja Werno; Antje van der Linden; Arlo Upton; Chris Mansell; David Hammer; Dragana Drinkovic; Erasmus Smit; Gary McAuliffe; Hana Sofia Andersson; Hermes Perez; James Ussher; Jill Sherwood; Jing Wang; Joep de Ligt; Josh Freeman; Julia Howard; Juliet Elvy; Lauren Jelly; Mary DeAlmeida; Matt Blakiston; Matt Storey; Matthew Rogers; Max Bloomfield; Michael Addidle; Michelle Balm; Muhammad Faisal; Nikki Freed; Olin Silander; Paula scholes; Sally Roberts; Sarah Jefferies; Sharmini Muttaiyah; Susan Lin; Susan Morpeth; Susan Taylor; Timothy Blackmore; Vani Sathyendran; Veronica Playle; Virginia Hope; Xiaoyun Ren
EPI_ISL_812249	Cantonal Hospital Baden	Institute of Medical Virology, University of Zurich	Alexandra Trkola; Annette Audigé; Cyril Shah; Guido Bloemberg; Jon Huder; Jürg Böni; Kevin Steiner; Maria Grünberg; Maryam Zaheri; Michael Huber; Riccarda Capaul; Stefan Schmutz; Verena Kufner
EPI_ISL_730623	Cantonal Hospital Frauenfeld	Institute of Medical Virology, University of Zurich	Alexandra Trkola; Gabriela Ziltener; Jürg Böni; Maryam Zaheri; Michael Huber; Stefan Schmutz; Verena Kufner
EPI_ISL_524478, EPI_ISL_811127	Cantonal Hospital Winterthur	Institute of Medical Virology, University of Zurich	Alexander Wepf; Alexandra Trkola; Andrea Zbinden; Annette Audigé; Cyril Shah; Fiona Steiner; Gabriela Ziltener; Guido Bloemberg; Jon Huder; Jürg Böni; Kevin Steiner; Maria Grünberg; Maryam Zaheri; Michael Huber; Patrick Redli; Riccarda Capaul; Stefan Schmutz; Urs Karrer; Verena Kufner
EPI_ISL_1300646, EPI_ISL_1300654 to 1300655	Cantonal Hospital Zenica; Department of Microbiological Diagnostics	Clinical Center, University of Sarajevo; Unit for Clinical Microbiology	Amela Dedei-Ljubovi; Edina Zahirovi; Golubinka Boshevska; Irma Salimovi-Beši; Maja Kuzmanovska; Sandra Vegar-Zubovi; Sebija Izetbegovi; Suzana Arapi
EPI_ISL_710612	Capio Bro VC	The Public Health Agency of Sweden	Department of Microbiology; The Public Health Agency of Sweden

EPI_ISL_534230 to 534233, EPI_ISL_560981	Capio S:t Gorans sjukhus	The Public Health Agency of Sweden	Anna Risberg; Anna-Malin Linde; Karin Tegmark-Wisell; Maria Lind Karlberg; Mattias Haukland; Mia Brytting; Olov Svartstrom; Oskar Karlsson Lindsjo; Petra Edquist; Reza Advani; Sandra Broddesson
EPI_ISL_491472	Cardinal Santos Medical Center	Research Institute for Tropical Medicine	Catalino Demetria; Criselda Bautista; Daria Manalo; Edelwisa Mercado; Francisco Gerardo Polotan; Inez Andrea Medado; Kirstyn Brunker; Ma. Angelica Tujan; Othoniel Jan Onza
EPI_ISL_771371	CardioVir EA-4684, University of Reims Champagne Ardenne	CardioVir EA-4684, University of Reims Champagne Ardenne	A.-L.; Callon, D.; Fornes; Lebreil; N'Guyen, Y.; P. and Andreoletti, L.
EPI_ISL_977538 to 977539, EPI_ISL_977581, EPI_ISL_977583 to 977584, EPI_ISL_977594	Caribbean Public Health Agency	Carrington Lab, Department of PreClinical Sciences	Adesh Ramsubhag; Arianne Brown-Jordan; Avery Hinds; Chinna Chinnadurai; Christine V. F. Carrington; Christopher Oura; Dr. Sharon Belmar-George; Dr. Sharon Belmar-George Avery Hinds; Gabriel Escobar; Jaya Jayaraman; Jerome Foster; Karla Georges; Marsha Ivey; Naresh Nandram; Nikita S. D. Sahadeo; Nuno Faria; Oliver Pybus; Rahul Naidu; Rajini Haraksingh; Risha Singh; Sarah Hill; Stanley Giddings; SueMin Nathaniel; Vernie Ramkissoon
EPI_ISL_977655 to 977659, EPI_ISL_978931, EPI_ISL_978933, EPI_ISL_978935 to 978936, EPI_ISL_978938, EPI_ISL_978944, EPI_ISL_978947, EPI_ISL_978949			
see above	Caribbean Public Health Agency	Carrington Lab, Department of PreClinical Sciences, Building 36, First Floor Biochemistry Unit, Faculty of Medical Sciences, The University of the West Indies	Adesh Ramsubhag; Arianne Brown-Jordan; Avery Hinds; Chinna Chinnadurai; Christine V. F. Carrington; Christopher Oura; Gabriel Escobar; Irad Potter; Jaya Jayaraman; Jerome Foster; John Lee; Karla Georges; Kenneth George; Marsha Ivey; Naresh Nandram; Nikita S. D. Sahadeo; Nuno Faria; Oliver Pybus; Rahul Naidu; Rajini Haraksingh; Risha Singh; Sarah Hill; Stanley Giddings; SueMin Nathaniel; Vernie Ramkissoon
EPI_ISL_857348, EPI_ISL_872194 to 872196	Caribbean Public Health Agency	Carrington Lab, Department of PreClinical Sciences, Faculty of Medical Sciences, The University of the West Indies	Adesh Ramsubhag; Arianne Brown-Jordan; Avery Hinds; Chinna Chinnadurai; Christine V. F. Carrington; Christopher Oura; Gabriel Escobar; Jaya Jayaraman; Jerome Foster; Karla Georges; Marsha Ivey; Naresh Nandram; Nikita S. D. Sahadeo; Nuno Faria; Oliver Pybus; Rahul Naidu; Rajini Haraksingh; Risha Singh; Roshan Parasram; Sarah Hill; Stanley Giddings; SueMin Nathaniel; Vernie Ramkissoon
EPI_ISL_872193	Caribbean Public Health Agency	Carrington Lab, Department ofBuilding 36, First Floor Biochemistry Unit, Faculty of Medical Sciences, The University of the West Indies	Adesh Ramsubhag; Arianne Brown-Jordan; Avery Hinds; Chinna Chinnadurai; Christine V. F. Carrington; Christopher Oura; Gabriel Escobar; Jaya Jayaraman; Jerome Foster; Karla Georges; Marsha Ivey; Naresh Nandram; Nikita S. D. Sahadeo; Nuno Faria; Oliver Pybus; Rahul Naidu; Rajini Haraksingh; Risha Singh; Sarah Hill; Stanley Giddings; SueMin Nathaniel; Vernie Ramkissoon
EPI_ISL_756360	Caribbean Regional Public Health Authority	Carrington Lab, Department of PreClinical Sciences, Faculty of Medical Sciences, The University of the West Indies	; Adesh Ramsubhag; Arianne Brown-Jordan; Avery Hinds; Chinna Chinnadurai; Christine V. F. Carrington; Christopher Oura; Gabriel Escobar; Jaya Jayaraman; Jerome Foster; Karla Georges; Marsha Ivey; Naresh Nandram; Nikita S. D. Sahadeo; Nuno Faria; Oliver Pybus; Rahul Naidu; Rajini Haraksingh; Risha Singh; Sarah Hill; Simone Keizer-Beache; Stanley Giddings; SueMin Nathaniel; Vernie Ramkissoon
EPI_ISL_409024, EPI_ISL_426407, EPI_ISL_602590, EPI_ISL_637029, EPI_ISL_1055249, EPI_ISL_1055260	Caritas Medical Centre	Hong Kong Department of Health	Chan Rickjason C.W.; Cheng Peter K.C.; Lam Edman T.K.; Mak Gannon C.K.; Tsang Dominic N.C.
EPI_ISL_1220046	Carvajal Laboratorio IPS SAS	Instituto Nacional de Salud- Dirección de Investigación en Salud Pública	Carlos Franco-Muñoz; Diego A. Álvarez-Díaz; Diego Andrés Prada; Gerardo Santamaría; Hector Alejandro Ruiz-Moreno; Jhonnatan Reales-González; Julian Naizaque; Katherine Laiton-Donato; Magdalena Wiesner; Marcela Mercado-Reyes.; Maria T. Herrera-Sepúlveda; Martha Lucia Ospina Martínez; Sheryll Corchuelo
EPI_ISL_794663	Carvajal Laboratorios	Instituto Nacional de Salud - Dirección de Investigación en Salud Pública	Carlos Franco-Muñoz; Diego A. Álvarez-Díaz; Diego Andrés Prada; Gerardo Santamaría; Jonathan Reales; Julian Naizaque; Katherine Laiton-Donato; Magdalena Wiesner; Marcela Mercado-Reyes; Maria T. Herrera; Martha Lucia Ospina Martinez; Mauricio Pacheco-Montealegre; Paola Muñoz-Laiton; Sheryl Corchuelo
EPI_ISL_583504 to 583505	Casa de Saude Stella Maris	Instituto Adolfo Lutz, Interdiciplinary Procedures Center, Strategic Laboratory	Claudia Regina Gonçalves; Claudio Tavares Sacchi; Erica Valessa Ramos Gomes; Karoline Rodrigues Campos
EPI_ISL_806783	Casa di cura Di Lorenzo- Avezzano	Istituto Zooprofilattico Sperimentale dell'Abruzzo e Molise "G. Caporale"	Ancora M; Calistri P; Cammà C; Curini V; Di Domenico M; Di Pasquale A; Lorusso A; Mangone I; Marcacci M; Puglia I; Rinaldi A; Savini G
EPI_ISL_693235	Casmi Centro Atendimento Saude da Mulher e Infancia	Instituto Adolfo Lutz, Interdiciplinary Procedures Center, Strategic Laboratory	Claudia Regina Gonçalves; Claudio Tavares Sacchi; Erica Valessa Ramos Gomes; Karoline Rodrigues Campos
EPI_ISL_1155982 to 1155993, EPI_ISL_1285343, EPI_ISL_1285346, EPI_ISL_1285348, EPI_ISL_1285351 to 1285352, EPI_ISL_1285354, EPI_ISL_1285356, EPI_ISL_1285359, EPI_ISL_1285362, EPI_ISL_1285364, EPI_ISL_1285367, EPI_ISL_1285374			
see above	CeGaT Management	Robert Koch Institute	
EPI_ISL_1122426 to 1122454, EPI_ISL_1213533, EPI_ISL_1213535, EPI_ISL_1213537, EPI_ISL_1213539 to 1213540, EPI_ISL_1213542, EPI_ISL_1213544, EPI_ISL_1213546 to 1213547, EPI_ISL_1213549, EPI_ISL_1213551 to 1213552, EPI_ISL_1213554, EPI_ISL_1213556, EPI_ISL_1213558 to 1213559, EPI_ISL_1213561, EPI_ISL_1219702			
see above	Cebu TB Reference Laboratory	Philippine Genome Center	Alethea R. de Guzman; Anna Ong-Lim; Arianne A. Zamora; Asia Louisa U. Chong; Benedict A. Maralit; Candice Francheska B. Tambaoan; Carlo M. Lapid; Celia Carlos; Devon Ray Pacial; Edsel Maurice Salvaña; El King D. Morado; Eva Maria Cutiongco-de la Paz; Francis A. Tablizo; Irish Coleen A. Asin; Jaime C. Montoya; Jan Michael C. Yap; Jo-Hannah S. Llames; John Q. Wong; Joshua Gregor A. Dizon; Juan Antonio R. Magalang; Karol Sophia Agape R. Padilla; Kenneth M. Kim; Kris P. Punayan; Marc Edsel C. Ayes; Marc Jerrone R. Castro; Maria Rosario Singh-Vergeire and Cynthia P. Saloma; Maria Sofia L. Yangzon; Marissa Alejandria; Razel Nikka M. Hao; Rianna Patricia S. Cruz; Sheila Mae M. Araiza
EPI_ISL_467809, EPI_ISL_475574 to 475716, EPI_ISL_824555 to 824616, EPI_ISL_824618 to 824656, EPI_ISL_824658 to 824741	Cedars-Sinai Medical Center, Department of Pathology & Laboratory Medicine, Molecular Pathology Laboratory	Cedars-Sinai Medical Center, Molecular Pathology Laboratory of Department of Pathology & Laboratory Medicine and Genomic Core	Brian Davis; Eric Vail; Jasmine T Plummer; Jean Lopategui; Jianbo Song; John Paul Govindavari; Jong Taek Kim; Jorge Mario Sincuir Martinez; Stephanie Chen; Wenjuan Zhang
EPI_ISL_971451	Cell culture Unit at CV-MIT belonging to HIMMV	Functional Genomic Platform UATRS-biology, CNRST	Abderrazzak Rfak; Amine Idriss Lahlou; Elmostafa EL FAHIME; Hicham Elannaz; Khalid ENNIBI; Marouane MELLOUL; Mly Abdelaziz ELALAOUI; Mostafa ELOUENNASS; Mouhssine Hemlali; Nadia Touil; Sanaa ALAOUI-Amine
EPI_ISL_1239674, EPI_ISL_1239736	Cell-T	1. National Institute of Public Health - National Institute of Hygiene; 2. Eurofins Genomics Europe Sequencing GmbH	ECDC COVID-19 WGS support team; Eurofins Genomics Europe Sequencing Team; Gierczyki Rafa; Sadkowska-Todys Magorzata; Wokowicz Tomasz; Zacharczuk Katarzyna
EPI_ISL_1239592	Cell-T Sp. z o.o.	1. National Institute of Public Health - National Institute of Hygiene; 2. Eurofins Genomics Europe Sequencing GmbH	ECDC COVID-19 WGS support team; Eurofins Genomics Europe Sequencing Team; Gierczyki Rafa; Sadkowska-Todys Magorzata; Wokowicz Tomasz; Zacharczuk Katarzyna
EPI_ISL_1201656 to 1201665, EPI_ISL_1273820	Cell-t	1. National Institute of Public Health - National Institute of Hygiene; 2. Eurofins Genomics Europe Sequencing GmbH	ECDC COVID-19 WGS support team; Eurofins Genomics Europe Sequencing Team; Gierczyki Rafa; Sadkowska-Todys Magorzata; Wokowicz Tomasz; Zacharczuk Katarzyna
EPI_ISL_756293 to 756294	Center for Biotechnology and Cell Therapy, São Rafael Hospital, Salvador, Brazil	Center for Biotechnology and Cell Therapy, São Rafael Hospital, Salvador, Brazil	Ana Verena Almeida Mendes; Bruno Solano de Freitas Souza; Carolina Kymie Vasques Nonaka; Marta Giovanetti; Marília Miranda Franco; Renato Santana de Aguiar; Tiago Gräf
EPI_ISL_1067728, EPI_ISL_1067732, EPI_ISL_1067736	Center for Biotechnology and Cell Therapy, São Rafael Hospital, Salvador, Brazil	Central Public Health Laboratory - LACEN -Bahia, Salvador, Brazil	Arabela Leal; Breno Dominguez; Felicidade Pereira; Jaqueline Gomes; Luciana Oliveira; Luiz Alcantara; Marcela Gómez; Marta Giovanetti; Patrícia Cajado; Stephane Tosta; Vagner Fonseca; Vanessa Nardy
EPI_ISL_450413	Center for Diagnostics, Institute of Medical Microbiology, Virology and Hygiene	University Medical Center Hamburg-Eppendorf	Huang, J.; Pfefferle; S. and Fischer, N.
EPI_ISL_459856 to 459864, EPI_ISL_468752, EPI_ISL_468760	Center for Genome Regulation (CRG)	Center for Mathematical Modeling and Center for Genome Regulation. Santiago, Chile	Allende ML; Gaete A; González M.; Maass A; Palma R; Travisany D; Urra C; Varas M
EPI_ISL_906780 to 906781, EPI_ISL_906790, EPI_ISL_906792, EPI_ISL_1036246 to 1036267	Center for Genome Research and Biocomputing	Center for Genome Research and Biocomputing	Oregon State University TRACE Project
EPI_ISL_890236	Center for Genome Sciences, US Army Medical Research Institute of Infectious Disease (USAMRIID)	Center for Genome Sciences, US Army Medical Research Institute of Infectious Disease (USAMRIID)	Bateman; Di Paola, N.; Gibson, W.; Kugelman, J.; Richardson, J.; S. and Jerke, K.
EPI_ISL_479663 to 479675, EPI_ISL_481283	Center for Genomics and System Biology, New York University	Center for Genomics and System Biology, New York University	Banakis, S.; Borenstein; Cornelius, A.; D. and Ghedin, E.; E.S.; Fleming, A.; Ghedin, E.; Gresham; Gresham, D.; Herati, R.; Johnson, K.; Khalfan, M.; Kottkamp, A.; M.J.; Mulligan; Raabe, V.; Roder, A.; Samanovic, M.; Ulrich, R.
EPI_ISL_516783 to 516785, EPI_ISL_522439 to 522453, EPI_ISL_522455 to 522464, EPI_ISL_522467 to 522473, EPI_ISL_522476, EPI_ISL_522479 to 522483, EPI_ISL_522485, EPI_ISL_522491 to 522497, EPI_ISL_522502, EPI_ISL_522505, EPI_ISL_522508, EPI_ISL_522510, EPI_ISL_522515 to 522516, EPI_ISL_522518, EPI_ISL_526730 to 526731, EPI_ISL_526734 to 526746			

see above	Center for Laboratory Control of Infectious Diseases, Korea Centers for Diseases Control and Prevention	Center for Laboratory Control of Infectious Diseases, Korea Centers for Diseases Control and Prevention	Ae Kyung Park; Eunkyung Shin; Heui Man Kim; Jeong-Min Kim; Jin Sun No; Junyoung Kim; Myung Guk Han; Yoon-Seok Chung
EPI_ISL_940771	Center for Laboratory Medicine St. Gallen	Center for Laboratory Medicine St. Gallen	Yannick Gerth
EPI_ISL_539870 to 539878	Center for Microbiology and Cell Biology, Instituto Venezolano de Investigaciones Científicas (CMBC, IVIC)	Center for Microbiology and Cell Biology, Instituto Venezolano de Investigaciones Científicas (CMBC, IVIC)	Aguilar, M.; Alarcon, V.; C.L.; D'Angelo, P.; Delgado, M.; F.H.; Garzaro, D.; H.R.; Jaspe; Loureiro; Pujol; R.C.; Rangel; Rodriguez, L.
EPI_ISL_512773, EPI_ISL_513296, EPI_ISL_513309	Center for Precision Medicine, Meizhou People's Hospital (Huangtang Hospital)	Center for Precision Medicine, Meizhou People's Hospital (Huangtang Hospital)	Guo, X.; Wu, H.; Yu; Z. and Huang, Q.
EPI_ISL_493137, EPI_ISL_493139	Center for Research and Innovation, Faculty of Medical Technology, Mahidol University	Center for Research and Innovation, Faculty of Medical Technology, Mahidol University	Anek Mungaomklang; Hatairat Lerdsamran; Jarunee Prasertsopon; Kamolthip Atsawawaranunt; Kantima Sangsiriwut; Nattakan Thinpan; Pilaipan Puthavathana; Prabda Praphasiri; Somrak Sirikhetkon; Tipsuda Chanmanee
EPI_ISL_1061415 to 1061416	Center for Vaccine Research, University of Pittsburgh School of Medicine	Center for Vaccine Research, University of Pittsburgh School of Medicine	A.K.; Bain; Duprex; Haidar, G.; K.R.; L.J.; L.R.; McCarthy; McElroy; Nambulli, S.; Rennick; Robinson-McCarthy; W.G.; W.P.
EPI_ISL_913064 to 913091, EPI_ISL_913093 to 913098, EPI_ISL_1020297 to 1020298, EPI_ISL_1020300 to 1020301, EPI_ISL_1116458 to 1116459, EPI_ISL_1191132 to 1191140	Center for Virology	Center for Virology	Irene Goerzer; Jeremy V. Camp; Monika Redlberger-Fritz; Stephan W. Aberle
EPI_ISL_419654 to 419674, EPI_ISL_437993 to 438128, EPI_ISL_475770 to 475812, EPI_ISL_583563 to 583571, EPI_ISL_583692 to 583726, EPI_ISL_583869 to 583882, EPI_ISL_853751, EPI_ISL_853759, EPI_ISL_853822 to 853853, EPI_ISL_853877 to 853913, EPI_ISL_853957 to 853961, EPI_ISL_854218 to 854219, EPI_ISL_854241, EPI_ISL_854253, EPI_ISL_854258, EPI_ISL_854295, EPI_ISL_854299, EPI_ISL_1117786, EPI_ISL_1117820, EPI_ISL_1117822 to 1117823, EPI_ISL_1117836 to 1117837, EPI_ISL_1117848 to 1117850, EPI_ISL_1117874, EPI_ISL_1117877 to 1117878, EPI_ISL_1117881, EPI_ISL_1117886, EPI_ISL_1118068, EPI_ISL_1118070			
see above	Center for Virology, Medical University of Vienna	Bergthaler laboratory, CeMM Research Center for Molecular Medicine of the Austrian Academy of Sciences	Adi Steinrigl; Alexander Lercher; Alexandra Popa; Andreas Bergthaler; Anna Schedl; Bekir Erguner; Benedikt Agerer; Christian Paar; Christoph Bock; Christoph Bock; Daniela Schmid; Dorothee von Laer; Elisabeth Puchhammer-Stoeckl; Elisabeth Puchhammer-Stöckl; Franz Allerberger; Gernot Walder; Gregor Hörmann; Guenter Weiss; Gunther Vogl; Henrique Colaco; Jakob-Wendelin Genger; Jan Laine; Judith Aberle; Kinga Rigler-Hohenwarter; Lukas Endler; Maelle Le Moing; Manfred Nairz; Mark Smyth; Martin Senekowitsch; Martin Senekowitsch; Michael Schuster; Michael Schuster; Peter Hufnagl; Peter Obrist; Rainer Gattringer; Sabine Sussitz-Rack; Stephan Aberle; Thomas Penz; Wegene Borena
EPI_ISL_516414 to 516425, EPI_ISL_677700 to 677702, EPI_ISL_677706, EPI_ISL_677708, EPI_ISL_678248 to 678249	Center for public health - Skopje	Research Center for Genetic Engineering and Biotechnology "Georgi D. Efremov" , Macedonian Academy of Sciences and Arts	RCGEB - MASA
EPI_ISL_755572 to 755574	Center of Advanced Studies and Technology, CAST	Center of Advanced Studies and Technology, CAST	De Fabritiis, S.; Ferrante, R.; Mandatori, D.
EPI_ISL_888674 to 888676, EPI_ISL_1034737 to 1034739, EPI_ISL_1034748 to 1034753, EPI_ISL_1036235 to 1036239, EPI_ISL_1093477 to 1093491, EPI_ISL_1097002, EPI_ISL_1201280 to 1201286, EPI_ISL_1260840 to 1260852			
see above	Center of Advanced Studies and Technology, Molecular Genetics Laboratory	Center of Advanced Studies and Technology, Molecular Genetics Laboratory	Anaclerio Federico; Damiani Verena; De Fabritiis Simone; Ferrante Rossella; Mandatori Domitilla
EPI_ISL_450875 to 451074	Center of Excellence in Clinical Virology	Center of Excellence in Clinical Virology	Auphimai, C.; Chansaenroj, J.; Nilyanimit, P.; Poovorawan, Y.; Puenpa, J.; Suwannakarn, K.; Yorsaeng, R.
EPI_ISL_1039057 to 1039123	Center of Hygiene and Epidemiology in Belgorod Region	WHO National Influenza Centre Russian Federation	Andrey Komissarov; Anna Ivanova; Artem Fadeev; Daria Danilenko; Dmitry Bazhenov; Dmitry Lioznov; Elena Nabieva; Georgii Bazykin; Ksenia Safina; Kseniya Komissarova; Lyudmila Berdinskikh; Maria Pisareva; Maria Timofeeva; Tamila Musaeva; Veronika Eder
EPI_ISL_523950	Center of Medical Microbiology, Virology, and Hospital Hygiene, University of Duesseldorf	Center of Medical Microbiology, Virology, and Hospital Hygiene, Heinrich Heine University Düsseldorf	Alexander Dilthey; Andreas Walker; Daniel Strelow; Hendrik Streeck; Jessica Nicolai; Jörg Timm; Klaus Pfeffer; Malte Kohns Vasconcelos; Marek Korencak; Maximilian Damagnez; Tobias Wienemann; Torsten Houwaart
EPI_ISL_413488, EPI_ISL_414497 to 414499, EPI_ISL_414504 to 414509, EPI_ISL_414574, EPI_ISL_417457 to 417468, EPI_ISL_419541 to 419552, EPI_ISL_425120 to 425140, EPI_ISL_452180, EPI_ISL_523927 to 523929, EPI_ISL_523931 to 523949, EPI_ISL_602510 to 602517, EPI_ISL_602533 to 602552, EPI_ISL_717976, EPI_ISL_779922 to 779953, EPI_ISL_832364 to 832374, EPI_ISL_1160123, EPI_ISL_1160125, EPI_ISL_1160133, EPI_ISL_1160137 to 1160138, EPI_ISL_1160140, EPI_ISL_1160146, EPI_ISL_1160148, EPI_ISL_1160150, EPI_ISL_1160152, EPI_ISL_1160155, EPI_ISL_1160157, EPI_ISL_1160159, EPI_ISL_1160161, EPI_ISL_1160165, EPI_ISL_1160167, EPI_ISL_1160169, EPI_ISL_1160171 to 1160172, EPI_ISL_1160174, EPI_ISL_1160176, EPI_ISL_1160178, EPI_ISL_1160180, EPI_ISL_1160182, EPI_ISL_1160184, EPI_ISL_1160186, EPI_ISL_1160188, EPI_ISL_1160190, EPI_ISL_1160192, EPI_ISL_1160194 to 1160195, EPI_ISL_1160200, EPI_ISL_1160202, EPI_ISL_1160205, EPI_ISL_1160207, EPI_ISL_1160209, EPI_ISL_1160211, EPI_ISL_1160213, EPI_ISL_1160215, EPI_ISL_1160217, EPI_ISL_1160219, EPI_ISL_1161006, EPI_ISL_1161009, EPI_ISL_1161011, EPI_ISL_1161013 to 1161014, EPI_ISL_1161016, EPI_ISL_1161018, EPI_ISL_1161022, EPI_ISL_1161026, EPI_ISL_1161030, EPI_ISL_1161049 to 1161050, EPI_ISL_1161052, EPI_ISL_1161054, EPI_ISL_1161056, EPI_ISL_1161058, EPI_ISL_1161069			
see above	Center of Medical Microbiology, Virology, and Hospital Hygiene, University of Duesseldorf	Center of Medical Microbiology, Virology, and Hospital Hygiene, University of Duesseldorf	Alexander Dilthey; Andreas Walker; Ashley-Jane Duplessis; Bjorn-Erik Jensen; Björn-Erik Jensen; Björn-ErikJensen; Daniel Strelow; Detlef Kindgen-Milles; Hendrik Streeck; Jessica Nicolai; Jorg Timm; Jörg Timm; Klaus Pfeffer; Lisanna Hülse; Malte Kohns Vasconcelos; Marcel Andree; Marek Korencak; Maximilian Damagnez; Nadine Lübke; Ortwin Adams; Sandra Hauka; Teresa Tamayo; Tina Senff; Tobias Wienemann; Torsten Feldt; Torsten Houwaart
EPI_ISL_523930	Center of Medical Microbiology, Virology, and Hospital Hygiene, University of Duesseldorf	Universitätstr.1 40225 Düsseldorf Germany	Alexander Dilthey; Andreas Walker; Daniel Strelow; Hendrik Streeck; Jessica Nicolai; Jörg Timm; Klaus Pfeffer; Malte Kohns Vasconcelos; Marek Korencak; Maximilian Damagnez; Tobias Wienemann; Torsten Houwaart
EPI_ISL_430820	Center of Scientific Excellence for Influenza Viruses, National Research Centre (NRC), Egypt.	Center of Scientific Excellence for Influenza Viruses, National Research Centre (NRC), Egypt.	Abo Shama; Ahmed E Kayed; Ahmed El-Taweel; Ahmed Kandeil; Ahmed Mostafa; Amal Naguib; M Noura; Mahmoud Shehata; Mina Kamel; Mohamed Ahmed Ali; Mohamed El Sayes; Mokhtar Gomaa; Nancy M. El Guindy; Omnia Kutkat; Rabeh El-Shesheny; Sara Mahmoud; Shymaa Showky Ahmed; Wael Roshdy; Yassmin Moatasim
EPI_ISL_430819	Center of Scientific Excellence for Influenza Viruses,National Research Centre (NRC), Egypt.	Center of Scientific Excellence for Influenza Viruses,National Research Centre (NRC), Egypt.	Abo Shama; Ahmed E Kayed; Ahmed El-Taweel; Ahmed Kandeil; Ahmed Mostafa; Amal Naguib; M Noura; Mahmoud Shehata; Mina Kamel; Mohamed Ahmed Ali; Mohamed El Sayes; Mokhtar Gomaa; Nancy M. El Guindy; Omnia Kutkat; Rabeh El-Shesheny; Sara Mahmoud; Shymaa Showky Ahmed; Wael Roshdy; Yassmin Moatasim
EPI_ISL_429852 to 429855, EPI_ISL_495459	Centers for Disease Control and Prevention of Lishui	Department of InspectionCenters for Disease Control and Prevention of Lishui	Ji Jiansong; Ji Qiaoying; Wang Xiaoguang; Ye Bifeng; Ye Ling
EPI_ISL_539495, EPI_ISL_940756, EPI_ISL_940895 to 940994, EPI_ISL_942007 to 942012, EPI_ISL_1168653 to 1168681	Centers for Disease Control and Prevention, Dengue Branch	Centers for Disease Control and Prevention, Dengue Branch	Betzabel Flores; Diego Sainz de la Peña; Gabriela Paz-Bailey; Gilberto A. Santiago; Glenda Gonzalez; Janice Perez; Jorge Bertran; Jorge L. Munoz-Jordan; Keyla Charriez; Vanessa Rivera-Amill
EPI_ISL_406031, EPI_ISL_420082 to 420085, EPI_ISL_421641, EPI_ISL_421651, EPI_ISL_428488 to 428491, EPI_ISL_429882 to 429884, EPI_ISL_857314			
see above	Centers for Disease Control, R.O.C. (Taiwan)	Centers for Disease Control, R.O.C. (Taiwan)	Ji-Rong Yang; Jung-Jung Mu; Jung-Jung-Mu; Ming-Tsan Liu; Ming-Tsan-Liu; Shu-Ying Li; Yu-Chi Lin; Yu-Chi-Lin
EPI_ISL_815255 to 815399	Centogene	Centogene	Krishna Kumar Kandaswamy; Peter Bauer; Vivi Hue-Trang Lieu
EPI_ISL_457750, EPI_ISL_459962 to 459964	Centogene AG	Centogene AG	Dr. Krishna Kumar Kandaswamy; Prof. Dr. Peter Bauer
EPI_ISL_455583, EPI_ISL_455594	Central Chest Institute of Thailand	National Institute of Health. Department of medical Sciences, Ministry of Public Health, Thailand	Chittaganpitch; Malinee; Okada; Parnmen; Phuygun; Pilailuk; Siripaporn; Sittiporn; Sunthareeya; Thanadachakul; Thanutsapa; Waicharoen; Warawan; Wongboot
EPI_ISL_463313	Central Health Medical Practice	Hong Kong Department of Health	Chan Rickjason C.W.; Cheng Peter K.C.; Lam Edman T.K.; Mak Gannon C.K.; Tsang Dominic N.C.
EPI_ISL_426410	Central Kowloon Health Centre	Hong Kong Department of Health	Chan Rickjason C.W.; Cheng Peter K.C.; Lam Edman T.K.; Mak Gannon C.K.; Tsang Dominic N.C.
EPI_ISL_794593 to 794604	Central Laboratories, Egyptian Ministry of Health and Population	Central Laboratories, Egyptian Ministry of Health and Population	A.E.; Ali; El Guindy; El Sayes; El Taweel, A.; El-Shesheny, R.; Gomaa, M.; Kamel; Kandeil, A.; Kayali, G.; Kayed; Khalifa; Kutkat, O.; M.A.; M.K.; M.N.; Mahmoud; Mahrous, N.; Moatasim, Y.; Mostafa, A.; N.M.; Naguib, A.; Roshdy; S.H.; Saleh, M.; Shawky, S.; Shehata, M.; W.H.
EPI_ISL_930854 to 930858, EPI_ISL_942374 to 942375, EPI_ISL_942407, EPI_ISL_942896 to 942899, EPI_ISL_942930 to 942931, EPI_ISL_943574 to 943613, EPI_ISL_983863 to 983869, EPI_ISL_984619 to 984621			
see above	Central Laboratory of Public Health of Rio Grande do Sul (Lacen-RS)	State Center for Health Surveillance of the Health Department of the State of Rio Grande do Sul (CEVS/SES-RS)	; Aline Campos; Amanda da Silva; Anelise Schaurich; Barcellos R; Campos A; Claudia Dornelles; Crescente L; Cynthia Molina; Da Silva A; Dornelles C; Fernanda Godinho; Fonseca V; Garay L; Godinho F; Gonzalez A; Gregianini T; Lara Crescente; Leticia Garay; Ludmila Fiorenzano Baethgen; Molina C; Regina Barcellos; Richard Salvato; Salvato R; Schaurich A; Tatiana Gregianini; Vagner Fonseca

EPI_ISL_529032	Central Molecular Microbiology Laboratory and Next Generation Sequencing Reference Laboratory, Clinical and Chemical Pathology Department, Faculty of Medicine, CAIRO UNIVERSITY	Next Generation Sequencing Reference Laboratory, Faculty of Medicine, CAIRO UNIVERSITY and The Center for Genome and Microbiome Research, Faculty of Pharmacy, CAIRO UNIVERSITY	May Abdelfattah; May Sherif Soliman; Ramy Karam Aziz
EPI_ISL_529031	Central Molecular Microbiology Laboratory, Clinical and Chemical Pathology Department, Faculty of Medicine, CAIRO UNIVERSITY	Next Generation Sequencing Reference Laboratory, Faculty of Medicine, Cairo University and The Center for Genome and Microbiome Research, Faculty of Pharmacy, CAIRO UNIVERSITY	May Abdelfattah; May Sherif Soliman; Ramy Karam Aziz
EPI_ISL_794605 to 794608	Central Public Health Laboratories, Egyptian Ministry of Health and Population	Central Public Health Laboratories, Egyptian Ministry of Health and Population	A.E.; Ali; El Guindy; El Sayes; El Taweel, A.; El-Shesheny, R.; Gomaa, M.; Kamel; Kandeil, A.; Kayali, G.; Kayed; Khalifa; Kutkat, O.; M.A.; M.K.; M.N.; Mahmoud; Mahrous, N.; Moatasim, Y.; Mostafa, A.; N.M.; Naguib, A.; Roshdy; S.H.; Saleh, M.; Shawky, S.; Shehata, M.; W.H.
EPI_ISL_693471 to 693483, EPI_ISL_1307639 to 1307681	Central Public Health Laboratory	National Public Health Laboratory, National Centre for Infectious Diseases	Esorom Daoni; Lin Cui; Raymond Tzer Pin Lin; Sophie Octavia; Theresa Palou; Tze Minn Mak; Zhenyang Zhou
EPI_ISL_978495 to 978533, EPI_ISL_1067729 to 1067731, EPI_ISL_1067733 to 1067735, EPI_ISL_1067737 to 1067738, EPI_ISL_1068315 to 1068323, EPI_ISL_1068363 to 1068394	Central Public Health Laboratory - LACEN -Bahia, Salvador, Brazil	Central Public Health Laboratory - LACEN -Bahia, Salvador, Brazil	Arabela Leal; Breno Dominguez; Felicidade Pereira; Jaqueline Gomes; Luciana Oliveira; Luiz Alcantara; Marcela Gómez; Marta Giovanetti; Patrícia Cajado; Stephane Tosta; Vagner Fonseca; Vanessa Nardy
EPI_ISL_429664 to 429703	Central Public Health Laboratory/Octávio Magalhães Institute (IOM) from the Ezequiel Dias Foundation (FUNED)	Instituto Octávio Magalhães / Fundação Ezequiel Dias (IOM/Funed)	Joilson Xavier; Luiz Carlos Junior Alcantara; Marcos Vinícius Silva; Marluce Aparecida Assunção Oliveira; Marta Giovanetti; Talita Adelino; Vagner Fonseca
EPI_ISL_1222766	Central Scientific Research Department, Gomel State Medical University,	Laboratory of Genomics and Bioinformatics of the Forest Research Institute of the NAS of Belarus	A.A.; A.S.; Baranov; E.V.; I.O.; Kiryanov; L.V.; O.V.; O.Y.; Osipkina; P.S. and Mozharovskaya; Padutov; Panteleev; S.V.; Shaforost; Stoma; V.E.; Voropaev; Zyatskov
EPI_ISL_447250 to 447251	Central Virology Laboratory	Central Virology Laboratory	Danit Sofer; Efrat Bucris; Ella Mendelson; Michal Mandelboim; Neta Zuckerman; Oran Erster; Orna Mor
EPI_ISL_419211	Central Virology Laboratory	Israel Institute for Biological Research	Adi Beth-Din; Anat Zvi; Boaz Politi; Dana Stein; Einat Vitner; Gadi Segal; Gili Regev-Yochay; Hadas Tamir; Hagit Achdout; Inbar Cohen-Gihon; Lilach Cherry; Michal Mandelboim; Nir Paran; Ofir Israeli; Ohad Shifman; Oran Erster; Orly Laskar; Sharon Melamed; Shay Weiss; Shmuel C. Shapira; Shmuel Yitzhaki; Tomer Israely; Yfat Yahalom Ronen
EPI_ISL_435284, EPI_ISL_435286 to 435287, EPI_ISL_435289, EPI_ISL_435291 to 435292, EPI_ISL_733498, EPI_ISL_737202 to 737204	Central Virology Laboratory, Israel Ministry of Health	Central Virology Laboratory, Israel Ministry of Health	Danit Sofer; Efrat Bucris; Efrat Dahan Bucris; Ella Mendelson; Michal Mandelboim; Neta S. Zuckerman; Neta Zuckerman; Oran Erster; Orna Mor; eta Zuckerman
EPI_ISL_430842	Central chest Institute of Thailand	National Institute of Health. Department of medical Sciences, Ministry of Public Health, Thailand	Chittaganpitch; Malinee; Okada; Parnmen; Phuygun; Pilailuk; Siripaporn; Sittiporn; Sunthareeya; Thanadachakul; Thanutsapa; Waicharoen; Warawan; Wongboot
EPI_ISL_693197	Central de Rede de Frio Municipal	Instituto Adolfo Lutz, Interdiciplinary Procedures Center, Strategic Laboratory	Claudia Regina Gonçalves; Claudio Tavares Sacchi; Erica Valessa Ramos Gomes; Karoline Rodrigues Campos
EPI_ISL_885143	Central public health laboratory	Molecular Diagnostics Department, Central public health laboratory	Dalia, F.; Dler, H.; Dlshad, H.; F.-A.; Fahmi, A.; Furat, S.; Hemdad, A.; Hemn; M. and Idrees, H.; Mohsen, A.; Sharmeen
EPI_ISL_486410, EPI_ISL_486417, EPI_ISL_961805, EPI_ISL_961809 to 961830, EPI_ISL_961833 to 961851, EPI_ISL_1219503, EPI_ISL_1219595, EPI_ISL_1312510 to 1312564, EPI_ISL_1312620 to 1312623, EPI_ISL_1312659 to 1312682			
see above	Centrala laboratorija	Latvian Biomedical Research and Study Centre	Davids Fridmanis; Davids Fridmanis; Guntars Zarins; Ivars Silamikelis; Ivars Silamielis; Ivars Silamikelis; Jana Osite; Jana Oste; Janis Klovins; Janis Pjalkovskis; Janis Klovins; Jurijs Perevoscikovs; Jnis Kloviš; Kaspars Megnis; Kaspars Megnis; Laila Silamikele; Laila Silamikele; Lauma Freimane; Lauma Freimane; Laura Ansone; Laura Ansone; Liga Birzniece; Liga Birzniece; Marta Priedite; Marta Priedte; Monta Ustinova; Monta Ustinova; Nikita Zrelovs; Nikita Zrelovs; Stella Lapia; Uga Dumpis; Uga Dumpis; Una Krumina; Vita Rovite; Vita Rovte; Vita Rovite; ikita Zrelovs
EPI_ISL_548256	Centralsjukhuset	The Public Health Agency of Sweden	Anna Risberg; Anna-Malin Linde; Karin Tegmark-Wisell; Maria Lind Karlberg; Mattias Haukland; Mia Brytting; Olov Svartstrom; Oskar Karlsson Lindsjo; Petra Edquist; Reza Advani; Sandra Broddesson
EPI_ISL_457999 to 458000	Centre For Biotechnology Research and Development	Centre For Biotechnology Research and Development	C.N. and Michuki; D.K.; G.N.; J.O.; Kimotho, J.; Matoke-Muhia; Matoke-Muhia, D.; Muuo; Ochwoto, M.; S.L.; S.N.; Symeker; Waruhiu; Zablon
EPI_ISL_1250633	Centre Hositalier Eure Seine	Centre Hospitalier Universitaire de Rouen Laboratoire de Virologie	Alice Moisan; Fabienne De Oliveira; Marie Leoz
EPI_ISL_414624, EPI_ISL_416494	Centre Hositalier Universitaire de Rouen Laboratoire de Virologie	National Reference Center for Viruses of Respiratory Infections, Institut Pasteur, Paris	Angela Brisebarre; Etienne Simon-Lorière; Flora Donati; Flora Donati Vincent Enouf; Jean-Christophe Plantier; Marion Barbet; Maud Vanpeene; Méline Bizard; Mélnie Albert; Sylvie Behillil; Sylvie van der Werf; Vincent Enouf
EPI_ISL_508958, EPI_ISL_509012	Centre Hospitalier Alpes Leman	CNR Virus des Infections Respiratoires - France SUD	Alexandre Gaymard; Antonin Bal; Bruno Lina; Carine Moustaud; Florence Morfin-Sherpa; Gregory Destras; Gwendolyne Burfin; Laurence Josset; Martine Valette; Maude Bouscambert-Duchamp; Raphaelle Lamy; Solenne Brun
EPI_ISL_414627 to 414630, EPI_ISL_414634 to 414638, EPI_ISL_415653 to 415654, EPI_ISL_416495 to 416497, EPI_ISL_418218, EPI_ISL_418220 to 418221, EPI_ISL_418223 to 418225, EPI_ISL_418227 to 418228, EPI_ISL_418231, EPI_ISL_418236 to 418239, EPI_ISL_429968			
see above	Centre Hospitalier Compiègne Laboratoire de Biologie	National Reference Center for Viruses of Respiratory Infections, Institut Pasteur, Paris	Angela Brisebarre; Etienne Simon-Lorière; Fabiana Gambaro; Flora Donati; Flora Donati Vincent Enouf; Marion Barbet; Maud Vanpeene; Mélanie Albert; Méline Bizard; Mélnie Albert; Raulin Olivia; Sylvie Behillil; Sylvie van der Werf; Vincent Enouf
EPI_ISL_860847, EPI_ISL_860898	Centre Hospitalier Compiègne-Noyon - Laboratoire	National Reference Center for Viruses of Respiratory Infections, Institut Pasteur, Paris	Angela Brisebarre; Camille Capel; Emond Jean-Philippe; Etienne Simon-Lorière; Foissaud Vincent; Marion Barbet; Maud Vanpeene; Méline Bizard; Sylvie Behillil; Sylvie van der Werf; Vincent Enouf
EPI_ISL_1250602	Centre Hospitalier Dieppe	Centre Hospitalier Universitaire de Rouen Laboratoire de Virologie	Alice Moisan; Fabienne De Oliveira; Marie Leoz
EPI_ISL_418428, EPI_ISL_508947	Centre Hospitalier Lucien Hussel	CNR Virus des Infections Respiratoires - France SUD	Alexandre Gaymard; Antonin Bal; Bruno Lina; Carine Moustaud; Florence Morfin-Sherpa; Gregory Destras; Gwendolyne Burfin; Laurence Josset; Martine Valette; Maude Bouscambert-Duchamp; Raphaelle Lamy; Solenne Brun
EPI_ISL_508966, EPI_ISL_508975, EPI_ISL_508998, EPI_ISL_636478, EPI_ISL_636480, EPI_ISL_636482 to 636483, EPI_ISL_636486			
see above	Centre Hospitalier Pierre Oudot	CNR Virus des Infections Respiratoires - France SUD	Alexandre Gaymard; Antonin Bal; Bruno Lina; Carine Moustaud; Florence Morfin-Sherpa; Gregory Destras; Gwendolyne Burfin; Hadrien Règue; Laurence Josset; Martine Valette; Maude Bouscambert-Duchamp; Raphaelle Lamy; Solenne Brun
EPI_ISL_414633	Centre Hospitalier René Dubois Laboratoire de Microbiologie - Bât A	National Reference Center for Viruses of Respiratory Infections, Institut Pasteur, Paris	Angela Brisebarre; Flora Donati Vincent Enouf; Marion Barbet; Maud Vanpeene; Méline Bizard; Mélnie Albert; Pascale Martres; Sylvie Behillil; Sylvie van der Werf
EPI_ISL_414625	Centre Hospitalier Régional Universitaire de Nantes Laboratoire de Virologie	National Reference Center for Viruses of Respiratory Infections, Institut Pasteur, Paris	Angela Brisebarre; Flora Donati Vincent Enouf; Marianne Coste-Burel; Marion Barbet; Maud Vanpeene; Méline Bizard; Mélnie Albert; Sylvie Behillil; Sylvie van der Werf
EPI_ISL_535787 to 535788, EPI_ISL_535790, EPI_ISL_535842 to 535844, EPI_ISL_535870 to 535871, EPI_ISL_535894 to 535896, EPI_ISL_535907, EPI_ISL_535931 to 535935, EPI_ISL_535955, EPI_ISL_536002, EPI_ISL_536005 to 536006, EPI_ISL_536009, EPI_ISL_536012, EPI_ISL_536014 to 536016, EPI_ISL_536047 to 536048, EPI_ISL_536050, EPI_ISL_536052 to 536054, EPI_ISL_536059 to 536060, EPI_ISL_536063, EPI_ISL_536073 to 536074, EPI_ISL_536076, EPI_ISL_536079, EPI_ISL_536119 to 536120, EPI_ISL_536123, EPI_ISL_536126, EPI_ISL_536129 to 536130, EPI_ISL_536133 to 536134, EPI_ISL_536138, EPI_ISL_536151, EPI_ISL_536153 to 536154, EPI_ISL_536381, EPI_ISL_560415, EPI_ISL_1241280			
see above	Centre Hospitalier Régional de Lanaudière	Laboratoire de santé publique du Québec	Guillaume Bourque; Ioannis Ragoussis; Jesse Shapiro; Mark Lathrop and Michel Roger; Mark Lathrop and Michel Roger on behalf of the CoVSeQ research group; Sandrine Moreira
EPI_ISL_418418 to 418419, EPI_ISL_420617, EPI_ISL_508879 to 508880, EPI_ISL_508959 to 508960, EPI_ISL_639978	Centre Hospitalier Saint Joseph Saint Luc	CNR Virus des Infections Respiratoires - France SUD	Alexandre Gaymard; Antonin Bal; Bruno Lina; Carine Moustaud; Florence Morfin-Sherpa; Gregory Destras; Gwendolyne Burfin; Hadrien Règue; Laurence Josset; Martine Valette; Maude Bouscambert-Duchamp; Raphaelle Lamy; Solenne Brun

EPI_ISL_1250601, EPI_ISL_1250603 to 1250608, EPI_ISL_1250610 to 1250612, EPI_ISL_1250614 to 1250622, EPI_ISL_1250625, EPI_ISL_1250630 to 1250632, EPI_ISL_1250634, EPI_ISL_1250636 to 1250644

see above	Centre Hospitalier Universitaire de Rouen Laboratoire de Virologie	Centre Hospitalier Universitaire de Rouen Laboratoire de Virologie	Alice Moisan; Fabienne De Oliveira; Marie Leoz

EPI_ISL_416757, EPI_ISL_417340, EPI_ISL_418426, EPI_ISL_419183, EPI_ISL_419185 to 419186, EPI_ISL_420620, EPI_ISL_508938, EPI_ISL_508944, EPI_ISL_508968, EPI_ISL_525539, EPI_ISL_582121, EPI_ISL_636481, EPI_ISL_639984

see above	Centre Hospitalier de Bourg en Bresse	CNR Virus des Infections Respiratoires - France SUD	Alexandre; Alexandre Gaymard; Antonin; Antonin Bal; Bal; Bouscambert-Duchamp; Brengel-Pesce; Bruno Lina; Bruno.; Carine Moustaud; Cheynet; Destras; Florence; Florence Morfin-Sherpa; Gaymard; Gregory; Gregory Destras; Gwendolyne Burfin; Hadrien Règue; Josset; Karen; Laurence; Laurence Josset; Lina; Martine; Martine Valette; Maude; Maude Bouscambert-Duchamp; Morfin-Sherpa; Raphaelle Lamy; Solenne Brun; Valette; Valérie
EPI_ISL_912612 to 912613	Centre Hospitalier de Compiègne Laboratoire Compiègne	National Reference Center for Viruses of Respiratory Infections, Institut Pasteur, Paris	Angela Brisebarre; Camille Capel; Etienne Simon-Lorière; Marion Barbet; Maud Vanpeene; Méline Bizard; Romand Sylvie; Sylvie Behillil; Sylvie van der Werf; Vincent Enouf
EPI_ISL_912557 to 912558	Centre Hospitalier de Gisors Route de Rouen	National Reference Center for Viruses of Respiratory Infections, Institut Pasteur, Paris	Angela Brisebarre; Camille Capel; Etienne Simon-Lorière; Marion Barbet; Maud Vanpeene; Méline Bizard; Sylvie Behillil; Sylvie van der Werf; Vincent Enouf
EPI_ISL_900530, EPI_ISL_900573	Centre Hospitalier de Guéret	CNR Virus des Infections Respiratoires - France SUD	Antonin Bal; Bruno Lina; Gregory Destras; Gwendolyne Burfin; Hadrien Règue; Laurence Josset; Martine Valette; Quentin Semanas; Sylvie Larrat

EPI_ISL_417338, EPI_ISL_418413, EPI_ISL_419174 to 419176, EPI_ISL_419187 to 419188, EPI_ISL_420612 to 420614, EPI_ISL_508875 to 508876, EPI_ISL_508941, EPI_ISL_508943, EPI_ISL_508946, EPI_ISL_508949 to 508950, EPI_ISL_508952, EPI_ISL_509005

see above	Centre Hospitalier de Macon	CNR Virus des Infections Respiratoires - France SUD	Alexandre Gaymard; Antonin Bal; Bruno Lina; Carine Moustaud; Florence Morfin-Sherpa; Gregory Destras; Gwendolyne Burfin; Laurence Josset; Martine Valette; Maude Bouscambert-Duchamp; Raphaelle Lamy; Solenne Brun
EPI_ISL_416749, EPI_ISL_418414 to 418415, EPI_ISL_418417, EPI_ISL_419168, EPI_ISL_508881	Centre Hospitalier de Valence	CNR Virus des Infections Respiratoires - France SUD	Alexandre; Alexandre Gaymard; Antonin; Antonin Bal; Bal; Bouscambert-Duchamp; Brengel-Pesce; Bruno Lina; Bruno.; Carine Moustaud; Cheynet; Destras; Florence; Florence Morfin-Sherpa; Gaymard; Gregory; Gregory Destras; Gwendolyne Burfin; Josset; Karen; Laurence; Laurence Josset; Lina; Martine; Martine Valette; Maude; Maude Bouscambert-Duchamp; Morfin-Sherpa; Raphaelle Lamy; Solenne Brun; Valette; Valérie
EPI_ISL_508932, EPI_ISL_508978, EPI_ISL_509006, EPI_ISL_509015	Centre Hospitalier de Villefranche	CNR Virus des Infections Respiratoires - France SUD	Alexandre Gaymard; Antonin Bal; Bruno Lina; Carine Moustaud; Florence Morfin-Sherpa; Gregory Destras; Gwendolyne Burfin; Laurence Josset; Martine Valette; Maude Bouscambert-Duchamp; Raphaelle Lamy; Solenne Brun
EPI_ISL_1079689 to 1079690, EPI_ISL_1079692 to 1079693, EPI_ISL_1079695 to 1079697, EPI_ISL_1079699 to 1079701, EPI_ISL_1079703, EPI_ISL_1081842	Centre Hospitalier de Wallonie Picarde (CHWaPi)	Institut de Pathologie et Génétique (IPG)	Jérémie Gras; Pascale Hilbert
EPI_ISL_1007746, EPI_ISL_1007810, EPI_ISL_1007860	Centre Hospitalier de Wallonie picarde (CHwapi)	Institut de Pathologie et Génétique (IPG)	Hilbert Pascale; Jeremie Gras; Jérémie Gras; Pascale Hilbert
EPI_ISL_1165834, EPI_ISL_1165846 to 1165849, EPI_ISL_1165852, EPI_ISL_1165854 to 1165857	Centre Hospitalier de l'Ardenne	GIGA Medical Genomics	Bouchra Boujemla; Cécile Meex; Keith Durkin; Maria Artesi; Marie-Pierre Hayette; Nathalie Renotte; Pierrette Melin; Raphaël Boreux; Sébastien Bontems; Vincent Bours
EPI_ISL_1250627 to 1250629	Centre Hospitalier de l'Austreberthe	Centre Hospitalier Universitaire de Rouen Laboratoire de Virologie	Alice Moisan; Fabienne De Oliveira; Marie Leoz
EPI_ISL_418412	Centre Hospitalier des Vals d'Ardeche	CNR Virus des Infections Respiratoires - France SUD	Alexandre Gaymard; Antonin Bal; Bruno Lina; Carine Moustaud; Florence Morfin-Sherpa; Gregory Destras; Gwendolyne Burfin; Laurence Josset; Martine Valette; Maude Bouscambert-Duchamp; Raphaelle Lamy; Solenne Brun
EPI_ISL_509016	Centre Hospitalier du Haut-Bugey	CNR Virus des Infections Respiratoires - France SUD	Alexandre Gaymard; Antonin Bal; Bruno Lina; Carine Moustaud; Florence Morfin-Sherpa; Gregory Destras; Gwendolyne Burfin; Laurence Josset; Martine Valette; Maude Bouscambert-Duchamp; Raphaelle Lamy; Solenne Brun
EPI_ISL_1299491 to 1299496, EPI_ISL_1299499	Centre Hospitalo Universitaire Souro Sanou	Centre Muraz	Abdoul-Salam Ouedraogo; Ange Badjo; Armel Poda; Arsène Somé; Arsène Zongo; Essia Belarbi; Fabian Leendertz; Firmin Kaboré; Grit Schubert; Jasmin Schlotterbeck; Soumeya Ouangraoua; Thérèse Kagone; Yacouba Sawadogo
EPI_ISL_779847	Centre Hospotalier Cannes	CNR Virus des Infections Respiratoires - France SUD	Antonin Bal; Bruno Lina; Gregory Destras; Gwendolyne Burfin; Hadrien Règue; Laurence Josset; Martine Valette; Quentin Semanas
EPI_ISL_1015368, EPI_ISL_1015371, EPI_ISL_1015373 to 1015375, EPI_ISL_1015389, EPI_ISL_1015392 to 1015400	Centre Marine La Villeneuve	IRBA, 2MI	BRAESCU J.; GORGE O.; NOLENT F.; SARILAR V.; VERMEULENE L.
EPI_ISL_660442	Centre Muraz	Project group Epidemology of Highly Pathogenic Microorganisms, Robert Koch Institut	Abdoul-Salam Ouedraogo; Arsène Zongo; Essia Belarbi; Fabian Leendertz; Grit Schubert; Soumeya Ouangraoua; Yacouba Sawadogo
EPI_ISL_512873	Centre Pasteur of Cameroun	Virology Service, Centre Pasteur of Cameroun	Richard Njouom and Serge Alain SADEUH-Mba
EPI_ISL_482820	Centre de Recerca en Sanitat Animal (IRTA-CReSA)	IrsiCaixa AIDS Research Lab	A. Valencia; B. Clotet; C. Avila-Nieto; E. Vidal; G. Cantero; I. Blanco; J. Blanco; J. Carrillo; J. Rodon; J. Segalés; J. Vergara-Alert; M. Noguera-Julián; M. Parera; M. Puig; M.T. Terrón; N. Izquierdo-Useros; S. Cruz; V. Guallar
EPI_ISL_788933 to 788938	Centre de Recherche et de Formation en Infectiologie Guinée	TransVIHMI, IRD/INSERM/Monpellier University	Abdoul Karim SOUMAH; Abdoulaye TOURE; Ahidjo AYOUBA; Alimou CAMARA; Alpha Kabinet KEITA; Bouna; Bouna Yatassaye; Christelle BUTEL; Eric DELAPORTE; Jean-louis MONEMOU; Joel KOIVOGUI; Kaba KOUROUMA; Laetitia SERRANO; Mamadou Bhoye KEITA; Mamadou Saliou BAH; Mamadou Saliou SOW; Mandiou DIAKITE; Martine PEETERS; Moriba POVOGUI; Penda Malhado DIALLO; Sakoba KEITA
EPI_ISL_539573 to 539576	Centre de Recherches Medicales de Lambarene (CERMEL)	Department of Emerging Infectious Diseases, Institute of Tropical Medicine, Nagasaki University	Akim A. Adegnika; Bertrand Lell; Haruka Abe; Jiro Yasuda; Rodrigue Bikangui; Yuri Ushijima
EPI_ISL_535845, EPI_ISL_535939 to 535940, EPI_ISL_560413 to 560414	Centre de SSS D'Arthabaska-et-de-l'érable - Hôtel-Dieu	Laboratoire de santé publique du Québec	Guillaume Bourque; Ioannis Ragoussis; Jesse Shapiro; Mark Lathrop and Michel Roger; Mark Lathrop and Michel Roger on behalf of the CoVSeQ research group; Sandrine Moreira
EPI_ISL_535724, EPI_ISL_535742 to 535743	Centre de SSS La Pommeraie	Laboratoire de santé publique du Québec	Guillaume Bourque; Ioannis Ragoussis; Jesse Shapiro; Mark Lathrop and Michel Roger; Mark Lathrop and Michel Roger on behalf of the CoVSeQ research group; Sandrine Moreira

EPI_ISL_535722, EPI_ISL_535747 to 535748, EPI_ISL_535849, EPI_ISL_535916, EPI_ISL_535976 to 535977, EPI_ISL_535979, EPI_ISL_536007, EPI_ISL_536031, EPI_ISL_536034 to 536036, EPI_ISL_536038

see above	Centre de SSS de Trois-Rivières	Laboratoire de santé publique du Québec	Guillaume Bourque; Ioannis Ragoussis; Jesse Shapiro; Mark Lathrop and Michel Roger; Mark Lathrop and Michel Roger on behalf of the CoVSeQ research group; Sandrine Moreira
EPI_ISL_535727, EPI_ISL_535776 to 535780, EPI_ISL_535803 to 535805, EPI_ISL_535816, EPI_ISL_535854 to 535855	Centre de SSS de la Haute-Yamaska	Laboratoire de santé publique du Québec	Guillaume Bourque; Ioannis Ragoussis; Jesse Shapiro; Mark Lathrop and Michel Roger; Mark Lathrop and Michel Roger on behalf of the CoVSeQ research group; Sandrine Moreira
EPI_ISL_978534 to 978552	Centre de Virologie des Maladies infectueuses Tropicales	Functional Genomic Platform UATRS-biology, CNRST	; Abdelilah LARAQUI; Abderrazzak Rfaki; Elmostafa BENAISSA; Elmostafa EL FAHIME; Elmostafa EL FAHIME.; Farida HILALI; Hemlali Mouhssine; Khalid ENNIBI; Khalid ENNIBI.; Marouane MELLOUL; Mly Abdelaziz ELALAOUI; Mostafa ELOUENNASS; Nadia Touil; Safaa GHOULAM; Safaa Ghoulam; Sanaa ALAOUI-Amine; Taha Chouati; Tahar BAJJOU; Yassine SEKHSOK; Yassine SEKHSOKH
EPI_ISL_471456 to 471460	Centre de Virologie des Maladies Tropicales	Functional Genomic Platform/Service Analyses Biologique/UATRS/ Centre National Pour la Recherche Scientifique Et Technique (CNRST)	Abdelillah LARAQUI; Abdelkader LAATIRIS; Ahmed REGGAD; Elmostafa EL FAHIME; Farida HILALI; Hicham EL ANNAZ; Idriss-Amine LAHLOU; Khalid ENNIBI; Marouane MELLOUL; Mly Abdelaziz ELALAOUI; Mostafa ELOUENNASS; Nadia TOUIL; Rachid ABI; Rida TAGAJDID; Safae ELKOCHRI; Sanaa ALAOUI-Amine; Tahar BAJJOU; Yassine SEKHSOKH; Youssef AKHOUAD; Zohour KASMY
EPI_ISL_535808	Centre de santé Chibougamau	Laboratoire de santé publique du Québec	Guillaume Bourque; Ioannis Ragoussis; Jesse Shapiro; Mark Lathrop and Michel Roger; Sandrine Moreira
EPI_ISL_443310	Centre de santé Filieris	National Reference Center for Viruses of Respiratory	Angela Brisebarre; Etienne Simon-Lorière; Flora Donati; Marion Barbet; Maud Vanpeene; Mélanie Albert; Méline Bizard; Sylvie Behillil; Sylvie van der

		Infections, Institut Pasteur, Paris	Werf; Vincent Enouf
EPI_ISL_465697, EPI_ISL_536353	Centre de santé Inuulitsivik	Laboratoire de santé publique du Québec	Guillaume Bourque; Ioannis Ragoussis; Jesse Shapiro; Mark Lathrop and Michel Roger on behalf of the CoVSeQ research group; Sandrine Moreira
EPI_ISL_483916 to 484217	Centre for Clinical Infection and Diagnostics Research and Genomics Innovation Unit, Guy's and St. Thomas' NHS Trust	COVID-19 Genomics UK (COG-UK) Consortium	Ali Raza Awan; Chloe Fisher; Jonathan Edgeworth; Luke Snell; Penny Cliff; Rahul Batra
EPI_ISL_560649 to 560740, EPI_ISL_561363 to 561369, EPI_ISL_561373 to 561374	Centre for Clinical Infection and Diagnostics Research and Genomics Innovation Unit, Guy's and St. Thomas' NHS Trust	Centre for Clinical Infection and Diagnostics Research and Genomics Innovation Unit, Guy's and St. Thomas' NHS Trust	Ali Raza Awan; Chloe Fisher; Jonathan Edgeworth; Luke Snell; Rahul Batra
EPI_ISL_428670 to 428673, EPI_ISL_525476, EPI_ISL_525478 to 525479, EPI_ISL_525481, EPI_ISL_525486, EPI_ISL_525488 to 525489	Centre for Dengue Research	Centre for Dengue Research	Ananda Wijewickrama; Chandima Jeewandara; Damayanthi Idampitiya; Damayanthi Tdampitiya; Deshni Jayathilaka; Dinuka Ariyaratne; Dinuka Ariyatane; Dinuka Guruge; Diyanath Ranasinghe; Eranga Narangoda; Gathsaurie Neelika Malavige; Laksiri Gomes; Malika Karunaratne; Neelika Malaige; Neelika Malavige; Ruwan Wijayamuni
EPI_ISL_525474	Centre for Dengue Research	Centre for Dengue Research, USJ, SL	Chandima Jeewandara; Deshni Jayathilaka; Dinuka Ariyaratne; Dinuka Guruge; Diyanath Ranasinghe; Gathsaurie Neelika Malavige; Laksiri Gomes; Ruwan Wijayamuni
EPI_ISL_792547 to 792556, EPI_ISL_862716 to 862721, EPI_ISL_862724 to 862727, EPI_ISL_872394, EPI_ISL_872478, EPI_ISL_872567 to 872568, EPI_ISL_1233054 to 1233138, EPI_ISL_1239361 to 1239368			
see above	Centre for Dengue Research and AICBU, Department of Immunology and Molecular Medicine	Centre for Dengue Research and AICBU, Department of Immunology and Molecular Medicine	Chandima Jeewandara; Deshni Jayathilaka; Dinuka Ariyaratne; Diyanath Ranasinghe; Gathsaurie Neelika Malavige; Laksiri Gomes; Tibutius Thanesh Pramanayagam
EPI_ISL_602564	Centre for Dengue Research, Department of Immunology and Molecular Medicine	Centre for Dengue Research	Ananda Wijewickrama; Chandima Jeewandara; Deshni Jayathilaka; Dinuka Ariyaratne; Diyanath Ranasinghe; Gathsaurie Neelika Malavige; Laksiri Gomes; Malika Karunaratne
EPI_ISL_602566 to 602576, EPI_ISL_668447 to 668455	Centre for Dengue Research, Department of Immunology and Molecular Medicine	Centre for Dengue Research, Department of Immunology and Molecular Medicine	Ananda Wijewickrama; Chandima Jeewandara; Deshni Jayathilaka; Dinuka Ariyaratne; Dinuka Guruge; Diyanath Ranasinghe; Gathsaurie Neelika Malavige; Laksiri Gomes; Malika Karunaratne; Ruwan Wiajayamuni
EPI_ISL_602565	Centre for Dengue Research, Department of Immunology and Molecular Medicine	Centre for Dengue Research, Department of Immunology and Molecular Medicine,	Chandima Jeewandara; Deshni Jayathilaka; Dinuka Ariyaratne; Dinuka Guruge; Diyanath Ranasinghe; Gathsaurie Neelika Malavige; Laksiri Gomes; Ruwan Wiajayamuni
EPI_ISL_453666 to 453695, EPI_ISL_461970 to 461998, EPI_ISL_475239 to 475341, EPI_ISL_479175 to 479194, EPI_ISL_484407 to 484432, EPI_ISL_493614 to 493665, EPI_ISL_499270 to 499329, EPI_ISL_507131 to 507159, EPI_ISL_512384 to 512435, EPI_ISL_514455 to 514513, EPI_ISL_517510 to 517530, EPI_ISL_517532 to 517542, EPI_ISL_526433 to 526436, EPI_ISL_529272, EPI_ISL_529303, EPI_ISL_529341 to 529352, EPI_ISL_529373 to 529377, EPI_ISL_529431 to 529432, EPI_ISL_529465, EPI_ISL_529481, EPI_ISL_529491, EPI_ISL_529511, EPI_ISL_529525, EPI_ISL_529668 to 529674, EPI_ISL_535024 to 535042, EPI_ISL_549341, EPI_ISL_549343, EPI_ISL_549360, EPI_ISL_549363 to 549364, EPI_ISL_549366, EPI_ISL_549460 to 549476, EPI_ISL_559983, EPI_ISL_572715 to 572716, EPI_ISL_572721, EPI_ISL_573253 to 573265, EPI_ISL_573870, EPI_ISL_577040 to 577067, EPI_ISL_577193 to 577219, EPI_ISL_577299 to 577345, EPI_ISL_577381 to 577382, EPI_ISL_577385 to 577400, EPI_ISL_577545 to 577546, EPI_ISL_584168 to 584187, EPI_ISL_584193 to 584195, EPI_ISL_584200 to 584206, EPI_ISL_584212 to 584216, EPI_ISL_584809 to 584810, EPI_ISL_595100 to 595114, EPI_ISL_611526, EPI_ISL_611532, EPI_ISL_611535, EPI_ISL_611537 to 611538, EPI_ISL_611555, EPI_ISL_611557, EPI_ISL_611585, EPI_ISL_611589 to 611590, EPI_ISL_611592, EPI_ISL_611638 to 611639, EPI_ISL_611689, EPI_ISL_611754 to 611755, EPI_ISL_611761 to 611763, EPI_ISL_611771 to 611773, EPI_ISL_611841, EPI_ISL_611916, EPI_ISL_611925, EPI_ISL_611927 to 611928, EPI_ISL_611933 to 611935, EPI_ISL_611957 to 611962, EPI_ISL_611971, EPI_ISL_611983, EPI_ISL_611998, EPI_ISL_612004, EPI_ISL_612028, EPI_ISL_612068 to 612069, EPI_ISL_612082, EPI_ISL_612086, EPI_ISL_612089, EPI_ISL_612092, EPI_ISL_612094, EPI_ISL_613282 to 613306, EPI_ISL_626677 to 626678, EPI_ISL_626684, EPI_ISL_626689 to 626690, EPI_ISL_626738, EPI_ISL_626778, EPI_ISL_626792, EPI_ISL_626878 to 626880, EPI_ISL_626895, EPI_ISL_626936, EPI_ISL_627056 to 627057, EPI_ISL_627116, EPI_ISL_627128, EPI_ISL_627144, EPI_ISL_627150, EPI_ISL_628312 to 628333, EPI_ISL_637278 to 637279, EPI_ISL_637305, EPI_ISL_637369 to 637372, EPI_ISL_637387, EPI_ISL_637402, EPI_ISL_637411, EPI_ISL_637432, EPI_ISL_637453, EPI_ISL_637459, EPI_ISL_637466 to 637467, EPI_ISL_637491 to 637499, EPI_ISL_637502, EPI_ISL_637528, EPI_ISL_637610, EPI_ISL_637659, EPI_ISL_637674, EPI_ISL_637714 to 637715, EPI_ISL_637717 to 637720, EPI_ISL_637780 to 637783, EPI_ISL_637853, EPI_ISL_637889, EPI_ISL_637892, EPI_ISL_637920 to 637921, EPI_ISL_637931 to 637932, EPI_ISL_637949, EPI_ISL_637951, EPI_ISL_637986, EPI_ISL_638002, EPI_ISL_638006, EPI_ISL_638026, EPI_ISL_638050, EPI_ISL_638065, EPI_ISL_638134 to 638137, EPI_ISL_638249 to 638257, EPI_ISL_638265 to 638292, EPI_ISL_638454 to 638458, EPI_ISL_638911 to 638913, EPI_ISL_639006 to 639007, EPI_ISL_650168 to 650169, EPI_ISL_650221, EPI_ISL_650248 to 650249, EPI_ISL_650379, EPI_ISL_650388, EPI_ISL_650401, EPI_ISL_650566 to 650571, EPI_ISL_650575 to 650579, EPI_ISL_650581, EPI_ISL_650584 to 650585, EPI_ISL_650600 to 650605, EPI_ISL_650608, EPI_ISL_650711, EPI_ISL_650941, EPI_ISL_650947, EPI_ISL_650949, EPI_ISL_650956 to 650957, EPI_ISL_650964 to 650966, EPI_ISL_650980 to 650982, EPI_ISL_651066 to 651070, EPI_ISL_651099, EPI_ISL_651187, EPI_ISL_651277, EPI_ISL_652103 to 652110, EPI_ISL_652620 to 652626, EPI_ISL_664137, EPI_ISL_664191, EPI_ISL_664302, EPI_ISL_664374 to 664377, EPI_ISL_664492, EPI_ISL_664526, EPI_ISL_664556 to 664559, EPI_ISL_664679, EPI_ISL_664690, EPI_ISL_664746, EPI_ISL_664763, EPI_ISL_664767, EPI_ISL_664840 to 664855, EPI_ISL_665267, EPI_ISL_665274, EPI_ISL_665301, EPI_ISL_665304, EPI_ISL_665330, EPI_ISL_665338, EPI_ISL_665341, EPI_ISL_665347 to 665348, EPI_ISL_665383, EPI_ISL_665391, EPI_ISL_665418, EPI_ISL_665434, EPI_ISL_665471 to 665472, EPI_ISL_665476 to 665480, EPI_ISL_665482 to 665488, EPI_ISL_665614 to 665616, EPI_ISL_665624 to 665625, EPI_ISL_665652, EPI_ISL_665698, EPI_ISL_665723, EPI_ISL_665726, EPI_ISL_665745, EPI_ISL_665849, EPI_ISL_665946 to 665955, EPI_ISL_666107 to 666109, EPI_ISL_678831, EPI_ISL_679969 to 679985, EPI_ISL_680016 to 680036, EPI_ISL_680562 to 680570, EPI_ISL_680578, EPI_ISL_702560, EPI_ISL_702585, EPI_ISL_702736, EPI_ISL_702744, EPI_ISL_702795, EPI_ISL_702837, EPI_ISL_702851, EPI_ISL_702858, EPI_ISL_702870, EPI_ISL_702915, EPI_ISL_702985, EPI_ISL_702987, EPI_ISL_703015, EPI_ISL_703053, EPI_ISL_703071, EPI_ISL_703082, EPI_ISL_703152, EPI_ISL_703267, EPI_ISL_703327, EPI_ISL_703338, EPI_ISL_703419, EPI_ISL_703446, EPI_ISL_703454, EPI_ISL_703489, EPI_ISL_703492, EPI_ISL_703495, EPI_ISL_703497, EPI_ISL_703500, EPI_ISL_703502, EPI_ISL_703548, EPI_ISL_703551, EPI_ISL_703554, EPI_ISL_703556, EPI_ISL_703559, EPI_ISL_703562, EPI_ISL_703564, EPI_ISL_703567, EPI_ISL_703666, EPI_ISL_703668, EPI_ISL_703682, EPI_ISL_703687, EPI_ISL_703772, EPI_ISL_703797, EPI_ISL_704243, EPI_ISL_704266, EPI_ISL_704322, EPI_ISL_704325, EPI_ISL_704585 to 704593, EPI_ISL_704783, EPI_ISL_705187, EPI_ISL_705234, EPI_ISL_705249, EPI_ISL_705294, EPI_ISL_705325, EPI_ISL_705369 to 705370, EPI_ISL_705379, EPI_ISL_706864 to 706890, EPI_ISL_723521 to 723557, EPI_ISL_727697 to 727806, EPI_ISL_741348, EPI_ISL_741357 to 741358, EPI_ISL_741658 to 741759, EPI_ISL_741772, EPI_ISL_742260, EPI_ISL_763396, EPI_ISL_763407, EPI_ISL_763421, EPI_ISL_763425, EPI_ISL_763430, EPI_ISL_763451, EPI_ISL_763465, EPI_ISL_763471, EPI_ISL_763476 to 763477, EPI_ISL_763495, EPI_ISL_763498, EPI_ISL_763503, EPI_ISL_763505, EPI_ISL_763512 to 763513, EPI_ISL_763515 to 763517, EPI_ISL_763521, EPI_ISL_763523, EPI_ISL_763541, EPI_ISL_763550, EPI_ISL_763586, EPI_ISL_763595, EPI_ISL_763598, EPI_ISL_763600 to 763627, EPI_ISL_763659 to 763661, EPI_ISL_763672, EPI_ISL_763676, EPI_ISL_763685, EPI_ISL_763690 to 763691, EPI_ISL_763693, EPI_ISL_763734, EPI_ISL_763755, EPI_ISL_763789, EPI_ISL_763791, EPI_ISL_763798 to 763800, EPI_ISL_763803 to 763808, EPI_ISL_763812, EPI_ISL_763823 to 763825, EPI_ISL_763827 to 763837, EPI_ISL_763865, EPI_ISL_764006 to 764031, EPI_ISL_764036, EPI_ISL_764081 to 764082, EPI_ISL_764094 to 764098, EPI_ISL_764101 to 764102, EPI_ISL_764118 to 764125, EPI_ISL_764136, EPI_ISL_764140, EPI_ISL_764152, EPI_ISL_764169, EPI_ISL_764172, EPI_ISL_764193, EPI_ISL_765112 to 765148, EPI_ISL_816137 to 816225, EPI_ISL_819364 to 819365, EPI_ISL_819367 to 819369, EPI_ISL_819371 to 819374, EPI_ISL_819379, EPI_ISL_819381 to 819383, EPI_ISL_819422 to 819423, EPI_ISL_819426, EPI_ISL_819432, EPI_ISL_819447, EPI_ISL_841755 to 841775, EPI_ISL_841777 to 841778, EPI_ISL_841780 to 841806, EPI_ISL_841808 to 841819, EPI_ISL_841821 to 841848, EPI_ISL_841850, EPI_ISL_841852 to 841864, EPI_ISL_841866 to 841872, EPI_ISL_841874 to 841876, EPI_ISL_841878 to 841879, EPI_ISL_841883 to 841924, EPI_ISL_841926 to 841943, EPI_ISL_841945 to 841956, EPI_ISL_841958 to 841959, EPI_ISL_841962 to 841967, EPI_ISL_841969 to 841970, EPI_ISL_841972 to 841976, EPI_ISL_841978 to 841981, EPI_ISL_841983 to 842008, EPI_ISL_842010 to 842099, EPI_ISL_867930 to 867981, EPI_ISL_867990, EPI_ISL_867993, EPI_ISL_867999, EPI_ISL_868001, EPI_ISL_868008, EPI_ISL_868023, EPI_ISL_868034, EPI_ISL_868102, EPI_ISL_868141 to 868148, EPI_ISL_868150, EPI_ISL_868152 to 868201, EPI_ISL_868203 to 868210, EPI_ISL_868212 to 868222, EPI_ISL_868224 to 868226, EPI_ISL_868228 to 868238, EPI_ISL_868240 to 868250, EPI_ISL_868252 to 868257, EPI_ISL_868309 to 868356, EPI_ISL_923212 to 923224, EPI_ISL_923226 to 923240, EPI_ISL_923242 to 923251, EPI_ISL_923256 to 923271, EPI_ISL_923273 to 923294, EPI_ISL_923296 to 923333, EPI_ISL_923335 to 923357, EPI_ISL_923359 to 923388, EPI_ISL_923390, EPI_ISL_923392 to 923399, EPI_ISL_923401 to 923424, EPI_ISL_923430, EPI_ISL_923436, EPI_ISL_923438, EPI_ISL_923443, EPI_ISL_923539, EPI_ISL_923577, EPI_ISL_923580, EPI_ISL_923589 to 923598, EPI_ISL_923605, EPI_ISL_923611, EPI_ISL_923613, EPI_ISL_923628, EPI_ISL_923660, EPI_ISL_923663 to 923683, EPI_ISL_952377 to 952448, EPI_ISL_952450 to 952458, EPI_ISL_952460 to 952485, EPI_ISL_952489 to 952490, EPI_ISL_952492 to 952499, EPI_ISL_952521 to 952526, EPI_ISL_952528 to 952532, EPI_ISL_952534 to 952547, EPI_ISL_952550, EPI_ISL_952553 to 952559, EPI_ISL_952561, EPI_ISL_952563, EPI_ISL_952565 to 952576, EPI_ISL_952578, EPI_ISL_952580 to 952592, EPI_ISL_952594 to 952595, EPI_ISL_952597 to 952602, EPI_ISL_952624, EPI_ISL_952673, EPI_ISL_952675, EPI_ISL_952677, EPI_ISL_952680, EPI_ISL_952689, EPI_ISL_952777 to 952815, EPI_ISL_952817 to 952842, EPI_ISL_952844 to 952855, EPI_ISL_952857, EPI_ISL_1000187 to 1000189, EPI_ISL_1000192 to 1000198, EPI_ISL_1000200 to 1000240, EPI_ISL_1000242 to 1000251, EPI_ISL_1000253 to 1000254, EPI_ISL_1000256 to 1000265, EPI_ISL_1000267 to 1000278, EPI_ISL_1000280 to 1000282, EPI_ISL_1000288, EPI_ISL_1000292 to 1000294, EPI_ISL_1000301 to 1000302, EPI_ISL_1000304 to 1000308, EPI_ISL_1000311 to 1000312, EPI_ISL_1000314, EPI_ISL_1000316, EPI_ISL_1000318 to 1000319, EPI_ISL_1000321 to 1000322, EPI_ISL_1000324, EPI_ISL_1000404 to 1000418, EPI_ISL_1000420 to 1000439, EPI_ISL_1000441 to 1000442, EPI_ISL_1000444 to 1000445, EPI_ISL_1000447 to 1000453, EPI_ISL_1000455 to 1000460, EPI_ISL_1000466 to 1000471, EPI_ISL_1000473 to 1000476, EPI_ISL_1000482 to 1000483, EPI_ISL_1000525, EPI_ISL_1000573, EPI_ISL_1000576, EPI_ISL_1000601 to 1000608, EPI_ISL_1000610, EPI_ISL_1000616 to 1000627, EPI_ISL_1050075 to 1050086, EPI_ISL_1050088 to 1050103, EPI_ISL_1050106 to 1050107, EPI_ISL_1050109 to 1050110, EPI_ISL_1050112, EPI_ISL_1050114 to 1050115, EPI_ISL_1050117, EPI_ISL_1050119, EPI_ISL_1050121 to 1050122, EPI_ISL_1050126 to 1050135, EPI_ISL_1050138 to 1050141, EPI_ISL_1050143 to 1050150, EPI_ISL_1050152 to 1050156, EPI_ISL_1050158, EPI_ISL_1050162 to 1050165, EPI_ISL_1050167, EPI_ISL_1050170 to 1050172, EPI_ISL_1050748 to 1050827, EPI_ISL_1050835 to 1050839, EPI_ISL_1050842 to 1050861, EPI_ISL_1050871 to 1050882, EPI_ISL_1050909 to 1050915, EPI_ISL_1053749 to 1053753, EPI_ISL_1053817 to 1053902, EPI_ISL_1053906 to 1053907, EPI_ISL_1053915 to 1053916, EPI_ISL_1053922 to 1053923, EPI_ISL_1053926 to 1053927, EPI_ISL_1053932 to 1053943, EPI_ISL_1053945, EPI_ISL_1053953 to 1053960, EPI_ISL_1053965, EPI_ISL_1053967 to 1053971, EPI_ISL_1053976, EPI_ISL_1103594 to 1103611, EPI_ISL_1103613, EPI_ISL_1103615, EPI_ISL_1103618, EPI_ISL_1103620, EPI_ISL_1103622, EPI_ISL_1103625, EPI_ISL_1103627 to 1103648, EPI_ISL_1104085, EPI_ISL_1104088, EPI_ISL_1105494 to 1105495, EPI_ISL_1105511 to 1105512, EPI_ISL_1105656, EPI_ISL_1107516 to 1107543, EPI_ISL_1107586 to 1107587, EPI_ISL_1107613, EPI_ISL_1107631 to 1107640, EPI_ISL_1107643 to 1107666, EPI_ISL_1107674 to 1107699, EPI_ISL_1107708, EPI_ISL_1107713 to 1107740, EPI_ISL_1107745 to 1107785, EPI_ISL_1107787 to 1107792, EPI_ISL_1108835, EPI_ISL_1177231 to 1177450, EPI_ISL_1178096 to 1178120, EPI_ISL_1179646 to 1179679, EPI_ISL_1179681, EPI_ISL_1179683, EPI_ISL_1179685, EPI_ISL_1179687, EPI_ISL_1179689, EPI_ISL_1179691 to 1179692, EPI_ISL_1179694 to 1179695, EPI_ISL_1179697, EPI_ISL_1179699 to 1179700, EPI_ISL_1179702, EPI_ISL_1179704 to 1179705, EPI_ISL_1179707, EPI_ISL_1179709, EPI_ISL_1179711 to 1179712, EPI_ISL_1179714, EPI_ISL_1179716 to 1179717, EPI_ISL_1179719, EPI_ISL_1179721 to 1179722, EPI_ISL_1179724, EPI_ISL_1179726, EPI_ISL_1179728 to 1179729, EPI_ISL_1179731, EPI_ISL_1179738 to 1179739, EPI_ISL_1179741, EPI_ISL_1179743 to 1179744, EPI_ISL_1179746, EPI_ISL_1179748, EPI_ISL_1179750 to 1179751, EPI_ISL_1179753, EPI_ISL_1179755 to 1179756, EPI_ISL_1179758, EPI_ISL_1179760, EPI_ISL_1179765, EPI_ISL_1179767, EPI_ISL_1179769, EPI_ISL_1179771, EPI_ISL_1179774, EPI_ISL_1179776, EPI_ISL_1179783, EPI_ISL_1179787, EPI_ISL_1179790, EPI_ISL_1179792, EPI_ISL_1179794, EPI_ISL_1179796, EPI_ISL_1179803, EPI_ISL_1179812, EPI_ISL_1179814, EPI_ISL_1179816, EPI_ISL_1179818 to 1179825, EPI_ISL_1179827 to 1179844, EPI_ISL_1179852 to 1179890, EPI_ISL_1247033 to 1247119, EPI_ISL_1248579 to 1248675, EPI_ISL_1249919, EPI_ISL_1249950, EPI_ISL_1249959, EPI_ISL_1249967 to 1249985, EPI_ISL_1249987, EPI_ISL_1249989, EPI_ISL_1249991 to 1249992, EPI_ISL_1249994 to 1249995, EPI_ISL_1249997 to 1249998, EPI_ISL_1250000 to 1250001, EPI_ISL_1250003 to 1250004, EPI_ISL_1250006 to 1250030, EPI_ISL_1250056, EPI_ISL_1250061, EPI_ISL_1250094 to 1250095, EPI_ISL_1250105, EPI_ISL_1250109 to 1250119, EPI_ISL_1309374 to 1309431, EPI_ISL_1310291 to 1310326, EPI_ISL_1310328 to 1310361, EPI_ISL_1310366 to 1310402, EPI_ISL_1310438 to 1310454			
see above	Centre for Enzyme Innovation, University of Portsmouth / Translational Research Laboratory, Portsmouth Hospitals NHS Trust	COVID-19 Genomics UK (COG-UK) Consortium	; Allyson Lloyd; Angela Beckett; Anoop Chauhan; Christopher Fearn; Ethan Butcher; Garry Scarlett; Kate Cook; Katie Loveson; Kelly Bicknell; Robert Impey; Salman Goudarzi; Samuel Robson; Sarah Wyllie; Scott Elliott; Sharon Glaysher; Yann Bourgeois
EPI_ISL_453665	Centre for Enzyme Innovation, University of Portsmouth / Translational Research Laboratory, Portsmouth Hospitals NHS Trust	University of Portsmouth	; Allyson Lloyd; Angela Beckett; Anoop Chauhan; Ethan Butcher; Garry Scarlett; Kelly Bicknell; Robert Impey; Samuel Robson; Sarah Wyllie; Scott Elliott; Sharon Glaysher; Yann Bourgeois
EPI_ISL_482781, EPI_ISL_733568	Centre for Health Protection	Hong Kong Department of Health	Alan K.L. Tsang; Chan Rickjason C.W.; Cheng Peter K.C.; Dominic N.C. Tsang; Edman T.K. Lam; Lam Edman T.K.; Mak Gannon C.K.; Peter C.W. Yip; Rickjason C.W. Chan; Tsang Dominic N.C.
EPI_ISL_654794	Centre for Human Virology & Genomics, Nigerian Institute of Medical Research	Centre for Human Virology & Genomics, Nigerian Institute of Medical Research	Shaibu, J.
EPI_ISL_413550	Centre for Human and Zoonotic Virology (CHAZVY), College of Medicine University of Lagos/Lagos University Teaching Hospital (LUTH), part of the Laboratory Network of the Nigeria Centre for Disease Control (NCDC)	African Centre of Excellence for Genomics of Infectious Diseases (ACEGID), Redeemer's University, Ede, Osun State, Nigeria	Ajogbasile F.V.; Folarin O.A.; Ihekweazu C. Happi C.T.; Kayode A.; Oguzie J.; Oluniyi P.E.
EPI_ISL_413596, EPI_ISL_413598 to 413600	Centre for Infectious Diseases and Microbiology - Public Health	NSW Health Pathology - Institute of Clinical Pathology and Medical Research; Westmead Hospital; University of Sydney	Carter I; Chen SC; Eden J-S; Gall; Gray K; Holmes EC; Kok J and Dwyer DE for the 2019-nCoV Study Group*; Lam C; M; Maddocks S; O'Sullivan MV; Rahman H; Rockett R; Sintchenko V; Timms; V

EPI_ISL_407893, EPI_ISL_408976, EPI_ISL_412975, EPI_ISL_413213 to 413214, EPI_ISL_413594 to 413595, EPI_ISL_417030	Centre for Infectious Diseases and Microbiology Laboratory Services	NSW Health Pathology - Institute of Clinical Pathology and Medical Research; Westmead Hospital; University of Sydney	; A; Alicia; Carter I; Chen SC; Eden J-S; Gall; Gray K; Holmes EC; Kok J and Dwyer DE for the 2019-nCoV Study Group; Kok J and Dwyer DE for the 2019-nCoV Study Group*; Lam C; M; Maddocks S; O'Sullivan MV; Rahman H; Rockett R; Sadsad R; Sintchenko V; Timms; V
EPI_ISL_417383 to 417412, EPI_ISL_427643 to 427710, EPI_ISL_427712 to 427716, EPI_ISL_427720, EPI_ISL_427722 to 427808	Centre for Infectious Diseases and Microbiology Public Health	NSW Health Pathology - Institute of Clinical Pathology and Medical Research; Westmead Hospital; University of Sydney	Arnott A; Arnott A and Sadsad R for the 2019-nCoV Study Group; Bachmann N; Basile K; Byun R; Carter I; Carter I and Rahman H for the 2019-nCoV Study Group; Chang S; Chen SC; Chen SC and Maddocks S for the 2019-nCoV Study Group; Draper J; Dwyer DE; Dwyer DE and Rockett R for the 2019-nCoV Study Group; Dwyer DE for the 2019-nCoV Study Group; Eden J-S; Eden J-S and Lam C for the 2019-nCoV Study Group; Eden JS; Gall M; Gall M and Arnott A for the 2019-nCoV Study Group; Gray K; Gray K and Timms V for the 2019-nCoV Study Group; Holmes EC; Holmes EC and O'Sullivan MV for the 2019-nCoV Study Group; Kok J; Kok J and Dwyer DE for the 2019-nCoV Study Group; Lam C; Lam C and Gray K for the 2019-nCoV Study Group; Maddocks S; Maddocks S and Kok J for the 2019-nCoV Study Group; O'Sullivan MV; O'Sullivan MV and Sintchenko V for the 2019-nCoV Study Group; Propenko M; Rahman H; Rahman H and Holmes EC for the 2019-nCoV Study Group; Rockett R; Rockett R and Eden J-S for the 2019-nCoV Study Group; Sadsad R; Sadsad R and Carter I for the 2019-nCoV Study Group; Sim E; Sintchenko V; Sintchenko V and Chen SC for the 2019-nCoV Study Group; Sorrell T; Timms; Timms V; Timms V and Gall M for the 2019-nCoV Study Group; V
EPI_ISL_413597	Centre for Infectious Diseases and Microbiology- Public Health	NSW Health Pathology - Institute of Clinical Pathology and Medical Research; Westmead Hospital; University of Sydney	Carter I; Chen SC; Eden J-S; Gall; Gray K; Holmes EC; Kok J and Dwyer DE for the 2019-nCoV Study Group*; Lam C; M; Maddocks S; O'Sullivan MV; Rahman H; Rockett R; Sintchenko V; Timms; V
EPI_ISL_450302 to 450303, EPI_ISL_465688 to 465690, EPI_ISL_535960, EPI_ISL_535980 to 535981, EPI_ISL_536000, EPI_ISL_536004, EPI_ISL_536023, EPI_ISL_536043, EPI_ISL_536045, EPI_ISL_536080, EPI_ISL_536104, EPI_ISL_536141 to 536142, EPI_ISL_536145, EPI_ISL_536155 to 536156, EPI_ISL_536159, EPI_ISL_536162, EPI_ISL_536182 to 536184, EPI_ISL_536211 to 536212, EPI_ISL_536218 to 536221, EPI_ISL_536224, EPI_ISL_536232, EPI_ISL_536240 to 536241, EPI_ISL_536255, EPI_ISL_536283, EPI_ISL_536315, EPI_ISL_536325, EPI_ISL_536329, EPI_ISL_536348, EPI_ISL_536368 to 536370, EPI_ISL_536376 to 536378			
see above	Centre hospitalier Anna-Laberge	Laboratoire de santé publique du Québec	Guillaume Bourque; Ioannis Ragoussis; Jesse Shapiro; Mark Lathrop and Michel Roger; Mark Lathrop and Michel Roger on behalf of the CoVSeQ research group; Sandrine Moreira
EPI_ISL_900512, EPI_ISL_900543	Centre hospitalier Emile Roux	CNR Virus des Infections Respiratoires - France SUD	Antonin Bal; Bruno Lina; Gregory Destras; Gwendolyne Burfin; Hadrien Règue; Laurence Josset; Martine Valette; Quentin Semanas
EPI_ISL_508934 to 508936, EPI_ISL_666682 to 666686, EPI_ISL_693391, EPI_ISL_700372 to 700410	Centre hospitalier Métropole Savoie	CNR Virus des Infections Respiratoires - France SUD	Alexandre Gaymard; Antonin Bal; Bruno Lina; Carine Dumollard; Carine Moustaud; Florence Morfin-Sherpa; Gregory Destras; Gwendolyne Burfin; Hadrien Règue; Jérôme Grosjean; Laurence Josset; Martine Valette; Maude Bouscambert-Duchamp; Quentin Semanas; Raphaelle Lamy; Solenne Brun
EPI_ISL_535746, EPI_ISL_535767 to 535768	Centre hospitalier de St-Mary	Laboratoire de santé publique du Québec	Guillaume Bourque; Ioannis Ragoussis; Jesse Shapiro; Mark Lathrop and Michel Roger; Sandrine Moreira
EPI_ISL_535807, EPI_ISL_535872 to 535873, EPI_ISL_535958 to 535959, EPI_ISL_536115, EPI_ISL_536199, EPI_ISL_536204	Centre hospitalier régional du Grand Portage	Laboratoire de santé publique du Québec	Guillaume Bourque; Ioannis Ragoussis; Jesse Shapiro; Mark Lathrop and Michel Roger; Mark Lathrop and Michel Roger on behalf of the CoVSeQ research group; Sandrine Moreira
EPI_ISL_639739	Centre of Nanotechnologies, INCD IMT-Bucuresti (National Institute for Research and Development in Microtechnologies - Bucharest)	Centre of Nanotechnologies, INCD IMT-Bucuresti (National Institute for Research and Development in Microtechnologies - Bucharest)	Gogianu; L. and Baisan, M.; Salceanu, A.
EPI_ISL_413647, EPI_ISL_417997 to 417999	Centro Hospital do Porto, E.P.E. - H. Geral de Santo Antonio	Instituto Nacional de Saude (INSA)	Guiomar et al; Helena Ramos; Inês Costa; Joana Isidro; Joana Mendonça; João Paulo Gomes; Luís Vieira; Pedro Pechirra; Raquel Guiomar; Vítor Borges
EPI_ISL_1164228 to 1164352	Centro Hospitalar de Entre o Douro e Vouga (CHEDV)	Institute of Biomedicine (IBiMED), Universidade de Aveiro	Gabriela Moura; Miguel Pinheiro and Manuel Santos; Patricia Arinto; Sofia Marques
EPI_ISL_1138761 to 1138827, EPI_ISL_1159127 to 1159128, EPI_ISL_1164184 to 1164227	Centro Hospitalar do Baixo Vouga (CHBV)	Institute of Biomedicine (IBiMED), Universidade de Aveiro	Gabriela Moura; Miguel Pinheiro and Manuel Santos; Patricia Arinto; Sofia Marques
EPI_ISL_417986 to 417987, EPI_ISL_417989	Centro Hospitalar e Universitario de Sao Joao, Porto	Instituto Nacional de Saude (INSA)	Guiomar et al
EPI_ISL_413648	Centro Hospitalar e Universitário de Sao Joao, Porto	Instituto Nacional de Saude (INSA)	Inês Costa; Joana Isidro; Joana Mendonça; João Paulo Gomes; João Tiago Guimarães; Luís Vieira; Pedro Pechirra; Raquel Guiomar; Vítor Borges
EPI_ISL_515547	Centro Medico da Policia Militar do Estado de Sao Paulo	Instituto Adolfo Lutz, Interdiciplinary Procedures Center, Strategic Laboratory	Claudia Regina Gonçalves; Claudio Tavares Sacchi; Erica Valessa Ramos Gomes
EPI_ISL_693248	Centro Municipal de Epidemiologia e Imunizações	Instituto Adolfo Lutz, Interdiciplinary Procedures Center, Strategic Laboratory	Claudia Regina Gonçalves; Claudio Tavares Sacchi; Erica Valessa Ramos Gomes; Karoline Rodrigues Campos
EPI_ISL_693244	Centro Médico da Polícia Militar do Estado de Sao Paulo	Instituto Adolfo Lutz, Interdiciplinary Procedures Center, Strategic Laboratory	Claudia Regina Gonçalves; Claudio Tavares Sacchi; Erica Valessa Ramos Gomes; Karoline Rodrigues Campos
EPI_ISL_512670, EPI_ISL_527742, EPI_ISL_527755	Centro Nacional De Rehabilitacion Humberto Araya Rojas (Cenare)	Inciensa, Instituto Costarricense de Investigación y Enseñanza en Nutrición y Salud	Adriana Godinez & Melany Calderon; Claudio Soto-Garita; Estela Cordero; Francisco Duarte; Hebleen Porras
EPI_ISL_837550, EPI_ISL_837553 to 837578	Centro Nacional de Enfermedades Tropicales (CENETROP)	Laboratory of Respiratory Viruses and Measles, Oswaldo Cruz Institute, FIOCRUZ	Ana Carolina Mendonca; Anna Carolina Paixao; Cinthia Avila; Fernando Motta; Luciana Appolinario; Marilda Siqueira on behalf of the Fiocruz COVID-19 Genomic Surveillance Network; Paola Resende; Roxana Loayza
EPI_ISL_1007893	Centro Nacional de Gripe (Valladolid)	SeqCOVID-SPAIN consortium/IBV(CSIC)	Diana Perez San José and SeqCOVID-SPAIN consortium; Iván Sanz Muñoz
EPI_ISL_629011 to 629028	Centro de Biotecnología Vegetal, Universidad Andrés Bello, Center for Genome Regulation	Center for Mathematical Modeling and Center for Genome Regulation. Santiago, Chile	Allende ML; Arriagada G; Bastias M; Bustos F; Castro E; González M; M; Maass A; Meneses C.; Montecino; Orellana A; Sanhueza D; Travisany D
EPI_ISL_483065, EPI_ISL_509430 to 509435, EPI_ISL_510536, EPI_ISL_529139	Centro de Desenvolvimento Tecnologico em Saude, Fundacao Oswaldo Cruz	Centro de Desenvolvimento Tecnologico em Saude, Fundacao Oswaldo Cruz	A.D.; Barroso; C.Q.; C.Q. and Medeiros; De Paula; F.B.; Ferreira; Fintelman-Rodrigues, N.; Gregorio; J.S.; M.A.; M.L.; Medeiros; Oliveira; S.P.; Sacramento; Saraiva; Souza; T.M.; Tschoeke, D.
EPI_ISL_510541, EPI_ISL_529140	Centro de Desenvolvimento Tecnologico em Saude, Fundacao Oswaldo Cruz	Centro de Desenvolvimento Tecnologico em Saude, Fundacao Oswaldo Cruz	A.D.; C.Q.; C.Q. and Medeiros; De Paula; F.B.; Ferreira; Fintelman-Rodrigues, N.; M.A.; Medeiros; Sacramento; Saraiva; Souza; T.M.
EPI_ISL_1023783 to 1023784, EPI_ISL_1023786, EPI_ISL_1023788, EPI_ISL_1023790, EPI_ISL_1023792, EPI_ISL_1023794, EPI_ISL_1023796, EPI_ISL_1023798, EPI_ISL_1023800 to 1023801, EPI_ISL_1023803, EPI_ISL_1023805, EPI_ISL_1023807, EPI_ISL_1023809, EPI_ISL_1023811 to 1023812, EPI_ISL_1023815 to 1023816, EPI_ISL_1023818, EPI_ISL_1023820, EPI_ISL_1023822, EPI_ISL_1023824, EPI_ISL_1023826 to 1023827, EPI_ISL_1023829, EPI_ISL_1023831, EPI_ISL_1023833, EPI_ISL_1023835, EPI_ISL_1023837, EPI_ISL_1023839, EPI_ISL_1023841, EPI_ISL_1023843, EPI_ISL_1023845			
see above	Centro de Desenvolvimento Tecnológico em Saúde - CDTS	Centro de Desenvolvimento Tecnológico em Saúde - CDTS	A.D.; C.Q.; De Paula; F.B.; Ferreira; Fintelman-Rodrigues, N.; M.A. and Sacramento; Saraiva; Souza; T.M.
EPI_ISL_635479 to 635576	Centro de Diagnstico COVID-19 UABC Tijuana	Andersen lab at Scripps Research	Germán Ibarra; Jonathan Vincent Baena; Jorge Luis Jiménez Niebla; Manuel Sánchez Alavez; Oscar Efrén Zazueta Fierro; SEARCH Alliance San Diego with Idanya Rubí Serafín Higuera
EPI_ISL_1195284	Centro de Especialidades Triunfo	Epiclin	Ana Paula Muterle; Carolina Comerlato; Eliana Márcia Da Ros Wendland; Fernando Hayashi Sant'Anna; Janira Prichula; Juliana Comerlato
EPI_ISL_941104 to 941162	Centro de Investigaciones en Microbiología y Biotecnología-UR (CIMBIUR), Facultad de Ciencias Naturales, Universidad del Rosario, Bogotá, Colombia	Centro de Investigaciones en Microbiología y Biotecnología-UR (CIMBIUR), Facultad de Ciencias Naturales, Universidad del Rosario, Bogotá, Colombia Icahn School of Medicine at Mount Sinai, New York, USA	Adriana van de Guchte; Alberto Paniz-Mondolfi; Alejandro Feged-Rivadeneira; Ana S. Gonzalez-Reiche; Andrés Angel; Carolina Flórez; Carolina Hernández; Emilia Mia Sordillo; Felipe González-Casabianca; Hala Alejel Alshammary; Harm van Bakel; Iván Carroll; Jaime Cascante; Jayeeta Dutta; Juan David Ramírez; Luz Helena Patiño; Marina Muñoz; Matthew M. Hernandez; Mauricio Santos-Vega; Mónica Palma-Cuero; Nathalia Ballesteros; Sergio Gomez; Viviana Simon; Zenab Khan
EPI_ISL_941941, EPI_ISL_941944 to 941948, EPI_ISL_941950, EPI_ISL_941952, EPI_ISL_941954, EPI_ISL_941959, EPI_ISL_941961 to 941962, EPI_ISL_941964 to 941983, EPI_ISL_941995 to 942002, EPI_ISL_942004 to 942005, EPI_ISL_944634 to 944638			
see above	Centro de Investigaciones en Microbiología y Biotecnología-UR (CIMBIUR), Facultad de Ciencias Naturales, Universidad del Rosario, Bogotá, Colombia	Centro de Investigaciones en Microbiología y Biotecnología-UR (CIMBIUR), Facultad de Ciencias Naturales, Universidad del Rosario, Bogotá, Colombia Instituto Nacional de Salud, Bogotá, Colombia Icahn School of Medicine at Mount Sinai, New York, USA	Adriana van de Guchte; Alberto Paniz-Mondolfi; Ana S. Gonzalez-Reiche; Carolina Flórez; Carolina Hernández; Emilia Mia Sordillo; Hala Alejel Alshammary; Harm van Bakel; Jayeeta Dutta; Juan David Ramírez; Luz Helena Patiño; Marina Muñoz; Matthew M. Hernandez; Nathalia Ballesteros; Sergio Gomez; Viviana Simon; Zenab Khan
EPI_ISL_491933 to 491935, EPI_ISL_491941, EPI_ISL_527818 to 527819, EPI_ISL_594118,	Centro de Investigaciones, Universidad de Especialidades Espíritu Santo	Institute of Microbiology, Universidad San Francisco de Quito	Belén Prado-Vivar; Bernardo Gutiérrez; Derly Andrade; Edith Lopez; Fernando Espinoza; Gabriel Morey; Gabriel Trueba; Jose Pedro Barberan; Juan Carlos Fernandez; Juan José Guadalupe; Michelle Grunauer; Monica Becerra-Wong; Patricio Rojas-Silva; Paúl Cárdenas; Ruben Armas; Sully Márquez;

EPI_ISL_697797			Verónica Barragán
EPI_ISL_468761 to 468764, EPI_ISL_500369 to 500372, EPI_ISL_500374 to 500381, EPI_ISL_500383, EPI_ISL_500385 to 500458, EPI_ISL_537382 to 537466, EPI_ISL_537811 to 537873, EPI_ISL_732748 to 732819, EPI_ISL_1093069 to 1093143, EPI_ISL_1217774, EPI_ISL_1217776 to 1217777, EPI_ISL_1217779, EPI_ISL_1217781, EPI_ISL_1217783 to 1217784, EPI_ISL_1217786, EPI_ISL_1217788 to 1217789, EPI_ISL_1217791, EPI_ISL_1217794, EPI_ISL_1217796, EPI_ISL_1217799, EPI_ISL_1217801, EPI_ISL_1217803, EPI_ISL_1217805, EPI_ISL_1217808, EPI_ISL_1217810, EPI_ISL_1217813, EPI_ISL_1217815, EPI_ISL_1217817, EPI_ISL_1217820, EPI_ISL_1217822, EPI_ISL_1217825, EPI_ISL_1217827, EPI_ISL_1217830, EPI_ISL_1217833, EPI_ISL_1217835, EPI_ISL_1217838, EPI_ISL_1217841, EPI_ISL_1217843, EPI_ISL_1217845, EPI_ISL_1217848, EPI_ISL_1217851, EPI_ISL_1217854, EPI_ISL_1217856, EPI_ISL_1217859, EPI_ISL_1217861, EPI_ISL_1217864, EPI_ISL_1217867 to 1217869, EPI_ISL_1217871, EPI_ISL_1217874, EPI_ISL_1217876, EPI_ISL_1217879, EPI_ISL_1217882, EPI_ISL_1217885, EPI_ISL_1217888, EPI_ISL_1217891, EPI_ISL_1217894, EPI_ISL_1217897, EPI_ISL_1217900, EPI_ISL_1217903, EPI_ISL_1217905, EPI_ISL_1217908, EPI_ISL_1217911, EPI_ISL_1217914, EPI_ISL_1217916, EPI_ISL_1217919, EPI_ISL_1217922, EPI_ISL_1217924, EPI_ISL_1217928, EPI_ISL_1217930			
see above	Centro de Investigación Biomédica de La Rioja - Hospital San Pedro Logroño	SeqCOVID-SPAIN consortium/IBV(CSIC)	José Manuel Azcona Gutiérrez; María Pilar Bea Escudero; María de Toro; Miriam Blasco Alberdi and SeqCOVID-SPAIN consortium
EPI_ISL_1279264, EPI_ISL_1279266 to 1279267, EPI_ISL_1279275 to 1279276, EPI_ISL_1279284 to 1279285, EPI_ISL_1279287, EPI_ISL_1279298, EPI_ISL_1279302, EPI_ISL_1279462 to 1279536, EPI_ISL_1287771 to 1287773			
see above	Centro de Investigación Biomédica del Noreste (CIBIN)	Instituto Nacional de Enfermedades Respiratorias (INER): Centro de Investigación en Enfermedades Infecciosas (CIENI)	Alejandro Sanchez-Flores; Alfredo Herrera-Estrella; Alicia Ocaña-Mondragón; Angel Gustavo Salas-Lais; Bernardo Martínez-Miguel; Blanca Taboada; Brenda Irasema Maldonado-Meza; Carla Ivón Herrera-Najera; Carlos F. Arias; Celia Boukadida; Clara Esperanza Santacruz-Tinoco; Concepción Grajales-Muñiz; Consorcio Mexicano de Vigilancia Genómica (CoViGen-Mex). Authors (in alphabetical order): Julio Elias Alvarado-Yaah; Fernando Fontove-Herrera; Francisco Pulido; Gloria Elena Espinoza-Ayala; Gloria María Molina-Salinas; Gloria Vazquez; Hector Esteban Paz-Juárez; Hector Montoya-Fuentes; Helen Haydee Fernanda Ramírez-Plascencia; Jorge Ivan Salinal-Nevarez; José Antonio Enciso-Moreno; José Esteban Muñoz-Medina; José de Jesús Nuñez-Contreras; Juan Bautista Chale-Dzul; Luis Alberto Ochoa-Carrera; Margarita Matías-Florentino; María Guadalupe Santiago-Mauricio; María Guadalupe de Jesús Mireles-Rivera; Nelly Sélem-Mojica; Pavel Isa; Ricardo Grande; Santiago Ávila-Ríos; Víctor Hugo Borja-Aburto
EPI_ISL_1171648 to 1171650	Centro de Saude Dr. Jose Paione em Mococa	Instituto Adolfo Lutz, Interdiciplinary Procedures Center, Strategic Laboratory	Caio Vinicius Dias Lopes; Claudia Regina Gonçalves; Claudio Tavares Sacchi; Erica Valessa Ramos Gomes; Karoline Rodrigues Campos
EPI_ISL_583491	Centro de Saude Esf IV Zona Rural Domingos de SJ Rio Pardo	Instituto Adolfo Lutz, Interdiciplinary Procedures Center, Strategic Laboratory	Claudia Regina Gonçalves; Claudio Tavares Sacchi; Erica Valessa Ramos Gomes; Karoline Rodrigues Campos
EPI_ISL_471543, EPI_ISL_583500	Centro de Saude I Tacito Leite de Carvalho e Silva	Instituto Adolfo Lutz, Interdiciplinary Procedures Center, Strategic Laboratory	Claudia Regina Gonçalves; Claudio Tavares Sacchi; Erica Valessa Ramos Gomes; Karoline Rodrigues Campos
EPI_ISL_735416	Centro de Saude II Dr Jose Paione Mococa	Instituto Adolfo Lutz, Interdiciplinary Procedures Center, Strategic Laboratory	Claudia Regina Gonçalves; Claudio Tavares Sacchi; Erica Valessa Ramos Gomes; Karoline Rodrigues Campos
EPI_ISL_1121311, EPI_ISL_1196296	Centro de Saude II Dr. Jose Paione Mococa	Instituto Adolfo Lutz, Interdiciplinary Procedures Center, Strategic Laboratory	Caio Vinicius Dias Lopes; Claudia Regina Gonçalves; Claudio Tavares Sacchi; Erica Valessa Ramos Gomes; Karoline Rodrigues Campos
EPI_ISL_861668, EPI_ISL_882659	Centro de Triagem Covid19	Instituto Adolfo Lutz, Interdiciplinary Procedures Center, Strategic Laboratory	Claudia Regina Gonçalves; Claudio Tavares Sacchi; Erica Valessa Ramos Gomes; Karoline Rodrigues Campos
EPI_ISL_468305, EPI_ISL_468307, EPI_ISL_735411, EPI_ISL_735423 to 735424, EPI_ISL_735426	Centro de Vigilancia a Saude de Diadema	Instituto Adolfo Lutz, Interdiciplinary Procedures Center, Strategic Laboratory	Claudia Regina Gonçalves; Claudio Tavares Sacchi; Erica Valessa Ramos Gomes; Karoline Rodrigues Campos
EPI_ISL_693240, EPI_ISL_693242	Centro de Vigilância a Saude de Diadema	Instituto Adolfo Lutz, Interdiciplinary Procedures Center, Strategic Laboratory	Claudia Regina Gonçalves; Claudio Tavares Sacchi; Erica Valessa Ramos Gomes; Karoline Rodrigues Campos
EPI_ISL_1239537, EPI_ISL_1239575 to 1239578, EPI_ISL_1239580 to 1239581, EPI_ISL_1239631, EPI_ISL_1239717 to 1239718, EPI_ISL_1273829 to 1273832	Centrum Medycyny Klinicznej i Estetycznej DiMedical Sp. z o.o.	1. National Institute of Public Health - National Institute of Hygiene; 2. Eurofins Genomics Europe Sequencing GmbH	ECDC COVID-19 WGS support team; Eurofins Genomics Europe Sequencing Team; Gierczyki Rafa; Sadkowska-Todys Magorzata; Wokowicz Tomasz; Zacharczuk Katarzyna
EPI_ISL_961286 to 961287, EPI_ISL_961322, EPI_ISL_961352 to 961355, EPI_ISL_961463	Centrum voor Medische Analyse	UAntwerp, Laboratory of Medical Microbiology	Basil Britto Xavier; Christine Lammens; Herman Goossens; Jasmine Coppens; Marie Le Mercier; Veerle Matheeussen
EPI_ISL_450520 to 450524, EPI_ISL_639632 to 639633, EPI_ISL_639636, EPI_ISL_639640, EPI_ISL_639642, EPI_ISL_639648, EPI_ISL_639650 to 639651, EPI_ISL_639654 to 639657, EPI_ISL_639663, EPI_ISL_639672, EPI_ISL_639676 to 639680, EPI_ISL_639683, EPI_ISL_770038 to 770039, EPI_ISL_770042, EPI_ISL_770045, EPI_ISL_770048 to 770049, EPI_ISL_770052, EPI_ISL_770054, EPI_ISL_770056, EPI_ISL_770058 to 770059, EPI_ISL_770061			
see above	Centrl Laboratorija	Latvian Biomedical Research and Study Centre	Ivars Silamielis; Jana Oste; Jnis Kloviš; Jnis Pjalkovskis; Kaspars Megnis; Marta Priedte; Monta Ustinova; Stella Lapia; Uga Dumpis; Vita Rovte; ikita Zrelovs
EPI_ISL_486390 to 486391, EPI_ISL_486411 to 486416, EPI_ISL_486418 to 486421, EPI_ISL_486437, EPI_ISL_492988 to 492992, EPI_ISL_501286 to 501289, EPI_ISL_501808, EPI_ISL_501817, EPI_ISL_501895 to 501896, EPI_ISL_501915, EPI_ISL_501922, EPI_ISL_515196, EPI_ISL_534200 to 534202, EPI_ISL_534206 to 534208, EPI_ISL_534210 to 534211, EPI_ISL_534220, EPI_ISL_534222 to 534223			
see above	Centrl laboratorija	Latvian Biomedical Research and Study Centre	Ivars Silamielis; Jana Oste; Jnis Kloviš; Jnis Pjalkovskis; Kaspars Megnis; Marta Priedte; Monta Ustinova; Stella Lapia; Uga Dumpis; Vita Rovte; ikita Zrelovs
EPI_ISL_861714	Cerba	CERBA LAB	Costa JM; Haïm-Boukobza S.; Hedbaut E; Lecorche E; Malek Ramdane; Olivi M; Roquebert B; Trombert S; Verdurme L
EPI_ISL_900527, EPI_ISL_913666, EPI_ISL_1228951, EPI_ISL_1228954 to 1228963, EPI_ISL_1228965 to 1228967, EPI_ISL_1228969, EPI_ISL_1228972, EPI_ISL_1228974, EPI_ISL_1228978, EPI_ISL_1228997, EPI_ISL_1229000, EPI_ISL_1229002 to 1229003, EPI_ISL_1229008, EPI_ISL_1229012 to 1229013, EPI_ISL_1229017, EPI_ISL_1229024, EPI_ISL_1229028 to 1229035, EPI_ISL_1229039			
see above	Cerballiance	CNR Virus des Infections Respiratoires - France SUD	Antonin Bal; Bruno Lina; Gregory Destras; Gwendolyne Burfin; Hadrien Regue; Hadrien Règue; Laurence Josset; Martine Valette; Quentin Semanas
EPI_ISL_1001461	Cerballiance	Cerba	Haim-Boukobza S; Lecorche E; Meyer S; Roig JC; Roquebert B; Trombert-Paolantoni S
EPI_ISL_861503	Cerballiance Alpes Durance	CERBA LAB	Dyen P; Prots L.
EPI_ISL_1005688	Cerballiance Antony Velpeau	Cerba	Benazra M; Collin T; Delaplace E; Gresset N; Haim-Boukobza S; Lecorche E; Lesenne A; Olivi M; Ricard B; Roquebert B; Trombert-Paolantoni S
EPI_ISL_1005686	Cerballiance Bligny	Cerba	Benazra M; Collin T; Delaplace E; Gresset N; Haim-Boukobza S; Lecorche E; Lesenne A; Olivi M; Ricard B; Roquebert B; Trombert-Paolantoni S
EPI_ISL_1009667	Cerballiance Bourg-la-Reine	Cerba	Benazra M; Collin T; Delaplace E; Gresset N; Haim-Boukobza S; Lecorche E; Lesenne A; Olivi M; Ricard B; Roquebert B; Trombert-Paolantoni S
EPI_ISL_1001465, EPI_ISL_1001467	Cerballiance Carpentras CV	Cerba	Haim-Boukobza S; Lecorche E; Meyer S; Roig JC; Roquebert B; Trombert-Paolantoni S
EPI_ISL_861497, EPI_ISL_861500 to 861502	Cerballiance Centre Est	CERBA LAB	Chanard C; Chanard E; Gazzano V; Munier C; Prots L.
EPI_ISL_1009618	Cerballiance Cerrieres-le-Buisson	Cerba	Benazra M; Collin T; Delaplace E; Gresset N; Haim-Boukobza S; Lecorche E; Lesenne A; Olivi M; Ricard B; Roquebert B; Trombert-Paolantoni S
EPI_ISL_860716, EPI_ISL_861488 to 861496, EPI_ISL_871876	Cerballiance Côte d'Azur	CERBA LAB	; Aankour N; Adjtoutah Z; Adoul O; Barrieu-Moussat S; Barrieu-Moussat S.; Barsanti M; Bausset O; Boudjahem Z; Chaudet L; Chobert F; Costa JM; De La Chapelle A; Francois J; Gandillet A; Gastaud S; Haïm-Boukobza S.; Hedbaut E; Jaugin P; Juvet O; Lecorche E; Malek Ramdane; Maltese S; Mangiarotti T; Marcadal M; Marius M; Martin L; Morello V; Olivi M; Pichon R; Pichon R. Quintle S; Prots L; Prots L.; Prots L. Raimondi V.; Prots.L; Roman E; Roquebert B; Sanza O; Sinsoulieu T; Trombert S; Van Der Rhyn I; Verdurme L; Viltard B
EPI_ISL_1003769	Cerballiance Endoume	Cerba	Haim-Boukobza S; Lecorche E; Meyer S; Roig JC; Roquebert B; Trombert-Paolantoni S
EPI_ISL_1011752	Cerballiance Grigny	Cerba	Benazra M; Collin T; Delaplace E; Gresset N; Haim-Boukobza S; Lecorche E; Lesenne A; Olivi M; Ricard B; Roquebert B; Trombert-Paolantoni S
EPI_ISL_1001466	Cerballiance Haifa	Cerba	Haim-Boukobza S; Lecorche E; Meyer S; Roig JC; Roquebert B; Trombert-Paolantoni S
EPI_ISL_861508	Cerballiance Haut de France	CERBA LAB	Felloni C; Lahmidi S.; Mainardi A
EPI_ISL_1222710	Cerballiance IDF site Vaureal	Cerba Lab	Benasra M; Haïm-Boukobza S; Lecorche E; Mallek R; Olivi M; Roquebert B; Trombert-Paolantoni S
EPI_ISL_1005687	Cerballiance Lardy	Cerba	Benazra M; Collin T; Delaplace E; Gresset N; Haim-Boukobza S; Lecorche E; Lesenne A; Olivi M; Ricard B; Roquebert B; Trombert-Paolantoni S

EPI_ISL_1001573	Cerballiance Luynes	Cerba	Haim-Boukobza S; Lecorche E; Meyer S; Roig JC; Roquebert B; Trombert-Paolantoni S
EPI_ISL_1222720	Cerballiance Maule	Cerba Lab	Besrana M; Haïm-Boukobza S; Lecorche E; Mallek R; Olivi M; Roquebert B; Trombert-Paolantoni S; Zimmer S
EPI_ISL_1005684	Cerballiance Monthléry	Cerba	Benazra M; Collin T; Delaplace E; Gresset N; Haim-Boukobza S; Lecorche E; Lesenne A; Olivi M; Ricard B; Roquebert B; Trombert-Paolantoni S
EPI_ISL_1005685	Cerballiance Montlhéry	Cerba	Benazra M; Collin T; Delaplace E; Gresset N; Haim-Boukobza S; Lecorche E; Lesenne A; Olivi M; Ricard B; Roquebert B; Trombert-Paolantoni S
EPI_ISL_871789, EPI_ISL_871792	Cerballiance Normandie	CERBA LAB	BRIAND G; COUDRAY J; COULON C; HAMEL A.; LAFOREST D; LEPIGEON; MARTINS-AMARAL F; THOMAS Johan; VITEL R; technicien V
EPI_ISL_1004174	Cerballiance Port de Bouc	Cerba	Haim-Boukobza S; Lecorche E; Meyer S; Roig JC; Roquebert B; Trombert-Paolantoni S
EPI_ISL_1001463	Cerballiance Port de bouc	Cerba	Haim-Boukobza S; Lecorche E; Meyer S; Roig JC; Roquebert B; Trombert-Paolantoni S
EPI_ISL_861504 to 861507	Cerballiance Provence	CERBA LAB	Delaunay E; Prots L.; Roig JC
EPI_ISL_1000968, EPI_ISL_1001462	Cerballiance Provence	Cerba	Haim-Boukobza S; Lecorche E; Meyer S; Roig JC; Roquebert B; Trombert-Paolantoni S
EPI_ISL_1002710	Cerballiance Rouviere	Cerba	Haim-Boukobza S; Lecorche E; Meyer S; Roig JC; Roquebert B; Trombert-Paolantoni S
EPI_ISL_862046 to 862047	Cerballiance Val de Loire	CERBA LAB	Abs G.
EPI_ISL_1060597	Cerballiance Vauréal	Cerba	Benazra M; Haim-Boukobza S; Lecorche E; Olivi M; Roquebert B; Trombert-Paolantoni S
EPI_ISL_1271520	Cerballiance Vauréal	Cerba Lab	Benazra M; Costa JM; Haïm-Boukobza S; Lecorche E; Olivi M; Roquebert B; Trombert-Paolantoni S; Verdurme L; Zimmer S
EPI_ISL_1222721	Cerballiance Viry Chatillon	Cerba LAb	Haïm-Boukobza S; Lecorche E; Lessenne A; Mallek R; Olivi M; Picard B; Roquebert B; Trombert-Paolantoni S; Zimmer S
EPI_ISL_855551	Cerballiance Wilson	CERBA LAB	Costa JM; Haïm-Boukobza S; Herhira S; Lecorche E; Malek R; Merah K; Olivi M; Roquebert B; Trombert S; Verdurme L; Zimmer S
EPI_ISL_1009674	Cerballiance Wilson	Cerba	Benazra M; Haïm-Boukobza S; Lecorche E; Merah K; Olivi M; Roquebert B; Trombert-Paolantoni S
EPI_ISL_802994 to 802995	Charite Universitatsmedizin Berlin, Institut fur Virologie, Charitéplatz 1, 10117 Berlin, Germany	Charite Universitatsmedizin Berlin, Institut fur Virologie, Charitéplatz 1, 10117 Berlin, Germany	Barbara Mühlemann; Christian Drosten; Julia Schneider; Julia Tesch; Jörn Beheim-Schwarzbach; Talitha Veith; Terry Jones; Tobias Bleicker; Victor M Corman
EPI_ISL_1164522 to 1164553, EPI_ISL_1164557 to 1164559, EPI_ISL_1164563 to 1164569, EPI_ISL_1164571 to 1164575, EPI_ISL_1164578 to 1164584, EPI_ISL_1164586 to 1164587, EPI_ISL_1164589 to 1164595			
see above	Charite Universitatsmedizin Berlin, Institut fur Virologie/Labor Berlin	Charite Universitatsmedizin Berlin, Institut fur Virologie	Barbara Mühlemann; Christian Drosten; Julia Schneider; Jörn Beheim-Schwarzbach; Talitha Veith; Terry Jones; Victor M Corman
EPI_ISL_516629 to 516645	Charite Universitatsmedizin Berlin, Institut fur Virologie/Labor Berlin	Charite Universitatsmedizin Berlin, Institut fur Virologie/Labor Berlin	Barbara Muhlemann; Christian Drosten; Julia Schneider; Jörn Beheim-Schwarzbach; Talitha Veith; Terry Jones; Victor M Corman
EPI_ISL_862124 to 862132, EPI_ISL_862134 to 862159, EPI_ISL_862163 to 862187, EPI_ISL_862659 to 862664	Charité Universitätsmedizin Berlin, Institut fur Virologie/Labor Berlin	Charité Universitätsmedizin Berlin, Institut für Virologie	Barbara Mühlemann; Christian Drosten; Julia Schneider; Julia Tesch; Jörn Beheim-Schwarzbach; Talitha Veith; Terry Jones; Tobias Bleicker; Victor M Corman
EPI_ISL_754174 to 754175	Charité Universitätsmedizin Berlin, Institut für Virologie	Charité Universitätsmedizin Berlin, Institut für Virologie	Barbara Mühlemann; Christian Drosten; Julia Schneider; Jörn Beheim-Schwarzbach; Talitha Veith; Terry Jones; Victor M Corman
EPI_ISL_729346, EPI_ISL_729348, EPI_ISL_729353, EPI_ISL_729355 to 729357, EPI_ISL_729360, EPI_ISL_729365 to 729366, EPI_ISL_729370, EPI_ISL_729377 to 729378, EPI_ISL_729382, EPI_ISL_729387 to 729388, EPI_ISL_729398, EPI_ISL_729400, EPI_ISL_729402, EPI_ISL_729404, EPI_ISL_729406, EPI_ISL_729408 to 729410, EPI_ISL_729412 to 729413, EPI_ISL_729415, EPI_ISL_729468, EPI_ISL_729473 to 729497, EPI_ISL_729499, EPI_ISL_729514, EPI_ISL_729529 to 729534, EPI_ISL_729548, EPI_ISL_729553 to 729557, EPI_ISL_729559 to 729561, EPI_ISL_729596 to 729607, EPI_ISL_753702 to 754058, EPI_ISL_754184 to 754194, EPI_ISL_806526 to 806531, EPI_ISL_806533 to 806537, EPI_ISL_806539 to 806542, EPI_ISL_853394 to 853404, EPI_ISL_869083, EPI_ISL_869085, EPI_ISL_869118 to 869119, EPI_ISL_869124 to 869136, EPI_ISL_869143, EPI_ISL_909747, EPI_ISL_909750 to 909760, EPI_ISL_909771, EPI_ISL_912227 to 912267			
see above	Charité Universitätsmedizin Berlin, Institut für Virologie/Labor Berlin	Charité Universitätsmedizin Berlin, Institut für Virologie	Barbara Mühlemann; Christian Drosten; Cornelia Schlee; Julia Schneider; Julia Tesch; Jörn Beheim-Schwarzbach; Talitha Veith; Terry Jones; Tobias Bleicker; Tomasz Zemojtel; Victor M Corman
EPI_ISL_818342	Charité Universitätsmedizin Berlin, Institute of Virology	Charité Universitätsmedizin Berlin, Institute of Virology	Barbara Mühlemann; Christian Drosten; Julia Schneider; Jörn Beheim-Schwarzbach; Talitha Veith; Terry Jones; Victor M Corman
EPI_ISL_824920 to 824921, EPI_ISL_824942 to 824944, EPI_ISL_824985 to 824986, EPI_ISL_848200 to 848202, EPI_ISL_856678, EPI_ISL_856680 to 856681, EPI_ISL_856683, EPI_ISL_860784 to 860790, EPI_ISL_868949, EPI_ISL_875344			
see above	Charité Universitätsmedizin Berlin, Institute of Virology, Charitéplatz 1, 10117 Berlin, Germany	Charité Universitätsmedizin Berlin, Institute of Virology, Charitéplatz 1, 10117 Berlin, Germany	Barbara Mühlemann; Christian Drosten; Julia Schneider; Julia Tesch; Jörn Beheim-Schwarzbach; Talitha Veith; Terry Jones; Tobias Bleicker; Victor M Corman
EPI_ISL_406862	Charité Universitätsmedizin Berlin, Institute of Virology; Institut für Mikrobiologie der Bundeswehr, Munich	Charité Universitätsmedizin Berlin, Institute of Virology	Barbara Mühlemann; Christian Drosten; Julia Schneider; Markus Antwerpen; Roman Wölfel; Talitha Veith; Victor M Corman
EPI_ISL_940075 to 940104, EPI_ISL_940106 to 940107, EPI_ISL_940109 to 940124, EPI_ISL_940126 to 940137, EPI_ISL_1239267 to 1239285, EPI_ISL_1239287 to 1239326, EPI_ISL_1239328 to 1239329			
see above	Charlotte Maxeke Johannesburg Academic Hospital, National Health Laboratory Services, Gauteng, South Africa	National Institute for Communicable Diseases of the National Health Laboratory Service	Allam M; Amoako DG; Bhiman JN; Ismail A; Mahlangu B; Mohale T; Ntuli N; Scheepers C
EPI_ISL_735496 to 735497, EPI_ISL_1123268	Chattogram Veterinary and Animal Sciences University	Central Biological Research Laboratory and Department of Biochemistry and Molecular Biology	H. M. Abdullah Al Masud; Imam Hossen; Md. Arif Hossain; Md. Imranul Hoq; Md. Khondakar Raziur Rahman; Md. Omer Faruq; Mohammad Omar Faruque; Robiul Hasan Bhuiyan; Sajib Rudra; Shanta Paul
EPI_ISL_1153448, EPI_ISL_1153530 to 1153538, EPI_ISL_1156362 to 1156370, EPI_ISL_1157100 to 1157104, EPI_ISL_1157106 to 1157116, EPI_ISL_1211988, EPI_ISL_1211990, EPI_ISL_1211992 to 1211993, EPI_ISL_1211996 to 1211997, EPI_ISL_1212000 to 1212005, EPI_ISL_1212007, EPI_ISL_1212009, EPI_ISL_1280590 to 1280600			
see above	Chemische und Veterinäruntersuchungsamt Münsterland-Emscher-Lippe	Robert Koch Institute	
EPI_ISL_1211989, EPI_ISL_1211991, EPI_ISL_1211994 to 1211995, EPI_ISL_1211998 to 1211999, EPI_ISL_1212006, EPI_ISL_1212008	Chemisches und Veterinäruntersuchungsamt Ostwestfalen-Lippe	Robert Koch Institute	
EPI_ISL_569612 to 569613	Cheyenne River Health Center	South Dakota Public Health Laboratory	Jacob Garfin; Matt Plumb; Xiong Wang; and Chris Carlson
EPI_ISL_419297 to 419298, EPI_ISL_419309 to 419311, EPI_ISL_479809 to 479811, EPI_ISL_480004 to 480014, EPI_ISL_684159 to 684161, EPI_ISL_686659 to 686844, EPI_ISL_691284 to 691288, EPI_ISL_901717 to 901721, EPI_ISL_1127138, EPI_ISL_1127144 to 1127145, EPI_ISL_1127164 to 1127183, EPI_ISL_1128018 to 1128056			
see above	Chiba Prefectural Institute of Public Health	Pathogen Genomics Center, National Institute of Infectious Diseases	Hajime Kamiya; Kentaro Itokawa; Makoto Kuroda; Masakatsu Taira; Masanori Hashino; Motoi Suzuki; Rina Tanaka; Tsuyoshi Sekizuka; Yushi Hachisu
EPI_ISL_468070 to 468078, EPI_ISL_477125 to 477140, EPI_ISL_492028 to 492031, EPI_ISL_625457 to 625477, EPI_ISL_700328 to 700347, EPI_ISL_768728 to 768742, EPI_ISL_906082 to 906090, EPI_ISL_906092 to 906097, EPI_ISL_906099 to 906116			
see above	Child Health Research Foundation	Child Health Research Foundation	Afroza Akter Tanni; Arif Mohammad Tanmoy; Hafizur Rahman; Maksuda Islam; Md Hafizur Rahman; Md Saiful Islam Sajib; Nikkon Sarkar; Roly Malaker; Samir K Saha; Senjuti Saha; Sharmistha Goswami; Syed Muktadir Al Sium
EPI_ISL_437912	Child Health Research Foundation	Child Health Research Lab	Maksuda Islam; Md Hafizur Rahman; Md Hasanuzzaman; Md Saiful Islam Sajib; Md Shahidul Islam; Roly Malaker; Samir K Saha; Senjuti Saha; Zabed B Ahmed
EPI_ISL_513343 to 513344	Children Westmead Hospital	NSW Health Pathology - Institute of Clinical Pathology and Medical Research; Westmead Hospital; University of Sydney	CIDM-PH et al.
EPI_ISL_507962, EPI_ISL_1314300	Children's Hospitals and Clinics of Minnesota	Minnesota Department of Health, Public Health Laboratory	Alexandra Lorentz; Jacob Garfin; Matt Plumb; and Xiong Wang
EPI_ISL_451548, EPI_ISL_451594,	Childrens Hospital Westmead	NSW Health Pathology - Institute of Clinical Pathology and	CIDM-PH et al.

EPI_ISL_451605, EPI_ISL_455068		Medical Research; Westmead Hospital; University of Sydney	
EPI_ISL_430722 to 430746	Chinese PLA Institute for Disease Control and Prevention	Chinese PLA Institute for Disease Control and Prevention	Lizhong Li; Peng LiJinhui Li
EPI_ISL_417316, EPI_ISL_417330 to 417332, EPI_ISL_429877 to 429878, EPI_ISL_847515, EPI_ISL_847517, EPI_ISL_847536, EPI_ISL_847540, EPI_ISL_847545 to 847546, EPI_ISL_847553, EPI_ISL_847563, EPI_ISL_847579, EPI_ISL_847619 to 847622, EPI_ISL_847640 to 847644, EPI_ISL_847716, EPI_ISL_847720, EPI_ISL_847730 to 847731, EPI_ISL_847736, EPI_ISL_847738, EPI_ISL_847741 to 847744, EPI_ISL_847746 to 847747, EPI_ISL_847782, EPI_ISL_855399 to 855401, EPI_ISL_977885 to 977983, EPI_ISL_977985 to 978168, EPI_ISL_978955 to 978973			
see above	Chiu Laboratory, University of California, San Francisco	Chiu Laboratory, University of California, San Francisco	Brian Bushnell; Candace Wang; Charles Chiu; Debra Wadford; Jill Hacker; Scot Federman; Venice Servellita; Wei Gu; Xianding (Wayne) Deng; Xianding Deng; and Charles Y. Chiu
EPI_ISL_1239330 to 1239358, EPI_ISL_1239360	Chris Hani Baragwanath Hospital, National Health Laboratory Services, Gauteng, South Africa	National Institute for Communicable Diseases of the National Health Laboratory Service	Amoako DG; Bhiman JN; Ismail A; Mahlangu B; Mohale T; Ntuli N; Scheepers C
EPI_ISL_833337	Christ the Saviour General Hospital - Batangas	Research Institute for Tropical Medicine	Catalino Demetria; Daria Manalo; Edelwisa Mercado; Francisco Gerardo Polotan; Hannah Leah Morito; Inez Andrea Medado; John Leonard Chan; Kirstyn Brunker; Ma Angelica Tujan; Othoniel Jan Onza
EPI_ISL_872100 to 872104	Chu Tivoli	GIGA Medical Genomics	Bouchra Boujemla; Cécile Meex; Keith Durkin; Maria Artesi; Marie-Pierre Hayette; Pierrette Melin; Raphaël Boreux; Sébastien Bontems; Vincent Bours
EPI_ISL_637033	Chun Yeung Estate Quarantine Center	Hong Kong Department of Health	Chan Rickjason C.W.; Lam Edman T.K.; Mak Gannon C.K.; Tsang Dominic N.C.
EPI_ISL_443315	Château de la Source	National Reference Center for Viruses of Respiratory Infections, Institut Pasteur, Paris	Angela Brisebarre; Etienne Simon-Lorière; Flora Donati; Marion Barbet; Maud Vanpeene; Mélanie Albert; Méline Bizard; Sylvie Behillil; Sylvie van der Werf; Vincent Enouf
EPI_ISL_491115	Cicin-Sain Lab	Cicin-Sain Lab	Kathrin Eschke; Luka Cicin-Sain; M. Zeeshan Chaudhry; Yeonsu Kim
EPI_ISL_877422	Citra Medika Hospital	Institute of Tropical Disease, Universitas Airlangga	Aldise M Nastri; Gatot Soegiarto; Jezzy R Dewantari; Kazufumi Shimizu; Krisnoadi Rahardjo; Laksmi Wulandari; Maria I Lusida; Resti Yudhawati; Rima R Prasetya; Soetjipto; Wahidah Rachmaniyah; Yasuko Mori
EPI_ISL_636981	City of Chimoio	KRISP, KZN Research Innovation and Sequencing Platform	Giandhari J; Ismael N; Nadia Sitoe; Nedio Mabunda; Paulo Arnaldo; Pillay S; Tegally H; Wilkinson E; de Oliveira T
EPI_ISL_454646	City of El Paso Department of Public Health Laboratory	Pathogen Discovery, Respiratory Viruses Branch, Division of Viral Diseases, Centers for Disease Control and Prevention	Anna Uehara; Clinton R. Paden; Haibin Wang; Jing Zhang; Krista Queen; Michael Bowen; Suxiang Tong; Yan Li; Ying Tao; Zachary Weiner
EPI_ISL_833413, EPI_ISL_1192363	City of Hope Comprehensive Cancer Center	Los Angeles County PHL	P. Hemarajata et al.
EPI_ISL_931472 to 931485, EPI_ISL_940715 to 940730, EPI_ISL_940732 to 940734, EPI_ISL_940784 to 940791, EPI_ISL_954753 to 954754, EPI_ISL_1015493 to 1015514, EPI_ISL_1262586 to 1262609			
see above	City of Milwaukee Health Department Laboratory	City of Milwaukee Health Department Laboratory	Sanjib Bhattacharyya
EPI_ISL_547585 to 547593, EPI_ISL_560318, EPI_ISL_560322 to 560324, EPI_ISL_561337 to 561343	Civil Hospital, Panchkula	CSIR-Institute of Microbial Technology	Amandeep Kaur; Anu Singh; Ashwani Kumar; Debarghya Ghose; Dipak Dutta; Harsh Goar; Kanika Bansal; Navin Baid; Poushali Chakraborty; Prabhu B. Patil; Rajesh Kumar Mishra; Sanjeet Kumar; Sanjeev Khosla
EPI_ISL_539483 to 539490, EPI_ISL_561344	Civil Hospital, Rupnagar	CSIR-Institute of Microbial Technology	Amandeep Kaur; Anu Singh; Ashwani Kumar; Debarghya Ghose; Dipak Dutta; Harsh Goar; Kanika Bansal; Navin Baid; Poushali Chakraborty; Prabhu B. Patil; Rajesh Kumar Mishra; Sanjeet Kumar; Sanjeev Khosla
EPI_ISL_1278373 to 1278942	Clalit Health Services Laboratories, Israel	Stern Lab	Stern Lab
EPI_ISL_1250842, EPI_ISL_1278275 to 1278276	Clin & Gen Lab	Molecular Genetics Laboratory, Instituto de Investigaciones Químicas, Universidad Mayor de San Andrés	Aneth Vasquez Michel; Oscar M. Rollano-Peñaloza
EPI_ISL_640068, EPI_ISL_700432, EPI_ISL_700519	Clinic-in-Asla	NHLS/UCT	Arash Iranzadeh; Bruna Galvao; Carolyn Williamson; Deelan Doolabh; Diana Hardie; Houriiyah Tegally; Innocent Mudau; Kruger Marais; Lynn Tyers; Marvin Hsiao; Stephen Korsman
EPI_ISL_414579	Clinica Alemana de Santiago, Chile	Instituto de Salud Publica de Chile	Alejandra Acevedo; Andrés E. Castillo; Bárbara Parra; Carolina Tambley; Gabriel Leal; Gisselle Barra; Jaime Lagos; Javier Tognarelli; Jorge Fernández.; Loredana Arata; Patricia Bustos; Paz Tapia; Rodrigo Fasce; Soledad Ulloa; Winston Andrade
EPI_ISL_527759, EPI_ISL_770004	Clinica Biblica	Inciensa, Instituto Costarricense de Investigación y Enseñanza en Nutrición y Salud	Adriana Godinez & Melany Calderon; Adriana Godínez; Claudio Soto-Garita; Estela Cordero; Francisco Duarte; Hebleen Porras; Melany Calderón & Karla Gutiérrez-González
EPI_ISL_414580	Clinica Santa Maria, Santiago, Chile	Instituto de Salud Publica de Chile	Alejandra Acevedo; Andrés E. Castillo; Bárbara Parra; Carolina Tambley; Gabriel Leal; Gisselle Barra; Jaime Lagos; Javier Tognarelli; Jorge Fernández.; Loredana Arata; Patricia Bustos; Paz Tapia; Rodrigo Fasce; Soledad Ulloa; Winston Andrade
EPI_ISL_1303368	Clinica de la Costa	Instituto Nacional de Salud- Dirección de Investigación en Salud Pública	Carlos Franco-Muñoz; Carmen Osorio; Diana Malo; Diego A. Álvarez-Díaz; Diego Andrés Prada; Gerardo Santamaría; Hector Alejandro Ruiz-Moreno; Jhonnatan Reales-González; Juan Camilo Martinez; Julian Naizaque; Katherine Laiton-Donato; Lisseth Pardo; Magdalena Wiesner; Marcela Mercado-Reyes; Maria T. Herrera-Sepúlveda; Marta Lopez Blanco; Martha Lucia Ospina Martinez; Sergio Gomez; Sheryll Corchuelo; Ángela Alarcon Cruz
EPI_ISL_1017711	Clinica del norte	Instituto Nacional de Salud- Dirección de Investigación en Salud Pública	Carlos Franco-Muñoz; Diego A. Álvarez-Díaz; Diego Andrés Prada; Gerardo Santamaría; Hector Alejandro Ruiz-Moreno; Jhonnatan Reales-González; Julian Naizaque; Katherine Laiton-Donato; Magdalena Wiesner; Marcela Mercado-Reyes; Maria T. Herrera-Sepúlveda; Martha Lucia Ospina Martinez; Sheryll Corchuelo
EPI_ISL_462450 to 462476	Clinical Center, University of Sarajevo	Charite Universitatsmedizin Berlin, Institute of Virology	Almedina Hadzihasanovic-Moro; Amela Dedeic-Ljubovic; Barbara Muehlemann; Christian Drosten; Irma Salimovic-Besic; Jorn Beheim-Schwarzbach; Julia Schneider; Selma Mutevelic; Suzana Arapcic; Talitha Veith; Terry Jones; Victor M Corman
EPI_ISL_1240071 to 1240076, EPI_ISL_1300645, EPI_ISL_1300647 to 1300651, EPI_ISL_1300653, EPI_ISL_1300656 to 1300659	Clinical Center, University of Sarajevo; Unit for Clinical Microbiology	Clinical Center, University of Sarajevo; Unit for Clinical Microbiology	Amela Dedei-Ljubovi; Edina Zahirovi; Golubinka Boshevska; Irma Salimovi-Beši; Maja Kuzmanovska; Sandra Vegar-Zubovi; Sebija Izetbegovi; Suzana Arapi
EPI_ISL_421279, EPI_ISL_421281, EPI_ISL_437455 to 437458, EPI_ISL_902950 to 902968, EPI_ISL_902970 to 902995, EPI_ISL_903218 to 903228, EPI_ISL_911353 to 911371, EPI_ISL_911402 to 911429, EPI_ISL_911443 to 911470, EPI_ISL_911549 to 911579, EPI_ISL_911604 to 911632, EPI_ISL_911710 to 911731, EPI_ISL_911733 to 911745, EPI_ISL_911751 to 911754, EPI_ISL_911924 to 911928			
see above	Clinical Diagnostics Laboratory, Diagnostic & Experimental Pathology, Lilly Research Laboratories	Clinical Diagnostics Laboratory, Diagnostic & Experimental Pathology, Lilly Research Laboratories	Andrew Schade; Angie Fulford; Erin Wray; Jeff Fill; Joe Oakley; John Calley; John McElwee; Leslie ONeill Reising; Leslie O'Neill Reising; Mayuri Vaidya; Pat Finnegan; Phil Ebert; Rachael Redmond; Sam McNeely; Tim Holzer
EPI_ISL_1233898	Clinical Division, Fred Hutchinson Cancer Research Center	Clinical Division, Fred Hutchinson Cancer Research Center	A.K.; C.-W.; D.E.; Geraghty; Greninger, A.; Ishitani, A.; Jerome, K.; L.P.; Li, R.; M.G.; Mills; Nelson; Pyo; Randhawa; Roychoudhury, P.; W.C.; Wang, R.; Zhao
EPI_ISL_677715, EPI_ISL_677718	Clinical Hospital - Bitola	Research Center for Genetic Engineering and Biotechnology "Georgi D. Efremov" , Macedonian Academy of Sciences and Arts	RCGEB - MASA
EPI_ISL_516426 to 516431, EPI_ISL_677676 to 677677, EPI_ISL_677716 to 677717, EPI_ISL_677720 to 677721, EPI_ISL_677724, EPI_ISL_678250, EPI_ISL_678259			
see above	Clinical Hospital - Shtip	Research Center for Genetic Engineering and Biotechnology "Georgi D. Efremov" , Macedonian Academy of Sciences and Arts	RCGEB - MASA
EPI_ISL_424352	Clinical Laboratory, Fuyang City Center for Disease Control and Prevention	Clinical Laboratory, Fuyang City Center for Disease Control and Prevention	Ge, B.
EPI_ISL_450506	Clinical Laboratory, Hospital Israelita Albert Einstein	Clinical Laboratory, Hospital Israelita Albert Einstein	Amgarten, D.; Araujo; C.L.P.; D.B.; D.B.L.; Durigon; E.L. and Pinho; J.R.R.; Machado; Malta, F.; Mangueira; R.A.F.; R.R.G.; Santana; de Oliveira
EPI_ISL_1005699	Clinical Laboratory, Vetsuisse Faculty, University of Zurich	Department of Biosystems Science and Engineering, ETH Zürich	Chaoran Chen; Christian Beisel; David Dreifuss; Elodie Burcklen; Ina Nissen; Ivan Topolsky; Julia Klaus; Katharina Jahn; Lara Fuhrmann; Marina Luisa Meli; Natascha Santacroce; Niko Beerenwinkel; Philipp Jablonski; Rebecca Denes; Regina Hofmann-Lehmann; Sarah Nadeau; Tanja Stadler

EPI_ISL_416432	Clinical Microbiology Lab	Infectious Disease Research Department, King Abdullah International Medical Research Center (KAIMRC)	Abdulrahman Alswaji; Liliane Okdah; Majed Alghoribi; Michel Doumith; Sadeem Alhayli; Sameera Al Johani
EPI_ISL_1248934, EPI_ISL_1249610, EPI_ISL_1250222, EPI_ISL_1258423 to 1258424, EPI_ISL_1258463 to 1258465, EPI_ISL_1263319 to 1263323	Clinical Microbiology Lab RSPTN Universitas Hasanuddin	Clinical Microbiology Lab RSPTN Universitas Hasanuddin	Gita Vita Soraya; Handayani; Handayani Halik; Muhammad Nasrum Massi; Muhammad Yusuf Usman; Rizalinda Sjahril; Rufika Shari; Triwibawa
EPI_ISL_1203843	Clinical Microbiology Laboratory	James Molecular Laboratory	Amy Leber; Dan Jones; Huanyu Wang; Pam Snyder; Zachary Rudy
EPI_ISL_747194, EPI_ISL_1122422 to 1122424	Clinical Microbiology Laboratory, Faculty of Medicine, Universitas Indonesia	Clinical Microbiology Laboratory, Faculty of Medicine, Universitas Indonesia	Andi Yasmon; Anis Karuniawati; Augustine Natasha; Erni J. Nelwan; Fera Ibrahim; Fithriyah; Pratiwi Sudarmono; Yulia Rosa Saharman
EPI_ISL_1196774, EPI_ISL_1196792 to 1196793, EPI_ISL_1196795, EPI_ISL_1196798	Clinical Microbiology Laboratory, Faculty of Medicine, Universitas Indonesia	Faculty of Medicine, Universitas Indonesia	Andi Yasmon; Anis Karuniawati; Ari Fahrial Syam; Badriul Hegar; Beti Ernawati dewi; Budi Wiweko; Dwiana Ocviyanti; Fadilah; Fera Ibrahim; Khaerunissa Anbar Istiadi; Linda Erlina; Pratiwi Sudarmono; Rafika Indah Paramita
EPI_ISL_1001460, EPI_ISL_1080751 to 1080752, EPI_ISL_1080756, EPI_ISL_1080759, EPI_ISL_1080761, EPI_ISL_1080767 to 1080769, EPI_ISL_1080772 to 1080773, EPI_ISL_1080775, EPI_ISL_1080777 to 1080779, EPI_ISL_1080782, EPI_ISL_1080794 to 1080795, EPI_ISL_1080797, EPI_ISL_1080802, EPI_ISL_1080804 to 1080808			
see above	Clinical Microbiology Laboratory, NewYork Presbyterian Hospital/Columbia University Irving Medical Center	Uhlemann Laboratory, Columbia University Irving Medical Center	Anne-Catrin Uhlemann; David Ho; Hiroshi Mohri; Hiroshi Mori; Medini K. Annavajhala
EPI_ISL_447419 to 447469	Clinical Microbiology Laboratory, Sheba Medical Center	Stern Lab	Stern Lab
EPI_ISL_447383 to 447406, EPI_ISL_447417 to 447418	Clinical Microbiology Laboratory, The Baruch Padeh Medical Center, Poriya	Stern Lab	Stern Lab
EPI_ISL_483570	Clinical Microbiology Laboratory- Basurto University Hospita	Biocruces-Bizkaia	Ana Belén Belén de la Hoz; Estibaliz Ugalde-Zarraga; José Luis Díaz de Tuesta del Arco; Matxalen Vidal-García; Mikel J. Urrutikoetxea-Gutierrez; Mº Carmen Nieto Toboso
EPI_ISL_483566, EPI_ISL_483571 to 483573, EPI_ISL_486876, EPI_ISL_489833 to 489835, EPI_ISL_490202 to 490204, EPI_ISL_490977	Clinical Microbiology Laboratory- Basurto University Hospital	Biocruces-Bizkaia	Ana Belén Belén de la Hoz; Estibaliz Ugalde-Zarraga; José Luis Díaz de Tuesta del Arco; Matxalen Vidal-García; Mikel J. Urrutikoetxea-Gutierrez; Mº Carmen Nieto Toboso
EPI_ISL_1081843 to 1081883, EPI_ISL_1081890 to 1081921, EPI_ISL_1081923, EPI_ISL_1081925, EPI_ISL_1081927, EPI_ISL_1081929, EPI_ISL_1081931, EPI_ISL_1081933, EPI_ISL_1081936, EPI_ISL_1081938, EPI_ISL_1081940, EPI_ISL_1081942, EPI_ISL_1081944, EPI_ISL_1128779, EPI_ISL_1128782, EPI_ISL_1128784, EPI_ISL_1128786, EPI_ISL_1128794, EPI_ISL_1128796, EPI_ISL_1128798, EPI_ISL_1128801, EPI_ISL_1128803, EPI_ISL_1128805, EPI_ISL_1128808, EPI_ISL_1128810, EPI_ISL_1128812, EPI_ISL_1128815, EPI_ISL_1128817, EPI_ISL_1128819, EPI_ISL_1128822, EPI_ISL_1128824, EPI_ISL_1128826, EPI_ISL_1128829, EPI_ISL_1128831, EPI_ISL_1128833, EPI_ISL_1128835, EPI_ISL_1128837, EPI_ISL_1128839, EPI_ISL_1128842, EPI_ISL_1128844, EPI_ISL_1128846, EPI_ISL_1128849, EPI_ISL_1128851, EPI_ISL_1128853, EPI_ISL_1128855, EPI_ISL_1128858, EPI_ISL_1128860, EPI_ISL_1128862, EPI_ISL_1128866, EPI_ISL_1128868, EPI_ISL_1128870, EPI_ISL_1128873, EPI_ISL_1128875, EPI_ISL_1128878, EPI_ISL_1128880, EPI_ISL_1128882, EPI_ISL_1128884, EPI_ISL_1128887, EPI_ISL_1128889, EPI_ISL_1128892, EPI_ISL_1128894, EPI_ISL_1128896, EPI_ISL_1128899, EPI_ISL_1128901, EPI_ISL_1128903, EPI_ISL_1128906, EPI_ISL_1128908 to 1128909, EPI_ISL_1128911, EPI_ISL_1128913 to 1128914, EPI_ISL_1128916, EPI_ISL_1128918 to 1128919, EPI_ISL_1128921, EPI_ISL_1128923, EPI_ISL_1128925, EPI_ISL_1128928, EPI_ISL_1128930, EPI_ISL_1128932, EPI_ISL_1128935, EPI_ISL_1128937, EPI_ISL_1128939, EPI_ISL_1128941, EPI_ISL_1128944, EPI_ISL_1128946, EPI_ISL_1176356 to 1176401, EPI_ISL_1239146 to 1239215			
see above	Clinical Microbiology, Infection Prevention and Control	Section for Molecular Diagnostics	Björn Hallström; Jonas Björkman
EPI_ISL_1164183	Clinical Microbiology, Nationwide Children's Hospital	James Molecular Laboratory	Amy Leber; Dan Jones; Huanyu Wang; Pam Snyder; Zachary Rudy
EPI_ISL_877585 to 877763, EPI_ISL_884291 to 884293, EPI_ISL_968140 to 968188, EPI_ISL_1067985 to 1068024, EPI_ISL_1163738 to 1163793, EPI_ISL_1168697	Clinical Molecular Microbiology Laboratory, UNC Hospital	Dirk Dittmer	Angelica Juarez; Blossom Damania.; Brent A. Eason; Cameroon Grant; Carolina Caro-Vegas; Cecilia Thompson; Dirk Dittmer; Evelyn Hoffman; Jason Wong; Justin T. Landis; Linda Pluta; Melissa B. Miller; Patricio Cano; Razia Moorad; Ryan McNamara; Shawn Hawken
EPI_ISL_831334 to 831369, EPI_ISL_832082 to 832089, EPI_ISL_832091 to 832094, EPI_ISL_832121 to 832126, EPI_ISL_861443 to 861455, EPI_ISL_911580 to 911603, EPI_ISL_911674, EPI_ISL_965157 to 965177, EPI_ISL_965906 to 965908, EPI_ISL_1137506 to 1137507, EPI_ISL_1137509 to 1137511, EPI_ISL_1137513 to 1137517, EPI_ISL_1137519 to 1137520, EPI_ISL_1137522 to 1137523, EPI_ISL_1137526, EPI_ISL_1137529, EPI_ISL_1137532, EPI_ISL_1137534, EPI_ISL_1137537 to 1137540, EPI_ISL_1137546 to 1137547, EPI_ISL_1137550 to 1137551, EPI_ISL_1137553 to 1137554, EPI_ISL_1137556 to 1137562, EPI_ISL_1137565 to 1137568, EPI_ISL_1137573 to 1137574, EPI_ISL_1137582, EPI_ISL_1137587 to 1137590, EPI_ISL_1137592 to 1137594, EPI_ISL_1137596, EPI_ISL_1137598, EPI_ISL_1163846 to 1163848, EPI_ISL_1163850, EPI_ISL_1163853 to 1163880, EPI_ISL_1163882 to 1163888, EPI_ISL_1164179 to 1164182, EPI_ISL_1192336 to 1192362, EPI_ISL_1240795 to 1240937			
see above	Clinical Molecular Microbiology Laboratory, UNC Hospitals	Jeremy Wang	Alexander Rubinsteyn; Benjamin Vincent; Cecilia Thompson; Clark Cunningham; Colleen Rice; Corbin Jones; Jason Smedberg; Jeremy Wang; Jonathan Juliano; Kaylee Gentry; Melissa Miller; Robert Hagan; Shawn Hawken
EPI_ISL_914003 to 914009, EPI_ISL_955264, EPI_ISL_955266 to 955268, EPI_ISL_955270, EPI_ISL_955276, EPI_ISL_955278 to 955282, EPI_ISL_955286 to 955287, EPI_ISL_955289, EPI_ISL_955294 to 955297, EPI_ISL_955301, EPI_ISL_955304 to 955306, EPI_ISL_955312, EPI_ISL_955317 to 955318, EPI_ISL_955321			
see above	Clinical Pathology Lab	Pathogen Discovery, Respiratory Viruses Branch, Division of Viral Diseases, Centers for Disease Control and Prevention	Anna Uehara; Clinton R. Paden; Haibin Wang; Jing Zhang; Krista Queen; Peter Cook; Suxiang Tong; Yan Li; Ying Tao
EPI_ISL_778842, EPI_ISL_778865 to 778866	Clinical Pathology Labs	Centers for Disease Control and Prevention Division of Viral Diseases, Pathogen Discovery	Ying Tao Yan Li Jing Zhang Krista Queen Anna Uehara Peter Cook Clinton R. Paden Haibin Wang Suxiang Tong
EPI_ISL_735511	Clinical Pathology and Microbiology, San Gallicano Dermatologic Institute IRCCS	National Institute for Infectious Diseases, INMI, "L. Spallanzani" IRCCS	A. Di Caro; B. Bartolini; C.E.M Gruber; E. Giombini; F. Carletti; F. Ensoli; F. Messina; F. Pimpinelli; M. Rueca; M.R. Capobianchi
EPI_ISL_578697, EPI_ISL_605799 to 605816	Clinical Virology Laboratory, Institute of Liver and Biliary Sciences	ILBS - IGIB	Abhishek Padhi; Ekta Gupta; Jaswinder Singh Maras; Reshu Agarwal; Sheetalnath Rooge; Shridhar Sivasubbu; Shvetank Sharma; Vinod Scaria
EPI_ISL_447311 to 447330	Clinical Virology Laboratory, Soroka Medical Center and the Faculty of Health Sciences, Ben-Gurion University of the Negev	Stern Lab	Stern Lab
EPI_ISL_447331 to 447382, EPI_ISL_447407 to 447416	Clinical Virology Unit, Hadassah Hebrew University Medical Center	Stern Lab	Stern Lab
EPI_ISL_1247019	Clinical microbiology lab, RSPTN Universitas Hasanuddin	Clinical microbiology lab, RSPTN Universitas Hasanuddin	Gita Vita Soraya; Handayani; Muhammad Nasrum Massi; Muhammad Yusuf Usman; Rizalinda Sjahril; Rufika Shari; Triwibawa
EPI_ISL_753262 to 753692, EPI_ISL_1295564 to 1295584	Clinical virology Laboratory, Children's Hospital Los Angeles	Center for Personalized Medicine, Children's Hospital Los Angeles	Alex Ryutov; Alexander R. Judkins; Ananthanarayanan Govindarajan; David Ruble; Dejerianne Ostrow; Dennis Maglinte; Gai et al; Jaclyn A. Biegel; Jennifer Dien Bard; Jennifer Han; Lishuang Shen; Moiz Bootwalla; Timothy J. Triche; Utsav Pandey; Xiaowu Gai
EPI_ISL_418230	Clinique AVERAY LA BROUSTE, Med. Polyvalente	National Reference Center for Viruses of Respiratory Infections, Institut Pasteur, Paris	Angela Brisebarre; Elsa Ngwem; Etienne Simon-Lorière; Flora Donati; Marion Barbet; Maud Vanpeene; Mélanie Albert; Méline Bizard; Sylvie Behillil; Sylvie van der Werf; Vincent Enouf
EPI_ISL_965393 to 965397, EPI_ISL_965402, EPI_ISL_965451, EPI_ISL_1058574, EPI_ISL_1076353 to 1076355, EPI_ISL_1076358, EPI_ISL_1076361 to 1076363, EPI_ISL_1076432 to 1076433, EPI_ISL_1076436, EPI_ISL_1165841 to 1165842, EPI_ISL_1171561, EPI_ISL_1241698 to 1241699, EPI_ISL_1241773			
see above	Clinique N.D de Grâce Gosselies	GIGA Medical Genomics	Bouchra Boujemla; Cécile Meex; Keith Durkin; Maria Artesi; Marie-Pierre Hayette; Nathalie Renotte; Pierrette Melin; Raphaël Boreux; Sébastien Bontems; Vincent Bours
EPI_ISL_1058533	Clinique St Joseph Saint Vith	GIGA Medical Genomics	Bouchra Boujemla; Cécile Meex; Keith Durkin; Maria Artesi; Marie-Pierre Hayette; Nathalie Renotte; Pierrette Melin; Raphaël Boreux; Sébastien Bontems; Vincent Bours
EPI_ISL_1171513 to 1171517, EPI_ISL_1171520, EPI_ISL_1171526, EPI_ISL_1171567	Cliniques Cud-Luxembourg	GIGA Medical Genomics	Bouchra Boujemla; Cécile Meex; Keith Durkin; Maria Artesi; Marie-Pierre Hayette; Nathalie Renotte; Pierrette Melin; Raphaël Boreux; Sébastien Bontems; Vincent Bours
EPI_ISL_949248	Cliniques Saint-Pierre Ottignies	UCLouvain/IREC/MBLG	Benoit Kabamba Mukadi; Jean Ruelle; Lysa Pinsmaye
EPI_ISL_1113066 to 1113074, EPI_ISL_1123370	Cliniques Sud-Luxembourg	GIGA Medical Genomics	Bouchra Boujemla; Cécile Meex; Keith Durkin; Maria Artesi; Marie-Pierre Hayette; Nathalie Renotte; Pierrette Melin; Raphaël Boreux; Sébastien Bontems; Vincent Bours

EPI_ISL_949244 to 949247, EPI_ISL_949249 to 949253, EPI_ISL_1029957 to 1029973	Cliniques universitaires Saint-Luc	UCLouvain/IREC/MBLG	Benoit Kabamba Mukadi; Jean Ruelle; Lysa Pinsmaye
EPI_ISL_862652 to 862653	Clínica Universidad de Navarra	Instituto de Salud Carlos III	A. Monzón; F. Casas; I. Fernández, M.; I. Jiménez; Iglesias-Caballero; M. Pozo; M. Cuesta; M. González-Esguevillas; M. Zaballos; M.Camarero; P. Jiménez; S. Juliá; S. Molinero Calamita; S. Varona
EPI_ISL_452472 to 452543, EPI_ISL_538082 to 538117, EPI_ISL_538608 to 538612, EPI_ISL_1179759, EPI_ISL_1179761 to 1179762, EPI_ISL_1179764, EPI_ISL_1179766, EPI_ISL_1179768, EPI_ISL_1179770, EPI_ISL_1179772 to 1179773, EPI_ISL_1179775, EPI_ISL_1179777 to 1179778, EPI_ISL_1179780, EPI_ISL_1179782, EPI_ISL_1179784 to 1179785, EPI_ISL_1179788 to 1179789, EPI_ISL_1179791, EPI_ISL_1179793, EPI_ISL_1179795, EPI_ISL_1179797 to 1179798, EPI_ISL_1179800, EPI_ISL_1179808 to 1179809, EPI_ISL_1179811, EPI_ISL_1179813, EPI_ISL_1179817			
see above	Clínica Universidad de Navarra. Servicio de Enfermedades Infecciosas y Microbiología clínica	SeqCOVID-SPAIN consortium/IBV(CSIC)	Jose Luis del Pozo and SeqCOVID-SPAIN consortium; Mirian Fernández-Alonso
EPI_ISL_745128, EPI_ISL_745153	CoVid EC Nelson Mandela Bay Metro	National Health Laboratory Service (NHLS), Tygerberg	Bronwyn Kleinhans; Eduan Wilkindon; Gert van Zyl; Houriiyah Tegally; Kayla Delaney; Susan Engelbrecht; Tulio de Oliveira; Wolfgang Preiser
EPI_ISL_640062 to 640063, EPI_ISL_1040819 to 1040821	CoVid WC Cape Town Metro	NHLS/UCT	Arash Iranzadeh; Bruna Galvao; Carolyn Williamson; Deelan Doolabh; Diana Hardie; Innocent Mudau; Kruger Marais; Lynn Tyers; Marvin Hsiao; Stephen Korsman
EPI_ISL_1015361	Coast Diagnostics	Synergy Laboratories	Megan Cornwell
EPI_ISL_1191727	Coast Diagnostics	Synergy Laboratories, Inc.	Tina Louise Sutton-Young
EPI_ISL_479662, EPI_ISL_591070	College of Veterinary Medicine, Chungnam National University	College of Veterinary Medicine, Chungnam National University	S.H. and Jang, Y.; Seo; Seo, S.
EPI_ISL_677241, EPI_ISL_751800	Colorado Department of Public Health and Environment	Colorado Department of Public Health & Environment	Diana Ir; Emily A. Travanty; Laura Bankers; Molly C. Hetherington-Rauth; Molly Hetherington-Rauth; Sarah Elizabeth Totten; Shannon Ely; Shannon R. Matzinger
EPI_ISL_1169609 to 1169817, EPI_ISL_1233975 to 1234044, EPI_ISL_1234046 to 1234381, EPI_ISL_1300053 to 1300174	Colorado Department of Public Health and Environment	Colorado Department of Public Health and Environment	Diana Ir; Emily A. Travanty; Laura Bankers; Molly C. Hetherington-Rauth; Sarah Elizabeth Totten; Shannon Ely; Shannon R. Matzinger
EPI_ISL_677242 to 677319, EPI_ISL_677637 to 677657, EPI_ISL_710130 to 710270, EPI_ISL_710272 to 710373, EPI_ISL_771152 to 771187, EPI_ISL_771189 to 771193, EPI_ISL_771195 to 771204, EPI_ISL_771206 to 771223, EPI_ISL_771225 to 771233, EPI_ISL_771245 to 771360, EPI_ISL_857043 to 857050, EPI_ISL_954818 to 954942, EPI_ISL_954944 to 955060, EPI_ISL_955131 to 955135, EPI_ISL_983705 to 983862, EPI_ISL_1038842 to 1038923, EPI_ISL_1097446 to 1097643			
see above	Colorado Department of Public Health and Environment	Colorado Department of Puplic Health and Environment	Diana Ir; Emily A. Travanty; Laura Bankers; Molly C. Hetherington-Rauth; Molly Hetherington-Rauth; Sarah Elizabeth Totten; Shannon Ely; Shannon R. Matzinger
EPI_ISL_765574 to 765582, EPI_ISL_872745 to 872777	Colorado Mesa University	Infectious Disease Program, Broad Institute of Harvard and MIT	A.E.; Adams, G.; Anahtar, M.; B.L.; B.W.; Bauer, M.; Birren; Branda, J.; Bronson, A.; Carter, A.; Cerrato, F.; Chaluvadi, S.; Chapman; Cusick, C.; D.J.; DeRuff, K.; E. and Sabeti; Flowers, K.; Gallagher, G.; Gladden-Young, A.; Gnirke, A.; Harris, J.; J.E.; K.J.; LaRocque, R.; Lagerborg, K.; Lemieux; Lin; Loreth, C.; MacInnis; Marshall, J.; Neumann, A.; Normandin, E.; ONeill, M.; P.C.; Park; Pierce, V.; Reilly, S.; Rosenberg; Rudy, M.; Ryan, E.; S.B.; Shaw, B.; Siddle; Slater, D.; Smole, S.; Tomkins-Tinch, C.; Turbett, S.; and Sabeti
EPI_ISL_527401 to 527488	Colorado State University - Ebel Lab	Colorado State University - Ebel Lab	Greg Ebel et al.
EPI_ISL_804980, EPI_ISL_896216 to 896217, EPI_ISL_896227, EPI_ISL_896231 to 896234, EPI_ISL_896236 to 896246, EPI_ISL_896382 to 896384, EPI_ISL_896386, EPI_ISL_896389, EPI_ISL_896391 to 896395, EPI_ISL_896422 to 896446, EPI_ISL_936036 to 936045, EPI_ISL_936063 to 936066, EPI_ISL_936069, EPI_ISL_936082, EPI_ISL_936086, EPI_ISL_936089, EPI_ISL_936092 to 936093, EPI_ISL_1015653 to 1015712, EPI_ISL_1016111 to 1016117, EPI_ISL_1016260 to 1016261, EPI_ISL_1016267 to 1016268, EPI_ISL_1016270, EPI_ISL_1016281 to 1016284, EPI_ISL_1016287 to 1016297, EPI_ISL_1016323 to 1016326, EPI_ISL_1016328 to 1016331, EPI_ISL_1020487 to 1020550, EPI_ISL_1226456, EPI_ISL_1226461, EPI_ISL_1226463, EPI_ISL_1226466, EPI_ISL_1226502 to 1226503, EPI_ISL_1226512, EPI_ISL_1226518, EPI_ISL_1226596, EPI_ISL_1226599, EPI_ISL_1226637 to 1226638, EPI_ISL_1226671, EPI_ISL_1226674, EPI_ISL_1226728, EPI_ISL_1226743, EPI_ISL_1226759, EPI_ISL_1226765, EPI_ISL_1226800 to 1226807, EPI_ISL_1226823 to 1226824, EPI_ISL_1226874 to 1226875, EPI_ISL_1226901 to 1226906, EPI_ISL_1226926, EPI_ISL_1226930, EPI_ISL_1226988 to 1226997, EPI_ISL_1227002 to 1227004, EPI_ISL_1227068 to 1227069, EPI_ISL_1227088 to 1227089, EPI_ISL_1227096, EPI_ISL_1227163, EPI_ISL_1227166, EPI_ISL_1227222, EPI_ISL_1227237, EPI_ISL_1227283, EPI_ISL_1227289 to 1227290, EPI_ISL_1227293, EPI_ISL_1227295 to 1227296, EPI_ISL_1227304 to 1227305, EPI_ISL_1227314, EPI_ISL_1227320, EPI_ISL_1227326, EPI_ISL_1227357, EPI_ISL_1227360 to 1227363, EPI_ISL_1227401 to 1227402, EPI_ISL_1227406 to 1227409, EPI_ISL_1227416, EPI_ISL_1227435, EPI_ISL_1227536, EPI_ISL_1227675, EPI_ISL_1227895 to 1227902, EPI_ISL_1227904 to 1227908, EPI_ISL_1227910, EPI_ISL_1227913 to 1227919, EPI_ISL_1227921 to 1227931, EPI_ISL_1227934, EPI_ISL_1228158, EPI_ISL_1228253, EPI_ISL_1228509, EPI_ISL_1228514, EPI_ISL_1228529, EPI_ISL_1228543 to 1228544, EPI_ISL_1228576			
see above	Columbia University Irving Medical Center	Wadsworth Center, New York State Department of Health	Alexis Russel; Daryl M. Lamson; Erasmus Schneider; Erica Lasek-Nesselquist; John Kelly; Jonathan Plitnick; Kirsten St. George; Matthew Shudt; Melissa A Leisner; Navjot Singh
EPI_ISL_794283 to 794307, EPI_ISL_802777 to 802789	Columbia University Irving Medical Center	Wadsworth Center, New York State Department.of Health	Alexis Russel; Daryl M. Lamson; Erasmus Schneider; Erica Lasek-Nesselquist; John Kelly; Jonathan Plitnick; Kirsten St. George; Matthew Shudt; Melissa A Leisner; Navjot Singh; Sara Griesemer
EPI_ISL_1169483 to 1169486, EPI_ISL_1169493 to 1169497, EPI_ISL_1169499, EPI_ISL_1169502, EPI_ISL_1169504 to 1169544, EPI_ISL_1169549	Commonwealth Healthcare Center	Genomics and Discovery, Respiratory Viruses Branch, Division of Viral Diseases, Centers for Disease Control and Prevention	Anna Montmayeur; Anna Uehara; Ben L. Rambo-Martin; Clinton R. Paden; Dhwani Batra; Haibin Wang; Jasmine Padilla; Jing Zhang; Justin Lee; Krista Queen; Lori Rowe; Mark Burroughs; Mili Sheth; Peter W. Cook; Rachel Marine; Sarah Nobles; Suxiang Tong; Yan Li; Ying Tao
EPI_ISL_510081 to 510082, EPI_ISL_523824, EPI_ISL_539822 to 539823	Communicable Disease Branch	Hong Kong Department of Health	Alan K.L. Tsang; Chan Rickjason C.W.; Dominic N.C. Tsang; Edman T.K. Lam; Lam Edman T.K.; Mak Gannon C.K.; Peter C.W. Yip; Rickjason C.W. Chan; Tsang Dominic N.C.
EPI_ISL_632250 to 632285, EPI_ISL_632899 to 632907, EPI_ISL_678261 to 678272, EPI_ISL_681298 to 681319, EPI_ISL_682299 to 682322, EPI_ISL_684028 to 684036, EPI_ISL_1233891			
see above	Communicable Disease Laboratory, Public Health Directorate	Communicable Disease Laboratory, Public Health Directorate	AlAbbas, Z.; AlHujairi, Z.; AlTaif, Z.; AlWasti, H.; Alhujairi, Z.; Altaif, Z.; Alwasti, H.; Marhoon, A.
EPI_ISL_485401, EPI_ISL_510528, EPI_ISL_510531	Communicable Disease Laboratory, Public Health Directorate	Communicable Disease Laboratory, Public Health Directorate	Al Wasti; Al-Taif; Al-Wasti, H.; AlWasti, H.; Altaif, Z.; H. and AlTaif, Z.; Shehab, F.; Z. and Shehab, F.; Zaed, A.
EPI_ISL_1055211 to 1055212, EPI_ISL_1055216, EPI_ISL_1055220, EPI_ISL_1055225, EPI_ISL_1055239, EPI_ISL_1055250, EPI_ISL_1055252 to 1055253			
see above	Community Testing Centre	Hong Kong Department of Health	Chan Rickjason C.W.; Lam Edman T.K.; Mak Gannon C.K.; Tsang Dominic N.C.
EPI_ISL_862544, EPI_ISL_862553 to 862555	Compejo Hospitalario Xeral-Calde	Instituto de Salud Carlos III	A. Monzón; F. Casas; I. Alonso, P.; I. Jiménez; Iglesias-Caballero; M. Pozo; M. Cuesta; M. González-Esguevillas; M. Zaballos; M.Camarero; P. Jiménez; S. Juliá; S. Molinero Calamita; S. Varona
EPI_ISL_913038	Complejo Asistencial Universitario de Burgos	Instituto de Salud Carlos III	A. Monzón; F. Casas; I. Jiménez; I. Megías, G.; Iglesias-Caballero; M. Camarero; P. Zaballos; S. Cuesta; S. Pozo; S. Sandonís; S. Varona; V. Vázquez
EPI_ISL_913052 to 913053, EPI_ISL_954785	Complejo Asistencial Universitario de Salamanca	Instituto de Salud Carlos III	A. Monzón; F. Casas; I. Jiménez; I. Ávila, A.; Iglesias-Caballero; M. Camarero; P. Zaballos; S. Cuesta; S. Pozo; S. Sandonís; S. Varona; V. Vázquez
EPI_ISL_455328 to 455331, EPI_ISL_539563 to 539564, EPI_ISL_578194	Complejo Hospitalario Universitario La Coruna	Instituto de Salud Carlos III	A. Monzón; F. Casas; I; I. Jiménez; Iglesias-Caballero; J. López; M. Camarero; M. Cuesta; M. González-Esguevillas; M. Molinero Calamita; M. Zaballos; M.A Canizares; M.I Paz; P. Jiménez; S. Juliá; S. Pozo; S. Varona
EPI_ISL_954789, EPI_ISL_954794, EPI_ISL_954800, EPI_ISL_954807 to 954808	Complejo Hospitalario Universitario La Coruña	Instituto de Salud Carlos III	A. Monzón; F. Casas; I. Jiménez; I. Peña, F.; Iglesias-Caballero; M. Camarero; P. Zaballos; S. Cuesta; S. Pozo; S. Sandonís; S. Varona; V. Vázquez
EPI_ISL_691684	Complejo Hospitalario Universitario de Albacete	Instituto de Salud Carlos III	A. Monzón; F. Casas; I. Jiménez; I. Martínez, E.; Iglesias-Caballero; M. Camarero; M. Cuesta; M. González-Esguevillas; M. Pozo; M. Zaballos; P. Jiménez; S. Juliá; S. Molinero Calamita; S. Varona
EPI_ISL_474798 to 474831, EPI_ISL_474837 to 474840, EPI_ISL_474847 to 474849, EPI_ISL_474853, EPI_ISL_474901 to 474906, EPI_ISL_474919, EPI_ISL_474921, EPI_ISL_474933, EPI_ISL_474940 to 474941, EPI_ISL_474945 to 474947, EPI_ISL_474951 to 474956, EPI_ISL_500157 to 500159, EPI_ISL_500166, EPI_ISL_500207, EPI_ISL_500218			
see above	Complejo Hospitalario Universitario de Albacete	SeqCOVID-SPAIN consortium/IBV(CSIC)	Caridad Sainz de Baranda Camino and SeqCOVID-SPAIN consortium; Encarnacion Simarro Córdoba; Julia Lozano Serra; Lorena Robles Fonseca; Monica Parra Grandes
EPI_ISL_455333, EPI_ISL_913024, EPI_ISL_913495	Complejo Hospitalario Universitario de Santiago	Instituto de Salud Carlos III	A. Monzón; F. Casas; I; I. Jiménez; I. Peña, F.; Iglesias-Caballero; J. Llovo; M. Camarero; M. Cuesta; M. González-Esguevillas; M. Molinero Calamita; M. Zaballos; P. Jiménez; P. Zaballos; S. Cuesta; S. Juliá; S. Pozo; S. Sandonís; S. Varona; V. Vázquez
EPI_ISL_537380 to 537381	Complejo Hospitalario Universitario de Vigo	SeqCOVID-SPAIN consortium/IBV(CSIC)	Benito Regueiro and SeqCOVID-SPAIN consortium

EPI_ISL_913039	Complejo Hospitalario Xeral-Calde	Instituto de Salud Carlos III	A. Monzón; F. Casas; I. Alonso, P.; I. Jiménez; Iglesias-Caballero; M. Camarero; P. Zaballos; S. Cuesta; S. Pozo; S. Sandonís; S. Varona; V. Vázquez
EPI_ISL_539569 to 539572, EPI_ISL_862550, EPI_ISL_862552, EPI_ISL_862570 to 862572, EPI_ISL_862645 to 862651, EPI_ISL_954784, EPI_ISL_954786, EPI_ISL_954791, EPI_ISL_954802, EPI_ISL_954804, EPI_ISL_965933, EPI_ISL_965937, EPI_ISL_965942 to 965944, EPI_ISL_965950, EPI_ISL_965956			
see above	Complejo Hospitalario de Navarra	Instituto de Salud Carlos III	A. Monzón; F. Casas; I; I. Ezpeleta, C.; I. Jiménez; I. Navascués, A.; Iglesias-Caballero; J. López; M. Pozo; M. Camarero; M. Cuesta; M. González-Esguevillas; M. Molinero Calamita; M. Sandonís; M. Zaballos; M.Camarero; P. Jiménez; P. Zaballos; S. Camarero; S. Cuesta; S. Iglesias-Caballero; S. Juliá; S. Molinero Calamita; S. Pozo; S. Sandonís; S. Varona; Sandonís; V. Vázquez
EPI_ISL_455334 to 455335, EPI_ISL_578195	Complejo Hospitalario de Orense	Instituto de Salud Carlos III	A. Monzón; F. Casas; I; I. Jiménez; Iglesias-Caballero; M. Camarero; M. Cuesta; M. García; M. González-Esguevillas; M. Molinero Calamita; M. Paz; M. Zaballos; P. Jiménez; S. Juliá; S. Pozo; S. Varona
EPI_ISL_583497	Complexo Hospitalar Ouro Verde de Campinas	Instituto Adolfo Lutz, Interdiciplinary Procedures Center, Strategic Laboratory	Claudia Regina Gonçalves; Claudio Tavares Sacchi; Erica Valessa Ramos Gomes; Karoline Rodrigues Campos
EPI_ISL_861683	Complexo Hospitalar Padre Bento de Guarulhos	Instituto Adolfo Lutz, Interdiciplinary Procedures Center, Strategic Laboratory	Claudia Regina Gonçalves; Claudio Tavares Sacchi; Erica Valessa Ramos Gomes; Karoline Rodrigues Campos
EPI_ISL_1121323 to 1121324	Complexo Hospitalar Padre Bentode Guarulhos	Instituto Adolfo Lutz, Interdiciplinary Procedures Center, Strategic Laboratory	Caio Vinicius Dias Lopes; Claudia Regina Gonçalves; Claudio Tavares Sacchi; Erica Valessa Ramos Gomes; Karoline Rodrigues Campos
EPI_ISL_523970	Conjunto Hospitalar do Mandaqui	Instituto Adolfo Lutz, Interdiciplinary Procedures Center, Strategic Laboratory	Claudia Regina Gonçalves; Claudio Tavares Sacchi; Erica Valessa Ramos Gomes
EPI_ISL_872191 to 872192	Conjunto Hospitalar do Mandaqui de Sao Paulo	Instituto Adolfo Lutz, Interdiciplinary Procedures Center, Strategic Laboratory	Ana Lucia de Carvalho Avelino; Claudia Regina Gonçalves; Claudio Tavares Sacchi; Erica Valessa Ramos Gomes; Fabiana Cristina Pereira dos Santos; Karoline Rodrigues Campos; Katia Correa de Oliveira Santos
EPI_ISL_1182027 to 1182030, EPI_ISL_1225640, EPI_ISL_1225654, EPI_ISL_1225661, EPI_ISL_1225722, EPI_ISL_1225728, EPI_ISL_1292971 to 1292978, EPI_ISL_1293140			
see above	Connecticut DPH	Grubaugh Lab - Yale School of Public Health	Anderson Brito; Annie Watkins; Anthony Muyombwe; Chaney Kalinich; Chantal Vogels; Isabell Ott; Jafar Razeq; Jessica Rothman; Joseph Fauver; Mallery Breban; Mary Petrone; Nathan Grubaugh; Randy Downing; Stephen M. Bart; Tara Alpert
EPI_ISL_729735 to 729736, EPI_ISL_729741, EPI_ISL_729743 to 729750, EPI_ISL_729752 to 729756, EPI_ISL_729758 to 729760, EPI_ISL_729765, EPI_ISL_729772 to 729773, EPI_ISL_729778 to 729780, EPI_ISL_729786, EPI_ISL_729789, EPI_ISL_729792, EPI_ISL_730053 to 730061, EPI_ISL_802502 to 802505, EPI_ISL_847834, EPI_ISL_847838, EPI_ISL_912163 to 912165, EPI_ISL_912185, EPI_ISL_1168737			
see above	Connecticut Department of Health	Grubaugh Lab - Yale School of Public Health	Adam Moore; Akiko Iwasaki; Albert Ko; Alice Lu; Allison Nelson; Anderson Brito; Anne Wyllie; Annie Watkins; Arnau Casanovas; Catherine Muenker; Chaney Kalinich; Chantal Vogels; Charlese Dela Cruz; Isabel Ott; Joseph Fauver; Mallery Breban; Maria Tokuyama; Mary Petrone; Nathan Grubaugh; Patrick Wong; Peiwen Lu; Richard Martinello; Saad Omer; Shelli Farhadian; Tara Alpert
EPI_ISL_1091805, EPI_ISL_1091808 to 1091809, EPI_ISL_1091811, EPI_ISL_1091821 to 1091823, EPI_ISL_1091828 to 1091829, EPI_ISL_1091835, EPI_ISL_1091838, EPI_ISL_1091840 to 1091841, EPI_ISL_1091843 to 1091848, EPI_ISL_1091854 to 1091857, EPI_ISL_1091865, EPI_ISL_1112933, EPI_ISL_1112940 to 1112943, EPI_ISL_1112945, EPI_ISL_1239849			
see above	Connecticut Department of Public Health	Grubaugh Lab - Yale School of Public Health	Anderson Brito; Anne Wyllie; Annie Watkins; Chaney Kalinich; Chantal Vogels; Isabel Ott; Joseph Fauver; Mallery Breban; Mary Petrone; Nathan Grubaugh; Tara Alpert
EPI_ISL_416416 to 416419, EPI_ISL_435710 to 435719, EPI_ISL_527738, EPI_ISL_527761 to 527785, EPI_ISL_792557	Connecticut State Department of Public Health	Grubaugh Lab - Yale School of Public Health	Adam Moore; Akiko Iwasaki; Albert Ko; Alice Lu; Allison Nelson; Anderson Brito; Anne Wyllie; Anthony Muyombwe; Arnau Casanovas; Catherine Muenker; Chaney Kalinich; Chantal Vogels; Charlese Dela Cruz; Cole Jensen; Ellen Foxman; Isabel Ott; Jafar Razeq; Joseph Fauver; Maria Tokuyama; Marie-Louise Landry; Mary Petrone; Nagarjuna Cheemarla; Nathan Grubaugh; Patrick Wong; Peiwen Lu; Richard Martinello; Saad Omer; Shelli Farhadian; Tara Alpert
EPI_ISL_738244 to 738289, EPI_ISL_738298 to 738312, EPI_ISL_1017585 to 1017677, EPI_ISL_1067639, EPI_ISL_1067642 to 1067644, EPI_ISL_1067646 to 1067647, EPI_ISL_1067649, EPI_ISL_1067655, EPI_ISL_1067660 to 1067667, EPI_ISL_1067670 to 1067671, EPI_ISL_1067673, EPI_ISL_1067675 to 1067676, EPI_ISL_1067678 to 1067679, EPI_ISL_1067681 to 1067684, EPI_ISL_1067686 to 1067687, EPI_ISL_1067689 to 1067691, EPI_ISL_1080455, EPI_ISL_1080457 to 1080459, EPI_ISL_1080462, EPI_ISL_1091799, EPI_ISL_1091839, EPI_ISL_1091869, EPI_ISL_1091883 to 1091890, EPI_ISL_1112935, EPI_ISL_1112938, EPI_ISL_1239847			
see above	Connecticut Veterans' Affairs Hospital	Grubaugh Lab - Yale School of Public Health	Chantal Vogels; Danielle Plank; Ellen Foxman; Isabel Ott; Joseph Fauver; Mary Petrone; Nathan Grubaugh; Shaili Gupta; Tara Alpert
EPI_ISL_536222, EPI_ISL_536288	Conseil Cri de la SSS de la Baie-James	Laboratoire de santé publique du Québec	Guillaume Bourque; Ioannis Ragoussis; Jesse Shapiro; Mark Lathrop and Michel Roger on behalf of the CoVSeQ research group; Sandrine Moreira
EPI_ISL_455327, EPI_ISL_539526 to 539530, EPI_ISL_691681	Consejeria de Sanidad y Asuntos Sociales	Instituto de Salud Carlos III	A. Monzón; F. Casas; G. Gutiérrez; I; I. Gutiérrez, G.; I. Jiménez; Iglesias-Caballero; M. Camarero; M. Cuesta; M. González-Esguevillas; M. Molinero Calamita; M. Pozo; M. Zaballos; P. Jiménez; S. Juliá; S. Molinero Calamita; S. Pozo; S. Varona
EPI_ISL_862546 to 862547, EPI_ISL_862557 to 862563, EPI_ISL_862566, EPI_ISL_862641 to 862644, EPI_ISL_913041, EPI_ISL_913059	Consejería de Sanidad y Asuntos Sociales	Instituto de Salud Carlos III	A. Monzón; F. Casas; I. Gutiérrez, G.; I. Jiménez; Iglesias-Caballero; M. Camarero; M. Cuesta; M. González-Esguevillas; M. Pozo; M. Zaballos; P. Jiménez; P. Zaballos; S. Cuesta; S. Juliá; S. Molinero Calamita; S. Pozo; S. Sandonís; S. Varona; V. Vázquez
EPI_ISL_965921, EPI_ISL_965938, EPI_ISL_965952, EPI_ISL_965957	Consejería de Sanidad y Asuntos Sociales de Castilla La Mancha	Instituto de Salud Carlos III	A. Monzón; F. Casas; I. Gutiérrez, G.; I. Jiménez; Iglesias-Caballero; M. Camarero; M. Sandonís; P. Zaballos; S. Camarero; S. Cuesta; S. Iglesias-Caballero; S. Pozo; S. Varona; Sandonís; V. Camarero; V. Vázquez; Vázquez
EPI_ISL_672488 to 672489, EPI_ISL_1030269, EPI_ISL_1030281 to 1030282, EPI_ISL_1030302 to 1030303, EPI_ISL_1030306 to 1030307, EPI_ISL_1030309 to 1030311, EPI_ISL_1030313 to 1030329, EPI_ISL_1030331 to 1030390, EPI_ISL_1234538, EPI_ISL_1234540, EPI_ISL_1234546, EPI_ISL_1234552 to 1234553, EPI_ISL_1234555 to 1234556, EPI_ISL_1234558 to 1234559, EPI_ISL_1234563, EPI_ISL_1234569 to 1234570, EPI_ISL_1234572 to 1234573, EPI_ISL_1234577, EPI_ISL_1234579 to 1234580, EPI_ISL_1234583, EPI_ISL_1234585, EPI_ISL_1234587, EPI_ISL_1234590, EPI_ISL_1234596, EPI_ISL_1234598, EPI_ISL_1234602, EPI_ISL_1234607, EPI_ISL_1234610, EPI_ISL_1234614 to 1234616, EPI_ISL_1234618, EPI_ISL_1234622, EPI_ISL_1234626, EPI_ISL_1234628 to 1234632, EPI_ISL_1234636, EPI_ISL_1234642 to 1234643, EPI_ISL_1234647, EPI_ISL_1234654 to 1234655, EPI_ISL_1234659, EPI_ISL_1234662, EPI_ISL_1234664, EPI_ISL_1234674 to 1234675, EPI_ISL_1234680, EPI_ISL_1234682, EPI_ISL_1234684 to 1234686, EPI_ISL_1234688, EPI_ISL_1234690 to 1234693, EPI_ISL_1234696 to 1234697, EPI_ISL_1234702, EPI_ISL_1234705, EPI_ISL_1234707 to 1234710, EPI_ISL_1234726 to 1234727, EPI_ISL_1234736, EPI_ISL_1234738, EPI_ISL_1234741, EPI_ISL_1234745 to 1234746, EPI_ISL_1234749, EPI_ISL_1234756 to 1234757, EPI_ISL_1234763 to 1234764, EPI_ISL_1234768, EPI_ISL_1234770, EPI_ISL_1234772, EPI_ISL_1234775, EPI_ISL_1234777, EPI_ISL_1234783, EPI_ISL_1235061 to 1235062, EPI_ISL_1235064 to 1235067, EPI_ISL_1235069, EPI_ISL_1235071 to 1235073, EPI_ISL_1235076, EPI_ISL_1235078, EPI_ISL_1235080 to 1235082, EPI_ISL_1235085 to 1235090, EPI_ISL_1235096, EPI_ISL_1235100 to 1235101, EPI_ISL_1235103, EPI_ISL_1235105 to 1235106, EPI_ISL_1235108 to 1235110, EPI_ISL_1235113 to 1235114, EPI_ISL_1235117, EPI_ISL_1235120, EPI_ISL_1235129 to 1235131, EPI_ISL_1235134, EPI_ISL_1235137, EPI_ISL_1235139, EPI_ISL_1235143, EPI_ISL_1235145 to 1235146, EPI_ISL_1235151, EPI_ISL_1235153 to 1235157, EPI_ISL_1235159 to 1235161, EPI_ISL_1235163 to 1235164, EPI_ISL_1235166 to 1235170, EPI_ISL_1235172 to 1235174, EPI_ISL_1235177, EPI_ISL_1235179 to 1235180, EPI_ISL_1235184, EPI_ISL_1235187, EPI_ISL_1235189, EPI_ISL_1235191, EPI_ISL_1235196, EPI_ISL_1235200 to 1235202, EPI_ISL_1235204 to 1235205, EPI_ISL_1235207 to 1235209, EPI_ISL_1235211, EPI_ISL_1235214 to 1235216, EPI_ISL_1235218, EPI_ISL_1235221, EPI_ISL_1235224 to 1235225, EPI_ISL_1235227 to 1235228, EPI_ISL_1235231, EPI_ISL_1235233, EPI_ISL_1235237, EPI_ISL_1235239 to 1235240, EPI_ISL_1235245 to 1235247, EPI_ISL_1235250, EPI_ISL_1235253, EPI_ISL_1235257 to 1235262, EPI_ISL_1235264 to 1235268, EPI_ISL_1235271, EPI_ISL_1235275, EPI_ISL_1235277 to 1235278, EPI_ISL_1235280, EPI_ISL_1235283 to 1235284, EPI_ISL_1235286, EPI_ISL_1235288, EPI_ISL_1235293, EPI_ISL_1235296 to 1235297, EPI_ISL_1235300 to 1235302, EPI_ISL_1235304 to 1235306, EPI_ISL_1235311, EPI_ISL_1235315 to 1235316, EPI_ISL_1235319 to 1235321, EPI_ISL_1235323 to 1235334, EPI_ISL_1235336 to 1235337, EPI_ISL_1235340 to 1235345, EPI_ISL_1235347 to 1235350, EPI_ISL_1235352 to 1235353, EPI_ISL_1235355 to 1235358			
see above	Contra Costa County Public Health Lab	Chan-Zuckerberg Biohub	CZB Cliahub Consortium
EPI_ISL_468615 to 468655, EPI_ISL_548491 to 548492, EPI_ISL_548495, EPI_ISL_548499, EPI_ISL_548501, EPI_ISL_548506 to 548508, EPI_ISL_548510, EPI_ISL_548515 to 548516, EPI_ISL_548520, EPI_ISL_548524, EPI_ISL_548527 to 548528, EPI_ISL_548531, EPI_ISL_548534 to 548535, EPI_ISL_548537 to 548540, EPI_ISL_548545, EPI_ISL_548548 to 548549, EPI_ISL_548552, EPI_ISL_548554, EPI_ISL_548556 to 548558, EPI_ISL_548561, EPI_ISL_548564 to 548567, EPI_ISL_548569, EPI_ISL_548572 to 548575, EPI_ISL_548577, EPI_ISL_548579			
see above	Contra Costa Public Health Lab	Chan-Zuckerberg Biohub	CZB Cliahub Consortium
EPI_ISL_640015, EPI_ISL_640118, EPI_ISL_700414, EPI_ISL_700441, EPI_ISL_700467, EPI_ISL_700488, EPI_ISL_700492, EPI_ISL_700496, EPI_ISL_700504, EPI_ISL_700506, EPI_ISL_700533, EPI_ISL_700536, EPI_ISL_700538 to 700539, EPI_ISL_700554, EPI_ISL_700556 to 700557, EPI_ISL_700563, EPI_ISL_700581, EPI_ISL_700587, EPI_ISL_700590, EPI_ISL_700599			
see above	Conville CDC wc CVC	NHLS/UCT	Arash Iranzadeh; Bruna Galvao; Carolyn Williamson; Deelan Doolabh; Diana Hardie; Houriiyah Tegally; Innocent Mudau; Kruger Marais; Lynn Tyers; Marvin Hsiao; Stephen Korsman
EPI_ISL_960119, EPI_ISL_960122, EPI_ISL_960127, EPI_ISL_960134	Conville CDC wc CVC	National Health Laboratory Service/UCT	Arash Iranzadeh; Bruna Galvao; Carolyn Williamson; Deelan Doolabh; Diana Hardie; Innocent Mudau; Kruger Marais; Lynn Tyers; Marvin Hsiao; Stephen Korsman
EPI_ISL_696472, EPI_ISL_696492, EPI_ISL_696508 to 696509, EPI_ISL_696512	Conville CDC wc CVC & NHLS/UCT	KRISP, KZN Research Innovation and Sequencing Platform	Arash Iranzadeh; Bruna Galvao; Carolyn Williamson; Deelan Doolabh; Diana Hardie; Emanuel James San; Houriiyah Tegally; Innocent Mudau; Jennifer Giandhari; Kruger Marais; Lynn Tyers; Marvin Hsiao; Stephen Korsman; Sureshnee Pillay; Tulio de Oliveira
EPI_ISL_1147265 to 1147266	Corona-Testzentrum ifp Institut für Produktqualität GmbH	Robert Koch Institute	
EPI_ISL_514751	CoronaNet Lab- TaskForce Regione Campania, CEINGE Biotecnologie Avanzate, Via G. Salvatore	CoronaNet Lab- TaskForce Regione Campania, CEINGE Biotecnologie Avanzate, Via G. Salvatore	Asadzadeh, F.; Atripaldi, L.; Bianchi, M.; Boccia, A.; Borriello, G.; Brandi, S.; Castaldo, G.; Cerino, R.; Chiariotti, L.; Comegna, M.; Ferrucci, V.; Fusco, G.; H.Y.; J.H.; J.M.; Jung; K.S. and Kim; Kong, Dy.; Lee; Marrone, L.; Paolella, G.; Pascarella, S.; Siciliano, R.; Tiberio, C.; Viscardi, M.; Yun; Zollo, M.
EPI_ISL_1213563	Cotabato Regional and Medical Center	Philippine Genome Center	Alethea R. de Guzman; Anna Ong-Lim; Arianne A. Zamora; Asia Louisa U. Chong; Benedict A. Maralit; Candice Francheska B. Tambaoan; Carlo M.

			Lapid; Celia Carlos; Devon Ray Pacial; Edsel Maurice Salvaña; El King D. Morado; Eva Maria Cutiongco-de la Paz; Francis A. Tablizo; Irish Coleen A. Asin; Jaime C. Montoya; Jan Michael C. Yap; Jo-Hannah S. Llames; John Q. Wong; Joshua Gregor A. Dizon; Juan Antonio R. Magalang; Karol Sophia Agape R. Padilla; Kenneth M. Kim; Kris P. Punayan; Marc Edsel C. Ayes; Marc Jerrone R. Castro; Maria Rosario Singh-Vergeire and Cynthia P. Saloma; Maria Sofia L. Yangzon; Marissa Alejandria; Razel Nikka M. Hao; Rianna Patricia S. Cruz; Sheila Mae M. Araiza
EPI_ISL_1116461	County Emergency Clinical Hospital of Targu Mures	National Institute of Infectious Diseases-Prof. Dr. Matei Bals Molecular Diagnostics Laboratory	Andreea Tudor; Corina Casangiu; Dan Otelea; Leontina Banica; Marius Surleac; Petre Milu; Simona Paraschiv
EPI_ISL_1116460	County Emergency Clinical Hospital of Tg. Mures	National Institute of Infectious Diseases-Prof. Dr. Matei Bals Molecular Diagnostics Laboratory	Andreea Tudor; Corina Casangiu; Dan Otelea; Leontina Banica; Marius Surleac; Petre Milu; Simona Paraschiv
EPI_ISL_454635	County Of San Luis Obispo Public Health Laboratory	Chan-Zuckerberg Biohub	CZB Cliahub Consortium
EPI_ISL_468388 to 468437, EPI_ISL_548265, EPI_ISL_548268 to 548269, EPI_ISL_548280 to 548281, EPI_ISL_548284, EPI_ISL_548286, EPI_ISL_548288, EPI_ISL_548292, EPI_ISL_548294 to 548295, EPI_ISL_548304, EPI_ISL_548311, EPI_ISL_548316, EPI_ISL_548322, EPI_ISL_548325, EPI_ISL_548329 to 548330, EPI_ISL_548335 to 548336, EPI_ISL_548347, EPI_ISL_548350 to 548351, EPI_ISL_548357, EPI_ISL_548359 to 548361, EPI_ISL_548365 to 548366, EPI_ISL_548369 to 548370, EPI_ISL_548391, EPI_ISL_548425, EPI_ISL_548428, EPI_ISL_548436, EPI_ISL_548453, EPI_ISL_548457, EPI_ISL_548493, EPI_ISL_548502, EPI_ISL_548504, EPI_ISL_548517, EPI_ISL_548519, EPI_ISL_548521, EPI_ISL_548532, EPI_ISL_548542, EPI_ISL_548544, EPI_ISL_548560, EPI_ISL_548603, EPI_ISL_548631, EPI_ISL_548644, EPI_ISL_548663, EPI_ISL_548671, EPI_ISL_625547 to 625549, EPI_ISL_625623 to 625626, EPI_ISL_672028, EPI_ISL_672068, EPI_ISL_672087, EPI_ISL_672354 to 672359, EPI_ISL_738890, EPI_ISL_738990, EPI_ISL_739017, EPI_ISL_739038, EPI_ISL_739095, EPI_ISL_739102, EPI_ISL_739363, EPI_ISL_739399, EPI_ISL_739480, EPI_ISL_739492, EPI_ISL_739588, EPI_ISL_979159 to 979161, EPI_ISL_1027366 to 1027367, EPI_ISL_1185288, EPI_ISL_1185387, EPI_ISL_1185443, EPI_ISL_1234806, EPI_ISL_1234938, EPI_ISL_1235029			
see above	County of San Luis Obispo Public Health Laboratory	Chan-Zuckerberg Biohub	CZB Cliahub Consortium
EPI_ISL_437043 to 437088, EPI_ISL_444023 to 444026	County of Santa Clara Public Health	Chan-Zuckerberg Biohub	CZB Cliahub Consortium
EPI_ISL_436641 to 436683, EPI_ISL_454653 to 454689, EPI_ISL_468345 to 468356, EPI_ISL_486115 to 486119, EPI_ISL_513773 to 513788, EPI_ISL_548264, EPI_ISL_548266 to 548267, EPI_ISL_548277, EPI_ISL_548279, EPI_ISL_548282, EPI_ISL_548290, EPI_ISL_548299, EPI_ISL_548302 to 548303, EPI_ISL_548307, EPI_ISL_548310, EPI_ISL_548314, EPI_ISL_548319 to 548321, EPI_ISL_548324, EPI_ISL_548327, EPI_ISL_548331, EPI_ISL_548333, EPI_ISL_548337 to 548345, EPI_ISL_548371, EPI_ISL_548379, EPI_ISL_548384 to 548385, EPI_ISL_548388, EPI_ISL_548392, EPI_ISL_548394 to 548396, EPI_ISL_548399, EPI_ISL_548401, EPI_ISL_548403, EPI_ISL_548407, EPI_ISL_548415, EPI_ISL_548417, EPI_ISL_548419 to 548423, EPI_ISL_548426, EPI_ISL_548429, EPI_ISL_548432 to 548434, EPI_ISL_548437, EPI_ISL_548446, EPI_ISL_548448, EPI_ISL_548451, EPI_ISL_548466, EPI_ISL_548468, EPI_ISL_548470, EPI_ISL_548472, EPI_ISL_548478, EPI_ISL_548482, EPI_ISL_548494, EPI_ISL_548496 to 548498, EPI_ISL_548500, EPI_ISL_548503, EPI_ISL_548509, EPI_ISL_548511 to 548512, EPI_ISL_548514, EPI_ISL_548518, EPI_ISL_548523, EPI_ISL_548525 to 548526, EPI_ISL_548529 to 548530, EPI_ISL_548533, EPI_ISL_548536, EPI_ISL_548541, EPI_ISL_548543, EPI_ISL_548546 to 548547, EPI_ISL_548550 to 548551, EPI_ISL_548553, EPI_ISL_548559, EPI_ISL_548562 to 548563, EPI_ISL_548568, EPI_ISL_548570, EPI_ISL_548576, EPI_ISL_548578, EPI_ISL_548582 to 548584, EPI_ISL_548588, EPI_ISL_548593, EPI_ISL_548595 to 548598, EPI_ISL_548600, EPI_ISL_548602, EPI_ISL_548604 to 548607, EPI_ISL_548610, EPI_ISL_548612, EPI_ISL_548616 to 548619, EPI_ISL_548621 to 548622, EPI_ISL_548624, EPI_ISL_548629 to 548630, EPI_ISL_548632 to 548633, EPI_ISL_548636, EPI_ISL_548639, EPI_ISL_548642 to 548643, EPI_ISL_548645, EPI_ISL_548647, EPI_ISL_548649 to 548650, EPI_ISL_548652, EPI_ISL_548655 to 548659, EPI_ISL_548661, EPI_ISL_548664, EPI_ISL_548669 to 548670, EPI_ISL_548672 to 548673, EPI_ISL_548676, EPI_ISL_548678 to 548679, EPI_ISL_582851 to 582958			
see above	County of Santa Clara Public Health Department	Chan-Zuckerberg Biohub	CZB Cliahub Consortium
EPI_ISL_735494	Cox's Bazar Medical College	Central Biological Research Laboratory and Department of Biochemistry and Molecular Biology	H. M. Abdullah Al Masud; Imam Hossen; Md. Arif Hossain; Md. Imranul Hoq; Md. Khondakar Raziur Rahman; Md. Omer Faruq; Mohammad Omar Faruque; Robiul Hasan Bhuiyan; Sajib Rudra; Shanta Paul
EPI_ISL_1123266 to 1123267	Cox's Bazar Medical College	Central Biological Research Laboratory and Department of Biochemistry and Molecular Biology	H. M. Abdullah Al Masud; Imam Hossen; Md. Arif Hossain; Md. Imranul Hoq; Md. Khondakar Raziur Rahman; Md. Omer Faruq; Mohammad Omar Faruque; Robiul Hasan Bhuiyan; Sajib Rudra; Shanta Paul
EPI_ISL_735493	Cox's Bazar Medicila College	Central Biological Research Laboratory and Department of Biochemistry and Molecular Biology Central Biological Research Laboratory and Department of Biochemistry and Molecular Biology	H. M. Abdullah Al Masud; Imam Hossen; Md. Arif Hossain; Md. Imranul Hoq; Md. Khondakar Raziur Rahman; Md. Omer Faruq; Mohammad Omar Faruque; Robiul Hasan Bhuiyan; Sajib Rudra; Shanta Paul
EPI_ISL_700443	Crags Clinic wc CRG	NHLS/UCT	Arash Iranzadeh; Bruna Galvao; Carolyn Williamson; Deelan Doolabh; Diana Hardie; Houriiyah Tegally; Innocent Mudau; Kruger Marais; Lynn Tyers; Marvin Hsiao; Stephen Korsman
EPI_ISL_696480	Crags Clinic wc CRG & NHLS/UCT	KRISP, KZN Research Innovation and Sequencing Platform	Arash Iranzadeh; Bruna Galvao; Carolyn Williamson; Deelan Doolabh; Diana Hardie; Emanuel James San; Houriiyah Tegally; Innocent Mudau; Jennifer Giandhari; Kruger Marais; Lynn Tyers; Marvin Hsiao; Stephen Korsman; Sureshnee Pillay; Tulio de Oliveira
EPI_ISL_1055795, EPI_ISL_1056961 to 1056967, EPI_ISL_1084742, EPI_ISL_1084747 to 1084760, EPI_ISL_1091176 to 1091178, EPI_ISL_1091180, EPI_ISL_1091182 to 1091187, EPI_ISL_1091189, EPI_ISL_1091191, EPI_ISL_1091193 to 1091198, EPI_ISL_1091200, EPI_ISL_1091202 to 1091203, EPI_ISL_1091205 to 1091207, EPI_ISL_1091211 to 1091212, EPI_ISL_1091216 to 1091217, EPI_ISL_1091219 to 1091222, EPI_ISL_1091224, EPI_ISL_1091226 to 1091227, EPI_ISL_1091229, EPI_ISL_1091231 to 1091234, EPI_ISL_1091236 to 1091240, EPI_ISL_1096344 to 1096346, EPI_ISL_1109489, EPI_ISL_1109495 to 1109497, EPI_ISL_1109499, EPI_ISL_1109510 to 1109511, EPI_ISL_1109516, EPI_ISL_1109519 to 1109520, EPI_ISL_1109525, EPI_ISL_1109529, EPI_ISL_1109548, EPI_ISL_1109553, EPI_ISL_1109563, EPI_ISL_1109571, EPI_ISL_1109573, EPI_ISL_1110766, EPI_ISL_1110780 to 1110782, EPI_ISL_1110792, EPI_ISL_1110804 to 1110833, EPI_ISL_1195672 to 1195676, EPI_ISL_1195730 to 1195741, EPI_ISL_1195743, EPI_ISL_1271988 to 1271995, EPI_ISL_1272050 to 1272057, EPI_ISL_1283137, EPI_ISL_1287369 to 1287410			
see above	Croatian Institute of Public Health	Croatian Institute of Public Health	Irena Tabain; Ivana Ferenak; Vlado Stevanovi
EPI_ISL_454602	Croatian Institute of Public Health	University of Zagreb, Centre for research and knowledge transfer in biotechnology	Anamarija Slovic; Irena Tabain; Jelena Ivancic Jelecki; Tatjana Vilibic-Cavlek
EPI_ISL_693207	Cs II Doutor Antonio Vicoso Moreira de Rezende	Instituto Adolfo Lutz, Interdiciplinary Procedures Center, Strategic Laboratory	Claudia Regina Gonçalves; Claudio Tavares Sacchi; Erica Valessa Ramos Gomes; Karoline Rodrigues Campos
EPI_ISL_1123265	Cumilla Medical College	Central Biological Research Laboratory and Department of Biochemistry and Molecular Biology	H. M. Abdullah Al Masud; Imam Hossen; Md. Arif Hossain; Md. Imranul Hoq; Md. Khondakar Raziur Rahman; Md. Omer Faruq; Mohammad Omar Faruque; Robiul Hasan Bhuiyan; Sajib Rudra; Shanta Paul
EPI_ISL_1195946, EPI_ISL_1255102	Curative Inc	Curative Inc	Katie Bond; Vladimir Slepnev
EPI_ISL_1009796 to 1009812, EPI_ISL_1009814 to 1009826, EPI_ISL_1009828 to 1009867	Curative Labs, Inc.	Wyoming Public Health Laboratory	Ashley Norberg; Bailey Bowcutt; Channing Weber; Lynette Gumbleton; Noah Hull; Taylor Fearing; and Wanda Manley
EPI_ISL_935155, EPI_ISL_1058076 to 1058078, EPI_ISL_1060594 to 1060596, EPI_ISL_1167666, EPI_ISL_1299220 to 1299221, EPI_ISL_1312364, EPI_ISL_1314262 to 1314263			
see above	Cytocheck Laboratory	Kansas Health and Environmental Lab	Ben Olsen; Carissa Robertson; Jonathan Barnell; Mike Grose; Paige Drury; and Phil Adam
EPI_ISL_640029, EPI_ISL_640048, EPI_ISL_700420, EPI_ISL_700427, EPI_ISL_700448, EPI_ISL_700470, EPI_ISL_700564, EPI_ISL_700569			
see above	D'Almeida Clinic wc DAL	NHLS/UCT	Arash Iranzadeh; Bruna Galvao; Carolyn Williamson; Deelan Doolabh; Diana Hardie; Houriiyah Tegally; Innocent Mudau; Kruger Marais; Lynn Tyers; Marvin Hsiao; Stephen Korsman
EPI_ISL_1069186	D.T. Protacio Hospital	Research Institute for Tropical Medicine	Catalino Demetria; Daria Manalo; Edelwisa Mercado; Francisco Gerardo Polotan; Hannah Leah Morito; Inez Andrea Medado; John Leonard Chan; Joseph Hughes; Kirstyn Brunker; Ma Angelica Tujan; Othoniel Jan Onza
EPI_ISL_833167 to 833176	DB Diagnosticos do Brasil	Instituto Adolfo Lutz, Interdiciplinary Procedures Center, Strategic Laboratory	Claudia Regina Gonçalves; Claudio Tavares Sacchi; Erica Valessa Ramos Gomes; Karoline Rodrigues Campos
EPI_ISL_1060876, EPI_ISL_1060884, EPI_ISL_1060886 to 1060887, EPI_ISL_1060891, EPI_ISL_1060900, EPI_ISL_1060902, EPI_ISL_1060904, EPI_ISL_1060910, EPI_ISL_1060914, EPI_ISL_1060918, EPI_ISL_1060920, EPI_ISL_1060923 to 1060928, EPI_ISL_1060943, EPI_ISL_1060947, EPI_ISL_1060949, EPI_ISL_1060954, EPI_ISL_1060963, EPI_ISL_1060967, EPI_ISL_1060971 to 1060972, EPI_ISL_1060974, EPI_ISL_1060978, EPI_ISL_1060981, EPI_ISL_1060990, EPI_ISL_1060994 to 1060995, EPI_ISL_1060997, EPI_ISL_1061006, EPI_ISL_1061025, EPI_ISL_1061027, EPI_ISL_1061029			
see above	DB Diagnosticos do Brasil	Instituto de Medicina Tropical de Sao Paulo	Brazil-UK Centre for Arbovirus Discovery Diagnosis Genomics and Epidemiology (CADDE) Genomic Network - Instituto de Medicina Tropical
EPI_ISL_672664 to 672668, EPI_ISL_672670 to 672686, EPI_ISL_722134 to 722169, EPI_ISL_804814 to 804844, EPI_ISL_904018 to 904034, EPI_ISL_1084726, EPI_ISL_1084730, EPI_ISL_1084737 to 1084738, EPI_ISL_1084740			
see above	DB Diagnosticos do Brasil	Laboratório de Parasitologia Médica - Instituto de Medicina Tropical - Universidade de São Paulo	Andrew Rambaut; Brazil-UK Centre for Arbovirus Discovery Diagnosis Genomics and Epidemiology (CADDE) Genomic Network - Instituto de Medicina Tropical; CADDE Genomic Network.; CDL; Camila A. Maia da Silva; Cecília da Cunha Camilo; DB; Darlan Candido; Erika Regina Manuli; Ester C. Sabino; Flavia Cristina Sales; HEMOAM; Ingra Morales Claro; Lucas A. Moyses Franco; Maria do Perpétuo Socorro Sampaio Carvalho; Myuki Alfaia Esashika Crispim; Nelson Abrahim Fraiji; Nelson Gaburo; Nick Loman; Nuno Faria; Oliver G. Pybus; Pamela dos Santos Andrade; Renato A. Santana; Thais de Moura Coletti
EPI_ISL_476171 to 476202, EPI_ISL_476209 to 476216, EPI_ISL_476218 to 476219, EPI_ISL_476224 to 476229, EPI_ISL_476232, EPI_ISL_476278 to 476336, EPI_ISL_476350 to 476371			
see above	DB Diagnósticos do Brasil	Instituto de Medicina Tropical da Univesidade de São Paulo	Camila Alves Maia da Silva; Darlan da Silva Candido; Erika Regina Manuli; Ester Sabino; Flavia Cristina da Silva Sales; Giula Magalhaes Ferreira;

			Jaqueline Goes de Jesus; Julien Theze; Mariana Severo Ramundo; Nuno Faria; Samples: Nelson Gaburo Jr; Sequencing: Ingra Morales Claro; Thais de Moura Coletti
EPI_ISL_594460	DC Department of Forensic Sciences	Pathogen Discovery, Respiratory Viruses Branch, Division of Viral Diseases, Centers for Disease Control and Prevention	Anna Uehara; Clinton Paden; Haibin Wang; Jing Zhang; Julu Bhatnagar; Krista Queen; Suxiang Tong; Yan Li; Ying Tao
EPI_ISL_436040 to 436043	DC Public Health Lab Dept of Forensic Science	Pathogen Discovery, Respiratory Viruses Branch, Division of Viral Diseases, Centers for Disease Control and Prevention	Anna Uehara; Bettina Bankamp; Clinton R. Paden; Haibin Wang; Jing Zhang; Krista Queen; Suxiang Tong; Yan Li; Ying Tao; Zachary Weiner
EPI_ISL_1272842 to 1272855	DC Public Health Lab/ Dept. of Forensic Sciences	Centers for Disease Control and Prevention Division of Viral Diseases, Pathogen Discovery	Anna Montmayeur; Anna Uehara; Ben L. Rambo-Martin; Clinton R. Paden; Dhwani Batra; Haibin Wang; Jasmine Padilla; Jing Zhang; Justin Lee; Katie Dillon; Krista Queen; Kristen Knipe; Kristine Lacek; Lori Rowe; Mark Burroughs; Matthew Schmerer; Mili Sheth; Peter W. Cook; Rachel Marine; Sam Shepard; Sarah Nobles; Shoshona Le; Suxiang Tong; Yan Li; Ying Tao
EPI_ISL_804845 to 804880, EPI_ISL_804882 to 804944, EPI_ISL_913740 to 913747, EPI_ISL_1121222 to 1121228, EPI_ISL_1121281 to 1121302, EPI_ISL_1133106 to 1133118, EPI_ISL_1159108 to 1159125, EPI_ISL_1168442 to 1168451, EPI_ISL_1180239 to 1180249, EPI_ISL_1181948 to 1181958, EPI_ISL_1191612 to 1191623, EPI_ISL_1195477 to 1195498, EPI_ISL_1295619 to 1295637, EPI_ISL_1297198 to 1297201			
see above	DC Public Health Lab/ Dept. of Forensic Sciences	DC Public Health Lab/ Dept. of Forensic Sciences	Brittany Hamilton; Connie Maza; David Payne; Elizabeth Zelaya; Janis Doss; Jocelyn Hauser; Monica Mann; Sarah Scott; Scott Nguyen
EPI_ISL_903743, EPI_ISL_903793, EPI_ISL_903864, EPI_ISL_903922, EPI_ISL_903959, EPI_ISL_903964, EPI_ISL_903969, EPI_ISL_903973, EPI_ISL_903976			
see above	DC Public Health Lab/ Dept. of Forensic Sciences	Genomics and Discovery, Respiratory Viruses Branch, Division of Viral Diseases, Centers for Disease Control and Prevention	Anna Montmayeur; Anna Uehara; Ben L. Rambo-Martin; Clinton R. Paden; Dhwani Batra; Haibin Wang; Jasmine Padilla; Jing Zhang; Justin Lee; Krista Queen; Lori Rowe; Mark Burroughs; Mili Sheth; Peter W. Cook; Rachel Marine; Sarah Nobles; Suxiang Tong; Yan Li; Ying Tao
EPI_ISL_424852, EPI_ISL_447840, EPI_ISL_576178	DC Public Health Lab/ Dept. of Forensic Sciences	Pathogen Discovery, Respiratory Viruses Branch, Division of Viral Diseases, Centers for Disease Control and Prevention	Alison S. Laufer Halpin; Anna Uehara; Brian Lynch; Christopher A. Elkins; Clinton R. Paden; Haibin Wang; Jasmine Padilla; Jing Zhang; Justin Lee; Krista Queen; Mary S. Keckler; Peter Cook; Rachel Marine; Suxiang Tong; Yan Li; Ying Tao
EPI_ISL_1094311, EPI_ISL_1094330 to 1094332, EPI_ISL_1094341, EPI_ISL_1094343 to 1094344, EPI_ISL_1094403 to 1094409, EPI_ISL_1094851 to 1094854	DC Public Health Lab/ Dept. of Forensic Sciences	Respiratory Viruses Branch, Division of Viral Diseases, Centers for Disease Control and Prevention	Anna Montmayeur; Anna Uehara; Ben L. Rambo-Martin; Clinton R. Paden; Dhwani Batra; Haibin Wang; Jasmine Padilla; Jing Zhang; Justin Lee; Krista Queen; Lori Rowe; Mark Burroughs; Mili Sheth; Peter W. Cook; Rachel Marine; Sarah Nobles; Suxiang Tong; Yan Li; Ying Tao
EPI_ISL_1020204	DD Klinik Ciputat, South Tangerang, Banten	Biosafety Level-3 Laboratory, Indonesian Institute of Sciences (LIPI)	Anggia Prasetyoputri; Eko Wahyu Putro; Fahrurrozi; Isa Nuryana; Ratih Asmana Ningrum; Sri Swasthikawati; Sugiyono Saputra; Syam Budi Iryanto
EPI_ISL_751543, EPI_ISL_751547, EPI_ISL_751602, EPI_ISL_751605, EPI_ISL_751621, EPI_ISL_751623, EPI_ISL_751627, EPI_ISL_751631, EPI_ISL_751726			
see above	DE Public Health Laboratory	Genomics and Discovery, Respiratory Viruses Branch, Division of Viral Diseases, Centers for Disease Control and Prevention	Anna Montmayeur; Anna Uehara; Clinton R. Paden; Haibin Wang; Jing Zhang; Justin Lee; Krista Queen; Mili Sheth; Peter W. Cook; Rachel Marine; Suxiang Tong; Yan Li; Ying Tao
EPI_ISL_1094269, EPI_ISL_1094375 to 1094376, EPI_ISL_1094467 to 1094472, EPI_ISL_1094622 to 1094638	DE Public Health Laboratory	Respiratory Viruses Branch, Division of Viral Diseases, Centers for Disease Control and Prevention	Anna Montmayeur; Anna Uehara; Ben L. Rambo-Martin; Clinton R. Paden; Dhwani Batra; Haibin Wang; Jasmine Padilla; Jing Zhang; Justin Lee; Krista Queen; Lori Rowe; Mark Burroughs; Mili Sheth; Peter W. Cook; Rachel Marine; Sarah Nobles; Suxiang Tong; Yan Li; Ying Tao
EPI_ISL_1228375 to 1228377	DEPARTMENT OF VETERANS AFFAIR MEDICAL CENTER	Wadsworth Center, New York State Department of Health	Alexis Russel; Daryl M. Lamson; Erasmus Schneider; Erica Lasek-Nesselquist; John Kelly; Jonathan Plitnick; Kirsten St. George; Matthew Shudt; Melissa A Leisner; Navjot Singh
EPI_ISL_486384, EPI_ISL_486387 to 486389, EPI_ISL_486401 to 486403, EPI_ISL_486405 to 486409, EPI_ISL_515932, EPI_ISL_515934 to 515936, EPI_ISL_515945 to 515949, EPI_ISL_515954 to 515955, EPI_ISL_515957 to 515959, EPI_ISL_515961 to 515962, EPI_ISL_515965, EPI_ISL_583914			
see above	DH	Department of Neurovirology, National Institute of Mental Health and Neuroscience (NIMHANS)	Anita Desai; Chitra Pattabiraman; Harsha PK; Manjunatha Venkataswamy; Pramada Prasad; Ravi Vasanthapuram; Risha Rasheed; Shafeeq S Hameed; Vijayalakshmi Reddy
EPI_ISL_1117488, EPI_ISL_1117521	DIP PREV AVEZZANO, SERVIZIO DI IGIENE EPIDEMIOLOGIA E SANITÀ PUBBLICA	Istituto Zooprofilattico Sperimentale dell'Abruzzo e Molise "G. Caporale"	Ancora M; Calistri P; Cammà C; Curini V; Di Domenico M; Di Pasquale A; Lorusso A; Mangone I; Marcacci M; Puglia I; Rinaldi A; Savini G; Scialabba S
EPI_ISL_1117675, EPI_ISL_1117686, EPI_ISL_1117690, EPI_ISL_1117697	DIP PREV AVEZZANO-SERVIZIO DI IGIENE EPIDEMIOLOGIA E SANITÀ PUBBLICA	Istituto Zooprofilattico Sperimentale dell'Abruzzo e Molise "G. Caporale"	Ancora M; Calistri P; Cammà C; Curini V; Di Domenico M; Di Pasquale A; Lorusso A; Mangone I; Marcacci M; Puglia I; Rinaldi A; Savini G; Scialabba S
EPI_ISL_1250981 to 1250982	DIP. PREV. AVEZZANO	Istituto Zooprofilattico Sperimentale dell'Abruzzo e Molise "G. Caporale"	Ancora M; Calistri P; Cammà C; Curini V; Di Domenico M; Di Pasquale A; Lorusso A; Mangone I; Marcacci M; Puglia I; Rinaldi A; Savini G; Scialabba S
EPI_ISL_1251039, EPI_ISL_1251061, EPI_ISL_1290805	DIP. PREV. AVEZZANO SERVIZIO DI IGIENE EPIDEMIOLOGIA E SANITA' PUBBLICA	Istituto Zooprofilattico Sperimentale dell'Abruzzo e Molise "G. Caporale"	Ancora M; Calistri P; Cammà C; Curini V; Di Domenico M; Di Pasquale A; Lorusso A; Mangone I; Marcacci M; Puglia I; Rinaldi A; Savini G; Scialabba S
EPI_ISL_833050 to 833051, EPI_ISL_833057 to 833067, EPI_ISL_961078 to 961080, EPI_ISL_961083, EPI_ISL_961094 to 961095, EPI_ISL_1055818	DIP. PREV. AVEZZANO SERVIZIO DI IGIENE EPIDEMIOLOGIAE SANITA' PUBBLICA	Istituto Zooprofilattico Sperimentale dell'Abruzzo e Molise "G. Caporale"	Ancora M; Calistri P; Cammà C; Curini V; Delli Compagni E; Di Domenico M; Di Pasquale A; Lorusso A; Mangone I; Marcacci M; Puglia I; Rinaldi A; Savini G; Scialabba S
EPI_ISL_849635 to 849636	DIPARTIMENTO PREVENZIONE AVEZZANO-SERVIZIO DI IGIENE EPIDEMIOLOGIA E SANITA' PUBBLICA	Istitituto Zooprofilattico Sperimentale dell'Abruzzo e Molise "G.Caporale"	Ancora M; Cammà C; Curini V; Di Domenico M; Di Pasquale A; Lorusso A; Mangone I; Marcacci M; Puglia I; Rinaldi A; Savini G.
EPI_ISL_806758 to 806761, EPI_ISL_806763 to 806765, EPI_ISL_806781 to 806782, EPI_ISL_833283, EPI_ISL_833301	DIPARTIMENTO PREVENZIONE AVEZZANO-SERVIZIO DI IGIENE EPIDEMIOLOGIA E SANITA' PUBBLICA	Istituto Zooprofilattico Sperimentale dell'Abruzzo e Molise "G. Caporale"	Ancora M; Calistri P; Cammà C; Curini V; Di Domenico M; Di Pasquale A; Lorusso A; Mangone I; Marcacci M; Puglia I; Rinaldi A; Savini G
EPI_ISL_794658	DIRECCION DE SANIDAD POLICIA NACIONAL	Instituto Nacional de Salud - Dirección de Investigación en Salud Pública	Carlos Franco-Muñoz; Diego A. Álvarez-Díaz; Diego Andrés Prada; Gerardo Santamaría; Jonathan Reales; Julian Naizaque; Katherine Laiton-Donato; Magdalena Wiesner; Marcela Mercado-Reyes; Maria T. Herrera; Martha Lucia Ospina Martinez; Mauricio Pacheco-Montealegre; Paola Muñoz-Laiton; Sheryl Corchuelo
EPI_ISL_906550 to 906551	DIRECCION DEPARTAMENTAL DE SALUD DE AMAZONAS	Instituto Nacional de Salud- Dirección de Investigación en Salud Pública, Universidad de los Andes- Applied genomics research group, Vicerrectoria de Investigación y Creación, Universidad de los Andes- Systems and Computing Engineering Department	Carlos Franco-Muñoz; Diego A. Álvarez-Díaz; Diego Andrés Prada; Gerardo Santamaría Jorge Duitama; Héctor Alejandro Ruiz-Moreno; Jhonnatan Reales-González; Jorge Ivan Diaz; Julian Naizaque; Katherine Laiton-Donato; Laura Natalia Gonzalez; Magdalena Wiesner; Marcela Mercado-Reyes; Maria T. Herrera-Sepúlveda; Martha Lucia Ospina Martinez; Mauricio Pacheco-Montealegre; Sheryll Corchuelo; Silvia Restrepo-Restrepo
EPI_ISL_906556	DIRECCION DEPARTAMENTAL DE SALUD DE GUAJIRA	Instituto Nacional de Salud- Dirección de Investigación en Salud Pública, Universidad de los Andes- Applied genomics research group, Vicerrectoria de Investigación y Creación, Universidad de los Andes- Systems and Computing Engineering Department	Carlos Franco-Muñoz; Diego A. Álvarez-Díaz; Diego Andrés Prada; Gerardo Santamaría Jorge Duitama; Héctor Alejandro Ruiz-Moreno; Jhonnatan Reales-González; Jorge Ivan Diaz; Julian Naizaque; Katherine Laiton-Donato; Laura Natalia Gonzalez; Magdalena Wiesner; Marcela Mercado-Reyes; Maria T. Herrera-Sepúlveda; Martha Lucia Ospina Martinez; Mauricio Pacheco-Montealegre; Sheryll Corchuelo; Silvia Restrepo-Restrepo
EPI_ISL_1195278, EPI_ISL_1195288, EPI_ISL_1195291	DIRETORIA DE VIGILANCIA EM SAUDE	Epiclin	Ana Paula Muterle; Carolina Comerlato; Eliana Márcia Da Ros Wendland; Fernando Hayashi Sant'Anna; Janira Prichula; Juliana Comerlato
EPI_ISL_1020205	DMC Dompet Dhuafa, South Tangerang, Banten	Biosafety Level-3 Laboratory, Indonesian Institute of Sciences (LIPI)	Anggia Prasetyoputri; Anik B. Dharmayanthi; Isa Nuryana; Nuruliawaty Utami; Ratih Asmana Ningrum; Sri Swasthikawati; Sugiyono Saputra; Syam Budi Iryanto
EPI_ISL_1059902	DNA Laboratories Sdn. Bhd.	Malaysia Genome Institute	Azrin Ahmad; Enizza Kasim; Irni Suhayu Sapian; Mohd Faizal Abu Bakar; Mohd Noor Mat Isa; Nor Azfa Johari.; Nurhezreen Md Iqbal; Shamsidar Sopie; Siti Noraini Othman; Wong Yong Wee; Yusuf Muhammad Noor
EPI_ISL_747283	DNA SOLUTION LTD.	DNA SOLUTION LTD.	Abdul Khaleque; Abu Sufian; Firoz Kabir; Jannatun Naima; Kazi Nadim Hasan; Md. Imran Khan; Mizanur Rahman; Mohammad Fazle Alam Rabbi; Sharif

			Akhteruzzaman
EPI_ISL_445213	DNA Solution Ltd	DNA Solution Ltd	Abdul Khaleque; Abu Sufian; Hasan Ul Haider; Kazi Nadim Hasan; MSM Chowdhury; Mala Khan; Mamudul Hasan Razu; Md. Imran Khan; Mizanur Rahman; Mohammad Fazle Alam Rabbi; Mohammed Nafiz Imtiaz Polol
EPI_ISL_445214 to 445217	DNA Solution Ltd.	DNA Solution Ltd.	Abdul Khaleque; Abu Sufian; Hasan Ul Haider; Kazi Nadim Hasan; MSM Chowdhury; Mala Khan; Mamudul Hasan Razu; Md. Imran Khan; Mizanur Rahman; Mohammad Fazle Alam Rabbi; Mohammed Nafiz Imtiaz Polol

EPI_ISL_633009 to 633010, EPI_ISL_633018, EPI_ISL_633037, EPI_ISL_633056, EPI_ISL_671689, EPI_ISL_671704, EPI_ISL_683756, EPI_ISL_683795, EPI_ISL_683813, EPI_ISL_683829, EPI_ISL_683834, EPI_ISL_683842, EPI_ISL_683848, EPI_ISL_683859, EPI_ISL_683877, EPI_ISL_683882 to 683883, EPI_ISL_683924, EPI_ISL_683927 to 683928, EPI_ISL_683933, EPI_ISL_683937, EPI_ISL_683962, EPI_ISL_683964, EPI_ISL_683968, EPI_ISL_683974, EPI_ISL_683976 to 683977, EPI_ISL_683982 to 683985, EPI_ISL_683991, EPI_ISL_745400, EPI_ISL_745404, EPI_ISL_745413, EPI_ISL_745425, EPI_ISL_745451, EPI_ISL_745469, EPI_ISL_823832 to 823834, EPI_ISL_823838 to 823840, EPI_ISL_823843 to 823844, EPI_ISL_823865, EPI_ISL_823869, EPI_ISL_823884 to 823887, EPI_ISL_823901 to 823902, EPI_ISL_823912, EPI_ISL_823927, EPI_ISL_823950, EPI_ISL_824298, EPI_ISL_824317 to 824318, EPI_ISL_832256, EPI_ISL_832266 to 832267, EPI_ISL_832284, EPI_ISL_832300, EPI_ISL_832312, EPI_ISL_832336 to 832337, EPI_ISL_832346 to 832350, EPI_ISL_857077 to 857080, EPI_ISL_857085 to 857086, EPI_ISL_857094, EPI_ISL_857100, EPI_ISL_857116, EPI_ISL_857118, EPI_ISL_857120, EPI_ISL_857126, EPI_ISL_857132 to 857133, EPI_ISL_857142, EPI_ISL_857163, EPI_ISL_857180 to 857183, EPI_ISL_861332, EPI_ISL_883309, EPI_ISL_883324, EPI_ISL_883327, EPI_ISL_883329, EPI_ISL_883371 to 883372, EPI_ISL_883376 to 883379, EPI_ISL_883409, EPI_ISL_883413 to 883415, EPI_ISL_937132 to 937133, EPI_ISL_937167, EPI_ISL_937172, EPI_ISL_937204 to 937205, EPI_ISL_937216, EPI_ISL_966389, EPI_ISL_966413, EPI_ISL_966416, EPI_ISL_966450, EPI_ISL_966475, EPI_ISL_1017982 to 1017985, EPI_ISL_1018001, EPI_ISL_1064775, EPI_ISL_1064803, EPI_ISL_1064809, EPI_ISL_1064820, EPI_ISL_1064825, EPI_ISL_1064831, EPI_ISL_1064876, EPI_ISL_1064909, EPI_ISL_1200522, EPI_ISL_1200541, EPI_ISL_1200552, EPI_ISL_1200556, EPI_ISL_1200577, EPI_ISL_1200594, EPI_ISL_1200621 to 1200622, EPI_ISL_1200624 to 1200625, EPI_ISL_1261801, EPI_ISL_1261804, EPI_ISL_1261807 to 1261808, EPI_ISL_1261824, EPI_ISL_1261843, EPI_ISL_1261856, EPI_ISL_1261858, EPI_ISL_1261864

see above	DOHMH Central Harlem	New York City Public Health Laboratory	Jade Wang; et al.

EPI_ISL_633014, EPI_ISL_633016 to 633017, EPI_ISL_633023, EPI_ISL_633025, EPI_ISL_633031, EPI_ISL_633033, EPI_ISL_633039 to 633040, EPI_ISL_633046 to 633047, EPI_ISL_633052, EPI_ISL_633066, EPI_ISL_633069, EPI_ISL_633076 to 633078, EPI_ISL_671684, EPI_ISL_671693, EPI_ISL_671702, EPI_ISL_671713, EPI_ISL_671722, EPI_ISL_671763, EPI_ISL_683765, EPI_ISL_683780, EPI_ISL_683788, EPI_ISL_683792, EPI_ISL_683807 to 683808, EPI_ISL_683828, EPI_ISL_683830, EPI_ISL_683865, EPI_ISL_683923, EPI_ISL_683931, EPI_ISL_683936, EPI_ISL_683941, EPI_ISL_683957, EPI_ISL_683961, EPI_ISL_683967, EPI_ISL_683971 to 683972, EPI_ISL_683980 to 683981, EPI_ISL_683989, EPI_ISL_745410, EPI_ISL_745421 to 745422, EPI_ISL_745436, EPI_ISL_745453, EPI_ISL_745457, EPI_ISL_823815 to 823816, EPI_ISL_823864, EPI_ISL_823924, EPI_ISL_823930, EPI_ISL_823934, EPI_ISL_823948, EPI_ISL_823954, EPI_ISL_823962, EPI_ISL_824300, EPI_ISL_824319, EPI_ISL_832282, EPI_ISL_832301 to 832302, EPI_ISL_832313 to 832314, EPI_ISL_832341, EPI_ISL_832362 to 832363, EPI_ISL_857076, EPI_ISL_857106 to 857110, EPI_ISL_857117, EPI_ISL_857130 to 857131, EPI_ISL_857136 to 857139, EPI_ISL_883335, EPI_ISL_883339, EPI_ISL_883368 to 883369, EPI_ISL_937136 to 937137, EPI_ISL_937174, EPI_ISL_937202 to 937203, EPI_ISL_937217 to 937218, EPI_ISL_966468, EPI_ISL_966483, EPI_ISL_1017978, EPI_ISL_1018011, EPI_ISL_1064766, EPI_ISL_1064779, EPI_ISL_1064782, EPI_ISL_1064797, EPI_ISL_1064872, EPI_ISL_1064895 to 1064896, EPI_ISL_1200518, EPI_ISL_1200528 to 1200530, EPI_ISL_1200534, EPI_ISL_1200560, EPI_ISL_1200564, EPI_ISL_1200574, EPI_ISL_1200584, EPI_ISL_1261817, EPI_ISL_1261852, EPI_ISL_1261855, EPI_ISL_1261862 to 1261863

see above	DOHMH Chelsea	New York City Public Health Laboratory	Jade Wang; et al.

EPI_ISL_632980 to 632993, EPI_ISL_633059, EPI_ISL_633074, EPI_ISL_671674, EPI_ISL_671677, EPI_ISL_671681 to 671683, EPI_ISL_671685, EPI_ISL_671691, EPI_ISL_671695, EPI_ISL_671697, EPI_ISL_671699 to 671700, EPI_ISL_671703, EPI_ISL_671705, EPI_ISL_671710 to 671711, EPI_ISL_671717 to 671721, EPI_ISL_671731 to 671732, EPI_ISL_671746 to 671747, EPI_ISL_671761, EPI_ISL_683753, EPI_ISL_683757 to 683758, EPI_ISL_683761, EPI_ISL_683764, EPI_ISL_683766, EPI_ISL_683770, EPI_ISL_683773, EPI_ISL_683777, EPI_ISL_683779, EPI_ISL_683785, EPI_ISL_683793 to 683794, EPI_ISL_683796, EPI_ISL_683798 to 683800, EPI_ISL_683802, EPI_ISL_683809, EPI_ISL_683811 to 683812, EPI_ISL_683815, EPI_ISL_683817 to 683818, EPI_ISL_683824, EPI_ISL_683831, EPI_ISL_683833, EPI_ISL_683841, EPI_ISL_683847, EPI_ISL_683849, EPI_ISL_683851, EPI_ISL_683855, EPI_ISL_683857, EPI_ISL_683860, EPI_ISL_683867, EPI_ISL_683870, EPI_ISL_683872 to 683873, EPI_ISL_683876, EPI_ISL_683881, EPI_ISL_683893, EPI_ISL_683896 to 683900, EPI_ISL_683908 to 683909, EPI_ISL_683911 to 683912, EPI_ISL_683926, EPI_ISL_683929, EPI_ISL_683932, EPI_ISL_683934, EPI_ISL_683952 to 683953, EPI_ISL_683960, EPI_ISL_683978 to 683979, EPI_ISL_745401, EPI_ISL_745403, EPI_ISL_745409, EPI_ISL_745411, EPI_ISL_745423 to 745424, EPI_ISL_745426 to 745427, EPI_ISL_745434 to 745435, EPI_ISL_745446 to 745447, EPI_ISL_745455, EPI_ISL_745458 to 745462, EPI_ISL_745464, EPI_ISL_745466 to 745467, EPI_ISL_823817 to 823819, EPI_ISL_823826 to 823828, EPI_ISL_823835 to 823837, EPI_ISL_823850, EPI_ISL_823861 to 823863, EPI_ISL_823868, EPI_ISL_823878 to 823882, EPI_ISL_823898 to 823899, EPI_ISL_823905 to 823906, EPI_ISL_823918 to 823922, EPI_ISL_823931, EPI_ISL_823933, EPI_ISL_823943 to 823945, EPI_ISL_823953, EPI_ISL_823957, EPI_ISL_823963 to 823967, EPI_ISL_824314 to 824315, EPI_ISL_824320, EPI_ISL_824328, EPI_ISL_824331, EPI_ISL_832243, EPI_ISL_832245, EPI_ISL_832251 to 832254, EPI_ISL_832260 to 832262, EPI_ISL_832277 to 832279, EPI_ISL_832290 to 832291, EPI_ISL_832303 to 832304, EPI_ISL_832320 to 832322, EPI_ISL_832325, EPI_ISL_832331 to 832335, EPI_ISL_832351, EPI_ISL_832355 to 832356, EPI_ISL_832359 to 832360, EPI_ISL_857067, EPI_ISL_857073, EPI_ISL_857082 to 857084, EPI_ISL_857101 to 857105, EPI_ISL_857135, EPI_ISL_857145 to 857147, EPI_ISL_857153, EPI_ISL_857156, EPI_ISL_857158, EPI_ISL_857165, EPI_ISL_857168, EPI_ISL_883312 to 883313, EPI_ISL_883348, EPI_ISL_883354 to 883356, EPI_ISL_883410 to 883411, EPI_ISL_937139 to 937144, EPI_ISL_937164 to 937165, EPI_ISL_937178 to 937184, EPI_ISL_937208, EPI_ISL_937214, EPI_ISL_937227 to 937231, EPI_ISL_966386, EPI_ISL_966392, EPI_ISL_966394, EPI_ISL_966399, EPI_ISL_966409 to 966410, EPI_ISL_966415, EPI_ISL_966425, EPI_ISL_966445 to 966446, EPI_ISL_966459, EPI_ISL_966467, EPI_ISL_966474, EPI_ISL_966476 to 966478, EPI_ISL_966480, EPI_ISL_966482, EPI_ISL_966491, EPI_ISL_1017975, EPI_ISL_1017986 to 1017988, EPI_ISL_1017995 to 1017999, EPI_ISL_1018005, EPI_ISL_1018027 to 1018031, EPI_ISL_1064757, EPI_ISL_1064760 to 1064761, EPI_ISL_1064767, EPI_ISL_1064784, EPI_ISL_1064786, EPI_ISL_1064789, EPI_ISL_1064808, EPI_ISL_1064811, EPI_ISL_1064847, EPI_ISL_1064870 to 1064871, EPI_ISL_1064873 to 1064874, EPI_ISL_1064888, EPI_ISL_1064893, EPI_ISL_1064897, EPI_ISL_1064905, EPI_ISL_1200533, EPI_ISL_1200540, EPI_ISL_1200545 to 1200546, EPI_ISL_1200565, EPI_ISL_1200568 to 1200570, EPI_ISL_1200591 to 1200593, EPI_ISL_1200597 to 1200598, EPI_ISL_1200603, EPI_ISL_1200613 to 1200614, EPI_ISL_1200626, EPI_ISL_1261806, EPI_ISL_1261810, EPI_ISL_1261812 to 1261813, EPI_ISL_1261832 to 1261833, EPI_ISL_1261835 to 1261837, EPI_ISL_1261844, EPI_ISL_1261859

see above	DOHMH Corona	New York City Public Health Laboratory	Jade Wang; et al.

EPI_ISL_632994 to 632998, EPI_ISL_633063, EPI_ISL_671690, EPI_ISL_671760, EPI_ISL_683821, EPI_ISL_683827, EPI_ISL_683856, EPI_ISL_683891, EPI_ISL_683904, EPI_ISL_683910, EPI_ISL_683935, EPI_ISL_683970, EPI_ISL_745419 to 745420, EPI_ISL_745445, EPI_ISL_745448, EPI_ISL_745452, EPI_ISL_745454, EPI_ISL_823852 to 823853, EPI_ISL_823925, EPI_ISL_823935 to 823936, EPI_ISL_823942, EPI_ISL_824301, EPI_ISL_824310, EPI_ISL_824316, EPI_ISL_832244, EPI_ISL_832275, EPI_ISL_832287 to 832288, EPI_ISL_832292 to 832293, EPI_ISL_832295, EPI_ISL_832338, EPI_ISL_832340, EPI_ISL_857089, EPI_ISL_857097, EPI_ISL_857172 to 857173, EPI_ISL_883337, EPI_ISL_883357 to 883360, EPI_ISL_883364, EPI_ISL_883370, EPI_ISL_883412, EPI_ISL_937138, EPI_ISL_937168, EPI_ISL_937173, EPI_ISL_966484, EPI_ISL_1018021, EPI_ISL_1018024, EPI_ISL_1064777, EPI_ISL_1064791, EPI_ISL_1064798, EPI_ISL_1064824, EPI_ISL_1064835, EPI_ISL_1064883, EPI_ISL_1064890, EPI_ISL_1064904, EPI_ISL_1200524, EPI_ISL_1200543, EPI_ISL_1200554, EPI_ISL_1200571, EPI_ISL_1200605, EPI_ISL_1261803, EPI_ISL_1261821, EPI_ISL_1261825, EPI_ISL_1261834, EPI_ISL_1261861

see above	DOHMH Crown Heights	New York City Public Health Laboratory	Jade Wang; et al.

EPI_ISL_633073, EPI_ISL_671678, EPI_ISL_671738, EPI_ISL_671759, EPI_ISL_683771, EPI_ISL_683816, EPI_ISL_683832, EPI_ISL_683868, EPI_ISL_683887 to 683888, EPI_ISL_683906, EPI_ISL_683913, EPI_ISL_683922, EPI_ISL_683963, EPI_ISL_683973, EPI_ISL_683996, EPI_ISL_745463, EPI_ISL_823849, EPI_ISL_823851, EPI_ISL_823866 to 823867, EPI_ISL_823904, EPI_ISL_823910 to 823911, EPI_ISL_823923, EPI_ISL_823938, EPI_ISL_824311 to 824312, EPI_ISL_824321 to 824322, EPI_ISL_832268 to 832269, EPI_ISL_832286, EPI_ISL_857095, EPI_ISL_857112 to 857115, EPI_ISL_857148, EPI_ISL_857166 to 857167, EPI_ISL_883416 to 883417, EPI_ISL_937134 to 937135, EPI_ISL_937200 to 937201, EPI_ISL_966443, EPI_ISL_1064762, EPI_ISL_1064771, EPI_ISL_1064780, EPI_ISL_1064785, EPI_ISL_1064790, EPI_ISL_1064794, EPI_ISL_1064836, EPI_ISL_1064885, EPI_ISL_1064891, EPI_ISL_1200523, EPI_ISL_1200620, EPI_ISL_1261809, EPI_ISL_1261818, EPI_ISL_1261847, EPI_ISL_1261850

see above	DOHMH Fort Greene	New York City Public Health Laboratory	Jade Wang; et al.

EPI_ISL_632999 to 633008, EPI_ISL_633060 to 633062, EPI_ISL_633064, EPI_ISL_671675, EPI_ISL_671692, EPI_ISL_671698, EPI_ISL_671706 to 671707, EPI_ISL_671712, EPI_ISL_671714, EPI_ISL_671723 to 671724, EPI_ISL_671730, EPI_ISL_671735, EPI_ISL_671739, EPI_ISL_671743, EPI_ISL_683775 to 683776, EPI_ISL_683781 to 683782, EPI_ISL_683786, EPI_ISL_683791, EPI_ISL_683822, EPI_ISL_683837 to 683838, EPI_ISL_683850, EPI_ISL_683858, EPI_ISL_683861 to 683862, EPI_ISL_683864, EPI_ISL_683874, EPI_ISL_683878, EPI_ISL_683885, EPI_ISL_683892, EPI_ISL_683901, EPI_ISL_683903, EPI_ISL_683914 to 683916, EPI_ISL_683920 to 683921, EPI_ISL_683925, EPI_ISL_683930, EPI_ISL_683939, EPI_ISL_683944 to 683945, EPI_ISL_683948, EPI_ISL_683951, EPI_ISL_683956, EPI_ISL_683958, EPI_ISL_683975, EPI_ISL_683986, EPI_ISL_683990, EPI_ISL_745398, EPI_ISL_745402, EPI_ISL_745405, EPI_ISL_745407, EPI_ISL_745414 to 745418, EPI_ISL_745440, EPI_ISL_745444, EPI_ISL_823813, EPI_ISL_823821 to 823822, EPI_ISL_823846 to 823848, EPI_ISL_823870 to 823871, EPI_ISL_823876 to 823877, EPI_ISL_823894 to 823895, EPI_ISL_823907 to 823908, EPI_ISL_823915 to 823917, EPI_ISL_823926, EPI_ISL_823928, EPI_ISL_823940 to 823941, EPI_ISL_823946, EPI_ISL_823949, EPI_ISL_823955 to 823956, EPI_ISL_823961, EPI_ISL_823968 to 823969, EPI_ISL_824296 to 824297, EPI_ISL_824302, EPI_ISL_824304 to 824305, EPI_ISL_824308 to 824309, EPI_ISL_824313, EPI_ISL_824329 to 824330, EPI_ISL_832246, EPI_ISL_832250, EPI_ISL_832263, EPI_ISL_832265, EPI_ISL_832272 to 832274, EPI_ISL_832276, EPI_ISL_832283, EPI_ISL_832294, EPI_ISL_832297 to 832298, EPI_ISL_832306 to 832311, EPI_ISL_832317 to 832319, EPI_ISL_832323 to 832324, EPI_ISL_832328 to 832330, EPI_ISL_832354, EPI_ISL_832357 to 832358, EPI_ISL_832361, EPI_ISL_857069, EPI_ISL_857075, EPI_ISL_857091, EPI_ISL_857096, EPI_ISL_857149 to 857152, EPI_ISL_857154 to 857155, EPI_ISL_857174 to 857177, EPI_ISL_857179, EPI_ISL_876519, EPI_ISL_883310, EPI_ISL_883314, EPI_ISL_883318 to 883323, EPI_ISL_883349, EPI_ISL_883373 to 883375, EPI_ISL_883418 to 883423, EPI_ISL_937145 to 937151, EPI_ISL_937157 to 937163, EPI_ISL_937175 to 937177, EPI_ISL_937213, EPI_ISL_937219 to 937226, EPI_ISL_966382, EPI_ISL_966384, EPI_ISL_966387 to 966388, EPI_ISL_966395, EPI_ISL_966398, EPI_ISL_966405, EPI_ISL_966412, EPI_ISL_966421 to 966422, EPI_ISL_966424, EPI_ISL_966432 to 966433, EPI_ISL_966447 to 966449, EPI_ISL_966461 to 966463, EPI_ISL_966479, EPI_ISL_966481, EPI_ISL_1017976 to 1017977, EPI_ISL_1017992 to 1017994, EPI_ISL_1018012 to 1018020, EPI_ISL_1018022 to 1018023, EPI_ISL_1064755, EPI_ISL_1064759, EPI_ISL_1064781, EPI_ISL_1064788, EPI_ISL_1064807, EPI_ISL_1064819, EPI_ISL_1064830, EPI_ISL_1064837 to 1064840, EPI_ISL_1064848, EPI_ISL_1064858, EPI_ISL_1064865 to 1064869, EPI_ISL_1064884, EPI_ISL_1064886, EPI_ISL_1064892, EPI_ISL_1064906 to 1064907, EPI_ISL_1200520 to 1200521, EPI_ISL_1200535 to 1200536, EPI_ISL_1200542, EPI_ISL_1200544, EPI_ISL_1200547, EPI_ISL_1200551, EPI_ISL_1200553, EPI_ISL_1200563, EPI_ISL_1200572 to 1200573, EPI_ISL_1200581, EPI_ISL_1200583, EPI_ISL_1200585, EPI_ISL_1200590, EPI_ISL_1200595 to 1200596, EPI_ISL_1200615 to 1200618, EPI_ISL_1261790 to 1261794, EPI_ISL_1261802, EPI_ISL_1261815, EPI_ISL_1261820, EPI_ISL_1261822, EPI_ISL_1261831, EPI_ISL_1261857, EPI_ISL_1261860, EPI_ISL_1261866

see above	DOHMH Jamaica	New York City Public Health Laboratory	Jade Wang; et al.

EPI_ISL_633015, EPI_ISL_633019 to 633022, EPI_ISL_633024, EPI_ISL_633027 to 633030, EPI_ISL_633032, EPI_ISL_633036, EPI_ISL_633044, EPI_ISL_633048 to 633049, EPI_ISL_633053, EPI_ISL_633057 to 633058, EPI_ISL_633065, EPI_ISL_633067 to 633068, EPI_ISL_633075, EPI_ISL_633080 to 633082, EPI_ISL_671679 to 671680, EPI_ISL_671686 to 671688, EPI_ISL_671708, EPI_ISL_671726 to 671729, EPI_ISL_671736, EPI_ISL_671740 to 671742, EPI_ISL_671751 to 671758, EPI_ISL_683754, EPI_ISL_683760, EPI_ISL_683762 to 683763, EPI_ISL_683772, EPI_ISL_683778, EPI_ISL_683790, EPI_ISL_683801, EPI_ISL_683803 to 683806, EPI_ISL_683814, EPI_ISL_683823, EPI_ISL_683825 to 683826, EPI_ISL_683839 to 683840, EPI_ISL_683843 to 683845, EPI_ISL_683853 to 683854, EPI_ISL_683863, EPI_ISL_683869, EPI_ISL_683879 to 683880, EPI_ISL_683884, EPI_ISL_683886, EPI_ISL_683889, EPI_ISL_683905, EPI_ISL_683917 to 683919, EPI_ISL_683938, EPI_ISL_683940, EPI_ISL_683946 to 683947, EPI_ISL_683959, EPI_ISL_683965 to 683966, EPI_ISL_683969, EPI_ISL_683992, EPI_ISL_745399, EPI_ISL_745406, EPI_ISL_745412, EPI_ISL_745430 to 745433, EPI_ISL_745441 to 745443, EPI_ISL_745449 to 745450, EPI_ISL_745470, EPI_ISL_823829 to 823831, EPI_ISL_823845, EPI_ISL_823854 to 823860, EPI_ISL_823872 to 823874, EPI_ISL_823892 to 823893, EPI_ISL_823903, EPI_ISL_823929, EPI_ISL_823932, EPI_ISL_823947, EPI_ISL_823959 to 823960, EPI_ISL_824295, EPI_ISL_824299, EPI_ISL_824303, EPI_ISL_824323 to 824327, EPI_ISL_832247 to 832248, EPI_ISL_832255, EPI_ISL_832257, EPI_ISL_832264, EPI_ISL_832280 to 832281, EPI_ISL_832285, EPI_ISL_832289, EPI_ISL_832299, EPI_ISL_832315 to 832316, EPI_ISL_832327, EPI_ISL_832342 to 832345, EPI_ISL_857071, EPI_ISL_857074, EPI_ISL_857081, EPI_ISL_857093, EPI_ISL_857098 to 857099, EPI_ISL_857119, EPI_ISL_857122 to 857125, EPI_ISL_857140 to 857141, EPI_ISL_857159 to 857160, EPI_ISL_857164, EPI_ISL_857169 to 857171, EPI_ISL_883331, EPI_ISL_883338, EPI_ISL_883347, EPI_ISL_883365 to 883367, EPI_ISL_937129 to 937131, EPI_ISL_937206 to 937207, EPI_ISL_937232 to 937236, EPI_ISL_937238 to 937239, EPI_ISL_966407 to 966408, EPI_ISL_966414, EPI_ISL_966420, EPI_ISL_966423, EPI_ISL_966434 to 966437, EPI_ISL_966451, EPI_ISL_966460, EPI_ISL_1017974, EPI_ISL_1017980 to 1017981, EPI_ISL_1018000, EPI_ISL_1018006 to 1018010, EPI_ISL_1018025 to 1018026, EPI_ISL_1064787, EPI_ISL_1064801 to 1064802, EPI_ISL_1064812, EPI_ISL_1064834, EPI_ISL_1064841 to 1064842, EPI_ISL_1064849 to 1064850, EPI_ISL_1064861 to 1064862, EPI_ISL_1064875, EPI_ISL_1200550, EPI_ISL_1200557 to 1200559, EPI_ISL_1200567, EPI_ISL_1200575, EPI_ISL_1200578 to 1200580, EPI_ISL_1200582, EPI_ISL_1200588 to 1200589, EPI_ISL_1200600, EPI_ISL_1200604, EPI_ISL_1200612, EPI_ISL_1261814, EPI_ISL_1261826, EPI_ISL_1261838 to 1261839, EPI_ISL_1261848, EPI_ISL_1261851

see above	DOHMH Morrisania	New York City Public Health Laboratory	Jade Wang; et al.

EPI_ISL_633011, EPI_ISL_633013, EPI_ISL_633026, EPI_ISL_633035, EPI_ISL_633038, EPI_ISL_633045, EPI_ISL_633050 to 633051, EPI_ISL_633054 to 633055, EPI_ISL_633071, EPI_ISL_633079, EPI_ISL_671676, EPI_ISL_671696, EPI_ISL_671715 to 671716, EPI_ISL_671734, EPI_ISL_671737, EPI_ISL_671744, EPI_ISL_683755, EPI_ISL_683759, EPI_ISL_683774, EPI_ISL_683783, EPI_ISL_683787, EPI_ISL_683789, EPI_ISL_683797, EPI_ISL_683810, EPI_ISL_683836, EPI_ISL_683846, EPI_ISL_683852, EPI_ISL_683871, EPI_ISL_683875, EPI_ISL_683890, EPI_ISL_683943, EPI_ISL_683954, EPI_ISL_683987 to 683988, EPI_ISL_683993, EPI_ISL_683995, EPI_ISL_745437 to 745438, EPI_ISL_745456, EPI_ISL_745465, EPI_ISL_823820, EPI_ISL_823842, EPI_ISL_823875, EPI_ISL_823889 to 823891, EPI_ISL_823896 to 823897, EPI_ISL_823909, EPI_ISL_823913 to 823914, EPI_ISL_823937, EPI_ISL_823958, EPI_ISL_824306 to 824307, EPI_ISL_832249, EPI_ISL_832258 to 832259, EPI_ISL_832352 to 832353, EPI_ISL_857063 to 857065, EPI_ISL_857068, EPI_ISL_857070, EPI_ISL_857072, EPI_ISL_857087 to 857088, EPI_ISL_857121, EPI_ISL_857127 to 857129, EPI_ISL_857134, EPI_ISL_857157, EPI_ISL_857161 to 857162, EPI_ISL_857185, EPI_ISL_883340 to 883343, EPI_ISL_883346, EPI_ISL_883361 to 883363, EPI_ISL_883380 to 883382, EPI_ISL_883424, EPI_ISL_937152 to 937156, EPI_ISL_937169 to 937171, EPI_ISL_937185, EPI_ISL_937209 to 937212, EPI_ISL_937215, EPI_ISL_966404, EPI_ISL_966426, EPI_ISL_966444, EPI_ISL_966464 to 966466, EPI_ISL_1017969 to 1017970, EPI_ISL_1017979, EPI_ISL_1017991, EPI_ISL_1018002 to 1018004, EPI_ISL_1064756, EPI_ISL_1064769, EPI_ISL_1064799, EPI_ISL_1064805 to 1064806, EPI_ISL_1064821 to 1064822, EPI_ISL_1064826, EPI_ISL_1064859 to 1064860, EPI_ISL_1064887, EPI_ISL_1064894, EPI_ISL_1200538, EPI_ISL_1200548, EPI_ISL_1200566, EPI_ISL_1200586 to 1200587, EPI_ISL_1200601 to 1200602, EPI_ISL_1200619, EPI_ISL_1200623, EPI_ISL_1261798, EPI_ISL_1261805, EPI_ISL_1261823

see above	DOHMH PHL	New York City Public Health Laboratory	Jade Wang; et al.

EPI_ISL_633012, EPI_ISL_633034, EPI_ISL_633041 to 633043, EPI_ISL_633072, EPI_ISL_671694, EPI_ISL_671701, EPI_ISL_671709, EPI_ISL_671725, EPI_ISL_671733, EPI_ISL_671745, EPI_ISL_671748 to 671750, EPI_ISL_671762, EPI_ISL_683767 to 683769, EPI_ISL_683784, EPI_ISL_683819 to 683820,

EPI_ISL_683866, EPI_ISL_683894 to 683895, EPI_ISL_683902, EPI_ISL_683907, EPI_ISL_683942, EPI_ISL_683949 to 683950, EPI_ISL_683955, EPI_ISL_683994, EPI_ISL_745408, EPI_ISL_745428 to 745429, EPI_ISL_745439, EPI_ISL_745468, EPI_ISL_823814, EPI_ISL_823823 to 823825, EPI_ISL_823841, EPI_ISL_823883, EPI_ISL_823888, EPI_ISL_823900, EPI_ISL_823939, EPI_ISL_823951 to 823952, EPI_ISL_832270 to 832271, EPI_ISL_832296, EPI_ISL_832305, EPI_ISL_832326, EPI_ISL_832339, EPI_ISL_857090, EPI_ISL_857092, EPI_ISL_857111, EPI_ISL_857143 to 857144, EPI_ISL_857178, EPI_ISL_857184, EPI_ISL_883333 to 883334, EPI_ISL_937126 to 937128, EPI_ISL_937166, EPI_ISL_937237, EPI_ISL_1017971 to 1017973, EPI_ISL_1064823, EPI_ISL_1064908, EPI_ISL_1200525, EPI_ISL_1200537, EPI_ISL_1200606, EPI_ISL_1200611, EPI_ISL_1261795, EPI_ISL_1261799, EPI_ISL_1261816, EPI_ISL_1261840 to 1261842, EPI_ISL_1261845, EPI_ISL_1261849			
see above	DOHMH Riverside	New York City Public Health Laboratory	Jade Wang; et al.
EPI_ISL_891153 to 891206, EPI_ISL_936464 to 936485, EPI_ISL_1018063 to 1018070	DPH, Massachusetts State Public Health Lab	DPH, Massachusetts State Public Health Lab	A.S.; Fink, T.; G.R.; Gallagher; Lang; S.C.; Smole
EPI_ISL_933735 to 933739, EPI_ISL_933741 to 933765, EPI_ISL_1009632 to 1009654, EPI_ISL_1181629 to 1181657, EPI_ISL_1181661 to 1181678, EPI_ISL_1181680 to 1181694, EPI_ISL_1268194 to 1268195, EPI_ISL_1268198, EPI_ISL_1268200, EPI_ISL_1268202, EPI_ISL_1268204 to 1268205, EPI_ISL_1268207, EPI_ISL_1268209, EPI_ISL_1268211, EPI_ISL_1268213, EPI_ISL_1268215, EPI_ISL_1268217, EPI_ISL_1268219, EPI_ISL_1268221, EPI_ISL_1268223 to 1268224, EPI_ISL_1268226, EPI_ISL_1268228, EPI_ISL_1268230, EPI_ISL_1268232, EPI_ISL_1268234, EPI_ISL_1268236, EPI_ISL_1268238, EPI_ISL_1268240, EPI_ISL_1268243 to 1268244, EPI_ISL_1268246, EPI_ISL_1268248, EPI_ISL_1268250, EPI_ISL_1268253, EPI_ISL_1268255, EPI_ISL_1268257, EPI_ISL_1268259, EPI_ISL_1268261, EPI_ISL_1268263			
see above	DPHL	Delaware Public Health Lab	Gregory Hovan; Rebecca Savage
EPI_ISL_794661	DRECCION DE SANIDAD POLICIA NACIONAL	Instituto Nacional de Salud - Dirección de Investigación en Salud Pública	Carlos Franco-Muñoz; Diego A. Álvarez-Díaz; Diego Andrés Prada; Gerardo Santamaría; Jonathan Reales; Julian Naizaque; Katherine Laiton-Donato; Magdalena Wiesner; Marcela Mercado-Reyes; Maria T. Herrera; Martha Lucia Ospina Martinez; Mauricio Pacheco-Montealegre; Paola Muñoz-Laiton; Sheryl Corchuelo
EPI_ISL_1229051, EPI_ISL_1229054 to 1229063, EPI_ISL_1229068 to 1229102, EPI_ISL_1312830 to 1312831, EPI_ISL_1312838, EPI_ISL_1312896	DYOMEDEA LYON	CNR Virus des Infections Respiratoires - France SUD	Antonin Bal; Bruno Lina; Gregory Destras; Gwendolyne Burfin; Hadrien Regue; Laurence Josset; Martine Valette; Quentin Semanas
EPI_ISL_1191703 to 1191704, EPI_ISL_1191716, EPI_ISL_1191718, EPI_ISL_1239448	DYOMEDEA-NEOLAB	CNR Virus des Infections Respiratoires - France SUD	Antonin Bal; Bruno Lina; Gregory Destras; Gwendolyne Burfin; Hadrien Regue; Laurence Josset; Martine Valette; Quentin Semanas
EPI_ISL_877459, EPI_ISL_1159389	Darmo Hospital	Institute of Tropical Disease, Universitas Airlangga	Aldise M Nastri; Gatot Soegiarto; Jezzy R Dewantari; Kazufumi Shimizu; Krisnoadi Rahardjo; Laksmi Wulandari; Maria I Lusida; Resti Yudhawati; Rima R Prasetya; Soetjipto; Sulung Budianto; Yasuko Mori
EPI_ISL_877420, EPI_ISL_882770, EPI_ISL_1165073	Darus Syifa' Islamic Hospital	Institute of Tropical Disease, Universitas Airlangga	Aldise M Nastri; Gatot Soegiarto; HM Faiz; Jezzy R Dewantari; Kazufumi Shimizu; Krisnoadi Rahardjo; Laksmi Wulandari; Maria I Lusida; Resti Yudhawati; Rima R Prasetya; Soetjipto; Yasuko Mori
EPI_ISL_416542 to 416543, EPI_ISL_421652	Dasman Diabetes Institute	Dasman Diabetes Institute	Ebaa Al-Ozairi; Ebaa AlOzairi; Fahd Al-Mulla; Motasem Melhem; Qais Al-Duwairi; Rasheeba Iqbal; Sara Al-Qabandi; Sumi John
EPI_ISL_416541	Dasman Diabetes Institute and Virology Laboratory Ministry of Health	Dasman Diabetes Institute	Ebaa AlOzairi; Fahd Al-Mulla; Motasem Melhem; Qais Al-Duwairi; Rasheeba Iqbal; Sara Al-Qabandi; Sumi John
EPI_ISL_1081828, EPI_ISL_1081840	Davao One World	Philippine Genome Center	Alethea R. de Guzman; Anna Ong-Lim; Arianne A. Zamora; Asia Louisa U. Chong; Benedict A. Maralit; Candice Francheska B. Tambaoan; Carlo M. Lapid; Celia Carlos; Cynthia P. Saloma; Devon Ray Pacial; Edsel Maurice Salvaña; El King D. Morado; Eva Maria Cutiongco-de la Paz; Francis A. Tablizo; Irish Coleen A. Asin; Jaime C. Montoya; Jan Michael C. Yap; Jo-Hannah S. Llames; John Q. Wong; Joshua Gregor A. Dizon; Juan Antonio R. Magalang; Karol Sophia Agape R. Padilla; Kenneth M. Kim; Kris P. Punayan; Marc Edsel C. Ayes; Marc Jerrone R. Castro; Maria Sofia L. Yangzon; Marissa Alejandria; Razel Nikka M. Hao; Rianna Patricia S. Cruz; Sheila Mae M. Araiza; and Maria Rosario Singh-Vergeire
EPI_ISL_861672, EPI_ISL_906065	Day Hospital de Ermelino Matarazzo	Instituto Adolfo Lutz, Interdiciplinary Procedures Center, Strategic Laboratory	Claudia Regina Gonçalves; Claudio Tavares Sacchi; Erica Valessa Ramos Gomes; Karoline Rodrigues Campos
EPI_ISL_1093602 to 1094191, EPI_ISL_1098800 to 1098829	DeRisi Lab, University of California, San Francisco	DeRisi Lab, University of California, San Francisco	Amy Kistler; Anthea Mitchell; Carina Marquez; Diane Havlir; Diane Jones; Douglas Black; Eric Chow; Gabriel Chamie; Genay Pilarowski; IDseq Team; Jackie Martinez; James Peng; Jamin Liu; Jon Jacobo; Lucy Li; Luis Rubio; Manu Vanaerschot; Matthew Laurie; Maya Petersen; Patrick Ayscue; Sabrina Mann; Sara Sunshine; Susana Rojas; Susy Rojas; Valerie Tulier-Laiwa; and Joseph DeRisi
EPI_ISL_476022	Defence Research & Development Establishment	Defence Research & Development Establishment	Ambuj Shrivastava; Jyoti S Kumar; Paban Kumar Dash; Shashi Sharma; Sushil Kumar Sharma
EPI_ISL_476023, EPI_ISL_476840, EPI_ISL_476842, EPI_ISL_476844, EPI_ISL_476846, EPI_ISL_476848 to 476850, EPI_ISL_476852 to 476854, EPI_ISL_476883 to 476896			
see above	Defence Research & Development Establishment (DRDE)	Defence Research & Development Establishment (DRDE)	Ambuj Shrivastava; Jyoti S. Kumar; Paban Kumar Dash; Shashi Sharma; Sushil Kumar Sharma
EPI_ISL_833041	Defence Services Medical Research Center, Biological Research Laboratory	Defence Services Medical Research Center, Biological Research Laboratory	Khine Zaw Oo; Ko Ko Lwin; Ko Ko Win; Kyaw Wanna; Kyee Myint; Nay Myo Aung; Phyo Kyaw Aung; Sat Paing Htoo; Thein Zaw; Zaw Win Htun
EPI_ISL_560307, EPI_ISL_560406, EPI_ISL_560567, EPI_ISL_560647 to 560648, EPI_ISL_560741 to 560742, EPI_ISL_561335 to 561336, EPI_ISL_561345 to 561353, EPI_ISL_561356 to 561360, EPI_ISL_561362, EPI_ISL_565830 to 565832, EPI_ISL_566084, EPI_ISL_566108 to 566109, EPI_ISL_574581, EPI_ISL_574584 to 574586, EPI_ISL_574599, EPI_ISL_575335, EPI_ISL_576150 to 576169, EPI_ISL_576222 to 576227, EPI_ISL_582133, EPI_ISL_583246 to 583248, EPI_ISL_583994 to 583995, EPI_ISL_584013 to 584014, EPI_ISL_593937 to 593981, EPI_ISL_708382 to 708452, EPI_ISL_812286 to 812307, EPI_ISL_849322 to 849349, EPI_ISL_1136975 to 1137047			
see above	Delaware Public Health Lab	Delaware Public Health Lab	Gregory Hovan
EPI_ISL_961105 to 961131	Delaware Public Health Laboratory	Delaware Public Health Lab	Gregory Hovan
EPI_ISL_693688 to 693757, EPI_ISL_766971 to 767012	Delaware Public Health Laboratory	Delaware Public Health Laboratory	Gregory Hovan
EPI_ISL_985239 to 985302	Delaware Public Health Laboratory (DPHL)	Delaware Public Health Lab	Gregory Hovan
EPI_ISL_745171 to 745172	Delft CHC wc DFP	National Health Laboratory Service (NHLS), Tygerberg	Bronwyn Kleinhans; Eduan Wilkindon; Gert van Zyl; Houriiyah Tegally; Kayla Delaney; Susan Engelbrecht; Tulio de Oliveira; Wolfgang Preiser
EPI_ISL_529147 to 529148	Democritus University of Thrace, Department of Medicine	Democritus University of Thrace, Department of Medicine	Bampali, M.; Dovrolis, N.; Froukala, E.; Gatzidou, E.; Karakasiliotis, I.; Kassela, K.; Spanakis, N.; Stavropoulou, A.; Tsakris, A.; Veletza, S.
EPI_ISL_430796 to 430798, EPI_ISL_430802, EPI_ISL_430805 to 430806, EPI_ISL_430808, EPI_ISL_792301	Departamento de Biología y genética molecular, IACA Laboratorios.	Área de Secuenciación del Laboratorio de Virología del Hospital de Niños Dr. Ricardo Gutierrez on behalf of 'Proyecto Argentino Interinstitucional de genomica de SARS-CoV-2' (PAIS Consortium)	A; AS; E; Goya; LE; Lusso; MI; MS; Masciovecchio MV; Mistchenko; Nabaes Jodar; Natale; S; Streitenberger ER; Suárez; Tittarelli; Valinotto; Viegas, M.
EPI_ISL_1096142, EPI_ISL_1096983, EPI_ISL_1096985	Departamento de Cuidados Intensivos-Unidad de Investigación Médica en Inmunoquímica, UMAE Hospital de Especialidades "Bernardo Sepúlveda Gutiérrez", Centro Médico Nacional Siglo XXI, Instituto Mexicano del Seguro Social (IMSS) México.	Laboratorio de Secuenciación-Centro de Instrumentos, IMSS México /Unidad de Investigación Médica en Inmunoquímica, UMAE Hospital de Especialidades "Bernardo Sepúlveda Gutiérrez", Centro Médico Nacional Siglo XXI, Instituto Mexicano del Seguro Social (IMSS), México.	Alejandro Torres Flores; Arturo Cérbulo-Vázquez; Aura Angélica López García; Carolina González-Torres; Constantino López-Macías.; César Raúl González-Bonilla; Eduardo Ferat-Osorio; Elizabeth Rubí Duran Castañeda; Horacio Zamudio-Meza; Javier Gaytán-Cervantes; Jesús Mena López; Joaquín González-Ibarra; José Manuel Ruiz Rincón; Juan Carlos Guerrero Escobar; Lourdes Arriaga-Pizano; Luis Sánchez Hurtado; Zuleima Maldonado Galeana
EPI_ISL_1096141	Departamento de Cuidados Intensivos-Unidad de Investigación Médica en Inmunoquímica, UMAE Hospital de Especialidades "Bernardo Sepúlveda Gutiérrez", Centro Médico Nacional Siglo XXI, Instituto Mexicano del Seguro Social (IMSS), México.	Laboratorio de Secuenciación-Centro de Instrumentos, IMSS México /Unidad de Investigación Médica en Inmunoquímica, UMAE Hospital de Especialidades "Bernardo Sepúlveda Gutiérrez", Centro Médico Nacional Siglo XXI, Instituto Mexicano del Seguro Social (IMSS), México.	Alejandro Torres Flores; Arturo Cérbulo-Vázquez; Aura Angélica López García; Carolina González-Torres; Constantino López-Macías.; César Raúl González-Bonilla; Eduardo Ferat-Osorio; Elizabeth Rubí Duran Castañeda; Horacio Zamudio-Meza; Javier Gaytán-Cervantes; Jesús Mena López; Joaquín González-Ibarra; José Manuel Ruiz Rincón; Juan Carlos Guerrero Escobar; Lourdes Arriaga-Pizano; Luis Sánchez Hurtado; Zuleima Maldonado Galeana
EPI_ISL_444493	Departamento de Laboratorios de Salud Publica (DLSP, Division Epidemiologia, Ministerio de Salud Publica)	Facultad de Ciencias (Sección Genética Evolutiva, Sección Virología).	Arbiza; Calleros, L.; Chiparelli, H.; Coppola, L.; Delfraro, A.; Frabasile, S.; Fuques, E.; Goni, N.; Grecco, S.; J. and Perez, R.; Panzera, Y.; Ramos, N.; Ramos, V.; Techera, C.
EPI_ISL_508615 to 508686, EPI_ISL_949193 to 949243, EPI_ISL_1194046 to 1194047, EPI_ISL_1194049 to 1194050, EPI_ISL_1194052 to 1194053, EPI_ISL_1194055, EPI_ISL_1194057 to 1194058, EPI_ISL_1194060 to 1194061, EPI_ISL_1194063 to 1194064, EPI_ISL_1194066 to 1194067, EPI_ISL_1194069 to 1194070, EPI_ISL_1194072 to 1194073, EPI_ISL_1194075 to 1194076, EPI_ISL_1194078 to 1194079, EPI_ISL_1194081 to 1194082, EPI_ISL_1194084 to 1194085, EPI_ISL_1194087 to 1194088, EPI_ISL_1194090 to 1194091, EPI_ISL_1194093, EPI_ISL_1194095 to 1194096, EPI_ISL_1194098 to 1194099, EPI_ISL_1194613 to 1194629			
see above	Departamento de Microbiología, CDB, Hospital Clínic,	SeqCOVID-SPAIN consortium/IBV(CSIC)	Aida Peiró and SeqCOVID-SPAIN consortium; Andrea Vergara; Elisa Rubio; Jéssica Navero; Mikel Martínez

	Barcelona		
EPI_ISL_603022, EPI_ISL_603034	Departamento de Vigilância à Saúde	Instituto Adolfo Lutz, Interdiciplinary Procedures Center, Strategic Laboratory	Claudia Regina Gonçalves; Claudio Tavares Sacchi; Erica Valessa Ramos Gomes; Karoline Rodrigues Campos
EPI_ISL_516922 to 516933	Department for Molecular Diagnostics, Centre for Medical Microbiology, Institute of Public Health of Montenegro	Charite Universitatsmedizin Berlin, Institut fur Virologie	Barbara Muehlemann; Christian Drosten; Julia Schneider; Jörn Beheim-Schwarzbach; Marija Govedarica and Danijela Vujoševi; Talitha Veith; Terry Jones; Victor M Corman
EPI_ISL_1013596 to 1013614	Department for Molecular Diagnostics, Centre for Medical Microbiology, Institute of Public Health, Montenegro	Charité Universitätsmedizin Berlin, Institut für Virologie	Barbara Mühlemann; Christian Drosten; Danijela Vujoševi; Julia Schneider; Julia Tesch; Jörn Beheim-Schwarzbach; Marija Govedarica; Talitha Veith; Terry Jones; Tobias Bleicker; Victor M Corman
EPI_ISL_420140, EPI_ISL_420142, EPI_ISL_420144	Department for Virology, Molecular Biology and Genome Research, R. G. Lugar Center for Public Health Research, National Center for Disease Control and Public Health (NCDC) of Georgia.	Department for Virology, Molecular Biology and Genome Research, R. G. Lugar Center for Public Health Research, National Center for Disease Control and Public Health (NCDC) of Georgia.	Adam Kotorashvili; Amiran Gamkrelidze; Amiran Gamkrelidze.; Ana Papkiauri; Ann Machablishvili; Anna Kasradze; Davit Tsaguria; Ekaterine Khmaladze; Ekaterine Zangaladze; Ekaterine Zhghenti; Giorgi Tomashvili; Gvantsa Brachveli; Gvantsa Chanturia; Irma Burjanadze; Ketevan Sidamonidze; Khatuna Zakhashvili; Lela Sabadze; Lela Urushadze; Magda Dgebuadze; Maia Alkhazashvili; Mari Gavashelidze; Mariam Zakalashvili; Marine Murtskhvaladze; Meri Pantsulaia; Nato Kotaria; Nino Berishvili; Paata Imnadze; Roena Sukhiashvili; Tamar Jashiashvili; Tata Imnadze; Tea Tevdoradze
EPI_ISL_447055 to 447056, EPI_ISL_470876 to 470877, EPI_ISL_471529, EPI_ISL_477169, EPI_ISL_481380, EPI_ISL_481483, EPI_ISL_754180 to 754181, EPI_ISL_754195, EPI_ISL_763062, EPI_ISL_1048367			
see above	Department for Virology, Molecular Biology and Genome Research, R. G. Lugar Center for Public Health Research, National Center for Disease Control and Public Health (NCDC) of Georgia.	Department for Virology, Molecular Biology and Genome Research, R. G. Lugar Center for Public Health Research, National Center for Disease Control and Public Health (NCDC) of Georgia.	Adam Kotorashvili; Amiran Gamkrelidze.; Ana Papkiauri; Ann Machablishvili; Anna Kasradze; Davit Tsaguria; Ekaterine Khmaladze; Ekaterine Zangaladze; Ekaterine Zhghenti; Giorgi Gogoladze; Giorgi Tomashvili; Gvantsa Brachveli; Gvantsa Chanturia; Irma Burjanadze; Ketevan Sidamonidze; Khatuna Zakhashvili; Lela Sabadze; Lela Urushadze; Magda Dgebuadze; Maia Alkhazashvili; Mari Gavashelidze; Mariam Zakalashvili; Marine Murtskhvaladze; Meri Pantsulaia; Nato Kotaria; Nino Berishvili; Paata Imnadze; Roena Sukhiashvili; Salome Javashvili; Tamar Jashiashvili; Tata Imnadze; Tea Tevdoradze
EPI_ISL_632935	Department of Acute Infectious Diseases Control and Prevention, Yunnan Provincial Center for Disease Control and Prevention	Department of Acute Infectious Diseases Control and Prevention, Yunnan Provincial Center for Disease Control and Prevention	Jienan Zhou; Meiling Zhang; Senquan Jia; Xiaonan Zhao; Xiaoqing Fu
EPI_ISL_632934	Department of Acute Infectious Diseases Control and Prevention,Yunnan Provincial Center for Disease Control and Prevention	Department of Acute Infectious Diseases Control and Prevention,Yunnan Provincial Center for Disease Control and Prevention	Jienan Zhou; Meiling Zhang; Senquan Jia; Xiaonan Zhao; Xiaoqing Fu
EPI_ISL_515082 to 515112	Department of Biochemistry, Cell and Molecular Biology	WACCBIP, University of Ghana	A.K.; Adu, B.; Amenga-Etego; Ampofo, W.; Amuzu; Anang; Arjarquah, A.; Asante, I.; Awandare; Bediako, Y.; Boatemaa, L.; Bonney, E.; Bonney, K.; C.M.; D.S.; Eshun, M.; G.A.; G.B.; J.K.; J.M.; Kotey, E.; Kumordjie, S.; Kyei; L.N.; Magnussen, V.; Morang'a; Mutungi; Ngoi; Quashie, P.; Tei-Maya, F.
EPI_ISL_884826 to 884856, EPI_ISL_944646 to 944735	Department of Biochemistry, Cell and Molecular Biology, West African Centre for Cell Biology of Infectious Pathogens (WACCBIP), University of Ghana	Department of Biochemistry, Cell and Molecular Biology, West African Centre for Cell Biology of Infectious Pathogens (WACCBIP), University of Ghana	A.-K.; A.B.; Abass; Adusei-Poku, M.; Akoriyea; Amenga-Etego; Amoako, E.; Ampofo; Amuzu; Asante, I.; Awandare; Bediako, Y.; Boakye; Bonney; Bonney, E.; C.M.; D.S.; Diallo; E.B.; G.A.; J.H.; J.K.; J.M.; Kibinge, N.; Kumi-Ansah, F.; L.N.; Magnussen, V.; Mohammed, A.; Morang'a; N.T.; Ndam; Ngoi; O.D.; Odoom; Odoom, T.; Ofori-Boadu, L.; Quansah; Quashie, P.; S.K.; Saiid, S.; Tapela, K.; Tei-Maya, F.; W.K.
EPI_ISL_907075, EPI_ISL_956332	Department of Biology, University of Basrah	Department of Biology, University of Basrah	A.I.; Abu-Ali; H.F. and Al-Badran; H.M. and Al-Badran; I.F.
EPI_ISL_1265370	Department of Biomedical, Surgical and Dental Sciences and Istituto Clinico Città Studi, University of Milan	Department of Biomedical, Surgical and Dental Sciences and Istituto Clinico Città Studi, University of Milan	Delbue Serena; Dolci Maria; Ferrante Pasquale; Signorini Lucia; Ticozzi Rosalia
EPI_ISL_411929	Department of Clinical Diagnostics	Department of Clinical Diagnostics	C.K.; Choe; Choi; G.-W.; J.-S. and Oh; J.Y.; Kang; Kim; Kim, N.; Kwon; Lee; M.-D.; M.-W.; N.-J.; P.G.; Park; S.-J.; Seo; Seong; W.B.; Yun, J.
EPI_ISL_412034 to 412039	Department of Clinical Laboratory, Tongji Medical College, Huazhong University of Science and Technology	Department of Clinical Laboratory, Tongji Medical College, Huazhong University of Science and Technology	Liu, W.; Song, H.; Sun; Xiang, R.; Y; Z. and Liu; Zhang, Q.
EPI_ISL_429239	Department of Clinical Laboratory, the First People's Hospital of Yunnan Province	Department of Clinical Laboratory, the First People's Hospital of Yunnan Province	Guiqian Zhang; Xin Fan; Ya Xu; Yi Sun; Yu Zhang; Ziqin Dian
EPI_ISL_416997, EPI_ISL_417004 to 417006, EPI_ISL_417008 to 417009, EPI_ISL_417011 to 417023, EPI_ISL_417025, EPI_ISL_418623 to 418666, EPI_ISL_421182 to 421214, EPI_ISL_424628 to 424665, EPI_ISL_427340 to 427390, EPI_ISL_447120 to 447162, EPI_ISL_455958 to 455979, EPI_ISL_471427 to 471437, EPI_ISL_484693 to 484708, EPI_ISL_498127 to 498151, EPI_ISL_498628 to 498629, EPI_ISL_515055 to 515081, EPI_ISL_540469 to 540578, EPI_ISL_581575 to 581667, EPI_ISL_582027, EPI_ISL_626231 to 626339, EPI_ISL_636605 to 636685, EPI_ISL_641557 to 641606, EPI_ISL_644611 to 644626, EPI_ISL_661201 to 661251, EPI_ISL_666871 to 666889, EPI_ISL_678399 to 678485, EPI_ISL_681264 to 681270, EPI_ISL_707714 to 707770, EPI_ISL_708453 to 708459, EPI_ISL_722926 to 722966, EPI_ISL_737310 to 737371, EPI_ISL_737393 to 737394, EPI_ISL_812122, EPI_ISL_824790 to 824863, EPI_ISL_832207 to 832242, EPI_ISL_833185 to 833190, EPI_ISL_856722 to 856754, EPI_ISL_872038 to 872050, EPI_ISL_872057 to 872064, EPI_ISL_872069 to 872087, EPI_ISL_872105 to 872109, EPI_ISL_872112 to 872116, EPI_ISL_872122, EPI_ISL_872127 to 872133, EPI_ISL_872135 to 872145, EPI_ISL_872148 to 872149, EPI_ISL_872153 to 872155, EPI_ISL_925362 to 925496, EPI_ISL_930569 to 930571, EPI_ISL_930575, EPI_ISL_930603, EPI_ISL_930609 to 930610, EPI_ISL_930615 to 930620, EPI_ISL_935654 to 935673, EPI_ISL_1036593, EPI_ISL_1040438 to 1040451, EPI_ISL_1058515 to 1058519, EPI_ISL_1058527, EPI_ISL_1058558 to 1058559, EPI_ISL_1058566, EPI_ISL_1058571, EPI_ISL_1058576 to 1058578, EPI_ISL_1058603, EPI_ISL_1058619 to 1058622, EPI_ISL_1076367, EPI_ISL_1076369, EPI_ISL_1076381, EPI_ISL_1076411 to 1076412, EPI_ISL_1076414, EPI_ISL_1076419, EPI_ISL_1076421, EPI_ISL_1076431, EPI_ISL_1076448 to 1076450, EPI_ISL_1076452 to 1076453, EPI_ISL_1076455 to 1076457, EPI_ISL_1076459 to 1076460, EPI_ISL_1081135, EPI_ISL_1081138, EPI_ISL_1081141, EPI_ISL_1121173, EPI_ISL_1121184, EPI_ISL_1121190, EPI_ISL_1121197, EPI_ISL_1121203, EPI_ISL_1121217, EPI_ISL_1123371, EPI_ISL_1171479 to 1171490, EPI_ISL_1241689, EPI_ISL_1241693 to 1241697, EPI_ISL_1241700, EPI_ISL_1241703, EPI_ISL_1241706 to 1241708, EPI_ISL_1241754 to 1241759, EPI_ISL_1241819 to 1241830			
see above	Department of Clinical Microbiology	GIGA Medical Genomics	Artesi Maria; Axelle Chaslain; Bontems Sébastien; Boreux Raphaël; Bouchra Boujemla; Bours Vincent.; Cecile Meex; Celine Fombellida-Lopez; Cécile Meex; Céline Fombellida-Lopez; Durkin Keith; Gilles Darcis; Hayette Marie-Pierre; Justine Defêche; Keith Durkin; Maria Artesi; Marie-Pierre Hayette; Meex Cécile; Melin Pierrette; Michel Moutschen; Nathalie Renotte; Pierrette Melin; Raphael Boreux; Raphaël Boreux; Sebastien Bontems; Sébastien Bontems; Vincent Bours; Vincent Bours.
EPI_ISL_429262 to 429318, EPI_ISL_429320 to 429332, EPI_ISL_451988 to 452100	Department of Clinical Microbiology, Copenhagen University Hospital, Hvidovre, Kettegaard Alle 30, 2650 Hvidovre.	Albertsen lab, Department of Chemistry and Bioscience, Aalborg University, Denmark	Rasmus Kirkegaard
EPI_ISL_417183 to 417184, EPI_ISL_417187 to 417188, EPI_ISL_417190, EPI_ISL_417193, EPI_ISL_417195, EPI_ISL_417197, EPI_ISL_417199, EPI_ISL_418815, EPI_ISL_419213 to 419216, EPI_ISL_419218 to 419219, EPI_ISL_419224 to 419229, EPI_ISL_419232, EPI_ISL_419241, EPI_ISL_419243 to 419252, EPI_ISL_420455			
see above	Department of Clinical Pathology, Pamela Youde Nethersole Eastern Hospital	Department of Health Technology and Informatics, Faculty of Health and Social Science, The Hong Kong Polytechnic University	Alan Ka-Lun WU; Alex Yat-Man HO; Barry Kin-Chung WONG; Chong-Yee YAU; David Ho-Keung SHUM; Eugene Yuk-Keung TSO; Gilman Kit-Hang SIU; Hiu-Yin LAO; Kam-Tong YIP; Kam-Tong Yip; Kenneth Siu-Sing LEUNG; Kingsley King-Gee TAM; Kit-Man SIN; Kitty Sau-Chun FUNG; Kwok-Cheung LUNG; Lam-Kwong LEE; Man-Chun CHAN; Ming-Pan CHOI; Miranda Chong-Yee YAU; Raymond Wai-To LIU; Sandy Ka-Yee CHAU; Shea Ping YIP; Tak-Lun QUE; Tak-Lun Que; Timothy Ting-Leung NG; Wai-Shing LEUNG; Wing Cheong YAM; Wing-Kin TO; Yuk-Yung NG
EPI_ISL_419242	Department of Clinical Pathology, Tuen Mun Hospital	Department of Health Technology and Informatics, Faculty of Health and Social Science, The Hong Kong Polytechnic University	Alan Ka-Lun WU; Alex Yat-Man HO; Barry Kin-Chung WONG; David Ho-Keung SHUM; Eugene Yuk-Keung TSO; Gilman Kit-Hang SIU; Hiu-Yin LAO; Kam-Tong YIP; Kenneth Siu-Sing LEUNG; Kingsley King-Gee TAM; Kit-Man SIN; Kitty Sau-Chun FUNG; Kwok-Cheung LUNG; Lam-Kwong LEE; Man-Chun CHAN; Ming-Pan CHOI; Miranda Chong-Yee YAU; Raymond Wai-To LIU; Sandy Ka-Yee CHAU; Shea Ping YIP; Tak-Lun QUE; Timothy Ting-Leung NG; Wai-Shing LEUNG; Wing Cheong YAM; Wing-Kin TO; Yuk-Yung NG
EPI_ISL_419231	Department of Clinical Pathology, Tuen Mun Hospital, 23 Tsing Chung Koon Road, Tuen Mun, N.T.	Department of Health Technology and Informatics, Faculty of Health and Social Science, The Hong Kong Polytechnic University	Alan Ka-Lun WU; Alex Yat-Man HO; Barry Kin-Chung WONG; David Ho-Keung SHUM; Eugene Yuk-Keung TSO; Gilman Kit-Hang SIU; Hiu-Yin LAO; Kam-Tong YIP; Kenneth Siu-Sing LEUNG; Kingsley King-Gee TAM; Kit-Man SIN; Kitty Sau-Chun FUNG; Kwok-Cheung LUNG; Lam-Kwong LEE; Man-Chun CHAN; Ming-Pan CHOI; Miranda Chong-Yee YAU; Raymond Wai-To LIU; Sandy Ka-Yee CHAU; Shea Ping YIP; Tak-Lun QUE; Timothy Ting-Leung NG; Wai-Shing LEUNG; Wing Cheong YAM; Wing-Kin TO; Yuk-Yung NG
EPI_ISL_1130784	Department of Dermatology	Switzerland	Department of Biosystems Science and Engineering; ETH Zürich
EPI_ISL_481251 to 481252, EPI_ISL_481254 to 481255, EPI_ISL_481257 to 481261, EPI_ISL_481263	Department of Emerging Infectious Diseases, Institute of Tropical Medicine, Nagasaki University	Department of Emerging Infectious Diseases, Institute of Tropical Medicine, Nagasaki University	Haruka Abe; Jiro Yasuda; Rokusuke Yoshikawa; Yuichiro Furusato
EPI_ISL_855345 to 855352	Department of Epidemiology, Infectious Disease Control and Prevention, Hiroshima University, Japan	Department of Epidemiology, Infectious Disease Control and Prevention, Hiroshima University, Japan	Eisaku Kishita; Junko Tanaka; Kazuaki Takahashi; Ko Ko; Masao Kuwabara; Shintaro Nagashima
EPI_ISL_507206 to 507215, EPI_ISL_605792	Department of Experimental Modeling and Pathogenesis of Infectious Diseases	WHO National Influenza Centre Russian Federation	Alekseev A.Yu.; Andrey Komissarov; Anna Ivanova; Artem Fadeev; Daria Danilenko; Kseniya Komissarova; Mariia Sergeeva; Shestopalov A.M.; Sobolev I.A.
EPI_ISL_487276	Department of Food Safety, Nutrition and Veterinary public health, Istituto Superiore di Sanita'	Department of Biomedical, Surgical and Dental Sciences and Department of Biomedical Sciences for Health	Anselmi, G.; Basilico, N.; Binda, S.; D'Alessandro, S.; Delbue, S.; Ferrante, P.; Galli, C.; Parapini, S.; Pariani, E.; Primache, V.; Signorini, L.

EPI_ISL_458008 to 458011, EPI_ISL_458014	Department of Food Safety, Nutrition and Veterinary public health, Istituto Superiore di Sanita'	Department of Food Safety, Nutrition and Veterinary public health, Istituto Superiore di Sanita'	Bonadonna, L.; Bonanno Ferraro, G.; Iaconelli, M.; La Rosa, G.; Lucentini, L.; Mancini, P.; Suffredini, E.; Veneri, C.
EPI_ISL_1280092 to 1280164	Department of Genetics, Medirex	Laboratory of Genomics and Bioinformatics, Comenius University Science Park	Anna Giová; Diana Rusáková; Gabriel Minárik; Jaroslav Budiš; Miroslav Böhmer; Renáta Lukaková; Tatiana Sedláková; Tomáš Szemes; Werner Krampl
EPI_ISL_1121893, EPI_ISL_1121900, EPI_ISL_1121945 to 1121946, EPI_ISL_1121950	Department of Health & Mental Hygiene Central Harlem	New York City Public Health Laboratory	Jade Wang; et al.
EPI_ISL_1121925, EPI_ISL_1121927 to 1121928, EPI_ISL_1121960	Department of Health & Mental Hygiene Chelsea	New York City Public Health Laboratory	Jade Wang; et al.
EPI_ISL_1121887, EPI_ISL_1121899, EPI_ISL_1121905 to 1121908, EPI_ISL_1121932 to 1121936	Department of Health & Mental Hygiene Corona	New York City Public Health Laboratory	Jade Wang; et al.
EPI_ISL_1121890 to 1121892, EPI_ISL_1121904, EPI_ISL_1121938 to 1121939	Department of Health & Mental Hygiene Crown Heights	New York City Public Health Laboratory	Jade Wang; et al.
EPI_ISL_1121889, EPI_ISL_1121901 to 1121903, EPI_ISL_1121937	Department of Health & Mental Hygiene Fort Greene	New York City Public Health Laboratory	Jade Wang; et al.
EPI_ISL_1121915 to 1121918, EPI_ISL_1121924, EPI_ISL_1121951 to 1121959	Department of Health & Mental Hygiene Jamaica	New York City Public Health Laboratory	Jade Wang; et al.
EPI_ISL_1121888, EPI_ISL_1121913, EPI_ISL_1121919 to 1121923, EPI_ISL_1121926, EPI_ISL_1121940 to 1121942, EPI_ISL_1121944, EPI_ISL_1121947 to 1121949			
see above	Department of Health & Mental Hygiene Morrisania	New York City Public Health Laboratory	Jade Wang; et al.
EPI_ISL_1121894, EPI_ISL_1121909 to 1121912, EPI_ISL_1121929 to 1121931	Department of Health & Mental Hygiene PHL	New York City Public Health Laboratory	Jade Wang; et al.
EPI_ISL_1121914, EPI_ISL_1121943	Department of Health & Mental Hygiene Riverside	New York City Public Health Laboratory	Jade Wang; et al.
EPI_ISL_610163 to 610226	Department of Health Technology and Informatics, The Hong Kong Polytechnic University	Department of Health Technology and Informatics, The Hong Kong Polytechnic University	A.K.-L.; A.Y.-M.; B.K.-C.; C.T.-M.; Chan; Chau; D.H.-K.; Fung; G.K.-H.; H.-Y.; Ho; J.S.-L.; K.-T.; K.K.-G.; K.S.-C.; K.S.-S.; L.-K.; Lai; Lao; Lee; Leung; Luk, K.; M.C.-Y.; Ng; Que; S.K.-Y.; S.P.; Shum; Siu; T.-L.; T.T.-L.; Tam; To; W.-K.; W.C.; Wong; Wu; Y.W.-M.; Yam; Yau; Yip
EPI_ISL_738084	Department of Health Technology and Informatics, Hong Kong Polytechnic University of Hong Kong	Department of Health Technology and Informatics, Hong Kong Polytechnic University of Hong Kong	A.K.-L.; A.Y.-M.; B.K.-C.; Chan; Chau; Choi; D.H.-K.; E.Y.-K.; Fung; G.K.-H.; H.-Y.; Ho; K.-C.; K.-T.; K.K.-G.; K.S.-C.; K.S.-S.; L.-K.; Lao; Lee; Leung; Liu; Lung; M.-C.; M.-P.; M.C.-Y.; Ng; Que; R.W.-T.; S.-P.; S.K.-Y.; Shum; T.-L.Q.; T.T.-L.; Tam; To; Tso; W.-K.; W.-S.; W.C. and Siu; Wong; Wu; Yam; Yau; Yip
EPI_ISL_1018118 to 1018217, EPI_ISL_1018229 to 1018328, EPI_ISL_1019637, EPI_ISL_1019640, EPI_ISL_1019642, EPI_ISL_1019645, EPI_ISL_1019647, EPI_ISL_1019650, EPI_ISL_1019653, EPI_ISL_1019655, EPI_ISL_1019658, EPI_ISL_1019660, EPI_ISL_1019663, EPI_ISL_1019665, EPI_ISL_1019668, EPI_ISL_1019671, EPI_ISL_1019674, EPI_ISL_1019676, EPI_ISL_1019678, EPI_ISL_1019681, EPI_ISL_1019683, EPI_ISL_1019686, EPI_ISL_1019688, EPI_ISL_1019691, EPI_ISL_1019693, EPI_ISL_1019696, EPI_ISL_1019699, EPI_ISL_1019702, EPI_ISL_1019705, EPI_ISL_1019707, EPI_ISL_1019710, EPI_ISL_1019713, EPI_ISL_1019715, EPI_ISL_1019718, EPI_ISL_1019720, EPI_ISL_1019722, EPI_ISL_1019724, EPI_ISL_1019726, EPI_ISL_1019729, EPI_ISL_1019731, EPI_ISL_1019734, EPI_ISL_1019737, EPI_ISL_1019739, EPI_ISL_1019742, EPI_ISL_1019745, EPI_ISL_1019748, EPI_ISL_1019750, EPI_ISL_1019753, EPI_ISL_1019756, EPI_ISL_1019759, EPI_ISL_1019762, EPI_ISL_1019764, EPI_ISL_1019767, EPI_ISL_1019770, EPI_ISL_1019772, EPI_ISL_1019775, EPI_ISL_1019778, EPI_ISL_1019780, EPI_ISL_1019783, EPI_ISL_1019785, EPI_ISL_1019788, EPI_ISL_1019790, EPI_ISL_1019793, EPI_ISL_1019795, EPI_ISL_1019798, EPI_ISL_1019800, EPI_ISL_1019803, EPI_ISL_1019805, EPI_ISL_1019807, EPI_ISL_1019810, EPI_ISL_1019813, EPI_ISL_1019815, EPI_ISL_1019818, EPI_ISL_1019820, EPI_ISL_1019823, EPI_ISL_1019825, EPI_ISL_1019828, EPI_ISL_1019830, EPI_ISL_1019833, EPI_ISL_1019836, EPI_ISL_1019838, EPI_ISL_1019841, EPI_ISL_1019843, EPI_ISL_1019846, EPI_ISL_1019849, EPI_ISL_1019851, EPI_ISL_1019853, EPI_ISL_1019855, EPI_ISL_1019858, EPI_ISL_1019861, EPI_ISL_1019863, EPI_ISL_1019866, EPI_ISL_1019869, EPI_ISL_1019872, EPI_ISL_1019875, EPI_ISL_1019877, EPI_ISL_1019880, EPI_ISL_1019882, EPI_ISL_1019885, EPI_ISL_1019888, EPI_ISL_1019890, EPI_ISL_1019893, EPI_ISL_1020021 to 1020120, EPI_ISL_1020208 to 1020284, EPI_ISL_1159070 to 1159106, EPI_ISL_1289435 to 1289446			
see above	Department of Health Technology and Informatics, The Hong Kong Polytechnic University	Department of Health Technology and Informatics, The Hong Kong Polytechnic University	Alan Ka-Lun Wu; Alex Yat-Man Ho; Barry Kin-Chung Wong; Chloe Toi-Mei Chan; David Ho-Keung Shum; Denise Sze-Hang Wong; Gilman Kit-Hang Siu; Hiu-Yin Lao; Jake Siu-Lun Leung; Kam-Tong Yip; Kenneth Siu-Sing Leung; Kingsley King-Gee Tam; Kitty Sau-Chun Fung; Kristine Luk; Lam-Kwong Lee; Miranda Chong-Yee Yau; Sandy Ka-Yee Chau; Shea Ping Yip; Tak-Lun Que; Timothy Ting-Leung Ng; Wing Cheong Yam; Wing-Kin To; Yvette Wai-Man Lai
EPI_ISL_1114470 to 1114471, EPI_ISL_1114492 to 1114493	Department of Health and Mental Hygiene Central Harlem	New York City Public Health Laboratory	Jade Wang; et al.
EPI_ISL_1114500 to 1114501, EPI_ISL_1114509, EPI_ISL_1114524	Department of Health and Mental Hygiene Chelsea	New York City Public Health Laboratory	Jade Wang; et al.
EPI_ISL_1114472 to 1114475, EPI_ISL_1114504 to 1114507, EPI_ISL_1114513 to 1114515	Department of Health and Mental Hygiene Corona	New York City Public Health Laboratory	Jade Wang; et al.
EPI_ISL_1114485, EPI_ISL_1114499, EPI_ISL_1114502, EPI_ISL_1114516 to 1114517	Department of Health and Mental Hygiene Crown Heights	New York City Public Health Laboratory	Jade Wang; et al.
EPI_ISL_1114494 to 1114496	Department of Health and Mental Hygiene Fort Greene	New York City Public Health Laboratory	Jade Wang; et al.
EPI_ISL_1114477 to 1114481, EPI_ISL_1114508, EPI_ISL_1114518	Department of Health and Mental Hygiene Jamaica	New York City Public Health Laboratory	Jade Wang; et al.
EPI_ISL_1114487 to 1114491	Department of Health and Mental Hygiene Morrisania	New York City Public Health Laboratory	Jade Wang; et al.
EPI_ISL_1114469, EPI_ISL_1114476, EPI_ISL_1114482 to 1114484, EPI_ISL_1114486, EPI_ISL_1114510, EPI_ISL_1114519 to 1114523	Department of Health and Mental Hygiene Public Health Laboratory	New York City Public Health Laboratory	Jade Wang; et al.
EPI_ISL_1114497 to 1114498, EPI_ISL_1114503	Department of Health and Mental Hygiene Riverside	New York City Public Health Laboratory	Jade Wang; et al.
EPI_ISL_417444	Department of Healthcare Biotechnology, National University of Sciences and Technology (NUST)	Department of Healthcare Biotechnology, National University of Sciences and Technology (NUST)	Corman; Ghani, E.; H.A.; Janjua; Javed, A.; Niazi; S.K.; Saqib, M.; V.M. and Zohaib, A.
EPI_ISL_632964, EPI_ISL_823970, EPI_ISL_823973, EPI_ISL_823975, EPI_ISL_883326, EPI_ISL_883332, EPI_ISL_883395 to 883396, EPI_ISL_883403, EPI_ISL_937191 to 937192, EPI_ISL_937248 to 937250, EPI_ISL_966385, EPI_ISL_966401, EPI_ISL_966469, EPI_ISL_1017989 to 1017990, EPI_ISL_1064758, EPI_ISL_1064768, EPI_ISL_1064810, EPI_ISL_1064827 to 1064828, EPI_ISL_1064851 to 1064852, EPI_ISL_1064889, EPI_ISL_1064898, EPI_ISL_1114511 to 1114512, EPI_ISL_1200562, EPI_ISL_1200599, EPI_ISL_1261800, EPI_ISL_1261830, EPI_ISL_1261853, EPI_ISL_1261867			
see above	Department of Homeless Services	New York City Public Health Laboratory	Jade Wang; et al.
EPI_ISL_529151 to 529154, EPI_ISL_529162 to 529166	Department of Immunology, The Scripps Research Institute	Andersen lab at Scripps Research	Allison Smither; Antoinette Bell; Arnaud Drouin; Austin; B. with SEARCH Alliance San Diego; Basler, T.; Dahlene Fusco; Gilberto Sabino-Santos; I. with SEARCH Alliance San Diego; Kaylynn Genemaras; Lilia Melnik; Mchardy; Patricia Snarski; Quigley, M.; Robert Garry with SEARCH Alliance San Diego; Shephard, J.; Stefanski, E.
EPI_ISL_524435 to 524436	Department of Immunology, The Scripps Research Institute	Department of Immunology, The Scripps Research Institute	Bell, A.; Drouin, A.; Fusco, D.; Garry, R.; Genemaras, K.; J.H.; Melnik, L.; Pride, D.; S.D.; SEARCH Alliance; Sabino-Santos, G.; Shin; Smither, A.; Snarski, P.
EPI_ISL_522407 to 522409	Department of Infection Prevention and Infectious Diseases,	Department of Infection Prevention and Infectious Diseases,	Fritsch, J.; Holzmann, T.; Schneider-Brachert, W.

	University Hospital Regensburg	University Hospital Regensburg	
EPI_ISL_513298 to 513307	Department of Infection Prevention and Infectious Diseases, University Hospital Regensburg	University Hospital Regensburg	Fritsch, J.; Holzmann, T.; Schneider-Brachert, W.
EPI_ISL_605929 to 605930	Department of Infectious Disease Prevention and Control, Henan Provincial Center for Disease Control and Prevention	Department of Infectious Disease Prevention and Control, Henan Provincial Center for Disease Control and Prevention	Guo, W.; Hu, X.; Huang, X.; Li, D.; Li, X.; Lu, S.; Wu, B.; Ye, Y.
EPI_ISL_1255104	Department of Infectious Disease Prevention and Control, Henan Provincial Center for Disease Control and Prevention	Department of Infectious Disease Prevention and Control, Henan Provincial Center for Disease Control and Prevention	B. and Huang, X.; Li, X.; Lu, S.; Wu
EPI_ISL_568556 to 568576, EPI_ISL_636989 to 637021, EPI_ISL_637075, EPI_ISL_918167 to 918176, EPI_ISL_1020317 to 1020322, EPI_ISL_1020324 to 1020327	Department of Infectious Diseases and Immunology, National Hospital Organization Nagoya Medical Center	Clinical Research Center, National Hospital Organization Nagoya Medical Center	Hirotaka Ode; Kazuhiro Matsuoka; Mai Kubota; Masakazu Matsuda; Mayumi Imahashi; Miho Nakasuji; Mikiko Mori; Nakasuji Miho; Yasumasa Iwatani; Yoshihiro Nakata; Yoshiyuki Yokomaku
EPI_ISL_578703	Department of Infectious Diseases, Gothenburg University	Department of Infectious Diseases, Gothenburg University	Nystrom, K.
EPI_ISL_524474, EPI_ISL_524480 to 524481	Department of Infectious Diseases, Cantonal Hospital Baden	Institute of Medical Virology, University of Zurich	Alexandra Trkola; Andrea Zbinden; Fiona Steiner; Gabriela Ziltener; Jon Huder; Jürg Böni; Maryam Zaheri; Michael Huber; Patrick Redli; Riccarda Capaul; Stefan Schmutz; Verena Kufner
EPI_ISL_457699 to 457700, EPI_ISL_457721, EPI_ISL_457724, EPI_ISL_457728, EPI_ISL_457732, EPI_ISL_457736, EPI_ISL_457749			
see above	Department of Infectious Diseases, Istituto Superiore di Sanità, Roma , Italy	Army Medical and Veterinary Research Center	Alessandra Lo Presti; Anna Anselmo; Antonella Fortunato; Antonella Marchi; Concetta Fabiani Silvia Fillo; Concetta Fabiani Silvia Fillo; Eleonora Benedetti; Florigio Lista; Francesco Giordani; Giovanni Faggioni; Nino D'Amore; Paola Stefanelli; Riccardo De Sanctis; Stefano Fiore; Vanessa Vera Fain
EPI_ISL_412973	Department of Infectious Diseases, Istituto Superiore di Sanità, Roma , Italy	Virology Laboratory, Scientific Department, Army Medical Center	Andrea Ciammaruconi; Anna Anselmo; Antonella Fortunato; Antonella Marchi; Concetta Fabiani; Eleonora Benedetti; Florigio Lista; Giovanni Faggioni; Paola Stefanelli; Riccardo De Santis; Silvia Fillo; Stefano Fiore; Stefano Palomba
EPI_ISL_856889, EPI_ISL_856904	Department of Infectious Diseases, Istituto Superiore di Sanità, Roma, Italy	Virology Laboratory, Scientific Department, Army Medical Center	Alessandra Lo Presti; Angela Di Martino; Anna Anselmo; Antonella Fortunato; Florigio Lista; Francesco Giordani; Giovanni Faggioni; Nino D'Amore; Paola Stefanelli; Riccardo De Sanctis; Silvia Fillo; Stefano Fiore; Vanessa Vera Fain
EPI_ISL_856888	Department of Infectious Diseases, Istituto Superiore di Sanità, Roma, Italy; A.O.R. San Carlo di Potenza, Laboratorio Microbiologia e Virologia, Potenza, Italy	Virology Laboratory, Scientific Department, Army Medical Center	Alessandra Lo Presti; Angela Di Martino; Anna Anselmo; Antonella Fortunato; Antonio Picerno; Florigio Lista; Francesco Giordani; Giovanni Faggioni; Nino D'Amore; Paola Stefanelli; Riccardo De Sanctis; Silvia Fillo; Stefano Fiore; Vanessa Vera Fain
EPI_ISL_856890	Department of Infectious Diseases, Istituto Superiore di Sanità, Roma, Italy; AO Annunziata, UOC Microbiologia e Virologia, Cosenza, Italy	Virology Laboratory, Scientific Department, Army Medical Center	Alessandra Lo Presti; Angela Di Martino; Anna Anselmo; Antonella Fortunato; Cristina Giraldi; Florigio Lista; Francesco Giordani; Giovanni Faggioni; Nino D'Amore; Paola Stefanelli; Riccardo De Sanctis; Silvia Fillo; Stefano Fiore; Vanessa Vera Fain
EPI_ISL_856905 to 856906	Department of Infectious Diseases, Istituto Superiore di Sanità, Roma, Italy; AO S.M. della Misericordia, S.C. Microbiologia, Perugia, Italy	Virology Laboratory, Scientific Department, Army Medical Center	Alessandra Lo Presti; Angela Di Martino; Anna Anselmo; Antonella Fortunato; Barbara Camilloni; Florigio Lista; Francesco Giordani; Giovanni Faggioni; Nino D'Amore; Paola Stefanelli; Riccardo De Sanctis; Silvia Fillo; Stefano Fiore; Vanessa Vera Fain
EPI_ISL_856871, EPI_ISL_856891 to 856892	Department of Infectious Diseases, Istituto Superiore di Sanità, Roma, Italy; AORN dei Colli, UOC Microbiologia Virologia, Napoli, Italy	Virology Laboratory, Scientific Department, Army Medical Center	Alessandra Lo Presti; Angela Di Martino; Anna Anselmo; Antonella Fortunato; Florigio Lista; Francesco Giordani; Giovanni Faggioni; Luigi Atripaldi; Nino D'Amore; Paola Stefanelli; Riccardo De Sanctis; Silvia Fillo; Stefano Fiore; Vanessa Vera Fain
EPI_ISL_856900 to 856901	Department of Infectious Diseases, Istituto Superiore di Sanità, Roma, Italy; AOUP V. Emanuele di Catania, P.O. Gaspare Rodolico, Catania, Italy	Virology Laboratory, Scientific Department, Army Medical Center	Alessandra Lo Presti; Angela Di Martino; Anna Anselmo; Antonella Fortunato; Florigio Lista; Francesco Giordani; Giovanni Faggioni; Guido Scalia; Nino D'Amore; Paola Stefanelli; Riccardo De Sanctis; Silvia Fillo; Stefano Fiore; Vanessa Vera Fain
EPI_ISL_856902	Department of Infectious Diseases, Istituto Superiore di Sanità, Roma, Italy; AS Alto Adige, Microbiologia e Virologia, Bolzano, Italy	Virology Laboratory, Scientific Department, Army Medical Center	Alessandra Lo Presti; Angela Di Martino; Anna Anselmo; Antonella Fortunato; Elisabetta Pagani; Florigio Lista; Francesco Giordani; Giovanni Faggioni; Nino D'Amore; Paola Stefanelli; Riccardo De Sanctis; Silvia Fillo; Stefano Fiore; Vanessa Vera Fain
EPI_ISL_856898 to 856899	Department of Infectious Diseases, Istituto Superiore di Sanità, Roma, Italy; ASL Città di Torino, S.C. Microbiologia e Virologia, Torino, Italy	Virology Laboratory, Scientific Department, Army Medical Center	Alessandra Lo Presti; Angela Di Martino; Anna Anselmo; Antonella Fortunato; Florigio Lista; Francesco Giordani; Giovanni Faggioni; Nino D'Amore; Paola Stefanelli; Riccardo De Sanctis; Silvia Fillo; Stefano Fiore; Valeria Ghisetti; Vanessa Vera Fain
EPI_ISL_856893 to 856894	Department of Infectious Diseases, Istituto Superiore di Sanità, Roma, Italy; ASUITS - Ospedale Maggiore, S.C. UOC Igiene e Sanità Pubblica, Trieste, Italy	Virology Laboratory, Scientific Department, Army Medical Center	Alessandra Lo Presti; Angela Di Martino; Anna Anselmo; Antonella Fortunato; Florigio Lista; Francesco Giordani; Giovanni Faggioni; Nino D'Amore; Paola Stefanelli; Pierlanfranco D'Agaro; Riccardo De Sanctis; Silvia Fillo; Stefano Fiore; Vanessa Vera Fain
EPI_ISL_856887	Department of Infectious Diseases, Istituto Superiore di Sanità, Roma, Italy; AUOC Policlinico Bari, UOC Igiene, Bari, Italy	Virology Laboratory, Scientific Department, Army Medical Center	Alessandra Lo Presti; Angela Di Martino; Anna Anselmo; Antonella Fortunato; Florigio Lista; Francesco Giordani; Giovanni Faggioni; Maria Chironna; Nino D'Amore; Paola Stefanelli; Riccardo De Sanctis; Silvia Fillo; Stefano Fiore; Vanessa Vera Fain
EPI_ISL_856907	Department of Infectious Diseases, Istituto Superiore di Sanità, Roma, Italy; AUSL Valle d'Aosta, Aosta, Italy	Virology Laboratory, Scientific Department, Army Medical Center	Alessandra Lo Presti; Angela Di Martino; Anna Anselmo; Antonella Fortunato; Florigio Lista; Francesco Giordani; Giovanni Faggioni; Massimo Di Benedetto; Nino D'Amore; Paola Stefanelli; Riccardo De Sanctis; Silvia Fillo; Stefano Fiore; Vanessa Vera Fain
EPI_ISL_856880, EPI_ISL_856895 to 856896	Department of Infectious Diseases, Istituto Superiore di Sanità, Roma, Italy; Ospedali Riuniti, Laboratorio Virologia, Ancona, Italy	Virology Laboratory, Scientific Department, Army Medical Center	Alessandra Lo Presti; Angela Di Martino; Anna Anselmo; Antonella Fortunato; Florigio Lista; Francesco Giordani; Giovanni Faggioni; Nino D'Amore; Paola Stefanelli; Patrizia Bagnarelli; Riccardo De Sanctis; Silvia Fillo; Stefano Fiore; Vanessa Vera Fain
EPI_ISL_856881, EPI_ISL_856897	Department of Infectious Diseases, Istituto Superiore di Sanità, Roma, Italy; PO Cardarelli, Laboratorio Analisi Microbiologia e Virologia, Campobasso, Italy	Virology Laboratory, Scientific Department, Army Medical Center	Alessandra Lo Presti; Angela Di Martino; Anna Anselmo; Antonella Fortunato; Florigio Lista; Francesco Giordani; Giovanni Faggioni; Massimiliano Scutellà; Nino D'Amore; Paola Stefanelli; Riccardo De Sanctis; Silvia Fillo; Stefano Fiore; Vanessa Vera Fain
EPI_ISL_856908	Department of Infectious Diseases, Istituto Superiore di Sanità, Roma, Italy; PO Madre Teresa di Calcutta, UOC. Laboratorio Analisi ULSS 6 Euganea, Padova, Italy	Virology Laboratory, Scientific Department, Army Medical Center	Alessandra Lo Presti; Angela Di Martino; Anna Anselmo; Antonella Fortunato; Florigio Lista; Francesco Giordani; Giacomo Mezzapelle; Giovanni Faggioni; Nino D'Amore; Paola Stefanelli; Riccardo De Sanctis; Silvia Fillo; Stefano Fiore; Vanessa Vera Fain
EPI_ISL_856884, EPI_ISL_856903	Department of Infectious Diseases, Istituto Superiore di Sanità, Roma, Italy; PO Santa Chiara, Microbiologia e Virologia, Trento, Italy	Virology Laboratory, Scientific Department, Army Medical Center	Alessandra Lo Presti; Angela Di Martino; Anna Anselmo; Antonella Fortunato; Florigio Lista; Francesco Giordani; Giovanni Faggioni; Nino D'Amore; Paola Stefanelli; Paolo Lanzafame; Riccardo De Sanctis; Silvia Fillo; Stefano Fiore; Vanessa Vera Fain
EPI_ISL_856886	Department of Infectious Diseases, Istituto Superiore di Sanità, Roma, Italy; PO Spirito Santo, UOC Microbiologia e Virologia Clinica, Pescara, Italy	Virology Laboratory, Scientific Department, Army Medical Center	Alessandra Lo Presti; Angela Di Martino; Anna Anselmo; Antonella Fortunato; Florigio Lista; Francesco Giordani; Giovanni Faggioni; Nino D'Amore; Paola Stefanelli; Paolo Fazii; Riccardo De Sanctis; Silvia Fillo; Stefano Fiore; Vanessa Vera Fain
EPI_ISL_856909	Department of Infectious Diseases, Istituto Superiore di Sanità, Roma, Italy; ULSS 8 Berica Vicenza, UOC Microbiologia, Vicenza, Italy	Virology Laboratory, Scientific Department, Army Medical Center	Alessandra Lo Presti; Angela Di Martino; Anna Anselmo; Antonella Fortunato; Florigio Lista; Francesco Giordani; Giovanni Faggioni; Mario Rassu; Nino D'Amore; Paola Stefanelli; Riccardo De Sanctis; Silvia Fillo; Stefano Fiore; Vanessa Vera Fain
EPI_ISL_855550, EPI_ISL_855553	Department of Infectious Diseases, Istituto Superiore di Sanità, Rome, Italy	Istituto Superiore di Sanità (ISS)	Alessandra Lo Presti; Angela Di Martino; Gabriele Vaccari; Giovanni Ianiro; Ilaria Di Bartolo; Luca De Sabato; Manuela Marra; Marco Crescenzi; Maria Carollo; Paola Stefanelli; Stefano Fiore
EPI_ISL_412974	Department of Infectious Diseases, Istituto Superiore di Sanità, Rome, Italy	Virology Laboratory, Scientific Department, Army Medical Center	Andrea Ciammaruconi; Antonella Fortunato; Antonella Marchi; Concetta Fabiani; Eleonora Benedetti; Filippo Molinari; Florigio Lista; Giancarlo Petralito; Giovanni Faggioni; Paola Stefanelli; Riccardo De Santis; Silvia Fillo; Stefano Fiore
EPI_ISL_1200635 to 1200637	Department of Infectious Diseases, Istituto Superiore di Sanità, Rome, Italy; AOR San Carlo, Potenza, Italy	Department of Infectious Diseases, Istituto Superiore di Sanità	Alessandra Lo Presti; Angela Di Martino; Angela Menchise; Anna Curci; Antonio Picerno; Lopizzo Teresa; Manuela Marra; Marco Crescenzi; Maria Carollo; Paola Stefanelli; Stefano Fiore

EPI_ISL_1181344	Department of Infectious Diseases, Istituto Superiore di Sanità, Rome, Italy; AOR San Carlo, Potenza, Italy	Istituto Superiore di Sanità (ISS)	Alessandra Lo Presti; Angela Di Martino; Angela Menchise; Anna Curci; Antonio Picerno; Lopizzo Teresa; Manuela Marra; Marco Crescenzi; Maria Carollo; Paola Stefanelli; Stefano Fiore
EPI_ISL_910331 to 910332	Department of Infectious Diseases, Istituto Superiore di Sanità, Rome, Italy; AOUP Paolo Giaccone, Palermo, Italy	Istituto Superiore di Sanità (ISS)	Alessandra Lo Presti; Angela Di Martino; Carmelo Maida; Daniela Di Naro; Fabio Tramuto; Francesco Vitale; Giulia Randazzo; Manuela Marra; Marco Crescenzi; Maria Carollo; Paola Stefanelli; Stefano Fiore
EPI_ISL_855554 to 855555	Department of Infectious Diseases, Istituto Superiore di Sanità, Rome, Italy; ASST G.O.M. Niguarda, Sezione Microbiologia e Virologia, Milano, Italy	Istituto Superiore di Sanità (ISS)	Alessandra Lo Presti; Angela Di Martino; Carlo Federico Perno; Gabriele Vaccari; Giovanni Ianiro; Ilaria Di Bartolo; Luca De Sabato; Manuela Marra; Marco Crescenzi; Maria Carollo; Paola Stefanelli; Stefano Fiore
EPI_ISL_1012923	Department of Infectious Diseases, Istituto Superiore di Sanità, Rome, Italy; ASST Sette Laghi, Varese, Italy	Istituto Superiore di Sanità (ISS)	Alessandra Lo Presti; Andreina Baj; Angela Di Martino; Angelo Genoni; Fabrizio Maggi; Federica Novazzi; Manuela Marra; Marco Crescenzi; Maria Carollo; Paola Stefanelli; Stefano Fiore
EPI_ISL_1181352, EPI_ISL_1198007	Department of Infectious Diseases, Istituto Superiore di Sanità, Rome, Italy; Azienda Ospedaliera Santa Maria di Terni, Terni, Italy	Istituto Superiore di Sanità (ISS)	Alessandra Lo Presti; Angela Di Martino; Augusto Scaccetti; Cinzia Di Giuli; Manuela Marra; Marco Crescenzi; Maria Carollo; Michele Palumbo; Paola Stefanelli; Stefano Fiore
EPI_ISL_1197038, EPI_ISL_1197040	Department of Infectious Diseases, Istituto Superiore di Sanità, Rome, Italy; Azienda Sanitaria Locale TO5, Torino, Italy	Istituto Superiore di Sanità (ISS)	Alessandra Allocco; Alessandra Lo Presti; Angela Di Martino; Cagnoli Claudia; Manuela Marra; Marco Crescenzi; Maria Carollo; Paola Stefanelli; Stefano Fiore
EPI_ISL_1181627	Department of Infectious Diseases, Istituto Superiore di Sanità, Rome, Italy; Azienda Usl Toscana sud est, Arezzo, Italy	Istituto Superiore di Sanità (ISS)	Agostino Ognibene; Alessandra Lo Presti; Angela Di Martino; Manuela Marra; Marco Crescenzi; Maria Carollo; Paola Stefanelli; Stefano Fiore
EPI_ISL_1198918, EPI_ISL_1199386, EPI_ISL_1199633	Department of Infectious Diseases, Istituto Superiore di Sanità, Rome, Italy; L.C. Laboratori Campisi s.r.l., Siracusa, Sicily, Italy	Istituto Superiore di Sanità (ISS)	Alessandra Lo Presti; Angela Di Martino; Campisi Corrado; Iemmolo Rosario; Manuela Marra; Marco Crescenzi; Maria Carollo; Paola Stefanelli; Stefano Fiore
EPI_ISL_855552	Department of Infectious Diseases, Istituto Superiore di Sanità, Rome, Italy; Ospedali Riuniti, Laboratorio Virologia, Ancona, Italy	Istituto Superiore di Sanità (ISS)	Alessandra Lo Presti; Angela Di Martino; Gabriele Vaccari; Giovanni Ianiro; Ilaria Di Bartolo; Luca De Sabato; Manuela Marra; Marco Crescenzi; Maria Carollo; Paola Stefanelli; Patrizia Bagnarelli; Stefano Fiore
EPI_ISL_1181347, EPI_ISL_1181349, EPI_ISL_1198794 to 1198796	Department of Infectious Diseases, Istituto Superiore di Sanità, Rome, Italy; U.O.S.D. Laboratorio Analisi Distretto 4 Ovest AULSS 9 Scaligera, Italy	Istituto Superiore di Sanità (ISS)	Alessandra Lo Presti; Angela Di Martino; Manuela Marra; Marco Crescenzi; Maria Carollo; Maria Pompilia Visconti; Paola Stefanelli; Rosa Sforza; Stefano Fiore
EPI_ISL_875687	Department of Infectious Diseases, Istituto Superiore di Sanità, Rome, Italy; UCO Igiene e Sanità Pubblica, ASUGI, Trieste, Italy; Laboratorio di Genomica ed Epigenomica sistema Argo, Area SciencePark, Trieste, Italy; Laboratorio di Virologia Molecolare, ICGEB, Trieste, Italy	Istituto Superiore di Sanità (ISS)	Alessandra Lo Presti; Alessandro Marcello; Angela Di Martino; Danilo Licastro; Ludovica Segat; Manuela Marra; Marco Crescenzi; Maria Carollo; Paola Stefanelli; Pierlanfranco D'Agaro; Stefano Fiore
EPI_ISL_1014545, EPI_ISL_1014675, EPI_ISL_1178582, EPI_ISL_1199634 to 1199635, EPI_ISL_1199707, EPI_ISL_1200004, EPI_ISL_1200515 to 1200516			
see above	Department of Infectious Diseases, Istituto Superiore di Sanità, Rome, Italy; Università degli Studi di Perugia, Perugia, Italy	Istituto Superiore di Sanità (ISS)	Alessandra Lo Presti; Angela Di Martino; Antonella Mencacci; Barbara Camilloni; Luca De Sabato; Manuela Marra; Marco Crescenzi; Maria Carollo; Paola Stefanelli; Stefano Fiore
EPI_ISL_1035871, EPI_ISL_1035900 to 1035901, EPI_ISL_1035966	Department of Infectious Diseases, Istituto Superiore di Sanità, Rome, Italy; Università degli Studi di Siena, Siena, Italy	Istituto Superiore di Sanità (ISS)	Alessandra Lo Presti; Angela Di Martino; Claudia Gandolfo; Gabriele Anichini; Gianni Gori Savellini; Manuela Marra; Marco Crescenzi; Maria Carollo; Maria Grazia Cusi; Paola Stefanelli; Stefano Fiore
EPI_ISL_644945 to 644999	Department of Infectious Diseases, Keio University School of Medicine, Tokyo, Japan	Center for Medical Genetics, Keio University School of Medicine, Tokyo, Japan	Haruhiko Siomi; Hirotsugu Ishizu; Kenjiro Kosaki; Kodai Abe; Yuka Iwasaki
EPI_ISL_893368 to 893719, EPI_ISL_939642, EPI_ISL_1168772 to 1169045	Department of Infectious Diseases, Kobe Institute of Health	Department of Infectious Diseases, Kobe Institute of Health	Kentaro Itokawa; Makoto Kuroda; Masanori Hashino; Noriko Nakanishi; Rina Tanaka; Ryohei Nomoto; Tomotada Iwamoto; Tsuyoshi Sekizuka
EPI_ISL_479821 to 479822, EPI_ISL_479855 to 479861, EPI_ISL_479868, EPI_ISL_479986 to 479989, EPI_ISL_480090 to 480102, EPI_ISL_480205 to 480207, EPI_ISL_480209 to 480220, EPI_ISL_892726 to 892807, EPI_ISL_894354 to 894363, EPI_ISL_895014 to 895094, EPI_ISL_896795 to 896854, EPI_ISL_898711 to 898809, EPI_ISL_902024 to 902053, EPI_ISL_902504 to 902552, EPI_ISL_906516 to 906518, EPI_ISL_906523, EPI_ISL_906527, EPI_ISL_1128148, EPI_ISL_1128158, EPI_ISL_1128161, EPI_ISL_1128198 to 1128272			
see above	Department of Infectious Diseases, Kobe Institute of Health	Pathogen Genomics Center, National Institute of Infectious Diseases	Hajime Kamiya; Kentaro Itokawa; Makoto Kuroda; Masanori Hashino; Motoi Suzuki; Rina Tanaka; Ryohei Nomoto; Tsuyoshi Sekizuka
EPI_ISL_411218 to 411220	Department of Infectious and Tropical Diseases, Bichat Claude Bernard Hospital, Paris	Laboratoire Virpath, CIRI U111, UCBL1, INSERM, CNRS, ENS Lyon	Alexandre Gaymard; Aurélien Traversier; Bruno Lina; Catherine Legras-Lachuer; Julien Fouret; Manuel Rosa-Calatrava; Olivier Terrier; Xavier Lescure; Yazdan Yazdanpanah
EPI_ISL_1301441	Department of Infectious and Tropical Diseases, Bichat Claude Bernard Hospital, Paris	NBACC	Aaron Phillips; Adam Bazinet; Amanda Graham; Brian Holland; David Miller; Denise Freeburger; Elyse Abbott; Gregory Williams; Jason Yolitz; Jennifer Biryukov; Jordan Bohannon; Karla Garcia; Katie Beck; M.J. Rosovitz; Melissa Krause; Michael Schuit; Paul Dabisch; Sean Lovett; Shanna Ratnesar-Shumate; Stewart Wood; Victoria Wahl; Wade Weaver
EPI_ISL_406596 to 406597, EPI_ISL_408430, EPI_ISL_410720, EPI_ISL_410984	Department of Infectious and Tropical Diseases, Bichat Claude Bernard Hospital, Paris	National Reference Center for Viruses of Respiratory Infections, Institut Pasteur, Paris	Angela Brisebarre; Flora Donati; Marion Barbet; Maud Vanpeene; Mélanie Albert; Méline Bizard; Sylvie Behillil; Sylvie van der Werf; Vincent Enouf; Xavier Lescure; Xavier Lescure.; Yazdan Yazdanpanah
EPI_ISL_485398, EPI_ISL_985394, EPI_ISL_1007662 to 1007679	Department of Internal Medicine, College of Medicine, Chosun University	Department of Internal Medicine, College of Medicine, Chosun University	D.-M.; Dong-Min Kim; Kim
EPI_ISL_413019 to 413020	Department of Internal Medicine, Triemli Hospital	Institute of Medical Virology, University of Zurich	Alexandra Trkola; Andrea Zbinden; Fiona Steiner; Gerhard Eich; Jon Huder; Jürg Böni; Maryam Zaheri; Michael Huber; Patrick Redli; Riccarda Capaul; Stefan Schmutz; Verena Kufner
EPI_ISL_476805 to 476812, EPI_ISL_476815	Department of Laboratory Medicine Tan Tock Seng Hospital	Department of Laboratory Medicine Tan Tock Seng Hospital	Barkham TMS; Chen YYC; Li C; Maurer-Stroh S; Nagarajan N; Sessions OM; Tang WY; Zair X
EPI_ISL_477172, EPI_ISL_477174 to 477175, EPI_ISL_477177 to 477178, EPI_ISL_477180, EPI_ISL_477182, EPI_ISL_477184, EPI_ISL_477187 to 477192, EPI_ISL_479482 to 479492, EPI_ISL_492978 to 492979			
see above	Department of Laboratory Medicine Tan Tock Seng Hospital	Department of Laboratory Medicine Tan Tock Seng Hospital	Barkham TMS; Chen YYC; Li C; Maurer-Stroh S; Nagarajan N; Sessions OM; Tang WY; Zair X
EPI_ISL_516801 to 516805	Department of Laboratory Medicine, Tan Tock Seng Hospital	Department of Laboratory Medicine, Tan Tock Seng Hospital	Barkham TMS; Chen YYC; Li C; Maurer-Stroh S; Nagarajan N; Sessions OM; Tang WY; Zair X
EPI_ISL_1233874, EPI_ISL_1233877 to 1233878, EPI_ISL_1306139 to 1306252	Department of Laboratory Medicine, Clinical Center, National Institutes of Health	Laboratory of Parasitic Diseases, Systems Genomics Section, National Institute of Allergy and Infectious Diseases, National Institutes of Health	Allison Roder; Elodie Ghedin; Heike Bailin; Jennifer Kwan; Jessica McCormick-Ell; Jung-ho Youn; Matthew Chung; Michael Bell; Rachel Mercado; Sanchita Das; Stephanie Banakis; Tara Palmore; Wei Wang
EPI_ISL_934548 to 934588, EPI_ISL_934655 to 934695, EPI_ISL_1008215 to 1008265, EPI_ISL_1008327 to 1008331, EPI_ISL_1008340 to 1008343, EPI_ISL_1008345, EPI_ISL_1008355 to 1008388, EPI_ISL_1117821, EPI_ISL_1180839 to 1180885, EPI_ISL_1209269 to 1209334			
see above	Department of Laboratory Medicine, Division of Clinical Virology, University of Medicine, Vienna	Bergthaler laboratory, CeMM Research Center for Molecular Medicine of the Austrian Academy of Sciences	Andreas Bergthaler; Anna Schedl; Bekir Erguner; Benedikt Agerer; Christoph Bock; Fabian Amman; Jan Laine; Lukas Endler; Maelle Le Moing; Martin Senekowitsch; Michael Schuster; Thomas Penz
EPI_ISL_408489, EPI_ISL_410218, EPI_ISL_413592, EPI_ISL_422407 to 422422, EPI_ISL_447614 to 447622, EPI_ISL_463007, EPI_ISL_534336, EPI_ISL_693302 to 693306, EPI_ISL_738064 to 738065, EPI_ISL_740547, EPI_ISL_872591 to 872599, EPI_ISL_1010728 to 1010731, EPI_ISL_1020315 to 1020316, EPI_ISL_1039160, EPI_ISL_1041957 to 1041958			
see above	Department of Laboratory Medicine, National Taiwan University Hospital	Microbial Genomics Core Lab, National Taiwan University Centers of Genomic and Precision Medicine	Chiao-Ling Li; Pei-Jer Chen; Shan-Chwen Chang; Shiou-Hwei Yeh; Sui-Yuan Chang; Ya-Yun Lai; You-Yu Lin
EPI_ISL_476795 to 476797, EPI_ISL_476813 to 476814, EPI_ISL_476816 to 476821, EPI_ISL_507002 to 507006, EPI_ISL_538435 to 538497, EPI_ISL_648043 to 648121, EPI_ISL_648610 to 648676, EPI_ISL_648678 to 648820			
see above	Department of Laboratory Medicine, Tan Tock Seng Hospital	Department of Laboratory Medicine, Tan Tock Seng Hospital	Barkham TMS; Chen YYC; Li C; Lim JX; Maurer-Stroh S; Nagarajan N; Sessions OM; Tang WY; Zair X
EPI_ISL_477170	Department of Laboratory, Medicine Tan Tock Seng Hospital	Department of Laboratory Medicine Tan Tock Seng Hospital	Barkham TMS; Chen YYC; Li C; Maurer-Stroh S; Nagarajan N; Sessions OM; Tang WY; Zair X

EPI_ISL_477171	Department of Laboratory, Medicine Tan Tock Seng Hospital	Department of Laboratory, Medicine Tan Tock Seng Hospital	Barkham TMS; Chen YYC; Li C; Maurer-Stroh S; Nagarajan N; Sessions OM; Tang WY; Zair X
EPI_ISL_803880 to 803888, EPI_ISL_803891 to 803893, EPI_ISL_803895	Department of Medical Biotechnologies, University of Siena	Laboratory of Infectious Diseases, Department of Biomedical and Clinical Sciences L. Sacco, University of Milan	Alessia Lai; Annalisa Bergna; Carla Della Ventura; Claudia Balotta; Filippo Dragoni; Gianguglielmo Zehender on behalf of SARS-CoV-2 ITALIAN RESEARCH ENTERPRISE-(SCIRE) Collaborative Group; Ilaria Vicenti; Maria Grazia Cusi; Massimo Galli; Maurizio Zazzi
EPI_ISL_710483 to 710484	Department of Medical Laboratory Sciences, Arab American University	Department of Medical Laboratory Sciences, Arab American University	Al-Jawabreh, A.; Al-Jawabreh, H.; Dumaidi, K.; Ereqat, S.; Nasereddin, A.
EPI_ISL_591012, EPI_ISL_591018 to 591019, EPI_ISL_775428 to 775437, EPI_ISL_775467, EPI_ISL_775523, EPI_ISL_796653, EPI_ISL_796681 to 796683, EPI_ISL_860262, EPI_ISL_906822 to 906825, EPI_ISL_964981 to 964983, EPI_ISL_1013457 to 1013458, EPI_ISL_1013488, EPI_ISL_1034324, EPI_ISL_1034330 to 1034331, EPI_ISL_1073945, EPI_ISL_1118459, EPI_ISL_1118520, EPI_ISL_1118602, EPI_ISL_1118610, EPI_ISL_1118614, EPI_ISL_1192252 to 1192253, EPI_ISL_1192266, EPI_ISL_1251417 to 1251419, EPI_ISL_1251470 to 1251471, EPI_ISL_1251631 to 1251633, EPI_ISL_1251824 to 1251826, EPI_ISL_1252003 to 1252004, EPI_ISL_1252491, EPI_ISL_1252551 to 1252555, EPI_ISL_1314528			
see above	Department of Medical Microbiology - section Molde, Molde Hospital	Norwegian Institute of Public Health, Department of Virology	Atiya R Ali; Debech Nadia; Engebretsen Serina Beate; Engebretsen Serina Beate Atiya R Ali; Garcia Llorente Ignacio; Hilde Elshaug; Hilde Vollan; Ignacio Garcia Llorente; Jon Bråte; Kamilla Heddeland Instefjord; Karoline Bragstad; Kathrine Stene-Johansen; Marie Paulsen Madsen; Olav Hungnes; Pedersen Benedikte Nevjen; Rasmus Riis Kopperud; Serina B Engebretsen
EPI_ISL_861717 to 861726, EPI_ISL_861729, EPI_ISL_977585 to 977590	Department of Medical Microbiology, Hospital Pengajar Universiti Putra Malaysia	Malaysia Genome Institute	Avisha Richards; Azrin Ahmad; Enizza Kasim; Hui-Yee Chee; Irni Suhayu Sapian; Mohd Faizal Abu Bakar; Mohd Noor Mat Isa; Muhammad MI; Narcisse Joseph; Nor Azfa Johari; Nor Zahrin Hasran; Nurhezreen Md Iqbal; Shamsidar Sopie; Siti Noraini Othman; Syafinaz Amin-Nordin; Yusuf Muhammad Noor
EPI_ISL_454416	Department of Medical Microbiology, Leiden University Medical Center	Department of Medical Microbiology, Leiden University Medical Center	Dalebout; E.J.; J.C.; J.J. and Sidorov, I.; N.S.; Ogando; Snijder; T.J.; Zevenhoven; de Vries
EPI_ISL_591007, EPI_ISL_591014, EPI_ISL_635096, EPI_ISL_635100, EPI_ISL_635110 to 635111, EPI_ISL_635126, EPI_ISL_635135 to 635136, EPI_ISL_635165, EPI_ISL_668404, EPI_ISL_668417 to 668418, EPI_ISL_668420 to 668421, EPI_ISL_668441, EPI_ISL_708042 to 708043, EPI_ISL_708106 to 708109, EPI_ISL_708142 to 708146, EPI_ISL_759969 to 759972, EPI_ISL_775306, EPI_ISL_775464 to 775466, EPI_ISL_775475, EPI_ISL_775481, EPI_ISL_775491 to 775494, EPI_ISL_775505 to 775508, EPI_ISL_796687, EPI_ISL_796706, EPI_ISL_796711, EPI_ISL_796717 to 796721, EPI_ISL_796725, EPI_ISL_796727 to 796728, EPI_ISL_842643, EPI_ISL_860284 to 860288, EPI_ISL_964971, EPI_ISL_964990, EPI_ISL_1013455 to 1013456, EPI_ISL_1034309, EPI_ISL_1034346 to 1034361, EPI_ISL_1040156, EPI_ISL_1073918, EPI_ISL_1073923 to 1073924, EPI_ISL_1073941 to 1073942, EPI_ISL_1118291, EPI_ISL_1118306, EPI_ISL_1118313, EPI_ISL_1118347, EPI_ISL_1118349, EPI_ISL_1118354 to 1118355, EPI_ISL_1118408, EPI_ISL_1118460, EPI_ISL_1118462 to 1118464, EPI_ISL_1118468 to 1118469, EPI_ISL_1118476, EPI_ISL_1118497, EPI_ISL_1118500, EPI_ISL_1118509 to 1118510, EPI_ISL_1118514, EPI_ISL_1186128 to 1186132, EPI_ISL_1192188, EPI_ISL_1192204, EPI_ISL_1192206, EPI_ISL_1192227, EPI_ISL_1192254, EPI_ISL_1192299 to 1192304, EPI_ISL_1251440, EPI_ISL_1251447 to 1251448, EPI_ISL_1251765 to 1251768, EPI_ISL_1251957, EPI_ISL_1251966, EPI_ISL_1251968, EPI_ISL_1251970, EPI_ISL_1252019, EPI_ISL_1254535, EPI_ISL_1254619 to 1254620, EPI_ISL_1254622 to 1254623, EPI_ISL_1254625, EPI_ISL_1254629 to 1254632			
see above	Department of Medical Microbiology, St. Olavs hospital	Norwegian Institute of Public Health, Department of Virology	Atiya R Ali; Debech Nadia; Engebretsen Serina Beate; Engebretsen Serina Beate Atiya R Ali; Garcia Llorente Ignacio; Hilde Elshaug; Hilde Vollan; Ignacio Garcia Llorente; Jon Bråte; Kamilla Heddeland Instefjord; Karoline Bragstad; Kathrine Stene-Johansen; Marie Paulsen Madsen; Olav Hungnes; Pedersen Benedikte Nevjen; Rasmus Riis Kopperud; Serina B Engebretsen
EPI_ISL_417917	Department of Medical Microbiology, University Malaya Medical Centre	Department of Medical Microbiology	Adeeba KAMARULZAMAN; Chee Kuan WONG; Cindy Shuan Ju TEH; I-Ching SAM; Sasheela PONNAMPALAVANAR; Sharifah Faridah SYED OMAR; University Malaya Medical Centre COVID Team; Vijayan MUNUSAMY; Yoke Fun Chan; Yoong Min CHONG
EPI_ISL_417918 to 417920, EPI_ISL_501176 to 501228, EPI_ISL_506996 to 507000	Department of Medical Microbiology, University Malaya Medical Centre	Department of Medical Microbiology, Faculty of Medicine, University of Malaya	Adeeba KAMARULZAMAN; Chee Kuan WONG; Cindy Shuan Ju TEH; Fadhil Hadi JAMALUDDIN; Han Ming GAN; I-Ching SAM; Jennifer Chong; Sasheela PONNAMPALAVANAR; Sharifah Faridah SYED OMAR; University Malaya Medical Centre COVID Team; Vijayan MUNUSAMY; Yoke Fun CHAN; Yoke Fun Chan; Yoong Min CHONG
EPI_ISL_488840, EPI_ISL_488845, EPI_ISL_488847, EPI_ISL_488853 to 488856, EPI_ISL_488861, EPI_ISL_488863 to 488864, EPI_ISL_488872, EPI_ISL_492836, EPI_ISL_492838 to 492839, EPI_ISL_492841 to 492842, EPI_ISL_492848 to 492849, EPI_ISL_492853 to 492855, EPI_ISL_492857 to 492861, EPI_ISL_492866 to 492867, EPI_ISL_492870 to 492871, EPI_ISL_492873, EPI_ISL_492875, EPI_ISL_492878, EPI_ISL_492881, EPI_ISL_492884, EPI_ISL_492886, EPI_ISL_492889, EPI_ISL_492891 to 492893, EPI_ISL_492895 to 492900, EPI_ISL_492903 to 492904, EPI_ISL_492907, EPI_ISL_492909 to 492910, EPI_ISL_492912 to 492913, EPI_ISL_492915, EPI_ISL_495081 to 495096, EPI_ISL_501557, EPI_ISL_501562, EPI_ISL_501602, EPI_ISL_501613			
see above	Department of Medical Microbiology, Western Sussex Hospitals NHS Foundation Trust, St Richard's Hospital	Wellcome Sanger Institute for the COVID-19 Genomics UK (COG-UK) consortium	Cordelia Langford; David K. Jackson; Dominic Kwiatkowski; Ewan Harrison; Ian Johnston; John Sillitoe on behalf of the Wellcome Sanger Institute COVID-19 Surveillance Team (http://www.sanger.ac.uk/covid-team); Jonathan Lewis; Manasa Mutingwende; Michelle Erkiert; Olga Podplomyk; Paul Randell and Alex Alderton; Roberto Amato; Sarah Lowdon; Sonia Goncalves
EPI_ISL_896572 to 896601	Department of Medical Research	DMR	Myat Htut Nyunt
EPI_ISL_512844	Department of Medical Research	DMR_Myanmar	Aung Kyaw Kyaw; Aung Zaw Latt; Hlaing Myat Thu; Hnin Ohnmar Soe; Htin Lin; Kay Thi Aye; Lai Lai San; Myat Htut Nyunt; Nan Aye Thida Oo; Ni Ni Zaw; Phyu Win Ei; Su Mon Win; Theingi Win Myat; Wah Wah Aung; Yi Yi Kyaw; Zaw Than Htun
EPI_ISL_454733	Department of Medical, Biotechnologies University of Siena	Department of Medical, Biotechnologies University of Siena	Anichini, G.; Cusi; G. and Santoro, F.; Gandolfo, C.; M.G.; Pinzauti, D.; Pozzi
EPI_ISL_528809 to 528822	Department of Medicine, Gandhi hospital, Hyderabad	CSIR-Centre for Cellular and Molecular Biology	Ajay Sarawagi; Archana Bharadwaj Siva; Dhiviya Vedagiri; Divya Gupta; Divya Tej Sowpati; G. Aditya Kumar; Gangumala Srinivas Reddy; Karthik Bharadwaj Tallapaka; Koushick Sivakumar; Krishnan Harinivas Harshan; Lamuk Zaveri; M Soujanya Reddy; Namami Gaur; Nikhil Hajirnis; Onkar Kulkarni; Payel Mukherjee; Pooja Ramesh Gupta; Pratheusa Maccha; Priya Singh; Priyanka Pant; Purushotham Vodnala; Rajan Kumar Jha; Rajarao Mesipogu; Rajkanwar Nathawat; Rakesh K Mishra; Renu Sudhakar; Sakshi Shambhavi; Santosh Kumar Kuncha; Shagufta Khan; Shraddha Vijay Lahoti; Sofia Banu; Somesh Gorde; Sujoy Deb; Swati Bayyana; Thrilok Chander Bingi; Tulasi Nagabandi; Umesh Kumar; Unis Ahmad Bhat; Vinayasekhar Aedula; Vishal Sah
EPI_ISL_437438 to 437444, EPI_ISL_475030 to 475046, EPI_ISL_476869 to 476882, EPI_ISL_477183, EPI_ISL_495070 to 495080, EPI_ISL_500947 to 500950, EPI_ISL_512070 to 512071			
see above	Department of MicroBiology, Government Medical College, Surat	Gujarat Biotechnology Research Centre	A M Kadri; Afzal Ansari; Akanksha Verma; Amit Kanani; Amit gamit; Anjali Rajwar; Ankit Hinsu; Apurvasinh Puvar; Armi Chaudhari; Bhavesh Modi; Bhavya Jindal; Binita Aring; Chaitanya Joshi; Dinesh Kumar; Dipa Kinariwala; Dipeshwari Shewale; Disha Patel; Fenil Patel; Gaurishankar Shrimali; Geeta Vaghela; Harsh Bakshi; Janvi Raval; Kairavi Joshi; Kamlesh J Upadhyay; Komal Patel; Labdhi Pandya; Madhvi Joshi; Maharshi Pandya; Monika Gandhi; Naresh Chauhan; Neelam Nathani; Neeta Khandelwal; Neha Rajpara; Nidhi Patel; Nidhi Sood; Nikha Trivedi; Nitin Savaliya; Pinal Trivedi; Pooja P Doshi; Pragya Sharma; Pranay Shah; Pritesh Sabara; Priti Pandita; Priyanka P Vatsa; R D Dixit; Raghawendra Kumar; Ramesh Pandit; Sharmistha Majumdar; Snehal Bagatharia; Sonia Barve; Summaiya Mullan; Tejas Shah; Zarna Patel; Zuber Saiyed
EPI_ISL_431102	Department of MicroBiology,Gandhi Medical College and Hospital,Secendrabad,Hyderabad,India	Department of Microbiology, Gandhi Medical College and Hospital, Secendrabad, Hyderabad	Amit A. Upadhyay; Anand Kumar K; Kalyani Putty; Muttineni Radhakrishna; Nagamani K; Pankaj Singh D; Raja Rao M; Rama Amara; Ravikumar P; Steven E. Bosinger; Sunitha P; Thrilok Chander B
EPI_ISL_1273103	Department of Microbial Biotechnology, Genetic Engineering Division, National Research Centre	Department of Microbial Biotechnology, Genetic Engineering Division, National Research Centre	A.E. and Shemis, M.; A.G.; Dawood; Farghaly, A.; Fikry; Khalifa; M.K.; R.M.; Taha
EPI_ISL_602562 to 602563	Department of Microbiology and Immunology, Stony Brook University	Department of Microbiology and Immunology, Stony Brook University	A.M.; E.E.; E.R.; Gorbunova; Luperchio; Mackow; Nascimento Conde, J.
EPI_ISL_507039	Department of Microbiology, College of Medicine and Medical Research Institute Chungbuk National University	Department of Microbiology, College of Medicine and Medical Research Institute Chungbuk National University	Eun-Ha Kim; Hye Won Jeong; Mark Anthony B. Casel; Se-Mi Kim; Seong-Gyu Kim; Su-Jin Park; Young Ki Choi; Young-Il Kim
EPI_ISL_1069194	Department of Microbiology, College of Medicine and Medical Research Institute Chungbuk National University	Department of Microbiology, College of Medicine and Medical Research Institute Chungbuk National University	Bum Sik Chin; Eun Ji Kim; Eun-Ha Kim; Haryoung Poo; Hye Won Jeong; In-Pil Mo; Jae U. Jung; Jae-Hyung Chang; Jihye Um; Jun-Sun Park; Ki-Hyun Chung; Kwang-Min Yu; Mark Anthony B. Casel; Min-Suk Song; Ok-Jun Lee; Richard J. Webby; Se-Mi Kim; Seong-Gyu Kim; Seunghun Lee; Su-Jin Park; Suan-Sin Foo; Van Dam Lai; Yeonjae Kim; Young Ki Choi; Young-Il Kim
EPI_ISL_416314 to 416315	Department of Microbiology, Faculty of Medicine, The Chinese University of Hong Kong, Hong Kong SAR, China	Department of Microbiology, Faculty of Medicine, Chinese University of Hong Kong, Hong Kong SAR, China	Paul KS Chan; Zigui Chen
EPI_ISL_1072965, EPI_ISL_1072986	Department of Microbiology, Faculty of Medicine, Universitas Sumatera Utara	Institute of Tropical Disease, Universitas Airlangga; Faculty of Medicine, Universitas Sumatera Utara	Aldise M Nastri; Franciscus Ginting; Inke N D Lubis; Irbah R Nainggolan; Jezzy R Dewantari; Kazufumi Shimizu; Krisnoadi Rahardjo; Maria I Lusida; Meliani; Mirzan Hasibuan; Muhammad Ichwan; R Andika D Cahyadi; R Lia Kusumawati; Ramadhan Bestari; Rima R Prasetya; Soetjipto; Yasuko Mori
EPI_ISL_431103, EPI_ISL_431117	Department of Microbiology, Gandhi Medical College and Hospital, Secendrabad, Hyderabad, India	Department of Microbiology, Gandhi Medical College and Hospital, Secendrabad, Hyderabad, India	Amit A. Upadhyay; Anand Kumar K; Kalyani Putty; Muttineni Radhakrishna; Nagamani K; Pankaj Singh D; Raja Rao M; Rama Amara; Ravikumar P; Steven E. Bosinger; Steven E.Bosinger; Sunitha P; Thrilok Chander B
EPI_ISL_483850 to 483879	Department of Microbiology, Government Medical College, Surat	Gujarat Biotechnology Research Centre	A M Kadri; Afzal Ansari; Amit gamit; Apurvasinh Puvar; Chaitanya Joshi; Dinesh Kumar; Harsh Bakshi; Janvi Raval; Komal Patel; Labdhi Pandya; Madhvi Joshi; Maharshi Pandya; Monika Gandhi; Naresh Chauhan; Nidhi Patel; Nikha Trivedi; Nitin Savaliya; Pinal Trivedi; R D Dixit; Raghawendra Kumar; Summaiya Mullan; Zarna Patel; Zuber Saiyed
EPI_ISL_1171857	Department of Microbiology, Icahn School of Medicine at Mount	MSHS Pathogen Surveillance Program	Aaron J. Schmitz; Ali H. Ellebedy; Ana Gonzalez Reiche; Daniel C. Adelsberg; Emilia Mia Sordillo; Fatima Amanat; Florian Krammer; Goran Bajic; Hala

	Sinai		Alshammary; Harm van Bakel; Jackson S. Turner; Juan Manuel Carreno; Julian Q Zhou; Komal Srivastava; Mahima Thapa; Nicholas Borcherding; Sarah Zafar; Shaza M. Sayed Ahmed; Shirin Strohmeier; The Personalized Virology Initiative; Tinting Lei; Viviana Simon; Willemijn Rijnink
EPI_ISL_413017 to 413018, EPI_ISL_413514, EPI_ISL_413516	Department of Microbiology, Institute for Viral Diseases, College of Medicine, Korea University	Department of Microbiology, Institute for Viral Diseases, College of Medicine, Korea University	Changmin Kang; Cui Chunguang; Dong Min Kim; Gee Eun Lee; Gee eun Lee; Hee Jin Cheong; Heedo Park; Jeonghun Kim; Ji Yun Noh; Jin Gu Yoon; Jin Il Kim; Joon Young Song; Joon-Yong Bae; Jungmin Lee; Juyoung Cho; Kyeong-ryeol Shin; Man-Seong Park; Woo Joo Kim
EPI_ISL_862049	Department of Microbiology, National Influenza Reference Laboratory for Northern Greece	Department of Microbiology, National Influenza Reference Laboratory for Northern Greece	Anna Papa; Styliani Pappa
EPI_ISL_1027637 to 1027657	Department of Microbiology, National Institute for Public Health of Kosova	Charité Universitätsmedizin Berlin, Institut für Virologie	Barbara Mühlemann; Christian Drosten; Donjeta Hajdari; Julia Schneider; Jörn Beheim-Schwarzbach; Talitha Veith; Terry Jones; Victor M Corman; Xhevat Jakupi; Zana Deva
EPI_ISL_420531 to 420534, EPI_ISL_420536 to 420539	Department of Microbiology, PathWest QEII Medical Centre	Department of Microbiology, PathWest QEII Medical Centre	Avram Levy; Chisha Sikazwe; David Speers and David Smith; Jurissa Lang
EPI_ISL_434560, EPI_ISL_497768 to 497870, EPI_ISL_498269 to 498271, EPI_ISL_510538 to 510540, EPI_ISL_516798 to 516799, EPI_ISL_538524 to 538551, EPI_ISL_1034368 to 1034735, EPI_ISL_1055452 to 1055455, EPI_ISL_1197067 to 1197128, EPI_ISL_1205464 to 1205467, EPI_ISL_1273444			
see above	Department of Microbiology, The University of Hong Kong	Department of Microbiology, The University of Hong Kong	A.C.P.; Chan; Fung; Fung, J.; H.K.H.; He, Z.; K.S.C. and Woo; K.S.M.; Kelvin K.W. To; Kwok-Yung Yuen; Lau; Li; Luk; P.C.Y.; S.K.P.; T.T.Y.; Wong; Zhu, L.
EPI_ISL_853718 to 853720, EPI_ISL_853722, EPI_ISL_853724 to 853728, EPI_ISL_853732, EPI_ISL_853739 to 853740, EPI_ISL_853742 to 853745, EPI_ISL_853747 to 853748, EPI_ISL_853750, EPI_ISL_853755 to 853756, EPI_ISL_853760 to 853761, EPI_ISL_853763, EPI_ISL_853765, EPI_ISL_853767, EPI_ISL_853783, EPI_ISL_853785 to 853786, EPI_ISL_853788, EPI_ISL_853792, EPI_ISL_853796, EPI_ISL_853802, EPI_ISL_853806, EPI_ISL_853814, EPI_ISL_853929 to 853956, EPI_ISL_854220 to 854222, EPI_ISL_854224 to 854226, EPI_ISL_854230, EPI_ISL_854232, EPI_ISL_854234, EPI_ISL_854236, EPI_ISL_854238, EPI_ISL_854240, EPI_ISL_934633 to 934649, EPI_ISL_1008280 to 1008291, EPI_ISL_1008321, EPI_ISL_1117775, EPI_ISL_1117782 to 1117784, EPI_ISL_1117787, EPI_ISL_1117790, EPI_ISL_1117807, EPI_ISL_1117809, EPI_ISL_1117888, EPI_ISL_1117896, EPI_ISL_1117913, EPI_ISL_1118083 to 1118085, EPI_ISL_1180886 to 1180894, EPI_ISL_1180896 to 1180899, EPI_ISL_1180901 to 1180903, EPI_ISL_1180905 to 1180906, EPI_ISL_1180908 to 1180909			
see above	Department of Microbiology, University Innsbruck	Bergthaler laboratory, CeMM Research Center for Molecular Medicine of the Austrian Academy of Sciences	Alexander Lercher; Alexandra Popa; Andreas Bergthaler; Anna Schedl; Bekir Erguner; Benedikt Agerer; Christoph Bock; Christoph Bock; Fabian Amman; Jakob-Wendelin Genger; Jan Laine; Lukas Endler; Maelle Le Moing; Martin Senekowitsch; Martin Senekowitsch; Michael Schuster; Michael Schuster; Thomas Penz
EPI_ISL_693298 to 693299	Department of Microbiology, Yokohama City University School of Medicine	Department of Microbiology, Yokohama City University School of Medicine	Akihide Ryo; Ichiro Takeuchi; Kazuya Sakai; Kei Miyakawa; Mototsugu Nishii; Reo Matsumura; Ryo Saji
EPI_ISL_438138	Department of Microbiology,Gandhi Medical College and Hospital	Department of Microbiology, Gandhi Medical College and Hospital Secendrabad, Hyderbad, India	Amit A. Upadhyay; Anand Kumar K; Kalyani Putty; Muttineni Radhakrishna; Nagamani K; Pankaj Singh D; Raja Rao Mesipogu; Rama Amara; Ravikumar P; Steven Bosinger; Sunitha P; Thrilok Chander B
EPI_ISL_437626	Department of Microbiology,Gandhi Medical College and Hospital	Department of Veterinary Biotechnalogy, College of Veterinary Science, Rajendranagar, PV Narsimha Rao Telengana Veterinary University	Amit A. Upadhyay; Anand Kumar K; Kalyani Putty; Muttineni Radhakrishna; Nagamani K; Pankaj Singh D; Raja Rao M; Rama Amara; Ravikumar P; Steven Bosinger; Sunitha P; Thrilok Chander B
EPI_ISL_431101	Department of Microbiology,Gandhi Medical College and Hospital	Virus Research Laboratory, Department of Zoology, Osmania University,Hyderabad,India	Amit A. Upadhyay Steven E. Bosinger; Anand Kumar K; Kalyani Putty; Muttineni Radhakrishna; Nagamani K; Pankaj Singh D; Raja Rao M; Rama Amara; Ravikumar P; Sunitha P; Thrilok Chander B
EPI_ISL_438139	Department of Microbiology,Gandhi Medical College and Hospital,Hyderabad	Virus Research Laboratory, Department of Zoology, Osmania University, Hyderabad, India	Amit A. Upadhyay; Anand Kumar K; Kalyani Putty; Muttineni Radhakrishna; Nagamani K; Pankaj Singh D; Raja Rao M; Rama Amara; Ravikumar P; Steven Bosinger; Sunitha P; Thrilok Chander B
EPI_ISL_434561 to 434562	Department of Microbiology; Ryota Kumagai Tokyo Metropolitan Institute of Public Health	Department of Microbiology; Ryota Kumagai Tokyo Metropolitan Institute of Public Health	Asakura, H.; Chiba; Kumagai, R.; Nagashima, M.; T. and Sadamasu, K.; Yoshida, I.
EPI_ISL_414511	Department of Microbiology; Ryota Kumagai Tokyo Metropolitan Institute of Public Health	Tokyo Metoropolitan Institute of Public Health	Chiba; Kumagai, R.; Nagashima, M.; T. and Sadamasu, K.; Yoshida, I.
EPI_ISL_812968	Department of Molecular Medicine, University of Padova	Department of Molecular Medicine, University of Padova	Abate, D.; Barzon, L.; Besutti, V.; De Canale, E.; Del Vecchio, C.; Franchin, E.; Lavezzo, E.; Loregian, A.; M.C.; Manganelli, R.; Manuto, L.; Masi, G.; Onelia, F.; Pacenti, M.; Parisi; Rossi, L.; S. and Crisanti, A.; S.G.; Saluzzo, F.; Sciro, M.; Toppo; Trevisan, M.; Vanuzzo
EPI_ISL_463741 to 463748, EPI_ISL_1164626 to 1164654, EPI_ISL_1164656 to 1164751	Department of Molecular Virology, Cyprus Institute of Neurology and Genetics	Department of Molecular Virology, Cyprus Institute of Neurology and Genetics	Anastasis Oulas; Andreas Hadjisavvas; Christina Christodoulou; Christina Tryfonos; Dana Koptides; Denise Alexandrou; George Krashias; George Spyrou; Jan Richter; Maria Loizidou; Mihalis Panayiotidis; Olga Kalakouta; Pavlos Fanis; Stavros Bashiardes
EPI_ISL_853819 to 853821, EPI_ISL_854239	Department of Pathology, Landeskrankenhaus Graz II, Medical University Graz	Bergthaler laboratory, CeMM Research Center for Molecular Medicine of the Austrian Academy of Sciences	Alexander Lercher; Alexandra Popa; Andreas Bergthaler; Anna Schedl; Benedikt Agerer; Christoph Bock; Jakob-Wendelin Genger; Jan Laine; Lukas Endler; Martin Senekowitsch; Michael Schuster; Thomas Penz
EPI_ISL_417176 to 417179, EPI_ISL_419217, EPI_ISL_419222 to 419223	Department of Pathology, Princess Margaret Hospital	Department of Health Technology and Informatics, Faculty of Health and Social Science, The Hong Kong Polytechnic University	Alan Ka-Lun WU; Alex Yat-Man HO; Barry Kin-Chung WONG; David Ho-Keung SHUM; Eugene Yuk-Keung TSO; Gilman Kit-Hang SIU; Hiu-Yin LAO; Kam-Tong YIP; Kam-Tong Yip; Kenneth Siu-Sing LEUNG; Kingsley King-Gee TAM; Kit-Man SIN; Kitty Sau-Chun FUNG; Kwok-Cheung LUNG; Lam-Kwong LEE; Man-Chun CHAN; Ming-Pan CHOI; Miranda Chong-Yee YAU; Raymond Wai-To LIU; Sandy Ka-Yee CHAU; Shea Ping YIP; Tak-Lun QUE; Tak-Lun Que; Timothy Ting-Leung NG; Wai-Shing LEUNG; Wing Cheong YAM; Wing-Kin TO; Yuk-Yung NG
EPI_ISL_594185 to 594188, EPI_ISL_596451 to 596452, EPI_ISL_596455	Department of Pathology, School of Medicine, Imam Khomeini Hospital, Tehran University of Medical Sciences	Genetics Research Center, University of Social Welfare and Rehabilitation Sciences	Ali Jafarpour; Alireza Abdollahi; Azam Ghaziasad; Azam Ghaziasadi; Azar Hadadi; Hossein Najmabadi; Khadijeh Jalalvand; Kimia Kahrizi; Marzieh Mohseni; Reza Najafipour; Saber Soltani; Seyed Mohammad Jazayeri; Seyedeh elham Mortazavi; Zohreh Fattahi
EPI_ISL_582033	Department of Pathology, School of Medicine, Imam Khomeini Hospital, Tehran University of Medical Sciences	Genetics Research Center. University Of Social Welfare And Rehabilitation Sciences	Ali Jafarpour; Alireza Abdollahi; Azam Ghaziasad; Azam Ghaziasadi; Azar Hadadi; Hossein Najmabadi; Khadijeh Jalalvand; Kimia Kahrizi; Marzieh Mohseni; Reza Najafipour; Saber Soltani; Seyed Mohammad Jazayeri; Seyedeh elham Mortazavi; Zohreh Fattahi
EPI_ISL_413459	Department of Pathology, Toshima Hospital	Pathogen Genomics Center, National Institute of Infectious Diseases	Haruka Nishioka; Harutaka Katano; Ippei Miyamoto; Ja-Mun Chong; Jun Yamazaki; Kentaro Itokawa; Makoto Kuroda; Masahiro Sano; Minoru Tobiume; Noriko Nakajima; Tadaki Suzuki; Takuya Adachi; Tsuyoshi Sekizuka; Yoko Sato
EPI_ISL_417180 to 417182, EPI_ISL_417185, EPI_ISL_419221, EPI_ISL_419253	Department of Pathology, United Christian Hospital	Department of Health Technology and Informatics, Faculty of Health and Social Science, The Hong Kong Polytechnic University	Alan Ka-Lun WU; Alex Yat-Man HO; Barry Kin-Chung WONG; David Ho-Keung SHUM; Eugene Yuk-Keung TSO; Gilman Kit-Hang SIU; Hiu-Yin LAO; Kam-Tong YIP; Kam-Tong Yip; Kenneth Siu-Sing LEUNG; Kingsley King-Gee TAM; Kit-Man SIN; Kitty Sau-Chun FUNG; Kwok-Cheung LUNG; Lam-Kwong LEE; Man-Chun CHAN; Ming-Pan CHOI; Miranda Chong-Yee YAU; Raymond Wai-To LIU; Sandy Ka-Yee CHAU; Shea Ping YIP; Tak-Lun QUE; Tak-Lun Que; Timothy Ting-Leung NG; Wai-Shing LEUNG; Wing Cheong YAM; Wing-Kin TO; Yuk-Yung NG
EPI_ISL_425231 to 425371, EPI_ISL_425373 to 425461, EPI_ISL_433466 to 433497, EPI_ISL_433666 to 434062, EPI_ISL_438550 to 438748, EPI_ISL_444313 to 444453, EPI_ISL_447945 to 448115, EPI_ISL_452857 to 453005, EPI_ISL_456677 to 456755, EPI_ISL_461546 to 461588, EPI_ISL_473451 to 473507, EPI_ISL_477783 to 477819, EPI_ISL_483913 to 483915, EPI_ISL_490317 to 490329, EPI_ISL_499853, EPI_ISL_499855, EPI_ISL_499905, EPI_ISL_499908, EPI_ISL_499966, EPI_ISL_499968 to 499973, EPI_ISL_512330 to 512335, EPI_ISL_514443 to 514451, EPI_ISL_516990 to 517000, EPI_ISL_529224 to 529225, EPI_ISL_529236, EPI_ISL_529257, EPI_ISL_529261, EPI_ISL_529265, EPI_ISL_529269, EPI_ISL_529283, EPI_ISL_529299, EPI_ISL_529445, EPI_ISL_529524, EPI_ISL_529656 to 529660, EPI_ISL_549374, EPI_ISL_549386, EPI_ISL_549388 to 549395, EPI_ISL_572471 to 572472, EPI_ISL_572484, EPI_ISL_572519, EPI_ISL_572521, EPI_ISL_572524, EPI_ISL_572541, EPI_ISL_572592, EPI_ISL_572616, EPI_ISL_572642, EPI_ISL_572699, EPI_ISL_572779, EPI_ISL_572792, EPI_ISL_572851 to 572852, EPI_ISL_572862 to 572868, EPI_ISL_573132 to 573151, EPI_ISL_573153, EPI_ISL_576897 to 576919, EPI_ISL_584283 to 584323, EPI_ISL_611593, EPI_ISL_611751, EPI_ISL_611937 to 611938, EPI_ISL_611964 to 611967, EPI_ISL_612007, EPI_ISL_612034, EPI_ISL_612099, EPI_ISL_612106, EPI_ISL_612123, EPI_ISL_612186, EPI_ISL_612261 to 612283, EPI_ISL_637260 to 637261, EPI_ISL_637264, EPI_ISL_637267 to 637272, EPI_ISL_637275 to 637277, EPI_ISL_637281 to 637282, EPI_ISL_637287, EPI_ISL_637292, EPI_ISL_637294, EPI_ISL_637299, EPI_ISL_637304, EPI_ISL_637307 to 637310, EPI_ISL_637314 to 637318, EPI_ISL_637321, EPI_ISL_637324, EPI_ISL_637328, EPI_ISL_637331, EPI_ISL_637334 to 637335, EPI_ISL_637340 to 637341, EPI_ISL_637345, EPI_ISL_637348, EPI_ISL_637353 to 637357, EPI_ISL_637360, EPI_ISL_637362 to 637368, EPI_ISL_637384, EPI_ISL_637386, EPI_ISL_637388 to 637389, EPI_ISL_637391 to 637394, EPI_ISL_637396 to 637399, EPI_ISL_637403 to 637408, EPI_ISL_637410, EPI_ISL_637413 to 637418, EPI_ISL_637420 to 637421, EPI_ISL_637423 to 637426, EPI_ISL_637428 to 637431, EPI_ISL_637433, EPI_ISL_637435, EPI_ISL_637440 to 637441, EPI_ISL_637451, EPI_ISL_637456 to 637457, EPI_ISL_637460 to 637462, EPI_ISL_637464 to 637465, EPI_ISL_637468 to 637490, EPI_ISL_637500 to 637501, EPI_ISL_637503 to 637519, EPI_ISL_637526 to 637527, EPI_ISL_637529 to 637530, EPI_ISL_637532, EPI_ISL_637534, EPI_ISL_637545, EPI_ISL_637547 to 637549, EPI_ISL_637552, EPI_ISL_637554, EPI_ISL_637560, EPI_ISL_637562 to 637579, EPI_ISL_637583, EPI_ISL_637590 to 637592, EPI_ISL_637595, EPI_ISL_637598, EPI_ISL_637613, EPI_ISL_637620, EPI_ISL_637623 to 637624, EPI_ISL_637628 to 637658, EPI_ISL_637662 to 637666, EPI_ISL_637668 to 637673, EPI_ISL_637676 to 637713, EPI_ISL_637716, EPI_ISL_637721 to 637779, EPI_ISL_637801 to 637852, EPI_ISL_637856, EPI_ISL_637859 to 637860, EPI_ISL_637873, EPI_ISL_637875, EPI_ISL_637877, EPI_ISL_637880, EPI_ISL_637888, EPI_ISL_637890, EPI_ISL_637903, EPI_ISL_637906, EPI_ISL_637913, EPI_ISL_637916, EPI_ISL_637936, EPI_ISL_637938 to 637940, EPI_ISL_637942, EPI_ISL_637947, EPI_ISL_637953, EPI_ISL_637955, EPI_ISL_637963, EPI_ISL_637966 to 637967, EPI_ISL_637974 to 637975, EPI_ISL_637977 to 637978, EPI_ISL_637980, EPI_ISL_637982 to 637983, EPI_ISL_637991, EPI_ISL_638000 to 638001, EPI_ISL_638008, EPI_ISL_638013 to 638014, EPI_ISL_638016, EPI_ISL_638018 to 638020, EPI_ISL_638027, EPI_ISL_638029 to 638030, EPI_ISL_638036, EPI_ISL_638038 to 638040, EPI_ISL_638066 to 638072, EPI_ISL_638079 to 638083, EPI_ISL_638131 to 638133, EPI_ISL_638138 to 638143, EPI_ISL_638146, EPI_ISL_638165 to 638167, EPI_ISL_638169, EPI_ISL_638174, EPI_ISL_638177 to 638205, EPI_ISL_638208 to 638248, EPI_ISL_638258 to 638264, EPI_ISL_638293 to 638448, EPI_ISL_638614 to 638681, EPI_ISL_638731 to 638735, EPI_ISL_638895 to 638910, EPI_ISL_638914 to 638927, EPI_ISL_638936 to 638943, EPI_ISL_639003 to 639005, EPI_ISL_639624 to 639625, EPI_ISL_650122, EPI_ISL_650127, EPI_ISL_650129 to 650130, EPI_ISL_650133, EPI_ISL_650143, EPI_ISL_650149, EPI_ISL_650200, EPI_ISL_650217, EPI_ISL_650228, EPI_ISL_650232, EPI_ISL_650236, EPI_ISL_650239 to 650240, EPI_ISL_650243, EPI_ISL_650262, EPI_ISL_650265, EPI_ISL_650273, EPI_ISL_650353, EPI_ISL_650356, EPI_ISL_650364 to 650365, EPI_ISL_650376, EPI_ISL_650383, EPI_ISL_650389 to 650392, EPI_ISL_650396 to 650398, EPI_ISL_650400, EPI_ISL_650447, EPI_ISL_650481, EPI_ISL_650553, EPI_ISL_650562, EPI_ISL_650564, EPI_ISL_650573 to 650574, EPI_ISL_650580, EPI_ISL_650583, EPI_ISL_650589 to 650596, EPI_ISL_650609, EPI_ISL_650612, EPI_ISL_650614 to 650617, EPI_ISL_650626 to 650633, EPI_ISL_650661, EPI_ISL_650664, EPI_ISL_650666, EPI_ISL_650669, EPI_ISL_650682, EPI_ISL_650697 to 650700, EPI_ISL_650707 to 650708, EPI_ISL_650768, EPI_ISL_650774, EPI_ISL_650836, EPI_ISL_650899, EPI_ISL_650910, EPI_ISL_650916, EPI_ISL_650922, EPI_ISL_650951 to 650952, EPI_ISL_650959 to 650962, EPI_ISL_650969 to 650973, EPI_ISL_650978, EPI_ISL_650983 to 650986, EPI_ISL_650988 to 650994, EPI_ISL_651001 to 651010, EPI_ISL_651014 to 651047, EPI_ISL_651049 to 651052, EPI_ISL_651080 to 651081, EPI_ISL_651122, EPI_ISL_651136, EPI_ISL_651172, EPI_ISL_651176, EPI_ISL_651186, EPI_ISL_651202, EPI_ISL_651235, EPI_ISL_651269, EPI_ISL_651276, EPI_ISL_651278, EPI_ISL_651280 to 651281, EPI_ISL_651283 to 651284, EPI_ISL_651292, EPI_ISL_651302, EPI_ISL_651331, EPI_ISL_651339, EPI_ISL_651346, EPI_ISL_651348, EPI_ISL_651360 to 651363, EPI_ISL_651365, EPI_ISL_651370, EPI_ISL_651382 to 651383, EPI_ISL_651423, EPI_ISL_651435 to 651436, EPI_ISL_651487 to 651492, EPI_ISL_651542, EPI_ISL_651550 to 651582, EPI_ISL_651653 to 651714, EPI_ISL_652157 to 652179, EPI_ISL_652286 to 652288, EPI_ISL_664108 to 664109, EPI_ISL_664112, EPI_ISL_664118 to 664120, EPI_ISL_664126, EPI_ISL_664130 to			

664133, EPI_ISL_664139 to 664141, EPI_ISL_664143, EPI_ISL_664149 to 664150, EPI_ISL_664152, EPI_ISL_664155 to 664157, EPI_ISL_664160 to 664161, EPI_ISL_664165 to 664168, EPI_ISL_664172, EPI_ISL_664174 to 664182, EPI_ISL_664185, EPI_ISL_664198 to 664201, EPI_ISL_664213, EPI_ISL_664217, EPI_ISL_664221, EPI_ISL_664234, EPI_ISL_664237, EPI_ISL_664249, EPI_ISL_664277, EPI_ISL_664280, EPI_ISL_664282, EPI_ISL_664286, EPI_ISL_664291, EPI_ISL_664303 to 664317, EPI_ISL_664320 to 664326, EPI_ISL_664346, EPI_ISL_664378 to 664384, EPI_ISL_664397, EPI_ISL_664421 to 664422, EPI_ISL_664428, EPI_ISL_664432 to 664434, EPI_ISL_664446, EPI_ISL_664451, EPI_ISL_664453 to 664455, EPI_ISL_664470, EPI_ISL_664514, EPI_ISL_664516, EPI_ISL_664518, EPI_ISL_664527 to 664538, EPI_ISL_664542 to 664543, EPI_ISL_664546 to 664555, EPI_ISL_664567 to 664591, EPI_ISL_664598 to 664599, EPI_ISL_664601, EPI_ISL_664610, EPI_ISL_664616, EPI_ISL_664622, EPI_ISL_664625, EPI_ISL_664627, EPI_ISL_664631, EPI_ISL_664635, EPI_ISL_664647, EPI_ISL_664655 to 664656, EPI_ISL_664658 to 664660, EPI_ISL_664662, EPI_ISL_664666, EPI_ISL_664676 to 664677, EPI_ISL_664680, EPI_ISL_664684, EPI_ISL_664697 to 664698, EPI_ISL_664708, EPI_ISL_664717, EPI_ISL_664719, EPI_ISL_664721, EPI_ISL_664731, EPI_ISL_664739, EPI_ISL_664748 to 664751, EPI_ISL_664762, EPI_ISL_664765 to 664766, EPI_ISL_664768 to 664837, EPI_ISL_664941 to 665022, EPI_ISL_678832 to 678915, EPI_ISL_679134 to 679248, EPI_ISL_680037 to 680100, EPI_ISL_680571 to 680577, EPI_ISL_681260, EPI_ISL_702474, EPI_ISL_702489, EPI_ISL_702500, EPI_ISL_702561, EPI_ISL_702703, EPI_ISL_702723, EPI_ISL_702734, EPI_ISL_702763, EPI_ISL_702782, EPI_ISL_702787, EPI_ISL_702834, EPI_ISL_702847 to 702848, EPI_ISL_702860, EPI_ISL_702876, EPI_ISL_702883, EPI_ISL_702886, EPI_ISL_702891, EPI_ISL_702971, EPI_ISL_702977, EPI_ISL_703032, EPI_ISL_703076, EPI_ISL_703093, EPI_ISL_703115, EPI_ISL_703118, EPI_ISL_703127, EPI_ISL_703213, EPI_ISL_703238, EPI_ISL_703406, EPI_ISL_703432, EPI_ISL_703435, EPI_ISL_703438, EPI_ISL_703440, EPI_ISL_703451, EPI_ISL_703457, EPI_ISL_703459, EPI_ISL_703462, EPI_ISL_703465, EPI_ISL_703468, EPI_ISL_703471, EPI_ISL_703473, EPI_ISL_703476, EPI_ISL_703479, EPI_ISL_703508, EPI_ISL_703511, EPI_ISL_703513, EPI_ISL_703516, EPI_ISL_703519, EPI_ISL_703521, EPI_ISL_703524, EPI_ISL_703619, EPI_ISL_703629, EPI_ISL_703632, EPI_ISL_703635, EPI_ISL_703679, EPI_ISL_703693, EPI_ISL_703708 to 703710, EPI_ISL_703713, EPI_ISL_703715 to 703716, EPI_ISL_703724, EPI_ISL_703741 to 703742, EPI_ISL_703747, EPI_ISL_704060, EPI_ISL_704076, EPI_ISL_704115, EPI_ISL_704203, EPI_ISL_704219, EPI_ISL_704222, EPI_ISL_704225, EPI_ISL_704247, EPI_ISL_704250, EPI_ISL_704252, EPI_ISL_704255, EPI_ISL_704257, EPI_ISL_704269, EPI_ISL_704271, EPI_ISL_704273, EPI_ISL_704276, EPI_ISL_704280, EPI_ISL_704283, EPI_ISL_704286, EPI_ISL_704289, EPI_ISL_704291, EPI_ISL_704294, EPI_ISL_704297, EPI_ISL_704299, EPI_ISL_704302, EPI_ISL_704305, EPI_ISL_704307, EPI_ISL_704309, EPI_ISL_704328, EPI_ISL_704331, EPI_ISL_704333, EPI_ISL_704336, EPI_ISL_704339, EPI_ISL_704341, EPI_ISL_704344, EPI_ISL_704347, EPI_ISL_704350, EPI_ISL_704353, EPI_ISL_704356, EPI_ISL_704359, EPI_ISL_704361, EPI_ISL_704364, EPI_ISL_704366, EPI_ISL_704369, EPI_ISL_704374, EPI_ISL_704377, EPI_ISL_704379, EPI_ISL_704382, EPI_ISL_704384, EPI_ISL_704389, EPI_ISL_704392, EPI_ISL_704395, EPI_ISL_704398, EPI_ISL_704401, EPI_ISL_704403, EPI_ISL_704406, EPI_ISL_704408, EPI_ISL_704411, EPI_ISL_704413, EPI_ISL_704416, EPI_ISL_704419, EPI_ISL_704427, EPI_ISL_704430, EPI_ISL_704433, EPI_ISL_704435, EPI_ISL_704438, EPI_ISL_704441, EPI_ISL_704443, EPI_ISL_704446, EPI_ISL_704449, EPI_ISL_704452, EPI_ISL_704454, EPI_ISL_704457, EPI_ISL_704460, EPI_ISL_704462, EPI_ISL_704465, EPI_ISL_704467, EPI_ISL_704470, EPI_ISL_704473, EPI_ISL_704475, EPI_ISL_704478, EPI_ISL_704481, EPI_ISL_704484, EPI_ISL_704486, EPI_ISL_704489, EPI_ISL_704492, EPI_ISL_704494, EPI_ISL_704497, EPI_ISL_704499, EPI_ISL_704502, EPI_ISL_704505, EPI_ISL_704507, EPI_ISL_704510, EPI_ISL_704513, EPI_ISL_704516, EPI_ISL_704518, EPI_ISL_704521, EPI_ISL_704524, EPI_ISL_704526, EPI_ISL_704529, EPI_ISL_704532, EPI_ISL_704535, EPI_ISL_704537, EPI_ISL_704540, EPI_ISL_704543, EPI_ISL_704545, EPI_ISL_704548, EPI_ISL_704551, EPI_ISL_704554, EPI_ISL_704557, EPI_ISL_704606, EPI_ISL_704610 to 704618, EPI_ISL_704640 to 704641, EPI_ISL_704643, EPI_ISL_704646, EPI_ISL_704648, EPI_ISL_704651, EPI_ISL_704654, EPI_ISL_704656, EPI_ISL_704659, EPI_ISL_704662, EPI_ISL_704664, EPI_ISL_704834, EPI_ISL_704957, EPI_ISL_705081, EPI_ISL_705110, EPI_ISL_705140, EPI_ISL_705217, EPI_ISL_705257, EPI_ISL_705289 to 705290, EPI_ISL_705333, EPI_ISL_705339, EPI_ISL_705346, EPI_ISL_705365, EPI_ISL_705376, EPI_ISL_705382, EPI_ISL_705410, EPI_ISL_705415, EPI_ISL_705501 to 705524, EPI_ISL_705529 to 705566, EPI_ISL_705629 to 705635, EPI_ISL_723851 to 723948, EPI_ISL_740953 to 741017, EPI_ISL_763366, EPI_ISL_763370, EPI_ISL_763375, EPI_ISL_763379 to 763380, EPI_ISL_763426, EPI_ISL_763438, EPI_ISL_763448, EPI_ISL_763464, EPI_ISL_763469, EPI_ISL_763508, EPI_ISL_763518, EPI_ISL_763526, EPI_ISL_763542, EPI_ISL_763569, EPI_ISL_763592 to 763593, EPI_ISL_763637 to 763638, EPI_ISL_763641, EPI_ISL_763648, EPI_ISL_763666, EPI_ISL_763688, EPI_ISL_763702, EPI_ISL_763790, EPI_ISL_763792 to 763796, EPI_ISL_763801 to 763802, EPI_ISL_763815 to 763816, EPI_ISL_763839, EPI_ISL_763843, EPI_ISL_763847 to 763850, EPI_ISL_763872 to 763891, EPI_ISL_764141, EPI_ISL_764151, EPI_ISL_764159, EPI_ISL_764162, EPI_ISL_764164, EPI_ISL_764171, EPI_ISL_764174, EPI_ISL_764176 to 764177, EPI_ISL_764191, EPI_ISL_764202, EPI_ISL_764204, EPI_ISL_764208 to 764209, EPI_ISL_764236, EPI_ISL_764272, EPI_ISL_764276, EPI_ISL_764280 to 764281, EPI_ISL_764284 to 764355, EPI_ISL_813154 to 813355, EPI_ISL_837837 to 837903, EPI_ISL_837905 to 837980, EPI_ISL_837982 to 838054, EPI_ISL_842110, EPI_ISL_864738 to 864756, EPI_ISL_864758, EPI_ISL_864760, EPI_ISL_864762, EPI_ISL_864764, EPI_ISL_864766, EPI_ISL_864768, EPI_ISL_864771, EPI_ISL_864773 to 864784, EPI_ISL_864786 to 864789, EPI_ISL_864791 to 864794, EPI_ISL_864796 to 864800, EPI_ISL_864802 to 864811, EPI_ISL_864813 to 864815, EPI_ISL_864818 to 864819, EPI_ISL_864821, EPI_ISL_864828 to 864829, EPI_ISL_864832 to 864835, EPI_ISL_864837 to 864847, EPI_ISL_864849 to 864852, EPI_ISL_864854, EPI_ISL_864856 to 864857, EPI_ISL_864859, EPI_ISL_864861 to 864876, EPI_ISL_864878, EPI_ISL_864880, EPI_ISL_864882 to 864884, EPI_ISL_864886, EPI_ISL_864888 to 864890, EPI_ISL_864892 to 864894, EPI_ISL_864896 to 864916, EPI_ISL_864919 to 864924, EPI_ISL_864926 to 864946, EPI_ISL_864949 to 864959, EPI_ISL_918985 to 918996, EPI_ISL_918998 to 919004, EPI_ISL_919006 to 919017, EPI_ISL_919029, EPI_ISL_919037, EPI_ISL_919039, EPI_ISL_919041, EPI_ISL_919043 to 919052, EPI_ISL_919056 to 919057, EPI_ISL_919059 to 919061, EPI_ISL_919064 to 919066, EPI_ISL_919068 to 919069, EPI_ISL_919071 to 919073, EPI_ISL_919076 to 919078, EPI_ISL_919081, EPI_ISL_919083, EPI_ISL_919085 to 919086, EPI_ISL_919088 to 919090, EPI_ISL_919094, EPI_ISL_919096, EPI_ISL_919098, EPI_ISL_919100, EPI_ISL_919104 to 919105, EPI_ISL_919108 to 919113, EPI_ISL_919115 to 919118, EPI_ISL_919120 to 919133, EPI_ISL_919135, EPI_ISL_919137 to 919138, EPI_ISL_919140 to 919142, EPI_ISL_919144 to 919177, EPI_ISL_919179 to 919189, EPI_ISL_920894 to 920909, EPI_ISL_920911 to 920923, EPI_ISL_920925 to 920933, EPI_ISL_920935 to 920942, EPI_ISL_920944 to 920974, EPI_ISL_923685 to 923689, EPI_ISL_923691 to 923715, EPI_ISL_923717, EPI_ISL_923719 to 923757, EPI_ISL_923759, EPI_ISL_923761 to 923776, EPI_ISL_923778 to 923779, EPI_ISL_923782 to 923800, EPI_ISL_923802 to 923805, EPI_ISL_923807 to 923808, EPI_ISL_923810 to 923816, EPI_ISL_923818, EPI_ISL_923820 to 923822, EPI_ISL_923824 to 923826, EPI_ISL_923828 to 923837, EPI_ISL_923841, EPI_ISL_923844 to 923845, EPI_ISL_923847 to 923851, EPI_ISL_923853 to 923854, EPI_ISL_923856 to 923874, EPI_ISL_923876, EPI_ISL_923878 to 923881, EPI_ISL_923883 to 923888, EPI_ISL_923890 to 923898, EPI_ISL_923900 to 923901, EPI_ISL_923904 to 923917, EPI_ISL_923919 to 923952, EPI_ISL_923954 to 923959, EPI_ISL_923961 to 923962, EPI_ISL_923964 to 923986, EPI_ISL_923989 to 923994, EPI_ISL_923996 to 923997, EPI_ISL_923999, EPI_ISL_924001 to 924006, EPI_ISL_924008 to 924009, EPI_ISL_924011 to 924036, EPI_ISL_924038 to 924039, EPI_ISL_924041 to 924060, EPI_ISL_924062 to 924076, EPI_ISL_949256, EPI_ISL_949258 to 949263, EPI_ISL_949265 to 949270, EPI_ISL_949272 to 949282, EPI_ISL_949284 to 949289, EPI_ISL_949291, EPI_ISL_949293 to 949309, EPI_ISL_949311 to 949326, EPI_ISL_949427 to 949429, EPI_ISL_949431 to 949432, EPI_ISL_949434 to 949478, EPI_ISL_949480 to 949484, EPI_ISL_949486, EPI_ISL_949488 to 949489, EPI_ISL_949491 to 949499, EPI_ISL_949501, EPI_ISL_949503 to 949507, EPI_ISL_949509, EPI_ISL_949511, EPI_ISL_949513, EPI_ISL_949515, EPI_ISL_949517, EPI_ISL_949522 to 949540, EPI_ISL_949542 to 949556, EPI_ISL_949567, EPI_ISL_949575, EPI_ISL_949579 to 949580, EPI_ISL_949583 to 949587, EPI_ISL_949593 to 949595, EPI_ISL_949597, EPI_ISL_949599 to 949600, EPI_ISL_949602, EPI_ISL_952858 to 952860, EPI_ISL_952862 to 952863, EPI_ISL_952865 to 952868, EPI_ISL_952871, EPI_ISL_952874, EPI_ISL_952876 to 952886, EPI_ISL_952888 to 952894, EPI_ISL_952896 to 952897, EPI_ISL_952899 to 952910, EPI_ISL_952912 to 952914, EPI_ISL_952916 to 952917, EPI_ISL_952919 to 952920, EPI_ISL_952923 to 952926, EPI_ISL_952929, EPI_ISL_952932 to 952935, EPI_ISL_952937 to 952941, EPI_ISL_952943 to 952949, EPI_ISL_996066 to 996067, EPI_ISL_996069 to 996071, EPI_ISL_996073, EPI_ISL_996075, EPI_ISL_996077, EPI_ISL_996079 to 996081, EPI_ISL_996598 to 996603, EPI_ISL_996637 to 996643, EPI_ISL_996647 to 996651, EPI_ISL_996653, EPI_ISL_996655 to 996656, EPI_ISL_996658 to 996667, EPI_ISL_996670, EPI_ISL_996673, EPI_ISL_996675 to 996677, EPI_ISL_996679 to 996680, EPI_ISL_996682, EPI_ISL_996686 to 996688, EPI_ISL_996691 to 996693, EPI_ISL_996697 to 996703, EPI_ISL_996705 to 996706, EPI_ISL_996709 to 996711, EPI_ISL_996713 to 996718, EPI_ISL_996720 to 996723, EPI_ISL_996725, EPI_ISL_996727, EPI_ISL_996729, EPI_ISL_996731 to 996734, EPI_ISL_996736, EPI_ISL_996738 to 996740, EPI_ISL_996743 to 996777, EPI_ISL_996779 to 996790, EPI_ISL_996792 to 996806, EPI_ISL_996808 to 996833, EPI_ISL_996835 to 996851, EPI_ISL_996854 to 996858, EPI_ISL_996860 to 996892, EPI_ISL_996894 to 996952, EPI_ISL_996954 to 996980, EPI_ISL_996982 to 997021, EPI_ISL_997023 to 997049, EPI_ISL_1000631, EPI_ISL_1000633 to 1000640, EPI_ISL_1047069 to 1047070, EPI_ISL_1047072, EPI_ISL_1047074, EPI_ISL_1047076, EPI_ISL_1047080, EPI_ISL_1047082 to 1047084, EPI_ISL_1047086, EPI_ISL_1047088, EPI_ISL_1047090 to 1047098, EPI_ISL_1047100 to 1047139, EPI_ISL_1047141 to 1047154, EPI_ISL_1047156 to 1047204, EPI_ISL_1047206 to 1047217, EPI_ISL_1047220 to 1047233, EPI_ISL_1047235 to 1047252, EPI_ISL_1047254 to 1047273, EPI_ISL_1047275 to 1047278, EPI_ISL_1047280 to 1047292, EPI_ISL_1047294 to 1047302, EPI_ISL_1047305 to 1047331, EPI_ISL_1047333 to 1047336, EPI_ISL_1047338 to 1047370, EPI_ISL_1047372 to 1047421, EPI_ISL_1047423 to 1047465, EPI_ISL_1047467 to 1047493, EPI_ISL_1047495 to 1047498, EPI_ISL_1047500 to 1047542, EPI_ISL_1047544 to 1047581, EPI_ISL_1050605 to 1050606, EPI_ISL_1050610, EPI_ISL_1050614, EPI_ISL_1050619, EPI_ISL_1050632 to 1050649, EPI_ISL_1050651 to 1050675, EPI_ISL_1050685 to 1050690, EPI_ISL_1050692 to 1050696, EPI_ISL_1050698 to 1050702, EPI_ISL_1050705 to 1050722, EPI_ISL_1103883 to 1103885, EPI_ISL_1103888, EPI_ISL_1103890 to 1103892, EPI_ISL_1103894 to 1103948, EPI_ISL_1176402 to 1176812, EPI_ISL_1177636 to 1177642, EPI_ISL_1177647, EPI_ISL_1177649, EPI_ISL_1177651 to 1177675, EPI_ISL_1177677 to 1177687, EPI_ISL_1179891 to 1180052, EPI_ISL_1247021 to 1247032, EPI_ISL_1247289 to 1247358, EPI_ISL_1247360 to 1247368, EPI_ISL_1247571 to 1247574, EPI_ISL_1247576, EPI_ISL_1247578, EPI_ISL_1247580 to 1247592, EPI_ISL_1247594 to 1247601, EPI_ISL_1247603, EPI_ISL_1247605, EPI_ISL_1247608, EPI_ISL_1247611 to 1247622, EPI_ISL_1247624 to 1247629, EPI_ISL_1247631 to 1247632, EPI_ISL_1250124 to 1250221, EPI_ISL_1308676 to 1308697, EPI_ISL_1308699 to 1308702, EPI_ISL_1310455 to 1310701			
see above	Department of Pathology, University of Cambridge	COVID-19 Genomics UK (COG-UK) Consortium	Aminu S. Jahun; Anna Yakovleva; Charlotte J. Houldcroft; Fahad A Khokhar; Grant Hall; Ian Goodfellow; Iliana Georgana; Laura G Caller; Luke W Meredith; M. Estee Torok; M. Estée Török; M. Est©e Trk; Malte Pinckert; Martin D. Curran; Myra Hosmillo; Rhys Izu; Rhys Izuagbe; Sarah L. Caddy; Surendra Parmar; Theresa Feltwell; William L. Hamilton; Yasmin Chaudhry
EPI_ISL_458571, EPI_ISL_459173, EPI_ISL_459241, EPI_ISL_459243, EPI_ISL_459258, EPI_ISL_524590, EPI_ISL_524595			
see above	Department of Pathology, University of Cambridge	Wellcome Sanger Institute for the COVID-19 Genomics UK (COG-UK) Consortium	Aminu S. Jahun; Anna Yakovleva; Charlotte J. Houldcroft; Cordelia Langford; David K. Jackson; Dominic Kwiatkowski; Ewan Harrison; Fahad A Khokhar; Grant Hall; Ian Goodfellow; Ian Johnston; John Sillitoe on behalf of the Wellcome Sanger Institute COVID-19 Surveillance Team; Laura G Caller; Luke W Meredith; M. Estée Török; Martin D. Curran; Myra Hosmillo; Roberto Amato; Sarah L. Caddy; Sonia Goncalves; Theresa Feltwell; William L. Hamilton; and Alex Alderton
EPI_ISL_438248 to 438545, EPI_ISL_439368 to 439664, EPI_ISL_439863 to 439952, EPI_ISL_439954 to 439956, EPI_ISL_439958, EPI_ISL_439961, EPI_ISL_439963, EPI_ISL_439968, EPI_ISL_439970, EPI_ISL_439975, EPI_ISL_439977, EPI_ISL_439980, EPI_ISL_439987, EPI_ISL_439989 to 439991, EPI_ISL_439993 to 439997, EPI_ISL_439999, EPI_ISL_440003 to 440004, EPI_ISL_440007, EPI_ISL_440009 to 440012, EPI_ISL_440014 to 440015, EPI_ISL_440017, EPI_ISL_440019, EPI_ISL_440024 to 440025, EPI_ISL_440030, EPI_ISL_440034, EPI_ISL_440040 to 440041, EPI_ISL_440044 to 440049, EPI_ISL_440053, EPI_ISL_440055, EPI_ISL_440057, EPI_ISL_440059 to 440062, EPI_ISL_440064, EPI_ISL_440066 to 440067, EPI_ISL_440069, EPI_ISL_440072 to 440080, EPI_ISL_440082 to 440083, EPI_ISL_440085 to 440086, EPI_ISL_440088 to 440090, EPI_ISL_440096 to 440097, EPI_ISL_440099, EPI_ISL_440104, EPI_ISL_440107 to 440112, EPI_ISL_440117, EPI_ISL_440120 to 440122, EPI_ISL_440125, EPI_ISL_440129, EPI_ISL_440132 to 440133, EPI_ISL_440138, EPI_ISL_440143 to 440144, EPI_ISL_440146 to 440147, EPI_ISL_440150, EPI_ISL_440152, EPI_ISL_440155, EPI_ISL_440159, EPI_ISL_440161, EPI_ISL_440163 to 440164, EPI_ISL_440166, EPI_ISL_440169 to 440171, EPI_ISL_440173, EPI_ISL_440176 to 440177, EPI_ISL_440179 to 440186, EPI_ISL_440188, EPI_ISL_440193, EPI_ISL_440195, EPI_ISL_440197, EPI_ISL_440202 to 440203, EPI_ISL_440207 to 440209, EPI_ISL_440211, EPI_ISL_440213, EPI_ISL_440217, EPI_ISL_440223, EPI_ISL_440226, EPI_ISL_440230 to 440231, EPI_ISL_440234, EPI_ISL_440237, EPI_ISL_440239 to 440240, EPI_ISL_440244, EPI_ISL_440246 to 440247, EPI_ISL_440254, EPI_ISL_440257 to 440261, EPI_ISL_440263 to 440265, EPI_ISL_440267 to 440268, EPI_ISL_440270 to 440271, EPI_ISL_440278 to 440285, EPI_ISL_440287 to 440290, EPI_ISL_440293, EPI_ISL_440295 to 440297, EPI_ISL_440300 to 440622, EPI_ISL_440810 to 440851, EPI_ISL_441052 to 441349, EPI_ISL_441547 to 441658, EPI_ISL_441660 to 441661, EPI_ISL_441664 to 441669, EPI_ISL_441671 to 441674, EPI_ISL_441677 to 441684, EPI_ISL_441686 to 441689, EPI_ISL_441692, EPI_ISL_441695, EPI_ISL_441700 to 441703, EPI_ISL_441705 to 441707, EPI_ISL_441709 to 441711, EPI_ISL_441713, EPI_ISL_441715, EPI_ISL_441718, EPI_ISL_441720 to 441729, EPI_ISL_441731 to 441732, EPI_ISL_441734 to 441736, EPI_ISL_441738 to 441746, EPI_ISL_441748 to 441750, EPI_ISL_441752, EPI_ISL_441754 to 441760, EPI_ISL_441762 to 441764, EPI_ISL_441767 to 441769, EPI_ISL_441772 to 441774, EPI_ISL_441777 to 441780, EPI_ISL_441782 to 441846, EPI_ISL_442045 to 442343, EPI_ISL_442524 to 442623, EPI_ISL_442811 to 443118, EPI_ISL_443318 to 443685, EPI_ISL_458516 to 458570, EPI_ISL_458572 to 458577, EPI_ISL_459166 to 459172, EPI_ISL_459174 to 459240, EPI_ISL_459242, EPI_ISL_459244 to 459257, EPI_ISL_459259 to 459297, EPI_ISL_459304 to 459323, EPI_ISL_459410 to 459504, EPI_ISL_469891 to 469913, EPI_ISL_470090 to 470355, EPI_ISL_470358 to 470528, EPI_ISL_470531 to 470533, EPI_ISL_470535 to 470538, EPI_ISL_489381 to 489390, EPI_ISL_489394 to 489395, EPI_ISL_489397 to 489400, EPI_ISL_489402 to 489406, EPI_ISL_489409 to 489412, EPI_ISL_489414 to 489417, EPI_ISL_489419 to 489428, EPI_ISL_489430 to 489441, EPI_ISL_489443 to 489448, EPI_ISL_489450 to 489454, EPI_ISL_489456 to 489469, EPI_ISL_489471 to 489479, EPI_ISL_489481 to 489488, EPI_ISL_489490 to 489522, EPI_ISL_489524 to 489529, EPI_ISL_489531 to 489535, EPI_ISL_489538 to 489542, EPI_ISL_489544 to 489546, EPI_ISL_489548 to 489566, EPI_ISL_489568 to 489577, EPI_ISL_492189 to 492194, EPI_ISL_492196, EPI_ISL_492198 to 492201, EPI_ISL_492204 to 492206, EPI_ISL_511959, EPI_ISL_511970, EPI_ISL_511982, EPI_ISL_524493, EPI_ISL_524495, EPI_ISL_524497 to 524498, EPI_ISL_524501 to 524503, EPI_ISL_524505 to 524506, EPI_ISL_524508 to 524509, EPI_ISL_524515, EPI_ISL_524521 to 524522, EPI_ISL_524525, EPI_ISL_524528 to 524531, EPI_ISL_524537 to 524538, EPI_ISL_524541, EPI_ISL_524544, EPI_ISL_524546, EPI_ISL_524548, EPI_ISL_524551, EPI_ISL_524553, EPI_ISL_524558, EPI_ISL_524560, EPI_ISL_524564, EPI_ISL_524568, EPI_ISL_524570, EPI_ISL_524573 to 524589, EPI_ISL_524591 to 524594, EPI_ISL_524596 to 524618, EPI_ISL_591076 to 591082, EPI_ISL_591091 to 591093			
see above	Department of Pathology, University of Cambridge	Wellcome Sanger Institute for the COVID-19 Genomics UK (COG-UK) consortium	Alex Alderton; Aminu S. Jahun; Anna Yakovleva; Charlotte J. Houldcroft; Cordelia Langford; David K. Jackson; Dominic Kwiatkowski; Ewan Harrison; Fahad A Khokhar; Grant Hall; Ian Goodfellow; Ian Johnston; John Sillitoe on behalf of the Wellcome Sanger Institute COVID-19 Surveillance Team; John Sillitoe on behalf of the Wellcome Sanger Institute COVID-19 Surveillance Team (http://www.sanger.ac.uk/covid-team); Laura G Caller; Luke W Meredith; M. Estée Török; Martin D. Curran; Myra Hosmillo; Roberto Amato; Sarah L. Caddy; Sonia Goncalves; Theresa Feltwell; William L. Hamilton; and Alex Alderton
EPI_ISL_984305, EPI_ISL_984307	Department of Poultry Diseases	Department of Omics Analysis	Arkadiusz Bomba; Domanska-Blicharz Katarzyna; Ewelina Iwan; Niemczuk Krzysztof; Orlowska Anna; Smreczak Marcin
EPI_ISL_1034281 to 1034301, EPI_ISL_1289789 to 1289826, EPI_ISL_1289829, EPI_ISL_1289833 to 1289955	Department of Public Health Microbiology Ljubljana, National Laboratory for Health, Environment and Food	Department of Public Health Microbiology Ljubljana, National Laboratory for Health, Environment and Food	José Gonçalves; Katarina Prosenc; Martin Bosilj; Metka Paragi; Tom Koritnik
EPI_ISL_576146 to 576149	Department of Respiratory & Other Viral Infections of L.V. Gromashevsky Institute of Epidemiology & Infectious Diseases NAMS of Ukraine	Department of Respiratory & Other Viral Infections of L.V. Gromashevsky Institute of Epidemiology & Infectious Diseases NAMS of Ukraine, JSC "Farmak"	Alla Mironenko; Andriy Goy; Ihor Kravchuk; Larysa Radchenko; Liudmyla Bolotova; Nataliia Teteriuk
EPI_ISL_1121961 to 1121962	Department of Respiratory Medicine, Nagasaki University Hospital, Nagasaki, Japan	Department of Virology, Institute of Tropical Medicine, Nagasaki University, Nagasaki, Japan	Ayumi Fujita; Katsunori Yanagihara; Kazuko Yamamoto; Kenji Ota; Koichi Izumikawa; Kouichi Morita; Masato Tashiro; Nobuyuki Ashizawa; Satoshi Irifune; Taiga Miyazaki; Takahiro Takazono; Takeshi Tanaka; Tatsuro Hirayama; Yoshifumi Imamura; and Hiroshi Mukae
EPI_ISL_582509	Department of Respiratory and other Viral Infections of	Department of Respiratory and other Viral Infections of	Alla Mironenko; Andriy Goy; Ihor Kravchuk; Larysa Radchenko; Ludmyla Bolotova; Nataliia Teteriuk

	L.V.Gromashevsky Institute of Epidemiology & Infectious Diseases NAMS of Ukraine	L.V.Gromashevsky Institute of Epidemiology & Infectious Diseases NAMS of Ukraine, JSC "Farmak"	
EPI_ISL_582510	Department of Respiratory and other Viral Infections of L.V.Gromashevsky Institute of Epidemiology & Infectious Diseases NAMS of Ukraine	Department of Respiratory and other Viral Infections of L.V.Gromashevsky Institute of Epidemiology & Infectious Diseases NAMS of Ukrain, JSC "Farmak"	Alla Mironenko; Andriy Goy; Ihor Kravchuk; Larysa Radchenko; Ludmyla Bolotova; Nataliia Teteriuk
EPI_ISL_582511 to 582513, EPI_ISL_654819 to 654820, EPI_ISL_979970 to 979973, EPI_ISL_1121990 to 1121992, EPI_ISL_1122014	Department of Respiratory and other Viral Infections of L.V.Gromashevsky Institute of Epidemiology & Infectious Diseases NAMS of Ukrain	Department of Respiratory and other Viral Infections of L.V.Gromashevsky Institute of Epidemiology & Infectious Diseases NAMS of Ukrain, JSC "Farmak"	Alla Mironenko; Andriy Goy; Ihor Kravchuk; Larysa Radchenko; Ludmyla Bolotova; Nataliia Teteriuk
EPI_ISL_654818	Department of Respiratory and other Viral Infections of L.V.Gromashevsky Institute of Epidemiology & Infectious Diseases NAMS of Ukrain	Department of Respiratory and other Viral Infections of L.V.Gromashevsky Institute of Epidemiology & Infectious Diseases NAMS of Ukrain, JSC "Farmak"	Alla Mironenko; Andriy Goy; Ihor Kravchuk; Larysa Radchenko; Ludmyla Bolotova; Nataliia Teteriuk
EPI_ISL_1215947, EPI_ISL_1216923, EPI_ISL_1217356, EPI_ISL_1217709	Department of Sciences for Health Promotion and Mother-Child Care "G. D'Alessandro", University of Palermo	Tumor Immunology Unit, Department of Health Sciences, University of Palermo School of Medicine; Section of Microbiology, University of Palermo School of Medicine; National Research Council of Italy - High Performance Computing and Networking Institute (CNR-ICAR) of Palermo	Alfonso Maurizio; Antonino; Antonio; Bonura; Cancila; Celestino; Claudio; Craxi; Davide; Di Bernardo; Fiannaca; Floriana; Francesca; Francesco; Gaia; Giammanco; La Paglia; La Rosa; Laura; Massimo; Maurizio Giovanni.; Morello; Tripodo; Urso; Vacca; Valeria; Vitale
EPI_ISL_414599	Department of Surgical Sciences, University of Cagliari	Universita' di Cagliari	Coghe; DelRio; F. and Orru, G.; Fais, S.; Loddo, M.; N.M.C.; Palmieri, G.; Scano, A.; Scioscia, R.
EPI_ISL_732948 to 732959	Department of Tropical Parasitology	Laboratory of Recombinant Vaccines	Boguslaw Szewczyk; Kirsi Aaltonen; Lukasz Rabalski; Maciej Grzybek; Maciej Kosinski; Ravi Kant; Tarja Sironen; Teemu Smura
EPI_ISL_487275	Department of Veterinary Pathology, University of Liege - FARAH	Department of Veterinary Pathology, University of Liege - FARAH	A.-S.; Clercx, C.; Desmecht, D.; Eloit, M.; Escriou, N.; Garigliany, M.; Giet, D.; Huon, C.; Vanlaere; van der Werf, S.
EPI_ISL_450198 to 450211, EPI_ISL_766055, EPI_ISL_807148 to 807149, EPI_ISL_1072998, EPI_ISL_1073001, EPI_ISL_1073004, EPI_ISL_1073012, EPI_ISL_1073015, EPI_ISL_1073021, EPI_ISL_1073023 to 1073028, EPI_ISL_1073030 to 1073033, EPI_ISL_1138735 to 1138742, EPI_ISL_1173257, EPI_ISL_1173505, EPI_ISL_1173507, EPI_ISL_1173510, EPI_ISL_1173513 to 1173514, EPI_ISL_1250319, EPI_ISL_1250574			
see above	Department of Virology	Department of Virology	Aamer Ikram; Abdul Ahad; Ackermann, N.; Adnan Khurshid; Antwerpen, M.; Bengs, K.; Berger, A.; Boehm, S.; Boehmer; Boender; Buchholz, U.; Cai, W.; Corman; D.V.; Dangel, A.; Drosten, C.; Eberle, U.; Fingerle, V.; Grahl, A.; Haas, W.; Hamouda, O.; Hamza Ahmad; Hamza Ahmed; Hoch, M.; Hoermansdorfer, S.; Ippisch, S.; Jones; Katz, K.; Konrad, R.; Liebl, B.; M.M.; Marosevic; Massab; Massab Umair; Muehlemann, B.; Muhammad Salman; Muller, N.; Nazish Badar; Poertner, K.; Protzer, U.; Reich, A.; Rexroth, U.; Salmaan Sharif; Samee Ullah; Sana Tamim; Schneider, J.; Sing, A.; T.C.; T.S.; Treis, B.; Umair; V.M.; Veith, T.; Walter, M.; Wicklein, B.; Woelfel, R.; Woudenberg, T.; Zaira Rehman; Zapf, A.; Zeitlmann, N.; an der Heiden, M.
EPI_ISL_872589 to 872590	Department of Virology Institute of Tropical Medicine Nagasaki University	Department of Virology Institute of Tropical Medicine Nagasaki University	Ph. D. Kouichi Morita; Takeshi Nabeshima Takahiro Takazono Nobuyuki Ashizawa Taiga Miyazaki Shingo Inoue Mya Myat Ngwe Tun Koichi Izumikawa Hiroshi Mukae Meng Ling Moi
EPI_ISL_1122890, EPI_ISL_1123289	Department of Virology I, National Institute of Infectious Diseases	Department of Veterinary Science, National Institute of Infectious Diseases	Keita Ishijima; Ken Maeda; Shuetsu Fukushi; Souichi Yamada; Tsukasa Yamamoto; Yudai Kuroda
EPI_ISL_768526 to 768527, EPI_ISL_804007 to 804008	Department of Virology I, National Institute of Infectious Diseases	Pathogen Genomics Center, National Institute of Infectious Diseases	Kentaro Itokawa; Makoto Kuroda; Masanori Hashino; Rina Tanaka; Shuetsu Fukushi; Souichi Yamada; Tsuyoshi Sekizuka
EPI_ISL_407084, EPI_ISL_529135	Department of Virology III, National Institute of Infectious Diseases	Pathogen Genomics Center, National Institute of Infectious Diseases	Kazuya Shirato; Kentaro Itokawa; Makoto Kuroda; Makoto Takeda; Masanori Hashino; Naganori Nao; Rina Tanaka; Shinji Watanabe; Shutoku Matsuyama; Takaji Wakita; Tsuyoshi Sekizuka
EPI_ISL_413602 to 413605, EPI_ISL_414640 to 414646, EPI_ISL_418385 to 418411, EPI_ISL_481513 to 481715, EPI_ISL_481717 to 481740, EPI_ISL_732565 to 732653, EPI_ISL_737034 to 737080, EPI_ISL_737209 to 737235, EPI_ISL_755943 to 756125, EPI_ISL_756165 to 756183, EPI_ISL_756247 to 756252, EPI_ISL_756282 to 756292, EPI_ISL_757288 to 757394, EPI_ISL_759763 to 759841, EPI_ISL_759899 to 759954, EPI_ISL_766016 to 766027, EPI_ISL_833201 to 833227, EPI_ISL_862052 to 862074, EPI_ISL_862082 to 862120, EPI_ISL_902758 to 902916, EPI_ISL_995839 to 996001, EPI_ISL_1081963 to 1082135, EPI_ISL_1082137 to 1082251, EPI_ISL_1184542 to 1184557, EPI_ISL_1217188, EPI_ISL_1217190, EPI_ISL_1217192 to 1217193, EPI_ISL_1217195, EPI_ISL_1217197 to 1217198, EPI_ISL_1217200, EPI_ISL_1217202, EPI_ISL_1217205, EPI_ISL_1217207, EPI_ISL_1217210, EPI_ISL_1217212, EPI_ISL_1217215, EPI_ISL_1217217, EPI_ISL_1217220, EPI_ISL_1217222, EPI_ISL_1217225, EPI_ISL_1217227, EPI_ISL_1217230, EPI_ISL_1217233, EPI_ISL_1217235, EPI_ISL_1218645 to 1218652, EPI_ISL_1219684 to 1219700, EPI_ISL_1219719 to 1219745, EPI_ISL_1222927 to 1222943, EPI_ISL_1229156 to 1229161, EPI_ISL_1229388 to 1229399, EPI_ISL_1231207 to 1231292, EPI_ISL_1233188 to 1233221, EPI_ISL_1239428 to 1239442, EPI_ISL_1240077 to 1240184, EPI_ISL_1240658 to 1240676, EPI_ISL_1240681 to 1240714, EPI_ISL_1240938 to 1240990, EPI_ISL_1241843 to 1241859, EPI_ISL_1278943 to 1279218			
see above	Department of Virology and Immunology, University of Helsinki and Helsinki University Hospital, Huslab Finland	Department of Virology, Faculty of Medicine, University of Helsinki, Helsinki, Finland	Essi Korhonen; Fathiah Zakham; Hanna Jarva; Hanna Liimatainen; Hannimari Kallio-Kokko; Harri Kangas; Hussein Alburkat; Jenni Virtanen; Maija Lappalainen; Maija Suvanto; Mert Erdin; Olli Vapalahti; Pekka Ellonen; Phuoc Truong; Ravi Kant; Sari Hannula; Satu Kurkela; Teemu Smura
EPI_ISL_1273443	Department of Virology, Bangabandhu Sheikh Mujib Medical University	Bioinformatics Division, National Institute of Biotechnology (NIB)	Arittra Bhattacharjee; Ishtiaque Ahammad; Md. Salimullah; Mohammad Uzzal Hossain; Munira Jahan; Raad Rahmat; S M Rashedul Islam; Saif Ullah Munshi; Tahmina Akhter
EPI_ISL_1299195 to 1299216	Department of Virology, Bangabandhu Sheikh Mujib Medical University	Department of Virology, Bangabandhu Sheikh Mujib Medical University	Arittra Bhattacharjee; Ishtiaque Ahammad; Md. Salimullah; Mohammad Uzzal Hossain; Munira Jahan; Raad Rahmat; S M Rashedul Islam; Saif Ullah Munshi; Tahmina Akhter
EPI_ISL_1259188 to 1259207	Department of Virology, Istituto Zooprofilattico Sperimentale del Lazio e della Toscana (IZSLT)	Department of General Diagnostics; Department of Virology; Istituto Zooprofilattico Sperimentale del Lazio e della Toscana (IZSLT)	Alessia Franco; Antonella Cersini; Antonio Battisti.; Elena L. Diaconu; Fabiola Feltrin; Giuseppe Manna; Patricia Alba; Raffaela Conti; Teresa Scicluna; Virginia Carfora
EPI_ISL_1014996 to 1015334, EPI_ISL_1063398, EPI_ISL_1063906	Department of Virology, Pitié-Salpêtrière hospital	Department of Virology, Pitié-Salpêtrière hospital	Anne-Geneviève Marcelin; Aude Jary; Karen Zafilaza; Stéphane Marot; Valentin Leducq; Vincent Calvez
EPI_ISL_855557 to 855572	Department of Virology, Principal Military Hospital of Instruction of Tunis	Bundeswehr Institute of Microbiology	Habiba Naija; Kilian Stoecker; Malena Bestehorn-Willmann; Markus H. Antwerpen; Mathias C. Walter; Roman Wölfel & Mohamed Ben Moussa; Simone Eckstein; Susann Handrick
EPI_ISL_757285 to 757286	Department of Virology, Public Health Laboratories Division	Department of Virology, Public Health Laboratories Division	Aamer Ikram; Massab Umair; Muhammad Salman
EPI_ISL_468161, EPI_ISL_468163, EPI_ISL_477164 to 477167	Department of Virology, Public Health Laboratories Division, National Institute of Health	Department of Virology, Public Health Laboratories Division, National Institute of Health	Aamer Ikram; Abdul Ahad; Adnan Khurshid; Hamza Ahmed Mirza; John Klena; Massab Umair; Muhammad Salman; Nazish Badar; Shannon Whitmer; Yasir Arshad
EPI_ISL_515974	Department of Virus and Microbiological Special Diagnostics, Statens Serum Institut	Albertsen lab, Department of Chemistry and Bioscience, Aalborg University, Denmark	Danish Corona Danica Consortia
EPI_ISL_855615 to 855901, EPI_ISL_855903, EPI_ISL_855905, EPI_ISL_855907, EPI_ISL_855909, EPI_ISL_855911, EPI_ISL_855913, EPI_ISL_855915, EPI_ISL_855918, EPI_ISL_855920, EPI_ISL_855922, EPI_ISL_855924, EPI_ISL_855926, EPI_ISL_855929, EPI_ISL_855931, EPI_ISL_855933, EPI_ISL_855935, EPI_ISL_855937, EPI_ISL_855939, EPI_ISL_855941, EPI_ISL_855943, EPI_ISL_855946, EPI_ISL_855948, EPI_ISL_855950, EPI_ISL_855952, EPI_ISL_855954, EPI_ISL_855956, EPI_ISL_855958, EPI_ISL_855961, EPI_ISL_855963, EPI_ISL_855965, EPI_ISL_855967, EPI_ISL_855969 to 856676, EPI_ISL_869265 to 870064, EPI_ISL_870066 to 870314, EPI_ISL_870316, EPI_ISL_870318, EPI_ISL_870320, EPI_ISL_870323 to 871324, EPI_ISL_871326 to 871787, EPI_ISL_925917 to 926445, EPI_ISL_926447 to 929762, EPI_ISL_929764 to 930565, EPI_ISL_970810 to 970817, EPI_ISL_970819 to 970825, EPI_ISL_970827 to 970839, EPI_ISL_970841 to 970844, EPI_ISL_970846 to 970855, EPI_ISL_970857 to 970859, EPI_ISL_970861 to 970874, EPI_ISL_970876 to 970877, EPI_ISL_970879 to 970898, EPI_ISL_970900 to 970901, EPI_ISL_970903 to 970924, EPI_ISL_970926 to 970930, EPI_ISL_970932 to 970943, EPI_ISL_970945 to 970946, EPI_ISL_970948 to 970964, EPI_ISL_970966 to 970967, EPI_ISL_970969 to 970985, EPI_ISL_970987 to 970994, EPI_ISL_970996 to 971007, EPI_ISL_971009 to 971011, EPI_ISL_971013 to 971022, EPI_ISL_971024 to 971035, EPI_ISL_971038, EPI_ISL_971041, EPI_ISL_971045, EPI_ISL_971048, EPI_ISL_971051, EPI_ISL_971056, EPI_ISL_971058, EPI_ISL_971062, EPI_ISL_971065, EPI_ISL_971067, EPI_ISL_971070, EPI_ISL_971073, EPI_ISL_971078, EPI_ISL_971081 to 971082, EPI_ISL_971085, EPI_ISL_971088, EPI_ISL_971092, EPI_ISL_971095, EPI_ISL_971098, EPI_ISL_971100, EPI_ISL_971103, EPI_ISL_971106, EPI_ISL_971110, EPI_ISL_971112, EPI_ISL_971115, EPI_ISL_971118, EPI_ISL_971121, EPI_ISL_971124, EPI_ISL_971127, EPI_ISL_971131, EPI_ISL_971133, EPI_ISL_971136, EPI_ISL_971140, EPI_ISL_971143, EPI_ISL_971145, EPI_ISL_971148, EPI_ISL_971151 to 971152, EPI_ISL_971155, EPI_ISL_971158 to 971159, EPI_ISL_971163, EPI_ISL_971166, EPI_ISL_971169, EPI_ISL_971172, EPI_ISL_971175, EPI_ISL_971178, EPI_ISL_971181, EPI_ISL_971184, EPI_ISL_971186, EPI_ISL_971188, EPI_ISL_971193, EPI_ISL_971195, EPI_ISL_971198, EPI_ISL_971201, EPI_ISL_971203, EPI_ISL_971207, EPI_ISL_971210, EPI_ISL_971213 to 971214, EPI_ISL_971217, EPI_ISL_971220, EPI_ISL_971223, EPI_ISL_971226, EPI_ISL_971229, EPI_ISL_971232, EPI_ISL_971237, EPI_ISL_971241, EPI_ISL_971244, EPI_ISL_971247, EPI_ISL_971250, EPI_ISL_971252, EPI_ISL_971257, EPI_ISL_971260, EPI_ISL_971263, EPI_ISL_971266, EPI_ISL_971270, EPI_ISL_971272, EPI_ISL_971275, EPI_ISL_971279 to 971280, EPI_ISL_971283, EPI_ISL_971287, EPI_ISL_971290, EPI_ISL_971293, EPI_ISL_971296, EPI_ISL_971299, EPI_ISL_971302, EPI_ISL_971306, EPI_ISL_971309, EPI_ISL_971312, EPI_ISL_971315, EPI_ISL_971318, EPI_ISL_971322 to 971323, EPI_ISL_971326, EPI_ISL_971329, EPI_ISL_971332, EPI_ISL_971335, EPI_ISL_971338, EPI_ISL_971341, EPI_ISL_971345 to 971346, EPI_ISL_971349, EPI_ISL_971353, EPI_ISL_971355, EPI_ISL_971358, EPI_ISL_971361, EPI_ISL_971363, EPI_ISL_971366, EPI_ISL_971368, EPI_ISL_971371, EPI_ISL_971375, EPI_ISL_971379, EPI_ISL_971381, EPI_ISL_971384, EPI_ISL_971387, EPI_ISL_971389, EPI_ISL_971391, EPI_ISL_971395, EPI_ISL_971398, EPI_ISL_971401, EPI_ISL_971404, EPI_ISL_971408, EPI_ISL_971411, EPI_ISL_971415, EPI_ISL_971418 to 971419, EPI_ISL_971422, EPI_ISL_971426, EPI_ISL_971429, EPI_ISL_971431, EPI_ISL_971434, EPI_ISL_971436 to 971437, EPI_ISL_971439, EPI_ISL_971441, EPI_ISL_971443, EPI_ISL_971445, EPI_ISL_971447, EPI_ISL_971450, EPI_ISL_971452, EPI_ISL_971455 to 971456, EPI_ISL_971458, EPI_ISL_971460 to 973476, EPI_ISL_973478, EPI_ISL_973480, EPI_ISL_973482, EPI_ISL_973484, EPI_ISL_973486 to 973487, EPI_ISL_973489, EPI_ISL_973492 to 973493, EPI_ISL_973495, EPI_ISL_973497, EPI_ISL_973499, EPI_ISL_973501, EPI_ISL_973503, EPI_ISL_973505, EPI_ISL_973508, EPI_ISL_973510, EPI_ISL_973512, EPI_ISL_973514, EPI_ISL_973516, EPI_ISL_973518 to 973519, EPI_ISL_973521, EPI_ISL_973523, EPI_ISL_973525, EPI_ISL_973527, EPI_ISL_973529, EPI_ISL_973532 to 973533, EPI_ISL_973535, EPI_ISL_973538, EPI_ISL_973540, EPI_ISL_973542, EPI_ISL_973544, EPI_ISL_973546, EPI_ISL_973548, EPI_ISL_973550, EPI_ISL_973553, EPI_ISL_973555, EPI_ISL_973557, EPI_ISL_973559, EPI_ISL_973561, EPI_ISL_973563, EPI_ISL_973566, EPI_ISL_973568, EPI_ISL_973570, EPI_ISL_973572, EPI_ISL_973574, EPI_ISL_973576, EPI_ISL_973578, EPI_ISL_973580, EPI_ISL_973582, EPI_ISL_973584, EPI_ISL_973586, EPI_ISL_973589, EPI_ISL_973591, EPI_ISL_973593, EPI_ISL_973595, EPI_ISL_973597, EPI_ISL_973599, EPI_ISL_973601, EPI_ISL_973603,			

EPI_ISL_973605, EPI_ISL_973607, EPI_ISL_973609, EPI_ISL_973611, EPI_ISL_973614, EPI_ISL_973616, EPI_ISL_973618, EPI_ISL_973621, EPI_ISL_973623 to 973624, EPI_ISL_973626, EPI_ISL_973628 to 973629, EPI_ISL_973631, EPI_ISL_973633, EPI_ISL_973635 to 973636, EPI_ISL_973638, EPI_ISL_973640 to 973641, EPI_ISL_973644 to 973645, EPI_ISL_973647 to 973648, EPI_ISL_973651 to 973652, EPI_ISL_973654, EPI_ISL_973656, EPI_ISL_973658, EPI_ISL_973660, EPI_ISL_973662, EPI_ISL_973664, EPI_ISL_973666, EPI_ISL_973668, EPI_ISL_973670, EPI_ISL_973672, EPI_ISL_973674, EPI_ISL_973676 to 973677, EPI_ISL_973680, EPI_ISL_973682, EPI_ISL_973684, EPI_ISL_973686, EPI_ISL_973688 to 973689, EPI_ISL_973691, EPI_ISL_973693 to 973694, EPI_ISL_973696, EPI_ISL_973698 to 973699, EPI_ISL_973701, EPI_ISL_973703 to 973704, EPI_ISL_973706, EPI_ISL_973708, EPI_ISL_973710 to 973711, EPI_ISL_973713, EPI_ISL_973715, EPI_ISL_973717, EPI_ISL_973720, EPI_ISL_973726, EPI_ISL_973728, EPI_ISL_973730, EPI_ISL_973732, EPI_ISL_973734 to 973735, EPI_ISL_973738 to 973739, EPI_ISL_973741, EPI_ISL_973743 to 973744, EPI_ISL_973746, EPI_ISL_973748, EPI_ISL_973750, EPI_ISL_973752, EPI_ISL_973754, EPI_ISL_973756 to 973757, EPI_ISL_973759, EPI_ISL_973761 to 973763, EPI_ISL_973765 to 973766, EPI_ISL_973768, EPI_ISL_973770, EPI_ISL_973772 to 973774, EPI_ISL_973777 to 973778, EPI_ISL_973780, EPI_ISL_973782, EPI_ISL_973784, EPI_ISL_973786 to 973787, EPI_ISL_973789 to 973790, EPI_ISL_973792 to 973793, EPI_ISL_973796, EPI_ISL_973798 to 973799, EPI_ISL_973801, EPI_ISL_973803 to 973804, EPI_ISL_973806, EPI_ISL_973808 to 973809, EPI_ISL_973811 to 973812, EPI_ISL_973814, EPI_ISL_973816 to 973817, EPI_ISL_973819 to 973820, EPI_ISL_973822, EPI_ISL_973824 to 973825, EPI_ISL_973827, EPI_ISL_973829, EPI_ISL_973831, EPI_ISL_973833, EPI_ISL_973835 to 973836, EPI_ISL_1022420 to 1023447, EPI_ISL_1023449, EPI_ISL_1023451, EPI_ISL_1023453, EPI_ISL_1023455, EPI_ISL_1023458, EPI_ISL_1023460, EPI_ISL_1023462, EPI_ISL_1023464, EPI_ISL_1023466, EPI_ISL_1023469, EPI_ISL_1023471 to 1023523, EPI_ISL_1023525 to 1023581, EPI_ISL_1023583, EPI_ISL_1023585, EPI_ISL_1023587, EPI_ISL_1023589, EPI_ISL_1023591, EPI_ISL_1023593, EPI_ISL_1023596, EPI_ISL_1023598, EPI_ISL_1023600, EPI_ISL_1023602, EPI_ISL_1023604, EPI_ISL_1023607, EPI_ISL_1023609, EPI_ISL_1023611, EPI_ISL_1023613, EPI_ISL_1023616, EPI_ISL_1023618 to 1023670, EPI_ISL_1023672, EPI_ISL_1023674, EPI_ISL_1023676, EPI_ISL_1023678, EPI_ISL_1023680, EPI_ISL_1023682, EPI_ISL_1023684, EPI_ISL_1023687, EPI_ISL_1023689, EPI_ISL_1023691, EPI_ISL_1023694, EPI_ISL_1023696, EPI_ISL_1023698, EPI_ISL_1023700, EPI_ISL_1023702, EPI_ISL_1023705, EPI_ISL_1023707, EPI_ISL_1023709, EPI_ISL_1023711, EPI_ISL_1023713, EPI_ISL_1023715, EPI_ISL_1023717, EPI_ISL_1023719, EPI_ISL_1023722, EPI_ISL_1023724, EPI_ISL_1023726, EPI_ISL_1023728, EPI_ISL_1023730 to 1023782, EPI_ISL_1023785, EPI_ISL_1023787, EPI_ISL_1023789, EPI_ISL_1023791, EPI_ISL_1023793, EPI_ISL_1023795, EPI_ISL_1023797, EPI_ISL_1023799, EPI_ISL_1023802, EPI_ISL_1023804, EPI_ISL_1023806, EPI_ISL_1023808, EPI_ISL_1023810, EPI_ISL_1023813 to 1023814, EPI_ISL_1023817, EPI_ISL_1023819, EPI_ISL_1023821, EPI_ISL_1023823, EPI_ISL_1023825, EPI_ISL_1023828, EPI_ISL_1023830, EPI_ISL_1023832, EPI_ISL_1023834, EPI_ISL_1023836, EPI_ISL_1023838, EPI_ISL_1023840, EPI_ISL_1023842, EPI_ISL_1023844, EPI_ISL_1023846 to 1025962, EPI_ISL_1065095 to 1066327, EPI_ISL_1066329 to 1066583, EPI_ISL_1066585 to 1066672, EPI_ISL_1066674 to 1066742, EPI_ISL_1066744 to 1066757, EPI_ISL_1066759, EPI_ISL_1066761, EPI_ISL_1066763, EPI_ISL_1066766, EPI_ISL_1066768, EPI_ISL_1066770, EPI_ISL_1066772, EPI_ISL_1066774, EPI_ISL_1066776, EPI_ISL_1066779, EPI_ISL_1066781 to 1066782, EPI_ISL_1066785, EPI_ISL_1066787, EPI_ISL_1066789, EPI_ISL_1066791, EPI_ISL_1066793, EPI_ISL_1066795, EPI_ISL_1066797 to 1067582, EPI_ISL_1123495, EPI_ISL_1123497, EPI_ISL_1123499 to 1123500, EPI_ISL_1123503, EPI_ISL_1123505, EPI_ISL_1123507, EPI_ISL_1123510, EPI_ISL_1123512, EPI_ISL_1123514, EPI_ISL_1123516, EPI_ISL_1123519, EPI_ISL_1123521, EPI_ISL_1123523, EPI_ISL_1123526, EPI_ISL_1123528, EPI_ISL_1123530, EPI_ISL_1123533, EPI_ISL_1123535, EPI_ISL_1123537, EPI_ISL_1123539, EPI_ISL_1123542, EPI_ISL_1123544, EPI_ISL_1123546, EPI_ISL_1123549, EPI_ISL_1123551, EPI_ISL_1123554, EPI_ISL_1123556, EPI_ISL_1123558, EPI_ISL_1123560, EPI_ISL_1123562, EPI_ISL_1123565, EPI_ISL_1123567, EPI_ISL_1123569, EPI_ISL_1123571, EPI_ISL_1123573, EPI_ISL_1123575, EPI_ISL_1123578, EPI_ISL_1123580, EPI_ISL_1123582, EPI_ISL_1123585, EPI_ISL_1123587, EPI_ISL_1123589, EPI_ISL_1123592, EPI_ISL_1123594, EPI_ISL_1123596, EPI_ISL_1123598, EPI_ISL_1123600, EPI_ISL_1123603, EPI_ISL_1123605, EPI_ISL_1123607, EPI_ISL_1123610, EPI_ISL_1123612, EPI_ISL_1123614, EPI_ISL_1123616, EPI_ISL_1123619, EPI_ISL_1123621, EPI_ISL_1123623, EPI_ISL_1123625, EPI_ISL_1123628, EPI_ISL_1123630, EPI_ISL_1123633, EPI_ISL_1123635, EPI_ISL_1123638, EPI_ISL_1123640, EPI_ISL_1123642, EPI_ISL_1123644, EPI_ISL_1123647, EPI_ISL_1123649, EPI_ISL_1123652, EPI_ISL_1123654, EPI_ISL_1123656, EPI_ISL_1123658, EPI_ISL_1123661, EPI_ISL_1123663, EPI_ISL_1123665, EPI_ISL_1123668, EPI_ISL_1123670, EPI_ISL_1123672, EPI_ISL_1123675, EPI_ISL_1123677, EPI_ISL_1123679, EPI_ISL_1123681, EPI_ISL_1123684, EPI_ISL_1123686, EPI_ISL_1123688, EPI_ISL_1123690, EPI_ISL_1123693, EPI_ISL_1123695, EPI_ISL_1123697, EPI_ISL_1123699, EPI_ISL_1123702, EPI_ISL_1123704, EPI_ISL_1123706, EPI_ISL_1123708, EPI_ISL_1123710, EPI_ISL_1123713, EPI_ISL_1123715, EPI_ISL_1123717, EPI_ISL_1123719, EPI_ISL_1123722, EPI_ISL_1123724, EPI_ISL_1123727, EPI_ISL_1123729, EPI_ISL_1123731, EPI_ISL_1123734, EPI_ISL_1123736, EPI_ISL_1123738, EPI_ISL_1123740, EPI_ISL_1123742, EPI_ISL_1123745, EPI_ISL_1123747, EPI_ISL_1123749, EPI_ISL_1123751, EPI_ISL_1123754, EPI_ISL_1123756, EPI_ISL_1123758, EPI_ISL_1123760, EPI_ISL_1123763, EPI_ISL_1123765, EPI_ISL_1123767, EPI_ISL_1123770, EPI_ISL_1123772, EPI_ISL_1123774, EPI_ISL_1123777, EPI_ISL_1123779, EPI_ISL_1123781, EPI_ISL_1123784, EPI_ISL_1123786, EPI_ISL_1123788, EPI_ISL_1123791, EPI_ISL_1123793, EPI_ISL_1123796, EPI_ISL_1123798, EPI_ISL_1123800, EPI_ISL_1123802, EPI_ISL_1123804, EPI_ISL_1123807, EPI_ISL_1123809, EPI_ISL_1123811, EPI_ISL_1123813, EPI_ISL_1123816, EPI_ISL_1123818, EPI_ISL_1123820, EPI_ISL_1123822, EPI_ISL_1123825, EPI_ISL_1123827, EPI_ISL_1123829, EPI_ISL_1123831, EPI_ISL_1123833, EPI_ISL_1123835, EPI_ISL_1123837, EPI_ISL_1123840, EPI_ISL_1123842, EPI_ISL_1123844, EPI_ISL_1123847, EPI_ISL_1123849, EPI_ISL_1123851, EPI_ISL_1123853, EPI_ISL_1123855, EPI_ISL_1123858, EPI_ISL_1123860, EPI_ISL_1123862, EPI_ISL_1123865, EPI_ISL_1123867, EPI_ISL_1123869, EPI_ISL_1123871, EPI_ISL_1123874, EPI_ISL_1123876, EPI_ISL_1123878, EPI_ISL_1123880, EPI_ISL_1123883, EPI_ISL_1123885, EPI_ISL_1123888, EPI_ISL_1123890, EPI_ISL_1123892, EPI_ISL_1123894, EPI_ISL_1123896, EPI_ISL_1123899, EPI_ISL_1123901, EPI_ISL_1123903, EPI_ISL_1123906, EPI_ISL_1123908, EPI_ISL_1123910, EPI_ISL_1123912, EPI_ISL_1123914, EPI_ISL_1123917, EPI_ISL_1123919, EPI_ISL_1123921, EPI_ISL_1123923, EPI_ISL_1123925, EPI_ISL_1123928, EPI_ISL_1123930, EPI_ISL_1123932, EPI_ISL_1123934, EPI_ISL_1123936, EPI_ISL_1123939, EPI_ISL_1123941, EPI_ISL_1123943, EPI_ISL_1123945, EPI_ISL_1123947, EPI_ISL_1123950, EPI_ISL_1123952, EPI_ISL_1123954, EPI_ISL_1123956, EPI_ISL_1123959, EPI_ISL_1123961, EPI_ISL_1123963, EPI_ISL_1123966, EPI_ISL_1123968, EPI_ISL_1123970, EPI_ISL_1123973, EPI_ISL_1123975, EPI_ISL_1123977, EPI_ISL_1123979, EPI_ISL_1123982, EPI_ISL_1123984, EPI_ISL_1123986, EPI_ISL_1123988, EPI_ISL_1123990, EPI_ISL_1123992, EPI_ISL_1123995, EPI_ISL_1123997, EPI_ISL_1124000, EPI_ISL_1124002, EPI_ISL_1124004, EPI_ISL_1124006, EPI_ISL_1124008, EPI_ISL_1124011, EPI_ISL_1124013, EPI_ISL_1124015, EPI_ISL_1124017, EPI_ISL_1124020, EPI_ISL_1124022, EPI_ISL_1124024, EPI_ISL_1124027, EPI_ISL_1124029, EPI_ISL_1124033, EPI_ISL_1124035, EPI_ISL_1124038, EPI_ISL_1124040, EPI_ISL_1124042, EPI_ISL_1124044, EPI_ISL_1124046, EPI_ISL_1124049, EPI_ISL_1124051, EPI_ISL_1124053, EPI_ISL_1124055, EPI_ISL_1124058, EPI_ISL_1124060, EPI_ISL_1124062, EPI_ISL_1124065, EPI_ISL_1124067, EPI_ISL_1124069, EPI_ISL_1124071, EPI_ISL_1124074, EPI_ISL_1124076, EPI_ISL_1124079, EPI_ISL_1124081, EPI_ISL_1124083, EPI_ISL_1124085, EPI_ISL_1124089, EPI_ISL_1124091, EPI_ISL_1124093, EPI_ISL_1124096, EPI_ISL_1124098, EPI_ISL_1124100, EPI_ISL_1124103, EPI_ISL_1124105, EPI_ISL_1124107, EPI_ISL_1124110, EPI_ISL_1124112, EPI_ISL_1124114, EPI_ISL_1124116, EPI_ISL_1124119, EPI_ISL_1124121, EPI_ISL_1124123, EPI_ISL_1124125, EPI_ISL_1124128, EPI_ISL_1124130, EPI_ISL_1124132, EPI_ISL_1124134, EPI_ISL_1124136, EPI_ISL_1124139, EPI_ISL_1124141, EPI_ISL_1124143, EPI_ISL_1124145, EPI_ISL_1124147, EPI_ISL_1124150, EPI_ISL_1124152, EPI_ISL_1124154, EPI_ISL_1124156, EPI_ISL_1124158, EPI_ISL_1124160, EPI_ISL_1124163, EPI_ISL_1124165, EPI_ISL_1124167, EPI_ISL_1124169, EPI_ISL_1124171, EPI_ISL_1124173, EPI_ISL_1124176, EPI_ISL_1124178, EPI_ISL_1124180, EPI_ISL_1124183, EPI_ISL_1124185, EPI_ISL_1124187, EPI_ISL_1124189, EPI_ISL_1124191, EPI_ISL_1124193, EPI_ISL_1124195, EPI_ISL_1124198, EPI_ISL_1124200, EPI_ISL_1124202, EPI_ISL_1124204, EPI_ISL_1124206, EPI_ISL_1124209, EPI_ISL_1124211, EPI_ISL_1124214, EPI_ISL_1124216, EPI_ISL_1124218, EPI_ISL_1124220, EPI_ISL_1124223, EPI_ISL_1124225, EPI_ISL_1124228, EPI_ISL_1124230, EPI_ISL_1124232, EPI_ISL_1124234, EPI_ISL_1124237, EPI_ISL_1124239, EPI_ISL_1124241, EPI_ISL_1124243, EPI_ISL_1124245, EPI_ISL_1124247, EPI_ISL_1124249, EPI_ISL_1124251, EPI_ISL_1124254, EPI_ISL_1124256, EPI_ISL_1124258, EPI_ISL_1124260, EPI_ISL_1124263, EPI_ISL_1124265, EPI_ISL_1124267, EPI_ISL_1124269, EPI_ISL_1124272, EPI_ISL_1124274, EPI_ISL_1124276, EPI_ISL_1124278, EPI_ISL_1124280, EPI_ISL_1124282, EPI_ISL_1124284, EPI_ISL_1124286, EPI_ISL_1124289 to 1124290, EPI_ISL_1124293 to 1124294, EPI_ISL_1124297, EPI_ISL_1124299, EPI_ISL_1124301, EPI_ISL_1124303, EPI_ISL_1124305, EPI_ISL_1124307, EPI_ISL_1124309, EPI_ISL_1124311 to 1124312, EPI_ISL_1124315, EPI_ISL_1124317, EPI_ISL_1124319, EPI_ISL_1124322, EPI_ISL_1124324, EPI_ISL_1124326, EPI_ISL_1124329, EPI_ISL_1124331, EPI_ISL_1124334, EPI_ISL_1124336, EPI_ISL_1124338, EPI_ISL_1124340, EPI_ISL_1124343, EPI_ISL_1124345, EPI_ISL_1124347, EPI_ISL_1124350, EPI_ISL_1124352, EPI_ISL_1124354, EPI_ISL_1124357, EPI_ISL_1124359, EPI_ISL_1124361, EPI_ISL_1124363, EPI_ISL_1124366, EPI_ISL_1124368, EPI_ISL_1124370, EPI_ISL_1124373, EPI_ISL_1124375, EPI_ISL_1124377, EPI_ISL_1124379, EPI_ISL_1124382, EPI_ISL_1124384, EPI_ISL_1124386, EPI_ISL_1124388, EPI_ISL_1124390, EPI_ISL_1124393, EPI_ISL_1124395, EPI_ISL_1124397, EPI_ISL_1124399, EPI_ISL_1124402, EPI_ISL_1124404, EPI_ISL_1124407, EPI_ISL_1124409, EPI_ISL_1124412, EPI_ISL_1124414, EPI_ISL_1124416, EPI_ISL_1124418, EPI_ISL_1124420, EPI_ISL_1124422, EPI_ISL_1124425, EPI_ISL_1124427, EPI_ISL_1124429, EPI_ISL_1124432, EPI_ISL_1124434, EPI_ISL_1124436, EPI_ISL_1124438, EPI_ISL_1124441, EPI_ISL_1124443, EPI_ISL_1124445, EPI_ISL_1124447, EPI_ISL_1124449, EPI_ISL_1124452, EPI_ISL_1124454, EPI_ISL_1124456, EPI_ISL_1124458, EPI_ISL_1124461, EPI_ISL_1124463, EPI_ISL_1124466, EPI_ISL_1124468, EPI_ISL_1124470, EPI_ISL_1124473, EPI_ISL_1124475, EPI_ISL_1124477, EPI_ISL_1124479, EPI_ISL_1124482, EPI_ISL_1124484, EPI_ISL_1124486, EPI_ISL_1124488, EPI_ISL_1124491, EPI_ISL_1124493, EPI_ISL_1124495, EPI_ISL_1124497, EPI_ISL_1124499, EPI_ISL_1124502, EPI_ISL_1124504, EPI_ISL_1124506, EPI_ISL_1124509, EPI_ISL_1124511, EPI_ISL_1124513, EPI_ISL_1124515, EPI_ISL_1124517, EPI_ISL_1124520, EPI_ISL_1124522, EPI_ISL_1124524, EPI_ISL_1124526, EPI_ISL_1124529, EPI_ISL_1124531, EPI_ISL_1124533, EPI_ISL_1124535, EPI_ISL_1124538, EPI_ISL_1124540, EPI_ISL_1124542, EPI_ISL_1124545, EPI_ISL_1124547, EPI_ISL_1124549, EPI_ISL_1124551, EPI_ISL_1124554, EPI_ISL_1124556, EPI_ISL_1124559, EPI_ISL_1124561, EPI_ISL_1124563, EPI_ISL_1124565, EPI_ISL_1124568, EPI_ISL_1124570, EPI_ISL_1124572, EPI_ISL_1124574, EPI_ISL_1124577, EPI_ISL_1124579, EPI_ISL_1124581, EPI_ISL_1124583, EPI_ISL_1124585, EPI_ISL_1124587, EPI_ISL_1124589, EPI_ISL_1124591, EPI_ISL_1124594, EPI_ISL_1124596, EPI_ISL_1124598, EPI_ISL_1124600, EPI_ISL_1124603, EPI_ISL_1124605, EPI_ISL_1124607, EPI_ISL_1124609, EPI_ISL_1124611, EPI_ISL_1124613, EPI_ISL_1124616, EPI_ISL_1124618, EPI_ISL_1124620, EPI_ISL_1124627, EPI_ISL_1124629, EPI_ISL_1124631, EPI_ISL_1124633, EPI_ISL_1124635, EPI_ISL_1124638, EPI_ISL_1124640, EPI_ISL_1124643, EPI_ISL_1124645, EPI_ISL_1124647, EPI_ISL_1124649, EPI_ISL_1124652, EPI_ISL_1124654, EPI_ISL_1124656, EPI_ISL_1124658, EPI_ISL_1124660, EPI_ISL_1124663, EPI_ISL_1124665, EPI_ISL_1124667, EPI_ISL_1124670, EPI_ISL_1124672, EPI_ISL_1124674, EPI_ISL_1124676, EPI_ISL_1124678, EPI_ISL_1124681, EPI_ISL_1124684, EPI_ISL_1124687, EPI_ISL_1124689, EPI_ISL_1124691, EPI_ISL_1124693, EPI_ISL_1124696, EPI_ISL_1124698, EPI_ISL_1124700, EPI_ISL_1124703, EPI_ISL_1124705, EPI_ISL_1124707, EPI_ISL_1124709, EPI_ISL_1124712, EPI_ISL_1124714, EPI_ISL_1124716, EPI_ISL_1124718, EPI_ISL_1124720, EPI_ISL_1124723, EPI_ISL_1124725, EPI_ISL_1124727, EPI_ISL_1124729, EPI_ISL_1124732, EPI_ISL_1124734, EPI_ISL_1124736, EPI_ISL_1124739, EPI_ISL_1124741, EPI_ISL_1124743, EPI_ISL_1124746, EPI_ISL_1124748, EPI_ISL_1124750, EPI_ISL_1124752, EPI_ISL_1124754, EPI_ISL_1124756, EPI_ISL_1124758, EPI_ISL_1124761, EPI_ISL_1124763, EPI_ISL_1124765, EPI_ISL_1124767, EPI_ISL_1124769, EPI_ISL_1124771, EPI_ISL_1124774, EPI_ISL_1124776, EPI_ISL_1124778, EPI_ISL_1124780, EPI_ISL_1124783, EPI_ISL_1124785, EPI_ISL_1124787, EPI_ISL_1124790, EPI_ISL_1124792, EPI_ISL_1124794, EPI_ISL_1124796, EPI_ISL_1124799, EPI_ISL_1124801, EPI_ISL_1124803, EPI_ISL_1124805, EPI_ISL_1124808, EPI_ISL_1124810, EPI_ISL_1124812, EPI_ISL_1124815, EPI_ISL_1124817, EPI_ISL_1124819, EPI_ISL_1124821, EPI_ISL_1124824, EPI_ISL_1124826, EPI_ISL_1124829, EPI_ISL_1124831, EPI_ISL_1124833, EPI_ISL_1124836, EPI_ISL_1124838, EPI_ISL_1124840, EPI_ISL_1124843, EPI_ISL_1124845, EPI_ISL_1124847, EPI_ISL_1124850, EPI_ISL_1124852, EPI_ISL_1124854, EPI_ISL_1124856, EPI_ISL_1124858, EPI_ISL_1124860, EPI_ISL_1124862, EPI_ISL_1124865, EPI_ISL_1124867, EPI_ISL_1124869, EPI_ISL_1124871, EPI_ISL_1124874, EPI_ISL_1124876, EPI_ISL_1124879, EPI_ISL_1124881, EPI_ISL_1124883, EPI_ISL_1124885, EPI_ISL_1124887, EPI_ISL_1124890, EPI_ISL_1124892, EPI_ISL_1124894, EPI_ISL_1124896, EPI_ISL_1124898, EPI_ISL_1124900, EPI_ISL_1124903, EPI_ISL_1124905, EPI_ISL_1124907, EPI_ISL_1124909, EPI_ISL_1124912, EPI_ISL_1124914, EPI_ISL_1124916, EPI_ISL_1124918, EPI_ISL_1124920, EPI_ISL_1124923, EPI_ISL_1124925, EPI_ISL_1124927, EPI_ISL_1124929, EPI_ISL_1124932, EPI_ISL_1124934, EPI_ISL_1124936, EPI_ISL_1124938, EPI_ISL_1124941, EPI_ISL_1124943, EPI_ISL_1124945, EPI_ISL_1124948, EPI_ISL_1124950, EPI_ISL_1124953, EPI_ISL_1124955, EPI_ISL_1124957, EPI_ISL_1124959, EPI_ISL_1124961, EPI_ISL_1124964, EPI_ISL_1124966, EPI_ISL_1124968, EPI_ISL_1124971, EPI_ISL_1124973, EPI_ISL_1124975, EPI_ISL_1124977, EPI_ISL_1124980, EPI_ISL_1124982, EPI_ISL_1124984, EPI_ISL_1124986, EPI_ISL_1124989, EPI_ISL_1124991, EPI_ISL_1124993, EPI_ISL_1124995, EPI_ISL_1124997, EPI_ISL_1124999, EPI_ISL_1125002, EPI_ISL_1125004, EPI_ISL_1125006, EPI_ISL_1125008, EPI_ISL_1125011, EPI_ISL_1125013, EPI_ISL_1125015, EPI_ISL_1125018, EPI_ISL_1125020, EPI_ISL_1125022, EPI_ISL_1125024, EPI_ISL_1125026, EPI_ISL_1125028, EPI_ISL_1125031, EPI_ISL_1125033, EPI_ISL_1125035, EPI_ISL_1125037, EPI_ISL_1125039, EPI_ISL_1125041, EPI_ISL_1125043, EPI_ISL_1125046, EPI_ISL_1125048, EPI_ISL_1125051, EPI_ISL_1125053, EPI_ISL_1125055, EPI_ISL_1125057, EPI_ISL_1125060, EPI_ISL_1125062, EPI_ISL_1125064, EPI_ISL_1125066, EPI_ISL_1125069, EPI_ISL_1125071, EPI_ISL_1125073, EPI_ISL_1125075, EPI_ISL_1125078, EPI_ISL_1125080, EPI_ISL_1125083, EPI_ISL_1125085, EPI_ISL_1125088, EPI_ISL_1125090, EPI_ISL_1125092, EPI_ISL_1125094, EPI_ISL_1125096, EPI_ISL_1125099, EPI_ISL_1125101, EPI_ISL_1125103, EPI_ISL_1125105, EPI_ISL_1125107 to 1125209, EPI_ISL_1125211 to 1125254, EPI_ISL_1125256, EPI_ISL_1125258, EPI_ISL_1125261, EPI_ISL_1125263, EPI_ISL_1125265, EPI_ISL_1125267, EPI_ISL_1125270, EPI_ISL_1125272, EPI_ISL_1125274, EPI_ISL_1125277, EPI_ISL_1125279, EPI_ISL_1125281, EPI_ISL_1125284, EPI_ISL_1125286, EPI_ISL_1125288, EPI_ISL_1125290, EPI_ISL_1125292, EPI_ISL_1125295, EPI_ISL_1125297, EPI_ISL_1125300 to 1125301, EPI_ISL_1125304, EPI_ISL_1125306, EPI_ISL_1125308, EPI_ISL_1125311, EPI_ISL_1125313, EPI_ISL_1125316, EPI_ISL_1125318, EPI_ISL_1125320, EPI_ISL_1125323 to 1125390, EPI_ISL_1125392, EPI_ISL_1125394, EPI_ISL_1125396 to 1125576, EPI_ISL_1125578 to 1125885, EPI_ISL_1125887 to 1127092			
see above	Department of Virus and Microbiological Special Diagnostics, Statens Serum Institut, Copenhagen, Denmark	Aalborg University	Danish Covid-19 Genome Consortium
EPI_ISL_668458 to 671269, EPI_ISL_682353 to 683328, EPI_ISL_711060 to 712059, EPI_ISL_712097 to 712567, EPI_ISL_712569 to 714937, EPI_ISL_747527 to 747614, EPI_ISL_747616 to 748137, EPI_ISL_748146 to 748666, EPI_ISL_748668 to 749035, EPI_ISL_749037 to 749147, EPI_ISL_749156 to 749237, EPI_ISL_749239 to 749473, EPI_ISL_749475 to 749705, EPI_ISL_749707 to 749905, EPI_ISL_749907 to 750107, EPI_ISL_750109 to 750160, EPI_ISL_750180 to 750255, EPI_ISL_750257 to 750429, EPI_ISL_750431 to 750819, EPI_ISL_750821 to 751010, EPI_ISL_751012 to 751183, EPI_ISL_757395 to 759700, EPI_ISL_792708 to 793012, EPI_ISL_793014 to 794013, EPI_ISL_794324 to 794591, EPI_ISL_794829 to 796010, EPI_ISL_815401 to 816134, EPI_ISL_817123 to 818341, EPI_ISL_818343 to 819123, EPI_ISL_843201 to 844200, EPI_ISL_844202 to 845542, EPI_ISL_846775 to 847514			
see above	Department of Virus and Microbiological Special Diagnostics, Statens Serum Institut, Copenhagen, Denmark	Albertsen Lab, Department of Chemistry and Bioscience, Aalborg University, Denmark	Danish Covid-19 Genome Consortium
EPI_ISL_641396 to 641516	Department of Virus and Microbiological Special Diagnostics, Statens Serum Institut, Copenhagen, Denmark	Albertsen lab, Department of Chemistry and Bioscience, Aalborg University, Denmark	Jannik Fonager; Morten Rasmussen; Thomas Bruun Rasmussen
EPI_ISL_429333 to 429590, EPI_ISL_436962 to 437042, EPI_ISL_437627 to 437683, EPI_ISL_444817 to 444968, EPI_ISL_452101 to 452102	Department of Virus and Microbiological Special Diagnostics, Statens Serum Institut, Copenhagen, Denmark, Artillerivej 5, 2300 Copenahgen S	Albertsen lab, Department of Chemistry and Bioscience, Aalborg University, Denmark	Rasmus Kirkegaard
EPI_ISL_613541 to 613544, EPI_ISL_614403 to 614762, EPI_ISL_614764 to 614888, EPI_ISL_615166 to 620405, EPI_ISL_620762 to 622760, EPI_ISL_625684 to 625932, EPI_ISL_625934 to 625959, EPI_ISL_625961 to 625971, EPI_ISL_625974 to 625997, EPI_ISL_625999 to 626009, EPI_ISL_626011 to 626210			
see above	Department of Virus and Microbiological Special Diagnostics, Statens Serum Institut, Denmark	Albertsen lab, Department of Chemistry and Bioscience, Aalborg University, Denmark	Danish Corona Genome Consortia; Danish Covid-19 Genome Consortia
EPI_ISL_415647, EPI_ISL_416140 to 416142	Department of Virus and Microbiological Special diagnostics, Statens Serum Institut, Copenhagen, Denmark.	Statens Serum Institute	Anders Fomsgaard; Maiken Worsoe Rosenstierne; Morten Rasmussen
EPI_ISL_415646, EPI_ISL_415648, EPI_ISL_416143 to 416144, EPI_ISL_416153	Department of Virus and Microbiological Special diagnostics, Statens Serum Institut, Copenhagen, Denmark.	ViFU	Anders Fomsgaard; Maiken Worsoe Rosenstierne; Morten Rasmussen
EPI_ISL_775346 to 775347, EPI_ISL_1251463	Department of medical microbiology, section Aalesund, Aalesund Hospital	Norwegian Institute of Public Health, Department of Virology	Atiya R Ali; Debech Nadia; Engebretsen Serina Beate; Garcia Llorente Ignacio; Hilde Elshaug; Hilde Vollan; Jon Bråte; Kamilla Heddeland Instefjord; Karoline Bragstad; Kathrine Stene-Johansen; Marie Paulsen Madsen; Olav Hungnes; Pedersen Benedikte Nevjen; Rasmus Riis Kopperud
EPI_ISL_413485	Department of microbiology laboratory,Anhui Provincial Center for Disease Control and Prevention	Department of microbiology laboratory,Anhui Provincial Center for Disease Control and Prevention	Bin Su; Jun He; Junling Yu; Qingqing Chen; Weidong Li; Weiwei Li; Yinglu Ge; Yong Sun; Yonglin Shi; Yuan Yuan; Zhirong Liu; Zhuhui Zhang

EPI_ISL_1231311 to 1231313, EPI_ISL_1231316 to 1231327, EPI_ISL_1251271	Depratment of Microbiology, Viral Research and Diagnostic Laboratory	Indian Council of Medical Research-National Institute of Virology, Microbial Containment Complex	Pragya D. Yadav; Tapan Majumdar
EPI_ISL_1176333 to 1176354	Dept of Microbiology, State Level Viral Research and Diagnostic Laboratory, Regional Institute of Medical Sciences	Indian Council of Medical Research-National Institute of Virology, Microbial Containment Complex	Pragya D. Yadav; Sulochna Devi
EPI_ISL_496482	Dept. Infectious, Tropical Diseases & Microbiology, IRCCS Sacro Cuore Don Calabria Hospital	1) Dept. Infectious, Tropical Diseases & Microbiology, IRCCS Sacro Cuore Don Calabria Hospital; 2) Centro Piattaforme Tecnologiche, University of Verona; 3) Dept. Neurosciences, Biomedicine and Movement Sciences, University of Verona.	1) Antonio Mori; 2) Monica Castellucci and Francesca Griggio; 3) Giovanni Malerba; Chiara Piubelli; Elena Pomari; Michela Deiana
EPI_ISL_449789 to 449794, EPI_ISL_590877, EPI_ISL_590953 to 590977, EPI_ISL_591021, EPI_ISL_635120, EPI_ISL_635144, EPI_ISL_635146, EPI_ISL_635166, EPI_ISL_635196, EPI_ISL_668407 to 668408, EPI_ISL_708044 to 708045, EPI_ISL_708081, EPI_ISL_708131 to 708135, EPI_ISL_759988, EPI_ISL_775272, EPI_ISL_775280, EPI_ISL_775314, EPI_ISL_775327 to 775329, EPI_ISL_775353 to 775355, EPI_ISL_775386, EPI_ISL_796658, EPI_ISL_796663, EPI_ISL_796677, EPI_ISL_796698, EPI_ISL_860289, EPI_ISL_906797, EPI_ISL_964961, EPI_ISL_965005 to 965007, EPI_ISL_1034318, EPI_ISL_1040151, EPI_ISL_1040154 to 1040155, EPI_ISL_1118283, EPI_ISL_1118311, EPI_ISL_1118421, EPI_ISL_1118435 to 1118438, EPI_ISL_1118442 to 1118443, EPI_ISL_1118447, EPI_ISL_1118489, EPI_ISL_1118545 to 1118546, EPI_ISL_1118558 to 1118559, EPI_ISL_1118564, EPI_ISL_1118570 to 1118571, EPI_ISL_1192249, EPI_ISL_1251296, EPI_ISL_1251306, EPI_ISL_1251344 to 1251357, EPI_ISL_1251539 to 1251547, EPI_ISL_1251706 to 1251711, EPI_ISL_1314458 to 1314465			
see above	Dept. of Medical Microbiology, Stavanger University Hospital, Helse Stavanger HF	Norwegian Institute of Public Health, Department of Virology	Atiya R Ali; Debech Nadia; Engebretsen Serina Beate; Engebretsen Serina Beate Atiya R Ali; Garcia Llorente Ignacio; Hilde Elshaug; Hilde Vollan; Ignacio Garcia Llorente; Iren Löhr; Jon Bråte; Kamilla Heddeland Instefjord; Karoline Bragstad; Kathrine Stene-Johansen; Marie Paulsen Madsen; Olav Hungnes; Pedersen Benedikte Nevjen; Rasmus Riis Kopperud; Serina B Engebretsen
EPI_ISL_447837	Dept. of Medical Microbiology, Stavanger University Hospital, Helse Stavanger HF,	Norwegian Institute of Public Health, Department of Virology	Hilde Elshaug; Kamilla Heddeland Instefjord; Karoline Bragstad; Kathrine Stene-Johansen; Olav Hungnes; Rasmus Riis Kopperud
EPI_ISL_664101, EPI_ISL_664103, EPI_ISL_664105 to 664106, EPI_ISL_665254 to 665260, EPI_ISL_666568, EPI_ISL_666593 to 666608, EPI_ISL_666610 to 666612, EPI_ISL_666614 to 666615, EPI_ISL_1118090 to 1118092, EPI_ISL_1118099 to 1118114, EPI_ISL_1118116 to 1118136, EPI_ISL_1118142 to 1118157			
see above	Dept. of Microbiology and Infection Control, Akershus University Hospital HF	Dept. of Microbiology and Infection Control, Akershus University Hospital HF	Alexander Hesselberg Løvestad; Hege Vangstein Aamot; Nina Handal; Ole Herman Ambur; Silje Bakken Jørgensen
EPI_ISL_1180182 to 1180185	Dept. of Microbiology, All India Institute of Medical Sciences, Jodhpur	Indian Council of Medical Research-National Institute of Virology, Microbial Containment Complex	Pragya D. Yadav; Vijaylakshmi Nag
EPI_ISL_1098653 to 1098655, EPI_ISL_1098657 to 1098659, EPI_ISL_1098893, EPI_ISL_1098897, EPI_ISL_1103550, EPI_ISL_1103558 to 1103559, EPI_ISL_1103568, EPI_ISL_1103571, EPI_ISL_1109904 to 1109905, EPI_ISL_1110027 to 1110028, EPI_ISL_1110288, EPI_ISL_1110293			
see above	Dept. of Molecular Biology and Genetics, Democritus University of Thrace, Alexandroupolis, Greece	Laboratory of Histology-Embryology, Molecular Carcinogenesis Group, Faculty of Medicine, National and Kapodistrian University of Athens	Gorgoulis V.G.
EPI_ISL_410531 to 410532	Dept. of Pathology, National Institute of Infectious Diseases	Pathogen Genomics Center, National Institute of Infectious Diseases	Harutaka Katano; Hideki Hasegawa; Kazuya Shirato; Makoto Kuroda; Makoto Takeda; Motoi Suzuki; Naganori Nao; Shutoku Matsuyama; Tadaki Suzuki; Takaji Wakita; Tsuyoshi Sekizuka
EPI_ISL_402126	Dept. of Virology III, National Institute of Infectious Diseases	Dept. of Virology III, National Institute of Infectious Diseases	Kazuya Shirato; Makoto Takeda; Naganori Nao; Shutoku Matsuyama
EPI_ISL_408665 to 408667, EPI_ISL_408669	Dept. of Virology III, National Institute of Infectious Diseases	Pathogen Genomics Center, National Institute of Infectious Diseases	Kazuya Shirato; Makoto Kuroda; Makoto Takeda; Naganori Nao; Shutoku Matsuyama; Tsuyoshi Sekizuka
EPI_ISL_774872 to 774975, EPI_ISL_774977 to 775018	Designated Reference Institute for Chemical Measurements (DRICM)	DNA SOLUTION LTD.	Abdul Khaleque; Abu Sufian; Hasan Ul Haider; Jannatun Naima; Kazi Nadim Hasan; MSM Chowdhury; Mala Khan; Mamudul Hasan Razu; Md. Imran Khan; Mizanur Rahman; Mohammad Fazle Alam Rabbi
EPI_ISL_1081796	Detoxicare	Philippine Genome Center	Alethea R. de Guzman; Anna Ong-Lim; Arianne A. Zamora; Asia Louisa U. Chong; Benedict A. Maralit; Candice Francheska B. Tambaoan; Carlo M. Lapid; Celia Carlos; Cynthia P. Saloma; Devon Ray Pacial; Edsel Maurice Salvaña; El King D. Morado; Eva Maria Cutiongco-de la Paz; Francis A. Tablizo; Irish Coleen A. Asin; Jaime C. Montoya; Jan Michael C. Yap; Jo-Hannah S. Llames; John Q. Wong; Joshua Gregor A. Dizon; Juan Antonio R. Magalang; Karol Sophia Agape R. Padilla; Kenneth M. Kim; Kris P. Punayan; Marc Edsel C. Ayes; Marc Jerrone R. Castro; Maria Sofia L. Yangzon; Marissa Alejandria; Razel Nikka M. Hao; Rianna Patricia S. Cruz; Sheila Mae M. Araiza; and Maria Rosario Singh-Vergeire
EPI_ISL_807154 to 807156	Deva County Emergency Hospital	National Institute of Infectious Diseases-Prof. Dr. Matei Bals Molecular Diagnostics Laboratory	Andreea Tudor; Corina Casangiu; Dan Otelea; Leontina Banica; Marius Surleac; Petre Milu; Simona Paraschiv
EPI_ISL_641320 to 641326, EPI_ISL_641330 to 641331, EPI_ISL_641359	Devki Devi Foundation, a unit of Max Healthcare	CSIR Institute of Genomics and Integrative Biology (CSIR-IGIB) / Max	Anurag Agrawal; Bansidhar Tarai; Bharathram Uppili; Mitali Mukerji; Mohammed Faruq; Nishu Tyagi; Pooja Sharma; Poonam Das; Rajesh Pandey#; Samreen Siddiqui; Saruchi Wadhwa; Sujeet Jha; Vinitaa Jha; Vivekanand A
EPI_ISL_459911 to 459952, EPI_ISL_751239 to 751257, EPI_ISL_751259 to 751260, EPI_ISL_751263 to 751267, EPI_ISL_751270 to 751273, EPI_ISL_751275 to 751313, EPI_ISL_751315 to 751317			
see above	Devki Devi Foundation, a unit of Max Healthcare	CSIR-IGIB/Max	Akansha Tyagi; Akshay Kanakan; Anurag Agrawal; Bansidhar Tarai; Bharathram Uppili; Janani Srinivasa Vasudevan; Mitali Mukerji; Mohammed Faruq; Nishu Tyagi; Pooja Sharma; Poonam Das; Rajesh Pandey#; Ranjeet Maurya; Samreen Siddiqui; Saruchi Wadhwa; Sujeet Jha; Uzma Shamim; Vinita Jha; Vivekanand A
EPI_ISL_811151 to 811193	Dharwad	CSIR Institute of Genomics and Integrative Biology	Abhinav Jain; Bani Jolly; Disha Sharma; Dr. Amit Ugargol; Dr. Rgavendra B Nayak; Dr. Shivarudrapp B Bhairappanavar; Dr. Suresh B Arakera; Dr. Vijay A Yenagi; Gyan Ranjan; Mohammed Imran; Mohit Divakar; Paras Sehgal; Rahul Bhoyar; Sridhar Sivasubbu; Vinod Scaria
EPI_ISL_953938, EPI_ISL_954750	Diagen	Diagen	Koliada O
EPI_ISL_1008205, EPI_ISL_1008210 to 1008211	Diagnostic & Research Inst. Of Hygiene, Microbiology and Environmental Medicine, Medical University Graz	Bergthaler laboratory, CeMM Research Center for Molecular Medicine of the Austrian Academy of Sciences	Andreas Bergthaler; Anna Schedl; Bekir Erguner; Benedikt Agerer; Christoph Bock; Jan Laine; Lukas Endler; Maelle Le Moing; Martin Senekowitsch; Michael Schuster; Thomas Penz
EPI_ISL_1164855	Diagnostic Genomics Lab and Functional Genomics Core University of South Carolina	Diagnostic Genomics Lab and Functional Genomics Core, University of South Carolina	Carolyn Banister; Diego Altomare; Hao Ji; Helmut Albrecht; Herr Doktor Professor Genomiklaborsdirektor Universitätkovidenpandemieretter Phillip Buckhaults; Michael Shtutman
EPI_ISL_1254646	Diagnostic Genomics Lab and Functional Genomics Core University of South Carolina	Diagnostic Genomics Lab and Functional Genomics Core, University of South Carolina	Carolyn Banister; Diego Altomare; Hao Ji; Helmut Albrecht; Herr Doktor Professor Genomiklaborsdirektor Universitätkovidenpandemieretter Phillip Buckhaults; Michael Shtutman
EPI_ISL_437197 to 437203, EPI_ISL_437298 to 437303, EPI_ISL_653916	Diagnostic- and Research Institute of Pathology, Medical University of Graz	Diagnostic- and Research Institute of Pathology, Medical University of Graz	Gregor Gorkiewicz; Karl Kashofer; Martin Zacharias; Peter Regitnig
EPI_ISL_763074 to 763075, EPI_ISL_1086034, EPI_ISL_1086037 to 1086043, EPI_ISL_1092725, EPI_ISL_1096121, EPI_ISL_1096135, EPI_ISL_1201894 to 1201896			
see above	Diagnosticos da America - DASA	Instituto Adolfo Lutz, Interdiciplinary Procedures Center, Strategic Laboratory	Caio Vinicius Dias Lopes; Claudia Regina Gonçalves; Claudio Tavares Sacchi; Erica Valessa Ramos Gomes; Karoline Rodrigues Campos
EPI_ISL_1142235 to 1142257, EPI_ISL_1143569 to 1143572, EPI_ISL_1143574 to 1143582, EPI_ISL_1143584 to 1143587, EPI_ISL_1143589 to 1143596, EPI_ISL_1143598 to 1143612, EPI_ISL_1143614 to 1143618, EPI_ISL_1143620 to 1143626, EPI_ISL_1143628 to 1143636, EPI_ISL_1143638, EPI_ISL_1143640 to 1143642, EPI_ISL_1143644 to 1143652, EPI_ISL_1144254, EPI_ISL_1144256, EPI_ISL_1146616 to 1146673, EPI_ISL_1146675 to 1146682, EPI_ISL_1146684 to 1146704, EPI_ISL_1149072 to 1149073, EPI_ISL_1149075 to 1149129, EPI_ISL_1149131 to 1149167, EPI_ISL_1149169 to 1149212, EPI_ISL_1149214 to 1149216, EPI_ISL_1149218 to 1149222, EPI_ISL_1149224 to 1149256, EPI_ISL_1150530 to 1150545, EPI_ISL_1150547 to 1150569, EPI_ISL_1150571 to 1150572, EPI_ISL_1150574 to 1150575, EPI_ISL_1150577 to 1150615, EPI_ISL_1150617 to 1150689, EPI_ISL_1150691 to 1150700, EPI_ISL_1151890 to 1151894, EPI_ISL_1152003 to 1152009, EPI_ISL_1156177 to 1156228, EPI_ISL_1156230 to 1156242, EPI_ISL_1156244 to 1156304, EPI_ISL_1156306 to 1156315, EPI_ISL_1156317 to 1156360, EPI_ISL_1217224, EPI_ISL_1217226, EPI_ISL_1217228 to 1217229, EPI_ISL_1217231 to 1217232, EPI_ISL_1217234, EPI_ISL_1217236 to 1217273, EPI_ISL_1217275 to 1217347, EPI_ISL_1218106 to 1218108, EPI_ISL_1284948 to 1284989, EPI_ISL_1284991 to 1285000, EPI_ISL_1285002 to 1285005, EPI_ISL_1285007 to 1285033, EPI_ISL_1285036 to 1285038, EPI_ISL_1285040 to 1285042			
see above	Diagnosticum - Labor Neukirchen	Robert Koch Institute	
EPI_ISL_1201680, EPI_ISL_1239536, EPI_ISL_1239547, EPI_ISL_1239593 to 1239596, EPI_ISL_1239675, EPI_ISL_1239689, EPI_ISL_1239712, EPI_ISL_1239737 to 1239740, EPI_ISL_1273834 to 1273835			
see above	Diagnostyka	1. National Institute of Public Health - National Institute of Hygiene; 2. Eurofins Genomics Europe Sequencing GmbH	ECDC COVID-19 WGS support team; Eurofins Genomics Europe Sequencing Team; Gierczyki Rafa; Sadkowska-Todys Magorzata; Wokowicz Tomasz; Zacharczuk Katarzyna
EPI_ISL_754236	Diagnósticos da América - DASA	Instituto de Medicina Tropical Universidade de São Paulo	Brazil-UK Centre for Arbovirus Discovery Diagnosis Genomics and Epidemiology (CADDE) Genomic Network - Instituto de Medicina Tropical

EPI_ISL_754237, EPI_ISL_1009675 to 1009678	Diagnósticos da América - DASA	Instituto de Medicina Tropical de Sao Paulo	Brazil-UK Centre for Arbovirus Discovery Diagnosis Genomics and Epidemiology (CADDE) Genomic Network - Instituto de Medicina Tropical
EPI_ISL_1144226 to 1144251, EPI_ISL_1147725 to 1147743, EPI_ISL_1147745 to 1147761, EPI_ISL_1155202 to 1155221, EPI_ISL_1155223 to 1155233, EPI_ISL_1211955 to 1211970, EPI_ISL_1211972 to 1211987, EPI_ISL_1285797 to 1285798, EPI_ISL_1285801 to 1285812, EPI_ISL_1285814 to 1285818, EPI_ISL_1285820 to 1285829, EPI_ISL_1285831 to 1285832, EPI_ISL_1285834, EPI_ISL_1285836 to 1285839			
see above	Dianovis GmbH Greiz	Robert Koch Institute	
EPI_ISL_413573	Dienst Gezondheid & Jeugd Zuid-Holland Zuid	Erasmus Medical Center	Anne van der Linden; Annemiek van der Eijk; Aura Timen; Bas Oude Munnink; Claudia Schapendonk; Corien Swaan; Corine GeurtsvanKessel; David Nieuwenhuijse; Irina Chestakova; Jeroen van Kampen; Jolanda Voermans; Madelief Mollers; Manon Haverkate; Marion Koopmans; Mark Pronk; Mart Stein; Pascal Lexmond; Reina Sikkema; Richard Molenkamp; Sandra Kengne Kamga Mobou; on behalf of the Dutch national COVID-19 response team.
EPI_ISL_751194 to 751200	Dienststelle Gesundheit und Sport Kanton Luzern	Institute of Medical Virology, University of Zurich	Alexandra Trkola; Claudia Schmutz; Eva Spieler; Gabriela Ziltener; Jürg Böni; Kevin Steiner; Maryam Zaheri; Michael Huber; Stefan Schmutz; Verena Kufner
EPI_ISL_1152566	Dietrich-Bonhoeffer-KlinikumInstitut für Laboratoriumsdiagnostik Mikrobiologie und Transfusionsmedizin	Robert Koch Institute	
EPI_ISL_452234, EPI_ISL_475512, EPI_ISL_475562 to 475563	Din Klinik	The Public Health Agency of Sweden	Anna Risberg; Anna-Malin Linde; Helene Warnborg; Karin Tegmark-Wisell; Maria Lind Karlberg; Mattias Haukland; Mia Brytting; Olov Svartstrom; Oskar Karlsson Lindsjo; Reza Advani; Sandra Broddesson; Theresa Enkirch
EPI_ISL_528753	Dinkes Kota Bogor	School of Life Sciences and Technology & School of Pharmacy-Institut Teknologi Bandung; Molecular Genetics Laboratory-Faculty of Medicine-Universitas Padjadjaran; Laboratorium Kesehatan Provinsi Jawa Barat	Agung Eru Wibowo; Azzania Fibriani; Catur Riani; Cut Nur Cinthia Alamanda; Davin H. E. Setiamarga; Ema Rahmawati; Hammam Riza; Hesti Lina Wiraswati; Husna Nugrahapraja; Irvan Faizal; Lia Faridah; Marselina Irasonia Tan; Rifky Waluyajati Rachman; Rizki Mardian; Ryan Bayusantika Ristandi; Savira Ekawardhani; Sony Solistia Wirawan; Tarwadi; Yunia Sribudiani
EPI_ISL_528748	Dinkes Provinsi Jawa Barat	School of Life Sciences and Technology & School of Pharmacy-Institut Teknologi Bandung; Molecular Genetics Laboratory-Faculty of Medicine-Universitas Padjadjaran; Laboratorium Kesehatan Provinsi Jawa Barat	Adelina Khristiani Rahayu; Agung Eru Wibowo; Azzania Fibriani; Catur Riani; Cut Nur Cinthia Alamanda; Ema Rahmawati; Gusti Ayu Prani Pradani; Hammam Riza; Hesti Lina Wiraswati; Husna Nugrahapraja; Irvan Faizal; Lia Faridah; Marselina Irasonia Tan; Rifky Waluyajati Rachman; Ryan Bayusantika Ristandi; Savira Ekawardhani; Sony Solistia Wirawan; Tarwadi; Yunia Sribudiani
EPI_ISL_467374	Dinkes Samarinda	Eijkman Institute for Molecular Biology, Ministry of Research and Technology/National Agency for Research and Innovation	Amin Soebandrio; David H Muljono; Edison Johar; Frilasita A Yudhaputri; Hidayat Trimarsanto; Khin Saw Myint; Safarina G Malik
EPI_ISL_754241	Dinkes Tasikmalaya	"School of Life Sciences and Technology & School of Pharmacy-Institut Teknologi Bandung; Molecular Genetics Laboratory-Faculty of Medicine-Universitas Padjadjaran; Laboratorium Kesehatan Provinsi Jawa Barat"	Agung Eru Wibowo; Azzania Fibriani; Catur Riani; Cut Nur Cinthia Alamanda; Ema Rahmawati; Hammam Riza; Hesti Lina Wiraswati; Husna Nugrahapraja; Irvan Faizal; Karimatu Khoirunnisa; Lia Faridah; Marselina Irasonia Tan; Miftahul Faridl; Rifky Waluyajati Rachman; Ryan Bayusantika Ristandi; Savira Ekawardhani; Soni Solistia Wirawan; Tarwadi; Yunia Sribudiani
EPI_ISL_1020197, EPI_ISL_1117591	Dinkes, Bogor, West Java	Biosafety Level-3 Laboratory, Indonesian Institute of Sciences (LIPI)	Ade Andriani; Anik Budhi Dharmayanthi; Asep M. Ridwanuloh; Eko Wahyu Putro; Gita Syahputra; Hariyatun; Inswasti Cahyani; Matthew W. Loose; Mohammad Ilyas; Susanti; Syam Budi Iryanto; Wien Kusharyoto
EPI_ISL_961730, EPI_ISL_961734, EPI_ISL_961747	Dipartimento Prevenzione Avezzano-Servizio Igiene epidemiologia Sanità Pubblica	Istituto Zooprofilattico Sperimentale dell'Abruzzo e Molise "G. Caporale"	Ancora M; Calistri P; Cammà C; Curini V; Di Domenico M; Di Pasquale A; Lorusso A; Mangone I; Marcacci M; Puglia I; Rinaldi A; Savini G; Scialabba S
EPI_ISL_591326 to 591340	Dipartimento di Biotecnologie Mediche, University of Siena	Dipartimento di Biotecnologie Mediche, University of Siena	Anichini, G.; COVID; Cusi; Gandolfo, C.; M.G.; Pinzauti, D.; Pozzi, G.; Santoro, F.
EPI_ISL_414512	Dipartimento di Medicina e Chirurgia, University of Insubria and Ospedale di Circolo e Fondazione Macchi	Dipartimento di Medicina e Chirurgia, University of Insubria and Ospedale di Circolo e Fondazione Macchi	A. and Rossi, A.; A.P.; Baj; Genoni
EPI_ISL_983327	Dipartimento di Prevenzione ASL Roma 4	INMI Lazzaro Spallanzani IRCCS	A Di Caro; B Bartolini; CEM Gruber; E Giombini; F Messina; M Rueca; MR Capobianchi; O Butera; S Ursino
EPI_ISL_722855 to 722858, EPI_ISL_722872, EPI_ISL_722896 to 722897, EPI_ISL_722899, EPI_ISL_722912, EPI_ISL_1112236 to 1112291, EPI_ISL_1116471 to 1116472, EPI_ISL_1116475			
see above	Dipartimento di Scienze Biomediche e Oncologia Umana - Azienda Ospedaliero Universitaria Consorziale Policlinico	Istituto Zooprofilattico Sperimentale della Puglia e della Basilicata	Bianco A.; Capozzi L.; Chironna M.; Del Sambro L.; Giannico A.; Loconsole D.; Parisi A.; Ridolfi D.; Sallustio A.; Simone D.
EPI_ISL_906529	Direccion de partamental de salud del Amazonas	Instituto Nacional de Salud- Dirección de Investigación en Salud Pública, Universidad de los Andes- Applied genomics research group, Vicerrectoria de Investigación y Creación, Universidad de los Andes- Systems and Computing Engineering Department	Carlos Franco-Muñoz; Diego A. Álvarez-Díaz; Diego Andrés Prada; Gerardo Santamaría Jorge Duitama; Héctor Alejandro Ruiz-Moreno; Jhonnatan Reales-González; Jorge Ivan Diaz; Julian Naizaque; Katherine Laiton-Donato; Laura Natalia Gonzalez; Magdalena Wiesner; Marcela Mercado-Reyes; Maria T. Herrera-Sepúlveda; Martha Lucia Ospina Martinez; Mauricio Pacheco-Montealegre; Sheryll Corchuelo; Silvia Restrepo-Restrepo
EPI_ISL_902736	Dirección Departamental de Salud de Pública Leticia	Instituto Nacional de Salud- Dirección de Investigación en Salud Pública, Universidad de los Andes- Applied genomics research group, Vicerrectoria de Investigación y Creación, Universidad de los Andes- Systems and Computing Engineering Department	Carlos Franco-Muñoz; Diego A. Álvarez-Díaz; Diego Andrés Prada; Jorge Duitama; Jorge Ivan Diaz; Katherine Laiton-Donato; Laura Natalia Gonzalez; Magdalena Wiesner; Marcela Mercado-Reyes; Maria T. Herrera-Sepúlveda; Martha Lucia Ospina Martinez; Mauricio Pacheco-Montealegre; Silvia Restrepo-Restrepo
EPI_ISL_845621 to 845625	Dirección de Sanidad Ejército	Instituto Nacional de Salud - Dirección de Investigación en Salud Pública	Carlos Franco-Muñoz; Diego A. Álvarez-Díaz; Diego Andrés Prada; Gerardo Santamaría; Jonathan Reales; Julian Naizaque; Katherine Laiton-Donato; Magdalena Wiesner; Marcela Mercado-Reyes; Maria T. Herrera-Sepúlveda; Martha Lucia Ospina Martinez; Mauricio Pacheco-Montealegre; Paola Muñoz-Laiton; Sheryll Corchuelo
EPI_ISL_1137618, EPI_ISL_1303371 to 1303373	Dirección de Sanidad Policia Nacional	Instituto Nacional de Salud- Dirección de Investigación en Salud Pública	Carlos Franco-Muñoz; Carmen Osorio; Diana Malo; Diego A. Álvarez-Díaz; Diego Andrés Prada; Gerardo Santamaría; Hector Alejandro Ruiz-Moreno; Jhonnatan Reales-González; Juan Camilo Martinez; Julian Naizaque; Katherine Laiton-Donato; Lisseth Pardo; Magdalena Wiesner; Marcela Mercado-Reyes; Marcela Mercado-Reyes.; Maria T. Herrera-Sepúlveda; Marta Lopez Blanco; Martha Lucia Ospina Martinez; Martha Lucia Ospina Martínez; Sergio Gomez; Sheryll Corchuelo; Ángela Alarcon Cruz
EPI_ISL_424353 to 424354, EPI_ISL_424983, EPI_ISL_424987, EPI_ISL_454412 to 454415, EPI_ISL_454796 to 454829, EPI_ISL_458001 to 458007, EPI_ISL_458291, EPI_ISL_458294 to 458297, EPI_ISL_524428 to 524432			
see above	Dirk Dittmer	Dirk Dittmer	A.B.; A.G.; Aubrey; B.G.; Bailey; C.P.; Caro-Vegas; Caro-Vegas, C.; Dittmer, D.; Eason; F. and Vahrson, W.; J.T.; Juarez, A.; L.J.; Landis; M.B.; McNamara; Miller; Moorad, R.; Pluta; R.P.; Seltzer; T.A.; Thompson, C.; Vahrson; Vahrson, W.; Villamor; Villamor, F.; W. and Villamor, F.
EPI_ISL_504185, EPI_ISL_515466	Discovery DNA	Discovery DNA	Alice Li; Aneal Khan; Desmond Koo; Dustin Hittel; Leo Dimnik; Marina Kerr
EPI_ISL_640044	District 6 CDC wc DSI	NHLS/UCT	Arash Iranzadeh; Bruna Galvao; Carolyn Williamson; Deelan Doolabh; Diana Hardie; Innocent Mudau; Kruger Marais; Lynn Tyers; Marvin Hsiao; Stephen Korsman
EPI_ISL_436137 to 436141, EPI_ISL_436156 to 436157	District Surveillance Unit	Department of Neurovirology, National Institute of Mental Health and Neuroscience (NIMHANS)	Anita Desai; Chitra Pattabiraman; Harsha PK; Manjunatha Venkataswamy; Ravi Vasanthapuram; Risha Rasheed; Shafeeq S Hameed; Vijayalakshmi Reddy
EPI_ISL_428479 to 428487, EPI_ISL_486382	District Surveillence Unit	Department of Neurovirology, National Institute of Mental Health and Neuroscience (NIMHANS)	Anita Desai; Chitra Pattabiraman; Harsha PK; Manjunatha Venkataswamy; Ravi Vasanthapuram; Risha Rasheed; Shafeeq S Hameed; Vijayalakshmi Reddy
EPI_ISL_534312	Distrito Sanitario Sul	Instituto Adolfo Lutz, Interdiciplinary Procedures Center, Strategic Laboratory	Claudia Regina Gonçalves; Claudio Tavares Sacchi; Erica Valessa Ramos Gomes
EPI_ISL_583499	Distrito Sanitario Sul Campinas	Instituto Adolfo Lutz, Interdiciplinary Procedures Center, Strategic Laboratory	Claudia Regina Gonçalves; Claudio Tavares Sacchi; Erica Valessa Ramos Gomes; Karoline Rodrigues Campos
EPI_ISL_738139, EPI_ISL_738141 to 738142, EPI_ISL_747245 to 747281, EPI_ISL_747284 to 747421, EPI_ISL_747425 to 747458, EPI_ISL_759991 to 760030, EPI_ISL_760060 to 760245, EPI_ISL_762992, EPI_ISL_833249, EPI_ISL_850181 to 850505, EPI_ISL_850653 to 850662, EPI_ISL_955939 to 955981, EPI_ISL_959476 to 959537, EPI_ISL_994668 to 994738, EPI_ISL_994741 to 994801, EPI_ISL_995192 to 995293, EPI_ISL_995308 to 995330, EPI_ISL_995765 to 995838, EPI_ISL_1007643 to 1007657, EPI_ISL_1063537 to 1063626, EPI_ISL_1063628 to 1063773, EPI_ISL_1138900 to 1138968, EPI_ISL_1138970 to 1138982, EPI_ISL_1165001 to 1165065, EPI_ISL_1209021, EPI_ISL_1209408 to 1209452, EPI_ISL_1228588 to 1228664, EPI_ISL_1252366 to 1252445			

see above	Division of Emerging Infectious Diseases, Bureau of Infectious Diseases Diagnosis Control, Korea Disease Control and Prevention Agency	Division of Emerging Infectious Diseases, Bureau of Infectious Diseases Diagnosis Control, Korea Disease Control and Prevention Agency	Ae Kyung Park; Chae Young Lee; Chaeyoung Lee; Eun-Jin Kim; Heui Man Kim; Il-Hwan Kim; Jeong-Ah Kimg; Jeong-Min Kim; Jin Sun No; Namjoo Lee; Sang Hee Woo
EPI_ISL_485603	Division of Infectious Disease	Steininger Lab	Christoph Steininger; Ingeborg Klymiuk; Jakob Thannesberger; Lorenz Schubert; Nicolas Rascovan; Oliver Robak
EPI_ISL_508864 to 508870, EPI_ISL_509222	Division of Infectious Diseases and Hospital Epidemiology, University Hospital Zürich	Institute of Medical Virology, University of Zurich	Aline Wolfensberger; Dana Weissberg; Hugo Sax; Irene A. Abela; Jürg Böni; Maryam Zaheri; Michael Huber; Peter W. Schreiber; Silvana K. Rampini; Verena Kufner
EPI_ISL_413513, EPI_ISL_413515	Division of Infectious Diseases, Department of Internal Medicine, Korea University College of Medicine	Department of Microbiology, Institute for Viral Diseases, College of Medicine, Korea University	Changmin Kang; Cui Chunguang; Gee Eun Lee; Hee Jin Cheong; Heedo Park; Jeonghun Kim; Ji Yun Noh; Jin Gu Yoon; Jin Il Kim; Joon Young Song; Joon-Yong Bae; Jungmin Lee; Juyoung Cho; Kyeong-ryeol Shin; Man-Seong Park; Woo Joo Kim
EPI_ISL_660109 to 660110	Division of Infectious Diseases, Department of Internal Medicine, Yonsei University College of Medicine	Department of biotechnology, Yonsei University	Jae sun Yoon; Jin Young Ahn; Jun Yong Choi*; Jung Ho Kim; Minwoo Kim; Youn-Jung Lee; and Jong-Won Oh*
EPI_ISL_413022 to 413024	Division of Infectious Diseases, University Hospital Zurich	Institute of Medical Virology, University of Zurich	Alexandra Trkola; Andrea Zbinden; Fiona Steiner; Gabriela Ziltener; Jon Huder; Jürg Böni; Maryam Zaheri; Michael Huber; Patrick Redli; Riccarda Capaul; Roberto Speck; Stefan Schmutz; Verena Kufner
EPI_ISL_524475, EPI_ISL_524477, EPI_ISL_524483, EPI_ISL_524485, EPI_ISL_939660 to 939672	Division of Infectious Diseases, University Hospital Zürich	Institute of Medical Virology, University of Zurich	Alexandra Trkola; Andrea Zbinden; Annette Audigé; Cyril Shah; Fiona Steiner; Gabriela Ziltener; Guido Bloemberg; Jon Huder; Jürg Böni; Kevin Steiner; Maria Grünberg; Maryam Zaheri; Michael Huber; Patrick Redli; Riccarda Capaul; Stefan Schmutz; Verena Kufner
EPI_ISL_812956 to 812965	Division of Pathogen Resource Management, Korea National Institute of Health, Korea Disease Control and Prevention Agency	Division of Pathogen Resource Management, Korea National Institute of Health, Korea Disease Control and Prevention Agency	Choi; Choi, C.; E.-J.; J.-M.; Kim; M.-r.; S.T.; S.Y.; Y.S.; Yun
EPI_ISL_1233936 to 1233937	Division of Viral Diseases, CDC Pathogen Discovery Team	Division of Viral Diseases, CDC Pathogen Discovery Team	B.L.; Batra, D.; Burroughs, M.; C.R.; Cook; J.S.; Knipe, K.; Lee; Li, Y.; Marine, R.; Montmayeur, A.; Nobles, S.; P.W.; Paden; Padilla, J.; Queen, K.; Rambo-Martin; Rowe, L.; Sheth, M.; Tao, Y.; Tong, S.; Uehara, A.; Wang, H.; Zhang, J.
EPI_ISL_412869 to 412873, EPI_ISL_425117 to 425118, EPI_ISL_426163 to 426164, EPI_ISL_426166 to 426169, EPI_ISL_426171, EPI_ISL_426173, EPI_ISL_426180 to 426183, EPI_ISL_426187, EPI_ISL_427809 to 427813, EPI_ISL_471425 to 471426, EPI_ISL_471438 to 471455, EPI_ISL_481370 to 481379, EPI_ISL_497951, EPI_ISL_497953 to 498053, EPI_ISL_506947 to 506995, EPI_ISL_510547 to 510688, EPI_ISL_514756 to 515054, EPI_ISL_522454, EPI_ISL_522465 to 522466, EPI_ISL_522474 to 522475, EPI_ISL_522477 to 522478, EPI_ISL_522484, EPI_ISL_522486 to 522490, EPI_ISL_522498 to 522501, EPI_ISL_522503 to 522504, EPI_ISL_522506 to 522507, EPI_ISL_522509, EPI_ISL_522511 to 522514, EPI_ISL_522517, EPI_ISL_522519 to 522544, EPI_ISL_526700 to 526729, EPI_ISL_526732 to 526733			
see above	Division of Viral Diseases, Center for Laboratory Control of Infectious Diseases, Korea Centers for Diseases Control and Prevention	Division of Viral Diseases, Center for Laboratory Control of Infectious Diseases, Korea Centers for Diseases Control and Prevention	Daesang Lee; Dong Hyun Song; Heui Man Kim; Hye-Jun Jo; Jeong-Min Kim; Jun-Sub Kim; Junhyeong Jang; Mi-Seon Kim; Myung Guk Han; Namjoo Lee; Sang Hee Woo; Sehee Park; Seong Tae Jeong; Yoon-Seok Chung
EPI_ISL_722209	Dom Zdravlja Sarajevo	Alea Genetic Center	Konjhodzic R.; Pecar D.; Salihefendic L.
EPI_ISL_513357, EPI_ISL_513389, EPI_ISL_699650	Douglas Hanly Moir	NSW Health Pathology - Institute of Clinical Pathology and Medical Research; Westmead Hospital; University of Sydney	CIDM-PH et al.
EPI_ISL_451601, EPI_ISL_455051 to 455053, EPI_ISL_455065	Douglas Hanly Moir Pathology	NSW Health Pathology - Institute of Clinical Pathology and Medical Research; Westmead Hospital; University of Sydney	CIDM-PH et al.
EPI_ISL_498773, EPI_ISL_526153, EPI_ISL_526213 to 526214, EPI_ISL_545027, EPI_ISL_593650 to 593651, EPI_ISL_740881 to 740882, EPI_ISL_779398, EPI_ISL_792026, EPI_ISL_792034			
see above	Douglass Hanly Moir Pathology	NSW Health Pathology - Institute of Clinical Pathology and Medical Research; Westmead Hospital; University of Sydney	CIDM-PH et al.
EPI_ISL_640100, EPI_ISL_700513, EPI_ISL_700517, EPI_ISL_700525, EPI_ISL_700547, EPI_ISL_700552, EPI_ISL_700567 to 700568, EPI_ISL_700570, EPI_ISL_700582, EPI_ISL_700591 to 700592			
see above	Dr Abdurahman CDC wc DAC	NHLS/UCT	Arash Iranzadeh; Bruna Galvao; Carolyn Williamson; Deelan Doolabh; Diana Hardie; Innocent Mudau; Kruger Marais; Lynn Tyers; Marvin Hsiao; Stephen Korsman
EPI_ISL_960160 to 960161, EPI_ISL_960171 to 960172	Dr Abdurahman CDC wc DAC	National Health Laboratory Service/UCT	Arash Iranzadeh; Bruna Galvao; Carolyn Williamson; Deelan Doolabh; Diana Hardie; Innocent Mudau; Kruger Marais; Lynn Tyers; Marvin Hsiao; Stephen Korsman
EPI_ISL_745113, EPI_ISL_745132, EPI_ISL_745154	Dr Ivan Toms Clinic wc IVT	National Health Laboratory Service (NHLS), Tygerberg	Bronwyn Kleinhans; Eduan Wilkindon; Gert van Zyl; Houriiyah Tegally; Kayla Delaney; Susan Engelbrecht; Tulio de Oliveira; Wolfgang Preiser
EPI_ISL_745112, EPI_ISL_745138 to 745139	Dr Van Niekerk Hospital	National Health Laboratory Service (NHLS), Tygerberg	Bronwyn Kleinhans; Eduan Wilkindon; Gert van Zyl; Houriiyah Tegally; Kayla Delaney; Susan Engelbrecht; Tulio de Oliveira; Wolfgang Preiser
EPI_ISL_1007612 to 1007613	Dr. Bard	Laboratory of genomics and metagenomics, Institute of Microbiology, University Hospital Centre and University of Lausanne, Switzerland	Claire Bertelli; Damien Jacot; Gilbert Greub; Sébastien Aeby; Trestan Pillonel
EPI_ISL_1007638	Dr. Borel	Laboratory of genomics and metagenomics, Institute of Microbiology, University Hospital Centre and University of Lausanne, Switzerland	Claire Bertelli; Damien Jacot; Gilbert Greub; Sébastien Aeby; Trestan Pillonel
EPI_ISL_766589 to 766590	Dr. Boubaker Karim laboratory	Institute of Medical Virology, University of Zurich	Alexandra Trkola; Annette Audigé; Cyril Shah; Jon Huder; Jürg Böni; Kevin Steiner; Maria Grünberg; Maryam Zaheri; Michael Huber; Riccarda Capaul; Stefan Schmutz; Verena Kufner
EPI_ISL_1041030	Dr. Cipto Mangunkusumo (RSCM) Hospital	Faculty of Medicine, Universitas Indonesia	Ari Fahrial Syam; Mei Riasanti; Rani Dwi Ningtias; Rina La Distia Nora
EPI_ISL_418811, EPI_ISL_429806, EPI_ISL_429811, EPI_ISL_429816, EPI_ISL_479625 to 479651, EPI_ISL_481742 to 481758, EPI_ISL_482471 to 482473			
see above	Dr. Georges-L.-Dumont University Hospital Centre	National Microbiology Laboratory	Anna Majer; Elsie Grudeski; Gary Van Domselaar; Grace Seo; Guillaume Desnoyers; Jennifer Tanner; Kristyn Burak; Matthew Gilmour; Morag Graham; Natalie Knox; Nathalie Bastien; Philip Mabon; Rhiannon Huzarewich; Richard Garceau; Russell Mandes; Shari Tyson; Timothy Booth; Yan Li
EPI_ISL_583727 to 583852	Dr. Gernot Walder GmbH	Bergthaler laboratory, CeMM Research Center for Molecular Medicine of the Austrian Academy of Sciences	Adi Steinrigl; Alexander Lercher; Alexandra Popa; Andreas Bergthaler; Benedikt Agerer; Christian Paar; Christoph Bock; Daniela Schmid; Dorothee von Laer; Elisabeth Puchhammer-Stoeckl; Franz Allerberger; Gernot Walder; Gregor Hörmann; Guenter Weiss; Gunther Vogl; Henrique Colaco; Jakob-Wendelin Genger; Jan Laine; Judith Aberle; Kinga Rigler-Hohenwarter; Lukas Endler; Manfred Nairz; Mark Smyth; Martin Senekowitsch; Michael Schuster; Peter Hufnagl; Peter Obrist; Rainer Gattringer; Sabine Sussitz-Rack; Stephan Aberle; Thomas Penz; Wegene Borena
EPI_ISL_528752	Dr. H. A. Rotinsulu Lung Hospital	School of Pharmacy & School of Life Sciences and Technology - Institut Teknologi Bandung; Molecular Genetics Laboratory-Faculty of Medicine-Universitas Padjadjaran; Laboratorium Kesehatan Provinsi Jawa Barat	Adelina Khristiani Rahayu; Agung Eru Wibowo; Azzania Fibriani; Catur Riani; Cut Nur Cinthia Alamanda; Ema Rahmawati; Gusti Ayu Prani Pradani; Hammam Riza; Hesti Lina Wiraswati; Husna Nugrahapraja; Irvan Faizal; Lia Faridah; Marselina Irasonia Tan; Rifky Waluyajati Rachman; Ryan Bayusantika Ristandi; Savira Ekawardhani; Sony Solistia Wirawan; Tarwadi; Yunia Sribudiani
EPI_ISL_906760 to 906776, EPI_ISL_1055385 to 1055451	Dr. Leonard A. Miller Centre for Health Services	National Microbiology Laboratory (NML)	Adel Malek; Anna Majer; Anneliese Landgraff; CanCOGeN's metadata curation team; Darian Hole; Elsie Grudeski; Gary Van Domselaar; George Zahariadis; Grace Seo; Jennifer Tanner; Kerri Smith; Kirsten Biggar; Laura Gilbert; Madison Chapel; Morag Graham; Natalie Knox; Nathalie Bastien; Philip Mabon; Public Health Agency of Canada CanCOGeN team; Rhiannon Huzarewich; Robert Needle; Russell Mandes; Shari Tyson; Timothy Booth; Yan Li; Yang Yu
EPI_ISL_469043 to 469047, EPI_ISL_475056 to 475057, EPI_ISL_495067 to 495069	Dr. N. D. Desai Medical College & Hospital	Gujarat Biotechnology Research Centre	A M Kadri; Afzal Ansari; Akanksha Verma; Ankit Hinsu; Apurvasinh Puvar; Bhavya Jindal; Chaitanya Joshi; Dinesh Kumar; Harsh Bakshi; J G Buch; Janvi Raval; Jigar Gusani; Komal Patel; Labdhi Pandya; Madhvi Joshi; Maharshi Pandya; Monika Gandhi; Neha Rajpara; Nidhi Patel; Nikha Trivedi; Nitin Savaliya; Pinal Trivedi; Pooja P Doshi; Pritesh Sabara; Priti Pandita; Priyanka P Vatsa; R D Dixit; Raghawendra Kumar; Snehal Bagatharia; Supreet Prabhu; Tejas Shah; Zarna Patel; Zuber Saiyed
EPI_ISL_512075 to 512077, EPI_ISL_514435 to 514437, EPI_ISL_524732	Dr. RSS Hospital, Modasa	Gujarat Biotechnology Research Centre	A M Kadri; Afzal Ansari; Apurvasinh Puvar; Chaitanya Joshi; Dinesh Kumar; Harsh Bakshi; Harsh Chaudhari; Janvi Raval; Komal Patel; Labdhi Pandya; Madhvi Joshi; Maharshi Pandya; Monika Gandhi; Neena Doshi; Nidhi Patel; Nikha Trivedi; Nitin Savaliya; Pinal Trivedi; R D Dixit; Raghawendra Kumar;

			Varsha Godbole; Zarna Patel; Zuber Saiyed
EPI_ISL_495012	Dr. Tony Mazzulli Microbiologist-in-Chief	Dr. Jeff Wrana, Senior Investigator	Bryn Hazlett; Christine Bruce; Dan Trcka; Javier Hernandez; Jeff Wrana; Jess Shen; Jessica Bourke; Kin Chan; Laurence Pelletier; Marie-Ming Aynaud; Seda Barutcu; Sue Poutanen; Tony Mazzulli
EPI_ISL_494970, EPI_ISL_495013	Dr. Tony Mazzulli Microbiologist-in-Chief	Dr. Jeff Wrana, Senior Investigator	Bryn Hazlett; Christine Bruce; Dan Trcka; Javier Hernandez; Jeff Wrana; Jess Shen; Jessica Bourke; Kin Chan; Laurence Pelletier; Marie-Ming Aynaud; Seda Barutcu; Sue Poutanen; Tony Mazzulli
EPI_ISL_640057, EPI_ISL_640059, EPI_ISL_640091, EPI_ISL_640094 to 640095	Du Noon CDC wc DNC	NHLS/UCT	Arash Iranzadeh; Bruna Galvao; Carolyn Williamson; Deelan Doolabh; Diana Hardie; Innocent Mudau; Kruger Marais; Lynn Tyers; Marvin Hsiao; Stephen Korsman
EPI_ISL_417211 to 417212	Dunedin Hospital	University of Otago	B. Lawley; J. Grant; J. Ussher; M.E. Quiñones-Mateu; R. Harfoot
EPI_ISL_414423 to 414446, EPI_ISL_414448 to 414471, EPI_ISL_414529 to 414566, EPI_ISL_415460 to 415535, EPI_ISL_422566 to 422635, EPI_ISL_422637 to 422960, EPI_ISL_423034, EPI_ISL_455112 to 455307, EPI_ISL_460636 to 461398, EPI_ISL_522987 to 523499, EPI_ISL_523501 to 523809, EPI_ISL_574779 to 574789, EPI_ISL_577743 to 577803, EPI_ISL_577805, EPI_ISL_577807 to 578079, EPI_ISL_632321 to 632795, EPI_ISL_722273 to 722850, EPI_ISL_763099 to 763137, EPI_ISL_763139 to 763317, EPI_ISL_763348 to 763349, EPI_ISL_763353 to 763359, EPI_ISL_801404 to 801515, EPI_ISL_802506 to 802544, EPI_ISL_904135 to 904625, EPI_ISL_1310833 to 1310834, EPI_ISL_1310837 to 1310841, EPI_ISL_1310843 to 1310850, EPI_ISL_1310852 to 1310864, EPI_ISL_1310866 to 1310869, EPI_ISL_1310871 to 1310879, EPI_ISL_1310881 to 1310882, EPI_ISL_1310884 to 1310895, EPI_ISL_1310898 to 1310900, EPI_ISL_1310902 to 1310903, EPI_ISL_1310905 to 1310915, EPI_ISL_1310917, EPI_ISL_1310919 to 1310922, EPI_ISL_1310924 to 1310939, EPI_ISL_1310942 to 1310943, EPI_ISL_1310945 to 1310960, EPI_ISL_1310962 to 1310965, EPI_ISL_1310967 to 1310973, EPI_ISL_1310976 to 1310979, EPI_ISL_1310981 to 1310985, EPI_ISL_1310987 to 1310995, EPI_ISL_1310997 to 1310998, EPI_ISL_1311000 to 1311007, EPI_ISL_1311009 to 1311010, EPI_ISL_1311012 to 1311013, EPI_ISL_1311017, EPI_ISL_1311020 to 1311031, EPI_ISL_1311033 to 1311041, EPI_ISL_1311043 to 1311056, EPI_ISL_1311058 to 1311093, EPI_ISL_1311095 to 1311098, EPI_ISL_1311100, EPI_ISL_1311102 to 1311114, EPI_ISL_1311116, EPI_ISL_1311119 to 1311121, EPI_ISL_1311127, EPI_ISL_1311136 to 1311148, EPI_ISL_1311154 to 1311158, EPI_ISL_1311169 to 1311183, EPI_ISL_1311185 to 1311186, EPI_ISL_1311188 to 1311194, EPI_ISL_1311196 to 1311203, EPI_ISL_1311205 to 1311208, EPI_ISL_1311211 to 1311236, EPI_ISL_1311238, EPI_ISL_1311240 to 1311241, EPI_ISL_1311244 to 1311247, EPI_ISL_1311249, EPI_ISL_1311251, EPI_ISL_1311253, EPI_ISL_1311255 to 1311259, EPI_ISL_1311261 to 1311281, EPI_ISL_1311283 to 1311294, EPI_ISL_1311297 to 1311300, EPI_ISL_1311304 to 1311308, EPI_ISL_1311310 to 1311320, EPI_ISL_1311322 to 1311328, EPI_ISL_1311331 to 1311344, EPI_ISL_1311356 to 1311366, EPI_ISL_1311370, EPI_ISL_1311372 to 1311375, EPI_ISL_1311377 to 1311388, EPI_ISL_1311390 to 1311402, EPI_ISL_1311404 to 1311407, EPI_ISL_1311413 to 1311435, EPI_ISL_1311439 to 1311470, EPI_ISL_1311472, EPI_ISL_1311474 to 1311526, EPI_ISL_1311535 to 1311537, EPI_ISL_1311539, EPI_ISL_1311541 to 1311551, EPI_ISL_1311558 to 1311565, EPI_ISL_1311569 to 1311577, EPI_ISL_1311581 to 1311586, EPI_ISL_1311591 to 1311594, EPI_ISL_1311597 to 1311618, EPI_ISL_1311624 to 1311627, EPI_ISL_1311630 to 1311635, EPI_ISL_1311640 to 1311667, EPI_ISL_1311670 to 1311688, EPI_ISL_1311696 to 1311701, EPI_ISL_1311710 to 1311723, EPI_ISL_1311725 to 1311743, EPI_ISL_1311752 to 1311815, EPI_ISL_1311818 to 1311825, EPI_ISL_1311829 to 1311832, EPI_ISL_1311834, EPI_ISL_1311836 to 1311847			
see above	Dutch COVID-19 response team	Erasmus Medical Center	Anne van der Linden; Annemiek van der Eijk; Aura Timen; Bas Oude Munnink; Claudia Schapendonk; Corien Swaan; Corine GeurtsvanKessel; David Nieuwenhuijse; Emmanuelle Munger; Irina Chestakova; Jeroen van Kampen; Jolanda Voermans; Madelief Mollers; Manon Haverkate; Marion Koopmans; Marjan Boter; Mark Pronk; Mart Stein; OH consortium; Pascal Lexmond; Reina Sikkema; Richard Molenkamp; Sandra Kengne Kamga Mobou; Stefan van Nieuwkoop; Theo Bestebroer; on behalf of the Dutch national COVID-19 response team.; on behalf of the OH consortium.
EPI_ISL_965529 to 965769, EPI_ISL_965771 to 965800, EPI_ISL_965802 to 965811, EPI_ISL_1120140 to 1120594, EPI_ISL_1158581 to 1158653, EPI_ISL_1158664 to 1158668, EPI_ISL_1158731 to 1158845, EPI_ISL_1158847 to 1158913, EPI_ISL_1158918 to 1158927, EPI_ISL_1210351 to 1210493			
see above	Dutch COVID-19 response team	Medical Microbiology, Maastricht University Medical Centre	Brian van der Veer*; Carmen Reumkens; Christian Hoebe; Erik Beuken; Jozef Dingemans*; Lieke van Alphen; Paul Savelkoul
EPI_ISL_454750 to 454794, EPI_ISL_547445 to 547568, EPI_ISL_636491 to 636603, EPI_ISL_723155 to 723467, EPI_ISL_728564 to 728763, EPI_ISL_747521 to 747525, EPI_ISL_790570 to 791083, EPI_ISL_804379 to 804462, EPI_ISL_823976 to 824283, EPI_ISL_826523 to 826536, EPI_ISL_904658 to 904934, EPI_ISL_904958 to 905730, EPI_ISL_942977 to 943547, EPI_ISL_1013616 to 1013618, EPI_ISL_1013620, EPI_ISL_1013625 to 1013630, EPI_ISL_1013633 to 1013642, EPI_ISL_1013644 to 1013650, EPI_ISL_1013652 to 1013677, EPI_ISL_1013680 to 1013687, EPI_ISL_1013689, EPI_ISL_1013691 to 1013696, EPI_ISL_1013698, EPI_ISL_1013700 to 1013707, EPI_ISL_1013713 to 1013722, EPI_ISL_1013726 to 1013727, EPI_ISL_1013729 to 1013733, EPI_ISL_1013737 to 1013741, EPI_ISL_1013743 to 1013745, EPI_ISL_1013747 to 1013748, EPI_ISL_1013752 to 1013755, EPI_ISL_1013759 to 1013770, EPI_ISL_1013773 to 1013778, EPI_ISL_1013780 to 1013786, EPI_ISL_1013788 to 1013791, EPI_ISL_1013793 to 1013794, EPI_ISL_1013798 to 1013818, EPI_ISL_1013821 to 1013825, EPI_ISL_1013829, EPI_ISL_1013831 to 1013834, EPI_ISL_1013837 to 1013838, EPI_ISL_1013842 to 1013846, EPI_ISL_1013849 to 1013854, EPI_ISL_1013857 to 1013888, EPI_ISL_1013890 to 1013895, EPI_ISL_1013897 to 1013905, EPI_ISL_1013907 to 1013909, EPI_ISL_1013911 to 1013912, EPI_ISL_1013917 to 1013918, EPI_ISL_1013921 to 1013922, EPI_ISL_1013924 to 1013961, EPI_ISL_1013963 to 1013964, EPI_ISL_1013969 to 1013983, EPI_ISL_1013985 to 1013994, EPI_ISL_1013996 to 1014017, EPI_ISL_1014021 to 1014101, EPI_ISL_1014103 to 1014120, EPI_ISL_1014128 to 1014130, EPI_ISL_1014150 to 1014151, EPI_ISL_1014156 to 1014218, EPI_ISL_1014220 to 1014235, EPI_ISL_1014239 to 1014344, EPI_ISL_1014346 to 1014434, EPI_ISL_1014437 to 1014448, EPI_ISL_1014450 to 1014496, EPI_ISL_1014498 to 1014510, EPI_ISL_1014512 to 1014516, EPI_ISL_1014518 to 1014541, EPI_ISL_1014543 to 1014544, EPI_ISL_1014546 to 1014592, EPI_ISL_1014594 to 1014674, EPI_ISL_1020302 to 1020304, EPI_ISL_1034917, EPI_ISL_1034919 to 1034923, EPI_ISL_1034925 to 1034952, EPI_ISL_1034954, EPI_ISL_1034961 to 1034964, EPI_ISL_1034968 to 1034971, EPI_ISL_1034974 to 1034981, EPI_ISL_1034987 to 1034989, EPI_ISL_1034991 to 1035023, EPI_ISL_1035030, EPI_ISL_1035032 to 1035034, EPI_ISL_1035039 to 1035075, EPI_ISL_1035078 to 1035087, EPI_ISL_1035089 to 1035098, EPI_ISL_1035100 to 1035117, EPI_ISL_1035120 to 1035130, EPI_ISL_1035134 to 1035135, EPI_ISL_1035138 to 1035151, EPI_ISL_1035154, EPI_ISL_1035156 to 1035174, EPI_ISL_1035176, EPI_ISL_1035179, EPI_ISL_1035181, EPI_ISL_1035183 to 1035198, EPI_ISL_1035200 to 1035201, EPI_ISL_1035203 to 1035209, EPI_ISL_1035211 to 1035215, EPI_ISL_1035217 to 1035234, EPI_ISL_1035236 to 1035256, EPI_ISL_1035258, EPI_ISL_1035260 to 1035275, EPI_ISL_1035277 to 1035280, EPI_ISL_1035282 to 1035283, EPI_ISL_1035286 to 1035439, EPI_ISL_1035442 to 1035452, EPI_ISL_1035454 to 1035458, EPI_ISL_1035461, EPI_ISL_1035467 to 1035476, EPI_ISL_1035479 to 1035487, EPI_ISL_1035500 to 1035513, EPI_ISL_1035525 to 1035557, EPI_ISL_1035560 to 1035633, EPI_ISL_1035635 to 1035636, EPI_ISL_1035639 to 1035640, EPI_ISL_1035643 to 1035645, EPI_ISL_1035647 to 1035650, EPI_ISL_1035653 to 1035657, EPI_ISL_1035659 to 1035675, EPI_ISL_1035680 to 1035710, EPI_ISL_1035712 to 1035720, EPI_ISL_1035723 to 1035742, EPI_ISL_1035744, EPI_ISL_1035746 to 1035756, EPI_ISL_1035763 to 1035808, EPI_ISL_1059903 to 1059921, EPI_ISL_1089434 to 1089481, EPI_ISL_1089503 to 1089594, EPI_ISL_1089597 to 1089615, EPI_ISL_1089617 to 1089629, EPI_ISL_1089631 to 1089667, EPI_ISL_1089690 to 1089775, EPI_ISL_1089777 to 1089782, EPI_ISL_1089784 to 1089788, EPI_ISL_1089790 to 1089807, EPI_ISL_1089809 to 1089899, EPI_ISL_1089957 to 1090273, EPI_ISL_1165144 to 1165217, EPI_ISL_1165219 to 1165227, EPI_ISL_1165229 to 1165234, EPI_ISL_1165236, EPI_ISL_1165238 to 1165313, EPI_ISL_1165315 to 1165370, EPI_ISL_1165372 to 1165380, EPI_ISL_1165382 to 1165406, EPI_ISL_1165408, EPI_ISL_1165410 to 1165441, EPI_ISL_1165443 to 1165482, EPI_ISL_1165484 to 1165554, EPI_ISL_1165556 to 1165561, EPI_ISL_1165563 to 1165576, EPI_ISL_1165578 to 1165666, EPI_ISL_1165668 to 1165671, EPI_ISL_1165673 to 1165674, EPI_ISL_1165678 to 1165755, EPI_ISL_1165757 to 1165769, EPI_ISL_1165771 to 1165784, EPI_ISL_1165786, EPI_ISL_1165788 to 1165800, EPI_ISL_1165802 to 1165803, EPI_ISL_1165805 to 1165810, EPI_ISL_1216226, EPI_ISL_1216228, EPI_ISL_1216230, EPI_ISL_1216232, EPI_ISL_1216234, EPI_ISL_1216236, EPI_ISL_1216238 to 1216239, EPI_ISL_1216241, EPI_ISL_1216243, EPI_ISL_1216245, EPI_ISL_1216247, EPI_ISL_1216249 to 1216250, EPI_ISL_1216252, EPI_ISL_1216254, EPI_ISL_1216256, EPI_ISL_1216258, EPI_ISL_1216260 to 1216261, EPI_ISL_1216263, EPI_ISL_1216265, EPI_ISL_1216267, EPI_ISL_1216269, EPI_ISL_1216271 to 1216272, EPI_ISL_1216274, EPI_ISL_1216276, EPI_ISL_1216278 to 1216279, EPI_ISL_1216281, EPI_ISL_1216283, EPI_ISL_1216285, EPI_ISL_1216287 to 1216288, EPI_ISL_1216290, EPI_ISL_1216292, EPI_ISL_1216294, EPI_ISL_1216296 to 1216297, EPI_ISL_1216299, EPI_ISL_1216301, EPI_ISL_1216303, EPI_ISL_1216305, EPI_ISL_1216307 to 1216308, EPI_ISL_1216310, EPI_ISL_1216312, EPI_ISL_1216314 to 1216315, EPI_ISL_1216317, EPI_ISL_1216319 to 1216320, EPI_ISL_1216322, EPI_ISL_1216324, EPI_ISL_1216326, EPI_ISL_1216328, EPI_ISL_1216330 to 1216331, EPI_ISL_1216333 to 1216334, EPI_ISL_1216336, EPI_ISL_1216338 to 1216339, EPI_ISL_1216341, EPI_ISL_1216343, EPI_ISL_1216345, EPI_ISL_1216347 to 1216348, EPI_ISL_1216350, EPI_ISL_1216352, EPI_ISL_1232243 to 1232905, EPI_ISL_1232908 to 1233007, EPI_ISL_1287783 to 1288152, EPI_ISL_1288534, EPI_ISL_1288536, EPI_ISL_1288539 to 1288804, EPI_ISL_1288871 to 1289402			
see above	Dutch COVID-19 response team	National Institute for Public Health and the Environment (RIVM)	Adam Meijer; AnneMarie van den Brandt; Bas van der Veer; Chantal Reusken; Dennis Schmitz; Dirk Eggink; Florian Zwagemaker; Harry Vennema; Jeroen Cremer; Matthijs Welkers; Pieter Overduin; Sharon van den Brink; on behalf of the national COVID-19 response team
EPI_ISL_913275, EPI_ISL_913277, EPI_ISL_913281, EPI_ISL_913309, EPI_ISL_913378 to 913392, EPI_ISL_913454, EPI_ISL_913459, EPI_ISL_934377, EPI_ISL_1008479 to 1008489, EPI_ISL_1064551, EPI_ISL_1064607, EPI_ISL_1064613 to 1064620, EPI_ISL_1168046			
see above	Dynamic Code AB	The Public Health Agency of Sweden	Anna Risberg; Anna-Malin Linde; Carlo Berg; Karin Tegmark-Wisell; Maria Lind Karlberg; Mattias Haukland; Mia Brytting; Noura Walai; Oskar Karlsson Lindsjo; Petra Edquist; Petra Holmstrom; Reza Advani; Samuel Ohman; Sofia Stamouli
EPI_ISL_640024, EPI_ISL_700520	Dysselsdorp Clinic wc DDC	NHLS/UCT	Arash Iranzadeh; Bruna Galvao; Carolyn Williamson; Deelan Doolabh; Diana Hardie; Houriiyah Tegally; Innocent Mudau; Kruger Marais; Lynn Tyers; Marvin Hsiao; Stephen Korsman
EPI_ISL_960096	Dysselsdorp Clinic wc DDC	National Health Laboratory Service/UCT	Arash Iranzadeh; Bruna Galvao; Carolyn Williamson; Deelan Doolabh; Diana Hardie; Innocent Mudau; Kruger Marais; Lynn Tyers; Marvin Hsiao; Stephen Korsman
EPI_ISL_1137609	Département de Maladies Infectieuses, CHU Farhat Hached Sousse, Tunisie	Laboratoire des Procédés de Criblage Moléculaire et Cellulaire-Centre de Biotechnologie de Sfax	A. and Masmoudi, S.; Abdelmoulah, F.; Abid, M.; Abid, N.; Ajili, F.; Aouni, M.; Ben Ayed, I.; Bensaid, M.; Chawech, H.; Chtourou, A.; Elargoubi, A.; Fki-berrajah, L.; Gaaloul, I.; Gargouri, S.; Hammami, A.; Kamoun, S.; Karray Hakim, H.; Kharat, N.; Letaief, A.; Mastouri, M.; Mhalla, S.; Nabli, A.; Rebai; Smeti, I.; Souissi, A.; Stambouli, N.; Turki, M.
EPI_ISL_419691	E. Gulbja Laboratorija	Charite Universitatsmedizin Berlin, Institute of Virology	Barbara Mühlemann; Christian Drosten; Dmitrijs Perminovs; Dr. Didzis Gavars; Jorn Beheim-Schwarzbach; Julia Schneider; Mikus Gavars; Talitha Veith; Terry Jones; Victor M Corman
EPI_ISL_421653 to 421656, EPI_ISL_426285 to 426289, EPI_ISL_450518 to 450519, EPI_ISL_492993 to 493000, EPI_ISL_501275, EPI_ISL_501284 to 501285, EPI_ISL_501823, EPI_ISL_501829, EPI_ISL_501833, EPI_ISL_501839, EPI_ISL_501849, EPI_ISL_501894, EPI_ISL_501929, EPI_ISL_501936, EPI_ISL_512313 to 512314, EPI_ISL_512645 to 512646, EPI_ISL_515187 to 515195, EPI_ISL_534199, EPI_ISL_534203, EPI_ISL_534212 to 534216, EPI_ISL_534218 to 534219, EPI_ISL_534221, EPI_ISL_639631, EPI_ISL_639635, EPI_ISL_639637 to 639638, EPI_ISL_639641, EPI_ISL_639643 to 639647, EPI_ISL_639649, EPI_ISL_639652 to 639653, EPI_ISL_639658 to 639662, EPI_ISL_639664 to 639671, EPI_ISL_639673 to 639675, EPI_ISL_639681 to 639682, EPI_ISL_639684 to 639694, EPI_ISL_770033 to 770037, EPI_ISL_770040 to 770041, EPI_ISL_770043 to 770044, EPI_ISL_770046, EPI_ISL_770050 to 770051, EPI_ISL_770053, EPI_ISL_770055, EPI_ISL_770057, EPI_ISL_770062			
see above	E. Gulbja Laboratorija	Latvian Biomedical Research and Study Centre	Dmitrijs Perminovs; Ivars Silamielis; Jnis Kloviš; Jnis Pjalkovskis; Kaspars Megnis; Mikus Gavars; Monta Ustinova; Uga Dumpis; Vita Rovte; ikita Zrelovs
EPI_ISL_486438, EPI_ISL_961801 to 961804, EPI_ISL_961807 to 961808, EPI_ISL_961832, EPI_ISL_961853 to 961861, EPI_ISL_961864 to 961865, EPI_ISL_961867 to 961868, EPI_ISL_961871 to 961875, EPI_ISL_961877 to 961882, EPI_ISL_1219194 to 1219231, EPI_ISL_1219254 to 1219267, EPI_ISL_1219321 to 1219351, EPI_ISL_1219458 to 1219471, EPI_ISL_1219504 to 1219558, EPI_ISL_1219579 to 1219594, EPI_ISL_1219596 to 1219608, EPI_ISL_1219658 to 1219683, EPI_ISL_1312390 to 1312497, EPI_ISL_1312565 to 1312619, EPI_ISL_1312624 to 1312658, EPI_ISL_1312683 to 1312703, EPI_ISL_1312721 to 1312729			
see above	E. Gulbja laboratorija	Latvian Biomedical Research and Study Centre	Davids Fridmanis; Davids Fridmanis; Dmitrijs Perminovs; Dmitrijs Perminovs; Guntars Zarins; Ivars Silamikelis; Ivars Silamielis; Ivars Silamikelis; Janis Klovins; Janis Pjalkovskis; Janis Klovins; Jurijs Perevoscikovs; Jnis Kloviš; Kaspars Megnis; Kaspars Megnis; Laila Silamikele; Laila Silamikele; Lauma Freimane; Lauma Freimane; Laura Ansone; Laura Ansone; Liga Birzniece; Liga Birzniece; Mikus Gavars; Mikus Gavars; Monta Ustinova; Monta Ustinova; Nikita Zrelovs; Nikita Zrelovs; Uga Dumpis; Uga Dumpis; Una Krumina; Vita Rovite; Vita Rovte; Vita Rovite; ikita Zrelovs
EPI_ISL_1085329, EPI_ISL_1085371, EPI_ISL_1085416, EPI_ISL_1085419	E.F.S B.F.C JEAN MINJOZ	Department of Virology, Henri Mondor University Hospital, Assistance Publique Hôpitaux de Paris, Université Paris-Est Créteil, INSERM U955	Alexandre Soulier; Christophe Rodriguez; Elisabeth Trawinski; Guillaume Gricourt; Jean-Michel Pawlotsky; Melissa N'Debi; Slim Fourati; Vanessa Demontant
EPI_ISL_1220066	E.S.E. HOSPITAL SAN JOSE DE MAICAO	Instituto Nacional de Salud- Dirección de Investigación en Salud Pública	Carlos Franco-Muñoz; Diego A. Álvarez-Díaz; Diego Andrés Prada; Gerardo Santamaría; Hector Alejandro Ruiz-Moreno; Jhonnatan Reales-González; Julian Naizaque; Katherine Laiton-Donato; Magdalena Wiesner; Marcela Mercado-Reyes.; Maria T. Herrera-Sepúlveda; Martha Lucia Ospina Martínez; Sheryll Corchuelo
EPI_ISL_906545	E.S.E. HOSPITAL SAN JOSE DE MAICAO	Instituto Nacional de Salud- Dirección de Investigación en	Carlos Franco-Muñoz; Diego A. Álvarez-Díaz; Diego Andrés Prada; Gerardo Santamaría Jorge Duitama; Héctor Alejandro Ruiz-Moreno; Jhonnatan

		Salud Pública, Universidad de los Andes- Applied genomics research group, Vicerrectoria de Investigación y Creación, Universidad de los Andes- Systems and Computing Engineering Department	Reales-González; Jorge Ivan Diaz; Julian Naizaque; Katherine Laiton-Donato; Laura Natalia Gonzalez; Magdalena Wiesner; Marcela Mercado-Reyes; Maria T. Herrera-Sepúlveda; Martha Lucia Ospina Martinez; Mauricio Pacheco-Montealegre; Sheryll Corchuelo; Silvia Restrepo-Restrepo
EPI_ISL_434540	EBAIS Concepción Norte	Inciensa, Instituto Costarricense de Investigación y Enseñanza en Nutrición y Salud	Adriana Godinez & Melany Calderon; Claudio Soto-Garita; Estela Cordero; Francisco Duarte; Hebleen Porras
EPI_ISL_982123	EFS ILE DE FRANCE BANQUE DE TISSUS	Department of Virology, Henri Mondor University Hospital, Assistance Publique Hôpitaux de Paris, Université Paris-Est Créteil, INSERM U955	Alexandre Soulier; Christophe Rodriguez; Elisabeth Trawinski; Guillaume Gricourt; Jean-Michel Pawlotsky; Melissa N'Debi; Slim Fourati; Vanessa Demontant
EPI_ISL_1007585	EHC MORGES	Laboratory of genomics and metagenomics, Institute of Microbiology, University Hospital Centre and University of Lausanne, Switzerland	Claire Bertelli; Damien Jacot; Gilbert Greub; Sébastien Aeby; Trestan Pillonel
EPI_ISL_970469, EPI_ISL_1007597 to 1007598, EPI_ISL_1007602, EPI_ISL_1007625 to 1007628, EPI_ISL_1007631, EPI_ISL_1007634 to 1007635, EPI_ISL_1120836 to 1120841, EPI_ISL_1120857 to 1120871			
see above	EHNV	Laboratory of genomics and metagenomics, Institute of Microbiology, University Hospital Centre and University of Lausanne, Switzerland	Claire Bertelli; Damien Jacot; Gilbert Greub; Sébastien Aeby; Trestan Pillonel
EPI_ISL_765478	EHPAD	National Reference Center for Viruses of Respiratory Infections, Institut Pasteur, Paris	Angela Brisebarre; Camille Capel; Etienne Simon-Lorière; Marion Barbet; Maud Vanpeene; Méline Bizard; Sylvie Behillil; Sylvie van der Werf; Vincent Enouf
EPI_ISL_418226	EHPAD - Résidences les Cèdres	National Reference Center for Viruses of Respiratory Infections, Institut Pasteur, Paris	Angela Brisebarre; Etienne Simon-Lorière; Flora Donati; Marion Barbet; Maud Vanpeene; Mélanie Albert; Méline Bizard; Sylvie Behillil; Sylvie van der Werf; Vincent Enouf
EPI_ISL_1285044, EPI_ISL_1285046, EPI_ISL_1285048, EPI_ISL_1285053, EPI_ISL_1285061 to 1285063, EPI_ISL_1285065, EPI_ISL_1285071, EPI_ISL_1285076, EPI_ISL_1285083, EPI_ISL_1285086, EPI_ISL_1285089, EPI_ISL_1285092, EPI_ISL_1285095, EPI_ISL_1285100, EPI_ISL_1285108 to 1285109, EPI_ISL_1285113, EPI_ISL_1285122 to 1285123, EPI_ISL_1285131, EPI_ISL_1285133, EPI_ISL_1285138, EPI_ISL_1285142, EPI_ISL_1285150, EPI_ISL_1285155, EPI_ISL_1285157			
see above	ELBLAB GmbH Zentrum für LaborMedizin	Robert Koch Institute	
EPI_ISL_1220053	EMPRESA SOCIAL DEL ESTADO HOSPITAL UNIVERSITARIO DE SANTANDER	Instituto Nacional de Salud- Dirección de Investigación en Salud Pública	Carlos Franco-Muñoz; Diego A. Álvarez-Díaz; Diego Andrés Prada; Gerardo Santamaría; Hector Alejandro Ruiz-Moreno; Jhonnatan Reales-González; Julian Naizaque; Katherine Laiton-Donato; Magdalena Wiesner; Marcela Mercado-Reyes.; Maria T. Herrera-Sepúlveda; Martha Lucia Ospina Martínez; Sheryll Corchuelo
EPI_ISL_956304 to 956305	ESE INSTITUTO NACIONAL DE CANCEROLOGIA	Instituto Nacional de Salud- Dirección de Investigación en Salud Pública	Carlos Franco-Muñoz; Diego A. Álvarez-Díaz; Diego Andrés Prada; Gerardo Santamaría; Hector Alejandro Ruiz-Moreno; Jhonnatan Reales-González; Julian Naizaque; Katherine Laiton-Donato; Magdalena Wiesner; Marcela Mercado-Reyes; Maria T. Herrera-Sepúlveda; Martha Lucia Ospina Martinez; Mauricio Pacheco-Montealegre; Sheryll Corchuelo
EPI_ISL_745198	ESI Hospital	CSIR-Centre for Cellular and Molecular Biology	Archana Bharadwaj Siva; B Himasri; Blessy B John; Divya Tej Sowpati; Dr.P.Shashikala Reddy; Dr.S.Pavani; Dr.Satyaprasad; Dr.V.Sudha Rani; Karthik Bharadwaj Tallapaka; Lamuk Zaveri; M Soujanya Reddy; Namami Gaur; Nikhil Hajirnis; Onkar Kulkarni; Payel Mukherjee; Pratheusa Maccha; Priya Singh; Purushotham Vodnala; Rakesh K Mishra; Sakshi Shambhavi; Shagufta Khan; Sofia Banu; Tulasi Nagabandi; Viswagithe S L
EPI_ISL_515943 to 515944	ESIC	Department of Neurovirology, National Institute of Mental Health and Neuroscience (NIMHANS)	Anita Desai; Chitra Pattabiraman; Harsha PK; Manjunatha Venkataswamy; Pramada Prasad; Ravi Vasanthapuram; Risha Rasheed; Shafeeq S Hameed; Vijayalakshmi Reddy
EPI_ISL_854305	ESOTERIX GENETIC LABORATORIES LLC	Wadsworth Center, New York State Department of Health	Alexis Russel; Daryl M. Lamson; Erasmus Schneider; Erica Lasek-Nesselquist; John Kelly; Jonathan Plitnick; Kirsten St. George; Matthew Shudt; Melissa A Leisner; Navjot Singh
EPI_ISL_1166049	EXALAB BIOLOGIE MEDICALE	CNR Virus des Infections Respiratoires - France SUD	Antonin Bal; Bruno Lina; Gregory Destras; Gwendolyne Burfin; Hadrien Regue; Laurence Josset; Martine Valette; Quentin Semanas
EPI_ISL_912169	East Gene	Grubaugh Lab - Yale School of Public Health	Anderson Brito; Anne Wyllie; Annie Watkins; Chaney Kalinich; Chantal Vogels; Isabel Ott; Joseph Fauver; Mallery Breban; Mary Petrone; Nathan Grubaugh; Tara Alpert
EPI_ISL_712077 to 712078, EPI_ISL_712092	East London NHLS Laboratory, Eastern Cape, South Africa	National Institute for Communicable Diseases of the National Health Laboratory Service	Allam M; Bhiman JN; Ismail A; Mahlangu B; Mohale T; Ntuli N
EPI_ISL_593481, EPI_ISL_593483, EPI_ISL_593486 to 593487, EPI_ISL_593489 to 593499, EPI_ISL_593501 to 593504, EPI_ISL_593506 to 593509, EPI_ISL_593511 to 593515, EPI_ISL_593517 to 593519, EPI_ISL_593523 to 593529, EPI_ISL_593531 to 593534, EPI_ISL_593536 to 593541, EPI_ISL_593543 to 593551, EPI_ISL_770822 to 770827			
see above	Eastern Ontario Regional Laboratory Association	McMaster University	Ahmed Draia; Andrew G. McArthur; Emily Panousis; Hooman Derakhshani; Jalees Nasir; Leanne Mortimer; Robert Slinger
EPI_ISL_437349	Ecole nationale vétérinaire d'Alfort-laboratoire de santé animale Anses UMR 1161 de virologie ENVA-Anses-INRAE	Institut Pasteur CIBU-ERI	Bernard Klonjkowski; Corinne Sailleau; Marine Dumarest; Sophie Le Poder; Stéphan Zientara
EPI_ISL_1238787 to 1238793	Ecological and Evolutionary Genomics. UGA-CINVESTAV	Unidad Universitaria de Secuenciación Masiva y Bioinformática (UUSMB). IBT-UNAM	Angelica Cibrian
EPI_ISL_745137, EPI_ISL_745151	Edameni Clinic	National Health Laboratory Service (NHLS), Tygerberg	Bronwyn Kleinhans; Eduan Wilkindon; Gert van Zyl; Houriiyah Tegally; Kayla Delaney; Susan Engelbrecht; Tulio de Oliveira; Wolfgang Preiser
EPI_ISL_745119, EPI_ISL_745173 to 745178	Eerste River Hospital wc ERH	National Health Laboratory Service (NHLS), Tygerberg	Bronwyn Kleinhans; Eduan Wilkindon; Gert van Zyl; Houriiyah Tegally; Kayla Delaney; Susan Engelbrecht; Tulio de Oliveira; Wolfgang Preiser
EPI_ISL_468044 to 468062, EPI_ISL_475722 to 475724, EPI_ISL_477161, EPI_ISL_478672, EPI_ISL_479686 to 479735, EPI_ISL_524426 to 524427, EPI_ISL_529141 to 529145			
see above	Egyptian National Cancer Institute (ENCI)	Egyptian National Cancer Institute (ENCI)	A.A.; A.N.; Abdel Rahman N; Abdel Rahman N.; Abdelhamid, W.; Abouelhoda; Abouelhoda, M.; Ahmed; Ali; Ali, M.; Amer; Bahnassy; Elkhateeb; Elsissy; Ezzelarab; Gad, A.; H.K.; Hafez; Hamdy; Hassan; Hassan, W.; K.E.; Khattab; M.A.; M.H.; M.M.; M.S.; Mahmoud; Mohamed; O.S.; Raouf, A.; S.M.; Samir, M.; Soliman; W.A.; Zekri
EPI_ISL_469275	Egyptian National Cancer Institute (ENCI)	Human Genome Center	A.A.; Abdel Rahman N; Abdelhamid, W.; Abouelhoda; Ahmed; Ali, M.; Amer; Bahnassy; Elkhateeb; Elsissy; Ezzelarab; Gad, A.; H.K.; Hafez; Hamdy; Hassan, W.; K.E.; M.H.; M.M.; M.S.; Mohamed; O.S.; Raouf, A.; S.M.; Samir, M.; Soliman; Zekri
EPI_ISL_862849 to 862851, EPI_ISL_862879	Ehime Prefectural Central Hospital	Fujita health University, School of Medicine, Department of Microbiology	Masahiro suzuki; Yohei Doi
EPI_ISL_889005, EPI_ISL_947293	Eijkman Institute for Molecular Biology, Ministry of Research and Technology/National Agency for Research and Innovation	Eijkman Institute for Molecular Biology, Ministry of Research and Technology/National Agency for Research and Innovation	Amin Soebandrio; Edison Johar; Frilasita A Yudhaputri; Hidayat Trimarsanto; Iskandar Adnan; Khin Saw Myint; Lydia V. Panggalo; Safarina G Malik; Sukma Oktavianthi; Willy Agustine
EPI_ISL_434652, EPI_ISL_710610 to 710611	Ektorps Vardcentral	The Public Health Agency of Sweden	Anna Risberg; Anna-Malin Linde; Department of Microbiology; Eva Espmark; Karin Tegmark-Wisell; Maria Lind Karlberg; Mia Brytting; Olov Svartstrom; Oskar Karlsson Lindsjo; The Public Health Agency of Sweden; Theresa Enkirch
EPI_ISL_1258425, EPI_ISL_1258443, EPI_ISL_1258452	Elias Emergency University Hospital, Bucharest	National Institute of Infectious Diseases-Prof. Dr. Matei Bals Molecular Diagnostics Laboratory	Corina Casangiu; Dan Otelea; Ionelia Nicolae; Leontina Banica; Marius Surleac; Ovidiu Vlaicu; Simona Paraschiv
EPI_ISL_1117780, EPI_ISL_1118072	Elling group, Institute of Molecular Biotechnology (IMBA)	Bergthaler laboratory, CeMM Research Center for Molecular Medicine of the Austrian Academy of Sciences	Andreas Bergthaler; Anna Schedl; Bekir Erguner; Benedikt Agerer; Christoph Bock; Jan Laine; Lukas Endler; Maelle Le Moing; Martin Senekowitsch; Michael Schuster; Thomas Penz
EPI_ISL_745110, EPI_ISL_745125, EPI_ISL_745162	Elsies River CHC wc ERP	National Health Laboratory Service (NHLS), Tygerberg	Bronwyn Kleinhans; Eduan Wilkindon; Gert van Zyl; Houriiyah Tegally; Kayla Delaney; Susan Engelbrecht; Tulio de Oliveira; Wolfgang Preiser
EPI_ISL_605924 to 605928	Emergence des Maladies Virales, Centre Interdisciplinaire de Recherches Medicales de Franceville	Emergence des Maladies Virales, Centre Interdisciplinaire de Recherches Medicales de Franceville	A.M.; Andeko; J.-B.; J.C.; Kandet Yattara, M.; Lekana-Douki; Mbongo kama, E.; Mintsa Ndong, A.; N'dilimabaka, N.; Ngonga Dikongo; S.E.; Zong Minko, O.
EPI_ISL_486856	Emergency County Hospital	Stefan cel Mare, University Metagenomics lab	Lobiuc Andrei et al.

EPI_ISL_486854	Emergency County Hospital Suceava	Stefan cel Mare, University Metagenomics lab	Lobiuc Andrei et al.
EPI_ISL_708822, EPI_ISL_708824 to 708826, EPI_ISL_768612 to 768613	Emergency Operation Center, (EOC)	National Institute of Health, Department of Medical Sciences, Ministry of Public Health, Thailand	; Malinee Chittaganpitch; Natchaya Khiadsang; Pakorn Piromtong; Pilailuk Okada; Ratana Tacharoenmuang; Siripaporn Phuygun; Sittiporn Parnmen; Sunthareeya Waicharoen; Thanutsapa Thanadachakul; Warawan Wongboot; sirikanda wimol
EPI_ISL_486855	Emergency county Hospital Suceava	"Stefan cel Mare" University Metagenomics Lab	Lobiuc Andrei et al.
EPI_ISL_1121993, EPI_ISL_1122015 to 1122016	Emergency department of COVID-19, Beijing Ditan Hospital	National Institute for Viral Disease Control and Prevention	3*; Di Tian1; Gang Liu1; Gang Wan1; Jianbo Tan1; Long Liu1; Pan Xiang1; Shuangli Zhu2; Shuping Cui1; Tingyu Zhang1; Wei Zhang1#; Wenbo Xu2# and Zhihai Chen1#; Xianbo Wang1; Xiang Zhao2; Yang Song2*; Yanhai Wang2; Yanli Xu1; Yu Wang1; Ziruo Ge1*
EPI_ISL_1122017	Emergency department of COVID-19, Beijing Ditan Hospital, Capital Medical University, Beijing, China.	National Institute for Viral Disease Control and Prevention	3*; Di Tian1; Gang Liu1; Gang Wan1; Jianbo Tan1; Long Liu1; Pan Xiang1; Shuangli Zhu2; Shuping Cui1; Tingyu Zhang1; Wei Zhang1#; Wenbo Xu2# and Zhihai Chen1#; Xianbo Wang1; Xiang Zhao2; Yang Song2*; Yanhai Wang2; Yanli Xu1; Yu Wang1; Ziruo Ge1*
EPI_ISL_454690, EPI_ISL_455355 to 455361, EPI_ISL_754746 to 754778, EPI_ISL_845754 to 845782, EPI_ISL_1278055 to 1278100	Emory Molecular Diagnostics Laboratory, Emory Healthcare	Piantadosi Lab, Emory Department of Pathology	Ahmed Babiker; Anne Piantadosi
EPI_ISL_632976 to 632979	Empire City Laboratories	New York City Public Health Laboratory	Jade Wang; et al.
EPI_ISL_424350 to 424351, EPI_ISL_468718	Environmental and Global Health	Environmental and Global Health	Alam, M.; C.J.; Elbadry; Gibson; J.A.; J.C.; J.G.; J.G. Jr. and Lednicky; L.H.; M.A.; Merck; Morris; Stephenson; Subramaniam, K.; T.B.; Waltzek
EPI_ISL_641318 to 641319, EPI_ISL_666617 to 666622	Environmental and Global Health, University of Florida	Environmental and Global Health, University of Florida	Alam; C.-Y.; C.J.; C.T.; Elbadry; Fan; J.A.; J.C.; J.G.; J.G. Jr.; J.G.; L.O.; Lauzardo, M.; Lednicky; Loeb; M.A.; M.M.; Morris; Nannu-Shankar, S.; Silva; Stephenson; Subramaniam, K.; T.B.; Waltzek; Witanachchi; Wu; Z.H.
EPI_ISL_524433 to 524434	Environmental and Global Health, University of Florida - Gainesville	University of Florida	Alam; C.J.; Elbadry; Gibson; J.A.; J.C.; J.G. Jr.; Lednicky; M.A.; M.M.; Morris; Stephenson; Subramaniam, K.; T.B.; Waltzek
EPI_ISL_632287, EPI_ISL_681322, EPI_ISL_873277	Environmental and Global Health, University of Florida	Environmental and Global Health, University of Florida	Alam; C.J.; Elbadry; Gibson; Iovine; J.A.; J.C.; J.G. and Lednicky; Lauzardo, M.; Lessard, K.; Loeb; M.A.; M.M.; Merck, L.; Morris; N.M.; Reeves, M.; Stephenson; Subramaniam, K.; T.B.; Waltzek; Wilkerson, G.
EPI_ISL_1300448	Epidemiology of Microbial Diseases, Yale School of Public Health	Yale School of Public Health	Fauver; J.R.
EPI_ISL_1220542	Equigerminal	Equigerminal	Isabel Fidalgo Carvalho
EPI_ISL_413578, EPI_ISL_413582	ErasmusMC	Erasmus Medical Center	Anne van der Linden; Annemiek van der Eijk; Aura Timen; Bas Oude Munnink; Claudia Schapendonk; Corien Swaan; Corine GeurtsvanKessel; David Nieuwenhuijse; Irina Chestakova; Jeroen van Kampen; Jolanda Voermans; Madelief Mollers; Manon Haverkate; Marion Koopmans; Mark Pronk; Mart Stein; Pascal Lexmond; Reina Sikkema; Richard Molenkamp; Sandra Kengne Kamga Mobou; on behalf of the Dutch national COVID-19 response team.
EPI_ISL_1009289 to 1009616	Erie County Public Health (ECPHL)	University at Buffalo Genomics and Bioinformatics Core	Alyssa Pohlman; Amanda Boccolucci; Brandon Marzullo; Donald Yergeau; Jennifer Surtees; Jonathan Bard; Natalie Lamb; Norma Nowak
EPI_ISL_648166 to 648168	Eskilstuna	The Public Health Agency of Sweden	Anna Risberg; Anna-Malin Linde; Karin Tegmark-Wisell; Maria Lind Karlberg; Mattias Haukland; Mia Brytting; Olov Svartstrom; Oskar Karlsson Lindsjo; Petra Edquist; Reza Advani; Sandra Broddesson
EPI_ISL_514648	Essentia Health-St. Joseph's Medical Center	Minnesota Department of Health, Public Health Laboratory	Jacob Garfin; Matt Plumb; and Xiong Wang
EPI_ISL_514642, EPI_ISL_603004 to 603005, EPI_ISL_603012 to 603014, EPI_ISL_603016 to 603017, EPI_ISL_644933 to 644935, EPI_ISL_644943, EPI_ISL_654696 to 654697, EPI_ISL_654699, EPI_ISL_683690 to 683694, EPI_ISL_683696 to 683713, EPI_ISL_708484, EPI_ISL_770808 to 770813, EPI_ISL_802567 to 802571, EPI_ISL_983699 to 983704			
see above	Essentia Health-St. Mary's Medical Center	Minnesota Department of Health, Public Health Laboratory	Alexandra Lorentz; Jacob Garfin; Matt Plumb; and Xiong Wang
EPI_ISL_640064	Ethembeni Clinic [Prieska]	NHLS/UCT	Arash Iranzadeh; Bruna Galvao; Carolyn Williamson; Deelan Doolabh; Diana Hardie; Innocent Mudau; Kruger Marais; Lynn Tyers; Marvin Hsiao; Stephen Korsman
EPI_ISL_636971	Etlik Veterinary Control Central Research Institute	Etlik Veterinary Control Central Research Institute	Ahu Pakdemirli; Cevdet Yarali; Dilek Dulger; Erdem Danyer; Ozcan Yildirim; Sabri Hacioglu; Ummu Sena Sari
EPI_ISL_614265 to 614280	Eurofins	National Reference Center for Viruses of Respiratory Infections, Institut Pasteur, Paris	Angela Brisebarre; Camille Capel; Etienne Simon-Lorière; Marion Barbet; Maud Vanpeene; Méline Bizard; Sylvie Behillil; Sylvie van der Werf; Vincent Enouf
EPI_ISL_871817, EPI_ISL_872395 to 872477, EPI_ISL_875570 to 875659, EPI_ISL_875874 to 875958, EPI_ISL_876331 to 876425, EPI_ISL_876428 to 876517, EPI_ISL_877237 to 877328, EPI_ISL_877331 to 877418, EPI_ISL_877461 to 877537, EPI_ISL_877539 to 877550, EPI_ISL_883066 to 883154, EPI_ISL_883195 to 883284, EPI_ISL_883501 to 883861, EPI_ISL_883867 to 883956, EPI_ISL_884089 to 884181, EPI_ISL_979459 to 979543, EPI_ISL_1097644 to 1097723			
see above	Eurofins Diatherix	Hudsonalpha Genome Sequencing Center	Ada Stewart; Jane Grimwood; Jenell Webber; Jennifer Cart; John Lovell; John T. Lovell; Joshua Stough; Leslie Malone; Lori H. Handley; Melissa Williams; Stefan Brzezinski; Teresa Jones; and Jeremy Schmutz
EPI_ISL_983321	Eurofins Genoma Group s.r.l.	INMI Lazzaro Spallanzani IRCCS	A Di Caro; B Bartolini; CEM Gruber; E Giombini; F Fiorentino; F Messina; F Spinella; M Rueca; MR Capobianchi; O Butera
EPI_ISL_1153163, EPI_ISL_1153165 to 1153174, EPI_ISL_1153176 to 1153187, EPI_ISL_1153189, EPI_ISL_1153192 to 1153197, EPI_ISL_1153199 to 1153239, EPI_ISL_1153241 to 1153242, EPI_ISL_1157070 to 1157083, EPI_ISL_1157085 to 1157099, EPI_ISL_1285843 to 1285852, EPI_ISL_1285855 to 1285857, EPI_ISL_1285859 to 1285884, EPI_ISL_1285886 to 1285898, EPI_ISL_1285900 to 1285909, EPI_ISL_1285911 to 1285925, EPI_ISL_1286138 to 1286226			
see above	Eurofins LifeCodexx GmbH	Robert Koch Institute	
EPI_ISL_450873 to 450874	Evandro Chagas Institute	Evandro Chagas Institute	A.M.; Barbagelata; E.C.; E.M.A.; Ferreira; G.M.R; J.A.; Junior; L.C.; L.S.; M.C.; Martins; P.S.; Santos; Silva; Sousa; Sousa Junior; Viana; W.D.C.; da Silva
EPI_ISL_1063789	Evandro Chagas Institute	Evandro Chagas Institute Virology	A.M.; Barbagelata; E.C.; E.M.A.; Ferreira; J.A.; Junior; K.C.; L.C.; L.S.; M.C.; P.S.; Pinheiro; Santos; Silva; Sousa; Sousa Junior; W.D.C.; da Silva
EPI_ISL_602282 to 602303	Evangelisches Klinikum Bethel, Institut für Laboratoriumsmedizin, Mikrobiologie und Hygiene	Bielefeld University	Alexander Sczyrba; Christiane Scherer; David Brandt; Jörn Kalinowski; Levin-Joe Klages; Marina Simunovic; Markus Haak; Svenja Vinke; Tobias Busche
EPI_ISL_1114374	Evergreen Health	Seattle Flu Study	Amanda Adler; Barry R. Lutz; Benjamin Pelle; Brian Hiatt; Caitlin R. Wolf; Chris D. Frazar; Deborah A. Nickerson; Elisabeth Brandstetter; Erica Ryke; Geoff Melly; Helen Y. Chu; Janet A. Englund; Jay Shendure; Jover Lee; Kairsten Fay; Kirsten Lacombe; Lea M. Starita; Mark J. Rieder; Matthew Richardson; Matthew Thompson; Melissa Truong; Michael Boeckh; Michael Famulare; Misja Ilcisin; Peter D. Han; Philip Dykema; Romesh Gautom; Scott Lindquist; Thomas R. Sibley; Trevor Bedford
EPI_ISL_962442, EPI_ISL_1222613 to 1222615, EPI_ISL_1222704 to 1222708	Evergreen Healthcare	Seattle Flu Study	Amanda Adler; Barry R. Lutz; Benjamin Pelle; Brian Hiatt; Caitlin R. Wolf; Chris D. Frazar; Deborah A. Nickerson; Elisabeth Brandstetter; Erica Ryke; Geoff Melly; Helen Y. Chu; Janet A. Englund; Jay Shendure; Jover Lee; Kairsten Fay; Kirsten Lacombe; Lea M. Starita; Mark J. Rieder; Matthew Richardson; Matthew Thompson; Melissa Truong; Michael Boeckh; Michael Famulare; Misja Ilcisin; Peter D. Han; Philip Dykema; Romesh Gautom; Scott Lindquist; Thomas R. Sibley; Trevor Bedford
EPI_ISL_548966 to 548971	Expo2020 Emergency Center	Agiomix	Cengiz Yakicier; Cherif Ben Hamada; Rashid Mohammed; Tamer Degheidy; Walaa Allam; Walid Dridi
EPI_ISL_1048246, EPI_ISL_1048248, EPI_ISL_1048254, EPI_ISL_1048259, EPI_ISL_1048266, EPI_ISL_1048270, EPI_ISL_1048276, EPI_ISL_1048279, EPI_ISL_1048281, EPI_ISL_1048283 to 1048284, EPI_ISL_1048298, EPI_ISL_1048304, EPI_ISL_1048307 to 1048309, EPI_ISL_1048312, EPI_ISL_1048314 to 1048315, EPI_ISL_1048324 to 1048335			
see above	FG17 - NRZ Influenzaviren	Robert Koch Institute, Influenza and respiratory viruses FG17 & Bioinformatics MF1, Berlin, Germany	Aleksandar Radonic; Marianne Wedde; Oliver Drechsel; R. Dürrwald; Ralf Duerrwald; Rene Kmiecinski; Stefan Kroeger; Stephan Fuchs; Thorsten Wolff
EPI_ISL_1272814 to 1272822	FL Bur. of Public Health Laboratories-Jacksonville	Centers for Disease Control and Prevention Division of Viral Diseases, Pathogen Discovery	Anna Montmayeur; Anna Uehara; Ben L. Rambo-Martin; Clinton R. Paden; Dhwani Batra; Haibin Wang; Jasmine Padilla; Jing Zhang; Justin Lee; Katie Dillon; Krista Queen; Kristen Knipe; Kristine Lacek; Lori Rowe; Mark Burroughs; Matthew Schmerer; Mili Sheth; Peter W. Cook; Rachel Marine; Sam Shepard; Sarah Nobles; Shoshona Le; Suxiang Tong; Yan Li; Ying Tao
EPI_ISL_424856 to 424857	FL Bur. of Public Health Laboratories-Jacksonville	Pathogen Discovery, Respiratory Viruses Branch, Division of Viral Diseases, Centers for Disease Control and Prevention	Alison S. Laufer Halpin; Anna Uehara; Christopher A. Elkins; Clinton R. Paden; Haibin Wang; Jing Zhang; Krista Queen; Mary S. Keckler; Rachel Marine; Suxiang Tong; Yan Li; Ying Tao
EPI_ISL_1094242, EPI_ISL_1094265, EPI_ISL_1094293, EPI_ISL_1094537 to	FL Bur. of Public Health Laboratories-Jacksonville	Respiratory Viruses Branch, Division of Viral Diseases, Centers for Disease Control and Prevention	Anna Montmayeur; Anna Uehara; Ben L. Rambo-Martin; Clinton R. Paden; Dhwani Batra; Haibin Wang; Jasmine Padilla; Jing Zhang; Justin Lee; Krista Queen; Lori Rowe; Mark Burroughs; Mili Sheth; Peter W. Cook; Rachel Marine; Sarah Nobles; Suxiang Tong; Yan Li; Ying Tao

1094539, EPI_ISL_1095186, EPI_ISL_1095287			
EPI_ISL_420785	FL Bureau of Health Laboratories Tampa	Pathogen Discovery, Respiratory Viruses Branch, Division of Viral Diseases, Centers for Disease Control and Prevention	Alison S. Laufer Halpin; Anne Uehara; Christopher A. Elkins; Clinton R. Paden; Haibin Wang; Jasmine Padilla; Jing Zhang; Justin Lee; Krista Queen; Mary S. Keckler; Rachel Marine; Suxiang Tong; Yan Li; Ying Tao
EPI_ISL_452110 to 452111, EPI_ISL_452132, EPI_ISL_594461, EPI_ISL_594465	FL Bureau of Public Health Laboratories	Pathogen Discovery, Respiratory Viruses Branch, Division of Viral Diseases, Centers for Disease Control and Prevention	Alison S. Laufer Halpin; Anna Montmayeur; Anna Uehara; Christopher A. Elkins; Clinton Paden; Clinton R. Paden; Haibin Wang; Jing Zhang; Julu Bhatnagar; Krista Queen; Mary S. Keckler; Rachel Marine; Suxiang Tong; Yan Li; Ying Tao; Zachary Weiner
EPI_ISL_1272971	FL Bureau of Public Health Laboratories-Miami	Centers for Disease Control and Prevention Division of Viral Diseases, Pathogen Discovery	Anna Montmayeur; Anna Uehara; Ben L. Rambo-Martin; Clinton R. Paden; Dhwani Batra; Haibin Wang; Jasmine Padilla; Jing Zhang; Justin Lee; Katie Dillon; Krista Queen; Kristen Knipe; Kristine Lacek; Lori Rowe; Mark Burroughs; Matthew Schmerer; Mili Sheth; Peter W. Cook; Rachel Marine; Sam Shepard; Sarah Nobles; Shoshona Le; Suxiang Tong; Yan Li; Ying Tao
EPI_ISL_1169489	FL Bureau of Public Health Laboratories-Miami	Genomics and Discovery, Respiratory Viruses Branch, Division of Viral Diseases, Centers for Disease Control and Prevention	Anna Montmayeur; Anna Uehara; Ben L. Rambo-Martin; Clinton R. Paden; Dhwani Batra; Haibin Wang; Jasmine Padilla; Jing Zhang; Justin Lee; Krista Queen; Lori Rowe; Mark Burroughs; Mili Sheth; Peter W. Cook; Rachel Marine; Sarah Nobles; Suxiang Tong; Yan Li; Ying Tao
EPI_ISL_424853 to 424854	FL Bureau of Public Health Laboratories-Miami	Pathogen Discovery, Respiratory Viruses Branch, Division of Viral Diseases, Centers for Disease Control and Prevention	Alison S. Laufer Halpin; Anna Uehara; Christopher A. Elkins; Clinton R. Paden; Haibin Wang; Jing Zhang; Krista Queen; Mary S. Keckler; Rachel Marine; Suxiang Tong; Yan Li; Ying Tao
EPI_ISL_1094299, EPI_ISL_1094545 to 1094546, EPI_ISL_1095290 to 1095294	FL Bureau of Public Health Laboratories-Miami	Respiratory Viruses Branch, Division of Viral Diseases, Centers for Disease Control and Prevention	Anna Montmayeur; Anna Uehara; Ben L. Rambo-Martin; Clinton R. Paden; Dhwani Batra; Haibin Wang; Jasmine Padilla; Jing Zhang; Justin Lee; Krista Queen; Lori Rowe; Mark Burroughs; Mili Sheth; Peter W. Cook; Rachel Marine; Sarah Nobles; Suxiang Tong; Yan Li; Ying Tao
EPI_ISL_1272713 to 1272719	FL Bureau of Public Health Laboratories-Tampa	Centers for Disease Control and Prevention Division of Viral Diseases, Pathogen Discovery	Anna Montmayeur; Anna Uehara; Ben L. Rambo-Martin; Clinton R. Paden; Dhwani Batra; Haibin Wang; Jasmine Padilla; Jing Zhang; Justin Lee; Katie Dillon; Krista Queen; Kristen Knipe; Kristine Lacek; Lori Rowe; Mark Burroughs; Matthew Schmerer; Mili Sheth; Peter W. Cook; Rachel Marine; Sam Shepard; Sarah Nobles; Shoshona Le; Suxiang Tong; Yan Li; Ying Tao
EPI_ISL_419559 to 419560, EPI_ISL_424855, EPI_ISL_447841, EPI_ISL_812138, EPI_ISL_812142	FL Bureau of Public Health Laboratories-Tampa	Pathogen Discovery, Respiratory Viruses Branch, Division of Viral Diseases, Centers for Disease Control and Prevention	Alison S. Laufer Halpin; Anna Montmayeur; Anna Uehara; Brian Lynch; Christopher A. Elkins; Clinton R. Paden; Haibin Wang; Jasmine Padilla; Jing Zhang; Justin Lee; Krista Queen; Mary S. Keckler; Peter Cook; Rachel Marine; Suxiang Tong; Yan Li; Ying Tao
EPI_ISL_1094315, EPI_ISL_1094540 to 1094541, EPI_ISL_1095288 to 1095289	FL Bureau of Public Health Laboratories-Tampa	Respiratory Viruses Branch, Division of Viral Diseases, Centers for Disease Control and Prevention	Anna Montmayeur; Anna Uehara; Ben L. Rambo-Martin; Clinton R. Paden; Dhwani Batra; Haibin Wang; Jasmine Padilla; Jing Zhang; Justin Lee; Krista Queen; Lori Rowe; Mark Burroughs; Mili Sheth; Peter W. Cook; Rachel Marine; Sarah Nobles; Suxiang Tong; Yan Li; Ying Tao
EPI_ISL_576173	FL Department of Health-Bureau of Epidemiology	Pathogen Discovery, Respiratory Viruses Branch, Division of Viral Diseases, Centers for Disease Control and Prevention	Anna Uehara; Brian Lynch; Clinton R. Paden; Haibin Wang; Jing Zhang; Krista Queen; Peter Cook; Suxiang Tong; Yan Li; Ying Tao
EPI_ISL_1036217	FONDAZIONE PICCOLA OPERA CARITAS	Istituto Zooprofilattico Sperimentale dell'Abruzzo e Molise "G. Caporale"	Ancora M; Calistri P; Cammà C; Curini V; Di Domenico M; Di Pasquale A; Lorusso A; Mangone I; Marcacci M; Puglia I; Rinaldi A; Savini G; Scialabba S
EPI_ISL_833046, EPI_ISL_833068 to 833075	FPAM	Istituto Zooprofilattico Sperimentale dell'Abruzzo e Molise "G. Caporale"	Ancora M; Calistri P; Cammà C; Curini V; Delli Compagni E; Di Domenico M; Di Pasquale A; Lorusso A; Mangone I; Marcacci M; Puglia I; Rinaldi A; Savini G
EPI_ISL_428851 to 428852	FSBSI "Chumakov Federal Scientific Center for Research and Development of Immune-and-Biological Products of Russian Academy of Sciences"	FSBSI "Chumakov Federal Scientific Center for Research and Development of Immune-and-Biological Products of Russian Academy of Sciences" & NRC "Kurchatov institute"	Anastasia Berestovskaya; Anastasia Piniaeva; Anna Shishova; Aydar Ishmukhametov; Denis Protsenko; Egor Prokhorchuk; Georgy Ignatyev; Liubov Kozlovskaya; Mikhail Rychev
EPI_ISL_1195273, EPI_ISL_1195281	FUNDACAO DE SAUDE PUBLICA SAO CAMILO DE ESTEIO	Epiclin	Ana Paula Muterle; Carolina Comerlato; Eliana Márcia Da Ros Wendland; Fernando Hayashi Sant'Anna; Janira Prichula; Juliana Comerlato
EPI_ISL_1195272	FUNDACAO HOSPITALAR DE SAPUCAIA DO SUL	Epiclin	Ana Paula Muterle; Carolina Comerlato; Eliana Márcia Da Ros Wendland; Fernando Hayashi Sant'Anna; Janira Prichula; Juliana Comerlato
EPI_ISL_845626	FUNDACION CARDIOINFANTIL	Instituto Nacional de Salud - Dirección de Investigación en Salud Pública	Carlos Franco-Muñoz; Diego A. Álvarez-Díaz; Diego Andrés Prada; Gerardo Santamaría; Jonathan Reales; Julian Naizaque; Katherine Laiton-Donato; Magdalena Wiesner; Marcela Mercado-Reyes; Maria T. Herrera-Sepúlveda; Martha Lucia Ospina Martinez; Mauricio Pacheco-Montealegre; Paola Muñoz-Laiton; Sheryll Corchuelo
EPI_ISL_445277	FUNDACION DE SALUD EL TENIENTE	Instituto de Salud Publica de Chile	Alejandra Acevedo; Andrés E Castillo; Bárbara Parra; Carolina Tambley; Gabriel Leal; Jaime Lagos; Jorge Fernandez; Loredana Arata; Patricia Bustos; Paz Tapia; Rodrigo Fasce; Winston Andrade
EPI_ISL_906543	FUNDACION HOSPITAL SAN PEDRO	Instituto Nacional de Salud- Dirección de Investigación en Salud Pública, Universidad de los Andes- Applied genomics research group, Vicerrectoria de Investigación y Creación, Universidad de los Andes- Systems and Computing Engineering Department	Carlos Franco-Muñoz; Diego A. Álvarez-Díaz; Diego Andrés Prada; Gerardo Santamaría Jorge Duitama; Héctor Alejandro Ruiz-Moreno; Jhonnatan Reales-González; Jorge Ivan Diaz; Julian Naizaque; Katherine Laiton-Donato; Laura Natalia Gonzalez; Magdalena Wiesner; Marcela Mercado-Reyes; Maria T. Herrera-Sepúlveda; Martha Lucia Ospina Martinez; Mauricio Pacheco-Montealegre; Sheryll Corchuelo; Silvia Restrepo-Restrepo
EPI_ISL_417010, EPI_ISL_418252	FUNDACION JIMENEZ DIAZ	Instituto de Salud Carlos III	A. Monzón; F. Casas; I. Fernández Roblas, R.; I. Jiménez; Iglesias-Caballero; M. Camarero; M. Cuesta; M. González-Esguevillas; M. Molinero Calamita; M. Zaballos; P. Jiménez; S. Juliá; S. Pozo; S. Varona
EPI_ISL_1220058 to 1220060	FUNDACION VALLE DEL LILI	Instituto Nacional de Salud- Dirección de Investigación en Salud Pública	Carlos Franco-Muñoz; Diego A. Álvarez-Díaz; Diego Andrés Prada; Gerardo Santamaría; Hector Alejandro Ruiz-Moreno; Jhonnatan Reales-González; Julian Naizaque; Katherine Laiton-Donato; Magdalena Wiesner; Marcela Mercado-Reyes.; Maria T. Herrera-Sepúlveda; Martha Lucia Ospina Martínez; Sheryll Corchuelo
EPI_ISL_468747 to 468751	Facultad de Medicina UC	Center for Mathematical Modeling and Center for Genome Regulation. Santiago, Chile	Allende ML; Ferres M.; Gaete A; González M; Maass A; Palma R; Travisany D; Urra C; Varas M
EPI_ISL_804016 to 804017	Facultad de Medicina, Universidad de Atacama	Facultad de Ciencias de la Vida, UNAB	Claudio Meneses; César Echeverría; Dayán Sanhueza; Eduardo Castro; Jorge Olivares; Macarena Bastías; Sebastián Wolter; Waldo Díaz
EPI_ISL_1273097 to 1273102	Faculty of Medicine, Al-Quds University	Faculty of Medicine, Al-Quds University	Al-Jawabreh, A.; Dumaidi, K.; Ereqat, S.; Nasereddin, A.
EPI_ISL_752605	Faculty of Medicine, Al-Quds University	Faculty of Medicine, Al-Quds University	A. and Al-Jawabreh, A.; Ereqat, S.; Nasereddin
EPI_ISL_510533 to 510534	Faculty of Medicine, Bursa Uludag University	Bursa Uludag University	Alemdar, A.; S.G.; Saglik, I.; Temel; Özemri Sa, .
EPI_ISL_1038997	Fair Haven CHC	Grubaugh Lab - Yale School of Public Health	Anderson Brito; Anne Wyllie; Annie Watkins; Chaney Kalinich; Chantal Vogels; Isabel Ott; Joseph Fauver; Mallery Breban; Mary Petrone; Nathan Grubaugh; Tara Alpert
EPI_ISL_526686 to 526688, EPI_ISL_548243 to 548244	Faith Laboratory, Immunology Institute, Icahn School of Medicine at Mount Sinai	van Bakel Laboratory, Genetics and Genomics Sciences, Icahn School of Medicine at Mount Sinai	Alexandra Livanos; Alice Chen-Liaw; Ana S. Gonzalez-Reiche; Benjamin R. tenOever; Daisy Hoagland; Divya Jha; Fatima Amanat; Florian Krammer; Francesca Cossarini; Gerold Bongers; Graham J. Britton; Gustavo Martinez-Delgado; Harm Van Bakel; Ilaria Mogno; Joseph Eggers; Lauren Tal Grinspan; Marla C. Dubinsky; Matthew P. Spindler; Michael Tankelevich; Miriam Merad; Rebekah E. Dixon; Saurabh Mehandru and Jeremiah J. Faith; Sophia Sui; Tamar Plitt
EPI_ISL_640033, EPI_ISL_640050, EPI_ISL_640088, EPI_ISL_640090, EPI_ISL_640116, EPI_ISL_640121, EPI_ISL_640123 to 640125, EPI_ISL_1040681 to 1040682, EPI_ISL_1040739 to 1040740, EPI_ISL_1040775, EPI_ISL_1040777, EPI_ISL_1040779 to 1040780			
see above	False Bay Hospital wc FBH	NHLS/UCT	Arash Iranzadeh; Bruna Galvao; Carolyn Williamson; Deelan Doolabh; Diana Hardie; Innocent Mudau; Kruger Marais; Lynn Tyers; Marvin Hsiao; Stephen Korsman
EPI_ISL_960105, EPI_ISL_960110, EPI_ISL_960112	False Bay Hospital wc FBH	National Health Laboratory Service/UCT	Arash Iranzadeh; Bruna Galvao; Carolyn Williamson; Deelan Doolabh; Diana Hardie; Innocent Mudau; Kruger Marais; Lynn Tyers; Marvin Hsiao; Stephen Korsman
EPI_ISL_536400	Fareham Creek Veterinary Surgery	MRC-University of Glasgow Centre for Virus Research	Ana Da Silva Filipe; Andrew Stevenson; Brian J Willett; Bryn Tennant; Daniel G Streicker; Daniel Goldfarb; Darcy Herrity; David L Robertson and William Weir; Dawn Dunbar; Emma MacDonald; Fiona Howie; Frazer Bell; Ilaria Epifano; Lynn Stevenson; Margaret J Hosie; Michael McDonald; Natasha Johnson; Pablo R Murcia; Richard J Orton; Ruth F Jarrett; Vanessa Herder
EPI_ISL_1257860 to 1257862	Farmalab Indoutama	National Institute of Health Research and Development	Arie Ardiansyah Nugraha; Hana Apsari Pawestri; Hartanti Dian Ikawati; Kartika Dewi Puspa; Krisna Pangesti; Nelly Puspandari; Subangkit; Vivi Setiawaty
EPI_ISL_1048372, EPI_ISL_1048375,	Farmalab Indoutama Jakarta	National Institute of Health Research and Development	Arie Ardiansyah Nugraha; Hana Apsari Pawestri; Hartanti Dian Ikawati; Kartika Dewi Puspa; Krisna Nur Andriana Pangesti; Nelly Puspandari; Subangkit;

EPI_ISL_1048381			Vivi Setiawaty.
EPI_ISL_526935 to 526948, EPI_ISL_614295 to 614317, EPI_ISL_854594	Faroese National Reference Laboratory for Fish and Animal Diseases	Faroese National Reference Laboratory for Fish and Animal Diseases	Arnfinnur Kallsberg Junior; Debes Hammershaimb Christiansen; Maria Marjunardóttir Dahl; Petra Elisabeth Petersen
EPI_ISL_451963 to 451970	Federal Budget Institution of Science, State Research Center for Applied Microbiology & Biotechnology	Federal Budget Institution of Science, State Research Center for Applied Microbiology & Biotechnology	Abaimova A; Bakhteeva I; Blagodatskikh S; Bogun A; Borzilov A; Chekan L; Chernysh S; Denisenko E; Dentovskaya S; Detushev K; Detusheva E; Dyatlov I; Firstova V; Frolov V; Fursov M; Fursova N; Galkina E; Gapelchenkova T; Goncharova J; Gorbatov A; Hlyntseva A; Ivanov S; Kalmantayev T; Kalmantayeva O; Kanashenko M; Kartsev N; Kartseva A; Khomyakov A; Khramov M; Kislichkina A; Kolchanova A; Koroleva-Ushakova A; Kosilova I; Krasilnikova E; Kuzin V; Kuzina E; Makarova M; Marin M; Novikova T; Platonov M; Podkopaev Y; Ryabko A; Shaikhutdinova R; Shemyakin I; Shishkina L; Silkina M; Sizova A; Skryabin Y; Slukin P; Slukina N; Solomentsev V; Solovieva A; Teymurazov M; Timofeev V; Titareva G; Trunyakova A; Tyurin E; Vagayskaya A; Zeninskaya N; Zhumakaev R
EPI_ISL_1181469, EPI_ISL_1219138	Federal University of Mato Grosso (UFMT)	Laboratory of Respiratory Viruses and Measles, Oswaldo Cruz Institute, FIOCRUZ	Alice Sampaio Rocha; Ana Carolina Mendonca; Anna Carolina Paixao; Fernando Motta; Luciana Appolinario; Marilda Siqueira on behalf of the Fiocruz COVID-19 Genomic Surveillance Network; Paola Resende; Renata Dezengrini; Renata Serrano Lopes
EPI_ISL_1181364, EPI_ISL_1181435 to 1181436, EPI_ISL_1181443, EPI_ISL_1181450	Federal University of Southern Bahia (UFSB - Universidade Federal do Sul da Bahia)	Laboratory of Respiratory Viruses and Measles, Oswaldo Cruz Institute, FIOCRUZ	Alice Sampaio Rocha; Ana Carolina Mendonca; Anna Carolina Paixao; Fernando Motta; Luciana Appolinario; Marilda Siqueira on behalf of the Fiocruz COVID-19 Genomic Surveillance Network; Paola Resende; Renata Serrano Lopes; Thiago Mafra
EPI_ISL_891059, EPI_ISL_960223, EPI_ISL_1114369 to 1114370	FidaLab	Seattle Flu Study	Amanda Adler; Barry R. Lutz; Benjamin Pelle; Brian Hiatt; Caitlin R. Wolf; Chris D. Frazar; Deborah A. Nickerson; Elisabeth Brandstetter; Erica Ryke; Geoff Melly; Helen Y. Chu; Janet A. Englund; Jay Shendure; Jover Lee; Kairsten Fay; Kirsten Lacombe; Lea M. Starita; Mark J. Rieder; Matthew Richardson; Matthew Thompson; Melissa Truong; Michael Boeckh; Michael Famulare; Misja Ilcisin; Peter D. Han; Philip Dykema; Romesh Gautom; Scott Lindquist; Thomas R. Sibley; Trevor Bedford
EPI_ISL_1085172	Fidalab	Hyde Lab	Jennifer L. Hyde; Quinne Feng; Roger Peck
EPI_ISL_1069189	Filinvest Tent Covid Quarantine Facility	Research Institute for Tropical Medicine	Catalino Demetria; Daria Manalo; Edelwisa Mercado; Francisco Gerardo Polotan; Hannah Leah Morito; Inez Andrea Medado; John Leonard Chan; Joseph Hughes; Kirstyn Brunker; Ma Angelica Tujan; Othoniel Jan Onza
EPI_ISL_1081812	First Aide Diagnostic Center	Philippine Genome Center	Alethea R. de Guzman; Anna Ong-Lim; Arianne A. Zamora; Asia Louisa U. Chong; Benedict A. Maralit; Candice Francheska B. Tambaoan; Carlo M. Lapid; Celia Carlos; Cynthia P. Saloma; Devon Ray Pacial; Edsel Maurice Salvaña; El King D. Morado; Eva Maria Cutiongco-de la Paz; Francis A. Tablizo; Irish Coleen A. Asin; Jaime C. Montoya; Jan Michael C. Yap; Jo-Hannah S. Llames; John Q. Wong; Joshua Gregor A. Dizon; Juan Antonio R. Magalang; Karol Sophia Agape R. Padilla; Kenneth M. Kim; Kris P. Punayan; Marc Edsel C. Ayes; Marc Jerrone R. Castro; Maria Sofia L. Yangzon; Marissa Alejandria; Razel Nikka M. Hao; Rianna Patricia S. Cruz; Sheila Mae M. Araiza; and Maria Rosario Singh-Vergeire
EPI_ISL_436505 to 436563, EPI_ISL_445003 to 445050, EPI_ISL_450844 to 450872, EPI_ISL_467667, EPI_ISL_480790 to 480951, EPI_ISL_489710 to 489832, EPI_ISL_495330 to 495410, EPI_ISL_508708 to 508763, EPI_ISL_508765 to 508861, EPI_ISL_509716 to 509798, EPI_ISL_512107, EPI_ISL_512547 to 512596, EPI_ISL_512676 to 512677, EPI_ISL_514139 to 514225, EPI_ISL_517771 to 517957, EPI_ISL_526553 to 526685, EPI_ISL_541338, EPI_ISL_549178 to 549270, EPI_ISL_568587 to 568685, EPI_ISL_568876, EPI_ISL_594277 to 594393, EPI_ISL_594466, EPI_ISL_613712 to 613950, EPI_ISL_614247, EPI_ISL_653098 to 653344, EPI_ISL_666814 to 666869, EPI_ISL_763331 to 763347, EPI_ISL_767416, EPI_ISL_790567 to 790569, EPI_ISL_802724 to 802725, EPI_ISL_848643 to 849147, EPI_ISL_852858 to 852861, EPI_ISL_876520 to 876587, EPI_ISL_876737, EPI_ISL_911518 to 911523, EPI_ISL_918493, EPI_ISL_918495, EPI_ISL_935359 to 935519, EPI_ISL_941927 to 941940, EPI_ISL_965016 to 965024, EPI_ISL_1029974 to 1030251, EPI_ISL_1049859 to 1050007, EPI_ISL_1097347 to 1097445, EPI_ISL_1132133 to 1132366, EPI_ISL_1191462 to 1191586, EPI_ISL_1218653 to 1219001, EPI_ISL_1238766, EPI_ISL_1239883 to 1240070, EPI_ISL_1250852, EPI_ISL_1259401 to 1259404			
see above	Florida Bureau of Public Health Laboratories	Florida Bureau of Public Health Laboratories	Jason Blanton; S. and Blanton, J.; Sarah Schmedes; Schmedes
EPI_ISL_541139 to 541331	Florida Bureau of Public Health Laboratories, Florida Department of Health	Florida Bureau of Public Health Laboratories, Florida Department of Health	Blanton, J.; Schmedes, S.
EPI_ISL_893739	Florida Department of Public Health	Pathogen Discovery, Respiratory Viruses Branch, Division of Viral Diseases, Centers for Disease Control and Prevention	Anna Montmayeur; Anna Uehara; Brian Lynch; Clinton R. Paden; Haibin Wang; Jing Zhang; Krista Queen; Peter Cook; Rachel Marine; Suxiang Tong; Yan Li; Ying Tao
EPI_ISL_917943, EPI_ISL_1097739, EPI_ISL_1098636 to 1098637, EPI_ISL_1098641, EPI_ISL_1192366, EPI_ISL_1192369 to 1192371, EPI_ISL_1197058 to 1197061, EPI_ISL_1198005, EPI_ISL_1303426, EPI_ISL_1303474 to 1303479, EPI_ISL_1303484 to 1303488, EPI_ISL_1307636 to 1307637			
see above	Flow Diagnostics Lab	Los Angeles County PHL	P. Hemarajata et al.
EPI_ISL_977120 to 977144, EPI_ISL_1253710 to 1253718, EPI_ISL_1254009 to 1254012	Flow Health	Infectious Disease Program, Broad Institute of Harvard and MIT	Adams, G.; B.L.; B.W.; Bauer, M.; Birren; Carter, A.; Chaluvadi, S.; D.J.; DeRuff, K.; Gallagher, G.; Gladden-Young, A.; J.E.; K.J.; Lagerborg, K.; Lemieux; Loreth, C.; MacInnis; Normandin, E.; P.C.; Park; Reilly, S.; Rudy, M.; Siddle; Smole, S.; Tomkins-Tinch, C.; and Sabeti
EPI_ISL_631563, EPI_ISL_631579, EPI_ISL_631586, EPI_ISL_631589, EPI_ISL_631602, EPI_ISL_631606, EPI_ISL_631614, EPI_ISL_631616, EPI_ISL_631620, EPI_ISL_631638, EPI_ISL_631692, EPI_ISL_631696, EPI_ISL_631699 to 631701, EPI_ISL_631707 to 631708, EPI_ISL_631710, EPI_ISL_631714 to 631715, EPI_ISL_631724 to 631725, EPI_ISL_631727, EPI_ISL_631732, EPI_ISL_631756, EPI_ISL_631766, EPI_ISL_631769 to 631771, EPI_ISL_631773 to 631774, EPI_ISL_631787, EPI_ISL_631826, EPI_ISL_631848, EPI_ISL_631851, EPI_ISL_631855, EPI_ISL_631885, EPI_ISL_631891, EPI_ISL_631893 to 631894, EPI_ISL_631906 to 631908, EPI_ISL_631916, EPI_ISL_631918, EPI_ISL_631923, EPI_ISL_631929, EPI_ISL_631931, EPI_ISL_631955, EPI_ISL_631959 to 631961, EPI_ISL_631964, EPI_ISL_631968, EPI_ISL_631986, EPI_ISL_631989, EPI_ISL_631991, EPI_ISL_632003 to 632004, EPI_ISL_632010, EPI_ISL_632018 to 632020, EPI_ISL_632022, EPI_ISL_632024, EPI_ISL_632027, EPI_ISL_632034, EPI_ISL_632087, EPI_ISL_632097, EPI_ISL_632103 to 632105, EPI_ISL_632119 to 632120, EPI_ISL_632122, EPI_ISL_632124 to 632127, EPI_ISL_632132 to 632134, EPI_ISL_632137 to 632140, EPI_ISL_632144, EPI_ISL_632174, EPI_ISL_632181, EPI_ISL_632185, EPI_ISL_632190, EPI_ISL_632194, EPI_ISL_632196 to 632197, EPI_ISL_632211 to 632213, EPI_ISL_632220, EPI_ISL_632222 to 632226, EPI_ISL_632236 to 632237, EPI_ISL_632947, EPI_ISL_632951 to 632952, EPI_ISL_632954 to 632956, EPI_ISL_632960, EPI_ISL_632966 to 632968, EPI_ISL_632973, EPI_ISL_633070			
see above	Flushing Hospital Medical Center	New York City Public Health Laboratory	Jade Wang; et al.
EPI_ISL_470882, EPI_ISL_500776 to 500778, EPI_ISL_590883 to 590884, EPI_ISL_590899, EPI_ISL_590981, EPI_ISL_590985, EPI_ISL_591008, EPI_ISL_635063 to 635065, EPI_ISL_635098, EPI_ISL_635127, EPI_ISL_635167, EPI_ISL_635170, EPI_ISL_635173, EPI_ISL_635176 to 635177, EPI_ISL_708073 to 708078, EPI_ISL_708176 to 708179, EPI_ISL_738313, EPI_ISL_775323 to 775326, EPI_ISL_775333, EPI_ISL_775362 to 775370, EPI_ISL_860274 to 860276, EPI_ISL_964959, EPI_ISL_964995, EPI_ISL_1013454, EPI_ISL_1118282, EPI_ISL_1118428 to 1118433, EPI_ISL_1118477, EPI_ISL_1118615, EPI_ISL_1192162, EPI_ISL_1192238 to 1192242, EPI_ISL_1192244 to 1192245, EPI_ISL_1192247 to 1192248, EPI_ISL_1192307 to 1192310, EPI_ISL_1251313 to 1251316, EPI_ISL_1251416, EPI_ISL_1251677 to 1251681, EPI_ISL_1251802, EPI_ISL_1251805, EPI_ISL_1251807, EPI_ISL_1251809 to 1251811			
see above	Foerde Hospital, Department of Microbiology	Norwegian Institute of Public Health, Department of Virology	Atiya R Ali; Debech Nadia; Engebretsen Serina Beate; Engebretsen Serina Beate Atiya R Ali; Garcia Llorente Ignacio; Hilde Elshaug; Hilde Vollan; Ignacio Garcia Llorente; Jon Bråte; Kamilla Heddeland Instefjord; Karoline Bragstad; Kathrine Stene-Johansen; Marie Paulsen Madsen; Olav Hungnes; Pedersen Benedikte Nevjen; Rasmus Riis Kopperud; Serina B Engebretsen
EPI_ISL_455903, EPI_ISL_475152, EPI_ISL_476139	Folkhalsomyndigheten	The Public Health Agency of Sweden	Anna Risberg; Anna-Malin Linde; Karin Tegmark-Wisell; Maria Lind Karlberg; Mattias Haukland; Olov Svartstrom; Oskar Karlsson Lindsjo; Petra Edquist; Reza Advani; Sandra Broddesson; Shamam Muradrasoli
EPI_ISL_434650, EPI_ISL_475536, EPI_ISL_475541	Follinge Halsocentral	The Public Health Agency of Sweden	Anna Risberg; Anna-Malin Linde; Karin Tegmark-Wisell; Kerstin Persson Moberg; Maria Lind Karlberg; Mattias Haukland; Mia Brytting; Olov Svartstrom; Oskar Karlsson Lindsjo; Reza Advani; Sandra Broddesson; Theresa Enkirch
EPI_ISL_581455, EPI_ISL_581462, EPI_ISL_581472, EPI_ISL_581486 to 581493	Fondation Congolaise pour la recherche medicale (FCRM)	NGS Competence Center Tübingen, Institut für Medizinische Mikrobiologie und Hygiene, Universitätsklinikum Tübingen	Angel Angelov
EPI_ISL_912353 to 912394	Fondation Congolaise pour la recherche medicale (FCRM), Francine Ntoumi	NGS Competence Center Tuebingen, Institut für Medizinische Mikrobiologie und Hygiene, Universitaetsklinikum Tübingen	Angel Angelov
EPI_ISL_902756, EPI_ISL_965137, EPI_ISL_965139, EPI_ISL_1009024 to 1009027, EPI_ISL_1295613	Fondazione Policlinico Universitario "A. Gemelli" IRCCS	INMI Lazzaro Spallanzani IRCCS	A Di Caro; B Bartolini; C.E.M Gruber; CEM Gruber; E Giombini; F Messina; F Santini; G Bonfiglio; M Rueca; M Sanguinetti; M. Rueca; MR Capobianchi; O Butera; P Cattani
EPI_ISL_420145	Forde Hospital Department of Microbiology	Norwegian Institute of Public Health, Department of Virology	Hilde Elshaug; Kamilla Heddeland Instefjord; Karoline Bragstad; Kathrine Stene-Johansen; Olav Hungnes
EPI_ISL_413586 to 413587	Foundation Elisabeth-Tweesteden Ziekenhuis	Erasmus Medical Center	Anne van der Linden; Annemiek van der Eijk; Aura Timen; Bas Oude Munnink; Claudia Schapendonk; Corien Swaan; Corine GeurtsvanKessel; David Nieuwenhuijse; Irina Chestakova; Jeroen van Kampen; Jolanda Voermans; Madelief Mollers; Manon Haverkate; Marion Koopmans; Mark Pronk; Mart Stein; Pascal Lexmond; Reina Sikkema; Richard Molenkamp; Sandra Kengne Kamga Mobou; on behalf of the Dutch national COVID-19 response team.
EPI_ISL_413565	Foundation Pamm	Erasmus Medical Center	Anne van der Linden; Annemiek van der Eijk; Aura Timen; Bas Oude Munnink; Claudia Schapendonk; Corien Swaan; Corine GeurtsvanKessel; David Nieuwenhuijse; Irina Chestakova; Jeroen van Kampen; Jolanda Voermans; Madelief Mollers; Manon Haverkate; Marion Koopmans; Mark Pronk; Mart Stein; Pascal Lexmond; Reina Sikkema; Richard Molenkamp; Sandra Kengne Kamga Mobou; on behalf of the Dutch national COVID-19 response team.
EPI_ISL_672423 to 672425, EPI_ISL_1235371, EPI_ISL_1235408, EPI_ISL_1235413, EPI_ISL_1235422, EPI_ISL_1235560, EPI_ISL_1235607			
see above	Fresno County Public Health Laboratory	Chan-Zuckerberg Biohub	CZB Cliahub Consortium
EPI_ISL_491111 to 491112	Friedrich-Loeffler-Institut, Laboratory for NGS and Microarray	Friedrich-Loeffler-Institut, Laboratory for NGS and Microarray	Dirk Höper; Laboratory for NGS and Microarray Diagnostics

	Diagnostics	Diagnostics	
EPI_ISL_411060, EPI_ISL_411066, EPI_ISL_431118, EPI_ISL_431180, EPI_ISL_431240, EPI_ISL_431292, EPI_ISL_431779 to 431785			
see above	Fujian Center for Disease Control and Prevention	Fujian Center for Disease Control and Prevention	Chen Wei; He Wenxiang; Huang Zhimiao; Lin Qi; Weng Yuwei; Zhang Yanhua
EPI_ISL_862839, EPI_ISL_862857, EPI_ISL_862868	Fujita Health University Bantane Hospital	Fujita health University, School of Medicine, Department of Microbiology	Masahiro suzuki; Yohei Doi
EPI_ISL_707928	Fujita Health University Hospital	Fujita Health University School of Medicine, Department of Microbiology	Aki Sakurai; Masahiro Suzuki; Yohei Doi
EPI_ISL_862837 to 862838, EPI_ISL_862840 to 862842	Fujita Health University Hospital	Fujita health University, School of Medicine, Department of Microbiology	Masahiro suzuki; Yohei Doi
EPI_ISL_728000	Fujita Health University Okazaki Medical Center	Department of Virology and Parasitology, Fujita Health University School of Medicin	Aki Sakurai; Masahiro suzuki; Satoshi Komoto; Takayuki Murata; Takuma Ishihara; Tomihiko Ide; Yohei Doi
EPI_ISL_718273, EPI_ISL_727998 to 727999, EPI_ISL_728153 to 728156, EPI_ISL_728159	Fujita Health University Okazaki Medical Center	Department of Virology and Parasitology, Fujita Health University School of Medicine	Aki Sakurai; Masahiro suzuki; Satoshi Komoto; Takayuki Murata; Takuma Ishihara; Tomihiko Ide; Yohei Doi
EPI_ISL_728206 to 728208	Fujita Health University Okazaki Medical Center	Fujita health University, Department of Microbiology	Aki Sakurai; Masahiro suzuki; Satoshi Komoto; Takayuki Murata; Takuma Ishihara; Tomihiko Ide; Yohei Doi
EPI_ISL_479967 to 479978, EPI_ISL_480120 to 480168, EPI_ISL_480225	Fukui Prefectural Institute of Public Health and Environmental Science	Pathogen Genomics Center, National Institute of Infectious Diseases	Hajime Kamiya; Kentaro Itokawa; Makoto Kuroda; Masanori Hashino; Miho Toho; Motoi Suzuki; Rina Tanaka; Tsuyoshi Sekizuka
EPI_ISL_1098660 to 1098754	Fukuoka Institute of Health and Environmental Sciences	Fukuoka Institute of Health and Environmental Sciences	Akira Ohishi; Asako Nakamura; Chiharu Katamune; Hiroaki Shigemura; Kentaro Itokawa; Makoto Kuroda; Masanori Hashino; Mitsuhiro Hamasaki; Rina Tanaka; Saori Ueda; Takayuki Kobayashi; Tsuyoshi Sekizuka; Yoshiki Etoh; Yuki Ashizuka
EPI_ISL_684408 to 684483, EPI_ISL_684634 to 684692, EPI_ISL_684723 to 684727, EPI_ISL_685918 to 685924, EPI_ISL_687533 to 687560, EPI_ISL_688612, EPI_ISL_688619 to 688623, EPI_ISL_688634, EPI_ISL_688897 to 688968, EPI_ISL_689460 to 689496, EPI_ISL_689646, EPI_ISL_689692 to 689697, EPI_ISL_689759 to 689825, EPI_ISL_690432 to 690526, EPI_ISL_690982 to 690986, EPI_ISL_691550 to 691551, EPI_ISL_691735, EPI_ISL_691756, EPI_ISL_691764 to 691765, EPI_ISL_692048 to 692050, EPI_ISL_692219 to 692229, EPI_ISL_692442 to 692497, EPI_ISL_894278 to 894279, EPI_ISL_894287, EPI_ISL_894301, EPI_ISL_894672 to 894816, EPI_ISL_895186 to 895188, EPI_ISL_902208			
see above	Fukuoka Institute of Health and Environmental Sciences	Pathogen Genomics Center, National Institute of Infectious Diseases	Kentaro Itokawa; Makoto Kuroda; Masanori Hashino; Rina Tanaka; Tsuyoshi Sekizuka
EPI_ISL_955261, EPI_ISL_955263, EPI_ISL_984753 to 984754, EPI_ISL_984948, EPI_ISL_1005583 to 1005654, EPI_ISL_1009868 to 1010307, EPI_ISL_1010309 to 1010698, EPI_ISL_1010737 to 1011508, EPI_ISL_1026866 to 1027247, EPI_ISL_1032946 to 1033023, EPI_ISL_1068395 to 1068592, EPI_ISL_1068594 to 1068681, EPI_ISL_1068683 to 1068848, EPI_ISL_1068852 to 1068926, EPI_ISL_1068930 to 1068995, EPI_ISL_1069000 to 1069075, EPI_ISL_1069085 to 1069152, EPI_ISL_1095645 to 1095912, EPI_ISL_1095914 to 1096029, EPI_ISL_1132845 to 1132970, EPI_ISL_1134840 to 1134903, EPI_ISL_1164360 to 1164438, EPI_ISL_1172046 to 1172109, EPI_ISL_1195947 to 1195949, EPI_ISL_1231437 to 1231530, EPI_ISL_1231533, EPI_ISL_1231537, EPI_ISL_1231541, EPI_ISL_1231545, EPI_ISL_1231549, EPI_ISL_1231553, EPI_ISL_1231557, EPI_ISL_1231561, EPI_ISL_1231565, EPI_ISL_1231569, EPI_ISL_1231573, EPI_ISL_1231577, EPI_ISL_1231581, EPI_ISL_1231585, EPI_ISL_1231590, EPI_ISL_1231594, EPI_ISL_1231598, EPI_ISL_1231602, EPI_ISL_1231606, EPI_ISL_1231610, EPI_ISL_1231615, EPI_ISL_1231618, EPI_ISL_1231623, EPI_ISL_1231626, EPI_ISL_1231630, EPI_ISL_1231634, EPI_ISL_1231638, EPI_ISL_1231642, EPI_ISL_1231646, EPI_ISL_1231651, EPI_ISL_1231655, EPI_ISL_1231659, EPI_ISL_1231663, EPI_ISL_1231666, EPI_ISL_1231670, EPI_ISL_1231675, EPI_ISL_1231678, EPI_ISL_1231682, EPI_ISL_1231686, EPI_ISL_1231690, EPI_ISL_1231694, EPI_ISL_1231698, EPI_ISL_1231702, EPI_ISL_1231706, EPI_ISL_1231710, EPI_ISL_1231714, EPI_ISL_1231719, EPI_ISL_1231723, EPI_ISL_1231727, EPI_ISL_1231730, EPI_ISL_1231735, EPI_ISL_1231739, EPI_ISL_1231743, EPI_ISL_1231747, EPI_ISL_1231751, EPI_ISL_1231755, EPI_ISL_1231758, EPI_ISL_1231762, EPI_ISL_1231767, EPI_ISL_1231771, EPI_ISL_1231774 to 1231913, EPI_ISL_1232029 to 1232192, EPI_ISL_1251260, EPI_ISL_1251273, EPI_ISL_1252050			
see above	Fulgent Genetics	Fulgent Genetics	Becky Tsai; Benafsh Sapra; Doreen Ng; Harry Gao; James Xie; John Gao; Joseph Fierro; Mickey Li; Yan Meng
EPI_ISL_492074	Functional Genomics Core University of South Carolina / Prisma Health-Midlands	Functional Genomics Core, University of South Carolina,	Alyssa Clay-Glimour; B.Celia Cui; Diego Altomare; Hao Ji; Helmut Albrecht; Mengqian Chen; Michael Shtutman; Michael Wyatt; Phillip Buckhaults
EPI_ISL_491481	Functional Genomics Core University of South Carolina / Prisma Health-Midlands	Functional Genomics Core, Center For Targeted Therapeutics,	Alyssa Clay-Glimour; B.Celia Cui; Diego Altomare; Hao Ji; Helmut Albrecht; Mengqian Chen; Michael Shtutman; Michael Wyatt; Phillip Buckhaults
EPI_ISL_493209 to 493212, EPI_ISL_493427, EPI_ISL_494553 to 494556, EPI_ISL_494558	Functional Genomics Core University of South Carolina / Prisma Health-Midlands	Functional Genomics Core, University of South Carolina	Alyssa Clay-Glimour; B.Celia Cui; Diego Altomare; Hao Ji; Helmut Albrecht; Mengqian Chen; Michael Shtutman; Michael Wyatt; Phillip Buckhaults
EPI_ISL_493129 to 493130, EPI_ISL_493426	Functional Genomics Core University of South Carolina / Prisma Health-Midlands	Functional Genomics Core, University of South Carolina,	Alyssa Clay-Glimour; B.Celia Cui; Diego Altomare; Hao Ji; Helmut Albrecht; Mengqian Chen; Michael Shtutman; Michael Wyatt; Phillip Buckhaults
EPI_ISL_419235 to 419237	Fundacion Jimenez Diaz	Instituto de Salud Carlos III	Camarero, S.; Casas, I.; Cuesta, I.; Fernández, R.; González-Esguevillas, M.; Iglesias-Caballero, M.; Jiménez, M.; Jiménez, P.; Juliá, M.; Molinero Calamita, M.; Monzón, S.; Pozo, F.; Varona, S.; Zaballos, A.
EPI_ISL_462447 to 462449	Fundació Lluita contra la SIDA (FLSida)/Hospital Universitari Germans Trias i Pujol	IrsiCaixa AIDS Research Lab	Bonaventura Clotet; Joaquim Segalés; Jorge Carrillo; Julia Blanco; Lidia Ruiz; Marc Corbacho; Marc Noguera-Julian; Maria Pilar Armengol; Maria Ubals; Mariona Parera; Nuria Izquierdo; Oriol Mitjà; Roger Paredes
EPI_ISL_794649 to 794651, EPI_ISL_794662	Fundación Cardio Infantil	Instituto Nacional de Salud - Dirección de Investigación en Salud Pública	Carlos Franco-Muñoz; Diego A. Álvarez-Díaz; Diego Andrés Prada; Gerardo Santamaría; Jonathan Reales; Julian Naizaque; Katherine Laiton-Donato; Magdalena Wiesner; Marcela Mercado-Reyes; Maria T. Herrera; Martha Lucia Ospina Martinez; Mauricio Pacheco-Montealegre; Paola Muñoz-Laiton; Sheryl Corchuelo
EPI_ISL_1137616 to 1137617, EPI_ISL_1201897	Fundación Cardio Infantil	Instituto Nacional de Salud- Dirección de Investigación en Salud Pública	Carlos Franco-Muñoz; Diego A. Álvarez-Díaz; Diego Andrés Prada; Gerardo Santamaría; Hector Alejandro Ruiz-Moreno; Jhonnatan Reales-González; Julian Naizaque; Katherine Laiton-Donato; Magdalena Wiesner; Marcela Mercado-Reyes.; Maria T. Herrera-Sepúlveda; Martha Lucia Ospina Martínez; Sheryll Corchuelo
EPI_ISL_462478	Fundación Jiménez Díaz	Instituto de Salud Carlos III	A. Monzón; F. Casas; I; I. Jiménez; Iglesias-Caballero; M. Camarero; M. Cuesta; M. González-Esguevillas; M. Molinero Calamita; M. Zaballos; P. Jiménez; R. Fernández; S. Juliá; S. Pozo; S. Varona
EPI_ISL_794654 to 794655, EPI_ISL_794664 to 794665	Fundación Valle del Lili	Instituto Nacional de Salud - Dirección de Investigación en Salud Pública	Carlos Franco-Muñoz; Diego A. Álvarez-Díaz; Diego Andrés Prada; Gerardo Santamaría; Jonathan Reales; Julian Naizaque; Katherine Laiton-Donato; Magdalena Wiesner; Marcela Mercado-Reyes; Maria T. Herrera; Martha Lucia Ospina Martinez; Mauricio Pacheco-Montealegre; Paola Muñoz-Laiton; Sheryl Corchuelo
EPI_ISL_1239124, EPI_ISL_1239137 to 1239139, EPI_ISL_1240642	Fundação Ezequiel Dias	Coordenação Geral de Laboratórios de Saúde Pública (CGLAB)	; Vagner Fonseca et al; Vagner Fonseca et al.
EPI_ISL_1182541, EPI_ISL_1182543 to 1182545, EPI_ISL_1182548 to 1182551, EPI_ISL_1182555, EPI_ISL_1182559 to 1182561, EPI_ISL_1182564, EPI_ISL_1182566, EPI_ISL_1182569 to 1182570, EPI_ISL_1182573 to 1182574, EPI_ISL_1182577 to 1182579, EPI_ISL_1182585 to 1182587, EPI_ISL_1182590 to 1182591, EPI_ISL_1182593, EPI_ISL_1182598, EPI_ISL_1182600 to 1182602, EPI_ISL_1182607, EPI_ISL_1182609, EPI_ISL_1182611 to 1182612, EPI_ISL_1182615, EPI_ISL_1182618, EPI_ISL_1182625			
see above	Fundação Ezequiel Dias (FUNED)	Coordenação Geral de Laboratórios de Saúde Pública (CGLAB/DAEVS/SVS/MS)	Vagner Fonseca; et al.
EPI_ISL_420141, EPI_ISL_420146, EPI_ISL_420313, EPI_ISL_449787 to 449788, EPI_ISL_481208, EPI_ISL_493356 to 493380, EPI_ISL_500768 to 500774, EPI_ISL_500784 to 500790, EPI_ISL_549027 to 549028, EPI_ISL_549038, EPI_ISL_549048 to 549049, EPI_ISL_549053 to 549058, EPI_ISL_549060 to 549069, EPI_ISL_549071 to 549080, EPI_ISL_549082, EPI_ISL_549088, EPI_ISL_549112, EPI_ISL_549119 to 549122, EPI_ISL_549124, EPI_ISL_549132, EPI_ISL_549143, EPI_ISL_549154, EPI_ISL_549165, EPI_ISL_549168, EPI_ISL_590881, EPI_ISL_590898, EPI_ISL_590901 to 590903, EPI_ISL_590912, EPI_ISL_635086 to 635087, EPI_ISL_668396 to 668397, EPI_ISL_668403, EPI_ISL_708035, EPI_ISL_708057 to 708059, EPI_ISL_708126 to 708130, EPI_ISL_759973 to 759974, EPI_ISL_759984 to 759985, EPI_ISL_775271, EPI_ISL_775273 to 775274, EPI_ISL_775279, EPI_ISL_775294, EPI_ISL_775299 to 775300, EPI_ISL_775303 to 775304, EPI_ISL_775427, EPI_ISL_775484, EPI_ISL_775488 to 775490, EPI_ISL_775496 to 775497, EPI_ISL_775524 to 775527, EPI_ISL_796671, EPI_ISL_796722 to 796724, EPI_ISL_796726, EPI_ISL_796730 to 796732, EPI_ISL_842644, EPI_ISL_860213, EPI_ISL_860281, EPI_ISL_906830, EPI_ISL_962901 to 962903, EPI_ISL_964979, EPI_ISL_964989, EPI_ISL_965001 to 965004, EPI_ISL_1013468, EPI_ISL_1013474, EPI_ISL_1013476, EPI_ISL_1013486 to 1013487, EPI_ISL_1013517 to 1013520, EPI_ISL_1034313, EPI_ISL_1040161 to 1040162, EPI_ISL_1040164, EPI_ISL_1058048, EPI_ISL_1058054, EPI_ISL_1058063, EPI_ISL_1073915, EPI_ISL_1073931, EPI_ISL_1073952, EPI_ISL_1110521 to 1110523, EPI_ISL_1110538 to 1110540, EPI_ISL_1118287, EPI_ISL_1118290, EPI_ISL_1118295, EPI_ISL_1118303, EPI_ISL_1118315, EPI_ISL_1118331, EPI_ISL_1118334, EPI_ISL_1118336 to 1118337, EPI_ISL_1118339, EPI_ISL_1118353, EPI_ISL_1118361 to 1118362, EPI_ISL_1118367, EPI_ISL_1118395, EPI_ISL_1118397, EPI_ISL_1118400 to 1118401, EPI_ISL_1118418 to 1118419, EPI_ISL_1118426, EPI_ISL_1118471, EPI_ISL_1118496, EPI_ISL_1118504 to 1118508, EPI_ISL_1118516, EPI_ISL_1118531, EPI_ISL_1118562, EPI_ISL_1118569, EPI_ISL_1118600, EPI_ISL_1118612, EPI_ISL_1118659, EPI_ISL_1118666, EPI_ISL_1118678, EPI_ISL_1118682 to 1118683, EPI_ISL_1118685 to 1118687, EPI_ISL_1186072 to 1186073, EPI_ISL_1186075 to 1186084, EPI_ISL_1186091 to 1186100, EPI_ISL_1186106 to 1186110, EPI_ISL_1186120, EPI_ISL_1186124 to 1186127, EPI_ISL_1186147 to 1186150, EPI_ISL_1186160 to 1186163, EPI_ISL_1186167 to 1186168, EPI_ISL_1186171, EPI_ISL_1186173 to 1186175, EPI_ISL_1186179 to 1186186, EPI_ISL_1192163, EPI_ISL_1192167, EPI_ISL_1192175 to 1192176, EPI_ISL_1192184 to 1192185, EPI_ISL_1192190, EPI_ISL_1192201 to 1192203, EPI_ISL_1192205, EPI_ISL_1192226, EPI_ISL_1192230, EPI_ISL_1192251, EPI_ISL_1192255, EPI_ISL_1192274 to 1192284, EPI_ISL_1192292 to 1192296, EPI_ISL_1251285, EPI_ISL_1251297, EPI_ISL_1251318, EPI_ISL_1251358 to 1251359, EPI_ISL_1251462, EPI_ISL_1251493 to 1251494, EPI_ISL_1251611 to 1251612, EPI_ISL_1251615, EPI_ISL_1251617, EPI_ISL_1251676, EPI_ISL_1251700 to 1251705, EPI_ISL_1251796 to 1251797, EPI_ISL_1251828, EPI_ISL_1251893 to 1251906, EPI_ISL_1251908, EPI_ISL_1251910 to 1251913, EPI_ISL_1251916 to 1251919, EPI_ISL_1251931 to 1251932, EPI_ISL_1251937,			

EPI_ISL_1251939, EPI_ISL_1251942 to 1251952, EPI_ISL_1251994 to 1251996, EPI_ISL_1251998 to 1252001, EPI_ISL_1252005, EPI_ISL_1252016, EPI_ISL_1252018, EPI_ISL_1252021 to 1252022, EPI_ISL_1252025 to 1252027, EPI_ISL_1252031, EPI_ISL_1252036 to 1252038, EPI_ISL_1252042 to 1252043, EPI_ISL_1252048 to 1252049, EPI_ISL_1252486, EPI_ISL_1252488, EPI_ISL_1252490, EPI_ISL_1252493 to 1252496, EPI_ISL_1252500 to 1252501, EPI_ISL_1252546 to 1252548, EPI_ISL_1254545, EPI_ISL_1254555, EPI_ISL_1254571, EPI_ISL_1254586, EPI_ISL_1254593 to 1254594, EPI_ISL_1254608, EPI_ISL_1314453 to 1314457, EPI_ISL_1314483 to 1314484, EPI_ISL_1314524, EPI_ISL_1314529, EPI_ISL_1314536 to 1314537			
see above	Furst Medical Laboratory	Norwegian Institute of Public Health, Department of Virology	Atiya R Ali; Debech Nadia; Engebretsen Serina Beate; Engebretsen Serina Beate Atiya R Ali; Garcia Llorente Ignacio; Hilde Elshaug; Hilde Synnøve Vollan; Hilde Vollan; Ignacio Garcia Llorente; Jon Bråte; Kamilla Heddeland Instefjord; Karoline Bragstad; Kathrine Stene-Johansen; Marie Paulsen Madsen; Olav Hungnes; Pedersen Benedikte Nevjen; Rasmus Riis Kopperud; Serina B Engebretsen
EPI_ISL_522549 to 522550	Félix Guyon Hospital	UMR PIMIT Université de La Réunion	Camille Lebarbenchon; David Wilkinson; Patrick Mavingui
EPI_ISL_912674 to 912676, EPI_ISL_981547, EPI_ISL_982162, EPI_ISL_982301, EPI_ISL_982307 to 982310, EPI_ISL_1085237, EPI_ISL_1085264, EPI_ISL_1085301, EPI_ISL_1085351, EPI_ISL_1085355, EPI_ISL_1085401, EPI_ISL_1110194 to 1110198			
see above	G.H.E.F.Grand Hôpital EST Francilien	Department of Virology, Henri Mondor University Hospital, Assistance Publique Hôpitaux de Paris, Université Paris-Est Créteil, INSERM U955	Alexandre Soulier; Christophe Rodriguez; Elisabeth Trawinski; Guillaume Gricourt; Jean-Michel Pawlotsky; Melissa N'Debi; Slim Fourati; Vanessa Demontant
EPI_ISL_965840 to 965854, EPI_ISL_1225320 to 1225329, EPI_ISL_1238994 to 1239011, EPI_ISL_1262459 to 1262500, EPI_ISL_1278007 to 1278028	GA Department of Public Health	GA Department of Public Health	Aliyah Fields; Cynthia Dixey; Jonathan Edwards; Stacy Reeves; Taylor Smith; Tonia Parrott
EPI_ISL_420786 to 420788	GA Department of Public Health	Pathogen Discovery, Respiratory Viruses Branch, Division of Viral Diseases, Centers for Disease Control and Prevention	Alison S. Laufer Halpin; Anne Uehara; Christopher A. Elkins; Clinton R. Paden; Haibin Wang; Jasmine Padilla; Jing Zhang; Justin Lee; Krista Queen; Mary S. Keckler; Rachel Marine; Suxiang Tong; Yan Li; Ying Tao
EPI_ISL_1272998 to 1273036	GA Department of Public Health Laboratory	Centers for Disease Control and Prevention Division of Viral Diseases, Pathogen Discovery	Anna Montmayeur; Anna Uehara; Ben L. Rambo-Martin; Clinton R. Paden; Dhwani Batra; Haibin Wang; Jasmine Padilla; Jing Zhang; Justin Lee; Katie Dillon; Krista Queen; Kristen Knipe; Kristine Lacek; Lori Rowe; Mark Burroughs; Matthew Schmerer; Mili Sheth; Peter W. Cook; Rachel Marine; Sam Shepard; Sarah Nobles; Shoshona Le; Suxiang Tong; Yan Li; Ying Tao
EPI_ISL_1167938 to 1167988, EPI_ISL_1168001 to 1168006, EPI_ISL_1168015, EPI_ISL_1168020 to 1168036, EPI_ISL_1226082 to 1226091, EPI_ISL_1226105	GA Department of Public Health Laboratory	Genomics and Discovery, Respiratory Viruses Branch, Division of Viral Diseases, Centers for Disease Control and Prevention	; Anna Montmayeur; Anna Uehara; Ben L. Rambo-Martin; Brian Lynch; Clinton R. Paden; Dhwani Batra; Haibin Wang; Jasmine Padilla; Jing Zhang; Justin Lee; Katie Dillon; Krista Queen; Kristen Knipe; Kristine Lacek; Lori Rowe; Mark Burroughs; Matthew Schmerer; Mili Sheth; Peter W. Cook; Rachel Marine; Sam Shepard; Sarah Nobles; Shoshona Le; Suxiang Tong; Yan Li; Ying Tao
EPI_ISL_419556 to 419557, EPI_ISL_424858 to 424864, EPI_ISL_426417 to 426419, EPI_ISL_527658 to 527665, EPI_ISL_566085 to 566107, EPI_ISL_576189 to 576195, EPI_ISL_647987 to 647989, EPI_ISL_648002 to 648003, EPI_ISL_648123, EPI_ISL_681776 to 681828, EPI_ISL_812131, EPI_ISL_812133, EPI_ISL_812136 to 812137, EPI_ISL_812140, EPI_ISL_812143 to 812197, EPI_ISL_955277, EPI_ISL_955288, EPI_ISL_955302 to 955303, EPI_ISL_955316, EPI_ISL_955319 to 955320, EPI_ISL_955329 to 955330, EPI_ISL_955334 to 955336, EPI_ISL_1008910 to 1008915			
see above	GA Department of Public Health Laboratory	Pathogen Discovery, Respiratory Viruses Branch, Division of Viral Diseases, Centers for Disease Control and Prevention	Alison S. Laufer Halpin; Anna Montmayeur; Anna Uehara; Brian Lynch; Christopher A. Elkins; Clinton R. Paden; Haibin Wang; Jasmine Padilla; Jing Zhang; Justin Lee; Krista Queen; Mary S. Keckler; Peter Cook; Rachel Marine; Suxiang Tong; Yan Li; Ying Tao
EPI_ISL_1094255 to 1094256, EPI_ISL_1094284, EPI_ISL_1094312, EPI_ISL_1094364, EPI_ISL_1094425 to 1094427, EPI_ISL_1094429 to 1094432, EPI_ISL_1095097 to 1095107, EPI_ISL_1095109, EPI_ISL_1095111 to 1095112, EPI_ISL_1095114 to 1095119			
see above	GA Department of Public Health Laboratory	Respiratory Viruses Branch, Division of Viral Diseases, Centers for Disease Control and Prevention	Anna Montmayeur; Anna Uehara; Ben L. Rambo-Martin; Clinton R. Paden; Dhwani Batra; Haibin Wang; Jasmine Padilla; Jing Zhang; Justin Lee; Krista Queen; Lori Rowe; Mark Burroughs; Mili Sheth; Peter W. Cook; Rachel Marine; Sarah Nobles; Suxiang Tong; Yan Li; Ying Tao
EPI_ISL_1048313	GA Kreis Wesel	Robert Koch Institute, Influenza and respiratory viruses FG17 & Bioinformatics MF1, Berlin, Germany	Aleksandar Radonic; B. Dittmar; Marianne Wedde; Oliver Drechsel; R. Dürrwald; Ralf Duerrwald; Rene Kmiecinski; Stefan Kroeger; Stephan Fuchs; Thorsten Wolff
EPI_ISL_475058 to 475059, EPI_ISL_495065 to 495066	GAIMS & G K General Hospital	Gujarat Biotechnology Research Centre	; A M Kadri; Afzal Ansari; Apurvasinh Puvar; Babulal Babhoria; Chaitanya Joshi; Dinesh Kumar; Harsh Bakshi; Hitesh Assudani; Janvi Raval; Komal Patel; Labdhi Pandya; Madhvi Joshi; Maharshi Pandya; Monika Gandhi; Neha Rajpara; Nidhi Patel; Nikha Trivedi; Nitin Savaliya; Pinal Trivedi; Pragya Sharma; Pritesh Sabara; R D Dixit; Raghawendra Kumar; Snehal Bagatharia; Zarna Patel; Zuber Saiyed
EPI_ISL_586569 to 586570	GCRI,Ahmedabad	Gujarat Biotechnology Research Centre	A M Kadri; Afzal Ansari; Apurvasinh Puvar; Chaitanya Joshi; Dinesh Kumar; Harsh Bakshi; Harsha Panchal; Janvi Raval; Komal Patel; Labdhi Pandya; Madhvi Joshi; Maharshi Pandya; Monika Gandhi; Nidhi Patel; Nikha Trivedi; Nitin Savaliya; Pinal Trivedi; R D Dixit; Raghawendra Kumar; Shashank Pandya; Zarna Patel; Zuber Saiyed
EPI_ISL_1059950, EPI_ISL_1059988	GERENCIA DE ASISITENCIA SANITARIA DE SORIA	Instituto de Salud Carlos III	A. Monzón; F. Casas; I. Aldea, C.; I. Jiménez; M. Sandonís; P. Zaballos; S. Cuesta; S. Iglesias-Caballero; S. Pozo; S. Varona; V. Camarero; Vázquez-Morón
EPI_ISL_1220316 to 1220321, EPI_ISL_1220323	GH BORDEAUX	CNR Virus des Infections Respiratoires - France SUD	Antonin Bal; Bruno Lina; Gregory Destras; Gwendolyne Burfin; Hadrien Regue; Laurence Josset; Martine Valette; Quentin Semanas
EPI_ISL_1313044, EPI_ISL_1313046, EPI_ISL_1313148, EPI_ISL_1313178, EPI_ISL_1313710	GH BOURG CENTRE PRELEVEMENT	CNR Virus des Infections Respiratoires - France SUD	Antonin Bal; Bruno Lina; Gregory Destras; Gwendolyne Burfin; Hadrien Regue; Laurence Josset; Martine Valette; Quentin Semanas
EPI_ISL_909686	GH Cochin-Broca Hôtel Dieu/Lab Virologie	National Reference Center for Viruses of Respiratory Infections, Institut Pasteur, Paris	Angela Brisebarre; Camille Capel; Etienne Simon-Lorière; L'Honneur Anne-Sophie; Marion Barbet; Maud Vanpeene; Méline Bizard; Sylvie Behillil; Sylvie van der Werf; Vincent Enouf
EPI_ISL_418416, EPI_ISL_508878, EPI_ISL_509003 to 509004	GH Les Portes du Sud	CNR Virus des Infections Respiratoires - France SUD	Alexandre Gaymard; Antonin Bal; Bruno Lina; Carine Moustaud; Florence Morfin-Sherpa; Gregory Destras; Gwendolyne Burfin; Laurence Josset; Martine Valette; Maude Bouscambert-Duchamp; Raphaelle Lamy; Solenne Brun
EPI_ISL_428351 to 428352, EPI_ISL_428363	GH Nord Essonne Service de Biologie clinique	National Reference Center for Viruses of Respiratory Infections, Institut Pasteur, Paris	Angela Brisebarre; Etienne Simon-Lorière; Flora Donati; Marion Barbet; Maud Vanpeene; Mélanie Albert; Méline Bizard; Sylvie Behillil; Sylvie van der Werf; Vincent Enouf
EPI_ISL_1261183, EPI_ISL_1261195 to 1261202, EPI_ISL_1261293 to 1261302, EPI_ISL_1261394, EPI_ISL_1261411	GHC Genetics s.r.o.	National Reference Laboratory for Influenza and Respiratory Viruses CZE	Alexander Nagy; Dusan Trnka; Helena Jirincova; Jaromira Vecerova; Timotej Suri
EPI_ISL_1265757, EPI_ISL_1265820 to 1265824, EPI_ISL_1265836 to 1265838, EPI_ISL_1265849 to 1265850	GHE REUNION	CNR Virus des Infections Respiratoires - France SUD	Antonin Bal; Bruno Lina; Gregory Destras; Gwendolyne Burfin; Hadrien Regue; Laurence Josset; Martine Valette; Quentin Semanas
EPI_ISL_1007633, EPI_ISL_1120825	GHOL	Laboratory of genomics and metagenomics, Institute of Microbiology, University Hospital Centre and University of Lausanne, Switzerland	Claire Bertelli; Damien Jacot; Gilbert Greub; Sébastien Aeby; Trestan Pillonel
EPI_ISL_1265490, EPI_ISL_1265597, EPI_ISL_1265701, EPI_ISL_1265719	GIVORS	CNR Virus des Infections Respiratoires - France SUD	Antonin Bal; Bruno Lina; Gregory Destras; Gwendolyne Burfin; Hadrien Regue; Laurence Josset; Martine Valette; Quentin Semanas
EPI_ISL_602621	GLENEAGLES HONG KONG (CLINICAL LAB)	Hong Kong Department of Health	Chan Rickjason C.W.; Lam Edman T.K.; Mak Gannon C.K.; Tsang Dominic N.C.
EPI_ISL_935973 to 935992, EPI_ISL_1226418, EPI_ISL_1226452, EPI_ISL_1228268 to 1228269, EPI_ISL_1228272 to 1228274, EPI_ISL_1228434 to 1228443, EPI_ISL_1228447 to 1228449, EPI_ISL_1228451, EPI_ISL_1228454 to 1228459, EPI_ISL_1228463 to 1228464, EPI_ISL_1228467 to 1228468, EPI_ISL_1228470 to 1228473, EPI_ISL_1228476			
see above	GLENS FALLS HOSPITAL LABORATORY	Wadsworth Center, New York State Department of Health	Alexis Russel; Daryl M. Lamson; Erasmus Schneider; Erica Lasek-Nesselquist; John Kelly; Jonathan Plitnick; Kirsten St. George; Matthew Shudt; Melissa A Leisner; Navjot Singh
EPI_ISL_794107 to 794118	GLENS FALLS HOSPITAL LABORATORY	Wadsworth Center, New York State Department.of Health	Alexis Russel; Daryl M. Lamson; Erasmus Schneider; Erica Lasek-Nesselquist; John Kelly; Jonathan Plitnick; Kirsten St. George; Matthew Shudt; Melissa A Leisner; Navjot Singh; Sara Griesemer
EPI_ISL_586562 to 586566	GMERS GMERS Medical College and Hospital, Junagadh	Gujarat Biotechnology Research Centre	A M Kadri; Afzal Ansari; Apurvasinh Puvar; Bhavesh Bagda; Chaitanya Joshi; Dinesh Kumar; Harsh Bakshi; Janvi Raval; Komal Patel; Labdhi Pandya; Madhvi Joshi; Maharshi Pandya; Monika Gandhi; Nidhi Patel; Nikha Trivedi; Nitin Savaliya; Pinal Trivedi; R D Dixit; Raghawendra Kumar; Ravi Dedaniya;

			Zarna Patel; Zuber Saiyed
EPI_ISL_469037 to 469042, EPI_ISL_475047 to 475055	GMERS Medical College & Hospital	Gujarat Biotechnology Research Centre	A M Kadri; Afzal Ansari; Ankit Hinsu; Apurvasinh Puvar; Armi Chaudhari; Bhavya Jindal; Chaitanya Joshi; Dinesh Kumar; Fenil Patel; Harsh Bakshi; Janvi Raval; Komal Patel; Labdhi Pandya; Madhvi Joshi; Maharshi Pandya; Meenakshi Shah; Monika Gandhi; Neelam Nathani; Neena Doshi; Neha Rajpara; Nidhi Patel; Nitin Savaliya; Pinal Trivedi; Pooja P Doshi; Pragya Sharma; Pritesh Sabara; Priyanka P Vatsa; R D Dixit; Raghawendra Kumar; Snehal Bagatharia; Tejas Shah; Varsha Godbole; Zarna Patel; Zuber Saiyed
EPI_ISL_476855 to 476862, EPI_ISL_495026 to 495062, EPI_ISL_500946, EPI_ISL_514610, EPI_ISL_524730 to 524731	GMERS Medical College & Hospital, Gotri, Vadodara	Gujarat Biotechnology Research Centre	; A M Kadri; Afzal Ansari; Apurvasinh Puvar; Chaitanya Joshi; Dinesh Kumar; Harsh Bakshi; Janvi Raval; Kamlesh J Upadhyay; Komal Patel; Labdhi Pandya; Madhvi Joshi; Maharshi Pandya; Meenakshi Shah; Monika Gandhi; Neena Doshi; Nidhi Patel; Nikha Trivedi; Nitin Savaliya; Pinal Trivedi; Pranay Shah; R D Dixit; Raghawendra Kumar; Sanjay Kapadia; Varsha Godbole; Zarna Patel; Zuber Saiyed
EPI_ISL_495063 to 495064	GMERS Medical College & Hospital, Himmatnagar	Gujarat Biotechnology Research Centre	; A M Kadri; Afzal Ansari; Apurvasinh Puvar; Chaitanya Joshi; Dinesh Kumar; Harsh Bakshi; Himanshu Khatri; Janvi Raval; Komal Patel; Labdhi Pandya; Madhvi Joshi; Maharshi Pandya; Mayur Gandhi; Monika Gandhi; Nidhi Patel; Nikha Trivedi; Nitin Savaliya; Pinal Trivedi; R D Dixit; Raghawendra Kumar; Zarna Patel; Zuber Saiyed
EPI_ISL_483823 to 483828	GMERS Medical College Himmatnagar	Gujarat Biotechnology Research Centre	A M Kadri; Afzal Ansari; Apurvasinh Puvar; Chaitanya Joshi; Dinesh Kumar; Harsh Bakshi; Himanshu Khatri; Janvi Raval; Komal Patel; Labdhi Pandya; Madhvi Joshi; Maharshi Pandya; Mayur Gandhi; Monika Gandhi; Nidhi Patel; Nikha Trivedi; Nitin Savaliya; Pinal Trivedi; R D Dixit; Raghawendra Kumar; Zarna Patel; Zuber Saiyed
EPI_ISL_524733 to 524734, EPI_ISL_524752 to 524766	GMERS Medical College and Hospital, Dharpur, Patan	Gujarat Biotechnology Research Centre	A M Kadri; A N Parmar; Afzal Ansari; Apurvasinh Puvar; Chaitanya Joshi; Dinesh Kumar; Harsh Bakshi; Janvi Raval; Komal Patel; Labdhi Pandya; Madhvi Joshi; Maharshi Pandya; Monika Gandhi; Nidhi Patel; Nikha Trivedi; Nitin Savaliya; Pinal Trivedi; R D Dixit; Raghawendra Kumar; Zarna Patel; Zuber Saiyed
EPI_ISL_447047 to 447053, EPI_ISL_447547 to 447555, EPI_ISL_467029 to 467038, EPI_ISL_476863 to 476866, EPI_ISL_483820, EPI_ISL_514611 to 514612, EPI_ISL_524737 to 524745, EPI_ISL_525419 to 525420			
see above	GMERS Medical College and Hospital, Gandhinagar	Gujarat Biotechnology Research Centre	A M Kadri; Afzal Ansari; Akanksha Verma; Amit Kanani; Anjali Rajwar; Ankit Hinsu; Apurvasinh Puvar; Armi Chaudhari; Bharti Rajani; Bhavesh Modi; Bhavya Jindal; Binita Aring; Chaitanya Joshi; Dinesh Kumar; Dipa Kinariwala; Dipeshwari Shewale; Disha Patel; Fenil Patel; Gaurishankar Shrimali; Geeta Vaghela; Harsh Bakshi; Janvi Raval; Kairavi Joshi; Kamlesh J Upadhyay; Komal Patel; Labdhi Pandya; Madhvi Joshi; Maharshi Pandya; Monika Gandhi; Neelam Nathani; Neeta Khandelwal; Neha Rajpara; Nidhi Patel; Nidhi Sood; Nikha Trivedi; Nitin Savaliya; Pinal Trivedi; Pooja P Doshi; Pragya Sharma; Pranay Shah; Pritesh Sabara; Priti Pandita; Priyanka P Vatsa; R D Dixit; Raghawendra Kumar; Ramesh Pandit; Sanjay Kapadia; Seema Bhatt; Sharmistha Majumdar; Snehal Bagatharia; Sonia Barve; Tejas Shah; Zarna Patel; Zuber Saiyed
EPI_ISL_825048 to 825049, EPI_ISL_825054 to 825058, EPI_ISL_825061 to 825062	GMERS Medical College and Hospital, Gotri, Vadodara	Gujarat Biotechnology Research Centre	Afzal Ansari; Apurvasinh Puvar; Bithika Duttaroy; Chaitanya Joshi; Dinesh Kumar; Janvi Raval; Kalpesh Mistry; Labdhi Pandya; Madhvi Joshi; Mitesh Kamothi; Nikha Trivedi; Nitin Savaliya; Ramesh Pandit; Zarna Patel; Zuber Saiyed
EPI_ISL_1228925 to 1228929, EPI_ISL_1228932, EPI_ISL_1228934 to 1228937, EPI_ISL_1228943 to 1228944, EPI_ISL_1228946, EPI_ISL_1228948, EPI_ISL_1228950, EPI_ISL_1228953, EPI_ISL_1265474			
see above	GROUPE HOSPITALIER SAINT ANDRE	CNR Virus des Infections Respiratoires - France SUD	Antonin Bal; Bruno Lina; Gregory Destras; Gwendolyne Burfin; Hadrien Regue; Laurence Josset; Martine Valette; Quentin Semanas
EPI_ISL_1085302, EPI_ISL_1085324 to 1085326, EPI_ISL_1085328, EPI_ISL_1085554, EPI_ISL_1085558, EPI_ISL_1085575 to 1085576, EPI_ISL_1085580, EPI_ISL_1085606, EPI_ISL_1085624			
see above	GROUPE HOSPITALIER SUD ILE DE FRANCE	Department of Virology, Henri Mondor University Hospital, Assistance Publique Hôpitaux de Paris, Université Paris-Est Créteil, INSERM U955	Alexandre Soulier; Christophe Rodriguez; Elisabeth Trawinski; Guillaume Gricourt; Jean-Michel Pawlotsky; Melissa N'Debi; Slim Fourati; Vanessa Demontant
EPI_ISL_888821	GZA Hospital Sint-Augustinus	UAntwerp, Laboratory of Medical Microbiology, Campus Drie Eiken S6.26, Universiteitsplein 1, 2610, Wilrijk, Antwerp, Belgium	Basil Britto Xavier; Christine Lammens; Herman Goossens; Jasmine Coppens; Veerle Matheeussen
EPI_ISL_1039222	GZA Sint-Augustinus	UAntwerp, Laboratory of Medical Microbiology	Basil Britto Xavier; Christine Lammens; Herman Goossens; Jasmine Coppens; Marie Le Mercier; Veerle Matheeussen
EPI_ISL_888822 to 888825, EPI_ISL_939654	GZA Sint-Augustinus Hospital	UAntwerp, Laboratory of Medical Microbiology, Campus Drie Eiken S6.26, Universiteitsplein 1, 2610, Wilrijk, Antwerp, Belgium	Basil Britto Xavier; Christine Lammens; Herman Goossens; Jasmine Coppens; Marie Le Mercier; Veerle Matheeussen
EPI_ISL_940757	GZA Sint-Augustinus Hospital	UAntwerp, Laboratory of Medical Microbiology, Campus Drie Eiken S6.26, Universiteitsplein 1, 2610, Wilrijk, Belgium	Basil Britto Xavier; Christine Lammens; Herman Goossens; Jasmine Coppens; Marie Le Mercier; Veerle Matheeussen
EPI_ISL_1169920	Galdakao University Hospital	Cruces University Hospital	Ana Belén de la Oz; Ana Gual-de-Torrella; Mikel Gallego; Pilar Berdonces
EPI_ISL_851048 to 851051	Gandhi Medical College and Hospital	CSIR-Centre for Cellular and Molecular Biology	Amrithesh Kumar; Archana GJ; Divya Tej Sowpati; Madhavi Latha Manolla; Manisha Rani; Nagamani Kammili; Raja Rao Mesepogu; Shailaja VV; Sofia Banu; Sudhamadhuri Devara; Sunitha Pakalapati; Sushma Rajyalakshmi Gudiseva; Thrilok Chander Bingi; Vanisree Rajoli; Vinay Shekar Reddy; Winnie Thomas
EPI_ISL_850510, EPI_ISL_850514	Gandhi Medical College and Hospital	MDRU-DHR,Gandhi Medical College	Amrithesh Kumar; Archana GJ; Divya Tej Sowpati; Madhavi Latha Manolla; Manisha Rani; Nagamani Kammili; Raja Rao Mesepogu; Shailaja VV; Sofia Banu; Sudhamadhuri Devara; Sunitha Pakalapati; Sushma Rajyalakshmi Gudiseva; Thrilok Chander Bingi; Vanisree Rajoli; Vinay Shekar Reddy; Winnie Thomas
EPI_ISL_850507, EPI_ISL_850509	Gandhi Medical College and Hospital	VRDL-Gandhi Medical College	Amrithesh Kumar; Archana GJ; Divya Tej Sowpati; Madhavi Latha Manolla; Manisha Rani; Nagamani Kammili; Raja Rao Mesepogu; Rajeshwar Rao; Shailaja VV; Sofia Banu; Sudhamadhuri Devara; Sunitha Pakalapati; Sushma Rajyalakshmi Gudiseva; Thrilok Chander Bingi; Vanisree Rajoli; Vinay Shekar Reddy; Winnie Thomas
EPI_ISL_583426, EPI_ISL_583429, EPI_ISL_583434 to 583436, EPI_ISL_583441 to 583442, EPI_ISL_583452 to 583454, EPI_ISL_583458 to 583459, EPI_ISL_583463 to 583464, EPI_ISL_583467, EPI_ISL_583471, EPI_ISL_583473, EPI_ISL_583477			
see above	Garcia-Sastre Laboratory, Department of Microbiology, Icahn School of Medicine at Mount Sinai	van Bakel Laboratory, Genetics and Genomics Sciences, Icahn School of Medicine at Mount Sinai	Adolfo García-Sastre; Adriana van de Guchte; Ajay Obla; Ana S. Gonzalez-Reiche; Genovefa Papanicolaou; Gunjan Shah; Harm van Bakel; Jayeeta Dutta; Judith Aberg; Kent Sepkowitz; Miguel-Angel Perales; Ngolela Esther Babady; Sadaf Aslam; Teresa Aydillo; Tobias Hohl; Zenab Khan; and Mini Kamboj
EPI_ISL_413517, EPI_ISL_414513 to 414514	Gastrointestinal and Liver Diseases Research Center, Iran University of Medical Sciences	Gastrointestinal and Liver Diseases Research Center, Iran University of Medical Sciences	Ajdarkosh, H.; Ameli, M.; Faraji, A.; Ghanbari, B.; Jamshidi Makiani; Karbalaie Niya; Keyvani, H.; Khoonsari, M.; Laali, A.; M. and Roham, M.; M. and Roham, M.; M.H.; M.R.; Nikkhah, M.; Ranjbar, M.; Rezaie, N.; Safarnezhad Tameshkel, F.; Savaj, S.; Sohrabi; Tabibzadeh, A.; Zamani, F.
EPI_ISL_534249 to 534251	Gavle Sjukhus	The Public Health Agency of Sweden	Anna Risberg; Anna-Malin Linde; Karin Tegmark-Wisell; Maria Lind Karlberg; Mattias Haukland; Mia Brytting; Olov Svartstrom; Oskar Karlsson Lindsjo; Petra Edquist; Reza Advani; Sandra Broddesson
EPI_ISL_475110, EPI_ISL_475115, EPI_ISL_582801, EPI_ISL_582809, EPI_ISL_582834, EPI_ISL_615101 to 615103, EPI_ISL_623095, EPI_ISL_648140 to 648142, EPI_ISL_648144, EPI_ISL_648163, EPI_ISL_661283, EPI_ISL_661286 to 661287, EPI_ISL_710608 to 710609, EPI_ISL_831977 to 831978			
see above	Gavle klinisk mikrobiologi	The Public Health Agency of Sweden	Anna Risberg; Anna-Malin Linde; Department of Microbiology; Karin Tegmark-Wisell; Maria Lind Karlberg; Mattias Haukland; Mia Brytting; Olov Svartstrom; Oskar Karlsson Lindsjo; Petra Edquist; Reza Advani; Sandra Broddesson; Shamam Muradrasoli; The Public Health Agency of Sweden
EPI_ISL_676514	Gavleborg	The Public Health Agency of Sweden	Department of Microbiology; The Public Health Agency of Sweden
EPI_ISL_730567 to 730577, EPI_ISL_730620, EPI_ISL_984736 to 984752, EPI_ISL_984980 to 984994	Gazi University Faculty of Medicine, Medical Virology Laboratory	Gazi University Faculty of Medicine, Medical Virology Laboratory	Erdem ahin; Gülendam Bozday; Hager Muftah; Il Fidan; Kayhan Çalar; Murat Dizbay; Selin Yiit; Shaknoza Sarzhanova; Özlem Güzel Tunçcan
EPI_ISL_420855, EPI_ISL_420876 to 420877, EPI_ISL_430064 to 430066	Geelong Centre for Emerging Infectious Diseases	Geelong Centre for Emerging Infectious Diseases	Alexandersen S.; Bhatta T.R.; Chamings A.; Chamings, A.; Raj Bhatta T.
EPI_ISL_900502, EPI_ISL_900536 to 900542	Gen-Bio	CNR Virus des Infections Respiratoires - France SUD	Antonin Bal; Bruno Lina; Gregory Destras; Gwendolyne Burfin; Hadrien Règue; Laurence Josset; Martine Valette; Quentin Semanas; Sylvie Larrat
EPI_ISL_1239609 to 1239612, EPI_ISL_1239761 to 1239762, EPI_ISL_1273840	GenXOne SA	1. National Institute of Public Health - National Institute of Hygiene; 2. Eurofins Genomics Europe Sequencing GmbH	ECDC COVID-19 WGS support team; Eurofins Genomics Europe Sequencing Team; Gierczyki Rafa; Sadkowska-Todys Magorzata; Wokowicz Tomasz; Zacharczuk Katarzyna

EPI_ISL_1184582, EPI_ISL_1185245, EPI_ISL_1186201	Genelabs Medical (Pvt) Ltd	Genelabs Medical (Pvt) Ltd	Chandanamali Punchihewa; K. Dasuki Hansana Perera; K.Dasuki Hansana Perera; Ravithra Roshan Rajasegar; Tharmini Sundralingam; Viveka Selvarasa
EPI_ISL_467300	General Hospital "Abdulah Nakas"	Alea Genetic Center	Damir Marjanovic; Dino Pecar; Enis Kandic; Lana Salihefendic; Nihad Fejzic; Rijad Konjhodzic; Sead Jazic; Teufik Goletic
EPI_ISL_959650, EPI_ISL_964882	General Hospital "Abdulah Nakas" Sarajevo	Alea Genetic Centre	Adis Kandic; Dino Pecar; Lana Salihefendic; Rijad Konjhodzic; Sead Jazic
EPI_ISL_922076	General Hospital #Abdulah Nakas" Sarajevo	Alea Genetic Centre	Dino Pecar; Lana Salihefendic; Rijad Konjhodzic; Sead Jazic
EPI_ISL_909738	General Hospital - Bitola	Research Center for Genetic Engineering and Biotechnology "Georgi D. Efremov" , Macedonian Academ	RCGEB - MASA
EPI_ISL_933566, EPI_ISL_933772, EPI_ISL_933786, EPI_ISL_934314, EPI_ISL_934418, EPI_ISL_942961, EPI_ISL_942963, EPI_ISL_942970, EPI_ISL_957423, EPI_ISL_958751, EPI_ISL_1011755			
see above	General Hospital - Bitola	Research Center for Genetic Engineering and Biotechnology "Georgi D. Efremov" , Macedonian Academy of Sciences and Arts	Aleksandar J. Dimovski; Aleksandar J.Dimovski; Dijana Plasheska-Karanfilska; Gjorgji Bozinovski; Milena Jakimovska; Predrag Noveski; RCGEB - MASA
EPI_ISL_516413, EPI_ISL_677705, EPI_ISL_677707, EPI_ISL_678252, EPI_ISL_910327, EPI_ISL_918492, EPI_ISL_933573, EPI_ISL_933776, EPI_ISL_934092, EPI_ISL_942962, EPI_ISL_942964, EPI_ISL_942966 to 942968, EPI_ISL_956421, EPI_ISL_956668, EPI_ISL_1013098, EPI_ISL_1013450 to 1013451, EPI_ISL_1018105, EPI_ISL_1143746			
see above	General Hospital - Kumanovo	Research Center for Genetic Engineering and Biotechnology "Georgi D. Efremov" , Macedonian Academy of Sciences and Arts	Aleksandar J. Dimovski; Aleksandar J.Dimovski; Dijana Plasheska-Karanfilska; Gjorgji Bozinovski; Milen; Milena Jakimovska; Predrag Noveski; RCGEB - MASA; leksandar J. Dimovski
EPI_ISL_910333	General Hospital - Ohrid	Research Center for Genetic Engineering and Biotechnology "Georgi D. Efremov" , Macedoni	RCGEB - MASA
EPI_ISL_677704, EPI_ISL_677709, EPI_ISL_677711, EPI_ISL_677722, EPI_ISL_677725 to 677726, EPI_ISL_678255 to 678256, EPI_ISL_909740, EPI_ISL_933775, EPI_ISL_933803, EPI_ISL_954745 to 954746, EPI_ISL_956402, EPI_ISL_959289, EPI_ISL_959291, EPI_ISL_1014934, EPI_ISL_1018103, EPI_ISL_1018107, EPI_ISL_1138222, EPI_ISL_1138410, EPI_ISL_1138423, EPI_ISL_1138752, EPI_ISL_1140098 to 1140100, EPI_ISL_1142575, EPI_ISL_1144121			
see above	General Hospital - Ohrid	Research Center for Genetic Engineering and Biotechnology "Georgi D. Efremov" , Macedonian Academy of Sciences and Arts	Aleksandar J. Dimovski; Dijana Plasheska-Karanfilska; Gjorgji Bozinovski; Milena Jakimovska; Predrag Noveski; RCGEB - MASA
EPI_ISL_514354, EPI_ISL_677674, EPI_ISL_677703, EPI_ISL_677712 to 677714, EPI_ISL_678258, EPI_ISL_909739, EPI_ISL_918497, EPI_ISL_933713, EPI_ISL_933766, EPI_ISL_944786, EPI_ISL_954265, EPI_ISL_956367, EPI_ISL_956382 to 956383, EPI_ISL_956419, EPI_ISL_959285, EPI_ISL_1013583, EPI_ISL_1014762, EPI_ISL_1014766, EPI_ISL_1014936, EPI_ISL_1137612, EPI_ISL_1137620, EPI_ISL_1138749, EPI_ISL_1140124, EPI_ISL_1142172			
see above	General Hospital - Prilep	Research Center for Genetic Engineering and Biotechnology "Georgi D. Efremov" , Macedonian Academy of Sciences and Arts	Aleksandar J. Dimovski; Dijana Plasheska-Karanfilska; Gjorgji Bozinovski; Milena Jakimovska; Predrag Noveski; RCGEB - MASA
EPI_ISL_933571	General Hospital - Shtip	Research Center for Genetic Engineering and Biotechnology "Georgi D. Efr	RCGEB - MASA
EPI_ISL_909742, EPI_ISL_933602, EPI_ISL_933797, EPI_ISL_934413, EPI_ISL_959288, EPI_ISL_1013521, EPI_ISL_1018104, EPI_ISL_1137623, EPI_ISL_1138406, EPI_ISL_1138412, EPI_ISL_1138748, EPI_ISL_1138751, EPI_ISL_1140120, EPI_ISL_1140123, EPI_ISL_1144534			
see above	General Hospital - Shtip	Research Center for Genetic Engineering and Biotechnology "Georgi D. Efremov" , Macedonian Academy of Sciences and Arts	Aleksandar J. Dimovski; Dijana Plasheska-Karanfilska; Gjorgji Bozinovski; Milena Jakimovska; Predrag Noveski; RCGEB - MASA
EPI_ISL_677719, EPI_ISL_678260, EPI_ISL_909736, EPI_ISL_918486, EPI_ISL_944108 to 944109, EPI_ISL_954611, EPI_ISL_1014939, EPI_ISL_1018106, EPI_ISL_1018218 to 1018219, EPI_ISL_1138424, EPI_ISL_1138743 to 1138744, EPI_ISL_1138746			
see above	General Hospital - Struga	Research Center for Genetic Engineering and Biotechnology "Georgi D. Efremov" , Macedonian Academy of Sciences and Arts	Aleksandar J. Dimovski; Dijana Plasheska-Karanfilska; Gjorgji Bozinovski; Milena Jakimovska; Predrag Noveski; RCGEB - MASA
EPI_ISL_943993	General Hospital - Strumica	Research Center for Genetic Engineering and Biotechnology "Georgi D. Efremov" , Macedon	Aleksandar J. Dimovski; Dijana Plasheska-Karanfilska; Gjorgji Bozinovski; Predrag Noveski
EPI_ISL_910326	General Hospital - Strumica	Research Center for Genetic Engineering and Biotechnology "Georgi D. Efremov" , Macedonian Academ	RCGEB - MASA
EPI_ISL_678254, EPI_ISL_918488, EPI_ISL_933579, EPI_ISL_933717, EPI_ISL_933726, EPI_ISL_943994, EPI_ISL_943998, EPI_ISL_944105 to 944106, EPI_ISL_956404, EPI_ISL_959286, EPI_ISL_1013452, EPI_ISL_1014763, EPI_ISL_1014933, EPI_ISL_1018102, EPI_ISL_1137622			
see above	General Hospital - Strumica	Research Center for Genetic Engineering and Biotechnology "Georgi D. Efremov" , Macedonian Academy of Sciences and Arts	Aleksandar J. Dimovski; Dijana Plasheska-Karanfilska; Gjorgji Bozinovski; Mi; Milena Jakimovska; Predrag Noveski; RCGEB - MASA
EPI_ISL_909654, EPI_ISL_910328, EPI_ISL_933574, EPI_ISL_933720, EPI_ISL_943996 to 943997, EPI_ISL_944107, EPI_ISL_944779, EPI_ISL_947678, EPI_ISL_957966, EPI_ISL_959274, EPI_ISL_1013522, EPI_ISL_1014764, EPI_ISL_1014912, EPI_ISL_1137611, EPI_ISL_1137614, EPI_ISL_1140512, EPI_ISL_1140936, EPI_ISL_1141320, EPI_ISL_1142968, EPI_ISL_1143347			
see above	General Hospital - Tetovo	Research Center for Genetic Engineering and Biotechnology "Georgi D. Efremov" , Macedonian Academy of Sciences and Arts	Aleksandar J. Dimovski; Dijana Plasheska-Karanfilska; Gjorgji Bozinovski; Milena Jakimovska; Predrag Noveski; RCGEB - MASA
EPI_ISL_677675, EPI_ISL_677710, EPI_ISL_910330, EPI_ISL_933578, EPI_ISL_933660, EPI_ISL_934415, EPI_ISL_942965, EPI_ISL_942969, EPI_ISL_943995, EPI_ISL_953939, EPI_ISL_956405, EPI_ISL_1011754, EPI_ISL_1011756, EPI_ISL_1013097, EPI_ISL_1014938, EPI_ISL_1141754			
see above	General Hospital - Veles	Research Center for Genetic Engineering and Biotechnology "Georgi D. Efremov" , Macedonian Academy of Sciences and Arts	Aleksandar J. Dimovski; Aleksandar J.Dimovski; Dijana Plasheska-Karanfilska; Gjorgji Bozinovski; Milena Jakimovska; Predrag Noveski; RCGEB - MASA
EPI_ISL_747243	General Hospital Laboratory	Pathogen Laboratory (BSL3), Biomedical Innovation Department, Experimental and Applied Biology Division, Scientific Research Center and High Education from Ensenada (CICESE)	Cervantes-Luevano K.; Galindo C. and Licea-Navarro A.; Martinez M.A.; Saavedra-Flores A.
EPI_ISL_406798 to 406801	General Hospital of Central Theater Command of People's Liberation Army of China	BGI & Institute of Microbiology, Chinese Academy of Sciences & Shandong First Medical University & Shandong Academy of Medical Sciences & General Hospital of Central Theater Command of People's Liberation Army of China	Weifeng Shi and Zhenhong Hu; Weijun Chen; Yuhai Bi
EPI_ISL_586531 to 586535	General Hospital,Chhotaudepur	Gujarat Biotechnology Research Centre	A M Kadri; Afzal Ansari; Apurvasinh Puvar; Arti Thakur; Chaitanya Joshi; Dharak Pandya; Dinesh Kumar; Harsh Bakshi; Janvi Raval; Komal Patel; Labdhi Pandya; Madhvi Joshi; Maharshi Pandya; Monika Gandhi; Nidhi Patel; Nikha Trivedi; Nitin Savaliya; Pinal Trivedi; R D Dixit; Raghawendra Kumar; Yogesh Parmar; Zarna Patel; Zuber Saiyed
EPI_ISL_586546 to 586548	General Hospital,Godhra	Gujarat Biotechnology Research Centre	A M Kadri; Afzal Ansari; Apurvasinh Puvar; Chaitanya Joshi; Dinesh Kumar; Harsh Bakshi; Janvi Raval; Komal Patel; Labdhi Pandya; Madhvi Joshi; Maharshi Pandya; Monika Gandhi; Neela Katara; Nidhi Patel; Nikha Trivedi; Nitin Savaliya; Pinal Gandhi; Pinal Trivedi; R D Dixit; Raghawendra Kumar; Sangeeta Kumar; Zarna Patel; Zuber Saiyed
EPI_ISL_430846	General Intensive Care Unit, Raymond Poincaré Hospital (AP-HP), Lab Inflammation & Infection, U1173 University Paris Saclay-UVSQ/INSERM, Garches, France.	Institut Pasteur, Laboratory for Urgent Response to biological Threats	Annane Djillali; Caro Valérie; Manuguerra Jean-Claude; Vanhomwegen Jessica

EPI_ISL_1261386 to 1261388	General University Hospital	National Reference Laboratory for Influenza and Respiratory Viruses CZE	Alexander Nagy; Dusan Trnka; Helena Jirincova; Jaromira Vecerova; Timotej Suri
EPI_ISL_614282 to 614293, EPI_ISL_623096 to 623097, EPI_ISL_623103, EPI_ISL_660324 to 660325, EPI_ISL_693766 to 693774, EPI_ISL_792042, EPI_ISL_792045 to 792048, EPI_ISL_792050, EPI_ISL_792053			
see above	General practitioner	National Reference Center for Viruses of Respiratory Infections, Institut Pasteur, Paris	; Angela Brisebarre; Brieuc Lefaure; Camille Capel; Etienne Simon-Lorière; Marion Barbet; Maud Vanpeene; Méline Bizard; Sylvie Behillil; Sylvie van der Werf; Vincent Enouf
EPI_ISL_888827 to 888829	General practitioner office	National Reference Center for Viruses of Respiratory Infections, Institut Pasteur, Paris	Angela Brisebarre; Camille Capel; Etienne Simon-Lorière; Marion Barbet; Maud Vanpeene; Méline Bizard; Sylvie Behillil; Sylvie van der Werf; Vincent Enouf
EPI_ISL_746478 to 746825, EPI_ISL_754391 to 754393, EPI_ISL_1167668 to 1167933, EPI_ISL_1300449 to 1300495, EPI_ISL_1300497 to 1300520	Genetica Molecular and Subdepartamento de Virologia ISP Chile	Instituto de Salud Publica de Chile	Andres Castillo; Barbara Parra; Gisselle Barra; Jaime Lagos; Javier Tognarelli; Jorge Fernandez; Karen Orostica; Loredana Arata; Patricia Bustos; Rodrigo Fasce
EPI_ISL_936380 to 936386	Genetica y Virologia, Facultad de Ciencias	Genetica y Virologia, Facultad de Ciencias	Arbiza, J.; Calleros, L.; Chiparelli, H.; Coppola, L.; Delfraro, A.; Frabasile, S.; Fuques, E.; Goni, N.; Grecco, S.; Marandino, A.; Mogdasy, C.; Panzera, Y.; Perez, R.; Ramas, V.; Ramos, N.; Sorhouet, C.; Techera, C.; Tomas, G.
EPI_ISL_978917, EPI_ISL_1164927 to 1164929	Genome Analysis Center, Yamanashi Central Hospital	Genome Analysis Center, Yamanashi Central Hospital	Yosuke Hirotsu
EPI_ISL_475570 to 475571, EPI_ISL_475573, EPI_ISL_548260 to 548263, EPI_ISL_561375 to 561377, EPI_ISL_561630, EPI_ISL_605783, EPI_ISL_746318 to 746319, EPI_ISL_746323 to 746324			
see above	Genome Center	Genome Center	A. S. M. Rubayet- Ul- Alam; Ashok Kumar; Chakrabortty Atonu; Habiba Ibnat; Hassan M. Al-Emran; M. Rafiul Islam; M. Shaminur Rahman; Md Nur Kabidul Azam; Md. Ali Ahasan Setu; Md. Aminul Islam; Md. Anwar Hossain; Md. Hasan al Pramanik; Md. Iqbal Kabir Jahid; Md. Iqbal Kabir Jahid Md. Anwar Hossain; Md. Muzahidul Islam; Md. Nazmul Hasan; Md. Shazid Hasan; Md. Taijul Islam; Md. Tanvir Islam; Md. Zannat Ali; Mithun Talukder Md. Tawyabur; Najmuj Sakib; Najuj Sakib; Nigar Sultana Meghla; Ovinu Kibria Islam; Pravas Chandra Roy; Proshanto Kumar Das; Provakar Mondol; Ruhul Amin; S. M. Tanjil Shah; Selina Akter; Shireen Nigar; Shohanur Rahaman; Shovon Lal Sarkar; Sourav Dutta; Tanay Chakrovarty
EPI_ISL_447590, EPI_ISL_447897, EPI_ISL_548259, EPI_ISL_572319, EPI_ISL_572325 to 572327, EPI_ISL_576389 to 576393, EPI_ISL_576396, EPI_ISL_583535 to 583556			
see above	Genome Centre	Genome Centre	; A. S. M. Rubayet Ul Alam; Amina Ferdaus manami; Habiba Ibnat; Hassan M. Al-Emran; Hassan Md. Al-Emran; Iqbal Kabir Jahid; M. Anwar Hossain; M. Rafiul Islam; M. Shaminur Rahman; M.Anwar Hossain; MD. Ali Ahasan Setu; Md Nur Kabidul Azam; Md. Anwar Hossain; Md. Nazmul Hasan; Md. Nur Kabidul Islam; Md. Shazid Hasan; Md. Tanvir Islam; Najmuj Sakib; Ovinu Kibria Islam; Pravas Chandra Roy; Ruhul Amin; Selina Akter; Selina Akter Pravas Chandra Roy Amina Ferdaus manami Habiba Ibnat A. S. M. Rubayet Ul Alam Shireen Nigar Iqbal Kabir Jahid and M. Anwar Hossain; Shireen Nigar; Sourav Dutta Dip; Tanay Chakrovarty
EPI_ISL_427391, EPI_ISL_428346, EPI_ISL_428368, EPI_ISL_480228 to 480292, EPI_ISL_735259 to 735373, EPI_ISL_735434 to 735435, EPI_ISL_833141 to 833148, EPI_ISL_885146, EPI_ISL_955153 to 955154			
see above	Genomic Laboratory (GLAB) (Conjoint lab of Health Directorate of Istanbul and Istanbul Technical University)	Genomic Laboratory (GLAB), Istanbul Technical University	Arzu Irvem; Ayse Serra Ozel; Betsi Kose; Betsi Köse; Bugra Agaoglu; Elifnaz Çelik; Gizem Alkurt; Gizem Dinler Doganay; Ilker Karacan; Jale Yildiz; Levent Doganay; Mehtap Aydin; Mehtap Aydn; Nihat Bugra Agaoglu; Nilsun Altunal; Nisan Denizce Can; Ozlem Akgun Dogan; Pari Sharifli; Payam Zolfagharian; Tugba Kizilboga Akgun; Yasemin Kendir Demirkol
EPI_ISL_882921	Genomic Medicine Laboratory, IRCCS Santa Lucia Foundation	INMI Lazzaro Spallanzani IRCCS	A Di Caro; B Bartolini; C.E.M Gruber; E Giardina; E Giombini; F Messina; M Rueca; MR Capobianchi; O Butera
EPI_ISL_803899	Genomic Medicine Laboratory, IRCCS Santa Lucia Foundation	National Institute for Infectious Diseases, INMI, "L. Spallanzani" IRCCS	A Di Caro; B Bartolini; C.E.M Gruber; E Giardina; E Giombini; F Messina; M. Rueca; MR Capobianchi; O Butera
EPI_ISL_512774	Genomic Research Lab, BCSIR	Bangladesh Council of Scientific and Industrial Research	A. K. M. Shamsuzzaman; Abu Sayeed Mohammad Mahmud; Asish Kumar Ghosh; Barna Goswami; Eshrar Osman; Iffat Jahan; Mahmuda Yeasmin; Md. Ahasan Habib; Md. Maruf Ahmed Molla; Md. Murshed Hasan Sarkar; Md. Saddam Hossain; Md. Salim Khan; Mohammad Samir Uzzaman; Salek Ahmed Sajib; Shahina Akter; Sheikh Md. Selim Al Din; Tanjina Akhter Banu; Tasnim Nafisa; Utpal Chandra Ray
EPI_ISL_483688	Genomic Research Lab, BCSIR	Genomic Research Lab, BCSIR	A. K. M. Shamsuzzaman; Abu Sayeed Mohammad Mahmud; Asish Kumar Ghosh; Barna Goswami; Eshrar Osman; Iffat Jahan; Mahmuda Yeasmin; Md. Ahasan Habib; Md. Maruf Ahmed Molla; Md. Murshed Hasan Sarkar; Md. Saddam Hossain; Md. Salim Khan; Mohammad Samir Uzzaman; Salek Ahmed Sajib; Shahina Akter; Sheikh Md. Selim Al Din; Tanjina Akhter Banu; Tasnim Nafisa; Utpal Chandra Ray
EPI_ISL_632908	Genomic Sciences, Rehman Medical Institute	Genomic Sciences, Rehman Medical Institute	Afridi; Ali, J.; H. and Jehanzeb, V.; Haider; Jan; S.A.; Sabiha, B.; U.K.
EPI_ISL_1290802	Genomic and molecular Biology Group, A.C.Camargo Cancer Center	Laboratory of Bioinformatics and Computational Biology, A.C.Camargo Cancer Center	Alexandre Defelicibus; Dirce Carraro; Giovana Torrezan; Israel Tojal
EPI_ISL_730197 to 730228, EPI_ISL_1081423 to 1081456, EPI_ISL_1081473 to 1081495	Genomica Lab Molecular, M©xico	Andersen lab at Scripps Research	Jose Horacio Reyna Verdugo; Jose Roman Chavez Mendez; Luis Alberto Rangel Gonzalez; Martin Gonzalez Ibarra; SEARCH Alliance San Diego with Jonathan Gonzalez Garcia
EPI_ISL_812783 to 812872	Genomics Program, Children Cancer Hospital	Genomics Program, Children Cancer Hospital	Abdo, I.; Abouelnaga, S.; Amer, K.; Bakry, U.; Diab, A.; El-Shaqnqery, H.; El-Zayat, M.; Farawyla, H.; Gomaa, C.; Hadad, A.; Halafawy, A.; Hammad, M.; Hassan, R.; Hassan, W.; Hatem, A.; Hussein, S.; Jalal, D.; Magdeldin, S.; Mansour, T.; Monuir, G.; Salah, H.; Samir, O.; Sayed, A.; Soliman, M.; Soliman, S.; shalaby, L.
EPI_ISL_436715 to 436717	Genomics and Computational Biology Lab, Scientific Research Institute of Physical-Chemical Medicine, FMBA of Russia	Genomics and Computational Biology Lab, Scientific Research Institute of Physical-Chemical Medicine, FMBA of Russia	A. Manolov; A. Pavlenko; D. Fedorov; K. Klimina; O. Guskova; V. Govorun and E. Ilina; V. Veselovsky
EPI_ISL_861889, EPI_ISL_861911, EPI_ISL_861914	Genomika Einstein	LATE - Laboratório de Técnicas Especiais - Hospital Israelita Albert Einstein	Ana Paula Moreira Salles; Deyvid Amgarten; Fernanda de Mello Malta; João Bosco Oliveira Filho; João Renato Rebello Pinho; Pedro Henrique Sebe Rodrigues; Raquel Riyuzo
EPI_ISL_640016, EPI_ISL_640027, EPI_ISL_700497	George Hospital wc GRH	NHLS/UCT	Arash Iranzadeh; Bruna Galvao; Carolyn Williamson; Deelan Doolabh; Diana Hardie; Innocent Mudau; Kruger Marais; Lynn Tyers; Marvin Hsiao; Stephen Korsman
EPI_ISL_960118	George Hospital wc GRH	National Health Laboratory Service/UCT	Arash Iranzadeh; Bruna Galvao; Carolyn Williamson; Deelan Doolabh; Diana Hardie; Innocent Mudau; Kruger Marais; Lynn Tyers; Marvin Hsiao; Stephen Korsman
EPI_ISL_696458, EPI_ISL_696471, EPI_ISL_696473, EPI_ISL_696481, EPI_ISL_696503	George Hospital wc GRH & NHLS/UCT	KRISP, KZN Research Innovation and Sequencing Platform	Arash Iranzadeh; Bruna Galvao; Carolyn Williamson; Deelan Doolabh; Diana Hardie; Emanuel James San; Houriiyah Tegally; Innocent Mudau; Jennifer Giandhari; Kruger Marais; Lynn Tyers; Marvin Hsiao; Stephen Korsman; Sureshnee Pillay; Tulio de Oliveira
EPI_ISL_640049	George Road Sat Clinic wc GWM	NHLS/UCT	Arash Iranzadeh; Bruna Galvao; Carolyn Williamson; Deelan Doolabh; Diana Hardie; Innocent Mudau; Kruger Marais; Lynn Tyers; Marvin Hsiao; Stephen Korsman
EPI_ISL_450801, EPI_ISL_452120, EPI_ISL_452125, EPI_ISL_509662	Georgia Department of Health	Pathogen Discovery, Respiratory Viruses Branch, Division of Viral Diseases, Centers for Disease Control and Prevention	Alison S. Laufer Halpin; Anna Montmayeur; Anna Uehara; Bettina Bankamp; Christopher A. Elkins; Clinton R. Paden; Haibin Wang; Jing Zhang; Krista Queen; Mary S. Keckler; Rachel Marine; Suxiang Tong; Yan Li; Ying Tao; Zachary Weiner
EPI_ISL_594456	Georgia Public Health Laboratory	Pathogen Discovery, Respiratory Viruses Branch, Division of Viral Diseases, Centers for Disease Control and Prevention	Anna Uehara; Clinton Paden; Haibin Wang; Jing Zhang; Julu Bhatnagar; Krista Queen; Suxiang Tong; Yan Li; Ying Tao
EPI_ISL_539524, EPI_ISL_862556, EPI_ISL_862564 to 862565, EPI_ISL_862567, EPI_ISL_862640, EPI_ISL_954806	Gerencia de Asistencia Sanitaria de Soria	Instituto de Salud Carlos III	A. Monzón; C. Aldea; F. Casas; I; I. Aldea, C.; I. Jiménez; Iglesias-Caballero; M. Pozo; M. Camarero; M. Cuesta; M. González-Esguevillas; M. Molinero Calamita; M. Zaballos; M.Camarero; P. Jiménez; P. Zaballos; S. Cuesta; S. Juliá; S. Molinero Calamita; S. Pozo; S. Sandonís; S. Varona; V. Vázquez
EPI_ISL_539557	Gerencia del área de salud de Badajoz, Llerena y Zafra	Instituto de Salud Carlos III	A. Monzón; C. Pazos; F. Casas; I; I. Jiménez; Iglesias-Caballero; M. Camarero; M. Cuesta; M. González-Esguevillas; M. Molinero Calamita; M. Zaballos; P. Jiménez; S. Juliá; S. Pozo; S. Varona
EPI_ISL_960664 to 960830	Germano de sousa	Instituto Gulbenkian de Ciencia	Cathy Paulino; João Costa; João Sobral; Maria Costa; Ricardo Leite; Susana Ladeiro
EPI_ISL_918484, EPI_ISL_933722,	Gerontology Institute - Skopje	Research Center for Genetic Engineering and Biotechnology	Aleksandar J. Dimovski; Dijana Plasheska-Karanfilska; Gjorgji Bozinovski; Milena Jakimovska; Predrag Noveski; RCGEB - MASA

EPI_ISL_933769, EPI_ISL_944777, EPI_ISL_954139, EPI_ISL_954141		"Georgi D. Efremov" , Macedonian Academy of Sciences and Arts	
EPI_ISL_1048250	Gesundheitsamt Region Kassel/Synlab Trier	Robert Koch Institute, Influenza and respiratory viruses FG17 & Bioinformatics MF1, Berlin, Germany	Aleksandar Radonic; Kapp; Marianne Wedde; Oliver Drechsel; R. Dürrwald; Ralf Duerrwald; Rene Kmiecinski; Stefan Kroeger; Stephan Fuchs; Thorsten Wolff; Volk
EPI_ISL_1156473, EPI_ISL_1156488, EPI_ISL_1156524, EPI_ISL_1156549, EPI_ISL_1156628, EPI_ISL_1156753, EPI_ISL_1211080, EPI_ISL_1211130, EPI_ISL_1211133, EPI_ISL_1211143, EPI_ISL_1211150, EPI_ISL_1211169, EPI_ISL_1211179, EPI_ISL_1211181, EPI_ISL_1211197, EPI_ISL_1211210, EPI_ISL_1211222, EPI_ISL_1211232, EPI_ISL_1211250, EPI_ISL_1211258, EPI_ISL_1211380, EPI_ISL_1211389, EPI_ISL_1216048, EPI_ISL_1216103, EPI_ISL_1216194, EPI_ISL_1216206, EPI_ISL_1216439			
see above	Gesundheitszentrum Brandenburg an der Havel GmbH bzw. Institut für Laboratoriumsmedizin Brandenburg an der Havel	Robert Koch Institute	
EPI_ISL_480083 to 480089	Gifu Prefectural Institute of Public Health and Environmental Sciences	Pathogen Genomics Center, National Institute of Infectious Diseases	Hajime Kamiya; Kentaro Itokawa; Makoto Kuroda; Masanori Hashino; Motoi Suzuki; Rina Tanaka; Tsuyoshi Sekizuka; Yoshihiko Kameyama
EPI_ISL_745471 to 746317	Ginkgo Bioworks Clinical Laboratory	Utah Public Health Laboratory	Alex Plocik; Becky Schilling; Birgitte Simen; David R. Hillyard; E. Susan Slechta; Erin L. Young; James McGann; Jeffrey B. Stevenson; Jim Griffin; Keith Robison; Kelly Oakeson; Malaika McKenzie-Bennett; Martha Pierson; Melanie A. Mallory; Michael T. Pyne; Michelle Spencer; Rebecca Littlefield; Salika M. Shakir; Tara Gallagher
EPI_ISL_833260 to 833261	Giulianova	Istituto Zooprofilattico Sperimentale dell'Abruzzo e Molise "G. Caporale"	Ancora M; Calistri P; Cammà C; Curini V; Di Domenico M; Di Pasquale A; Lorusso A; Mangone I; Marcacci M; Puglia I; Rinaldi A; Savini G
EPI_ISL_447608 to 447613, EPI_ISL_452218 to 452223	Goethe University Hospital Frankfurt	Institute for Medical Virology, Goethe University Hospital Frankfurt	Annemarie Berger; Björn Rotter; Denisa Bojkova; Jindrich Cinatl; Klaus Hoffmeier; Sandra Westhaus; Sandra Ciesek; Sebastian Hoehl; Tuna Toptan; and Marek Widera
EPI_ISL_529146	Goethe University Hospital Frankfurt, Institute for Medical Virology	Goethe University Hospital Frankfurt, Institute for Medical Virology	Berger, A.; Bojkova, D.; Ciesek, S.; Cinatl, J.; Grabmair; Hoehl, S.; Hoffmeier, K.; Rotter, B.; T.T.; Westhaus, S.; Widera, M.
EPI_ISL_890188 to 890194, EPI_ISL_1233922 to 1233923	Gonoshasthaya-RNA Research Center, Gonoshasthaya-RNA Molecular Diagnostics and Research Center	Gonoshasthaya-RNA Research Center, Gonoshasthaya-RNA Molecular Diagnostics and Research Center	Adnan, N.; Ahmed; Akter, S.; Ali, T.; Azmuda, N.; Chaity, M.; Haq; Jahan, N.; Jamiruddin; Jamiruddin, M.; Khandker; Khondoker; Liza; M.A.; M.F.; M.R.; M.U.; Marzan, M.; Mou; Nahar, S.; Oishee, M.; S.M.; S.S.; Sharif, N.; Sharmin, S.; T.J.
EPI_ISL_774976	Gonoshasthya-RNA Molecular Diagnostic and Research Center	Gonoshasthya-RNA Molecular Diagnostic and Research Center	Firoz Ahmed; Maha Jamiruddin; Mahfuza Marzan; Md. Ahsanul Haq; Mohd. Raeed Jamiruddin; Mohib Ullah Khondoker; Mousumi Chaity; Mumtarin Jannat Oishee; Nafisa Azmuda; Nihad Adnan; Nowshin Jahan; Salma Akter; Sayeda Moriam Liza; Shahad Saif Khandker; Shahana Sharmin; Tamanna Ali; Taslin Jahan Mou
EPI_ISL_775019 to 775020, EPI_ISL_775213 to 775218	Gonoshasthya-RNA Molecular Research Center	Gonoshasthya-RNA Molecular Research Center	Firoz Ahmed; Maha Jamiruddin; Mahfuza Marzan; Md. Ahsanul Haq; Mohd. Raeed Jamiruddin; Mohib Ullah Khondoker; Mousumi Chaity; Mumtarin Jannat Oishee; Nafisa Azmuda; Nihad Adnan; Nowshin Jahan; Salma Akter; Sayeda Moriam Liza; Shahad Saif Khandker; Shahana Sharmin; Tamanna Ali; Taslin Jahan Mou
EPI_ISL_1181354 to 1181355, EPI_ISL_1181379 to 1181380, EPI_ISL_1181408, EPI_ISL_1181419, EPI_ISL_1181424, EPI_ISL_1181429, EPI_ISL_1181440 to 1181441, EPI_ISL_1181444 to 1181445, EPI_ISL_1181449, EPI_ISL_1181484 to 1181485, EPI_ISL_1219136			
see above	Gonçalo Moniz Institute, FIOCRUZ, Bahia	Laboratory of Respiratory Viruses and Measles, Oswaldo Cruz Institute, FIOCRUZ	Alice Sampaio Rocha; Ana Carolina Mendonca; Anna Carolina Paixao; Fernando Motta; Luciana Appolinario; Marilda Siqueira on behalf of the Fiocruz COVID-19 Genomic Surveillance Network; Paola Resende; Renata Serrano Lopes; Ricardo Khouri; Tiago Graf
EPI_ISL_415152	Gorgas Memorial Institute for Health Studies	Gorgas Memorial Institute for Health Studies	Alexander A. Martinez.; Ambar Moreno; Claudia Gonzalez; Danilo Franco; Elimelec Valdespino; Juan M. Pascale; Leyda Abrego; Oris Chavarria; Sandra Lopez-Verges; Yamilka Diaz
EPI_ISL_496603 to 496915, EPI_ISL_1225330 to 1225583	Gorgas Memorial Laboratory of Health Studies	Gorgas Memorial Laboratory of Health Studies	Adriana Weeden; Alejandra Valoy; Alexander A Martinez; Alexander Martinez; Ambar Moreno; Anyuri Ortiz; Brechla Moreno; Claudia Gonzalez; Claudia Gonzalez Sandra Lopez-Verges; Daniel Castillo; Danilo Franco; Davis Beltran; Dimelza Arauz; Elimelec Valdespino; Gretel Vasquez; Ilka Guerra; Isela Guerrero; Jessica Gondola; Jim Chang; Juan Miguel Pascale; Layda Abrego; Lisseth Saenz; Mabel Martinez-Montero; Maria Chen-German; Marlene Castillo; Melissa Gaitan; Oris Chavarria; Rita Corrales; Rita Rodriguez; Sandra Lopez-Verges; Yamilka Diaz; Yaneth Pitti; Zumara Chaverra
EPI_ISL_906306, EPI_ISL_1001457	Gorgas memorial Institute For Health Studies	Gorgas memorial Institute For Health Studies	Abrego L; Arauz D; Castillo D; Castillo M; Chavarria O; Diaz Y; Díaz Y; Franco D; Gaitan M; Gondola J; Gondola Y; González C; Lopez-Verges S.; Lopez-Verguez S; Martinez AA; Martinez Alexander A.; Moreno A; Moreno B; Pitti Y; Saenz L
EPI_ISL_1001458	Gorgas memorial Institute for Health Studies	Gorgas memorial Institute for Health Studies	Arauz D; Castillo D; Castillo M; Chavarria O; Díaz Y; Franco D; Gaitan M; Gondola J; González C; Lopez-Verges S.; Martinez AA; Moreno A; Moreno B; Pitti Y; Saenz L
EPI_ISL_1258459 to 1258462	Gorj County Directorate of Public Health	National Institute of Infectious Diseases-Prof. Dr. Matei Bals Molecular Diagnostics Laboratory	Corina Casangiu; Dan Otelea; Ionelia Nicolae; Leontina Banica; Marius Surleac; Ovidiu Vlaicu; Simona Paraschiv
EPI_ISL_524735 to 524736, EPI_ISL_586542 to 586545	Government General Hospital, Jam Khambhaliya, Devbhoomi Dwarka	Gujarat Biotechnology Research Centre	A M Kadri; Afzal Ansari; Apurvasinh Puvar; Chaitanya Joshi; Dinesh Kumar; Harish Matani; Harsh Bakshi; Janvi Raval; Komal Patel; Labdhi Pandya; Madhvi Joshi; Maharshi Pandya; Monika Gandhi; Nidhi Patel; Nikha Trivedi; Nitin Savaliya; Pinal Trivedi; R D Dixit; Raghawendra Kumar; Zarna Patel; Zuber Saiyed
EPI_ISL_586551 to 586554, EPI_ISL_590691 to 590692	Government Hospital,Veraval	Gujarat Biotechnology Research Centre	A M Kadri; Afzal Ansari; Apurvasinh Puvar; Chaitanya Joshi; Dinesh Kumar; Harsh Bakshi; Janvi Raval; Jignesh Parmar; Jitendra Bamrotia; Komal Patel; Labdhi Pandya; Madhvi Joshi; Maharshi Pandya; Monika Gandhi; Nidhi Patel; Nikha Trivedi; Nitin Savaliya; Pinal Trivedi; R D Dixit; Raghawendra Kumar; Zarna Patel; Zuber Saiyed
EPI_ISL_508207 to 508286	Government Medical College	National Institute of Biomedical Genomics	Arindam Maitra; Dhaval Khatri; Jyoti Iravane; Maitrik Dave; Saumitra Das
EPI_ISL_511930 to 511941	Government Medical College	National Institute of Biomedical Genomics - DBT's PAN-INDIA 1000 SARS--CoV-2 RNA Genome Sequencing Consortium	Arindam Maitra; Dhaval Khatri; Jyoti Iravane; Maitrik Dave; Saumitra Das
EPI_ISL_495020 to 495025	Government Medical College, Bhavnagar	Gujarat Biotechnology Research Centre	A M Kadri; Afzal Ansari; Apurvasinh Puvar; Chaitanya Joshi; Dinesh Kumar; Harsh Bakshi; Janvi Raval; Kairavi Desai; Komal Patel; Labdhi Pandya; Madhvi Joshi; Maharshi Pandya; Monika Gandhi; Nidhi Patel; Nikha Trivedi; Nitin Savaliya; Pinal Trivedi; R D Dixit; Raghawendra Kumar; Saklin Malek; Shirish Patel; Zarna Patel; Zuber Saiyed
EPI_ISL_1165088 to 1165092, EPI_ISL_1165096 to 1165134, EPI_ISL_1165136 to 1165137	Government Medical College, Patiala, Punjab	Indian Council of Medical Research-National Institute of Virology, Microbial Containment Complex	Pragya D. Yadav; Rupinder Bakshi
EPI_ISL_451154 to 451163, EPI_ISL_455017 to 455027, EPI_ISL_461478 to 461480, EPI_ISL_467039 to 467040, EPI_ISL_469029 to 469036, EPI_ISL_483821 to 483822	Government Medical College, Vadodara	Gujarat Biotechnology Research Centre	; A M Kadri; Afzal Ansari; Akanksha Verma; Amit Kanani; Ankit Hinsu; Apurvasinh Puvar; Armi Chaudhari; Bhavesh Modi; Bhavya Jindal; Camellia Chakraborty; Chaitanya Joshi; Dinesh Kumar; Fenil Patel; Gaurishankar Shrimali; Harsh Bakshi; Janvi Raval; Komal Patel; Labdhi Pandya; Madhvi Joshi; Maharshi Pandya; Manish Pattani; Meenakshi Shah; Monika Gandhi; Neelam Nathani; Neena Doshi; Nidhi Patel; Nikha Trivedi; Nitin Savaliya; Pinal Trivedi; Pooja P Doshi; Pragya Sharma; Pritesh Sabara; Priti Pandita; Priyanka P Vatsa; R D Dixit; R N Daveshwar; Raghawendra Kumar; Ramesh Pandit; Sharmistha Majumdar; Siddhant Kumar; Snehal Bagatharia; Tanuja Javadekar; Tejas Shah; Umang Mishra; Varsha Godbole; Zarna Patel; Zuber Saiyed
EPI_ISL_450781 to 450784	Government Medical College-Bhavnagar	Gujarat Biotechnology Research Centre	A M Kadri; Afzal Ansari; Akanksha Verma; Amit Kanani; Ankit Hinsu; Apurvasinh Puvar; Bhavesh Modi; Chaitanya Joshi; Dinesh Kumar; Gaurishankar Shrimali; Janvi Raval; Kairavi Desai; Komal Patel; Labdhi Pandya; Madhvi Joshi; Maharshi Pandya; Monika Gandhi; Neha Rajpara; Nidhi Patel; Nitin Savaliya; Pinal Trivedi; Pritesh Sabara; Priti Pandita; R D Dixit; Raghawendra Kumar; Ramesh Pandit; Saklain Malek; Shirish Patel; Snehal Bagatharia; Tejas Shah; Zarna Patel; Zuber Saiyed
EPI_ISL_1159385	Graha Medika Maternity Hospital	Institute of Tropical Disease, Universitas Airlangga	Aldise M Nastri; Diah R Kusumawati; Gatot Soegiarto; Jezzy R Dewantari; Kazufumi Shimizu; Krisnoadi Rahardjo; Laksmi Wulandari; Maria I Lusida; Resti Yudhawati; Rima R Prasetya; Soetjipto; Yasuko Mori
EPI_ISL_982857 to 982859, EPI_ISL_983063 to 983072, EPI_ISL_983075 to 983081, EPI_ISL_983083 to 983084, EPI_ISL_983086 to 983091, EPI_ISL_1302685 to 1302686, EPI_ISL_1302694 to 1302697, EPI_ISL_1302699 to 1302700, EPI_ISL_1302708 to 1302716, EPI_ISL_1312344 to 1312345, EPI_ISL_1312354 to 1312363, EPI_ISL_1314168 to 1314169, EPI_ISL_1314178 to 1314189, EPI_ISL_1314198 to 1314207			
see above	Gravity Diagnostics	Kentucky State Public Health Lab	Joshua Tobias; Karim George; Matthew Johnson; Rachel Zinner; Rhonda Lucas; Stephanie Lunn; Vaneet Arora; William Grooms
EPI_ISL_700543, EPI_ISL_700550	Great Brak River Clinic wc GBC	NHLS/UCT	Arash Iranzadeh; Bruna Galvao; Carolyn Williamson; Deelan Doolabh; Diana Hardie; Houriiyah Tegally; Innocent Mudau; Kruger Marais; Lynn Tyers;

			Marvin Hsiao; Stephen Korsman
EPI_ISL_960149	Great Brak River Clinic wc GBC	National Health Laboratory Service/UCT	Arash Iranzadeh; Bruna Galvao; Carolyn Williamson; Deelan Doolabh; Diana Hardie; Innocent Mudau; Kruger Marais; Lynn Tyers; Marvin Hsiao; Stephen Korsman
EPI_ISL_696466, EPI_ISL_696497, EPI_ISL_696501, EPI_ISL_696507	Great Brak River Clinic wc GBC & NHLS/UCT	KRISP, KZN Research Innovation and Sequencing Platform	Arash Iranzadeh; Bruna Galvao; Carolyn Williamson; Deelan Doolabh; Diana Hardie; Emanuel James San; Houriiyah Tegally; Innocent Mudau; Jennifer Giandhari; Kruger Marais; Lynn Tyers; Marvin Hsiao; Stephen Korsman; Sureshnee Pillay; Tulio de Oliveira
EPI_ISL_794818 to 794820, EPI_ISL_794822 to 794824	Greek Genome Center, Biomedical Research Foundation of the Academy of Athens (BRFAA)	Greek Genome Center, Biomedical Research Foundation of the Academy of Athens (BRFAA)	Christina Maria Kravvari; Dimitrios Thanos; Emmanouil Athanasiadis; Ioannis Vatsellas; Katerina Zoi; Thodoris Loupis
EPI_ISL_640041	Green Point CDC wc GPH	NHLS/UCT	Arash Iranzadeh; Bruna Galvao; Carolyn Williamson; Deelan Doolabh; Diana Hardie; Innocent Mudau; Kruger Marais; Lynn Tyers; Marvin Hsiao; Stephen Korsman
EPI_ISL_640040, EPI_ISL_640043, EPI_ISL_640045, EPI_ISL_640051 to 640055, EPI_ISL_640058, EPI_ISL_640060 to 640061, EPI_ISL_640066 to 640067, EPI_ISL_640084, EPI_ISL_640111 to 640112, EPI_ISL_640119 to 640120, EPI_ISL_640122, EPI_ISL_640128 to 640135, EPI_ISL_640137 to 640140, EPI_ISL_700493, EPI_ISL_700507, EPI_ISL_700534, EPI_ISL_700577, EPI_ISL_1040644 to 1040646, EPI_ISL_1040648 to 1040654, EPI_ISL_1040656 to 1040661, EPI_ISL_1040663, EPI_ISL_1040668, EPI_ISL_1040672 to 1040673, EPI_ISL_1040676, EPI_ISL_1040683 to 1040687, EPI_ISL_1040689 to 1040693, EPI_ISL_1040696 to 1040700, EPI_ISL_1040704 to 1040708, EPI_ISL_1040711, EPI_ISL_1040714, EPI_ISL_1040716, EPI_ISL_1040720, EPI_ISL_1040722 to 1040723, EPI_ISL_1040725 to 1040729, EPI_ISL_1040731, EPI_ISL_1040733, EPI_ISL_1040735, EPI_ISL_1040738, EPI_ISL_1040745, EPI_ISL_1040747 to 1040748, EPI_ISL_1040750 to 1040756, EPI_ISL_1040758 to 1040759, EPI_ISL_1040763, EPI_ISL_1040765, EPI_ISL_1040767 to 1040768, EPI_ISL_1040781 to 1040783, EPI_ISL_1040785 to 1040787, EPI_ISL_1040789, EPI_ISL_1040795 to 1040796, EPI_ISL_1040800 to 1040802, EPI_ISL_1040804, EPI_ISL_1040807 to 1040808, EPI_ISL_1040811 to 1040813, EPI_ISL_1040815 to 1040818, EPI_ISL_1040822			
see above	Groote Schuur Hospital wc GSH	NHLS/UCT	Arash Iranzadeh; Bruna Galvao; Carolyn Williamson; Deelan Doolabh; Diana Hardie; Innocent Mudau; Kruger Marais; Lynn Tyers; Marvin Hsiao; Stephen Korsman
EPI_ISL_960102 to 960103, EPI_ISL_960107, EPI_ISL_960109, EPI_ISL_960117, EPI_ISL_960126, EPI_ISL_960132 to 960133, EPI_ISL_960135 to 960136, EPI_ISL_960140, EPI_ISL_960142 to 960143, EPI_ISL_960145			
see above	Groote Schuur Hospital wc GSH	National Health Laboratory Service/UCT	Arash Iranzadeh; Bruna Galvao; Carolyn Williamson; Deelan Doolabh; Diana Hardie; Innocent Mudau; Kruger Marais; Lynn Tyers; Marvin Hsiao; Stephen Korsman
EPI_ISL_696464	Groote Schuur Hospital wc GSH & NHLS/UCT	KRISP, KZN Research Innovation and Sequencing Platform	Arash Iranzadeh; Bruna Galvao; Carolyn Williamson; Deelan Doolabh; Diana Hardie; Emanuel James San; Houriiyah Tegally; Innocent Mudau; Jennifer Giandhari; Kruger Marais; Lynn Tyers; Marvin Hsiao; Stephen Korsman; Sureshnee Pillay; Tulio de Oliveira
EPI_ISL_698105 to 698169, EPI_ISL_698172 to 699161, EPI_ISL_708827 to 708838	Group 42 (G42) Healthcare, Abu Dhabi, United Arab Emirates; Department of Health, The United Arab Emirates	G42 Healthcare	Ashish Koshy; Budoor Alqarni; Denghui Liu; Fang Chen; Hanif Khalak; Huanming Yang; Javier Quilez; Jian Wang; Junhua Li; Ke Liang; Long Lin; Mohammed Saifuddin Fasihuddin; Nan Qiao; Nawal Ahmed Mohamed Al Kaabi; Pauline Ogrodzki; Pei Wu; Peng Xiao; Pengjuan Liu; Rong Liu; Sally Mahmoud; Siyang Liu; Stephen S. Francis; Tao Ma; Vinay Kusuma; Walid Abbas Zaher; Weibin Liu; Wenjun He; Xavier Anton; Xin Jin; Xin Meng; Xinyu Huang; Xun Xu; Zhaorong Yuan
EPI_ISL_1259282 to 1259283	Group for molecular genetics of pathogens	Group for molecular genetics of pathogens	Anna S. Gladkikh; Areg A.Totolian; Ekaterina A. Goncharova; Valerya A. Sbarzaglia; Vladimir G. Dedkov
EPI_ISL_699570 to 699577, EPI_ISL_735250 to 735258	Group of Genetic Engeneering and Biotechnology, Federal Budget Institution of Science 'Central Research Institute of Epidemiology' of The Federal Service on Customers' Rights Protection and Human Well-being Surveillance	Group of Genetic Engeneering and Biotechnology, Federal Budget Institution of Science 'Central Research Institute of Epidemiology' of The Federal Service on Customers' Rights Protection and Human Well-being Surveillance	A.G.; A.S.; A.V.; Akimkin; Berlina; Bulanenko; Cherkashina; E.D.; E.V.; Golubeva; M.I.; O.Y.; Shipulina; Soloviova; Solovyova; Speranskaya; Tivanova; V.G.; V.P.; Valdokhina; Y.Y.; Zotova
EPI_ISL_710479	Group of Genetic Engineering and Biotechnology, Federal Budget Institution of Science 'Central Research Institute of Epidemiology' of The Federal Service on Customers' Rights Protection and Human Well-being Surveillance	Group of Genetic Engineering and Biotechnology, Federal Budget Institution of Science 'Central Research Institute of Epidemiology' of The Federal Service on Customers' Rights Protection and Human Well-being Surveillance	A.G.; A.S.; A.V.; Berlina; Bulanenko; Cherkashina; E.D.; E.V.; Golubeva; M.I.; O.Y. and Akimkin; Shipulina; Soloviova; Speranskaya; Tivanova; V.G.; V.P.; Valdokhina; Y.Y.; Zotova
EPI_ISL_981401 to 981405, EPI_ISL_981409 to 981424, EPI_ISL_981426 to 981451, EPI_ISL_981489 to 981491, EPI_ISL_981493 to 981496, EPI_ISL_981498 to 981531, EPI_ISL_982145, EPI_ISL_1085422 to 1085423, EPI_ISL_1085426, EPI_ISL_1085429 to 1085432, EPI_ISL_1085434, EPI_ISL_1085437, EPI_ISL_1085439, EPI_ISL_1085442, EPI_ISL_1085444 to 1085446, EPI_ISL_1085448, EPI_ISL_1085450, EPI_ISL_1085456 to 1085460, EPI_ISL_1085462 to 1085471, EPI_ISL_1085479 to 1085496, EPI_ISL_1085498, EPI_ISL_1085502 to 1085503, EPI_ISL_1085514, EPI_ISL_1085517, EPI_ISL_1085523 to 1085524, EPI_ISL_1085549, EPI_ISL_1085572, EPI_ISL_1085595, EPI_ISL_1085622, EPI_ISL_1085844 to 1085845, EPI_ISL_1085878, EPI_ISL_1085907, EPI_ISL_1088560, EPI_ISL_1088564, EPI_ISL_1088567 to 1088571, EPI_ISL_1110142 to 1110150, EPI_ISL_1110176			
see above	Groupe LCD	Department of Virology, Henri Mondor University Hospital, Assistance Publique Hôpitaux de Paris, Université Paris-Est Créteil, INSERM U955	Alexandre Soulier; Christophe Rodriguez; Elisabeth Trawinski; Guillaume Gricourt; Jean-Michel Pawlotsky; Melissa N'Debi; Slim Fourati; Vanessa Demontant
EPI_ISL_729737, EPI_ISL_729739 to 729740, EPI_ISL_729771, EPI_ISL_729774 to 729776, EPI_ISL_729781 to 729785, EPI_ISL_729787 to 729788, EPI_ISL_729790 to 729791, EPI_ISL_729793, EPI_ISL_792558 to 792559, EPI_ISL_1039001 to 1039007, EPI_ISL_1091882, EPI_ISL_1168736, EPI_ISL_1225677, EPI_ISL_1293217			
see above	Grubaugh Lab - Yale School of Public Health	Grubaugh Lab - Yale School of Public Health	Adam Moore; Akiko Iwasaki; Albert Ko; Alice Lu; Allison Nelson; Anderson Brito; Anne Wyllie; Annie Watkins; Arnau Casanovas; Catherine Muenker; Chaney Kalinich; Chantal Vogels; Charlese Dela Cruz; Isabel Ott; Isabell Ott; Jessica Rothman; Joseph Fauver; Mallery Breban; Maria Tokuyama; Marie L. Landry; Mary Petrone; Nathan Grubaugh; Patrick Wong; Peiwen Lu; Richard Martinello; Saad Omer; Shelli Farhadian; Tara Alpert
EPI_ISL_1123373	Grupo Tecnico de Vigilancia Sanitaria e Epidemiologica	Instituto Adolfo Lutz, Interdiciplinary Procedures Center, Strategic Laboratory	Caio Vinicius Dias Lopes; Claudia Regina Gonçalves; Claudio Tavares Sacchi; Erica Valessa Ramos Gomes; Karoline Rodrigues Campos
EPI_ISL_447734 to 447754	Grupo de Investigaciones Microbiológicas-UR (GIMUR), Departamento de Biología, Facultad de Ciencias Naturales, Universidad del Rosario, Bogotá, Colombia	Grupo de Investigaciones Microbiológicas-UR (GIMUR), Departamento de Biología, Facultad de Ciencias Naturales, Universidad del Rosario, Bogotá, Colombia Instituto Nacional de Salud, Bogotá, Colombia Icahn School of Medicine at Mount Sinai, New York, USA	Adriana Castillo; Alberto Paniz-Mondolfi; Ana S. Gonzalez-Reiche; Angelica Rico; Aníbal A. Teherán; Carolina Florez; Carolina Hernandez; David Martínez; Emilia Mia Sordillo; Esther C. Barros; Harm van Bakel; Jesús E. Jaimes; Juan David Ramírez; Laura Vega; Lisseth Pardo; Marina Muñoz; Martha L. Ospina; Matthew M. Hernandez; Nathalia Ballesteros; Sergio Castañeda; Sergio Gomez; Viviana Simon
EPI_ISL_1040921 to 1040926	Grupo de Investigación en Enfermedades Tropicales del Ejército (GINETEJ), Laboratorio de Referencia e Investigación, Dirección de Sanidad Ejército, Bogotá, Colombia	Centro de Investigaciones en Microbiología y Biotecnología-UR (CIMBIUR), Facultad de Ciencias Naturales, Universidad del Rosario, Bogotá, Colombia	Alberto Paniz-Mondolfi; Camilo Correa-Cardenas; Carlos A. Rincón; Claudia P. Mendez; Elizabeth K. Marquez; Frank de los Santos Ortiz; Juan David Ramírez; Julie J. Perez; Luz H. Patiño; Magda C. Oliveros; Maria C. Duque; Marina Muñoz; Nathalia Ballesteros; Sergio Castañeda
EPI_ISL_804009 to 804015	Grupo de Resistencia Antimicrobiana en bacterias patógenas y ambientales GRABPA	Facultad de Ciencias de la Vida, UNAB	Claudio Meneses; César Echeverría; Dayán Sanhueza; Eduardo Castro; Jorge Olivares; Macarena Bastías; Sebastián Wolter; Waldo Díaz
EPI_ISL_1273048 to 1273073	Guam Public Health Laboratory	Centers for Disease Control and Prevention Division of Viral Diseases, Pathogen Discovery	Anna Montmayeur; Anna Uehara; Ben L. Rambo-Martin; Clinton R. Paden; Dhwani Batra; Haibin Wang; Jasmine Padilla; Jing Zhang; Justin Lee; Katie Dillon; Krista Queen; Kristen Knipe; Kristine Lacek; Lori Rowe; Mark Burroughs; Matthew Schmerer; Mili Sheth; Peter W. Cook; Rachel Marine; Sam Shepard; Sarah Nobles; Shoshona Le; Suxiang Tong; Yan Li; Ying Tao
EPI_ISL_406531	Guangdong Provincial Center for Diseases Contorl and Prevention; Guangdong Provincial Public Health	Guangdong Provincial Center for Disease Control and Prevention	Baisheng Li; Changwen Ke; Feng Ruan; Guanhao He; Haojie Zhong; Huihong Deng; Jianfeng He; Jianpeng Xiao; Jianxiang Geng; Jianxiong Hu; Jie Wu; Jing Lu; Lifeng Lin; Lijun Liang; Lirong Zou; Min Kang; Qi Zhu; Shujiang Mei; Songjian Xiao; Tao Liu; Tie Song; Weilin Zeng; Wenjun Ma; Xing Li; Xiujuan Tang; Xue Zhuang; Xuguang Chen; Ying Wang; Yingchao Song; Yingtao Zhang; Yuhuang Liao; Zhe Liu
EPI_ISL_406533	Guangdong Provincial Center for Diseases Contorl and Prevention; Guangdong Provinical Public Health	Guangdong Provincial Center for Diseases Control and Prevention	Baisheng Li; Changwen Ke; Feng Ruan; Guanhao He; Haojie Zhong; Huihong Deng; Jianfeng He; Jianpeng Xiao; Jianxiang Geng; Jianxiong Hu; Jie Wu; Jing Lu; Lifeng Lin; Lijun Liang; Lirong Zou; Min Kang; Qi Zhu; Shujiang Mei; Songjian Xiao; Tao Liu; Tie Song; Weilin Zeng; Wenjun Ma; Xing Li; Xiujuan Tang; Xue Zhuang; Xuguang Chen; Ying Wang; Yingchao Song; Yingtao Zhang; Yuhuang Liao; Zhe Liu
EPI_ISL_403932 to 403937	Guangdong Provincial Center for Diseases Control and Prevention; Guangdong Provincial Public Health	Department of Microbiology, Guangdong Provincial Center for Diseases Control and Prevention	Baisheng Li; Changwen Ke; Feng Ruan; Guanhao He; Haojie Zhong; Huihong Deng; Jianfeng He; Jianpeng Xiao; Jianxiang Geng; Jianxiong Hu; Jie Wu; Jing Lu; Lifeng Lin; Lijun Liang; Lirong Zou; Min Kang; Qi Zhu; Shujiang Mei; Songjian Xiao; Tao Liu; Tie Song; Weilin Zeng; Wenjun Ma; Xing Li; Xiujuan Tang; Xue Zhuang; Xuguang Chen; Ying Wang; Yingchao Song; Yingtao Zhang; Yuhuang Liao; Zhe Liu
EPI_ISL_406534 to 406536	Guangdong Provincial Center for Diseases Control and Prevention; Guangdong Provincial Public Health	Guangdong Provincial Center for Diseases Control and Prevention	Baisheng Li; Changwen Ke; Feng Ruan; Guanhao He; Haojie Zhong; Huihong Deng; Jianfeng He; Jianpeng Xiao; Jianxiang Geng; Jianxiong Hu; Jie Wu; Jing Lu; Lifeng Lin; Lijun Liang; Lirong Zou; Min Kang; Qi Zhu; Shujiang Mei; Songjian Xiao; Tao Liu; Tie Song; Weilin Zeng; Wenjun Ma; Xing Li; Xiujuan Tang; Xue Zhuang; Xuguang Chen; Ying Wang; Yingchao Song; Yingtao Zhang; Yuhuang Liao; Zhe Liu
EPI_ISL_406538	Guangdong Provincial Center for Diseases Control and Prevention;Guangdong Provincial Institute of Public Health	Guangdong Provincial Center for Diseases Control and Prevention	Baisheng Li; Changwen Ke; Feng Ruan; Guanhao He; Haojie Zhong; Huihong Deng; Jianfeng He; Jianpeng Xiao; Jianxiang Geng; Jianxiong Hu; Jie Wu; Jing Lu; Lifeng Lin; Lijun Liang; Lirong Zou; Min Kang; Qi Zhu; Shujiang Mei; Songjian Xiao; Tao Liu; Tie Song; Weilin Zeng; Wenjun Ma; Xing Li; Xiujuan

			Tang; Xue Zhuang; Xuguang Chen; Ying Wang; Yingchao Song; Yingtao Zhang; Yuhuang Liao; Zhe Liu
EPI_ISL_428440 to 428478	Guangdong Provincial Center for Diseases Control and Prevention;Guangdong Provincial Institute of Public Health	School of Public Health, The University of Hong Kong	Bosheng Li; Hanri Zeng; Haogao Gu; Hui-Ling Yen; Jie Wu; Leo L.M. Poon; Lijun Liang; Tie Song; Yao Hu; Yingchao Song; Zhencui Li
EPI_ISL_413850 to 413902	Guangdong Provincial Institution of Public Health, Guangdong Provinical Center for Disease Control and Prevention	Guangdong Provincial Institution of Public Health	Andrew Rambaut; Bo Peng; Changwen Ke; Chuming Liang; Huanying Zheng; Huifang Lin; Jing Lu; Jingju Peng; Jiufeng Sun; Josh Quick; Juan Su; Kang Min; Kuibiao Li; Lilian Zeng; Liu Zhe; Louis du Plessis; Minfeng Liang; Moritz Kraemer; Nick Loman; Nuno Faria; Oliver Pybus; Pingping Zhou; Qianlin Xiong; Ru bai; Ruilin Sun; Runyu Yuan; Sarah François; Shisong Fang; Song Tie; Tao Liu; Verity Hill; Wei Li; Wenjun Ma; Wenzhe Su; Xi Tang; Jing Lu
EPI_ISL_444969	Guangzhou Eighth People's Hospital (Jiahe Sector)	Institute of Human Virology, Zhongshan School of Medicine, Sun Yat-sen University	Bingfeng Liu; Fang Li; Fei Yu; Feng Huang; Fengyu Hu; Hui Zhang; Huimin Fan; Jun Liu; Junsong Zhang; Kai Deng; Mang Shi; Ruosu Ying; Ting Pan; Xu Zhang; Yiwen Zhang
EPI_ISL_509695 to 509703, EPI_ISL_509710	Guatemala Ministry of Public Health	Pathogen Discovery, Respiratory Viruses Branch, Division of Viral Diseases, Centers for Disease Control and Prevention	Anna Uehara; Clinton Paden; Haibin Wang; Jing Zhang; Krista Queen; Suxiang Tong; Yan Li; Ying Tao
EPI_ISL_1040761	Guguletu CHC	NHLS/UCT	Arash Iranzadeh; Bruna Galvao; Carolyn Williamson; Deelan Doolabh; Diana Hardie; Innocent Mudau; Kruger Marais; Lynn Tyers; Marvin Hsiao; Stephen Korsman
EPI_ISL_640069 to 640071, EPI_ISL_640104, EPI_ISL_700415, EPI_ISL_700455, EPI_ISL_700481, EPI_ISL_700491, EPI_ISL_700503, EPI_ISL_700512, EPI_ISL_700514, EPI_ISL_700530, EPI_ISL_700576, EPI_ISL_700578, EPI_ISL_1040666 to 1040667, EPI_ISL_1040669, EPI_ISL_1040671, EPI_ISL_1040675, EPI_ISL_1040678, EPI_ISL_1040710, EPI_ISL_1040713, EPI_ISL_1040715, EPI_ISL_1040718, EPI_ISL_1040721, EPI_ISL_1040724, EPI_ISL_1040732, EPI_ISL_1040734, EPI_ISL_1040793, EPI_ISL_1040805			
see above	Guguletu CHC wc GDH	NHLS/UCT	Arash Iranzadeh; Bruna Galvao; Carolyn Williamson; Deelan Doolabh; Diana Hardie; Innocent Mudau; Kruger Marais; Lynn Tyers; Marvin Hsiao; Stephen Korsman
EPI_ISL_960154 to 960158, EPI_ISL_960163, EPI_ISL_960167, EPI_ISL_960174	Guguletu CHC wc GDH	National Health Laboratory Service/UCT	Arash Iranzadeh; Bruna Galvao; Carolyn Williamson; Deelan Doolabh; Diana Hardie; Innocent Mudau; Kruger Marais; Lynn Tyers; Marvin Hsiao; Stephen Korsman
EPI_ISL_435055 to 435056, EPI_ISL_444482 to 444486, EPI_ISL_447534 to 447546, EPI_ISL_458103 to 458113	Gujarat Biotechnology Research Centre	Gujarat Biotechnology Research Centre	; A M Kadri; Afzal Ansari; Akanksha Verma; Amit Kanani; Anjali Rajwal; Anjali Rajwar; Ankit Hinsu; Apurvasinh Puvar; Armi Chaudhari; Bhavesh Modi; Bhavya Jindal; Binita Aring; Camellia Chakraborty; Chaitanya Joshi; Dinesh Kumar; Dipa Kinariwala; Dipeshwari Shewale; Disha Patel; Gaurishankar Shrimali; Geeta Vaghela; Janvi Raval; Kairavi Joshi; Kamlesh J Upadhyay; Komal Patel; Labdhi Pandya; Madhvi Joshi; Maharshi Pandya; Monika Gandhi; Neelam Nathani; Neeta Khandelwal; Neha Rajpara; Nidhi Patel; Nidhi Sood; Nitin Savaliya; Pinal Trivedi; Pooja P Doshi; Pragya Sharma; Pranay Shah; Pritesh Sabara; Priti Pandita; Priyanka P Vatsa; R D Dixit; Raghawendra Kumar; Ramesh Pandit; Sharmistha Majumdar; Siddhant Kumar; Snehal Bagatharia; Sonia Barve; Tejas Shah; Umang Mishra; Zarna Patel; Zuber Saiyed
EPI_ISL_455573 to 455574, EPI_ISL_455580 to 455582, EPI_ISL_471157, EPI_ISL_489904, EPI_ISL_489956 to 489958, EPI_ISL_547608 to 547610, EPI_ISL_547666 to 547676, EPI_ISL_547683 to 547684, EPI_ISL_547705, EPI_ISL_660802 to 660805, EPI_ISL_660869 to 660881, EPI_ISL_660892 to 660893, EPI_ISL_942415 to 942418, EPI_ISL_942482 to 942494, EPI_ISL_942505 to 942506, EPI_ISL_942941, EPI_ISL_1113182 to 1113185			
see above	Gundersen Clinical Microbiology Laboratory	Kabara Cancer Research Institute	Craig S. Richmond; Paraic A. Kenny
EPI_ISL_418185 to 418186	Gundersen Molecular Diagnostic Laboratory	Kabara Cancer Research Institute	Craig S. Richmond & Paraic A. Kenny
EPI_ISL_418184, EPI_ISL_418187 to 418189, EPI_ISL_419651 to 419652, EPI_ISL_422453, EPI_ISL_422459, EPI_ISL_422461 to 422465, EPI_ISL_426159 to 426161, EPI_ISL_429843 to 429848, EPI_ISL_435394 to 435402, EPI_ISL_436891 to 436900, EPI_ISL_455568 to 455572, EPI_ISL_455575 to 455579, EPI_ISL_471144 to 471156, EPI_ISL_489896 to 489903, EPI_ISL_489905 to 489955, EPI_ISL_547602 to 547607, EPI_ISL_547611 to 547665, EPI_ISL_547677 to 547682, EPI_ISL_547685 to 547704, EPI_ISL_547706 to 547869, EPI_ISL_660795 to 660801, EPI_ISL_660806 to 660868, EPI_ISL_660882 to 660891, EPI_ISL_660894 to 661173, EPI_ISL_806807 to 806808, EPI_ISL_830649, EPI_ISL_861472 to 861475, EPI_ISL_906146, EPI_ISL_942408 to 942414, EPI_ISL_942419 to 942481, EPI_ISL_942495 to 942504, EPI_ISL_942507 to 942514, EPI_ISL_942516 to 942894, EPI_ISL_942940, EPI_ISL_942942 to 942950, EPI_ISL_1113176 to 1113181, EPI_ISL_1113186 to 1113225, EPI_ISL_1239453 to 1239457			
see above	Gundersen Molecular Diagnostics Laboratory	Kabara Cancer Research Institute	Craig S. Richmond; Craig S. Richmond & Paraic A. Kenny; Paraic A. Kenny
EPI_ISL_1257796 to 1257811	Gunma Prefectural Institute of Public Health and Environmental Sciences	Gunma Prefectural Institute of Public Health and Environmental Sciences	Daisuke Shinoda; Hiroyuki Tsukagoshi; Kentaro Itokawa; Makoto Kuroda; Mariko Saito; Masanori Hashino; Rina Tanaka; Tsuyoshi Sekizuka
EPI_ISL_479850 to 479854, EPI_ISL_479896 to 479901, EPI_ISL_480015 to 480020, EPI_ISL_480169 to 480177, EPI_ISL_684127, EPI_ISL_684220 to 684288, EPI_ISL_684325 to 684338, EPI_ISL_684566 to 684577, EPI_ISL_685279 to 685281, EPI_ISL_689047 to 689076, EPI_ISL_689612, EPI_ISL_689617, EPI_ISL_689686 to 689691, EPI_ISL_690210 to 690253, EPI_ISL_692047, EPI_ISL_692316 to 692327, EPI_ISL_896764 to 896794, EPI_ISL_901458 to 901490, EPI_ISL_901966 to 902018, EPI_ISL_1123435, EPI_ISL_1123437, EPI_ISL_1123451 to 1123452, EPI_ISL_1123467, EPI_ISL_1123525, EPI_ISL_1123527, EPI_ISL_1123529, EPI_ISL_1123531 to 1123532, EPI_ISL_1123534, EPI_ISL_1123536, EPI_ISL_1123538, EPI_ISL_1123540 to 1123541, EPI_ISL_1123543, EPI_ISL_1123545, EPI_ISL_1123547 to 1123548, EPI_ISL_1123550, EPI_ISL_1123552 to 1123553, EPI_ISL_1123555, EPI_ISL_1123557, EPI_ISL_1123559, EPI_ISL_1123561, EPI_ISL_1123563 to 1123564, EPI_ISL_1123566, EPI_ISL_1123568, EPI_ISL_1123570, EPI_ISL_1123572, EPI_ISL_1123574, EPI_ISL_1123576 to 1123577, EPI_ISL_1123579, EPI_ISL_1123581, EPI_ISL_1123583 to 1123584, EPI_ISL_1123586, EPI_ISL_1123588, EPI_ISL_1123590 to 1123591, EPI_ISL_1123593, EPI_ISL_1123595, EPI_ISL_1123597, EPI_ISL_1123599, EPI_ISL_1123601 to 1123602, EPI_ISL_1123604, EPI_ISL_1123606, EPI_ISL_1123608 to 1123609, EPI_ISL_1123611, EPI_ISL_1123613, EPI_ISL_1123615, EPI_ISL_1123617 to 1123618, EPI_ISL_1123620, EPI_ISL_1123622, EPI_ISL_1123624, EPI_ISL_1123626 to 1123627, EPI_ISL_1123629, EPI_ISL_1123631 to 1123632, EPI_ISL_1123634, EPI_ISL_1123636 to 1123637, EPI_ISL_1123639, EPI_ISL_1123641, EPI_ISL_1123643, EPI_ISL_1123645 to 1123646, EPI_ISL_1123648, EPI_ISL_1123650 to 1123651, EPI_ISL_1123653, EPI_ISL_1123655, EPI_ISL_1123657, EPI_ISL_1123659 to 1123660, EPI_ISL_1123662, EPI_ISL_1123664, EPI_ISL_1123666			
see above	Gunma Prefectural Institute of Public Health and Environmental Sciences	Pathogen Genomics Center, National Institute of Infectious Diseases	Hajime Kamiya; Hiroyuki Tsukagoshi; Kentaro Itokawa; Makoto Kuroda; Masanori Hashino; Motoi Suzuki; Rina Tanaka; Tsuyoshi Sekizuka
EPI_ISL_745201	Guntur General Hospital	CSIR-Centre for Cellular and Molecular Biology	Archana Bharadwaj Siva; B Himasri; Blessy B John; Divya Tej Sowpati; Dr.P.Shashikala Reddy; Dr.S.Pavani; Dr.Satyaprasad; Dr.V.Sudha Rani; Karthik Bharadwaj Tallapaka; Lamuk Zaveri; M Soujanya Reddy; Namami Gaur; Nikhil Hajirnis; Onkar Kulkarni; Payel Mukherjee; Pratheusa Maccha; Priya Singh; Purushotham Vodnala; Rakesh K Mishra; Sakshi Shambhavi; Shagufta Khan; Sofia Banu; Tulasi Nagabandi; Viswagithe S L
EPI_ISL_421454, EPI_ISL_421458, EPI_ISL_421481, EPI_ISL_421487, EPI_ISL_421491	H Beatriz Angelo	Instituto Nacional de Saude (INSA)	Guiomar et al
EPI_ISL_418003, EPI_ISL_418007 to 418008, EPI_ISL_418019 to 418022	H Braga	Instituto Nacional de Saude (INSA)	Guiomar et al
EPI_ISL_421457	H Dr Nelio Mendonca - Funchal	Instituto Nacional de Saude (INSA)	Guiomar et al
EPI_ISL_418026, EPI_ISL_421449, EPI_ISL_421459 to 421461	H Dr. Nelio Mendonca - Funchal	Instituto Nacional de Saude (INSA)	Guiomar et al
EPI_ISL_418023, EPI_ISL_421466 to 421467	H Evora	Instituto Nacional de Saude (INSA)	Guiomar et al
EPI_ISL_418018	H Garcia de Orta	Instituto Nacional de Saude (INSA)	Guiomar et al
EPI_ISL_421446 to 421448	H Guimaraes	Instituto Nacional de Saude (INSA)	Guiomar et al
EPI_ISL_418025, EPI_ISL_421462, EPI_ISL_421468 to 421471, EPI_ISL_421488, EPI_ISL_421490, EPI_ISL_421492, EPI_ISL_421495			
see above	H Santarem	Instituto Nacional de Saude (INSA)	Guiomar et al
EPI_ISL_1069185	H.M. Corachea General Hospital	Research Institute for Tropical Medicine	Catalino Demetria; Daria Manalo; Edelwisa Mercado; Francisco Gerardo Polotan; Hannah Leah Morito; Inez Andrea Medado; John Leonard Chan; Joseph Hughes; Kirstyn Brunker; Ma Angelica Tujan; Othoniel Jan Onza
EPI_ISL_455587	H.R.H. Maha Chakri Sirindhorn Medical Center	National Institute of Health. Department of medical Sciences, Ministry of Public Health, Thailand	Chittaganpitch; Malinee; Okada; Parnmen; Phuygun; Pilailuk; Siripaporn; Sittiporn; Sunthareeya; Thanadachakul; Thanutsapa; Waicharoen; Warawan; Wongboot
EPI_ISL_1084739, EPI_ISL_1172014 to 1172017	HC_FMUSP	Laboratório de Parasitologia Médica - Instituto de Medicina Tropical - Universidade de São Paulo	Brazil-UK Centre for Arbovirus Discovery Diagnosis Genomics and Epidemiology (CADDE) Genomic Network - Instituto de Medicina Tropical
EPI_ISL_955234, EPI_ISL_955239	HE CMN LA RAZA	BIOBANCO / COCTI	Arias C; Borja-Aburto VH; Grajales-Muñiz C; Grande R; Isa P; López S; Muñoz-Medina JE; Ochoa Carrera LA; Rojas-Mendoza T; Santacruz Tinoco CE; Sánchez A; Taboada B
EPI_ISL_596250 to 596263, EPI_ISL_596268 to 596317, EPI_ISL_596357 to 596377	HELIX LCC	WHO National Influenza Centre Russian Federation	Andrey Komissarov; Anna Ivanova; Artem Fadeev; Daria Danilenko; Dmitry Bazhenov; Kseniya Komissarova
EPI_ISL_602329 to 602411, EPI_ISL_602417 to 602461, EPI_ISL_639948 to 639973, EPI_ISL_733023 to 733153, EPI_ISL_733301 to 733391, EPI_ISL_733393, EPI_ISL_733396 to 733401, EPI_ISL_733417 to 733458, EPI_ISL_872972 to 873039, EPI_ISL_1039124 to 1039137			

see above	HELIX LLC	WHO National Influenza Centre Russian Federation	Andrey Komissarov; Anna Ivanova; Artem Fadeev; Daria Danilenko; Dmitry Bazhenov; Dmitry Lioznov; Elena Nabieva; Georgii Bazykin; Ksenia Safina; Kseniya Komissarova; Maria Pisareva; Maria Timofeeva; Mikhail Bakaev; Tamila Musaeva; Veronika Eder
EPI_ISL_1117779, EPI_ISL_1117791 to 1117792, EPI_ISL_1117797, EPI_ISL_1117800 to 1117801, EPI_ISL_1117806, EPI_ISL_1117808, EPI_ISL_1117831 to 1117833, EPI_ISL_1117870 to 1117872, EPI_ISL_1117882 to 1117885, EPI_ISL_1117919 to 1117920, EPI_ISL_1117922, EPI_ISL_1117928 to 1117936, EPI_ISL_1117951 to 1118028, EPI_ISL_1118081 to 1118082, EPI_ISL_1180715 to 1180751			
see above	HG Pharma GmbH	Bergthaler laboratory, CeMM Research Center for Molecular Medicine of the Austrian Academy of Sciences	Andreas Bergthaler; Anna Schedl; Bekir Erguner; Benedikt Agerer; Christoph Bock; Fabian Amman; Jan Laine; Lukas Endler; Maelle Le Moing; Martin Senekowitsch; Michael Schuster; Thomas Penz
EPI_ISL_1173206	HG Pharma GmbH	Institute of Virology Department of Hygiene, Microbiology and Public Health at Innsbruck Medical University	Andre Volland; Barbara Falkensammer; David Bante; Dorothee von Laer; Heribert Stoiber; Janine Kimpel; Lisa Pipperger; Lydia Riepler; Wegene Borena
EPI_ISL_955246	HGOMF 60 TLANEPANTLA	BIOBANCO / COCTI	Arias C; Borja-Aburto VH; Grajales-Muñiz C; Grande R; Isa P; López S; Muñoz-Medina JE; Ochoa Carrera LA; Rojas-Mendoza T; Santacruz Tinoco CE; Sánchez A; Taboada B
EPI_ISL_955236, EPI_ISL_955245	HGR 1 VICENTE GUERRERO	BIOBANCO / COCTI	Arias C; Borja-Aburto VH; Grajales-Muñiz C; Grande R; Isa P; López S; Muñoz-Medina JE; Ochoa Carrera LA; Rojas-Mendoza T; Santacruz Tinoco CE; Sánchez A; Taboada B
EPI_ISL_955247	HGR 200 TECAMAC	BIOBANCO / COCTI	Arias C; Borja-Aburto VH; Grajales-Muñiz C; Grande R; Isa P; López S; Muñoz-Medina JE; Ochoa Carrera LA; Rojas-Mendoza T; Santacruz Tinoco CE; Sánchez A; Taboada B
EPI_ISL_955242 to 955243, EPI_ISL_955250 to 955253, EPI_ISL_955255	HGSMF 26 CABO SAN LUCAS	BIOBANCO / COCTI	Arias C; Borja-Aburto VH; Grajales-Muñiz C; Grande R; Isa P; López S; Muñoz-Medina JE; Ochoa Carrera LA; Rojas-Mendoza T; Santacruz Tinoco CE; Sánchez A; Taboada B
EPI_ISL_955256	HGZ 1 CARLOS MC GREGOR	BIOBANCO / COCTI	Arias C; Borja-Aburto VH; Grajales-Muñiz C; Grande R; Isa P; López S; Muñoz-Medina JE; Ochoa Carrera LA; Rojas-Mendoza T; Santacruz Tinoco CE; Sánchez A; Taboada B
EPI_ISL_955249	HGZ 2A PASO-TRONCOSO	BIOBANCO / COCTI	Arias C; Borja-Aburto VH; Grajales-Muñiz C; Grande R; Isa P; López S; Muñoz-Medina JE; Ochoa Carrera LA; Rojas-Mendoza T; Santacruz Tinoco CE; Sánchez A; Taboada B
EPI_ISL_955240 to 955241	HGZ 30 MEXICALI	BIOBANCO / COCTI	Arias C; Borja-Aburto VH; Grajales-Muñiz C; Grande R; Isa P; López S; Muñoz-Medina JE; Ochoa Carrera LA; Rojas-Mendoza T; Santacruz Tinoco CE; Sánchez A; Taboada B
EPI_ISL_955257	HGZ 32 VILLA COAPA	BIOBANCO / COCTI	Arias C; Borja-Aburto VH; Grajales-Muñiz C; Grande R; Isa P; López S; Muñoz-Medina JE; Ochoa Carrera LA; Rojas-Mendoza T; Santacruz Tinoco CE; Sánchez A; Taboada B
EPI_ISL_955233	HGZMF 1 LA PAZ	BIOBANCO / COCTI	Arias C; Borja-Aburto VH; Grajales-Muñiz C; Grande R; Isa P; López S; Muñoz-Medina JE; Ochoa Carrera LA; Rojas-Mendoza T; Santacruz Tinoco CE; Sánchez A; Taboada B
EPI_ISL_955238	HI CMN LA RAZA	BIOBANCO / COCTI	Arias C; Borja-Aburto VH; Grajales-Muñiz C; Grande R; Isa P; López S; Muñoz-Medina JE; Ochoa Carrera LA; Rojas-Mendoza T; Santacruz Tinoco CE; Sánchez A; Taboada B
EPI_ISL_1272638 to 1272652	HI Dept. of Health, State Laboratories Division	Centers for Disease Control and Prevention Division of Viral Diseases, Pathogen Discovery	Anna Montmayeur; Anna Uehara; Ben L. Rambo-Martin; Clinton R. Paden; Dhwani Batra; Haibin Wang; Jasmine Padilla; Jing Zhang; Justin Lee; Katie Dillon; Krista Queen; Kristen Knipe; Kristine Lacek; Lori Rowe; Mark Burroughs; Matthew Schmerer; Mili Sheth; Peter W. Cook; Rachel Marine; Sam Shepard; Sarah Nobles; Shoshona Le; Suxiang Tong; Yan Li; Ying Tao
EPI_ISL_751578, EPI_ISL_751603, EPI_ISL_751651, EPI_ISL_751673 to 751675, EPI_ISL_751758, EPI_ISL_751761, EPI_ISL_751767, EPI_ISL_751772, EPI_ISL_903583			
see above	HI Dept. of Health, State Laboratories Division	Genomics and Discovery, Respiratory Viruses Branch, Division of Viral Diseases, Centers for Disease Control and Prevention	Anna Montmayeur; Anna Uehara; Ben L. Rambo-Martin; Clinton R. Paden; Dhwani Batra; Haibin Wang; Jasmine Padilla; Jing Zhang; Justin Lee; Krista Queen; Lori Rowe; Mark Burroughs; Mili Sheth; Peter W. Cook; Rachel Marine; Sarah Nobles; Suxiang Tong; Yan Li; Ying Tao
EPI_ISL_426420 to 426421	HI Dept. of Health, State Laboratories Division	Pathogen Discovery, Respiratory Viruses Branch, Division of Viral Diseases, Centers for Disease Control and Prevention	Alison S. Laufer Halpin; Anna Uehara; Christopher A. Elkins; Clinton R. Paden; Haibin Wang; Jing Zhang; Krista Queen; Mary S. Keckler; Rachel Marine; Suxiang Tong; Yan Li; Ying Tao
EPI_ISL_1094209, EPI_ISL_1094221, EPI_ISL_1094276, EPI_ISL_1094772 to 1094775	HI Dept. of Health, State Laboratories Division	Respiratory Viruses Branch, Division of Viral Diseases, Centers for Disease Control and Prevention	Anna Montmayeur; Anna Uehara; Ben L. Rambo-Martin; Clinton R. Paden; Dhwani Batra; Haibin Wang; Jasmine Padilla; Jing Zhang; Justin Lee; Krista Queen; Lori Rowe; Mark Burroughs; Mili Sheth; Peter W. Cook; Rachel Marine; Sarah Nobles; Suxiang Tong; Yan Li; Ying Tao
EPI_ISL_860849	HIA BÉGIN Service de Biologie	National Reference Center for Viruses of Respiratory Infections, Institut Pasteur, Paris	Angela Brisebarre; Camille Capel; Etienne Simon-Lorière; Marion Barbet; Maud Vanpeene; Merens Audrey; Méline Bizard; Sylvie Behillil; Sylvie van der Werf; Vincent Enouf
EPI_ISL_941270	HIA PERCY - lab biologie	National Reference Center for Viruses of Respiratory Infections, Institut Pasteur, Paris	Angela Brisebarre; Camille Capel; Etienne Simon-Lorière; Marion Barbet; Maud Vanpeene; Méline Bizard; Sylvie Behillil; Sylvie van der Werf; Vincent Enouf; Vincent Foissaud
EPI_ISL_860850 to 860851	HIA PERCY, Biologie médicale	National Reference Center for Viruses of Respiratory Infections, Institut Pasteur, Paris	Angela Brisebarre; Camille Capel; Etienne Simon-Lorière; Foissaud Vincent; Marion Barbet; Maud Vanpeene; Méline Bizard; Sylvie Behillil; Sylvie van der Werf; Vincent Enouf
EPI_ISL_1007590, EPI_ISL_1007641	HIB	Laboratory of genomics and metagenomics, Institute of Microbiology, University Hospital Centre and University of Lausanne, Switzerland	Claire Bertelli; Damien Jacot; Gilbert Greub; Sébastien Aeby; Trestan Pillonel
EPI_ISL_1170956	HIV Molecular Lab, Ethiopian Public Health Institute	HIV Molecular Lab	A.G.; Abichu, G.; Ahyong, V.; D. and Tato, C.; Damena, D.; Detweiler, A.; Fahsbender, E.; Gutema, G.; Kalantar, K.; Kidane, E.; Leta; Neff, N.; S.K.; Tan, M.; Tessema; Vanaerschot, M.; Weldemariam
EPI_ISL_1169919	HIV Molecular Laboratory, Ethiopian Public Health Institute	HIV Molecular Laboratory, Ethiopian Public Health Institute	A.G.; Abichu, G.; Ahyong, V.; D. and Tato, C.; Damena, D.; Detweiler, A.; Fahsbender, E.; Gutema, G.; Kalantar, K.; Kidane, E.; Leta; Neff, N.; S.K.; Tan, M.; Tessema; Vanaerschot, M.; Weldemariam
EPI_ISL_1170958	HIV Molecular Laboratory, Ethiopian Public Health Institute, Ethiopia	HIV molecular lab, Ethiopian Public Health Institute, Ethiopian	A.G.; Abichu, G.; Ahyong, V.; D. and Tato, C.; Damena, D.; Detweiler, A.; Fahsbender, E.; Gutema, G.; Kalantar, K.; Kidane, E.; Leta; Neff, N.; S.K.; Tan, M.; Tessema; Vanaerschot, M.; Weldemariam
EPI_ISL_1170957	HIV Molecular laboratory, Ethiopian Public Health Institute	HIV Molecular Laboratory	A.G.; Abichu, G.; Ahyong, V.; D. and Tato, C.; Damena, D.; Detweiler, A.; Fahsbender, E.; Gutema, G.; Kalantar, K.; Kidane, E.; Leta; Neff, N.; S.K.; Tan, M.; Tessema; Vanaerschot, M.; Weldemariam
EPI_ISL_463307	HK Molecular Pathology Disgnostic Centre	Hong Kong Department of Health	Chan Rickjason C.W.; Cheng Peter K.C.; Lam Edman T.K.; Mak Gannon C.K.; Tsang Dominic N.C.
EPI_ISL_914808	HLE - ASOCIACION HOGAR PARA ANCIANOS PRESBITERO JAFET JIMENEZ MORALES DE GRECIA	Inciensa, Instituto Costarricense de Investigación y Enseñanza en Nutrición y Salud	Adriana Godínez; Claudio Soto-Garita; Estela Cordero; Francisco Duarte; Hebleen Porras; Melany Calderón & Mariel López
EPI_ISL_914827	HLE - ASOCIACION DE ALBERGUE PARA ANCIANOS DE GOLFITO	Inciensa, Instituto Costarricense de Investigación y Enseñanza en Nutrición y Salud	Adriana Godínez; Claudio Soto-Garita; Estela Cordero; Francisco Duarte; Hebleen Porras; Melany Calderón & Mariel López
EPI_ISL_914807	HLE - ASOCIACION HOGAR DE ANCIANOS SANTIAGO CRESPO CALVO	Inciensa, Instituto Costarricense de Investigación y Enseñanza en Nutrición y Salud	Adriana Godínez; Claudio Soto-Garita; Estela Cordero; Francisco Duarte; Hebleen Porras; Melany Calderón & Mariel López
EPI_ISL_914826	HLE - MANOS ABIERTAS	Inciensa, Instituto Costarricense de Investigación y Enseñanza en Nutrición y Salud	Adriana Godínez; Claudio Soto-Garita; Estela Cordero; Francisco Duarte; Hebleen Porras; Melany Calderón & Mariel López
EPI_ISL_1239447	HOPITAL MARIN HENDAYE	CNR Virus des Infections Respiratoires - France SUD	Antonin Bal; Bruno Lina; Gregory Destras; Gwendolyne Burfin; Hadrien Regue; Laurence Josset; Martine Valette; Quentin Semanas
EPI_ISL_1313712	HOPITAL NORD OUEST - CH VILLEFRANCHE	CNR Virus des Infections Respiratoires - France SUD	Antonin Bal; Bruno Lina; Gregory Destras; Gwendolyne Burfin; Hadrien Regue; Laurence Josset; Martine Valette; Quentin Semanas
EPI_ISL_1253543	HOPITAL NOTRE-DAME LA MISERICORDE	CNR Virus des Infections Respiratoires - France SUD	Antonin Bal; Bruno Lina; Gregory Destras; Gwendolyne Burfin; Hadrien Regue; Laurence Josset; Martine Valette; Quentin Semanas
EPI_ISL_860899	HOPITAL PAUL BROUSSE - Service Microbiologie	National Reference Center for Viruses of Respiratory	Angela Brisebarre; Camille Capel; Etienne Simon-Lorière; Marion Barbet; Maud Vanpeene; Méline Bizard; Roque-Afonso Anne-Marie; Sylvie Behillil;

		Infections, Institut Pasteur, Paris	Sylvie van der Werf; Vincent Enouf
EPI_ISL_1200838 to 1200842, EPI_ISL_1200844 to 1200846, EPI_ISL_1200848, EPI_ISL_1200851, EPI_ISL_1200853, EPI_ISL_1200855 to 1200859, EPI_ISL_1241876, EPI_ISL_1265479			
see above	HOPITAL SAINT ANDRE	CNR Virus des Infections Respiratoires - France SUD	Antonin Bal; Bruno Lina; Gregory Destras; Gwendolyne Burfin; Hadrien Regue; Laurence Josset; Martine Valette; Quentin Semanas
EPI_ISL_912560 to 912562, EPI_ISL_912599	HOPITAL SIMONE VEIL	National Reference Center for Viruses of Respiratory Infections, Institut Pasteur, Paris	Angela Brisebarre; Camille Capel; Etienne Simon-Lorière; Marion Barbet; Maud Vanpeene; Moreau Farida; Méline Bizard; Sylvie Behillil; Sylvie van der Werf; Vincent Enouf
EPI_ISL_1191681 to 1191682, EPI_ISL_1191705, EPI_ISL_1191722 to 1191725, EPI_ISL_1265464, EPI_ISL_1265477, EPI_ISL_1265486, EPI_ISL_1265494, EPI_ISL_1265500, EPI_ISL_1265546, EPI_ISL_1265608 to 1265609			
see above	HOPITAL UNIVERSITAIRE DE FORT DE FRANCE	CNR Virus des Infections Respiratoires - France SUD	Antonin Bal; Bruno Lina; Gregory Destras; Gwendolyne Burfin; Hadrien Regue; Laurence Josset; Martine Valette; Quentin Semanas
EPI_ISL_912621	HOPITAL YVES LE FOLL Laboratoire de Biologie Médicale	National Reference Center for Viruses of Respiratory Infections, Institut Pasteur, Paris	Angela Brisebarre; Camille Capel; Chrevet Laurent; Etienne Simon-Lorière; Marion Barbet; Maud Vanpeene; Méline Bizard; Sylvie Behillil; Sylvie van der Werf; Vincent Enouf
EPI_ISL_445359	HOSP. SANTIAGO ORIENTE DR. LUIS TISNE B.	Instituto de Salud Publica de Chile	Alejandra Acevedo; Andrés E Castillo; Bárbara Parra; Carolina Tambley; Gabriel Leal; Jaime Lagos; Jorge Fernandez; Loredana Arata; Patricia Bustos; Paz Tapia; Rodrigo Fasce; Winston Andrade
EPI_ISL_445323	HOSP.ENFERMEDADES INFECCIOSAS	Instituto de Salud Publica de Chile	Alejandra Acevedo; Andrés E Castillo; Bárbara Parra; Carolina Tambley; Gabriel Leal; Jaime Lagos; Jorge Fernandez; Loredana Arata; Patricia Bustos; Paz Tapia; Rodrigo Fasce; Winston Andrade
EPI_ISL_682239	HOSPITAL CIUDAD NEILY	Inciensa, Instituto Costarricense de Investigación y Enseñanza en Nutrición y Salud	Adriana Godinez & Melany Calderon; Claudio Soto-Garita; Estela Cordero; Francisco Duarte; Hebleen Porras
EPI_ISL_418250, EPI_ISL_419707, EPI_ISL_1159173 to 1159182	HOSPITAL CLINIC	Instituto de Salud Carlos III	A. Monzón; F. Casas; I. Jiménez; I. Marcos; I. Marcos M.A; I. VILA ESTAPE; Iglesias-Caballero; JORDI; M. Camarero; M. Camarero S. Pozo F. Casas I. Jiménez; M. Cuesta; M. González-Esguevillas; M. Molinero Calamita; M. Zaballos; M.A; P. Jiménez; P. Zaballos; S. Cuesta; S. Iglesias-Caballero; S. Juliá; S. Pozo; S. Varona; Sandonís; V. Vázquez-Morón
EPI_ISL_445281	HOSPITAL CLINICO DEL SUR	Instituto de Salud Publica de Chile	Alejandra Acevedo; Andrés E Castillo; Bárbara Parra; Carolina Tambley; Gabriel Leal; Jaime Lagos; Jorge Fernandez; Loredana Arata; Patricia Bustos; Paz Tapia; Rodrigo Fasce; Winston Andrade
EPI_ISL_445275 to 445276	HOSPITAL CLINICO FUSAT	Instituto de Salud Publica de Chile	Alejandra Acevedo; Andrés E Castillo; Bárbara Parra; Carolina Tambley; Gabriel Leal; Jaime Lagos; Jorge Fernandez; Loredana Arata; Patricia Bustos; Paz Tapia; Rodrigo Fasce; Winston Andrade
EPI_ISL_954792, EPI_ISL_954796, EPI_ISL_1059955, EPI_ISL_1059976 to 1059977, EPI_ISL_1159133 to 1159135, EPI_ISL_1159137 to 1159147, EPI_ISL_1167198, EPI_ISL_1261460 to 1261462, EPI_ISL_1261476 to 1261477, EPI_ISL_1261517, EPI_ISL_1261532, EPI_ISL_1262118, EPI_ISL_1262120 to 1262121, EPI_ISL_1262123 to 1262124, EPI_ISL_1262126			
see above	HOSPITAL COMARCAL DE MELILLA	Instituto de Salud Carlos III	A. Monzón; F. Casas; I. Illescas, S.; I. Jiménez; I. Roman, S.; I. Román, S.; I.ROMAN SOTO; I.Roman, S.; I.Roman. S.; Iglesias-Caballero; M. Camarero; M. Sandonís; P. Zaballos; S. Camarero; S. Cuesta; S. Iglesias-Caballero; S. Pozo; S. Sandonís; S. Varona; SERGIO; V. Camarero; V. Vázquez; V. Vázquez-Morón; Vázquez-Morón
EPI_ISL_445378	HOSPITAL DE BULNES	Instituto de Salud Publica de Chile	Alejandra Acevedo; Andrés E Castillo; Bárbara Parra; Carolina Tambley; Gabriel Leal; Jaime Lagos; Jorge Fernandez; Loredana Arata; Patricia Bustos; Paz Tapia; Rodrigo Fasce; Winston Andrade
EPI_ISL_445335	HOSPITAL DE CALBUCO	Instituto de Salud Publica de Chile	Alejandra Acevedo; Andrés E Castillo; Bárbara Parra; Carolina Tambley; Gabriel Leal; Jaime Lagos; Jorge Fernandez; Loredana Arata; Patricia Bustos; Paz Tapia; Rodrigo Fasce; Winston Andrade
EPI_ISL_445251, EPI_ISL_445305, EPI_ISL_445354, EPI_ISL_445369 to 445370	HOSPITAL DE CARABINEROS	Instituto de Salud Publica de Chile	Alejandra Acevedo; Andrés E Castillo; Bárbara Parra; Carolina Tambley; Gabriel Leal; Jaime Lagos; Jorge Fernandez; Loredana Arata; Patricia Bustos; Paz Tapia; Rodrigo Fasce; Winston Andrade
EPI_ISL_419238	HOSPITAL DE CRUCES.	Instituto de Salud Carlos III	A. Monzón; F. Casas; I. Aranzamendi, M.; I. Jiménez; Iglesias-Caballero; M. Camarero; M. Cuesta; M. González-Esguevillas; M. Molinero Calamita; M. Zaballos; P. Jiménez; S. Juliá; S. Pozo; S. Varona
EPI_ISL_1059937	HOSPITAL DE LA VEGA LORENZO GUIRAO	Instituto de Salud Carlos III	A. Monzón; F. Casas; I. Jiménez; I. Álvarez, J.; Iglesias-Caballero; M. Sandonís; P. Zaballos; S. Camarero; S. Cuesta; S. Pozo; S. Varona; V. Vázquez-Morón
EPI_ISL_1059934 to 1059935	HOSPITAL DE MERIDA - SERVICIO DE CONTABILIDAD - C.D.02	Instituto de Salud Carlos III	A. Monzón; F. Casas; I. Jiménez; I. Sánchez, J.; Iglesias-Caballero; M. Sandonís; P. Zaballos; S. Camarero; S. Cuesta; S. Pozo; S. Varona; V. Vázquez-Morón
EPI_ISL_1059981	HOSPITAL DE MOSTOLES	Instituto de Salud Carlos III	A. Monzón; F. Casas; I. Jiménez; I. López, M.; M. Sandonís; P. Zaballos; S. Cuesta; S. Iglesias-Caballero; S. Pozo; S. Varona; V. Camarero; Vázquez-Morón
EPI_ISL_914815 to 914819	HOSPITAL DE NIÑOS DR. CARLOS SAENZ HERRERA	Inciensa, Instituto Costarricense de Investigación y Enseñanza en Nutrición y Salud	Adriana Godínez; Claudio Soto-Garita; Estela Cordero; Francisco Duarte; Hebleen Porras; Melany Calderón & Cristian Pérez-Corrales
EPI_ISL_445300	HOSPITAL DE RANCAGUA	Instituto de Salud Publica de Chile	Alejandra Acevedo; Andrés E Castillo; Bárbara Parra; Carolina Tambley; Gabriel Leal; Jaime Lagos; Jorge Fernandez; Loredana Arata; Patricia Bustos; Paz Tapia; Rodrigo Fasce; Winston Andrade
EPI_ISL_445298	HOSPITAL DE SAN FERNANDO	Instituto de Salud Publica de Chile	Alejandra Acevedo; Andrés E Castillo; Bárbara Parra; Carolina Tambley; Gabriel Leal; Jaime Lagos; Jorge Fernandez; Loredana Arata; Patricia Bustos; Paz Tapia; Rodrigo Fasce; Winston Andrade
EPI_ISL_445352, EPI_ISL_445360, EPI_ISL_445368	HOSPITAL DEL PROFESOR	Instituto de Salud Publica de Chile	Alejandra Acevedo; Andrés E Castillo; Bárbara Parra; Carolina Tambley; Gabriel Leal; Jaime Lagos; Jorge Fernandez; Loredana Arata; Patricia Bustos; Paz Tapia; Rodrigo Fasce; Winston Andrade
EPI_ISL_906539	HOSPITAL DEPARTAMENTAL DE VILLAVICENCIO	Instituto Nacional de Salud- Dirección de Investigación en Salud Pública, Universidad de los Andes- Applied genomics research group, Vicerrectoria de Investigación y Creación, Universidad de los Andes- Systems and Computing Engineering Department	Carlos Franco-Muñoz; Diego A. Álvarez-Díaz; Diego Andrés Prada; Gerardo Santamaría Jorge Duitama; Héctor Alejandro Ruiz-Moreno; Jhonnatan Reales-González; Jorge Ivan Diaz; Julian Naizaque; Katherine Laiton-Donato; Laura Natalia Gonzalez; Magdalena Wiesner; Marcela Mercado-Reyes; Maria T. Herrera-Sepúlveda; Martha Lucia Ospina Martinez; Mauricio Pacheco-Montealegre; Sheryll Corchuelo; Silvia Restrepo-Restrepo
EPI_ISL_906535, EPI_ISL_906537, EPI_ISL_906547	HOSPITAL DEPARTAMENTAL DE VILLAVICENCIO E.S.E.	Instituto Nacional de Salud- Dirección de Investigación en Salud Pública, Universidad de los Andes- Applied genomics research group, Vicerrectoria de Investigación y Creación, Universidad de los Andes- Systems and Computing Engineering Department	Carlos Franco-Muñoz; Diego A. Álvarez-Díaz; Diego Andrés Prada; Gerardo Santamaría Jorge Duitama; Héctor Alejandro Ruiz-Moreno; Jhonnatan Reales-González; Jorge Ivan Diaz; Julian Naizaque; Katherine Laiton-Donato; Laura Natalia Gonzalez; Magdalena Wiesner; Marcela Mercado-Reyes; Maria T. Herrera-Sepúlveda; Martha Lucia Ospina Martinez; Mauricio Pacheco-Montealegre; Sheryll Corchuelo; Silvia Restrepo-Restrepo
EPI_ISL_914821 to 914823, EPI_ISL_1067596, EPI_ISL_1067611, EPI_ISL_1196433	HOSPITAL DR. ENRIQUE BALTODANO BRICEÑO	Inciensa, Instituto Costarricense de Investigación y Enseñanza en Nutrición y Salud	Adriana Godínez; Caterina Guzmán; Claudio Soto-Garita; Estela Cordero; Francisco Duarte; Hebleen Porras; Melany Calderón; Melany Calderón & Adriana Bermúdez-Espinoza; Melany Calderón & Mariel López; Nazareth Ruiz & Adriana Bermúdez-Espinoza; Nazareth Ruiz & Mariel López
EPI_ISL_914795, EPI_ISL_1067589, EPI_ISL_1201438	HOSPITAL DR. FERNANDO ESCALANTE PRADILLA	Inciensa, Instituto Costarricense de Investigación y Enseñanza en Nutrición y Salud	Adriana Godínez; Caterina Guzmán; Claudio Soto-Garita; Estela Cordero; Francisco Duarte; Hebleen Porras; Melany Calderón; Melany Calderón & Mariel López; Melany Calderón & Nicole Vargas-Víquez; Nazareth Ruiz & Nicole Vargas-Víquez
EPI_ISL_1196430	HOSPITAL DR. MAX PERALTA JIMENEZ	Inciensa, Instituto Costarricense de Investigación y Enseñanza en Nutrición y Salud	Adriana Godínez; Claudio Soto-Garita; Estela Cordero; Francisco Duarte; Hebleen Porras; Melany Calderón & Mónica Charpentier-Artavia
EPI_ISL_914840	HOSPITAL DR. RAUL BLANCO CERVANTES	Inciensa, Instituto Costarricense de Investigación y Enseñanza en Nutrición y Salud	Adriana Godínez; Claudio Soto-Garita; Estela Cordero; Francisco Duarte; Hebleen Porras; Melany Calderón & Rebeca Ruiz-Sánchez
EPI_ISL_914801	HOSPITAL DR. TOMAS CASAS CASAJUS	Inciensa, Instituto Costarricense de Investigación y Enseñanza en Nutrición y Salud	Adriana Godínez; Claudio Soto-Garita; Estela Cordero; Francisco Duarte; Hebleen Porras; Melany Calderón & Mariel López
EPI_ISL_445270, EPI_ISL_445338 to 445348, EPI_ISL_447119	HOSPITAL DR.HERNAN HENRIQUEZ ARAVENA	Instituto de Salud Publica de Chile	Alejandra Acevedo; Andrés E Castillo; Bárbara Parra; Carolina Tambley; Gabriel Leal; Jaime Lagos; Jorge Fernandez; Loredana Arata; Patricia Bustos; Paz Tapia; Rodrigo Fasce; Winston Andrade

EPI_ISL_445310, EPI_ISL_445325, EPI_ISL_445329, EPI_ISL_445365 to 445366, EPI_ISL_445371	HOSPITAL DR.SOTERO DEL RIO	Instituto de Salud Publica de Chile	Alejandra Acevedo; Andrés E Castillo; Bárbara Parra; Carolina Tambley; Gabriel Leal; Jaime Lagos; Jorge Fernandez; Loredana Arata; Patricia Bustos; Paz Tapia; Rodrigo Fasce; Winston Andrade
EPI_ISL_445308, EPI_ISL_445313 to 445314, EPI_ISL_445364	HOSPITAL EL CARMEN DR.LUIS VALENTIN F.	Instituto de Salud Publica de Chile	Alejandra Acevedo; Andrés E Castillo; Bárbara Parra; Carolina Tambley; Gabriel Leal; Jaime Lagos; Jorge Fernandez; Loredana Arata; Patricia Bustos; Paz Tapia; Rodrigo Fasce; Winston Andrade
EPI_ISL_445319, EPI_ISL_445321	HOSPITAL FELIX BULNES	Instituto de Salud Publica de Chile	Alejandra Acevedo; Andrés E Castillo; Bárbara Parra; Carolina Tambley; Gabriel Leal; Jaime Lagos; Jorge Fernandez; Loredana Arata; Patricia Bustos; Paz Tapia; Rodrigo Fasce; Winston Andrade
EPI_ISL_445372	HOSPITAL FF.AA. "CIRUJANO C. GUZMAN	Instituto de Salud Publica de Chile	Alejandra Acevedo; Andrés E Castillo; Bárbara Parra; Carolina Tambley; Gabriel Leal; Jaime Lagos; Jorge Fernandez; Loredana Arata; Patricia Bustos; Paz Tapia; Rodrigo Fasce; Winston Andrade
EPI_ISL_418247	HOSPITAL GENERAL DE SEGOVIA	Instituto de Salud Carlos III	A. Monzón; F. Casas; I. Hernando-Real S.; I. Jiménez; Iglesias-Caballero; M. Camarero; M. Cuesta; M. González-Esguevillas; M. Molinero Calamita; M. Zaballos; P. Jiménez; S. Juliá; S. Pozo; S. Varona
EPI_ISL_954795, EPI_ISL_1159132	HOSPITAL GENERAL RIO CARRION	Instituto de Salud Carlos III	A. Monzón; ANTONIA; F. Casas; I. García, A.; I. Jiménez; I.GARCIA CASTRO; Iglesias-Caballero; M. Camarero; M. Sandonís; P. Zaballos; S. Cuesta; S. Iglesias-Caballero; S. Pozo; S. Sandonís; S. Varona; V. Camarero; V. Vázquez; Vázquez-Morón
EPI_ISL_1059982, EPI_ISL_1159227 to 1159228, EPI_ISL_1262050	HOSPITAL GENERAL UNIVERSITARIO DE CIUDAD REAL	Instituto de Salud Carlos III	A. Monzón; F. Casas; I. Illescas; I. Jiménez; I.ILLESCAS FERNANDEZ-BERMEJO; I.Illescas; Iglesias-Caballero; M. S.; M. SOLEDAD; M. Sandonís; M.S.; P. Zaballos; S. Camarero; S. Cuesta; S. Pozo; S. Varona; V. Vázquez-Morón
EPI_ISL_1059945	HOSPITAL GENERAL UNIVERSITARIO DE ELCHE	Instituto de Salud Carlos III	A. Monzón; F. Casas; I. De Gonzalo, N.; I. Jiménez; Iglesias-Caballero; M. Sandonís; P. Zaballos; S. Camarero; S. Cuesta; S. Pozo; S. Varona; V. Vázquez-Morón
EPI_ISL_1159198 to 1159209, EPI_ISL_1261519	HOSPITAL GENERAL UNIVERSITARIO REINA SOFIA	Instituto de Salud Carlos III	A. Monzón; F. Casas; I. Jiménez; I.NUÑEZ TRIGUEROS; I.Nuñez; Iglesias-Caballero; M. Sandonís; M.L; Mª LUZ; P. Zaballos; S. Camarero; S. Cuesta; S. Pozo; S. Varona; V. Vázquez-Morón
EPI_ISL_1059923, EPI_ISL_1059927 to 1059933, EPI_ISL_1159155 to 1159166, EPI_ISL_1159222 to 1159224, EPI_ISL_1261464 to 1261468, EPI_ISL_1261473 to 1261475, EPI_ISL_1261481, EPI_ISL_1261520, EPI_ISL_1261531, EPI_ISL_1262066, EPI_ISL_1262068 to 1262069, EPI_ISL_1262071 to 1262072, EPI_ISL_1262074 to 1262075, EPI_ISL_1262077			
see above	HOSPITAL GENERAL UNIVERSITARIO SANTA LUCIA	Instituto de Salud Carlos III	A; A. Monzón; ANA; F. Casas; I. BLAZQUEZ ABELLAN; I. Blazquez, A.; I. Blázquez, A.; I. Jiménez; I.BLAZQUEZ ABELLAN; I.Blazquez; I.Blazquez, A.; Iglesias-Caballero; M. Camarero; M. Sandonís; P. Zaballos; S. Camarero; S. Cuesta; S. Iglesias-Caballero; S. Pozo; S. Varona; Sandonís; V. Camarero; V. Vázquez-Morón; Vázquez-Morón
EPI_ISL_1261478, EPI_ISL_1261494	HOSPITAL GENERAL VIRGEN DE LA LUZ	Instituto de Salud Carlos III	A. Monzón; F. Casas; I. Jiménez; I.Rodriguez; M. Sandonís; M.J; P. Zaballos; S. Cuesta; S. Iglesias-Caballero; S. Pozo; S. Varona; V. Camarero; Vázquez-Morón
EPI_ISL_1067592	HOSPITAL GOLFITO MANUEL MORA VALVERDE	Inciensa, Instituto Costarricense de Investigación y Enseñanza en Nutrición y Salud	Adriana Godínez; Claudio Soto-Garita; Estela Cordero; Francisco Duarte; Hebleen Porras; Melany Calderón & Mariel López
EPI_ISL_445286, EPI_ISL_445337	HOSPITAL HANGA ROA	Instituto de Salud Publica de Chile	Alejandra Acevedo; Andrés E Castillo; Bárbara Parra; Carolina Tambley; Gabriel Leal; Jaime Lagos; Jorge Fernandez; Loredana Arata; Patricia Bustos; Paz Tapia; Rodrigo Fasce; Winston Andrade
EPI_ISL_445331 to 445332	HOSPITAL HERMINDA MARTIN CHILLAN	Instituto de Salud Publica de Chile	Alejandra Acevedo; Andrés E Castillo; Bárbara Parra; Carolina Tambley; Gabriel Leal; Jaime Lagos; Jorge Fernandez; Loredana Arata; Patricia Bustos; Paz Tapia; Rodrigo Fasce; Winston Andrade
EPI_ISL_1059938 to 1059942, EPI_ISL_1059948 to 1059949, EPI_ISL_1059951 to 1059954, EPI_ISL_1059957 to 1059974, EPI_ISL_1059980, EPI_ISL_1059993 to 1059995, EPI_ISL_1261482, EPI_ISL_1262039, EPI_ISL_1262078			
see above	HOSPITAL J.M. MORALES MESEGUER	Instituto de Salud Carlos III	A. Monzón; F. Casas; I. Antequera, P.; I. Antequera; I. Antequera, P.; I. Jiménez; I.Antequera, P.; Iglesias-Caballero; M. Camarero; M. Sandonís; P; P. Zaballos; S. Camarero; S. Cuesta; S. Iglesias-Caballero; S. Pozo; S. Varona; Sandonís; V. Camarero; V. Vázquez-Morón; Vázquez-Morón
EPI_ISL_445336	HOSPITAL LAS HIGUERAS DE TALCAHUANO	Instituto de Salud Publica de Chile	Alejandra Acevedo; Andrés E Castillo; Bárbara Parra; Carolina Tambley; Gabriel Leal; Jaime Lagos; Jorge Fernandez; Loredana Arata; Patricia Bustos; Paz Tapia; Rodrigo Fasce; Winston Andrade
EPI_ISL_1159183 to 1159193, EPI_ISL_1261471 to 1261472, EPI_ISL_1261483 to 1261493, EPI_ISL_1261500 to 1261501	HOSPITAL LOS ARCOS	Instituto de Salud Carlos III	A. Monzón; F. Casas; I. CAMARA SIMON; I. Camara; I. Jiménez; I.Camara; M; M. Camarero; M. Sandonís; MARGARITA; P. Zaballos; S. Cuesta; S. Iglesias-Caballero; S. Pozo; S. Varona; Sandonís; V. Camarero; V. Vázquez-Morón; Vázquez-Morón
EPI_ISL_914814, EPI_ISL_914834	HOSPITAL METROPOLITANO	Inciensa, Instituto Costarricense de Investigación y Enseñanza en Nutrición y Salud	Adriana Godínez; Claudio Soto-Garita; Estela Cordero; Francisco Duarte; Hebleen Porras; Melany Calderón & Margarita Lee-Lui
EPI_ISL_914836 to 914839, EPI_ISL_1067613 to 1067614, EPI_ISL_1067623 to 1067625, EPI_ISL_1196431	HOSPITAL MEXICO	Inciensa, Instituto Costarricense de Investigación y Enseñanza en Nutrición y Salud	Adriana Godínez; Caterina Guzmán; Claudio Soto-Garita; Estela Cordero; Francisco Duarte; Hebleen Porras; Melany Calderón; Melany Calderón & Mariel López; Melany Calderón & Teresita Somogyi; Nazareth Ruiz & Teresita Somogyi
EPI_ISL_445289, EPI_ISL_445301	HOSPITAL NAVAL PUERTO WILLIAMS	Instituto de Salud Publica de Chile	Alejandra Acevedo; Andrés E Castillo; Bárbara Parra; Carolina Tambley; Gabriel Leal; Jaime Lagos; Jorge Fernandez; Loredana Arata; Patricia Bustos; Paz Tapia; Rodrigo Fasce; Winston Andrade
EPI_ISL_445327, EPI_ISL_445353	HOSPITAL PADRE HURTADO	Instituto de Salud Publica de Chile	Alejandra Acevedo; Andrés E Castillo; Bárbara Parra; Carolina Tambley; Gabriel Leal; Jaime Lagos; Jorge Fernandez; Loredana Arata; Patricia Bustos; Paz Tapia; Rodrigo Fasce; Winston Andrade
EPI_ISL_445246	HOSPITAL PUERTO MONTT	Instituto de Salud Publica de Chile	Alejandra Acevedo; Andrés E Castillo; Bárbara Parra; Carolina Tambley; Gabriel Leal; Jaime Lagos; Jorge Fernandez; Loredana Arata; Patricia Bustos; Paz Tapia; Rodrigo Fasce; Winston Andrade
EPI_ISL_1261540	HOSPITAL RAFAEL MENDEZ	Instituto de Salud Carlos III	A. Monzón; E; F. Casas; I. Jiménez; I.Cascales; M. Sandonís; P. Zaballos; S. Cuesta; S. Iglesias-Caballero; S. Pozo; S. Varona; V. Camarero; Vázquez-Morón
EPI_ISL_445268 to 445269, EPI_ISL_445280, EPI_ISL_445284, EPI_ISL_445288, EPI_ISL_445293 to 445295	HOSPITAL REG.LAUTARO NAVARRO AVARIA	Instituto de Salud Publica de Chile	Alejandra Acevedo; Andrés E Castillo; Bárbara Parra; Carolina Tambley; Gabriel Leal; Jaime Lagos; Jorge Fernandez; Loredana Arata; Patricia Bustos; Paz Tapia; Rodrigo Fasce; Winston Andrade
EPI_ISL_445296	HOSPITAL REGIONAL DE COYHAIQUE	Instituto de Salud Publica de Chile	Alejandra Acevedo; Andrés E Castillo; Bárbara Parra; Carolina Tambley; Gabriel Leal; Jaime Lagos; Jorge Fernandez; Loredana Arata; Patricia Bustos; Paz Tapia; Rodrigo Fasce; Winston Andrade
EPI_ISL_593772 to 593776	HOSPITAL REGIONAL LAMBAYEQUE	GENOMA MAYOR	Franklin R. Aguilar-Gamboa; Heber Silva-Díaz; Juan J. Bonifacio-Briceño; Luis M. López-Serquén; Luis Salcedo-Mejía; Marco E. Mechán-Llontop and Juan P. Cárdenas.; Percy O. Tullume-Vergara; Ramsés Salas-Asencios
EPI_ISL_906541	HOSPITAL SAN JOSE DE MAICAO	Instituto Nacional de Salud- Dirección de Investigación en Salud Pública, Universidad de los Andes- Applied genomics research group, Vicerrectoria de Investigación y Creación, Universidad de los Andes- Systems and Computing Engineering Department	Carlos Franco-Muñoz; Diego A. Álvarez-Díaz; Diego Andrés Prada; Gerardo Santamaría Jorge Duitama; Héctor Alejandro Ruiz-Moreno; Jhonnatan Reales-González; Jorge Ivan Diaz; Julian Naizaque; Katherine Laiton-Donato; Laura Natalia Gonzalez; Magdalena Wiesner; Marcela Mercado-Reyes; Maria T. Herrera-Sepúlveda; Martha Lucia Ospina Martinez; Mauricio Pacheco-Montealegre; Sheryll Corchuelo; Silvia Restrepo-Restrepo
EPI_ISL_445307	HOSPITAL SAN JOSE DE MAIPO	Instituto de Salud Publica de Chile	Alejandra Acevedo; Andrés E Castillo; Bárbara Parra; Carolina Tambley; Gabriel Leal; Jaime Lagos; Jorge Fernandez; Loredana Arata; Patricia Bustos; Paz Tapia; Rodrigo Fasce; Winston Andrade
EPI_ISL_682241, EPI_ISL_682243, EPI_ISL_682245 to 682246, EPI_ISL_682253 to 682254, EPI_ISL_682257, EPI_ISL_682262 to 682264, EPI_ISL_682266 to 682274, EPI_ISL_1067604, EPI_ISL_1196434 to 1196437			
see above	HOSPITAL SAN JUAN DE DIOS	Inciensa, Instituto Costarricense de Investigación y Enseñanza en Nutrición y Salud	Adriana Godinez & Melany Calderon; Adriana Godínez; Claudio Soto-Garita; Estela Cordero; Francisco Duarte; Hebleen Porras; Melany Calderón & Daniel Cascante-Serrano; Melany Calderón & Melissa Carazo-Gutiérrez
EPI_ISL_445304, EPI_ISL_445349 to 445351,	HOSPITAL SAN JUAN DE DIOS	Instituto de Salud Publica de Chile	Alejandra Acevedo; Andrés E Castillo; Bárbara Parra; Carolina Tambley; Gabriel Leal; Jaime Lagos; Jorge Fernandez; Loredana Arata; Patricia Bustos;

EPI_ISL_445373 to 445377, EPI_ISL_449800			Paz Tapia; Rodrigo Fasce; Winston Andrade
EPI_ISL_1159168 to 1159172	HOSPITAL SAN PEDRO DE ALCANTARA - C.D. 05	Instituto de Salud Carlos III	A. Monzón; F. Casas; GUADALUPE; I. Jiménez; I. RODRIGUEZ RODRIGUEZ; M. Camarero; P. Zaballos; S. Cuesta; S. Iglesias-Caballero; S. Pozo; S. Varona; Sandonís; V. Vázquez-Morón
EPI_ISL_682242, EPI_ISL_682255	HOSPITAL SAN VICENTE DE PAUL	Inciensa, Instituto Costarricense de Investigación y Enseñanza en Nutrición y Salud	Adriana Godinez; Adriana Godinez & Melany Calderon; Claudio Soto-Garita; Estela Cordero; Francisco Duarte; Hebleen Porras; Melany Calderon & Mariel López
EPI_ISL_417007	HOSPITAL SANTA MARIA NAI	Instituto de Salud Carlos III	A. Monzón; I. García Costa, J.; Iglesias-Caballero; M. Camarero S. Pozo F. Casas I. Jiménez; M. Cuesta; M. González-Esguevillas; M. Molinero Calamita; M. Zaballos; P. Jiménez; S. Juliá; S. Varona
EPI_ISL_1060011	HOSPITAL SEVERO OCHOA	Instituto de Salud Carlos III	A. Monzón; F. Casas; I. Jiménez; I. Reyes, S.; Iglesias-Caballero; M. Sandonís; P. Zaballos; S. Camarero; S. Cuesta; S. Pozo; S. Varona; V. Vázquez-Morón
EPI_ISL_418253, EPI_ISL_419240, EPI_ISL_419709	HOSPITAL TXAGORRITXU	Instituto de Salud Carlos III	A. Monzón; C; F. Casas; I. Gomez-Gonzalez C.; I. Gómez; I. Gómez, C.; I. Jiménez; Iglesias-Caballero; M. Camarero; M. Camarero S. Pozo F. Casas I. Jiménez; M. Cuesta; M. González-Esguevillas; M. Molinero Calamita; M. Zaballos; P. Jiménez; S. Juliá; S. Pozo; S. Varona
EPI_ISL_1059936, EPI_ISL_1059975, EPI_ISL_1059978 to 1059979, EPI_ISL_1059983, EPI_ISL_1059996 to 1060010, EPI_ISL_1060012, EPI_ISL_1261459, EPI_ISL_1261469 to 1261470, EPI_ISL_1261504 to 1261506, EPI_ISL_1261521 to 1261530, EPI_ISL_1262040, EPI_ISL_1262042 to 1262044, EPI_ISL_1262046 to 1262047, EPI_ISL_1262049			
see above	HOSPITAL UNIV. MIGUEL SERVET	Instituto de Salud Carlos III	A; A. M.; A. Monzón; F. Casas; I. Milagro; I. Jiménez; I. Martinez, A.; I. Milagro; I.Milagro; I.Milagro, A.; Iglesias-Caballero; M. Camarero; M. Sandonís; P. Zaballos; S. Camarero; S. Cuesta; S. Iglesias-Caballero; S. Pozo; S. Varona; Sandonís; V. Vázquez-Morón
EPI_ISL_1159216 to 1159217, EPI_ISL_1261457, EPI_ISL_1261498 to 1261499, EPI_ISL_1261534 to 1261537	HOSPITAL UNIVERS MARQUES DE VALDECILLA	Instituto de Salud Carlos III	A. Monzón; C; CARLOS ANTONIO; F. Casas; I. Jiménez; I.SALAS VENERO; I.Salas; Iglesias-Caballero; M. Sandonís; P. Zaballos; S. Camarero; S. Cuesta; S. Iglesias-Caballero; S. Pozo; S. Varona; V. Camarero; V. Vázquez-Morón; Vázquez-Morón
EPI_ISL_1059922, EPI_ISL_1059989 to 1059992, EPI_ISL_1262111 to 1262112, EPI_ISL_1262114 to 1262115, EPI_ISL_1262117	HOSPITAL UNIVERSITARIO DE CEUTA	Instituto de Salud Carlos III	A. Monzón; F. Casas; I. López, J.; I. Jiménez; I. López, J.; I.Hijano, S.; Iglesias-Caballero; M. Camarero; M. Sandonís; P. Zaballos; S. Camarero; S. Cuesta; S. Iglesias-Caballero; S. Pozo; S. Varona; Sandonís; V. Camarero; V. Vázquez-Morón; Vázquez-Morón
EPI_ISL_954811, EPI_ISL_954813, EPI_ISL_1059944, EPI_ISL_1059956, EPI_ISL_1159167, EPI_ISL_1159215, EPI_ISL_1261681			
see above	HOSPITAL UNIVERSITARIO DE GUADALAJARA	Instituto de Salud Carlos III	A; A. Monzón; ALEJANDRO; F. Casas; I. GONZALEZ PRAETORIUS; I. González, A.; I. Jiménez; I.GONZALEZ PRAETORIUS; I.Gonzalez; Iglesias-Caballero; M. Camarero; M. Sandonís; P. Zaballos; S. Camarero; S. Cuesta; S. Iglesias-Caballero; S. Pozo; S. Sandonís; S. Varona; Sandonís; V. Camarero; V. Vázquez; V. Vázquez-Morón; Vázquez-Morón
EPI_ISL_1159131, EPI_ISL_1261510 to 1261515, EPI_ISL_1262087 to 1262088, EPI_ISL_1262090, EPI_ISL_1262096, EPI_ISL_1262099 to 1262100, EPI_ISL_1262102 to 1262103, EPI_ISL_1262105 to 1262106, EPI_ISL_1262108 to 1262109			
see above	HOSPITAL UNIVERSITARIO INSULAR DE GRAN CANARIA	Instituto de Salud Carlos III	A. Monzón; C; CRISTOBAL; F. Casas; I. Del Rosario, C.; I. Jiménez; I.DEL ROSARIO QUINTANA; I.Del Rosario; I.Del Rosario, C.; Iglesias-Caballero; M. Camarero; M. Sandonís; P. Zaballos; S. Camarero; S. Cuesta; S. Iglesias-Caballero; S. Pozo; S. Varona; Sandonís; V. Camarero; V. Vázquez-Morón; Vázquez-Morón
EPI_ISL_418251	HOSPITAL UNIVERSITARIO LA PAZ	Instituto de Salud Carlos III	A. Monzón; F. Casas; I. Jiménez; I. Romero P.; Iglesias-Caballero; M. Camarero; M. Cuesta; M. González-Esguevillas; M. Molinero Calamita; M. Zaballos; P. Jiménez; S. Juliá; S. Pozo; S. Varona
EPI_ISL_794659 to 794660	HOSPITAL UNIVERSITARIO SAN IGNACIO	Instituto Nacional de Salud - Dirección de Investigación en Salud Pública	Carlos Franco-Muñoz; Diego A. Álvarez-Díaz; Diego Andrés Prada; Gerardo Santamaría; Jonathan Reales; Julian Naizaque; Katherine Laiton-Donato; Magdalena Wiesner; Marcela Mercado-Reyes; Maria T. Herrera; Martha Lucia Ospina Martinez; Mauricio Pacheco-Montealegre; Paola Muñoz-Laiton; Sheryl Corchuelo
EPI_ISL_1159148 to 1159154, EPI_ISL_1159212, EPI_ISL_1167199, EPI_ISL_1261496 to 1261497, EPI_ISL_1262052 to 1262053, EPI_ISL_1262055 to 1262056, EPI_ISL_1262058 to 1262060, EPI_ISL_1262062 to 1262063, EPI_ISL_1262065			
see above	HOSPITAL UNIVERSITARIO VIRGEN DE LA ARRIXACA	Instituto de Salud Carlos III	A. Monzón; F. Casas; I. Jiménez; I.MORENO PARRADO; I.Moreno; I.Moreno, L.; Iglesias-Caballero; L; LAURA; M. Sandonís; P. Zaballos; S. Camarero; S. Cuesta; S. Iglesias-Caballero; S. Pozo; S. Varona; V. Camarero; V. Vázquez-Morón; Vázquez-Morón
EPI_ISL_418243 to 418244	HOSPITAL UNIVERSITARIO VIRGEN DE LAS NIEVES	Instituto de Salud Carlos III	A. Monzón; F. Casas; I. Jiménez; I. Sanbonmatsu S.; Iglesias-Caballero; M. Camarero; M. Cuesta; M. González-Esguevillas; M. Molinero Calamita; M. Zaballos; P. Jiménez; S. Juliá; S. Pozo; S. Varona
EPI_ISL_682265	HOSPITAL UPALA	Inciensa, Instituto Costarricense de Investigación y Enseñanza en Nutrición y Salud	Adriana Godinez & Melany Calderon; Claudio Soto-Garita; Estela Cordero; Francisco Duarte; Hebleen Porras
EPI_ISL_636975 to 636979	HP Pemba	KRISP, KZN Research Innovation and Sequencing Platform	Giandhari J; Ismael N; Nadia Sitoe; Nedio Mabunda; Paulo Arnaldo; Pillay S; Tegally H; Wilkinson E; de Oliveira T
EPI_ISL_430847	HS mikrobiologi virus	The Public Health Agency of Sweden	Anna Risberg; Anna-Malin Linde; Karin Tegmark-Wisell; Maria Lind Karlberg; Olov Svartstrom; Oskar Karlsson Lindsjo; Shaman Muradrasoli; Zhibing Yun
EPI_ISL_418009, EPI_ISL_421463, EPI_ISL_421489, EPI_ISL_421493 to 421494	HSE Ilha Terceira - Angra do Heroismo	Instituto Nacional de Saude (INSA)	Guiomar et al
EPI_ISL_412971	HUS Diagnostiikkakeskus, Hallinto	Department of Virology Faculty of Medicine, Medicum University of Helsinki	Hannimari Kallio-Kokko; Olli Vapalahti; Suvi Kuivanen; Teemu Smura
EPI_ISL_648151	Halmstad	The Public Health Agency of Sweden	Anna Risberg; Anna-Malin Linde; Karin Tegmark-Wisell; Maria Lind Karlberg; Mattias Haukland; Mia Brytting; Olov Svartstrom; Oskar Karlsson Lindsjo; Petra Edquist; Reza Advani; Sandra Broddesson
EPI_ISL_454436 to 454446, EPI_ISL_455902, EPI_ISL_469073 to 469074, EPI_ISL_475093 to 475094, EPI_ISL_475096 to 475098, EPI_ISL_475100, EPI_ISL_475114, EPI_ISL_475116 to 475117, EPI_ISL_475120 to 475121, EPI_ISL_475123, EPI_ISL_475125, EPI_ISL_475156 to 475159, EPI_ISL_475164, EPI_ISL_475548, EPI_ISL_475553, EPI_ISL_475556 to 475557, EPI_ISL_510831, EPI_ISL_510866, EPI_ISL_548247, EPI_ISL_548249, EPI_ISL_582803 to 582805, EPI_ISL_594148, EPI_ISL_615104 to 615107, EPI_ISL_623086, EPI_ISL_648148			
see above	Halmstad klinisk mikrobiologi	The Public Health Agency of Sweden	Anna Risberg; Anna-Malin Linde; Karin Tegmark-Wisell; Maria Lind Karlberg; Mattias Haukland; Mia Brytting; Olov Svartstrom; Oskar Karlsson Lindsjo; Petra Edquist; Reza Advani; Sandra Broddesson; Shamam Muradrasoli; Shaman Muradrasoli
EPI_ISL_710606 to 710607, EPI_ISL_766591	Halsomedicinskt Center	The Public Health Agency of Sweden	Department of Microbiology; The Public Health Agency of Sweden
EPI_ISL_660137 to 660139, EPI_ISL_660141 to 660142	Hamadi	National Health Laboratory Service (NHLS), Tygerberg	Bronwyn Kleinhans; Davis M-A; Draper C; Eduan Wilkindon; Gert van Zyl; Houriiyah Tegally; Hsiao M; Kayla Delaney; Siegfried N; Susan Engelbrecht; Tulio de Oliveira; Williamson C; Wolfgang Preiser
EPI_ISL_486884 to 486885	Hamedan University of Medical Sciences	Hamedan University of Medical Sciences	Ansari, N.; Azizi jalilian, F.; Jamehdor, S.; N. and Mazaheri, Z.; Nazari, A.; Saadat; Teimoori, A.; Zanjani, M.
EPI_ISL_407313	Hangzhou Center for Disease Control and Prevention	Hangzhou Center for Disease Control and Prevention	Haoqiu Wang; Hua Yu; Jun Li; Lingfeng Mao; Qingxin Kong; Shuchang Chen; Xin Qian; Xinfen Yu; Xuchu Wang; Zhou Sun
EPI_ISL_418510	Hangzhou Center for Disease Control and Prevention	Insepction Center of Hangzhou Center for Disease Control and Prevention	Li jun; Pan jingcao; Wang haoqiu; Yu hua; Yu xinfeng
EPI_ISL_418506	Hangzhou Center for Disease Control and Prevention	Inspection Center of Hanghzou Center for Disease Control and Prevention	Li jun; Pan jingcao; Wang haoqiu; Yu hua; Yu xinfeng
EPI_ISL_418441 to 418442, EPI_ISL_418502 to 418504, EPI_ISL_418507 to 418509, EPI_ISL_418511 to 418515	Hangzhou Center for Disease Control and Prevention	Inspection Center of Hangzhou Center for Disease Control and Prevention	Li jun; Pan jingcao; Wang haoqiu; Yu hua; Yu xinfeng
EPI_ISL_406970	Hangzhou Center for Disease and Control Microbiology Lab	Hangzhou Center for Disease and Control Microbiology Lab	Li Jun; Wang Haoqiu; Yu Hua; Yu Xinfeng
EPI_ISL_421221 to 421236, EPI_ISL_482575 to 482586	Hangzhou Center for Diseases Control and Prevention	Hangzhou Center for Diseases Control and Prevention	Haoqiu Wang; Hua Yu; Jun Li; Junfang Chen; Lingfeng Mao; Shuchang Chen; Xin Qian; Xinfen Yu; Xuchu Wang; Zhou Sun
EPI_ISL_766028, EPI_ISL_883157 to 883175, EPI_ISL_1268648, EPI_ISL_1268650 to 1268651, EPI_ISL_1268661 to 1268662, EPI_ISL_1268664 to 1268665, EPI_ISL_1268698, EPI_ISL_1268700, EPI_ISL_1268702 to 1268703, EPI_ISL_1268705, EPI_ISL_1268707, EPI_ISL_1268709, EPI_ISL_1268711, EPI_ISL_1268713,			

EPI_ISL_1268715, EPI_ISL_1268717, EPI_ISL_1268719, EPI_ISL_1268721, EPI_ISL_1268723, EPI_ISL_1268725, EPI_ISL_1268727, EPI_ISL_1268729, EPI_ISL_1268731, EPI_ISL_1268771, EPI_ISL_1268773, EPI_ISL_1268778, EPI_ISL_1268782, EPI_ISL_1268784, EPI_ISL_1268794, EPI_ISL_1268796, EPI_ISL_1268799, EPI_ISL_1268807, EPI_ISL_1268809, EPI_ISL_1268811, EPI_ISL_1268813, EPI_ISL_1268815, EPI_ISL_1268828, EPI_ISL_1268830 to 1268832, EPI_ISL_1268834, EPI_ISL_1268836 to 1268838, EPI_ISL_1268841, EPI_ISL_1268843, EPI_ISL_1268845 to 1268846, EPI_ISL_1268849 to 1268850, EPI_ISL_1268852 to 1268853, EPI_ISL_1268855 to 1268856, EPI_ISL_1268858 to 1268859, EPI_ISL_1268861 to 1268862, EPI_ISL_1268864 to 1268865, EPI_ISL_1268867 to 1268869, EPI_ISL_1268887, EPI_ISL_1268889 to 1268890, EPI_ISL_1268892			
see above	Hannover Medical School, Institute of Virology	Hannover Medical School, Institute of Virology	Jasper Götting; Lars Steinbrück
EPI_ISL_700411, EPI_ISL_700418, EPI_ISL_700457, EPI_ISL_700463, EPI_ISL_700477, EPI_ISL_700480, EPI_ISL_700494, EPI_ISL_700515 to 700516, EPI_ISL_700561 to 700562, EPI_ISL_700566, EPI_ISL_700573, EPI_ISL_700585, EPI_ISL_700593, EPI_ISL_1040694 to 1040695, EPI_ISL_1040717, EPI_ISL_1040730, EPI_ISL_1040764, EPI_ISL_1040797, EPI_ISL_1040809			
see above	Hanover Park CHC wc HPH	NHLS/UCT	Arash Iranzadeh; Bruna Galvao; Carolyn Williamson; Deelan Doolabh; Diana Hardie; Innocent Mudau; Kruger Marais; Lynn Tyers; Marvin Hsiao; Stephen Korsman
EPI_ISL_960165	Hanover Park CHC wc HPH	National Health Laboratory Service/UCT	Arash Iranzadeh; Bruna Galvao; Carolyn Williamson; Deelan Doolabh; Diana Hardie; Innocent Mudau; Kruger Marais; Lynn Tyers; Marvin Hsiao; Stephen Korsman
EPI_ISL_413025	Harborview Medical Center	UW Virology Lab	Alexander Greninger; Arun Nalla; Hong Xie; Keith Jerome; Pavitra Roychoudhury
EPI_ISL_413487	Harborview Medical Center	University of Washington Virology Lab	Alexander Greninger; Arun Nalla; Hong Xie; Keith Jerome; Pavitra Roychoudhury
EPI_ISL_635080 to 635084, EPI_ISL_635089 to 635091, EPI_ISL_635133, EPI_ISL_635184	Haukeland University Hospital, Department of Medical Microbiology	Norwegian Institute of Public Health, Department of Virology	Hilde Elshaug; Hilde Vollan; Kamilla Heddeland Instefjord; Karoline Bragstad; Kathrine Stene-Johansen; Marie Paulsen Madsen; Olav Hungnes; Rasmus Riis Kopperud
EPI_ISL_635128, EPI_ISL_668392, EPI_ISL_668405, EPI_ISL_759975, EPI_ISL_775276, EPI_ISL_775335 to 775336, EPI_ISL_775357, EPI_ISL_775407 to 775409, EPI_ISL_796652, EPI_ISL_860208, EPI_ISL_860211, EPI_ISL_860278, EPI_ISL_964951, EPI_ISL_1034310, EPI_ISL_1040153, EPI_ISL_1058047, EPI_ISL_1058049 to 1058053, EPI_ISL_1058055 to 1058058, EPI_ISL_1058060, EPI_ISL_1058062, EPI_ISL_1110512 to 1110513, EPI_ISL_1110536, EPI_ISL_1110541 to 1110542, EPI_ISL_1118289, EPI_ISL_1118327, EPI_ISL_1186177 to 1186178, EPI_ISL_1251530			
see above	Haukeland University Hospital, Dept. of Microbiology	Norwegian Institute of Public Health, Department of Virology	Atiya R Ali; Debech Nadia; Engebretsen Serina Beate; Engebretsen Serina Beate Atiya R Ali; Garcia Llorente Ignacio; Hilde Elshaug; Hilde Vollan; Ignacio Garcia Llorente; Jon Bråte; Kamilla Heddeland Instefjord; Karoline Bragstad; Kathrine Stene-Johansen; Marie Paulsen Madsen; Olav Hungnes; Pedersen Benedikte Nevjen; Rasmus Riis Kopperud; Serina B Engebretsen
EPI_ISL_450498	Health Board Laboratory of Communicable Diseases	Charite Universitatsmedizin Berlin, Institute of Virology	Barbara Muhlemann; Christian Drosten; Julia Schneider; Jörn Beheim-Schwarzbach; Liidia Dotsenko; Natalja Kuznetsova; Talitha Veith; Terry Jones; Victor M Corman
EPI_ISL_420065 to 420067	Health Board Laboratory of Communicable Diseases	Charite Universitatsmedizin Berlin, Institute of Virology	Barbara Mühlemann; Christian Drosten; Jorn Beheim-Schwarzbach; Julia Schneider; Liidia Dotsenko; Natalja Kuznetsova; Talitha Veith; Terry Jones; Victor M Corman
EPI_ISL_429256	Health Sciences Technology Park, Avicena, 8, 18016 Granada. Spain	Sequencing and Bioinformatics Service FISABIO-Public Health	Almudena Rojas; Joaquín Mendoza; Pablo Mendoza
EPI_ISL_541942	Health and Environmental Research Institute of Gwangju	Health & Environment Institute of Gwangju	Ji-eun Lee; Min Ji Kim
EPI_ISL_540993 to 541007	Health and Environmental Research Institute of Gwangju Metropolitan city	Health and Environmental Research Institute of Gwangju Metropolitan city	Ji-eun Lee; Min Ji Kim
EPI_ISL_634864 to 634865, EPI_ISL_1132743	HealthPartners Central Lab	Minnesota Department of Health, Public Health Laboratory	Alexandra Lorentz; Jacob Garfin; Matt Plumb; and Xiong Wang
EPI_ISL_1200519	HealthQuest Esoterics	New York City Public Health Laboratory	Jade Wang; et al.
EPI_ISL_796012 to 796026	Hebei Provincial Center for Disease Control and Prevention, Shijiazhuang, Hebei Province; National Institute for Viral Disease Control and Prevention, China CDC	Hebei Provincial Center for Disease Control and Prevention, Shijiazhuang, Hebei Province; National Institute for Viral Disease Control and Prevention, China CDC	George F. Gao; Nankun Liu; Qi Li; Shunxiang Qi; Wenbo Xu; Xiang Zhao; Yang Song
EPI_ISL_640018	Heidelberg Clinic wc HBC	NHLS/UCT	Arash Iranzadeh; Bruna Galvao; Carolyn Williamson; Deelan Doolabh; Diana Hardie; Innocent Mudau; Kruger Marais; Lynn Tyers; Marvin Hsiao; Stephen Korsman
EPI_ISL_640101, EPI_ISL_640103, EPI_ISL_640106, EPI_ISL_700412, EPI_ISL_700454, EPI_ISL_700459, EPI_ISL_700473, EPI_ISL_700499, EPI_ISL_700508, EPI_ISL_700522, EPI_ISL_700549, EPI_ISL_1040719			
see above	Heideveld CDC wc HVP	NHLS/UCT	Arash Iranzadeh; Bruna Galvao; Carolyn Williamson; Deelan Doolabh; Diana Hardie; Innocent Mudau; Kruger Marais; Lynn Tyers; Marvin Hsiao; Stephen Korsman
EPI_ISL_960164, EPI_ISL_960168 to 960170, EPI_ISL_960173	Heideveld CDC wc HVP	National Health Laboratory Service/UCT	Arash Iranzadeh; Bruna Galvao; Carolyn Williamson; Deelan Doolabh; Diana Hardie; Innocent Mudau; Kruger Marais; Lynn Tyers; Marvin Hsiao; Stephen Korsman
EPI_ISL_640082, EPI_ISL_700445, EPI_ISL_700545, EPI_ISL_700559, EPI_ISL_700574, EPI_ISL_700584, EPI_ISL_700595, EPI_ISL_700598, EPI_ISL_1040662, EPI_ISL_1040665, EPI_ISL_1040670, EPI_ISL_1040680, EPI_ISL_1040688, EPI_ISL_1040757, EPI_ISL_1040798, EPI_ISL_1040803, EPI_ISL_1040806, EPI_ISL_1040810			
see above	Heideveld Emergency Centre	NHLS/UCT	Arash Iranzadeh; Bruna Galvao; Carolyn Williamson; Deelan Doolabh; Diana Hardie; Houriiyah Tegally; Innocent Mudau; Kruger Marais; Lynn Tyers; Marvin Hsiao; Stephen Korsman
EPI_ISL_960124, EPI_ISL_960153, EPI_ISL_960162, EPI_ISL_960166	Heideveld Emergency Centre	National Health Laboratory Service/UCT	Arash Iranzadeh; Bruna Galvao; Carolyn Williamson; Deelan Doolabh; Diana Hardie; Innocent Mudau; Kruger Marais; Lynn Tyers; Marvin Hsiao; Stephen Korsman
EPI_ISL_1176311, EPI_ISL_1176313, EPI_ISL_1176316, EPI_ISL_1176318, EPI_ISL_1176320	Heilig Hart Hospital	Imelda Hospital	Dagmar Obbels; Dagmar obbels; Hanne Valgaeren; Johan Frans
EPI_ISL_1265163	Heilig Hart Hospital Lier	Imelda Hospital	Dagmar Obbels; Hanne Valgaeren; Johan Frans
EPI_ISL_940767	Heilig Hart Lier	UAntwerp, Laboratory of Medical Microbiology, Campus Drie Eiken S6.26, Universiteitsplein 1, 2610, Wilrijk, Belgium	Basil Britto Xavier; Christine Lammens; Herman Goossens; Jasmine Coppens; Marie Le Mercier; Veerle Matheeussen
EPI_ISL_961104	Heilig Hart Mol	UAntwerp, Laboratory of Medical Microbiology	Basil Britto Xavier; Christine Lammens; Herman Goossens; Jasmine Coppens; Marie Le Mercier; Veerle Matheeussen
EPI_ISL_1252474 to 1252477, EPI_ISL_1265166, EPI_ISL_1281693	Heilig hart Lier	Imelda hospital Bonheiden	Dagmar Obbels; Hanne Valgaeren; Johan Frans
EPI_ISL_1144020 to 1144021, EPI_ISL_1153463, EPI_ISL_1153508, EPI_ISL_1156095 to 1156098, EPI_ISL_1156101, EPI_ISL_1156115, EPI_ISL_1156122, EPI_ISL_1156152, EPI_ISL_1214847			
see above	Helios Universitätsklinikum Wuppertal: Universität Witten/Herdecke: Institut für Medizinische Labordiagnostik	Robert Koch Institute	
EPI_ISL_850689 to 850944	Helix / Illumina	Genomics and Discovery, Respiratory Viruses Branch, Division of Viral Diseases, Centers for Disease Control and Prevention	Alexandre Bolze; Ary Ascencio; Ben L. Rambo-Martin Eileen de Feo; Brad Sickler; Charlotte Rivera-Garcia; Christine Tran; Clinton R. Paden; David Becker; Dhwani Batra; Duncan MacCannell; Efren Sandoval; Elizabeth Cirulli; Eric Allen; Geraint Levan; James Lu; Jan Antico; Jason Nguyen; Jimmy Ramirez; Jingtao Liu; Kelly Schiabor Barrett; Kim Gietzen; Magnus Isaksson; Marc Laurent; Matthew Tolentino; Nicole L. Washington; Peter W. Cook; Phil Febbo; Ryan Cho; Shannon Wickline; Sherry Wang; Simon White; Summer Galloway; Suxiang Tong; Tyler Cassens; William Lee
EPI_ISL_1088585 to 1089433, EPI_ISL_1255398 to 1255414	Helix / Illumina	Respiratory Viruses Branch, Division of Viral Diseases, Centers for Disease Control and Prevention	Alexandre Bolze; Ary Ascencio; Ben L. Rambo-Martin; Brad Sickler; Charlotte Rivera-Garcia; Christine Tran; Clinton R. Paden; Dakota Howard; David Becker; Dhwani Batra; Duncan MacCannell; Efren Sandoval; Eileen de Feo; Elizabeth Cirulli; Eric Allen; Geraint Levan; James Lu; Jan Antico; Jason Nguyen; Jimmy Ramirez; Jingtao Liu; Kelly Schiabor Barrett; Kim Gietzen; Magnus Isaksson; Marc Laurent; Matthew Tolentino; Nicole L. Washington; Peter W. Cook; Phil Febbo; Ryan Cho; Shannon Wickline; Sherry Wang; Simon White; Summer Galloway; Suxiang Tong; Tyler Cassens; William Lee
EPI_ISL_1194731 to 1194865, EPI_ISL_1261895 to 1262038, EPI_ISL_1262041, EPI_ISL_1262045, EPI_ISL_1262048, EPI_ISL_1262051, EPI_ISL_1262054, EPI_ISL_1262057, EPI_ISL_1262061, EPI_ISL_1262064, EPI_ISL_1262067, EPI_ISL_1262070, EPI_ISL_1262073, EPI_ISL_1262076, EPI_ISL_1262080, EPI_ISL_1262083, EPI_ISL_1262086, EPI_ISL_1262089, EPI_ISL_1262092, EPI_ISL_1262095, EPI_ISL_1262098, EPI_ISL_1262101, EPI_ISL_1262104, EPI_ISL_1262107, EPI_ISL_1262110, EPI_ISL_1262113, EPI_ISL_1262116, EPI_ISL_1262119, EPI_ISL_1262122, EPI_ISL_1262125, EPI_ISL_1262127 to 1262454, EPI_ISL_1267502 to 1267589, EPI_ISL_1267592, EPI_ISL_1267595, EPI_ISL_1267598, EPI_ISL_1267601, EPI_ISL_1267603 to 1268193, EPI_ISL_1268196 to 1268197, EPI_ISL_1268199, EPI_ISL_1268201, EPI_ISL_1268203, EPI_ISL_1268206, EPI_ISL_1268208, EPI_ISL_1268210, EPI_ISL_1268212, EPI_ISL_1268214, EPI_ISL_1268216, EPI_ISL_1268218, EPI_ISL_1268220, EPI_ISL_1268222, EPI_ISL_1268225, EPI_ISL_1268227, EPI_ISL_1268229, EPI_ISL_1268231, EPI_ISL_1268233, EPI_ISL_1268235, EPI_ISL_1268237, EPI_ISL_1268239, EPI_ISL_1268241 to 1268242, EPI_ISL_1268245, EPI_ISL_1268247, EPI_ISL_1268249, EPI_ISL_1268251 to 1268252, EPI_ISL_1268254, EPI_ISL_1268256, EPI_ISL_1268258, EPI_ISL_1268260, EPI_ISL_1268262, EPI_ISL_1268264 to 1268647, EPI_ISL_1268649, EPI_ISL_1268652, EPI_ISL_1268654, EPI_ISL_1268657, EPI_ISL_1268660, EPI_ISL_1268663, EPI_ISL_1268666, EPI_ISL_1268669,			

EPI_ISL_1268672, EPI_ISL_1268675, EPI_ISL_1268678, EPI_ISL_1268681, EPI_ISL_1268684, EPI_ISL_1268687, EPI_ISL_1268690, EPI_ISL_1268693, EPI_ISL_1268696, EPI_ISL_1268699, EPI_ISL_1268701, EPI_ISL_1268704, EPI_ISL_1268706, EPI_ISL_1268708, EPI_ISL_1268710, EPI_ISL_1268712, EPI_ISL_1268714, EPI_ISL_1268716, EPI_ISL_1268718, EPI_ISL_1268720, EPI_ISL_1268722, EPI_ISL_1268724, EPI_ISL_1268726, EPI_ISL_1268728, EPI_ISL_1268730, EPI_ISL_1268732, EPI_ISL_1268734, EPI_ISL_1268736, EPI_ISL_1268738, EPI_ISL_1268740, EPI_ISL_1268742, EPI_ISL_1268744, EPI_ISL_1268746, EPI_ISL_1268748, EPI_ISL_1268751 to 1268752, EPI_ISL_1268754, EPI_ISL_1268756, EPI_ISL_1268758, EPI_ISL_1268760, EPI_ISL_1268762, EPI_ISL_1268764, EPI_ISL_1268766, EPI_ISL_1268768, EPI_ISL_1268770, EPI_ISL_1268772, EPI_ISL_1268774, EPI_ISL_1268777, EPI_ISL_1268779, EPI_ISL_1268781, EPI_ISL_1268783, EPI_ISL_1268785, EPI_ISL_1268787, EPI_ISL_1268789, EPI_ISL_1268791, EPI_ISL_1268793, EPI_ISL_1268795, EPI_ISL_1268798, EPI_ISL_1268800, EPI_ISL_1268802, EPI_ISL_1268804, EPI_ISL_1268806, EPI_ISL_1268808, EPI_ISL_1268810, EPI_ISL_1268812, EPI_ISL_1268814, EPI_ISL_1268816, EPI_ISL_1268818, EPI_ISL_1268820, EPI_ISL_1268822, EPI_ISL_1268824, EPI_ISL_1268826 to 1268827, EPI_ISL_1268829, EPI_ISL_1268833, EPI_ISL_1268835, EPI_ISL_1268839 to 1268840, EPI_ISL_1268842, EPI_ISL_1268844, EPI_ISL_1268847 to 1268848, EPI_ISL_1268851, EPI_ISL_1268854, EPI_ISL_1268857, EPI_ISL_1268860, EPI_ISL_1268863, EPI_ISL_1268866, EPI_ISL_1268870, EPI_ISL_1268873, EPI_ISL_1268876, EPI_ISL_1268879, EPI_ISL_1268882, EPI_ISL_1268885, EPI_ISL_1268888, EPI_ISL_1268891, EPI_ISL_1268894 to 1269017, EPI_ISL_1269019 to 1269313, EPI_ISL_1269316, EPI_ISL_1269320, EPI_ISL_1269323, EPI_ISL_1269326, EPI_ISL_1269329, EPI_ISL_1269332, EPI_ISL_1269335, EPI_ISL_1269339, EPI_ISL_1269342, EPI_ISL_1269345, EPI_ISL_1269349, EPI_ISL_1269352, EPI_ISL_1269355, EPI_ISL_1269358, EPI_ISL_1269361, EPI_ISL_1269364, EPI_ISL_1269368, EPI_ISL_1269371, EPI_ISL_1269374, EPI_ISL_1269377, EPI_ISL_1269380, EPI_ISL_1269383, EPI_ISL_1269386, EPI_ISL_1269389, EPI_ISL_1269392, EPI_ISL_1269395, EPI_ISL_1269399, EPI_ISL_1269402, EPI_ISL_1269405, EPI_ISL_1269408, EPI_ISL_1269412, EPI_ISL_1269415, EPI_ISL_1269418, EPI_ISL_1269422, EPI_ISL_1269425, EPI_ISL_1269428, EPI_ISL_1269431, EPI_ISL_1269434, EPI_ISL_1269437, EPI_ISL_1269441, EPI_ISL_1269444, EPI_ISL_1269447, EPI_ISL_1269450, EPI_ISL_1269453, EPI_ISL_1269457, EPI_ISL_1269460, EPI_ISL_1269463, EPI_ISL_1269466, EPI_ISL_1269470, EPI_ISL_1269473, EPI_ISL_1269476, EPI_ISL_1269479, EPI_ISL_1269482, EPI_ISL_1269485, EPI_ISL_1269488, EPI_ISL_1269491, EPI_ISL_1269495, EPI_ISL_1269498, EPI_ISL_1269503, EPI_ISL_1269507, EPI_ISL_1269509, EPI_ISL_1269513, EPI_ISL_1269516, EPI_ISL_1269519, EPI_ISL_1269522, EPI_ISL_1269525, EPI_ISL_1269528, EPI_ISL_1269532, EPI_ISL_1269534, EPI_ISL_1269538, EPI_ISL_1269541, EPI_ISL_1269544, EPI_ISL_1269547, EPI_ISL_1269551, EPI_ISL_1269554, EPI_ISL_1269557, EPI_ISL_1269560, EPI_ISL_1269564, EPI_ISL_1269567, EPI_ISL_1269570, EPI_ISL_1269573, EPI_ISL_1269576, EPI_ISL_1269579, EPI_ISL_1269582, EPI_ISL_1269585, EPI_ISL_1269588, EPI_ISL_1269591, EPI_ISL_1269593, EPI_ISL_1269596, EPI_ISL_1269599, EPI_ISL_1269603, EPI_ISL_1269606, EPI_ISL_1269609, EPI_ISL_1269611, EPI_ISL_1269615, EPI_ISL_1269618, EPI_ISL_1269621, EPI_ISL_1269624, EPI_ISL_1269627, EPI_ISL_1269630, EPI_ISL_1269633, EPI_ISL_1269636, EPI_ISL_1269639, EPI_ISL_1269642, EPI_ISL_1269646, EPI_ISL_1269648, EPI_ISL_1269651, EPI_ISL_1269654, EPI_ISL_1269657, EPI_ISL_1269660, EPI_ISL_1269663, EPI_ISL_1269666, EPI_ISL_1269670, EPI_ISL_1269673, EPI_ISL_1269676, EPI_ISL_1269679, EPI_ISL_1269682, EPI_ISL_1269685, EPI_ISL_1269688, EPI_ISL_1269691, EPI_ISL_1269694, EPI_ISL_1269697, EPI_ISL_1269700, EPI_ISL_1269704, EPI_ISL_1269707, EPI_ISL_1269710, EPI_ISL_1269713, EPI_ISL_1269716, EPI_ISL_1269718, EPI_ISL_1269722, EPI_ISL_1269724, EPI_ISL_1269727, EPI_ISL_1269730, EPI_ISL_1269733, EPI_ISL_1269736, EPI_ISL_1269739, EPI_ISL_1269742, EPI_ISL_1269745, EPI_ISL_1269749, EPI_ISL_1269752, EPI_ISL_1269755, EPI_ISL_1269759, EPI_ISL_1269762, EPI_ISL_1269764, EPI_ISL_1269767, EPI_ISL_1269770, EPI_ISL_1269774, EPI_ISL_1269777, EPI_ISL_1269779, EPI_ISL_1269782, EPI_ISL_1269786, EPI_ISL_1269789, EPI_ISL_1269792, EPI_ISL_1269795, EPI_ISL_1269798, EPI_ISL_1269801, EPI_ISL_1269804, EPI_ISL_1269808, EPI_ISL_1269811, EPI_ISL_1269814, EPI_ISL_1269817, EPI_ISL_1269820, EPI_ISL_1269823, EPI_ISL_1269826, EPI_ISL_1269829, EPI_ISL_1269832, EPI_ISL_1269836, EPI_ISL_1269839, EPI_ISL_1269842, EPI_ISL_1269846, EPI_ISL_1269849, EPI_ISL_1269852, EPI_ISL_1269855, EPI_ISL_1269858, EPI_ISL_1269861, EPI_ISL_1269864, EPI_ISL_1269867, EPI_ISL_1269870, EPI_ISL_1269873, EPI_ISL_1269876, EPI_ISL_1269879, EPI_ISL_1269882, EPI_ISL_1269885, EPI_ISL_1269889, EPI_ISL_1269892, EPI_ISL_1269894, EPI_ISL_1269897, EPI_ISL_1269900, EPI_ISL_1269903, EPI_ISL_1269907, EPI_ISL_1269910, EPI_ISL_1269913, EPI_ISL_1269916, EPI_ISL_1269919, EPI_ISL_1269922, EPI_ISL_1269925, EPI_ISL_1269928, EPI_ISL_1269931, EPI_ISL_1269934, EPI_ISL_1269936, EPI_ISL_1269940, EPI_ISL_1269943, EPI_ISL_1269946, EPI_ISL_1269948 to 1270201, EPI_ISL_1270203, EPI_ISL_1270205, EPI_ISL_1270207, EPI_ISL_1270209, EPI_ISL_1270211, EPI_ISL_1270213, EPI_ISL_1270215, EPI_ISL_1270217, EPI_ISL_1270219, EPI_ISL_1270221, EPI_ISL_1270223, EPI_ISL_1270225 to 1270226, EPI_ISL_1270228, EPI_ISL_1270230, EPI_ISL_1270232, EPI_ISL_1270234, EPI_ISL_1270236, EPI_ISL_1270238, EPI_ISL_1270240, EPI_ISL_1270242, EPI_ISL_1270244, EPI_ISL_1270246, EPI_ISL_1270248 to 1270249, EPI_ISL_1270251, EPI_ISL_1270253, EPI_ISL_1270255 to 1270786, EPI_ISL_1283377, EPI_ISL_1283379, EPI_ISL_1283382, EPI_ISL_1283384, EPI_ISL_1283387, EPI_ISL_1283389, EPI_ISL_1283392, EPI_ISL_1283394, EPI_ISL_1283397, EPI_ISL_1283399, EPI_ISL_1283402, EPI_ISL_1283404, EPI_ISL_1283407, EPI_ISL_1283409, EPI_ISL_1283412, EPI_ISL_1283414, EPI_ISL_1283417, EPI_ISL_1283419, EPI_ISL_1283422, EPI_ISL_1283425, EPI_ISL_1283427, EPI_ISL_1283430, EPI_ISL_1283433, EPI_ISL_1283435, EPI_ISL_1283438, EPI_ISL_1283440, EPI_ISL_1283443, EPI_ISL_1283445, EPI_ISL_1283448, EPI_ISL_1283450, EPI_ISL_1283453, EPI_ISL_1283456, EPI_ISL_1283458, EPI_ISL_1283460, EPI_ISL_1283463, EPI_ISL_1283465, EPI_ISL_1283468, EPI_ISL_1283470, EPI_ISL_1283473, EPI_ISL_1283476, EPI_ISL_1283478, EPI_ISL_1283481, EPI_ISL_1283484, EPI_ISL_1283486, EPI_ISL_1283489, EPI_ISL_1283492, EPI_ISL_1283494, EPI_ISL_1283497, EPI_ISL_1283499, EPI_ISL_1283502, EPI_ISL_1283504, EPI_ISL_1283507, EPI_ISL_1283509, EPI_ISL_1283512, EPI_ISL_1283515, EPI_ISL_1283517, EPI_ISL_1283520, EPI_ISL_1283522, EPI_ISL_1283524, EPI_ISL_1283527, EPI_ISL_1283529, EPI_ISL_1283531, EPI_ISL_1283534, EPI_ISL_1283537, EPI_ISL_1283540, EPI_ISL_1283542, EPI_ISL_1283544, EPI_ISL_1283547, EPI_ISL_1283549, EPI_ISL_1283552, EPI_ISL_1283554, EPI_ISL_1283557, EPI_ISL_1283559, EPI_ISL_1283562, EPI_ISL_1283564, EPI_ISL_1283567, EPI_ISL_1283570, EPI_ISL_1283572, EPI_ISL_1283575, EPI_ISL_1283577, EPI_ISL_1283580, EPI_ISL_1283582, EPI_ISL_1283585, EPI_ISL_1283587, EPI_ISL_1283590, EPI_ISL_1283592, EPI_ISL_1283595, EPI_ISL_1283598, EPI_ISL_1283600, EPI_ISL_1283602, EPI_ISL_1283605, EPI_ISL_1283607, EPI_ISL_1283610, EPI_ISL_1283612, EPI_ISL_1283615, EPI_ISL_1283617, EPI_ISL_1283620, EPI_ISL_1283622, EPI_ISL_1283625, EPI_ISL_1283627, EPI_ISL_1283629, EPI_ISL_1283631, EPI_ISL_1283634, EPI_ISL_1283636, EPI_ISL_1283639, EPI_ISL_1283642, EPI_ISL_1283644, EPI_ISL_1283647, EPI_ISL_1283650, EPI_ISL_1283652, EPI_ISL_1283655, EPI_ISL_1283658, EPI_ISL_1283660, EPI_ISL_1283662, EPI_ISL_1283664, EPI_ISL_1283667, EPI_ISL_1283670, EPI_ISL_1283672, EPI_ISL_1283674, EPI_ISL_1283677, EPI_ISL_1283679, EPI_ISL_1283682, EPI_ISL_1283685, EPI_ISL_1283687, EPI_ISL_1283689, EPI_ISL_1283692, EPI_ISL_1283695, EPI_ISL_1283697, EPI_ISL_1283699, EPI_ISL_1283702, EPI_ISL_1283704, EPI_ISL_1283707, EPI_ISL_1283709, EPI_ISL_1283712, EPI_ISL_1283714, EPI_ISL_1283717, EPI_ISL_1283719, EPI_ISL_1283722, EPI_ISL_1283724, EPI_ISL_1283727, EPI_ISL_1283730, EPI_ISL_1283732, EPI_ISL_1283735, EPI_ISL_1283737, EPI_ISL_1283740, EPI_ISL_1283742, EPI_ISL_1283745, EPI_ISL_1283747, EPI_ISL_1283750, EPI_ISL_1283752, EPI_ISL_1283754, EPI_ISL_1283756, EPI_ISL_1283759, EPI_ISL_1283762, EPI_ISL_1283764, EPI_ISL_1283767, EPI_ISL_1283771, EPI_ISL_1283773, EPI_ISL_1283776, EPI_ISL_1283778, EPI_ISL_1283781, EPI_ISL_1283783, EPI_ISL_1283786, EPI_ISL_1283788, EPI_ISL_1283791, EPI_ISL_1283794, EPI_ISL_1283796, EPI_ISL_1283799, EPI_ISL_1283801, EPI_ISL_1283804, EPI_ISL_1283806, EPI_ISL_1283808, EPI_ISL_1283811, EPI_ISL_1283813, EPI_ISL_1283816, EPI_ISL_1283819, EPI_ISL_1283821, EPI_ISL_1283823, EPI_ISL_1283826, EPI_ISL_1283828, EPI_ISL_1283831, EPI_ISL_1283833, EPI_ISL_1283835, EPI_ISL_1283838, EPI_ISL_1283840, EPI_ISL_1283843, EPI_ISL_1283845, EPI_ISL_1283848, EPI_ISL_1283851, EPI_ISL_1283853, EPI_ISL_1283856, EPI_ISL_1283859, EPI_ISL_1283862, EPI_ISL_1283864, EPI_ISL_1283867, EPI_ISL_1283869, EPI_ISL_1283872, EPI_ISL_1283875, EPI_ISL_1283877 to 1283879, EPI_ISL_1283882, EPI_ISL_1283884, EPI_ISL_1283886, EPI_ISL_1283889, EPI_ISL_1283891, EPI_ISL_1283893, EPI_ISL_1283896, EPI_ISL_1283898, EPI_ISL_1283901, EPI_ISL_1283904, EPI_ISL_1283906, EPI_ISL_1283909, EPI_ISL_1283911, EPI_ISL_1283914, EPI_ISL_1283916, EPI_ISL_1283919, EPI_ISL_1283921, EPI_ISL_1283926, EPI_ISL_1283930, EPI_ISL_1283935, EPI_ISL_1283940, EPI_ISL_1283943, EPI_ISL_1283946, EPI_ISL_1283948, EPI_ISL_1283952, EPI_ISL_1283956, EPI_ISL_1283959, EPI_ISL_1283962, EPI_ISL_1283965, EPI_ISL_1283968, EPI_ISL_1283971, EPI_ISL_1283975, EPI_ISL_1283977, EPI_ISL_1283981, EPI_ISL_1283985, EPI_ISL_1283990, EPI_ISL_1283993, EPI_ISL_1283997, EPI_ISL_1284001, EPI_ISL_1284006, EPI_ISL_1284010, EPI_ISL_1284013, EPI_ISL_1284016, EPI_ISL_1284019, EPI_ISL_1284023, EPI_ISL_1284028, EPI_ISL_1284032, EPI_ISL_1284036, EPI_ISL_1284039, EPI_ISL_1284043, EPI_ISL_1284047, EPI_ISL_1284051, EPI_ISL_1284056, EPI_ISL_1284060, EPI_ISL_1284065, EPI_ISL_1284070, EPI_ISL_1284075, EPI_ISL_1284079, EPI_ISL_1284082, EPI_ISL_1284085, EPI_ISL_1284088, EPI_ISL_1284091, EPI_ISL_1284094, EPI_ISL_1284097, EPI_ISL_1284100, EPI_ISL_1284103, EPI_ISL_1284107, EPI_ISL_1284110, EPI_ISL_1284113, EPI_ISL_1284116, EPI_ISL_1284120, EPI_ISL_1284123, EPI_ISL_1284127, EPI_ISL_1284131, EPI_ISL_1284134, EPI_ISL_1284138, EPI_ISL_1284141, EPI_ISL_1284144, EPI_ISL_1284148, EPI_ISL_1284151, EPI_ISL_1284154, EPI_ISL_1284158, EPI_ISL_1284161, EPI_ISL_1284166, EPI_ISL_1284171, EPI_ISL_1284175, EPI_ISL_1284179, EPI_ISL_1284183, EPI_ISL_1284185, EPI_ISL_1284189, EPI_ISL_1284192, EPI_ISL_1284195, EPI_ISL_1284199, EPI_ISL_1284202, EPI_ISL_1284205, EPI_ISL_1284209, EPI_ISL_1284213, EPI_ISL_1284217, EPI_ISL_1284220, EPI_ISL_1284224, EPI_ISL_1284227, EPI_ISL_1284231, EPI_ISL_1284233, EPI_ISL_1284236, EPI_ISL_1284239, EPI_ISL_1284243, EPI_ISL_1284246, EPI_ISL_1284249, EPI_ISL_1284253, EPI_ISL_1284256, EPI_ISL_1284260, EPI_ISL_1284264, EPI_ISL_1284267, EPI_ISL_1284271, EPI_ISL_1284275, EPI_ISL_1284278, EPI_ISL_1284282, EPI_ISL_1284285, EPI_ISL_1284289, EPI_ISL_1284291, EPI_ISL_1284295, EPI_ISL_1284298, EPI_ISL_1284302, EPI_ISL_1284306, EPI_ISL_1284309, EPI_ISL_1284312, EPI_ISL_1284316, EPI_ISL_1284320, EPI_ISL_1284323, EPI_ISL_1284326, EPI_ISL_1284329, EPI_ISL_1284333, EPI_ISL_1284336, EPI_ISL_1284339, EPI_ISL_1284343, EPI_ISL_1284346, EPI_ISL_1284349, EPI_ISL_1284351, EPI_ISL_1284354, EPI_ISL_1284356, EPI_ISL_1284359, EPI_ISL_1284361, EPI_ISL_1284363, EPI_ISL_1284366, EPI_ISL_1284368, EPI_ISL_1284370, EPI_ISL_1284372, EPI_ISL_1284375, EPI_ISL_1284377, EPI_ISL_1284380, EPI_ISL_1284382, EPI_ISL_1284385, EPI_ISL_1284387, EPI_ISL_1284389, EPI_ISL_1284392, EPI_ISL_1284394, EPI_ISL_1284397, EPI_ISL_1284400, EPI_ISL_1284402, EPI_ISL_1284405, EPI_ISL_1284407, EPI_ISL_1284409 to 1284410, EPI_ISL_1284412, EPI_ISL_1284419, EPI_ISL_1284422, EPI_ISL_1284424, EPI_ISL_1284426, EPI_ISL_1284429, EPI_ISL_1284432, EPI_ISL_1284434, EPI_ISL_1284437, EPI_ISL_1284439, EPI_ISL_1284442, EPI_ISL_1284444, EPI_ISL_1284447, EPI_ISL_1284449, EPI_ISL_1284451, EPI_ISL_1284454, EPI_ISL_1284456, EPI_ISL_1297851 to 1298177, EPI_ISL_1298179, EPI_ISL_1298182, EPI_ISL_1298185, EPI_ISL_1298187, EPI_ISL_1298190, EPI_ISL_1298193, EPI_ISL_1298195 to 1298225

see above	Helix/Illumina	Centers for Disease Control and Prevention Division of Viral Diseases, Pathogen Discovery	Alexandre Bolze; Ary Ascencio; Ben L. Rambo-Martin; Brad Sickler; Charlotte Rivera-Garcia; Christine Tran; Clinton R. Paden; Dakota Howard; David Becker; Dhwani Batra; Duncan MacCannell; Efren Sandoval; Eileen de Feo; Elizabeth Cirulli; Eric Allen; Geraint Levan; James Lu; Jan Antico; Jason Nguyen; Jimmy Ramirez; Jingtao Liu; Kelly Schiabor Barrett; Kim Gietzen; Magnus Isaksson; Marc Laurent; Matthew Tolentino; Nicole L. Washington; Peter W. Cook; Phil Febbo; Ryan Cho; Shannon Wickline; Sherry Wang; Simon White; Summer Galloway; Suxiang Tong; Tyler Cassens; William Lee
EPI_ISL_755590 to 755618, EPI_ISL_802599 to 802723, EPI_ISL_850517 to 850651, EPI_ISL_850952 to 851040, EPI_ISL_876625 to 876736	Helix/Illumina	Genomics and Discovery, Respiratory Viruses Branch, Division of Viral Diseases, Centers for Disease Control and Prevention	; Alexandre Bolze; Ary Ascencio; Ben L. Rambo-Martin; Ben L. Rambo-Martin Eileen de Feo; Brad Sickler; Charlotte Rivera-Garcia; Christine Tran; Clinton R. Paden; David Becker; Dhwani Batra; Duncan MacCannell; Efren Sandoval; Eileen de Feo; Elizabeth Cirulli; Eric Allen; Geraint Levan; James Lu; Jan Antico; Jason Nguyen; Jimmy Ramirez; Jingtao Liu; Kelly Schiabor Barrett; Kim Gietzen; Magnus Isaksson; Marc Laurent; Matthew Tolentino; Nicole L. Washington; Peter W. Cook; Phil Febbo; Ryan Cho; Shannon Wickline; Sherry Wang; Simon White; Summer Galloway; Suxiang Tong; Tyler Cassens; William Lee

EPI_ISL_966497 to 966512, EPI_ISL_966514 to 966532, EPI_ISL_966534 to 966548, EPI_ISL_966553 to 966564, EPI_ISL_966566 to 966590, EPI_ISL_966592 to 966604, EPI_ISL_966606 to 966622, EPI_ISL_966624 to 966645, EPI_ISL_966647 to 966662, EPI_ISL_966664 to 966681, EPI_ISL_966683 to 966706, EPI_ISL_966708 to 966755, EPI_ISL_966852 to 966939, EPI_ISL_966941 to 967522, EPI_ISL_967524, EPI_ISL_967531, EPI_ISL_967537, EPI_ISL_967547, EPI_ISL_967554, EPI_ISL_967567, EPI_ISL_967576, EPI_ISL_967583, EPI_ISL_967590, EPI_ISL_967599, EPI_ISL_967609, EPI_ISL_967617, EPI_ISL_967629, EPI_ISL_967642, EPI_ISL_967655, EPI_ISL_967662, EPI_ISL_967671, EPI_ISL_967678, EPI_ISL_967684, EPI_ISL_967693, EPI_ISL_967700, EPI_ISL_967710, EPI_ISL_967719, EPI_ISL_967726, EPI_ISL_967736, EPI_ISL_967748, EPI_ISL_967753, EPI_ISL_967761, EPI_ISL_967767, EPI_ISL_967772 to 967881, EPI_ISL_978555 to 978787, EPI_ISL_1016509 to 1016689, EPI_ISL_1016691 to 1016821, EPI_ISL_1016823 to 1016841, EPI_ISL_1036684 to 1036726, EPI_ISL_1081045 to 1081096, EPI_ISL_1110650 to 1110666, EPI_ISL_1133792 to 1134839, EPI_ISL_1255415

see above	Helix/Illumina	Respiratory Viruses Branch, Division of Viral Diseases, Centers for Disease Control and Prevention	; Alexandre Bolze; Ary Ascencio; Ben L. Rambo-Martin; Brad Sickler; Charlotte Rivera-Garcia; Christine Tran; Clinton R. Paden; Dakota Howard; David Becker; Dhwani Batra; Duncan MacCannell; Efren Sandoval; Eileen de Feo; Elizabeth Cirulli; Eric Allen; Geraint Levan; James Lu; Jan Antico; Jason Nguyen; Jimmy Ramirez; Jingtao Liu; Kelly Schiabor Barrett; Kim Gietzen; Magnus Isaksson; Marc Laurent; Matthew Tolentino; Nicole L. Washington; Peter W. Cook; Phil Febbo; Ryan Cho; Shannon Wickline; Sherry Wang; Simon White; Summer Galloway; Suxiang Tong; Tyler Cassens; William Lee

EPI_ISL_451310 to 451311, EPI_ISL_487370, EPI_ISL_487377, EPI_ISL_487379, EPI_ISL_487381, EPI_ISL_501231 to 501238, EPI_ISL_501241 to 501242, EPI_ISL_501246, EPI_ISL_501248 to 501251

see above	Hellenic Pasteur Institute, National Influenza Reference laboratory of Southern Greece & Unit of Bioinformatics and Applied Genomics	Hellenic Pasteur Institute, National Influenza Reference laboratory of Southern Greece & Unit of Bioinformatics and Applied Genomics	Andreas Mentis; Androniki Voulgari-Kokota; Antonios Kalliaropoulos; Aspasia Kontou; Athanasios Kossyvakis; Evangelidou Maria; Horefti Elina; Timokratis Karamitros; Vasiliki Pogka
EPI_ISL_501230	Hellenic Pasteur Institute, Public Health Laboratories	Hellenic Pasteur Institute, National Influenza Reference laboratory of Southern Greece & Unit of Bioinformatics and Applied Genomics	Andreas Mentis; Androniki Voulgari-Kokota; Antonios Kalliaropoulos; Aspasia Kontou; Athanasios Kossyvakis; Evangelidou Maria; Horefti Elina; Timokratis Karamitros; Vasiliki Pogka
EPI_ISL_430469	Hellenic Pasteur Institute, Public Health Laboratories	Hellenic Pasteur Institute, Public Health Laboratories, Unit of Bioinformatics and Applied Genomics	Andreas Mentis; Androniki Voulgari-Kokota; Antonios Kalliaropoulos; Aspasia Kontou; Athanasios Kossyvakis; Evangelidou Maria; Horefti Elina; Timokratis Karamitros; Vasiliki Pogka
EPI_ISL_1017712	Hemato Oncologos S.A	Instituto Nacional de Salud- Dirección de Investigación en Salud Pública	Carlos Franco-Muñoz; Diego A. Álvarez-Díaz; Diego Andrés Prada; Gerardo Santamaría; Hector Alejandro Ruiz-Moreno; Jhonnatan Reales-González; Julian Naizaque; Katherine Laiton-Donato; Magdalena Wiesner; Marcela Mercado-Reyes; Maria T. Herrera-Sepúlveda; Martha Lucia Ospina Martinez; Sheryll Corchuelo
EPI_ISL_428232 to 428236	Hematology Laboratory, Section of Molecular Diagnostics, University Clinical Centre, Medical University of Gdansk	Department of Virology, Faculty of Medicine, University of Helsinki, Helsinki, Finland	Aneta Szulc; Maciej Grzybek; Marlena Robakowska; Olli Vapalahti; Teemu Smura
EPI_ISL_1229508, EPI_ISL_1229528, EPI_ISL_1229533 to 1229534, EPI_ISL_1239033 to 1239046	Hematology Laboratory, Section of Molecular Diagnostics, University Clinical Centre, Medical University of Gdansk	1. Laboratory of Recombinant Vaccines 2. Department of Biology and Medical Genetics 3. Laboratory of Clinical Genetics	Adam Sodol; Aneta Szulc; Bartosz Wasag; Boguslaw Szewczyk; Ewa Milosz; Krystyna Bienkowska-Szewczyk; Krzysztof Lewandowski; Lukasz Rabalski; Maciej Kosinski; Marlena Robakowska
EPI_ISL_450525 to 450530, EPI_ISL_512296 to 512303	Hematology Laboratory, Section of Molecular Diagnostics, University Clinical Centre, Medical University of Gdansk	Department of Virology, Faculty of Medicine, University of Helsinki, Helsinki, Finland	Aneta Szulc; Ewa Miosz; Maciej Grzybek; Marlena Robakowska; Olli Vapalahti; Teemu Smura

EPI_ISL_451648 to 451654, EPI_ISL_906711 to 906712, EPI_ISL_906714 to 906719, EPI_ISL_906721 to 906726, EPI_ISL_906728 to 906729, EPI_ISL_906731 to 906735, EPI_ISL_906737 to 906739, EPI_ISL_906741 to 906742, EPI_ISL_906744 to 906754, EPI_ISL_906756, EPI_ISL_930784, EPI_ISL_1040939 to 1040986, EPI_ISL_1040994 to 1040996, EPI_ISL_1096155 to 1096166, EPI_ISL_1096170 to 1096195, EPI_ISL_1096204 to 1096209, EPI_ISL_1229400 to 1229414, EPI_ISL_1229507, EPI_ISL_1229509 to 1229527, EPI_ISL_1229529 to 1229532, EPI_ISL_1239047

see above	Hematology Laboratory, Section of Molecular Diagnostics, University Clinical Centre, Medical University of Gdansk	Laboratory of Recombinant Vaccines	Adam Sodol; Aneta Szulc; Anna Piotrowska-Mietelska; Boguslaw Szewczyk; Ewa Milosz; Izabela Szczygielska; Krystyna Bienkowska-Szewczyk; Krzysztof Lewandowski; Lukasz Rabalski; Maciej Grzybek; Maciej Kosinski; Marlena Robakowska
EPI_ISL_699658 to 700325, EPI_ISL_723045 to 723076	Hematopathology Laboratory, ACTREC, TMC	Hematopathology Laboratory, ACTREC, TMC	ACTREC; Hematopathology Laboratory
EPI_ISL_530169 to 530171, EPI_ISL_644942, EPI_ISL_683689, EPI_ISL_770793 to 770794, EPI_ISL_770797, EPI_ISL_873276	Hennepin County Medical Center	Minnesota Department of Health, Public Health Laboratory	Alexandra Lorentz; Jacob Garfin; Matt Plumb; and Xiong Wang
EPI_ISL_1303471	Henry Mayo Newhall Hospital	Los Angeles County PHL	P. Hemarajata et al.
EPI_ISL_700565, EPI_ISL_700571	Herbertsdale Sat Clinic wc HBD	NHLS/UCT	Arash Iranzadeh; Bruna Galvao; Carolyn Williamson; Deelan Doolabh; Diana Hardie; Innocent Mudau; Kruger Marais; Lynn Tyers; Marvin Hsiao; Stephen Korsman
EPI_ISL_470568 to 470588, EPI_ISL_470598 to 470614, EPI_ISL_470651 to 470655	Hermes Pardini	Bioinformatics Laboratory / LNCC	Alexandra Gerber; Amilcar Tanuri; Ana Paula Guimarães; CADDE-group; Carolina Voloch; Ester Cerdeira Sabino; Filipe Romero; Ingra Morales Claro; Jaqueline Goes de Jesus; Laboratorio Hermes Pardini; Laboratorio Simile; Luiz Gonzaga Paula de Almeida; Mariane Talon; Nuno Rodrigues Faria; Renato Santana Aguiar e Ana Tereza Vasconcelos; Ronaldo da Silva Francisco Junior; Terezinha Marta Pereira; working group UFMG; working group UFRJ; Átila Duque Rossi
EPI_ISL_1141141 to 1141152, EPI_ISL_1141612 to 1141626, EPI_ISL_1144803 to 1144816, EPI_ISL_1147578 to 1147592, EPI_ISL_1147594 to 1147606	Herz- und Diabeteszentrum NRW: Universitätsklinik der Ruhr-Universität Bochum --> Institut für Laboratoriums- und Transfusionsmedizin	Robert Koch Institute	
EPI_ISL_1213565 to 1213566	Hi-Precision Diagnostic Center (QC)	Philippine Genome Center	Alethea R. de Guzman; Anna Ong-Lim; Arianne A. Zamora; Asia Louisa U. Chong; Benedict A. Maralit; Candice Francheska B. Tambaoan; Carlo M. Lapid; Celia Carlos; Devon Ray Pacial; Edsel Maurice Salvaña; El King D. Morado; Eva Maria Cutiongco-de la Paz; Francis A. Tablizo; Irish Coleen A. Asin; Jaime C. Montoya; Jan Michael C. Yap; Jo-Hannah S. Llames; John Q. Wong; Joshua Gregor A. Dizon; Juan Antonio R. Magalang; Karol Sophia Agape R. Padilla; Kenneth M. Kim; Kris P. Punayan; Marc Edsel C. Ayes; Marc Jerrone R. Castro; Maria Rosario Singh-Vergeire and Cynthia P. Saloma; Maria Sofia L. Yangzon; Marissa Alejandria; Razel Nikka M. Hao; Rianna Patricia S. Cruz; Sheila Mae M. Araiza
EPI_ISL_1122457	Hi-Precision Diagnostics	Philippine Genome Center	Alethea R. de Guzman; Anna Ong-Lim; Arianne A. Zamora; Asia Louisa U. Chong; Benedict A. Maralit; Candice Francheska B. Tambaoan; Carlo M. Lapid; Celia Carlos; Devon Ray Pacial; Edsel Maurice Salvaña; El King D. Morado; Eva Maria Cutiongco-de la Paz; Francis A. Tablizo; Irish Coleen A. Asin; Jaime C. Montoya; Jan Michael C. Yap; Jo-Hannah S. Llames; John Q. Wong; Joshua Gregor A. Dizon; Juan Antonio R. Magalang; Karol Sophia Agape R. Padilla; Kenneth M. Kim; Kris P. Punayan; Marc Edsel C. Ayes; Marc Jerrone R. Castro; Maria Rosario Singh-Vergeire and Cynthia P. Saloma; Maria Sofia L. Yangzon; Marissa Alejandria; Razel Nikka M. Hao; Rianna Patricia S. Cruz; Sheila Mae M. Araiza
EPI_ISL_479903 to 479912	Himeji City Institute of Environment and Health	Pathogen Genomics Center, National Institute of Infectious Diseases	Hajime Kamiya; Kentaro Itokawa; Makoto Kuroda; Masanori Hashino; Motoi Suzuki; Rina Tanaka; Tsuyoshi Sekizuka
EPI_ISL_480178 to 480179	Hiroshima City Institute of Public Health	Pathogen Genomics Center, National Institute of Infectious Diseases	Hajime Kamiya; Kentaro Itokawa; Kota Noritsune; Makoto Kuroda; Masanori Hashino; Motoi Suzuki; Rina Tanaka; Tsuyoshi Sekizuka
EPI_ISL_745093 to 745094	Histopath	CIDM-PH, Westmead Hospital	CIDM-PH et al.
EPI_ISL_498774 to 498775, EPI_ISL_513318 to 513320, EPI_ISL_544960, EPI_ISL_544975, EPI_ISL_544987, EPI_ISL_545007, EPI_ISL_545034 to 545036, EPI_ISL_547600, EPI_ISL_591485 to 591486, EPI_ISL_593652, EPI_ISL_672574, EPI_ISL_740879 to 740880, EPI_ISL_740896, EPI_ISL_767872 to 767875, EPI_ISL_767925 to 767926, EPI_ISL_792031, EPI_ISL_806721, EPI_ISL_845796, EPI_ISL_872580			
see above	Histopath	NSW Health Pathology - Institute of Clinical Pathology and Medical Research; Westmead Hospital; University of Sydney	CIDM-PH et al.
EPI_ISL_1196013	Hlathikhulu Government Hospital	National Institute for Communicable Diseases of the National Health Laboratory Service	Amoako DG; Bhiman JN; Ismail A; Mahlangu B; Maphalala GP; Mohale T; Ntuli N; Scheepers C
EPI_ISL_770013	Hle - Asilos De Ancianos	Inciensa, Instituto Costarricense de Investigación y Enseñanza en Nutrición y Salud	Adriana Godínez; Claudio Soto-Garita; Estela Cordero; Francisco Duarte; Hebleen Porras; Melany Calderón & Mariel López
EPI_ISL_770006	Hle - Asociacion Hogar De Ancianos De Palmar Sur De Osa	Inciensa, Instituto Costarricense de Investigación y Enseñanza en Nutrición y Salud	Adriana Godínez; Claudio Soto-Garita; Estela Cordero; Francisco Duarte; Hebleen Porras; Melany Calderón & Mariel López
EPI_ISL_770024	Hle - Asociacion Hogar De Ancianos Santiago Crespo Calvo	Inciensa, Instituto Costarricense de Investigación y Enseñanza en Nutrición y Salud	Adriana Godínez; Claudio Soto-Garita; Estela Cordero; Francisco Duarte; Hebleen Porras; Melany Calderón & Mariel López
EPI_ISL_770031 to 770032	Hle-Asociacion De Atencion Integral Del Anciano San Cayetano	Inciensa, Instituto Costarricense de Investigación y Enseñanza en Nutrición y Salud	Adriana Godínez; Claudio Soto-Garita; Estela Cordero; Francisco Duarte; Hebleen Porras; Melany Calderón & Mariel López
EPI_ISL_1158549 to 1158577	Hokkaido Institute of Public Health	Hokkaido Institute of Public Health	Hiroki Yamaguchi; Kazuhiro Okubo; Kentaro Itokawa; Makoto Kuroda; Masanori Hashino; Rina Tanaka; Tetsuya Ikeda; Tsuyoshi Sekizuka; Yuta Ohno
EPI_ISL_479792 to 479795, EPI_ISL_479801, EPI_ISL_479812 to 479820	Hokkaido Institute of Public Health	Pathogen Genomics Center, National Institute of Infectious Diseases	Hajime Kamiya; Kentaro Itokawa; Makoto Kuroda; Masanori Hashino; Motoi Suzuki; Rika Komagome; Rina Tanaka; Tsuyoshi Sekizuka
EPI_ISL_730578 to 730581, EPI_ISL_1055256	Home Quarantine Taskforce	Hong Kong Department of Health	Chan Rickjason C.W.; Lam Edman T.K.; Mak Gannon C.K.; Tsang Dominic N.C.
EPI_ISL_1196003, EPI_ISL_1196011 to 1196012, EPI_ISL_1196014	Homecare	National Institute for Communicable Diseases of the National Health Laboratory Service	Amoako DG; Bhiman JN; Ismail A; Mahlangu B; Maphalala GP; Mohale T; Ntuli N; Scheepers C
EPI_ISL_444037	Hong Kong Adventist Hospital	Hong Kong Department of Health	Chan Rickjason C.W.; Cheng Peter K.C.; Lam Edman T.K.; Mak Gannon C.K.; Tsang Dominic N.C.
EPI_ISL_730582, EPI_ISL_1055202, EPI_ISL_1055210	Hong Kong Adventist Hospital - Tsuen Wan	Hong Kong Department of Health	Chan Rickjason C.W.; Lam Edman T.K.; Mak Gannon C.K.; Tsang Dominic N.C.
EPI_ISL_510090, EPI_ISL_523853, EPI_ISL_637027, EPI_ISL_1055232	Hong Kong Baptist Hospital	Hong Kong Department of Health	Chan Rickjason C.W.; Lam Edman T.K.; Mak Gannon C.K.; Tsang Dominic N.C.
EPI_ISL_412028, EPI_ISL_412030	Hong Kong Department of Health	School of Public Health, The University of Hon g Kong	Daniel K.W. Chu; Dominic N.C. Tsang; Leo L.M. Poon; Malik Peiris
EPI_ISL_414517, EPI_ISL_414519, EPI_ISL_414527 to 414528, EPI_ISL_414567, EPI_ISL_414569, EPI_ISL_414571, EPI_ISL_476801 to 476804, EPI_ISL_760031 to 760058			
see above	Hong Kong Department of Health	School of Public Health, The University of Hong Kong	Carrie Wan; Daisy Ng; Daniel Chu; Daniel K.W. Chu; Dominic N.C. Tsang; Gigi Liu; Haogao Gu; Leo L.M. Poon; Leo Poon; Malik Peiris; Pavithra Krishnan
EPI_ISL_412029	Hong Kong Department of Health	The University of Hong Kong	Daniel K.W. Chu; Dominic N.C. Tsang; Leo L.M. Poon; Malik Peiris
EPI_ISL_1055227	Hong Kong Police Force	Hong Kong Department of Health	Chan Rickjason C.W.; Lam Edman T.K.; Mak Gannon C.K.; Tsang Dominic N.C.
EPI_ISL_426380, EPI_ISL_730583 to 730584	Hong Kong Sanatorium & Hospital	Hong Kong Department of Health	Chan Rickjason C.W.; Cheng Peter K.C.; Lam Edman T.K.; Mak Gannon C.K.; Tsang Dominic N.C.
EPI_ISL_560581 to 560590, EPI_ISL_792056, EPI_ISL_846631 to 846636, EPI_ISL_846644, EPI_ISL_846649 to 846650, EPI_ISL_872198 to 872199, EPI_ISL_872206, EPI_ISL_872210 to 872211, EPI_ISL_872213 to 872220, EPI_ISL_872227 to 872231, EPI_ISL_872233, EPI_ISL_872236 to 872237, EPI_ISL_872249 to 872253, EPI_ISL_872255, EPI_ISL_872261 to 872263, EPI_ISL_872266 to 872270, EPI_ISL_872274 to 872278, EPI_ISL_872291 to 872305, EPI_ISL_872307 to 872308, EPI_ISL_872315 to 872319, EPI_ISL_872325, EPI_ISL_872339 to 872348, EPI_ISL_883972 to 883989, EPI_ISL_894219, EPI_ISL_912566, EPI_ISL_935551 to 935559, EPI_ISL_935561 to 935562, EPI_ISL_935565 to 935575, EPI_ISL_935577 to 935586, EPI_ISL_935639, EPI_ISL_940995, EPI_ISL_941000 to 941003, EPI_ISL_941016, EPI_ISL_941019, EPI_ISL_941021 to 941022, EPI_ISL_941027, EPI_ISL_953940 to 953951, EPI_ISL_953953, EPI_ISL_953955, EPI_ISL_953957 to 953962, EPI_ISL_953964 to 953967, EPI_ISL_953969 to 953985, EPI_ISL_953987, EPI_ISL_953989 to 953993, EPI_ISL_953995 to 953997, EPI_ISL_954009 to 954012, EPI_ISL_954014 to 954020, EPI_ISL_954032 to 954033, EPI_ISL_954047 to 954048, EPI_ISL_954071 to 954072, EPI_ISL_954076 to 954077, EPI_ISL_954081, EPI_ISL_954103 to 954104, EPI_ISL_954106 to 954107, EPI_ISL_954112 to 954113, EPI_ISL_954115, EPI_ISL_954120, EPI_ISL_954122, EPI_ISL_954126, EPI_ISL_955061, EPI_ISL_955064 to 955065, EPI_ISL_955074 to 955075, EPI_ISL_955077 to 955078, EPI_ISL_955081, EPI_ISL_955083 to 955085, EPI_ISL_955095 to 955100, EPI_ISL_955174 to 955177, EPI_ISL_964916, EPI_ISL_977664, EPI_ISL_995462 to 995467, EPI_ISL_995471, EPI_ISL_995474 to 995490, EPI_ISL_1001312 to 1001317, EPI_ISL_1001319 to 1001325, EPI_ISL_1001328, EPI_ISL_1001330, EPI_ISL_1001336 to 1001337, EPI_ISL_1001352, EPI_ISL_1001354,			

EPI_ISL_1001365 to 1001367, EPI_ISL_1001372 to 1001373, EPI_ISL_1001386, EPI_ISL_1001391 to 1001393, EPI_ISL_1001403 to 1001407, EPI_ISL_1001415, EPI_ISL_1001450 to 1001451, EPI_ISL_1001456, EPI_ISL_1060333 to 1060336, EPI_ISL_1060338 to 1060358, EPI_ISL_1060368, EPI_ISL_1060379 to 1060386, EPI_ISL_1060389 to 1060394, EPI_ISL_1060430 to 1060432, EPI_ISL_1060441 to 1060442, EPI_ISL_1060447 to 1060448, EPI_ISL_1060450 to 1060460, EPI_ISL_1060557 to 1060560, EPI_ISL_1060575, EPI_ISL_1096242, EPI_ISL_1096244 to 1096246, EPI_ISL_1096253 to 1096261, EPI_ISL_1096264 to 1096266, EPI_ISL_1096270 to 1096273, EPI_ISL_1096275 to 1096289, EPI_ISL_1096291 to 1096297, EPI_ISL_1096299 to 1096302, EPI_ISL_1096304 to 1096312, EPI_ISL_1096317 to 1096320, EPI_ISL_1110390 to 1110398, EPI_ISL_1110406 to 1110407, EPI_ISL_1110409 to 1110419, EPI_ISL_1110421, EPI_ISL_1110428, EPI_ISL_1110431, EPI_ISL_1110435 to 1110438, EPI_ISL_1110440 to 1110441, EPI_ISL_1110444 to 1110459, EPI_ISL_1110462, EPI_ISL_1110464 to 1110466, EPI_ISL_1110475 to 1110510, EPI_ISL_1110877 to 1110878, EPI_ISL_1110881 to 1110887, EPI_ISL_1110890 to 1110891, EPI_ISL_1110895 to 1110916, EPI_ISL_1110918 to 1110923, EPI_ISL_1110928 to 1110951, EPI_ISL_1110953 to 1110958, EPI_ISL_1110960 to 1110963, EPI_ISL_1110966 to 1110970, EPI_ISL_1110972 to 1110979, EPI_ISL_1110981 to 1111017, EPI_ISL_1111025 to 1111046, EPI_ISL_1111048, EPI_ISL_1111050 to 1111056, EPI_ISL_1111059, EPI_ISL_1111061, EPI_ISL_1118876 to 1118878, EPI_ISL_1118883, EPI_ISL_1118892 to 1118893, EPI_ISL_1118898 to 1118899, EPI_ISL_1118901, EPI_ISL_1166891 to 1166917, EPI_ISL_1166919 to 1167021, EPI_ISL_1168191 to 1168194, EPI_ISL_1168202, EPI_ISL_1168206 to 1168211, EPI_ISL_1168218 to 1168220, EPI_ISL_1201507 to 1201510, EPI_ISL_1201520, EPI_ISL_1201522 to 1201525, EPI_ISL_1201539 to 1201543, EPI_ISL_1219924 to 1219929, EPI_ISL_1219932 to 1219934, EPI_ISL_1219937, EPI_ISL_1219939 to 1219941, EPI_ISL_1219943 to 1219948, EPI_ISL_1219951 to 1219958, EPI_ISL_1233224 to 1233236, EPI_ISL_1233247 to 1233253, EPI_ISL_1239369 to 1239371, EPI_ISL_1259284 to 1259296, EPI_ISL_1259299 to 1259300, EPI_ISL_1259307, EPI_ISL_1259310, EPI_ISL_1262718 to 1262746, EPI_ISL_1262751 to 1262759, EPI_ISL_1262815 to 1262818, EPI_ISL_1262898 to 1262907, EPI_ISL_1262914, EPI_ISL_1262917 to 1262933, EPI_ISL_1262981 to 1262989, EPI_ISL_1301804 to 1301815, EPI_ISL_1301831, EPI_ISL_1301838 to 1301858, EPI_ISL_1301882, EPI_ISL_1301890 to 1301894, EPI_ISL_1301896 to 1301898, EPI_ISL_1301921 to 1301922, EPI_ISL_1301930 to 1301937			
see above	Hopital	National Reference Center for Viruses of Respiratory Infections, Institut Pasteur, Paris	; Amade (W); Andre-Garnier Elisabeth; Angela Brisebarre; Bernardi Franck; Bigaillon Christine; Billy Pierre Alain; Bisiau H; Blazejewski C.; Bour Jean Baptiste; Bressollette (C) CéLine; Bret Laurent; Breuil J.; Brichler SéGolèNe; Brichler Ségolène; Cady Anne; Cady Anne; Camille Capel; Capron Nicolas; Cariou Marie-Estelle; Cartier Riviere B; Castelain Sandrine; Centre Hospitalier De Manosque; Combe Patrice; Coste-Burel Marianne; Cron Sophie; Delamare Catherine; Delepine Alain; Descamps Dominique; Desroches Marine; Djamdjian Laura; Ducancelle Alexandra; Ducancelle Alexandra; Dufour Mj; Durivault JéRôMe; Eloy Clarence; Engelmann Ilka; Etienne Simon-Lorière; Fabiana Gambaro; Farfour Eric; Febreau Christine; Fiette; Florin CéCile; Foissaud Vincent; Fougeaud Jacques; Gaschet Anne; Gastli Nabil; Goudeau Alain; Goudeau Alain; Griscelli Franck; Gros Isabelle; Guiard Carine; Guigon AuréLie; Guillaume CléMence; Guillotel Eleha; Guinard JéRome; Guinoiseau Thibault; Hermann CéCile; Hochart A; Irimia Alix; Joly Isabelle; Labourdette Isabelle; Lagathu GisèLe; Laplanche Sophie; Larreche; Ledru Sylvie; LefeuvreCaroline; Lepiller Quentin; Leruez-Ville Marianne; Lesimple BéAtrice; Louis Mathilde; Louise Lefrançois; Louvetl; Luizy Nelly; Maneglie Benjamin; Marichal A.; Marion Barbet; Marque Juillet StéPhanie; Martres Pascale; Marty Quinternet Solene; Maud Vanpeene; Merens Audrey; Morvan Odile; Morvan Pauline; Méline Bizard; Patoz Pierre; Patricia Stoessel; Pham StéPhanie; Philippi Jean-FrançOis; Pilorge LéA; Ramanantsoa CéLine; Ramirez José-Manuel; Raulin Olivia; Riverain Emeline; Serazin V; Smati-Lafarge Mounira; Stoessel-Thouvenin Patricia; Sylvie Behillil; Sylvie van der Werf; Thellier; Thibault Vincent; Vachee Anne; Van Agt S.; Vincent Enouf; Woercel Isabelle
EPI_ISL_912559, EPI_ISL_912598, EPI_ISL_913607	Hopital Avicenne Laboratoire central de bactériologie virologie hygiène	National Reference Center for Viruses of Respiratory Infections, Institut Pasteur, Paris	Angela Brisebarre; Brichler Ségolène; Camille Capel; Etienne Simon-Lorière; Marion Barbet; Maud Vanpeene; Méline Bizard; Sylvie Behillil; Sylvie van der Werf; Vincent Enouf
EPI_ISL_909666, EPI_ISL_909682 to 909683	Hopital Avicenne. Laboratoire central de bactériologie virologie hygiène	National Reference Center for Viruses of Respiratory Infections, Institut Pasteur, Paris	Angela Brisebarre; Brichler Ségolène; Camille Capel; Etienne Simon-Lorière; Marion Barbet; Maud Vanpeene; Méline Bizard; Sylvie Behillil; Sylvie van der Werf; Vincent Enouf
EPI_ISL_1035958	Hopital CH Jeanne de Navarre Laboratoire de Biologie	National Reference Center for Viruses of Respiratory Infections, Institut Pasteur, Paris	Angela Brisebarre; Camille Capel; Etienne Simon-Lorière; Marion Barbet; Maud Vanpeene; Méline Bizard; Sylvie Behillil; Sylvie van der Werf; Thellier; Vincent Enouf
EPI_ISL_1036398 to 1036402	Hopital CH René Dubos - Laboratoire de Biologie médicale	National Reference Center for Viruses of Respiratory Infections, Institut Pasteur, Paris	Angela Brisebarre; Blanchard GenevièVe; Camille Capel; Etienne Simon-Lorière; Marion Barbet; Maud Vanpeene; Méline Bizard; Sylvie Behillil; Sylvie van der Werf; Vincent Enouf
EPI_ISL_1035904 to 1035916, EPI_ISL_1035924, EPI_ISL_1035927	Hopital CH de Troyes	National Reference Center for Viruses of Respiratory Infections, Institut Pasteur, Paris	Angela Brisebarre; Camille Capel; Etienne Simon-Lorière; Marion Barbet; Maud Vanpeene; Méline Bizard; Stoessel-Thouvenin Patricia; Sylvie Behillil; Sylvie van der Werf; Vincent Enouf
EPI_ISL_1035925 to 1035926, EPI_ISL_1035929, EPI_ISL_1035952 to 1035957	Hopital CHRU Tours	National Reference Center for Viruses of Respiratory Infections, Institut Pasteur, Paris	Angela Brisebarre; Camille Capel; Etienne Simon-Lorière; Guinoiseau Thibault; Marion Barbet; Maud Vanpeene; Méline Bizard; Sylvie Behillil; Sylvie van der Werf; Vincent Enouf
EPI_ISL_1035930	Hopital Centre Hospitalier d'Arpajon	National Reference Center for Viruses of Respiratory Infections, Institut Pasteur, Paris	Angela Brisebarre; Barboux; Camille Capel; Etienne Simon-Lorière; Marion Barbet; Maud Vanpeene; Méline Bizard; Sylvie Behillil; Sylvie van der Werf; Vincent Enouf
EPI_ISL_1036407	Hopital HIA PERCY	National Reference Center for Viruses of Respiratory Infections, Institut Pasteur, Paris	Angela Brisebarre; Camille Capel; Etienne Simon-Lorière; Foissaud Vincent; Marion Barbet; Maud Vanpeene; Méline Bizard; Sylvie Behillil; Sylvie van der Werf; Vincent Enouf
EPI_ISL_1036426	Hopital Laboratoire CH Laon	National Reference Center for Viruses of Respiratory Infections, Institut Pasteur, Paris	Angela Brisebarre; Camille Capel; Etienne Simon-Lorière; Marion Barbet; Maud Vanpeene; Méline Bizard; Ravaux Jean Jacques; Sylvie Behillil; Sylvie van der Werf; Vincent Enouf
EPI_ISL_1036427 to 1036432	Hopital Laboratoire de Virologie	National Reference Center for Viruses of Respiratory Infections, Institut Pasteur, Paris	Angela Brisebarre; Camille Capel; Etienne Simon-Lorière; Griscelli Franck; Marion Barbet; Maud Vanpeene; Méline Bizard; Sylvie Behillil; Sylvie van der Werf; Vincent Enouf
EPI_ISL_418427	Hopital Privé de l'Est Lyonnais	CNR Virus des Infections Respiratoires - France SUD	Alexandre Gaymard; Antonin Bal; Bruno Lina; Carine Moustaud; Florence Morfin-Sherpa; Gregory Destras; Gwendolyne Burfin; Laurence Josset; Martine Valette; Maude Bouscambert-Duchamp; Raphaelle Lamy; Solenne Brun
EPI_ISL_1058581	Hopital Sainte Therese Bastogne	GIGA Medical Genomics	Bouchra Boujemla; Cécile Meex; Keith Durkin; Maria Artesi; Marie-Pierre Hayette; Nathalie Renotte; Pierrette Melin; Raphaël Boreux; Sébastien Bontems; Vincent Bours
EPI_ISL_418229	Hopital franco britannique - Laboratoire	National Reference Center for Viruses of Respiratory Infections, Institut Pasteur, Paris	Angela Brisebarre; Etienne Simon-Lorière; Flora Donati; Marianne Asso Bonnet; Marion Barbet; Maud Vanpeene; Mélanie Albert; Méline Bizard; Sylvie Behillil; Sylvie van der Werf; Vincent Enouf
EPI_ISL_416501	Hopital franco britannique - Service des Urgences	National Reference Center for Viruses of Respiratory Infections, Institut Pasteur, Paris	Angela Brisebarre; Etienne Simon-Lorière; Flora Donati; Marion Barbet; Maud Vanpeene; Méline Bizard; Mélnie Albert; Sylvie Behillil; Sylvie van der Werf; Vincent Enouf
EPI_ISL_434666	Hornefors Halsocentral	The Public Health Agency of Sweden	Anna Risberg; Anna-Malin Linde; Camilla Eiback; Karin Tegmark-Wisell; Maria Lind Karlberg; Mia Brytting; Olov Svartstrom; Oskar Karlsson Lindsjo; Theresa Enkirch
EPI_ISL_700458, EPI_ISL_700524, EPI_ISL_700558	Hornlee Clinic wc HLC	NHLS/UCT	Arash Iranzadeh; Bruna Galvao; Carolyn Williamson; Deelan Doolabh; Diana Hardie; Houriiyah Tegally; Innocent Mudau; Kruger Marais; Lynn Tyers; Marvin Hsiao; Stephen Korsman
EPI_ISL_696457, EPI_ISL_696479, EPI_ISL_696483, EPI_ISL_696486, EPI_ISL_696502	Hornlee Clinic wc HLC & NHLS/UCT	KRISP, KZN Research Innovation and Sequencing Platform	Arash Iranzadeh; Bruna Galvao; Carolyn Williamson; Deelan Doolabh; Diana Hardie; Emanuel James San; Houriiyah Tegally; Innocent Mudau; Jennifer Giandhari; Kruger Marais; Lynn Tyers; Marvin Hsiao; Stephen Korsman; Sureshnee Pillay; Tulio de Oliveira
EPI_ISL_471562, EPI_ISL_471581 to 471582, EPI_ISL_515564, EPI_ISL_523977	Hosp. Municipal Prof. Dr. Alípio Corrêa Netto	Instituto Adolfo Lutz, Interdiciplinary Procedures Center, Strategic Laboratory	Claudia Regina Gonçalves; Claudio Tavares Sacchi; Erica Valessa Ramos Gomes
EPI_ISL_560598 to 560636, EPI_ISL_560643 to 560646, EPI_ISL_660323, EPI_ISL_693758 to 693764, EPI_ISL_718226 to 718249, EPI_ISL_832130 to 832166, EPI_ISL_832169 to 832180, EPI_ISL_832197, EPI_ISL_832201 to 832202, EPI_ISL_855353 to 855356, EPI_ISL_855358 to 855380, EPI_ISL_855382 to 855386, EPI_ISL_855388, EPI_ISL_888826, EPI_ISL_890355 to 890359, EPI_ISL_890889 to 890894			
see above	Hospital	National Reference Center for Viruses of Respiratory Infections, Institut Pasteur, Paris	Alexandra Ducancelle; Angela Brisebarre; Aurélie Guigon; Camille Capel; Clémence Guillaume; Combe Patrice; Ducancelle Alexandra; Esther Gyde; Etienne Simon-Lorière; Fabiana Gambaro; Fourgeaud Jacques; Gisèle Lagathu; Griscelli Franck; Guigon AuréLie; Laurent Andreoletti; Laurent Roudiere; Léa Pilorge; Marion Barbet; Maud Vanpeene; Méline Bizard; Patricia Stoessel-Thouvenin; Pilorge Léa; Sylvie Behillil; Sylvie van der Werf; Vincent Enouf
EPI_ISL_693229	Hospital 8 de Maio	Instituto Adolfo Lutz, Interdiciplinary Procedures Center, Strategic Laboratory	Claudia Regina Gonçalves; Claudio Tavares Sacchi; Erica Valessa Ramos Gomes; Karoline Rodrigues Campos
EPI_ISL_434366 to 434386, EPI_ISL_450724 to 450737	Hospital AZ Rivierenland	Institute of Tropical Medicine	; Colin Anthony; Philippe Selhorst
EPI_ISL_1196864	Hospital Alor Gajah	Institute for Medical Research, Infectious Disease Research Centre, National Institutes of Health, Ministry of Health Malaysia	Kamel K; Mohd Zawawi Z; Ramly N; Robert F; Suppiah J; Thayan R
EPI_ISL_1219021	Hospital Aviccena	Instituto Adolfo Lutz, Interdiciplinary Procedures Center, Strategic Laboratory	Caio Vinicius Dias Lopes; Claudia Regina Gonçalves; Claudio Tavares Sacchi; Erica Valessa Ramos Gomes; Karoline Rodrigues Campos

EPI_ISL_906080 to 906081	Hospital Beneficiencia Portuguesa	Instituto Adolfo Lutz, Interdiciplinary Procedures Center, Strategic Laboratory	Claudia Regina Gonçalves; Claudio Tavares Sacchi; Erica Valessa Ramos Gomes; Karoline Rodrigues Campos
EPI_ISL_471554	Hospital Bosque da Saúde	Instituto Adolfo Lutz, Interdiciplinary Procedures Center, Strategic Laboratory	Claudia Regina Gonçalves; Claudio Tavares Sacchi; Erica Valessa Ramos Gomes
EPI_ISL_491439	Hospital Calderon Guardia	Inciensa, Instituto Costarricense de Investigación y Enseñanza en Nutrición y Salud	Adriana Godinez & Melany Calderon; Claudio Soto-Garita; Estela Cordero; Francisco Duarte; Hebleen Brenes
EPI_ISL_539559 to 539560	Hospital Campo Arañuelo	Instituto de Salud Carlos III	A. Monzón; F. Casas; G. Rodríguez; I; I. Jiménez; Iglesias-Caballero; J. López; M. Camarero; M. Cuesta; M. González-Esguevillas; M. Molinero Calamita; M. Zaballos; P. Jiménez; S. Juliá; S. Pozo; S. Varona
EPI_ISL_906078	Hospital Carlos Chagas	Instituto Adolfo Lutz, Interdiciplinary Procedures Center, Strategic Laboratory	Claudia Regina Gonçalves; Claudio Tavares Sacchi; Erica Valessa Ramos Gomes; Karoline Rodrigues Campos
EPI_ISL_940626 to 940627	Hospital Central Sao Caetano do Sul	Instituto Adolfo Lutz, Interdiciplinary Procedures Center, Strategic Laboratory	Claudia Regina Gonçalves; Claudio Tavares Sacchi; Erica Valessa Ramos Gomes; Karoline Rodrigues Campos
EPI_ISL_574580	Hospital Cidade Tiradentes Carmen Prudente	Instituto Adolfo Lutz, Interdiciplinary Procedures Center, Strategic Laboratory	Claudia Regina Gonçalves; Claudio Tavares Sacchi; Erica Valessa Ramos Gomes; Karoline Rodrigues Campos
EPI_ISL_527743 to 527744	Hospital Cima	Inciensa, Instituto Costarricense de Investigación y Enseñanza en Nutrición y Salud	Adriana Godinez & Melany Calderon; Claudio Soto-Garita; Estela Cordero; Francisco Duarte; Hebleen Porras
EPI_ISL_462479	Hospital Clinic	Instituto de Salud Carlos III	A. Monzón; F. Casas; I; I. Jiménez; Iglesias-Caballero; M. Camarero; M. Cuesta; M. González-Esguevillas; M. Molinero Calamita; M. Zaballos; M.A Marcos; P. Jiménez; S. Juliá; S. Pozo; S. Varona
EPI_ISL_491441 to 491442, EPI_ISL_491455	Hospital Clinica Biblica	Inciensa, Instituto Costarricense de Investigación y Enseñanza en Nutrición y Salud	Adriana Godinez & Melany Calderon; Claudio Soto-Garita; Estela Cordero; Francisco Duarte; Hebleen Brenes
EPI_ISL_539533 to 539556, EPI_ISL_862573 to 862588, EPI_ISL_862627 to 862636	Hospital Clínic	Instituto de Salud Carlos III	A. Monzón; F. Casas; I; I. Jiménez; I. Marcos; Iglesias-Caballero; M. Pozo; M. Camarero; M. Cuesta; M. González-Esguevillas; M. Molinero Calamita; M. Zaballos; M.A Marcos; M.A.; M.Camarero; P. Jiménez; S. Juliá; S. Molinero Calamita; S. Pozo; S. Varona
EPI_ISL_802726 to 802749	Hospital Clínic de Barcelona	Instituto de Salud Carlos III	A. Monzón; F. Casas; I; I. Jiménez; Iglesias-Caballero; M. Camarero; M. Cuesta; M. González-Esguevillas; M. Molinero Calamita; M. Zaballos; M.A Marcos.; P. Jiménez; S. Juliá; S. Pozo; S. Varona
EPI_ISL_480315 to 480320	Hospital Clínica Bíblica	Charité Virology-University of Costa Rica	Andres Moreira-Soto; Eugenia Corrales-Aguilar; Ignacio Postigo-Hidalgo; Jan Felix Drexler; Karla Sofía Gutiérrez
EPI_ISL_691700 to 691728, EPI_ISL_862548, EPI_ISL_862569, EPI_ISL_871985 to 871990, EPI_ISL_871992 to 871993	Hospital Clínico San Carlos	Instituto de Salud Carlos III	A. Monzón; F. Casas; I. Jiménez; I. Rodríguez, I.; Iglesias-Caballero; M. Camarero; M. Cuesta; M. González-Esguevillas; M. Pozo; M. Zaballos; P. Jiménez; S. Juliá; S. Molinero Calamita; S. Varona
EPI_ISL_539500 to 539502, EPI_ISL_539509 to 539512, EPI_ISL_578190 to 578191	Hospital Clínico Universitario Lozano Blesa	Instituto de Salud Carlos III	A. Monzón; F. Casas; I; I. Jiménez; Iglesias-Caballero; M. Camarero; M. Cuesta; M. González-Esguevillas; M. Molinero Calamita; M. Zaballos; P. Jiménez; R. Benito; S. Juliá; S. Pozo; S. Varona
EPI_ISL_671794 to 671804, EPI_ISL_683595 to 683597, EPI_ISL_1007859, EPI_ISL_1007861 to 1007892, EPI_ISL_1225136 to 1225154	Hospital Clínico Universitario Lozano Blesa de Zaragoza (España)	SeqCOVID-SPAIN consortium/IBV(CSIC)	Jessica Bueno and SeqCOVID-SPAIN consortium; Rafael Benito; Sonia Algarate
EPI_ISL_1194516 to 1194517, EPI_ISL_1194519 to 1194520, EPI_ISL_1194522 to 1194523, EPI_ISL_1194525 to 1194526, EPI_ISL_1194528 to 1194537, EPI_ISL_1194539, EPI_ISL_1194541 to 1194542, EPI_ISL_1194544 to 1194545, EPI_ISL_1194547, EPI_ISL_1194549 to 1194558, EPI_ISL_1194560, EPI_ISL_1194562 to 1194563, EPI_ISL_1194565 to 1194566, EPI_ISL_1194568 to 1194569, EPI_ISL_1194571 to 1194572, EPI_ISL_1194574, EPI_ISL_1194576 to 1194577			
see above	Hospital Clínico Universitario Virgen de la Arrixaca (Murcia)	SeqCOVID-SPAIN consortium/IBV(CSIC)	Antonio Moreno Docón; Genoveva Yagüe and SeqCOVID-SPAIN consortium; Manuel Segovia Hernández
EPI_ISL_467262 to 467297, EPI_ISL_500162, EPI_ISL_500167, EPI_ISL_500209, EPI_ISL_500219, EPI_ISL_500221, EPI_ISL_510268 to 510304			
see above	Hospital Clínico Universitario de Santiago de Compostela	SeqCOVID-SPAIN consortium/IBV(CSIC)	Amparo Coira Nieto; Antonio Aguilera Guirao; Gema Barbeito Castiñeiras; José Javier Costa Alcalde; Mª Luisa Pérez del Molino Bernal; Rocio Trastoy Pena and SeqCOVID-SPAIN consortium
EPI_ISL_541037 to 541066	Hospital Clínico Universitario de Santiago de Compostela	SeqCOVID-SPAIN consortium/Institute of Biomedicine of Valencia, IBV-CSIC	Amparo Coira Nieto; Antonio Aguilera Guirao; Gema Barbeito Castiñeiras; José Javier Costa Alcalde; Mª Luisa Pérez del Molino Bernal; Rocio Trastoy Pena and SeqCOVID-SPAIN consortium
EPI_ISL_1007680 to 1007697	Hospital Clínico Universitario de Valladolid-Hospital Universitario Río Hortega	SeqCOVID-SPAIN consortium/IBV(CSIC)	Antonio Orduña-Domingo; Carmen Aldea-Mansilla; Jose María Eiros and SeqCOVID-SPAIN consortium; Juan Luis Muñoz-Bellido; M. Isabel Fernandez-Natal; Marta Domínguez-Gil; Marta Hernández; Mª Fe Brezmes-Valdivieso; Silvia Rojo
EPI_ISL_913042 to 913051	Hospital Comarcal Sierrallana	Instituto de Salud Carlos III	A. Monzón; F. Casas; I. De Benito, I.; I. Jiménez; Iglesias-Caballero; M. Camarero; P. Zaballos; S. Cuesta; S. Pozo; S. Sandonís; S. Varona; V. Vázquez
EPI_ISL_455344 to 455349, EPI_ISL_539567 to 539568, EPI_ISL_804949 to 804979, EPI_ISL_862589 to 862626, EPI_ISL_954798, EPI_ISL_965930, EPI_ISL_965936, EPI_ISL_965949			
see above	Hospital Comarcal de Melilla	Instituto de Salud Carlos III	A. Monzón; C. Ezpeleta; F. Casas; I; I. Jiménez; I. López, J.; I. Pérez; I. Roman, S.; I. Román, S.; Iglesias-Caballero; J. López; M. Camarero; M. Cuesta; M. González-Esguevillas; M. Molinero Calamita; M. Pozo; M. Sandonís; M. Zaballos; P. Jiménez; P. Zaballos; S. Camarero; S. Cuesta; S. Iglesias-Caballero; S. Juliá; S. Molinero Calamita; S. Pozo; S. Sandonís; S. Varona; Sandonís; V. Vázquez
EPI_ISL_462477, EPI_ISL_539499	Hospital Costa del Sol	Instituto de Salud Carlos III	A. Monzón; F. Casas; F. Fernández; I; I. Jiménez; Iglesias-Caballero; M. Camarero; M. Cuesta; M. González-Esguevillas; M. Molinero Calamita; M. Zaballos; P. Jiménez; S. Juliá; S. Pozo; S. Varona
EPI_ISL_512665	Hospital De Las Mujeres Dr. Adolfo Carit	Inciensa, Instituto Costarricense de Investigación y Enseñanza en Nutrición y Salud	Adriana Godinez & Melany Calderon; Claudio Soto-Garita; Estela Cordero; Francisco Duarte; Hebleen Porras
EPI_ISL_512655, EPI_ISL_512672 to 512675, EPI_ISL_527750	Hospital De Niños Dr. Carlos Saenz Herrera [San Jose/San Jose]	Inciensa, Instituto Costarricense de Investigación y Enseñanza en Nutrición y Salud	Adriana Godinez & Melany Calderon; Claudio Soto-Garita; Estela Cordero; Francisco Duarte; Hebleen Porras
EPI_ISL_1091919 to 1091921	Hospital Departamental María Inmaculada	Instituto Nacional de Salud- Dirección de Investigación en Salud Pública	Carlos Franco-Muñoz; Diego A. Álvarez-Díaz; Diego Andrés Prada; Gerardo Santamaría; Hector Alejandro Ruiz-Moreno; Jhonnatan Reales-González; Julian Naizaque; Katherine Laiton-Donato; Magdalena Wiesner; Marcela Mercado-Reyes; Maria T. Herrera-Sepúlveda; Martha Lucia Ospina Martinez; Sheryll Corchuelo
EPI_ISL_574591 to 574592, EPI_ISL_603030, EPI_ISL_693218	Hospital Domingos Leonardo Ceravolo Presidente Prudente	Instituto Adolfo Lutz, Interdiciplinary Procedures Center, Strategic Laboratory	Claudia Regina Gonçalves; Claudio Tavares Sacchi; Erica Valessa Ramos Gomes; Karoline Rodrigues Campos
EPI_ISL_547578	Hospital Doutor Domingos Leonardo Cerávolo	Instituto Adolfo Lutz, Interdiciplinary Procedures Center, Strategic Laboratory	Claudia Regina Gonçalves; Claudio Tavares Sacchi; Erica Valessa Ramos Gomes; Karoline Rodrigues Campos
EPI_ISL_527752, EPI_ISL_769990	Hospital Dr. Rafael A. Calderon Guardia	Inciensa, Instituto Costarricense de Investigación y Enseñanza en Nutrición y Salud	Adriana Godinez & Melany Calderon; Adriana Godínez; Claudio Soto-Garita; Estela Cordero; Francisco Duarte; Hebleen Porras; Melany Calderón & Mariel López
EPI_ISL_512654	Hospital Dr. Rafael A. Calderon Guardia [San Jose/San Jose]	Inciensa, Instituto Costarricense de Investigación y Enseñanza en Nutrición y Salud	Adriana Godinez & Melany Calderon; Claudio Soto-Garita; Estela Cordero; Francisco Duarte; Hebleen Porras
EPI_ISL_770028	Hospital Dr. Raul Blanco Cervantes	Inciensa, Instituto Costarricense de Investigación y Enseñanza en Nutrición y Salud	Adriana Godínez; Claudio Soto-Garita; Estela Cordero; Francisco Duarte; Hebleen Porras; Melany Calderón & Mariel López
EPI_ISL_1121307	Hospital E Antonio Policarpo de Oliveira	Instituto Adolfo Lutz, Interdiciplinary Procedures Center, Strategic Laboratory	Caio Vinicius Dias Lopes; Claudia Regina Gonçalves; Claudio Tavares Sacchi; Erica Valessa Ramos Gomes; Karoline Rodrigues Campos
EPI_ISL_693213	Hospital E Maternidade Municipal Governador Mario Covas	Instituto Adolfo Lutz, Interdiciplinary Procedures Center,	Claudia Regina Gonçalves; Claudio Tavares Sacchi; Erica Valessa Ramos Gomes; Karoline Rodrigues Campos

		Strategic Laboratory	
EPI_ISL_936494	Hospital Enche Besar Hajjah Khalsom Kluang	Institute for Medical Research, Infectious Disease Research Centre, National Institutes of Health, Ministry of Health Malaysia	Azizan MA; Kamel K; Suppiah J; Thayan R
EPI_ISL_574594	Hospital Escola da Universidade de Taubate	Instituto Adolfo Lutz, Interdiciplinary Procedures Center, Strategic Laboratory	Claudia Regina Gonçalves; Claudio Tavares Sacchi; Erica Valessa Ramos Gomes; Karoline Rodrigues Campos
EPI_ISL_574588, EPI_ISL_583490	Hospital Estadual Sumare	Instituto Adolfo Lutz, Interdiciplinary Procedures Center, Strategic Laboratory	Claudia Regina Gonçalves; Claudio Tavares Sacchi; Erica Valessa Ramos Gomes; Karoline Rodrigues Campos
EPI_ISL_1303542 to 1303543	Hospital Estadual de Campanha Barradas	Instituto Adolfo Lutz, Interdiciplinary Procedures Center, Strategic Laboratory	Caio Vinicius Dias Lopes; Claudia Regina Gonçalves; Claudio Tavares Sacchi; Erica Valessa Ramos Gomes; Karoline Rodrigues Campos
EPI_ISL_583501	Hospital Estadual de CampanhaCOVID 19 Barradas	Instituto Adolfo Lutz, Interdiciplinary Procedures Center, Strategic Laboratory	Claudia Regina Gonçalves; Claudio Tavares Sacchi; Erica Valessa Ramos Gomes; Karoline Rodrigues Campos
EPI_ISL_1303537	Hospital Estadual de Vila Alpina	Instituto Adolfo Lutz, Interdiciplinary Procedures Center, Strategic Laboratory	Caio Vinicius Dias Lopes; Claudia Regina Gonçalves; Claudio Tavares Sacchi; Erica Valessa Ramos Gomes; Karoline Rodrigues Campos
EPI_ISL_491443, EPI_ISL_491447	Hospital Fernando Escalante Pradilla	Inciensa, Instituto Costarricense de Investigación y Enseñanza en Nutrición y Salud	Adriana Godinez & Melany Calderon; Claudio Soto-Garita; Estela Cordero; Francisco Duarte; Hebleen Brenes
EPI_ISL_476496, EPI_ISL_476561	Hospital Garrahan	Héritas	Andrea Mangano; Cristian Rohr; Dalmacio Pereyra; Fabian Fay; Maria Florencia Fernandez; Martin Vazquez; Mauricio Grisolia; Roberta Crespo
EPI_ISL_578186	Hospital General Juan Ramón Jiménez	Instituto de Salud Carlos III	A. Monzón; F. Casas; I; I. Jiménez; Iglesias-Caballero; J. Saavedra; M. Camarero; M. Cuesta; M. González-Esguevillas; M. Molinero Calamita; M. Zaballos; P. Jiménez; S. Juliá; S. Pozo; S. Varona
EPI_ISL_424731	Hospital General Regional No.66, Ciudad Juárez, Chihuahua.	Laboratorio Central de Epidemiología-DLVIE / Laboratorio de Secuenciación-Centro de Instrumentos. Instituto Mexicano del Seguro Social	Araiza-Rodríguez A; Arias C; Barrera-Badillo G; Becerril-Vargas E; Borja-Aburto VH.; Boukadida C; Garcés-Ayala F; García-Andrade LA; Gaytán-Cervantes FJ; González-Bonilla CR; González-Torres C; Grajales-Muñiz C; Grande R; Hernández-Rivas L; Ibarra-González V; Isa P; Ledesma-Barrientos F; López-Martínez I; Martínez-Orozco JA; Mendieta-Condado E; Muñoz-Medina JE; Mújica-Sánchez M; Pulido F. López S; Ramos-Cervantes P; Ramírez-González JE; Ruiz-Palacios GM; Sánchez A; Taboada B; Vázquez G; Vázquez-Pérez JA; de León-Garduño AP; Ávila-Ríos S
EPI_ISL_756271, EPI_ISL_756273 to 756274, EPI_ISL_776770, EPI_ISL_776772, EPI_ISL_776870 to 776871, EPI_ISL_776875, EPI_ISL_776877, EPI_ISL_777490, EPI_ISL_777902, EPI_ISL_778445, EPI_ISL_778644 to 778645, EPI_ISL_803854 to 803864, EPI_ISL_885150, EPI_ISL_1173765, EPI_ISL_1180260 to 1180268, EPI_ISL_1180270 to 1180278, EPI_ISL_1184469 to 1184503, EPI_ISL_1185847, EPI_ISL_1185849, EPI_ISL_1185851, EPI_ISL_1185853, EPI_ISL_1201203 to 1201279, EPI_ISL_1201298, EPI_ISL_1233377, EPI_ISL_1233379 to 1233423, EPI_ISL_1233819, EPI_ISL_1233873, EPI_ISL_1307715, EPI_ISL_1314364 to 1314401, EPI_ISL_1314403 to 1314406			
see above	Hospital General Universitario Gregorio Marañón	Hospital General Universitario Gregorio Marañón	Cristina Rodriguez-Grande; Darío García de Viedma; Darío García de Viedma.; Darío García de Viedma"; Laura Perez-Lago; Laura Pérez-Lago; Patricia Muñoz; Pedro Sola Campoy; Pilar Catalán; Sergio Buenestado Serrano; "Sergio Buenestado Serrano
EPI_ISL_912268 to 912352	Hospital General Universitario Gregorio Marañón	SeqCOVID-SPAIN consortium / IBV (CSIC)	Andrés Von Wernitz; Arturo fraile Torres; Carmen del Arco; Darío García de Viedma; Darío García de Viedma and SeqCOVID-SPAIN consortium; Julia Suárez; Laura Cardeñoso; Laura Pérez-Lago; Marta Herranz; Patricia Muñoz; Patricia Muñoz and SeqCOVID-SPAIN consortium; Pedro J Sola-Campoy; Pedro J. Sola Campoy; Pilar Catalán; Sergio Buenestado Serrano; Sergio Buenestado-Serrano; Victor Manuel de la Cueva
EPI_ISL_467184 to 467261, EPI_ISL_481041 to 481109, EPI_ISL_510103 to 510151, EPI_ISL_510165 to 510245, EPI_ISL_541878 to 541941, EPI_ISL_582812 to 582832, EPI_ISL_582843 to 582844, EPI_ISL_654021 to 654080, EPI_ISL_654086 to 654087, EPI_ISL_654091, EPI_ISL_654097, EPI_ISL_654099, EPI_ISL_654102, EPI_ISL_654116, EPI_ISL_654135, EPI_ISL_654139, EPI_ISL_654142, EPI_ISL_654154, EPI_ISL_654159, EPI_ISL_654162 to 654400, EPI_ISL_779955 to 780095, EPI_ISL_780414 to 780415, EPI_ISL_813980 to 814061, EPI_ISL_852947 to 853032, EPI_ISL_861773 to 861855, EPI_ISL_1272405 to 1272431, EPI_ISL_1272434 to 1272437, EPI_ISL_1272439 to 1272471, EPI_ISL_1272473 to 1272548			
see above	Hospital General Universitario Gregorio Marañón	SeqCOVID-SPAIN consortium/IBV(CSIC)	Darío García de Viedma; Darío García de Viedma and SeqCOVID-SPAIN consortium; Jon Sicilia; Julia Suárez; Laura Pérez-Lago; Marta Herranz; Patricia Muñoz; Patricia Muñoz and SeqCOVID-SPAIN consortium; Pedro J Sola-Campoy; Pilar Catalán; Sergio Buenestado-Serrano; Victor Manuel de la Cueva
EPI_ISL_756272	Hospital General Universitario La Princesa	Hospital General Universitario Gregorio Marañón	Andrés Von Wernitz; Arturo fraile Torres; Carmen del Arco; Darío García de Viedma.; Laura Cardeñoso; Patricia Muñoz; Pedro Sola Campoy; Pilar Catalán; Sergio Buenestado Serrano
EPI_ISL_961204 to 961285, EPI_ISL_1311868 to 1311870, EPI_ISL_1311874 to 1311877, EPI_ISL_1311882, EPI_ISL_1311884 to 1311890, EPI_ISL_1311893 to 1311901, EPI_ISL_1311906, EPI_ISL_1311909 to 1311913, EPI_ISL_1311915 to 1311919, EPI_ISL_1311923 to 1311930, EPI_ISL_1311932 to 1311936, EPI_ISL_1311938 to 1311939, EPI_ISL_1311941 to 1311945, EPI_ISL_1311947 to 1311948			
see above	Hospital General Universitario de Alicante - Instituto de Investigación Sanitaria y Biomédica de Alicante	SeqCOVID-SPAIN consortium/IBV(CSIC)	Carmen Molina Pardines and SeqCOVID-SPAIN consortium; Maripaz Ventero Martín
EPI_ISL_691682 to 691683, EPI_ISL_954781, EPI_ISL_954805, EPI_ISL_954810, EPI_ISL_965914 to 965915, EPI_ISL_965926, EPI_ISL_965934, EPI_ISL_965940, EPI_ISL_965946, EPI_ISL_965959			
see above	Hospital General Universitario de Ciudad Real	Instituto de Salud Carlos III	A. Monzón; F. Casas; I. Illescas, S.; I. Jarilla, M.; I. Jiménez; I. Madrigal, M.; Iglesias-Caballero; M. Camarero; M. Cuesta; M. González-Esguevillas; M. Pozo; M. Sandonís; M. Zaballos; P. Jiménez; P. Zaballos; S. Camarero; S. Cuesta; S. Iglesias-Caballero; S. Juliá; S. Molinero Calamita; S. Pozo; S. Sandonís; S. Varona; Sandonís; V. Camarero; V. Vázquez; Vázquez
EPI_ISL_954809	Hospital General de Lanzarote	Instituto de Salud Carlos III	A. Monzón; F. Casas; I. Copado, R.; I. Jiménez; Iglesias-Caballero; M. Camarero; P. Zaballos; S. Cuesta; S. Pozo; S. Sandonís; S. Varona; V. Vázquez
EPI_ISL_1110240 to 1110247	Hospital General de Requena	SeqCOVID-SPAIN consortium/IBV(CSIC)	Carolina Pla Cortes and SeqCOVID-SPAIN consortium; Martín Yago López; María Ángeles Goberna Bravo
EPI_ISL_539523	Hospital General de Segovia	Instituto de Salud Carlos III	A. Monzón; F. Casas; I; I. Jiménez; Iglesias-Caballero; M. Camarero; M. Cuesta; M. González-Esguevillas; M. Molinero Calamita; M. Zaballos; P. Jiménez; S. Hernando; S. Juliá; S. Pozo; S. Varona
EPI_ISL_418245 to 418246	Hospital General y Universitario de Guadalajara	Instituto de Salud Carlos III	A. Monzón; F. Casas; I. Gonzalez-Praetorius A.; I. Jiménez; Iglesias-Caballero; M. Camarero; M. Cuesta; M. González-Esguevillas; M. Molinero Calamita; M. Zaballos; P. Jiménez; S. Juliá; S. Pozo; S. Varona
EPI_ISL_471541, EPI_ISL_523971	Hospital Geral Santa Marcelina	Instituto Adolfo Lutz, Interdiciplinary Procedures Center, Strategic Laboratory	Claudia Regina Gonçalves; Claudio Tavares Sacchi; Erica Valessa Ramos Gomes
EPI_ISL_534317	Hospital Geral de Itapevi	Instituto Adolfo Lutz, Interdiciplinary Procedures Center, Strategic Laboratory	Claudia Regina Gonçalves; Claudio Tavares Sacchi; Erica Valessa Ramos Gomes
EPI_ISL_603037	Hospital Geral de Pedreira	Instituto Adolfo Lutz, Interdiciplinary Procedures Center, Strategic Laboratory	Claudia Regina Gonçalves; Claudio Tavares Sacchi; Erica Valessa Ramos Gomes; Karoline Rodrigues Campos
EPI_ISL_861636	Hospital Geral de Sao Mateus São Paulo	Instituto Adolfo Lutz, Interdiciplinary Procedures Center, Strategic Laboratory	Claudia Regina Gonçalves; Claudio Tavares Sacchi; Erica Valessa Ramos Gomes; Karoline Rodrigues Campos
EPI_ISL_940630, EPI_ISL_943967 to 943972	Hospital Geral de Sao Paulo	Instituto Adolfo Lutz, Interdiciplinary Procedures Center, Strategic Laboratory	Claudia Regina Gonçalves; Claudio Tavares Sacchi; Erica Valessa Ramos Gomes; Karoline Rodrigues Campos
EPI_ISL_515555	Hospital Geral de Vila Nova Cachoeirinha	Instituto Adolfo Lutz, Interdiciplinary Procedures Center, Strategic Laboratory	Claudia Regina Gonçalves; Claudio Tavares Sacchi; Erica Valessa Ramos Gomes
EPI_ISL_906075	Hospital Geral de Vila Penteado Dr Jose Pangella Sao Paulo	Instituto Adolfo Lutz, Interdiciplinary Procedures Center, Strategic Laboratory	Claudia Regina Gonçalves; Claudio Tavares Sacchi; Erica Valessa Ramos Gomes; Karoline Rodrigues Campos
EPI_ISL_574595	Hospital Geral de Vila Penteado Dr. Jose Pamgella	Instituto Adolfo Lutz, Interdiciplinary Procedures Center, Strategic Laboratory	Claudia Regina Gonçalves; Claudio Tavares Sacchi; Erica Valessa Ramos Gomes; Karoline Rodrigues Campos
EPI_ISL_1138411	Hospital HIA Percy, Medical Biology	National Reference Center for Viruses of Respiratory Infections, Institut Pasteur, Paris	Angela Brisebarre; Camille Capel; Etienne Simon-Lorière; Marion Barbet; Maud Vanpeene; Méline Bizard; Sylvie Behillil; Sylvie van der Werf; Vincent Enouf; Vincent Foissaud

EPI_ISL_1303535 to 1303536	Hospital Heliopolis	Instituto Adolfo Lutz, Interdiciplinary Procedures Center, Strategic Laboratory	Caio Vinicius Dias Lopes; Claudia Regina Gonçalves; Claudio Tavares Sacchi; Erica Valessa Ramos Gomes; Karoline Rodrigues Campos
EPI_ISL_471267 to 471268, EPI_ISL_481244 to 481248, EPI_ISL_574431	Hospital IESS Babahoyo	Institute of Microbiology, Universidad San Francisco de Quito	Belén Prado-Vivar; Bernardo Gutiérrez; Carla Torres; Fernanda Zurita; Francisco Cordova; Gabriel Trueba; Juan José Guadalupe; Killen Briones-Claudette; Killen Briones-Zamora; Michelle Grunauer; Monica Becerra-Wong; Ninfa Henriquez; Patricio Rojas-Silva; Paúl Cárdenas; Sully Márquez; Verónica Barragán
EPI_ISL_871974 to 871975, EPI_ISL_871979	Hospital Infanta Cristina	Instituto de Salud Carlos III	A. Monzón; F. Casas; I. García, P.; I. Jiménez; Iglesias-Caballero; M. Camarero; M. Cuesta; M. González-Esguevillas; M. Pozo; M. Zaballos; P. Jiménez; S. Juliá; S. Molinero Calamita; S. Varona
EPI_ISL_412964	Hospital Israelita Albert Einstein	Instituto Adolfo Lutz Interdisciplinary Procedures Center Strategic Laboratory	Andrew Rambaut; Claudia Regina Gonçalves; Claudio Tavares Sacchi; Daniela Bernardes Borges da Silva; Ester Cerdeira Sabino; Flávia Cristina da Silva Sales; Ingra Morales Claro; Jaqueline Goes de Jesus; Joshua Quick; Maria do Carmo; Nicholas James Loman; Nuno Rodrigues Faria; Sampaio Tavares Timenetsky
EPI_ISL_414014, EPI_ISL_416033 to 416034	Hospital Israelita Albert Einstein	Instituto Adolfo Lutz, Interdiciplinary Procedures Center, Strategic Laboratory	Adriana Bugno; Adriano Abbud; Carlos Henrique Camargo; Claudia Regina Gonçalves; Claudio Tavares Sacchi; Daniela Bernardes Borges da Silva; Erica Valessa Ramos Gomes; Ester Cerdeira Sabino; Fabiana Cristina Pereira dos Santos; Katia Correia dos Santos; Maria do Carmo Sampaio Tavares Timenetsky; Maria do Carmo Sampaito Tavares Timenetsky; Simone Guadagnucci Morillo; Terezinha Maria de Paiva
EPI_ISL_413016	Hospital Israelita Albert Einstein	Instituto Adolfo Lutz, Interdisciplinary Procedures Center, Strategic Laboratory	Andrew Rambaut; Claudia Regina Gonçalves; Claudio Tavares Sacchi; Ester Cerdeira Sabino; Fabiana Cristina Pereira dos Santos; Flávia Cristina da Silva Sales; Ingra Morales Claro; Jaqueline Goes de Jesus; Joshua Quick; Maria do Carmo Sampaio Tavares Timenetsky; Nicholas James Loman; Nuno Rodrigues Faria
EPI_ISL_523957	Hospital Itamaraty	Instituto Adolfo Lutz, Interdiciplinary Procedures Center, Strategic Laboratory	Claudia Regina Gonçalves; Claudio Tavares Sacchi; Erica Valessa Ramos Gomes
EPI_ISL_862551, EPI_ISL_954787, EPI_ISL_954790	Hospital J.M. Morales Meseguer	Instituto de Salud Carlos III	A. Monzón; F. Casas; I. Guerrero, C.; I. Jiménez; I. Navarro, D.; Iglesias-Caballero; M. . Pozo; M. Camarero; M. Cuesta; M. González-Esguevillas; M. Zaballos; P. Jiménez; P. Zaballos; S. Cuesta; S. Juliá; S. Molinero Calamita; S. Pozo; S. Sandonís; S. Varona; V. Vázquez
EPI_ISL_954797, EPI_ISL_954801, EPI_ISL_954803	Hospital Los Arcos	Instituto de Salud Carlos III	A. Monzón; F. Casas; I. Camara, M.; I. Jiménez; Iglesias-Caballero; M. Camarero; P. Zaballos; S. Cuesta; S. Pozo; S. Sandonís; S. Varona; V. Vázquez
EPI_ISL_965911, EPI_ISL_965913, EPI_ISL_965917 to 965919, EPI_ISL_965923, EPI_ISL_965928 to 965929, EPI_ISL_965932, EPI_ISL_965935, EPI_ISL_965939, EPI_ISL_965941, EPI_ISL_965945, EPI_ISL_965947, EPI_ISL_965951, EPI_ISL_965955, EPI_ISL_965958			
see above	Hospital Los Arcos del Mar Menor	Instituto de Salud Carlos III	A. Monzón; F. Casas; I. Cámara, M.; I. Jiménez; Iglesias-Caballero; M. Camarero; M. Sandonís; P. Zaballos; S. Camarero; S. Cuesta; S. Iglesias-Caballero; S. Pozo; S. Varona; Sandonís; V. Camarero; V. Vázquez; Vázquez
EPI_ISL_1120614	Hospital Margarita Maza de Juarez	CIAD LDM-LGM	Bruno Gome-Gil; Julissa Enciso-Ibarra
EPI_ISL_1196660, EPI_ISL_1196705 to 1196706, EPI_ISL_1196768, EPI_ISL_1196770 to 1196771	Hospital Melaka	Institute for Medical Research, Infectious Disease Research Centre, National Institutes of Health, Ministry of Health Malaysia	Kamel K; Mohd Zawawi Z; Ramly N; Robert F; Suppiah J; Thayan R
EPI_ISL_527741, EPI_ISL_769992, EPI_ISL_769995	Hospital Metropolitano	Inciensa, Instituto Costarricense de Investigación y Enseñanza en Nutrición y Salud	Adriana Godinez & Melany Calderon; Adriana Godínez; Claudio Soto-Garita; Estela Cordero; Francisco Duarte; Hebleen Porras; Melany Calderón & Margarita Lee Lui
EPI_ISL_524462	Hospital Metropolitano	Instituto Adolfo Lutz, Interdiciplinary Procedures Center, Strategic Laboratory	Claudia Regina Gonçalves; Claudio Tavares Sacchi; Erica Valessa Ramos Gomes
EPI_ISL_458236, EPI_ISL_480312 to 480314	Hospital Mexico	Charité Virology-University of Costa Rica	Andres Moreira-Soto; Eugenia Corrales-Aguilar; Ignacio Postigo-Hidalgo; Jan Felix Drexler; Teresita Somogyi
EPI_ISL_527739, EPI_ISL_527758	Hospital Mexico [San Jose/San Jose]	Inciensa, Instituto Costarricense de Investigación y Enseñanza en Nutrición y Salud	Adriana Godinez & Melany Calderon; Claudio Soto-Garita; Estela Cordero; Francisco Duarte; Hebleen Porras
EPI_ISL_515541, EPI_ISL_515561	Hospital Montemagno	Instituto Adolfo Lutz, Interdiciplinary Procedures Center, Strategic Laboratory	Claudia Regina Gonçalves; Claudio Tavares Sacchi; Erica Valessa Ramos Gomes
EPI_ISL_534324	Hospital Mun Ver Jose Storopolli	Instituto Adolfo Lutz, Interdiciplinary Procedures Center, Strategic Laboratory	Claudia Regina Gonçalves; Claudio Tavares Sacchi; Erica Valessa Ramos Gomes
EPI_ISL_534318, EPI_ISL_693208 to 693209	Hospital Municipal Antonio Giglio	Instituto Adolfo Lutz, Interdiciplinary Procedures Center, Strategic Laboratory	Claudia Regina Gonçalves; Claudio Tavares Sacchi; Erica Valessa Ramos Gomes; Karoline Rodrigues Campos
EPI_ISL_547571, EPI_ISL_861658	Hospital Municipal Antônio Giglio	Instituto Adolfo Lutz, Interdiciplinary Procedures Center, Strategic Laboratory	Claudia Regina Gonçalves; Claudio Tavares Sacchi; Erica Valessa Ramos Gomes; Karoline Rodrigues Campos
EPI_ISL_471549, EPI_ISL_523991 to 523992, EPI_ISL_527869	Hospital Municipal Carmen Prudente	Instituto Adolfo Lutz, Interdiciplinary Procedures Center, Strategic Laboratory	Claudia Regina Gonçalves; Claudio Tavares Sacchi; Erica Valessa Ramos Gomes
EPI_ISL_1303540 to 1303541, EPI_ISL_1303544 to 1303545	Hospital Municipal Cidade Tiradentes Carmen Prudente	Instituto Adolfo Lutz, Interdiciplinary Procedures Center, Strategic Laboratory	Caio Vinicius Dias Lopes; Claudia Regina Gonçalves; Claudio Tavares Sacchi; Erica Valessa Ramos Gomes; Karoline Rodrigues Campos
EPI_ISL_515562	Hospital Municipal Doutor Alexandre Zaio	Instituto Adolfo Lutz, Interdiciplinary Procedures Center, Strategic Laboratory	Claudia Regina Gonçalves; Claudio Tavares Sacchi; Erica Valessa Ramos Gomes
EPI_ISL_693203	Hospital Municipal Doutor Arthur Ribeiro de Saboya	Instituto Adolfo Lutz, Interdiciplinary Procedures Center, Strategic Laboratory	Claudia Regina Gonçalves; Claudio Tavares Sacchi; Erica Valessa Ramos Gomes; Karoline Rodrigues Campos
EPI_ISL_468311 to 468312	Hospital Municipal Dr Ignacio Proenca de Gouvea	Instituto Adolfo Lutz, Interdiciplinary Procedures Center, Strategic Laboratory	Claudia Regina Gonçalves; Claudio Tavares Sacchi; Erica Valessa Ramos Gomes
EPI_ISL_515521	Hospital Municipal Dr Waldemar Tebaldi	Instituto Adolfo Lutz, Interdiciplinary Procedures Center, Strategic Laboratory	Claudia Regina Gonçalves; Claudio Tavares Sacchi; Erica Valessa Ramos Gomes
EPI_ISL_523985	Hospital Municipal Dr. Benedicto Montenegro	Instituto Adolfo Lutz, Interdiciplinary Procedures Center, Strategic Laboratory	Claudia Regina Gonçalves; Claudio Tavares Sacchi; Erica Valessa Ramos Gomes
EPI_ISL_882671 to 882673	Hospital Municipal Dr. Guido Guida	Instituto Adolfo Lutz, Interdiciplinary Procedures Center, Strategic Laboratory	Claudia Regina Gonçalves; Claudio Tavares Sacchi; Erica Valessa Ramos Gomes; Karoline Rodrigues Campos
EPI_ISL_515553, EPI_ISL_574577, EPI_ISL_574579	Hospital Municipal Dr. Ignacio Proença de Gouvea	Instituto Adolfo Lutz, Interdiciplinary Procedures Center, Strategic Laboratory	Claudia Regina Gonçalves; Claudio Tavares Sacchi; Erica Valessa Ramos Gomes; Karoline Rodrigues Campos
EPI_ISL_515548, EPI_ISL_515563, EPI_ISL_574582, EPI_ISL_574589	Hospital Municipal Dr. Jose Soares Hungria	Instituto Adolfo Lutz, Interdiciplinary Procedures Center, Strategic Laboratory	Claudia Regina Gonçalves; Claudio Tavares Sacchi; Erica Valessa Ramos Gomes; Karoline Rodrigues Campos
EPI_ISL_861638, EPI_ISL_861640 to 861641	Hospital Municipal Dr. Moyses Deutsch	Instituto Adolfo Lutz, Interdiciplinary Procedures Center, Strategic Laboratory	Claudia Regina Gonçalves; Claudio Tavares Sacchi; Erica Valessa Ramos Gomes; Karoline Rodrigues Campos
EPI_ISL_515557, EPI_ISL_524467	Hospital Municipal Dr. Moysés Deutsch	Instituto Adolfo Lutz, Interdiciplinary Procedures Center, Strategic Laboratory	Claudia Regina Gonçalves; Claudio Tavares Sacchi; Erica Valessa Ramos Gomes
EPI_ISL_583498, EPI_ISL_861681	Hospital Municipal Dr. Waldemar Tebaldi	Instituto Adolfo Lutz, Interdiciplinary Procedures Center, Strategic Laboratory	Claudia Regina Gonçalves; Claudio Tavares Sacchi; Erica Valessa Ramos Gomes; Karoline Rodrigues Campos
EPI_ISL_1220095	Hospital Municipal Gov. Mario Covas Jr.	Instituto Butantan (genome assembly and bioinformatics) and	Antonio Jorge Martins; Dimas Tadeu Covas; Jose Patane; Maria Carolina Quartim Barbosa Elias Sabbaga; Rafael dos Santos Bezerra; Sandra Coccuzzo

		Mendelics (sequencing)	Sampaio Vessoni; Simone Haddad
EPI_ISL_977490	Hospital Municipal Guido Guida	Instituto Adolfo Lutz, Interdiciplinary Procedures Center, Strategic Laboratory	Claudia Regina Gonçalves; Claudio Tavares Sacchi; Erica Valessa Ramos Gomes; Karoline Rodrigues Campos
EPI_ISL_940629	Hospital Municipal Josanias Castanha Braga	Instituto Adolfo Lutz, Interdiciplinary Procedures Center, Strategic Laboratory	Claudia Regina Gonçalves; Claudio Tavares Sacchi; Erica Valessa Ramos Gomes; Karoline Rodrigues Campos
EPI_ISL_693206	Hospital Municipal Mario Gatti	Instituto Adolfo Lutz, Interdiciplinary Procedures Center, Strategic Laboratory	Claudia Regina Gonçalves; Claudio Tavares Sacchi; Erica Valessa Ramos Gomes; Karoline Rodrigues Campos
EPI_ISL_527870, EPI_ISL_574578, EPI_ISL_603029, EPI_ISL_603039	Hospital Municipal Mário Gatti	Instituto Adolfo Lutz, Interdiciplinary Procedures Center, Strategic Laboratory	Claudia Regina Gonçalves; Claudio Tavares Sacchi; Erica Valessa Ramos Gomes; Karoline Rodrigues Campos
EPI_ISL_861671, EPI_ISL_882660	Hospital Municipal Prefeito Waldemar Costa Filho	Instituto Adolfo Lutz, Interdiciplinary Procedures Center, Strategic Laboratory	Claudia Regina Gonçalves; Claudio Tavares Sacchi; Erica Valessa Ramos Gomes; Karoline Rodrigues Campos
EPI_ISL_527856	Hospital Municipal Prof. Waldomiro de Paula	Instituto Adolfo Lutz, Interdiciplinary Procedures Center, Strategic Laboratory	Claudia Regina Gonçalves; Claudio Tavares Sacchi; Erica Valessa Ramos Gomes
EPI_ISL_603028	Hospital Municipal Santa Ana	Instituto Adolfo Lutz, Interdiciplinary Procedures Center, Strategic Laboratory	Claudia Regina Gonçalves; Claudio Tavares Sacchi; Erica Valessa Ramos Gomes; Karoline Rodrigues Campos
EPI_ISL_524468, EPI_ISL_527859	Hospital Municipal Vereador Jose Storopolli	Instituto Adolfo Lutz, Interdiciplinary Procedures Center, Strategic Laboratory	Claudia Regina Gonçalves; Claudio Tavares Sacchi; Erica Valessa Ramos Gomes
EPI_ISL_471647	Hospital Municipal de Barueri Dr. Francisco Moran	Instituto Adolfo Lutz, Interdiciplinary Procedures Center, Strategic Laboratory	Claudia Regina Gonçalves; Claudio Tavares Sacchi; Erica Valessa Ramos Gomes
EPI_ISL_527860	Hospital Municipal de Parelheiros Josanias Castanha Braga	Instituto Adolfo Lutz, Interdiciplinary Procedures Center, Strategic Laboratory	Claudia Regina Gonçalves; Claudio Tavares Sacchi; Erica Valessa Ramos Gomes
EPI_ISL_527862	Hospital Municipal de Urgência	Instituto Adolfo Lutz, Interdiciplinary Procedures Center, Strategic Laboratory	Claudia Regina Gonçalves; Claudio Tavares Sacchi; Erica Valessa Ramos Gomes
EPI_ISL_468308, EPI_ISL_468315, EPI_ISL_515520, EPI_ISL_515526, EPI_ISL_515546, EPI_ISL_515551 to 515552, EPI_ISL_523955, EPI_ISL_523974, EPI_ISL_523976, EPI_ISL_527863			
see above	Hospital Municipal do Tatuape Carmino Caricchio	Instituto Adolfo Lutz, Interdiciplinary Procedures Center, Strategic Laboratory	Claudia Regina Gonçalves; Claudio Tavares Sacchi; Erica Valessa Ramos Gomes
EPI_ISL_735399	Hospital Municipiplal Dr Ignacio de gouvea	Instituto Adolfo Lutz, Interdiciplinary Procedures Center, Strategic Laboratory	Claudia Regina Gonçalves; Claudio Tavares Sacchi; Erica Valessa Ramos Gomes; Karoline Rodrigues Campos
EPI_ISL_836977	Hospital Municipiplal Dr. Jose de Carvalho Florence	Instituto Adolfo Lutz, Interdiciplinary Procedures Center, Strategic Laboratory	Claudia Regina Gonçalves; Claudio Tavares Sacchi; Erica Valessa Ramos Gomes; Karoline Rodrigues Campos
EPI_ISL_491451, EPI_ISL_491453	Hospital México	Inciensa, Instituto Costarricense de Investigación y Enseñanza en Nutrición y Salud	Adriana Godinez & Melany Calderon; Claudio Soto-Garita; Estela Cordero; Francisco Duarte; Hebleen Brenes
EPI_ISL_480322 to 480327	Hospital Nacional de Niños	Charité Virology-University of Costa Rica	Andrei Montero Bonilla; Andres Moreira-Soto; Cristian Pérez Corrales; Eugenia Corrales-Aguilar; Ignacio Postigo-Hidalgo; Jan Felix Drexler
EPI_ISL_735428 to 735429, EPI_ISL_735431 to 735432, EPI_ISL_861637, EPI_ISL_906066	Hospital Nipo Brasileiro	Instituto Adolfo Lutz, Interdiciplinary Procedures Center, Strategic Laboratory	Claudia Regina Gonçalves; Claudio Tavares Sacchi; Erica Valessa Ramos Gomes; Karoline Rodrigues Campos
EPI_ISL_539496	Hospital Nostra Senyora de Meritxell	Instituto de Salud Carlos III	A. Monzón; F. Casas; F. Fernández; I; I. Jiménez; Iglesias-Caballero; M. Camarero; M. Cuesta; M. González-Esguevillas; M. Molinero Calamita; M. Zaballos; P. Jiménez; S. Juliá; S. Pozo; S. Varona
EPI_ISL_539525, EPI_ISL_862637 to 862639, EPI_ISL_913061 to 913062	Hospital Nuestra Señora de Sonsoles	Instituto de Salud Carlos III	A. Monzón; A. San Pedro; F. Casas; I; I. Jiménez; I. Sánchez, R.; Iglesias-Caballero; M. Camarero; M. Cuesta; M. González-Esguevillas; M. Molinero Calamita; M. Zaballos; P. Jiménez; P. Zaballos; S. Cuesta; S. Juliá; S. Pozo; S. Sandonís; S. Varona; V. Vázquez
EPI_ISL_471269 to 471271	Hospital Oncológico Solca Núcleo de Quito	Institute of Microbiology, Universidad San Francisco de Quito	Belén Prado-Vivar; Bernardo Gutiérrez; Gabriel Trueba; Grace Salazar; Juan José Guadalupe; Marcos Di Stefano; Michelle Grunauer; Patricio Rojas-Silva; Paúl Cárdenas; Sully Márquez; Verónica Barragán
EPI_ISL_1015358 to 1015360	Hospital Pablo Tobón Uribe	Universidad Nacional de Colombia - Laboratorio Genómico One Health	Andres F. Cardona-Rios; Carlos Franco-Muñoz; Daniel O. Maldonado-Perez; Diego A. Álvarez-Díaz; Idabely Betancur Ortiz; Jorge E. Osorio; Juan P. Hernandez-Ortiz; Karl A Ciuoderis; Laura Silvana Perez; Marcela Mercado-Reyes; Sandra Ines Cano
EPI_ISL_906079, EPI_ISL_940610 to 940611	Hospital Paulistano Paulista	Instituto Adolfo Lutz, Interdiciplinary Procedures Center, Strategic Laboratory	Claudia Regina Gonçalves; Claudio Tavares Sacchi; Erica Valessa Ramos Gomes; Karoline Rodrigues Campos
EPI_ISL_1303538 to 1303539	Hospital Presidente	Instituto Adolfo Lutz, Interdiciplinary Procedures Center, Strategic Laboratory	Caio Vinicius Dias Lopes; Claudia Regina Gonçalves; Claudio Tavares Sacchi; Erica Valessa Ramos Gomes; Karoline Rodrigues Campos
EPI_ISL_419386 to 419387	Hospital Prof. Doutor Fernando Fonseca, EPE	Instituto Gulbenkian de Ciência	Cathy Paulino; Joao Sobral; João Costa; Ricardo Leite; Susana Ladeiro
EPI_ISL_861684 to 861685	Hospital Pronto Socorro Itaquera	Instituto Adolfo Lutz, Interdiciplinary Procedures Center, Strategic Laboratory	Claudia Regina Gonçalves; Claudio Tavares Sacchi; Erica Valessa Ramos Gomes; Karoline Rodrigues Campos
EPI_ISL_1196794, EPI_ISL_1196796 to 1196797	Hospital Pulau Pinang	Institute for Medical Research, Infectious Disease Research Centre, National Institutes of Health, Ministry of Health Malaysia	Kamel K; Mohd Zawawi Z; Ramly N; Robert F; Suppiah J; Thayan R
EPI_ISL_936488, EPI_ISL_1186114	Hospital Queen Elizabeth	Institute for Medical Research, Infectious Disease Research Centre, National Institutes of Health, Ministry of Health Malaysia	Azizan MA; Kamel K; Mohd Zawawi Z; Ramly N; Robert F; Suppiah J; Thayan R
EPI_ISL_936489	Hospital Raja Perempuan Zainab II	Institute for Medical Research, Infectious Disease Research Centre, National Institutes of Health, Ministry of Health Malaysia	Azizan MA; Kamel K; Suppiah J; Thayan R
EPI_ISL_882612 to 882613, EPI_ISL_882615 to 882618, EPI_ISL_882620 to 882621, EPI_ISL_882623 to 882625, EPI_ISL_882627 to 882629, EPI_ISL_882631 to 882632	Hospital Ramón y Cajal	Hospital Ramón y Cajal	Concepción Rodríguez; JC Galán; José M Gonzalez-Alba; Laura Martínez; Luz Leticia Olavarrieta; Melanie Abreu; Rafael Cantón; Val F Lanza
EPI_ISL_527857	Hospital Regional Vale do Ribeira	Instituto Adolfo Lutz, Interdiciplinary Procedures Center, Strategic Laboratory	Claudia Regina Gonçalves; Claudio Tavares Sacchi; Erica Valessa Ramos Gomes
EPI_ISL_523956	Hospital Regional de Assis	Instituto Adolfo Lutz, Interdiciplinary Procedures Center, Strategic Laboratory	Claudia Regina Gonçalves; Claudio Tavares Sacchi; Erica Valessa Ramos Gomes
EPI_ISL_524463	Hospital Regional de Cotia	Instituto Adolfo Lutz, Interdiciplinary Procedures Center, Strategic Laboratory	Claudia Regina Gonçalves; Claudio Tavares Sacchi; Erica Valessa Ramos Gomes
EPI_ISL_735418	Hospital Regional do Vale do Paraiba	Instituto Adolfo Lutz, Interdiciplinary Procedures Center, Strategic Laboratory	Claudia Regina Gonçalves; Claudio Tavares Sacchi; Erica Valessa Ramos Gomes; Karoline Rodrigues Campos

EPI_ISL_861649	Hospital Renascença Campinas	Instituto Adolfo Lutz, Interdiciplinary Procedures Center, Strategic Laboratory	Claudia Regina Gonçalves; Claudio Tavares Sacchi; Erica Valessa Ramos Gomes; Karoline Rodrigues Campos
EPI_ISL_833165 to 833166	Hospital Samaritano	Instituto Adolfo Lutz, Interdiciplinary Procedures Center, Strategic Laboratory	Claudia Regina Gonçalves; Claudio Tavares Sacchi; Erica Valessa Ramos Gomes; Karoline Rodrigues Campos
EPI_ISL_882670	Hospital Samaritano Paulista	Instituto Adolfo Lutz, Interdiciplinary Procedures Center, Strategic Laboratory	Claudia Regina Gonçalves; Claudio Tavares Sacchi; Erica Valessa Ramos Gomes; Karoline Rodrigues Campos
EPI_ISL_970179	Hospital San Agustin, Avilés	Laboratorio de Virología HUCA	Abreu F; Alvarez-Arguelles ME; Boga JA; Castelló C; Costales I; Coto E; Gómez de Oña J; Martín-Rodríguez G; Melón S; Perez-Martínez Z; Rojo S; Sandoval M
EPI_ISL_1020017	Hospital San Agustín	Laboratorio de Virología HUCA	Abreu F; Alvarez-Arguelles ME; Boga JA; Castelló C; Costales I; Coto E; Gómez de Oña J; Martín-Rodríguez G; Melón S; Perez-Martínez Z; Rojo S; Sandoval M
EPI_ISL_993630	Hospital San Agustín	Laboratorio de Virología Hospital Universitario Central de Asturias (HUCA)	Abreu F; Alvarez-Arguelles ME; Boga JA; Castelló C; Costales I; Coto E; Gómez de Oña J; Martín-Rodríguez G; Melón S; Perez-Martínez Z; Rojo S; Sandoval M
EPI_ISL_906532	Hospital San Jose de Maicao	Instituto Nacional de Salud- Dirección de Investigación en Salud Pública, Universidad de los Andes- Applied genomics research group, Vicerrectoria de Investigación y Creación, Universidad de los Andes- Systems and Computing Engineering Department	Carlos Franco-Muñoz; Diego A. Álvarez-Díaz; Diego Andrés Prada; Gerardo Santamaría Jorge Duitama; Héctor Alejandro Ruiz-Moreno; Jhonnatan Reales-González; Jorge Ivan Diaz; Julian Naizaque; Katherine Laiton-Donato; Laura Natalia Gonzalez; Magdalena Wiesner; Marcela Mercado-Reyes; Maria T. Herrera-Sepúlveda; Martha Lucia Ospina Martinez; Mauricio Pacheco-Montealegre; Sheryll Corchuelo; Silvia Restrepo-Restrepo
EPI_ISL_491450, EPI_ISL_491454, EPI_ISL_491456	Hospital San Juan de Dios	Inciensa, Instituto Costarricense de Investigación y Enseñanza en Nutrición y Salud	Adriana Godinez & Melany Calderon; Claudio Soto-Garita; Estela Cordero; Francisco Duarte; Hebleen Brenes
EPI_ISL_419234, EPI_ISL_455336 to 455343, EPI_ISL_578196 to 578201	Hospital San Pedro	Instituto de Salud Carlos III	A. Monzón; Alonso, C.; C. Alonso; Camarero, S.; Casas, I.; Cuesta, I.; F. Casas; González-Esguevillas, M.; I; I. Jiménez; Iglesias-Caballero; Iglesias-Caballero, M.; J.M Azcona; Jiménez, M.; Jiménez, P.; Juliá, M.; M. Blasco; M. Camarero; M. Cuesta; M. González-Esguevillas; M. Molinero Calamita; M. Zaballos; Molinero Calamita, M.; Monzón, S.; P. Jiménez; Pozo, F.; S. Juliá; S. Pozo; S. Varona; Varona, S.; Zaballos, A.
EPI_ISL_539558, EPI_ISL_539561 to 539562, EPI_ISL_862654, EPI_ISL_913021 to 913023, EPI_ISL_913032 to 913035, EPI_ISL_913057	Hospital San Pedro de Alcántara	Instituto de Salud Carlos III	A. Monzón; E. Cerro; F. Casas; I; I. Jiménez; I. Rodríguez, G.; Iglesias-Caballero; J. López; M. Pozo; M. Camarero; M. Cuesta; M. González-Esguevillas; M. Molinero Calamita; M. Zaballos; M.A Cañizares; M.Camarero; P. Jiménez; P. Zaballos; S. Cuesta; S. Juliá; S. Molinero Calamita; S. Pozo; S. Sandonís; S. Varona; V. Vázquez
EPI_ISL_510305 to 510332, EPI_ISL_613410 to 613411	Hospital San Pedro de Alcántara (Cáceres)	SeqCOVID-SPAIN consortium/IBV(CSIC)	Cristina Muñoz Cuevas; Guadalupe Rodriguez Rodriguez and SeqCOVID-SPAIN consortium
EPI_ISL_491438, EPI_ISL_491440, EPI_ISL_491448, EPI_ISL_491452	Hospital San Rafael de Alajuela	Inciensa, Instituto Costarricense de Investigación y Enseñanza en Nutrición y Salud	Adriana Godinez & Melany Calderon; Claudio Soto-Garita; Estela Cordero; Francisco Duarte; Hebleen Brenes
EPI_ISL_527753 to 527754	Hospital San Vicente De Paul	Inciensa, Instituto Costarricense de Investigación y Enseñanza en Nutrición y Salud	Adriana Godinez & Melany Calderon; Claudio Soto-Garita; Estela Cordero; Francisco Duarte; Hebleen Porras
EPI_ISL_434536	Hospital San Vicente de Paul	Inciensa, Instituto Costarricense de Investigación y Enseñanza en Nutrición y Salud	Adriana Godinez & Melany Calderon; Claudio Soto-Garita; Estela Cordero; Francisco Duarte; Hebleen Porras
EPI_ISL_471552, EPI_ISL_523967	Hospital Sancta Maggiore	Instituto Adolfo Lutz, Interdiciplinary Procedures Center, Strategic Laboratory	Claudia Regina Gonçalves; Claudio Tavares Sacchi; Erica Valessa Ramos Gomes
EPI_ISL_603036	Hospital Santa Ana	Instituto Adolfo Lutz, Interdiciplinary Procedures Center, Strategic Laboratory	Claudia Regina Gonçalves; Claudio Tavares Sacchi; Erica Valessa Ramos Gomes; Karoline Rodrigues Campos
EPI_ISL_515527, EPI_ISL_693196	Hospital Santa Clara	Instituto Adolfo Lutz, Interdiciplinary Procedures Center, Strategic Laboratory	Claudia Regina Gonçalves; Claudio Tavares Sacchi; Erica Valessa Ramos Gomes; Karoline Rodrigues Campos
EPI_ISL_693233	Hospital Santa Cruz	Instituto Adolfo Lutz, Interdiciplinary Procedures Center, Strategic Laboratory	Claudia Regina Gonçalves; Claudio Tavares Sacchi; Erica Valessa Ramos Gomes; Karoline Rodrigues Campos
EPI_ISL_693236, EPI_ISL_861646 to 861647, EPI_ISL_861650, EPI_ISL_861654 to 861655	Hospital Santa Marcelina Sao Paulo	Instituto Adolfo Lutz, Interdiciplinary Procedures Center, Strategic Laboratory	Claudia Regina Gonçalves; Claudio Tavares Sacchi; Erica Valessa Ramos Gomes; Karoline Rodrigues Campos
EPI_ISL_802750 to 802776	Hospital Santa María Nai	Instituto de Salud Carlos III	A. Monzón; F. Casas; I; I. Jiménez; Iglesias-Caballero; J. García.; M. Camarero; M. Cuesta; M. González-Esguevillas; M. Molinero Calamita; M. Zaballos; P. Jiménez; S. Juliá; S. Pozo; S. Varona
EPI_ISL_861644	Hospital Santa Virginia	Instituto Adolfo Lutz, Interdiciplinary Procedures Center, Strategic Laboratory	Claudia Regina Gonçalves; Claudio Tavares Sacchi; Erica Valessa Ramos Gomes; Karoline Rodrigues Campos
EPI_ISL_861653	Hospital Santa Virgínia	Instituto Adolfo Lutz, Interdiciplinary Procedures Center, Strategic Laboratory	Claudia Regina Gonçalves; Claudio Tavares Sacchi; Erica Valessa Ramos Gomes; Karoline Rodrigues Campos
EPI_ISL_940619 to 940625	Hospital Sao Joaquim - Beneficiencia Portuguesa	Instituto Adolfo Lutz, Interdiciplinary Procedures Center, Strategic Laboratory	Claudia Regina Gonçalves; Claudio Tavares Sacchi; Erica Valessa Ramos Gomes; Karoline Rodrigues Campos
EPI_ISL_906076 to 906077	Hospital Sao Luiz Sao Caetano	Instituto Adolfo Lutz, Interdiciplinary Procedures Center, Strategic Laboratory	Claudia Regina Gonçalves; Claudio Tavares Sacchi; Erica Valessa Ramos Gomes; Karoline Rodrigues Campos
EPI_ISL_468310	Hospital Sao Paulo de Ensino da UNIFESP	Instituto Adolfo Lutz, Interdiciplinary Procedures Center, Strategic Laboratory	Claudia Regina Gonçalves; Claudio Tavares Sacchi; Erica Valessa Ramos Gomes
EPI_ISL_471545, EPI_ISL_471551, EPI_ISL_515528, EPI_ISL_515545, EPI_ISL_515559 to 515560, EPI_ISL_523969, EPI_ISL_523981, EPI_ISL_523988, EPI_ISL_693201, EPI_ISL_861639			
see above	Hospital Sao Paulo de Ensino da Unifesp	Instituto Adolfo Lutz, Interdiciplinary Procedures Center, Strategic Laboratory	Claudia Regina Gonçalves; Claudio Tavares Sacchi; Erica Valessa Ramos Gomes; Karoline Rodrigues Campos
EPI_ISL_524484	Hospital Schwyz	Institute of Medical Virology, University of Zurich	Alexandra Trkola; Andrea Zbinden; Fiona Steiner; Gabriela Ziltener; Jon Huder; Jürg Böni; Maryam Zaheri; Michael Huber; Patrick Redli; Riccarda Capaul; Stefan Schmutz; Verena Kufner
EPI_ISL_414015 to 414017	Hospital São Joaquim Beneficencia Portuguesa	Instituto Adolfo Lutz, Interdiciplinary Procedures Center, Strategic Laboratory	Audrey Cilli; Carlos Henrique Camargo; Claudia Regina Gonçalves; Claudio Tavares Sacchi; Daniela Bernardes Borges da Silva; Ester Cerdeira Sabino; Fabiana Cristina Pereira dos Santos; Fabiana Cristina Pereira dos Santos Terezinha Maria de Paiva; Maria do Carmo Sampaito Tavares Timenetsky; SimoneGuadagnucci Morillo; Terezinha Maria de Paiva
EPI_ISL_455350 to 455351	Hospital Txagorritxu	Instituto de Salud Carlos III	A. Monzón; C. Gómez; F. Casas; I; I. Jiménez; Iglesias-Caballero; M. Camarero; M. Cuesta; M. González-Esguevillas; M. Molinero Calamita; M. Zaballos; P. Jiménez; S. Juliá; S. Pozo; S. Varona
EPI_ISL_1057039, EPI_ISL_1058151 to 1058158, EPI_ISL_1058167 to 1058175, EPI_ISL_1063791, EPI_ISL_1063795, EPI_ISL_1169865 to 1169866, EPI_ISL_1169868, EPI_ISL_1169875 to 1169880, EPI_ISL_1264385, EPI_ISL_1264387, EPI_ISL_1264390, EPI_ISL_1264393, EPI_ISL_1264395, EPI_ISL_1264397, EPI_ISL_1264400, EPI_ISL_1264403, EPI_ISL_1264405, EPI_ISL_1264408, EPI_ISL_1264410, EPI_ISL_1264412			
see above	Hospital Universitari Arnau de Vilanova	Hospital Universitari Vall d'Hebron - Vall d'Hebron Institut de Recerca	Andrés Antón; Ariadna Rando; Carla Castillo; Cristina Andrés; Damir Garcia-Cehic; Josep F Abril; Josep Quer; Juliana Esperalba; Maria Carmen Martin; Maria Gema Codina; Maria Piñana; Tomàs Pumarola
EPI_ISL_1194101 to 1194102, EPI_ISL_1194104 to 1194106, EPI_ISL_1194108 to 1194109, EPI_ISL_1194111 to 1194112, EPI_ISL_1194114 to 1194115, EPI_ISL_1194117 to 1194118, EPI_ISL_1194120 to 1194121, EPI_ISL_1194123 to 1194124, EPI_ISL_1194126 to 1194127, EPI_ISL_1194129 to 1194130, EPI_ISL_1194132 to 1194133, EPI_ISL_1194135 to 1194136, EPI_ISL_1194138 to 1194139, EPI_ISL_1194141 to 1194142, EPI_ISL_1194144, EPI_ISL_1194146 to 1194147, EPI_ISL_1194497, EPI_ISL_1194499 to 1194500, EPI_ISL_1194502 to 1194503, EPI_ISL_1194505, EPI_ISL_1194507 to 1194508, EPI_ISL_1194510 to 1194511,			

EPI_ISL_1194513 to 1194514			
see above	Hospital Universitari Arnau de Vilanova de Lleida	SeqCOVID-SPAIN consortium/IBV(CSIC)	Alba Bellés Bellés; Albert Bernet Sánchez; Eric López González; Iván Prats Sánchez; Mercè García González and SeqCOVID-SPAIN consortium
EPI_ISL_483059	Hospital Universitari Germans Trias i Pujol	IrsiCaixa AIDS Research Lab	A. Valencia; B. Clotet; C. Avila-Nieto; E. Vidal; G. Cantero; I. Blanco; J. Blanco; J. Carrillo; J. Rodon; J. Segalés; J. Vergara-Alert; M. Noguera-Julián; M. Parera; M. Puig; M.T. Terrón; N. Izquierdo-Useros; S. Cruz; V. Guallar
EPI_ISL_902738 to 902748	Hospital Universitari Germans Trias i Pujol (HUGTiP) / Fundació Lluita contra la SIDA (FLSida)	IrsiCaixa - Can Ruti CovidSeq	2a planta; Badalona Marta Massanella; Bonaventura Clotet; Ester Ballana; Fundació irsiCaixa. Hospital Universitari Germans Trias i Pujol(HUGTiP); Joaquim Segalés; Jorge Carrillo; Julia Blanco; Lidia Ruiz; Nuria Izquierdo; Roger Paredes; maternal Ctra Canyet s/n
EPI_ISL_1012925	Hospital Universitari Germans Trias i Pujol(HUGTiP)/Fundació Lluita contra la SIDA (FLSida)	IrsiCaixa - Can Ruti CovidSeq	Bonaventura Clotet; Bonaventura Clotet Marta Massanella; Ester Ballana; Francesc Catala-Moll; Joaquim Segalés; Jorge Carrillo; Julia Blanco; Lidia Ruiz; Marc Noguera-Julian; Maria Casadellà; Mariona Parera; Nuria Izquierdo; Pilar Armengol; Roger Paredes
EPI_ISL_535575, EPI_ISL_538552, EPI_ISL_832101 to 832104	Hospital Universitari Germans Trias i Pujol(HUGTiP)/Fundació Lluita contra la SIDA (FLSida)	IrsiCaixa AIDS Research Lab	Bonaventura Clotet; Ester Ballana; Joaquim Segalés; Jorge Carrillo; Julia Blanco; Lidia Ruiz; Marc Noguera-Julian; Maria Pilar Armengol; Mariona Parera; Marta Massanella; Nuria Izquierdo; Roger Paredes
EPI_ISL_418268, EPI_ISL_471472, EPI_ISL_510689	Hospital Universitari Germans Trias i Pujol(HUGTiP)/Fundació Lluita contra la SIDA (FLSida)/IRTA-CReSA	IrsiCaixa AIDS Research Lab	Albert Bensaid; Bonaventura Clotet; Joaquim Segalés; Jordi Rodón; Jorge Carrillo; Julia Blanco; Julia Vergara; Lidia Ruiz; Marc Noguera-Julian; Nuria Izquierdo; Pilar Armengol; Roger Paredes
EPI_ISL_516193	Hospital Universitari Germans Trias i Pujol.	IrsiCaixa AIDS Research Lab	Bonaventura Clotet; Ester Ballana; Joaquim Segalés; Jorge Carrillo; Julia Blanco; Lidia Ruiz; Marc Noguera-Julian; Maria Pilar Armengol; Mariona Parera; Marta Massanella; Nuria Izquierdo; Roger Paredes
EPI_ISL_1058159 to 1058160, EPI_ISL_1058181 to 1058189, EPI_ISL_1063790, EPI_ISL_1063792 to 1063794, EPI_ISL_1117529 to 1117549, EPI_ISL_1117551 to 1117588, EPI_ISL_1169853 to 1169859, EPI_ISL_1169861 to 1169862, EPI_ISL_1169869, EPI_ISL_1169881 to 1169882, EPI_ISL_1264414, EPI_ISL_1264416, EPI_ISL_1264418, EPI_ISL_1264421 to 1264422, EPI_ISL_1264425, EPI_ISL_1264427, EPI_ISL_1264430, EPI_ISL_1264433, EPI_ISL_1264435			
see above	Hospital Universitari Joan XXIII de Tarragona	Hospital Universitari Vall d'Hebron - Vall d'Hebron Institut de Recerca	Andrés Antón; Ariadna Rando; Carla Castillo; Cristina Andrés; Damir Garcia-Cehic; Josep F Abril; Josep Quer; Juliana Esperalba; Maria Carmen Martin; Maria Gema Codina; Maria Piñana; Tomàs Pumarola
EPI_ISL_418860 to 418861, EPI_ISL_444971 to 444990, EPI_ISL_447532 to 447533, EPI_ISL_450337, EPI_ISL_458130 to 458132, EPI_ISL_728361 to 728362, EPI_ISL_728364 to 728365, EPI_ISL_819293 to 819363, EPI_ISL_824409 to 824512, EPI_ISL_825326 to 825384, EPI_ISL_825618 to 825631, EPI_ISL_850516, EPI_ISL_918273 to 918338, EPI_ISL_954142 to 954182, EPI_ISL_954747, EPI_ISL_981297 to 981304, EPI_ISL_981308 to 981316, EPI_ISL_981319 to 981322, EPI_ISL_981324 to 981344, EPI_ISL_981350, EPI_ISL_981352, EPI_ISL_981354 to 981367, EPI_ISL_1020140, EPI_ISL_1020551 to 1020581, EPI_ISL_1020583 to 1020591, EPI_ISL_1059022 to 1059041, EPI_ISL_1059044 to 1059065, EPI_ISL_1063998 to 1064004, EPI_ISL_1064006 to 1064007, EPI_ISL_1064009 to 1064010, EPI_ISL_1064012 to 1064015, EPI_ISL_1080811 to 1080852, EPI_ISL_1116556 to 1116591, EPI_ISL_1117550, EPI_ISL_1125255, EPI_ISL_1125257, EPI_ISL_1125259 to 1125260, EPI_ISL_1125262, EPI_ISL_1125264, EPI_ISL_1125266, EPI_ISL_1125268 to 1125269, EPI_ISL_1125271, EPI_ISL_1125273, EPI_ISL_1125275 to 1125276, EPI_ISL_1125278, EPI_ISL_1125280, EPI_ISL_1125282 to 1125283, EPI_ISL_1125285, EPI_ISL_1125287, EPI_ISL_1125289, EPI_ISL_1125291, EPI_ISL_1125293 to 1125294, EPI_ISL_1125296, EPI_ISL_1125298 to 1125299, EPI_ISL_1125302 to 1125303, EPI_ISL_1125305, EPI_ISL_1125307, EPI_ISL_1125309 to 1125310, EPI_ISL_1125312, EPI_ISL_1125314 to 1125315, EPI_ISL_1125317, EPI_ISL_1125319, EPI_ISL_1125321 to 1125322, EPI_ISL_1137608, EPI_ISL_1169860, EPI_ISL_1169863 to 1169864, EPI_ISL_1169867, EPI_ISL_1169872 to 1169874, EPI_ISL_1195208 to 1195271, EPI_ISL_1216811, EPI_ISL_1216813, EPI_ISL_1216815, EPI_ISL_1216818, EPI_ISL_1216820, EPI_ISL_1216823, EPI_ISL_1216825, EPI_ISL_1216827, EPI_ISL_1216829, EPI_ISL_1216832, EPI_ISL_1216835, EPI_ISL_1216837, EPI_ISL_1216840, EPI_ISL_1216842, EPI_ISL_1216845, EPI_ISL_1216847, EPI_ISL_1216850, EPI_ISL_1216853, EPI_ISL_1216855, EPI_ISL_1216858, EPI_ISL_1216861, EPI_ISL_1216863, EPI_ISL_1216866, EPI_ISL_1216868, EPI_ISL_1216871, EPI_ISL_1216874, EPI_ISL_1216877, EPI_ISL_1216879, EPI_ISL_1216881, EPI_ISL_1216884, EPI_ISL_1216886, EPI_ISL_1216889, EPI_ISL_1216892, EPI_ISL_1216895, EPI_ISL_1216898, EPI_ISL_1234498 to 1234524, EPI_ISL_1250738 to 1250813, EPI_ISL_1289403 to 1289434, EPI_ISL_1298430 to 1298433			
see above	Hospital Universitari Vall d'Hebron - Vall d'Hebron Institut de Recerca	Hospital Universitari Vall d'Hebron - Vall d'Hebron Institut de Recerca	Andrés Antón; Ariadna Rando; Aroa Silgado; Carla Castillo; Cristina Andrés; DAmir Garcia-Cehic; Damir Garcia-Cehic; Dàmir Garcia-Cehic; Elena Sulleiro; Josep F Abril; Josep Gregori; Josep Quer; Juliana Esperalba; Maria Carmen Martin; Maria Carmen Martín; Maria Gema Codina; Maria Piñana; Mercedes Guerrero-Murillo; Tomàs Pumarola
EPI_ISL_850511 to 850513, EPI_ISL_850515, EPI_ISL_981270 to 981296	Hospital Universitari de Bellvitge	Hospital Universitari Vall d'Hebron - Vall d'Hebron Institut de Recerca	Andrés Antón; Ariadna Rando; Carla Castillo; Cristina Andrés; Damir Garcia-Cehic; Josep F Abril; Josep Quer; Juliana Esperalba; Maria Carmen Martin; Maria Gema Codina; Maria Piñana; Tomàs Pumarola
EPI_ISL_1060491	Hospital Universitari de La Plana	SeqCOVID-SPAIN consortium/IBV(CSIC)	Alberto Yagüe Muñoz; Helena Buj Jordá; Juan B. Bellido Blasco; Maria Gil Fortuño and SeqCOVID-SPAIN consortium; Noelia Henández Pérez; Óscar Pérez Olaso
EPI_ISL_538003 to 538054, EPI_ISL_539245, EPI_ISL_941165 to 941173	Hospital Universitari i Politècnic La Fe de València	SeqCOVID-SPAIN consortium/IBV(CSIC)	Ana Gil Brusola; Eva González Barbera; Eva González Barberá; José Luis López Hontangas and SeqCOVID-SPAIN consortium; María Dolores Gómez Ruiz; Salvador Giner Almaraz
EPI_ISL_671863	Hospital Universitari i Politècnic la Fe, València, Spain	SeqCOVID-SPAIN consortium/IBV(CSIC)	Manuel Guerreiro; Marta Montero-Alonso; María Dolores Gómez and SeqCOVID-SPAIN consortium
EPI_ISL_421171 to 421180, EPI_ISL_428683 to 428711, EPI_ISL_430718, EPI_ISL_529972 to 530024, EPI_ISL_534327 to 534329, EPI_ISL_737930	Hospital Universitario 12 de Octubre	Hospital Universitario 12 de Octubre	Elias Dahdouh; Esther Viedma; Fernando Lázaro; Irene Múñoz-Gallego; Jesús Mingorance; Juan Carlos Galán; Julio García; Ma Dolores Folgueira; Mª Dolores Folgueira; Natalia Stella; Rafael Cantón; Rafael Delgado; Raul Recio; Raúl Recio; Sara González
EPI_ISL_417952, EPI_ISL_417954, EPI_ISL_417956 to 417957, EPI_ISL_417961, EPI_ISL_417963, EPI_ISL_417967, EPI_ISL_418182			
see above	Hospital Universitario 12 de Octubre	Hospital Universitario La Paz	Elias Dahdouh; Esther Viedma; Fernando Lázaro; Jesús Mingorance; Juan Carlos Galán; Julio García; Mª Dolores Folgueira; Natalia Stella; Rafael Cantón; Rafael Delgado; Sara González
EPI_ISL_1120908 to 1120962, EPI_ISL_1195499 to 1195617, EPI_ISL_1201047, EPI_ISL_1272549 to 1272553, EPI_ISL_1272555 to 1272593, EPI_ISL_1312301 to 1312343	Hospital Universitario 12 de Octubre	SeqCOVID-SPAIN consortium/IBV(CSIC)	Esther Viedma; Irene Múñoz-Gallego; Jennifer Villa; M.ª Dolores Folgueira and SeqCOVID-SPAIN consortiumconsortium; Mar Aguilera; Mª Dolores Folgueira and SeqCOVID-SPAIN consortiumconsortium; Rafael Delgado; Raúl Recio; Sagrario Zurita
EPI_ISL_467092 to 467183, EPI_ISL_500228 to 500286, EPI_ISL_509616 to 509618, EPI_ISL_537874 to 537945, EPI_ISL_538118, EPI_ISL_538231 to 538232	Hospital Universitario Araba. Vitoria-Gasteiz	SeqCOVID-SPAIN consortium/IBV(CSIC)	Amaia Aguirre Quiñonero; Andrés Canut Blasco and SeqCOVID-SPAIN consortium; Andrés Canut Blasco. and SeqCOVID-SPAIN consortium; Carmen Gómez González; Ma Concepción Lecaroz Agara; Ma Rosario Almela Ferrer; Marina Fernández Torres; Mª Concepción Lecaroz Agara; Mª Rosario Almela Ferrer; Silvia Hernáez Crespo
EPI_ISL_452692 to 452786	Hospital Universitario Araba. Vitoria-Gasteiz,	SeqCOVID-SPAIN consortium/IBV(CSIC)	Amaia Aguirre Quiñonero; Andrés Canut Blasco and SeqCOVID-SPAIN consortium; Carmen Gómez González; Maria Concepción Lecaroz Agara; Maria Rosario Almela Ferrer; Marina Fernández Torres; Silvia Hernáez Crespo
EPI_ISL_1017706 to 1017710	Hospital Universitario Hernando Moncaleano Perdomo	Instituto Nacional de Salud- Dirección de Investigación en Salud Pública	Carlos Franco-Muñoz; Diego A. Álvarez-Díaz; Diego Andrés Prada; Gerardo Santamaría; Hector Alejandro Ruiz-Moreno; Jhonnatan Reales-González; Julian Naizaque; Katherine Laiton-Donato; Magdalena Wiesner; Marcela Mercado-Reyes; Maria T. Herrera-Sepúlveda; Martha Lucia Ospina Martinez; Sheryll Corchuelo
EPI_ISL_455326	Hospital Universitario Insular de Gran Canaria	Instituto de Salud Carlos III	A. Hernández; A. Monzón; F. Casas; I; I. Jiménez; Iglesias-Caballero; M. Camarero; M. Cuesta; M. González-Esguevillas; M. Molinero Calamita; M. Zaballos; P. Jiménez; S. Juliá; S. Pozo; S. Varona
EPI_ISL_428674 to 428682, EPI_ISL_430719 to 430721, EPI_ISL_435144	Hospital Universitario La Paz	Hospital Universitario 12 de Octubre	Elias Dahdouh; Esther Viedma; Fernando Lázaro; Jesús Mingorance; Juan Carlos Galán; Julio García; Mª Dolores Folgueira; Natalia Stella; Rafael Cantón; Rafael Delgado; Raúl Recio; Sara González
EPI_ISL_417969, EPI_ISL_417972, EPI_ISL_417975, EPI_ISL_417978, EPI_ISL_530025 to 530093, EPI_ISL_534330 to 534333	Hospital Universitario La Paz	Hospital Universitario La Paz	Elias Dahdouh; Esther Viedma; Fernando Lázaro; Jesús Mingorance; Juan Carlos Galán; Julio García; Ma Dolores Folgueira; María Rodríguez; Mª Dolores Folgueira; Natalia Stella; Rafael Cantón; Rafael Delgado; Raúl Recio; Sara González
EPI_ISL_831021 to 831288, EPI_ISL_1279903 to 1279935	Hospital Universitario La Paz (Madrid)	SeqCOVID-SPAIN consortium/IBV(CSIC)	Elias Dahdouh; Fernando Lázaro-Perona; Jesús Mingorance and SeqCOVID-SPAIN consortium; María Rodríguez-Tejedor
EPI_ISL_871976 to 871977	Hospital Universitario Lozano Bielsa	Instituto de Salud Carlos III	A. Monzón; F. Casas; I. Benito, R.; I. Jiménez; Iglesias-Caballero; M. Camarero; M. Cuesta; M. González-Esguevillas; M. Pozo; M. Zaballos; P. Jiménez; S. Juliá; S. Molinero Calamita; S. Varona
EPI_ISL_537809 to 537810	Hospital Universitario Marqués de Valdecilla (Santander), Servicio de Microbiología	SeqCOVID-SPAIN consortium/IBV(CSIC)	Daniel Pablo Marcos; Jesús Rodríguez Lozano; Jose Manuel Méndez Legaza; María Eliecer Cano García; María Siller Ruiz and SeqCOVID-SPAIN consortium; Mónica Gozalo Margüello
EPI_ISL_538690 to 538747, EPI_ISL_582091 to 582109, EPI_ISL_871879 to 871890, EPI_ISL_871892 to 871907, EPI_ISL_871911, EPI_ISL_1007698 to 1007712, EPI_ISL_1007768 to 1007808, EPI_ISL_1007811 to 1007832, EPI_ISL_1008981 to 1009010, EPI_ISL_1192424 to 1192467			
see above	Hospital Universitario Marqués de Valdecilla - IDIVAL	SeqCOVID-SPAIN consortium/IBV(CSIC)	Daniel Pablo Marcos; Jesús Rodríguez Rodríguez; Jose Manuel Méndez Legaza; María Eliecer Cano García; María Siller Ruiz and SeqCOVID-SPAIN

	(Santander, Cantabria)		consortium; Mónica Gozalo Margüello
EPI_ISL_539503 to 539508, EPI_ISL_539513 to 539519, EPI_ISL_578189, EPI_ISL_578192 to 578193, EPI_ISL_871978, EPI_ISL_871980 to 871983	Hospital Universitario Miguel Servet	Instituto de Salud Carlos III	A. Monzón; A. Rezusta; F. Casas; I; I. Jiménez; I. Rezusta, A.; Iglesias-Caballero; M. Camarero; M. Cuesta; M. González-Esguevillas; M. Molinero Calamita; M. Pozo; M. Zaballos; P. Jiménez; R. Benito; S. Juliá; S. Molinero Calamita; S. Pozo; S. Varona
EPI_ISL_691731	Hospital Universitario Puerta de Hierro	Instituto de Salud Carlos III	A. Monzón; F. Casas; I. Jiménez; I. Velasco, A.; Iglesias-Caballero; M. Camarero; M. Cuesta; M. González-Esguevillas; M. Pozo; M. Zaballos; P. Jiménez; S. Juliá; S. Molinero Calamita; S. Varona
EPI_ISL_452453 to 452471, EPI_ISL_467064 to 467085	Hospital Universitario Puerta del Mar de Cádiz - INIBICA	SeqCOVID-SPAIN consortium/IBV(CSIC)	Fátima-Galán-Sánchez; Manuel Rodríguez-Iglesias and SeqCOVID-SPAIN consortium; Manuel Rodrí-guez-Iglesias and SeqCOVID-SPAIN consortium; Salud Rodríguez-Pallares
EPI_ISL_530094 to 530099	Hospital Universitario Ramón y Cajal	Hospital Universitario 12 de Octubre	Elias Dahdouh; Esther Viedma; Fernando Lázaro; Jesús Mingorance; Juan Carlos Galán; Julio García; Mª Dolores Folgueira; Natalia Stella; Rafael Cantón; Rafael Delgado; Raúl Recio; Sara González
EPI_ISL_417979 to 417981, EPI_ISL_530100 to 530119, EPI_ISL_534334	Hospital Universitario Ramón y Cajal	Hospital Universitario La Paz	Elias Dahdouh; Esther Viedma; Fernando Lázaro; Jesús Mingorance; Juan Carlos Galán; Julio García; Ma Dolores Folgueira; María Rodríguez; Mª Dolores Folgueira; Natalia Stella; Rafael Cantón; Rafael Delgado; Raúl Recio; Sara González
EPI_ISL_691729 to 691730, EPI_ISL_871973, EPI_ISL_871984, EPI_ISL_871991	Hospital Universitario Severo Ochoa	Instituto de Salud Carlos III	A. Monzón; F. Casas; I. García; I. García, M.; I. Jiménez; Iglesias-Caballero; M. Camarero; M. Cuesta; M. González-Esguevillas; M. Pozo; M. Zaballos; M.L.; P. Jiménez; S. Juliá; S. Molinero Calamita; S. Varona
EPI_ISL_862545, EPI_ISL_862549, EPI_ISL_862568, EPI_ISL_913060, EPI_ISL_954782 to 954783, EPI_ISL_954793, EPI_ISL_954799, EPI_ISL_954812			
see above	Hospital Universitario Virgen de la Arrixaca	Instituto de Salud Carlos III	A. Monzón; F. Casas; I; I. Jiménez; I. Moreno, A.; I. Moreno, L.; Iglesias-Caballero; M. Camarero; M. Camarero; M. Cuesta; M. González-Esguevillas; M. Pozo; M. Zaballos; Moreno, A.; P. Jiménez; P. Zaballos; S. Cuesta; S. Juliá; S. Molinero Calamita; S. Pozo; S. Sandonís; S. Varona; V. Vázquez
EPI_ISL_419230	Hospital Universitario Virgen de las Nieves	Instituto de Salud Carlos III	Camarero, S.; Casas, I.; Cuesta, I.; González-Esguevillas, M.; Iglesias-Caballero, M.; Jiménez, M.; Jiménez, P.; Juliá, M.; Molinero Calamita, M.; Monzón, S.; Pozo, F.; Sanbonmatsu, S.; Varona, S.; Zaballos, A.
EPI_ISL_467059 to 467063, EPI_ISL_474797, EPI_ISL_474832 to 474836, EPI_ISL_474841 to 474846, EPI_ISL_474850 to 474852, EPI_ISL_474854 to 474900, EPI_ISL_474907 to 474909, EPI_ISL_474920, EPI_ISL_474922 to 474932, EPI_ISL_474934 to 474939, EPI_ISL_474942 to 474944, EPI_ISL_474948 to 474950, EPI_ISL_474957, EPI_ISL_500160 to 500161, EPI_ISL_500163 to 500165, EPI_ISL_500168 to 500206, EPI_ISL_500208, EPI_ISL_500210 to 500217, EPI_ISL_500220, EPI_ISL_500222 to 500227, EPI_ISL_510424 to 510460, EPI_ISL_538672 to 538689, EPI_ISL_654626, EPI_ISL_1007747 to 1007767, EPI_ISL_1272058, EPI_ISL_1272061 to 1272065, EPI_ISL_1272067 to 1272073			
see above	Hospital Universitario Virgen de las Nieves de Granada-SAS	SeqCOVID-SPAIN consortium/IBV(CSIC)	Irene Pedrosa Corral; José M. Navarro-Marí and SeqCOVID-SPAIN consortium; Mercedes Pérez Ruiz; Sara Sanbonmatsu Gámez
EPI_ISL_468318, EPI_ISL_468321, EPI_ISL_547574	Hospital Universitario da USP	Instituto Adolfo Lutz, Interdiciplinary Procedures Center, Strategic Laboratory	Claudia Regina Gonçalves; Claudio Tavares Sacchi; Erica Valessa Ramos Gomes; Karoline Rodrigues Campos
EPI_ISL_471539	Hospital Universitario da USP Sao Paulo	Instituto Adolfo Lutz, Interdiciplinary Procedures Center, Strategic Laboratory	Claudia Regina Gonçalves; Claudio Tavares Sacchi; Erica Valessa Ramos Gomes
EPI_ISL_534314	Hospital Universitario da USP de SP	Instituto Adolfo Lutz, Interdiciplinary Procedures Center, Strategic Laboratory	Claudia Regina Gonçalves; Claudio Tavares Sacchi; Erica Valessa Ramos Gomes
EPI_ISL_419233, EPI_ISL_455325, EPI_ISL_539532	Hospital Universitario de Canarias	Instituto de Salud Carlos III	A. Monzón; B. Castro; Camarero, S.; Casas, I.; Castro, B.; Cuesta, I.; F. Casas; González-Esguevillas, M.; I; I. Jiménez; Iglesias-Caballero; Iglesias-Caballero, M.; Jiménez, M.; Jiménez, P.; Juliá, M.; M. Camarero; M. Cuesta; M. González-Esguevillas; M. Molinero Calamita; M. Zaballos; Molinero Calamita, M.; Monzón, S.; P. Jiménez; Pozo, F.; S. Juliá; S. Pozo; S. Varona; Varona, S.; Zaballos, A.
EPI_ISL_539520 to 539522, EPI_ISL_691686 to 691699, EPI_ISL_862655 to 862658, EPI_ISL_871995, EPI_ISL_913020, EPI_ISL_913054 to 913056, EPI_ISL_913058			
see above	Hospital Universitario de Ceuta	Instituto de Salud Carlos III	A. Monzón; F. Casas; G. Sánchez; I; I. Hijano, S.; I. Jiménez; I. López, J.; Iglesias-Caballero; J. López; M. Camarero; M. Cuesta; M. González-Esguevillas; M. Molinero Calamita; M. Pozo; M. Zaballos; P. Jiménez; P. Zaballos; S. Cuesta; S. Juliá; S. Molinero Calamita; S. Pozo; S. Sandonís; S. Varona; V. Vázquez
EPI_ISL_467086 to 467091, EPI_ISL_474910 to 474918, EPI_ISL_537691 to 537728, EPI_ISL_537788 to 537808, EPI_ISL_561370 to 561372, EPI_ISL_962929 to 962974, EPI_ISL_1000972 to 1000992			
see above	Hospital Universitario de Gran Canaria Dr. Negrín	SeqCOVID-SPAIN consortium/IBV(CSIC)	Ana Bordes Benítez and SeqCOVID-SPAIN consortium; Francisco J. Chamizo López; M. Carmen Pérez González
EPI_ISL_913040	Hospital Universitario de Guadalajara	Instituto de Salud Carlos III	A. Monzón; F. Casas; I. González, A.; I. Jiménez; Iglesias-Caballero; M. Camarero; P. Zaballos; S. Cuesta; S. Pozo; S. Sandonís; S. Varona; V. Vázquez
EPI_ISL_539246 to 539283, EPI_ISL_941233 to 941239	Hospital Universitario de La Ribera (Alzira, València)	SeqCOVID-SPAIN consortium/IBV(CSIC)	Julia González Cantó and SeqCOVID-SPAIN consortium; Julia González and SeqCOVID-SPAIN consortium; Olalla Martínez Macias
EPI_ISL_1220047	Hospital Universitario de San Rafael de Tunja	Instituto Nacional de Salud- Dirección de Investigación en Salud Pública	Carlos Franco-Muñoz; Diego A. Álvarez-Díaz; Diego Andrés Prada; Gerardo Santamaría; Hector Alejandro Ruiz-Moreno; Jhonnatan Reales-González; Julian Naizaque; Katherine Laiton-Donato; Magdalena Wiesner; Marcela Mercado-Reyes.; Maria T. Herrera-Sepúlveda; Martha Lucia Ospina Martínez; Sheryll Corchuelo
EPI_ISL_1103551, EPI_ISL_1123376	Hospital Valle del Nalón	Laboratorio de Virología HUCA	Abreu F; Alvarez-Arguelles ME; Boga JA; Castelló C; Costales I; Coto E; Gómez de Oña J; Martín-Rodríguez G; Melón S; Perez-Martínez Z; Rojo S; Sandoval M
EPI_ISL_1124087	Hospital Valle del Nalón	Laboratorio de Virología Hospital Universitario Central de Asturias (HUCA)	Abreu F; Alvarez-Arguelles ME; Boga JA; Castelló C; Costales I; Coto E; Gómez de Oña J; Martín-Rodríguez G; Melón S; Perez-Martínez Z; Rojo S; Sandoval M
EPI_ISL_693516, EPI_ISL_693518 to 693520, EPI_ISL_693523 to 693524, EPI_ISL_693534 to 693537, EPI_ISL_693539, EPI_ISL_693630 to 693657	Hospital Vila Franca de Xira	Instituto Nacional de Saude (INSA)	Borges et al
EPI_ISL_906073	Hospital Vila Lobos	Instituto Adolfo Lutz, Interdiciplinary Procedures Center, Strategic Laboratory	Claudia Regina Gonçalves; Claudio Tavares Sacchi; Erica Valessa Ramos Gomes; Karoline Rodrigues Campos
EPI_ISL_455315 to 455322, EPI_ISL_455324, EPI_ISL_539497 to 539498, EPI_ISL_578187 to 578188	Hospital Virgen de las Nieves	Instituto de Salud Carlos III	A. Monzón; F. Casas; I; I. Jiménez; Iglesias-Caballero; J. Lepe; J.M Navarro; M. Camarero; M. Cuesta; M. González-Esguevillas; M. Molinero Calamita; M. Zaballos; P. Jiménez; S. Juliá; S. Pozo; S. Sanbonmatsu; S. Varona
EPI_ISL_455314, EPI_ISL_455323	Hospital Virgen del Rocio	Instituto de Salud Carlos III	A. Monzón; F. Casas; I; I. Jiménez; Iglesias-Caballero; J. Lepe; M. Camarero; M. Cuesta; M. González-Esguevillas; M. Molinero Calamita; M. Zaballos; P. Jiménez; S. Juliá; S. Pozo; S. Varona
EPI_ISL_476203 to 476208, EPI_ISL_476217, EPI_ISL_476222 to 476223, EPI_ISL_476237 to 476277, EPI_ISL_476372 to 476386, EPI_ISL_476428 to 476490	Hospital da Clínicas da Faculdade de Medicina da Universidade de São Paulo	Instituto de Medicina Tropical da Univesidade de São Paulo	Camila Alves Maia da Silva; Carolina S. Lazar; Cecilia Salete Alencar; Darlan da Silva Candido; Erika Regina Manuli; Ester Sabino; Flavia Cristina da Silva Sales; Giula Magalhaes Ferreira; Jaqueline Goes de Jesus; Julien Theze; Mariana Severo Ramundo; Nuno Faria; Samples: Ingra Morales Claro; Sequencing: Ingra Morales Claro; Silvia F. Costa; Thais de Moura Coletti
EPI_ISL_534313	Hospital da Sta Casa de Sto Amaro	Instituto Adolfo Lutz, Interdiciplinary Procedures Center, Strategic Laboratory	Claudia Regina Gonçalves; Claudio Tavares Sacchi; Erica Valessa Ramos Gomes
EPI_ISL_721987 to 722129	Hospital das Clínicas Universidade de São Paulo Medical School	Laboratório de Parasitologia Médica - Instituto de Medicina Tropical - Universidade de São Paulo	Brazil-UK Centre for Arbovirus Discovery Diagnosis Genomics and Epidemiology (CADDE) Genomic Network - Instituto de Medicina Tropical
EPI_ISL_672669, EPI_ISL_672687 to 672700, EPI_ISL_672722 to 672745	Hospital das Clínicas da Faculdade de Medicina da Universidade de São Paulo (HC-FMUSP)	Laboratório de Parasitologia Médica - Instituto de Medicina Tropical - Universidade de São Paulo	Brazil-UK Centre for Arbovirus Discovery Diagnosis Genomics and Epidemiology (CADDE) Genomic Network - Instituto de Medicina Tropical
EPI_ISL_511577 to 511578, EPI_ISL_660443 to	Hospital de Braga	Instituto Nacional de Saude (INSA)	Borges et al

660444			
EPI_ISL_1098843, EPI_ISL_1123364, EPI_ISL_1138407, EPI_ISL_1242040, EPI_ISL_1310782 to 1310783	Hospital de Cabueñes	Laboratorio de Virología HUCA	Abreu F; Alvarez-Arguelles ME; Boga JA; Castelló C; Costales I; Coto E; Gómez de Oña J; Martín-Rodríguez G; Melón S; Perez-Martínez Z; Rojo S; Sandoval M
EPI_ISL_1121318 to 1121319	Hospital de Campanha COVID 19 Caieiras	Instituto Adolfo Lutz, Interdiciplinary Procedures Center, Strategic Laboratory	Caio Vinicius Dias Lopes; Claudia Regina Gonçalves; Claudio Tavares Sacchi; Erica Valessa Ramos Gomes; Karoline Rodrigues Campos
EPI_ISL_836143	Hospital de Campanha COVID-19 de Mairipora	Instituto Adolfo Lutz, Interdiciplinary Procedures Center, Strategic Laboratory	Claudia Regina Gonçalves; Claudio Tavares Sacchi; Erica Valessa Ramos Gomes; Karoline Rodrigues Campos
EPI_ISL_693205	Hospital de Campanha Covid-19 Assis	Instituto Adolfo Lutz, Interdiciplinary Procedures Center, Strategic Laboratory	Claudia Regina Gonçalves; Claudio Tavares Sacchi; Erica Valessa Ramos Gomes; Karoline Rodrigues Campos
EPI_ISL_940612	Hospital de Campanha para Enfrentamento do Coronavirus - Goiania	Instituto Adolfo Lutz, Interdiciplinary Procedures Center, Strategic Laboratory	Claudia Regina Gonçalves; Claudio Tavares Sacchi; Erica Valessa Ramos Gomes; Karoline Rodrigues Campos
EPI_ISL_735396	Hospital de Camplanha COVID 19 SER	Instituto Adolfo Lutz, Interdiciplinary Procedures Center, Strategic Laboratory	Claudia Regina Gonçalves; Claudio Tavares Sacchi; Erica Valessa Ramos Gomes; Karoline Rodrigues Campos
EPI_ISL_882667	Hospital de Clinicas Caieiras	Instituto Adolfo Lutz, Interdiciplinary Procedures Center, Strategic Laboratory	Claudia Regina Gonçalves; Claudio Tavares Sacchi; Erica Valessa Ramos Gomes; Karoline Rodrigues Campos
EPI_ISL_455352 to 455354	Hospital de Cruces	Instituto de Salud Carlos III	A. Monzón; F. Casas; I; I. Jiménez; Iglesias-Caballero; M. Aranzamendi; M. Camarero; M. Cuesta; M. González-Esguevillas; M. Molinero Calamita; M. Zaballos; P. Jiménez; S. Juliá; S. Pozo; S. Varona
EPI_ISL_691685	Hospital de Leon	Instituto de Salud Carlos III	A. Monzón; F. Casas; I. Jiménez; I. Vidan, J.; Iglesias-Caballero; M. Camarero; M. Cuesta; M. González-Esguevillas; M. Pozo; M. Zaballos; P. Jiménez; S. Juliá; S. Molinero Calamita; S. Varona
EPI_ISL_691732, EPI_ISL_871994	Hospital de Madrid	Instituto de Salud Carlos III	A. Monzón; F. Casas; I. Alhambra, A.; I. Jiménez; Iglesias-Caballero; M. Camarero; M. Cuesta; M. González-Esguevillas; M. Pozo; M. Zaballos; P. Jiménez; S. Juliá; S. Molinero Calamita; S. Varona
EPI_ISL_476563, EPI_ISL_476565, EPI_ISL_476567 to 476568, EPI_ISL_476571, EPI_ISL_476573, EPI_ISL_615121	Hospital de Pediatría "Prof. Dr. Juan P Garrahan"	Héritas	Andrea Mangano; Bianca Brun; Cristian Rohr; Dalmacio Pereyra; Fabian Fay; Maria Florencia Fernandez; Martin Vazquez; Mauricio Grisolia; Priscila Aldabe; Roberta Crespo
EPI_ISL_1110214 to 1110239, EPI_ISL_1110248 to 1110287	Hospital de Sagunto	SeqCOVID-SPAIN consortium/IBV(CSIC)	Noelia Lozano Rodríguez; Sandra Moreno and SeqCOVID-SPAIN consortium
EPI_ISL_882661 to 882662	Hospital de Santa Barbara de Goias	Instituto Adolfo Lutz, Interdiciplinary Procedures Center, Strategic Laboratory	Claudia Regina Gonçalves; Claudio Tavares Sacchi; Erica Valessa Ramos Gomes; Karoline Rodrigues Campos
EPI_ISL_414577 to 414578	Hospital de Talca, Chile	Instituto de Salud Publica de Chile	Alejandra Acevedo; Andrés E. Castillo; Bárbara Parra; Carolina Tambley; Gabriel Leal; Gisselle Barra; Jaime Lagos; Javier Tognarelli; Jorge Fernández.; Loredana Arata; Patricia Bustos; Paz Tapia; Rodrigo Fasce; Soledad Ulloa; Winston Andrade
EPI_ISL_850508	Hospital de Tortosa Verge de la Cinta	Hospital Universitari Vall d'Hebron - Vall d'Hebron Institut de Recerca	Andrés Antón; Ariadna Rando; Carla Castillo; Cristina Andrés; Damir Garcia-Cehic; Josep F Abril; Josep Quer; Juliana Esperalba; Maria Carmen Martin; Maria Gema Codina; Maria Piñana; Tomàs Pumarola
EPI_ISL_510246 to 510267, EPI_ISL_671805 to 671833	Hospital de la Santa Creu i Sant Pau. Servicio de Microbiología	SeqCOVID-SPAIN consortium/IBV(CSIC)	Elisenda Miró and SeqCOVID-SPAIN consortium; Ferran Navarro; Núria Rabella
EPI_ISL_541067 to 541081	Hospital de la Santa Creu i Sant Pau. Servicio de Microbiología	SeqCOVID-SPAIN consortium/Institute of Biomedicine of Valencia, IBV-CSIC	Elisenda Miró and SeqCOVID-SPAIN consortium; Ferran Navarro; Núria Rabella
EPI_ISL_1138405	Hospital del Oriente de Asturias	Laboratorio de Virología HUCA	Abreu F; Alvarez-Arguelles ME; Boga JA; Castelló C; Costales I; Coto E; Gómez de Oña J; Martín-Rodríguez G; Melón S; Perez-Martínez Z; Rojo S; Sandoval M
EPI_ISL_534319 to 534320	Hospital do Serv Pub ESTAFCO Morato de Oliveira	Instituto Adolfo Lutz, Interdiciplinary Procedures Center, Strategic Laboratory	Claudia Regina Gonçalves; Claudio Tavares Sacchi; Erica Valessa Ramos Gomes
EPI_ISL_875689	Hospital do Servidor Publico	Instituto Adolfo Lutz, Interdiciplinary Procedures Center, Strategic Laboratory	Claudia Regina Gonçalves; Claudio Tavares Sacchi; Erica Valessa Ramos Gomes; Karoline Rodrigues Campos
EPI_ISL_693199	Hospital do Servidor Publico Estadual Francisco Morato de Oliveira	Instituto Adolfo Lutz, Interdiciplinary Procedures Center, Strategic Laboratory	Claudia Regina Gonçalves; Claudio Tavares Sacchi; Erica Valessa Ramos Gomes; Karoline Rodrigues Campos
EPI_ISL_471548, EPI_ISL_515565, EPI_ISL_523965, EPI_ISL_523972, EPI_ISL_523978, EPI_ISL_523982, EPI_ISL_524470			
see above	Hospital do Servidor Público Estadual Francisco Morato de Oliveira	Instituto Adolfo Lutz, Interdiciplinary Procedures Center, Strategic Laboratory	Claudia Regina Gonçalves; Claudio Tavares Sacchi; Erica Valessa Ramos Gomes
EPI_ISL_527861, EPI_ISL_940609	Hospital e Maternidade Celso Pierro	Instituto Adolfo Lutz, Interdiciplinary Procedures Center, Strategic Laboratory	01246-1301; 355 - Brazil; Av. Dr. Arnaldo; Cerqueira Cesar; Claudia Regina Gonçalves; Claudio Tavares Sacchi; Erica Valessa Ramos Gomes; Karoline Rodrigues Campos; São Paulo - SP
EPI_ISL_861635	Hospital e Maternidade Madre Theodora	Instituto Adolfo Lutz, Interdiciplinary Procedures Center, Strategic Laboratory	Claudia Regina Gonçalves; Claudio Tavares Sacchi; Erica Valessa Ramos Gomes; Karoline Rodrigues Campos
EPI_ISL_693200	Hospital e Maternidade Mairipora	Instituto Adolfo Lutz, Interdiciplinary Procedures Center, Strategic Laboratory	Claudia Regina Gonçalves; Claudio Tavares Sacchi; Erica Valessa Ramos Gomes; Karoline Rodrigues Campos
EPI_ISL_547577	Hospital e Maternidade Nossa Senhora das Graças	Instituto Adolfo Lutz, Interdiciplinary Procedures Center, Strategic Laboratory	Claudia Regina Gonçalves; Claudio Tavares Sacchi; Erica Valessa Ramos Gomes; Karoline Rodrigues Campos
EPI_ISL_693241, EPI_ISL_735425	Hospital e Maternidade Sao Lucas	Instituto Adolfo Lutz, Interdiciplinary Procedures Center, Strategic Laboratory	Claudia Regina Gonçalves; Claudio Tavares Sacchi; Erica Valessa Ramos Gomes; Karoline Rodrigues Campos
EPI_ISL_861657	Hospital e Maternidade Sino Brasileiro	Instituto Adolfo Lutz, Interdiciplinary Procedures Center, Strategic Laboratory	Claudia Regina Gonçalves; Claudio Tavares Sacchi; Erica Valessa Ramos Gomes; Karoline Rodrigues Campos
EPI_ISL_527865	Hospital e Maternidade São Cristóvão	Instituto Adolfo Lutz, Interdiciplinary Procedures Center, Strategic Laboratory	Claudia Regina Gonçalves; Claudio Tavares Sacchi; Erica Valessa Ramos Gomes
EPI_ISL_527868	Hospital e Maternidade do Braz	Instituto Adolfo Lutz, Interdiciplinary Procedures Center, Strategic Laboratory	Claudia Regina Gonçalves; Claudio Tavares Sacchi; Erica Valessa Ramos Gomes
EPI_ISL_861645	Hospital e Pronto Socorro Comunitario Vila Iolanda	Instituto Adolfo Lutz, Interdiciplinary Procedures Center, Strategic Laboratory	Claudia Regina Gonçalves; Claudio Tavares Sacchi; Erica Valessa Ramos Gomes; Karoline Rodrigues Campos
EPI_ISL_534323	Hospital e Pronto Socorro Comunitario Vila Yolanda	Instituto Adolfo Lutz, Interdiciplinary Procedures Center, Strategic Laboratory	Claudia Regina Gonçalves; Claudio Tavares Sacchi; Erica Valessa Ramos Gomes
EPI_ISL_527864	Hospital e Pronto Socorro Comunitário Vila Iolanda	Instituto Adolfo Lutz, Interdiciplinary Procedures Center, Strategic Laboratory	Claudia Regina Gonçalves; Claudio Tavares Sacchi; Erica Valessa Ramos Gomes

EPI_ISL_693195, EPI_ISL_693230, EPI_ISL_693232, EPI_ISL_735412, EPI_ISL_861648, EPI_ISL_861680, EPI_ISL_1121308

see above	Hospital e Pronto Socorro Portinari	Instituto Adolfo Lutz, Interdiciplinary Procedures Center, Strategic Laboratory	Caio Vinicius Dias Lopes; Claudia Regina Gonçalves; Claudio Tavares Sacchi; Erica Valessa Ramos Gomes; Karoline Rodrigues Campos
EPI_ISL_1084635	Hospital for Infectious Diseases, Molecular Diagnostics Laboratory, Warsaw, Poland	0. Laboratory of Recombinant Vaccines, Intercollegiate Faculty of Biotechnology University of Gdansk and Medical University of Gdansk, 2. ViroGenetics - BSL3 Laboratory of Virology, Maopolska Centre of Biotechnology, Jagiellonian University	Andrzej Horban; Aneta Kopacz; Krystyna Bienkowska-Szewczyk; Krzysztof Pyrc; Lukasz Rabalski; Maciej Kosinski; Natalia Mazur-Panasiuk; Piotr Zabek; Tomasz Dyda
EPI_ISL_1084636	Hospital for Infectious Diseases, Molecular Diagnostics Laboratory, Warsaw, Poland	1. Laboratory of Recombinant Vaccines, Intercollegiate Faculty of Biotechnology University of Gdansk and Medical University of Gdansk, 2. ViroGenetics - BSL3 Laboratory of Virology, Maopolska Centre of Biotechnology, Jagiellonian University	Andrzej Horban; Aneta Kopacz; Krystyna Bienkowska-Szewczyk; Krzysztof Pyrc; Lukasz Rabalski; Maciej Kosinski; Natalia Mazur-Panasiuk; Piotr Zabek; Tomasz Dyda
EPI_ISL_1084620	Hospital for Infectious Diseases, Molecular Diagnostics Laboratory, Warsaw, Poland	10. Laboratory of Recombinant Vaccines, Intercollegiate Faculty of Biotechnology University of Gdansk and Medical University of Gdansk, 2. ViroGenetics - BSL3 Laboratory of Virology, Maopolska Centre of Biotechnology, Jagiellonian University	Andrzej Horban; Aneta Kopacz; Krystyna Bienkowska-Szewczyk; Krzysztof Pyrc; Lukasz Rabalski; Maciej Kosinski; Natalia Mazur-Panasiuk; Piotr Zabek; Tomasz Dyda
EPI_ISL_1084642	Hospital for Infectious Diseases, Molecular Diagnostics Laboratory, Warsaw, Poland	11. Laboratory of Recombinant Vaccines, Intercollegiate Faculty of Biotechnology University of Gdansk and Medical University of Gdansk, 2. ViroGenetics - BSL3 Laboratory of Virology, Maopolska Centre of Biotechnology, Jagiellonian University	Andrzej Horban; Aneta Kopacz; Krystyna Bienkowska-Szewczyk; Krzysztof Pyrc; Lukasz Rabalski; Maciej Kosinski; Natalia Mazur-Panasiuk; Piotr Zabek; Tomasz Dyda
EPI_ISL_1084643	Hospital for Infectious Diseases, Molecular Diagnostics Laboratory, Warsaw, Poland	12. Laboratory of Recombinant Vaccines, Intercollegiate Faculty of Biotechnology University of Gdansk and Medical University of Gdansk, 2. ViroGenetics - BSL3 Laboratory of Virology, Maopolska Centre of Biotechnology, Jagiellonian University	Andrzej Horban; Aneta Kopacz; Krystyna Bienkowska-Szewczyk; Krzysztof Pyrc; Lukasz Rabalski; Maciej Kosinski; Natalia Mazur-Panasiuk; Piotr Zabek; Tomasz Dyda
EPI_ISL_1084607	Hospital for Infectious Diseases, Molecular Diagnostics Laboratory, Warsaw, Poland	13. Laboratory of Recombinant Vaccines, Intercollegiate Faculty of Biotechnology University of Gdansk and Medical University of Gdansk, 2. ViroGenetics - BSL3 Laboratory of Virology, Maopolska Centre of Biotechnology, Jagiellonian University	Andrzej Horban; Aneta Kopacz; Krystyna Bienkowska-Szewczyk; Krzysztof Pyrc; Lukasz Rabalski; Maciej Kosinski; Natalia Mazur-Panasiuk; Piotr Zabek; Tomasz Dyda
EPI_ISL_1084644	Hospital for Infectious Diseases, Molecular Diagnostics Laboratory, Warsaw, Poland	14. Laboratory of Recombinant Vaccines, Intercollegiate Faculty of Biotechnology University of Gdansk and Medical University of Gdansk, 2. ViroGenetics - BSL3 Laboratory of Virology, Maopolska Centre of Biotechnology, Jagiellonian University	Andrzej Horban; Aneta Kopacz; Krystyna Bienkowska-Szewczyk; Krzysztof Pyrc; Lukasz Rabalski; Maciej Kosinski; Natalia Mazur-Panasiuk; Piotr Zabek; Tomasz Dyda
EPI_ISL_1084645	Hospital for Infectious Diseases, Molecular Diagnostics Laboratory, Warsaw, Poland	15. Laboratory of Recombinant Vaccines, Intercollegiate Faculty of Biotechnology University of Gdansk and Medical University of Gdansk, 2. ViroGenetics - BSL3 Laboratory of Virology, Maopolska Centre of Biotechnology, Jagiellonian University	Andrzej Horban; Aneta Kopacz; Krystyna Bienkowska-Szewczyk; Krzysztof Pyrc; Lukasz Rabalski; Maciej Kosinski; Natalia Mazur-Panasiuk; Piotr Zabek; Tomasz Dyda
EPI_ISL_1084646	Hospital for Infectious Diseases, Molecular Diagnostics Laboratory, Warsaw, Poland	16. Laboratory of Recombinant Vaccines, Intercollegiate Faculty of Biotechnology University of Gdansk and Medical University of Gdansk, 2. ViroGenetics - BSL3 Laboratory of Virology, Maopolska Centre of Biotechnology, Jagiellonian University	Andrzej Horban; Aneta Kopacz; Krystyna Bienkowska-Szewczyk; Krzysztof Pyrc; Lukasz Rabalski; Maciej Kosinski; Natalia Mazur-Panasiuk; Piotr Zabek; Tomasz Dyda
EPI_ISL_1084647	Hospital for Infectious Diseases, Molecular Diagnostics Laboratory, Warsaw, Poland	17. Laboratory of Recombinant Vaccines, Intercollegiate Faculty of Biotechnology University of Gdansk and Medical University of Gdansk, 2. ViroGenetics - BSL3 Laboratory of Virology, Maopolska Centre of Biotechnology, Jagiellonian University	Andrzej Horban; Aneta Kopacz; Krystyna Bienkowska-Szewczyk; Krzysztof Pyrc; Lukasz Rabalski; Maciej Kosinski; Natalia Mazur-Panasiuk; Piotr Zabek; Tomasz Dyda
EPI_ISL_1084621	Hospital for Infectious Diseases, Molecular Diagnostics Laboratory, Warsaw, Poland	18. Laboratory of Recombinant Vaccines, Intercollegiate Faculty of Biotechnology University of Gdansk and Medical University of Gdansk, 2. ViroGenetics - BSL3 Laboratory of Virology, Maopolska Centre of Biotechnology, Jagiellonian University	Andrzej Horban; Aneta Kopacz; Krystyna Bienkowska-Szewczyk; Krzysztof Pyrc; Lukasz Rabalski; Maciej Kosinski; Natalia Mazur-Panasiuk; Piotr Zabek; Tomasz Dyda
EPI_ISL_1084648	Hospital for Infectious Diseases, Molecular Diagnostics Laboratory, Warsaw, Poland	19. Laboratory of Recombinant Vaccines, Intercollegiate Faculty of Biotechnology University of Gdansk and Medical University of Gdansk, 2. ViroGenetics - BSL3 Laboratory of Virology, Maopolska Centre of Biotechnology, Jagiellonian University	Andrzej Horban; Aneta Kopacz; Krystyna Bienkowska-Szewczyk; Krzysztof Pyrc; Lukasz Rabalski; Maciej Kosinski; Natalia Mazur-Panasiuk; Piotr Zabek; Tomasz Dyda
EPI_ISL_1084616, EPI_ISL_1084627	Hospital for Infectious Diseases, Molecular Diagnostics Laboratory, Warsaw, Poland	2. Laboratory of Recombinant Vaccines, Intercollegiate Faculty of Biotechnology University of Gdansk and Medical University of Gdansk, 2. ViroGenetics - BSL3 Laboratory of Virology, Maopolska Centre of Biotechnology, Jagiellonian University	Andrzej Horban; Aneta Kopacz; Krystyna Bienkowska-Szewczyk; Krzysztof Pyrc; Lukasz Rabalski; Maciej Kosinski; Natalia Mazur-Panasiuk; Piotr Zabek; Tomasz Dyda
EPI_ISL_1084649	Hospital for Infectious Diseases, Molecular Diagnostics Laboratory, Warsaw, Poland	20. Laboratory of Recombinant Vaccines, Intercollegiate Faculty of Biotechnology University of Gdansk and Medical University of Gdansk, 2. ViroGenetics - BSL3 Laboratory of Virology, Maopolska Centre of Biotechnology, Jagiellonian University	Andrzej Horban; Aneta Kopacz; Krystyna Bienkowska-Szewczyk; Krzysztof Pyrc; Lukasz Rabalski; Maciej Kosinski; Natalia Mazur-Panasiuk; Piotr Zabek; Tomasz Dyda
EPI_ISL_1084650	Hospital for Infectious Diseases, Molecular Diagnostics Laboratory, Warsaw, Poland	21. Laboratory of Recombinant Vaccines, Intercollegiate Faculty of Biotechnology University of Gdansk and Medical University of Gdansk, 2. ViroGenetics - BSL3 Laboratory of Virology, Maopolska Centre of Biotechnology, Jagiellonian University	Andrzej Horban; Aneta Kopacz; Krystyna Bienkowska-Szewczyk; Krzysztof Pyrc; Lukasz Rabalski; Maciej Kosinski; Natalia Mazur-Panasiuk; Piotr Zabek; Tomasz Dyda
EPI_ISL_1084622	Hospital for Infectious Diseases, Molecular Diagnostics	22. Laboratory of Recombinant Vaccines, Intercollegiate	Andrzej Horban; Aneta Kopacz; Krystyna Bienkowska-Szewczyk; Krzysztof Pyrc; Lukasz Rabalski; Maciej Kosinski; Natalia Mazur-Panasiuk; Piotr Zabek;

	Laboratory, Warsaw, Poland	Faculty of Biotechnology University of Gdansk and Medical University of Gdansk, 2. ViroGenetics - BSL3 Laboratory of Virology, Maopolska Centre of Biotechnology, Jagiellonian University	Tomasz Dyda
EPI_ISL_1084651	Hospital for Infectious Diseases, Molecular Diagnostics Laboratory, Warsaw, Poland	23. Laboratory of Recombinant Vaccines, Intercollegiate Faculty of Biotechnology University of Gdansk and Medical University of Gdansk, 2. ViroGenetics - BSL3 Laboratory of Virology, Maopolska Centre of Biotechnology, Jagiellonian University	Andrzej Horban; Aneta Kopacz; Krystyna Bienkowska-Szewczyk; Krzysztof Pyrc; Lukasz Rabalski; Maciej Kosinski; Natalia Mazur-Panasiuk; Piotr Zabek; Tomasz Dyda
EPI_ISL_1084652	Hospital for Infectious Diseases, Molecular Diagnostics Laboratory, Warsaw, Poland	24. Laboratory of Recombinant Vaccines, Intercollegiate Faculty of Biotechnology University of Gdansk and Medical University of Gdansk, 2. ViroGenetics - BSL3 Laboratory of Virology, Maopolska Centre of Biotechnology, Jagiellonian University	Andrzej Horban; Aneta Kopacz; Krystyna Bienkowska-Szewczyk; Krzysztof Pyrc; Lukasz Rabalski; Maciej Kosinski; Natalia Mazur-Panasiuk; Piotr Zabek; Tomasz Dyda
EPI_ISL_1084653	Hospital for Infectious Diseases, Molecular Diagnostics Laboratory, Warsaw, Poland	25. Laboratory of Recombinant Vaccines, Intercollegiate Faculty of Biotechnology University of Gdansk and Medical University of Gdansk, 2. ViroGenetics - BSL3 Laboratory of Virology, Maopolska Centre of Biotechnology, Jagiellonian University	Andrzej Horban; Aneta Kopacz; Krystyna Bienkowska-Szewczyk; Krzysztof Pyrc; Lukasz Rabalski; Maciej Kosinski; Natalia Mazur-Panasiuk; Piotr Zabek; Tomasz Dyda
EPI_ISL_1084611	Hospital for Infectious Diseases, Molecular Diagnostics Laboratory, Warsaw, Poland	26. Laboratory of Recombinant Vaccines, Intercollegiate Faculty of Biotechnology University of Gdansk and Medical University of Gdansk, 2. ViroGenetics - BSL3 Laboratory of Virology, Maopolska Centre of Biotechnology, Jagiellonian University	Andrzej Horban; Aneta Kopacz; Krystyna Bienkowska-Szewczyk; Krzysztof Pyrc; Lukasz Rabalski; Maciej Kosinski; Natalia Mazur-Panasiuk; Piotr Zabek; Tomasz Dyda
EPI_ISL_1084617	Hospital for Infectious Diseases, Molecular Diagnostics Laboratory, Warsaw, Poland	27. Laboratory of Recombinant Vaccines, Intercollegiate Faculty of Biotechnology University of Gdansk and Medical University of Gdansk, 2. ViroGenetics - BSL3 Laboratory of Virology, Maopolska Centre of Biotechnology, Jagiellonian University	Andrzej Horban; Aneta Kopacz; Krystyna Bienkowska-Szewczyk; Krzysztof Pyrc; Lukasz Rabalski; Maciej Kosinski; Natalia Mazur-Panasiuk; Piotr Zabek; Tomasz Dyda
EPI_ISL_1084623	Hospital for Infectious Diseases, Molecular Diagnostics Laboratory, Warsaw, Poland	28. Laboratory of Recombinant Vaccines, Intercollegiate Faculty of Biotechnology University of Gdansk and Medical University of Gdansk, 2. ViroGenetics - BSL3 Laboratory of Virology, Maopolska Centre of Biotechnology, Jagiellonian University	Andrzej Horban; Aneta Kopacz; Krystyna Bienkowska-Szewczyk; Krzysztof Pyrc; Lukasz Rabalski; Maciej Kosinski; Natalia Mazur-Panasiuk; Piotr Zabek; Tomasz Dyda
EPI_ISL_1084654	Hospital for Infectious Diseases, Molecular Diagnostics Laboratory, Warsaw, Poland	29. Laboratory of Recombinant Vaccines, Intercollegiate Faculty of Biotechnology University of Gdansk and Medical University of Gdansk, 2. ViroGenetics - BSL3 Laboratory of Virology, Maopolska Centre of Biotechnology, Jagiellonian University	Andrzej Horban; Aneta Kopacz; Krystyna Bienkowska-Szewczyk; Krzysztof Pyrc; Lukasz Rabalski; Maciej Kosinski; Natalia Mazur-Panasiuk; Piotr Zabek; Tomasz Dyda
EPI_ISL_1084634, EPI_ISL_1084637	Hospital for Infectious Diseases, Molecular Diagnostics Laboratory, Warsaw, Poland	3. Laboratory of Recombinant Vaccines, Intercollegiate Faculty of Biotechnology University of Gdansk and Medical University of Gdansk, 2. ViroGenetics - BSL3 Laboratory of Virology, Maopolska Centre of Biotechnology, Jagiellonian University	Andrzej Horban; Aneta Kopacz; Krystyna Bienkowska-Szewczyk; Krzysztof Pyrc; Lukasz Rabalski; Maciej Kosinski; Natalia Mazur-Panasiuk; Piotr Zabek; Tomasz Dyda
EPI_ISL_1084655	Hospital for Infectious Diseases, Molecular Diagnostics Laboratory, Warsaw, Poland	30. Laboratory of Recombinant Vaccines, Intercollegiate Faculty of Biotechnology University of Gdansk and Medical University of Gdansk, 2. ViroGenetics - BSL3 Laboratory of Virology, Maopolska Centre of Biotechnology, Jagiellonian University	Andrzej Horban; Aneta Kopacz; Krystyna Bienkowska-Szewczyk; Krzysztof Pyrc; Lukasz Rabalski; Maciej Kosinski; Natalia Mazur-Panasiuk; Piotr Zabek; Tomasz Dyda
EPI_ISL_1084629	Hospital for Infectious Diseases, Molecular Diagnostics Laboratory, Warsaw, Poland	31. Laboratory of Recombinant Vaccines, Intercollegiate Faculty of Biotechnology University of Gdansk and Medical University of Gdansk, 2. ViroGenetics - BSL3 Laboratory of Virology, Maopolska Centre of Biotechnology, Jagiellonian University	Andrzej Horban; Aneta Kopacz; Krystyna Bienkowska-Szewczyk; Krzysztof Pyrc; Lukasz Rabalski; Maciej Kosinski; Natalia Mazur-Panasiuk; Piotr Zabek; Tomasz Dyda
EPI_ISL_1084656	Hospital for Infectious Diseases, Molecular Diagnostics Laboratory, Warsaw, Poland	32. Laboratory of Recombinant Vaccines, Intercollegiate Faculty of Biotechnology University of Gdansk and Medical University of Gdansk, 2. ViroGenetics - BSL3 Laboratory of Virology, Maopolska Centre of Biotechnology, Jagiellonian University	Andrzej Horban; Aneta Kopacz; Krystyna Bienkowska-Szewczyk; Krzysztof Pyrc; Lukasz Rabalski; Maciej Kosinski; Natalia Mazur-Panasiuk; Piotr Zabek; Tomasz Dyda
EPI_ISL_1084657	Hospital for Infectious Diseases, Molecular Diagnostics Laboratory, Warsaw, Poland	33. Laboratory of Recombinant Vaccines, Intercollegiate Faculty of Biotechnology University of Gdansk and Medical University of Gdansk, 2. ViroGenetics - BSL3 Laboratory of Virology, Maopolska Centre of Biotechnology, Jagiellonian University	Andrzej Horban; Aneta Kopacz; Krystyna Bienkowska-Szewczyk; Krzysztof Pyrc; Lukasz Rabalski; Maciej Kosinski; Natalia Mazur-Panasiuk; Piotr Zabek; Tomasz Dyda
EPI_ISL_1084630	Hospital for Infectious Diseases, Molecular Diagnostics Laboratory, Warsaw, Poland	35. Laboratory of Recombinant Vaccines, Intercollegiate Faculty of Biotechnology University of Gdansk and Medical University of Gdansk, 2. ViroGenetics - BSL3 Laboratory of Virology, Maopolska Centre of Biotechnology, Jagiellonian University	Andrzej Horban; Aneta Kopacz; Krystyna Bienkowska-Szewczyk; Krzysztof Pyrc; Lukasz Rabalski; Maciej Kosinski; Natalia Mazur-Panasiuk; Piotr Zabek; Tomasz Dyda
EPI_ISL_1084624	Hospital for Infectious Diseases, Molecular Diagnostics Laboratory, Warsaw, Poland	37. Laboratory of Recombinant Vaccines, Intercollegiate Faculty of Biotechnology University of Gdansk and Medical University of Gdansk, 2. ViroGenetics - BSL3 Laboratory of Virology, Maopolska Centre of Biotechnology, Jagiellonian University	Andrzej Horban; Aneta Kopacz; Krystyna Bienkowska-Szewczyk; Krzysztof Pyrc; Lukasz Rabalski; Maciej Kosinski; Natalia Mazur-Panasiuk; Piotr Zabek; Tomasz Dyda
EPI_ISL_1084625	Hospital for Infectious Diseases, Molecular Diagnostics Laboratory, Warsaw, Poland	38. Laboratory of Recombinant Vaccines, Intercollegiate Faculty of Biotechnology University of Gdansk and Medical University of Gdansk, 2. ViroGenetics - BSL3 Laboratory of Virology, Maopolska Centre of Biotechnology, Jagiellonian University	Andrzej Horban; Aneta Kopacz; Krystyna Bienkowska-Szewczyk; Krzysztof Pyrc; Lukasz Rabalski; Maciej Kosinski; Natalia Mazur-Panasiuk; Piotr Zabek; Tomasz Dyda

EPI_ISL_1084658	Hospital for Infectious Diseases, Molecular Diagnostics Laboratory, Warsaw, Poland	39. Laboratory of Recombinant Vaccines, Intercollegiate Faculty of Biotechnology University of Gdansk and Medical University of Gdansk, 2. ViroGenetics - BSL3 Laboratory of Virology, Maopolska Centre of Biotechnology, Jagiellonian University	Andrzej Horban; Aneta Kopacz; Krystyna Bienkowska-Szewczyk; Krzysztof Pyrc; Lukasz Rabalski; Maciej Kosinski; Natalia Mazur-Panasiuk; Piotr Zabek; Tomasz Dyda
EPI_ISL_1084615, EPI_ISL_1084638	Hospital for Infectious Diseases, Molecular Diagnostics Laboratory, Warsaw, Poland	4. Laboratory of Recombinant Vaccines, Intercollegiate Faculty of Biotechnology University of Gdansk and Medical University of Gdansk, 2. ViroGenetics - BSL3 Laboratory of Virology, Maopolska Centre of Biotechnology, Jagiellonian University	Andrzej Horban; Aneta Kopacz; Krystyna Bienkowska-Szewczyk; Krzysztof Pyrc; Lukasz Rabalski; Maciej Kosinski; Natalia Mazur-Panasiuk; Piotr Zabek; Tomasz Dyda
EPI_ISL_1084612	Hospital for Infectious Diseases, Molecular Diagnostics Laboratory, Warsaw, Poland	40. Laboratory of Recombinant Vaccines, Intercollegiate Faculty of Biotechnology University of Gdansk and Medical University of Gdansk, 2. ViroGenetics - BSL3 Laboratory of Virology, Maopolska Centre of Biotechnology, Jagiellonian University	Andrzej Horban; Aneta Kopacz; Krystyna Bienkowska-Szewczyk; Krzysztof Pyrc; Lukasz Rabalski; Maciej Kosinski; Natalia Mazur-Panasiuk; Piotr Zabek; Tomasz Dyda
EPI_ISL_1084659	Hospital for Infectious Diseases, Molecular Diagnostics Laboratory, Warsaw, Poland	41. Laboratory of Recombinant Vaccines, Intercollegiate Faculty of Biotechnology University of Gdansk and Medical University of Gdansk, 2. ViroGenetics - BSL3 Laboratory of Virology, Maopolska Centre of Biotechnology, Jagiellonian University	Andrzej Horban; Aneta Kopacz; Krystyna Bienkowska-Szewczyk; Krzysztof Pyrc; Lukasz Rabalski; Maciej Kosinski; Natalia Mazur-Panasiuk; Piotr Zabek; Tomasz Dyda
EPI_ISL_1084626	Hospital for Infectious Diseases, Molecular Diagnostics Laboratory, Warsaw, Poland	42. Laboratory of Recombinant Vaccines, Intercollegiate Faculty of Biotechnology University of Gdansk and Medical University of Gdansk, 2. ViroGenetics - BSL3 Laboratory of Virology, Maopolska Centre of Biotechnology, Jagiellonian University	Andrzej Horban; Aneta Kopacz; Krystyna Bienkowska-Szewczyk; Krzysztof Pyrc; Lukasz Rabalski; Maciej Kosinski; Natalia Mazur-Panasiuk; Piotr Zabek; Tomasz Dyda
EPI_ISL_1084608	Hospital for Infectious Diseases, Molecular Diagnostics Laboratory, Warsaw, Poland	43. Laboratory of Recombinant Vaccines, Intercollegiate Faculty of Biotechnology University of Gdansk and Medical University of Gdansk, 2. ViroGenetics - BSL3 Laboratory of Virology, Maopolska Centre of Biotechnology, Jagiellonian University	Andrzej Horban; Aneta Kopacz; Krystyna Bienkowska-Szewczyk; Krzysztof Pyrc; Lukasz Rabalski; Maciej Kosinski; Natalia Mazur-Panasiuk; Piotr Zabek; Tomasz Dyda
EPI_ISL_1084660	Hospital for Infectious Diseases, Molecular Diagnostics Laboratory, Warsaw, Poland	44. Laboratory of Recombinant Vaccines, Intercollegiate Faculty of Biotechnology University of Gdansk and Medical University of Gdansk, 2. ViroGenetics - BSL3 Laboratory of Virology, Maopolska Centre of Biotechnology, Jagiellonian University	Andrzej Horban; Aneta Kopacz; Krystyna Bienkowska-Szewczyk; Krzysztof Pyrc; Lukasz Rabalski; Maciej Kosinski; Natalia Mazur-Panasiuk; Piotr Zabek; Tomasz Dyda
EPI_ISL_1084661	Hospital for Infectious Diseases, Molecular Diagnostics Laboratory, Warsaw, Poland	45. Laboratory of Recombinant Vaccines, Intercollegiate Faculty of Biotechnology University of Gdansk and Medical University of Gdansk, 2. ViroGenetics - BSL3 Laboratory of Virology, Maopolska Centre of Biotechnology, Jagiellonian University	Andrzej Horban; Aneta Kopacz; Krystyna Bienkowska-Szewczyk; Krzysztof Pyrc; Lukasz Rabalski; Maciej Kosinski; Natalia Mazur-Panasiuk; Piotr Zabek; Tomasz Dyda
EPI_ISL_1084662	Hospital for Infectious Diseases, Molecular Diagnostics Laboratory, Warsaw, Poland	46. Laboratory of Recombinant Vaccines, Intercollegiate Faculty of Biotechnology University of Gdansk and Medical University of Gdansk, 2. ViroGenetics - BSL3 Laboratory of Virology, Maopolska Centre of Biotechnology, Jagiellonian University	Andrzej Horban; Aneta Kopacz; Krystyna Bienkowska-Szewczyk; Krzysztof Pyrc; Lukasz Rabalski; Maciej Kosinski; Natalia Mazur-Panasiuk; Piotr Zabek; Tomasz Dyda
EPI_ISL_1084663	Hospital for Infectious Diseases, Molecular Diagnostics Laboratory, Warsaw, Poland	47. Laboratory of Recombinant Vaccines, Intercollegiate Faculty of Biotechnology University of Gdansk and Medical University of Gdansk, 2. ViroGenetics - BSL3 Laboratory of Virology, Maopolska Centre of Biotechnology, Jagiellonian University	Andrzej Horban; Aneta Kopacz; Krystyna Bienkowska-Szewczyk; Krzysztof Pyrc; Lukasz Rabalski; Maciej Kosinski; Natalia Mazur-Panasiuk; Piotr Zabek; Tomasz Dyda
EPI_ISL_1084664	Hospital for Infectious Diseases, Molecular Diagnostics Laboratory, Warsaw, Poland	48. Laboratory of Recombinant Vaccines, Intercollegiate Faculty of Biotechnology University of Gdansk and Medical University of Gdansk, 2. ViroGenetics - BSL3 Laboratory of Virology, Maopolska Centre of Biotechnology, Jagiellonian University	Andrzej Horban; Aneta Kopacz; Krystyna Bienkowska-Szewczyk; Krzysztof Pyrc; Lukasz Rabalski; Maciej Kosinski; Natalia Mazur-Panasiuk; Piotr Zabek; Tomasz Dyda
EPI_ISL_1084665	Hospital for Infectious Diseases, Molecular Diagnostics Laboratory, Warsaw, Poland	49. Laboratory of Recombinant Vaccines, Intercollegiate Faculty of Biotechnology University of Gdansk and Medical University of Gdansk, 2. ViroGenetics - BSL3 Laboratory of Virology, Maopolska Centre of Biotechnology, Jagiellonian University	Andrzej Horban; Aneta Kopacz; Krystyna Bienkowska-Szewczyk; Krzysztof Pyrc; Lukasz Rabalski; Maciej Kosinski; Natalia Mazur-Panasiuk; Piotr Zabek; Tomasz Dyda
EPI_ISL_1084633, EPI_ISL_1084639	Hospital for Infectious Diseases, Molecular Diagnostics Laboratory, Warsaw, Poland	5. Laboratory of Recombinant Vaccines, Intercollegiate Faculty of Biotechnology University of Gdansk and Medical University of Gdansk, 2. ViroGenetics - BSL3 Laboratory of Virology, Maopolska Centre of Biotechnology, Jagiellonian University	Andrzej Horban; Aneta Kopacz; Krystyna Bienkowska-Szewczyk; Krzysztof Pyrc; Lukasz Rabalski; Maciej Kosinski; Natalia Mazur-Panasiuk; Piotr Zabek; Tomasz Dyda
EPI_ISL_1084666	Hospital for Infectious Diseases, Molecular Diagnostics Laboratory, Warsaw, Poland	50. Laboratory of Recombinant Vaccines, Intercollegiate Faculty of Biotechnology University of Gdansk and Medical University of Gdansk, 2. ViroGenetics - BSL3 Laboratory of Virology, Maopolska Centre of Biotechnology, Jagiellonian University	Andrzej Horban; Aneta Kopacz; Krystyna Bienkowska-Szewczyk; Krzysztof Pyrc; Lukasz Rabalski; Maciej Kosinski; Natalia Mazur-Panasiuk; Piotr Zabek; Tomasz Dyda
EPI_ISL_1084667	Hospital for Infectious Diseases, Molecular Diagnostics Laboratory, Warsaw, Poland	51. Laboratory of Recombinant Vaccines, Intercollegiate Faculty of Biotechnology University of Gdansk and Medical University of Gdansk, 2. ViroGenetics - BSL3 Laboratory of Virology, Maopolska Centre of Biotechnology, Jagiellonian University	Andrzej Horban; Aneta Kopacz; Krystyna Bienkowska-Szewczyk; Krzysztof Pyrc; Lukasz Rabalski; Maciej Kosinski; Natalia Mazur-Panasiuk; Piotr Zabek; Tomasz Dyda
EPI_ISL_1084619	Hospital for Infectious Diseases, Molecular Diagnostics Laboratory, Warsaw, Poland	52. Laboratory of Recombinant Vaccines, Intercollegiate Faculty of Biotechnology University of Gdansk and Medical University of Gdansk, 2. ViroGenetics - BSL3 Laboratory of Virology, Maopolska Centre of Biotechnology, Jagiellonian University	Andrzej Horban; Aneta Kopacz; Krystyna Bienkowska-Szewczyk; Krzysztof Pyrc; Lukasz Rabalski; Maciej Kosinski; Natalia Mazur-Panasiuk; Piotr Zabek; Tomasz Dyda

EPI_ISL_1084668	Hospital for Infectious Diseases, Molecular Diagnostics Laboratory, Warsaw, Poland	53. Laboratory of Recombinant Vaccines, Intercollegiate Faculty of Biotechnology University of Gdansk and Medical University of Gdansk, 2. ViroGenetics - BSL3 Laboratory of Virology, Maopolska Centre of Biotechnology, Jagiellonian University	Andrzej Horban; Aneta Kopacz; Krystyna Bienkowska-Szewczyk; Krzysztof Pyrc; Lukasz Rabalski; Maciej Kosinski; Natalia Mazur-Panasiuk; Piotr Zabek; Tomasz Dyda
EPI_ISL_1084669	Hospital for Infectious Diseases, Molecular Diagnostics Laboratory, Warsaw, Poland	54. Laboratory of Recombinant Vaccines, Intercollegiate Faculty of Biotechnology University of Gdansk and Medical University of Gdansk, 2. ViroGenetics - BSL3 Laboratory of Virology, Maopolska Centre of Biotechnology, Jagiellonian University	Andrzej Horban; Aneta Kopacz; Krystyna Bienkowska-Szewczyk; Krzysztof Pyrc; Lukasz Rabalski; Maciej Kosinski; Natalia Mazur-Panasiuk; Piotr Zabek; Tomasz Dyda
EPI_ISL_1084614	Hospital for Infectious Diseases, Molecular Diagnostics Laboratory, Warsaw, Poland	56. Laboratory of Recombinant Vaccines, Intercollegiate Faculty of Biotechnology University of Gdansk and Medical University of Gdansk, 2. ViroGenetics - BSL3 Laboratory of Virology, Maopolska Centre of Biotechnology, Jagiellonian University	Andrzej Horban; Aneta Kopacz; Krystyna Bienkowska-Szewczyk; Krzysztof Pyrc; Lukasz Rabalski; Maciej Kosinski; Natalia Mazur-Panasiuk; Piotr Zabek; Tomasz Dyda
EPI_ISL_1084670	Hospital for Infectious Diseases, Molecular Diagnostics Laboratory, Warsaw, Poland	58. Laboratory of Recombinant Vaccines, Intercollegiate Faculty of Biotechnology University of Gdansk and Medical University of Gdansk, 2. ViroGenetics - BSL3 Laboratory of Virology, Maopolska Centre of Biotechnology, Jagiellonian University	Andrzej Horban; Aneta Kopacz; Krystyna Bienkowska-Szewczyk; Krzysztof Pyrc; Lukasz Rabalski; Maciej Kosinski; Natalia Mazur-Panasiuk; Piotr Zabek; Tomasz Dyda
EPI_ISL_1084618	Hospital for Infectious Diseases, Molecular Diagnostics Laboratory, Warsaw, Poland	59. Laboratory of Recombinant Vaccines, Intercollegiate Faculty of Biotechnology University of Gdansk and Medical University of Gdansk, 2. ViroGenetics - BSL3 Laboratory of Virology, Maopolska Centre of Biotechnology, Jagiellonian University	Andrzej Horban; Aneta Kopacz; Krystyna Bienkowska-Szewczyk; Krzysztof Pyrc; Lukasz Rabalski; Maciej Kosinski; Natalia Mazur-Panasiuk; Piotr Zabek; Tomasz Dyda
EPI_ISL_1084632, EPI_ISL_1084640	Hospital for Infectious Diseases, Molecular Diagnostics Laboratory, Warsaw, Poland	6. Laboratory of Recombinant Vaccines, Intercollegiate Faculty of Biotechnology University of Gdansk and Medical University of Gdansk, 2. ViroGenetics - BSL3 Laboratory of Virology, Maopolska Centre of Biotechnology, Jagiellonian University	Andrzej Horban; Aneta Kopacz; Krystyna Bienkowska-Szewczyk; Krzysztof Pyrc; Lukasz Rabalski; Maciej Kosinski; Natalia Mazur-Panasiuk; Piotr Zabek; Tomasz Dyda
EPI_ISL_1084671	Hospital for Infectious Diseases, Molecular Diagnostics Laboratory, Warsaw, Poland	60. Laboratory of Recombinant Vaccines, Intercollegiate Faculty of Biotechnology University of Gdansk and Medical University of Gdansk, 2. ViroGenetics - BSL3 Laboratory of Virology, Maopolska Centre of Biotechnology, Jagiellonian University	Andrzej Horban; Aneta Kopacz; Krystyna Bienkowska-Szewczyk; Krzysztof Pyrc; Lukasz Rabalski; Maciej Kosinski; Natalia Mazur-Panasiuk; Piotr Zabek; Tomasz Dyda
EPI_ISL_1084609	Hospital for Infectious Diseases, Molecular Diagnostics Laboratory, Warsaw, Poland	61. Laboratory of Recombinant Vaccines, Intercollegiate Faculty of Biotechnology University of Gdansk and Medical University of Gdansk, 2. ViroGenetics - BSL3 Laboratory of Virology, Maopolska Centre of Biotechnology, Jagiellonian University	Andrzej Horban; Aneta Kopacz; Krystyna Bienkowska-Szewczyk; Krzysztof Pyrc; Lukasz Rabalski; Maciej Kosinski; Natalia Mazur-Panasiuk; Piotr Zabek; Tomasz Dyda
EPI_ISL_1084672	Hospital for Infectious Diseases, Molecular Diagnostics Laboratory, Warsaw, Poland	62. Laboratory of Recombinant Vaccines, Intercollegiate Faculty of Biotechnology University of Gdansk and Medical University of Gdansk, 2. ViroGenetics - BSL3 Laboratory of Virology, Maopolska Centre of Biotechnology, Jagiellonian University	Andrzej Horban; Aneta Kopacz; Krystyna Bienkowska-Szewczyk; Krzysztof Pyrc; Lukasz Rabalski; Maciej Kosinski; Natalia Mazur-Panasiuk; Piotr Zabek; Tomasz Dyda
EPI_ISL_1084613	Hospital for Infectious Diseases, Molecular Diagnostics Laboratory, Warsaw, Poland	63. Laboratory of Recombinant Vaccines, Intercollegiate Faculty of Biotechnology University of Gdansk and Medical University of Gdansk, 2. ViroGenetics - BSL3 Laboratory of Virology, Maopolska Centre of Biotechnology, Jagiellonian University	Andrzej Horban; Aneta Kopacz; Krystyna Bienkowska-Szewczyk; Krzysztof Pyrc; Lukasz Rabalski; Maciej Kosinski; Natalia Mazur-Panasiuk; Piotr Zabek; Tomasz Dyda
EPI_ISL_1084628, EPI_ISL_1084631	Hospital for Infectious Diseases, Molecular Diagnostics Laboratory, Warsaw, Poland	7. Laboratory of Recombinant Vaccines, Intercollegiate Faculty of Biotechnology University of Gdansk and Medical University of Gdansk, 2. ViroGenetics - BSL3 Laboratory of Virology, Maopolska Centre of Biotechnology, Jagiellonian University	Andrzej Horban; Aneta Kopacz; Krystyna Bienkowska-Szewczyk; Krzysztof Pyrc; Lukasz Rabalski; Maciej Kosinski; Natalia Mazur-Panasiuk; Piotr Zabek; Tomasz Dyda
EPI_ISL_1084610	Hospital for Infectious Diseases, Molecular Diagnostics Laboratory, Warsaw, Poland	8. Laboratory of Recombinant Vaccines, Intercollegiate Faculty of Biotechnology University of Gdansk and Medical University of Gdansk, 2. ViroGenetics - BSL3 Laboratory of Virology, Maopolska Centre of Biotechnology, Jagiellonian University	Andrzej Horban; Aneta Kopacz; Krystyna Bienkowska-Szewczyk; Krzysztof Pyrc; Lukasz Rabalski; Maciej Kosinski; Natalia Mazur-Panasiuk; Piotr Zabek; Tomasz Dyda
EPI_ISL_1084641	Hospital for Infectious Diseases, Molecular Diagnostics Laboratory, Warsaw, Poland	9. Laboratory of Recombinant Vaccines, Intercollegiate Faculty of Biotechnology University of Gdansk and Medical University of Gdansk, 2. ViroGenetics - BSL3 Laboratory of Virology, Maopolska Centre of Biotechnology, Jagiellonian University	Andrzej Horban; Aneta Kopacz; Krystyna Bienkowska-Szewczyk; Krzysztof Pyrc; Lukasz Rabalski; Maciej Kosinski; Natalia Mazur-Panasiuk; Piotr Zabek; Tomasz Dyda
EPI_ISL_458023 to 458028, EPI_ISL_498255 to 498261, EPI_ISL_707709 to 707710	Hospital for Tropical Diseases	COVID-19 Network Investigations (CONI) Alliance	Akanitt Jittmittraphap; Angkana Huang; Anthony R. Jones; Arporn Wangwiwatsin; Bhakbhoom Panthan; Chonticha Klungtong; Ekawat Pasomsub; Elizabeth Batty; Insee Sensorn; Janjira Thaipadungpanit; Khajohn Joonsalak; Kingkan Rakmanee; Krittikorn Kumpornsin; Namfon Kotanan; Nantarat Chantawat; Pornsawan Leaungwutiwong; Stefan Fernandez; Thanat Chookajorn; Theerarat Kochakarn; Treewat Watthanachockchai; Viravarn Luvira; Wasun Chantratita; Weena Janwitthayanan; Wudtichai Manasatienkij
EPI_ISL_913025 to 913031, EPI_ISL_913036 to 913037	Hospital los Arcos	Instituto de Salud Carlos III	A. Monzón; F. Casas; I. Cámara, M.; I. Jiménez; Iglesias-Caballero; M. Camarero; P. Zaballos; S. Cuesta; S. Pozo; S. Sandonís; S. Varona; V. Vázquez
EPI_ISL_417486 to 417487, EPI_ISL_420147, EPI_ISL_471173, EPI_ISL_471176, EPI_ISL_481206, EPI_ISL_493384, EPI_ISL_500775, EPI_ISL_500791 to 500792, EPI_ISL_500797 to 500799, EPI_ISL_549036 to 549037, EPI_ISL_549052, EPI_ISL_549167, EPI_ISL_590904 to 590907, EPI_ISL_590915, EPI_ISL_590936 to 590937, EPI_ISL_590984, EPI_ISL_635097, EPI_ISL_635121 to 635123, EPI_ISL_635138, EPI_ISL_635143, EPI_ISL_635161, EPI_ISL_635189, EPI_ISL_668394 to 668395, EPI_ISL_668427 to 668428, EPI_ISL_708055 to 708056, EPI_ISL_708122 to 708125, EPI_ISL_759976, EPI_ISL_775285, EPI_ISL_775371, EPI_ISL_775518 to 775522, EPI_ISL_796664, EPI_ISL_796688, EPI_ISL_796714 to 796716, EPI_ISL_796729, EPI_ISL_813977, EPI_ISL_842645, EPI_ISL_860214, EPI_ISL_860222, EPI_ISL_860236, EPI_ISL_860295, EPI_ISL_906831, EPI_ISL_964984 to 964986, EPI_ISL_1034321, EPI_ISL_1040152, EPI_ISL_1110546 to 1110547, EPI_ISL_1118294, EPI_ISL_1118537, EPI_ISL_1118557, EPI_ISL_1118671 to 1118673, EPI_ISL_1186158 to 1186159, EPI_ISL_1192170, EPI_ISL_1192179 to 1192180, EPI_ISL_1192197, EPI_ISL_1192229, EPI_ISL_1192257 to 1192265, EPI_ISL_1192334, EPI_ISL_1251320 to 1251321, EPI_ISL_1251399, EPI_ISL_1251420, EPI_ISL_1251486 to 1251487, EPI_ISL_1251610, EPI_ISL_1251757, EPI_ISL_1251847, EPI_ISL_1251981 to 1251983, EPI_ISL_1251985, EPI_ISL_1252047, EPI_ISL_1252529 to 1252530, EPI_ISL_1254531, EPI_ISL_1254542, EPI_ISL_1254552, EPI_ISL_1254565, EPI_ISL_1254567, EPI_ISL_1254604, EPI_ISL_1254607, EPI_ISL_1254628, EPI_ISL_1314435 to 1314452, EPI_ISL_1314527, EPI_ISL_1314534			
see above	Hospital of Southern Norway - Kristiansand, Department of Medical Microbiology	Norwegian Institute of Public Health, Department of Virology	Atiya R Ali; Debech Nadia; Engebretsen Serina Beate; Engebretsen Serina Beate Atiya R Ali; Garcia Llorente Ignacio; Hilde Elshaug; Hilde Synnøve Vollan; Hilde Vollan; Ignacio Garcia Llorente; Jon Bråte; Kamilla Heddeland Instefjord; Karoline Bragstad; Kathrine Stene-Johansen; Marie Paulsen

			Madsen; Olav Hungnes; Pedersen Benedikte Nevjen; Rasmus Riis Kopperud; Serina B Engebretsen
EPI_ISL_593915	Hospital, Amneville	National Reference Center for Viruses of Respiratory Infections, Institut Pasteur, Paris	Etienne Simon-Lorière; Fabiana Gambaro; Maud Vanpeene; Sylvie Behillil; Sylvie van der Werf; Vincent Enouf
EPI_ISL_593903	Hospital, Argenteuil	National Reference Center for Viruses of Respiratory Infections, Institut Pasteur, Paris	Etienne Simon-Lorière; Fabiana Gambaro; Maud Vanpeene; Sylvie Behillil; Sylvie van der Werf; Vincent Enouf
EPI_ISL_593910	Hospital, Boulogne-Billancourt	National Reference Center for Viruses of Respiratory Infections, Institut Pasteur, Paris	Etienne Simon-Lorière; Fabiana Gambaro; Maud Vanpeene; Sylvie Behillil; Sylvie van der Werf; Vincent Enouf
EPI_ISL_593921	Hospital, Fameck	National Reference Center for Viruses of Respiratory Infections, Institut Pasteur, Paris	Etienne Simon-Lorière; Fabiana Gambaro; Maud Vanpeene; Sylvie Behillil; Sylvie van der Werf; Vincent Enouf
EPI_ISL_593918	Hospital, Joeuf	National Reference Center for Viruses of Respiratory Infections, Institut Pasteur, Paris	Etienne Simon-Lorière; Fabiana Gambaro; Maud Vanpeene; Sylvie Behillil; Sylvie van der Werf; Vincent Enouf
EPI_ISL_593911	Hospital, Le Chesnay Cédex	National Reference Center for Viruses of Respiratory Infections, Institut Pasteur, Paris	Etienne Simon-Lorière; Fabiana Gambaro; Maud Vanpeene; Sylvie Behillil; Sylvie van der Werf; Vincent Enouf
EPI_ISL_593922 to 593923	Hospital, Maizières les Metz	National Reference Center for Viruses of Respiratory Infections, Institut Pasteur, Paris	Etienne Simon-Lorière; Fabiana Gambaro; Maud Vanpeene; Sylvie Behillil; Sylvie van der Werf; Vincent Enouf
EPI_ISL_593912	Hospital, Marange Silvange	National Reference Center for Viruses of Respiratory Infections, Institut Pasteur, Paris	Etienne Simon-Lorière; Fabiana Gambaro; Maud Vanpeene; Sylvie Behillil; Sylvie van der Werf; Vincent Enouf
EPI_ISL_593914, EPI_ISL_593920	Hospital, Metz	National Reference Center for Viruses of Respiratory Infections, Institut Pasteur, Paris	Etienne Simon-Lorière; Fabiana Gambaro; Maud Vanpeene; Sylvie Behillil; Sylvie van der Werf; Vincent Enouf
EPI_ISL_593917	Hospital, Montigny les Metz	National Reference Center for Viruses of Respiratory Infections, Institut Pasteur, Paris	Etienne Simon-Lorière; Fabiana Gambaro; Maud Vanpeene; Sylvie Behillil; Sylvie van der Werf; Vincent Enouf
EPI_ISL_593919	Hospital, Ottonville	National Reference Center for Viruses of Respiratory Infections, Institut Pasteur, Paris	Etienne Simon-Lorière; Fabiana Gambaro; Maud Vanpeene; Sylvie Behillil; Sylvie van der Werf; Vincent Enouf
EPI_ISL_593916	Hospital, Sarreguemines	National Reference Center for Viruses of Respiratory Infections, Institut Pasteur, Paris	Etienne Simon-Lorière; Fabiana Gambaro; Maud Vanpeene; Sylvie Behillil; Sylvie van der Werf; Vincent Enouf
EPI_ISL_593913	Hospital, Talange	National Reference Center for Viruses of Respiratory Infections, Institut Pasteur, Paris	Etienne Simon-Lorière; Fabiana Gambaro; Maud Vanpeene; Sylvie Behillil; Sylvie van der Werf; Vincent Enouf
EPI_ISL_1233900	Houston Health Department, Disease Prevention and Control	Houston Health Department	Brown, P.; Lara, A.; Penn, R.
EPI_ISL_956340 to 956366, EPI_ISL_968082 to 968088, EPI_ISL_968090 to 968139	Houston Health Department, Disease Prevention and Control	Houston Health Department, Disease Prevention and Control	Brown, P.; Lara, A.; Penn, R.
EPI_ISL_900054, EPI_ISL_911471 to 911493, EPI_ISL_935696 to 935752, EPI_ISL_936182 to 936211, EPI_ISL_940793 to 940820, EPI_ISL_942900 to 942926, EPI_ISL_979379 to 979409, EPI_ISL_1059239 to 1059295, EPI_ISL_1163717 to 1163735, EPI_ISL_1168698 to 1168727, EPI_ISL_1195903 to 1195909, EPI_ISL_1224913 to 1224940, EPI_ISL_1260610 to 1260632, EPI_ISL_1260634 to 1260660			
see above	Houston Health Dept.	Houston Health Dept.	Adolpho Lara; Pamela Brown; Ryker Penn
EPI_ISL_434712 to 435031, EPI_ISL_542487 to 542934, EPI_ISL_543007 to 544954, EPI_ISL_545051 to 545574, EPI_ISL_545581 to 545953, EPI_ISL_545958 to 546435, EPI_ISL_546437 to 546934, EPI_ISL_546937 to 547432, EPI_ISL_780097 to 780374, EPI_ISL_780427 to 780669, EPI_ISL_780671 to 781112, EPI_ISL_781114 to 781177, EPI_ISL_783536 to 783849, EPI_ISL_783851 to 788897, EPI_ISL_789105 to 790566, EPI_ISL_1074302 to 1074621, EPI_ISL_1074624 to 1074742, EPI_ISL_1074744 to 1075266, EPI_ISL_1075268 to 1075349, EPI_ISL_1075351 to 1075643, EPI_ISL_1075645 to 1075656, EPI_ISL_1075658 to 1076022, EPI_ISL_1076024 to 1076305, EPI_ISL_1076307 to 1076352, EPI_ISL_1076357, EPI_ISL_1076360, EPI_ISL_1076365, EPI_ISL_1076368, EPI_ISL_1076372, EPI_ISL_1076375, EPI_ISL_1076378, EPI_ISL_1076382, EPI_ISL_1076386, EPI_ISL_1076389, EPI_ISL_1076393, EPI_ISL_1076396, EPI_ISL_1076400, EPI_ISL_1076403, EPI_ISL_1076406, EPI_ISL_1076410, EPI_ISL_1076413, EPI_ISL_1076416, EPI_ISL_1076420, EPI_ISL_1076423, EPI_ISL_1076427, EPI_ISL_1076430, EPI_ISL_1076434, EPI_ISL_1076437, EPI_ISL_1076440, EPI_ISL_1076444, EPI_ISL_1076447, EPI_ISL_1076451, EPI_ISL_1076454, EPI_ISL_1076458, EPI_ISL_1076461 to 1076536, EPI_ISL_1076538 to 1076688, EPI_ISL_1076690 to 1077347, EPI_ISL_1077350 to 1078588, EPI_ISL_1078591, EPI_ISL_1078595, EPI_ISL_1078598, EPI_ISL_1078602, EPI_ISL_1078605, EPI_ISL_1078609, EPI_ISL_1078612, EPI_ISL_1078615, EPI_ISL_1078619, EPI_ISL_1078622 to 1078980, EPI_ISL_1078982, EPI_ISL_1078985, EPI_ISL_1078990, EPI_ISL_1078994, EPI_ISL_1078998, EPI_ISL_1079001, EPI_ISL_1079005, EPI_ISL_1079009 to 1079157, EPI_ISL_1079160, EPI_ISL_1079164, EPI_ISL_1079167 to 1079338, EPI_ISL_1079340 to 1079341, EPI_ISL_1079343 to 1079351, EPI_ISL_1079354, EPI_ISL_1079357, EPI_ISL_1079361, EPI_ISL_1079365, EPI_ISL_1079367, EPI_ISL_1079369, EPI_ISL_1079372, EPI_ISL_1079374 to 1079375, EPI_ISL_1079377, EPI_ISL_1079379, EPI_ISL_1079381, EPI_ISL_1079384 to 1079385, EPI_ISL_1079387, EPI_ISL_1079389, EPI_ISL_1079393, EPI_ISL_1079396 to 1079397, EPI_ISL_1079399, EPI_ISL_1079401 to 1079435, EPI_ISL_1079437 to 1079688, EPI_ISL_1079691, EPI_ISL_1079694, EPI_ISL_1079698, EPI_ISL_1079702, EPI_ISL_1079704 to 1079889, EPI_ISL_1079892, EPI_ISL_1079895 to 1079991, EPI_ISL_1079997, EPI_ISL_1080000, EPI_ISL_1080002 to 1080045, EPI_ISL_1080047 to 1080333, EPI_ISL_1080335, EPI_ISL_1080340, EPI_ISL_1080345, EPI_ISL_1080349, EPI_ISL_1080352 to 1080374, EPI_ISL_1080377, EPI_ISL_1080383, EPI_ISL_1080392 to 1080432, EPI_ISL_1080858 to 1080930, EPI_ISL_1080934, EPI_ISL_1080938, EPI_ISL_1080942, EPI_ISL_1080946, EPI_ISL_1080951, EPI_ISL_1080955, EPI_ISL_1080960, EPI_ISL_1080964, EPI_ISL_1080968, EPI_ISL_1080973, EPI_ISL_1080977, EPI_ISL_1080980, EPI_ISL_1080985 to 1081044, EPI_ISL_1201898 to 1202565, EPI_ISL_1202567 to 1203815, EPI_ISL_1235691 to 1236444, EPI_ISL_1236446 to 1236900, EPI_ISL_1236902 to 1238194, EPI_ISL_1238196 to 1238303, EPI_ISL_1238305 to 1238765, EPI_ISL_1303551 to 1303775, EPI_ISL_1303777 to 1305773			
see above	Houston Methodist Hospital	Houston Methodist Hospital	Chia-Wei Chou; Concepcion C. Cantu; Daniel Boutz; David W. Bernard; Ghazaleh Eskandari; Hakon Jonsson; Heather Hendrickson; Hoang A. T. Nguyen; Hung-Che Kuo; Ilya J. Finkelstein; J. Hunter Long; James J. Davis; Jason S. McLellan; Jessica Cambric; Jimmy Gollihar; Jule Goike; Kamyab Javanmardi; Kari Stefansson; Kristina Reppond; Layne Pruitt; Madison N. Shyer; Marcus Nguyen; Matthew Ojeda Saavedra; Maulik Shukla; Muthiah Kumaraswami; Paul A. Christensen; Prasanti Yerramilli; Randall J. Olsen; Robert Olson; S. Wesley Long; Sishir Subedi; and James M. Musser
EPI_ISL_450819, EPI_ISL_469059, EPI_ISL_469068	Hovas Askim Familjelakare och BVC	The Public Health Agency of Sweden	Anna Risberg; Anna Wendel; Anna-Malin Linde; Karin Tegmark-Wisell; Maria Lind Karlberg; Mattias Haukland; Mia Brytting; Olov Svartstrom; Oskar Karlsson Lindsjo; Petra Edquist; Reza Advani; Sandra Broddesson; Shamam Muradrasoli; Theresa Enkirch
EPI_ISL_1085592, EPI_ISL_1085605	Hpital Avicenne	Department of Virology, Henri Mondor University Hospital, Assistance Publique Hôpitaux de Paris, Université Paris-Est Créteil, INSERM U955	Alexandre Soulier; Christophe Rodriguez; Elisabeth Trawinski; Guillaume Gricourt; Jean-Michel Pawlotsky; Melissa N'Debi; Slim Fourati; Vanessa Demontant
EPI_ISL_1085552	Hpital Necker-Enfants malades	Department of Virology, Henri Mondor University Hospital, Assistance Publique Hôpitaux de Paris, Université Paris-Est Créteil, INSERM U955	Alexandre Soulier; Christophe Rodriguez; Elisabeth Trawinski; Guillaume Gricourt; Jean-Michel Pawlotsky; Melissa N'Debi; Slim Fourati; Vanessa Demontant
EPI_ISL_1085435, EPI_ISL_1085447, EPI_ISL_1085475, EPI_ISL_1085506, EPI_ISL_1085513, EPI_ISL_1085515 to 1085516	Hpital Piti-Salptrire	Department of Virology, Henri Mondor University Hospital, Assistance Publique Hôpitaux de Paris, Université Paris-Est Créteil, INSERM U955	Alexandre Soulier; Christophe Rodriguez; Elisabeth Trawinski; Guillaume Gricourt; Jean-Michel Pawlotsky; Melissa N'Debi; Slim Fourati; Vanessa Demontant
EPI_ISL_452237, EPI_ISL_452240, EPI_ISL_469062, EPI_ISL_469064 to 469065, EPI_ISL_475513, EPI_ISL_475522 to 475523, EPI_ISL_475525, EPI_ISL_475567			
see above	Huddinge VC	The Public Health Agency of Sweden	Anders Johansson; Anna Risberg; Anna-Malin Linde; Karin Tegmark-Wisell; Maria Lind Karlberg; Mattias Haukland; Mia Brytting; Olov Svartstrom; Oskar Karlsson Lindsjo; Petra Edquist; Reza Advani; Sandra Broddesson; Shamam Muradrasoli; Theresa Enkirch
EPI_ISL_424349, EPI_ISL_437512, EPI_ISL_445088	Human Genetic Research Center, Kawsar Biotech Company	Human Genetic Research Center, Kawsar Biotech Company	Abbasalipour Bashash, M.; Abbasalipour, M.; F. and Haghdoost, Y.; Firoozi, M.; Hosseini; Jadaliha; Kehsvar, Y.; Keshvar, Y.; Khateri, E.; Khosravi; M. and Yazdani, R.; M.A.; Mostafavi Jabbari, S.; Pourtavakoli, S.; R. and Hoseini, F.; Sabeghi, S.; Zeinali; Zeinali, S.
EPI_ISL_645000 to 645109	Human Genome Variation Research Group, Malopolska Centre of Biotechnology	Human Genome Variation Research Group, Malopolska Centre of Biotechnology	Botwina, P.; Branicki, W.; Dabrowska, A.; Foremny, J.; Gromowski, T.; Klajmon, A.; Kopera, K.; Kowalski, M.; Labaj; Marszalek, K.; Owczarek, K.; P.P.; Pisarek, A.; Pospiech, E.; Pyrc, K.; Sanak, M.; Swadzba, J.; Szczepanski, A.
EPI_ISL_454636 to 454641, EPI_ISL_468438 to 468461, EPI_ISL_486280 to 486286, EPI_ISL_513840 to 513855, EPI_ISL_583046 to 583170, EPI_ISL_738504 to 738505, EPI_ISL_738530, EPI_ISL_738539, EPI_ISL_738563, EPI_ISL_738600, EPI_ISL_738647, EPI_ISL_738649, EPI_ISL_738651, EPI_ISL_738742, EPI_ISL_738756, EPI_ISL_738759, EPI_ISL_738778 to 738779, EPI_ISL_738785 to 738786, EPI_ISL_738795, EPI_ISL_738801, EPI_ISL_738806 to 738807, EPI_ISL_738809 to 738810, EPI_ISL_738827, EPI_ISL_738832, EPI_ISL_738844, EPI_ISL_738849, EPI_ISL_738852, EPI_ISL_738854, EPI_ISL_738865, EPI_ISL_738875, EPI_ISL_738879, EPI_ISL_738902, EPI_ISL_738912 to 738913, EPI_ISL_738917 to 738918, EPI_ISL_738923, EPI_ISL_738929, EPI_ISL_738938, EPI_ISL_738947, EPI_ISL_738951, EPI_ISL_738953, EPI_ISL_738958, EPI_ISL_738961, EPI_ISL_738969, EPI_ISL_738980, EPI_ISL_738985, EPI_ISL_738988, EPI_ISL_738999 to 739001, EPI_ISL_739007, EPI_ISL_739011, EPI_ISL_739032, EPI_ISL_739043, EPI_ISL_739051 to 739052, EPI_ISL_739079, EPI_ISL_739086, EPI_ISL_739106, EPI_ISL_739112, EPI_ISL_739114, EPI_ISL_739123, EPI_ISL_739126, EPI_ISL_739131, EPI_ISL_739148, EPI_ISL_739155, EPI_ISL_739159, EPI_ISL_739165, EPI_ISL_739170, EPI_ISL_739177, EPI_ISL_739183, EPI_ISL_739194, EPI_ISL_739198, EPI_ISL_739210, EPI_ISL_739225, EPI_ISL_739228, EPI_ISL_739235, EPI_ISL_739240, EPI_ISL_739242, EPI_ISL_739250, EPI_ISL_739264, EPI_ISL_739269, EPI_ISL_739272, EPI_ISL_739274, EPI_ISL_739287, EPI_ISL_739292, EPI_ISL_739297, EPI_ISL_739299, EPI_ISL_739311, EPI_ISL_739313, EPI_ISL_739318, EPI_ISL_739330, EPI_ISL_739366 to 739367, EPI_ISL_739389, EPI_ISL_739403, EPI_ISL_739405, EPI_ISL_739411, EPI_ISL_739417 to 739418, EPI_ISL_739425, EPI_ISL_739430, EPI_ISL_739433, EPI_ISL_739436,			

EPI_ISL_739442, EPI_ISL_739447, EPI_ISL_739452, EPI_ISL_739461, EPI_ISL_739475, EPI_ISL_739489, EPI_ISL_739499, EPI_ISL_739525, EPI_ISL_739527, EPI_ISL_739530 to 739531, EPI_ISL_739533, EPI_ISL_739542, EPI_ISL_739553, EPI_ISL_739558, EPI_ISL_739571, EPI_ISL_739585, EPI_ISL_739608, EPI_ISL_739611, EPI_ISL_739621, EPI_ISL_739651, EPI_ISL_955496 to 955560, EPI_ISL_955693 to 955777, EPI_ISL_979162 to 979245, EPI_ISL_979710 to 979797, EPI_ISL_1027476, EPI_ISL_1027517, EPI_ISL_1027535 to 1027541, EPI_ISL_1027561, EPI_ISL_1030499, EPI_ISL_1185287, EPI_ISL_1185291 to 1185292, EPI_ISL_1185294, EPI_ISL_1185298, EPI_ISL_1185300 to 1185302, EPI_ISL_1185304, EPI_ISL_1185306, EPI_ISL_1185308 to 1185309, EPI_ISL_1185312, EPI_ISL_1185318 to 1185319, EPI_ISL_1185323 to 1185326, EPI_ISL_1185328 to 1185329, EPI_ISL_1185340, EPI_ISL_1185345 to 1185346, EPI_ISL_1185352 to 1185353, EPI_ISL_1185355, EPI_ISL_1185359 to 1185361, EPI_ISL_1185365, EPI_ISL_1185369, EPI_ISL_1185382, EPI_ISL_1185385, EPI_ISL_1185394 to 1185395, EPI_ISL_1185399, EPI_ISL_1185407 to 1185410, EPI_ISL_1185412 to 1185413, EPI_ISL_1185417, EPI_ISL_1185424 to 1185425, EPI_ISL_1185428, EPI_ISL_1185432, EPI_ISL_1185436, EPI_ISL_1185438 to 1185440, EPI_ISL_1185448, EPI_ISL_1185454 to 1185455, EPI_ISL_1185460, EPI_ISL_1185463, EPI_ISL_1185468 to 1185469, EPI_ISL_1185471, EPI_ISL_1185474, EPI_ISL_1185476, EPI_ISL_1185479, EPI_ISL_1185481, EPI_ISL_1185486 to 1185489, EPI_ISL_1185497 to 1185498, EPI_ISL_1185504 to 1185505, EPI_ISL_1185507, EPI_ISL_1185509, EPI_ISL_1185516, EPI_ISL_1185518 to 1185519, EPI_ISL_1185521, EPI_ISL_1185526, EPI_ISL_1185528, EPI_ISL_1234786, EPI_ISL_1234788 to 1234789, EPI_ISL_1234792 to 1234794, EPI_ISL_1234799, EPI_ISL_1234801, EPI_ISL_1234803, EPI_ISL_1234805, EPI_ISL_1234807, EPI_ISL_1234811, EPI_ISL_1234814, EPI_ISL_1234816 to 1234817, EPI_ISL_1234821 to 1234824, EPI_ISL_1234826, EPI_ISL_1234831, EPI_ISL_1234833, EPI_ISL_1234836, EPI_ISL_1234852 to 1234853, EPI_ISL_1234856, EPI_ISL_1234859, EPI_ISL_1234861, EPI_ISL_1234863, EPI_ISL_1234868, EPI_ISL_1234871, EPI_ISL_1234874, EPI_ISL_1234882, EPI_ISL_1234888 to 1234890, EPI_ISL_1234893, EPI_ISL_1234896, EPI_ISL_1234898, EPI_ISL_1234903, EPI_ISL_1234905 to 1234906, EPI_ISL_1234908, EPI_ISL_1234910 to 1234912, EPI_ISL_1234914, EPI_ISL_1234916, EPI_ISL_1234922, EPI_ISL_1234927 to 1234928, EPI_ISL_1234930 to 1234931, EPI_ISL_1234934, EPI_ISL_1234936, EPI_ISL_1234948 to 1234949, EPI_ISL_1234955, EPI_ISL_1234960, EPI_ISL_1234963, EPI_ISL_1234966, EPI_ISL_1234971, EPI_ISL_1234974, EPI_ISL_1234980 to 1234981, EPI_ISL_1234984, EPI_ISL_1234989, EPI_ISL_1234998, EPI_ISL_1235001 to 1235005, EPI_ISL_1235011 to 1235012, EPI_ISL_1235023, EPI_ISL_1235030, EPI_ISL_1235033, EPI_ISL_1235037, EPI_ISL_1235039 to 1235040, EPI_ISL_1235045 to 1235046, EPI_ISL_1235053			
see above	Humboldt County Public Health Laboratory	Chan-Zuckerberg Biohub	CZB Cliahub Consortium
EPI_ISL_526215 to 526238, EPI_ISL_1041195 to 1041212	Hungarian Defence Forces Military Medical Centre	National Laboratory of Virology, Szentágothai Research Centre	Balázs Somogyi; Bálint Eszenyi; Csaba Pereszlényi; Endre Gábor Tóth; Ferenc Jakab; Gábor Kemenesi; Ágnes Balázs-Nagy
EPI_ISL_1192364 to 1192365	Huntington Memorial Hospital	Los Angeles County PHL	P. Hemarajata et al.
EPI_ISL_529961, EPI_ISL_759956	Husada Utama Hospital	Institute of Tropical Disease, Universitas Airlangga	Aldise M Nastri; Didi Dewanto; Gatot Soegiarto; Jezzy R Dewantari; Kazufumi Shimizu; Krisnoadi Rahardjo; Laksmi Wulandari; Maria I Lusida; Mitsuhiro Nishimura; Resti Yudhawati; Retno A Setyoningrum; Rima R Prasetya; Soetjipto; Yasuko Mori; Yohko K Shimizu
EPI_ISL_450814, EPI_ISL_452241, EPI_ISL_455103	Huslakarna Varmbadhuset Varberg	The Public Health Agency of Sweden	Anna Risberg; Anna-Malin Linde; Johanna Hilmersson; Karin Tegmark-Wisell; Maria Lind Karlberg; Mia Brytting; Olov Svartstrom; Oskar Karlsson Lindsjo; Theresa Enkirch
EPI_ISL_1085921 to 1085924, EPI_ISL_1110182	Hôpital Ambroise-Paré	Department of Virology, Henri Mondor University Hospital, Assistance Publique Hôpitaux de Paris, Université Paris-Est Créteil, INSERM U955	Alexandre Soulier; Christophe Rodriguez; Elisabeth Trawinski; Guillaume Gricourt; Jean-Michel Pawlotsky; Melissa N'Debi; Slim Fourati; Vanessa Demontant
EPI_ISL_1085835, EPI_ISL_1085875, EPI_ISL_1085948	Hôpital Antoine-Béclère	Department of Virology, Henri Mondor University Hospital, Assistance Publique Hôpitaux de Paris, Université Paris-Est Créteil, INSERM U955	Alexandre Soulier; Christophe Rodriguez; Elisabeth Trawinski; Guillaume Gricourt; Jean-Michel Pawlotsky; Melissa N'Debi; Slim Fourati; Vanessa Demontant
EPI_ISL_1085825, EPI_ISL_1085882, EPI_ISL_1085916, EPI_ISL_1085920, EPI_ISL_1085947, EPI_ISL_1085949	Hôpital Armand-Trousseau	Department of Virology, Henri Mondor University Hospital, Assistance Publique Hôpitaux de Paris, Université Paris-Est Créteil, INSERM U955	Alexandre Soulier; Christophe Rodriguez; Elisabeth Trawinski; Guillaume Gricourt; Jean-Michel Pawlotsky; Melissa N'Debi; Slim Fourati; Vanessa Demontant
EPI_ISL_981533 to 981534, EPI_ISL_1085311 to 1085313, EPI_ISL_1085315, EPI_ISL_1085332, EPI_ISL_1085352, EPI_ISL_1085402, EPI_ISL_1085697 to 1085698, EPI_ISL_1085800 to 1085801, EPI_ISL_1085810, EPI_ISL_1085824, EPI_ISL_1085892 to 1085893, EPI_ISL_1085925 to 1085927, EPI_ISL_1110174, EPI_ISL_1110181, EPI_ISL_1110207, EPI_ISL_1110212			
see above	Hôpital Avicenne	Department of Virology, Henri Mondor University Hospital, Assistance Publique Hôpitaux de Paris, Université Paris-Est Créteil, INSERM U955	Alexandre Soulier; Christophe Rodriguez; Elisabeth Trawinski; Guillaume Gricourt; Jean-Michel Pawlotsky; Melissa N'Debi; Slim Fourati; Vanessa Demontant
EPI_ISL_833191 to 833200, EPI_ISL_940159 to 940552, EPI_ISL_1296364 to 1296407	Hôpital Bichat Claude Bernard, Laboratoire de Virologie	IAME UMR1137 Inserm, Université de Paris, Hôpital Bichat	Alexandre Storto; Amélie Recoing; Antoine Bridier; Antoine Bridier-Nahmias; Benoit Visseaux; Charlotte Charpentier; Diane Descamps; Gilles Collin; Lena Daniel; Mélanie Bertine; Nadhira Houhou-Fidouh; Quentin Le Hingrat; Siham Hamri
EPI_ISL_1085852 to 1085853, EPI_ISL_1085936	Hôpital Bichat-Claude Bernard	Department of Virology, Henri Mondor University Hospital, Assistance Publique Hôpitaux de Paris, Université Paris-Est Créteil, INSERM U955	Alexandre Soulier; Christophe Rodriguez; Elisabeth Trawinski; Guillaume Gricourt; Jean-Michel Pawlotsky; Melissa N'Debi; Slim Fourati; Vanessa Demontant
EPI_ISL_1085322, EPI_ISL_1085327, EPI_ISL_1085417, EPI_ISL_1085917, EPI_ISL_1110175, EPI_ISL_1110188	Hôpital Bicêtre	Department of Virology, Henri Mondor University Hospital, Assistance Publique Hôpitaux de Paris, Université Paris-Est Créteil, INSERM U955	Alexandre Soulier; Christophe Rodriguez; Elisabeth Trawinski; Guillaume Gricourt; Jean-Michel Pawlotsky; Melissa N'Debi; Slim Fourati; Vanessa Demontant
EPI_ISL_450304 to 450305, EPI_ISL_450308, EPI_ISL_450310, EPI_ISL_450316, EPI_ISL_465681 to 465682, EPI_ISL_465694, EPI_ISL_465698 to 465699, EPI_ISL_535738, EPI_ISL_535796, EPI_ISL_535819, EPI_ISL_535848, EPI_ISL_535851, EPI_ISL_535853, EPI_ISL_535866, EPI_ISL_535869, EPI_ISL_535874, EPI_ISL_535888, EPI_ISL_535890 to 535891, EPI_ISL_535920 to 535923, EPI_ISL_535927, EPI_ISL_535944, EPI_ISL_535949 to 535950, EPI_ISL_535952 to 535953, EPI_ISL_535957, EPI_ISL_535961 to 535962, EPI_ISL_535966, EPI_ISL_535972 to 535973, EPI_ISL_535983, EPI_ISL_536017 to 536018, EPI_ISL_536020, EPI_ISL_536024 to 536025, EPI_ISL_536075, EPI_ISL_536077 to 536078, EPI_ISL_536081 to 536082, EPI_ISL_536087 to 536091, EPI_ISL_536093 to 536094, EPI_ISL_536100, EPI_ISL_536146 to 536149, EPI_ISL_536185 to 536187, EPI_ISL_536191, EPI_ISL_536194, EPI_ISL_536203, EPI_ISL_536214, EPI_ISL_536223, EPI_ISL_536225, EPI_ISL_536233, EPI_ISL_536247, EPI_ISL_536268 to 536270, EPI_ISL_536279 to 536282, EPI_ISL_536284 to 536285, EPI_ISL_536290, EPI_ISL_536292 to 536294, EPI_ISL_536296 to 536298, EPI_ISL_536313 to 536314, EPI_ISL_536316 to 536317, EPI_ISL_536326 to 536328, EPI_ISL_536331, EPI_ISL_536336, EPI_ISL_536338 to 536343, EPI_ISL_536350, EPI_ISL_536371 to 536373, EPI_ISL_1241283			
see above	Hôpital Charles-LeMoyne	Laboratoire de santé publique du Québec	Guillaume Bourque; Ioannis Ragoussis; Jesse Shapiro; Mark Lathrop and Michel Roger; Mark Lathrop and Michel Roger on behalf of the CoVSeQ research group; Sandrine Moreira
EPI_ISL_535839	Hôpital Cité de la Santé	Laboratoire de santé publique du Québec	Guillaume Bourque; Ioannis Ragoussis; Jesse Shapiro; Mark Lathrop and Michel Roger; Sandrine Moreira
EPI_ISL_1085849, EPI_ISL_1085854, EPI_ISL_1085909	Hôpital Cochin	Department of Virology, Henri Mondor University Hospital, Assistance Publique Hôpitaux de Paris, Université Paris-Est Créteil, INSERM U955	Alexandre Soulier; Christophe Rodriguez; Elisabeth Trawinski; Guillaume Gricourt; Jean-Michel Pawlotsky; Melissa N'Debi; Slim Fourati; Vanessa Demontant
EPI_ISL_1085250, EPI_ISL_1085296 to 1085297	Hôpital Européen G-Pompidou-Broussais	Department of Virology, Henri Mondor University Hospital, Assistance Publique Hôpitaux de Paris, Université Paris-Est Créteil, INSERM U955	Alexandre Soulier; Christophe Rodriguez; Elisabeth Trawinski; Guillaume Gricourt; Jean-Michel Pawlotsky; Melissa N'Debi; Slim Fourati; Vanessa Demontant
EPI_ISL_710532, EPI_ISL_710534, EPI_ISL_710537, EPI_ISL_710540 to 710541, EPI_ISL_710575, EPI_ISL_711057, EPI_ISL_712060			
see above	Hôpital Fattouma-Bourguiba de Monastir	Laboratoire des Procédés de Criblage Moléculaire et Cellulaire-Centre de Biotechnologie de Sfax	A. and Masmoudi, S.; Abdelmoulah, F.; Abid, N.; Ajili, F.; Aouni, M.; Ben Ayed, I.; Bensaid, M.; Chtourou, A.; Elargoubi, A.; Fki-berrajah, L.; Gaaloul, I.; Gargouri, S.; Hammami, A.; Kamoun, S.; Karray Hakim, H.; Kharat, N.; Mastouri, M.; Mhalla, S.; Nabli, A.; Rebai; Smeti, I.; Souissi, A.; Stambouli, N.; Turki, M.
EPI_ISL_535792	Hôpital Fleurimont	Laboratoire de santé publique du Québec	Guillaume Bourque; Ioannis Ragoussis; Jesse Shapiro; Mark Lathrop and Michel Roger; Sandrine Moreira
EPI_ISL_961555 to 961672, EPI_ISL_1164947 to 1164968	Hôpital Georges L. Dumont	National Microbiology Laboratory (NML)	Anna Majer; Anneliese Landgraff; CanCOGeN's metadata curation team; Darian Hole; Elsie Grudeski; Gary Van Domselaar; Grace Seo; Guillaume Desnoyers; Jennifer Tanner; Kirsten Biggar; Madison Chapel; Morag Graham; Natalie Knox; Nathalie Bastien; Philip Mabon; Public Health Agency of Canada CanCOGeN team; Rhiannon Huzarewich; Richard Garceau; Russell Mandes; Shari Tyson; Timothy Booth; Yan Li
EPI_ISL_912635 to 912636, EPI_ISL_912638 to 912642, EPI_ISL_912645 to 912648, EPI_ISL_912650 to 912654, EPI_ISL_912656 to 912659, EPI_ISL_912661, EPI_ISL_912663 to 912666, EPI_ISL_912668 to 912673, EPI_ISL_912677 to 912680, EPI_ISL_912682 to 912686, EPI_ISL_912688 to 912718, EPI_ISL_912720 to 912780, EPI_ISL_912795 to 912890, EPI_ISL_912892 to 912906, EPI_ISL_912908, EPI_ISL_912921 to 912922, EPI_ISL_912927 to 912932, EPI_ISL_912935, EPI_ISL_912937 to 912939, EPI_ISL_912941 to 912960, EPI_ISL_912962 to 913019, EPI_ISL_982116 to 982117, EPI_ISL_982119, EPI_ISL_982121 to 982122, EPI_ISL_982124 to 982127, EPI_ISL_982129, EPI_ISL_982133, EPI_ISL_982135 to 982137, EPI_ISL_982140 to 982141, EPI_ISL_982148, EPI_ISL_982150 to 982153, EPI_ISL_982157, EPI_ISL_982160 to 982161, EPI_ISL_982163, EPI_ISL_982166 to 982168, EPI_ISL_982170, EPI_ISL_982172 to 982173, EPI_ISL_982177, EPI_ISL_982182 to 982205, EPI_ISL_982214, EPI_ISL_982223 to 982225, EPI_ISL_982300, EPI_ISL_982302, EPI_ISL_982304, EPI_ISL_982311, EPI_ISL_982321 to 982343, EPI_ISL_1085231, EPI_ISL_1085233 to 1085234, EPI_ISL_1085239 to 1085240, EPI_ISL_1085243, EPI_ISL_1085247 to 1085249, EPI_ISL_1085252, EPI_ISL_1085256, EPI_ISL_1085267 to 1085285, EPI_ISL_1085303 to 1085310, EPI_ISL_1085314, EPI_ISL_1085335 to 1085336, EPI_ISL_1085343, EPI_ISL_1085346 to 1085347, EPI_ISL_1085354, EPI_ISL_1085356 to 1085357, EPI_ISL_1085360, EPI_ISL_1085372, EPI_ISL_1085384 to 1085391, EPI_ISL_1085396 to 1085400, EPI_ISL_1085440, EPI_ISL_1085451, EPI_ISL_1085453 to 1085454, EPI_ISL_1085473, EPI_ISL_1085500, EPI_ISL_1085504, EPI_ISL_1085526, EPI_ISL_1085533 to 1085534, EPI_ISL_1085540 to 1085541, EPI_ISL_1085544 to 1085545, EPI_ISL_1085553, EPI_ISL_1085556, EPI_ISL_1085559 to 1085560, EPI_ISL_1085568, EPI_ISL_1085571, EPI_ISL_1085583, EPI_ISL_1085593, EPI_ISL_1085600, EPI_ISL_1085607 to 1085608, EPI_ISL_1085610 to 1085613, EPI_ISL_1085616, EPI_ISL_1085621, EPI_ISL_1085626 to 1085628, EPI_ISL_1085637, EPI_ISL_1085640 to 1085641, EPI_ISL_1085646 to 1085651, EPI_ISL_1085668 to 1085675, EPI_ISL_1085706 to 1085735, EPI_ISL_1085767, EPI_ISL_1085784 to 1085789, EPI_ISL_1085792, EPI_ISL_1085795 to 1085796, EPI_ISL_1085808 to 1085809, EPI_ISL_1085811 to 1085815, EPI_ISL_1085831, EPI_ISL_1085837, EPI_ISL_1085843, EPI_ISL_1085856, EPI_ISL_1085858 to 1085868, EPI_ISL_1085895, EPI_ISL_1085898, EPI_ISL_1085901, EPI_ISL_1085903, EPI_ISL_1085905, EPI_ISL_1085908, EPI_ISL_1085928 to 1085929, EPI_ISL_1085941 to 1085946, EPI_ISL_1088552 to 1088556, EPI_ISL_1088558 to 1088559, EPI_ISL_1088561 to 1088563, EPI_ISL_1088565 to 1088566, EPI_ISL_1088575, EPI_ISL_1088577 to 1088578, EPI_ISL_1088580 to 1088584, EPI_ISL_1110139, EPI_ISL_1110156 to 1110157, EPI_ISL_1110161 to 1110162, EPI_ISL_1110171 to 1110173, EPI_ISL_1110177 to 1110179, EPI_ISL_1110190 to 1110191, EPI_ISL_1110203 to 1110204			
see above	Hôpital Henri Mondor	Department of Virology, Henri Mondor University Hospital, Assistance Publique Hôpitaux de Paris, Université Paris-Est Créteil, INSERM U955	Alexandre Soulier; Christophe Rodriguez; Elisabeth Trawinski; Guillaume Gricourt; Jean-Michel Pawlotsky; Melissa N'Debi; Slim Fourati; Vanessa Demontant

EPI_ISL_447654 to 447655, EPI_ISL_447657 to 447733	Hôpital Henri-Mondor Ap-Hp	Hôpital Henri-Mondor Ap-Hp	Canoui-Poitrine, F.; Clinical Study Group, C.; De Prost, N.; Demontant, V.; Deveaux, I.; Fourati, S.; Gricourt, G.; J.-M.; Lamoureux, C.; Lepeule, R.; Mekontso-Dessap, A.; N'debi, M.; P.-L.; Pawlotsky; Picard, O.; Rodriguez, C.; Schmitz, D.; Surgers, L.; Trawinski, E.; Woerther
EPI_ISL_535717, EPI_ISL_535797, EPI_ISL_535912, EPI_ISL_535915, EPI_ISL_536013, EPI_ISL_536029, EPI_ISL_536055, EPI_ISL_536177, EPI_ISL_536180, EPI_ISL_536271, EPI_ISL_536330, EPI_ISL_536379			
see above	Hôpital Honoré-Mercier	Laboratoire de santé publique du Québec	Guillaume Bourque; Ioannis Ragoussis; Jesse Shapiro; Mark Lathrop and Michel Roger; Mark Lathrop and Michel Roger on behalf of the CoVSeQ research group; Sandrine Moreira
EPI_ISL_415650	Hôpital Instruction des Armées - BEGIN	National Reference Center for Viruses of Respiratory Infections, Institut Pasteur, Paris	Angela Brisebarre; Christine Bigaillon; Flora Donati Vincent Enouf; Marion Barbet; Maud Vanpeene; Méline Bizard; Mélnie Albert; Sylvie Behillil; Sylvie van der Werf
EPI_ISL_535745, EPI_ISL_535994	Hôpital Laurentien	Laboratoire de santé publique du Québec	Guillaume Bourque; Ioannis Ragoussis; Jesse Shapiro; Mark Lathrop and Michel Roger; Mark Lathrop and Michel Roger on behalf of the CoVSeQ research group; Sandrine Moreira
EPI_ISL_536108	Hôpital Le Royer	Laboratoire de santé publique du Québec	Guillaume Bourque; Ioannis Ragoussis; Jesse Shapiro; Mark Lathrop and Michel Roger on behalf of the CoVSeQ research group; Sandrine Moreira
EPI_ISL_1085884	Hôpital Louis Mourier	Department of Virology, Henri Mondor University Hospital, Assistance Publique Hôpitaux de Paris, Université Paris-Est Créteil, INSERM U955	Alexandre Soulier; Christophe Rodriguez; Elisabeth Trawinski; Guillaume Gricourt; Jean-Michel Pawlotsky; Melissa N'Debi; Slim Fourati; Vanessa Demontant
EPI_ISL_535755, EPI_ISL_535817, EPI_ISL_535831	Hôpital Maisonneuve-Rosemont	Laboratoire de santé publique du Québec	Guillaume Bourque; Ioannis Ragoussis; Jesse Shapiro; Mark Lathrop and Michel Roger; Sandrine Moreira
EPI_ISL_1013210 to 1013213, EPI_ISL_1013235, EPI_ISL_1013415 to 1013416	Hôpital Necker	National Reference Center for Viruses of Respiratory Infections, Institut Pasteur, Paris	Angela Brisebarre; Camille Capel; Etienne Simon-Lorière; Fourgeaud Jacques; Leruez-Ville Marianne; Marion Barbet; Maud Vanpeene; Méline Bizard; Sylvie Behillil; Sylvie van der Werf; Vincent Enouf
EPI_ISL_443295 to 443299	Hôpital Necker - Enfants - Malades Laboratoire de Virologie	National Reference Center for Viruses of Respiratory Infections, Institut Pasteur, Paris	Angela Brisebarre; Etienne Simon-Lorière; Flora Donati; Marianne Leruez-Ville; Marion Barbet; Maud Vanpeene; Mélanie Albert; Méline Bizard; Sylvie Behillil; Sylvie van der Werf; Vincent Enouf
EPI_ISL_1085846	Hôpital Necker-Enfants malades	Department of Virology, Henri Mondor University Hospital, Assistance Publique Hôpitaux de Paris, Université Paris-Est Créteil, INSERM U955	Alexandre Soulier; Christophe Rodriguez; Elisabeth Trawinski; Guillaume Gricourt; Jean-Michel Pawlotsky; Melissa N'Debi; Slim Fourati; Vanessa Demontant
EPI_ISL_535721, EPI_ISL_535795	Hôpital Notre-Dame	Laboratoire de santé publique du Québec	Guillaume Bourque; Ioannis Ragoussis; Jesse Shapiro; Mark Lathrop and Michel Roger; Mark Lathrop and Michel Roger on behalf of the CoVSeQ research group; Sandrine Moreira
EPI_ISL_900503, EPI_ISL_900535, EPI_ISL_900569 to 900572	Hôpital Paris Saint-Joseph	CNR Virus des Infections Respiratoires - France SUD	Antonin Bal; Bruno Lina; Camille Ciccone; Gregory Destras; Gwendolyne Burfin; Hadrien Règue; Isabelle Garrigue; Laurence Josset; Marie-Edith Lafon; Martine Valette; Pantxika Bellecave; Pascale Trimoulet; Quentin Semanas; Sylvie Larrat
EPI_ISL_450309, EPI_ISL_450314 to 450315, EPI_ISL_450317 to 450318, EPI_ISL_465695 to 465696, EPI_ISL_465700, EPI_ISL_465702 to 465703, EPI_ISL_535718, EPI_ISL_535733 to 535734, EPI_ISL_535763, EPI_ISL_535786, EPI_ISL_535818, EPI_ISL_535840, EPI_ISL_535857, EPI_ISL_535879, EPI_ISL_535908 to 535909, EPI_ISL_535948, EPI_ISL_536021, EPI_ISL_536039, EPI_ISL_536041, EPI_ISL_536092, EPI_ISL_536096, EPI_ISL_536099, EPI_ISL_536103, EPI_ISL_536173 to 536176, EPI_ISL_536178 to 536179, EPI_ISL_536188 to 536189, EPI_ISL_536195 to 536196, EPI_ISL_536229, EPI_ISL_536231, EPI_ISL_536234, EPI_ISL_536236, EPI_ISL_536245, EPI_ISL_536253, EPI_ISL_536276 to 536277, EPI_ISL_536295, EPI_ISL_536300, EPI_ISL_536303, EPI_ISL_536305 to 536309, EPI_ISL_536322 to 536324, EPI_ISL_536332 to 536335, EPI_ISL_536346 to 536347, EPI_ISL_536351 to 536352, EPI_ISL_536360, EPI_ISL_536374 to 536375, EPI_ISL_536380, EPI_ISL_536388 to 536389, EPI_ISL_536391, EPI_ISL_536395, EPI_ISL_536397			
see above	Hôpital Pierre-Boucher	Laboratoire de santé publique du Québec	Guillaume Bourque; Ioannis Ragoussis; Jesse Shapiro; Mark Lathrop and Michel Roger; Mark Lathrop and Michel Roger on behalf of the CoVSeQ research group; Sandrine Moreira
EPI_ISL_535731, EPI_ISL_535752, EPI_ISL_535762, EPI_ISL_535766, EPI_ISL_535770 to 535771, EPI_ISL_535850, EPI_ISL_535861 to 535863, EPI_ISL_535881 to 535883, EPI_ISL_535892 to 535893, EPI_ISL_535936, EPI_ISL_535938, EPI_ISL_535954, EPI_ISL_535956, EPI_ISL_536061 to 536062, EPI_ISL_536064 to 536068, EPI_ISL_536070, EPI_ISL_536083 to 536086, EPI_ISL_536125, EPI_ISL_536128, EPI_ISL_536131, EPI_ISL_536137, EPI_ISL_536139 to 536140, EPI_ISL_536143 to 536144, EPI_ISL_536152, EPI_ISL_536157			
see above	Hôpital Pierre-Le Gardeur	Laboratoire de santé publique du Québec	Guillaume Bourque; Ioannis Ragoussis; Jesse Shapiro; Mark Lathrop and Michel Roger; Mark Lathrop and Michel Roger on behalf of the CoVSeQ research group; Sandrine Moreira
EPI_ISL_912637, EPI_ISL_912643 to 912644, EPI_ISL_912649, EPI_ISL_912655, EPI_ISL_912660, EPI_ISL_912662, EPI_ISL_912667, EPI_ISL_912719, EPI_ISL_912786 to 912794, EPI_ISL_912909 to 912920, EPI_ISL_912923 to 912926, EPI_ISL_912933 to 912934, EPI_ISL_912936, EPI_ISL_912940, EPI_ISL_981532, EPI_ISL_981535 to 981537, EPI_ISL_982156, EPI_ISL_1085238, EPI_ISL_1085242, EPI_ISL_1085244, EPI_ISL_1085246, EPI_ISL_1085257 to 1085258, EPI_ISL_1085323, EPI_ISL_1085338 to 1085341, EPI_ISL_1085345, EPI_ISL_1085348 to 1085350, EPI_ISL_1085353, EPI_ISL_1085381 to 1085382, EPI_ISL_1085678 to 1085696, EPI_ISL_1085699 to 1085705, EPI_ISL_1085736 to 1085766, EPI_ISL_1085770, EPI_ISL_1085772 to 1085782, EPI_ISL_1085802, EPI_ISL_1085819, EPI_ISL_1085821, EPI_ISL_1085823, EPI_ISL_1085827, EPI_ISL_1085833, EPI_ISL_1085841, EPI_ISL_1085873 to 1085874, EPI_ISL_1085888 to 1085891, EPI_ISL_1085894, EPI_ISL_1085900, EPI_ISL_1110163 to 1110170, EPI_ISL_1110183 to 1110184, EPI_ISL_1110186 to 1110187, EPI_ISL_1110210 to 1110211			
see above	Hôpital Pitié-Salpêtrière	Department of Virology, Henri Mondor University Hospital, Assistance Publique Hôpitaux de Paris, Université Paris-Est Créteil, INSERM U955	Alexandre Soulier; Christophe Rodriguez; Elisabeth Trawinski; Guillaume Gricourt; Jean-Michel Pawlotsky; Melissa N'Debi; Slim Fourati; Vanessa Demontant
EPI_ISL_414631 to 414632	Hôpital Robert Debré Laboratoire de Virologie	National Reference Center for Viruses of Respiratory Infections, Institut Pasteur, Paris	Angela Brisebarre; Flora Donati Vincent Enouf; Laurent Andreoletti; Marion Barbet; Maud Vanpeene; Méline Bizard; Mélnie Albert; Sylvie Behillil; Sylvie van der Werf
EPI_ISL_1085375	Hôpital Robert Schuman-Laboratoire	Department of Virology, Henri Mondor University Hospital, Assistance Publique Hôpitaux de Paris, Université Paris-Est Créteil, INSERM U955	Alexandre Soulier; Christophe Rodriguez; Elisabeth Trawinski; Guillaume Gricourt; Jean-Michel Pawlotsky; Melissa N'Debi; Slim Fourati; Vanessa Demontant
EPI_ISL_1085855	Hôpital Robert- Debré	Department of Virology, Henri Mondor University Hospital, Assistance Publique Hôpitaux de Paris, Université Paris-Est Créteil, INSERM U955	Alexandre Soulier; Christophe Rodriguez; Elisabeth Trawinski; Guillaume Gricourt; Jean-Michel Pawlotsky; Melissa N'Debi; Slim Fourati; Vanessa Demontant
EPI_ISL_1085840, EPI_ISL_1085877, EPI_ISL_1085885 to 1085886, EPI_ISL_1085937	Hôpital Saint-Louis	Department of Virology, Henri Mondor University Hospital, Assistance Publique Hôpitaux de Paris, Université Paris-Est Créteil, INSERM U955	Alexandre Soulier; Christophe Rodriguez; Elisabeth Trawinski; Guillaume Gricourt; Jean-Michel Pawlotsky; Melissa N'Debi; Slim Fourati; Vanessa Demontant
EPI_ISL_535810, EPI_ISL_535859 to 535860, EPI_ISL_535897 to 535901, EPI_ISL_535969, EPI_ISL_536003, EPI_ISL_536010 to 536011, EPI_ISL_536056 to 536057, EPI_ISL_536252			
see above	Hôpital Sainte-Croix	Laboratoire de santé publique du Québec	Guillaume Bourque; Ioannis Ragoussis; Jesse Shapiro; Mark Lathrop and Michel Roger; Mark Lathrop and Michel Roger on behalf of the CoVSeQ research group; Sandrine Moreira
EPI_ISL_535963, EPI_ISL_536046	Hôpital d'Alma	Laboratoire de santé publique du Québec	Guillaume Bourque; Ioannis Ragoussis; Jesse Shapiro; Mark Lathrop and Michel Roger on behalf of the CoVSeQ research group; Sandrine Moreira
EPI_ISL_768829	Hôpital d'Instruction des Armées Sainte-Anne	CNR Virus des Infections Respiratoires - France SUD	Antonin Bal; Bruno Lina; Gregory Destras; Gwendolyne Burfin; Hadrien Règue; Laurence Josset; Martine Valette; Quentin Semanas
EPI_ISL_536265	Hôpital de Chandler	Laboratoire de santé publique du Québec	Guillaume Bourque; Ioannis Ragoussis; Jesse Shapiro; Mark Lathrop and Michel Roger on behalf of the CoVSeQ research group; Sandrine Moreira
EPI_ISL_535829, EPI_ISL_535905 to 535906, EPI_ISL_535964 to 535965, EPI_ISL_536019, EPI_ISL_536107	Hôpital de Chicoutimi	Laboratoire de santé publique du Québec	Guillaume Bourque; Ioannis Ragoussis; Jesse Shapiro; Mark Lathrop and Michel Roger; Mark Lathrop and Michel Roger on behalf of the CoVSeQ research group; Sandrine Moreira
EPI_ISL_465691, EPI_ISL_535858, EPI_ISL_536266, EPI_ISL_536311, EPI_ISL_536384	Hôpital de Gatineau	Laboratoire de santé publique du Québec	Guillaume Bourque; Ioannis Ragoussis; Jesse Shapiro; Mark Lathrop and Michel Roger on behalf of the CoVSeQ research group; Sandrine Moreira
EPI_ISL_465686, EPI_ISL_535794, EPI_ISL_535809, EPI_ISL_535886 to 535887, EPI_ISL_535911, EPI_ISL_535989, EPI_ISL_535993, EPI_ISL_535995, EPI_ISL_536051, EPI_ISL_536117 to 536118, EPI_ISL_536122, EPI_ISL_536124, EPI_ISL_536127, EPI_ISL_536132, EPI_ISL_536135 to 536136, EPI_ISL_536168 to 536169, EPI_ISL_536207 to 536210, EPI_ISL_536230, EPI_ISL_536238 to 536239, EPI_ISL_536242, EPI_ISL_536244, EPI_ISL_536267, EPI_ISL_536275, EPI_ISL_536291, EPI_ISL_536312, EPI_ISL_536363 to 536364, EPI_ISL_536367, EPI_ISL_536385 to 536387, EPI_ISL_536392 to 536394, EPI_ISL_536396			
see above	Hôpital de Hull	Laboratoire de santé publique du Québec	Guillaume Bourque; Ioannis Ragoussis; Jesse Shapiro; Mark Lathrop and Michel Roger; Mark Lathrop and Michel Roger on behalf of the CoVSeQ research group; Sandrine Moreira
EPI_ISL_535744	Hôpital de Lasalle	Laboratoire de santé publique du Québec	Guillaume Bourque; Ioannis Ragoussis; Jesse Shapiro; Mark Lathrop and Michel Roger; Sandrine Moreira

EPI_ISL_465683 to 465684, EPI_ISL_535885, EPI_ISL_536111 to 536112, EPI_ISL_536262, EPI_ISL_536289	Hôpital de Maria	Laboratoire de santé publique du Québec	Guillaume Bourque; Ioannis Ragoussis; Jesse Shapiro; Mark Lathrop and Michel Roger on behalf of the CoVSeQ research group; Sandrine Moreira
EPI_ISL_535945	Hôpital de Mont-Laurier	Laboratoire de santé publique du Québec	Guillaume Bourque; Ioannis Ragoussis; Jesse Shapiro; Mark Lathrop and Michel Roger; Sandrine Moreira
EPI_ISL_536171	Hôpital de Papineau	Laboratoire de santé publique du Québec	Guillaume Bourque; Ioannis Ragoussis; Jesse Shapiro; Mark Lathrop and Michel Roger on behalf of the CoVSeQ research group; Sandrine Moreira
EPI_ISL_535985 to 535987	Hôpital de Rouyn-Noranda	Laboratoire de santé publique du Québec	Guillaume Bourque; Ioannis Ragoussis; Jesse Shapiro; Mark Lathrop and Michel Roger on behalf of the CoVSeQ research group; Sandrine Moreira
EPI_ISL_535793, EPI_ISL_535865, EPI_ISL_535867, EPI_ISL_535878, EPI_ISL_535889, EPI_ISL_535924, EPI_ISL_535926, EPI_ISL_535929 to 535930, EPI_ISL_535941 to 535943, EPI_ISL_535947, EPI_ISL_535951, EPI_ISL_535982, EPI_ISL_536008, EPI_ISL_536049, EPI_ISL_536058, EPI_ISL_536069, EPI_ISL_536071 to 536072			
see above	Hôpital de Saint-Eustache	Laboratoire de santé publique du Québec	Guillaume Bourque; Ioannis Ragoussis; Jesse Shapiro; Mark Lathrop and Michel Roger; Mark Lathrop and Michel Roger on behalf of the CoVSeQ research group; Sandrine Moreira
EPI_ISL_535716, EPI_ISL_535723, EPI_ISL_535728, EPI_ISL_535856	Hôpital de Verdun	Laboratoire de santé publique du Québec	Guillaume Bourque; Ioannis Ragoussis; Jesse Shapiro; Mark Lathrop and Michel Roger; Mark Lathrop and Michel Roger on behalf of the CoVSeQ research group; Sandrine Moreira
EPI_ISL_535739	Hôpital de l'Enfant-Jésus	Laboratoire de santé publique du Québec	Guillaume Bourque; Ioannis Ragoussis; Jesse Shapiro; Mark Lathrop and Michel Roger; Sandrine Moreira
EPI_ISL_535914, EPI_ISL_535937, EPI_ISL_535968, EPI_ISL_535971, EPI_ISL_535974 to 535975, EPI_ISL_535978, EPI_ISL_535996 to 535999, EPI_ISL_536001, EPI_ISL_536030, EPI_ISL_536032 to 536033, EPI_ISL_536037, EPI_ISL_536246, EPI_ISL_536248 to 536251			
see above	Hôpital du Centre-de-la-Mauricie	Laboratoire de santé publique du Québec	Guillaume Bourque; Ioannis Ragoussis; Jesse Shapiro; Mark Lathrop and Michel Roger; Mark Lathrop and Michel Roger on behalf of the CoVSeQ research group; Sandrine Moreira
EPI_ISL_535812	Hôpital du Sacré-Coeur de Montréal	Laboratoire de santé publique du Québec	Guillaume Bourque; Ioannis Ragoussis; Jesse Shapiro; Mark Lathrop and Michel Roger on behalf of the CoVSeQ research group; Sandrine Moreira
EPI_ISL_450307, EPI_ISL_450311 to 450313, EPI_ISL_465679 to 465680, EPI_ISL_465693, EPI_ISL_465701, EPI_ISL_535847, EPI_ISL_535868, EPI_ISL_535880, EPI_ISL_535913, EPI_ISL_535917 to 535919, EPI_ISL_535988, EPI_ISL_536026, EPI_ISL_536095, EPI_ISL_536097 to 536098, EPI_ISL_536102, EPI_ISL_536105, EPI_ISL_536163, EPI_ISL_536165, EPI_ISL_536167, EPI_ISL_536170, EPI_ISL_536190, EPI_ISL_536192 to 536193, EPI_ISL_536198, EPI_ISL_536200 to 536202, EPI_ISL_536226 to 536228, EPI_ISL_536254, EPI_ISL_536257, EPI_ISL_536259, EPI_ISL_536261, EPI_ISL_536273 to 536274, EPI_ISL_536287, EPI_ISL_536318 to 536320, EPI_ISL_536349, EPI_ISL_536354 to 536359, EPI_ISL_536362, EPI_ISL_536390			
see above	Hôpital du Suroît	Laboratoire de santé publique du Québec	Guillaume Bourque; Ioannis Ragoussis; Jesse Shapiro; Mark Lathrop and Michel Roger; Mark Lathrop and Michel Roger on behalf of the CoVSeQ research group; Sandrine Moreira
EPI_ISL_535991, EPI_ISL_536113 to 536114, EPI_ISL_1241279	Hôpital et Centre d'Hébergement de Sept-Iles	Laboratoire de santé publique du Québec	Guillaume Bourque; Ioannis Ragoussis; Jesse Shapiro; Mark Lathrop and Michel Roger on behalf of the CoVSeQ research group; Sandrine Moreira
EPI_ISL_535719 to 535720, EPI_ISL_535756 to 535757, EPI_ISL_535769	Hôpital général Juif	Laboratoire de santé publique du Québec	Guillaume Bourque; Ioannis Ragoussis; Jesse Shapiro; Mark Lathrop and Michel Roger; Mark Lathrop and Michel Roger on behalf of the CoVSeQ research group; Sandrine Moreira
EPI_ISL_535852, EPI_ISL_535875	Hôpital général du Lakeshore	Laboratoire de santé publique du Québec	Guillaume Bourque; Ioannis Ragoussis; Jesse Shapiro; Mark Lathrop and Michel Roger; Mark Lathrop and Michel Roger on behalf of the CoVSeQ research group; Sandrine Moreira
EPI_ISL_536106, EPI_ISL_536205	Hôpital régional de Rimouski	Laboratoire de santé publique du Québec	Guillaume Bourque; Ioannis Ragoussis; Jesse Shapiro; Mark Lathrop and Michel Roger on behalf of the CoVSeQ research group; Sandrine Moreira
EPI_ISL_535791, EPI_ISL_535798, EPI_ISL_535837 to 535838, EPI_ISL_536022	Hôpital régional de Saint-Jérôme	Laboratoire de santé publique du Québec	Guillaume Bourque; Ioannis Ragoussis; Jesse Shapiro; Mark Lathrop and Michel Roger; Mark Lathrop and Michel Roger on behalf of the CoVSeQ research group; Sandrine Moreira
EPI_ISL_536101	Hôpital, CLSC et Centre d'Hébergement de Roberval	Laboratoire de santé publique du Québec	Guillaume Bourque; Ioannis Ragoussis; Jesse Shapiro; Mark Lathrop and Michel Roger on behalf of the CoVSeQ research group; Sandrine Moreira
EPI_ISL_535725, EPI_ISL_535750 to 535751, EPI_ISL_535758 to 535759, EPI_ISL_535820 to 535821, EPI_ISL_535833 to 535836	Hôtel-Dieu de Lévis	Laboratoire de santé publique du Québec	Guillaume Bourque; Ioannis Ragoussis; Jesse Shapiro; Mark Lathrop and Michel Roger; Mark Lathrop and Michel Roger on behalf of the CoVSeQ research group; Sandrine Moreira
EPI_ISL_535903 to 535904, EPI_ISL_535990, EPI_ISL_535992, EPI_ISL_536116, EPI_ISL_536121	Hôtel-Dieu de Sorel	Laboratoire de santé publique du Québec	Guillaume Bourque; Ioannis Ragoussis; Jesse Shapiro; Mark Lathrop and Michel Roger; Mark Lathrop and Michel Roger on behalf of the CoVSeQ research group; Sandrine Moreira
EPI_ISL_1058582 to 1058583, EPI_ISL_1058585 to 1058586, EPI_ISL_1165811 to 1165833, EPI_ISL_1171568 to 1171584	I.F.A.C. Hopital Princesse Paola	GIGA Medical Genomics	Bouchra Boujemla; Cécile Meex; Keith Durkin; Maria Artesi; Marie-Pierre Hayette; Nathalie Renotte; Pierrette Melin; Raphaël Boreux; Sébastien Bontems; Vincent Bours
EPI_ISL_649785, EPI_ISL_649938 to 649939, EPI_ISL_653763 to 653783, EPI_ISL_653787 to 653812, EPI_ISL_653814 to 653823, EPI_ISL_722851 to 722854, EPI_ISL_722859 to 722871, EPI_ISL_722895, EPI_ISL_722898, EPI_ISL_722900			
see above	I.R.C.C.S. "S. De Bellis" - Ente Ospedaliero	Istituto Zooprofilattico Sperimentale della Puglia e della Basilicata	Bianco A.; Capozzi L.; Cipolletta D.; Del Sambro L.; Galante D.; Lippolis A.; Manzulli V; Notarnicola M.; Pace L.; Parisi A.; Rondinone V.
EPI_ISL_1272928 to 1272941	IA State Hygienic Laboratory	Centers for Disease Control and Prevention Division of Viral Diseases, Pathogen Discovery	Anna Montmayeur; Anna Uehara; Ben L. Rambo-Martin; Clinton R. Paden; Dhwani Batra; Haibin Wang; Jasmine Padilla; Jing Zhang; Justin Lee; Katie Dillon; Krista Queen; Kristen Knipe; Kristine Lacek; Lori Rowe; Mark Burroughs; Matthew Schmerer; Mili Sheth; Peter W. Cook; Rachel Marine; Sam Shepard; Sarah Nobles; Shoshona Le; Suxiang Tong; Yan Li; Ying Tao
EPI_ISL_751553, EPI_ISL_751565, EPI_ISL_751666, EPI_ISL_751687, EPI_ISL_751692, EPI_ISL_751721, EPI_ISL_751742, EPI_ISL_751779, EPI_ISL_751783, EPI_ISL_903621, EPI_ISL_903629, EPI_ISL_903669, EPI_ISL_903772, EPI_ISL_903815, EPI_ISL_903840, EPI_ISL_903900, EPI_ISL_903920, EPI_ISL_903927			
see above	IA State Hygienic Laboratory	Genomics and Discovery, Respiratory Viruses Branch, Division of Viral Diseases, Centers for Disease Control and Prevention	Anna Montmayeur; Anna Uehara; Ben L. Rambo-Martin; Clinton R. Paden; Dhwani Batra; Haibin Wang; Jasmine Padilla; Jing Zhang; Justin Lee; Krista Queen; Lori Rowe; Mark Burroughs; Mili Sheth; Peter W. Cook; Rachel Marine; Sarah Nobles; Suxiang Tong; Yan Li; Ying Tao
EPI_ISL_424865, EPI_ISL_424895 to 424900, EPI_ISL_447842, EPI_ISL_647990 to 648001	IA State Hygienic Laboratory	Pathogen Discovery, Respiratory Viruses Branch, Division of Viral Diseases, Centers for Disease Control and Prevention	Alison S. Laufer Halpin; Anna Uehara; Brian Lynch; Christopher A. Elkins; Clinton R. Paden; Haibin Wang; Jasmine Padilla; Jing Zhang; Justin Lee; Krista Queen; Mary S. Keckler; Rachel Marine; Suxiang Tong; Yan Li; Ying Tao
EPI_ISL_1094300, EPI_ISL_1094329, EPI_ISL_1094388, EPI_ISL_1094687 to 1094706, EPI_ISL_1095225 to 1095226	IA State Hygienic Laboratory	Respiratory Viruses Branch, Division of Viral Diseases, Centers for Disease Control and Prevention	Anna Montmayeur; Anna Uehara; Ben L. Rambo-Martin; Clinton R. Paden; Dhwani Batra; Haibin Wang; Jasmine Padilla; Jing Zhang; Justin Lee; Krista Queen; Lori Rowe; Mark Burroughs; Mili Sheth; Peter W. Cook; Rachel Marine; Sarah Nobles; Suxiang Tong; Yan Li; Ying Tao
EPI_ISL_981383, EPI_ISL_981385, EPI_ISL_981387, EPI_ISL_984263, EPI_ISL_1078981, EPI_ISL_1078983 to 1078984, EPI_ISL_1078986 to 1078989, EPI_ISL_1078991 to 1078993, EPI_ISL_1078995 to 1078997, EPI_ISL_1078999 to 1079000, EPI_ISL_1079002 to 1079004, EPI_ISL_1079006 to 1079008, EPI_ISL_1079158 to 1079159, EPI_ISL_1079161 to 1079163, EPI_ISL_1079165 to 1079166, EPI_ISL_1086035 to 1086036, EPI_ISL_1086044 to 1086057, EPI_ISL_1092360, EPI_ISL_1095913, EPI_ISL_1096120, EPI_ISL_1096122 to 1096134, EPI_ISL_1096136, EPI_ISL_1121306, EPI_ISL_1121310, EPI_ISL_1121312 to 1121315, EPI_ISL_1121317, EPI_ISL_1121320 to 1121321, EPI_ISL_1121326			
see above	IAL Regional de Bauru	Instituto Adolfo Lutz, Interdiciplinary Procedures Center, Strategic Laboratory	Caio Vinicius Dias Lopes; Claudia Regina Gonçalves; Claudio Tavares Sacchi; Erica Valessa Ramos Gomes; Karoline Rodrigues Campos
EPI_ISL_984247 to 984262, EPI_ISL_1171641 to 1171647, EPI_ISL_1196297 to 1196302, EPI_ISL_1201889 to 1201892	IAL Regional de Marilia	Instituto Adolfo Lutz, Interdiciplinary Procedures Center, Strategic Laboratory	Caio Vinicius Dias Lopes; Claudia Regina Gonçalves; Claudio Tavares Sacchi; Erica Valessa Ramos Gomes; Karoline Rodrigues Campos
EPI_ISL_1171619, EPI_ISL_1171651 to 1171674, EPI_ISL_1219027, EPI_ISL_1219037	IAL Regional de Presidente Prudente	Instituto Adolfo Lutz, Interdiciplinary Procedures Center, Strategic Laboratory	Caio Vinicius Dias Lopes; Claudia Regina Gonçalves; Claudio Tavares Sacchi; Erica Valessa Ramos Gomes; Karoline Rodrigues Campos
EPI_ISL_1139070	IAL Regional de Ribeirao Preto	Instituto Adolfo Lutz, Interdiciplinary Procedures Center, Strategic Laboratory	Caio Vinicius Dias Lopes; Claudia Regina Gonçalves; Claudio Tavares Sacchi; Erica Valessa Ramos Gomes; Karoline Rodrigues Campos
EPI_ISL_1121325, EPI_ISL_1123374 to	IAL Regional de Santos	Instituto Adolfo Lutz, Interdiciplinary Procedures Center,	Caio Vinicius Dias Lopes; Claudia Regina Gonçalves; Claudio Tavares Sacchi; Erica Valessa Ramos Gomes; Karoline Rodrigues Campos

1123375, EPI_ISL_1171622 to 1171640		Strategic Laboratory	
EPI_ISL_1201893, EPI_ISL_1219022 to 1219026, EPI_ISL_1293056 to 1293081	IAL Regional de Sorocaba	Instituto Adolfo Lutz, Interdiciplinary Procedures Center, Strategic Laboratory	Caio Vinicius Dias Lopes; Claudia Regina Gonçalves; Claudio Tavares Sacchi; Erica Valessa Ramos Gomes; Karoline Rodrigues Campos
EPI_ISL_1303518 to 1303534	IAL Regional de São Jose do Rio Preto	Instituto Adolfo Lutz, Interdiciplinary Procedures Center, Strategic Laboratory	Caio Vinicius Dias Lopes; Claudia Regina Gonçalves; Claudio Tavares Sacchi; Erica Valessa Ramos Gomes; Karoline Rodrigues Campos
EPI_ISL_452787 to 452795	ICAR-National Institute of High Security Animal Diseases	ICAR-National Institute of High Security Animal Diseases	Anamika Mishra; Ashutosh Aasdev; Ashwin Ashok Raut; Atul Kumar Pateriya; Chakradhar Tosh; Chandan Kumar Dubey; Dipesh Kumar Nayak; Fateh Singh; G Venkatesh; Harshad Murugkar; Katherukamem Rajukumar; Manoj Kumar; Meghna Tripathi; Naveen Kumar; Niranjan Mishra; Pradeep Gandhale; Pushpendra Namdeo; Pushpendra Singh; Richa Sood; Rupal Singh; Sandeep Bhatia; Sandeep Kumar Jhade; Senmannan Kalaiyarasu; Sethil Kumar D; Shanmugasundaram Nagarajan; Shashi Bhushan Sudhakar; Suman Kumari Shah; Suman Mishra; Sushil Tripathi; Vijendra Pal Singh; Vishnupriya Patil
EPI_ISL_1007617, EPI_ISL_1007629 to 1007630, EPI_ISL_1120821 to 1120823, EPI_ISL_1120828 to 1120830	ICH-SION	Laboratory of genomics and metagenomics, Institute of Microbiology, University Hospital Centre and University of Lausanne, Switzerland	Claire Bertelli; Damien Jacot; Gilbert Greub; Sébastien Aeby; Trestan Pillonel
EPI_ISL_1231365 to 1231373, EPI_ISL_1231376 to 1231381, EPI_ISL_1231383	ICMR-National Institute of Cholera and Enteric Diseases	Indian Council of Medical Research-National Institute of Virology, Microbial Containment Complex	Pragya D. Yadav; Shanta Dutta
EPI_ISL_430464 to 430468, EPI_ISL_437536 to 437539, EPI_ISL_455640 to 455679, EPI_ISL_508441 to 508488	ICMR-National Institute of Cholera and Enteric Diseases	National Institute of Biomedical Genomics	Ananya Chatterjee; Arindam Maitra; Hasina Banu; Mamta Chawla Sarkar; Saumitra Das; Shanta Dutta; Sreedhar Chinnaswamy
EPI_ISL_511899	ICMR-National Institute of Cholera and Enteric Diseases	National Institute of Biomedical Genomics - DBT's PAN-INDIA 1000 SARS--CoV-2 RNA Genome Sequencing Consortium	Ananya Chatterjee; Arindam Maitra; Hasina Banu; Mamta Chawla Sarkar; Saumitra Das; Shanta Dutta; Sreedhar Chinnaswamy
EPI_ISL_1139114 to 1139121, EPI_ISL_1139128 to 1139145	ICMR-RMRC, Dibrugarh	Indian Council of Medical Research-National Institute of Virology, Microbial Containment Complex	Biswajyoti Borkakoty; Pragya D. Yadav
EPI_ISL_1231293 to 1231301	ICMR-Regional Medical Research Center (RMRC), Gorakhpur	Indian Council of Medical Research-National Institute of Virology, Microbial Containment Complex	Hirawati Deval; Pragya D. Yadav
EPI_ISL_1091779 to 1091781	ICMT	Universidad Nacional de Colombia - Laboratorio Genómico One Health	Andres F. Cardona-Rios; Carlos Franco-Muñoz; Daniel O. Maldonado-Perez; Diego A. Álvarez-Díaz; Idabely Betancur Ortiz; Jorge E. Osorio; Juan P. Hernandez-Ortiz; Karl A Ciuoderis; Laura Silvana Perez; Marcela Mercado-Reyes; Sandra Ines Cano
EPI_ISL_751567, EPI_ISL_751590, EPI_ISL_751612, EPI_ISL_751614, EPI_ISL_751618, EPI_ISL_751719, EPI_ISL_751748, EPI_ISL_751750, EPI_ISL_751780, EPI_ISL_751782, EPI_ISL_751789, EPI_ISL_903571, EPI_ISL_903603, EPI_ISL_903619, EPI_ISL_903624, EPI_ISL_903753, EPI_ISL_903887, EPI_ISL_903908, EPI_ISL_903923			
see above	ID Bureau of Laboratories	Genomics and Discovery, Respiratory Viruses Branch, Division of Viral Diseases, Centers for Disease Control and Prevention	Anna Montmayeur; Anna Uehara; Ben L. Rambo-Martin; Clinton R. Paden; Dhwani Batra; Haibin Wang; Jasmine Padilla; Jing Zhang; Justin Lee; Krista Queen; Lori Rowe; Mark Burroughs; Mili Sheth; Peter W. Cook; Rachel Marine; Sarah Nobles; Suxiang Tong; Yan Li; Ying Tao
EPI_ISL_1229142 to 1229143, EPI_ISL_1299217	IDI-IRCCS	INMI Lazzaro Spallanzani IRCCS	A Di Caro; B Bartolini; CEM Gruber; D Castiglia; E Giombini; F Messina; F Santini; G Bonfiglio; G Russo; M Rueca; MR Capobianchi; O Butera; S Madonna
EPI_ISL_845630 to 845633	IDIME S.A	Instituto Nacional de Salud - Dirección de Investigación en Salud Pública	Carlos Franco-Muñoz; Diego A. Álvarez-Díaz; Diego Andrés Prada; Gerardo Santamaría; Jonathan Reales; Julian Naizaque; Katherine Laiton-Donato; Magdalena Wiesner; Marcela Mercado-Reyes; Maria T. Herrera-Sepúlveda; Martha Lucia Ospina Martinez; Mauricio Pacheco-Montealegre; Paola Muñoz-Laiton; Sheryll Corchuelo
EPI_ISL_539491, EPI_ISL_560319	IDSP unit, Dehradun	CSIR-Institute of Microbial Technology	Amandeep Kaur; Anu Singh; Ashwani Kumar; Debarghya Ghose; Dipak Dutta; Harsh Goar; Kanika Bansal; Navin Baid; Poushali Chakraborty; Prabhu B. Patil; Rajesh Kumar Mishra; Sanjeet Kumar; Sanjeev Khosla
EPI_ISL_1081170	IEH Laboratories & Consulting Group	ID Genomics	Curtis Fowler; Evgeni Sokurenko; Hao Geng; Hemantha Kulakesara; Kai Deng; Kirthi Kutumbaka; Lydia Larson; Mansour Samadpour; Veronika Tchesnokova
EPI_ISL_1081332, EPI_ISL_1081337, EPI_ISL_1081339, EPI_ISL_1081341, EPI_ISL_1081346 to 1081347	IEH Laboratories and Consulting Group	IEH and ID Genomics	Curtis Fowler; Evgeni Sokurenko; Hao Geng; Hemantha Kulakesara; Kai Deng; Kirthi Kutumbaka; Lydia Larson; Mansour Samadpour; Veronika Tchesnokova
EPI_ISL_569614	IHS Fort Yates Hospital	South Dakota Public Health Laboratory	Jacob Garfin; Matt Plumb; Xiong Wang; and Chris Carlson
EPI_ISL_1097023 to 1097024	IHU Mediterranee Infection	IHU Mediterranee Infection	Philippe Colson et al.
EPI_ISL_891255	IHU Méditerranée Infection	19-21, boulevard Jean Moulin, 13005 Marseille	Philippe Colson et al.
EPI_ISL_891254	IHU Méditerranée Infection	IHU Méditerranée Infection	Philippe Colson et al.
EPI_ISL_528685	IIP Institute of Genomics and Integrative Biology	IIP Institute of Genomics and Integrative Biology	Ajit Shewale; Akshay Kanakan; Anurag Agrawal; Azka Khan; Bharathram Uppili; Mahesh S. Dhar; Mohammed Faruq; Partha Rakshit; Pooja Sharma; RadhaKrishnan VS; Rajesh Pandey; Robin Marwal; Sandhya Kabra; Simmi Tiwari; Sujeet Singh; Tushar Nale; Vivekanand A
EPI_ISL_404253, EPI_ISL_410045	IL Department of Public Health Chicago Laboratory	Pathogen Discovery, Respiratory Viruses Branch, Division of Viral Diseases, Centers for Dieases Control and Prevention	Anna Uehara; Brett L. Whitaker; Brian Lynch; Clinton R. Paden; Janna' R. Murray; Jing Zhang; Krista Queen; Lijuan Wang; Senthil Kumar K. Sakthivel; Shifaq Kamili; Stephen Lindstrom; Susan I. Gerber; Suxiang Tong; Xiaoyan Lu; Yan Li; Ying Tao
EPI_ISL_424850 to 424851, EPI_ISL_452134, EPI_ISL_913990, EPI_ISL_914013 to 914029, EPI_ISL_1008919 to 1008920, EPI_ISL_1111065, EPI_ISL_1111067, EPI_ISL_1196886			
see above	IL Department of Public Health Chicago Laboratory	Pathogen Discovery, Respiratory Viruses Branch, Division of Viral Diseases, Centers for Disease Control and Prevention	Alison S. Laufer Halpin; Anna Kelleher; Anna Montmayeur; Anna Uehara; Brian Lynch; Christopher A. Elkins; Clinton R. Paden; Haibin Wang; Han Jia Justin Ng; Han Jia Ng; Jing Zhang; Krista Queen; Mary S. Keckler; Peter Cook; Rachel Marine; Suxiang Tong; Yan Li; Ying Tao; Zachary Weiner
EPI_ISL_911923	IL Department of Public Health Chicago Laboratory/Tempus Labs	Pathogen Discovery, Respiratory Viruses Branch, Division of Viral Diseases, Centers for Disease Control and Prevention	Anna Uehara; Clinton R. Paden; Haibin Wang; Jing Zhang; Krista Queen; Peter Cook; Suxiang Tong; Yan Li; Ying Tao
EPI_ISL_914030 to 914034	IL Department of Public Health Springfield Laboratory	Pathogen Discovery, Respiratory Viruses Branch, Division of Viral Diseases, Centers for Disease Control and Prevention	Anna Uehara; Clinton R. Paden; Haibin Wang; Jing Zhang; Krista Queen; Peter Cook; Suxiang Tong; Yan Li; Ying Tao
EPI_ISL_1196887	IL Dept. of Public Health Carbondale Laboratory	Pathogen Discovery, Respiratory Viruses Branch, Division of Viral Diseases, Centers for Disease Control and Prevention	Anna Kelleher; Anna Uehara; Brian Lynch; Clinton R. Paden; Haibin Wang; Han Jia Justin Ng; Jing Zhang; Krista Queen; Peter Cook; Suxiang Tong; Yan Li; Ying Tao
EPI_ISL_1272697 to 1272712	IL Dept. of Public Health Springfield Laboratory	Centers for Disease Control and Prevention Division of Viral Diseases, Pathogen Discovery	Anna Montmayeur; Anna Uehara; Ben L. Rambo-Martin; Clinton R. Paden; Dhwani Batra; Haibin Wang; Jasmine Padilla; Jing Zhang; Justin Lee; Katie Dillon; Krista Queen; Kristen Knipe; Kristine Lacek; Lori Rowe; Mark Burroughs; Matthew Schmerer; Mili Sheth; Peter W. Cook; Rachel Marine; Sam Shepard; Sarah Nobles; Shoshona Le; Suxiang Tong; Yan Li; Ying Tao
EPI_ISL_903587 to 903588, EPI_ISL_903607, EPI_ISL_903663, EPI_ISL_903682, EPI_ISL_903742, EPI_ISL_903779, EPI_ISL_903789, EPI_ISL_903807, EPI_ISL_903812, EPI_ISL_903828, EPI_ISL_903847, EPI_ISL_903896, EPI_ISL_903911, EPI_ISL_1225872, EPI_ISL_1226092 to 1226104			
see above	IL Dept. of Public Health Springfield Laboratory	Genomics and Discovery, Respiratory Viruses Branch, Division of Viral Diseases, Centers for Disease Control and Prevention	Anna Montmayeur; Anna Uehara; Ben L. Rambo-Martin; Clinton R. Paden; Dhwani Batra; Haibin Wang; Jasmine Padilla; Jing Zhang; Justin Lee; Katie Dillon; Krista Queen; Kristen Knipe; Kristine Lacek; Lori Rowe; Mark Burroughs; Matthew Schmerer; Mili Sheth; Peter W. Cook; Rachel Marine; Sam Shepard; Sarah Nobles; Shoshona Le; Suxiang Tong; Yan Li; Ying Tao
EPI_ISL_913991, EPI_ISL_913995 to 913996, EPI_ISL_955291, EPI_ISL_955309, EPI_ISL_955328, EPI_ISL_1008893 to 1008896, EPI_ISL_1196868, EPI_ISL_1196895 to 1196896			
see above	IL Dept. of Public Health Springfield Laboratory	Pathogen Discovery, Respiratory Viruses Branch, Division of Viral Diseases, Centers for Disease Control and Prevention	Anna Kelleher; Anna Uehara; Brian Lynch; Clinton R. Paden; Haibin Wang; Han Jia Justin Ng; Jing Zhang; Krista Queen; Peter Cook; Suxiang Tong; Yan Li; Ying Tao

EPI_ISL_1094202, EPI_ISL_1094222, EPI_ISL_1094249 to 1094250, EPI_ISL_1094258, EPI_ISL_1094289, EPI_ISL_1094488 to 1094491, EPI_ISL_1094723 to 1094725, EPI_ISL_1095233 to 1095234			
see above	IL Dept. of Public Health Springfield Laboratory	Respiratory Viruses Branch, Division of Viral Diseases, Centers for Disease Control and Prevention	Anna Montmayeur; Anna Uehara; Ben L. Rambo-Martin; Clinton R. Paden; Dhwani Batra; Haibin Wang; Jasmine Padilla; Jing Zhang; Justin Lee; Krista Queen; Lori Rowe; Mark Burroughs; Mili Sheth; Peter W. Cook; Rachel Marine; Sarah Nobles; Suxiang Tong; Yan Li; Ying Tao
EPI_ISL_853854 to 853855, EPI_ISL_853914, EPI_ISL_853924 to 853928, EPI_ISL_934425 to 934433, EPI_ISL_1209267	ILV Kärnten	Bergthaler laboratory, CeMM Research Center for Molecular Medicine of the Austrian Academy of Sciences	Alexander Lercher; Alexandra Popa; Andreas Bergthaler; Anna Schedl; Bekir Erguner; Benedikt Agerer; Christoph Bock; Christoph Bock; Fabian Amman; Jakob-Wendelin Genger; Jan Laine; Lukas Endler; Maelle Le Moing; Martin Senekowitsch; Martin Senekowitsch; Michael Schuster; Michael Schuster; Thomas Penz
EPI_ISL_445379	IMALAB- HOSPITAL FACH	Instituto de Salud Publica de Chile	Alejandra Acevedo; Andrés E Castillo; Bárbara Parra; Carolina Tambley; Gabriel Leal; Jaime Lagos; Jorge Fernandez; Loredana Arata; Patricia Bustos; Paz Tapia; Rodrigo Fasce; Winston Andrade
EPI_ISL_1141654 to 1141655, EPI_ISL_1143471, EPI_ISL_1143473, EPI_ISL_1143490, EPI_ISL_1143533 to 1143537, EPI_ISL_1143539, EPI_ISL_1143546 to 1143550, EPI_ISL_1143553, EPI_ISL_1147417 to 1147420, EPI_ISL_1147429 to 1147430, EPI_ISL_1151966, EPI_ISL_1151969 to 1151970, EPI_ISL_1151973, EPI_ISL_1151979, EPI_ISL_1151982 to 1151984, EPI_ISL_1156380 to 1156382, EPI_ISL_1156386, EPI_ISL_1156388, EPI_ISL_1156392, EPI_ISL_1156442, EPI_ISL_1212019, EPI_ISL_1212023 to 1212024, EPI_ISL_1212032 to 1212033, EPI_ISL_1212036, EPI_ISL_1212039, EPI_ISL_1212041, EPI_ISL_1212048, EPI_ISL_1212052, EPI_ISL_1212058, EPI_ISL_1212060, EPI_ISL_1212063 to 1212064, EPI_ISL_1282310, EPI_ISL_1282312 to 1282313, EPI_ISL_1282319 to 1282320, EPI_ISL_1282325 to 1282326, EPI_ISL_1282330 to 1282331, EPI_ISL_1282339, EPI_ISL_1282341, EPI_ISL_1282364, EPI_ISL_1282371, EPI_ISL_1282373			
see above	IMD - Institut für Medizinische Diagnostik Berlin-Potsdam	Robert Koch Institute	
EPI_ISL_1143449 to 1143454, EPI_ISL_1143456 to 1143467, EPI_ISL_1143469 to 1143470, EPI_ISL_1143472, EPI_ISL_1143474, EPI_ISL_1143476 to 1143487, EPI_ISL_1143501, EPI_ISL_1143518, EPI_ISL_1143522, EPI_ISL_1143525, EPI_ISL_1143527, EPI_ISL_1143532, EPI_ISL_1143551, EPI_ISL_1147454, EPI_ISL_1147457 to 1147458, EPI_ISL_1147460, EPI_ISL_1147464 to 1147465, EPI_ISL_1147467 to 1147469, EPI_ISL_1147473 to 1147474, EPI_ISL_1147478, EPI_ISL_1147480, EPI_ISL_1147486 to 1147487, EPI_ISL_1147491, EPI_ISL_1147495 to 1147496, EPI_ISL_1147500, EPI_ISL_1147502 to 1147505, EPI_ISL_1147509, EPI_ISL_1147511 to 1147513, EPI_ISL_1147515 to 1147516, EPI_ISL_1147518 to 1147528, EPI_ISL_1151895, EPI_ISL_1151897 to 1151898, EPI_ISL_1151900 to 1151901, EPI_ISL_1151904, EPI_ISL_1151910 to 1151914, EPI_ISL_1151918, EPI_ISL_1151922, EPI_ISL_1151924 to 1151925, EPI_ISL_1151931 to 1151932, EPI_ISL_1151934, EPI_ISL_1151936, EPI_ISL_1151940, EPI_ISL_1151943, EPI_ISL_1151946, EPI_ISL_1151951, EPI_ISL_1151953, EPI_ISL_1151955, EPI_ISL_1151959, EPI_ISL_1151962, EPI_ISL_1156376, EPI_ISL_1156385, EPI_ISL_1156400, EPI_ISL_1156415, EPI_ISL_1156419, EPI_ISL_1156426, EPI_ISL_1156429 to 1156430, EPI_ISL_1156435 to 1156436, EPI_ISL_1212022, EPI_ISL_1212035, EPI_ISL_1212038, EPI_ISL_1212046, EPI_ISL_1212051, EPI_ISL_1212056, EPI_ISL_1212059, EPI_ISL_1212061, EPI_ISL_1212065, EPI_ISL_1217355, EPI_ISL_1217358, EPI_ISL_1217362, EPI_ISL_1217364, EPI_ISL_1217367, EPI_ISL_1217369 to 1217370, EPI_ISL_1217372 to 1217373, EPI_ISL_1217379, EPI_ISL_1217382, EPI_ISL_1217389 to 1217391, EPI_ISL_1217394 to 1217396, EPI_ISL_1217398, EPI_ISL_1217401 to 1217402, EPI_ISL_1217405, EPI_ISL_1217407 to 1217408, EPI_ISL_1217410 to 1217412, EPI_ISL_1217415, EPI_ISL_1217419 to 1217422, EPI_ISL_1282324, EPI_ISL_1282332, EPI_ISL_1282335, EPI_ISL_1282337, EPI_ISL_1282356, EPI_ISL_1282365 to 1282366, EPI_ISL_1282369, EPI_ISL_1282374, EPI_ISL_1282383, EPI_ISL_1285734, EPI_ISL_1285736, EPI_ISL_1285739, EPI_ISL_1285741, EPI_ISL_1285744 to 1285748, EPI_ISL_1285750, EPI_ISL_1285754, EPI_ISL_1285757 to 1285758, EPI_ISL_1285760, EPI_ISL_1285764, EPI_ISL_1285766, EPI_ISL_1285768 to 1285770, EPI_ISL_1285772 to 1285775, EPI_ISL_1285778 to 1285780, EPI_ISL_1285784, EPI_ISL_1285787 to 1285790, EPI_ISL_1285792, EPI_ISL_1285794 to 1285795			
see above	IMD - Labor Oderland	Robert Koch Institute	
EPI_ISL_1143448, EPI_ISL_1143488 to 1143489, EPI_ISL_1143491 to 1143492, EPI_ISL_1143494 to 1143500, EPI_ISL_1143502 to 1143505, EPI_ISL_1143507 to 1143515, EPI_ISL_1143517, EPI_ISL_1143519 to 1143521, EPI_ISL_1143523 to 1143524, EPI_ISL_1143528 to 1143531, EPI_ISL_1143542 to 1143543, EPI_ISL_1143552, EPI_ISL_1147453, EPI_ISL_1147456, EPI_ISL_1147459, EPI_ISL_1147471, EPI_ISL_1147482, EPI_ISL_1147492, EPI_ISL_1147497, EPI_ISL_1147499, EPI_ISL_1147501, EPI_ISL_1151971 to 1151972, EPI_ISL_1151974 to 1151975, EPI_ISL_1151989, EPI_ISL_1156374, EPI_ISL_1156377 to 1156379, EPI_ISL_1156383, EPI_ISL_1156387, EPI_ISL_1156391, EPI_ISL_1156397 to 1156399, EPI_ISL_1156403, EPI_ISL_1156412, EPI_ISL_1156416, EPI_ISL_1156420, EPI_ISL_1156423, EPI_ISL_1156427 to 1156428, EPI_ISL_1156433, EPI_ISL_1156439, EPI_ISL_1156441, EPI_ISL_1210790, EPI_ISL_1210797 to 1210798, EPI_ISL_1212020 to 1212021, EPI_ISL_1212025, EPI_ISL_1212027 to 1212028, EPI_ISL_1212034, EPI_ISL_1212040, EPI_ISL_1212042, EPI_ISL_1212045, EPI_ISL_1212047, EPI_ISL_1212049, EPI_ISL_1212053, EPI_ISL_1217357, EPI_ISL_1217359, EPI_ISL_1217375, EPI_ISL_1217384 to 1217385, EPI_ISL_1217393, EPI_ISL_1217400, EPI_ISL_1217403, EPI_ISL_1217413 to 1217414, EPI_ISL_1217423, EPI_ISL_1282311, EPI_ISL_1282314 to 1282315, EPI_ISL_1282317 to 1282318, EPI_ISL_1282322 to 1282323, EPI_ISL_1282327 to 1282329, EPI_ISL_1282333, EPI_ISL_1282336, EPI_ISL_1282338, EPI_ISL_1282342 to 1282343, EPI_ISL_1282345 to 1282346, EPI_ISL_1282349 to 1282350, EPI_ISL_1282352 to 1282354, EPI_ISL_1282357 to 1282363, EPI_ISL_1282372, EPI_ISL_1282375 to 1282379, EPI_ISL_1282381 to 1282382			
see above	IMD - MVZ Labor Greifswald	Robert Koch Institute	
EPI_ISL_1048286, EPI_ISL_1048306, EPI_ISL_1048310, EPI_ISL_1048316 to 1048317	IMD - MVZ Labor Greifswald	Robert Koch Institute, Influenza and respiratory viruses FG17 & Bioinformatics MF1, Berlin, Germany	A. Kruggel; Aleksandar Radonic; K. Meinck; Marianne Wedde; Oliver Drechsel; Ralf Duerrwald; Rene Kmiecinski; Stefan Kroeger; Stephan Fuchs; Thorsten Wolff
EPI_ISL_1143538, EPI_ISL_1143540 to 1143541, EPI_ISL_1143545, EPI_ISL_1143554, EPI_ISL_1147476, EPI_ISL_1147481, EPI_ISL_1147483, EPI_ISL_1147485, EPI_ISL_1147494, EPI_ISL_1147508, EPI_ISL_1151896, EPI_ISL_1151907, EPI_ISL_1151920, EPI_ISL_1151923, EPI_ISL_1151944, EPI_ISL_1151960, EPI_ISL_1210777, EPI_ISL_1210783, EPI_ISL_1210786, EPI_ISL_1210791 to 1210792, EPI_ISL_1210794 to 1210795, EPI_ISL_1210802, EPI_ISL_1210815, EPI_ISL_1217363, EPI_ISL_1217365, EPI_ISL_1217381, EPI_ISL_1217406, EPI_ISL_1217409, EPI_ISL_1217416 to 1217417, EPI_ISL_1285735, EPI_ISL_1285738, EPI_ISL_1285749, EPI_ISL_1285751 to 1285753, EPI_ISL_1285765, EPI_ISL_1285771, EPI_ISL_1285776 to 1285777, EPI_ISL_1285786, EPI_ISL_1285791, EPI_ISL_1285796			
see above	IMD - Medizinisches Labor Rostock	Robert Koch Institute	
EPI_ISL_1140525 to 1140528, EPI_ISL_1140534 to 1140539, EPI_ISL_1144370, EPI_ISL_1144372, EPI_ISL_1144377 to 1144383, EPI_ISL_1146717, EPI_ISL_1146721 to 1146726, EPI_ISL_1147981 to 1147988, EPI_ISL_1148468, EPI_ISL_1148470, EPI_ISL_1148472, EPI_ISL_1148477, EPI_ISL_1148487 to 1148490, EPI_ISL_1148493, EPI_ISL_1148496, EPI_ISL_1148501, EPI_ISL_1148509, EPI_ISL_1152513, EPI_ISL_1152516 to 1152517, EPI_ISL_1152519, EPI_ISL_1152521, EPI_ISL_1155431 to 1155432, EPI_ISL_1155437 to 1155438, EPI_ISL_1155443 to 1155444, EPI_ISL_1155446, EPI_ISL_1155455, EPI_ISL_1155457, EPI_ISL_1155462, EPI_ISL_1155464 to 1155467, EPI_ISL_1216787, EPI_ISL_1216802, EPI_ISL_1216817, EPI_ISL_1216828, EPI_ISL_1216838, EPI_ISL_1216841, EPI_ISL_1216846, EPI_ISL_1216851, EPI_ISL_1216856, EPI_ISL_1216870, EPI_ISL_1216875, EPI_ISL_1216905, EPI_ISL_1216909, EPI_ISL_1216920, EPI_ISL_1216924, EPI_ISL_1216929, EPI_ISL_1216934 to 1216936, EPI_ISL_1216983, EPI_ISL_1216985 to 1216986, EPI_ISL_1216989, EPI_ISL_1216994, EPI_ISL_1280680, EPI_ISL_1280683, EPI_ISL_1280693 to 1280694, EPI_ISL_1280696, EPI_ISL_1280717, EPI_ISL_1284516, EPI_ISL_1284518, EPI_ISL_1284520, EPI_ISL_1284523, EPI_ISL_1284528, EPI_ISL_1284538, EPI_ISL_1284541, EPI_ISL_1284547, EPI_ISL_1284549, EPI_ISL_1284553			
see above	IMD Labor Frankfurt	Robert Koch Institute	
EPI_ISL_1213173, EPI_ISL_1213175, EPI_ISL_1213177 to 1213178, EPI_ISL_1213180, EPI_ISL_1213182 to 1213183, EPI_ISL_1213185, EPI_ISL_1213187, EPI_ISL_1213192, EPI_ISL_1213194, EPI_ISL_1213196 to 1213197, EPI_ISL_1213199, EPI_ISL_1213209, EPI_ISL_1213275, EPI_ISL_1213277, EPI_ISL_1213279, EPI_ISL_1213281 to 1213282, EPI_ISL_1213284, EPI_ISL_1213286, EPI_ISL_1213288 to 1213289, EPI_ISL_1213291, EPI_ISL_1213293, EPI_ISL_1213315, EPI_ISL_1213317, EPI_ISL_1213319 to 1213320, EPI_ISL_1213322, EPI_ISL_1213329, EPI_ISL_1213345 to 1213346, EPI_ISL_1213348, EPI_ISL_1213350, EPI_ISL_1213352 to 1213353, EPI_ISL_1213355, EPI_ISL_1213357 to 1213358, EPI_ISL_1213360, EPI_ISL_1213362, EPI_ISL_1213364			
see above	IMT-UFRN/RN	Bioinformatics Laboratory / LNCC	Alessandra P Lamarca; Alexandra L Gerber; Ana Paula Melo Mariano; Ana Paula de C Guimarães; Ana Tereza R Vasconcelos; Angela Maria Guimarães Santos; Bianca Mendes Maciel; Danielle Angst Secco; Eduardo Sérgio Soares Sousa; Eloiza Helena Campana; Francisco Paulo Freire Neto; George Rego Albuquerque; Kátia Castanho Scortecci; Lucymara Fassarella Agnez Lima; Luiz G P de Almeida; Luís Cristóvão Porto; Otavio J. Brustolini; Paulo Ricardo Nascimento; Ronaldo da Silva Francisco Jr; Sandra Rocha Gadelha; Selma Maria Bezerra Jeronimo; Vinícius Pietta Perez
EPI_ISL_1084723 to 1084725, EPI_ISL_1084727 to 1084729, EPI_ISL_1084731 to 1084736, EPI_ISL_1171871	IMT_USP	Laboratório de Parasitologia Médica - Instituto de Medicina Tropical - Universidade de São Paulo	Brazil-UK Centre for Arbovirus Discovery Diagnosis Genomics and Epidemiology (CADDE) Genomic Network - Instituto de Medicina Tropical
EPI_ISL_914035	IN State Department of Health Laboratory Service	Pathogen Discovery, Respiratory Viruses Branch, Division of Viral Diseases, Centers for Disease Control and Prevention	Anna Uehara; Clinton R. Paden; Haibin Wang; Jing Zhang; Krista Queen; Peter Cook; Suxiang Tong; Yan Li; Ying Tao
EPI_ISL_1272653 to 1272659	IN State Department of Health Laboratory Services	Centers for Disease Control and Prevention Division of Viral Diseases, Pathogen Discovery	Anna Montmayeur; Anna Uehara; Ben L. Rambo-Martin; Clinton R. Paden; Dhwani Batra; Haibin Wang; Jasmine Padilla; Jing Zhang; Justin Lee; Katie Dillon; Krista Queen; Kristen Knipe; Kristine Lacek; Lori Rowe; Mark Burroughs; Matthew Schmerer; Mili Sheth; Peter W. Cook; Rachel Marine; Sam Shepard; Sarah Nobles; Shoshona Le; Suxiang Tong; Yan Li; Ying Tao
EPI_ISL_751579, EPI_ISL_751599 to 751600, EPI_ISL_751626, EPI_ISL_751632, EPI_ISL_751672, EPI_ISL_751676, EPI_ISL_751727, EPI_ISL_751768, EPI_ISL_751770, EPI_ISL_903616, EPI_ISL_903754, EPI_ISL_903773, EPI_ISL_903875, EPI_ISL_903883, EPI_ISL_903886, EPI_ISL_903904, EPI_ISL_1225987 to 1225991			
see above	IN State Department of Health Laboratory Services	Genomics and Discovery, Respiratory Viruses Branch, Division of Viral Diseases, Centers for Disease Control and Prevention	Anna Montmayeur; Anna Uehara; Ben L. Rambo-Martin; Clinton R. Paden; Dhwani Batra; Haibin Wang; Jasmine Padilla; Jing Zhang; Justin Lee; Katie Dillon; Krista Queen; Kristen Knipe; Kristine Lacek; Lori Rowe; Mark Burroughs; Matthew Schmerer; Mili Sheth; Peter W. Cook; Rachel Marine; Sam Shepard; Sarah Nobles; Shoshona Le; Suxiang Tong; Yan Li; Ying Tao
EPI_ISL_424866, EPI_ISL_426422 to 426424, EPI_ISL_452131, EPI_ISL_452135 to 452136, EPI_ISL_527666, EPI_ISL_540431, EPI_ISL_1008917 to 1008918, EPI_ISL_1008924 to 1008927			
see above	IN State Department of Health Laboratory Services	Pathogen Discovery, Respiratory Viruses Branch, Division of Viral Diseases, Centers for Disease Control and Prevention	Alison S. Laufer Halpin; Anna Montmayeur; Anna Uehara; Brian Lynch; Christopher A. Elkins; Clinton R. Paden; Haibin Wang; Jing Zhang; Krista Queen; Mary S. Keckler; Peter Cook; Rachel Marine; Suxiang Tong; Yan Li; Ying Tao; Zachary Weiner
EPI_ISL_1094241, EPI_ISL_1094326, EPI_ISL_1094339, EPI_ISL_1094776, EPI_ISL_1094778 to 1094790, EPI_ISL_1095243 to 1095248	IN State Department of Health Laboratory Services	Respiratory Viruses Branch, Division of Viral Diseases, Centers for Disease Control and Prevention	Anna Montmayeur; Anna Uehara; Ben L. Rambo-Martin; Clinton R. Paden; Dhwani Batra; Haibin Wang; Jasmine Padilla; Jing Zhang; Justin Lee; Krista Queen; Lori Rowe; Mark Burroughs; Mili Sheth; Peter W. Cook; Rachel Marine; Sarah Nobles; Suxiang Tong; Yan Li; Ying Tao
EPI_ISL_648209 to 648216, EPI_ISL_648218, EPI_ISL_648677	INBIRS-UBA	Laboratorio Mixto de Biotecnología Acuática (LMBA)	Adriana Giri; Agustina Cerri; Ana Cavatorta; Ana Paletta; Diego Chouhy; Elisa Bolatti; Elizabeth Tapia; Federico Remes Lenicov; Flavio Spetale; Gastón Viarengo; Ignacio García Labari; Javier Murillo; Joaquín Ezpeleta; Julian Acosta; Laura Angelone; Leandro Ciappina; María Re; Pablo Casal; Pilar Bulacio; Silvana Spinelli; Silvia Arranz; Sofía Lavista Llanos; Vanina Villanova; Victoria Posner
EPI_ISL_419255, EPI_ISL_493328 to 493331, EPI_ISL_590693 to 590698, EPI_ISL_603137 to 603187, EPI_ISL_609989 to 609999, EPI_ISL_882918 to 882920, EPI_ISL_882924 to 882932, EPI_ISL_913448, EPI_ISL_941338, EPI_ISL_965127 to 965128, EPI_ISL_983323 to 983325, EPI_ISL_1034280, EPI_ISL_1039782 to 1039783, EPI_ISL_1063911, EPI_ISL_1079995, EPI_ISL_1091276 to 1091279, EPI_ISL_1091788 to 1091789, EPI_ISL_1169883 to 1169886, EPI_ISL_1195950, EPI_ISL_1198698, EPI_ISL_1210494 to 1210496, EPI_ISL_1229131, EPI_ISL_1259405 to 1259408, EPI_ISL_1259410 to 1259411, EPI_ISL_1259413 to 1259415,			

EPI_ISL_1290804, EPI_ISL_1295606, EPI_ISL_1295609, EPI_ISL_1295616 to 1295617, EPI_ISL_1295642, EPI_ISL_1296237, EPI_ISL_1296292, EPI_ISL_1299218			
see above	INMI Lazzaro Spallanzani IRCCS	INMI Lazzaro Spallanzani IRCCS	A Di Caro; Alessandra D'Abramo; Annunziata Tamburro; Antonino Di Caro; B Bartolini; Barbara Bartolini; Beatrice Valli; C.E.M Gruber; CEM Gruber; Cesare E. M. Gruber; Cesare E.M. Gruber; Cesare EM Gruber; Concetta Castilletti; Daniele Castiglia; Daniele Lapa; E Giombini; Eleonora Lalle; Emanuela Giombini; Emanuele Nicastri; F Messina; F Santini; Fabrizio Carletti; Francesca Colavita; Francesco Messina; Francesco Vairo; Fulvia Pimpinelli; G Bonfiglio; Giandomenico Russo; Giuseppe Ippolito.; Giuseppina Cappiello; M Rueca; M Rueca0; M. Rueca; MR Capobianchi; Marcello Meledandri; Maria Letizia Schiavone; Maria R Capobianchi; Maria R. Capobianchi; Mariarosa Gaudio; Martina Rueca; O Butera; Ornella Butera; Patrizia Massarelli; Silvia Meschi; Simone Lanini; Stefania Madonna
EPI_ISL_410545 to 410546, EPI_ISL_417921 to 417923, EPI_ISL_419254, EPI_ISL_424342 to 424344	INMI Lazzaro Spallanzani IRCCS	Laboratory of Virology, INMI Lazzaro Spallanzani IRCCS	Antonino Di Caro; Barbara Bartolini; Cesare E. M. Gruber; Cesare Ernesto Maria Gruber; Concetta Castilletti; Daniele Lapa; Eleonora Lalle; Emanuela Giombini; Emanuele Nicastri; Fabrizio Carletti; Francesca Colavita; Francesco Messina; Francesco Vairo; Giulia Matusali; Giuseppe Ippolito; Giuseppe Ippolito.; Licia Bordi; Maria R. Capobianchi; Maria Rosaria Capobianchi; Martina Rueca
EPI_ISL_1109633, EPI_ISL_1201486, EPI_ISL_1235668 to 1235677, EPI_ISL_1252844 to 1252846	INNO Diagnostics Reference Laboratory	RCMI-Center for Research Resources, Ponce Research Institute	Andrea Arias-García; Raphael Sánchez-Torres; Vanessa Rivera-Amill
EPI_ISL_806544	INSPI Instituto Nacional de Investigación en Salud Pública	Av. Julián Coronel 905 entre Esmeraldas y José Mascote Av. Juan Tanca Marengo No. 100 y Av. de las Américas	Alberto Orlando.; Alfredo Bruno; Andrés Carrazco; Doménica de Mora; Leandro Patiño; Manuel González; Maritza Olmedo; Mary Regato; Melissa Zambrano; Orson Mestanza
EPI_ISL_826798, EPI_ISL_826800, EPI_ISL_826802, EPI_ISL_826804 to 826805, EPI_ISL_826807, EPI_ISL_826809, EPI_ISL_826811 to 826812, EPI_ISL_826814, EPI_ISL_826816, EPI_ISL_826818, EPI_ISL_826820, EPI_ISL_826822 to 826823, EPI_ISL_826825, EPI_ISL_826827, EPI_ISL_826829 to 826830, EPI_ISL_826832, EPI_ISL_826834, EPI_ISL_826836, EPI_ISL_831020			
see above	INSPI-CRN DE INFLUENZA Y OTROS VIRUS RESPIRATORIOS	Instituto de Salud Publica de Chile	Alfredo Bruno; Andres Castillo; Barbara Parra; Domenica de Mora; Gisselle Barra; Jaime Lagos; Javier Tognarelli; Jimmy Garcez; Jorge Fernandez; Loredana Arata; Manuel Gonzalez; Martiza Olmedo; Michelle Paez; Patricia Bustos; Rodrigo Fasce; Solon Narvaez
EPI_ISL_940769, EPI_ISL_940773, EPI_ISL_940775, EPI_ISL_940781, EPI_ISL_940821	INSPI-CRN de Influenza y otros virus respiratorios	INSPI-Centro de Investigación Multidisciplinaria de la DTIDI	Alberto Orlando; Alberto Orlando.; Alfredo Bruno; Andrés Carrazco-Montalvo; Doménica de Mora; Leandro Patiño; Manuel González; Maritza Olmedo; Mary Regato-Arrata; Melissa Zambrano; Orson Mestanza
EPI_ISL_1191683, EPI_ISL_1312947	INSTITUT BERGONIE	CNR Virus des Infections Respiratoires - France SUD	Antonin Bal; Bruno Lina; Gregory Destras; Gwendolyne Burfin; Hadrien Regue; Laurence Josset; Martine Valette; Quentin Semanas
EPI_ISL_981485	INSTITUT GUSTAVE ROUSSY	Department of Virology, Henri Mondor University Hospital, Assistance Publique Hôpitaux de Paris, Université Paris-Est Créteil, INSERM U955	Alexandre Soulier; Christophe Rodriguez; Elisabeth Trawinski; Guillaume Gricourt; Jean-Michel Pawlotsky; Melissa N'Debi; Slim Fourati; Vanessa Demontant
EPI_ISL_445302	INSTITUTO MEDICO LEGAL	Instituto de Salud Publica de Chile	Alejandra Acevedo; Andrés E Castillo; Bárbara Parra; Carolina Tambley; Gabriel Leal; Jaime Lagos; Jorge Fernandez; Loredana Arata; Patricia Bustos; Paz Tapia; Rodrigo Fasce; Winston Andrade
EPI_ISL_492184, EPI_ISL_493197, EPI_ISL_493213, EPI_ISL_494756 to 494757, EPI_ISL_494759, EPI_ISL_494761 to 494763, EPI_ISL_494771, EPI_ISL_494774 to 494776, EPI_ISL_775239 to 775240, EPI_ISL_775243 to 775244, EPI_ISL_775246, EPI_ISL_775257, EPI_ISL_775259, EPI_ISL_775261, EPI_ISL_1082292 to 1082293, EPI_ISL_1082295, EPI_ISL_1257899 to 1257900			
see above	INT Fondazione Pascale	INT Fondazione Pascale	INT Fondazione Pascale; Pascale
EPI_ISL_445357	INTEGRAMEDICA CENTROS MEDICOS S.A.	Instituto de Salud Publica de Chile	Alejandra Acevedo; Andrés E Castillo; Bárbara Parra; Carolina Tambley; Gabriel Leal; Jaime Lagos; Jorge Fernandez; Loredana Arata; Patricia Bustos; Paz Tapia; Rodrigo Fasce; Winston Andrade
EPI_ISL_445261	INTEGRAMEDICA LAB. CLINICO LTDA.	Instituto de Salud Publica de Chile	Alejandra Acevedo; Andrés E Castillo; Bárbara Parra; Carolina Tambley; Gabriel Leal; Jaime Lagos; Jorge Fernandez; Loredana Arata; Patricia Bustos; Paz Tapia; Rodrigo Fasce; Winston Andrade
EPI_ISL_445324	INTEGRAMEDICA S.A	Instituto de Salud Publica de Chile	Alejandra Acevedo; Andrés E Castillo; Bárbara Parra; Carolina Tambley; Gabriel Leal; Jaime Lagos; Jorge Fernandez; Loredana Arata; Patricia Bustos; Paz Tapia; Rodrigo Fasce; Winston Andrade
EPI_ISL_492980 to 492987, EPI_ISL_751318 to 751447, EPI_ISL_1064018 to 1064021	IRCCS Sacro Cuore Don Calabria Hospital, Department of Infectious, Tropical Diseases & Microbiology	University of Verona, Department of Biotechnology	Antonio Mori; Chiara Degli Esposti; Chiara Piubelli; Cristina Beltrami; Elena Pomari; Emanuela Cosentino; Giulia Lopatriello; Luca Marcolungo; Massimo Delledonne; Michela Deiana
EPI_ISL_902755, EPI_ISL_1229130	IRCCS San Raffaele	INMI Lazzaro Spallanzani IRCCS	A Di Caro; B Bartolini; C.E.M Gruber; CEM Gruber; D Limongi; D Russo; E Giombini; F Ciatti; F Messina; M Rueca; MR Capobianchi; O Butera
EPI_ISL_1027628	IRESSEF GENOMICS LAB	IRESSEF GENOMICS LAB	Abdoulie Kanteh; Dr Abdou PADANE; Dr Abdul Karim Sesay; Dr Aminata MBOUP; Dr Badara CISSE; Dr Birahim Pierre NDIAYE; Dr Moustapha MBOW; Dr Papa Alassane DIAW; Professor Ndéye Coumba Touré KANE; Professor Souleymane MBOUP
EPI_ISL_480961, EPI_ISL_480975, EPI_ISL_480981, EPI_ISL_480989, EPI_ISL_480995, EPI_ISL_481003, EPI_ISL_481017, EPI_ISL_481025, EPI_ISL_481029, EPI_ISL_481034 to 481035			
see above	ISGlobal, Institut de Salut Global de Barcelona	SeqCOVID-SPAIN consortium/IBV(CSIC)	Alberto L Garcia-Basteiro; Alfredo Mayor; Carlota Dobaño; Gemma Moncunill; Pau Cisteró and SeqCOVID-SPAIN consortium
EPI_ISL_1117526	IZS TERAMO	Istituto Zooprofilattico Sperimentale dell'Abruzzo e Molise "G. Caporale"	Ancora M; Calistri P; Cammà C; Curini V; Di Domenico M; Di Pasquale A; Lorusso A; Mangone I; Marcacci M; Puglia I; Rinaldi A; Savini G; Scialabba S
EPI_ISL_833076	IZSAM	Istituto Zooprofilattico Sperimentale dell'Abruzzo e Molise "G. Caporale"	Ancora M; Calistri P; Cammà C; Curini V; Delli Compagni E; Di Domenico M; Di Pasquale A; Lorusso A; Mangone I; Marcacci M; Puglia I; Rinaldi A; Savini G
EPI_ISL_572320 to 572324, EPI_ISL_584069, EPI_ISL_584071 to 584072	IZSM	IZSM	Giovanna Fusco; Lorena Cardillo; Maurizio Viscardi
EPI_ISL_1163889 to 1163913, EPI_ISL_1166877 to 1166882	IZSM	IZSM-U.O.C. Virologia	Antonio Limone; Esterina De Carlo; Giovanna Fusco; Giuseppe Prota; Lorena Cardillo; Luigi Atripaldi; Maurizio Viscardi; Vincenzo Petrella
EPI_ISL_900574 to 900690, EPI_ISL_1086058 to 1086064, EPI_ISL_1086066 to 1086067, EPI_ISL_1086069 to 1086072, EPI_ISL_1086074 to 1086075, EPI_ISL_1086077 to 1086081, EPI_ISL_1086085 to 1086091, EPI_ISL_1086093 to 1086094, EPI_ISL_1086096, EPI_ISL_1086098, EPI_ISL_1086100 to 1086102, EPI_ISL_1086104 to 1086106, EPI_ISL_1086108 to 1086109, EPI_ISL_1086111 to 1086112, EPI_ISL_1086114, EPI_ISL_1086116 to 1086118, EPI_ISL_1086121 to 1086122, EPI_ISL_1086131, EPI_ISL_1086133 to 1086142, EPI_ISL_1086144 to 1086146, EPI_ISL_1086149 to 1086150, EPI_ISL_1086153 to 1086155, EPI_ISL_1086157 to 1086160, EPI_ISL_1086163 to 1086166, EPI_ISL_1086168 to 1086173, EPI_ISL_1086175, EPI_ISL_1086177, EPI_ISL_1086179 to 1086188, EPI_ISL_1086190 to 1086195, EPI_ISL_1086198 to 1086214, EPI_ISL_1086217, EPI_ISL_1185704 to 1185708, EPI_ISL_1185711 to 1185714, EPI_ISL_1185716 to 1185717, EPI_ISL_1196955 to 1196970, EPI_ISL_1196986, EPI_ISL_1196988 to 1197005, EPI_ISL_1197013 to 1197036			
see above	IZSM	TIGEM	Andrea Ballabio; Anna Manfredi; Antonio Grimaldi; Antonio Grimaldi Patrizia Annunziata Francesco Panariello Biancamaria Pierri Valentina Bouche Chiara Colantuono Maria Concetta Cuomo Denise Di Concilio Lucio Di Filippo Anna Manfredi Marcello Salvi Antonio Limone Pellegrino Cerino Andrea Ballabio Davide Cacchiarelli; Antonio Limone; Biancamaria Pierri; Chiara Colantuono; Davide Cacchiarelli.; Denise Di Concilio; Francesco Panariello; Lucio Di Filippo; Marcello Salvi; Maria Concetta Cuomo; Patrizia Annunziata; Pellegrino Cerino; Valentina Bouche
EPI_ISL_738121 to 738132	IZSM-U.O.C. Virologia	Istituto Zooprofilattico Sperimentale del Mezzogiorno	Giovanna Fusco; Lorena Cardillo; Maurizio Viscardi
EPI_ISL_918377 to 918406, EPI_ISL_1122349 to 1122356	Ibaraki Prefectural Institute of Public Health	Ibaraki Prefectural Institute of Public Health	Keiko Goto; Kentaro Itokawa; Makoto Kuroda; Masanori Hashino; Rina Tanaka; Tsuyoshi Sekizuka
EPI_ISL_479936 to 479943, EPI_ISL_479985, EPI_ISL_480021 to 480029, EPI_ISL_480180 to 480189	Ibaraki Prefectural Institute of Public Health	Pathogen Genomics Center, National Institute of Infectious Diseases	Hajime Kamiya; Keiko Goto; Kentaro Itokawa; Makoto Kuroda; Masanori Hashino; Motoi Suzuki; Rina Tanaka; Tsuyoshi Sekizuka
EPI_ISL_683657 to 683677, EPI_ISL_779715 to 779729, EPI_ISL_779738 to 779739, EPI_ISL_779741 to 779752, EPI_ISL_779754 to 779773, EPI_ISL_855402 to 855408, EPI_ISL_962914			
see above	IdISSC/Hospital Clínico San Carlos de Madrid	SeqCOVID-SPAIN consortium/IBV(CSIC)	Alberto Delgado-Iribarren; Esther Culebras Lopez; Esther Culebras López; Jorge Matias-Guiu; Luis Ortega Medina; Silvia Sánchez Ramón; Ulises Gómez-Pinedo and SeqCOVID-SPAIN consortium; Vicente Estrada Pérez
EPI_ISL_632847 to 632889, EPI_ISL_1190729 to 1190737	Idaho Bureau of Laboratories	Center for Global Health, University of New Mexico Health Sciences Center	Christopher Ball; Darrell Dinwiddie; Daryl Domman; Kurt Schwalm; Matthew Burns; Robert Voermans

EPI_ISL_1164782 to 1164793, EPI_ISL_1164795 to 1164815	Idaho Bureau of Laboratories	IBL	"R. Beukelman; Aimee Ceniseros; Christopher Ball"; Matthew C. Burns; Robert L. Voermans
EPI_ISL_1219911 to 1219923	Idaho Bureau of Laboratories	Idaho Bureau of Laboratories	"R. Beukelman; Aimee Ceniseros; Christopher Ball"; Matthew Charles Burns; Robert L. Voermans
EPI_ISL_848203 to 848545, EPI_ISL_961883 to 962178, EPI_ISL_1032667 to 1032945, EPI_ISL_1138425 to 1138511, EPI_ISL_1225736 to 1225779	Illinois Department of Public Health	Gagnon Lab, Southern Illinois University	Keith Gagnon
EPI_ISL_420789 to 420790	Illinois Department of Public Health Chicago Laboratory	Pathogen Discovery, Respiratory Viruses Branch, Division of Viral Diseases, Centers for Disease Control and Prevention	Alison S. Laufer Halpin; Anne Uehara; Christopher A. Elkins; Clinton R. Paden; Haibin Wang; Jasmine Padilla; Jing Zhang; Justin Lee; Krista Queen; Mary S. Keckler; Rachel Marine; Suxiang Tong; Yan Li; Ying Tao
EPI_ISL_1013094, EPI_ISL_1013096, EPI_ISL_1013594 to 1013595, EPI_ISL_1013771, EPI_ISL_1169896 to 1169904, EPI_ISL_1173355, EPI_ISL_1173509, EPI_ISL_1173517 to 1173518, EPI_ISL_1173766 to 1173768, EPI_ISL_1176298, EPI_ISL_1176300, EPI_ISL_1176303			
see above	Imelda Hospital	Imelda Hospital	Dagmar Obbels; Hanne Valgaeren; Hanne valgaeren; Johan Frans
EPI_ISL_1252479 to 1252484, EPI_ISL_1265169	Imelda hospital	Imelda hospital Bonheiden	Dagmar Obbels; Hanne Valgaeren; Johan Frans
EPI_ISL_1281680, EPI_ISL_1281682, EPI_ISL_1281686, EPI_ISL_1281688, EPI_ISL_1281690, EPI_ISL_1281692	Imelda hospital Bonheiden	Imelda hospital Bonheiden	Dagmar Obbels; Hanne Valgaeren; Johan Frans
EPI_ISL_528746	Immanuel Hospital	Molecular Genetics Laboratory-Faculty of Medicine-Universitas Padjadjaran; School of Life Sciences and Technology & School of Pharmacy-Institut Teknologi Bandung; Laboratorium Kesehatan Provinsi Jawa Barat	Agung Eru Wibowo; Azzania Fibriani; Catur Riani; Cut Nur Cinthia Alamanda; Ema Rahmawati; Fensi Amalina; Hammam Riza; Hesti Lina Wiraswati; Husna Nugrahapraja; Irvan Faizal; Lia Faridah; Marselina Irasonia Tan; Mas Rizky A.A. Syamsunarno; Rifky Waluyajati Rachman; Ryan Bayusantika Ristandi; Savira Ekawardhani; Sony Solistia Wirawan; Tarwadi; Tri Hanggono Achmad; Yunia Sribudiani
EPI_ISL_481110 to 481205, EPI_ISL_1063796 to 1063805, EPI_ISL_1063808, EPI_ISL_1063810 to 1063839, EPI_ISL_1063862 to 1063896, EPI_ISL_1063899	Immunogenomics lab, Institute of Life Sciences, Bhubaneswar	Immunogenomics lab, Institute of Life Sciences, Bhubaneswar	Aditi Chatterjee; Ajay Parida; Ajit Singh; Amol M. Kanampalliwar; Amol Ratnakar Suryawanshi; Ankita Datey; Arup Ghosh; Atimukta Jha; Auromira Khuntia; Bharati Singh; DBT's PAN-INDIA 1000 SARS-CoV2 RNA genome sequencing consortium; Deepika Singh; Dileep Vasudeva; Dileep Vasudevan; Ghulam Hussain Syed; INSACOG Consortium; Kirtal Hansdah; Manasi Priyadarshini; Naga Jogayya Kothakota; Neha Singh; Orissa COVID-19 Study Group; P. Sushree Shyamli; Punit Prasad; Rahul Das; Rajeeb Swain; Rupesh Dash; Safal Walia; Shanti Senapati; Shifu Aggarwal; Sivaram Krishna; Sneha Dutta; Soma Chattopadhyay; Soumyajit Ghosh; Sunil K. Raghav; Sunil Raghav; Swati Madhulika; Tsheten Sheroa; Tushar K. Beuria; Viplov K. Biswas
EPI_ISL_1018071 to 1018101	Immunology, Noguchi Memorial Institute for Medical Research	Immunology, Noguchi Memorial Institute for Medical Research	Adu, B.; Adusei-Poku; Agbodzi, B.; Ampofo; Appiah-Kubi, J.; Asare; Bonney; Egyir, B.; J.K.; K.M.; Kumordjie, S.; M.A.; Mohktar, Q.; Odoom; Oteng, F.; Owusu-Nyantakyi, C.; W.K.; Yeboah, C.
EPI_ISL_475572	Imperial College London	Imperial College London	Jie Zhou; Wendy Barclay
EPI_ISL_476702 to 476704	Incubadora Venezolana de Ciencia, Venezuela	Incubadora Venezolana de Ciencia, Venezuela / Instituto Nacional de Salud, Bogotá, Colombia / Grupo de Investigaciones Microbiológicas-UR (GIMUR), Departamento de Biología, Facultad de Ciencias Naturales, Universidad del Rosario, Bogotá, Colombia / Icahn School of Medicine at Mount Sinai, New York, USA	Alberto Paniz-Mondolfi; Ana S. Gonzalez-Reiche; Angelica Rico; Aníbal A. Teherán; Carolina Florez; Carolina Hernández; Emilia Mia Sordillo; Esther C. Barros; Harm van Bakel; Jesús E. Jaimes; Juan David Ramírez; Lisseth Pardo; Lourdes Delgado; Luis Perez-Garcia; Marina Muñoz; Matthew M. Hernandez; Sergio Gomez; Viviana Simon
EPI_ISL_413522	Indian Council of Medical Research - National Institute of Virology	National Influenza Center, Indian Council of Medical Research - National Institute of Virology	Choudhary ML; Potdar V; Shete-Aich A; Yadav PD
EPI_ISL_413523	Indian Council of Medical Research-National Institute of Virology	National Influenza Center, Indian Council of Medical Research-National Institute of Virology	Choudhary ML; Potdar V; Shete-Aich A; Yadav PD
EPI_ISL_1201048 to 1201058, EPI_ISL_1201061 to 1201127, EPI_ISL_1251262 to 1251263	Indian Council of Medical Research-National Institute of Virology, Kerala Unit	Indian Council of Medical Research-National Institute of Virology, Microbial Containment Complex	A.P Sugunan; Pragya D. Yadav
EPI_ISL_420544, EPI_ISL_420546, EPI_ISL_420548, EPI_ISL_420550, EPI_ISL_420552, EPI_ISL_420554, EPI_ISL_420556			
see above	Indian Council of Medical Research-National Institute of Virology, Microbial Containment Complex	Indian Council of Medical Research-National Institute of Virology, Microbial Containment Complex	Amita Jain; Anita M. Shete; Atanu Basu; Bharati Malhotra; Dimpal A. Nyayanit; Gajanan Sapkal; Lalit Dar; M Choudhary; Pragya D. Yadav. Savita Patil; Pranita Gawande; Prasad Sarkale; Priya Abraham; Sarah Cherian; Varsha Potdar
EPI_ISL_825084, EPI_ISL_825086 to 825089	Indian Council of Medical Research-National Institute of Virology,Maximum Containment Laboratory	Indian Council of Medical Research-National Institute of Virology,Maximum Containment Laboratory	Deepak Y Patil; Dimpal A Nyayanit; Jayshri Pethani; Pragya D Yadav; Prasad Sarkale; Rima R Sahay; Savita Patil; Shrikant Baradkar; Varsha Potdar
EPI_ISL_1173175 to 1173201	Indian Council of Medical Research-National Institute of Virology, Banglore Unit	Indian Council of Medical Research-National Institute of Virology, Microbial Containment Complex	Ashok Munivenkatappa; Pragya D. Yadav
EPI_ISL_637099 to 637104, EPI_ISL_1061417, EPI_ISL_1061426, EPI_ISL_1061428, EPI_ISL_1061433 to 1061434	Indian Council of Medical Research-National Institute of Virology, Microbial Containment Complex	Indian Council of Medical Research-National Institute of Virology, Microbial Containment Complex	Anita Shete-Aich; Dimpal A. Nyayanit; Gururaj Rao Deshpande; Padinjaremattathil Thankappan Ullas; Pragya D. Yadav; Prasad Sarkale; Priya Abraham; Varsha Potdar
EPI_ISL_969397, EPI_ISL_969509, EPI_ISL_969740	Indian Council of Medical Research-National Institute of Virology, Pune	Indian Council of Medical Research-National Institute of Virology, Maximum Containment Laboratory	Abhinendra Kumar; Anita Shete-Aich; Dimpal A. Nyayanit; Pragya D. Yadav; Savita Patil; Triparna D. Majumdar
EPI_ISL_1129275, EPI_ISL_1129277 to 1129278	Indian Council of Medical Research-National Institute of Virology, Pune	Indian Council of Medical Research-National Institute of Virology, Microbial Containment Complex	Pragya D. Yadav
EPI_ISL_508287 to 508337	Indian Institute of Science	National Institute of Biomedical Genomics	Amit Singh; Arindam Maitra; Bharath K Sundararaj; Deepak K Saini; Harsha Raheja; N. Srinivasan; Saumitra Das
EPI_ISL_511945 to 511954, EPI_ISL_518031 to 518032	Indian Institute of Science	National Institute of Biomedical Genomics - DBT's PAN-INDIA 1000 SARS--CoV-2 RNA Genome Sequencing Consortium	Amit Singh; Arindam Maitra; Bharath K Sundararaj; Deepak K Saini; Harsha Raheja; N. Srinivasan; Saumitra Das
EPI_ISL_891141 to 891144, EPI_ISL_893214, EPI_ISL_955212 to 955228, EPI_ISL_1184169 to 1184219, EPI_ISL_1255279 to 1255295	Indiana Animal Disease Diagnostic Laboratory	Carpi Laboratory - Purdue University	Abebe A Fola; G Kenitra Hendrix; Giovanna Carpi; Ilinca I Ciubotariu; Jack Dorman; Jobin J Kattoor; Lev Gorenstein; Nicole M Perry; Rebecca P Wilkes
EPI_ISL_647986	Indiana State Department of Health	Pathogen Discovery, Respiratory Viruses Branch, Division of Viral Diseases, Centers for Disease Control and Prevention	Anna Montmayeur; Anna Uehara; Brian Lynch; Clinton R. Paden; Haibin Wang; Jing Zhang; Krista Queen; Rachel Marine; Suxiang Tong; Yan Li; Ying Tao
EPI_ISL_1192076, EPI_ISL_1192092, EPI_ISL_1192098 to 1192099, EPI_ISL_1192135 to 1192148	Indira Gandhi Medical College & Hospital, Shimla	Indian Council of Medical Research-National Institute of Virology, Microbial Containment Complex	Pragya D. Yadav; Santwana Verma
EPI_ISL_755587	Infection and Immunology, Translational Health Science and Technology Institute	Infection and Immunology, Translational Health Science and Technology Institute	A. and Medigeshi; A.K.; Das; G.R.; Kumar, S.; Pandey; Wangchuk, J.
EPI_ISL_936487	Infectious Disease Biology, Institute of Life Sciences	Infectious Disease Biology, Institute of Life Sciences	Avula, K.; Beuria; Biswas; Chattopadhyay, S.; Das, R.; Datey, A.; G.H.; Ghosh, A.; I.C. and Parida, A.; Jha, A.; Laha, E.; Madhulika, S.; Prasad, P.; Priyadarhini, M.; Puwan; Raghav, S.; S.S.; Senapati, S.; Singh, B.; Singh, D.; Singh, N.; Swain, R.; Syed; T.K.; Team; V.K.
EPI_ISL_413518 to 413521, EPI_ISL_514752	Infectious Disease Control Center, Center for Disease Control and Prevention of PLA	Infectious Disease Control Center, Center for Disease Control and Prevention of PLA	Li; Li, J.; Li, L.; Li, P.; Li, Z.; P. and Li, P.; Qiu, S.; Song, H.

EPI_ISL_962528 to 962584, EPI_ISL_962868 to 962875	Infectious Disease Control and Prevention Institute	Infectious Disease Control and Prevention Institute	Bo Pang; Jianxing Wang; Julong Wu; Mingxiao Yao; Ti Liu; Xiaolin Jiang; Yan Li; Yujie He; Yuwei Zhang; Zengqiang Kou
EPI_ISL_450437	Infectious Disease Hospital, Central Laboratory	Infectious Disease Hospital, Central Laboratory	Yan, Y.
EPI_ISL_596454	Infectious Disease and Tropical Medicine Research Center, Resistant Tuberculosis Institute, Zahedan University of Medical Sciences, Zahedan, Iran.	Genetics Research Center, University of Social Welfare and Rehabilitation Sciences	Ali Jafarpour; Azam Ghaziasadi; Ebrahim Kord; Hossein Najmabadi; Khadijeh Jalalvand; Kimia Kahrizi; Marzieh Mohseni; Seyed Mohammad Hashemi-Shahri; Seyed Mohammad Jazayeri; Seyedeh elham Mortazavi; Zohreh Fattahi
EPI_ISL_1229134 to 1229135	Infectious Diseases Clinic, Udine University Hospital	INMI Lazzaro Spallanzani IRCCS	A Di Caro; B Bartolini; C Tascini; CEM Gruber; D Pecori; E Giombini; F Messina; M Rueca; MR Capobianchi; O Butera
EPI_ISL_856712 to 856721	Infectious Diseases Unit, Department of Internal Medicine, Azienda Ospedaliera-Universitaria di Padova	Laboratory of Infectious Diseases, Department of Biomedical and Clinical Sciences L. Sacco, University of Milan	Alessia Lai; Anna Maria Cattelan; Annalisa Bergna; Carla Della Ventura; Claudia Balotta; Davide Leoni; Gianguglielmo Zehender on behalf of SARS-CoV-2 ITALIAN RESEARCH ENTERPRISE-(SCIRE) Collaborative Group; Lolita Sasset; Massimo Galli
EPI_ISL_577606, EPI_ISL_582003 to 582016, EPI_ISL_591059 to 591065, EPI_ISL_591341 to 591346, EPI_ISL_660111 to 660118, EPI_ISL_672575 to 672607	Infectious Diseases and Tropical Medicine Research Center, Infectious Diseases and Tropical Medicine Research Center	Infectious Diseases and Tropical Medicine Research Center, Infectious Diseases and Tropical Medicine Research Center	Aboutalebian, S.; Abutalebian, S.; Ahangarzadeh, S.; Ataei, B.; Haghjoo Javanmard, S.; Haghjoo javanmard, S.; Haghjooy Javanmard, S.; Haghjooy javanmard, S.; M.M.; Ranjbar; Shariati, L.; Shariati, l.; Shoaei, P.
EPI_ISL_1233893 to 1233894	Infectious Diseases, Chan-Zuckerberg Biohub Covidtracker	Infectious Diseases, Chan-Zuckerberg Biohub Covidtracker	CZB Cliahub Consortium
EPI_ISL_825028 to 825032	Infectious Diseases, NC SLPH COVID-19 Response Team	Infectious Diseases, NC SLPH COVID-19 Response Team	Chase, K.; M.C.; Miller
EPI_ISL_538514 to 538521	Infectious Diseases, North Carolina State Laboratory of Public Health COVID-19 Response Team	Infectious Diseases, North Carolina State Laboratory of Public Health COVID-19 Response Team	Chase, K.
EPI_ISL_538522 to 538523	Infectious Diseases, North Carolina State Laboratory of Public Health COVID-19 Response Team	North Carolina State Laboratory of Public Health	Chase, K.
EPI_ISL_884303 to 884444, EPI_ISL_906920 to 907074, EPI_ISL_1233901 to 1233903, EPI_ISL_1233919 to 1233921, EPI_ISL_1233924	Infectious Diseases, Quest Diagnostics	Infectious Diseases, Quest Diagnostics	Anderson, B.; Bernstein; D.F.; Gerasimova, A.; Hua, M.; I.A.; K.E.; Kagan; L.E.; Lacbawan, F.; Liu, Y.; Livingston; Owen, R.; Perez, A.; R.M.; Rosenthal; S.H.; Shalhout; Shlyakhter; Tanpaiboon, P.
EPI_ISL_649152	Infectious diseases, Chan Zuckerberg Biohub	Infectious diseases, Chan Zuckerberg Biohub	Bhatt; K.D.
EPI_ISL_496339 to 496376	Infectolab	Andersen lab at Scripps Research	Carlos A. Cota Haros; Octavio Renteria Pacheco; SEARCH Alliance San Diego with Samuel Navarro Alvarez
EPI_ISL_541970	Influenza Centre, University of Bergen	Norwegian Institute of Public Health, Department of Virology	Bjørn Blomberg; Fan Zhou; Hilde Elshaug; Hilde Synnøve Vollan; Kamilla Heddeland Instefjord; Karl A Brokstad; Karoline Bragstad; Kathrine Stene-Johansen; Olav Hungnes; Rasmus Riis Kopperud; Rebecca J Cox
EPI_ISL_1240719 to 1240725	Influenza and respiratory viruses	Human virology Department	A.Bensalem; A.Hachid; F.Derrar; F.Khardine; M.A.Beloufa
EPI_ISL_1093429	Influenza and respiratory viruses Lab. Pasteur Institute in Algeria	Human Virology Department	A.Hachid; F.A.Khardine; F.Derrar; M.A.Beloufa
EPI_ISL_1093427	Influenza and respiratory viruses lab. Pasteur Institute in Algeria	Human virology Department	A.Hachid; F.A.Khardine; F.Derrar; M.A. Beloufa
EPI_ISL_1093428	Influenza and respiratory viruses. Pasteur Institute in Algeria	Human virology deprtment	A.Hachid; F.A.Khardine; F.Derrar
EPI_ISL_1093430	Influenza and respiratory viurses Lab. Pasteur Institute in Algeria	Human Virology Department	A.Hachid; F.A.Khardine; F.Derrar; M.A.Beloufa
EPI_ISL_470900 to 470904, EPI_ISL_872641 to 872642, EPI_ISL_875515	Influenza etiology and epidemiology laboratory	Pathogenic Microorganisms Variability Laboratory	Alexander Gintsburg; Alexey Shchetinin; Andrei Siniavin; Andrey Botikov; Andrey Pochtovyy; Anna Ignatieva; Anna Ignatjeva; Daria Grousova; Denis Logunov; Elena Burtseva; Elena Shidlovskaya; Elizaveta Divisenko; Evgeniya Mukasheva; Evgeny Usachev; Inna Dolzhikova; Kirill Krasnoslobotsev; Ludmila Kolobukhina; Maria Nikiforova; Nadezhda Kuznetsova; Olesya Venchakova; Olga Burgasova; Svetlana Smetanina; Svetlana Trushakova; Valeria Bacalin; Vladimir Gushchin
EPI_ISL_469057	Inger Landgren	The Public Health Agency of Sweden	Anna Risberg; Anna-Malin Linde; Karin Tegmark-Wisell; Maria Lind Karlberg; Mattias Haukland; Olov Svartstrom; Oskar Karlsson Lindsjo; Petra Edquist; Reza Advani; Sandra Broddesson; Shamam Muradrasoli
EPI_ISL_635124 to 635125, EPI_ISL_635130 to 635131, EPI_ISL_635183, EPI_ISL_668412 to 668416, EPI_ISL_708030 to 708031, EPI_ISL_708036 to 708039, EPI_ISL_708151 to 708153, EPI_ISL_775440 to 775441, EPI_ISL_860226 to 860228, EPI_ISL_860291 to 860292, EPI_ISL_906793, EPI_ISL_906796, EPI_ISL_906812, EPI_ISL_964996 to 964997, EPI_ISL_1034327, EPI_ISL_1040157, EPI_ISL_1058059, EPI_ISL_1073948, EPI_ISL_1118308, EPI_ISL_1118565, EPI_ISL_1118577, EPI_ISL_1118583, EPI_ISL_1118588 to 1118589, EPI_ISL_1118598 to 1118599, EPI_ISL_1118609, EPI_ISL_1118656 to 1118657, EPI_ISL_1118669 to 1118670, EPI_ISL_1192166, EPI_ISL_1192189, EPI_ISL_1251360 to 1251362, EPI_ISL_1251370 to 1251376, EPI_ISL_1251398, EPI_ISL_1251408 to 1251415, EPI_ISL_1251532, EPI_ISL_1251585, EPI_ISL_1251587, EPI_ISL_1251589, EPI_ISL_1251776 to 1251779, EPI_ISL_1251786, EPI_ISL_1254537, EPI_ISL_1254579, EPI_ISL_1314540			
see above	Innlandet Hospital Trust, Division Lillehammer, Department for Medical Microbiology	Norwegian Institute of Public Health, Department of Virology	Atiya R Ali; Debech Nadia; Engebretsen Serina Beate; Engebretsen Serina Beate Atiya R Ali; Garcia Llorente Ignacio; Hilde Elshaug; Hilde Vollan; Ignacio Garcia Llorente; Jon Bråte; Kamilla Heddeland Instefjord; Karoline Bragstad; Kathrine Stene-Johansen; Marie Paulsen Madsen; Olav Hungnes; Pedersen Benedikte Nevjen; Rasmus Riis Kopperud; Serina B Engebretsen
EPI_ISL_1120708, EPI_ISL_1168682 to 1168685	Inno Diagnostics/Center for Research Resources, Ponce Medical School Foundation, Inc.	Centers for Disease Control and Prevention, Dengue Branch	Andrea Arias-Garcia; Betzabel Flores; Gabriela Paz-Bailey; Gilberto A. Santiago; Glenda Gonzalez; Jorge L. Munoz-Jordan; Keyla Charriez; Raphael Sanchez-Torres; Vanessa Rivera-Amill
EPI_ISL_467946 to 467949, EPI_ISL_495122 to 495160, EPI_ISL_511805 to 511851, EPI_ISL_511862, EPI_ISL_569887 to 569948, EPI_ISL_576518 to 576556, EPI_ISL_677821 to 677907, EPI_ISL_754857 to 754901, EPI_ISL_756312 to 756356, EPI_ISL_918177 to 918262, EPI_ISL_955126 to 955129, EPI_ISL_980840 to 981003, EPI_ISL_984659 to 984662, EPI_ISL_1041034 to 1041053, EPI_ISL_1041055 to 1041107, EPI_ISL_1041181 to 1041194, EPI_ISL_1049262 to 1049337, EPI_ISL_1060040 to 1060191, EPI_ISL_1081284 to 1081290			
see above	Innovative Genomics Institute, UC Berkeley	Innovative Genomics Institute, UC Berkeley	Alison Ciling; Haridha Shivram; Justin Choi; Liana Lareau; Netravathi Krishnappa; Phil Frankino; Shana McDevitt; Stacia Wyman
EPI_ISL_466648	Innovative Genomics Institute, UCB	Innovative Genomics Institute, UCB	Haridha Shivram; Justin Choi; Liana Lareau; Shana McDevitt; Stacia Wyman
EPI_ISL_418215	Instirut Pasteur Dakar	Institut Pasteur de Dakar	Amadou Alpha Sall; Ndongo Dia; Ousmane Faye
EPI_ISL_811130	Institut Central des Hôpitaux Valaisans ICHV/ZIWS Service des Maladies Infectieuses	Department of Biosystems Science and Engineering, ETH Zürich	Andrea Patrignani; Andreia Cabral de Gouvea; Catharine Aquino; Chaoran Chen; Christiane Beckmann; Christoph Noppen; David Dreifuss; Doris Popovic; Griffin White; Ivan Topolsky; Jay Tracy; Katharina Jahn; Lara Fuhrmann; Laura Neff; Lennart Opitz; Maria Domenica Moccia; Maurice Redondo; Niko Beerenwinkel; Noemie Santamaria de Souza; Olivier Kobel; Philipp Jablonski; Ralph Schlapbach; Sarah Nadeau; Simon Grüter; Sophie Seidel; Tanja Stadler; Timothy Sykes
EPI_ISL_416498, EPI_ISL_428355 to 428357	Institut Médico légal- Hop R. Poincaré	National Reference Center for Viruses of Respiratory Infections, Institut Pasteur, Paris	Angela Brisebarre; Etienne Simon-Lorière; Flora Donati; Marion Barbet; Maud Vanpeene; Mélanie Albert; Méline Bizard; Mélnie Albert; Sylvie Behillil; Sylvie van der Werf; Vincent Enouf
EPI_ISL_984270, EPI_ISL_984273, EPI_ISL_984308	Institut National d'hygiène	"Unité Mixte Internationale TransVIHMI (UMI 233 IRD - U1175 INSERM - Université de Montpellier) IRD (Institut de recherche pour le développement)"	Abla A. KONOU; Adodo SADJI; Ahidjo AYOUBA; Akoélé SILIADIN; Alassane OURO-MEDELI; Amivi EHLAN; Améyo DORKENOO; Anoumou DAGNRA; Christelle BUTEL; Déléma MABA; Eric DELAPORTE; Issaka Maman; Kokou TEGUENI; Laetitia SERRANO; Martine PEETERS; Messanh DOUFFAN; Mireille PRINCE-DAVID; Mounerou SALOU; Sidonie A.M.KAGNISSODE; Sika DOSSIM; Wembo A. HALATOKO
EPI_ISL_982526	Institut National d'Hygiène (INH)	Unité Mixte Internationale TransVIHMI (UMI 233 IRD - U1175 INSERM - Université de Montpellier)IRD (Institut de recherche pour le développement)	Ahidjo Ayouba; Ahouefa Abla Konou; Amivi Ehlan; Anoumou DAGNRA; Christelle Butel; Eric DELAPORTE; Issaka Maman; Kokou Tegueni; Martine PEETERS; Mounerou SALOU; Nicole VIDAL; Wemboo Afiwa Halatoko
EPI_ISL_1293351, EPI_ISL_1303031	Institut National d'Hygiène (INH)	Unité Mixte Internationale TransVIHMI (UMI 233 IRD - U1175 INSERM - Université de Montpellier)IRD (Institut de recherche pour le développement)	Abla A. KONOU; Adodo SADJI; Ahidjo AYOUBA; Akoélé SILIADIN; Alassane OURO-MEDELI; Amivi EHLAN; Améyo DORKENOO; Anoumou DAGNRA; Christelle BUTEL; Déléma MABA; Eric DELAPORTE; Issaka Maman; Kokou TEGUENI; Laetitia SERRANO; Martine PEETERS; Messanh DOUFFAN; Mireille PRINCE-DAVID; Mounerou SALOU; Sidonie A.M.KAGNISSODE; Sika DOSSIM; Wembo A. HALATOKO
EPI_ISL_485712	Institut Pasteur	Institut Pasteur de Dakar	Amadou Alpha Sall.; Mamadou Malado Jallow; Mamadou diop; Marie Henriette Dior Ndione; Moussa Moise Diagne; Ndongo Dia; Ousmane Faye;

			Safietou Sanke
EPI_ISL_418206 to 418214, EPI_ISL_418216 to 418217, EPI_ISL_420069 to 420074, EPI_ISL_420076 to 420079, EPI_ISL_476148 to 476151, EPI_ISL_476491 to 476495, EPI_ISL_476497, EPI_ISL_476514 to 476516, EPI_ISL_476558, EPI_ISL_476560, EPI_ISL_476562, EPI_ISL_476564, EPI_ISL_476569 to 476570, EPI_ISL_476572, EPI_ISL_476574, EPI_ISL_477141 to 477159, EPI_ISL_480554, EPI_ISL_480556, EPI_ISL_480782 to 480783, EPI_ISL_480786 to 480789, EPI_ISL_481220, EPI_ISL_481234 to 481240, EPI_ISL_481243, EPI_ISL_482874 to 482878, EPI_ISL_485635, EPI_ISL_485708, EPI_ISL_485710 to 485711, EPI_ISL_485713, EPI_ISL_485715 to 485717, EPI_ISL_486857 to 486873			
see above	Institut Pasteur Dakar	Institut Pasteur de Dakar	Amadou Alpha Sall; Amadou Alpha Sall.; Amadou Alpha sall; Amadou alpha Sall; Mamadou Diop; Mamadou Malado Jallow; Mamadou diop; Mamadou malado Jallow; Marie Henriette Dior Ndione; Moussa Moise; Moussa Moise Diagne; Ndongo Dia; Ousmane Faye; Ousmane faye; Safietou Sanke; Safietou Sankhe; mamadou Diop
EPI_ISL_498229 to 498252	Institut Pasteur de Dakar	Institut Pasteur de Dakar	Amadou Alpha Sall.; Mamadou Diop; Mamadou Malado Jallow; Marie Henriette Dior Ndione; Moussa Moise Diagne; Ndongo Dia; Ousmane Faye; Safietou Sankhe Mbengue
EPI_ISL_1013214 to 1013216, EPI_ISL_1013236, EPI_ISL_1013417 to 1013436	Institut Pasteur de Guadeloupe	National Reference Center for Viruses of Respiratory Infections, Institut Pasteur, Paris	Angela Brisebarre; Camille Capel; Etienne Simon-Lorière; Marion Barbet; Maud Vanpeene; Méline Bizard; Sylvie Behillil; Sylvie van der Werf; Talarmin Antoine; Vincent Enouf
EPI_ISL_999030, EPI_ISL_999032	Institut Pasteur de Guinée	Institut Pasteur de Dakar	Dia Ndongo; Diagne Moussa Moïse; Diallo Amadou; Diop Mamadou; Faye Ousmane; Grayo Solene; Loucoubar Cheikh; Mbengue Safietou Sankhe; Ndiaye Ndack; Sall Amadou Alpha; Tordo Noel
EPI_ISL_613419 to 613456	Institut Pasteur de la Guadeloupe	Institut Pasteur de la Guadeloupe	Angela Brisebarre; Antoine Talarmin; Camille Capel; Cherina Fleming; Etienne Simon-Lorière; Marion Barbet; Maud Vanpeene; Méline Bizard; Radjin Steingrover; Stéphanie Guyomard; Sylvie Behillil; Sylvie van der Werf; Sébastien Breurec; Vincent Enouf
EPI_ISL_459965 to 459984	Institut Pasteur du Maroc	Institut Pasteur du Maroc	Abdellah Faouzi; Anass Abbad; Anderrahmane Maaroufi; Angela Brisebarre; Camille Capel; Etienne Simon-Lorière; Jalal Nourlil; Latifa Anga; Marion Barbet; Maud Vanpeene; Mjid Eloualid; Méline Bizard; Sylvie Behillil; Sylvie van der Werf; Vincent Enouf
EPI_ISL_965146	Institut de Pathologie et Genetique	Institut de Pathologie et Genetique	Jeremie Gras; Pascale Hilbert
EPI_ISL_965141, EPI_ISL_965144	Institut de Pathologie et Génétique	Institut de Pathologie et Génétique	Jeremie Gras; Pascale Hilbert
EPI_ISL_1173508, EPI_ISL_1176297, EPI_ISL_1176305, EPI_ISL_1176307, EPI_ISL_1186115, EPI_ISL_1186199, EPI_ISL_1186205 to 1186208, EPI_ISL_1186212, EPI_ISL_1186216, EPI_ISL_1196764, EPI_ISL_1196766 to 1196767, EPI_ISL_1196769, EPI_ISL_1196772 to 1196773, EPI_ISL_1196799, EPI_ISL_1196865, EPI_ISL_1205461 to 1205463, EPI_ISL_1205468 to 1205470, EPI_ISL_1205486, EPI_ISL_1205488, EPI_ISL_1205699, EPI_ISL_1206480, EPI_ISL_1208665, EPI_ISL_1208950, EPI_ISL_1208953 to 1208955, EPI_ISL_1209015 to 1209016, EPI_ISL_1209019, EPI_ISL_1222775 to 1222778, EPI_ISL_1246958, EPI_ISL_1246961, EPI_ISL_1246965 to 1246970, EPI_ISL_1257812 to 1257813, EPI_ISL_1257815, EPI_ISL_1293331, EPI_ISL_1293338 to 1293339, EPI_ISL_1293343, EPI_ISL_1293345 to 1293349, EPI_ISL_1293352, EPI_ISL_1293354, EPI_ISL_1293356, EPI_ISL_1293642, EPI_ISL_1295013, EPI_ISL_1295177 to 1295178, EPI_ISL_1295180, EPI_ISL_1307687 to 1307689, EPI_ISL_1307691 to 1307694, EPI_ISL_1307696 to 1307701, EPI_ISL_1307704, EPI_ISL_1307707, EPI_ISL_1307710 to 1307714			
see above	Institut de Virologie du CHU de Strasbourg	Institut de Virologie du CHU de Strasbourg	Fafi-Kremer Samira; Gallais Floriane; Gantner Pierre; Laugel Elodie; Solis Morgane; Velay Aurélie; Wendling Maire-Josée; Wendling Marie-Josée
EPI_ISL_416747 to 416748, EPI_ISL_416750, EPI_ISL_416753 to 416754, EPI_ISL_416756, EPI_ISL_416758	Institut des Agents Infectieux (IAI) Hospices Civils de Lyon	CNR Virus des Infections Respiratoires - France SUD	Alexandre; Antonin; Bal; Bouscambert-Duchamp; Brengel-Pesce; Bruno.; Cheynet; Destras; Florence; Gaymard; Gregory; Josset; Karen; Laurence; Lina; Martine; Maude; Morfin-Sherpa; Valette; Valérie
EPI_ISL_417333 to 417337, EPI_ISL_417339, EPI_ISL_418420 to 418425, EPI_ISL_418429 to 418432, EPI_ISL_419169 to 419173, EPI_ISL_419177 to 419182, EPI_ISL_419184, EPI_ISL_420604 to 420611, EPI_ISL_420615 to 420616, EPI_ISL_420618 to 420619, EPI_ISL_420621 to 420625, EPI_ISL_508871 to 508874, EPI_ISL_508877, EPI_ISL_508882 to 508911, EPI_ISL_509007 to 509011, EPI_ISL_509013 to 509014			
see above	Institut des Agents Infectieux (IAI), Hospices Civils de Lyon	CNR Virus des Infections Respiratoires - France SUD	Alexandre Gaymard; Antonin Bal; Bruno Lina; Carine Moustaud; Florence Morfin-Sherpa; Gregory Destras; Gwendolyne Burfin; Laurence Josset; Martine Valette; Maude Bouscambert-Duchamp; Raphaelle Lamy; Solenne Brun
EPI_ISL_1140522 to 1140524, EPI_ISL_1140529 to 1140533, EPI_ISL_1144363, EPI_ISL_1148474, EPI_ISL_1148476, EPI_ISL_1148504, EPI_ISL_1217519 to 1217520, EPI_ISL_1284532, EPI_ISL_1284544			
see above	Institut für Immunologie und Genetik Kaiserslautern: Medizinisches Labor Dr. med. B. Thiele	Robert Koch Institute	
EPI_ISL_1149299, EPI_ISL_1153471, EPI_ISL_1214986	Institut für Klinische Chemie Transfusions- und LaboratoriumsmedizinBerufsgenossenschaftliches Universitätsklinikum Bergmannsheil	Robert Koch Institute	
EPI_ISL_1143653, EPI_ISL_1156445 to 1156446, EPI_ISL_1285733	Institut für Medizinische Mikrobiologie - Universitätsmedizin Göttingen	Robert Koch Institute	
EPI_ISL_1048262, EPI_ISL_1048265, EPI_ISL_1048300, EPI_ISL_1048311, EPI_ISL_1048336	Institut für Medizinische Mikrobiologie und Hygiene, Institut für Virologie,Technische Universität Dresden	Robert Koch Institute, Influenza and respiratory viruses FG17 & Bioinformatics MF1, Berlin, Germany	A. Dalpke; Aleksandar Radonic; K. Hochauf-Stange; Marianne Wedde; Oliver Drechsel; Ralf Duerrwald; Rene Kmiecinski; Stefan Kroeger; Stephan Fuchs; Thorsten Wolff
EPI_ISL_1138605, EPI_ISL_1138614, EPI_ISL_1138618, EPI_ISL_1138675, EPI_ISL_1138682, EPI_ISL_1138724, EPI_ISL_1138734			
see above	Institut für Medizinische Virologie, Universitätsklinikum Frankfurt	Charité Universitätsmedizin Berlin, Institut für Virologie	Barbara Mühlemann; Christian Drosten; Christiane Pallas; Julia Schneider; Julia Tesch; Jörn Beheim-Schwarzbach; Marek Widera; Niko Kohmer; Sandra Ciesek; Talitha Veith; Terry Jones; Tobias Bleicker; Tuna Toptan Grabmair; Victor M Corman
EPI_ISL_1138599 to 1138604, EPI_ISL_1138606 to 1138613, EPI_ISL_1138615 to 1138617, EPI_ISL_1138619 to 1138674, EPI_ISL_1138676 to 1138681, EPI_ISL_1138683 to 1138723, EPI_ISL_1138725 to 1138733			
see above	Institut für Medizinische Virologie, Universitätsklinikum Frankfurt	Institut für Medizinische Virologie, Universitätsklinikum Frankfurt	Barbara Muehlemann et al
EPI_ISL_1117810 to 1117816, EPI_ISL_1117846 to 1117847, EPI_ISL_1118062	Institut für Pathologie, Salzkammergut Klinikum Vöcklabruck	Bergthaler laboratory, CeMM Research Center for Molecular Medicine of the Austrian Academy of Sciences	Andreas Bergthaler; Anna Schedl; Bekir Erguner; Benedikt Agerer; Christoph Bock; Jan Laine; Lukas Endler; Maelle Le Moing; Martin Senekowitsch; Michael Schuster; Thomas Penz
EPI_ISL_1008010 to 1008018, EPI_ISL_1008192 to 1008201, EPI_ISL_1180671 to 1180674	Institut für Pathologie, Salzkammergut Klinikum Vöcklabruck	Bergthaler laboratory, CeMM Research Center for Molecular Medicine of the Austrian Academy of Sciences	Andreas Bergthaler; Anna Schedl; Bekir Erguner; Benedikt Agerer; Christoph Bock; Fabian Amman; Jan Laine; Lukas Endler; Maelle Le Moing; Martin Senekowitsch; Michael Schuster; Thomas Penz
EPI_ISL_437913 to 437932, EPI_ISL_437974 to 437992, EPI_ISL_475763, EPI_ISL_475769, EPI_ISL_475813 to 475829, EPI_ISL_475916 to 475928, EPI_ISL_583572, EPI_ISL_583600 to 583616, EPI_ISL_583691, EPI_ISL_934540 to 934547, EPI_ISL_1008299 to 1008310, EPI_ISL_1117910			
see above	Institut für Virologie am Department für Hygiene, Mikrobiologie und Public Health	Bergthaler laboratory, CeMM Research Center for Molecular Medicine of the Austrian Academy of Sciences	Adi Steinrigl; Alexander Lercher; Alexandra Popa; Andreas Bergthaler; Anna Schedl; Bekir Erguner; Benedikt Agerer; Christian Paar; Christoph Bock; Daniela Schmid; Dorothee von Laer; Elisabeth Puchhammer-Stoeckl; Franz Allerberger; Gernot Walder; Gregor Hörmann; Guenter Weiss; Gunther Vogl; Henrique Colaco; Jakob-Wendelin Genger; Jan Laine; Judith Aberle; Kinga Rigler-Hohenwarter; Lukas Endler; Maelle Le Moing; Manfred Nairz; Mark Smyth; Martin Senekowitsch; Michael Schuster; Peter Hufnagl; Peter Obrist; Rainer Gattringer; Sabine Sussitz-Rack; Stephan Aberle; Thomas Penz; Wegene Borena
EPI_ISL_1140505 to 1140511, EPI_ISL_1140513 to 1140518, EPI_ISL_1141627 to 1141653	Institut für Virologie des Universitätsklinikums Tübingen	Robert Koch Institute	
EPI_ISL_485809 to 485813, EPI_ISL_508687 to 508704	Institut für Virologie und Epidemiologie der Viruskrankheiten, Universitätsklinikum Tübingen	NGS Competence Center Tübingen, Institut für Medizinische Mikrobiologie und Hygiene, Universitätsklinikum Tübingen	Angel Angelov; Angelov at al.; Angelov et al.
EPI_ISL_831427 to 831470	Institut für Virologie, Charite - Universitätsmedizin Berlin	Robert Koch Institute, Influenza and respiratory viruses FG17 & Bioinformatics MF1, Berlin, Germany	Aleksandar Radonic; Dr. Victor Corman; Marianne Wedde; Oliver Drechsel; Ralf Duerrwald; Rene Kmiecinski; Stefan Kroeger; Stephan Fuchs; Thorsten Wolff
EPI_ISL_1155565 to 1155566, EPI_ISL_1155568 to 1155570, EPI_ISL_1281287, EPI_ISL_1281291	Institut für med. Mikrobiologie u. Virologie Klinikum Oldenburg	Robert Koch Institute	
EPI_ISL_420075, EPI_ISL_476566	Institut pasteur Dakar	Institut Pasteur de Dakar	Amadou Alpha Sall; Mamadou Diop; Moussa Moise Diagne; Ndongo Dia; Ousmane Faye

EPI_ISL_955158 to 955168	Institute for Biocides and Medical Ecology	Institute of microbiology and Immunology, Faculty of Medicine, University of Belgrade	Jankovic, M.; Jovanovic, T.; Knezevic, A.; Milicevic, O.; Sekler, M.; Tesovic, B.; Vidanovic, D.
EPI_ISL_855186 to 855339	Institute for Computational Biomedicine, Weill Cornell Medicine	Institute for Computational Biomedicine, Weill Cornell Medicine	A.J.; A.M.; Afshinnekoo, E.; B.W.; Bezdan, D.; Bhattacharya, C.; Butler; C.D.; C.E.; Cong, L.; Craney, A.; Cushing, M.; D.J.; Danko, D.; F.J.; Foox, J.; Gawrys, J.; Gisladottir, U.; Hajirasouliha, I.; Horner; Iftner, A.; Iftner, T.; Imielinski, M.; Ivanov; L.F.; Langhorst; Levy, S.; Loda, M.; MacKay, M.; Mason; Meleshko, D.; Melnick; Meydan, C.; Mozsary, C.; N.A.; P.A.D.; Pohle, D.; R.E.; Ramlall, V.; Rennert, H.; Rosiene, J.; Ruggiero, P.; Ryon, K.; S.M.; Salvatore, M.; Schwartz; Sedlazeck; Shaiber, A.; Shemesh; Sierra, M.; Sipley, J.; Steel; Tanner, N.; Tatonetti, N.; Thierry-Mieg, D.; Thierry-Mieg, J.; Velu, P.; Westblade; Westover; Wu, S.; Xiang, J.; Xu, D.; Young, B.; Zietz, M.
EPI_ISL_454419, EPI_ISL_812969	Institute for Forensic Medicine, Faculty of Medicine, University of Belgrade	Institute for Forensic Medicine, Faculty of Medicine, University of Belgrade	I.R. and Bogdanovic; M.D.; Petrovic; Skadric; Skadric, I.; Stojkovic, O.; T. and Tolic, A.
EPI_ISL_1020296	Institute for Hygiene and Microbiology	Center for Virology	Irene Goerzer; Jeremy V. Camp; Monika Redlberger-Fritz; Stephan W. Aberle
EPI_ISL_574790 to 574862	Institute for Infectious Diseases, University of Bern	Institute for Infectious Diseases, University of Bern	Alban Ramette; Christian Baumann; Cora Sägesser; Franziska Suter-Riniker; Michel C Koch; Miguel A Terrazos Miani; Peter Keller; Stephen L Leib
EPI_ISL_830648, EPI_ISL_831649 to 831659, EPI_ISL_860719 to 860726, EPI_ISL_913436 to 913445, EPI_ISL_918164	Institute for Infectious Diseases, University of Bern, Switzerland	Institute for Infectious Diseases, University of Bern, Switzerland	Alban Ramette; Christian Baumann; Cora Sägesser; Franziska Suter-Riniker; Michel C Koch; Miguel A Terrazos Miani; Pascal Bittel; Peter Keller; Stephen L Leib
EPI_ISL_583853 to 583868, EPI_ISL_853784, EPI_ISL_853856 to 853873, EPI_ISL_853915 to 853916, EPI_ISL_854227	Institute for Laboratory Diagnostics and Microbiology, Klinikum Klagenfurt am Worthersee	Bergthaler laboratory, CeMM Research Center for Molecular Medicine of the Austrian Academy of Sciences	Adi Steinrigl; Alexander Lercher; Alexandra Popa; Andreas Bergthaler; Anna Schedl; Benedikt Agerer; Christian Paar; Christoph Bock; Christoph Bock; Daniela Schmid; Dorothee von Laer; Elisabeth Puchhammer-Stoeckl; Franz Allerberger; Gernot Walder; Gregor Hörmann; Guenter Weiss; Gunther Vogl; Henrique Colaco; Jakob-Wendelin Genger; Jan Laine; Judith Aberle; Kinga Rigler-Hohenwarter; Lukas Endler; Manfred Nairz; Mark Smyth; Martin Senekowitsch; Martin Senekowitsch; Michael Schuster; Michael Schuster; Peter Hufnagl; Peter Obrist; Rainer Gattringer; Sabine Sussitz-Rack; Stephan Aberle; Thomas Penz; Wegene Borena
EPI_ISL_1209263 to 1209266	Institute for Laboratory Diagnostics and Microbiology, Klinikum Klagenfurt am Wörthersee	Bergthaler laboratory, CeMM Research Center for Molecular Medicine of the Austrian Academy of Sciences	Andreas Bergthaler; Anna Schedl; Bekir Erguner; Benedikt Agerer; Christoph Bock; Fabian Amman; Jan Laine; Lukas Endler; Maelle Le Moing; Martin Senekowitsch; Michael Schuster; Thomas Penz
EPI_ISL_677723, EPI_ISL_678251, EPI_ISL_678253, EPI_ISL_909744, EPI_ISL_933804, EPI_ISL_954301, EPI_ISL_1013453, EPI_ISL_1013615, EPI_ISL_1014765, EPI_ISL_1140122, EPI_ISL_1140424			
see above	Institute for Lung Diseases in Children - Skopje	Research Center for Genetic Engineering and Biotechnology "Georgi D. Efremov" , Macedonian Academy of Sciences and Arts	Aleksandar J. Dimovski; Dijana Plasheska-Karanfilska; Gjorgji Bozinovski; Milena Jakimovska; Predrag Noveski; RCGEB - MASA
EPI_ISL_455790 to 455793	Institute for Medical Research, Infectious Disease Research Centre, National Institutes of Health, Ministry of Health Malaysia	Malaysia Genome Institute	Azrin Ahmad; Enizza Kasim; Irni Suhayu Sapian; Jeyanthi Suppiah; Mohd Faizal Abu Bakar; Mohd Noor Mat Isa; Norazah Ahmad; Nurhezreen Md Iqbal; Ravindran Thayan; Shahrul Hisham Zainal Ariffin; Shamsidar Sopie; Siti Noraini Othman; Tahir Aris; Yusuf Muhammad Noor; Zarina Mohd Zawawi
EPI_ISL_430439	Institute for Medical Research, Infectious Disease Research Centre, National Institutes of Health, Ministry of Health Malaysia	Institute for Medical Research Infectious Disease Research Centre, National Institutes of Health, Ministry of Health Malaysia	Abdul-Wahid.M-Z; Ahmad.N; Azizan.M-A; Hisham.H-A; Kalyanasundram.J; Mat-Sharani.S; Mohd-Zain.R; Mohd-Zawawi.Z; Suppiah.J; Tan.L-P; Thayan.R
EPI_ISL_459957	Institute for Medical Research, Infectious Disease Research Centre, National Institutes of Health, Minis	Institute for Medical Research, Infectious Disease Research Centre, National Institutes of Health, Minis	Ellan K; Kalyanasundram J; Kamel KA; Mohd-Zain R; Mohd-Zawawi Z; Suppiah J; Thayan R
EPI_ISL_459953	Institute for Medical Research, Infectious Disease Research Centre, National Institutes of Health, Ministry of Health Malaysia	Institute for Medical Research Infectious Disease Research Centre, National Institutes of Health, Ministry of Health Malaysia	Ellan K; Kalyanasundram J; Kamel KA; Mohd-Zain R; Mohd-Zawawi Z; Suppiah J; Thayan R
EPI_ISL_430440 to 430444, EPI_ISL_459954 to 459956, EPI_ISL_489992 to 489994, EPI_ISL_490014 to 490016, EPI_ISL_490047 to 490048, EPI_ISL_490089 to 490103, EPI_ISL_596449, EPI_ISL_644671 to 644672, EPI_ISL_718267 to 718272, EPI_ISL_718274 to 718315, EPI_ISL_728157 to 728158, EPI_ISL_728160 to 728167, EPI_ISL_728169 to 728170, EPI_ISL_728178, EPI_ISL_728209 to 728212, EPI_ISL_728241 to 728253, EPI_ISL_877228, EPI_ISL_912397, EPI_ISL_912399 to 912400, EPI_ISL_934424, EPI_ISL_936491, EPI_ISL_936496, EPI_ISL_944094 to 944096, EPI_ISL_944099 to 944104, EPI_ISL_962203 to 962207, EPI_ISL_962523 to 962527, EPI_ISL_968089, EPI_ISL_1055037, EPI_ISL_1055261 to 1055264, EPI_ISL_1055361 to 1055365, EPI_ISL_1068849 to 1068851, EPI_ISL_1068927 to 1068929, EPI_ISL_1081327, EPI_ISL_1081333, EPI_ISL_1081350 to 1081352, EPI_ISL_1114717 to 1114724, EPI_ISL_1114726, EPI_ISL_1114734, EPI_ISL_1114744, EPI_ISL_1114765, EPI_ISL_1138598, EPI_ISL_1138750, EPI_ISL_1186028, EPI_ISL_1196866 to 1196867, EPI_ISL_1196930, EPI_ISL_1204503, EPI_ISL_1263458, EPI_ISL_1263460, EPI_ISL_1263540, EPI_ISL_1264762, EPI_ISL_1265161			
see above	Institute for Medical Research, Infectious Disease Research Centre, National Institutes of Health, Ministry of Health Malaysia	Institute for Medical Research, Infectious Disease Research Centre, National Institutes of Health, Ministry of Health Malaysia	Abdul-Wahid.M-Z; Ahmad.N; Azizan MA; Azizan.M-A; Ellan K; Hisham.H-A; Kalyanasundram J; Kalyanasundram.J; Kamel K; Kamel KA; Mat-Sharani.S; Moh Zawawi Z; Mohd Zawawi Z; Mohd-Zain R; Mohd-Zain.R; Mohd-Zawawi Z; Mohd-Zawawi.Z; Ramly MN; Ramly N; Robert F; Suppiah J; Suppiah.J; Tan.L-P; Tengku-Abd-Rashid.T-R; Tengku-Rogayah.TAR; Thayan R; Thayan.R
EPI_ISL_583579 to 583598, EPI_ISL_1008212 to 1008214	Institute for Medical and Chemical Laboratory Diagnostics, Kepler Universitätsklinikum	Bergthaler laboratory, CeMM Research Center for Molecular Medicine of the Austrian Academy of Sciences	Adi Steinrigl; Alexander Lercher; Alexandra Popa; Andreas Bergthaler; Anna Schedl; Bekir Erguner; Benedikt Agerer; Christian Paar; Christoph Bock; Daniela Schmid; Dorothee von Laer; Elisabeth Puchhammer-Stoeckl; Franz Allerberger; Gernot Walder; Gregor Hörmann; Guenter Weiss; Gunther Vogl; Henrique Colaco; Jakob-Wendelin Genger; Jan Laine; Judith Aberle; Kinga Rigler-Hohenwarter; Lukas Endler; Maelle Le Moing; Manfred Nairz; Mark Smyth; Martin Senekowitsch; Michael Schuster; Peter Hufnagl; Peter Obrist; Rainer Gattringer; Sabine Sussitz-Rack; Stephan Aberle; Thomas Penz; Wegene Borena
EPI_ISL_429659, EPI_ISL_429663, EPI_ISL_429705, EPI_ISL_429792, EPI_ISL_429802 to 429803, EPI_ISL_429805, EPI_ISL_454605 to 454606, EPI_ISL_455566 to 455567, EPI_ISL_468591, EPI_ISL_468656			
see above	Institute for Public Health	Laboratory for advanced genomics	Filip Roki; Igor Jurak; Lovro Trgovec-Greif; Neven Sui; Oliver Vugrek; Tomislav Rukavina
EPI_ISL_492075 to 492086	Institute for Public Health of the Republic of North Macedonia	Charite Universitatsmedizin Berlin, Institute of Virology	Barbara Muhlemann; Christian Drosten; Elizabeta Jancheska; Golubinka Bosevska; Joern Beheim-Schwarzbach; Julia Schneider; Maja Kuzmanovska; Talitha Veith; Terry Jones; Victor M Corman
EPI_ISL_477168, EPI_ISL_477205 to 477263, EPI_ISL_479736 to 479755, EPI_ISL_480293 to 480296, EPI_ISL_486666 to 486674, EPI_ISL_486835 to 486841	Institute for Stem Cell Science and Regenerative Medicine	National Centre for Biological Sciences	Aswin Seshasayee; Awadhesh Pandit; Dasaradhi Palakodeti; Farhan Ali; Mohak Sharda; Shah-e-Jahan Gulzar; Srikar Krishna; Uma Ramakrishnan; Vanessa Molin Paynter; Varadha Sundarmurthy
EPI_ISL_707707 to 707708, EPI_ISL_728557 to 728563, EPI_ISL_733528 to 733567, EPI_ISL_812929 to 812955, EPI_ISL_882773 to 882779, EPI_ISL_882781 to 882794, EPI_ISL_882800 to 882804, EPI_ISL_887105, EPI_ISL_1138828 to 1138829			
see above	Institute for Urban Disease Control and Prevention	COVID-19 Network Investigations (CONI) Alliance	Amornmas Kongklieng; Anek Mungaomklang; Angkana Huang; Anthony R. Jones; Arporn Wangwiwatsin; Bhakbhoom Panthan; Chonticha Klungtong; Duangkamon Loesbanluechai; Ekawat Pasomsub; Elizabeth Batty; Insee Sensorn; Janjira Thaipadungpanit; Kamolthip Atsawawaranunt; Khajohn Joonlasak; Khajohn Joonsalak; Kingkan Rakmanee; Krittikorn Kumpornsin; Namfon Kotanan; Prayuth Kaewmalang; Pukkaporn Parnwijitkul; Stefan Fernandez; Thanat Chookajorn; Theerarat Kochakarn; Treewat Watthanachockchai; Vichan Pawun; Wasun Chantratita; Wudtichai Manasatienkij
EPI_ISL_572330 to 572331, EPI_ISL_572333, EPI_ISL_572397, EPI_ISL_573152, EPI_ISL_574259	Institute for Virology, University Hospital Duesseldorf, Medical Faculty, Heinrich-Heine-University Duesseldorf	Institute for Virology, University Hospital Duesseldorf, Medical Faculty, Heinrich-Heine-University Duesseldorf	; Alexander Killer; Andreas Walker; Annemarie Mohring; Anselm Kunstein; Ansgar Schulz; Björn Jensen; Caroline Klindt; Edwin Bölke; Gerald Antoch; Heiner Schaal; Jennifer Neubert; Johannes Bode; Johannes C. Fischer; Jörg Timm; Lisa Müller; Maximilian Damagnez; Nadine Lübke; Ortwin Adams; Philipp Albrecht; Philipp Ostermann; Saskia Elben; Tina Senff; Tom Lüdde; Torsten Feldt; Verena Keitel
EPI_ISL_602462 to 602509, EPI_ISL_602518 to 602532, EPI_ISL_626211 to 626230, EPI_ISL_717977	Institute for Virology, University Hospital Essen	Center of Medical Microbiology, Virology, and Hospital Hygiene, University of Duesseldorf	Alexander Dilthey; Andreas Walker; Daniel Strelow; Jessica Nicolai; Jörg Timm; Klaus Pfeffer; Lisanna Hülse; Malte Kohns Vasconcelos; Maximilian Damagnez; Nadine Lübke; Olympia E. Anastasiou; Tobias Wienemann; Torsten Houwaart; Ulf Dittmer
EPI_ISL_853723, EPI_ISL_853752, EPI_ISL_853762, EPI_ISL_853787, EPI_ISL_853816, EPI_ISL_854233, EPI_ISL_934650 to 934651, EPI_ISL_934696, EPI_ISL_1008266 to 1008279, EPI_ISL_1008311 to 1008313, EPI_ISL_1117781, EPI_ISL_1117793, EPI_ISL_1117803, EPI_ISL_1117903, EPI_ISL_1117912, EPI_ISL_1117915, EPI_ISL_1180910 to 1180931, EPI_ISL_1209335 to 1209364, EPI_ISL_1209369			
see above	Institute for Water Quality and Resource Management, Technical University Vienna	Bergthaler laboratory, CeMM Research Center for Molecular Medicine of the Austrian Academy of Sciences	Alexander Lercher; Alexandra Popa; Andreas Bergthaler; Anna Schedl; Bekir Erguner; Benedikt Agerer; Christoph Bock; Christoph Bock; Fabian Amman; Jakob-Wendelin Genger; Jan Laine; Lukas Endler; Maelle Le Moing; Martin Senekowitsch; Martin Senekowitsch; Michael Schuster; Michael Schuster; Thomas Penz
EPI_ISL_956403, EPI_ISL_1140101	Institute for prevention, treatment and rehabilitation of cardiovascular diseases - Ohrid	Research Center for Genetic Engineering and Biotechnology "Georgi D. Efremov" , Macedonian Academy of Sciences and	Aleksandar J. Dimovski; Dijana Plasheska-Karanfilska; Gjorgji Bozinovski; Milena Jakimovska; Predrag Noveski

		Arts	
EPI_ISL_418988	Institute information KU Leuven, Clinical and Epidemiological Virology	Institute information KU Leuven, Clinical and Epidemiological Virology	Bert Vanmechelen; Joan Marti-Carreras; Piet Maes; Tony Wawina
EPI_ISL_918411	Institute of Biocides and Medical Ecology	Virology Department Institute of Microbiology and Immunology Faculty of Medicine University of Belgrade	Abazovic Dzihan; Banko Ana; Despot Dragana; Loncar Ana; Milicevic Ognjen; Miljanovic Danijela
EPI_ISL_1055778 to 1055779, EPI_ISL_1055782 to 1055783, EPI_ISL_1055786 to 1055787, EPI_ISL_1055790 to 1055791, EPI_ISL_1055793 to 1055794, EPI_ISL_1056956 to 1056957, EPI_ISL_1056959, EPI_ISL_1056968, EPI_ISL_1056970, EPI_ISL_1056972, EPI_ISL_1056974, EPI_ISL_1057035 to 1057036, EPI_ISL_1057038, EPI_ISL_1057040 to 1057041, EPI_ISL_1057097 to 1057098, EPI_ISL_1057102 to 1057105, EPI_ISL_1061425, EPI_ISL_1061427, EPI_ISL_1061429 to 1061431			
see above	Institute of Biocides and Medical Ecology, Belgarde, Serbia	Virology Department Institute of Microbiology and Immunology Faculty of Medicine University of Belgrade	Abazovic Dzihan; Banko Ana; Despot Dragana; Loncar Ana; Milicevic Ognjen; Miljanovic Danijela
EPI_ISL_891267 to 891272	Institute of Biocides and Medical Ecology, Belgarde, Serbia	Virology department Institute of microbiology and immunology Faculty of Medicine University of Belgrade	Abazovic Dzihan; Banko Ana; Despot Dragana; Loncar Ana; Milicevic Ognjen; Miljanovic Danijela
EPI_ISL_909737	Institute of Biocides and Medical Ecology, Belgrade, Serbia	Virology Department, Institute of microbiology and immunology, Faculty of Medicine University of Blegrade	Abazovic Dzihan; Banko Ana; Despot Dragana; Loncar Ana; Milicevic Ognjen; Miljanovic Danijela
EPI_ISL_891266	Institute of Biocides and Medical Ecology, Belgrade, Serbia	Virology department Institute of microbiology and immunology Faculty of Medicine University of Belgrade	Abazovic Dzihan; Banko Ana; Despot Dragana; Loncar Ana; Milicevic Ognjen; Miljanovic Danijela
EPI_ISL_450488	Institute of Biomedical & Genetic Engineering	Institute of Biomedical & Genetic Engineering	A.H.; Ajmal; Hashmi; M. and Ahmad, N.
EPI_ISL_1118929	Institute of Clinical Hygiene, Medical Microbiology and Infectiology, General Hospital Nürnberg, Paracelsus Medical University, Nuremberg, Germany	Department of Molecular & Medical Virology, Ruhr University Bochum, 44801 Bochum, Germany | Institute of Virology, Charité - Universitätsmedizin Berlin, corporate member of Freie Universität Berlin, Humboldt-Universität zu Berlin and Berlin Institute of Health (BIH), Berlin, Germany	Baxolele Mhlekude; Christine Goffinet; Daniel Todt; Eike Steinmann; Jörg Steinmann; Stephanie Pfaender; Toni Luise Meister; Yannick Brüggemann
EPI_ISL_525472 to 525473	Institute of Clinical Microbiology and Hygiene, University Hospital Regensburg	Institute of Clinical Microbiology and Hygiene, University Hospital Regensburg	Hiergeist, A.
EPI_ISL_539333 to 539339	Institute of Disease Control and Prevention, People's Liberation Army	Institute of Disease Control and Prevention, People's Liberation Army	Li, P.; Qiu, S.
EPI_ISL_600428 to 600446, EPI_ISL_600449, EPI_ISL_600451, EPI_ISL_600454, EPI_ISL_600456, EPI_ISL_600459, EPI_ISL_600461, EPI_ISL_600463, EPI_ISL_600466, EPI_ISL_600468, EPI_ISL_600471, EPI_ISL_600473, EPI_ISL_600476, EPI_ISL_600478, EPI_ISL_600480, EPI_ISL_600484, EPI_ISL_600487, EPI_ISL_600489, EPI_ISL_600492, EPI_ISL_600495, EPI_ISL_600498, EPI_ISL_600500, EPI_ISL_600502, EPI_ISL_600505, EPI_ISL_600507, EPI_ISL_600510, EPI_ISL_600512, EPI_ISL_600515, EPI_ISL_600517, EPI_ISL_600519, EPI_ISL_600522, EPI_ISL_600524, EPI_ISL_600527, EPI_ISL_600530, EPI_ISL_600533, EPI_ISL_600535, EPI_ISL_600538, EPI_ISL_600541, EPI_ISL_600543, EPI_ISL_600546, EPI_ISL_600549, EPI_ISL_600552, EPI_ISL_600554, EPI_ISL_600557, EPI_ISL_600559, EPI_ISL_600562, EPI_ISL_600564, EPI_ISL_600567, EPI_ISL_600570			
see above	Institute of Epidemiology Disease Control And Research	Institute for Developing Science and Health Initiatives	Firadausi Qadri; Lauren Cowley; Md. Mahfuz-Al-mamun; Mokibul Hassan Afrad; Sadia Isfat Ara Rahman; Tahmina Shirin
EPI_ISL_1061277 to 1061278	Institute of Hematology and Transfusion Medicine	Laboratory of Recombinant Vaccines	Aneta Kopacz; Boguslaw Szewczyk; Krystyna Bienkowska-Szewczyk; Krzysztof Pyr; Lukasz Rabalski; Maciej Kosinski; Natalia Mazur-Panasiuk
EPI_ISL_462480, EPI_ISL_485399, EPI_ISL_582031 to 582032, EPI_ISL_1048376 to 1048380	Institute of Human Genetics, Polish Academy of Sciences	Institute of Human Genetics, Polish Academy of Sciences	Adam Ustaszewski; Andrzej Pawski; Emilia Lis; Ewa Zitkiewicz; Marta Kaczmarek-Ry; Micha Witt; Szymon Hryhorowicz; Tomasz Woniak
EPI_ISL_485400	Institute of Human Genetics, Polish Academy of Sciences	Institute of Human Genetics, Polish Academy of Sciences,	Adam Ustaszewski; Andrzej Pawski; Emilia Lis; Ewa Zitkiewicz; Marta Kaczmarek-Ry; Micha Witt; Szymon Hryhorowicz
EPI_ISL_450294	Institute of Human Genetics, Polish Academy of Sciences Sanitary and Epidemiological Station in Pozna	Institute of Human Genetics, Polish Academy of Sciences	Adam Ustaszewski; Andrzej Pawski; Emilia Lis; Marta Kaczmarek-Ry; Micha Witt; Szymon Hryhorowicz
EPI_ISL_450295	Institute of Human Genetics, Polish Academy of Sciences; Sanitary and Epidemiological Station in Pozna	Institute of Human Genetics, Polish Academy of Sciences	Adam Ustaszewski; Andrzej Pawski; Emilia Lis; Marta Kaczmarek-Ry; Micha Witt; Szymon Hryhorowicz
EPI_ISL_450338	Institute of Human Genetics, Polish Academy of Sciences; Sanitary and Epidemiological Station in Pozna	Institute of Human Genetics, Polish Academy of Sciences	Adam Ustaszewski; Andrzej Pawski; Emilia Lis; Marta Kaczmarek-Ry; Micha Witt; Szymon Hryhorowicz
EPI_ISL_854229, EPI_ISL_854237, EPI_ISL_934589 to 934592, EPI_ISL_1008202 to 1008204, EPI_ISL_1117897 to 1117899, EPI_ISL_1117902, EPI_ISL_1117905, EPI_ISL_1180831, EPI_ISL_1209268			
see above	Institute of Legal Medicine, Medical University of Innsbruck	Bergthaler laboratory, CeMM Research Center for Molecular Medicine of the Austrian Academy of Sciences	Alexander Lercher; Alexandra Popa; Andreas Bergthaler; Anna Schedl; Bekir Erguner; Benedikt Agerer; Christoph Bock; Christoph Bock; Fabian Amman; Jakob-Wendelin Genger; Jan Laine; Lukas Endler; Maelle Le Moing; Martin Senekowitsch; Martin Senekowitsch; Michael Schuster; Michael Schuster; Thomas Penz
EPI_ISL_1190402, EPI_ISL_1196305, EPI_ISL_1241861, EPI_ISL_1242007, EPI_ISL_1242030, EPI_ISL_1255308, EPI_ISL_1255389 to 1255390			
see above	Institute of Life Sciences	Institute of Life Sciences	Amol Suryawanshi; Ankita Datey; Arup Ghosh; Atimukta Jha; Eshna Laha; Gargee Bhattacharya; Gulam Hussain Syed; ILS COVID-19 Team and Ajay Parida; ILS COVID-19 Team and Ajay Parida.; Odisha COVID-19 Study Group; Punit Prasad; Rajeeb Swain; Rupesh Dash; Safal Rajesh Walia; Saikat De; Sanchari Chatterjee; Sandhya Suranjika; Shantibhushan Senapati; Soma Chattopadhyay; Soumya Sengupta; Soumyajit Ghosh; Sunil Raghav; Supriya Suman Keshry; Tushar K. Beuria
EPI_ISL_463010 to 463093	Institute of Life Sciences, Bhubaneswar	Immunogenomics lab, Institute of Life Sciences, Bhubaneswar	Ajay Parida; Aliva Minz; Arup Ghosh; Atimukta Jha; Avula Kiran; DBT's PAN-INDIA 1000 SARS-CoV2 RNA genome sequencing consortium; Debyashrita Barik; Deepak Singh; Eshna Laha; Ghulam Hussain Syed; Hiren G. Dodia; ILS COVID-19 TEAM; Jeky Chawla; Kaushik Sen; Kautilya Kumar Jena; Manasi Priyadarshini; Neha Singh; O. P. Shriwas; Orissa COVID-19 Study Group; Parej Nath; Priyanka Mohapatra; Punit Prasad; Rajeeb Swain; Rina Yadav; Rupesh Dash; Saiket De; Sanchari Chatterjee; Sandhya Suranjika; Satya Ranjan Sahu; Shamima Ansari; Shanti Senapati; Shuchi Smita; Sifu Agarwal; Soma Chattopadhyay; Sunil Raghav; Supriya Suman; Swati Madhulika; Tushar K. Beuria; Viplov K. Biswas
EPI_ISL_527871	Institute of Medical Biology, Chinese Academy of Medical Sciences and Peking Union Medical College	Institute of Medical Biology, Chinese Academy of Medical Sciences and Peking Union Medical College	Chen, H.; Fan, S.; G. and Li, Q.; Jiang; Liao, Y.; Liu, L.; Wang, L.; Xie, Z.; Xu, X.; Zheng, H.; Zhou, X.
EPI_ISL_723077 to 723117	Institute of Medical Genetics and Applied Genomics	Institute of Medical Genetics and Applied Genomics	Angel Angelov; Caspar Gross; Daniela Bezdan; Michael Bitzer; Michael Sonnabend; Michaela Pogoda; Nicolas Casadei; Siri Göpel; Stephan Ossowski; Thomas Iftner; Tina Ganzenmüller
EPI_ISL_803871, EPI_ISL_803874 to 803875, EPI_ISL_864544 to 864546, EPI_ISL_864563 to 864579, EPI_ISL_884260 to 884290, EPI_ISL_887115 to 887144, EPI_ISL_894161 to 894169, EPI_ISL_960834, EPI_ISL_960836 to 960837, EPI_ISL_960842, EPI_ISL_960845, EPI_ISL_960847 to 960848, EPI_ISL_960850, EPI_ISL_960853 to 960864, EPI_ISL_960866 to 960882, EPI_ISL_960900 to 960901, EPI_ISL_1034799, EPI_ISL_1034806 to 1034811, EPI_ISL_1034815 to 1034816, EPI_ISL_1034827 to 1034829, EPI_ISL_1034831 to 1034834, EPI_ISL_1034840 to 1034847, EPI_ISL_1034854, EPI_ISL_1034878 to 1034879, EPI_ISL_1034893, EPI_ISL_1034897, EPI_ISL_1034901, EPI_ISL_1034907 to 1034910, EPI_ISL_1279618 to 1279619, EPI_ISL_1279622, EPI_ISL_1279625 to 1279626, EPI_ISL_1279631 to 1279635, EPI_ISL_1279647 to 1279648, EPI_ISL_1279651 to 1279652, EPI_ISL_1279660 to 1279664, EPI_ISL_1279671 to 1279677, EPI_ISL_1279685 to 1279690, EPI_ISL_1279698 to 1279699, EPI_ISL_1279706 to 1279708, EPI_ISL_1279713 to 1279718, EPI_ISL_1279732, EPI_ISL_1279734, EPI_ISL_1279745 to 1279747, EPI_ISL_1279754 to 1279756, EPI_ISL_1279766 to 1279768, EPI_ISL_1279771 to 1279773, EPI_ISL_1279775 to 1279782, EPI_ISL_1279784 to 1279785, EPI_ISL_1279791 to 1279792, EPI_ISL_1279805 to 1279806, EPI_ISL_1279813, EPI_ISL_1279815, EPI_ISL_1279819, EPI_ISL_1279821, EPI_ISL_1279828, EPI_ISL_1279831, EPI_ISL_1279844 to 1279848, EPI_ISL_1279851 to 1279856, EPI_ISL_1279865 to 1279869, EPI_ISL_1279875 to 1279879			
see above	Institute of Medical Microbiology and Hospital Hygiene	Institute of Medical Microbiology and Hospital Hygiene	Aljoscha Tersteegen; Prof. Dr. Achim Kaasch
EPI_ISL_886157, EPI_ISL_887106 to 887114	Institute of Medical Microbiology and Virology, University Hospital Carl Gustav Carus, TU Dresden	DRESDEN-concept Genome Center, CMCB, TU Dresden	Beil J.; Brown T.; Büttner L.; Gscheidel N.; Hochauf-Stange K.; Klemroth S.; Lindemann D.; Mehnert G.; Petzold A.; Reinhardt S.; Rost F.; Sameith K.; Winkler S.
EPI_ISL_517611	Institute of Medical Microbiology, University Medical Center Goettingen	Institute of Human Genetics, University Medical Center Goettingen	Andreas E Zautner; Antje Dickmanns; Arne Zibat; Bernd Wollnik; Gabriela Salinas-Riester; Maren Sitte; Maren Stegmann; Matthias Dobbelstein; Uwe Groß
EPI_ISL_590823 to 590836	Institute of Medical Virology, University of Zurich	Institute of Medical Virology, University of Zurich	Alexandra Trkola; Benjamin G. Hale; Idoia Busnadiego; Irene Abela; Marie O. Pohl; Maryam Zaheri; Michael Huber; Silke Stertz; Stefan Schmutz; Verena Kufner
EPI_ISL_422563 to 422565	Institute of Microbiology Universidad San Francisco de Quito	Institute of Microbiology Universidad San Francisco de Quito	Alejandra Ramones; Belen Prado-Vivar; Bernardo Gutierrez; Edison Ligña; Francisco Mora; Franklin Espinoza; Gabriel Trueba; Jorge Reyes; Juan Gaviria; Juan Jose Guadalupe; Michelle Grunauer; Patricio Rojas-Silva; Paul Cardenas; Sully Marquez; Veronica Barragan

EPI_ISL_420294 to 420295, EPI_ISL_420541, EPI_ISL_635200 to 635203, EPI_ISL_635205 to 635299, EPI_ISL_636459 to 636461, EPI_ISL_877424 to 877431, EPI_ISL_877437 to 877453, EPI_ISL_877455, EPI_ISL_906117 to 906130, EPI_ISL_979246 to 979270, EPI_ISL_1020294, EPI_ISL_1063495, EPI_ISL_1063497 to 1063500, EPI_ISL_1063504 to 1063513, EPI_ISL_1063519 to 1063520, EPI_ISL_1063523 to 1063525, EPI_ISL_1063996, EPI_ISL_1098755 to 1098797, EPI_ISL_1118849 to 1118870, EPI_ISL_1181822 to 1181834, EPI_ISL_1181836 to 1181849, EPI_ISL_1181851 to 1181852, EPI_ISL_1181854 to 1181864, EPI_ISL_1185774 to 1185775, EPI_ISL_1190964 to 1190965, EPI_ISL_1190967, EPI_ISL_1190969 to 1190973, EPI_ISL_1190975, EPI_ISL_1190977, EPI_ISL_1190979, EPI_ISL_1190981 to 1190987, EPI_ISL_1190990, EPI_ISL_1190992 to 1190994, EPI_ISL_1190997 to 1191001, EPI_ISL_1191004 to 1191006, EPI_ISL_1191008 to 1191009, EPI_ISL_1191011 to 1191015, EPI_ISL_1191018, EPI_ISL_1191020 to 1191022, EPI_ISL_1191024 to 1191025, EPI_ISL_1191027 to 1191028, EPI_ISL_1191030 to 1191032, EPI_ISL_1191035, EPI_ISL_1191037 to 1191041, EPI_ISL_1191044, EPI_ISL_1191046, EPI_ISL_1191048 to 1191052, EPI_ISL_1201128, EPI_ISL_1201131, EPI_ISL_1210501 to 1210529, EPI_ISL_1228587, EPI_ISL_1250645 to 1250694, EPI_ISL_1250713 to 1250737, EPI_ISL_1250814 to 1250841, EPI_ISL_1265165, EPI_ISL_1265167, EPI_ISL_1265174 to 1265234, EPI_ISL_1265264 to 1265272, EPI_ISL_1265274 to 1265352, EPI_ISL_1273382 to 1273384, EPI_ISL_1273386 to 1273390			
see above	Institute of Microbiology and Immunology, Faculty of Medicine, University of Ljubljana	Institute of Microbiology and Immunology, Faculty of Medicine, University of Ljubljana	Alen Sulji; Andrej Celar; Dominika Šturm; Doroteja Vlaj; Lucijan Skubic; Mario Poljak; Matic Brvar; Miša Korva; Patricija Pozvek; Samo Zakotnik; Tatjana Avši - Županc; Tatjana Avši - Županc; Tomaž Mark Zorec; Špela Pleh
EPI_ISL_635204	Institute of Microbiology and Immunology, Faculty of Medicine, University of Ljubljana	The National Laboratory of Health, Environment and Food - Centre for Medical Microbiology Maribor	Mario Poljak; Miša Korva; Mojca Cimerman; Samo Zakotnik; Tatjana Avši - Županc; Tomaž Mark Zorec
EPI_ISL_548942 to 548946	Institute of Microbiology, University of Veterinary and Animal sciences	Institute of Microbiology, University of Veterinary and Animal sciences	Ali; Altaf, I.; Anwar, M.; Ashraf; Asif, A.; Attique; Awan; Aziz; Bhatti; Cheema; Fazal, S.; Hassan, S.; Khan; Khan, N.; M.A.; M.B.; M.M.; M.S.; M.T.; M.U.; M.W.; M.Z.; Mehmood, A.; Mukhtar, N.; N.A.; Nawaz, M.; Rafique, S.; Rana; Raza, S.; S.Q.; S.Z.; Sardar, N.; Sarwar, H.; Shabbir; Shah; Tahir, Z.; Yaqub, T.; Younis
EPI_ISL_417482, EPI_ISL_477014 to 477016, EPI_ISL_486842 to 486851, EPI_ISL_491932, EPI_ISL_491936 to 491940, EPI_ISL_516648 to 516652, EPI_ISL_525430 to 525438, EPI_ISL_527809 to 527817, EPI_ISL_539785 to 539793, EPI_ISL_660529 to 660539, EPI_ISL_697783 to 697795, EPI_ISL_697798 to 697800, EPI_ISL_728202 to 728205, EPI_ISL_803098, EPI_ISL_824284 to 824294, EPI_ISL_877551 to 877562			
see above	Institute of Microbiology, Universidad San Francisco de Quito	Institute of Microbiology, Universidad San Francisco de Quito	Adriana Arnao; Alejandra Ramones; Alexandra Noboa; Alexandra Tino; Alvaro Davalos de Castro; Ana Diaz; Andrea Cunguan; Andrea Macias; Andres Moreno; Anita Garcia; Belen Prado-Vivar; Belén Prado-Vivar; Bernardo Gutierrez; Bernardo Gutiérrez; Carla Torres; Carlos Guerrero; Carlos Mena; Carlos Tobar; Carolina Pacheco; Cristian Pelaez; Damaris Zandoya; DamarisSandoya; David Acosta-España; Dayron Brossard; Diana Zambrano; Diego Egas; Diego Mora; Diego Morocho; Eddy Chavez; Edgar Espinoza; Edison Chavez; Edison Ligña; Edmundo Encalada; Eduardo Males; Edy Quizhpe; Eulalia Pazmiño; Fabricio Picoita; Fausto Maldonado; Fernanda Garcia; Fernando Martinez; Francisco Cordova; Francisco Mora; Francisco Rodriguez; Franklin Espinoza; Freddy Iza; Fredy Loor; Gabriel Trueba; Geovanny Carzola; Hector Moya; Hermelinda Paguay; Jonathan Araujo; Jorge Caamaño; Jorge Luis Velez; Jorge Montaño; Jorge Reyes; Juan Gaviria; Juan Jose Guadalupe; Juan José Guadalupe; Juan Monge; Juan Zuñiga; Karina Barragan; Katalina Pacheco; Khurram Mahbbob; Kyllen Briones; Ligia Briceño; Manuel Jaramillo; Manuel Jibaja; Marco Jimenez; Marcos DiStefano; Maribel Cruz; Maureen Mosquera; Michelle Grunauer; Milton Tobar; Monica Becerra-Wong; Nabih Dahik; Nelson Remache; Ninfa Hernandez; Pablo Flores; Patricio Reyes; Patricio Rojas-Silva; Paul Cardenas; Paúl Cárdenas; Piedad Villacis; Prado-Vivar; Rene Bracho; Rosa Avila; Rosario Erazo; Sayra Caiza; Stalin Castillo; Stephanie Arregui; Sully Marquez; Sully Márquez; Tania Guayasamin; Vanesa Ramirez; Veronica Barragan; Veronica Paredes; Verónica Barragán; Viviana Medina; Yomara Napa
EPI_ISL_1121467 to 1121468, EPI_ISL_1121470, EPI_ISL_1121472 to 1121474, EPI_ISL_1121477, EPI_ISL_1121479, EPI_ISL_1121482 to 1121483, EPI_ISL_1121485			
see above	Institute of Microbiology, Universidad San Francisco de Quito	Omics Sciences Laboratory	Belén Prado-Vivar; Bernardo Gutiérrez; Darlyn Amaya; Derly Andrade Molina; Emily Sulay Saltos Montalvo; Gabriel Morey León; Gabriel Trueba; Juan Carlos Fernández Cadena; Juan José Guadalupe; Katheryn Sacheri Viteri; Michelle Grunauer; Monica Becerra-Wong; Patricio Reyes; Patricio Rojas-Silva; Paula Juliana Gavilanes Jarrín; Paúl Cárdenas; Rubén Armas González; Sully Márquez; Tania Guayasamin; Verónica Barragán
EPI_ISL_634976	Institute of Molecular Biology and Biotechnology, National Institutes of Health	Institute of Molecular Biology and Biotechnology, National Institutes of Health	Arevalo, G.; Dungca; E.M.; N.T. and Salvana; Schwem, B.
EPI_ISL_463008	Institute of Molecular Virology, University Münster	Institute of Molecular Virology, University Münster	Alexander Mellmann; Angeles Mecate Zambrano; Joachim Kühn; Linda Brunotte; Stephan Ludwig
EPI_ISL_1233820 to 1233867	Institute of Molecular and Translational Medicine / Laboratory of Experimental Medicine, Faculty of Medicine and Dentistry, Palacky University	Institute of Molecular and Translational Medicine / Laboratory of Experimental Medicine	Hana Jaworek; Marián Hajdúch; Rastislav Slavkovský; Vladimíra Koudeláková
EPI_ISL_1140094	Institute of Nephrology - Struga	Research Center for Genetic Engineering and Biotechnology "Georgi D. Efremov" , Macedonian Academy of Sciences and Arts	Aleksandar J. Dimovski; Dijana Plasheska-Karanfilska; Gjorgji Bozinovski; Milena Jakimovska; Predrag Noveski
EPI_ISL_402123, EPI_ISL_403928 to 403931	Institute of Pathogen Biology, Chinese Academy of Medical Sciences & Peking Union Medical College	Institute of Pathogen Biology, Chinese Academy of Medical Sciences & Peking Union Medical College	Chao Wu; Jianwei Wang; Lili Ren; Qi Jin; Yiwei Liu; Zhiqiang Wu; Zichun Xiang
EPI_ISL_508338 to 508414, EPI_ISL_653826 to 653832	Institute of Post Graduate Medical Education & Research	National Institute of Biomedical Genomics	Arindam Maitra; Aritra Biswas; Jayeeta Haldar; Monimoy Banerjee; Raja Ray; Saumitra Das
EPI_ISL_511900 to 511907	Institute of Post Graduate Medical Education & Research	National Institute of Biomedical Genomics - DBT's PAN-INDIA 1000 SARS--CoV-2 RNA Genome Sequencing Consortium	Arindam Maitra; Aritra Biswas; Jayeeta Haldar; Monimoy Banerjee; Raja Ray; Saumitra Das
EPI_ISL_1300652	Institute of Public Health of Central Bosnia Canton, Travnik	Clinical Center, University of Sarajevo; Unit for Clinical Microbiology	Amela Dedei-Ljubovi; Edina Zahirovi; Golubinka Boshevska; Irma Salimovi-Beši; Maja Kuzmanovska; Sandra Vegar-Zubovi; Sebija Izetbegovi; Suzana Arapi
EPI_ISL_877421, EPI_ISL_882769, EPI_ISL_956280, EPI_ISL_956312, EPI_ISL_956316, EPI_ISL_1159384, EPI_ISL_1165072, EPI_ISL_1165074			
see above	Institute of Tropical Disease	Institute of Tropical Disease, Universitas Airlangga	Aldise M Nastri; Gatot Soegiarto; Jezzy R Dewantari; Kazufumi Shimizu; Krisnoadi Rahardjo; Laksmi Wulandari; Maria I Lusida; Resti Yudhawati; Rima R Prasetya; Soetjipto; Yasuko Mori
EPI_ISL_672701 to 672721, EPI_ISL_672746 to 672751	Institute of Tropical Medicine at the University of São Paulo (IMT-USP)	Laboratório de Parasitologia Médica - Instituto de Medicina Tropical - Universidade de São Paulo	Brazil-UK Centre for Arbovirus Discovery Diagnosis Genomics and Epidemiology (CADDE) Genomic Network - Instituto de Medicina Tropical
EPI_ISL_408511 to 408515	Institute of Viral Disease Control and Prevention, China CDC	Institute of Viral Disease Control and Prevention, China CDC	Beiwei Ye; Dayan Wang; George F. Gao; Guizhen Wu; Juan Song; Jun Han; Mengjie Yang; Peihua Niu; Peipei Liu; Roujian Lu; Shumei Zou; Wei Zhen; Weifeng Shi; Weimin Zhou; Wenbo Xu; Wenjie Tan; Wenwen Lei; William J. Liu; Xiang Zhao; Yingze Zhao; Yuchao Wu; Zhixiao Chen; Ziqian Xu
EPI_ISL_964878 to 964879	Institute of Virology Ulm University Medical Center Prof. Dr. Thomas Stamminger	Münch Lab / Ulm University Medical Center Kirchhoff Lab / Ulm University Medical Center Sparrer Lab / Ulm University Medical Center Blum Lab / LMU Munich	Alexander Graf; Carina Conzelmann; Frank Kirchhoff; Helmut Blum; Jan Münch; Janis Müller; Konstantin Sparrer; Rüdiger Gross; Stefan Krebs; Thomas Stamminger
EPI_ISL_577734 to 577742, EPI_ISL_583481 to 583489, EPI_ISL_717975, EPI_ISL_718250 to 718260, EPI_ISL_779403, EPI_ISL_779651 to 779660, EPI_ISL_788979 to 788988, EPI_ISL_791990, EPI_ISL_831663 to 831676, EPI_ISL_875516 to 875539, EPI_ISL_903979 to 904003, EPI_ISL_959604 to 959649, EPI_ISL_1091488 to 1091492, EPI_ISL_1234384 to 1234473, EPI_ISL_1239372 to 1239427, EPI_ISL_1299292 to 1299315, EPI_ISL_1310813 to 1310831			
see above	Institute of Virology, Biomedical Research Center of the Slovak Academy of Sciences, Bratislava	Faculty of Natural Sciences, Comenius University, Bratislava	Alena Košálová; Andrej Belák; Boris Klempa; Brona Brejova; Broa Brejová; Dominika Friová; Edita Staroová; Elena Tichá; Jozef Nosek; Juraj Kopacek; Juraj Kopáek; Kristina Borsova; Kristína Boršová; Lubomira Lukacikova; Martina Lickova; Martina Liková; Martina Nebohacova; Martina Neboháová; Monika Slavikova; Monika Sláviková; Peter Sabaka; Sabina Fumacova Havlikova; Sabina Fumaová Havlíková; Tomas Vinar; Tomáš Vina; Viktoria Cabanova; Viktoria Hodorova; Viktória Hodorová; Viktória abanová; ubomíra Lukáiková
EPI_ISL_417877 to 417880	Institute of Virology, Biomedical Research Center of the Slovak Academy of Sciences, Bratislava; Public Health Authority of the Slovak Republic, Bratislava	Institute of Virology, Biomedical Research Center of the Slovak Academy of Sciences, Bratislava; Comenius University Science Park, Bratislava	Boris Klempa; Diana Rusáková; Edita Staroová; Elena Tichá; Jaroslav Budiš; Juraj Kopáek; Juraj Koi; Martina Liková; Miroslav Böhmer; Monika Slávikova; Sabina Fumaová Havlíková; Tomáš Szemeš; Werner Krampl
EPI_ISL_852633 to 852812, EPI_ISL_893743 to 893744, EPI_ISL_893746, EPI_ISL_893748, EPI_ISL_893750, EPI_ISL_893752, EPI_ISL_893755, EPI_ISL_893757, EPI_ISL_893759, EPI_ISL_893761, EPI_ISL_893764, EPI_ISL_893766, EPI_ISL_893769, EPI_ISL_893771, EPI_ISL_893773 to 893775, EPI_ISL_893777 to 893778, EPI_ISL_893781, EPI_ISL_893783, EPI_ISL_893785, EPI_ISL_918339 to 918358, EPI_ISL_1195302 to 1195372, EPI_ISL_1195376, EPI_ISL_1195378 to 1195411, EPI_ISL_1195413 to 1195421, EPI_ISL_1195425 to 1195426			
see above	Institute of Virology, Medical Center, University of Freiburg, Freiburg, Germany	Institute of Virology, Clinial Virus Genomics, Medical Center, University of Freiburg, Freiburg, Germany	Hajo Grundmann; Jonas Fuchs; Lisa Kern; Marcus Panning; Sandra Reuter
EPI_ISL_983501 to 983587	Institute of Virology, University Hospital, University of Bonn and German Center for Infection Research (DZIF), Bonn-Cologne, Bonn, Germany	Institute of Virology, University Hospital, University of Bonn and German Center for Infection Research (DZIF), Bonn-Cologne, Bonn, Germany	Marek Korencak et al

EPI_ISL_796027 to 796063, EPI_ISL_803866, EPI_ISL_827042 to 827043	Institute of Virology, University of Cologne	Institute of Virology, University of Cologne	Alex Thielen; Alexander Dilthey; Andreas Walker; Dominik Aschenmeier; Elena Knops; Eva Heger; Gibran Rubio; Jörg Timm; Martin Däumer; Maximilian Damagnez; Rolf Kaiser; Saleta Sierra; Zevanya Tessalonica
EPI_ISL_1017679 to 1017689, EPI_ISL_1017691 to 1017700	Institute of Virology, Vaccines and Sera "Torlak"	Institute of microbiology and Immunology, Faculty of Medicine, University of Belgrade	Jankovic, M.; Jovanovic, T.; Knezevic, A.; Milicevic, O.; Sekler, M.; Tesovic, B.; Vidanovic, D.
EPI_ISL_728346, EPI_ISL_728348, EPI_ISL_728350 to 728351, EPI_ISL_728354, EPI_ISL_728356 to 728357, EPI_ISL_776711, EPI_ISL_776771			
see above	Institute of medical Microbiology and hospital Hygiene	Institute of medical Microbiology and hospital Hygiene	Aljoscha Tersteegen; Prof. Dr. Achim Kaasch
EPI_ISL_455680	Institute of pathogenic microbiology, Jiangsu Provincial Center for Disease Control and Prevention	Institute of pathogenic microbiology, Jiangsu Provincial Center for Disease Control and Prevention	Cui, L.
EPI_ISL_933732	Institution of Nefrology-Struga	Research Center for Genetic Engineering and Biotechnology "Georgi D. Efremov" , Macedonian Academy of Sciences and Arts	RCGEB - MASA
EPI_ISL_526246 to 526252	Instituto Adolfo Lutz	Instituto Adolfo Lutz Laboratório de Vírus Respiratórios	Ana Maria Sardinha Afonso; Cecília Simões Santos; Fabiana Cristina Pereira dos Santos; Katia Corrêa de Oliveira Santos; Maira do Carmo Sampaio Tavares Timenetsky; Maíra Marcele Birochi
EPI_ISL_755640, EPI_ISL_755642 to 755643, EPI_ISL_755651, EPI_ISL_755653 to 755654, EPI_ISL_776750 to 776756, EPI_ISL_776759 to 776763, EPI_ISL_792101 to 792102, EPI_ISL_792104 to 792114, EPI_ISL_833152 to 833155, EPI_ISL_833159, EPI_ISL_833161, EPI_ISL_861625 to 861627, EPI_ISL_861629 to 861634, EPI_ISL_861642 to 861643, EPI_ISL_861663, EPI_ISL_861677			
see above	Instituto Adolfo Lutz - Central	Instituto Adolfo Lutz, Interdiciplinary Procedures Center, Strategic Laboratory	Claudia Regina Gonçalves; Claudio Tavares Sacchi; Erica Valessa Ramos Gomes; Karoline Rodrigues Campos
EPI_ISL_776768, EPI_ISL_977482, EPI_ISL_1039702	Instituto Adolfo Lutz - Regional de Aracatuba	Instituto Adolfo Lutz, Interdiciplinary Procedures Center, Strategic Laboratory	Claudia Regina Gonçalves; Claudio Tavares Sacchi; Erica Valessa Ramos Gomes; Karoline Rodrigues Campos
EPI_ISL_755655, EPI_ISL_861664, EPI_ISL_906068 to 906069	Instituto Adolfo Lutz - Regional de Campinas	Instituto Adolfo Lutz, Interdiciplinary Procedures Center, Strategic Laboratory	Claudia Regina Gonçalves; Claudio Tavares Sacchi; Erica Valessa Ramos Gomes; Karoline Rodrigues Campos
EPI_ISL_776757 to 776758, EPI_ISL_776767, EPI_ISL_833163, EPI_ISL_984243 to 984244	Instituto Adolfo Lutz - Regional de Marilia	Instituto Adolfo Lutz, Interdiciplinary Procedures Center, Strategic Laboratory	Claudia Regina Gonçalves; Claudio Tavares Sacchi; Erica Valessa Ramos Gomes; Karoline Rodrigues Campos
EPI_ISL_977471, EPI_ISL_977475, EPI_ISL_977478, EPI_ISL_977480 to 977481, EPI_ISL_977485, EPI_ISL_977488, EPI_ISL_985170, EPI_ISL_1039696			
see above	Instituto Adolfo Lutz - Regional de Presidente Prudente	Instituto Adolfo Lutz, Interdiciplinary Procedures Center, Strategic Laboratory	Claudia Regina Gonçalves; Claudio Tavares Sacchi; Erica Valessa Ramos Gomes; Karoline Rodrigues Campos
EPI_ISL_735401 to 735404, EPI_ISL_735410, EPI_ISL_861667	Instituto Adolfo Lutz - Regional de Rio Claro	Instituto Adolfo Lutz, Interdiciplinary Procedures Center, Strategic Laboratory	Claudia Regina Gonçalves; Claudio Tavares Sacchi; Erica Valessa Ramos Gomes; Karoline Rodrigues Campos
EPI_ISL_755641, EPI_ISL_755646 to 755647, EPI_ISL_755649, EPI_ISL_776764 to 776766, EPI_ISL_776769, EPI_ISL_792103, EPI_ISL_833157 to 833158, EPI_ISL_833160, EPI_ISL_977486			
see above	Instituto Adolfo Lutz - Regional de Santo Andre	Instituto Adolfo Lutz, Interdiciplinary Procedures Center, Strategic Laboratory	Claudia Regina Gonçalves; Claudio Tavares Sacchi; Erica Valessa Ramos Gomes; Karoline Rodrigues Campos
EPI_ISL_735400, EPI_ISL_735427, EPI_ISL_735430	Instituto Adolfo Lutz - Regional de Santos	Instituto Adolfo Lutz, Interdiciplinary Procedures Center, Strategic Laboratory	Claudia Regina Gonçalves; Claudio Tavares Sacchi; Erica Valessa Ramos Gomes; Karoline Rodrigues Campos
EPI_ISL_833156, EPI_ISL_860317, EPI_ISL_860633	Instituto Adolfo Lutz - Regional de Sorocaba	Instituto Adolfo Lutz, Interdiciplinary Procedures Center, Strategic Laboratory	Claudia Regina Gonçalves; Claudio Tavares Sacchi; Erica Valessa Ramos Gomes; Karoline Rodrigues Campos
EPI_ISL_755648, EPI_ISL_755650, EPI_ISL_792115 to 792116, EPI_ISL_861665, EPI_ISL_861670, EPI_ISL_861679, EPI_ISL_985171 to 985174, EPI_ISL_1039699, EPI_ISL_1039703			
see above	Instituto Adolfo Lutz - Regional de Taubate	Instituto Adolfo Lutz, Interdiciplinary Procedures Center, Strategic Laboratory	Claudia Regina Gonçalves; Claudio Tavares Sacchi; Erica Valessa Ramos Gomes; Karoline Rodrigues Campos
EPI_ISL_977472 to 977474, EPI_ISL_977476 to 977477, EPI_ISL_977483 to 977484, EPI_ISL_977487, EPI_ISL_984242, EPI_ISL_984245 to 984246, EPI_ISL_985175 to 985177, EPI_ISL_1039697, EPI_ISL_1039700 to 1039701, EPI_ISL_1039705 to 1039710, EPI_ISL_1139071 to 1139074, EPI_ISL_1171620			
see above	Instituto Adolfo Lutz Central	Instituto Adolfo Lutz, Interdiciplinary Procedures Center, Strategic Laboratory	Caio Vinicius Dias Lopes; Claudia Regina Gonçalves; Claudio Tavares Sacchi; Erica Valessa Ramos Gomes; Karoline Rodrigues Campos
EPI_ISL_1181362	Instituto Aggeu Magalhães - Oswaldo Cruz Foundation, FIOCRUZ (FIOCRUZ-PE)	Laboratory of Respiratory Viruses and Measles, Oswaldo Cruz Institute, FIOCRUZ	Alice Sampaio Rocha; Ana Carolina Mendonca; Anna Carolina Paixao; Fernando Motta; Gabriel Wallau; Luciana Appolinario; Marilda Siqueira on behalf of the Fiocruz COVID-19 Genomic Surveillance Network; Paola Resende; Renata Serrano Lopes
EPI_ISL_491174 to 491297	Instituto Gulbenkian de Ciência	Instituto Gulbenkian de Ciência	Cathy Paulino; Joao Sobral; João Costa; Ricardo Leite; Susana Ladeiro
EPI_ISL_1015735	Instituto Nacional de Cancerologia	Instituto Nacional de Salud	Carlos Franco-Muñoz; Diego A. Álvarez-Díaz; Diego Andrés Prada; Gerardo Santamaría; Hector Alejandro Ruiz-Moreno; Jhonnatan Reales-González; Julian Naizaque; Katherine Laiton-Donato; Magdalena Wiesner; Marcela Mercado-Reyes; Maria T. Herrera-Sepúlveda; Martha Lucia Ospina Martinez; Sheryll Corchuelo
EPI_ISL_845634	Instituto Nacional de Cancerologia	Instituto Nacional de Salud - Dirección de Investigación en Salud Pública	Carlos Franco-Muñoz; Diego A. Álvarez-Díaz; Diego Andrés Prada; Gerardo Santamaría; Jonathan Reales; Julian Naizaque; Katherine Laiton-Donato; Magdalena Wiesner; Marcela Mercado-Reyes; Maria T. Herrera-Sepúlveda; Martha Lucia Ospina Martinez; Mauricio Pacheco-Montealegre; Paola Muñoz-Laiton; Sheryll Corchuelo
EPI_ISL_426364	Instituto Nacional de Ciencias Medicas y Nutricion Salvador Zubiran	Instituto Nacional de Ciencias Medicas y Nutricion	Adnan Araiza Rodríguez; Alejandro Sánchez; Alfredo Ponce de León Garduño; Blanca Taboada; Carlos F. Arias; Carolina González Torres; Celia Boukadida; Cesar Raúl González Bonilla; Concepción Grajales Muñiz; Edgar Mendieta Condado; Eduardo Becerril Vargas; Fabiola Garcés Ayala; Fernando Ledesma Barrientos; Francisco Javier Gaytán Cervantes; Francisco Pulido; Gisela Barrera Badillo; Gloria Vázquez; Guillermo M. Ruiz-Palacios; Irma López Martínez; Joel Armando Vázquez Pérez; José Arturo Martínez Orozco; José Ernesto Ramírez González; José Esteban Muñoz Medina; Lucia Hernández Rivas; Luis Alberto García Andrade; Mario Mújica Sánchez; Pavel Isa; Pilar Ramos Cervantes; Ricardo Grande; Santiago Avila Ríos; Victor Hugo Borja Aburto; Violeta Ibarra Gonzalez
EPI_ISL_426361 to 426363, EPI_ISL_426365	Instituto Nacional de Ciencias Medicas y Nutricion Salvador Zubiran	Instituto Nacional de Ciencias Medicas y Nutricion Salvador Zubiran	Adnan Araiza Rodríguez; Alejandro Sánchez; Alfredo Ponce de León Garduño; Blanca Taboada; Carlos F. Arias; Carolina González Torres; Celia Boukadida; Cesar Raúl González Bonilla; Concepción Grajales Muñiz; Edgar Mendieta Condado; Eduardo Becerril Vargas; Fabiola Garcés Ayala; Fernando Ledesma Barrientos; Francisco Javier Gaytán Cervantes; Francisco Pulido; Gisela Barrera Badillo; Gloria Vázquez; Guillermo M. Ruiz-Palacios; Irma López Martínez; Joel Armando Vázquez Pérez; José Arturo Martínez Orozco; José Ernesto Ramírez González; José Esteban Muñoz Medina; Lucia Hernández Rivas; Luis Alberto García Andrade; Mario Mújica Sánchez; Pavel Isa; Pilar Ramos Cervantes; Ricardo Grande; Santiago Avila Ríos; Victor Hugo Borja Aburto; Violeta Ibarra Gonzalez
EPI_ISL_424345, EPI_ISL_424348, EPI_ISL_424625 to 424627	Instituto Nacional de Enfermedades Respiratorias	Instituto Nacional de Enfermedades Respiratorias	Adnan Araiza Rodríguez; Alejandro Sánchez; Alfredo Ponce de León Garduño; Blanca Taboada; Carlos F. Arias.; Carolina González Torres; Celia Boukadida; Cesar Raúl González Bonilla; Concepción Grajales Muñiz; Edgar Mendieta Condado; Eduardo Becerril Vargas; Fabiola Garcés Ayala; Fernando Ledesma Barrientos; Francisco Javier Gaytán Cervantes; Francisco Pulido; Gisela Barrera Badillo; Gloria Vázquez; Guillermo M. Ruiz-Palacios; Irma López Martínez; Joel Armando Vázquez Pérez; Jorge Salas Hernández; José Arturo Martínez Orozco; José Ernesto Ramírez González; José Esteban Muñoz Medina; Lucia Hernández Rivas; Luis Alberto García Andrade; Mario Mújica Sánchez; Pavel Isa; Pilar Ramos Cervantes; Ricardo Grande; Santiago Avila Ríos; Victor Hugo Borja Aburto; Violeta Ibarra Gonzalez
EPI_ISL_412972	Instituto Nacional de Enfermedades Respiratorias	Instituto de Diagnostico y Referencia Epidemiologicos (INDRE)	Araiza-Rodriguez Adnan; Arias Carlos; Barrera-Badillo Gisela; Boukadida Celia; Garces-Ayala Fabiola; Hernandez-Rivas Lucia; Isa Pavel; Lopez Susana; Lopez-Martinez Irma; Martinez Arturo; Mendieta-Condado Edgar; Munoz-Medina Esteban; Ramirez-Gonzalez Ernesto; Rodriguez-Maldonado Abril; Sanchez Alejandro; Taboada Blanca; Vazquez-Perez Joel; Wong-Arambula Claudia
EPI_ISL_837600 to 837814, EPI_ISL_843191 to	Instituto Nacional de Enfermedades Respiratorias (INER)	Instituto Nacional de Enfermedades Respiratorias (INER)	Alejandra Hernández-Terán; Alma Rincón-Rubio; Celia Boukadida; Edgar Sevilla-Reyes; Eduardo Becerril-Vargas; Fidencio Mejía-Nepomuceno; Hector

843192			Esteban Paz-Juárez; Joel Armando Vázquez-Pérez; Jorge Salas-Hernández; José Arturo Martínez-Orozco; Margarita Matías-Florentino; Mario Müjica-Sánchez; Olivia Briceño; Santiago Ávila-Ríos
EPI_ISL_1301445, EPI_ISL_1301452 to 1301453, EPI_ISL_1301465, EPI_ISL_1301471 to 1301472, EPI_ISL_1301488 to 1301490, EPI_ISL_1301499, EPI_ISL_1301503 to 1301504, EPI_ISL_1301506 to 1301507, EPI_ISL_1301510, EPI_ISL_1301515, EPI_ISL_1301520, EPI_ISL_1301522, EPI_ISL_1301524, EPI_ISL_1301533, EPI_ISL_1301539, EPI_ISL_1301545, EPI_ISL_1301550 to 1301551, EPI_ISL_1301554, EPI_ISL_1301598, EPI_ISL_1301600, EPI_ISL_1301602 to 1301619, EPI_ISL_1301717 to 1301729, EPI_ISL_1301731 to 1301733, EPI_ISL_1302152, EPI_ISL_1302155, EPI_ISL_1302159 to 1302160, EPI_ISL_1302163, EPI_ISL_1302170 to 1302171, EPI_ISL_1302179 to 1302180, EPI_ISL_1302185 to 1302186, EPI_ISL_1302191 to 1302194, EPI_ISL_1302198, EPI_ISL_1302205 to 1302208, EPI_ISL_1302227 to 1302234, EPI_ISL_1302245 to 1302247, EPI_ISL_1302249 to 1302250, EPI_ISL_1302253, EPI_ISL_1302255 to 1302258, EPI_ISL_1302263, EPI_ISL_1302266 to 1302267, EPI_ISL_1302271, EPI_ISL_1302273, EPI_ISL_1302277 to 1302278, EPI_ISL_1302280, EPI_ISL_1302290 to 1302292, EPI_ISL_1302297 to 1302299, EPI_ISL_1302301 to 1302304, EPI_ISL_1302306 to 1302308, EPI_ISL_1302312, EPI_ISL_1302316 to 1302317, EPI_ISL_1302319 to 1302320, EPI_ISL_1302325 to 1302330, EPI_ISL_1302334, EPI_ISL_1302336, EPI_ISL_1302338 to 1302339, EPI_ISL_1302345 to 1302346, EPI_ISL_1302349 to 1302350, EPI_ISL_1302356 to 1302357, EPI_ISL_1302360 to 1302361, EPI_ISL_1302364 to 1302366, EPI_ISL_1302368 to 1302369, EPI_ISL_1302373 to 1302377, EPI_ISL_1302381, EPI_ISL_1302383 to 1302387, EPI_ISL_1302389, EPI_ISL_1302391, EPI_ISL_1302394 to 1302395, EPI_ISL_1302399 to 1302400			
see above	Instituto Nacional de Enfermedades Respiratorias (INER)	Instituto de Biotecnología de la UNAM	Alejandra Hernández-Terán; Alejandro Sanchez-Flores; Alma Rincón-Rubio; Andrea Santos Coy-Arechavaleta; Authors from IBT; Blanca Taboada; Celia Boukadida; Clara Esperanza Santacruz-Tinoco; Edgar Mendieta-Condado; Eduardo Becerril-Vargas; Fidencio Mejía-Nepomuceno; Francisco Pulido; Gisela Barrera-Badillo; Gloria Vazquez; Hector Esteban Paz-Juárez; IMSS; InDRE and INER (in alphabetical order): Carlos F. Arias; Irma Lopez-Martinez; Jerome Jean Verleyen; Joel Armando Vázquez-Pérez; Jorge Salas-Hernández; José Arturo Martínez-Orozco; José Ernesto Ramírez-González; José Esteban Muñoz-Medina; Larissa Fernandes-Matano; Lucia Hernandez-Rivas; Luis Alberto Ochoa-Carrera; Margarita Matías-Florentino; Mario Müjica-Sánchez; Natividad Cruz-Ortiz; Pavel Isa; Ricardo Grande; Santiago Ávila-Ríos; Tatiana Nunez-Garcia; Teresita Rojas-Mendoza
EPI_ISL_792351 to 792353	Instituto Nacional de Epidemiología Dr. Jara	Área de Secuenciación del Laboratorio de Virología del Hospital de Niños Dr. Ricardo Gutierrez on behalf of 'Proyecto Argentino Interinstitucional de genomica de SARS-CoV-2' (PAIS Consortium)	CJ; Cimmino; Goya; I; LE; Lusso; MI; MS; Nabaes Jodar; Natale; O; Pagano; S; Uez; Valinotto; Viegas, M.
EPI_ISL_574294	Instituto Nacional de Investigacion en Salud Pública	Instituto Nacional de Investigación en Salud Pública	Alberto Orlando Narvaez; Alfredo Bruno; Andrés Carrasco; Denisse Portugal; Doménica de Mora Coloma; Leandro Patino Patino; Manuel Gonzalez; Orson Mestanza
EPI_ISL_574292	Instituto Nacional de Investigación en Salud Pública	Instituto Nacional de Investigación en Salud Pública	Alberto Orlando Narvaez.; Alfredo Bruno Caicedo; Andrés Carrasco; Denisse Portugal; Domenica de Mora Coloma; Leandro Patino Patino; Manuel Gonzalez; Orson Mestanza
EPI_ISL_574293	Instituto Nacional de Investigación en Salud Pública	Instituto Nacional de Investigación en Salud Publica	Alberto Orlando Narvaez.; Alfredo Bruno Caicedo; Andrés Carrasco; Denisse Portugal; Doménica de Mora Coloma; Leandro Patino Patino; Manuel Gonzalez; Orson Mestanza
EPI_ISL_491944 to 491954	Instituto Nacional de Investigación en Salud Pública - INSPI	INSPI - Charité	Alberto Orlando; Alexandra Usiña; Alfredo Bruno Caicedo; Andres Moreira-Soto; Anna-Lena Sander; Denisses Portugal; Domenica de Mora Coloma; Jan Felix Drexler; Juan Carlos Zeballos; Manuel Gonzalez; Maritza Olmedo; Nina Krause; Silvia Salgado
EPI_ISL_770129	Instituto Nacional de Investigación y Tecnología Agraria y Alimentaria (INIA)/ Departamento de Mejora Genética Animal	Instituto Nacional de Investigación y Tecnología Agraria y Alimentaria (INIA)/ Departamento de Mejora Genética Animal	Jovita Fernández-Pinero and SeqCOVID-SPAIN consortium; Miguel Ángel Jiménez-Clavero; Mónica Gutiérrez-Rivas; Oscar González-Recio; Ramón Peiró-Pastor
EPI_ISL_522873 to 522878, EPI_ISL_930783, EPI_ISL_933512 to 933531, EPI_ISL_944611 to 944633, EPI_ISL_955189 to 955209, EPI_ISL_985149 to 985167, EPI_ISL_1005559 to 1005582, EPI_ISL_1005656 to 1005673, EPI_ISL_1040535 to 1040643, EPI_ISL_1055005 to 1055025, EPI_ISL_1058046, EPI_ISL_1060598 to 1060615, EPI_ISL_1075267, EPI_ISL_1075657, EPI_ISL_1080433 to 1080453, EPI_ISL_1081152 to 1081169, EPI_ISL_1085421, EPI_ISL_1095493 to 1095516, EPI_ISL_1117353 to 1117375, EPI_ISL_1120878 to 1120896, EPI_ISL_1133103, EPI_ISL_1137460 to 1137474, EPI_ISL_1168634 to 1168652, EPI_ISL_1181700 to 1181719, EPI_ISL_1193558, EPI_ISL_1205203 to 1205218, EPI_ISL_1262630 to 1262717, EPI_ISL_1265902 to 1265908, EPI_ISL_1298451 to 1298473, EPI_ISL_1300425, EPI_ISL_1310808, EPI_ISL_1310811			
see above	Instituto Nacional de Medicina Genomica	Instituto Nacional de Medicina Genomica	Alcaraz N; Alcaraz-Millman M; Cedro-Tanda A; Cisneros-Villanueva M; Gonzalez-Barrera D; Herrera-Montalvo LA; Herrera-Montalvo LA.; Hidalgo-Miranda A; Hurtado-Cordova E; Mendoza-Vargas A; Peñaloza-Figueroa F; Ramirez-Vega O; Rangel-DeLeon D; Reyes-Grajeda JP; Sifuentes-Rojas C
EPI_ISL_522872, EPI_ISL_522879 to 522880, EPI_ISL_522940 to 522942, EPI_ISL_522978 to 522986, EPI_ISL_523500	Instituto Nacional de Medicina Genómica	Instituto Nacional de Medicina Genómica	Cedro-Tanda A; Cisneros-Villanueva M; Herrera-Montalvo LA; Hidalgo-Miranda A; Hurtado-Cordova E; Mendoza-Vargas A; Peñaloza-Figueroa F; Reyes-Grajeda JP
EPI_ISL_418262	Instituto Nacional de Salud	Instituto Nacional de Salud Universidad Cooperativa de Colombia Instituto Alexander von Humboldt Imperial College-London London School of Hygiene & Tropical Medicine	Astrid C. Flórez; Carlos Franco-Muñoz; Christian Julian VillabonaArenas; Diana Marcela Walteros-Acero; Diego A. Álvarez-Díaz; Erika Ospitia; Gloria Puerto; Jose A. Usme-Ciro; Juliana Barbosa; Katherine Laiton-Donato; Liz Villabona-Arenas; Luz Dary Rodriguez; Mailyn A.Gonzalez; Marcela Mercado-Reyes; Martha Lucia Ospina Martinez; Nicolas D. Franco-Sierra; Nuno Rodrigues Faria; Sergio Gomez Rangel; Sussy Echeverria; Zulma M. Cucunubá
EPI_ISL_536477 to 536565	Instituto Nacional de Salud	Laboratorio de Infecciones Respiratorias Agudas	David Tarazona; Dennis Carhuaricra; Eduardo Juscamayta Lopez; Faviola Valdivia Guerrero; Lenin Maturrano Hernandez; Nancy Rojas Serrano; Ronnie Gavilan Chavez
EPI_ISL_791084 to 791096	Instituto Nacional de Salud - Unidad de Secuenciación y Análisis Genómico	Instituto Nacional de Salud - Dirección de Investigación en Salud Pública	Carlos Franco-Muñoz; Diego A. Álvarez-Díaz; Diego Andrés Prada; Gerardo Santamaría; Jeadran Malagon-Rojas; Jesith Toloza; Jonathan Reales; Julia Almentero; Julian Naizaque; Katherine Laiton-Donato; Magdalena Wiesner; Marcela Mercado-Reyes; Maria T. Herrera; Martha Lucia Ospina Martinez; Mauricio Pacheco-Montealegre; Paola Muñoz-Laiton; Ronald Lopez; Sheryl Corchuelo
EPI_ISL_456116 to 456156	Instituto Nacional de Salud - Unidad de Secuenciación y Análisis Genómico	Instituto Nacional de Salud, Universidad Cooperativa de Colombia, Instituto Alexander von Humboldt, Imperial College-London, London School of Hygiene & Tropical Medicine	Astrid C. Flórez; Carlos Franco-Muñoz; Christian Julian VillabonaArenas; Diana Marcela Walteros-Acero; Diego A. Álvarez-Díaz; Erika Ospitia; Gloria Puerto; Jose A. Usme-Ciro; Juliana Barbosa; Katherine Laiton-Donato; Liz Villabona-Arenas; Luz Dary Rodriguez; Mailyn A.Gonzalez; Marcela Mercado-Reyes.; Martha Lucia Ospina Martinez; Nicolas D. Franco-Sierra; Sergio Gomez-Rangel; Sussy Echeverria; Zulma M. Cucunubá
EPI_ISL_941942 to 941943, EPI_ISL_941949, EPI_ISL_941951, EPI_ISL_941953, EPI_ISL_941955 to 941958, EPI_ISL_941960, EPI_ISL_941963, EPI_ISL_941984 to 941994, EPI_ISL_942003, EPI_ISL_942006			
see above	Instituto Nacional de Salud, Bogotá, Colombia	Centro de Investigaciones en Microbiología y Biotecnología-UR (CIMBIUR), Facultad de Ciencias Naturales, Universidad del Rosario, Bogotá, Colombia Instituto Nacional de Salud, Bogotá, Colombia Icahn School of Medicine at Mount Sinai, New York, USA	Adriana van de Guchte; Alberto Paniz-Mondolfi; Ana S. Gonzalez-Reiche; Carolina Flórez; Carolina Hernández; Emilia Mia Sordillo; Hala Alejel Alshammary; Harm van Bakel; Jayeeta Dutta; Juan David Ramírez; Luz Helena Patiño; Marina Muñoz; Matthew M. Hernandez; Nathalia Ballesteros; Sergio Gomez; Viviana Simon; Zenab Khan
EPI_ISL_447755 to 447769, EPI_ISL_447771 to 447772, EPI_ISL_447774 to 447787, EPI_ISL_447789 to 447817	Instituto Nacional de Salud, Bogotá, Colombia	Grupo de Investigaciones Microbiológicas-UR (GIMUR), Departamento de Biología, Facultad de Ciencias Naturales, Universidad del Rosario, Bogotá, Colombia Instituto Nacional de Salud, Bogotá, Colombia Icahn School of Medicine at Mount Sinai, New York, USA	Adriana Castillo; Alberto Paniz-Mondolfi; Ana S. Gonzalez-Reiche; Angelica Rico; Aníbal A. Teherán; Carolina Florez; Carolina Hernandez; David Martínez; Emilia Mia Sordillo; Esther C. Barros; Harm van Bakel; Jesús E. Jaimes; Juan David Ramírez; Laura Vega; Lisseth Pardo; Marina Muñoz; Martha L. Ospina; Matthew M. Hernandez; Nathalia Ballesteros; Sergio Castañeda; Sergio Gomez; Viviana Simon
EPI_ISL_497736, EPI_ISL_497738, EPI_ISL_497744 to 497745, EPI_ISL_498152 to 498170, EPI_ISL_526932 to 526934, EPI_ISL_526949 to 526951, EPI_ISL_526953 to 526965, EPI_ISL_526967 to 526971, EPI_ISL_526973 to 526974, EPI_ISL_653745 to 653762, EPI_ISL_653824 to 653825, EPI_ISL_654793, EPI_ISL_739663 to 739683			
see above	Instituto Nacional de Salud, Bogotá, Colombia	Instituto Nacional de Salud, Bogotá, Colombia	Astrid C. Flórez; Carlos Andrés Durán; Carlos Franco-Muñoz; Carolina Ferro; Christian Julian VillabonaArenas; Diana Marcela Walteros-Acero; Diego A. Álvarez-Díaz; Diego Andrés Prada; Edmilson F. de Oliveira-Filho; Felix Betzler; Franklin Prieto; Jeadran Malagón-Rojas; Jonathan Reales; Jose A. Usme-Ciro; Katherine Laiton-Donato; Liz Villabona-Arenas; Magdalena Weisner; Marcela Mercado-Reyes; Martha Lucia Ospina Martinez; Mauricio Pacheco-Montealegre; Nicolas D. Franco-Sierra; Sheryl Corchuelo; Sussy Echeverria; Wendy K. Jo; Zulma M. Cucunubá
EPI_ISL_956282 to 956297, EPI_ISL_1017705, EPI_ISL_1303366 to 1303367, EPI_ISL_1303376 to 1303377	Instituto Nacional de Salud- Dirección de Redes de Laboratorios de Salud Pública	Instituto Nacional de Salud- Dirección de Investigación en Salud Pública	Carlos Franco-Muñoz; Carmen Osorio; Diana Malo; Diego A. Álvarez-Díaz; Diego Andrés Prada; Gerardo Santamaría; Hector Alejandro Ruiz-Moreno; Jhonnatan Reales-González; Juan Camilo Martinez; Julian Naizaque; Katherine Laiton-Donato; Lisseth Pardo; Magdalena Wiesner; Marcela Mercado-Reyes; Maria T. Herrera-Sepúlveda; Marta Lopez Blanco; Martha Lucia Ospina Martinez; Mauricio Pacheco-Montealegre; Sergio Gomez; Sheryll Corchuelo; Ángela Alarcon Cruz
EPI_ISL_887419 to 887505, EPI_ISL_964918 to 964948, EPI_ISL_1132818 to 1132844	Instituto Nacional de Saude (INS), Mozambique	KRISP, KZN Research Innovation and Sequencing Platform	Emmanuel S; Giandhari J; Nadia Sitoe; Nalia Ismael; Nedio Mabunda; Paulo Arnaldo; Pillay S; Tegally H; Wilkinson E; de Oliveira T
EPI_ISL_421450 to 421452, EPI_ISL_421456, EPI_ISL_421472 to 421478, EPI_ISL_421496 to 421499, EPI_ISL_510889 to 511181, EPI_ISL_511189, EPI_ISL_511316 to 511483, EPI_ISL_511509 to 511576, EPI_ISL_511579 to 511643, EPI_ISL_511645 to 511750, EPI_ISL_693517, EPI_ISL_693521 to 693522, EPI_ISL_693525 to 693533, EPI_ISL_693538, EPI_ISL_693540 to 693629, EPI_ISL_731895 to 731896, EPI_ISL_731898 to 731899, EPI_ISL_731901 to 731902, EPI_ISL_731904 to 731905, EPI_ISL_731908 to 731911, EPI_ISL_731913, EPI_ISL_731916, EPI_ISL_731918 to 731923, EPI_ISL_731931 to 731939, EPI_ISL_731941 to 732117, EPI_ISL_738088 to 738092, EPI_ISL_738094 to 738095, EPI_ISL_738103 to 738108, EPI_ISL_738110 to 738120, EPI_ISL_738146, EPI_ISL_759707 to 759723, EPI_ISL_759761 to 759762, EPI_ISL_765210 to 765215, EPI_ISL_765219 to 765221, EPI_ISL_796733 to 796781, EPI_ISL_801516 to 801521, EPI_ISL_861529 to 861624, EPI_ISL_861707 to 861709, EPI_ISL_941339 to 941646, EPI_ISL_1023448, EPI_ISL_1023450, EPI_ISL_1023452, EPI_ISL_1023454, EPI_ISL_1023456 to 1023457, EPI_ISL_1023459, EPI_ISL_1023461, EPI_ISL_1023463, EPI_ISL_1023465, EPI_ISL_1023467 to 1023468, EPI_ISL_1023470, EPI_ISL_1116594,			

EPI_ISL_1116596 to 1116598, EPI_ISL_1116602 to 1116605, EPI_ISL_1116607 to 1116612, EPI_ISL_1116614 to 1116623, EPI_ISL_1116625 to 1116636, EPI_ISL_1116638 to 1116646, EPI_ISL_1116649 to 1116659, EPI_ISL_1116661, EPI_ISL_1116665 to 1116666, EPI_ISL_1116670 to 1116671, EPI_ISL_1116688 to 1116757, EPI_ISL_1116759 to 1116775, EPI_ISL_1116777 to 1116779, EPI_ISL_1116783, EPI_ISL_1116786 to 1116788, EPI_ISL_1116790 to 1116795, EPI_ISL_1116797 to 1116815, EPI_ISL_1116857, EPI_ISL_1116860 to 1116861, EPI_ISL_1116865, EPI_ISL_1116867 to 1116871, EPI_ISL_1116890 to 1116946, EPI_ISL_1116995, EPI_ISL_1116999 to 1117000, EPI_ISL_1117002 to 1117006, EPI_ISL_1117009 to 1117013, EPI_ISL_1117016 to 1117017, EPI_ISL_1117019, EPI_ISL_1117021, EPI_ISL_1117023, EPI_ISL_1117025 to 1117030, EPI_ISL_1117035 to 1117042, EPI_ISL_1117044 to 1117045, EPI_ISL_1117047 to 1117056, EPI_ISL_1117063 to 1117064, EPI_ISL_1117084 to 1117150, EPI_ISL_1117152 to 1117199, EPI_ISL_1117201 to 1117230, EPI_ISL_1170126, EPI_ISL_1170131 to 1170134, EPI_ISL_1170138, EPI_ISL_1170140 to 1170146, EPI_ISL_1170150, EPI_ISL_1170154 to 1170158, EPI_ISL_1170161 to 1170162, EPI_ISL_1170164 to 1170175, EPI_ISL_1170177 to 1170189, EPI_ISL_1170192, EPI_ISL_1170194 to 1170204, EPI_ISL_1170207 to 1170208, EPI_ISL_1170210, EPI_ISL_1170214 to 1170223, EPI_ISL_1170225 to 1170226, EPI_ISL_1170232, EPI_ISL_1171966 to 1172013, EPI_ISL_1191070 to 1191074, EPI_ISL_1260855 to 1260861			
see above	Instituto Nacional de Saude (INSA)	Instituto Nacional de Saude (INSA)	Borges et al; Guiomar et al
EPI_ISL_1170127 to 1170130, EPI_ISL_1170139, EPI_ISL_1170151, EPI_ISL_1170153, EPI_ISL_1170163, EPI_ISL_1170211 to 1170213	Instituto Nacional de Saude (INSA)	Instituto Nacional de Saude (INSA) and Institute of Biomedicine (iBiMed), Universidade de Aveiro	Borges et al
EPI_ISL_511484 to 511508, EPI_ISL_511751 to 511771, EPI_ISL_1170135 to 1170137, EPI_ISL_1170147 to 1170149, EPI_ISL_1170152, EPI_ISL_1170159 to 1170160, EPI_ISL_1170176, EPI_ISL_1170190 to 1170191, EPI_ISL_1170193, EPI_ISL_1170205 to 1170206, EPI_ISL_1170209, EPI_ISL_1170224			
see above	Instituto Nacional de Saude (INSA)	Instituto Nacional de Saude (INSA) and Instituto Gulbenkian de Ciencia (IGC)	Borges et al
EPI_ISL_1116599, EPI_ISL_1116662 to 1116664, EPI_ISL_1116672 to 1116687, EPI_ISL_1116863, EPI_ISL_1116883 to 1116889, EPI_ISL_1117018, EPI_ISL_1117031, EPI_ISL_1117043, EPI_ISL_1117057, EPI_ISL_1117065 to 1117083			
see above	Instituto Nacional de Saude (INSA) and Institute of Biomedicine (iBiMed), Universidade de Aveiro	Instituto Nacional de Saude (INSA) and Institute of Biomedicine (iBiMed), Universidade de Aveiro	Borges et al
EPI_ISL_511182 to 511188, EPI_ISL_511190 to 511315, EPI_ISL_731897, EPI_ISL_731900, EPI_ISL_731903, EPI_ISL_731906 to 731907, EPI_ISL_731912, EPI_ISL_731914 to 731915, EPI_ISL_731917, EPI_ISL_731924 to 731930, EPI_ISL_731940, EPI_ISL_732118 to 732293, EPI_ISL_765216 to 765218, EPI_ISL_941647 to 941900, EPI_ISL_1116595, EPI_ISL_1116606, EPI_ISL_1116624, EPI_ISL_1116647 to 1116648, EPI_ISL_1116660, EPI_ISL_1116667 to 1116669, EPI_ISL_1116816 to 1116856, EPI_ISL_1116858 to 1116859, EPI_ISL_1116862, EPI_ISL_1116864, EPI_ISL_1116866, EPI_ISL_1116872 to 1116882, EPI_ISL_1116947 to 1116994, EPI_ISL_1117007 to 1117008, EPI_ISL_1117032 to 1117034, EPI_ISL_1117046, EPI_ISL_1117058 to 1117062, EPI_ISL_1117231 to 1117282, EPI_ISL_1117284 to 1117288, EPI_ISL_1117290 to 1117352			
see above	Instituto Nacional de Saude (INSA) and Instituto Gulbenkian de Ciencia (IGC)	Instituto Nacional de Saude (INSA) and Instituto Gulbenkian de Ciencia (IGC)	Borges et al
EPI_ISL_426580	Instituto Sabin	Laboratory of Virology	Bergmann M Ribeiro; Fernando L Melo; Gustavo Barra; Ikaro A Andrade; Pedro G Mesquita; Tatsuya Nagata; Ticiane H Santa-Rita
EPI_ISL_492032 to 492048	Instituto de Biologia do Exército	Laboratório Metabolismo Macromolecular FirminoTorres de Castro, Instituto de Biofísica Carlos Chagas Filho, Universidade Federal do Rio de Janeiro	Aline Rosa Vianna de Souza; Bianca Catarina Azevedo Cabral; Caleb GM Santos; Clarissa Damaso; Elizabeth Valentin; Marcio da Costa Cipitelli; Marcos Dornelas-Ribeiro; Nádia Vaez Gonçalves da Cruz; Rodrigo Soares de Moura Neto; Rosane Silva; Tatiana LS Nogueira; Virginia Sara Grancieri do Amaral
EPI_ISL_861242, EPI_ISL_875540 to 875550, EPI_ISL_888671 to 888672, EPI_ISL_1000668, EPI_ISL_1000670 to 1000671, EPI_ISL_1000673, EPI_ISL_1000675, EPI_ISL_1000677, EPI_ISL_1109632			
see above	Instituto de Biotecnologia - UNESP-Botucatu-SP	Instituto de Biotecnologia - UNESP-Botucatu-SP	Camila Dantas Malossi; Cecília Artico Banho; Cíntia Bittar; Fábio Sossai Possebon; Guilherme Campos; Helena Lage Ferreira; Jorge A. Petroli Marchesi; João Pessoa Araújo Jr.; Leila Sabrina Ullmann; Lívia Sacchetto; Maisa C. Pereira Parra; Marília Moraes; Maurício L. Nogueira; Paula Rahal; Paulo Inacio da Costa
EPI_ISL_424673	Instituto de Diagnostico y Referencia Epidemiologicos	Instituto de Diagnostico y Referencia Epidemiologicos	Adnan Araiza Rodríguez; Alejandro Sánchez; Alfredo Ponce de León Garduño; Blanca Taboada; Carlos F. Arias; Carolina González Torres; Celia Boukadida; Cesar Raúl González Bonilla; Concepción Grajales Muñiz; Edgar Mendieta Condado; Eduardo Becerril Vargas; Fabiola Garcés Ayala; Fernando Ledesma Barrientos; Francisco Javier Gaytán Cervantes; Francisco Pulido; Gisela Barrera Badillo; Gloria Vázquez; Guillermo M. Ruiz-Palacios; Irma López Martínez; Joel Armando Vázquez Pérez; José Arturo Martínez Orozco; José Ernesto Ramírez González; José Esteban Muñoz Medina; Lucia Hernández Rivas; Luis Alberto García Andrade; Mario Mújica Sánchez; Pavel Isa; Pilar Ramos Cervantes; Ricardo Grande; Santiago Avila Ríos; Victor Hugo Borja Aburto; Violeta Ibarra Gonzalez
EPI_ISL_455432, EPI_ISL_455434 to 455439, EPI_ISL_455454 to 455456, EPI_ISL_493334 to 493349, EPI_ISL_516608 to 516625, EPI_ISL_576257 to 576279, EPI_ISL_658863, EPI_ISL_658865, EPI_ISL_658868, EPI_ISL_658870, EPI_ISL_658873, EPI_ISL_658875, EPI_ISL_658878, EPI_ISL_658880, EPI_ISL_658883, EPI_ISL_658886, EPI_ISL_658888, EPI_ISL_658891, EPI_ISL_658893, EPI_ISL_658896, EPI_ISL_658899, EPI_ISL_658901, EPI_ISL_658904, EPI_ISL_861419 to 861421, EPI_ISL_872088 to 872093, EPI_ISL_872097 to 872099			
see above	Instituto de Diagnostico y Referencia Epidemiologicos (INDRE)	Instituto de Diagnostico y Referencia Epidemiologicos (INDRE)	; Abril Rodriguez-Maldonado; Adnan Araiza-Rodriguez; Adnan-Araiza Rodríguez; Araiza-Rodriguez Adnan; Barrera-Badillo Gisela; Claudia Wong-Arambula; Dayanira Arellano-Suarez; Edgar Mendieta-Condado; Ernesto Ramirez-Gonzalez; Ernesto Ramirez-Gonzalez.; Fabiola Garces-Ayala; Garces-Ayala Fabiola; Garces-Ayala Fabiola. Ramirez-Gonzalez Ernesto; Garces-Ayala Fabiola. Taboada Ramírez Blanca. Ramirez-Gonzalez Ernesto; Garces-Ayala Fabiola. Taboada Ramirez Blanca. Ramirez-Gonzalez Ernesto; Garces-Ayala Fabiola. Taboada Ramírez Blanca. Ramirez-Gonzalez Ernesto; Gaudalupe Herrera-Ramirez; Gisela Barrera-Badillo; Gisela Barrera-Badillo.; Hernandez-Rivas Lucia; Irma Lopez-Martinez; Irma Lopez-Martinez.; Jazmin Galicia-Hernandez.; Linda Andarade-Sanchez; Lopez-Martinez Irma; Lopez-Martinez Irma.; Lucia Hernandez-Rivas; Marisol Galindo-Galindo; Mendieta-Condado Edgar; Natividad Cruz-Ortiz; Octavio Ruiz-Muñiz; Pamela Ramirez-Medina; Ramirez-Gonzalez Ernesto; Ramirez-Gonzalez Ernesto.; Rodriguez-Maldonado Abril; Roman Canul-Aguilar; Taboada Ramirez Blanca; Taboada Ramírez Blanca; Taboada Ramírez Blanca.; Taboada Ramírez Blanca. Ramirez-Gonzalez Ernesto; Tatiana Nunez-Garcia; Wong-Arambula Claudia; Wong-Arambula Claudia.
EPI_ISL_452139, EPI_ISL_452141	Instituto de Diagnostico y Referencia Epidemiologicos (INDRE)	Instituto de diagnóstico y Referencia Epidemiologicos (INDRE)	Araiza-Rodriguez Adnan; Barrera-Badillo Gisela; Garces-Ayala Fabiola; Hernandez-Rivas Lucia; Lopez-Martinez Irma; Lopez-Martinez Irma.; Mendieta-Condado Edgar; Ramirez-Gonzalez Ernesto; Rodriguez-Maldonado Abril; Wong-Arambula Claudia
EPI_ISL_660068	Instituto de Diagnostico y Referencia Epidemiologicos (INDRE)	Instituto de diagnóstico y Referencia Epidemiologicos (INDRE) Departamento de Virología	Abril Rodriguez-Maldonado; Claudia Wong-Arambula; Dayanira Arellano-Suarez; Ernesto Ramirez-Gonzalez.; Fabiola Garces-Ayala; Gisela Barrera-Badillo; Irma Lopez-Martinez; Lucia Hernandez-Rivas; Natividad Cruz-Ortiz; Tatiana Nunez-Garcia
EPI_ISL_1060676 to 1060763	Instituto de Diagnostico y Referencia Epidemiologicos (INDRE)_RNLSP	Instituto de Diagnostico y Referencia Epidemiologicos (INDRE)	Abril Rodriguez-Maldonado; Adnan Araiza-Rodriguez; Claudia Wong-Arambula; David Fragoso-Fonseca; Ernesto Ramirez-Gonzalez.; Fabiola Garces-Ayala; Gisela Barrera-Badillo; Irma Lopez-Martinez; Lucia Hernandez-Rivas; Mayra Jimenez-Morales; Nancy Munoz-Hernandez; Natividad Cruz-Ortiz; Sergio Rangel-Guerrero; Tatiana Nunez-Garcia
EPI_ISL_913911 to 913984, EPI_ISL_914878 to 914880, EPI_ISL_933661 to 933712, EPI_ISL_942928 to 942929, EPI_ISL_1054940 to 1054996, EPI_ISL_1168452 to 1168633, EPI_ISL_1219711 to 1219713, EPI_ISL_1219715 to 1219718			
see above	Instituto de Diagnostico y Referencia Epidemiologicos INDRE_RNLSP	Instituto de Diagnostico y Referencia Epidemiologicos (INDRE)	Abril Rodriguez-Maldonado; Adnan Araiza-Rodriguez; Ariadna Medina-Benitez; Claudia Wong-Arambula; David Fragoso-Fonseca; Ernesto Ramirez-Gonzalez.; Fabiola Garces-Ayala; Gisela Barrera-Badillo; Irma Lopez-Martinez; Joaquin Quiroz-Mercado; Lucia Hernandez-Rivas; Mayra Jimenez-Morales; Nancy Munoz-Hernandez; Natividad Cruz-Ortiz; Sergio Rangel-Guerrero; Tatiana Nunez-Garcia; Vanessa Rivero-Arredondo
EPI_ISL_1301444, EPI_ISL_1301447 to 1301449, EPI_ISL_1301451, EPI_ISL_1301454 to 1301461, EPI_ISL_1301464, EPI_ISL_1301469 to 1301470, EPI_ISL_1301474 to 1301483, EPI_ISL_1301485 to 1301487, EPI_ISL_1301491 to 1301495, EPI_ISL_1301497, EPI_ISL_1301500 to 1301502, EPI_ISL_1301505, EPI_ISL_1301508, EPI_ISL_1301511 to 1301514, EPI_ISL_1301517, EPI_ISL_1301519, EPI_ISL_1301521, EPI_ISL_1301523, EPI_ISL_1301526 to 1301529, EPI_ISL_1301531 to 1301532, EPI_ISL_1301534, EPI_ISL_1301537, EPI_ISL_1301540 to 1301544, EPI_ISL_1301546 to 1301549, EPI_ISL_1301555 to 1301592, EPI_ISL_1301679 to 1301713, EPI_ISL_1301730, EPI_ISL_1302154, EPI_ISL_1302162, EPI_ISL_1302166 to 1302168, EPI_ISL_1302174 to 1302177, EPI_ISL_1302181 to 1302184, EPI_ISL_1302187 to 1302189, EPI_ISL_1302197, EPI_ISL_1302199 to 1302202, EPI_ISL_1302209 to 1302219, EPI_ISL_1302241 to 1302242, EPI_ISL_1302254, EPI_ISL_1302259, EPI_ISL_1302262, EPI_ISL_1302265, EPI_ISL_1302274, EPI_ISL_1302276, EPI_ISL_1302279, EPI_ISL_1302283, EPI_ISL_1302286 to 1302287, EPI_ISL_1302289, EPI_ISL_1302293, EPI_ISL_1302295 to 1302296, EPI_ISL_1302300, EPI_ISL_1302309 to 1302310, EPI_ISL_1302313 to 1302314, EPI_ISL_1302321 to 1302323, EPI_ISL_1302331, EPI_ISL_1302333, EPI_ISL_1302335, EPI_ISL_1302337, EPI_ISL_1302340, EPI_ISL_1302342 to 1302344, EPI_ISL_1302358, EPI_ISL_1302363, EPI_ISL_1302371, EPI_ISL_1302380, EPI_ISL_1302382, EPI_ISL_1302388, EPI_ISL_1302396 to 1302398			
see above	Instituto de Diagnostico y Referencia Epidemiologicos InDRE_RNLSP	Instituto de Biotecnología de la UNAM	Alejandra Hernández-Terán; Alejandro Sanchez-Flores; Alma Rincón-Rubio; Andrea Santos Coy-Arechavaleta; Authors from IBT; Blanca Taboada; Celia Boukadida; Clara Esperanza Santacruz-Tinoco; Edgar Mendieta-Condado; Eduardo Becerril-Vargas; Fidencio Mejía-Nepomuceno; Francisco Pulido; Gisela Barrera-Badillo; Gloria Vazquez; Hector Esteban Paz-Juárez; IMSS; InDRE and INER (in alphabetical order): Carlos F. Arias; Irma Lopez-Martinez; Jerome Jean Verleyen; Joel Armando Vázquez-Pérez; Jorge Salas-Hernández; José Arturo Martínez-Orozco; José Ernesto Ramírez-González; José Esteban Muñoz-Medina; Larissa Fernandes-Matano; Lucia Hernandez-Rivas; Luis Alberto Ochoa-Carrera; Margarita Matías-Florentino; Mario Mújica-Sánchez; Natividad Cruz-Ortiz; Pavel Isa; Ricardo Grande; Santiago Ávila-Ríos; Tatiana Nunez-Garcia; Teresita Rojas-Mendoza
EPI_ISL_424666	Instituto de Diagnóstico y Referencia Epidemiológicos	Instituto de Diagnóstico y Referencia Epidemiológicos	Adnan Araiza Rodríguez; Alejandro Sánchez; Alfredo Ponce de León Garduño; Blanca Taboada; Carlos F. Arias.; Carolina González Torres; Celia Boukadida; Cesar Raúl González Bonilla; Concepción Grajales Muñiz; Edgar Mendieta Condado; Eduardo Becerril Vargas; Fabiola Garcés Ayala; Fernando Ledesma Barrientos; Francisco Javier Gaytán Cervantes; Francisco Pulido; Gisela Barrera Badillo; Gloria Vázquez; Guillermo M. Ruiz-Palacios; Irma López Martínez; Joel Armando Vázquez Pérez; José Arturo Martínez Orozco; José Ernesto Ramírez González; José Esteban Muñoz Medina; Lucia Hernández Rivas; Luis Alberto García Andrade; Mario Mújica Sánchez; Pavel Isa; Pilar Ramos Cervantes; Ricardo Grande; Santiago Avila Ríos; Victor Hugo Borja Aburto; Violeta Ibarra Gonzalez
EPI_ISL_747615, EPI_ISL_748138 to 748145	Instituto de Investigaciones Biologicas Clemente Estable	Institut Pasteur de Montevideo	Ana Carolina Mendonça; Andres Lizasoain; Camila Simoes; Cecilia Alonso; Cecilia Salazar; Daiana Mir; Fernando Lopez-Tort; Fernando Motta; Gonzalo

			Bello; Ighor Arantes; Ignacio Ferrés; Jose Sotelo; Leticia Maya; Letícia Garay Martins; Luciana Appolinario; Lucia Spangenberg; Mailen Arleo; Mariana Brandes; Marilda Mendonça Siqueira; Marilda Tereza Mar da Rosa; Maria Jose Benitez-Galeano; Martín Graña; Matias Castells; Matias Victoria; Matías Salvo; Natalia Rego; Natalia Reyes; Pablo Smircich; Paola Cristina Resende; Rodney Colina; Tamara Fernandez-Calero; Tania Possi; Tatiana Schäffer Gregianini; Veronica Noya; Yasser Vega
EPI_ISL_510064 to 510077, EPI_ISL_510461 to 510466	Instituto de Investigaciones Biomédicas de Barcelona (CSIC), Hospital Clinic i Provincial de Barcelona, Instituto de Biomedicina de Valencia (CSIC), Hospital de Sant Pau	SeqCOVID-SPAIN consortium/IBV(CSIC)	Alex Soriano; Andrea Vergara; Anna M. Planas; Israel Fernández Cadenas and SeqCOVID-SPAIN consortium; Jordi Pérez Tur; Miguel J. Martínez; Mª Angeles Marcos
EPI_ISL_792320 to 792332	Instituto de Investigaciones Biomédicas en Retrovirus y SIDA	Área de Secuenciación del Laboratorio de Virología del Hospital de Niños Dr. Ricardo Gutierrez on behalf of 'Proyecto Argentino Interinstitucional de genomica de SARS-CoV-2' (PAIS Consortium)	F; Goya; H; LE; Lusso; MI; MS; Nabaes Jodar; Natale; Remes Lenicov; S; Salomón; Seery; V; Valinotto; Viegas, M.
EPI_ISL_722130 to 722133	Instituto de Medicina Tropical Universidade de São Paulo	Laboratório de Parasitologia Médica - Instituto de Medicina Tropical - Universidade de São Paulo	Brazil-UK Centre for Arbovirus Discovery Diagnosis Genomics and Epidemiology (CADDE) Genomic Network - Instituto de Medicina Tropical
EPI_ISL_729869 to 729871, EPI_ISL_729902 to 729919	Instituto de Medicina Tropical, Universidad Nacional Toribio Rodríguez de Mendoza de Amazonas	Laboratorio de Genómica Microbiana, Universidad Peruana Cayetano Heredia	Alejandra Dávila-Barclay; Brenda Ayzanoa; Carla Montenegro; Cecilia Pajuelo; Janet Huancachoque; Luis González; Pablo Tsukayama; Pedro E. Romero; Pool Marcos; Rafael Tapia; Stella Chenet
EPI_ISL_500864	Instituto de Pesquisas Biomédicas, Hospital Naval Marcílio Dias	Bioinformatics Laboratory / LNCC	Albina Luciana da Silva Freitas; Alexandra Lehmkuhl Gerber; Amilcar Tanuri; Ana Tereza Vasconcelos; Andressa Rangel de Oliveira Lima; Carolina Moreira Voloch; Claudio Alberto Mule Monteiro; Cynthia Chester Cardoso; Fernanda Conceição Silva do Amorim; Isabel de Medeiros Magalhaes de As; Luana Ferreira Martins de Toledo; Luiz Gonzaga Paula de Almeida; Marlon Daniel Lima Tonin; Mônica Gomes Monteiro da R. Santos; Shana Priscila Coutinho Barroso; Átila Duque Rossi
EPI_ISL_871972, EPI_ISL_871996 to 871998, EPI_ISL_1059943	Instituto de Salud Carlos III	Instituto de Salud Carlos III	A. Monzón; F. Casas; I. Gil, G.; I. Jiménez; Iglesias-Caballero; M. Camarero; M. Cuesta, I.; M. González-Esguevillas; M. Pozo; M. Sandonís; M. Zaballos; P. Jiménez; P. Zaballos; S. Camarero; S. Cuesta; S. Juliá; S. Molinero Calamita; S. Pozo; S. Varona; V. Vázquez-Morón
EPI_ISL_499083	Instituto de Virologia "Dr. J. M. Vanella", Facultad de Ciencias Medicas, Universidad Nacional de Cordoba. Laboratorio Central de la Provincia de Cordoba, Argentina. Ministerio de Salud de la provincia de Cordoba, Argentina.	Laboratorio de Virología, Hospital de Niños Ricardo Gutiérrez, CABA, Argentina.	Adrian Diaz; Brenda Konigheim; Gabriela Barbas; Gonzalo Castro; Javier Aguilar; Lorena Spinsanti; Mariana Viegas.; Mercedes Nabaes; Monica Natale; Sandra Gallego; Sebastian Blanco; Silvina Lusso; Stephanie Goya
EPI_ISL_485575 to 485582, EPI_ISL_485584 to 485602	Instituto de diagnóstico y Referencia Epidemiologicos (INDRE)	Instituto de diagnóstico y Referencia Epidemiologicos (INDRE)	Barrera-Badillo, G.; Ramirez-Gonzalez, E.
EPI_ISL_745090	Instutite for Medical Microbiology, School of Medicine, University of Goettingen	Institut of Human Genetics, University Medicine Goettingen	Gross U; Wollnik B; Zautner A; Zibat A
EPI_ISL_812250	Invenimus AG	Institute of Medical Virology, University of Zurich	Alexandra Trkola; Annette Audigé; Cyril Shah; Guido Bloemberg; Jon Huder; Jürg Böni; Kevin Steiner; Maria Grünberg; Maryam Zaheri; Michael Huber; Riccarda Capaul; Stefan Schmutz; Verena Kufner
EPI_ISL_1220061 to 1220065	Investigaciones Biomédicas - Universidad de Sucre	Instituto Nacional de Salud- Dirección de Investigación en Salud Pública	Carlos Franco-Muñoz; Diego A. Álvarez-Díaz; Diego Andrés Prada; Gerardo Santamaría; Hector Alejandro Ruiz-Moreno; Jhonnatan Reales-González; Julian Naizaque; Katherine Laiton-Donato; Magdalena Wiesner; Marcela Mercado-Reyes.; Maria T. Herrera-Sepúlveda; Martha Lucia Ospina Martínez; Sheryll Corchuelo
EPI_ISL_413553 to 413554, EPI_ISL_413904 to 413906, EPI_ISL_413949, EPI_ISL_413955, EPI_ISL_414371 to 414374, EPI_ISL_414475, EPI_ISL_414945 to 414946, EPI_ISL_414948			
see above	Iran National Influenza Center	Iran National Influenza Center	Ahmad Nejati; Fatemeh Ajami Nejad; Fatemeh AjamiNejad; Fatemeh Ajaminejad; Fatemeh Ajaminejad and Talat Mokhtari Azad; Fatemeh Saadatmand and Talat Mokhtari Azad; Jila Yavarian; Kaveh Sadeghi; Kaveh Sadeghi and Talat Mokhtari Azad; Najmeh Parhizghari; Nastaran Ghavvami; Nastaran Ghavvami and Talat Mokhtari Azad; Nazanin Zahra Shafiei Jandaghi; Nazanin Zahra Shafiei Jandaghi and Talat Mokhtari Azad; Saeedeh Mahfozi; Saeedeh Mahfozi and Talat Mokhtari Azad; Simin Abbasi; Simin Abbasi and Talat Mokhtari Azad; Soad Ghabeshi and Talat Mokhtari Azad; Vahid Salimi
EPI_ISL_1261701 to 1261774	Iressef Genomics lab	IRESSEF	Abdou PADANE; Abdoulie KANTEH; Abdul Karim SESAY; Ambroise AHOUIDI; Aminata DIA; Aminata MBOUP; Astou Gaye GAYE; Barada CISSE; Birahim Piere NDIAYE; Gora LO; Khadim GUEYE; Moustapha MBOW; Nafisatou LEYE; Ndeye Coumba Toure KANE; Papa Alassane DIAW; Souleymane MBOUP; Yacine DIA
EPI_ISL_1167125 to 1167184	Iressef Genomics lab	L'institut de Recherche en Santé, de Surveillance Épidémiologique et de Formation (IRESSEF)	Abdou PADANE; Abdoulie KANTEH; Abdul Karim SESAY; Ambroise AHOUIDI; Aminata DIA; Aminata MBOUP; Astou Gaye GAYE; Barada CISSE; Birahim Piere NDIAYE; Gora LO; Khadim GUEYE; Moustapha MBOW; Nafisatou LEYE; Ndeye Coumba Toure KANE; Papa Alassane DIAW; Souleymane MBOUP; Yacine DIA
EPI_ISL_836978	Irmandade da Santa Casa de Misericordia de Lorena	Instituto Adolfo Lutz, Interdiciplinary Procedures Center, Strategic Laboratory	Claudia Regina Gonçalves; Claudio Tavares Sacchi; Erica Valessa Ramos Gomes; Karoline Rodrigues Campos
EPI_ISL_918428	Isar-Amper-Klinikum Haar	Bundeswehr Institute of Microbiology	Alexandra Rehn; Enrico Georgi; Malena Bestehorn-Willmann; Markus Antwerpen; Mathias Walter; Roman Wölfel; Sabine Zange
EPI_ISL_862858	Isehara Kyodo Hospital	Fujita health University, School of Medicine, Department of Microbiology	Masahiro suzuki; Yohei Doi
EPI_ISL_419299 to 419300, EPI_ISL_479796	Ishikawa Prefectural Institute of Public Health and Environmental Science	Pathogen Genomics Center, National Institute of Infectious Diseases	Eri Nariai; Hajime Kamiya; Kentaro Itokawa; Makoto Kuroda; Masanori Hashino; Motoi Suzuki; Rina Tanaka; Sanae Kuramoto; Tsuyoshi Sekizuka
EPI_ISL_956311	Islamic Hospital	Institute of Tropical Disease, Universitas Airlangga	Aldise M Nastri; Dodo Anondo; Gatot Soegiarto; Jezzy R Dewantari; Kazufumi Shimizu; Krisnoadi Rahardjo; Laksmi Wulandari; Maria I Lusida; Resti Yudhawati; Rima R Prasetya; Soetjipto; Yasuko Mori
EPI_ISL_956385 to 956401	Isolation - Virology Unit, Institut Pasteur du Cambodge; Sequencing - US National Institute of Allergy and Infectious Diseases Cambodia, US Naval Medical Research Unit -2, Cambodia National Institute for Public Health	Virology Unit, Institut Pasteur du Cambodge	Chau Darapheak; Chin Savuth; Erik A Karlsson; Jennifer Bohl; Jessica Manning; Jose A Garcia-Rivera; Kraing Sidonn; Ly Sovann; Sophana Chea; Sreyngim Lay; Veasna Duong; Vireak Heang; Yi Sengdoeurn
EPI_ISL_956384	Isolation - Virology Unit, Institut Pasteur du Cambodge; Sequencing - US National Institute of Allergy and Infectious Diseases Cambodia, US Naval Medical Research Unit -2, National Public Health Laboratory, National Institute for Public Health, Cambodia	Virology Unit, Institut Pasteur du Cambodge	Chau Darapheak; Chin Savuth; Erik A Karlsson; Jennifer Bohl; Jessica Manning; Jose A Garcia-Rivera; Kraing Sidonn; Ly Sovann; Sophana Chea; Sreyngim Lay; Veasna Duong; Vireak Heang; Yi Sengdoeurn
EPI_ISL_1240645 to 1240650	Israel Central Virology Laboratory	Israel National Consortium for SARS-CoV-2 sequencing	Assaf Rokney; Dana Bar-Ilan; David A. Zeevi; Efrat Dahan Bucris; Efrat Glick-Saar; Efrat Rorman; Ella Mendelson; Ephraim Fass; Eva Nachum; Gal Zizelski Valenci; Gideon Rechavi; Israel Nissan; Joseph Jaffe; Maya Davidovich Cohen; Michal Mandelboim; Mor Rubinstein; Neta Zuckerman; Omer Murik; Omri Nayshool; Oran Erster; Orna Mor; Tzvia Mann
EPI_ISL_474959 to 475025, EPI_ISL_483701, EPI_ISL_483704, EPI_ISL_483706, EPI_ISL_483708 to 483709, EPI_ISL_483711 to 483725, EPI_ISL_514266 to 514274, EPI_ISL_514276 to 514316, EPI_ISL_516884 to 516891, EPI_ISL_516893 to 516921, EPI_ISL_575332 to 575334, EPI_ISL_649060 to 649107, EPI_ISL_745034 to 745081, EPI_ISL_745083 to 745086, EPI_ISL_776569 to 776656, EPI_ISL_776775 to 776868			
see above	Israel Central Virology laboratory	Israel Central Virology laboratory	Efrat Dahan Bucris; Ella Mendelson; Michal Mandelboim; Neta Zuckerman; Oran Erster; Orna Mor
EPI_ISL_804056 to 804083, EPI_ISL_804085 to 804213, EPI_ISL_814092 to 814273, EPI_ISL_889023 to 889306, EPI_ISL_944188 to 944570, EPI_ISL_1073142 to 1073385, EPI_ISL_1073387 to 1073546, EPI_ISL_1073548, EPI_ISL_1073551 to 1073553, EPI_ISL_1073555 to 1073556, EPI_ISL_1073559 to 1073637, EPI_ISL_1209469 to 1210216, EPI_ISL_1210218 to 1210297, EPI_ISL_1210299 to 1210350, EPI_ISL_1240651 to 1240654, EPI_ISL_1240656 to 1240657, EPI_ISL_1240678 to 1240679, EPI_ISL_1259181, EPI_ISL_1259208 to 1259213, EPI_ISL_1259263 to 1259265, EPI_ISL_1259278 to 1259281			
see above	Israel Central Virology laboratory	Israel National Consortium for SARS-CoV-2 sequencing	"Neta Zuckerman; Assaf Rokney; Dana Bar-Ilan; David A. Zeevi; Efrat Dahan Bucris; Efrat Glick-Saar; Efrat Rorman; Ella Mendelson; Ephraim Fass; Eva Nachum; Gal Zizelski Valenci; Gideon Rechavi; Israel Nissan; Joseph Jaffe; Maya Davidovich Cohen; Michal Mandelboim; Mor Rubinstein; Neta Zuckerman; Omer Murik; Omri Nayshool; Oran Erster; Orna Mor; Orna Mor"; Tzvia Mann

EPI_ISL_594155 to 594162	Israel Institute for Biological Research	Israel Institute for Biological Research	Adi Beth-Din and Anat Zvi; Dana Stein; Emanuelle Mamroud; Galia Zaide; Inbar Cohen-Gihon; Libby Weiss; Ofir Israeli; Orly Laskar; Shay Weiss; Yoav Gal
EPI_ISL_474958, EPI_ISL_514275, EPI_ISL_516892	Israeli Central Virology laboratory	Israel Central Virology laboratory	Efrat Dahan Bucris; Ella Mendelson; Michal Mandelboim; Neta Zuckerman; Oran Erster
EPI_ISL_1061413	Istanbul University-Cerrahpasa, Cerrahpasa School of Medicine, COVID-19 Laboratory	Istanbul University-Cerrahpasa, Cerrahpasa School of Medicine, COVID-19 Laboratory	Ebru Yucebag; Fusun Can; Haluk Eraksoy; Kenan Midilli; Mert Ahmet Kuskucu; Okan Kadir Nohut; Serap imek Yavuz; Yesim Tuyji Tok; Zarifa Abullayeva
EPI_ISL_1042001, EPI_ISL_1063399	Istinye university genetic diseases assessment center	Istinye university genetic diseases assessment center	Msc; Mursel Caliskan; Prof Dr; Zeynep Ocak MD
EPI_ISL_824407 to 824408	Istituto Nazionale Malattie Infettive Lazzaro Spallanzani IRCCS	Istituto Nazionale Malattie Infettive Lazzaro Spallanzani IRCCS	Antonino Di Caro; Barbara Bartolini; Cesare E.M. Gruber; Concetta Castilletti; Emanuela Giombini; Francesca Colavita; Francesco Messina; Maria R. Capobianchi; Martina Rueca; Ornella Butera; Silvia Meschi
EPI_ISL_983329, EPI_ISL_1009018	Istituto Zooprofilattico Sperimentale Lazio e Toscana "M. Aleandri"	INMI Lazzaro Spallanzani IRCCS	A Cersini; A Di Caro; B Bartolini; CEM Gruber; E Giombini; F Messina; G Manna; M Rueca; MR Capobianchi; MT Scicluna; O Butera
EPI_ISL_477193 to 477194, EPI_ISL_477202 to 477203	Istituto Zooprofilattico Sperimentale Puglia e Basilicata;	Beaconlab (Bioinformatics, Evolution and Comparative Genomics lab), Dept of Biosciences, University on Mila	Chiara M.; Manzari C.; Parisi A.; Pesole G.
EPI_ISL_477195 to 477201	Istituto Zooprofilattico Sperimentale Puglia e Basilicata;	Beaconlab (Bioinformatics, Evolution and Comparative Genomics lab), Dept of Biosciences, University on Milan	Chiara M.; Manzari C.; Parisi A.; Pesole G.
EPI_ISL_451961	Istituto Zooprofilattico Sperimentale Puglia e Basilicata; Dipartimento di Bioscienze, Biotecnologie e Biofarmaceutica dell'Università degli Studi di Bari "A.Moro"; Istituto di Biomembrane. Bioenergetica e Biotecnologie Molecolari del Consiglio Nazionale delle Ricerche di Bari	Beaconlab (Bioinformatics Evolution and Comparative Genomics lab), Dept of Biosciences, University of Milan	Chiara M.; Manzari C.; Parisi A.; Pesole G.
EPI_ISL_451962	Istituto Zooprofilattico Sperimentale Puglia e Basilicata; Dipartimento di Bioscienze, Biotecnologie e Biofarmaceutica dell'Università degli Studi di Bari "A.Moro"; Istituto di Biomembrane. Bioenergetica e Biotecnologie Molecolari del Consiglio Nazionale delle Ricerche di Bari	Beaconlab (Bioinformatics, Evolution and Comparative Genomics lab), Dept of Biosciences, University on Milan	Chiara M.; Manzari C.; Parisi A.; Pesole G.
EPI_ISL_468914, EPI_ISL_469016, EPI_ISL_469018 to 469023, EPI_ISL_469050, EPI_ISL_525553 to 525574, EPI_ISL_527380	Istituto Zooprofilattico Sperimentale Puglia e Basilicata; Dipartimento di Bioscienze, Biotecnologie e Biofarmaceutica dell'Università degli Studi di Bari "A.Moro"; Istituto di Biomembrane. Bioenergetica e Biotecnologie Molecolari del Consiglio Nazionale delle Ricerche di Bari	Beaconlab (Bioinformatics, Evolution and Comparative Genomics lab), Dept of Biosciences, University on Milan	Chiara M; Chiara M.; Manzari C.; Parisi A.; Pesole G.
EPI_ISL_1163829 to 1163830, EPI_ISL_1166172 to 1166188, EPI_ISL_1169895, EPI_ISL_1212673, EPI_ISL_1212675, EPI_ISL_1212677, EPI_ISL_1212679, EPI_ISL_1212681, EPI_ISL_1212684, EPI_ISL_1212686, EPI_ISL_1212689, EPI_ISL_1212691, EPI_ISL_1212693, EPI_ISL_1212696, EPI_ISL_1212698, EPI_ISL_1212700, EPI_ISL_1296261 to 1296262, EPI_ISL_1299527 to 1299531, EPI_ISL_1299533 to 1299542, EPI_ISL_1299544			
see above	Istituto Zooprofilattico Sperimentale Umbria e Marche "Togo Rosati"	Istituto Zooprofilattico Sperimentale dell'Abruzzo e Molise "G. Caporale"	Ancora M; Biagetti M; Biagetti M.; Biagetti m; Calistri P; Cammà C; Curini V; Di Domenico M; Di Pasquale A; Giammarioli M; Giammarioli M.; Lorusso A; Mangone I; Marcacci M; Puglia I; Rinaldi A; Savini G; Scialabba S
EPI_ISL_493332 to 493333, EPI_ISL_560407, EPI_ISL_882923, EPI_ISL_913449	Istituto Zooprofilattico Sperimentale del Mezzogiorno	INMI Lazzaro Spallanzani IRCCS	A Di Caro; Antonino Di Caro; B Bartolini; B Pierri; Barbara Bartolini; C Buonerba; C.E.M Gruber; Cesare E.M. Gruber; Cesare EM Gruber; Concetta Castilletti; D Di Concilio; E Giombini; Emanuela Giombini; F Messina; Francesca Colavita; Francesco Messina; Giorgia Borriello; Giovanna Fusco; M. Rueca; MC Cuomo; MR Capobianchi; Maria R Capobianchi; Maria R. Capobianchi; Martina Rueca; Maurizio Viscardi; O Butera; Ornella Butera; P Cerino; Sergio Brandi; Silvia Meschi
EPI_ISL_1035873 to 1035899	Istituto Zooprofilattico Sperimentale del Mezzogiorno	IZSM-U.O.C. Virologia	Antonio Limone; Claudio de Martinis; Esterina De Carlo; Giovanna Fusco; Lorena Cardillo; Massimo Zollo; Maurizio Viscardi; Pellegrino Cerino; Sergio Brandi
EPI_ISL_509418 to 509423	Istituto Zooprofilattico Sperimentale del Mezzogiorno	Istituto Zooprofilattico Sperimentale del Mezzogiorno	Cozzolino, L.; Fusco, G.; Viscardi, M.
EPI_ISL_1080375 to 1080376, EPI_ISL_1080378 to 1080382, EPI_ISL_1080384 to 1080391, EPI_ISL_1080481 to 1080495, EPI_ISL_1080580 to 1080602, EPI_ISL_1173204 to 1173205	Istituto Zooprofilattico Sperimentale del Mezzogiorno	Istituto Zooprofilattico Sperimentale del Mezzogiorno-U.O.C. Virologia	Antonio Limone; Esterina De Carlo; Giovanna Fusco; Loredana Cozzolino; Loredana Cozzolino Pellegrino Cerino; Lorena Cardillo; Luigi Atripaldi; Massimo Zollo; Maurizio Viscardi; Pellegrino Cerino
EPI_ISL_1095366 to 1095383, EPI_ISL_1095385 to 1095407, EPI_ISL_1129215	Istituto Zooprofilattico Sperimentale del Mezzogiorno	Lab. Microbiologia e Virologia, Cotugno, A.O. dei Colli	Anna Perfetti; Biancamaria Pierri; Claudia Tiberio; Luigi Atripaldi; Maria Concetta Cuomo; Pellegrino Cerino
EPI_ISL_736996 to 736997	Istituto Zooprofilattico Sperimentale del Mezzogiorno	National Institute for Infectious Diseases, INMI, "L. Spallanzani" IRCCS	A. Di Caro; B. Bartolini; B. Pierri; C. Buonerba; C.E.M Gruber; D. Di Concilio; E. Giombini; F. Carletti; F. Messina; M. Rueca; M.C. Cuomo; M.R Capobianchi; M.R. Capobianchi; P. Cerino
EPI_ISL_736777 to 736890, EPI_ISL_776879 to 776993, EPI_ISL_776995 to 777009, EPI_ISL_778646 to 778817, EPI_ISL_833522 to 833570, EPI_ISL_837247 to 837330, EPI_ISL_837433 to 837504, EPI_ISL_960442 to 960447, EPI_ISL_960449 to 960661, EPI_ISL_1287411 to 1287736, EPI_ISL_1307722 to 1307799, EPI_ISL_1307842 to 1307941, EPI_ISL_1310795, EPI_ISL_1310799			
see above	Istituto Zooprofilattico Sperimentale del Mezzogiorno	TIGEM	Andrea Ballabio; Anna Manfredi; Antonio Grimaldi; Antonio Grimaldi Patrizia Annunziata Francesco Panariello Biancamaria Pierri Claudia Tiberio Valentina Bouche Chiara Colantuono Maria Concetta Cuomo Denise Di Concilio Lucio Di Filippo Anna Manfredi Marcello Salvi Antonio Limone Luigi Atripaldi Pellegrino Cerino Andrea Ballabio Davide Cacchiarelli; Antonio Grimaldi Patrizia Annunziata Francesco Panariello Biancamaria Pierri Claudia Tiberio Valentina Bouche Chiara Colantuono Maria Concetta Cuomo Denise Di Concilio Lucio Di Filippo Anna Manfredi Marcello Salvi Antonio Limone Luigi Atripaldi Pellegrino Cerino Andrea Ballabio Davide Cacchiarelli; Antonio Limone; Biancamaria Pierri; Chiara Colantuono; Davide Cacchiarelli.; Denise Di Concilio; Francesco Panariello; Lucio Di Filippo; Marcello Salvi; Maria Concetta Cuomo; Patrizia Annunziata; Pellegrino Cerino; Valentina Bouche
EPI_ISL_1254715 to 1254823, EPI_ISL_1254825 to 1255050	Istituto Zooprofilattico Sperimentale del Mezzogiorno	Telethon Institute of Genetics and Medicine (TIGEM)	Antonio Grimaldi Patrizia Annunziata Francesco Panariello Biancamaria Pierri Claudia Tiberio Valentina Bouche Chiara Colantuono Maria Concetta Cuomo Denise Di Concilio Lucio Di Filippo Anna Manfredi Marcello Salvi Antonio Limone Luigi Atripaldi Pellegrino Cerino Andrea Ballabio Davide Cacchiarelli
EPI_ISL_602304	Istituto Zooprofilattico Sperimentale del Mezzogiorno	U.O. Diagnostica Virologica Dip. Sanità Animale IZSM	Lorena Cardillo; Maurizio Viscardi; e Giovanna Fusco
EPI_ISL_1061067, EPI_ISL_1061091 to 1061098, EPI_ISL_1061100 to 1061130, EPI_ISL_1061141, EPI_ISL_1061150 to 1061154, EPI_ISL_1061157, EPI_ISL_1061192, EPI_ISL_1061206 to 1061210, EPI_ISL_1061225, EPI_ISL_1061252 to 1061259, EPI_ISL_1086065			
see above	Istituto Zooprofilattico Sperimentale del Mezzogiorno (IZSM)	Telethon Institute of Genetics and Medicine (TIGEM)	Andrea Ballabio; Anna Manfredi; Antonio Grimaldi; Antonio Limone; Biancamaria Pierri; Chiara Colantuono; Davide Cacchiarelli.; Denise Di Concilio; Francesco Panariello; Lucio Di Filippo; Marcello Salvi; Maria Concetta Cuomo; Patrizia Annunziata; Pellegrino Cerino; Valentina Bouche
EPI_ISL_763069, EPI_ISL_763071 to 763073, EPI_ISL_763094 to 763098, EPI_ISL_763138, EPI_ISL_763318, EPI_ISL_763320, EPI_ISL_763322 to 763330			
see above	Istituto Zooprofilattico Sperimentale dell' Umbria e delle Marche -Togo Rosati	Istituto Superiore di Sanità	Gabriele Vaccari; Giovanni Ianiro; Ilaria Di Bartolo; Luca De Sabato; Massimo Biagetti; Monica Giammarioli
EPI_ISL_1020160 to 1020170, EPI_ISL_1020306 to 1020309, EPI_ISL_1020313, EPI_ISL_1020328, EPI_ISL_1020330	Istituto Zooprofilattico Sperimentale dell' Umbria e delle Marche -Togo Rosati Università degli Studi di Perugia	Department of Food safety, Nutrition and Veterinary public health - Istituto Superiore di Sanità	Antonella Mencacci; Barbara Camilloni; Gabriele Vaccari; Giovanni Ianiro; Ilaria Di Bartolo; Luca De Sabato; Massimo Biagetti; Monica Giammarioli

EPI_ISL_649189 to 649191, EPI_ISL_649784, EPI_ISL_649940, EPI_ISL_653784 to 653786, EPI_ISL_653813, EPI_ISL_722873 to 722894, EPI_ISL_722901 to 722911, EPI_ISL_722913 to 722925, EPI_ISL_794745, EPI_ISL_794749, EPI_ISL_794752, EPI_ISL_794754 to 794755, EPI_ISL_794759 to 794817, EPI_ISL_876738 to 876741, EPI_ISL_876744 to 876813, EPI_ISL_1008663, EPI_ISL_1008665 to 1008677, EPI_ISL_1008679 to 1008699, EPI_ISL_1008701 to 1008702, EPI_ISL_1112227 to 1112234, EPI_ISL_1116473 to 1116474, EPI_ISL_1254652 to 1254656, EPI_ISL_1254671 to 1254674, EPI_ISL_1254688 to 1254695, EPI_ISL_1261870			
see above	Istituto Zooprofilattico Sperimentale della Puglia e della Basilicata	Istituto Zooprofilattico Sperimentale della Puglia e della Basilicata	Bianco A.; Capozzi L.; Chironna M.; Cipolletta D.; Del Sambro L.; Galante D.; Giannico A.; Loconsole D.; Manzulli V; Pace L.; Parisi A.; Ridolfi D.; Rondinone V.; Sallustio A.; Simone D.
EPI_ISL_1298476 to 1298477, EPI_ISL_1298481	Ivano-Frankivsk CCH#1	The Institute of Molecular Biology and Genetics of NASU	M.Tukalo et al.
EPI_ISL_422426 to 422427	JABER AL AHMAD AL SABAH HOSPITAL - KUWAIT CITY	Dasman Diabetes Institute	Ebaa Al-Ozairi; Fahd Al-Mulla; Qais Al-Duwairi; Rasheeba Iqbal; Sumi John
EPI_ISL_995700 to 995701, EPI_ISL_995720 to 995721	JAYANAGAR GENERAL HOSPITAL	Department of Neurovirology, National Institute of Mental Health and Neurosciences (NIMHANS)	Anita S Desai; Anson Kunjumon George; Chitra Pattabiraman; Darshan Sreenivas; Nakka Vijay Kiran Reddy; Pramada Prasad; Risha Rasheed; V Ravi
EPI_ISL_515960	JGH	Department of Neurovirology, National Institute of Mental Health and Neuroscience (NIMHANS)	Anita Desai; Chitra Pattabiraman; Harsha PK; Manjunatha Venkataswamy; Pramada Prasad; Ravi Vasanthapuram; Risha Rasheed; Shafeeq S Hameed; Vijayalakshmi Reddy
EPI_ISL_422424	Jaber Al Ahmad Al Sabah Hospital	Dasman diabetes Institute	Ebaa Al-Ozairi; Fahd Al-Mulla; Qais Al-Duwairi; Rasheeba Iqbal; Sumi John
EPI_ISL_631516, EPI_ISL_631524, EPI_ISL_631550 to 631551, EPI_ISL_631554, EPI_ISL_631571, EPI_ISL_631574 to 631575, EPI_ISL_631578, EPI_ISL_631582, EPI_ISL_631585, EPI_ISL_631590, EPI_ISL_631592 to 631593, EPI_ISL_631595, EPI_ISL_631597, EPI_ISL_631599, EPI_ISL_631601, EPI_ISL_631604, EPI_ISL_631608 to 631610, EPI_ISL_631612 to 631613, EPI_ISL_631615, EPI_ISL_631621, EPI_ISL_631634, EPI_ISL_631636 to 631637, EPI_ISL_631697 to 631698, EPI_ISL_631702 to 631706, EPI_ISL_631709, EPI_ISL_631711 to 631713, EPI_ISL_631716 to 631723, EPI_ISL_631726, EPI_ISL_631728 to 631731, EPI_ISL_631733 to 631747, EPI_ISL_631767 to 631768, EPI_ISL_631772, EPI_ISL_631775 to 631777, EPI_ISL_631788 to 631791, EPI_ISL_631795, EPI_ISL_631798, EPI_ISL_631809 to 631810, EPI_ISL_631812 to 631813, EPI_ISL_631820, EPI_ISL_631822 to 631823, EPI_ISL_631825, EPI_ISL_631833, EPI_ISL_631835 to 631836, EPI_ISL_631847, EPI_ISL_631849 to 631850, EPI_ISL_631852 to 631854, EPI_ISL_631866 to 631867, EPI_ISL_631870 to 631872, EPI_ISL_631876 to 631879, EPI_ISL_631882 to 631883, EPI_ISL_631887, EPI_ISL_631889 to 631890, EPI_ISL_631895 to 631896, EPI_ISL_631901 to 631902, EPI_ISL_631904, EPI_ISL_631909 to 631910, EPI_ISL_631921 to 631922, EPI_ISL_631925 to 631928, EPI_ISL_631930, EPI_ISL_631950 to 631951, EPI_ISL_631963, EPI_ISL_631966, EPI_ISL_631969 to 631970, EPI_ISL_631973, EPI_ISL_631979, EPI_ISL_631994, EPI_ISL_631996 to 631997, EPI_ISL_632005, EPI_ISL_632007 to 632009, EPI_ISL_632011 to 632012, EPI_ISL_632017, EPI_ISL_632021, EPI_ISL_632023, EPI_ISL_632025, EPI_ISL_632028 to 632029, EPI_ISL_632035, EPI_ISL_632037, EPI_ISL_632051, EPI_ISL_632061, EPI_ISL_632065 to 632066, EPI_ISL_632075, EPI_ISL_632086, EPI_ISL_632088, EPI_ISL_632090, EPI_ISL_632093 to 632096, EPI_ISL_632106 to 632110, EPI_ISL_632128, EPI_ISL_632130, EPI_ISL_632143, EPI_ISL_632145, EPI_ISL_632149 to 632152, EPI_ISL_632162, EPI_ISL_632166 to 632172, EPI_ISL_632180, EPI_ISL_632182, EPI_ISL_632184, EPI_ISL_632188 to 632189, EPI_ISL_632191, EPI_ISL_632195, EPI_ISL_632198, EPI_ISL_632204, EPI_ISL_632209 to 632210, EPI_ISL_632214, EPI_ISL_632219, EPI_ISL_632221, EPI_ISL_632227 to 632229, EPI_ISL_632239 to 632241, EPI_ISL_632939, EPI_ISL_632941, EPI_ISL_632944, EPI_ISL_632949 to 632950, EPI_ISL_632953, EPI_ISL_632957 to 632958, EPI_ISL_632961, EPI_ISL_632969 to 632972, EPI_ISL_632974			
see above	Jamaica Hospital Medical Center	New York City Public Health Laboratory	Jade Wang; et al.
EPI_ISL_450792 to 450799	Jamaica Ministry of Health and Wellness	Pathogen Discovery, Respiratory Viruses Branch, Division of Viral Diseases, Centers for Disease Control and Prevention	Anna Montmayeur; Anna Uehara; Bettina Bankamp; Clinton R. Paden; Haibin Wang; Jasmine Padilla; Jing Zhang; Justin Lee; Krista Queen; Rachel Marine; Suxiang Tong; Yan Li; Ying Tao; Zachary Weiner
EPI_ISL_631596, EPI_ISL_631600, EPI_ISL_631874, EPI_ISL_632179, EPI_ISL_632200	James J. Peters VA Medical Center	New York City Public Health Laboratory	Jade Wang; et al.
EPI_ISL_779255 to 779290, EPI_ISL_1233953	Jamil-ur-Rahman Center for Genome Research, Dr. Panjwani Center for Molecular Medicine and Drug Research	Jamil-ur-Rahman Center for Genome Research, Dr. Panjwani Center for Molecular Medicine and Drug Research	Ansari; Ansari, S.; I.A.; Irfan, M.; Khan; Khan, I.; Nisa, Z.; Rashid, M.; S.K.; Shakeel, M.; Zaibunnisa
EPI_ISL_451958	Jamil-ur-Rahman Center for Genome Research, Dr. Panjwani Center for Molecular Medicine and Drug Research, International Center for Chemical and Biological Sciences, University of Karachi	Jamil-ur-Rahman Center for Genome Research, Dr. Panjwani Center for Molecular Medicine and Drug Research, International Center for Chemical and Biological Sciences, University of Karachi	B.A.; I.A; Khan; Khan, S.; M.A.and Khan; Qureshi; Raza; S.A.; Shakeel, M.; Zahid, M.
EPI_ISL_416565 to 416634, EPI_ISL_454749	Japanese Quarantine Stations	Pathogen Genomics Center, National Institute of Infectious Diseases	Hajime Kamiya; Hideki Hasegawa; Ikuyo Takayama; Kentaro Itokawa; Makoto Kuroda; Masanori Hashino; Motoi Suzuki; Rina Tanaka; Shinji Saito; Takaji Wakita; Takuri Takahashi; Takuya Yamagishi; Tsutomu Kageyama; Tsuyoshi Sekizuka
EPI_ISL_862874	Japanese Red Cross Nagoya Daiichi Hospital	Fujita health University, School of Medicine, Department of Microbiology	Masahiro suzuki; Yohei Doi
EPI_ISL_862848, EPI_ISL_862856, EPI_ISL_862872 to 862873, EPI_ISL_862885	Japanese Red Cross Narita Hospital	Fujita health University, School of Medicine, Department of Microbiology	Masahiro suzuki; Yohei Doi
EPI_ISL_450821 to 450822	Jarpens HC	The Public Health Agency of Sweden	Anna Risberg; Anna-Malin Linde; Gunilla Johansson; Karin Tegmark-Wisell; Maria Lind Karlberg; Mia Brytting; Olov Svartstrom; Oskar Karlsson Lindsjo; Theresa Enkirch
EPI_ISL_860193, EPI_ISL_995731, EPI_ISL_995743 to 995745	Jayanagar General Hospital	Department of Neurovirology, National Institute of Mental Health and Neurosciences (NIMHANS)	Anita S Desai; Anson Kunjumon George; Chitra Pattabiraman; Darshan Sreenivas; Harsha.P.K; Nakka Vijay Kiran Reddy; Pramada Prasad; Risha Rasheed; V Ravi
EPI_ISL_486396	Jayanagar General Hospital to Victoria Hospital	Department of Neurovirology, National Institute of Mental Health and Neuroscience (NIMHANS)	Anita Desai; Chitra Pattabiraman; Harsha PK; Manjunatha Venkataswamy; Ravi Vasanthapuram; Risha Rasheed; Shafeeq S Hameed; Vijayalakshmi Reddy
EPI_ISL_728288	Jena University Hospital, Institute for Infectious Diseases and Infection Control	Institute for infectious medicine & hospital hygiene, CaSe-Group	Brandt; Christian; Marquet; Matthias W.; Mike; Pletz; Riccardo; Spott
EPI_ISL_728292 to 728294, EPI_ISL_728296, EPI_ISL_728298, EPI_ISL_728302 to 728303, EPI_ISL_728321, EPI_ISL_728323 to 728324, EPI_ISL_728331, EPI_ISL_728333, EPI_ISL_728335, EPI_ISL_728337 to 728338, EPI_ISL_728341, EPI_ISL_728345, EPI_ISL_763077, EPI_ISL_763086 to 763093, EPI_ISL_768633, EPI_ISL_768725 to 768727			
see above	Jena University Hospital, Institute for Infectious Diseases and Infection Control	Institute of infectious medicine & hospital hygiene, CaSe-Group	Brandt; Christian; Marquet; Matthias W.; Mike; Pletz; Riccardo; Spott
EPI_ISL_632938	Jena University Hospital; Institute for Infectious Diseases and Infection Control	Institute of infectious medicine & hospital hygiene, CaSe-Group	Brandt; Christian; Marquet; Matthias W.; Mike; Pletz; Riccardo; Spott
EPI_ISL_871820 to 871839, EPI_ISL_940566 to 940572, EPI_ISL_940576 to 940579, EPI_ISL_940584 to 940606, EPI_ISL_948973, EPI_ISL_948975, EPI_ISL_948978, EPI_ISL_948981, EPI_ISL_948984, EPI_ISL_948986, EPI_ISL_948989, EPI_ISL_948991, EPI_ISL_948994, EPI_ISL_948997, EPI_ISL_949000, EPI_ISL_949003, EPI_ISL_949005, EPI_ISL_949008, EPI_ISL_949011, EPI_ISL_949013, EPI_ISL_949016, EPI_ISL_949019, EPI_ISL_949022, EPI_ISL_949024, EPI_ISL_949070 to 949078, EPI_ISL_949082 to 949084, EPI_ISL_949086 to 949089, EPI_ISL_949092 to 949095, EPI_ISL_949097 to 949123, EPI_ISL_949126 to 949127, EPI_ISL_949129 to 949156, EPI_ISL_949158 to 949172, EPI_ISL_949174 to 949175, EPI_ISL_949254 to 949255, EPI_ISL_953376 to 953382, EPI_ISL_953384, EPI_ISL_953428 to 953429, EPI_ISL_953431 to 953435, EPI_ISL_953437, EPI_ISL_953439 to 953444, EPI_ISL_953446 to 953449, EPI_ISL_953451 to 953457, EPI_ISL_953463 to 953464, EPI_ISL_953474 to 953475, EPI_ISL_953485 to 953488, EPI_ISL_960176 to 960182, EPI_ISL_960185, EPI_ISL_960187, EPI_ISL_995495 to 995517, EPI_ISL_995520 to 995525, EPI_ISL_995527 to 995539, EPI_ISL_995541 to 995547, EPI_ISL_1014904, EPI_ISL_1014906 to 1014911, EPI_ISL_1014913 to 1014916, EPI_ISL_1014918 to 1014923, EPI_ISL_1014925 to 1014932, EPI_ISL_1014935, EPI_ISL_1041969, EPI_ISL_1041971 to 1041973, EPI_ISL_1041975 to 1041980, EPI_ISL_1041982 to 1041985, EPI_ISL_1041987 to 1041991, EPI_ISL_1041993 to 1041997, EPI_ISL_1041999 to 1042000, EPI_ISL_1098844 to 1098865, EPI_ISL_1098872 to 1098890, EPI_ISL_1128118 to 1128127, EPI_ISL_1128132 to 1128139, EPI_ISL_1138883 to 1138885, EPI_ISL_1138887, EPI_ISL_1138891 to 1138895, EPI_ISL_1138897 to 1138898, EPI_ISL_1164439 to 1164465, EPI_ISL_1193398 to 1193399, EPI_ISL_1193401 to 1193402, EPI_ISL_1193404, EPI_ISL_1193406 to 1193407, EPI_ISL_1193409 to 1193410, EPI_ISL_1193412, EPI_ISL_1223329, EPI_ISL_1223332, EPI_ISL_1223335, EPI_ISL_1223338, EPI_ISL_1223341, EPI_ISL_1223344, EPI_ISL_1223347, EPI_ISL_1223349, EPI_ISL_1223352, EPI_ISL_1223355, EPI_ISL_1223358, EPI_ISL_1223361, EPI_ISL_1223364, EPI_ISL_1223367, EPI_ISL_1223370, EPI_ISL_1223373, EPI_ISL_1223376, EPI_ISL_1223378, EPI_ISL_1223381, EPI_ISL_1223384, EPI_ISL_1223387, EPI_ISL_1223390, EPI_ISL_1223393, EPI_ISL_1223396, EPI_ISL_1223399, EPI_ISL_1223402, EPI_ISL_1223405, EPI_ISL_1223408, EPI_ISL_1240185 to 1240221, EPI_ISL_1296656 to 1296676, EPI_ISL_1297165 to 1297193, EPI_ISL_1311949 to 1311963			
see above	Jessa	Jessa	Bert Cruys; Brigitte Maes; Cruys et al. on behalf of the Jessa_cmdLab; Jessa_cmdLab
EPI_ISL_421237 to 421254, EPI_ISL_421256 to 421262, EPI_ISL_455460 to 455467	Jiangxi Province Center for Disease Control and Prevention	Jiangxi Province Center for Disease Control and Prevention	DaJin Xiao; Fang Xiao; Gang Xu; JianXiong Li; Jun Zhou; ShiWen Liu; Tian Gong; XiaoQing Liu; Xin Ran; YanNi Zhang; Ying Xiong; Yong Shi
EPI_ISL_417420	Jiangxi province Center for Disease Control and Prevention	Jiangxi province Center for Disease Control and Prevention	Li jian Xiong
EPI_ISL_412459	Jingzhou Center for Disease Control and Prevention	Hubei Provincial Center for Disease Control and Prevention	Bin Fang; Bo Yang; Bo Yu; Chunlin Mao; Faxian Zhan; Guojun Ye; Jie Hu; Jing Li; Junqiang Xu; Kun Cai; Linlin Liu; Maoyi Chen; Xiang Li; Xiao Yu; Xixiang Huo; Yongzhong Jiang.
EPI_ISL_410314, EPI_ISL_410344 to 410345	Joanna Ina Manalo Research Institute for Tropical Medicine, Molecular Biology Laboratory	Joanna Ina Manalo Research Institute for Tropical Medicine, Molecular Biology Laboratory	A.D.; Cruz; E.S.; F.M.; I.P.; J.I.; J.I. and Nicolasora; K.M. and Polotan; M.A.; Manalo; Medado; Mercado; Nicolasora; O.T.; Onza; Tujan
EPI_ISL_745135, EPI_ISL_745144	Joe Slovo Hospital	National Health Laboratory Service (NHLS), Tygerberg	Bronwyn Kleinhans; Eduan Wilkindon; Gert van Zyl; Houriiyah Tegally; Kayla Delaney; Susan Engelbrecht; Tulio de Oliveira; Wolfgang Preiser
EPI_ISL_434586 to 434588, EPI_ISL_434638 to 434640, EPI_ISL_434682 to 434691, EPI_ISL_438222 to 438247, EPI_ISL_457751 to 457823, EPI_ISL_511863 to 511877, EPI_ISL_791334 to 791487, EPI_ISL_860932 to 861104, EPI_ISL_862036 to 862038, EPI_ISL_887508 to 887586, EPI_ISL_890360, EPI_ISL_911755 to			

911917, EPI_ISL_981057 to 981201, EPI_ISL_1036273 to 1036372, EPI_ISL_1091924 to 1092004, EPI_ISL_1171771 to 1171855, EPI_ISL_1238903 to 1238993, EPI_ISL_1305819 to 1305973			
see above	Johns Hopkins Hospital Department of Pathology	Johns Hopkins Hospital Department of Pathology	Adannaya Amadi; Amanda Ernlund; C. Paul Morris; Chun Huai Luo; Craig Howser; Heba H. Mostafa; Heba Mostafa; Kristina Zudock; Mark Hopkins; Matthew Schwartz; Melanie Kirsche; Michael C. Schatz; Michael Schatz; N_di_ Trovao; Nicholas Gallagher; Nidia Trovao; Norah Sadowski; Nídiá Trovao; Oluwaseun Falade-Nwulia; Oluwaseun Nwulia-Falade; Paul Morris; Peter M. Thielen; Shirlee Wohl; Srividya Ramakrishnan; Stuart C. Ray; Thomas Mehoke; Timothy Gilpatrick; Victoria Gniazdowski; Winston Timp; Yunfan Fan; _Amanda Ernlund; _Oluwaseun Falade-Nwulia
EPI_ISL_825013	Johns Hopkins Pathology	Johns Hopkins Hospital Department of Pathology	Adannaya Amadi; C. Paul Morris; Chun Huai Luo; Heba H. Mostafa; Nicholas Gallagher
EPI_ISL_445242	Jokkmokks Halsocentral	The Public Health Agency of Sweden	Anna Risberg; Anna-Malin Linde; Karin Tegmark-Wisell; Maria Lind Karlberg; Markus Beland; Mia Brytting; Olov Svartstrom; Oskar Karlsson Lindsjo; Theresa Enkirch
EPI_ISL_455101, EPI_ISL_469055 to 469056	Jourcentralen	The Public Health Agency of Sweden	Anna Risberg; Anna-Malin Linde; Karin Tegmark-Wisell; Maria Lind Karlberg; Mattias Haukland; Mia Brytting; Olov Svartstrom; Oskar Karlsson Lindsjo; Petra Edquist; Reza Advani; Salvatore Ascione; Sandra Broddesson; Shamam Muradrasoli; Theresa Enkirch
EPI_ISL_830208, EPI_ISL_830210, EPI_ISL_830212, EPI_ISL_830223, EPI_ISL_830225, EPI_ISL_830227, EPI_ISL_830229 to 830230, EPI_ISL_830232, EPI_ISL_830234, EPI_ISL_830236, EPI_ISL_830238 to 830239, EPI_ISL_830241, EPI_ISL_830243, EPI_ISL_830571, EPI_ISL_830573, EPI_ISL_830575, EPI_ISL_830579 to 830627, EPI_ISL_861106 to 861108, EPI_ISL_861134, EPI_ISL_861144 to 861174, EPI_ISL_936060 to 936062, EPI_ISL_936067 to 936068, EPI_ISL_936070 to 936081, EPI_ISL_936083 to 936085, EPI_ISL_936087 to 936088, EPI_ISL_936090 to 936091, EPI_ISL_936094 to 936102, EPI_ISL_936144, EPI_ISL_983240 to 983242, EPI_ISL_983244, EPI_ISL_983246 to 983247, EPI_ISL_983250 to 983282, EPI_ISL_983284 to 983288, EPI_ISL_983296 to 983299, EPI_ISL_1226436, EPI_ISL_1226458, EPI_ISL_1226488, EPI_ISL_1226499, EPI_ISL_1226541 to 1226542, EPI_ISL_1226617, EPI_ISL_1226684, EPI_ISL_1226696, EPI_ISL_1226846 to 1226848, EPI_ISL_1227546 to 1227554, EPI_ISL_1227965 to 1227972, EPI_ISL_1228042			
see above	KALEIDA CENTER FOR LABORATORY MEDICINE	Wadsworth Center, New York State Department of Health	Alexis Russel; Daryl M. Lamson; Erasmus Schneider; Erica Lasek-Nesselquist; John Kelly; Jonathan Plitnick; Kirsten St. George; Matthew Shudt; Melissa A Leisner; Navjot Singh
EPI_ISL_995185 to 995188	KARWAR INSTITUTE OF MEDICAL SCIENCES	Department of Neurovirology, National Institute of Mental Health and Neurosciences (NIMHANS)	Anita S Desai; Anson Kunjumon George; Chitra Pattabiraman; Darshan Sreenivas; Nakka Vijay Kiran Reddy; Pramada Prasad; Risha Rasheed; V Ravi
EPI_ISL_995695	KASTURBA MEDICAL COLLEGE, MANGALORE	Department of Neurovirology, National Institute of Mental Health and Neurosciences (NIMHANS)	Anita S Desai; Anson Kunjumon George; Chitra Pattabiraman; Darshan Sreenivas; Nakka Vijay Kiran Reddy; Pramada Prasad; Risha Rasheed; V Ravi
EPI_ISL_486397	KC General Hospital	Department of Neurovirology, National Institute of Mental Health and Neuroscience (NIMHANS)	Anita Desai; Chitra Pattabiraman; Harsha PK; Manjunatha Venkataswamy; Ravi Vasanthapuram; Risha Rasheed; Shafeeq S Hameed; Vijayalakshmi Reddy
EPI_ISL_995189 to 995190, EPI_ISL_995694, EPI_ISL_995697 to 995699, EPI_ISL_995713 to 995714, EPI_ISL_995759 to 995762, EPI_ISL_995764	KEMPEGOWDA INTERNATIONAL AIRPORT	Department of Neurovirology, National Institute of Mental Health and Neurosciences (NIMHANS)	Anita S Desai; Anson Kunjumon George; Chitra Pattabiraman; Darshan Sreenivas; Harsha.P.K; Nakka Vijay Kiran Reddy; Pramada Prasad; Risha Rasheed; V Ravi
EPI_ISL_457845 to 457931	KEMRI-CGMR-C	KEMRI-Wellcome Trust Research Programme/KEMRI-CGMR-C Kilifi	Githinji G. et al 2020
EPI_ISL_457932 to 457936	KEMRI-Centre for Virus Research	KEMRI-Wellcome Trust Research Programme/KEMRI-CGMR-C Kilifi	Githinji G. et al 2020
EPI_ISL_568695 to 568872, EPI_ISL_806548 to 806717, EPI_ISL_855494 to 855531, EPI_ISL_855533 to 855548, EPI_ISL_968807 to 968808, EPI_ISL_968810, EPI_ISL_968813, EPI_ISL_968815, EPI_ISL_968817, EPI_ISL_968819 to 968822, EPI_ISL_968825 to 968829, EPI_ISL_968831 to 968832, EPI_ISL_968834 to 968835, EPI_ISL_968837 to 968839, EPI_ISL_968841 to 968844, EPI_ISL_968846 to 968847, EPI_ISL_968849 to 968851, EPI_ISL_968853 to 968856, EPI_ISL_968858, EPI_ISL_968860 to 968861, EPI_ISL_968863 to 968865, EPI_ISL_968868 to 968869, EPI_ISL_968871, EPI_ISL_968873, EPI_ISL_968877 to 968878, EPI_ISL_968881 to 968885, EPI_ISL_968887, EPI_ISL_968889 to 968892, EPI_ISL_968894, EPI_ISL_968896 to 968899, EPI_ISL_968901 to 968905, EPI_ISL_968907 to 968911, EPI_ISL_968913 to 968914, EPI_ISL_968916, EPI_ISL_968919 to 968920, EPI_ISL_968922, EPI_ISL_968924 to 968927, EPI_ISL_968929 to 968930, EPI_ISL_968941 to 968943, EPI_ISL_968955 to 968956, EPI_ISL_968958 to 968959, EPI_ISL_968961, EPI_ISL_968972, EPI_ISL_968984 to 968990, EPI_ISL_968992 to 968993, EPI_ISL_968996 to 968999, EPI_ISL_969001 to 969005, EPI_ISL_969007 to 969014, EPI_ISL_969016 to 969021, EPI_ISL_969023 to 969031, EPI_ISL_969041 to 969044, EPI_ISL_969046 to 969054, EPI_ISL_969056 to 969061, EPI_ISL_969063 to 969069, EPI_ISL_969071 to 969074, EPI_ISL_969245, EPI_ISL_1039223 to 1039229			
see above	KEMRI-Wellcome Trust Research Programme/KEMRI-CGMR-C Kilifi	KEMRI-Wellcome Trust Research Programme/KEMRI-CGMR-C Kilifi	Githinji et al; Githinji et al 2020
EPI_ISL_1257859	KKP Soekarno Hatta	National Institute of Health Research and Development	Arie Ardiansyah Nugraha; Hana Apsari Pawestri; Hartanti Dian Ikawati; Kartika Dewi Puspa; Krisna Pangesti; Nelly Puspandari; Subangkit; Vivi Setiawaty
EPI_ISL_1284924 to 1284941, EPI_ISL_1284943 to 1284946	KRH Klinikum Region Hannover: Institut für Med. Mikrobiologie und Krankenhaushygiene	Robert Koch Institute	
EPI_ISL_436684 to 436687, EPI_ISL_455629 to 455639, EPI_ISL_660221 to 660222, EPI_ISL_660228 to 660233, EPI_ISL_660256 to 660258, EPI_ISL_660664	KRISP, KZN Research Innovation and Sequencing Platform	KRISP, KZN Research Innovation and Sequencing Platform	Chimukangara B; Deforche K; Giandhari J; Khan S; Lessells R; Mdlalose K; Pillay S; Tegally H; Wilkinson E; York D; de Oliveira T
EPI_ISL_751572, EPI_ISL_751677, EPI_ISL_751707, EPI_ISL_751724, EPI_ISL_751730	KS Health and Environmental Laboratories	Genomics and Discovery, Respiratory Viruses Branch, Division of Viral Diseases, Centers for Disease Control and Prevention	Anna Montmayeur; Anna Uehara; Clinton R. Paden; Haibin Wang; Jing Zhang; Justin Lee; Krista Queen; Mili Sheth; Peter W. Cook; Rachel Marine; Suxiang Tong; Yan Li; Ying Tao
EPI_ISL_424867, EPI_ISL_452114	KS Health and Environmental Laboratories	Pathogen Discovery, Respiratory Viruses Branch, Division of Viral Diseases, Centers for Disease Control and Prevention	Alison S. Laufer Halpin; Anna Montmayeur; Anna Uehara; Christopher A. Elkins; Clinton R. Paden; Haibin Wang; Jing Zhang; Krista Queen; Mary S. Keckler; Rachel Marine; Suxiang Tong; Yan Li; Ying Tao; Zachary Weiner
EPI_ISL_1094246, EPI_ISL_1094254, EPI_ISL_1094270, EPI_ISL_1094313, EPI_ISL_1094349, EPI_ISL_1094639 to 1094647, EPI_ISL_1094649 to 1094650, EPI_ISL_1094653 to 1094655, EPI_ISL_1095210 to 1095214			
see above	KS Health and Environmental Laboratories	Respiratory Viruses Branch, Division of Viral Diseases, Centers for Disease Control and Prevention	Anna Montmayeur; Anna Uehara; Ben L. Rambo-Martin; Clinton R. Paden; Dhwani Batra; Haibin Wang; Jasmine Padilla; Jing Zhang; Justin Lee; Krista Queen; Lori Rowe; Mark Burroughs; Mili Sheth; Peter W. Cook; Rachel Marine; Sarah Nobles; Suxiang Tong; Yan Li; Ying Tao
EPI_ISL_415154	KU Leuven, Clincal and Epidemiological Virology	KU Leuven, Clincal and Epidemiological Virology	Bert Vanmechelen; Joan Marti-Careras; Marc Van Ranst; Piet Maes.; Tony Wawina
EPI_ISL_407976, EPI_ISL_415153, EPI_ISL_415155 to 415159, EPI_ISL_416467 to 416472, EPI_ISL_416475 to 416476, EPI_ISL_417422, EPI_ISL_417424 to 417430, EPI_ISL_418270, EPI_ISL_418792 to 418798, EPI_ISL_418800, EPI_ISL_418805 to 418806, EPI_ISL_418863, EPI_ISL_418981 to 418987, EPI_ISL_418989, EPI_ISL_420314 to 420454			
see above	KU Leuven, Clinical and Epidemiological Virology	KU Leuven, Clinical and Epidemiological Virology	Annabel Rector; Bert Vanmechelen; Elke Wollants; Els Keyaerts; Joan Marti-Carerras; Joan Marti-Carreras; Lies Laenen; Marc Van Ranst; Piet Maes; Tony Wawina; and Piet Maes
EPI_ISL_458156 to 458235, EPI_ISL_462151 to 462275, EPI_ISL_464065 to 464090, EPI_ISL_476941 to 477007, EPI_ISL_522349 to 522350, EPI_ISL_845566 to 845617, EPI_ISL_888683 to 888790, EPI_ISL_890238 to 890351, EPI_ISL_894171 to 894217, EPI_ISL_896447 to 896494, EPI_ISL_897986 to 898032, EPI_ISL_912404 to 912455, EPI_ISL_913748 to 913770, EPI_ISL_913772 to 913773, EPI_ISL_913775 to 913777, EPI_ISL_913779 to 913781, EPI_ISL_913783 to 913784, EPI_ISL_913786 to 913787, EPI_ISL_913789 to 913790, EPI_ISL_913792 to 913794, EPI_ISL_913796 to 913797, EPI_ISL_913799 to 913801, EPI_ISL_913803 to 913804, EPI_ISL_913806 to 913807, EPI_ISL_913809 to 913810, EPI_ISL_913812 to 913814, EPI_ISL_913816 to 913817, EPI_ISL_913819 to 913821, EPI_ISL_913823 to 913824, EPI_ISL_913826 to 913828, EPI_ISL_913830 to 913831, EPI_ISL_913833 to 913835, EPI_ISL_913837 to 913838, EPI_ISL_913840 to 913841, EPI_ISL_913843 to 913845, EPI_ISL_913847 to 913848, EPI_ISL_913850 to 913851, EPI_ISL_913853 to 913855, EPI_ISL_913857 to 913858, EPI_ISL_913860 to 913861, EPI_ISL_913863 to 913865, EPI_ISL_913867 to 913869, EPI_ISL_913871 to 913872, EPI_ISL_913874 to 913876, EPI_ISL_913878, EPI_ISL_935187 to 935358, EPI_ISL_995556 to 995600, EPI_ISL_995602 to 995613, EPI_ISL_995623 to 995650, EPI_ISL_995663 to 995682, EPI_ISL_995692 to 995693, EPI_ISL_1000993, EPI_ISL_1001041, EPI_ISL_1048814 to 1048821, EPI_ISL_1048843 to 1049202, EPI_ISL_1050358 to 1050368, EPI_ISL_1050370 to 1050384, EPI_ISL_1050387 to 1050391, EPI_ISL_1050393 to 1050400, EPI_ISL_1050402 to 1050416, EPI_ISL_1051042, EPI_ISL_1051044, EPI_ISL_1051046 to 1051047, EPI_ISL_1051049, EPI_ISL_1051051, EPI_ISL_1051053, EPI_ISL_1051055, EPI_ISL_1051057, EPI_ISL_1051059, EPI_ISL_1051061, EPI_ISL_1051064, EPI_ISL_1051066, EPI_ISL_1051068, EPI_ISL_1051070, EPI_ISL_1051072, EPI_ISL_1051074, EPI_ISL_1051076, EPI_ISL_1051078 to 1051079, EPI_ISL_1051081, EPI_ISL_1051083, EPI_ISL_1051085 to 1051086, EPI_ISL_1051088, EPI_ISL_1051090, EPI_ISL_1051092, EPI_ISL_1051094, EPI_ISL_1051096, EPI_ISL_1051098, EPI_ISL_1051100, EPI_ISL_1051102, EPI_ISL_1051104, EPI_ISL_1051106, EPI_ISL_1051108 to 1051109, EPI_ISL_1051111, EPI_ISL_1051113, EPI_ISL_1051115, EPI_ISL_1055821 to 1055823, EPI_ISL_1056984 to 1056995, EPI_ISL_1057008 to 1057009, EPI_ISL_1057032 to 1057034, EPI_ISL_1057043, EPI_ISL_1057055, EPI_ISL_1057057 to 1057060, EPI_ISL_1057066, EPI_ISL_1057068, EPI_ISL_1057072 to 1057083, EPI_ISL_1057096, EPI_ISL_1064405 to 1064407, EPI_ISL_1064409 to 1064441, EPI_ISL_1064444 to 1064448, EPI_ISL_1064450 to 1064460, EPI_ISL_1064462 to 1064476, EPI_ISL_1064479 to 1064490, EPI_ISL_1091292 to 1091323, EPI_ISL_1091325 to 1091400, EPI_ISL_1093183 to 1093396, EPI_ISL_1094192 to 1094195, EPI_ISL_1103612, EPI_ISL_1103614, EPI_ISL_1103616 to 1103617, EPI_ISL_1103619, EPI_ISL_1103621, EPI_ISL_1103623 to 1103624, EPI_ISL_1103626, EPI_ISL_1111057 to 1111058, EPI_ISL_1120963 to 1120966, EPI_ISL_1312067, EPI_ISL_1312070 to 1312074, EPI_ISL_1312076 to 1312120, EPI_ISL_1312123 to 1312300			
see above	KU Leuven, Rega Institute, Clinical and Epidemiological Virology	KU Leuven, Rega Institute, Clinical and Epidemiological Virology	Bert Vanmechelen; Joan Marti-Carerras; Piet Maes; Tony Wawina-Bokalanga
EPI_ISL_539300 to 539325, EPI_ISL_549006 to 549023	KWR Watercycle Research Institute	Erasmus Medical Center	Bas B. Oude Munnink; Claudia M. E. Schapendonk; David Nieuwenhuijse; Frank M. Aarestrup; Gertjan Medema; Goffe Elsinga; Leo Heijnen; Lu Lu; Marion P.G. Koopmans; Matthijs Kon; Miranda de Graaf; Ray Izquierdo-Lara; Samantha Lycett
EPI_ISL_751570, EPI_ISL_751594, EPI_ISL_751596, EPI_ISL_751648, EPI_ISL_751670, EPI_ISL_751754, EPI_ISL_751794, EPI_ISL_1167934 to 1167935, EPI_ISL_1225992 to 1226002			

see above	KY State Public Health Lab	Genomics and Discovery, Respiratory Viruses Branch, Division of Viral Diseases, Centers for Disease Control and Prevention	; Anna Montmayeur; Anna Uehara; Ben L. Rambo-Martin; Brian Lynch; Clinton R. Paden; Dhwani Batra; Haibin Wang; Jasmine Padilla; Jing Zhang; Justin Lee; Katie Dillon; Kristen Queen; Kristen Knipe; Kristine Lacek; Lori Rowe; Mark Burroughs; Matthew Schmerer; Mili Sheth; Peter W. Cook; Rachel Marine; Sam Shepard; Sarah Nobles; Shoshona Le; Suxiang Tong; Yan Li; Ying Tao
EPI_ISL_1094298, EPI_ISL_1094345, EPI_ISL_1094945 to 1094961	KY State Public Health Lab	Respiratory Viruses Branch, Division of Viral Diseases, Centers for Disease Control and Prevention	Anna Montmayeur; Anna Uehara; Ben L. Rambo-Martin; Clinton R. Paden; Dhwani Batra; Haibin Wang; Jasmine Padilla; Jing Zhang; Justin Lee; Krista Queen; Lori Rowe; Mark Burroughs; Mili Sheth; Peter W. Cook; Rachel Marine; Sarah Nobles; Suxiang Tong; Yan Li; Ying Tao
EPI_ISL_495411 to 495457	Kafkas University, Faculty of Medicine, Department of Medical Microbiology	Kafkas University, Faculty of Medicine, Department of Medical Microbiology	Didem Ozgur; E. Ediz Tutuncu; Murat Karamese
EPI_ISL_1114385	Kaiser Permanente	Seattle Flu Study	Amanda Adler; Barry R. Lutz; Benjamin Pelle; Brian Hiatt; Caitlin R. Wolf; Chris D. Frazar; Deborah A. Nickerson; Elisabeth Brandstetter; Erica Ryke; Geoff Melly; Helen Y. Chu; Janet A. Englund; Jay Shendure; Jover Lee; Kairsten Fay; Kirsten Lacombe; Lea M. Starita; Mark J. Rieder; Matthew Richardson; Matthew Thompson; Melissa Truong; Michael Boeckh; Michael Famulare; Misja Ilcisin; Peter D. Han; Philip Dykema; Romesh Gautom; Scott Lindquist; Thomas R. Sibley; Trevor Bedford
EPI_ISL_745197	Kakatiya Medical College	CSIR-Centre for Cellular and Molecular Biology	Archana Bharadwaj Siva; B Himasri; Blessy B John; Divya Tej Sowpati; Dr.P.Shashikala Reddy; Dr.S.Pavani; Dr.Satyaprasad; Dr.V.Sudha Rani; Karthik Bharadwaj Tallapaka; Lamuk Zaveri; M Soujanya Reddy; Namami Gaur; Nikhil Hajirnis; Onkar Kulkarni; Payel Mukherjee; Pratheusa Maccha; Priya Singh; Purushotham Vodnala; Rakesh K Mishra; Sakshi Shambhavi; Shagufta Khan; Sofia Banu; Tulasi Nagabandi; Viswagithe S L
EPI_ISL_475137 to 475138, EPI_ISL_510825 to 510826, EPI_ISL_510832 to 510833, EPI_ISL_510836 to 510837, EPI_ISL_510859, EPI_ISL_510864 to 510865, EPI_ISL_623091, EPI_ISL_648164, EPI_ISL_676534, EPI_ISL_710605			
see above	Kalmar klinisk mikrobiologi	The Public Health Agency of Sweden	Anna Risberg; Anna-Malin Linde; Department of Microbiology; Karin Tegmark-Wisell; Maria Lind Karlberg; Mattias Haukland; Mia Brytting; Olov Svartstrom; Oskar Karlsson Lindsjo; Petra Edquist; Reza Advani; Sandra Broddesson; Shamam Muradrasoli; The Public Health Agency of Sweden
EPI_ISL_1298487	Kalush CRH	The Institute of Molecular Biology and Genetics of NASU	M.Tukalo et al.
EPI_ISL_872571, EPI_ISL_1039012 to 1039056	Kamchatka Regional Children's Infectious Diseases Hospital	WHO National Influenza Centre Russian Federation	Andrey Komissarov; Anna Ivanova; Artem Fadeev; Daria Danilenko; Dmitry Bazhenov; Dmitry Lioznov; Elena Nabieva; Georgii Bazykin; Ksenia Safina; Kseniya Komissarova; Maria Pisareva; Maria Timofeeva; Mikhail Bakaev; Tamila Musaeva; Veronika Eder
EPI_ISL_687043 to 687078, EPI_ISL_690818 to 690836, EPI_ISL_898523 to 898550, EPI_ISL_1127143, EPI_ISL_1127999 to 1128017	Kanagawa Prefectural Institute of Public Health	Pathogen Genomics Center, National Institute of Infectious Diseases	Kentaro Itokawa; Makoto Kuroda; Masanori Hashino; Rina Tanaka; Tsuyoshi Sekizuka
EPI_ISL_1287778 to 1287782	Kansas Health and Environmental Lab	Cytocheck Laboratory	Ben Olsen; Jonathan Barnell; Mike Grose; and Phil Adam
EPI_ISL_875675 to 875684, EPI_ISL_883037 to 883065, EPI_ISL_884223 to 884248, EPI_ISL_914866 to 914874, EPI_ISL_966334 to 966337, EPI_ISL_966339 to 966354, EPI_ISL_978553, EPI_ISL_983357 to 983358, EPI_ISL_983360 to 983367, EPI_ISL_983494 to 983499, EPI_ISL_1000971, EPI_ISL_1004218 to 1004232, EPI_ISL_1048830 to 1048842, EPI_ISL_1049843 to 1049858, EPI_ISL_1054886 to 1054891, EPI_ISL_1058067 to 1058075, EPI_ISL_1064711 to 1064735, EPI_ISL_1067717 to 1067725, EPI_ISL_1073813 to 1073824, EPI_ISL_1079890 to 1079891, EPI_ISL_1079893 to 1079894, EPI_ISL_1092009, EPI_ISL_1110553 to 1110579, EPI_ISL_1113042 to 1113055, EPI_ISL_1118885 to 1118889, EPI_ISL_1120108 to 1120119, EPI_ISL_1120121 to 1120132, EPI_ISL_1121338 to 1121354, EPI_ISL_1133100 to 1133102, EPI_ISL_1137048 to 1137080, EPI_ISL_1159682 to 1159696, EPI_ISL_1163794, EPI_ISL_1167547 to 1167567, EPI_ISL_1167664 to 1167665, EPI_ISL_1229369 to 1229387, EPI_ISL_1231192 to 1231206, EPI_ISL_1261124 to 1261134, EPI_ISL_1263324 to 1263330, EPI_ISL_1272075, EPI_ISL_1298489 to 1298490, EPI_ISL_1299222 to 1299226, EPI_ISL_1314258 to 1314261			
see above	Kansas Health and Environmental Lab	Kansas Health and Environmental Lab	Ben Olsen; Carissa Robertson; Jonathan Barnell; Mike Grose; Paige Drury; and Phil Adam
EPI_ISL_812251 to 812254	Kantonsarztamt Bern	Institute of Medical Virology, University of Zurich	Alexandra Trkola; Annette Audigé; Cyril Shah; Guido Bloemberg; Jon Huder; Jürg Böni; Kevin Steiner; Maria Grünberg; Maryam Zaheri; Michael Huber; Riccarda Capaul; Stefan Schmutz; Verena Kufner
EPI_ISL_896091, EPI_ISL_896101 to 896102, EPI_ISL_896116	Kantonsspital Aarau, Institut für Labormedizin	University Hospital Basel, Clinical Bacteriology	Adrian Egli; Alfredo Mari; Hans Hirsch; Helena MB Seth-Smith; Julia Bielicki; Karoline Leuzinger; Madlen Stange; Manuel Battegay; Michael Oberle; Tim Roloff
EPI_ISL_1039788, EPI_ISL_1039805 to 1039808, EPI_ISL_1082343, EPI_ISL_1082346 to 1082347, EPI_ISL_1082382 to 1082383	Kantonsspital Baden AG	Institute of Medical Virology, University of Zurich	Alexandra Trkola; Annette Audigé; Cyril Shah; Gabriela Ziltener; Guido Bloemberg; Jon Huder; Jürg Böni; Kevin Steiner; Maria Grünberg; Maryam Zaheri; Michael Huber; Riccarda Capaul; Stefan Schmutz; Verena Kufner
EPI_ISL_1039809, EPI_ISL_1082331, EPI_ISL_1082355, EPI_ISL_1082380	Kantonsspital Frauenfeld	Institute of Medical Virology, University of Zurich	Alexandra Trkola; Annette Audigé; Cyril Shah; Gabriela Ziltener; Guido Bloemberg; Jon Huder; Jürg Böni; Kevin Steiner; Maria Grünberg; Maryam Zaheri; Michael Huber; Riccarda Capaul; Stefan Schmutz; Verena Kufner
EPI_ISL_1039786, EPI_ISL_1082350, EPI_ISL_1082362, EPI_ISL_1082385	Kantonsspital Münsterlingen	Institute of Medical Virology, University of Zurich	Alexandra Trkola; Annette Audigé; Cyril Shah; Gabriela Ziltener; Guido Bloemberg; Jon Huder; Jürg Böni; Kevin Steiner; Maria Grünberg; Maryam Zaheri; Michael Huber; Riccarda Capaul; Stefan Schmutz; Verena Kufner
EPI_ISL_812255	Kantonsärztlicher Dienst Aargau	Institute of Medical Virology, University of Zurich	Alexandra Trkola; Annette Audigé; Cyril Shah; Guido Bloemberg; Jon Huder; Jürg Böni; Kevin Steiner; Maria Grünberg; Maryam Zaheri; Michael Huber; Riccarda Capaul; Stefan Schmutz; Verena Kufner
EPI_ISL_444487 to 444492	Karolinska Universitetslaboratoriet	CTMR, Karolinska Institutet, Stockholm, Sweden	Caroline Bjurnemark; Fredrik Boulund; Jing Wang; Jingkai Ji; Lars Engstrand; Linnéa Påvénius; Marica Hamsten; Shuiqin Li; Stefanie Prast-Nielsen; Vivien Lan Yang Swartz; Yinghua Zha; Yue Hu
EPI_ISL_454447 to 454493, EPI_ISL_454868 to 454902, EPI_ISL_455846 to 455901, EPI_ISL_469075, EPI_ISL_469077 to 469079, EPI_ISL_475086 to 475091, EPI_ISL_475095, EPI_ISL_475136, EPI_ISL_475141 to 475142, EPI_ISL_475149 to 475150, EPI_ISL_475544 to 475547, EPI_ISL_475551 to 475552, EPI_ISL_475558 to 475561, EPI_ISL_510834 to 510835, EPI_ISL_510838 to 510840, EPI_ISL_510852, EPI_ISL_510860 to 510863, EPI_ISL_560974, EPI_ISL_615086 to 615089, EPI_ISL_676533			
see above	Karolinska Universitetslaboratoriet	The Public Health Agency of Sweden	Anna Risberg; Anna-Malin Linde; Department of Microbiology; Karin Tegmark-Wisell; Maria Lind Karlberg; Mattias Haukland; Mia Brytting; Olov Svartstrom; Oskar Karlsson Lindsjo; Petra Edquist; Reza Advani; Sandra Broddesson; Shamam Muradrasoli; Shaman Muradrasoli; The Public Health Agency of Sweden
EPI_ISL_1313181 to 1313279, EPI_ISL_1313282 to 1313541	Karolinska University Hospital	Karolinska University Hospital	Annelie Bjerkner; Isak Sylvin; Jan Albert; Karolina Ininbergs; Lina Guerra Blomqvist; Lynda Eneh; Martin Ekman; Martina Wahlund; Robert Dyrdak; Sandra Broddesson; Tanja Normark; Tobias Allander; Valtteri Wirta; Zhibing Yun
EPI_ISL_534259, EPI_ISL_548257 to 548258, EPI_ISL_560989 to 560990	Karolinska universitetslaboratoriet	The Public Health Agency of Sweden	Anna Risberg; Anna-Malin Linde; Karin Tegmark-Wisell; Maria Lind Karlberg; Mattias Haukland; Mia Brytting; Olov Svartstrom; Oskar Karlsson Lindsjo; Petra Edquist; Reza Advani; Sandra Broddesson
EPI_ISL_534234 to 534236, EPI_ISL_560982 to 560985	Karolinska universitetslaboratoriet SOLNA	The Public Health Agency of Sweden	Anna Risberg; Anna-Malin Linde; Karin Tegmark-Wisell; Maria Lind Karlberg; Mattias Haukland; Mia Brytting; Olov Svartstrom; Oskar Karlsson Lindsjo; Petra Edquist; Reza Advani; Sandra Broddesson
EPI_ISL_833498	Kartika Pulomas Hospital, Jakarta, Indonesia	Biosafety Level-3 Laboratory, Indonesian Institute of Sciences (LIPI)	Ade Andriani; Ahmad Fathoni; Andri Wardiana; Anggia Prasetyoputri; Anik Budhi Dharmayanthi; Isa Nuryana; Pinka Alisa Diena Shafa; Ratih Asmana Ningrum; Syam Budi Iryanto; Winda Tasia
EPI_ISL_1073037, EPI_ISL_1075644	Kasane Primary Hospital Laboratory	Botswana Harvard HIV Reference Laboratory	Boitumelo Zuze; Botshelo Radibe; David Lawrence; Dorcas Maruapula; Joseph; Joseph Makhema; Legodile Kooepile; Makhema; Mosepele Mosepele; Roger Shapiro; Shahin Lockman; Sikhulile Moyo; Simani Gaseitsiwe; Wonderful T. Choga
EPI_ISL_1113704 to 1113708, EPI_ISL_1113754, EPI_ISL_1113759 to 1113763, EPI_ISL_1113843, EPI_ISL_1114066, EPI_ISL_1121041 to 1121045, EPI_ISL_1121047 to 1121048, EPI_ISL_1121050 to 1121056, EPI_ISL_1121221, EPI_ISL_1121229 to 1121232, EPI_ISL_1121304, EPI_ISL_1121331 to 1121335, EPI_ISL_1121337, EPI_ISL_1121355, EPI_ISL_1233424 to 1233425, EPI_ISL_1233436, EPI_ISL_1233438, EPI_ISL_1233483, EPI_ISL_1233868 to 1233872			
see above	Kashi Clinical Laboratory	Kashi Clinical Laboratory	Jenefer DeKoning; Zahra Kashi
EPI_ISL_1239550, EPI_ISL_1239559, EPI_ISL_1239621 to 1239623, EPI_ISL_1239765 to 1239768, EPI_ISL_1273841	Katedra Medycyny Sdowej PUM w Szczecinie	1. National Institute of Public Health - National Institute of Hygiene; 2. Eurofins Genomics Europe Sequencing GmbH	ECDC COVID-19 WGS support team; Eurofins Genomics Europe Sequencing Team; Gierczyki Rafa; Sadkowska-Todys Magorzata; Wokowicz Tomasz; Zacharczuk Katarzyna
EPI_ISL_1145218 to 1145219, EPI_ISL_1149270, EPI_ISL_1149281, EPI_ISL_1149296, EPI_ISL_1150458, EPI_ISL_1153480, EPI_ISL_1153487, EPI_ISL_1153499 to 1153500, EPI_ISL_1217522, EPI_ISL_1217530, EPI_ISL_1217559, EPI_ISL_1281972			
see above	Kath. St.-Johannes-Gesellschaft Dortmund gGmbH	Robert Koch Institute	
EPI_ISL_754062 to 754067	Kathmandu University	Nepal Health Research Council	Meghnath Dhimal; Pradip Gyanwali
EPI_ISL_1150459	Katholisches Klinikum Lünen/Werne GmbH :	Robert Koch Institute	

	St.-Marien-Hospital Lünen		
EPI_ISL_442044	Kawsar Human Genetic Research Center	Kawsar Human Genetic Research Center	Fatemeh Hosseini; Maryam Abbasalipour Bashash; Mohammad Ali Khosravi; Sirous Zeinali; Solmaz Sabeghi; Yeganeh Haghdoost; Yeganeh Keshvar
EPI_ISL_469287 to 469296	Keio University Hospital	Keio University Hospital	Kenjiro Kosaki
EPI_ISL_437601, EPI_ISL_438947 to 438949, EPI_ISL_438951 to 438956, EPI_ISL_438958 to 438959, EPI_ISL_438961 to 438972, EPI_ISL_803879, EPI_ISL_860117 to 860181, EPI_ISL_915358 to 915420, EPI_ISL_1137610			
see above	Keio University School of Medicine	Keio University School of Medicine	; Haruhiko Siomi; Hirotsugu Ishizu; Kenjiro Kosaki; Kodai Abe; Toshiki Takenouchi; Yuka Iwasaki
EPI_ISL_512811 to 512821	Kenema Government Hospital, Ministry of Health and Sanitation	Kenema Government Hospital, Ministry of Health and Sanitation	Andersen, K.; Garry, R.; Goba, A.; Grant, D.; Happi, C.; Jalloh, S.; Mehta, S.; Momoh, M.; Olawoye, I.; Oluniyi, P.; Park, D.; Sandi, J.; Siddle, K.; Tomkins-Tinch, C.
EPI_ISL_1040760	Kensington CDC	NHLS/UCT	Arash Iranzadeh; Bruna Galvao; Carolyn Williamson; Deelan Doolabh; Diana Hardie; Innocent Mudau; Kruger Marais; Lynn Tyers; Marvin Hsiao; Stephen Korsman
EPI_ISL_640092 to 640093	Kensington CDC wc KSC	NHLS/UCT	Arash Iranzadeh; Bruna Galvao; Carolyn Williamson; Deelan Doolabh; Diana Hardie; Innocent Mudau; Kruger Marais; Lynn Tyers; Marvin Hsiao; Stephen Korsman
EPI_ISL_965815 to 965825, EPI_ISL_965827 to 965835, EPI_ISL_966315 to 966332, EPI_ISL_966355 to 966356, EPI_ISL_966358 to 966374, EPI_ISL_982506 to 982525, EPI_ISL_982762 to 982780, EPI_ISL_982842 to 982844, EPI_ISL_982853 to 982856, EPI_ISL_983073 to 983074, EPI_ISL_983082, EPI_ISL_983085, EPI_ISL_983092 to 983095, EPI_ISL_1121994 to 1122013, EPI_ISL_1171858 to 1171870, EPI_ISL_1184115 to 1184134, EPI_ISL_1302682 to 1302684, EPI_ISL_1302687 to 1302693, EPI_ISL_1302698, EPI_ISL_1302701 to 1302707, EPI_ISL_1312346 to 1312353, EPI_ISL_1314170 to 1314177, EPI_ISL_1314190 to 1314197			
see above	Kentucky State Public Health Lab	Kentucky State Public Health Lab	Joshua Tobias; Karim George; Matthew Johnson; Rachel Zinner; Rhonda Lucas; Stephanie Lunn; Vaneet Arora; William Grooms
EPI_ISL_456405	Kerman University of Medical Sciences	Kerman University of Medical Sciences, Afzalipour School of Medicine	Abbas Aghaei Afshar; Davood Kalantar-Neyestanaki; Hamidreza R. Mollaei
EPI_ISL_813655 to 813656	Kettering General Hospital	COVID-19 Genomics UK (COG-UK) Consortium	Christopher Moore; Fei Sang; Johnny Debebe; Jonathan Ball; Joseph Chappell; Matthew Carlisle; Matthew Loose; Nadine Holmes; Patrick McClure; Theocharis Tsoleridis; Victoria Wright
EPI_ISL_696511	Keurhoek Clinic wc KTT & NHLS/UCT	KRISP, KZN Research Innovation and Sequencing Platform	Arash Iranzadeh; Bruna Galvao; Carolyn Williamson; Deelan Doolabh; Diana Hardie; Emanuel James San; Houriiyah Tegally; Innocent Mudau; Jennifer Giandhari; Kruger Marais; Lynn Tyers; Marvin Hsiao; Stephen Korsman; Sureshnee Pillay; Tulio de Oliveira
EPI_ISL_411957	Key Laboratory of Human Diseases, Comparative Medicine, Institute of Laboratory Animal Science	Key Laboratory of Human Diseases, Comparative Medicine, Institute of Laboratory Animal Science	Conghui, W.; Feifei, Q.; Fengdi, L.; Guanpeng, W.; Hong, G.; J. and Chuan, Q.; Jiangning, L.; Jing, X.; Lan, C.; Li, G.; Lili, R.; Linlin, B.; Mingya, L.; Pin, Y.; Qi; Qi, L.; Qiang, W.; Shunyi, W.; Shuran, G.; Wei, D.; Xinming, W.; Yajin, Q.; Yan, X.; Yanfeng, X.; Ying, W.; Zhiqi, S.
EPI_ISL_414510	Key Laboratory of Medical Molecular Virology (MOE/NHC/CAMS), School of Basic Medicine, Shanghai Medical College, Fudan University	Key Laboratory of Medical Molecular Virology (MOE/NHC/CAMS), School of Basic Medicine, Shanghai Medical College, Fudan University	Cai, X.; Chen, H.; Fang, F.; Feng, F.; Fu, C.; Gu, C.; Han, W.; Li, C.; Qu, D.; Song, W.; Sun, Z.; Teng, Z.; Wang, Y.; Xie; Xu, W.; Y. and Yuan, Z.; Ye, R.; Yi, Z.; Zhang, R.; Zhang, X.; Zhou, Y.; Zhu, Y.
EPI_ISL_700423, EPI_ISL_700464, EPI_ISL_700468, EPI_ISL_700485, EPI_ISL_700505, EPI_ISL_700540	Khayelethu Clinic wc KLC	NHLS/UCT	Arash Iranzadeh; Bruna Galvao; Carolyn Williamson; Deelan Doolabh; Diana Hardie; Houriiyah Tegally; Innocent Mudau; Kruger Marais; Lynn Tyers; Marvin Hsiao; Stephen Korsman
EPI_ISL_696484, EPI_ISL_696505	Khayelethu Clinic wc KLC & NHLS/UCT	KRISP, KZN Research Innovation and Sequencing Platform	Arash Iranzadeh; Bruna Galvao; Carolyn Williamson; Deelan Doolabh; Diana Hardie; Emanuel James San; Houriiyah Tegally; Innocent Mudau; Jennifer Giandhari; Kruger Marais; Lynn Tyers; Marvin Hsiao; Stephen Korsman; Sureshnee Pillay; Tulio de Oliveira
EPI_ISL_640083	Khayelitsha (Site B) CHC wc KHC	NHLS/UCT	Arash Iranzadeh; Bruna Galvao; Carolyn Williamson; Deelan Doolabh; Diana Hardie; Innocent Mudau; Kruger Marais; Lynn Tyers; Marvin Hsiao; Stephen Korsman
EPI_ISL_745111, EPI_ISL_745127, EPI_ISL_745163	Khayelitsha (Site B) CHC wc KHC	National Health Laboratory Service (NHLS), Tygerberg	Bronwyn Kleinhans; Eduan Wilkindon; Gert van Zyl; Houriiyah Tegally; Kayla Delaney; Susan Engelbrecht; Tulio de Oliveira; Wolfgang Preiser
EPI_ISL_745114 to 745116, EPI_ISL_745120, EPI_ISL_745122 to 745123, EPI_ISL_745145, EPI_ISL_745166	Khayelitsha Hospital wc KHA	National Health Laboratory Service (NHLS), Tygerberg	Bronwyn Kleinhans; Eduan Wilkindon; Gert van Zyl; Houriiyah Tegally; Kayla Delaney; Susan Engelbrecht; Tulio de Oliveira; Wolfgang Preiser
EPI_ISL_803906 to 803907, EPI_ISL_803910, EPI_ISL_803914 to 803915, EPI_ISL_825146, EPI_ISL_825150 to 825151, EPI_ISL_845881	Kidwai Memorial Institute of Oncology	Department of Neurovirology, National Institute of Mental Health and Neurosciences (NIMHANS)	Anita S Desai; Chitra Pattabiraman; Darshan Sreenivas; Nakka Vijay Kiran Reddy; Pramada Prasad; Risha Rasheed; V Ravi
EPI_ISL_1239225 to 1239266, EPI_ISL_1239458, EPI_ISL_1239460 to 1239461, EPI_ISL_1239463, EPI_ISL_1239466 to 1239468, EPI_ISL_1239471, EPI_ISL_1239474 to 1239478, EPI_ISL_1239480 to 1239481, EPI_ISL_1239483, EPI_ISL_1239485 to 1239486, EPI_ISL_1239488 to 1239489, EPI_ISL_1239508, EPI_ISL_1239512, EPI_ISL_1239518 to 1239520, EPI_ISL_1239522, EPI_ISL_1239527, EPI_ISL_1239529, EPI_ISL_1239531			
see above	Kimberley Hospital, National Health Laboratory Services, Northern Cape, South Africa	National Institute for Communicable Diseases of the National Health Laboratory Service	Amoako DG; Bhiman JN; Ismail A; Mahlangu B; Mohale T; Ntuli N; Scheepers C
EPI_ISL_489996 to 490012	King Fahad Medical City	King Fahad Medical City	Alghoraibi, M.; Alosaimi, B.; Enani, M.; Naeem, A.
EPI_ISL_1231306 to 1231310	King George's Medical University,Lucknow	Indian Council of Medical Research-National Institute of Virology, Microbial Containment Complex	Amita Jain; Pragya D. Yadav
EPI_ISL_516940 to 516986	King Georges Medical University	CSIR-National Botanical Research Institute	Amita Jain; Babita Singh; Danish Nasar Khan; Hricha Mishra; Kishan Sahu; MLB Bhatt; Mehar H.Asif; Om Prakash; Priti Prasad; SK Barik; Samir V. Sawant; Shantanu Prakash; Sumit Kr. Bag; Suruchi Shukla
EPI_ISL_458030 to 458044, EPI_ISL_471583 to 471584	King Institute of Preventive Medicine & Research	CSIR-Centre for Cellular and Molecular Biology	Archana Bharadwaj Siva; Dhiviya Vedagiri; Divya Gupta; Divya Tej Sowpati; G. Dhinakar Raj; G. Ravikumar; K Thangaraj; K.Kaveri; Karthik Bharadwaj Tallapaka; Krishnan Harinivas Harshan; Lamuk Zaveri; M. Sekar; Namami Gaur; P. Azhahianambi; P.Padmapriya; Payel Mukherjee; Priya Singh; Purushotham Vodnala; R. P. Aravindh Babu; R.Kiruba; Rakesh K Mishra; S.Magesh; S.Sivasubramanian; S.Vennila; Sakshi Shambhavi; Santosh Kumar Kuncha; Shagufta Khan; Sofia Banu; Tulasi Nagabandi; Vishal Sah
EPI_ISL_1231384 to 1231398, EPI_ISL_1231408 to 1231418	King Institute of Preventive Medicine & Research	Indian Council of Medical Research-National Institute of Virology, Microbial Containment Complex	K. Kaveri; Pragya D. Yadav
EPI_ISL_483542 to 483565, EPI_ISL_483638 to 483640	Kingdom of Bahrein Ministry of Health	Erasmus Medical Center	Amjad Ghanem Mohamed; Anne van der Linden; Bas Oude Munnink; Claudia Schapendonk; David Nieuwenhuijse; Ebrahim Shehad; Fatema; Hashmeya Al Wasti; Irina Chestakova; Marion Koopmans; Mark Pronk; Pascal Lexmond; Reina Sikkema; Richard Molenkamp; Stefan van Nieuwkoop; Theo Bestebroer; on behalf of the Dutch national COVID-19 response team.
EPI_ISL_459866 to 459869, EPI_ISL_459871 to 459875, EPI_ISL_459877 to 459892, EPI_ISL_529029 to 529030	Kingston Health Sciences Center	Queen's Genomics Lab at Ongwanada (Q-GLO)	Ayub M; Colautti R; Evans GA; Guan H; Huang D; Hudson ML; Perez-Patrigeon S; Rustom N; Sheth P; Sjaarda CP; Soares CN; Wong H
EPI_ISL_538321 to 538386	Kingston Health Sciences Centre / Queen's University	Ontario Institute for Cancer Research	Bernard Lam; Calvin Sjaarda; Ilinca Lungu; Jared T. Simpson; Katya Douchant; Lawrence E Heisler; Michael Laszloffy; Paul Krzyzanowski; Prameet M. Sheth; Richard de Borja; Robert Colautti
EPI_ISL_717723 to 717764, EPI_ISL_718105, EPI_ISL_1015363 to 1015367, EPI_ISL_1015530	Kingston Health Sciences Centre and Queen's University	Ontario Institute for Cancer Research	Bernard Lam; Calvin Sjaarda; Felicia Vincelli; Ilinca Lungu; Jared T. Simpson; Jeremy Johns; Katya Douchant; Lawrence E. Heisler; Michael Laszloffy; Paul Krzyzanowski; Philip Zuzarte; Prameet M. Sheth; Richard de Borja; Robert Colautti
EPI_ISL_479990	Kitakyushu City Institute of Health and Environmental Sciences	Pathogen Genomics Center, National Institute of Infectious Diseases	Asuka Kikuchi Kentaro Itokawa; Hajime Kamiya; Katsuya Obata; Makoto Kuroda; Masanori Hashino; Motoi Suzuki; Rina Tanaka; Tsuyoshi Sekizuka
EPI_ISL_862881	Kitakyushu Municipal Medical Center	Fujita health University, School of Medicine, Department of Microbiology	Masahiro suzuki; Yohei Doi
EPI_ISL_1252692, EPI_ISL_1252696, EPI_ISL_1252703, EPI_ISL_1252726 to 1252727, EPI_ISL_1252733 to 1252734, EPI_ISL_1252739, EPI_ISL_1252744, EPI_ISL_1252809, EPI_ISL_1252815 to 1252836			

see above	Klaipeda University Hospital	Vilnius University Hospital Santaros Klinikos, Center of Laboratory Medicine	Daniel Naumovas; Dovile Ezerskyte; Gytis Dudas; Ingrida Olendraite; Laimonas Griskevicius; Ligita Raugaite; Mindaugas Stoskus; Monika Katenaite; Rimvydas Norvilas
EPI_ISL_518820, EPI_ISL_833388 to 833391, EPI_ISL_833495, EPI_ISL_833497, EPI_ISL_833500, EPI_ISL_833508	Klinik Apotek Dein, Jakarta, Indonesia	Biosafety Level-3 Laboratory, Indonesian Institute of Sciences (LIPI)	Ade Andriani; Ahmad Fathoni; Akhirta Atikana; Alfi Taufik Fathurahman; Andri Wardiana; Anggia Prasetyoputri; Anik Budhi Dharmayanthi; Ario Betha Juanssilfero; Asep M Ridwanuloh; Asrul M. Fuad; Dadang Supriatna; Dian Fitria Agustiyanti; Fahrurrozy; Hariyatun; Herman Irawan; Isa Nuryana; Pamella Apriliana; Popi Hadi Wisnuwardhani; Puspita Lisdiyanti; Ratih Asmana Ningrum; Rifki Sadikin; Saiful anwar; Sri Swasthikawati; Syam Budi Iryanto; Wien Kusharyoto
EPI_ISL_1020203, EPI_ISL_1117457	Klinik Apotik Dien, Jakarta	Biosafety Level-3 Laboratory, Indonesian Institute of Sciences (LIPI)	Ade Andriani; Anik Budhi Dharmayanthi; Asep M. Ridwanuloh; Eko Wahyu Putro; Eko Wahyu Putro; Gita Syahputra; Hariyatun; Inswasti Cahyani; Matthew W. Loose; Mohammad Ilyas; Susanti; Syam Budi Iryanto; Wien Kusharyoto
EPI_ISL_1284204	Klinik Ciputra Medical Center	Eijkman Institute for Molecular Biology, Ministry of Research and Technology/National Agency for Research and Innovation	Amin Soebandrio; Edison Johar; Frilasita A Yudhaputri; Hidayat Trimarsanto; Iskandar Adnan; Khin Saw Myint; Lydia V. Panggalo; Safarina G Malik; Sukma Oktavianthi; Willy Agustine
EPI_ISL_413021	Klinik Hirslanden Zurich	Institute of Medical Virology, University of Zurich	Alexandra Trkola; Andrea Zbinden; Christian Ruef; Fiona Steiner; Gabriela Ziltener; Jon Huder; Jürg Böni; Maryam Zaheri; Michael Huber; Patrick Redli; Riccarda Capaul; Stefan Schmutz; Verena Kufner
EPI_ISL_524479	Klinik Hirslanden Zürich	Institute of Medical Virology, University of Zurich	Alexandra Trkola; Andrea Zbinden; Fiona Steiner; Gabriela Ziltener; Jon Huder; Jürg Böni; Maryam Zaheri; Michael Huber; Patrick Redli; Riccarda Capaul; Stefan Schmutz; Verena Kufner
EPI_ISL_1181337 to 1181340	Klinikum Bielefeld Mitte	Bielefeld University	Alexander Sczyrba; David Brandt; Jörn Kalinowski; Levin-Joe Klages; Marina Simunovic; Markus Haak; Svenja Vinke; Tobias Busche
EPI_ISL_1217964 to 1217971, EPI_ISL_1217973 to 1218006, EPI_ISL_1218008 to 1218015, EPI_ISL_1218018 to 1218030, EPI_ISL_1218032 to 1218037, EPI_ISL_1218039, EPI_ISL_1218041 to 1218043, EPI_ISL_1218045 to 1218047, EPI_ISL_1218049 to 1218065, EPI_ISL_1218068 to 1218069, EPI_ISL_1218071 to 1218085, EPI_ISL_1218087 to 1218105, EPI_ISL_1218188 to 1218200, EPI_ISL_1282754 to 1282764, EPI_ISL_1285166 to 1285176			
see above	Klinikum Ernst von Bergmann gemeinnützige GmbH - stationärer Bereich	Robert Koch Institute	
EPI_ISL_1156471, EPI_ISL_1156502, EPI_ISL_1282146	Klinikum Fulda gAG	Robert Koch Institute	
EPI_ISL_1286893, EPI_ISL_1286895, EPI_ISL_1286897, EPI_ISL_1286900, EPI_ISL_1286902, EPI_ISL_1286904	Klinikum Stuttgart	Robert Koch Institute	
EPI_ISL_475910 to 475915, EPI_ISL_934652 to 934654, EPI_ISL_1007993 to 1008009, EPI_ISL_1008292 to 1008298, EPI_ISL_1117777, EPI_ISL_1117788 to 1117789, EPI_ISL_1117794, EPI_ISL_1117834 to 1117835, EPI_ISL_1117840 to 1117845, EPI_ISL_1117894, EPI_ISL_1117900 to 1117901, EPI_ISL_1117904, EPI_ISL_1117909, EPI_ISL_1117921, EPI_ISL_1118050 to 1118061, EPI_ISL_1118063, EPI_ISL_1180832 to 1180838			
see above	Klinikum Wels-Grieskirchen	Bergthaler laboratory, CeMM Research Center for Molecular Medicine of the Austrian Academy of Sciences	Alexander Lercher; Alexandra Popa; Andreas Bergthaler; Anna Schedl; Bekir Erguner; Benedikt Agerer; Christoph Bock; Daniela Schmid; Dorothee von Laer; Elisabeth Puchhammer-Stoeckl; Fabian Amman; Franz Allerberger; Gregor Hörmann; Guenter Weiss; Henrique Colaco; Jakob-Wendelin Genger; Jan Laine; Judith Aberle; Kinga Rigler-Hohenwarter; Lukas Endler; Maelle Le Moing; Manfred Nairz; Mark Smyth; Martin Senekowitsch; Michael Schuster; Peter Hufnagl; Rainer Gattringer; Stephan Aberle; Thomas Penz; Wegene Borena
EPI_ISL_1154022, EPI_ISL_1157057, EPI_ISL_1283690	Klinikum Wolfsburg - Institut für Klinische Chemie Laboratoriums- und Transfusionsmedizin	Robert Koch Institute	
EPI_ISL_1148479, EPI_ISL_1148499, EPI_ISL_1148505 to 1148506, EPI_ISL_1148508, EPI_ISL_1216790, EPI_ISL_1216793, EPI_ISL_1216814, EPI_ISL_1216869, EPI_ISL_1216894, EPI_ISL_1216907, EPI_ISL_1216911, EPI_ISL_1216918 to 1216919, EPI_ISL_1216921, EPI_ISL_1216928, EPI_ISL_1216931, EPI_ISL_1216942, EPI_ISL_1216944, EPI_ISL_1216948, EPI_ISL_1216951, EPI_ISL_1216954, EPI_ISL_1216958, EPI_ISL_1216960, EPI_ISL_1216968, EPI_ISL_1216970, EPI_ISL_1216991, EPI_ISL_1216993, EPI_ISL_1216995, EPI_ISL_1217001, EPI_ISL_1280666 to 1280673, EPI_ISL_1280675 to 1280679, EPI_ISL_1280681 to 1280682, EPI_ISL_1280684 to 1280692, EPI_ISL_1280697 to 1280706, EPI_ISL_1280708 to 1280716, EPI_ISL_1280718 to 1280734, EPI_ISL_1280736, EPI_ISL_1284534			
see above	Klinikum der Stadt Ludwigshafen - Institut für Labordiagnostik Hygiene und Transfusionsmedizin	Robert Koch Institute	
EPI_ISL_1259313, EPI_ISL_1265740 to 1265750	Klinisch Laboratorium ZNA	Klinisch Laboratorium ZNA	Verstrepen et al.
EPI_ISL_475101 to 475104, EPI_ISL_476140 to 476142, EPI_ISL_582808, EPI_ISL_615108, EPI_ISL_654954, EPI_ISL_660427 to 660431, EPI_ISL_661301, EPI_ISL_676492, EPI_ISL_676497 to 676501, EPI_ISL_676518, EPI_ISL_766696, EPI_ISL_766713, EPI_ISL_775546 to 775548, EPI_ISL_789017, EPI_ISL_832005, EPI_ISL_913298 to 913299, EPI_ISL_913315, EPI_ISL_913334 to 913336, EPI_ISL_913370, EPI_ISL_934388 to 934396, EPI_ISL_934407 to 934409, EPI_ISL_1008459 to 1008469, EPI_ISL_1008471 to 1008478, EPI_ISL_1008552 to 1008556, EPI_ISL_1008559 to 1008566, EPI_ISL_1008569, EPI_ISL_1008571 to 1008573, EPI_ISL_1008575, EPI_ISL_1008577, EPI_ISL_1008580 to 1008581, EPI_ISL_1008585, EPI_ISL_1008598 to 1008600, EPI_ISL_1064576 to 1064580, EPI_ISL_1064583 to 1064588, EPI_ISL_1064698 to 1064699, EPI_ISL_1168052 to 1168053, EPI_ISL_1168085, EPI_ISL_1168162 to 1168167, EPI_ISL_1190856 to 1190860, EPI_ISL_1190866 to 1190867, EPI_ISL_1190881 to 1190882, EPI_ISL_1190884, EPI_ISL_1231914, EPI_ISL_1231919, EPI_ISL_1231931 to 1231939, EPI_ISL_1231948 to 1231950, EPI_ISL_1231953 to 1231956, EPI_ISL_1231958 to 1231964, EPI_ISL_1231968, EPI_ISL_1231971 to 1231976, EPI_ISL_1231982, EPI_ISL_1231985, EPI_ISL_1231994 to 1231996, EPI_ISL_1232015 to 1232028			
see above	Klinisk Mikrobiologi	The Public Health Agency of Sweden	Anna Risberg; Anna-Malin Linde; Carlo Berg; Department of Microbiology; Karin Tegmark-Wisell; Maria Lind Karlberg; Mattias Haukland; Mia Brytting; Noura Walai; Olov Svartstrom; Oskar Karlsson Lindsjo; Petra Edquist; Petra Holmstrom; Reza Advani; Samuel Ohman; Sandra Broddesson; Shamam Muradrasoli; Sofia Stamouli; The Public Health Agency of Sweden
EPI_ISL_654892 to 654899, EPI_ISL_654942, EPI_ISL_654944 to 654951, EPI_ISL_660416 to 660421, EPI_ISL_661274 to 661278, EPI_ISL_661280 to 661281, EPI_ISL_661288 to 661289, EPI_ISL_661293, EPI_ISL_661302 to 661303, EPI_ISL_676489 to 676491, EPI_ISL_676494 to 676496, EPI_ISL_676502 to 676508, EPI_ISL_676520, EPI_ISL_676531 to 676532, EPI_ISL_710595 to 710604, EPI_ISL_732654, EPI_ISL_766592 to 766593, EPI_ISL_766608 to 766611, EPI_ISL_766613, EPI_ISL_766615 to 766629, EPI_ISL_766633 to 766639, EPI_ISL_766694, EPI_ISL_766707, EPI_ISL_766710 to 766712, EPI_ISL_766714, EPI_ISL_766725 to 766730, EPI_ISL_775549, EPI_ISL_775572 to 775574, EPI_ISL_775584, EPI_ISL_775588, EPI_ISL_775590 to 775592, EPI_ISL_788989 to 788990, EPI_ISL_789036 to 789045, EPI_ISL_789047, EPI_ISL_831941 to 831942, EPI_ISL_831958, EPI_ISL_831973, EPI_ISL_831980 to 831981, EPI_ISL_831990 to 831992, EPI_ISL_831996 to 831997, EPI_ISL_832001 to 832002, EPI_ISL_913258 to 913262, EPI_ISL_913264 to 913265, EPI_ISL_913268 to 913274, EPI_ISL_913278 to 913280, EPI_ISL_913283, EPI_ISL_913285, EPI_ISL_913295 to 913297, EPI_ISL_913300, EPI_ISL_913306 to 913308, EPI_ISL_913310 to 913311, EPI_ISL_913313, EPI_ISL_913317, EPI_ISL_913319 to 913322, EPI_ISL_913325 to 913329, EPI_ISL_913344 to 913369, EPI_ISL_913371 to 913374, EPI_ISL_913453, EPI_ISL_913457 to 913458, EPI_ISL_913460 to 913463, EPI_ISL_913465, EPI_ISL_913469 to 913485, EPI_ISL_913487 to 913493, EPI_ISL_934323 to 934363, EPI_ISL_934375 to 934376, EPI_ISL_934378 to 934387, EPI_ISL_1008400, EPI_ISL_1008412 to 1008445, EPI_ISL_1008447 to 1008450, EPI_ISL_1008455 to 1008458, EPI_ISL_1008470, EPI_ISL_1008490 to 1008533, EPI_ISL_1008548, EPI_ISL_1008589 to 1008592, EPI_ISL_1008594 to 1008595, EPI_ISL_1008602, EPI_ISL_1064552 to 1064554, EPI_ISL_1064556 to 1064574, EPI_ISL_1064581 to 1064582, EPI_ISL_1064589 to 1064606, EPI_ISL_1064621 to 1064622, EPI_ISL_1064624, EPI_ISL_1064626 to 1064629, EPI_ISL_1064632, EPI_ISL_1064634 to 1064643, EPI_ISL_1064645 to 1064647, EPI_ISL_1064649 to 1064652, EPI_ISL_1064655, EPI_ISL_1064660 to 1064674, EPI_ISL_1064676, EPI_ISL_1064678 to 1064681, EPI_ISL_1064691 to 1064692, EPI_ISL_1064695 to 1064697, EPI_ISL_1064703 to 1064707, EPI_ISL_1168039 to 1168045, EPI_ISL_1168048 to 1168050, EPI_ISL_1168060 to 1168063, EPI_ISL_1168065 to 1168068, EPI_ISL_1168070 to 1168084, EPI_ISL_1168086 to 1168093, EPI_ISL_1168107 to 1168122, EPI_ISL_1168124 to 1168134, EPI_ISL_1168137 to 1168161, EPI_ISL_1168168 to 1168190, EPI_ISL_1190861 to 1190865, EPI_ISL_1190869 to 1190872, EPI_ISL_1231920 to 1231923, EPI_ISL_1231940 to 1231943, EPI_ISL_1231947, EPI_ISL_1231951, EPI_ISL_1231965 to 1231967, EPI_ISL_1231970, EPI_ISL_1231977, EPI_ISL_1231983, EPI_ISL_1231986 to 1231990, EPI_ISL_1231997 to 1232003, EPI_ISL_1232008 to 1232010, EPI_ISL_1300277 to 1300285, EPI_ISL_1300287 to 1300289, EPI_ISL_1300292 to 1300334			
see above	Klinisk mikrobiologi	The Public Health Agency of Sweden	Anna Risberg; Anna-Malin Linde; Carlo Berg; Department of Microbiology; Karin Tegmark-Wisell; Maria Lind Karlberg; Mattias Haukland; Mia Brytting; Noura Walai; Olov Svartstrom; Oskar Karlsson Lindsjo; Petra Edquist; Petra Holmstrom; Reza Advani; Samuel Ohman; Sandra Broddesson; Sofia Stamouli; The Public Health Agency of Sweden
EPI_ISL_615097 to 615100, EPI_ISL_623092 to 623094	Klinisk mikrobiologi Lanssjukhuset Ryhov, Jonkoping	The Public Health Agency of Sweden	Anna Risberg; Anna-Malin Linde; Karin Tegmark-Wisell; Maria Lind Karlberg; Mattias Haukland; Mia Brytting; Olov Svartstrom; Oskar Karlsson Lindsjo; Petra Edquist; Reza Advani; Sandra Broddesson
EPI_ISL_623089	Klinisk mikrobiologi Linkoping	The Public Health Agency of Sweden	Anna Risberg; Anna-Malin Linde; Karin Tegmark-Wisell; Maria Lind Karlberg; Mattias Haukland; Mia Brytting; Olov Svartstrom; Oskar Karlsson Lindsjo; Petra Edquist; Reza Advani; Sandra Broddesson
EPI_ISL_510841 to 510844, EPI_ISL_510868 to 510870, EPI_ISL_548250 to 548251, EPI_ISL_548253 to 548254, EPI_ISL_560973, EPI_ISL_615090 to 615091, EPI_ISL_615109, EPI_ISL_623087 to 623088, EPI_ISL_648169, EPI_ISL_648173, EPI_ISL_831974			
see above	Klinisk mikrobiologi NAL Trollhattan	The Public Health Agency of Sweden	Anna Risberg; Anna-Malin Linde; Department of Microbiology; Karin Tegmark-Wisell; Maria Lind Karlberg; Mattias Haukland; Mia Brytting; Olov Svartstrom; Oskar Karlsson Lindsjo; Petra Edquist; Reza Advani; Sandra Broddesson; The Public Health Agency of Sweden
EPI_ISL_429136 to 429148, EPI_ISL_429150 to 429158, EPI_ISL_429160, EPI_ISL_430860 to 430861, EPI_ISL_430863	Klinisk mikrobiologi Orebro	The Public Health Agency of Sweden	Anna Risberg; Anna-Malin Linde; Karin Tegmark-Wisell; Maria Lind Karlberg; Martin Sundqvist; Olov Svartstrom; Oskar Karlsson Lindsjo; Shaman Muradrasoli
EPI_ISL_582806 to 582807, EPI_ISL_648193	Klinisk mikrobiologi SAS Boras	The Public Health Agency of Sweden	Anna Risberg; Anna-Malin Linde; Karin Tegmark-Wisell; Maria Lind Karlberg; Mattias Haukland; Mia Brytting; Olov Svartstrom; Oskar Karlsson Lindsjo; Petra Edquist; Reza Advani; Sandra Broddesson
EPI_ISL_475144 to 475148, EPI_ISL_475153 to 475155, EPI_ISL_475160 to 475163, EPI_ISL_510818 to 510819, EPI_ISL_510853 to 510858, EPI_ISL_582797 to 582798, EPI_ISL_582800, EPI_ISL_582835 to 582838, EPI_ISL_623090, EPI_ISL_648162, EPI_ISL_654504 to 654505, EPI_ISL_654957, EPI_ISL_710594			

see above	Klinisk mikrobiologi Vasternorrland	The Public Health Agency of Sweden	Anna Risberg; Anna-Malin Linde; Department of Microbiology; Karin Tegmark-Wisell; Maria Lind Karlberg; Mattias Haukland; Mia Brytting; Olov Svartstrom; Oskar Karlsson Lindsjo; Petra Edquist; Reza Advani; Sandra Broddesson; Shamam Muradrasoli; The Public Health Agency of Sweden
EPI_ISL_510820 to 510824, EPI_ISL_510867, EPI_ISL_548252, EPI_ISL_560975, EPI_ISL_648133	Klinisk mikrobiologi centralsjukhuset Karlstad	The Public Health Agency of Sweden	Anna Risberg; Anna-Malin Linde; Karin Tegmark-Wisell; Maria Lind Karlberg; Mattias Haukland; Mia Brytting; Olov Svartstrom; Oskar Karlsson Lindsjo; Petra Edquist; Reza Advani; Sandra Broddesson
EPI_ISL_429115 to 429125, EPI_ISL_430848 to 430855, EPI_ISL_450834	Klinisk mikrobiologi och vardhygien Halmstad	The Public Health Agency of Sweden	Anna Risberg; Anna-Malin Linde; Arne Kotz; Karin Tegmark-Wisell; Maria Lind Karlberg; Mia Brytting; Olov Svartstrom; Oskar Karlsson Lindsjo; Shaman Muradrasoli; Theresa Enkirch
EPI_ISL_766692 to 766693, EPI_ISL_789012 to 789013, EPI_ISL_789029 to 789035	Klinisk mikrobiologi, Laboratoriemedicin	The Public Health Agency of Sweden	Department of Microbiology; The Public Health Agency of Sweden
EPI_ISL_534256, EPI_ISL_560988	Klinisk mikrobiologi, Laboratoriemedicin Gavleborg	The Public Health Agency of Sweden	Anna Risberg; Anna-Malin Linde; Karin Tegmark-Wisell; Maria Lind Karlberg; Mattias Haukland; Mia Brytting; Olov Svartstrom; Oskar Karlsson Lindsjo; Petra Edquist; Reza Advani; Sandra Broddesson
EPI_ISL_984733 to 984735, EPI_ISL_984950 to 984970, EPI_ISL_984973 to 984979, EPI_ISL_985041 to 985057, EPI_ISL_985331 to 985392, EPI_ISL_1091493 to 1091575, EPI_ISL_1093492 to 1093514, EPI_ISL_1093516 to 1093530, EPI_ISL_1093532 to 1093545, EPI_ISL_1166556 to 1166614, EPI_ISL_1224950 to 1224988, EPI_ISL_1303381, EPI_ISL_1303383 to 1303398, EPI_ISL_1303400 to 1303403, EPI_ISL_1305974 to 1306012			
see above	Klinisk mikrobiologi, Region Västerbotten	CBRN Defence and Security, Swedish Defence Research Agency	Andreas Sjödin; Anna-Lena Johansson; Annika Allard; Annika Osterman; Carolin Öhrman; Caroline Öhrman; David Sundell; Emelie Näslund Salomonsson; Ingrid Dacklin; Jonas Näslund; Kerstin Myrtennäs; Linda Karlsson; Malin Granberg; Mats Forsman; Stina Bäckman
EPI_ISL_424703, EPI_ISL_428148, EPI_ISL_428201, EPI_ISL_548962 to 548963, EPI_ISL_549024, EPI_ISL_549026, EPI_ISL_549176			
see above	Klinisk mikrobiologi, Region Västerbotten	Unit for Biological Agents, Department for CBRN Defence and Security, Swedish Defence Research Agency	FOI Bioinformatics team; FOI bioinformatics team
EPI_ISL_615080 to 615085, EPI_ISL_623080 to 623085	Klinisk mikrobiologi, Skanes universitetssjukhus, Lund	The Public Health Agency of Sweden	Anna Risberg; Anna-Malin Linde; Karin Tegmark-Wisell; Maria Lind Karlberg; Mattias Haukland; Mia Brytting; Olov Svartstrom; Oskar Karlsson Lindsjo; Petra Edquist; Reza Advani; Sandra Broddesson
EPI_ISL_454903, EPI_ISL_455904 to 455907	Klinisk mikrobiologi, UAS	The Public Health Agency of Sweden	Anna Risberg; Anna-Malin Linde; Karin Tegmark-Wisell; Maria Lind Karlberg; Mattias Haukland; Olov Svartstrom; Oskar Karlsson Lindsjo; Petra Edquist; Reza Advani; Shamam Muradrasoli
EPI_ISL_661290, EPI_ISL_676515, EPI_ISL_766630 to 766632, EPI_ISL_775531 to 775532, EPI_ISL_775542 to 775545, EPI_ISL_789048 to 789053, EPI_ISL_789055 to 789059, EPI_ISL_831979, EPI_ISL_913323 to 913324, EPI_ISL_934322			
see above	Klinisk mikrobiologi, Viruslab	The Public Health Agency of Sweden	Anna Risberg; Anna-Malin Linde; Carlo Berg; Department of Microbiology; Karin Tegmark-Wisell; Maria Lind Karlberg; Mattias Haukland; Mia Brytting; Noura Walai; Oskar Karlsson Lindsjo; Petra Edquist; Petra Holmstrom; Reza Advani; Sofia Stamouli; The Public Health Agency of Sweden
EPI_ISL_766697, EPI_ISL_1064709	Klinisk mikrobiologi, bakteriologi	The Public Health Agency of Sweden	Anna Risberg; Anna-Malin Linde; Carlo Berg; Department of Microbiology; Karin Tegmark-Wisell; Maria Lind Karlberg; Mattias Haukland; Mia Brytting; Noura Walai; Oskar Karlsson Lindsjo; Petra Edquist; Petra Holmstrom; Reza Advani; Samuel Ohman; Sofia Stamouli; The Public Health Agency of Sweden
EPI_ISL_1231926	Klinisk mikrobiologi, molekylar virologi	The Public Health Agency of Sweden	Anna Risberg; Anna-Malin Linde; Carlo Berg; Karin Tegmark-Wisell; Maria Lind Karlberg; Mattias Haukland; Mia Brytting; Noura Walai; Oskar Karlsson Lindsjo; Petra Edquist; Petra Holmstrom; Reza Advani; Sofia Stamouli
EPI_ISL_788999, EPI_ISL_831945 to 831946, EPI_ISL_1231916	Klinisk mikrobiologi, virus F68	The Public Health Agency of Sweden	Anna Risberg; Anna-Malin Linde; Carlo Berg; Department of Microbiology; Karin Tegmark-Wisell; Maria Lind Karlberg; Mattias Haukland; Mia Brytting; Noura Walai; Oskar Karlsson Lindsjo; Petra Edquist; Petra Holmstrom; Reza Advani; Sofia Stamouli; The Public Health Agency of Sweden
EPI_ISL_534245, EPI_ISL_560987	Kliniskt mikrobiologiska laboratoriet	The Public Health Agency of Sweden	Anna Risberg; Anna-Malin Linde; Karin Tegmark-Wisell; Maria Lind Karlberg; Mattias Haukland; Mia Brytting; Olov Svartstrom; Oskar Karlsson Lindsjo; Petra Edquist; Reza Advani; Sandra Broddesson
EPI_ISL_510871 to 510877, EPI_ISL_560976, EPI_ISL_582771 to 582775, EPI_ISL_594149 to 594153, EPI_ISL_615110, EPI_ISL_615117 to 615119, EPI_ISL_654952, EPI_ISL_660384 to 660414, EPI_ISL_661279, EPI_ISL_661284 to 661285, EPI_ISL_676512, EPI_ISL_676519			
see above	Klinsisk mikrobiologi Linkoping	The Public Health Agency of Sweden	Anna Risberg; Anna-Malin Linde; Department of Microbiology; Karin Tegmark-Wisell; Maria Lind Karlberg; Mattias Haukland; Mia Brytting; Olov Svartstrom; Oskar Karlsson Lindsjo; Petra Edquist; Reza Advani; Sandra Broddesson; The Public Health Agency of Sweden
EPI_ISL_864547 to 864562	Knappschaftskrankenhaus Bochum	Bundeswehr Institute of Microbiology	Alexandra Rehn; Antonios Katsounas; Enrico Georgi; Malena Bestehorn-Willmann; Markus Antwerpen; Mathias Walter; Mustafa Özçürümez; Roman Wölfel; Sabine Zange
EPI_ISL_434659, EPI_ISL_450811	Knivsta VC	The Public Health Agency of Sweden	Anna Risberg; Anna-Malin Linde; Johanna Carlson; Karin Tegmark-Wisell; Maria Lind Karlberg; Mia Brytting; Olov Svartstrom; Oskar Karlsson Lindsjo; Theresa Enkirch
EPI_ISL_700433, EPI_ISL_700436, EPI_ISL_700440, EPI_ISL_700450, EPI_ISL_700469, EPI_ISL_700474, EPI_ISL_700526, EPI_ISL_700529, EPI_ISL_700531, EPI_ISL_700542			
see above	Knysna CDC wc WLC	NHLS/UCT	Arash Iranzadeh; Bruna Galvao; Carolyn Williamson; Deelan Doolabh; Diana Hardie; Houriiyah Tegally; Innocent Mudau; Kruger Marais; Lynn Tyers; Marvin Hsiao; Stephen Korsman
EPI_ISL_696467, EPI_ISL_696495, EPI_ISL_696506	Knysna CDC wc WLC & NHLS/UCT	KRISP, KZN Research Innovation and Sequencing Platform	Arash Iranzadeh; Bruna Galvao; Carolyn Williamson; Deelan Doolabh; Diana Hardie; Emanuel James San; Houriiyah Tegally; Innocent Mudau; Jennifer Giandhari; Kruger Marais; Lynn Tyers; Marvin Hsiao; Stephen Korsman; Sureshnee Pillay; Tulio de Oliveira
EPI_ISL_640022, EPI_ISL_640038, EPI_ISL_640076, EPI_ISL_700419, EPI_ISL_700422, EPI_ISL_700426, EPI_ISL_700429, EPI_ISL_700456, EPI_ISL_700484, EPI_ISL_700501, EPI_ISL_700509 to 700510, EPI_ISL_700583			
see above	Knysna Hospital wc KNY	NHLS/UCT	Arash Iranzadeh; Bruna Galvao; Carolyn Williamson; Deelan Doolabh; Diana Hardie; Houriiyah Tegally; Innocent Mudau; Kruger Marais; Lynn Tyers; Marvin Hsiao; Stephen Korsman
EPI_ISL_960120	Knysna Hospital wc KNY	National Health Laboratory Service/UCT	Arash Iranzadeh; Bruna Galvao; Carolyn Williamson; Deelan Doolabh; Diana Hardie; Innocent Mudau; Kruger Marais; Lynn Tyers; Marvin Hsiao; Stephen Korsman
EPI_ISL_696463, EPI_ISL_696469, EPI_ISL_696477 to 696478, EPI_ISL_696482, EPI_ISL_696491	Knysna Hospital wc KNY & NHLS/UCT	KRISP, KZN Research Innovation and Sequencing Platform	Arash Iranzadeh; Bruna Galvao; Carolyn Williamson; Deelan Doolabh; Diana Hardie; Emanuel James San; Houriiyah Tegally; Innocent Mudau; Jennifer Giandhari; Kruger Marais; Lynn Tyers; Marvin Hsiao; Stephen Korsman; Sureshnee Pillay; Tulio de Oliveira
EPI_ISL_419296, EPI_ISL_479823	Kochi Prefectural Institute of Public Health	Pathogen Genomics Center, National Institute of Infectious Diseases	Akihiko Tokaji; Hajime Kamiya; Kentaro Itokawa; Makoto Kuroda; Masanori Hashino; Motoi Suzuki; Rina Tanaka; Tsuyoshi Sekizuka
EPI_ISL_862875	Komatsu Municipal Hospital	Fujita health University, School of Medicine, Department of Microbiology	Masahiro suzuki; Yohei Doi
EPI_ISL_407193	Korea Centers for Disease Control & Prevention (KCDC) Center for Laboratory Control of Infectious Diseases Division of Viral Diseases	Korea Centers for Disease Control & Prevention (KCDC) Center for Laboratory Control of Infectious Diseases Division of Viral Diseases	Heui Man Kim; Hye-Joon Jo; Jeong-Min Kim; Mi-Seon Kim; Myung Guk Han; Namjoo Lee; SangHee Woo; Sehee Park; Yoon-Seok Chung
EPI_ISL_754847	Korian Villa d'Azon	National Reference Center for Viruses of Respiratory Infections, Institut Pasteur, Paris	Angela Brisebarre; Camille Capel; Etienne Simon-Lorière; Marion Barbet; Maud Vanpeene; Méline Bizard; Sylvie Behillil; Sylvie van der Werf; Vincent Enouf
EPI_ISL_480103 to 480108, EPI_ISL_480221 to 480223	Koshigaya City Public Health Center	Pathogen Genomics Center, National Institute of Infectious Diseases	Aya Tamura; Hajime Kamiya; Kentaro Itokawa; Kyohei Sakata; Makoto Kuroda; Masanori Hashino; Motoi Suzuki; Rina Tanaka; Takumi Daimon; Tsuyoshi Sekizuka; Yoko Togawa; Yoshiko Hamada; Yuka Furui
EPI_ISL_1298475, EPI_ISL_1298478 to 1298479, EPI_ISL_1298485 to 1298486	Kosov CRH	The Institute of Molecular Biology and Genetics of NASU	M.Tukalo et al.
EPI_ISL_1072962	Kota Nopan Primary Health Centre, Mandailing Natal District	Institute of Tropical Disease, Universitas Airlangga; Faculty of Medicine, Universitas Sumatera Utara	Aldise M Nastri; Franciscus Ginting; Inke N D Lubis; Irbah R Nainggolan; Jezzy R Dewantari; Kazufumi Shimizu; Krisnoadi Rahardjo; Maria I Lusida; Meliani; Mirzan Hasibuan; Muhammad Ichwan; R Andika D Cahyadi; R Lia Kusumawati; Ramadhan Bestari; Rima R Prasetya; Soetjipto; Yasuko Mori
EPI_ISL_745109, EPI_ISL_745131,	Kraaifontein CHC wc KFP	National Health Laboratory Service (NHLS), Tygerberg	Bronwyn Kleinhans; Eduan Wilkindon; Gert van Zyl; Houriiyah Tegally; Kayla Delaney; Susan Engelbrecht; Tulio de Oliveira; Wolfgang Preiser

EPI_ISL_745133, EPI_ISL_745136, EPI_ISL_745164, EPI_ISL_745179			
EPI_ISL_696460, EPI_ISL_696498	Kranshoek Clinic wc KSH & NHLS/UCT	KRISP, KZN Research Innovation and Sequencing Platform	Arash Iranzadeh; Bruna Galvao; Carolyn Williamson; Deelan Doolabh; Diana Hardie; Emanuel James San; Houriiyah Tegally; Innocent Mudau; Jennifer Giandhari; Kruger Marais; Lynn Tyers; Marvin Hsiao; Stephen Korsman; Sureshnee Pillay; Tulio de Oliveira
EPI_ISL_869247, EPI_ISL_869250, EPI_ISL_869262 to 869264, EPI_ISL_872570	Krasnoyarsk Regional Clinical Hospital	WHO National Influenza Centre Russian Federation	Andrey Komissarov; Anna Ivanova; Artem Fadeev; Daria Danilenko; Dmitry Bazhenov; Dmitry Lioznov; Elena Nabieva; Georgii Bazykin; Ksenia Safina; Kseniya Komissarova; Mikhail Bakaev
EPI_ISL_434657	Kristianstadkliniken	The Public Health Agency of Sweden	Anna Risberg; Anna-Malin Linde; Karin Tegmark-Wisell; Maria Lind Karlberg; Mia Brytting; Mia Settergren Hammer; Olov Svartstrom; Oskar Karlsson Lindsjo; Theresa Enkirch
EPI_ISL_434651	Krokoms Halsocentral	The Public Health Agency of Sweden	Anna Risberg; Anna-Malin Linde; Karin Tegmark-Wisell; Maria Lind Karlberg; Martin Ersson; Mia Brytting; Olov Svartstrom; Oskar Karlsson Lindsjo; Theresa Enkirch
EPI_ISL_479991 to 479996	Kumamoto City Public Health Research Institute	Pathogen Genomics Center, National Institute of Infectious Diseases	Hajime Kamiya; Kaori Tashiro; Kentaro Itokawa; Makoto Kuroda; Masanori Hashino; Motoi Suzuki; Rina Tanaka; Tsuyoshi Sekizuka
EPI_ISL_479824	Kumamoto PrefecturalInstituteof Public-Health and Environmental Science	Pathogen Genomics Center, National Institute of Infectious Diseases	Hajime Kamiya; Kentaro Itokawa; Makoto Kuroda; Masanori Hashino; Motoi Suzuki; Rina Tanaka; Shunsuke Yahiro; Tsuyoshi Sekizuka
EPI_ISL_515181 to 515184, EPI_ISL_515247 to 515249	Kumasi Centre for Collaborative Research in Tropical Medicine, Kumasi.	Institute of Virology, Charité - Universitätsmedizin Berlin	Augustina Sylverken; Christian Drosten; Eric Adu; Jesse Addo Asamoah; Julia Schneider; Jörn Beheim-Schwarzbach; Michael Owusu; Philip El-Duah; Richard Phillips.; Richmond Gorman; Richmond Yeboah; Sherihane Aryeetey; Victor Max Corman
EPI_ISL_735492	Kumilla Medical College	Central Biological Research Laboratory and Department of Biochemistry and Molecular Biology	H. M. Abdullah Al Masud; Imam Hossen; Md. Arif Hossain; Md. Imranul Hoq; Md. Khondakar Raziur Rahman; Md. Omer Faruq; Mohammad Omar Faruque; Robiul Hasan Bhuiyan; Sajib Rudra; Shanta Paul
EPI_ISL_539881	Kungsbacka Narakut	The Public Health Agency of Sweden	Anna Risberg; Anna-Malin Linde; Karin Tegmark-Wisell; Maria Lind Karlberg; Mattias Haukland; Mia Brytting; Olov Svartstrom; Oskar Karlsson Lindsjo; Reza Advani; Sandra Broddesson; Theresa Enkirch
EPI_ISL_434644, EPI_ISL_450809, EPI_ISL_469067, EPI_ISL_475542, EPI_ISL_475569	Kungsholmsdoktorn	The Public Health Agency of Sweden	Anna Risberg; Anna-Malin Linde; Karin Tegmark-Wisell; Linus Hammar; Maria Lind Karlberg; Mattias Haukland; Mia Brytting; Olov Svartstrom; Oskar Karlsson Lindsjo; Petra Edquist; Reza Advani; Sandra Broddesson; Shamam Muradrasoli; Theresa Enkirch
EPI_ISL_434668, EPI_ISL_445232, EPI_ISL_455102, EPI_ISL_475529 to 475532, EPI_ISL_475568	Kungsors VC	The Public Health Agency of Sweden	Anna Risberg; Anna-Malin Linde; Jessica Karlsson; Karin Tegmark-Wisell; Maria Lind Karlberg; Mattias Haukland; Mia Brytting; Olov Svartstrom; Oskar Karlsson Lindsjo; Reza Advani; Sandra Broddesson; Theresa Enkirch
EPI_ISL_747239	Kuningan Publlic Health Office	West Java Health Laboratory; School of Life Sciences and Technology, Institut Teknologi Bandung	Azzania Fibriani; Cut Nur Cinthia Alamanda; Ema Rahmawati; Isak Solihin; Karimatu Khoirunnisa; Miftahul Faridl; Rifky Waluyajati Rachman; Rini Robiani; Ryan Bayusantika Ristandi
EPI_ISL_862268 to 862543, EPI_ISL_1103556 to 1103557	Kurnool Medical College (KMC)	CSIR Institute of Genomics and Integrative Biology	A. Surekha; Abhinav Jain; Avileli Surekha; Avula Renuka Devi; Bani Jolly; Damam Srinivasulu; Dasari Ankaiah; Disha Sharma; Gyan Ranjan; J. Vijaya Lakshmi; Juturu Vijaya Lakshmi; Mercy Rophina; Mohamed Imran; Mohit Kumar Divakar; Nambi Rajesh; P R Vanaja; PR Vanaja; Pallavali Roja Rani; Panyam Suresh; Paras Sehgal; Pulala Chandra; Pulala Chandrasekhar; Rahul C Bhoyar; Rajamadugu Hymavathy; S. Afsar; S. Umadevi; Sanaga Shanthi Kumari; Sridhar Sivasubbu; Valluri Anitha Lavanya; Vigneshwar Senthivel; Vinod Scaria
EPI_ISL_640030, EPI_ISL_700532, EPI_ISL_1040674	Kwamandlenkosi Clinic wc KMN	NHLS/UCT	Arash Iranzadeh; Bruna Galvao; Carolyn Williamson; Deelan Doolabh; Diana Hardie; Innocent Mudau; Kruger Marais; Lynn Tyers; Marvin Hsiao; Stephen Korsman
EPI_ISL_700437, EPI_ISL_700495, EPI_ISL_700535, EPI_ISL_700537, EPI_ISL_700541, EPI_ISL_700579	Kwanokuthula CDC wc KWA	NHLS/UCT	Arash Iranzadeh; Bruna Galvao; Carolyn Williamson; Deelan Doolabh; Diana Hardie; Houriiyah Tegally; Innocent Mudau; Kruger Marais; Lynn Tyers; Marvin Hsiao; Stephen Korsman
EPI_ISL_960123	Kwanokuthula CDC wc KWA	National Health Laboratory Service/UCT	Arash Iranzadeh; Bruna Galvao; Carolyn Williamson; Deelan Doolabh; Diana Hardie; Innocent Mudau; Kruger Marais; Lynn Tyers; Marvin Hsiao; Stephen Korsman
EPI_ISL_696465	Kwanokuthula CDC wc KWA & NHLS/UCT	KRISP, KZN Research Innovation and Sequencing Platform	Arash Iranzadeh; Bruna Galvao; Carolyn Williamson; Deelan Doolabh; Diana Hardie; Emanuel James San; Houriiyah Tegally; Innocent Mudau; Jennifer Giandhari; Kruger Marais; Lynn Tyers; Marvin Hsiao; Stephen Korsman; Sureshnee Pillay; Tulio de Oliveira
EPI_ISL_1061406, EPI_ISL_1061408	Kwong Wah Hospital	Hong Kong Children's Hospital	David Christopher Lung; Garnet Kwan-Yue Choi; Herman Tse; Sally Cheuk-Ying Wong
EPI_ISL_426393, EPI_ISL_510086, EPI_ISL_510091, EPI_ISL_523842, EPI_ISL_523855, EPI_ISL_539805, EPI_ISL_602616, EPI_ISL_637026			
see above	Kwong Wah Hospital	Hong Kong Department of Health	Alan K.L. Tsang; Chan Rickjason C.W.; Cheng Peter K.C.; Dominic N.C. Tsang; Edman T.K. Lam; Lam Edman T.K.; Mak Gannon C.K.; Peter C.W. Yip; Rickjason C.W. Chan; Tsang Dominic N.C.
EPI_ISL_420039 to 420040	L'Air du Temps	National Reference Center for Viruses of Respiratory Infections, Institut Pasteur, Paris	Angela Brisebarre; Etienne Simon-Lorière; Flora Donati; Marion Barbet; Maud Vanpeene; Mélanie Albert; Méline Bizard; Sylvie Behillil; Sylvie van der Werf; Vincent Enouf
EPI_ISL_508931	L'Hôpital Nord-Ouest Tarare-Grandris	CNR Virus des Infections Respiratoires - France SUD	Alexandre Gaymard; Antonin Bal; Bruno Lina; Carine Moustaud; Florence Morfin-Sherpa; Gregory Destras; Gwendolyne Burfin; Laurence Josset; Martine Valette; Maude Bouscambert-Duchamp; Raphaelle Lamy; Solenne Brun
EPI_ISL_913667, EPI_ISL_1272782 to 1272797	LA Office of Public Health Laboratories	Centers for Disease Control and Prevention Division of Viral Diseases, Pathogen Discovery	Anna Montmayeur; Anna Uehara; Ben L. Rambo-Martin; Clinton R. Paden; Dhwani Batra; Haibin Wang; Jasmine Padilla; Jing Zhang; Justin Lee; Katie Dillon; Krista Queen; Kristen Knipe; Kristine Lacek; Lori Rowe; Mark Burroughs; Matthew Schmerer; Mili Sheth; Peter Cook; Peter W. Cook; Rachel Marine; Sam Shepard; Sarah Nobles; Shoshona Le; Suxiang Tong; Yan Li; Ying Tao
EPI_ISL_751620, EPI_ISL_751669, EPI_ISL_751696, EPI_ISL_751722, EPI_ISL_751752, EPI_ISL_903613, EPI_ISL_903627, EPI_ISL_903651, EPI_ISL_903689, EPI_ISL_903693, EPI_ISL_903709, EPI_ISL_903720, EPI_ISL_903728, EPI_ISL_903739, EPI_ISL_903752, EPI_ISL_903765, EPI_ISL_903776, EPI_ISL_903832, EPI_ISL_903877, EPI_ISL_903906			
see above	LA Office of Public Health Laboratories	Genomics and Discovery, Respiratory Viruses Branch, Division of Viral Diseases, Centers for Disease Control and Prevention	Anna Montmayeur; Anna Uehara; Ben L. Rambo-Martin; Clinton R. Paden; Dhwani Batra; Haibin Wang; Jasmine Padilla; Jing Zhang; Justin Lee; Krista Queen; Lori Rowe; Mark Burroughs; Mili Sheth; Peter W. Cook; Rachel Marine; Sarah Nobles; Suxiang Tong; Yan Li; Ying Tao
EPI_ISL_424868, EPI_ISL_452106 to 452107, EPI_ISL_535305 to 535358, EPI_ISL_913997 to 913998, EPI_ISL_914012, EPI_ISL_955269, EPI_ISL_955275, EPI_ISL_1008897 to 1008900, EPI_ISL_1008921 to 1008923, EPI_ISL_1196872 to 1196885			
see above	LA Office of Public Health Laboratories	Pathogen Discovery, Respiratory Viruses Branch, Division of Viral Diseases, Centers for Disease Control and Prevention	Alison S. Laufer Halpin; Anna Kelleher; Anna Montmayeur; Anna Uehara; Brian Lynch; Christopher A. Elkins; Clinton Paden; Clinton R. Paden; Haibin Wang; Han Jia Justin Ng; Jing Zhang; Krista Queen; Mary S. Keckler; Peter Cook; Rachel Marine; Suxiang Tong; Yan Li; Ying Tao; Zachary Weiner
EPI_ISL_1094333, EPI_ISL_1094410, EPI_ISL_1094412, EPI_ISL_1094871 to 1094873, EPI_ISL_1094875 to 1094877, EPI_ISL_1094881 to 1094884, EPI_ISL_1094886, EPI_ISL_1095260 to 1095261			
see above	LA Office of Public Health Laboratories	Respiratory Viruses Branch, Division of Viral Diseases, Centers for Disease Control and Prevention	Anna Montmayeur; Anna Uehara; Ben L. Rambo-Martin; Clinton R. Paden; Dhwani Batra; Haibin Wang; Jasmine Padilla; Jing Zhang; Justin Lee; Krista Queen; Lori Rowe; Mark Burroughs; Mili Sheth; Peter W. Cook; Rachel Marine; Sarah Nobles; Suxiang Tong; Yan Li; Ying Tao
EPI_ISL_1007632, EPI_ISL_1120842, EPI_ISL_1120845 to 1120854	LA SOURCE	Laboratory of genomics and metagenomics, Institute of Microbiology, University Hospital Centre and University of Lausanne, Switzerland	Claire Bertelli; Damien Jacot; Gilbert Greub; Sébastien Aeby; Trestan Pillonel
EPI_ISL_1118159 to 1118204, EPI_ISL_1121034 to 1121036	LAB ANALISI PO CARDARELLI CAMPOBASSO	Istituto Zooprofilattico Sperimentale dell'Abruzzo e Molise "G. Caporale"	Ancora M; Calistri P; Cammà C; Curini V; Di Domenico M; Di Pasquale A; Felice V. Lorusso A; Mangone I; Marcacci M; Niro G; Puglia I; Rinaldi A; Savini G; Scialabba S; Scutellà M
EPI_ISL_1110581 to 1110595	LAB ANALISI PO CARDARELLI CB	Istituto Zooprofilattico Sperimentale dell'Abruzzo e Molise "G. Caporale"	Ancora M; Calistri P; Cammà C; Curini V; Di Domenico M; Di Pasquale A; Lorusso A; Mangone I; Marcacci M; Niro G; Puglia I; Rinaldi A; Savini G; Scialabba S; Scutellà M
EPI_ISL_1163736	LABCOVID_HCPA	LABCOVID_HCPA	Barth AL; Martins AF; Monteiro F; Rosset C; Volpato F; Wink PL; Zavascki AP; de Paris F

EPI_ISL_1133120 to 1133144, EPI_ISL_1161401 to 1161415, EPI_ISL_1163530, EPI_ISL_1163532, EPI_ISL_1163693 to 1163705, EPI_ISL_1163709 to 1163716, EPI_ISL_1163737			
see above	LABCOVID_HCPA	LABRESIS_HCPA	Barth AL; Martins AF; Monteiro F; Rosset C; Volpato F; Wink PL; Zavascki AP; de Paris F
EPI_ISL_1137459, EPI_ISL_1137475 to 1137477, EPI_ISL_1158060 to 1158062	LABDIS-UAGro	Instituto Nacional de Medicina Genomica	Adolfo Roman-Roman; Alcaraz N; Azucena D. Alvarez-Diaz; Berenice Illades-Aguiar; Cedro-Tanda A; Ciresthel Bello-Rios; Cisneros-Villanueva M; Daniel Hernandez-Sotelo; Fredy O Beltran-Anaya; Gladys W. Valente-Nio; Gonzalez-Barrera D; Herrera-Montalvo LA; Hidalgo-Miranda A; Hugo A. Rodriguez-Ruiz; Jaqueline Loaeza-Loaeza; Julio C. Azucar-Hesiquio; Luis I. Basilio-Leyva.; Madian Valadez-Quinones; Marco Antonio-Leyva; Mendoza-Vargas A; Oscar del Moral-Hernandez; Ramirez-Vega O; Rangel-DeLeon D; Reyes-Grajeda JP; Roberto Dircio-Maldonado; Sifuentes-Rojas C
EPI_ISL_981492, EPI_ISL_981497	LABM BIOLAM CLINIQUE ESTREE	Department of Virology, Henri Mondor University Hospital, Assistance Publique Hôpitaux de Paris, Université Paris-Est Créteil, INSERM U955	Alexandre Soulier; Christophe Rodriguez; Elisabeth Trawinski; Guillaume Gricourt; Jean-Michel Pawlotsky; Melissa N'Debi; Slim Fourati; Vanessa Demontant
EPI_ISL_416499 to 416500, EPI_ISL_418234, EPI_ISL_418240	LABM GH nord Essonne	National Reference Center for Viruses of Respiratory Infections, Institut Pasteur, Paris	Angela Brisebarre; Christine Lambert; Etienne Simon-Lorière; Flora Donati; Marion Barbet; Maud Vanpeene; Mélanie Albert; Méline Bizard; Mélnie Albert; Sylvie Behillil; Sylvie van der Werf; Vincent Enouf
EPI_ISL_428354, EPI_ISL_428361 to 428362, EPI_ISL_428365, EPI_ISL_443260, EPI_ISL_443305, EPI_ISL_443314	LABM GH nord Essonne de Longjumeau - BP 125	National Reference Center for Viruses of Respiratory Infections, Institut Pasteur, Paris	Angela Brisebarre; Etienne Simon-Lorière; Flora Donati; Marion Barbet; Maud Vanpeene; Mélanie Albert; Méline Bizard; Sylvie Behillil; Sylvie van der Werf; Vincent Enouf
EPI_ISL_1085290 to 1085291, EPI_ISL_1085317, EPI_ISL_1085404	LABM SAINT MARTIN	Department of Virology, Henri Mondor University Hospital, Assistance Publique Hôpitaux de Paris, Université Paris-Est Créteil, INSERM U955	Alexandre Soulier; Christophe Rodriguez; Elisabeth Trawinski; Guillaume Gricourt; Jean-Michel Pawlotsky; Melissa N'Debi; Slim Fourati; Vanessa Demontant
EPI_ISL_1147555 to 1147556, EPI_ISL_1151781, EPI_ISL_1157261, EPI_ISL_1217618, EPI_ISL_1217652, EPI_ISL_1217702, EPI_ISL_1285088, EPI_ISL_1285090, EPI_ISL_1285105			
see above	LABOR CHEMNITZ - Zentrum für Diagnostik am Klinikum Chemnitz	Robert Koch Institute	
EPI_ISL_1312950 to 1312951, EPI_ISL_1312989, EPI_ISL_1313016 to 1313017, EPI_ISL_1313039 to 1313043	LABORATOIRE ALPIGENE	CNR Virus des Infections Respiratoires - France SUD	Antonin Bal; Bruno Lina; Gregory Destras; Gwendolyne Burfin; Hadrien Regue; Laurence Josset; Martine Valette; Quentin Semanas
EPI_ISL_1313091 to 1313092	LABORATOIRE ANABIO BERGSON	CNR Virus des Infections Respiratoires - France SUD	Antonin Bal; Bruno Lina; Gregory Destras; Gwendolyne Burfin; Hadrien Regue; Laurence Josset; Martine Valette; Quentin Semanas
EPI_ISL_1135045	LABORATOIRE BIOAXIOME	CNR Virus des Infections Respiratoires - France SUD	Antonin Bal; Bruno Lina; Gregory Destras; Gwendolyne Burfin; Hadrien Regue; Laurence Josset; Martine Valette; Quentin Semanas
EPI_ISL_1190838 to 1190839	LABORATOIRE BIOESTEREL	CNR Virus des Infections Respiratoires - France SUD	Antonin Bal; Bruno Lina; Gregory Destras; Gwendolyne Burfin; Hadrien Règue; Laurence Josset; Martine Valette; Quentin Semanas
EPI_ISL_1135046 to 1135052	LABORATOIRE BIOFUSION	CNR Virus des Infections Respiratoires - France SUD	Antonin Bal; Bruno Lina; Gregory Destras; Gwendolyne Burfin; Hadrien Regue; Laurence Josset; Martine Valette; Quentin Semanas
EPI_ISL_1165966 to 1165967	LABORATOIRE CERBALLIANCE PLT VILLON	CNR Virus des Infections Respiratoires - France SUD	Antonin Bal; Bruno Lina; Gregory Destras; Gwendolyne Burfin; Hadrien Regue; Laurence Josset; Martine Valette; Quentin Semanas
EPI_ISL_1313005	LABORATOIRE CERDIBIO-CHARENTES	CNR Virus des Infections Respiratoires - France SUD	Antonin Bal; Bruno Lina; Gregory Destras; Gwendolyne Burfin; Hadrien Regue; Laurence Josset; Martine Valette; Quentin Semanas
EPI_ISL_1190815	LABORATOIRE LABOSUD BIO 2000	CNR Virus des Infections Respiratoires - France SUD	Antonin Bal; Bruno Lina; Gregory Destras; Gwendolyne Burfin; Hadrien Règue; Laurence Josset; Martine Valette; Quentin Semanas
EPI_ISL_1265846	LABORATOIRE LARTAUD et MONNERY	CNR Virus des Infections Respiratoires - France SUD	Antonin Bal; Bruno Lina; Gregory Destras; Gwendolyne Burfin; Hadrien Regue; Laurence Josset; Martine Valette; Quentin Semanas
EPI_ISL_1201857 to 1201866, EPI_ISL_1313168, EPI_ISL_1313713, EPI_ISL_1313923	LABORATOIRE NOVELAB	CNR Virus des Infections Respiratoires - France SUD	Antonin Bal; Bruno Lina; Gregory Destras; Gwendolyne Burfin; Hadrien Regue; Laurence Josset; Martine Valette; Quentin Semanas
EPI_ISL_1265760, EPI_ISL_1265785, EPI_ISL_1265880 to 1265882	LABORATOIRE ORIADE	CNR Virus des Infections Respiratoires - France SUD	Antonin Bal; Bruno Lina; Gregory Destras; Gwendolyne Burfin; Hadrien Regue; Laurence Josset; Martine Valette; Quentin Semanas
EPI_ISL_956303	LABORATORIO ANALIZAR SYNLAB	Instituto Nacional de Salud- Dirección de Investigación en Salud Pública	Carlos Franco-Muñoz; Diego A. Álvarez-Díaz; Diego Andrés Prada; Gerardo Santamaría; Hector Alejandro Ruiz-Moreno; Jhonnatan Reales-González; Julian Naizaque; Katherine Laiton-Donato; Magdalena Wiesner; Marcela Mercado-Reyes; Maria T. Herrera-Sepúlveda; Martha Lucia Ospina Martinez; Mauricio Pacheco-Montealegre; Sheryll Corchuelo
EPI_ISL_1220055 to 1220057	LABORATORIO BIOLOGIA MOLECULAR IMAT SAS	Instituto Nacional de Salud- Dirección de Investigación en Salud Pública	Carlos Franco-Muñoz; Diego A. Álvarez-Díaz; Diego Andrés Prada; Gerardo Santamaría; Hector Alejandro Ruiz-Moreno; Jhonnatan Reales-González; Julian Naizaque; Katherine Laiton-Donato; Magdalena Wiesner; Marcela Mercado-Reyes.; Maria T. Herrera-Sepúlveda; Martha Lucia Ospina Martínez; Sheryll Corchuelo
EPI_ISL_445271	LABORATORIO CLINICA CHILLAN	Instituto de Salud Publica de Chile	Alejandra Acevedo; Andrés E Castillo; Bárbara Parra; Carolina Tambley; Gabriel Leal; Jaime Lagos; Jorge Fernandez; Loredana Arata; Patricia Bustos; Paz Tapia; Rodrigo Fasce; Winston Andrade
EPI_ISL_445333	LABORATORIO CLINICA UNIVERSITARIA DE CONCEPCION	Instituto de Salud Publica de Chile	Alejandra Acevedo; Andrés E Castillo; Bárbara Parra; Carolina Tambley; Gabriel Leal; Jaime Lagos; Jorge Fernandez; Loredana Arata; Patricia Bustos; Paz Tapia; Rodrigo Fasce; Winston Andrade
EPI_ISL_1196426 to 1196427	LABORATORIO CLINICO CENAHCE	Inciensa, Instituto Costarricense de Investigación y Enseñanza en Nutrición y Salud	Adriana Godínez; Claudio Soto-Garita; Estela Cordero; Francisco Duarte; Hebleen Porras; Melany Calderón & Andrés Feoli-Grant
EPI_ISL_914832 to 914833, EPI_ISL_1067593, EPI_ISL_1067620, EPI_ISL_1196432	LABORATORIO CLINICO LABIN	Inciensa, Instituto Costarricense de Investigación y Enseñanza en Nutrición y Salud	Adriana Godínez; Caterina Guzmán; Claudio Soto-Garita; Estela Cordero; Francisco Duarte; Hebleen Porras; Melany Calderón; Melany Calderón & David Rodríguez-Masís; Melany Calderón & Pei Ling Chan Ma; Nazareth Ruiz & Pei Ling Chan Ma
EPI_ISL_1220048	LABORATORIO CLÍNICO FUNDACIÓN HOSPITAL SAN PEDRO	Instituto Nacional de Salud- Dirección de Investigación en Salud Pública	Carlos Franco-Muñoz; Diego A. Álvarez-Díaz; Diego Andrés Prada; Gerardo Santamaría; Hector Alejandro Ruiz-Moreno; Jhonnatan Reales-González; Julian Naizaque; Katherine Laiton-Donato; Magdalena Wiesner; Marcela Mercado-Reyes.; Maria T. Herrera-Sepúlveda; Martha Lucia Ospina Martínez; Sheryll Corchuelo
EPI_ISL_792402 to 792423	LABORATORIO DE CAMPAÑA COVID 19 INTA	Instituto de Biotecnología, IABIMO (CONICET), Instituto de Virología, IVIT(CONICET), Instituto de Patobiología, IPVET(CONICET), CICVyA, INTA on behalf of 'Proyecto Argentino Interinstitucional de genomica de SARS-CoV-2' (PAIS Consortium)	A; AF; AJ; AV; Asurmendi; B; Bengoa Luoni; Cacciabué; Chimeno; Craig; D; Del Médico; Delgado; Diagiacomo; Distéfano; Dus Santos; Díaz Carrasco; E; F; Farber; Fass; Ferández PC; Franco; Fusco; GA; Garbaccio; Gioffré; Gómez; Huertas; I; J; K; Klepp; König; L; LC; Lozano Calderón; Lucero; M; MD; MG; MI; MJ; MPD; MS; Muñoz Hidalgo; NB; Olivera; P; PA; Paniego; Pedroarias; Peralta; Perea; Pereda; Puebla; Rimondi; Rivarola; Rodríguez; Rosende; S; Schammas; Sioya; Soria; Trono; V; VC; Vera; Viegas, M.; Vissani; W; Zavallo; Zumárraga; Álvarez
EPI_ISL_956302	LABORATORIO IMAT	Instituto Nacional de Salud- Dirección de Investigación en Salud Pública	Carlos Franco-Muñoz; Diego A. Álvarez-Díaz; Diego Andrés Prada; Gerardo Santamaría; Hector Alejandro Ruiz-Moreno; Jhonnatan Reales-González; Julian Naizaque; Katherine Laiton-Donato; Magdalena Wiesner; Marcela Mercado-Reyes; Maria T. Herrera-Sepúlveda; Martha Lucia Ospina Martinez; Mauricio Pacheco-Montealegre; Sheryll Corchuelo
EPI_ISL_445279	LABORATORIO INMUNOLAB SPA	Instituto de Salud Publica de Chile	Alejandra Acevedo; Andrés E Castillo; Bárbara Parra; Carolina Tambley; Gabriel Leal; Jaime Lagos; Jorge Fernandez; Loredana Arata; Patricia Bustos; Paz Tapia; Rodrigo Fasce; Winston Andrade
EPI_ISL_445273 to 445274, EPI_ISL_445278	LABORATORIO TORRE MEDICA LTDA.	Instituto de Salud Publica de Chile	Alejandra Acevedo; Andrés E Castillo; Bárbara Parra; Carolina Tambley; Gabriel Leal; Jaime Lagos; Jorge Fernandez; Loredana Arata; Patricia Bustos; Paz Tapia; Rodrigo Fasce; Winston Andrade
EPI_ISL_682275 to 682277	LABORATORIOS LABIN	Inciensa, Instituto Costarricense de Investigación y Enseñanza en Nutrición y Salud	Adriana Godinez; Claudio Soto-Garita; Estela Cordero; Francisco Duarte; Hebleen Porras; Melany Calderon & Pei Ling Chan
EPI_ISL_1239554, EPI_ISL_1239560, EPI_ISL_1239563, EPI_ISL_1239583 to 1239584, EPI_ISL_1239624, EPI_ISL_1239656 to 1239660, EPI_ISL_1239705, EPI_ISL_1239724 to 1239726, EPI_ISL_1239728 to 1239729			
see above	LABORATORIUM BADA KLINICZNYCH WSSE w OPOLU	1. National Institute of Public Health - National Institute of Hygiene; 2. Eurofins Genomics Europe Sequencing GmbH	ECDC COVID-19 WGS support team; Eurofins Genomics Europe Sequencing Team; Gierczyki Rafa; Sadkowska-Todys Magorzata; Wokowicz Tomasz; Zacharczuk Katarzyna
EPI_ISL_1313049 to 1313051,	LABOSUD PROVENCE	CNR Virus des Infections Respiratoires - France SUD	Antonin Bal; Bruno Lina; Gregory Destras; Gwendolyne Burfin; Hadrien Regue; Laurence Josset; Martine Valette; Quentin Semanas

EPI_ISL_1313150			
EPI_ISL_1261698 to 1261699	LACEN - Laboratório Central de Saúde Pública de Pernambuco	Evandro Chagas Institute	A.M.; Barbagelata; E.C.; E.M.A.; Ferreira; J.A.; Junior; K.C.; L.C.; L.S.; M.C.; P.S.; Pinheiro; Santos; Silva; Sousa; Sousa Junior; W.D.C.; da Silva
EPI_ISL_1261687	LACEN - Laboratório Central de Saúde Pública de Roraima	Evandro Chagas Institute	A.M.; Barbagelata; E.C.; E.M.A.; Ferreira; J.A.; Junior; K.C.; L.C.; L.S.; M.C.; P.S.; Pinheiro; Santos; Silva; Sousa; Sousa Junior; W.D.C.; da Silva
EPI_ISL_918551, EPI_ISL_918553 to 918561	LACEN - Laboratório Central de Saúde Pública do Amapa	Evandro Chagas Institute	A.M.; Barbagelata; E.C.; E.M.A.; Ferreira; J.A.; Junior; K.C.; L.C.; L.S.; M.C.; P.S.; Pinheiro; Santos; Silva; Sousa; Sousa Junior; W.D.C.; da Silva
EPI_ISL_1164976, EPI_ISL_1164981 to 1164982, EPI_ISL_1164984 to 1164985, EPI_ISL_1261686, EPI_ISL_1261688 to 1261689, EPI_ISL_1261692, EPI_ISL_1261695 to 1261696			
see above	LACEN - Laboratório Central de Saúde Pública do Amapá	Evandro Chagas Institute	A.M.; Barbagelata; E.C.; E.M.A.; Ferreira; J.A.; Junior; K.C.; L.C.; L.S.; M.C.; P.S.; Pinheiro; Santos; Silva; Sousa; Sousa Junior; W.D.C.; da Silva
EPI_ISL_925846, EPI_ISL_925916, EPI_ISL_926446	LACEN - Laboratório Central de Saúde Pública do Amazonas	Evandro Chagas Institute Virology	A.M.; Barbagelata; E.C.; E.M.A.; Ferreira; J.A.; Junior; K.C.; L.C.; L.S.; M.C.; P.S.; Pinheiro; Santos; Silva; Sousa; Sousa Junior; W.D.C.; da Silva
EPI_ISL_918499 to 918512, EPI_ISL_918531 to 918535, EPI_ISL_1261683, EPI_ISL_1261685, EPI_ISL_1261690, EPI_ISL_1261694	LACEN - Laboratório Central de Saúde Pública do Amazonas	Evandro Chagas Institute	A.M.; Barbagelata; E.C.; E.M.A.; Ferreira; J.A.; Junior; K.C.; L.C.; L.S.; M.C.; P.S.; Pinheiro; Santos; Silva; Sousa; Sousa Junior; W.D.C.; da Silva
EPI_ISL_918536 to 918544	LACEN - Laboratório Central de Saúde Pública do Ceara	Evandro Chagas Institute	A.M.; Barbagelata; E.C.; E.M.A.; Ferreira; J.A.; Junior; K.C.; L.C.; L.S.; M.C.; P.S.; Pinheiro; Santos; Silva; Sousa; Sousa Junior; W.D.C.; da Silva
EPI_ISL_1164970 to 1164971, EPI_ISL_1164973, EPI_ISL_1164980, EPI_ISL_1164986, EPI_ISL_1164993, EPI_ISL_1164995, EPI_ISL_1261684, EPI_ISL_1261693, EPI_ISL_1261697			
see above	LACEN - Laboratório Central de Saúde Pública do Ceará	Evandro Chagas Institute	A.M.; Barbagelata; E.C.; E.M.A.; Ferreira; J.A.; Junior; K.C.; L.C.; L.S.; M.C.; P.S.; Pinheiro; Santos; Silva; Sousa; Sousa Junior; W.D.C.; da Silva
EPI_ISL_1086373 to 1086374, EPI_ISL_1164979, EPI_ISL_1164996, EPI_ISL_1164998, EPI_ISL_1261700	LACEN - Laboratório Central de Saúde Pública do Maranhao	Evandro Chagas Institute	A.M.; Barbagelata; E.C.; E.M.A.; Ferreira; J.A.; Junior; K.C.; L.C.; L.S.; M.C.; P.S.; Pinheiro; Santos; Silva; Sousa; Sousa Junior; W.D.C.; da Silva
EPI_ISL_918515 to 918517, EPI_ISL_918522 to 918530, EPI_ISL_918545 to 918550, EPI_ISL_918552	LACEN - Laboratório Central de Saúde Pública do Para	Evandro Chagas Institute	A.M.; Barbagelata; E.C.; E.M.A.; Ferreira; J.A.; Junior; K.C.; L.C.; L.S.; M.C.; P.S.; Pinheiro; Santos; Silva; Sousa; Sousa Junior; W.D.C.; da Silva
EPI_ISL_1086377, EPI_ISL_1164989 to 1164992, EPI_ISL_1164994, EPI_ISL_1164999	LACEN - Laboratório Central de Saúde Pública do Paraiba	Evandro Chagas Institute	A.M.; Barbagelata; E.C.; E.M.A.; Ferreira; J.A.; Junior; K.C.; L.C.; L.S.; M.C.; P.S.; Pinheiro; Santos; Silva; Sousa; Sousa Junior; W.D.C.; da Silva
EPI_ISL_904120 to 904121, EPI_ISL_1164972, EPI_ISL_1164974 to 1164975, EPI_ISL_1164978, EPI_ISL_1164983	LACEN - Laboratório Central de Saúde Pública do Pará	Evandro Chagas Institute	A.M.; Barbagelata; E.C.; E.M.A.; Ferreira; J.A.; Junior; K.C.; L.C.; L.S.; M.C.; P.S.; Pinheiro; Santos; Silva; Sousa; Sousa Junior; W.D.C.; da Silva
EPI_ISL_1166615	LACEN - Laboratório Central de Saúde Pública do Rio Grande do Norte	Evandro Chagas Institute Virology	A.M.; Barbagelata; E.C.; E.M.A.; Ferreira; J.A.; Junior; K.C.; L.C.; L.S.; M.C.; P.S.; Pinheiro; Santos; Silva; Sousa; Sousa Junior; W.D.C.; da Silva
EPI_ISL_1086375 to 1086376, EPI_ISL_1164977, EPI_ISL_1164987 to 1164988, EPI_ISL_1164997	LACEN - Laboratório Central de Saúde Pública do Rio Grande do Norte	Evandro Chagas Institute	A.M.; Barbagelata; E.C.; E.M.A.; Ferreira; J.A.; Junior; K.C.; L.C.; L.S.; M.C.; P.S.; Pinheiro; Santos; Silva; Sousa; Sousa Junior; W.D.C.; da Silva
EPI_ISL_918513	LACEN - Laboratório Central de Saúde Pública do Roraima	Evandro Chagas Institute	A.M.; Barbagelata; E.C.; E.M.A.; Ferreira; J.A.; Junior; K.C.; L.C.; L.S.; M.C.; P.S.; Pinheiro; Santos; Silva; Sousa; Sousa Junior; W.D.C.; da Silva
EPI_ISL_717809, EPI_ISL_717832 to 717836, EPI_ISL_717841, EPI_ISL_717910 to 717919, EPI_ISL_717958, EPI_ISL_717963 to 717964	LACEN Dr. Francisco Rimolo Neto	Bioinformatics Laboratory / LNCC	Alexandra L Gerber; Amilcar Tanuri; Ana Paula de C Guimarães; Ana Tereza R de Vasconcelos; Andréa Cony Cavalcanti; Carolina M Voloch; Claudia dos Santos Rodrigues; Cynthia C Cardoso; Diana Mariani; Luiz G P de Almeida; Otavio Bustrolini; Ronaldo da Silva F Jr; Terezinha M P P Castiñeira
EPI_ISL_717785 to 717790, EPI_ISL_717792, EPI_ISL_717794 to 717800, EPI_ISL_717803 to 717804, EPI_ISL_717899 to 717909, EPI_ISL_717962	LACEN RJ - Noel Nutels	Bioinformatics Laboratory / LNCC	Alexandra L Gerber; Amilcar Tanuri; Ana Paula de C Guimarães; Ana Tereza R de Vasconcelos; Andréa Cony Cavalcanti; Carolina M Voloch; Claudia dos Santos Rodrigues; Cynthia C Cardoso; Diana Mariani; Luiz G P de Almeida; Otavio Bustrolini; Ronaldo da Silva F Jr; Terezinha M P P Castiñeira
EPI_ISL_1293052 to 1293055, EPI_ISL_1303499 to 1303505	LACEN de Rondonia	Instituto Adolfo Lutz, Interdiciplinary Procedures Center, Strategic Laboratory	Caio Vinicius Dias Lopes; Claudia Regina Gonçalves; Claudio Tavares Sacchi; Erica Valessa Ramos Gomes; Karoline Rodrigues Campos
EPI_ISL_985318 to 985319	LACEN de Santa Catarina	Instituto Adolfo Lutz, Interdiciplinary Procedures Center, Strategic Laboratory	Claudia Regina Gonçalves; Claudio Tavares Sacchi; Erica Valessa Ramos Gomes; Karoline Rodrigues Campos
EPI_ISL_882663 to 882664, EPI_ISL_1196285 to 1196294, EPI_ISL_1293051, EPI_ISL_1303506 to 1303508	LACEN do Distrito Federal	Instituto Adolfo Lutz, Interdiciplinary Procedures Center, Strategic Laboratory	Caio Vinicius Dias Lopes; Claudia Regina Gonçalves; Claudio Tavares Sacchi; Erica Valessa Ramos Gomes; Karoline Rodrigues Campos
EPI_ISL_943988 to 943990, EPI_ISL_985303 to 985317, EPI_ISL_1039691 to 1039695, EPI_ISL_1041509, EPI_ISL_1303510 to 1303517	LACEN do Estado de Goias	Instituto Adolfo Lutz, Interdiciplinary Procedures Center, Strategic Laboratory	Caio Vinicius Dias Lopes; Claudia Regina Gonçalves; Claudio Tavares Sacchi; Erica Valessa Ramos Gomes; Karoline Rodrigues Campos
EPI_ISL_943973 to 943987, EPI_ISL_943991, EPI_ISL_1303509	LACEN do Estado de Tocantins	Instituto Adolfo Lutz, Interdiciplinary Procedures Center, Strategic Laboratory	Caio Vinicius Dias Lopes; Claudia Regina Gonçalves; Claudio Tavares Sacchi; Erica Valessa Ramos Gomes; Karoline Rodrigues Campos
EPI_ISL_882657	LACEN do Estado do Piaui, Dr. Costa Alvarenga	Instituto Adolfo Lutz, Interdiciplinary Procedures Center, Strategic Laboratory	Claudia Regina Gonçalves; Claudio Tavares Sacchi; Erica Valessa Ramos Gomes; Karoline Rodrigues Campos
EPI_ISL_1040824 to 1040851, EPI_ISL_1121327 to 1121330, EPI_ISL_1139052 to 1139068, EPI_ISL_1171621, EPI_ISL_1196282 to 1196284, EPI_ISL_1201883	LACEN do Mato Grosso do Sul	Instituto Adolfo Lutz, Interdiciplinary Procedures Center, Strategic Laboratory	Caio Vinicius Dias Lopes; Claudia Regina Gonçalves; Claudio Tavares Sacchi; Erica Valessa Ramos Gomes; Karoline Rodrigues Campos
EPI_ISL_1121316	LACEN do Rio Grande do Sul	Instituto Adolfo Lutz, Interdiciplinary Procedures Center, Strategic Laboratory	Caio Vinicius Dias Lopes; Claudia Regina Gonçalves; Claudio Tavares Sacchi; Erica Valessa Ramos Gomes; Karoline Rodrigues Campos
EPI_ISL_906071, EPI_ISL_940613 to 940618	LACEN-PI DR. Costa Alvarenga	Instituto Adolfo Lutz, Interdiciplinary Procedures Center, Strategic Laboratory	Claudia Regina Gonçalves; Claudio Tavares Sacchi; Erica Valessa Ramos Gomes; Karoline Rodrigues Campos
EPI_ISL_502779, EPI_ISL_502875	LACEN/PE	LABBE, Federal University of Pernambuco	ANTONIO CARLOS DE FREITAS; BRUNO SAMPAIO; HEIDI LACERDA ALVES DA CRUZ; MAIRA GALDINO DA ROCHA PITTA; MARCOS ANTONIO DE MORAIS JUNIOR; MARCOS DA SILVEIRA REGUEIRA NETO; MICHELLY CRISTINY PEREIRA; REGINALDO GONCALVES DE LIMA NETO; SERGIO DE SA LEITAO PAIVA JUNIOR; VALDIR DE QUEIROZ BALBINO.; WILSON JOSE DA SILVA JUNIOR; ZILDENE DE SOUSA SILVEIRA
EPI_ISL_1140481 to 1140484, EPI_ISL_1140540 to 1140551, EPI_ISL_1141656 to 1141658, EPI_ISL_1147413 to 1147414, EPI_ISL_1147423, EPI_ISL_1147425 to 1147426, EPI_ISL_1147432 to 1147435, EPI_ISL_1147489, EPI_ISL_1147493, EPI_ISL_1147498, EPI_ISL_1147506, EPI_ISL_1147510, EPI_ISL_1147517, EPI_ISL_1147565 to 1147568, EPI_ISL_1147570 to 1147577, EPI_ISL_1151899, EPI_ISL_1151902 to 1151903, EPI_ISL_1151906, EPI_ISL_1151908 to 1151909, EPI_ISL_1151915 to 1151917, EPI_ISL_1151919, EPI_ISL_1151921, EPI_ISL_1151930, EPI_ISL_1151935, EPI_ISL_1151937 to 1151938, EPI_ISL_1151942, EPI_ISL_1151945, EPI_ISL_1151954, EPI_ISL_1151957, EPI_ISL_1151964 to 1151965, EPI_ISL_1151967, EPI_ISL_1151976, EPI_ISL_1151978, EPI_ISL_1151980, EPI_ISL_1151985 to 1151988, EPI_ISL_1151991, EPI_ISL_1156371 to 1156373, EPI_ISL_1156375, EPI_ISL_1156384, EPI_ISL_1156389 to 1156390, EPI_ISL_1156394 to 1156396, EPI_ISL_1156401 to 1156402, EPI_ISL_1156404 to 1156411, EPI_ISL_1156413 to 1156414, EPI_ISL_1156418, EPI_ISL_1156421 to 1156422, EPI_ISL_1156424 to 1156425, EPI_ISL_1156431 to 1156432, EPI_ISL_1156434, EPI_ISL_1156437 to 1156438, EPI_ISL_1156440, EPI_ISL_1156443 to 1156444, EPI_ISL_1210772 to 1210776, EPI_ISL_1210778 to 1210782, EPI_ISL_1210784 to 1210785, EPI_ISL_1210787 to 1210789, EPI_ISL_1210793, EPI_ISL_1210796, EPI_ISL_1210799 to 1210801, EPI_ISL_1210803 to 1210814, EPI_ISL_1212026, EPI_ISL_1212030 to 1212031, EPI_ISL_1212037, EPI_ISL_1212043 to			

1212044, EPI_ISL_1212050, EPI_ISL_1212054 to 1212055, EPI_ISL_1212057, EPI_ISL_1212062, EPI_ISL_1217360 to 1217361, EPI_ISL_1217366, EPI_ISL_1217368, EPI_ISL_1217371, EPI_ISL_1217374, EPI_ISL_1217376, EPI_ISL_1217378, EPI_ISL_1217387, EPI_ISL_1217392, EPI_ISL_1217397, EPI_ISL_1217399, EPI_ISL_1217404, EPI_ISL_1217418, EPI_ISL_1282321, EPI_ISL_1282334, EPI_ISL_1282340, EPI_ISL_1282347, EPI_ISL_1282351, EPI_ISL_1282355, EPI_ISL_1282367 to 1282368, EPI_ISL_1282370, EPI_ISL_1282380, EPI_ISL_1285737, EPI_ISL_1285740, EPI_ISL_1285743, EPI_ISL_1285755 to 1285756, EPI_ISL_1285759, EPI_ISL_1285761 to 1285762, EPI_ISL_1285767, EPI_ISL_1285781, EPI_ISL_1285783, EPI_ISL_1285785, EPI_ISL_1285793			
see above	LADR MVZ Zweigpraxis Bernau	Robert Koch Institute	
EPI_ISL_1141702 to 1141710, EPI_ISL_1141712 to 1141750, EPI_ISL_1141752 to 1141753, EPI_ISL_1141755 to 1141758, EPI_ISL_1142580 to 1142596, EPI_ISL_1142598 to 1142625, EPI_ISL_1142627 to 1142628, EPI_ISL_1142630 to 1142657, EPI_ISL_1145494, EPI_ISL_1145496 to 1145497, EPI_ISL_1145499 to 1145503, EPI_ISL_1145506 to 1145507, EPI_ISL_1145509 to 1145521, EPI_ISL_1145523, EPI_ISL_1145525, EPI_ISL_1145527			
see above	LADR Zentrallabor DR. Kramer & Kollegen Geesthacht	Robert Koch Institute	
EPI_ISL_1213168, EPI_ISL_1213170, EPI_ISL_1213211, EPI_ISL_1213216, EPI_ISL_1213218, EPI_ISL_1213220, EPI_ISL_1213222, EPI_ISL_1213224, EPI_ISL_1213226 to 1213227, EPI_ISL_1213229 to 1213230, EPI_ISL_1213232, EPI_ISL_1213234, EPI_ISL_1213236 to 1213237, EPI_ISL_1213239, EPI_ISL_1213241 to 1213242, EPI_ISL_1213244 to 1213245, EPI_ISL_1213251 to 1213252, EPI_ISL_1213254, EPI_ISL_1213256, EPI_ISL_1213258 to 1213259, EPI_ISL_1213261, EPI_ISL_1213263, EPI_ISL_1213265, EPI_ISL_1213267, EPI_ISL_1213269, EPI_ISL_1213274, EPI_ISL_1213294, EPI_ISL_1213297 to 1213298, EPI_ISL_1213300, EPI_ISL_1213302 to 1213303, EPI_ISL_1213305, EPI_ISL_1213324, EPI_ISL_1213326, EPI_ISL_1213328, EPI_ISL_1213331, EPI_ISL_1213333, EPI_ISL_1213335, EPI_ISL_1213341 to 1213342, EPI_ISL_1213365, EPI_ISL_1213384, EPI_ISL_1213386, EPI_ISL_1213390, EPI_ISL_1213399, EPI_ISL_1213401 to 1213402, EPI_ISL_1213411, EPI_ISL_1213413, EPI_ISL_1213415			
see above	LAFEM/UESC	Bioinformatics Laboratory / LNCC	Alessandra P Lamarca; Alexandra L Gerber; Ana Paula Melo Mariano; Ana Paula de C Guimarães; Ana Tereza R Vasconcelos; Angela Maria Guimarães Santos; Bianca Mendes Maciel; Danielle Angst Secco; Eduardo Sérgio Soares Sousa; Eloiza Helena Campana; Francisco Paulo Freire Neto; George Rego Albuquerque; Kátia Castanho Scortecci; Lucymara Fassarella Agnez Lima; Luiz G P de Almeida; Luís Cristóvão Porto; Otavio J. Brustolini; Paulo Ricardo Nascimento; Ronaldo da Silva Francisco Jr; Sandra Rocha Gadelha; Selma Maria Bezerra Jeronimo; Vinícius Pietta Perez
EPI_ISL_1241983	LAM BIO 86 SITE DE CHAUMONT	CNR Virus des Infections Respiratoires - France SUD	Antonin Bal; Bruno Lina; Gregory Destras; Gwendolyne Burfin; Hadrien Regue; Laurence Josset; Martine Valette; Quentin Semanas
EPI_ISL_1209376	LAM BIOCENTRE MERCURE	CHU Lille - Laboratoire de Virologie	AIT YAHYA Emilie; ALIDJINOU Enagnon Kazali; BOCKET Laurence; CREPIN Michel; DEMAY Christophe; ENGELMANN Ilka; GEFFROY Sandrine; GUIGON Aurélie; LAZREK Mouna; NOBILLIAUX Florian; PREVOST Brigitte; THUILLIER Caroline; TINEZ Claire
EPI_ISL_1209380, EPI_ISL_1209394	LAM BIOPATH	CHU Lille - Laboratoire de Virologie	AIT YAHYA Emilie; ALIDJINOU Enagnon Kazali; BOCKET Laurence; CREPIN Michel; DEMAY Christophe; ENGELMANN Ilka; GEFFROY Sandrine; GUIGON Aurélie; LAZREK Mouna; NOBILLIAUX Florian; PREVOST Brigitte; THUILLIER Caroline; TINEZ Claire
EPI_ISL_1209378 to 1209379, EPI_ISL_1209406	LAM DIAGNOVIE RONCHIN	CHU Lille - Laboratoire de Virologie	AIT YAHYA Emilie; ALIDJINOU Enagnon Kazali; BOCKET Laurence; CREPIN Michel; DEMAY Christophe; ENGELMANN Ilka; GEFFROY Sandrine; GUIGON Aurélie; LAZREK Mouna; NOBILLIAUX Florian; PREVOST Brigitte; THUILLIER Caroline; TINEZ Claire
EPI_ISL_1265816	LAM LXBIO	CNR Virus des Infections Respiratoires - France SUD	Antonin Bal; Bruno Lina; Gregory Destras; Gwendolyne Burfin; Hadrien Regue; Laurence Josset; Martine Valette; Quentin Semanas
EPI_ISL_1265762 to 1265764	LAM PALAIS DES PYRENEES	CNR Virus des Infections Respiratoires - France SUD	Antonin Bal; Bruno Lina; Gregory Destras; Gwendolyne Burfin; Hadrien Regue; Laurence Josset; Martine Valette; Quentin Semanas
EPI_ISL_1313708	LAM SANTA DEVOTA	CNR Virus des Infections Respiratoires - France SUD	Antonin Bal; Bruno Lina; Gregory Destras; Gwendolyne Burfin; Hadrien Regue; Laurence Josset; Martine Valette; Quentin Semanas
EPI_ISL_861867 to 861888, EPI_ISL_861890 to 861910, EPI_ISL_861912 to 861913, EPI_ISL_861915, EPI_ISL_884249 to 884250, EPI_ISL_977500	LATE - Laboratório de Técnicas Especiais - Hospital Israelita Albert Einstein	LATE - Laboratório de Técnicas Especiais - Hospital Israelita Albert Einstein	Ana Paula Moreira Salles; Deyvid Amgarten; Fernanda de Mello Malta; João Renato Rebello Pinho; Pedro Henrique Sebe Rodrigues; Raquel Riyuzo
EPI_ISL_1313008	LBA	CNR Virus des Infections Respiratoires - France SUD	Antonin Bal; Bruno Lina; Gregory Destras; Gwendolyne Burfin; Hadrien Regue; Laurence Josset; Martine Valette; Quentin Semanas
EPI_ISL_1314018, EPI_ISL_1314043 to 1314051	LBM BIOLYSS	CNR Virus des Infections Respiratoires - France SUD	Antonin Bal; Bruno Lina; Gregory Destras; Gwendolyne Burfin; Hadrien Regue; Laurence Josset; Martine Valette; Quentin Semanas
EPI_ISL_1297489 to 1297490	LBM EXALAB	CNR Virus des Infections Respiratoires - France SUD	Antonin Bal; Bruno Lina; Gregory Destras; Gwendolyne Burfin; Hadrien Regue; Laurence Josset; Martine Valette; Quentin Semanas
EPI_ISL_1085817, EPI_ISL_1085850 to 1085851, EPI_ISL_1085933	LBM NEVERS	Department of Virology, Henri Mondor University Hospital, Assistance Publique Hôpitaux de Paris, Université Paris-Est Créteil, INSERM U955	Alexandre Soulier; Christophe Rodriguez; Elisabeth Trawinski; Guillaume Gricourt; Jean-Michel Pawlotsky; Melissa N'Debi; Slim Fourati; Vanessa Demontant
EPI_ISL_1297491	LBM SEALAB DARRASSE ET ASSOCIES	CNR Virus des Infections Respiratoires - France SUD	Antonin Bal; Bruno Lina; Gregory Destras; Gwendolyne Burfin; Hadrien Regue; Laurence Josset; Martine Valette; Quentin Semanas
EPI_ISL_1190801 to 1190804	LBM SEALAB PAU LOUP	CNR Virus des Infections Respiratoires - France SUD	Antonin Bal; Bruno Lina; Gregory Destras; Gwendolyne Burfin; Hadrien Règue; Laurence Josset; Martine Valette; Quentin Semanas
EPI_ISL_1313170	LBM TRONQUIERES	CNR Virus des Infections Respiratoires - France SUD	Antonin Bal; Bruno Lina; Gregory Destras; Gwendolyne Burfin; Hadrien Regue; Laurence Josset; Martine Valette; Quentin Semanas
EPI_ISL_1253552 to 1253554, EPI_ISL_1265827, EPI_ISL_1265842 to 1265843, EPI_ISL_1287741 to 1287747, EPI_ISL_1287749 to 1287751, EPI_ISL_1312994, EPI_ISL_1313000 to 1313001, EPI_ISL_1313003, EPI_ISL_1314034 to 1314035, EPI_ISL_1314038 to 1314041			
see above	LBM UNIBIO VALENTIN	CNR Virus des Infections Respiratoires - France SUD	Antonin Bal; Bruno Lina; Gregory Destras; Gwendolyne Burfin; Hadrien Regue; Laurence Josset; Martine Valette; Quentin Semanas
EPI_ISL_1213153, EPI_ISL_1213156, EPI_ISL_1213160 to 1213161, EPI_ISL_1213165 to 1213166, EPI_ISL_1213189 to 1213190, EPI_ISL_1213201 to 1213202, EPI_ISL_1213204, EPI_ISL_1213206 to 1213207, EPI_ISL_1213213, EPI_ISL_1213215, EPI_ISL_1213336, EPI_ISL_1213343 to 1213344, EPI_ISL_1213367, EPI_ISL_1213369 to 1213370, EPI_ISL_1213388, EPI_ISL_1213392, EPI_ISL_1213404, EPI_ISL_1213406, EPI_ISL_1213427, EPI_ISL_1213429, EPI_ISL_1213431, EPI_ISL_1213433 to 1213434, EPI_ISL_1213436, EPI_ISL_1213438 to 1213439, EPI_ISL_1213441, EPI_ISL_1213443 to 1213444, EPI_ISL_1213446, EPI_ISL_1213448 to 1213449, EPI_ISL_1213451, EPI_ISL_1213453 to 1213454, EPI_ISL_1213456, EPI_ISL_1213458 to 1213459, EPI_ISL_1213461, EPI_ISL_1213463			
see above	LBM/UFPB	Bioinformatics Laboratory / LNCC	Alessandra P Lamarca; Alexandra L Gerber; Ana Paula Melo Mariano; Ana Paula de C Guimarães; Ana Tereza R Vasconcelos; Angela Maria Guimarães Santos; Bianca Mendes Maciel; Danielle Angst Secco; Eduardo Sérgio Soares Sousa; Eloiza Helena Campana; Francisco Paulo Freire Neto; George Rego Albuquerque; Kátia Castanho Scortecci; Lucymara Fassarella Agnez Lima; Luiz G P de Almeida; Luís Cristóvão Porto; Otavio J. Brustolini; Paulo Ricardo Nascimento; Ronaldo da Silva Francisco Jr; Sandra Rocha Gadelha; Selma Maria Bezerra Jeronimo; Vinícius Pietta Perez
EPI_ISL_1091784 to 1091786	LDSP	Universidad Nacional de Colombia - Laboratorio Genómico One Health	Andres F. Cardona-Rios; Carlos Franco-Muñoz; Daniel O. Maldonado-Perez; Diego A. Álvarez-Díaz; Idabely Betancur Ortiz; Jorge E. Osorio; Juan P. Hernandez-Ortiz; Karl A Ciuoderis; Laura Silvana Perez; Marcela Mercado-Reyes; Maria Angélica Maya; Sandra Ines Cano
EPI_ISL_942927, EPI_ISL_961467 to 961469	LESP Nuevo Leon/Grupo de Diagnostico ARIES	Instituto de Diagnostico y Referencia Epidemiologicos (INDRE)	Abril Rodriguez-Maldonado; Claudia Wong-Arambula; Ernesto Ramirez-Gonzalez.; Fabiola Garces-Ayala; Gisela Barrera-Badillo; Irma Lopez-Martinez; Lucia Hernandez-Rivas; Natividad Cruz-Ortiz; Tatiana Nunez-Garcia
EPI_ISL_1180935	LHUB-ULB	LHUB-ULB	Charlotte Michel; Marie Hallin; Olivier Vandenberg
EPI_ISL_984297, EPI_ISL_1039248, EPI_ISL_1039250, EPI_ISL_1039711, EPI_ISL_1039832 to 1039834, EPI_ISL_1039948 to 1039949, EPI_ISL_1040472 to 1040473, EPI_ISL_1164846, EPI_ISL_1164848, EPI_ISL_1164852, EPI_ISL_1166890, EPI_ISL_1190842 to 1190843, EPI_ISL_1259242 to 1259249, EPI_ISL_1259251, EPI_ISL_1259253 to 1259258, EPI_ISL_1259260 to 1259262, EPI_ISL_1259267 to 1259269, EPI_ISL_1259271 to 1259277, EPI_ISL_1279945 to 1279946, EPI_ISL_1298181, EPI_ISL_1298186, EPI_ISL_1298191, EPI_ISL_1305783, EPI_ISL_1305786 to 1305788			
see above	LHUB-ULB	UAntwerp, Laboratory of Medical Microbiology	Basil Britto Xavier; Christine Lammens; Herman Goossens; Jasmine Coppens; Marie Le Mercier; Veerle Matheeussen
EPI_ISL_498226	LIC	LIC	LIC
EPI_ISL_1219139 to 1219158, EPI_ISL_1219269 to 1219279, EPI_ISL_1219393 to 1219411, EPI_ISL_1219559 to 1219578, EPI_ISL_1312498 to 1312509	LIC	Latvian Biomedical Research and Study Centre	Davids Fridmanis; Diana Dusacka; Guntars Zarins; Ivars Silamikelis; Janis Klovins; Janis Pjalkovskis; Jurijs Perevoscikovs; Kaspars Megnis; Laila Silamikele; Lauma Freimane; Laura Ansone; Liga Birzniece; Monta Ustinova; Nikita Zrelovs; Reinis Zeltmatis; Uga Dumpis; Una Krumina; Vita Rovite
EPI_ISL_1085288	LISIEUX Cerballiance	Department of Virology, Henri Mondor University Hospital, Assistance Publique Hôpitaux de Paris, Université Paris-Est Créteil, INSERM U955	Alexandre Soulier; Christophe Rodriguez; Elisabeth Trawinski; Guillaume Gricourt; Jean-Michel Pawlotsky; Melissa N'Debi; Slim Fourati; Vanessa Demontant
EPI_ISL_995548 to 995549, EPI_ISL_995551 to 995555, EPI_ISL_1138890	LKOLMC	Jessa	Cruys et al. on behalf of the Jessa_cmdLab; Jessa_cmdLab
EPI_ISL_469017, EPI_ISL_469049, EPI_ISL_469051 to 469054, EPI_ISL_482732 to 482740, EPI_ISL_728221 to 728224, EPI_ISL_728235 to 728237, EPI_ISL_728239			
see above	LNR National Reference Laboratory, Mohammed VI University of Health Sciences	Medical Biotechnology Laboratory, Rabat Medical and Pharmacy School, Mohammed The Vth University in Rabat	Adib Ghassan; Chakib NEJJARI; Houda BENRAHMA; Housna ARROUCHI; Idrissa DIAWARA; Idrissa Diawara; Imane SMYEJ; Jalil El ATAR; Jalil El Atar; Jalila RAHOUI; Lahcen BELYAMANI and Azeddine IBRAHIMI; Laila SBABOU; Laila SBABOU; Loubna ALLAM; M.W. CHEMAO-ELFIHRI; Meriem

			LAAMARTI; Mouna OUADGHIRI; Mouneem Essabbar; Nabila Soara; Rachid EL JAOUDI; Rachid MENTAG; Rokaia LAAMRTI; Rokia LAAMARTI; Saaid AMZAZI; Souad KARTTI; Tarek Aanniz
EPI_ISL_794652 to 794653	LSP DEL TOLIMA	Instituto Nacional de Salud - Dirección de Investigación en Salud Pública	Carlos Franco-Muñoz; Diego A. Álvarez-Díaz; Diego Andrés Prada; Gerardo Santamaría; Jonathan Reales; Julian Naizaque; Katherine Laiton-Donato; Magdalena Wiesner; Marcela Mercado-Reyes; Maria T. Herrera; Martha Lucia Ospina Martinez; Mauricio Pacheco-Montealegre; Paola Muñoz-Laiton; Sheryl Corchuelo
EPI_ISL_435034 to 435044, EPI_ISL_435550 to 435579, EPI_ISL_451203 to 451297, EPI_ISL_455980 to 456002, EPI_ISL_574295 to 574430, EPI_ISL_578698 to 578700, EPI_ISL_578704 to 579057, EPI_ISL_653345 to 653744, EPI_ISL_666609, EPI_ISL_666613, EPI_ISL_768325 to 768520, EPI_ISL_768525, EPI_ISL_778918 to 779128, EPI_ISL_889444 to 889541, EPI_ISL_889543 to 889801, EPI_ISL_890361 to 890364, EPI_ISL_890366 to 890370, EPI_ISL_1039521 to 1039541, EPI_ISL_1039543 to 1039689, EPI_ISL_1040072 to 1040118, EPI_ISL_1164856 to 1164926, EPI_ISL_1168348 to 1168353			
see above	LSUHS Emerging Viral Threat Laboratory	Microbial Genome Sequencing Center	; Abida Siddiqa; Adam Greer; Andrew D. Yurochko; Byeong-Jae Lee; Camille F. Abshire; Chan-ki Min; Christopher G. Kevil; Daniel J. Snyder; Edna Ondari; Jason M. Bodily; Jennifer L. Carroll; Jeremy P. Kamil; John A. Vanchiere; Katarzyna Zwolinska; Maarten Van Diest; Malgorzata Bienkowska-Haba; Martin J. Sapp; Md Maksudul Alam; Michelle M. Arnold; Mohammed N.A. Siddiquey; Monica Gestal-Cartele; Paul M. Weinberger; Rona S. Scott; Vaughn S. Cooper
EPI_ISL_528637 to 528638	LVM/UFRJ	Bioinformatics Laboratory / LNCC	Amilcar Tanuri; Ana Teresa R. Vasconcelos; Bruno B. Bezerra; Diana Marianni; Elena Cobos; Fabio Limonte; Gustavo D. P. Silva; Isadora A. Correa; Luciana B. Arruda; Luciana J. Costa; Lucio A. Caldas; Luiz Almeida; Luiza Higga; M. Romário M. de Souza; Marcelo Bozza; Orlando Ferreira; Sharton V. A. Coelho; Terezinha M. Castineiras; Wanderley de Souza
EPI_ISL_528539	LVM/UFRJ	LNCC	Amilcar Tanuri; Ana Teresa R. Vasconcelos; Bruno B. Bezerra; Diana Marianni; Elena Cobos; Fabio Limonte; Gustavo M. Romário M. de Souza; Isadora A. Correa; Luciana B. Arruda; Luciana J. Costa.; Lucio A. Caldas; Luiz Almeida; Luiza Higga; Marcelo Bozza; Orlando Ferreira; Sharton V. A. Coelho; Terezinha M. Castineiras; Wanderley de Souza
EPI_ISL_1313015	LX BIO	CNR Virus des Infections Respiratoires - France SUD	Antonin Bal; Bruno Lina; Gregory Destras; Gwendolyne Burfin; Hadrien Regue; Laurence Josset; Martine Valette; Quentin Semanas
EPI_ISL_443307	La Villa Papyri	National Reference Center for Viruses of Respiratory Infections, Institut Pasteur, Paris	Angela Brisebarre; Etienne Simon-Lorière; Flora Donati; Marion Barbet; Maud Vanpeene; Mélanie Albert; Méline Bizard; Sylvie Behillil; Sylvie van der Werf; Vincent Enouf
EPI_ISL_1060792, EPI_ISL_1060796	Lab Corp of America-Raritan	New Jersey Public Health Environmental Laboratories (NJ_PHEL)	Byeong Jeong; Dana Woell; Lindsey Bodnar; Shiv Verma
EPI_ISL_755644 to 755645, EPI_ISL_755652, EPI_ISL_833162, EPI_ISL_861669	Lab LOC - Itapecerica da Serra	Instituto Adolfo Lutz, Interdiciplinary Procedures Center, Strategic Laboratory	Claudia Regina Gonçalves; Claudio Tavares Sacchi; Erica Valessa Ramos Gomes; Karoline Rodrigues Campos
EPI_ISL_977479, EPI_ISL_985178, EPI_ISL_1039698, EPI_ISL_1039704, EPI_ISL_1139075, EPI_ISL_1139077	Lab Loc - Itapecerica da Serra	Instituto Adolfo Lutz, Interdiciplinary Procedures Center, Strategic Laboratory	Caio Vinicius Dias Lopes; Claudia Regina Gonçalves; Claudio Tavares Sacchi; Erica Valessa Ramos Gomes; Karoline Rodrigues Campos
EPI_ISL_501614 to 501631	Lab Microbiology, Pathology Department, William Harvey Hospital	Wellcome Sanger Institute for the COVID-19 Genomics UK (COG-UK) consortium	Cordelia Langford; David K. Jackson; Dominic Kwiatkowski; Ewan Harrison; Felicity Ryan and Alex Alderton; Hannah Lowe; Ian Johnston; John Sillitoe on behalf of the Wellcome Sanger Institute COVID-19 Surveillance Team (http://www.sanger.ac.uk/covid-team); Roberto Amato; Samuel Moses; Sonia Goncalves
EPI_ISL_846541 to 846580, EPI_ISL_977491, EPI_ISL_977493, EPI_ISL_982237 to 982273, EPI_ISL_982275 to 982280, EPI_ISL_982283 to 982296, EPI_ISL_1073827 to 1073833, EPI_ISL_1073835 to 1073868, EPI_ISL_1073870 to 1073914, EPI_ISL_1109737 to 1109751, EPI_ISL_1109753 to 1109758, EPI_ISL_1109760 to 1109763, EPI_ISL_1109765 to 1109778, EPI_ISL_1109780 to 1109782, EPI_ISL_1109784, EPI_ISL_1109786 to 1109792, EPI_ISL_1109794 to 1109795, EPI_ISL_1109798 to 1109818, EPI_ISL_1109822, EPI_ISL_1109825 to 1109861, EPI_ISL_1109863 to 1109879, EPI_ISL_1109881 to 1109887, EPI_ISL_1109889 to 1109902, EPI_ISL_1117593 to 1117596, EPI_ISL_1118818 to 1118848, EPI_ISL_1273105 to 1273213			
see above	Lab voor klinische biologie	Lab voor klinische biologie	Bruno Verhasselt; Hannelore Hamerlinck; Marija Janevska
EPI_ISL_419259, EPI_ISL_419264 to 419266, EPI_ISL_425048, EPI_ISL_425050 to 425064, EPI_ISL_434347 to 434365, EPI_ISL_451164 to 451182, EPI_ISL_468727 to 468746, EPI_ISL_475060 to 475082, EPI_ISL_481221 to 481233, EPI_ISL_634876 to 634895, EPI_ISL_717573 to 717633, EPI_ISL_717980 to 717983, EPI_ISL_717985 to 718026, EPI_ISL_745202 to 745221, EPI_ISL_794825, EPI_ISL_855902, EPI_ISL_855904, EPI_ISL_855906, EPI_ISL_855908, EPI_ISL_855910, EPI_ISL_855912, EPI_ISL_855914, EPI_ISL_855916 to 855917, EPI_ISL_855919, EPI_ISL_855921, EPI_ISL_855923, EPI_ISL_855925, EPI_ISL_855927 to 855928, EPI_ISL_855930, EPI_ISL_855932, EPI_ISL_855934, EPI_ISL_855936, EPI_ISL_855938, EPI_ISL_855940, EPI_ISL_855942, EPI_ISL_855944 to 855945, EPI_ISL_855947, EPI_ISL_855949, EPI_ISL_855951, EPI_ISL_855953, EPI_ISL_855955, EPI_ISL_855957, EPI_ISL_855959 to 855960, EPI_ISL_855962, EPI_ISL_855964, EPI_ISL_855966, EPI_ISL_855968			
see above	Lab voor klinische biologie	Onderzoeksgroep Virologie	Basiel Cole; Bruno Verhasselt; Hans Nauwynck; Jozefien De Clercq; Laurens Lambrechts; Linos Vandekerckhove; Marthe Pauwels; Nick Vereecke; Sebastiaan Theuns
EPI_ISL_775596	Lab. Biologi Molekular FKIK Unwar, Bali	National Institute of Health Research and Development	AA; Dewi; HA; HD; Ikawati; KD; KNA; N; Nugraha; Pangesti; Pawestri; Puspa; Puspandari; SR; Setiawaty; Soekarso; Subangkit; T; V
EPI_ISL_1239548, EPI_ISL_1239553, EPI_ISL_1239587 to 1239590, EPI_ISL_1239665 to 1239671, EPI_ISL_1239698, EPI_ISL_1239707 to 1239708, EPI_ISL_1239730 to 1239735, EPI_ISL_1273837			
see above	Lab. Biologii molekularnej USK	1. National Institute of Public Health - National Institute of Hygiene; 2. Eurofins Genomics Europe Sequencing GmbH	ECDC COVID-19 WGS support team; Eurofins Genomics Europe Sequencing Team; Gierczyki Rafa; Sadkowska-Todys Magorzata; Wokowicz Tomasz; Zacharczuk Katarzyna
EPI_ISL_1307800 to 1307841	Lab. Microbiologia e Virologia - Cotugno - A.O. dei Colli	TIGEM	Antonio Grimaldi Patrizia Annunziata Francesco Panariello Biancamaria Pierri Claudia Tiberio Valentina Bouche Chiara Colantuono Maria Concetta Cuomo Denise Di Concilio Lucio Di Filippo Anna Manfredi Marcello Salvi Antonio Limone Luigi Atripaldi Pellegrino Cerino Andrea Ballabio Davide Cacchiarelli
EPI_ISL_1307942 to 1308009	Lab. Microbiologia e Virologia Cotugno A.O. dei Colli	TIGEM	Antonio Grimaldi Patrizia Annunziata Francesco Panariello Biancamaria Pierri Claudia Tiberio Valentina Bouche Chiara Colantuono Maria Concetta Cuomo Denise Di Concilio Lucio Di Filippo Anna Manfredi Marcello Salvi Antonio Limone Luigi Atripaldi Pellegrino Cerino Andrea Ballabio Davide Cacchiarelli
EPI_ISL_847825, EPI_ISL_852619, EPI_ISL_852622, EPI_ISL_852628 to 852632, EPI_ISL_852813, EPI_ISL_852819, EPI_ISL_852821, EPI_ISL_852823, EPI_ISL_852825, EPI_ISL_852828, EPI_ISL_855556, EPI_ISL_855573, EPI_ISL_855610 to 855614, EPI_ISL_856679, EPI_ISL_856682, EPI_ISL_856694, EPI_ISL_856698, EPI_ISL_856700, EPI_ISL_860095, EPI_ISL_860203 to 860205, EPI_ISL_860257, EPI_ISL_860290, EPI_ISL_860297, EPI_ISL_860794			
see above	Lab. Microbiologia e Virologia, Cotugno, A.O. dei Colli	Lab. Microbiologia e Virologia, Cotugno, A.O. dei Colli	Anna Perfetti; Claudia Tiberio; Luigi Atripaldi
EPI_ISL_911967	LabCorp	Wyoming Public Health Laboratory	Ashley Norberg; Bailey Bowcutt; Channing Weber; Lynette Gumbleton; Noah Hull; Taylor Fearing; and Wanda Manley
EPI_ISL_1114257, EPI_ISL_1114375	LabCorp Seattle	Seattle Flu Study	Amanda Adler; Barry R. Lutz; Benjamin Pelle; Brian Hiatt; Caitlin R. Wolf; Chris D. Frazar; Deborah A. Nickerson; Elisabeth Brandstetter; Erica Ryke; Geoff Melly; Helen Y. Chu; Janet A. Englund; Jay Shendure; Jover Lee; Kairsten Fay; Kirsten Lacombe; Lea M. Starita; Mark J. Rieder; Matthew Richardson; Matthew Thompson; Melissa Truong; Michael Boeckh; Michael Famulare; Misja Ilcisin; Peter D. Han; Philip Dykema; Romesh Gautom; Scott Lindquist; Thomas R. Sibley; Trevor Bedford
EPI_ISL_1141223, EPI_ISL_1141239, EPI_ISL_1141243, EPI_ISL_1141251, EPI_ISL_1141263, EPI_ISL_1141277, EPI_ISL_1141284, EPI_ISL_1141293, EPI_ISL_1141308, EPI_ISL_1141310, EPI_ISL_1141313, EPI_ISL_1141315, EPI_ISL_1141318, EPI_ISL_1141326, EPI_ISL_1141335, EPI_ISL_1141340, EPI_ISL_1141342 to 1141343, EPI_ISL_1141354, EPI_ISL_1141356, EPI_ISL_1141361, EPI_ISL_1141368, EPI_ISL_1141373, EPI_ISL_1141379, EPI_ISL_1141383, EPI_ISL_1141389, EPI_ISL_1141392 to 1141393, EPI_ISL_1141398, EPI_ISL_1141404, EPI_ISL_1141426, EPI_ISL_1141430, EPI_ISL_1141440 to 1141441, EPI_ISL_1141447, EPI_ISL_1141454, EPI_ISL_1141458 to 1141461, EPI_ISL_1141464, EPI_ISL_1141477 to 1141478, EPI_ISL_1141482 to 1141483, EPI_ISL_1141486, EPI_ISL_1141488 to 1141489, EPI_ISL_1141492, EPI_ISL_1141495, EPI_ISL_1141504, EPI_ISL_1141507 to 1141508, EPI_ISL_1141523 to 1141524, EPI_ISL_1142274, EPI_ISL_1142277, EPI_ISL_1142285 to 1142287, EPI_ISL_1142300, EPI_ISL_1142305, EPI_ISL_1142310 to 1142311, EPI_ISL_1142327 to 1142328, EPI_ISL_1142341 to 1142343, EPI_ISL_1142346, EPI_ISL_1142362 to 1142363, EPI_ISL_1142374, EPI_ISL_1142391, EPI_ISL_1142395, EPI_ISL_1142401, EPI_ISL_1142412, EPI_ISL_1142419, EPI_ISL_1142426 to 1142427, EPI_ISL_1142432, EPI_ISL_1142434, EPI_ISL_1142439, EPI_ISL_1142443 to 1142444, EPI_ISL_1142449, EPI_ISL_1142455, EPI_ISL_1142467, EPI_ISL_1142470, EPI_ISL_1142475, EPI_ISL_1142479, EPI_ISL_1142482 to 1142483, EPI_ISL_1142493, EPI_ISL_1142497, EPI_ISL_1142502, EPI_ISL_1142510, EPI_ISL_1142730 to 1142731, EPI_ISL_1142733, EPI_ISL_1142736, EPI_ISL_1142740, EPI_ISL_1142748, EPI_ISL_1142760, EPI_ISL_1142780, EPI_ISL_1142785, EPI_ISL_1142807, EPI_ISL_1142811, EPI_ISL_1142818 to 1142819, EPI_ISL_1142829, EPI_ISL_1142847, EPI_ISL_1142856, EPI_ISL_1142875, EPI_ISL_1142889, EPI_ISL_1142910, EPI_ISL_1142915, EPI_ISL_1142920 to 1142921, EPI_ISL_1142924 to 1142925, EPI_ISL_1142930, EPI_ISL_1142939, EPI_ISL_1142942, EPI_ISL_1142944, EPI_ISL_1142946, EPI_ISL_1142950, EPI_ISL_1142953, EPI_ISL_1142974, EPI_ISL_1142976, EPI_ISL_1142980, EPI_ISL_1142987, EPI_ISL_1142992 to 1142993, EPI_ISL_1142996, EPI_ISL_1143002, EPI_ISL_1143024 to 1143026, EPI_ISL_1143038, EPI_ISL_1145911, EPI_ISL_1145983, EPI_ISL_1145987, EPI_ISL_1145994, EPI_ISL_1145998, EPI_ISL_1146020, EPI_ISL_1146047, EPI_ISL_1146049, EPI_ISL_1146102, EPI_ISL_1146142, EPI_ISL_1146146, EPI_ISL_1146148, EPI_ISL_1146161, EPI_ISL_1146163, EPI_ISL_1146165, EPI_ISL_1146168 to 1146169, EPI_ISL_1148376, EPI_ISL_1150020, EPI_ISL_1150025, EPI_ISL_1150039, EPI_ISL_1150046, EPI_ISL_1150049, EPI_ISL_1150052, EPI_ISL_1150056, EPI_ISL_1150063, EPI_ISL_1150067, EPI_ISL_1150070, EPI_ISL_1150079, EPI_ISL_1150091, EPI_ISL_1150095, EPI_ISL_1150099, EPI_ISL_1150102, EPI_ISL_1150107, EPI_ISL_1150109, EPI_ISL_1150112, EPI_ISL_1150117, EPI_ISL_1150125, EPI_ISL_1150131, EPI_ISL_1150136, EPI_ISL_1150139, EPI_ISL_1150143, EPI_ISL_1150148, EPI_ISL_1150150 to 1150151, EPI_ISL_1150169, EPI_ISL_1150173, EPI_ISL_1150179, EPI_ISL_1150194 to 1150196, EPI_ISL_1150199, EPI_ISL_1150206, EPI_ISL_1150210, EPI_ISL_1150218, EPI_ISL_1150225, EPI_ISL_1150227, EPI_ISL_1150234, EPI_ISL_1150238, EPI_ISL_1150240, EPI_ISL_1150243, EPI_ISL_1150246 to 1150247, EPI_ISL_1150252, EPI_ISL_1150255, EPI_ISL_1150259 to 1150260, EPI_ISL_1150271, EPI_ISL_1150273, EPI_ISL_1150281, EPI_ISL_1150291, EPI_ISL_1150302, EPI_ISL_1150308 to 1150309, EPI_ISL_1150321, EPI_ISL_1150330, EPI_ISL_1150337, EPI_ISL_1150343, EPI_ISL_1150346, EPI_ISL_1150354, EPI_ISL_1150358, EPI_ISL_1150363, EPI_ISL_1150376 to 1150377, EPI_ISL_1154441, EPI_ISL_1154447, EPI_ISL_1154449, EPI_ISL_1154452, EPI_ISL_1154454, EPI_ISL_1154460, EPI_ISL_1154462 to 1154463, EPI_ISL_1154471, EPI_ISL_1154474, EPI_ISL_1154492, EPI_ISL_1154497, EPI_ISL_1154499, EPI_ISL_1154506 to 1154507, EPI_ISL_1154511, EPI_ISL_1154515, EPI_ISL_1154521, EPI_ISL_1154529, EPI_ISL_1154539 to 1154542, EPI_ISL_1154550, EPI_ISL_1154560, EPI_ISL_1154564, EPI_ISL_1154573, EPI_ISL_1154575 to 1154576, EPI_ISL_1154578, EPI_ISL_1154581, EPI_ISL_1154583 to 1154586, EPI_ISL_1154588, EPI_ISL_1154599, EPI_ISL_1154601, EPI_ISL_1154603, EPI_ISL_1154606, EPI_ISL_1154613, EPI_ISL_1154615, EPI_ISL_1154617, EPI_ISL_1154619, EPI_ISL_1154621 to 1154622, EPI_ISL_1154630 to 1154631, EPI_ISL_1154636 to 1154637, EPI_ISL_1154643 to 1154645, EPI_ISL_1154650 to 1154651, EPI_ISL_1154656, EPI_ISL_1154661, EPI_ISL_1154666, EPI_ISL_1154670, EPI_ISL_1154672, EPI_ISL_1154678, EPI_ISL_1154685, EPI_ISL_1154691, EPI_ISL_1154694, EPI_ISL_1154696 to 1154697, EPI_ISL_1154700, EPI_ISL_1154704, EPI_ISL_1154707, EPI_ISL_1154723, EPI_ISL_1154726 to 1154727, EPI_ISL_1154732 to 1154734, EPI_ISL_1154738, EPI_ISL_1154740, EPI_ISL_1154747, EPI_ISL_1154751, EPI_ISL_1154755 to 1154756, EPI_ISL_1154762 to 1154765, EPI_ISL_1154767, EPI_ISL_1154770, EPI_ISL_1154885, EPI_ISL_1154913, EPI_ISL_1156483,			

EPI_ISL_1156514, EPI_ISL_1156521, EPI_ISL_1156584, EPI_ISL_1156589, EPI_ISL_1156593, EPI_ISL_1156597, EPI_ISL_1156612, EPI_ISL_1156616, EPI_ISL_1156623, EPI_ISL_1156638, EPI_ISL_1156644, EPI_ISL_1156666, EPI_ISL_1156670, EPI_ISL_1156675, EPI_ISL_1156688, EPI_ISL_1156734, EPI_ISL_1156760, EPI_ISL_1156762, EPI_ISL_1212500 to 1212501, EPI_ISL_1212503, EPI_ISL_1212506, EPI_ISL_1212510, EPI_ISL_1212515, EPI_ISL_1212520, EPI_ISL_1212524, EPI_ISL_1212529, EPI_ISL_1212532, EPI_ISL_1212534, EPI_ISL_1212538, EPI_ISL_1212540, EPI_ISL_1212542, EPI_ISL_1212545, EPI_ISL_1212547, EPI_ISL_1212549, EPI_ISL_1212551, EPI_ISL_1212556, EPI_ISL_1212562, EPI_ISL_1212565, EPI_ISL_1212573, EPI_ISL_1212575 to 1212576, EPI_ISL_1212582, EPI_ISL_1212584, EPI_ISL_1212588, EPI_ISL_1212594, EPI_ISL_1212596 to 1212598, EPI_ISL_1212601, EPI_ISL_1212607, EPI_ISL_1212611 to 1212613, EPI_ISL_1212616, EPI_ISL_1212642, EPI_ISL_1212648, EPI_ISL_1212651, EPI_ISL_1212653, EPI_ISL_1212655, EPI_ISL_1212657, EPI_ISL_1212662, EPI_ISL_1212667, EPI_ISL_1212672, EPI_ISL_1212678, EPI_ISL_1212680, EPI_ISL_1212688, EPI_ISL_1212697, EPI_ISL_1212703, EPI_ISL_1212705, EPI_ISL_1212711, EPI_ISL_1212714, EPI_ISL_1212726, EPI_ISL_1212731, EPI_ISL_1212733 to 1212734, EPI_ISL_1212741, EPI_ISL_1212744, EPI_ISL_1212751, EPI_ISL_1212754, EPI_ISL_1212757 to 1212761, EPI_ISL_1212766 to 1212767, EPI_ISL_1212773, EPI_ISL_1212775, EPI_ISL_1212778 to 1212779, EPI_ISL_1212783, EPI_ISL_1212788, EPI_ISL_1212793, EPI_ISL_1212801, EPI_ISL_1212804, EPI_ISL_1212810, EPI_ISL_1212813, EPI_ISL_1212818, EPI_ISL_1212822, EPI_ISL_1212825, EPI_ISL_1212827, EPI_ISL_1212830 to 1212833, EPI_ISL_1212836, EPI_ISL_1212844, EPI_ISL_1212850 to 1212852, EPI_ISL_1212854, EPI_ISL_1212857 to 1212858, EPI_ISL_1214350, EPI_ISL_1214355, EPI_ISL_1214374, EPI_ISL_1214379, EPI_ISL_1214402, EPI_ISL_1214432, EPI_ISL_1214434, EPI_ISL_1214448, EPI_ISL_1214459, EPI_ISL_1214468, EPI_ISL_1214477, EPI_ISL_1214486, EPI_ISL_1214495, EPI_ISL_1214500, EPI_ISL_1214526, EPI_ISL_1214537, EPI_ISL_1214547, EPI_ISL_1214561, EPI_ISL_1214570, EPI_ISL_1214581, EPI_ISL_1214590, EPI_ISL_1214599, EPI_ISL_1214612, EPI_ISL_1214621, EPI_ISL_1214633, EPI_ISL_1214645, EPI_ISL_1214654, EPI_ISL_1214664, EPI_ISL_1214684, EPI_ISL_1214686, EPI_ISL_1214695, EPI_ISL_1214697, EPI_ISL_1214704, EPI_ISL_1214721, EPI_ISL_1214723, EPI_ISL_1214752, EPI_ISL_1281992 to 1281994, EPI_ISL_1281999, EPI_ISL_1282006, EPI_ISL_1282010, EPI_ISL_1282012 to 1282013, EPI_ISL_1282016, EPI_ISL_1282022, EPI_ISL_1282026, EPI_ISL_1282029, EPI_ISL_1282033, EPI_ISL_1282038 to 1282041, EPI_ISL_1282048 to 1282049, EPI_ISL_1282053 to 1282054, EPI_ISL_1282056 to 1282057, EPI_ISL_1282061, EPI_ISL_1282063, EPI_ISL_1282065, EPI_ISL_1282070, EPI_ISL_1282075, EPI_ISL_1282079 to 1282080, EPI_ISL_1282087, EPI_ISL_1282097, EPI_ISL_1282100, EPI_ISL_1282106 to 1282107, EPI_ISL_1282109, EPI_ISL_1282111, EPI_ISL_1282120 to 1282121, EPI_ISL_1282126 to 1282127, EPI_ISL_1282130 to 1282131, EPI_ISL_1282142, EPI_ISL_1282144 to 1282145, EPI_ISL_1282149, EPI_ISL_1282151, EPI_ISL_1282153 to 1282154, EPI_ISL_1282156 to 1282157, EPI_ISL_1282161, EPI_ISL_1282165, EPI_ISL_1282169, EPI_ISL_1282172 to 1282175, EPI_ISL_1282178, EPI_ISL_1282185 to 1282186, EPI_ISL_1282192, EPI_ISL_1282198 to 1282199, EPI_ISL_1282202, EPI_ISL_1282207, EPI_ISL_1282212, EPI_ISL_1282214, EPI_ISL_1282216, EPI_ISL_1282219, EPI_ISL_1282227, EPI_ISL_1282230, EPI_ISL_1282233, EPI_ISL_1282235, EPI_ISL_1282241 to 1282243, EPI_ISL_1282255 to 1282256, EPI_ISL_1282262, EPI_ISL_1282264, EPI_ISL_1282269, EPI_ISL_1282271 to 1282272, EPI_ISL_1282276 to 1282277, EPI_ISL_1282281 to 1282283, EPI_ISL_1282288, EPI_ISL_1282290 to 1282291, EPI_ISL_1282293 to 1282294, EPI_ISL_1282302 to 1282303, EPI_ISL_1282306 to 1282307, EPI_ISL_1285432, EPI_ISL_1285442, EPI_ISL_1285447, EPI_ISL_1285450 to 1285451, EPI_ISL_1285453 to 1285455, EPI_ISL_1285460, EPI_ISL_1285462 to 1285463, EPI_ISL_1285465, EPI_ISL_1285467, EPI_ISL_1285469 to 1285472, EPI_ISL_1285476, EPI_ISL_1285478, EPI_ISL_1285480 to 1285481, EPI_ISL_1285483, EPI_ISL_1285486, EPI_ISL_1285492, EPI_ISL_1285495, EPI_ISL_1285498, EPI_ISL_1285500, EPI_ISL_1285502, EPI_ISL_1285504, EPI_ISL_1285510 to 1285511, EPI_ISL_1285513, EPI_ISL_1285518, EPI_ISL_1285520, EPI_ISL_1285523 to 1285524, EPI_ISL_1285528 to 1285529, EPI_ISL_1285533, EPI_ISL_1285535, EPI_ISL_1285537, EPI_ISL_1285543, EPI_ISL_1285545, EPI_ISL_1285549, EPI_ISL_1285553, EPI_ISL_1285555, EPI_ISL_1285559, EPI_ISL_1285563, EPI_ISL_1285572 to 1285573, EPI_ISL_1285575, EPI_ISL_1285577, EPI_ISL_1285580 to 1285582, EPI_ISL_1285585, EPI_ISL_1285591, EPI_ISL_1285593, EPI_ISL_1285595, EPI_ISL_1285597, EPI_ISL_1285601, EPI_ISL_1285603, EPI_ISL_1285606, EPI_ISL_1285608 to 1285609, EPI_ISL_1285613 to 1285614, EPI_ISL_1285616 to 1285618, EPI_ISL_1285625, EPI_ISL_1285628, EPI_ISL_1285631, EPI_ISL_1285634, EPI_ISL_1285636 to 1285637, EPI_ISL_1285640 to 1285641, EPI_ISL_1285644, EPI_ISL_1285651 to 1285652, EPI_ISL_1285654 to 1285659, EPI_ISL_1285662 to 1285663, EPI_ISL_1285667, EPI_ISL_1285672, EPI_ISL_1285679, EPI_ISL_1285682, EPI_ISL_1285685, EPI_ISL_1285694, EPI_ISL_1285698 to 1285702, EPI_ISL_1285705 to 1285706, EPI_ISL_1285709 to 1285710, EPI_ISL_1285716

see above | LabKom - Labor Augsburg MVZ GmbH | Robert Koch Institute

EPI_ISL_1142332, EPI_ISL_1142347, EPI_ISL_1142352, EPI_ISL_1142490, EPI_ISL_1143084, EPI_ISL_1143105, EPI_ISL_1143131, EPI_ISL_1143176, EPI_ISL_1143300, EPI_ISL_1145942, EPI_ISL_1154478, EPI_ISL_1154509, EPI_ISL_1154535, EPI_ISL_1154563, EPI_ISL_1154596, EPI_ISL_1154702, EPI_ISL_1154746, EPI_ISL_1154768, EPI_ISL_1285435, EPI_ISL_1285487, EPI_ISL_1285550, EPI_ISL_1285554, EPI_ISL_1285598

see above | LabKom - Labor Hamburg-Lübeck | Robert Koch Institute

EPI_ISL_1142281, EPI_ISL_1142312, EPI_ISL_1142314, EPI_ISL_1142378, EPI_ISL_1142380, EPI_ISL_1142425, EPI_ISL_1142429, EPI_ISL_1142450, EPI_ISL_1142477 to 1142478, EPI_ISL_1142516, EPI_ISL_1142518, EPI_ISL_1142750, EPI_ISL_1142758, EPI_ISL_1142772, EPI_ISL_1142796, EPI_ISL_1142804, EPI_ISL_1142808, EPI_ISL_1142810, EPI_ISL_1142848, EPI_ISL_1142886, EPI_ISL_1142899, EPI_ISL_1142907, EPI_ISL_1142931, EPI_ISL_1142948, EPI_ISL_1142973, EPI_ISL_1142981, EPI_ISL_1143018, EPI_ISL_1143022, EPI_ISL_1143028 to 1143029, EPI_ISL_1143032, EPI_ISL_1143036, EPI_ISL_1145912, EPI_ISL_1145914, EPI_ISL_1145918 to 1145919, EPI_ISL_1145925, EPI_ISL_1145949, EPI_ISL_1145968, EPI_ISL_1145982, EPI_ISL_1145986, EPI_ISL_1145988, EPI_ISL_1145995, EPI_ISL_1146014, EPI_ISL_1146026, EPI_ISL_1146037, EPI_ISL_1146044, EPI_ISL_1146048, EPI_ISL_1146058, EPI_ISL_1146066, EPI_ISL_1146072, EPI_ISL_1146080, EPI_ISL_1146085, EPI_ISL_1146089, EPI_ISL_1146096, EPI_ISL_1146109, EPI_ISL_1146118, EPI_ISL_1146123, EPI_ISL_1146125, EPI_ISL_1146154, EPI_ISL_1154439 to 1154440, EPI_ISL_1154446, EPI_ISL_1154450, EPI_ISL_1154453, EPI_ISL_1154455, EPI_ISL_1154464 to 1154465, EPI_ISL_1154468 to 1154469, EPI_ISL_1154481 to 1154482, EPI_ISL_1154484, EPI_ISL_1154491, EPI_ISL_1154494 to 1154495, EPI_ISL_1154500, EPI_ISL_1154503, EPI_ISL_1154510, EPI_ISL_1154512, EPI_ISL_1154523, EPI_ISL_1154530, EPI_ISL_1154536, EPI_ISL_1154543 to 1154544, EPI_ISL_1154546, EPI_ISL_1154549, EPI_ISL_1154555, EPI_ISL_1154559, EPI_ISL_1154561, EPI_ISL_1154565, EPI_ISL_1154567, EPI_ISL_1154580, EPI_ISL_1154589, EPI_ISL_1154593, EPI_ISL_1154600, EPI_ISL_1154605, EPI_ISL_1154623 to 1154624, EPI_ISL_1154626, EPI_ISL_1154629, EPI_ISL_1154633, EPI_ISL_1154640, EPI_ISL_1154646, EPI_ISL_1154655, EPI_ISL_1154660, EPI_ISL_1154665, EPI_ISL_1154667, EPI_ISL_1154669, EPI_ISL_1154671, EPI_ISL_1154676 to 1154677, EPI_ISL_1154680, EPI_ISL_1154683 to 1154684, EPI_ISL_1154687, EPI_ISL_1154695, EPI_ISL_1154706, EPI_ISL_1154709, EPI_ISL_1154712, EPI_ISL_1154715, EPI_ISL_1154718, EPI_ISL_1154722, EPI_ISL_1154724 to 1154725, EPI_ISL_1154743, EPI_ISL_1154754, EPI_ISL_1154761, EPI_ISL_1154773, EPI_ISL_1154789, EPI_ISL_1154794, EPI_ISL_1154799 to 1154800, EPI_ISL_1154816, EPI_ISL_1154830, EPI_ISL_1154834 to 1154835, EPI_ISL_1154850, EPI_ISL_1154895 to 1154896, EPI_ISL_1154907, EPI_ISL_1154924, EPI_ISL_1154949, EPI_ISL_1154963, EPI_ISL_1154971, EPI_ISL_1154999, EPI_ISL_1155007, EPI_ISL_1155029, EPI_ISL_1155032, EPI_ISL_1155061, EPI_ISL_1155064, EPI_ISL_1155092, EPI_ISL_1155096, EPI_ISL_1155101, EPI_ISL_1155120, EPI_ISL_1211088, EPI_ISL_1211092, EPI_ISL_1211098, EPI_ISL_1211101, EPI_ISL_1211114, EPI_ISL_1211116, EPI_ISL_1211120, EPI_ISL_1211124, EPI_ISL_1211132, EPI_ISL_1211134, EPI_ISL_1211141, EPI_ISL_1211147, EPI_ISL_1211154, EPI_ISL_1211161, EPI_ISL_1211193, EPI_ISL_1211198, EPI_ISL_1211204, EPI_ISL_1211208, EPI_ISL_1211217, EPI_ISL_1211233, EPI_ISL_1211244, EPI_ISL_1211247, EPI_ISL_1211257, EPI_ISL_1211259 to 1211261, EPI_ISL_1211274, EPI_ISL_1211287, EPI_ISL_1211297, EPI_ISL_1211309, EPI_ISL_1211318, EPI_ISL_1211321, EPI_ISL_1211354, EPI_ISL_1211360, EPI_ISL_1211367, EPI_ISL_1211372, EPI_ISL_1211391, EPI_ISL_1211393 to 1211394, EPI_ISL_1211420

see above | LabKom - Labor Hannover MVZ GmbH | Robert Koch Institute

EPI_ISL_1142284, EPI_ISL_1142293, EPI_ISL_1142315, EPI_ISL_1142317, EPI_ISL_1142321, EPI_ISL_1142326, EPI_ISL_1142340, EPI_ISL_1142356, EPI_ISL_1142361, EPI_ISL_1142367, EPI_ISL_1142376, EPI_ISL_1142385, EPI_ISL_1142389, EPI_ISL_1142402, EPI_ISL_1142404, EPI_ISL_1142408, EPI_ISL_1142415, EPI_ISL_1142424, EPI_ISL_1142431, EPI_ISL_1142433, EPI_ISL_1142463, EPI_ISL_1142472 to 1142473, EPI_ISL_1142498, EPI_ISL_1142501, EPI_ISL_1142757, EPI_ISL_1142815, EPI_ISL_1142838, EPI_ISL_1142896, EPI_ISL_1142967, EPI_ISL_1142997, EPI_ISL_1143001, EPI_ISL_1143030, EPI_ISL_1143090, EPI_ISL_1143113, EPI_ISL_1143119, EPI_ISL_1143122, EPI_ISL_1143126 to 1143127, EPI_ISL_1143135, EPI_ISL_1143159, EPI_ISL_1143164, EPI_ISL_1143167, EPI_ISL_1143187, EPI_ISL_1143193, EPI_ISL_1143198, EPI_ISL_1143211, EPI_ISL_1143221, EPI_ISL_1143223, EPI_ISL_1143226, EPI_ISL_1143234, EPI_ISL_1143241, EPI_ISL_1143245, EPI_ISL_1143255, EPI_ISL_1143260, EPI_ISL_1143289, EPI_ISL_1143294, EPI_ISL_1143296, EPI_ISL_1143302, EPI_ISL_1145952, EPI_ISL_1145989, EPI_ISL_1146153, EPI_ISL_1154456, EPI_ISL_1154459, EPI_ISL_1154472 to 1154473, EPI_ISL_1154480, EPI_ISL_1154485, EPI_ISL_1154488, EPI_ISL_1154505, EPI_ISL_1154508, EPI_ISL_1154516 to 1154517, EPI_ISL_1154528, EPI_ISL_1154531 to 1154532, EPI_ISL_1154538, EPI_ISL_1154557, EPI_ISL_1154570, EPI_ISL_1154579, EPI_ISL_1154582, EPI_ISL_1154587, EPI_ISL_1154594, EPI_ISL_1154608, EPI_ISL_1154639, EPI_ISL_1154642, EPI_ISL_1154647, EPI_ISL_1154649, EPI_ISL_1154652, EPI_ISL_1154654, EPI_ISL_1154679, EPI_ISL_1154689, EPI_ISL_1154693, EPI_ISL_1154705, EPI_ISL_1154720, EPI_ISL_1154731, EPI_ISL_1154735, EPI_ISL_1154752, EPI_ISL_1154769, EPI_ISL_1154855, EPI_ISL_1154927, EPI_ISL_1154977, EPI_ISL_1155017, EPI_ISL_1155044, EPI_ISL_1155051, EPI_ISL_1155069, EPI_ISL_1156708, EPI_ISL_1211071, EPI_ISL_1211086, EPI_ISL_1211099, EPI_ISL_1211104, EPI_ISL_1211113, EPI_ISL_1211136, EPI_ISL_1211139, EPI_ISL_1211149, EPI_ISL_1211167, EPI_ISL_1211174 to 1211175, EPI_ISL_1211192, EPI_ISL_1211195, EPI_ISL_1211205, EPI_ISL_1211212, EPI_ISL_1211256, EPI_ISL_1211262, EPI_ISL_1211269, EPI_ISL_1211285, EPI_ISL_1211291, EPI_ISL_1211301, EPI_ISL_1211322, EPI_ISL_1211329, EPI_ISL_1211334, EPI_ISL_1211351, EPI_ISL_1211363, EPI_ISL_1211395, EPI_ISL_1211402, EPI_ISL_1211404, EPI_ISL_1212572, EPI_ISL_1212702, EPI_ISL_1212835, EPI_ISL_1282117

see above | LabKom - Labor Mainz MVZ GmbH | Robert Koch Institute

EPI_ISL_1142275, EPI_ISL_1142288, EPI_ISL_1142303, EPI_ISL_1142316, EPI_ISL_1142323, EPI_ISL_1142330 to 1142331, EPI_ISL_1142333 to 1142334, EPI_ISL_1142349 to 1142351, EPI_ISL_1142359, EPI_ISL_1142373, EPI_ISL_1142377, EPI_ISL_1142383, EPI_ISL_1142390, EPI_ISL_1142394, EPI_ISL_1142409, EPI_ISL_1142417, EPI_ISL_1142420 to 1142423, EPI_ISL_1142442, EPI_ISL_1142446, EPI_ISL_1142465 to 1142466, EPI_ISL_1142487, EPI_ISL_1142504, EPI_ISL_1142753, EPI_ISL_1142763, EPI_ISL_1142781, EPI_ISL_1142790, EPI_ISL_1142817, EPI_ISL_1142827, EPI_ISL_1142849, EPI_ISL_1142954, EPI_ISL_1142963, EPI_ISL_1142975, EPI_ISL_1142979, EPI_ISL_1142989, EPI_ISL_1142998, EPI_ISL_1143013, EPI_ISL_1143035, EPI_ISL_1145966 to 1145967, EPI_ISL_1145975, EPI_ISL_1146000, EPI_ISL_1146004, EPI_ISL_1146032, EPI_ISL_1146069, EPI_ISL_1146081, EPI_ISL_1148173, EPI_ISL_1148177, EPI_ISL_1148191, EPI_ISL_1148235, EPI_ISL_1148272, EPI_ISL_1148278, EPI_ISL_1148283, EPI_ISL_1148305, EPI_ISL_1148308, EPI_ISL_1148316, EPI_ISL_1148325, EPI_ISL_1148343, EPI_ISL_1148366, EPI_ISL_1148383, EPI_ISL_1148396, EPI_ISL_1148429, EPI_ISL_1150045, EPI_ISL_1150058, EPI_ISL_1150068, EPI_ISL_1150114, EPI_ISL_1150116, EPI_ISL_1150141, EPI_ISL_1150163, EPI_ISL_1150178, EPI_ISL_1150182, EPI_ISL_1150187, EPI_ISL_1150224, EPI_ISL_1150311, EPI_ISL_1150316, EPI_ISL_1150323, EPI_ISL_1150368, EPI_ISL_1154458, EPI_ISL_1154466, EPI_ISL_1154477, EPI_ISL_1154519, EPI_ISL_1154534, EPI_ISL_1154545, EPI_ISL_1154553 to 1154554, EPI_ISL_1154556, EPI_ISL_1154566, EPI_ISL_1154614, EPI_ISL_1154627, EPI_ISL_1154635, EPI_ISL_1154638, EPI_ISL_1154641, EPI_ISL_1154658, EPI_ISL_1154681, EPI_ISL_1154690, EPI_ISL_1154698, EPI_ISL_1154703, EPI_ISL_1154710 to 1154711, EPI_ISL_1154717, EPI_ISL_1154728, EPI_ISL_1154744 to 1154745, EPI_ISL_1154748 to 1154749, EPI_ISL_1154758, EPI_ISL_1154760, EPI_ISL_1154776, EPI_ISL_1154837, EPI_ISL_1154839, EPI_ISL_1154846, EPI_ISL_1154868, EPI_ISL_1154872, EPI_ISL_1154879, EPI_ISL_1154882, EPI_ISL_1154920, EPI_ISL_1154932, EPI_ISL_1154951, EPI_ISL_1155009, EPI_ISL_1155020, EPI_ISL_1155050, EPI_ISL_1155056 to 1155057, EPI_ISL_1155075, EPI_ISL_1155080, EPI_ISL_1155083, EPI_ISL_1155090, EPI_ISL_1155104, EPI_ISL_1155116, EPI_ISL_1156455, EPI_ISL_1156459, EPI_ISL_1156463, EPI_ISL_1156469, EPI_ISL_1156475, EPI_ISL_1156479 to 1156481, EPI_ISL_1156484, EPI_ISL_1156493, EPI_ISL_1156497, EPI_ISL_1156500, EPI_ISL_1156506, EPI_ISL_1156508, EPI_ISL_1156515, EPI_ISL_1156520, EPI_ISL_1156533, EPI_ISL_1156546 to 1156547, EPI_ISL_1156559, EPI_ISL_1156565, EPI_ISL_1156585, EPI_ISL_1156596, EPI_ISL_1156610, EPI_ISL_1156617, EPI_ISL_1156624, EPI_ISL_1156627, EPI_ISL_1156632, EPI_ISL_1156635 to 1156636, EPI_ISL_1156641, EPI_ISL_1156654, EPI_ISL_1156656, EPI_ISL_1156665, EPI_ISL_1156669, EPI_ISL_1156672, EPI_ISL_1156680, EPI_ISL_1156683, EPI_ISL_1156693, EPI_ISL_1156698, EPI_ISL_1156700, EPI_ISL_1156716, EPI_ISL_1156719 to 1156721, EPI_ISL_1156739, EPI_ISL_1156742 to 1156743, EPI_ISL_1156756, EPI_ISL_1156758, EPI_ISL_1156761, EPI_ISL_1156764, EPI_ISL_1156769, EPI_ISL_1156771, EPI_ISL_1211074, EPI_ISL_1211102, EPI_ISL_1211152 to 1211153, EPI_ISL_1211165, EPI_ISL_1211178, EPI_ISL_1211288, EPI_ISL_1211326, EPI_ISL_1211338, EPI_ISL_1211378, EPI_ISL_1211405, EPI_ISL_1212523, EPI_ISL_1212528, EPI_ISL_1212563, EPI_ISL_1212615, EPI_ISL_1212671, EPI_ISL_1212676, EPI_ISL_1212708, EPI_ISL_1212768, EPI_ISL_1212797, EPI_ISL_1212855, EPI_ISL_1281995, EPI_ISL_1282011, EPI_ISL_1282014, EPI_ISL_1282020, EPI_ISL_1282024, EPI_ISL_1282027, EPI_ISL_1282032, EPI_ISL_1282034 to 1282035, EPI_ISL_1282042, EPI_ISL_1282052, EPI_ISL_1282055, EPI_ISL_1282058 to 1282059, EPI_ISL_1282067 to 1282069, EPI_ISL_1282076, EPI_ISL_1282078, EPI_ISL_1282083 to 1282085, EPI_ISL_1282093 to 1282094, EPI_ISL_1282096, EPI_ISL_1282099, EPI_ISL_1282112, EPI_ISL_1282116, EPI_ISL_1282119, EPI_ISL_1282122, EPI_ISL_1282124, EPI_ISL_1282135, EPI_ISL_1282140, EPI_ISL_1282150, EPI_ISL_1282176 to 1282177, EPI_ISL_1282183 to 1282184, EPI_ISL_1282187 to 1282188, EPI_ISL_1282190, EPI_ISL_1282194 to 1282195, EPI_ISL_1282204 to 1282206, EPI_ISL_1282208, EPI_ISL_1282213, EPI_ISL_1282217 to 1282218, EPI_ISL_1282229, EPI_ISL_1282231, EPI_ISL_1282273, EPI_ISL_1282304 to 1282305, EPI_ISL_1282308, EPI_ISL_1285587

see above | LabKom - Labor an der Salzbrücke MVZ GmbH | Robert Koch Institute

EPI_ISL_1142735, EPI_ISL_1142745, EPI_ISL_1142751, EPI_ISL_1142756, EPI_ISL_1142761, EPI_ISL_1142769, EPI_ISL_1142786, EPI_ISL_1142795, EPI_ISL_1142805 to 1142806, EPI_ISL_1142809, EPI_ISL_1142813 to 1142814, EPI_ISL_1142822 to 1142823, EPI_ISL_1142832 to 1142833, EPI_ISL_1142842, EPI_ISL_1142846, EPI_ISL_1142850, EPI_ISL_1142852, EPI_ISL_1142855, EPI_ISL_1142864 to 1142865, EPI_ISL_1142870, EPI_ISL_1142876, EPI_ISL_1142878, EPI_ISL_1142883, EPI_ISL_1142890, EPI_ISL_1142892 to 1142893, EPI_ISL_1142908, EPI_ISL_1142912, EPI_ISL_1142918, EPI_ISL_1142927, EPI_ISL_1142941, EPI_ISL_1142947, EPI_ISL_1142956 to 1142957, EPI_ISL_1142959 to 1142960, EPI_ISL_1142965, EPI_ISL_1142969, EPI_ISL_1142971, EPI_ISL_1142978, EPI_ISL_1143009, EPI_ISL_1143021, EPI_ISL_1143023, EPI_ISL_1143031, EPI_ISL_1143033 to 1143034, EPI_ISL_1143053, EPI_ISL_1143062, EPI_ISL_1143066, EPI_ISL_1143077, EPI_ISL_1143079, EPI_ISL_1143088, EPI_ISL_1143100, EPI_ISL_1143111, EPI_ISL_1143144, EPI_ISL_1143153, EPI_ISL_1143162 to 1143163, EPI_ISL_1143166, EPI_ISL_1143247 to 1143248, EPI_ISL_1143251, EPI_ISL_1143266, EPI_ISL_1143270, EPI_ISL_1143274, EPI_ISL_1143277, EPI_ISL_1143283 to 1143284, EPI_ISL_1143288, EPI_ISL_1143297, EPI_ISL_1145993, EPI_ISL_1146054, EPI_ISL_1146160, EPI_ISL_1148169, EPI_ISL_1148189, EPI_ISL_1148203, EPI_ISL_1148255, EPI_ISL_1148266, EPI_ISL_1148401, EPI_ISL_1150065, EPI_ISL_1150081, EPI_ISL_1150156, EPI_ISL_1150162, EPI_ISL_1150222, EPI_ISL_1150245, EPI_ISL_1150250, EPI_ISL_1150282 to 1150284, EPI_ISL_1150342, EPI_ISL_1154682, EPI_ISL_1154791, EPI_ISL_1154793, EPI_ISL_1154797, EPI_ISL_1154804, EPI_ISL_1154823, EPI_ISL_1154832, EPI_ISL_1154844, EPI_ISL_1154847, EPI_ISL_1154871, EPI_ISL_1154908, EPI_ISL_1154912, EPI_ISL_1154917, EPI_ISL_1154931, EPI_ISL_1154935, EPI_ISL_1154942, EPI_ISL_1154954 to 1154955, EPI_ISL_1154960, EPI_ISL_1154975, EPI_ISL_1154981, EPI_ISL_1155013, EPI_ISL_1155028, EPI_ISL_1155033, EPI_ISL_1155037, EPI_ISL_1155062 to 1155063, EPI_ISL_1155091, EPI_ISL_1155093, EPI_ISL_1155126, EPI_ISL_1156453, EPI_ISL_1156464, EPI_ISL_1156467, EPI_ISL_1156485, EPI_ISL_1156490 to 1156491, EPI_ISL_1156496, EPI_ISL_1156498 to 1156499, EPI_ISL_1156505, EPI_ISL_1156509, EPI_ISL_1156512 to 1156513, EPI_ISL_1156516, EPI_ISL_1156519, EPI_ISL_1156525, EPI_ISL_1156529, EPI_ISL_1156542, EPI_ISL_1156548, EPI_ISL_1156551, EPI_ISL_1156557, EPI_ISL_1156566, EPI_ISL_1156568, EPI_ISL_1156570, EPI_ISL_1156573, EPI_ISL_1156576, EPI_ISL_1156583, EPI_ISL_1156598 to 1156599, EPI_ISL_1156601, EPI_ISL_1156603, EPI_ISL_1156608, EPI_ISL_1156621, EPI_ISL_1156634, EPI_ISL_1156643, EPI_ISL_1156652, EPI_ISL_1156655, EPI_ISL_1156660, EPI_ISL_1156668, EPI_ISL_1156671, EPI_ISL_1156674, EPI_ISL_1156681, EPI_ISL_1156687, EPI_ISL_1156690, EPI_ISL_1156710, EPI_ISL_1156714, EPI_ISL_1156725, EPI_ISL_1156729, EPI_ISL_1156736, EPI_ISL_1156740 to 1156741, EPI_ISL_1156745 to 1156746, EPI_ISL_1156754 to 1156755, EPI_ISL_1156763, EPI_ISL_1156778, EPI_ISL_1212509, EPI_ISL_1212514, EPI_ISL_1212517, EPI_ISL_1212539, EPI_ISL_1212555, EPI_ISL_1212557, EPI_ISL_1212559 to 1212560, EPI_ISL_1212567, EPI_ISL_1212580 to 1212581, EPI_ISL_1212585, EPI_ISL_1212592, EPI_ISL_1212606, EPI_ISL_1212620, EPI_ISL_1212624, EPI_ISL_1212635, EPI_ISL_1212640, EPI_ISL_1212712, EPI_ISL_1212722, EPI_ISL_1212755, EPI_ISL_1212763, EPI_ISL_1212790, EPI_ISL_1212819, EPI_ISL_1212821, EPI_ISL_1212826, EPI_ISL_1212837, EPI_ISL_1212840, EPI_ISL_1212843, EPI_ISL_1212853, EPI_ISL_1216577, EPI_ISL_1216638, EPI_ISL_1216663, EPI_ISL_1216708 to 1216709, EPI_ISL_1216711, EPI_ISL_1216740, EPI_ISL_1216749, EPI_ISL_1216751, EPI_ISL_1216765, EPI_ISL_1285431, EPI_ISL_1285433, EPI_ISL_1285436, EPI_ISL_1285440 to 1285441, EPI_ISL_1285444, EPI_ISL_1285452, EPI_ISL_1285456, EPI_ISL_1285458 to 1285459, EPI_ISL_1285466, EPI_ISL_1285479, EPI_ISL_1285482, EPI_ISL_1285491, EPI_ISL_1285503, EPI_ISL_1285505 to 1285506, EPI_ISL_1285508, EPI_ISL_1285514 to 1285515, EPI_ISL_1285519, EPI_ISL_1285527, EPI_ISL_1285530, EPI_ISL_1285536, EPI_ISL_1285538, EPI_ISL_1285540 to 1285542, EPI_ISL_1285544, EPI_ISL_1285548, EPI_ISL_1285558, EPI_ISL_1285567, EPI_ISL_1285569, EPI_ISL_1285578 to 1285579, EPI_ISL_1285584, EPI_ISL_1285586, EPI_ISL_1285589, EPI_ISL_1285592, EPI_ISL_1285599, EPI_ISL_1285607, EPI_ISL_1285611, EPI_ISL_1285620, EPI_ISL_1285622, EPI_ISL_1285627, EPI_ISL_1285629, EPI_ISL_1285635, EPI_ISL_1285645, EPI_ISL_1285647, EPI_ISL_1285649, EPI_ISL_1285660 to 1285661, EPI_ISL_1285665, EPI_ISL_1285668, EPI_ISL_1285676 to 1285677, EPI_ISL_1285680, EPI_ISL_1285683 to 1285684, EPI_ISL_1285687 to 1285688, EPI_ISL_1285691, EPI_ISL_1285693, EPI_ISL_1285695, EPI_ISL_1285703, EPI_ISL_1285708, EPI_ISL_1285712 to 1285715, EPI_ISL_1285717

see above | LabKom - MVZ Labor Bochum MLB GmbH | Robert Koch Institute

EPI_ISL_456157, EPI_ISL_456172 to 456180, EPI_ISL_456196 to 456212, EPI_ISL_456300, EPI_ISL_456379 to 456384, EPI_ISL_456388 to 456394, EPI_ISL_534335, EPI_ISL_547969, EPI_ISL_547976, EPI_ISL_547978 to 547979, EPI_ISL_547990 to 547992, EPI_ISL_547996 to 548000, EPI_ISL_548017, EPI_ISL_548021 to

548027, EPI_ISL_548040 to 548041, EPI_ISL_548049 to 548051, EPI_ISL_548068 to 548073, EPI_ISL_548077 to 548080, EPI_ISL_548083 to 548093, EPI_ISL_548106 to 548111, EPI_ISL_548119 to 548120, EPI_ISL_548122, EPI_ISL_548128, EPI_ISL_548131, EPI_ISL_548135 to 548137, EPI_ISL_548141 to 548142, EPI_ISL_579059, EPI_ISL_579097 to 579098, EPI_ISL_579105 to 579117, EPI_ISL_579119 to 579120, EPI_ISL_579122 to 579123, EPI_ISL_579156, EPI_ISL_579233 to 579293, EPI_ISL_579315 to 579378, EPI_ISL_579403, EPI_ISL_579405, EPI_ISL_579415 to 579417, EPI_ISL_579425, EPI_ISL_579514, EPI_ISL_582017, EPI_ISL_622773 to 622774, EPI_ISL_622777, EPI_ISL_622779, EPI_ISL_622803, EPI_ISL_622807, EPI_ISL_622814 to 622816, EPI_ISL_622821 to 622823, EPI_ISL_626352 to 626356, EPI_ISL_637089 to 637095, EPI_ISL_661255 to 661259, EPI_ISL_661261 to 661264, EPI_ISL_682291 to 682294, EPI_ISL_707795 to 707805, EPI_ISL_732962, EPI_ISL_732964 to 732967, EPI_ISL_755627 to 755633, EPI_ISL_755637, EPI_ISL_794610 to 794623, EPI_ISL_843193, EPI_ISL_843196, EPI_ISL_877205 to 877213, EPI_ISL_877221 to 877226, EPI_ISL_1016846 to 1016847, EPI_ISL_1016849, EPI_ISL_1016851, EPI_ISL_1016853 to 1016854, EPI_ISL_1016858 to 1016862, EPI_ISL_1016864, EPI_ISL_1016867, EPI_ISL_1016869, EPI_ISL_1016871, EPI_ISL_1016873, EPI_ISL_1016877, EPI_ISL_1016880, EPI_ISL_1016883, EPI_ISL_1082260, EPI_ISL_1082263, EPI_ISL_1082265 to 1082266, EPI_ISL_1082268, EPI_ISL_1172028 to 1172033, EPI_ISL_1250698 to 1250701, EPI_ISL_1250703 to 1250705, EPI_ISL_1250708 to 1250711			
see above	LabPLUS	Institute of Environmental Science and Research (ESR)	Anja Werno; Antje van der Linden; Arlo Upton; Chris Mansell; David Hammer; Dragana Drinkovic; Erasmus Smit; Gary McAuliffe; Hana Sofia Andersson; Hermes Perez; James Ussher; Jill Sherwood; Jing Wang; Joep de Ligt; Josh Freeman; Julia Howard; Juliet Elvy; Lauren Jelly; Mary DeAlmeida; Matt Blakiston; Matt Storey; Matthew Rogers; Max Bloomfield; Michael Addidle; Michelle Balm; Muhammad Faisal; Nikki Freed; Olin Silander; Olivia Stroeven; Rachel Boyle; Sally Roberts; SallyAnn Harbison; Sarah Jefferies; Sharmini Muttaiyah; Susan Morpeth; Susan Taylor; Timothy Blackmore; Vani Sathyendran; Veronica Playle; Virginia Hope; Xiaoyun Ren
EPI_ISL_547970 to 547975, EPI_ISL_547977, EPI_ISL_547986 to 547989, EPI_ISL_547993 to 547995, EPI_ISL_548013 to 548016, EPI_ISL_548046, EPI_ISL_548104 to 548105, EPI_ISL_579058, EPI_ISL_579099, EPI_ISL_579421 to 579424, EPI_ISL_622778, EPI_ISL_622793, EPI_ISL_637084, EPI_ISL_637096, EPI_ISL_1016856, EPI_ISL_1082261, EPI_ISL_1113757			
see above	LabTests	Institute of Environmental Science and Research (ESR)	Anja Werno; Antje van der Linden; Arlo Upton; Chris Mansell; David Hammer; Dragana Drinkovic; Erasmus Smit; Gary McAuliffe; Hana Sofia Andersson; Hermes Perez; James Ussher; Jill Sherwood; Jing Wang; Joep de Ligt; Josh Freeman; Julia Howard; Juliet Elvy; Lauren Jelly; Mary DeAlmeida; Matt Blakiston; Matt Storey; Matthew Rogers; Max Bloomfield; Michael Addidle; Michelle Balm; Muhammad Faisal; Nikki Freed; Olin Silander; Olivia Stroeven; Rachel Boyle; Sally Roberts; SallyAnn Harbison; Sarah Jefferies; Sharmini Muttaiyah; Susan Morpeth; Susan Taylor; Timothy Blackmore; Vani Sathyendran; Veronica Playle; Virginia Hope; Xiaoyun Ren
EPI_ISL_886158 to 886169, EPI_ISL_886172 to 886241, EPI_ISL_886243 to 886263, EPI_ISL_886265 to 886356, EPI_ISL_886358 to 886695, EPI_ISL_886697 to 886993, EPI_ISL_886995 to 887054, EPI_ISL_887056, EPI_ISL_887058, EPI_ISL_887061 to 887062, EPI_ISL_887067, EPI_ISL_887069, EPI_ISL_887071, EPI_ISL_887073, EPI_ISL_887075 to 887091, EPI_ISL_887093 to 887104, EPI_ISL_887591 to 887911, EPI_ISL_887913 to 887921, EPI_ISL_887923 to 887924, EPI_ISL_887926 to 887933, EPI_ISL_887935 to 887936, EPI_ISL_887938 to 888119, EPI_ISL_888121 to 888277, EPI_ISL_888279 to 888289, EPI_ISL_888291 to 888292, EPI_ISL_888294 to 888295, EPI_ISL_888297 to 888300, EPI_ISL_888302 to 888304, EPI_ISL_888310, EPI_ISL_888312 to 888435, EPI_ISL_888438 to 888439, EPI_ISL_888443, EPI_ISL_888448 to 888483, EPI_ISL_888485 to 888499, EPI_ISL_888502 to 888588			
see above	Labcorp	Genomics and Discovery, Respiratory Viruses Branch, Division of Viral Diseases, Centers for Disease Control and Prevention	; Amanda Douglas; Amanda Suchanek; Andrea Throop; Ayla Burns; Ben L. Rambo-Martin; Bobbi Croy; Brian Krueger; Brian Norvell; Christos Petropoulos; Clinton R. Paden; Craig Lukasik; Debbie Boles; Dhwani Batra; Duncan MacCannell; Eyad Almasri; Goran Stevovic; Howard Engler; Hrushikesh Deshmukh; Jake Humphrey; Jana Schroth; Joe Voshell; John Pruitt; Jonathan Meltzer; Jonathan Williams; Kimberly Wagner; Lax Iyer; Lyndon Tilson; Manoj Jain; Marcia Eisenberg; Mary Ann Cristobal; Mary Williamson; Michael Levandoski; Mike Sapeta; Mindy Nye; Minoo Agarwal; Mohan Kolli; Nuthawin Charoensri; Oren Cohen; Peter W. Cook; Prashant Gupta; Qian Zeng; Rama Ghatti; Scott Parker; Scott Ryan; Stanley Letovsky; Steven Ragan; Summer Galloway; Suresh Babu Selvaraju; Susan Countryman; Susan Hicks; Suxiang Tong; Suzanne Dale; Thomas Urban; Tim Kuphal; Tricia Zwiefelhofer; Vincent Drouillon
EPI_ISL_560637 to 560642, EPI_ISL_846642 to 846643, EPI_ISL_846645 to 846648, EPI_ISL_855357, EPI_ISL_855381, EPI_ISL_855387, EPI_ISL_860824, EPI_ISL_860829 to 860831, EPI_ISL_860833 to 860841, EPI_ISL_860852 to 860859, EPI_ISL_860864, EPI_ISL_860866, EPI_ISL_860869 to 860889, EPI_ISL_860931, EPI_ISL_861234, EPI_ISL_861237, EPI_ISL_872201, EPI_ISL_872203 to 872205, EPI_ISL_872207 to 872209, EPI_ISL_872234 to 872235, EPI_ISL_872239, EPI_ISL_872256 to 872260, EPI_ISL_872279 to 872280, EPI_ISL_872282 to 872290, EPI_ISL_872320 to 872324, EPI_ISL_883959 to 883963, EPI_ISL_883997 to 884005, EPI_ISL_890352 to 890354, EPI_ISL_909665, EPI_ISL_909667 to 909668, EPI_ISL_909675 to 909681, EPI_ISL_909707, EPI_ISL_909724 to 909727, EPI_ISL_909729 to 909731, EPI_ISL_909733 to 909735, EPI_ISL_912545, EPI_ISL_912547 to 912549, EPI_ISL_912569, EPI_ISL_912584 to 912597, EPI_ISL_912600 to 912601, EPI_ISL_912603 to 912605, EPI_ISL_912607 to 912611, EPI_ISL_912617 to 912620, EPI_ISL_912622 to 912623, EPI_ISL_935527 to 935539, EPI_ISL_935546 to 935550, EPI_ISL_935563 to 935564, EPI_ISL_935576, EPI_ISL_935590, EPI_ISL_935605 to 935606, EPI_ISL_935637 to 935638, EPI_ISL_935642 to 935649, EPI_ISL_940997 to 940999, EPI_ISL_941004 to 941014, EPI_ISL_941026, EPI_ISL_941028 to 941103, EPI_ISL_953956, EPI_ISL_953968, EPI_ISL_953986, EPI_ISL_953988, EPI_ISL_953994, EPI_ISL_954007, EPI_ISL_954022 to 954025, EPI_ISL_954030 to 954031, EPI_ISL_954036 to 954038, EPI_ISL_954040 to 954041, EPI_ISL_954049 to 954052, EPI_ISL_954054, EPI_ISL_954059 to 954070, EPI_ISL_954078 to 954080, EPI_ISL_954091 to 954093, EPI_ISL_954105, EPI_ISL_954114, EPI_ISL_954119, EPI_ISL_954121, EPI_ISL_954123 to 954124, EPI_ISL_954127 to 954128, EPI_ISL_955073, EPI_ISL_955094, EPI_ISL_955180 to 955183, EPI_ISL_995468 to 995470, EPI_ISL_995491 to 995494, EPI_ISL_1001318, EPI_ISL_1001329, EPI_ISL_1035960, EPI_ISL_1035962 to 1035965, EPI_ISL_1036403 to 1036404, EPI_ISL_1060369 to 1060370, EPI_ISL_1060376, EPI_ISL_1060396, EPI_ISL_1060399, EPI_ISL_1060401, EPI_ISL_1060404, EPI_ISL_1060407 to 1060408, EPI_ISL_1060414, EPI_ISL_1060422 to 1060428, EPI_ISL_1060434 to 1060437, EPI_ISL_1060439 to 1060440, EPI_ISL_1060443, EPI_ISL_1060445, EPI_ISL_1060463 to 1060468, EPI_ISL_1060476, EPI_ISL_1060479 to 1060480, EPI_ISL_1060483 to 1060490, EPI_ISL_1060562, EPI_ISL_1060568 to 1060569, EPI_ISL_1060572, EPI_ISL_1060574, EPI_ISL_1060576, EPI_ISL_1060593, EPI_ISL_1096243, EPI_ISL_1096247, EPI_ISL_1096250 to 1096252, EPI_ISL_1096268 to 1096269, EPI_ISL_1096303, EPI_ISL_1096313 to 1096316, EPI_ISL_1110399 to 1110405, EPI_ISL_1110408, EPI_ISL_1110420, EPI_ISL_1110422 to 1110427, EPI_ISL_1110430, EPI_ISL_1110432 to 1110434, EPI_ISL_1110439, EPI_ISL_1110442 to 1110443, EPI_ISL_1110460 to 1110461, EPI_ISL_1110467 to 1110471, EPI_ISL_1110473 to 1110474, EPI_ISL_1110888 to 1110889, EPI_ISL_1110892 to 1110894, EPI_ISL_1110924 to 1110927, EPI_ISL_1110980, EPI_ISL_1111060, EPI_ISL_1111062 to 1111064, EPI_ISL_1118902 to 1118903, EPI_ISL_1118905, EPI_ISL_1168195 to 1168201, EPI_ISL_1168205, EPI_ISL_1201521, EPI_ISL_1201544 to 1201553, EPI_ISL_1219935 to 1219936, EPI_ISL_1219938, EPI_ISL_1219949 to 1219950, EPI_ISL_1219959 to 1219960, EPI_ISL_1219962 to 1219963, EPI_ISL_1233237 to 1233246, EPI_ISL_1259298, EPI_ISL_1259301 to 1259303, EPI_ISL_1262763 to 1262812, EPI_ISL_1262881 to 1262897, EPI_ISL_1262915 to 1262916, EPI_ISL_1262934 to 1262980, EPI_ISL_1262990 to 1263089, EPI_ISL_1301821 to 1301822, EPI_ISL_1301861 to 1301862, EPI_ISL_1301865, EPI_ISL_1301884 to 1301886, EPI_ISL_1301901 to 1301902, EPI_ISL_1301916 to 1301919, EPI_ISL_1301923, EPI_ISL_1301945 to 1301947, EPI_ISL_1301959			
see above	Labo Analyses Med	National Reference Center for Viruses of Respiratory Infections, Institut Pasteur, Paris	; Amzalag Jonas; Angela Brisebarre; Bendayan James; Besson J; Bonnaudet GéRaldine; Bonnet Cyrile; Bour Jean Baptiste; Breton-Cazaux JéRôMe; Cady Anne; Camille Capel; Cerballiance Paris; Chauvet; Coignard Catherine; Delamare Catherine; Desjardins Nicolas; Dlab Dieppe; Dornic Catherine; Ducancelle Alexandra; Dufour Mj; Durivault JéRôMe; Dynalab; Ebel Anne; Etienne Elodie; Etienne Simon-Lorière; Fabiana Gambaro; Felloni Claire; Fiorina Jc; Foissaud Vincent; Garand Anne-Laure; Garel FréDéRick; Gaumery Bertrand; Gestin (B) Brieuc; Gestin Brieuc; Girard Sophie; Griscelli Franck; Harich; Holstein Anne; Jacques Alexandra; Jouard Charles; Labm Hignard; Laroche Christine; Le Berre (D) David; Le Vicky; Lebihan David; Lecorche; Lefaure Brieuc; Leonard Pierre-Yves; Lescieux Alexandre; Lesimple BéAtrice; Louise Lefrançois; Marion Barbet; Martres Pascale; Maud Vanpeene; Merah Kader; Michez Karine; Morvan Odile; Méline Bizard; Nativelle; Nizard Patrice; Oliel Jonathan; Pasquier; Potiron GréGoire; Ravaux Jean Jacques; Roquebert BéNéDicte; Rouah Raquel; Rousset Dominique; Said-Delattre OphéLie; Sarret Benoit; Selas Labomaine; Serazin V; Sylvie Behillil; Sylvie van der Werf; Takoudju Eve-Marie; Tomasi Florent; Vincent Enouf; Wittersheim Pascal
EPI_ISL_593924 to 593933	Labo Analyses Med, Puteaux	National Reference Center for Viruses of Respiratory Infections, Institut Pasteur, Paris	Etienne Simon-Lorière; Fabiana Gambaro; Maud Vanpeene; Sylvie Behillil; Sylvie van der Werf; Vincent Enouf
EPI_ISL_593904 to 593909	Labo Analyses Med, Sarcelles	National Reference Center for Viruses of Respiratory Infections, Institut Pasteur, Paris	Etienne Simon-Lorière; Fabiana Gambaro; Maud Vanpeene; Sylvie Behillil; Sylvie van der Werf; Vincent Enouf
EPI_ISL_860826, EPI_ISL_860842 to 860845, EPI_ISL_860860 to 860862, EPI_ISL_861239, EPI_ISL_883964 to 883971, EPI_ISL_894223, EPI_ISL_894232, EPI_ISL_909655 to 909664, EPI_ISL_909669 to 909673, EPI_ISL_935587 to 935589, EPI_ISL_935591 to 935604, EPI_ISL_935607 to 935636, EPI_ISL_955178 to 955179			
see above	Labo Analyses med	National Reference Center for Viruses of Respiratory Infections, Institut Pasteur, Paris	Amzalag Jonas; Angela Brisebarre; Camille Capel; Etienne Simon-Lorière; Marion Barbet; Maud Vanpeene; Méline Bizard; Sylvie Behillil; Sylvie van der Werf; Vincent Enouf
EPI_ISL_420063	Labo BM - Site de Juvisy - Hopital Général	National Reference Center for Viruses of Respiratory Infections, Institut Pasteur, Paris	Angela Brisebarre; Etienne Simon-Lorière; Flora Donati; Marion Barbet; Maud Vanpeene; Mélanie Albert; Méline Bizard; Sylvie Behillil; Sylvie van der Werf; Vincent Enouf
EPI_ISL_930594 to 930595, EPI_ISL_959569 to 959588, EPI_ISL_965306 to 965308, EPI_ISL_965318 to 965319, EPI_ISL_965328, EPI_ISL_965330 to 965331, EPI_ISL_965343, EPI_ISL_965399, EPI_ISL_965406 to 965407, EPI_ISL_965409, EPI_ISL_965411 to 965413, EPI_ISL_965419 to 965420, EPI_ISL_965469, EPI_ISL_965474, EPI_ISL_965476 to 965478, EPI_ISL_965504, EPI_ISL_1171562 to 1171566			
see above	Labo Luc Olivier	GIGA Medical Genomics	Bouchra Boujemla; Cécile Meex; Keith Durkin; Maria Artesi; Marie-Pierre Hayette; Nathalie Renotte; Pierrette Melin; Raphaël Boreux; Sébastien Bontems; Vincent Bours
EPI_ISL_1281683, EPI_ISL_1281685	Labo Somedi	Imelda hospital Bonheiden	Dagmar Obbels; Hanne Valgaeren; Johan Frans
EPI_ISL_1060378, EPI_ISL_1060397	Labo analyse Med	National Reference Center for Viruses of Respiratory Infections, Institut Pasteur, Paris	Angela Brisebarre; Camille Capel; Etienne Simon-Lorière; Goubard Agathe; Marion Barbet; Maud Vanpeene; Méline Bizard; Sylvie Behillil; Sylvie van der Werf; Vincent Enouf
EPI_ISL_955062 to 955063, EPI_ISL_955068 to 955072, EPI_ISL_955076, EPI_ISL_955079 to 955080, EPI_ISL_955082, EPI_ISL_955086 to 955093	Labo analyses Med	National Reference Center for Viruses of Respiratory Infections, Institut Pasteur, Paris	Angela Brisebarre; Camille Capel; Etienne Simon-Lorière; Marion Barbet; Maud Vanpeene; Méline Bizard; Potiron GréGoire; Sylvie Behillil; Sylvie van der Werf; Vincent Enouf
EPI_ISL_872212, EPI_ISL_872238, EPI_ISL_872264 to 872265, EPI_ISL_872309 to 872314, EPI_ISL_955066 to 955067, EPI_ISL_977661 to 977663, EPI_ISL_1035919 to 1035922, EPI_ISL_1035961, EPI_ISL_1036373 to 1036375, EPI_ISL_1036409 to 1036425			
see above	Labo analyses med	National Reference Center for Viruses of Respiratory Infections, Institut Pasteur, Paris	Amzalag Jonas; Angela Brisebarre; Camille Capel; Etienne Simon-Lorière; Goubard Agathe; Marion Barbet; Maud Vanpeene; Méline Bizard; Sylvie Behillil; Sylvie van der Werf; Vincent Enouf
EPI_ISL_416029	Laboiratório Fleury	Instituto Adolfo Lutz, Interdiciplinary Procedures Center, Strategic Laboratory	Adriana Bugno; Adriano Abbud; Carlos Henrique Camargo; Claudia Regina Gonçalves; Claudio Tavares Sacchi; Daniela Bernardes Borges da Silva; Fabiana Cristina Pereira dos Santos; Maria do Carmo Sampaio Tavares Timenetsky; Simone Guadagnucci Morillo; Terezinha Maria de Paiva
EPI_ISL_1143041, EPI_ISL_1143043, EPI_ISL_1143052, EPI_ISL_1143058, EPI_ISL_1143083, EPI_ISL_1143092, EPI_ISL_1143123, EPI_ISL_1143136, EPI_ISL_1143146, EPI_ISL_1143157, EPI_ISL_1143165, EPI_ISL_1143188, EPI_ISL_1143209, EPI_ISL_1143224, EPI_ISL_1143232, EPI_ISL_1143235, EPI_ISL_1143258, EPI_ISL_1143273, EPI_ISL_1143276, EPI_ISL_1143281, EPI_ISL_1145916, EPI_ISL_1145921, EPI_ISL_1145943, EPI_ISL_1145953, EPI_ISL_1145958, EPI_ISL_1146018, EPI_ISL_1146030, EPI_ISL_1146059, EPI_ISL_1146061, EPI_ISL_1146067, EPI_ISL_1146071, EPI_ISL_1146087, EPI_ISL_1146091, EPI_ISL_1146130,			

EPI_ISL_1146135, EPI_ISL_1154792, EPI_ISL_1154806, EPI_ISL_1154821, EPI_ISL_1154841, EPI_ISL_1154848, EPI_ISL_1154856, EPI_ISL_1154883, EPI_ISL_1154889, EPI_ISL_1154938, EPI_ISL_1154948, EPI_ISL_1154967, EPI_ISL_1154991, EPI_ISL_1155026, EPI_ISL_1155040, EPI_ISL_1155070 to 1155071, EPI_ISL_1155099, EPI_ISL_1155105, EPI_ISL_1156456, EPI_ISL_1156494, EPI_ISL_1156538, EPI_ISL_1156553, EPI_ISL_1156663, EPI_ISL_1156759, EPI_ISL_1156765 to 1156766, EPI_ISL_1156768, EPI_ISL_1212504, EPI_ISL_1212507, EPI_ISL_1212512, EPI_ISL_1212518, EPI_ISL_1212521, EPI_ISL_1212550, EPI_ISL_1212570, EPI_ISL_1212629, EPI_ISL_1212644, EPI_ISL_1212649, EPI_ISL_1212669, EPI_ISL_1212674, EPI_ISL_1212683, EPI_ISL_1212690, EPI_ISL_1212749 to 1212750, EPI_ISL_1212769 to 1212770, EPI_ISL_1212774, EPI_ISL_1212776, EPI_ISL_1212785, EPI_ISL_1212817, EPI_ISL_1212820, EPI_ISL_1212823, EPI_ISL_1212838, EPI_ISL_1212848, EPI_ISL_1212856, EPI_ISL_1212859, EPI_ISL_1212861 to 1212863			
see above	Labor 28 MVZ GmbH	Robert Koch Institute	
EPI_ISL_1211426 to 1211441, EPI_ISL_1287320 to 1287348	Labor Becker & Kollegen (Standort München)	Robert Koch Institute	
EPI_ISL_1144290 to 1144291, EPI_ISL_1144296 to 1144297, EPI_ISL_1144302, EPI_ISL_1144304, EPI_ISL_1144306 to 1144308, EPI_ISL_1144311, EPI_ISL_1144316, EPI_ISL_1144321, EPI_ISL_1144335, EPI_ISL_1144341, EPI_ISL_1144343, EPI_ISL_1144345 to 1144346, EPI_ISL_1144348 to 1144349, EPI_ISL_1144351 to 1144352, EPI_ISL_1144354, EPI_ISL_1147140 to 1147141, EPI_ISL_1147368 to 1147374, EPI_ISL_1147377, EPI_ISL_1147383 to 1147384, EPI_ISL_1147387 to 1147394, EPI_ISL_1147396 to 1147404, EPI_ISL_1147407 to 1147408, EPI_ISL_1147411 to 1147412			
see above	Labor Blackholm MVZ	Robert Koch Institute	
EPI_ISL_1144907 to 1144912, EPI_ISL_1150465, EPI_ISL_1150475 to 1150476, EPI_ISL_1152013, EPI_ISL_1156975, EPI_ISL_1156977, EPI_ISL_1156979, EPI_ISL_1156984, EPI_ISL_1156994, EPI_ISL_1156999 to 1157000, EPI_ISL_1157014, EPI_ISL_1157028, EPI_ISL_1157037, EPI_ISL_1157046, EPI_ISL_1157051, EPI_ISL_1157054 to 1157055, EPI_ISL_1217123 to 1217126, EPI_ISL_1217128, EPI_ISL_1217130 to 1217131, EPI_ISL_1217133, EPI_ISL_1217135 to 1217137, EPI_ISL_1217141, EPI_ISL_1217144 to 1217146, EPI_ISL_1217149, EPI_ISL_1217152, EPI_ISL_1217156, EPI_ISL_1217158, EPI_ISL_1217161 to 1217163, EPI_ISL_1217167 to 1217168, EPI_ISL_1217172, EPI_ISL_1217174 to 1217176, EPI_ISL_1286094 to 1286095, EPI_ISL_1286098 to 1286101, EPI_ISL_1286103 to 1286105, EPI_ISL_1286107, EPI_ISL_1286110, EPI_ISL_1286112 to 1286113, EPI_ISL_1286115, EPI_ISL_1286117, EPI_ISL_1286119, EPI_ISL_1286123 to 1286124, EPI_ISL_1286127 to 1286133			
see above	Labor Dr. Heidrich & Kollegen MVZ GmbH Hamburg	Robert Koch Institute	
EPI_ISL_831404 to 831405, EPI_ISL_831407, EPI_ISL_831412 to 831413, EPI_ISL_831417 to 831419, EPI_ISL_831423, EPI_ISL_831426, EPI_ISL_1048243, EPI_ISL_1048263, EPI_ISL_1048274, EPI_ISL_1048292 to 1048293, EPI_ISL_1048296, EPI_ISL_1048299, EPI_ISL_1048301, EPI_ISL_1048318, EPI_ISL_1048337 to 1048343			
see above	Labor Dr. Krause & Kollegen MVZ GmbH	Robert Koch Institute, Influenza and respiratory viruses FG17 & Bioinformatics MF1, Berlin, Germany	A. Krumbholz; Aleksandar Radonic; Dr. Lorentz; Lorentz; Marianne Wedde; Oliver Drechsel; Ralf Duerrwald; Rene Kmiecinski; Stefan Kroeger; Stephan Fuchs; Thorsten Wolff
EPI_ISL_1145970, EPI_ISL_1146024, EPI_ISL_1146083, EPI_ISL_1146086, EPI_ISL_1146093, EPI_ISL_1146104, EPI_ISL_1146121, EPI_ISL_1146138, EPI_ISL_1151429, EPI_ISL_1151469, EPI_ISL_1151506, EPI_ISL_1151547, EPI_ISL_1151687, EPI_ISL_1151741, EPI_ISL_1154808, EPI_ISL_1154822, EPI_ISL_1154843, EPI_ISL_1154851 to 1154852, EPI_ISL_1154940, EPI_ISL_1154946, EPI_ISL_1155003, EPI_ISL_1155068, EPI_ISL_1155123, EPI_ISL_1211093, EPI_ISL_1211119, EPI_ISL_1211125, EPI_ISL_1211140, EPI_ISL_1211157, EPI_ISL_1211172, EPI_ISL_1211187, EPI_ISL_1211206, EPI_ISL_1211215 to 1211216, EPI_ISL_1211218, EPI_ISL_1211223, EPI_ISL_1211231, EPI_ISL_1211236, EPI_ISL_1211272, EPI_ISL_1211302, EPI_ISL_1211304, EPI_ISL_1211306, EPI_ISL_1211335, EPI_ISL_1211344, EPI_ISL_1211366, EPI_ISL_1211396, EPI_ISL_1211415, EPI_ISL_1211424, EPI_ISL_1216455, EPI_ISL_1216480, EPI_ISL_1216483, EPI_ISL_1216498 to 1216499, EPI_ISL_1216524, EPI_ISL_1216548 to 1216549, EPI_ISL_1216581 to 1216582, EPI_ISL_1216592, EPI_ISL_1216599, EPI_ISL_1216601, EPI_ISL_1216605, EPI_ISL_1216608, EPI_ISL_1216662, EPI_ISL_1216691, EPI_ISL_1216694, EPI_ISL_1216720, EPI_ISL_1216735, EPI_ISL_1216738, EPI_ISL_1216748, EPI_ISL_1216750, EPI_ISL_1216767, EPI_ISL_1284570, EPI_ISL_1284599, EPI_ISL_1284616, EPI_ISL_1284628, EPI_ISL_1284653, EPI_ISL_1284664, EPI_ISL_1284687, EPI_ISL_1284701, EPI_ISL_1284709, EPI_ISL_1284724, EPI_ISL_1284764, EPI_ISL_1284768, EPI_ISL_1284799, EPI_ISL_1284801, EPI_ISL_1284806, EPI_ISL_1284844, EPI_ISL_1284860, EPI_ISL_1284872, EPI_ISL_1284874, EPI_ISL_1285525, EPI_ISL_1285600, EPI_ISL_1285678			
see above	Labor Dr. Schumacher MVZ	Robert Koch Institute	
EPI_ISL_1140136, EPI_ISL_1140179, EPI_ISL_1140839 to 1140841, EPI_ISL_1140849 to 1140850, EPI_ISL_1140852, EPI_ISL_1140855, EPI_ISL_1140857, EPI_ISL_1140865 to 1140867, EPI_ISL_1140869, EPI_ISL_1140879, EPI_ISL_1140881 to 1140882, EPI_ISL_1140885, EPI_ISL_1140888, EPI_ISL_1140893, EPI_ISL_1140900 to 1140901, EPI_ISL_1140913, EPI_ISL_1140998, EPI_ISL_1141807, EPI_ISL_1141823, EPI_ISL_1141828, EPI_ISL_1144513, EPI_ISL_1146545			
see above	Labor Dr. Wisplinghoff - Berlin	Robert Koch Institute	
EPI_ISL_1148588, EPI_ISL_1211444 to 1211450, EPI_ISL_1211452 to 1211455, EPI_ISL_1211458, EPI_ISL_1211460, EPI_ISL_1211462 to 1211466, EPI_ISL_1211468 to 1211469, EPI_ISL_1211471, EPI_ISL_1211473, EPI_ISL_1211476 to 1211477, EPI_ISL_1211480 to 1211481, EPI_ISL_1211483 to 1211488, EPI_ISL_1211490 to 1211494, EPI_ISL_1211496, EPI_ISL_1211499 to 1211500, EPI_ISL_1211503 to 1211506, EPI_ISL_1211508 to 1211509, EPI_ISL_1211512, EPI_ISL_1211515 to 1211523, EPI_ISL_1211525 to 1211526, EPI_ISL_1211528 to 1211533, EPI_ISL_1211535, EPI_ISL_1211538 to 1211539, EPI_ISL_1211541 to 1211542, EPI_ISL_1211544 to 1211548, EPI_ISL_1211550 to 1211554, EPI_ISL_1211557 to 1211559, EPI_ISL_1211562 to 1211570, EPI_ISL_1211572, EPI_ISL_1211574, EPI_ISL_1211576 to 1211579, EPI_ISL_1211583, EPI_ISL_1211587, EPI_ISL_1211590 to 1211598, EPI_ISL_1211600 to 1211601, EPI_ISL_1211603 to 1211606, EPI_ISL_1211611, EPI_ISL_1211618, EPI_ISL_1211622, EPI_ISL_1211625 to 1211626, EPI_ISL_1211629, EPI_ISL_1211633, EPI_ISL_1211635, EPI_ISL_1211642, EPI_ISL_1211644, EPI_ISL_1211646, EPI_ISL_1211648, EPI_ISL_1211650 to 1211651, EPI_ISL_1211653, EPI_ISL_1211655, EPI_ISL_1211657, EPI_ISL_1211659, EPI_ISL_1211661, EPI_ISL_1211663, EPI_ISL_1211665, EPI_ISL_1211667 to 1211668, EPI_ISL_1211670, EPI_ISL_1211672, EPI_ISL_1211674, EPI_ISL_1211683, EPI_ISL_1211685, EPI_ISL_1211687, EPI_ISL_1211689, EPI_ISL_1211691, EPI_ISL_1211693 to 1211694, EPI_ISL_1211698, EPI_ISL_1211701 to 1211702, EPI_ISL_1211704, EPI_ISL_1211708 to 1211709, EPI_ISL_1211713, EPI_ISL_1211715, EPI_ISL_1211722, EPI_ISL_1211724, EPI_ISL_1211727, EPI_ISL_1211731, EPI_ISL_1211733, EPI_ISL_1211735, EPI_ISL_1211744, EPI_ISL_1211746, EPI_ISL_1211748, EPI_ISL_1211750, EPI_ISL_1211754, EPI_ISL_1211758 to 1211759, EPI_ISL_1211765, EPI_ISL_1211767, EPI_ISL_1211771 to 1211772, EPI_ISL_1211774, EPI_ISL_1211776 to 1211777, EPI_ISL_1211779 to 1211783, EPI_ISL_1211785 to 1211787, EPI_ISL_1211789 to 1211790, EPI_ISL_1211792, EPI_ISL_1211796, EPI_ISL_1211799, EPI_ISL_1211802 to 1211804, EPI_ISL_1211806, EPI_ISL_1211811, EPI_ISL_1211813 to 1211816, EPI_ISL_1211818 to 1211819, EPI_ISL_1211821, EPI_ISL_1211823 to 1211831, EPI_ISL_1211834, EPI_ISL_1211836 to 1211839, EPI_ISL_1211841 to 1211842, EPI_ISL_1211844, EPI_ISL_1211846 to 1211849, EPI_ISL_1211854, EPI_ISL_1211857 to 1211861, EPI_ISL_1211863 to 1211864, EPI_ISL_1211866 to 1211872, EPI_ISL_1211874 to 1211877, EPI_ISL_1215812, EPI_ISL_1215814 to 1215817, EPI_ISL_1215820 to 1215825, EPI_ISL_1215828 to 1215829, EPI_ISL_1215831 to 1215837, EPI_ISL_1215839 to 1215841, EPI_ISL_1215843 to 1215846, EPI_ISL_1215848 to 1215852, EPI_ISL_1215855 to 1215856, EPI_ISL_1215860 to 1215873, EPI_ISL_1215877, EPI_ISL_1215880 to 1215881, EPI_ISL_1215883 to 1215885, EPI_ISL_1215887, EPI_ISL_1215890 to 1215892, EPI_ISL_1215895, EPI_ISL_1215897, EPI_ISL_1215899 to 1215900, EPI_ISL_1215902 to 1215904, EPI_ISL_1215906 to 1215907, EPI_ISL_1215909, EPI_ISL_1215913 to 1215914, EPI_ISL_1215916, EPI_ISL_1215920 to 1215922, EPI_ISL_1215925 to 1215928, EPI_ISL_1215931, EPI_ISL_1215933 to 1215936, EPI_ISL_1215938 to 1215942, EPI_ISL_1215945, EPI_ISL_1215949, EPI_ISL_1215951, EPI_ISL_1215954, EPI_ISL_1215956, EPI_ISL_1215959, EPI_ISL_1215964 to 1215968, EPI_ISL_1215970, EPI_ISL_1215973, EPI_ISL_1215975 to 1215980, EPI_ISL_1215983, EPI_ISL_1215986, EPI_ISL_1215989, EPI_ISL_1215991 to 1215992, EPI_ISL_1215995 to 1215997, EPI_ISL_1215999 to 1216000, EPI_ISL_1216003 to 1216007, EPI_ISL_1216010, EPI_ISL_1216012, EPI_ISL_1216015 to 1216016, EPI_ISL_1216018 to 1216019, EPI_ISL_1216022 to 1216023, EPI_ISL_1216026 to 1216027, EPI_ISL_1216029, EPI_ISL_1216032, EPI_ISL_1216036, EPI_ISL_1216038 to 1216041, EPI_ISL_1217110, EPI_ISL_1217113, EPI_ISL_1217116 to 1217117, EPI_ISL_1282384, EPI_ISL_1282391 to 1282392, EPI_ISL_1282400 to 1282401, EPI_ISL_1282404, EPI_ISL_1282406 to 1282408, EPI_ISL_1282410, EPI_ISL_1282416 to 1282417, EPI_ISL_1282425, EPI_ISL_1282432, EPI_ISL_1282436 to 1282437, EPI_ISL_1282439, EPI_ISL_1282442 to 1282443, EPI_ISL_1282448, EPI_ISL_1282451, EPI_ISL_1282455 to 1282457, EPI_ISL_1282459 to 1282461, EPI_ISL_1282463, EPI_ISL_1282471 to 1282473, EPI_ISL_1282475 to 1282476, EPI_ISL_1282481, EPI_ISL_1282484 to 1282486, EPI_ISL_1282490 to 1282491, EPI_ISL_1282493, EPI_ISL_1282498, EPI_ISL_1282501 to 1282503, EPI_ISL_1282507 to 1282508, EPI_ISL_1282511, EPI_ISL_1282514, EPI_ISL_1282519 to 1282520, EPI_ISL_1282524 to 1282525, EPI_ISL_1282527, EPI_ISL_1282529, EPI_ISL_1282539 to 1282541, EPI_ISL_1282547 to 1282548, EPI_ISL_1282550 to 1282553, EPI_ISL_1282555 to 1282557, EPI_ISL_1282560, EPI_ISL_1282562 to 1282563, EPI_ISL_1282566, EPI_ISL_1282569, EPI_ISL_1282571 to 1282572, EPI_ISL_1282578, EPI_ISL_1282581, EPI_ISL_1282584 to 1282585, EPI_ISL_1282592 to 1282593, EPI_ISL_1282597 to 1282600, EPI_ISL_1282602, EPI_ISL_1282605 to 1282609, EPI_ISL_1282611 to 1282612, EPI_ISL_1282616, EPI_ISL_1282619, EPI_ISL_1282624, EPI_ISL_1282628 to 1282632, EPI_ISL_1282635 to 1282636, EPI_ISL_1282638, EPI_ISL_1282640, EPI_ISL_1282642 to 1282643, EPI_ISL_1282654, EPI_ISL_1282657, EPI_ISL_1282659, EPI_ISL_1282661, EPI_ISL_1282663, EPI_ISL_1282665, EPI_ISL_1282669, EPI_ISL_1282672, EPI_ISL_1282674 to 1282675, EPI_ISL_1282680, EPI_ISL_1282688 to 1282689, EPI_ISL_1282692 to 1282698, EPI_ISL_1282700, EPI_ISL_1282704, EPI_ISL_1282707, EPI_ISL_1282709 to 1282710, EPI_ISL_1282714, EPI_ISL_1282718, EPI_ISL_1282720 to 1282721, EPI_ISL_1282723, EPI_ISL_1282726, EPI_ISL_1282728, EPI_ISL_1282731, EPI_ISL_1282733 to 1282734, EPI_ISL_1282737 to 1282738, EPI_ISL_1282740 to 1282741, EPI_ISL_1282743 to 1282744, EPI_ISL_1282747 to 1282748, EPI_ISL_1282752, EPI_ISL_1286907, EPI_ISL_1286909 to 1286911, EPI_ISL_1286913, EPI_ISL_1286920, EPI_ISL_1286941, EPI_ISL_1286962, EPI_ISL_1286964, EPI_ISL_1286967, EPI_ISL_1286972, EPI_ISL_1286974, EPI_ISL_1286979 to 1286981, EPI_ISL_1286987, EPI_ISL_1287001, EPI_ISL_1287027, EPI_ISL_1287030, EPI_ISL_1287040, EPI_ISL_1287045 to 1287046, EPI_ISL_1287048, EPI_ISL_1287053, EPI_ISL_1287060, EPI_ISL_1287063, EPI_ISL_1287066, EPI_ISL_1287069, EPI_ISL_1287075, EPI_ISL_1287077 to 1287078, EPI_ISL_1287080, EPI_ISL_1287082, EPI_ISL_1287108, EPI_ISL_1287112 to 1287113, EPI_ISL_1287115, EPI_ISL_1287120, EPI_ISL_1287126, EPI_ISL_1287145, EPI_ISL_1287156, EPI_ISL_1287165, EPI_ISL_1287177, EPI_ISL_1287190, EPI_ISL_1287198 to 1287199, EPI_ISL_1287203, EPI_ISL_1287210, EPI_ISL_1287216, EPI_ISL_1287220, EPI_ISL_1287225, EPI_ISL_1287235, EPI_ISL_1287242, EPI_ISL_1287248, EPI_ISL_1287256, EPI_ISL_1287262 to 1287263			
see above	Labor Dr. Wisplinghoff - Köln	Robert Koch Institute	
EPI_ISL_751799	Labor Dr. Wisplinghoff - Köln	Robert Koch Institute, Bioinformatics MF1, Berlin, Germany	Aleksandar Radonic; Dr. R. Grosser; Marianne Wedde; Oliver Drechsel; Ralf Duerrwald; Rene Kmiecinski; Stefan Kroeger; Stephan Fuchs; Thorsten Wolff
EPI_ISL_803955 to 803957, EPI_ISL_831402, EPI_ISL_831409, EPI_ISL_831411, EPI_ISL_831420 to 831422, EPI_ISL_831424 to 831425, EPI_ISL_1048247, EPI_ISL_1048253, EPI_ISL_1048255, EPI_ISL_1048271, EPI_ISL_1048275, EPI_ISL_1048282, EPI_ISL_1048285, EPI_ISL_1048290, EPI_ISL_1048302 to 1048303, EPI_ISL_1048319, EPI_ISL_1048344 to 1048349			
see above	Labor Dr. Wisplinghoff - Köln	Robert Koch Institute, Influenza and respiratory viruses FG17 & Bioinformatics MF1, Berlin, Germany	Aleksandar Radonic; Dr. R. Grosser; Marianne Wedde; Oliver Drechsel; R. Grosser; Ralf Duerrwald; Rene Kmiecinski; Stefan Kroeger; Stephan Fuchs; Thorsten Wolff
EPI_ISL_476705, EPI_ISL_487398 to 487431	Labor Kneißler GmbH & Co. KG	Heinrich Pette Institute, Leibniz Institute for Experimental Virology	Adam Grundhoff; Manja Czech-Sioli; Matthias Ottinger; Melanie M. Brinkmann; Nicole Fischer; Thomas Günther
EPI_ISL_1141009 to 1141010, EPI_ISL_1141013, EPI_ISL_1141015 to 1141016, EPI_ISL_1142229 to 1142231, EPI_ISL_1142233 to 1142234, EPI_ISL_1142658 to 1142660, EPI_ISL_1145658 to 1145661, EPI_ISL_1148450 to 1148451, EPI_ISL_1148453 to 1148455, EPI_ISL_1148458 to 1148459, EPI_ISL_1211879, EPI_ISL_1211881, EPI_ISL_1211883 to 1211887, EPI_ISL_1217348 to 1217354, EPI_ISL_1281777 to 1281784			
see above	Labor Kneißler GmbH & Co. KG	Robert Koch Institute	
EPI_ISL_831370 to 831372, EPI_ISL_831374, EPI_ISL_831379, EPI_ISL_831382 to 831385, EPI_ISL_831390, EPI_ISL_831392, EPI_ISL_831394 to 831395, EPI_ISL_831397 to 831399, EPI_ISL_831401			
see above	Labor Krone	Robert Koch Institute, Influenza and respiratory viruses FG17 & Bioinformatics MF1, Berlin, Germany	Aleksandar Radonic; Dr. Münstermann. Prof. Tiemann; Marianne Wedde; Oliver Drechsel; Ralf Duerrwald; Rene Kmiecinski; Stefan Kroeger; Stephan Fuchs; Thorsten Wolff
EPI_ISL_1153472 to 1153473, EPI_ISL_1153483, EPI_ISL_1153486, EPI_ISL_1153492, EPI_ISL_1153503 to 1153504, EPI_ISL_1153522, EPI_ISL_1153526, EPI_ISL_1156099, EPI_ISL_1156124, EPI_ISL_1212257, EPI_ISL_1212273, EPI_ISL_1212284, EPI_ISL_1212287, EPI_ISL_1212290, EPI_ISL_1212294, EPI_ISL_1214866, EPI_ISL_1214906, EPI_ISL_1214925, EPI_ISL_1214971, EPI_ISL_1214975, EPI_ISL_1214977, EPI_ISL_1217540, EPI_ISL_1217549, EPI_ISL_1281915, EPI_ISL_1281923, EPI_ISL_1281964 to 1281965, EPI_ISL_1281968, EPI_ISL_1281976, EPI_ISL_1285290, EPI_ISL_1285306, EPI_ISL_1285317, EPI_ISL_1285319, EPI_ISL_1285340			
see above	Labor Lübeck bzw. Laborärztliche Gemeinschaftspraxis Lübeck	Robert Koch Institute	
EPI_ISL_913063, EPI_ISL_913092	Labor Mustafa Wien	Center for Virology	Irene Goerzer; Jeremy V. Camp; Monika Redlberger-Fritz; Stephan W. Aberle
EPI_ISL_1153340, EPI_ISL_1153345, EPI_ISL_1153361, EPI_ISL_1153369, EPI_ISL_1153390, EPI_ISL_1153398, EPI_ISL_1153401, EPI_ISL_1153404, EPI_ISL_1153425, EPI_ISL_1153971, EPI_ISL_1153973, EPI_ISL_1153976, EPI_ISL_1153978, EPI_ISL_1154006, EPI_ISL_1155483, EPI_ISL_1155557, EPI_ISL_1155561, EPI_ISL_1157061, EPI_ISL_1215776, EPI_ISL_1217016 to 1217017, EPI_ISL_1217039, EPI_ISL_1217043, EPI_ISL_1217064, EPI_ISL_1217066, EPI_ISL_1217082, EPI_ISL_1217088, EPI_ISL_1217091, EPI_ISL_1283131, EPI_ISL_1283133 to 1283136, EPI_ISL_1283138, EPI_ISL_1283142, EPI_ISL_1283149 to 1283150, EPI_ISL_1283152, EPI_ISL_1283154, EPI_ISL_1283166, EPI_ISL_1283175, EPI_ISL_1283177, EPI_ISL_1283182, EPI_ISL_1283189, EPI_ISL_1283193, EPI_ISL_1283201, EPI_ISL_1283203, EPI_ISL_1283206, EPI_ISL_1283209, EPI_ISL_1283211, EPI_ISL_1283214, EPI_ISL_1283227, EPI_ISL_1283242, EPI_ISL_1283705,			

EPI_ISL_1283733, EPI_ISL_1283739, EPI_ISL_1283758, EPI_ISL_1283770, EPI_ISL_1283787, EPI_ISL_1283805, EPI_ISL_1283825, EPI_ISL_1283836, EPI_ISL_1283852, EPI_ISL_1286136 to 1286137			
see above	Labor MönchengladbachMVZ Dr. Stein + Kollegen GbR	Robert Koch Institute	
EPI_ISL_1285345, EPI_ISL_1285347, EPI_ISL_1285349 to 1285350, EPI_ISL_1285353, EPI_ISL_1285355, EPI_ISL_1285357 to 1285358, EPI_ISL_1285360, EPI_ISL_1285363, EPI_ISL_1285365, EPI_ISL_1285369 to 1285370, EPI_ISL_1285372 to 1285373, EPI_ISL_1285375 to 1285384			
see above	Labor Prof. Dr. G. Enders MVZ GbR	Robert Koch Institute	
EPI_ISL_831414 to 831416, EPI_ISL_1048252, EPI_ISL_1048258, EPI_ISL_1048260, EPI_ISL_1048267, EPI_ISL_1048295, EPI_ISL_1048350 to 1048351			
see above	Labor Prof. Dr. G. Enders MVZ GbR	Robert Koch Institute, Influenza and respiratory viruses FG17 & Bioinformatics MF1, Berlin, Germany	Aleksandar Radonic; Dr. Friedemann Tewald; F. Tewald; Marianne Wedde; Oliver Drechsel; Ralf Duerrwald; Rene Kmiecinski; Stefan Kroeger; Stephan Fuchs; Thorsten Wolff
EPI_ISL_1140190 to 1140388, EPI_ISL_1140390 to 1140422, EPI_ISL_1140427 to 1140456, EPI_ISL_1140459 to 1140477, EPI_ISL_1143555 to 1143567, EPI_ISL_1143847 to 1143881, EPI_ISL_1143883 to 1143915, EPI_ISL_1211903 to 1211905, EPI_ISL_1211909, EPI_ISL_1211912, EPI_ISL_1211914 to 1211921, EPI_ISL_1211924 to 1211927, EPI_ISL_1211929, EPI_ISL_1211931 to 1211932, EPI_ISL_1212417 to 1212419, EPI_ISL_1212422 to 1212426, EPI_ISL_1212428, EPI_ISL_1212430, EPI_ISL_1212432 to 1212434, EPI_ISL_1212436, EPI_ISL_1212438, EPI_ISL_1212440, EPI_ISL_1212443, EPI_ISL_1212445 to 1212450, EPI_ISL_1212452 to 1212458, EPI_ISL_1212461 to 1212462, EPI_ISL_1212465 to 1212467, EPI_ISL_1212469 to 1212472, EPI_ISL_1212476 to 1212478, EPI_ISL_1212480 to 1212488, EPI_ISL_1212490 to 1212499, EPI_ISL_1218111 to 1218112, EPI_ISL_1218114, EPI_ISL_1218118, EPI_ISL_1218122 to 1218124, EPI_ISL_1218126, EPI_ISL_1218128 to 1218131, EPI_ISL_1218133, EPI_ISL_1218137, EPI_ISL_1218139, EPI_ISL_1218141 to 1218145, EPI_ISL_1218149 to 1218152, EPI_ISL_1218154 to 1218156, EPI_ISL_1218159 to 1218160, EPI_ISL_1218162 to 1218163, EPI_ISL_1218165 to 1218166, EPI_ISL_1218169 to 1218174, EPI_ISL_1218176 to 1218178, EPI_ISL_1218180 to 1218182, EPI_ISL_1218186 to 1218187, EPI_ISL_1282836, EPI_ISL_1282838, EPI_ISL_1282842 to 1282843, EPI_ISL_1282845 to 1282851, EPI_ISL_1282853 to 1282854, EPI_ISL_1282857, EPI_ISL_1282859, EPI_ISL_1282861, EPI_ISL_1282864, EPI_ISL_1282866 to 1282867, EPI_ISL_1282869, EPI_ISL_1282871 to 1282875, EPI_ISL_1282879, EPI_ISL_1282881, EPI_ISL_1282885, EPI_ISL_1282888 to 1282891, EPI_ISL_1282894 to 1282895, EPI_ISL_1282897, EPI_ISL_1282899 to 1282901, EPI_ISL_1282908 to 1282913, EPI_ISL_1282915 to 1282916, EPI_ISL_1282918 to 1282919, EPI_ISL_1282922, EPI_ISL_1282924, EPI_ISL_1282927, EPI_ISL_1282930, EPI_ISL_1282932, EPI_ISL_1282936, EPI_ISL_1282942 to 1282944, EPI_ISL_1282950 to 1282951, EPI_ISL_1282953 to 1282955, EPI_ISL_1282957, EPI_ISL_1282960, EPI_ISL_1282962 to 1282965, EPI_ISL_1285178 to 1285179, EPI_ISL_1285181 to 1285182, EPI_ISL_1285184, EPI_ISL_1285190 to 1285193, EPI_ISL_1285195, EPI_ISL_1285197, EPI_ISL_1285200, EPI_ISL_1285203, EPI_ISL_1285209 to 1285210, EPI_ISL_1285212, EPI_ISL_1285215, EPI_ISL_1285217, EPI_ISL_1285219, EPI_ISL_1285222 to 1285223, EPI_ISL_1285225, EPI_ISL_1285228, EPI_ISL_1285232 to 1285234, EPI_ISL_1285240, EPI_ISL_1285243, EPI_ISL_1285245, EPI_ISL_1285248, EPI_ISL_1285250, EPI_ISL_1285258, EPI_ISL_1285262 to 1285264, EPI_ISL_1285266 to 1285267, EPI_ISL_1285269 to 1285271, EPI_ISL_1285273 to 1285276, EPI_ISL_1285280			
see above	Labor ZOTZ|KLIMAS; MVZ Düsseldorf-Centrum	Robert Koch Institute	
EPI_ISL_1151430, EPI_ISL_1151497, EPI_ISL_1151512, EPI_ISL_1151563, EPI_ISL_1151616, EPI_ISL_1151661, EPI_ISL_1151674, EPI_ISL_1151676, EPI_ISL_1151683, EPI_ISL_1151690, EPI_ISL_1151692, EPI_ISL_1151720, EPI_ISL_1151732, EPI_ISL_1152624, EPI_ISL_1152644, EPI_ISL_1152657, EPI_ISL_1152660, EPI_ISL_1152763, EPI_ISL_1152776, EPI_ISL_1152791, EPI_ISL_1152842 to 1152843, EPI_ISL_1213592, EPI_ISL_1213666, EPI_ISL_1213671, EPI_ISL_1213676, EPI_ISL_1213716, EPI_ISL_1213724, EPI_ISL_1213729, EPI_ISL_1213732, EPI_ISL_1213758, EPI_ISL_1213761, EPI_ISL_1213811, EPI_ISL_1213839, EPI_ISL_1213855, EPI_ISL_1213860, EPI_ISL_1213908, EPI_ISL_1213914 to 1213915, EPI_ISL_1213924, EPI_ISL_1213975, EPI_ISL_1213988, EPI_ISL_1214007, EPI_ISL_1214038, EPI_ISL_1214046, EPI_ISL_1214087, EPI_ISL_1218301, EPI_ISL_1218307, EPI_ISL_1218314, EPI_ISL_1218332, EPI_ISL_1280789, EPI_ISL_1280807, EPI_ISL_1280830, EPI_ISL_1280867, EPI_ISL_1280871, EPI_ISL_1280879, EPI_ISL_1280897			
see above	Labor für medizinische Mikrobiologie Bakteriologie Mykologie Virologie & Infektionsserologie	Robert Koch Institute	
EPI_ISL_445220	Labolarory for Respiratory Viruses, "Cantacuzino" National Military-Medical Institute for Resararch and Development	Cantacuzino Institute	A.Cretu; L.Ustea; M.Lazar
EPI_ISL_981372	Labolarory for Respiratory Viruses, "Cantacuzino" National Military-Medical Institute for Resararch and Development	Cantacuzino Institute Virology	Catalina Pascu; Luiza Ustea; Mihaela Lazar; Nicoleta Paraschiv
EPI_ISL_768818	Laboratoire 2A2B	CNR Virus des Infections Respiratoires - France SUD	Antonin Bal; Bruno Lina; Gregory Destras; Gwendolyne Burfin; Hadrien Règue; Laurence Josset; Martine Valette; Quentin Semanas
EPI_ISL_768825 to 768826	Laboratoire AX Bio Ocean	CNR Virus des Infections Respiratoires - France SUD	Antonin Bal; Bruno Lina; Gregory Destras; Gwendolyne Burfin; Hadrien Règue; Laurence Josset; Martine Valette; Quentin Semanas
EPI_ISL_768824	Laboratoire BIOLAB33	CNR Virus des Infections Respiratoires - France SUD	Antonin Bal; Bruno Lina; Gregory Destras; Gwendolyne Burfin; Hadrien Règue; Laurence Josset; Martine Valette; Quentin Semanas
EPI_ISL_768830, EPI_ISL_779848 to 779849	Laboratoire BIOMED 05	CNR Virus des Infections Respiratoires - France SUD	Antonin Bal; Bruno Lina; Gregory Destras; Gwendolyne Burfin; Hadrien Règue; Laurence Josset; Martine Valette; Quentin Semanas
EPI_ISL_768819 to 768820	Laboratoire BPO-BIOEPINE	CNR Virus des Infections Respiratoires - France SUD	Antonin Bal; Bruno Lina; Gregory Destras; Gwendolyne Burfin; Hadrien Règue; Laurence Josset; Martine Valette; Quentin Semanas
EPI_ISL_723469, EPI_ISL_728219, EPI_ISL_728272 to 728277, EPI_ISL_728289 to 728291, EPI_ISL_728295, EPI_ISL_728297, EPI_ISL_728301, EPI_ISL_728322, EPI_ISL_728332, EPI_ISL_728334, EPI_ISL_728336, EPI_ISL_728339 to 728340, EPI_ISL_728342, EPI_ISL_728344, EPI_ISL_728347, EPI_ISL_728349, EPI_ISL_728352 to 728353, EPI_ISL_728355, EPI_ISL_728359 to 728360, EPI_ISL_728363, EPI_ISL_728366 to 728367, EPI_ISL_768831 to 768832, EPI_ISL_768834 to 768840, EPI_ISL_769863, EPI_ISL_775219 to 775226, EPI_ISL_775247, EPI_ISL_775249 to 775250, EPI_ISL_775252, EPI_ISL_775254 to 775256, EPI_ISL_775258, EPI_ISL_775260, EPI_ISL_775262 to 775265, EPI_ISL_778870			
see above	Laboratoire Biolife	Laboratoire de Biotechnologie	Afaf Alaoui; Amina Benouda; Bouchra Belfquih; Hanae Dakka; Lahcen Belyamani; Lahcen belyamani; Lahecn Belyamani; Mohamed Chenaoui; Mohammed Walid Chemao Elfihri; Mouna Ouadghiri; Otmane Touzani; Saaid Amzazi and Azeddine Ibrahim; Saaid Amzazi and Azeddine Ibrahimi; Tarik Aanniz
EPI_ISL_912563 to 912564, EPI_ISL_912574	Laboratoire Central - American Hospital of Paris	National Reference Center for Viruses of Respiratory Infections, Institut Pasteur, Paris	Angela Brisebarre; Camille Capel; Etienne Simon-Lorière; Marion Barbet; Maud Vanpeene; Méline Bizard; Ramirez José-Manuel; Sylvie Behillil; Sylvie van der Werf; Vincent Enouf
EPI_ISL_1009627	Laboratoire Cerballiance Bretigny	Cerba	Benazra M; Collin T; Delaplace E; Gresset N; Haim-Boukobza S; Lecorche E; Lesenne A; Olivi M; Ricard B; Roquebert B; Trombert-Paolantoni S
EPI_ISL_1001459	Laboratoire Cerballiance Port de Bouc	Cerba	Haim-Boukobza S; Lecorche E; Meyer S; Roig JC; Roquebert B; Trombert-Paolantoni S
EPI_ISL_1001464	Laboratoire Cerballiance du pole santé	Cerba	Haim-Boukobza S; Lecorche E; Meyer S; Roig JC; Roquebert B; Trombert-Paolantoni S
EPI_ISL_1250623 to 1250624	Laboratoire D Lab	Centre Hospitalier Universitaire de Rouen Laboratoire de Virologie	Alice Moisan; Fabienne De Oliveira; Marie Leoz
EPI_ISL_1259250, EPI_ISL_1259252, EPI_ISL_1259270, EPI_ISL_1279944	Laboratoire Hospitalier Universitaire de Bruxelles (LHUB-ULB)	UAntwerp, Laboratory of Medical Microbiology	Basil Britto Xavier; Christine Lammens; Herman Goossens; Jasmine Coppens; Marie Le Mercier; Veerle Matheeussen
EPI_ISL_768817, EPI_ISL_779836	Laboratoire Mirialis	CNR Virus des Infections Respiratoires - France SUD	Antonin Bal; Bruno Lina; Gregory Destras; Gwendolyne Burfin; Hadrien Règue; Laurence Josset; Martine Valette; Quentin Semanas
EPI_ISL_421734 to 421763, EPI_ISL_428939 to 428962, EPI_ISL_429706 to 429791, EPI_ISL_429793 to 429800, EPI_ISL_434487 to 434515, EPI_ISL_445053 to 445076, EPI_ISL_459893 to 459906			
see above	Laboratoire National de Sante, Microbiology, Virology	Laboratoire National de Sante, Microbiology, Epidemiology and Microbial Genomics	Anke Wienecke-Baldacchino; Ardashel Latsuzbaia; Catherine Ragimbeau; Guillaume Fournier; Jessica Tapp; Joel Mossong; Tamir Abdelrahman; Trung Nguyen Nguyen
EPI_ISL_413593	Laboratoire National de Santé	Erasmus Medical Center	Anne van der Linden; Annemiek van der Eijk; Bas Oude Munnink; Claudia Schapendonk; Corine GeurtsvanKessel; David Nieuwenhuijse; G. Fournier; Irina Chestakova; J. Mossong; Jeroen van Kampen; Jolanda Voermans; Marion Koopmans; Mark Pronk; Pascal Lexmond; Reina Sikkema; Richard Molenkamp; T. Abdelrahman; T. Nguyen; on behalf of the Dutch national COVID-19 response team.
EPI_ISL_419562 to 419607	Laboratoire National de Santé, Microbiology, Virology	Laboratoire National de Santé, Microbiology, Epidemiology and Microbial Genomics	Anke Wienecke-Baldacchino; Ardashel Latsuzbaia; Catherine Ragimbeau; Guillaume Fournier; Jessica Tapp; Joel Mossong; Tamir Abdelrahman; Trung Nguyen Nguyen
EPI_ISL_417526 to 417534	Laboratoire Nationale de Santé, Microbiology, Virology	Laboratoire Nationale de Santé, Microbiology, Epidemiology and Microbial Genomics	Anke Wienecke-Baldacchino; Ardashel Latsuzbaia; Catherine Ragimbeau; Guillaume Fournier; Jessica Tapp; Joel Mossong; Tamir Abdelrahman; Trung Nguyen Nguyen
EPI_ISL_768821	Laboratoire Novabio	CNR Virus des Infections Respiratoires - France SUD	Antonin Bal; Bruno Lina; Gregory Destras; Gwendolyne Burfin; Hadrien Règue; Laurence Josset; Martine Valette; Quentin Semanas
EPI_ISL_1158547 to 1158548, EPI_ISL_1158579 to 1158580, EPI_ISL_1158928, EPI_ISL_1159069, EPI_ISL_1159107, EPI_ISL_1159126, EPI_ISL_1159129 to 1159130, EPI_ISL_1159229, EPI_ISL_1159231 to 1159232			
see above	Laboratoire Vivalia - Cliniques St-Joseph	Plateforme de testing Namuroise	Degosserie Jonathan; Denis Olivier; Maschietto Céline; Mullier François; Otto Gaetan; Technologue
EPI_ISL_1158578	Laboratoire Vivalia -Cliniques St-Joseph	Plateforme de testing Namuroise	Degosserie Jonathan; Denis Olivier; Maschietto Céline; Mullier François; Otto Gaetan
EPI_ISL_1013438 to 1013449	Laboratoire associé au CNR des Virus Influenzae	National Reference Center for Viruses of Respiratory Infections, Institut Pasteur, Paris	Angela Brisebarre; Camille Capel; Etienne Simon-Lorière; Marion Barbet; Maud Vanpeene; Méline Bizard; Rousset (Guy) Dominique; Sylvie Behillil; Sylvie van der Werf; Vincent Enouf
EPI_ISL_1103576, EPI_ISL_1107189, EPI_ISL_1109622 to 1109623, EPI_ISL_1116465 to 1116466, EPI_ISL_1116470, EPI_ISL_1116555, EPI_ISL_1116592, EPI_ISL_1117289, EPI_ISL_1120732, EPI_ISL_1137621, EPI_ISL_1159697 to 1159700			
see above	Laboratoire central de Virologie	Laboratoire de Biotechnologie	Abdelomunim Essabbar; Amal Zouaki; Ghizlane EL Amin; Hakima Kabbaj; Lahcen Belyamani and Azeddine Ibrahim; Lahcen Belyamani and Azeddine Ibrahimi; Mouna Ouadghiri; Myriam Seffar; Saaïd Amzazi; Tarik Aanniz
EPI_ISL_1103584	Laboratoire central de Virologie, Centre hospitalo-universitaire Ibn Sina, Hôpital des spécialités	Laboratoire de Biotechnologie	Abdelomunim Essabbar; Amal Zouaki; Ghizlane EL Amin; Hakima Kabbaj; Lahcen Belyamani and Azeddine Ibrahimi; Mouna Ouadghiri; Myriam Seffar; Saaïd Amzazi; Tarik Aanniz

EPI_ISL_1105946	Laboratoire central de Virologie, Centre hospitalo-universitaire Ibn Sina Hôpital des spécialités	Laboratoire de Biotechnologie	Abdelomunim Essabbar; Amal Zouaki; Ghizlane EL Amin; Hakima Kabbaj; Lahcen Belyamani and Azeddine Ibrahimi; Mouna Ouadghiri; Myriam Seffar; Saaïd Amzazi; Tarik Aanniz
EPI_ISL_1103579	Laboratoire central de Virologie, Centre hospitalo-universitaire Ibn Sina, Hôpital des spécialités	Laboratoire de Biotechnologie	Abdelomunim Essabbar; Amal Zouaki; Ghizlane EL Amin; Hakima Kabbaj; Lahcen Belyamani and Azeddine Ibrahimi; Mouna Ouadghiri; Myriam Seffar; Saaïd Amzazi; Tarik Aanniz
EPI_ISL_1103569	Laboratoire central de Virologie, Centre hospitalo-universitaire Ibn Sina, Hopital des spécialités, Rabat	Laboratoire de Biotechnologie	Abdelomunim Essabbar; Amal Zouaki; Ghizlane EL Amin; Hakima Kabbaj; Lahcen Belyamani and Azeddine Ibrahimi; Mouna Ouadghiri; Myriam Seffar; Saaïd Amzazi; Tarik Aanniz
EPI_ISL_754141 to 754172, EPI_ISL_1013218 to 1013221	Laboratoire de Microbiologie	National Reference Center for Viruses of Respiratory Infections, Institut Pasteur, Paris	Angela Brisebarre; Camille Capel; Etienne Simon-Lorière; Marie-Sarah Fangous; Marion Barbet; Martres Pascale; Maud Vanpeene; Méline Bizard; Sylvie Behillil; Sylvie van der Werf; Vincent Enouf
EPI_ISL_443284 to 443288	Laboratoire de Microbiologie - Bât A - CH René Dubois	National Reference Center for Viruses of Respiratory Infections, Institut Pasteur, Paris	Angela Brisebarre; Etienne Simon-Lorière; Flora Donati; Marion Barbet; Maud Vanpeene; Mélanie Albert; Méline Bizard; Pascale Martres; Sylvie Behillil; Sylvie van der Werf; Vincent Enouf
EPI_ISL_660445 to 660528	Laboratoire de Microbiologie CHU Souro Sanou	Centre Muraz	Abdoul-Salam Ouedraogo; Arsène Zongo; Essia Belarbi; Fabian Leendertz; Grit Schubert; Halidou Tinto; Lassana Sangaré; Soumeya Ouangraoua; Yacouba Sawadogo; Zekiba Tarnagda
EPI_ISL_1116464, EPI_ISL_1116467 to 1116469, EPI_ISL_1118675, EPI_ISL_1118884, EPI_ISL_1118923, EPI_ISL_1118926, EPI_ISL_1138747			
see above	Laboratoire de Microbiologie- CHU Habib Bourguiba - Sfax	Laboratoire des Procédés de Criblage Moléculaire et Cellulaire-Centre de Biotechnologie de Sfax	A. and Masmoudi, S.; Abdelmoulah, F.; Abid, N.; Ajili, F.; Aouni, M.; Ben Ayed, I.; Bensaid, M.; Chtourou, A.; Elargoubi, A.; Fki-berrajah, L.; Gaaloul, I.; Gargouri, S.; Hammami, A.; Kamoun, S.; Karray Hakim, H.; Kharat, N.; Mastouri, M.; Mhalla, S.; Nabli, A.; Rebai; Smeti, I.; Souissi, A.; Stambouli, N.; Turki, M.
EPI_ISL_712062 to 712069, EPI_ISL_712568	Laboratoire de Microbiologie- CHU Habib Bourguiba - Sfax adresse	Laboratoire des Procédés de Criblage Moléculaire et Cellulaire-Centre de Biotechnologie de Sfax	A. and Masmoudi, S.; Abdelmoulah, F.; Abid, N.; Ajili, F.; Aouni, M.; Ben Ayed, I.; Bensaid, M.; Chtourou, A.; Elargoubi, A.; Fki-berrajah, L.; Gaaloul, I.; Gargouri, S.; Hammami, A.; Kamoun, S.; Karray Hakim, H.; Kharat, N.; Mastouri, M.; Mhalla, S.; Nabli, A.; Rebai; Smeti, I.; Souissi, A.; Stambouli, N.; Turki, M.
EPI_ISL_451400	Laboratoire de Recherche et d'Analyse Médicale de la Gendarmerie Royale	Laboratoire de Recherche et d'Analyse Médicale de la Gendarmerie Royale	Amal SOUIRI; Sanaâ LEMRISS; Saâd EL KABBAJ
EPI_ISL_933715, EPI_ISL_933719	Laboratoire de Recherche et d'Analyses Medicales de la Gendarmerie Royale	Laboratoire de Recherche et d'Analyses Medicales de la Gendarmerie Royale	Amal Souiri; Mohammed Labioui; Momammed Labioui; Nabil Lemzaoui; S. El kabbaj; Sanaâ Lemriss; Saâd El kabbaj; elmostafa El Fahime
EPI_ISL_476024 to 476026	Laboratoire de Recherche et d'Analyses Médicales de la Gendarmerie Royale	Laboratoire de Recherche et d'Analyses Médicales de la Gendarmerie Royale	Amal SOUIRI; Amal Souiri; Ayoub Jibjibe; Mahmoud Yartaoui; Marouane El Rhouila; Mohamed Chahmi; Mohamed Labioui; Nabil Lemzaoui; Nabil Ouaariba; Nadia Kandoussi; Omar Mestoui; Samiha Sellak; Sanaâ LEMRISS; Sanaâ Lemriss; Saâd EL KABBAJ; Saâd El Kabbaj.
EPI_ISL_754843, EPI_ISL_754845	Laboratoire de Virologie Hôpital Robert Debré	National Reference Center for Viruses of Respiratory Infections, Institut Pasteur, Paris	Andreoletti Laurent; Angela Brisebarre; Camille Capel; Etienne Simon-Lorière; Marion Barbet; Maud Vanpeene; Méline Bizard; Sylvie Behillil; Sylvie van der Werf; Vincent Enouf
EPI_ISL_792000 to 792007	Laboratoire de Virologie Hôpital Robert Debré	National Reference Center for Viruses of Respiratory Infections, Institut Pasteur, Paris	Andreoletti Laurent; Angela Brisebarre; Camille Capel; Etienne Simon-Lorière; Marion Barbet; Maud Vanpeene; Méline Bizard; Sylvie Behillil; Sylvie van der Werf; Vincent Enouf
EPI_ISL_414600, EPI_ISL_414623	Laboratoire de Virologie Institut de Virologie - INSERM U 1109 Hôpitaux Universitaires de Strasbourg	National Reference Center for Viruses of Respiratory Infections, Institut Pasteur, Paris	Angela Brisebarre; Flora Donati Vincent Enouf; Marion Barbet; Maud Vanpeene; Méline Bizard; Mélnie Albert; Samira Fafi-Kremer; Sylvie Behillil; Sylvie van der Werf
EPI_ISL_912565, EPI_ISL_912568, EPI_ISL_912575 to 912578, EPI_ISL_912602, EPI_ISL_913608 to 913610	Laboratoire de Virologie du CHU de Lille Bâtiment Paul Boulanger	National Reference Center for Viruses of Respiratory Infections, Institut Pasteur, Paris	Angela Brisebarre; Camille Capel; Etienne Simon-Lorière; Guigon Aurélie; Marion Barbet; Maud Vanpeene; Méline Bizard; Sylvie Behillil; Sylvie van der Werf; Vincent Enouf
EPI_ISL_666870	Laboratoire de Virologie, CHU de Caen, Normandie, France.	GRAM2.0, Université de Caen Normandie	Astrid VABRET; Estelle LEPERCHOIS; Januel VOUTEAU; Juliette ECHE; Meriadeg LE GOUIL
EPI_ISL_911513	Laboratoire de Virologie, CHU de Caen, Normandie, France.	GRAM2.0, Université de Caen Normandie Laboratoire de Virologie, CHU de Caen	Astrid VABRET; Estelle LEPERCHOIS; Januel VOUTEAU; Juliette ECHE; Meriadeg LE GOUIL
EPI_ISL_413996 to 413997, EPI_ISL_413999, EPI_ISL_414019 to 414023, EPI_ISL_548147 to 548242	Laboratoire de Virologie, HUG	Swiss National Reference Centre for Influenza	LAUBSCHER F.; LAUBSCHER Florian et al.
EPI_ISL_765479	Laboratoire de Virologie, Hôpital Robert Debré	National Reference Center for Viruses of Respiratory Infections, Institut Pasteur, Paris	Andreoletti Laurent; Angela Brisebarre; Camille Capel; Etienne Simon-Lorière; Marion Barbet; Maud Vanpeene; Méline Bizard; Sylvie Behillil; Sylvie van der Werf; Vincent Enouf
EPI_ISL_1190738	Laboratoire de biologie moléculaire - Plateforme Clinique	Laboratoire de biologie moléculaire - Plateforme Clinique	Bogaerts Pierre; Denis Olivier; Evrard Stephanie; Huang Te-Din; Mullier François; Rouze Pauline
EPI_ISL_1220862	Laboratoire de microbiologie CHU NIMES	Laboratoire de microbiologie CHU NIMES	Agathe Boudet; Marie-josée Carles; Sophie Bravo; Stephan Robin
EPI_ISL_435343 to 435347, EPI_ISL_444494 to 444516, EPI_ISL_450638 to 450653, EPI_ISL_463903 to 463969, EPI_ISL_476498 to 476513, EPI_ISL_515197 to 515246	Laboratoire de microbiologie, Hopital de Verdun	Smith Laboratory, Centre de Recherche CHU Sainte-Justine	Ivan Pavlov; Marieke Rozendaal; Martin Smith
EPI_ISL_825634 to 825712, EPI_ISL_825715 to 825806, EPI_ISL_825808 to 825948, EPI_ISL_825950 to 826268, EPI_ISL_852834 to 852841, EPI_ISL_853405 to 853415, EPI_ISL_889802 to 890173, EPI_ISL_890371 to 890888, EPI_ISL_954302 to 954610, EPI_ISL_954612 to 954744, EPI_ISL_1001069 to 1001108, EPI_ISL_1004175 to 1004177, EPI_ISL_1009129 to 1009158, EPI_ISL_1167298 to 1167307, EPI_ISL_1241005 to 1241278, EPI_ISL_1241281 to 1241282, EPI_ISL_1241284 to 1241385, EPI_ISL_1241387 to 1241679, EPI_ISL_1314968 to 1314969			
see above	Laboratoire de santé publique du Québec	Laboratoire de santé publique du Québec	Guillaume Bourque; Ioannis Ragoussis; Jesse Shapiro; Mark Lathrop and Michel Roger on behalf of the CoVSeQ research group; Mark Lathrop and Michel Roger on behalf of the CoVSeQ research group (http://covseq.ca/researchgroup); Sandrine Moreira
EPI_ISL_1197037, EPI_ISL_1208399	Laboratoire de virologie clinique - Institut Pasteur de Tunis	1-Laboratory of Microbiology, National Reference Lab, Charles Nicolle Hospital; 2-University of Tunis ElManar, Faculty of Medicine of Tunis, LR99ES09, Tunis, Tunisia	Alia BenKahla; Anissa Chouikha; Fares Wasfi; Henda Triki; Ilhem Boutiba-Ben Boubaker.; Imen Kacem; Ines Mdini; Jalila Ben Khelil; Maher Kharrat; Manel Ben Sassi; Mariem Gdoura; Mouna Ben Sassi; Mouna Safer; Nissaf Ben Alaya; Riadh Daghfous; Riadh Gouider; Roua Ben Othman; Salma Abid; Sameh Trabelsi; Sana Ferjani; Sara Chammam; Sondes Haddad
EPI_ISL_779781 to 779782, EPI_ISL_779814 to 779835	Laboratoire de virologie, CHU de Grenoble	CNR Virus des Infections Respiratoires - France SUD	Antonin Bal; Bruno Lina; Gregory Destras; Gwendolyne Burfin; Hadrien Règue; Laurence Josset; Martine Valette; Quentin Semanas; Sylvie Larrat
EPI_ISL_634818, EPI_ISL_700349	Laboratoire de virologie, CHU de Grenoble - CS 10217 - 38043 Grenoble cedex 10	CNR Virus des Infections Respiratoires - France SUD	Antonin Bal; Bruno Lina; Gregory Destras; Gwendolyne Burfin; Hadrien Règue; Laurence Josset; Martine Valette; Quentin Semanas; Sylvie Larrat
EPI_ISL_634819, EPI_ISL_700350	Laboratoire de virologie, CHU de Grenoble - CS 10217 - 38043 Grenoble cedex 11	CNR Virus des Infections Respiratoires - France SUD	Antonin Bal; Bruno Lina; Gregory Destras; Gwendolyne Burfin; Hadrien Règue; Laurence Josset; Martine Valette; Quentin Semanas; Sylvie Larrat
EPI_ISL_634820, EPI_ISL_700351	Laboratoire de virologie, CHU de Grenoble - CS 10217 - 38043 Grenoble cedex 12	CNR Virus des Infections Respiratoires - France SUD	Antonin Bal; Bruno Lina; Gregory Destras; Gwendolyne Burfin; Hadrien Règue; Laurence Josset; Martine Valette; Quentin Semanas; Sylvie Larrat
EPI_ISL_634821, EPI_ISL_700352	Laboratoire de virologie, CHU de Grenoble - CS 10217 - 38043 Grenoble cedex 13	CNR Virus des Infections Respiratoires - France SUD	Antonin Bal; Bruno Lina; Gregory Destras; Gwendolyne Burfin; Hadrien Règue; Laurence Josset; Martine Valette; Quentin Semanas; Sylvie Larrat
EPI_ISL_634822, EPI_ISL_700353	Laboratoire de virologie, CHU de Grenoble - CS 10217 - 38043 Grenoble cedex 14	CNR Virus des Infections Respiratoires - France SUD	Antonin Bal; Bruno Lina; Gregory Destras; Gwendolyne Burfin; Hadrien Règue; Laurence Josset; Martine Valette; Quentin Semanas; Sylvie Larrat
EPI_ISL_634823, EPI_ISL_700354	Laboratoire de virologie, CHU de Grenoble - CS 10217 - 38043 Grenoble cedex 15	CNR Virus des Infections Respiratoires - France SUD	Antonin Bal; Bruno Lina; Gregory Destras; Gwendolyne Burfin; Hadrien Règue; Laurence Josset; Martine Valette; Quentin Semanas; Sylvie Larrat
EPI_ISL_634824, EPI_ISL_700355	Laboratoire de virologie, CHU de Grenoble - CS 10217 - 38043 Grenoble cedex 16	CNR Virus des Infections Respiratoires - France SUD	Antonin Bal; Bruno Lina; Gregory Destras; Gwendolyne Burfin; Hadrien Règue; Laurence Josset; Martine Valette; Quentin Semanas; Sylvie Larrat

EPI_ISL_634825, EPI_ISL_700356	Laboratoire de virologie, CHU de Grenoble - CS 10217 - 38043 Grenoble cedex 17	CNR Virus des Infections Respiratoires - France SUD	Antonin Bal; Bruno Lina; Gregory Destras; Gwendolyne Burfin; Hadrien Règue; Laurence Josset; Martine Valette; Quentin Semanas; Sylvie Larrat
EPI_ISL_634826, EPI_ISL_700357	Laboratoire de virologie, CHU de Grenoble - CS 10217 - 38043 Grenoble cedex 18	CNR Virus des Infections Respiratoires - France SUD	Antonin Bal; Bruno Lina; Gregory Destras; Gwendolyne Burfin; Hadrien Règue; Laurence Josset; Martine Valette; Quentin Semanas; Sylvie Larrat
EPI_ISL_634827, EPI_ISL_700358	Laboratoire de virologie, CHU de Grenoble - CS 10217 - 38043 Grenoble cedex 19	CNR Virus des Infections Respiratoires - France SUD	Antonin Bal; Bruno Lina; Gregory Destras; Gwendolyne Burfin; Hadrien Règue; Laurence Josset; Martine Valette; Quentin Semanas; Sylvie Larrat
EPI_ISL_634828, EPI_ISL_700359	Laboratoire de virologie, CHU de Grenoble - CS 10217 - 38043 Grenoble cedex 20	CNR Virus des Infections Respiratoires - France SUD	Antonin Bal; Bruno Lina; Gregory Destras; Gwendolyne Burfin; Hadrien Règue; Laurence Josset; Martine Valette; Quentin Semanas; Sylvie Larrat
EPI_ISL_634829, EPI_ISL_700360	Laboratoire de virologie, CHU de Grenoble - CS 10217 - 38043 Grenoble cedex 21	CNR Virus des Infections Respiratoires - France SUD	Antonin Bal; Bruno Lina; Gregory Destras; Gwendolyne Burfin; Hadrien Règue; Laurence Josset; Martine Valette; Quentin Semanas; Sylvie Larrat
EPI_ISL_634830, EPI_ISL_700361	Laboratoire de virologie, CHU de Grenoble - CS 10217 - 38043 Grenoble cedex 22	CNR Virus des Infections Respiratoires - France SUD	Antonin Bal; Bruno Lina; Gregory Destras; Gwendolyne Burfin; Hadrien Règue; Laurence Josset; Martine Valette; Quentin Semanas; Sylvie Larrat
EPI_ISL_634831, EPI_ISL_700362	Laboratoire de virologie, CHU de Grenoble - CS 10217 - 38043 Grenoble cedex 23	CNR Virus des Infections Respiratoires - France SUD	Antonin Bal; Bruno Lina; Gregory Destras; Gwendolyne Burfin; Hadrien Règue; Laurence Josset; Martine Valette; Quentin Semanas; Sylvie Larrat
EPI_ISL_634832, EPI_ISL_700363	Laboratoire de virologie, CHU de Grenoble - CS 10217 - 38043 Grenoble cedex 24	CNR Virus des Infections Respiratoires - France SUD	Antonin Bal; Bruno Lina; Gregory Destras; Gwendolyne Burfin; Hadrien Règue; Laurence Josset; Martine Valette; Quentin Semanas; Sylvie Larrat
EPI_ISL_634833, EPI_ISL_700364	Laboratoire de virologie, CHU de Grenoble - CS 10217 - 38043 Grenoble cedex 25	CNR Virus des Infections Respiratoires - France SUD	Antonin Bal; Bruno Lina; Gregory Destras; Gwendolyne Burfin; Hadrien Règue; Laurence Josset; Martine Valette; Quentin Semanas; Sylvie Larrat
EPI_ISL_634834, EPI_ISL_700365	Laboratoire de virologie, CHU de Grenoble - CS 10217 - 38043 Grenoble cedex 26	CNR Virus des Infections Respiratoires - France SUD	Antonin Bal; Bruno Lina; Gregory Destras; Gwendolyne Burfin; Hadrien Règue; Laurence Josset; Martine Valette; Quentin Semanas; Sylvie Larrat
EPI_ISL_634835, EPI_ISL_700366	Laboratoire de virologie, CHU de Grenoble - CS 10217 - 38043 Grenoble cedex 27	CNR Virus des Infections Respiratoires - France SUD	Antonin Bal; Bruno Lina; Gregory Destras; Gwendolyne Burfin; Hadrien Règue; Laurence Josset; Martine Valette; Quentin Semanas; Sylvie Larrat
EPI_ISL_634836, EPI_ISL_700367	Laboratoire de virologie, CHU de Grenoble - CS 10217 - 38043 Grenoble cedex 28	CNR Virus des Infections Respiratoires - France SUD	Antonin Bal; Bruno Lina; Gregory Destras; Gwendolyne Burfin; Hadrien Règue; Laurence Josset; Martine Valette; Quentin Semanas; Sylvie Larrat
EPI_ISL_634837, EPI_ISL_700368	Laboratoire de virologie, CHU de Grenoble - CS 10217 - 38043 Grenoble cedex 29	CNR Virus des Infections Respiratoires - France SUD	Antonin Bal; Bruno Lina; Gregory Destras; Gwendolyne Burfin; Hadrien Règue; Laurence Josset; Martine Valette; Quentin Semanas; Sylvie Larrat
EPI_ISL_700369	Laboratoire de virologie, CHU de Grenoble - CS 10217 - 38043 Grenoble cedex 30	CNR Virus des Infections Respiratoires - France SUD	Antonin Bal; Bruno Lina; Gregory Destras; Gwendolyne Burfin; Hadrien Règue; Laurence Josset; Martine Valette; Quentin Semanas; Sylvie Larrat
EPI_ISL_700370	Laboratoire de virologie, CHU de Grenoble - CS 10217 - 38043 Grenoble cedex 31	CNR Virus des Infections Respiratoires - France SUD	Antonin Bal; Bruno Lina; Gregory Destras; Gwendolyne Burfin; Hadrien Règue; Laurence Josset; Martine Valette; Quentin Semanas; Sylvie Larrat
EPI_ISL_634817, EPI_ISL_700348, EPI_ISL_900510	Laboratoire de virologie, CHU de Grenoble - CS 10217 - 38043 Grenoble cedex 9	CNR Virus des Infections Respiratoires - France SUD	Antonin Bal; Bruno Lina; Gregory Destras; Gwendolyne Burfin; Hadrien Règue; Laurence Josset; Martine Valette; Quentin Semanas; Sylvie Larrat
EPI_ISL_476822 to 476831, EPI_ISL_476833 to 476834	Laboratoire des Fièvres Hémorragiques Virales du Benin	Charité-Universitätsmedizin Berlin	Anges; Drexler; Jan Felix; Moreira-Soto Andres; Sander Anna-Lena; Yadouleton
EPI_ISL_629081 to 629108, EPI_ISL_666653 to 666681, EPI_ISL_683331 to 683333	Laboratoire du Centre Hospitalier Annecy Genevois	CNR Virus des Infections Respiratoires - France SUD	Antonin Bal; Bruno Chanzy; Bruno Lina; Gregory Destras; Gwendolyne Burfin; Hadrien Règue; Hélène Petitprez; Laurence Josset; Martine Valette; Quentin Semanas
EPI_ISL_910342 to 911241	Laboratoire national de sante, Microbiology, Virology	Laboratoire national de sante, Microbiology, Microbial Genomics Platform	Anke Wienecke-Baldacchino; Catherine Ragimbeau; Fatu Djabi; Jessica Tapp; Lise Pignon; Raoul Salmon; Tamir Abdelrahman
EPI_ISL_739700, EPI_ISL_739880, EPI_ISL_740129, EPI_ISL_744295, EPI_ISL_744819, EPI_ISL_744830	Laboratoire national de santé, Microbiology, Virology	Laboratoire national de santé, Microbiology, Epidemiology and Microbial Genomics	Anke Wienecke-Baldacchino; Catherine Ragimbeau; Fatu Djabi; Jessica Tapp; Tamir Abdelrahman; Trung Nguyen Nguyen
EPI_ISL_739693 to 739699, EPI_ISL_739701 to 739711, EPI_ISL_739713, EPI_ISL_739715 to 739849, EPI_ISL_739851 to 739879, EPI_ISL_739881 to 739909, EPI_ISL_739911 to 739936, EPI_ISL_739938 to 739970, EPI_ISL_739972 to 740055, EPI_ISL_740057 to 740076, EPI_ISL_740078 to 740128, EPI_ISL_740130 to 740164, EPI_ISL_740166 to 740204, EPI_ISL_740206 to 740266, EPI_ISL_740268 to 740423, EPI_ISL_740425 to 740545, EPI_ISL_744135 to 744196, EPI_ISL_744198 to 744202, EPI_ISL_744204 to 744210, EPI_ISL_744212 to 744294, EPI_ISL_744296 to 744482, EPI_ISL_744484 to 744675, EPI_ISL_744677 to 744755, EPI_ISL_744757 to 744808, EPI_ISL_744810 to 744818, EPI_ISL_744820 to 744829, EPI_ISL_744831 to 744878, EPI_ISL_744880 to 744892, EPI_ISL_744894 to 745027, EPI_ISL_755589, EPI_ISL_770845 to 771150			
see above	Laboratoire national de santé, Microbiology, Virology	Laboratoire national de santé, Microbiology, Microbial Genomics Platform	Anke Wienecke-Baldacchino; Catherine Ragimbeau; Fatu Djabi; Jessica Tapp; Lise Pignon; Raoul Salmon; Tamir Abdelrahman
EPI_ISL_1058161 to 1058166	Laboratori de Referència de Catalunya	Hospital Universitari Vall d'Hebron - Vall d'Hebron Institut de Recerca	Andrés Antón; Ariadna Rando; Carla Castillo; Cristina Andrés; Damir Garcia-Cehic; Josep F Abril; Josep Quer; Juliana Esperalba; Maria Carmen Martin; Maria Gema Codina; Maria Piñana; Tomàs Pumarola
EPI_ISL_1194149 to 1194150, EPI_ISL_1194152 to 1194153, EPI_ISL_1194155 to 1194156, EPI_ISL_1194158 to 1194159, EPI_ISL_1194161, EPI_ISL_1194163 to 1194164, EPI_ISL_1194166			
see above	Laboratori de Referència de Catalunya	SeqCOVID-SPAIN consortium/IBV(CSIC)	Aida Esperanza Ramírez Marinero; Alba Vilas Basil; Eduardo Padilla León; Mireia Canal Aranda and SeqCOVID-SPAIN consortium
EPI_ISL_1313179	LaboratorieMedicinskt Centrum Gotland	Karolinska University Hospital	Annelie Bjerkner; Isak Sylvin; Jan Albert; Karolina Ininbergs; Lina Guerra Blomqvist; Lynda Eneh; Martin Ekman; Martina Wahlund; Robert Dyrdak; Sandra Broddesson; Tanja Normark; Tobias Allander; Valtteri Wirta; Zhibing Yun
EPI_ISL_429128 to 429135, EPI_ISL_430856 to 430859, EPI_ISL_434641 to 434642, EPI_ISL_450836 to 450837	Laboratoriemedicin	The Public Health Agency of Sweden	Anna Risberg; Anna-Malin Linde; Karin Tegmark-Wisell; Maria Lind Karlberg; Mia Brytting; Olov Svartstrom; Oskar Karlsson Lindsjo; Shaman Muradrasoli; Theresa Enkirch
EPI_ISL_534248, EPI_ISL_534252 to 534255, EPI_ISL_534257 to 534258	Laboratoriemedicin Vasternorrland	The Public Health Agency of Sweden	Anna Risberg; Anna-Malin Linde; Karin Tegmark-Wisell; Maria Lind Karlberg; Mattias Haukland; Mia Brytting; Olov Svartstrom; Oskar Karlsson Lindsjo; Petra Edquist; Reza Advani; Sandra Broddesson
EPI_ISL_661299 to 661300, EPI_ISL_676516 to 676517, EPI_ISL_676535 to 676540, EPI_ISL_766640 to 766641, EPI_ISL_831953 to 831957, EPI_ISL_831998 to 831999, EPI_ISL_913294, EPI_ISL_913318, EPI_ISL_934316 to 934321, EPI_ISL_1168094, EPI_ISL_1232007			
see above	Laboratoriemedicin, Klinisk mikrobiologi	The Public Health Agency of Sweden	Anna Risberg; Anna-Malin Linde; Carlo Berg; Department of Microbiology; Karin Tegmark-Wisell; Maria Lind Karlberg; Mattias Haukland; Mia Brytting; Noura Walai; Oskar Karlsson Lindsjo; Petra Edquist; Petra Holmstrom; Reza Advani; Sofia Stamouli; The Public Health Agency of Sweden
EPI_ISL_1166147 to 1166148, EPI_ISL_1296253 to 1296257, EPI_ISL_1296259 to 1296260, EPI_ISL_1299545 to 1299553, EPI_ISL_1299555	Laboratorio Analisi Osp. Città di Castello - Azienda USL Umbria1	Istituto Zooprofilattico Sperimentale dell'Abruzzo e Molise "G. Caporale"	Ancora M; Calistri P; Camilloni B; Cammà C; Curini V; Di Domenico M; Di Pasquale A; Lorusso A; Malagigi V; Mangone I; Marcacci M; Mencacci A; Puglia I; Rinaldi A; Savini G; Scialabba S; Tacconi P
EPI_ISL_906552	Laboratorio Angel Diagnostica	Instituto Nacional de Salud- Dirección de Investigación en Salud Pública, Universidad de los Andes- Applied genomics research group, Vicerrectoria de Investigación y Creación, Universidad de los Andes- Systems and Computing Engineering Department	Carlos Franco-Muñoz; Diego A. Álvarez-Díaz; Diego Andrés Prada; Gerardo Santamaría Jorge Duitama; Héctor Alejandro Ruiz-Moreno; Jhonnatan Reales-González; Jorge Ivan Diaz; Julian Naizaque; Katherine Laiton-Donato; Laura Natalia Gonzalez; Magdalena Wiesner; Marcela Mercado-Reyes; Maria T. Herrera-Sepúlveda; Martha Lucia Ospina Martinez; Mauricio Pacheco-Montealegre; Sheryll Corchuelo; Silvia Restrepo-Restrepo
EPI_ISL_430793 to 430794	Laboratorio Análisis Clínicos, Unidad de Servicios	Área de Secuenciación del Laboratorio de Virología del	AS; D; Goya; Guevara; LE; Lusso; MI; MS; Mistchenko; Nabaes Jodar; Natale; O; S; SM; Sanchez; Valinotto; Vicario; Viegas, M.

	Diagnósticos, Swiss Medical Group	Hospital de Niños Dr. Ricardo Gutierrez on behalf of 'Proyecto Argentino Interinstitucional de genomica de SARS-CoV-2' (PAIS Consortium)	
EPI_ISL_906553 to 906555	Laboratorio Bienestar	Instituto Nacional de Salud- Dirección de Investigación en Salud Pública, Universidad de los Andes- Applied genomics research group, Vicerrectoria de Investigación y Creación, Universidad de los Andes- Systems and Computing Engineering Department	Carlos Franco-Muñoz; Diego A. Álvarez-Díaz; Diego Andrés Prada; Gerardo Santamaría Jorge Duitama; Héctor Alejandro Ruiz-Moreno; Jhonnatan Reales-González; Jorge Ivan Diaz; Julian Naizaque; Katherine Laiton-Donato; Laura Natalia Gonzalez; Magdalena Wiesner; Marcela Mercado-Reyes; Maria T. Herrera-Sepúlveda; Martha Lucia Ospina Martinez; Mauricio Pacheco-Montealegre; Sheryll Corchuelo; Silvia Restrepo-Restrepo
EPI_ISL_965179	Laboratorio Biologia Molecolare SARS-COV2- UOC Laboratorio Analisi- Servizio Medicina di Laboratorio, Ospedale San Francesco - ATS-ASSLNUoro	LABORATORIO SPECIALISTICO UOC EMATOLOGIA- Ospedale San Francesco - ATS-ASSLNUoro	ASPRONI ROSANNA; FANCELLO TATIANA; FIAMMA MAURA; FLORE ANNA RITA. PALMAS ANGELO DOMENICO; IANA LO MAGLIO; MAMELI GIUSEPPE; MONNE MARIA ITRIA; PIRAS GIOVANNA; SANNA FILOMENA; SULIS VINCENZO; TOJA ALESSANDRO
EPI_ISL_458085	Laboratorio Biologia Molecolare Sars Cov2 - UOC Laboratorio Analisi - Servizio Medicina di Laboratorio , Ospedale "San Francesco" - ATS- ASSL Nuoro	Laboratorio specialistico UOC Ematologia - Ospedale "San Francesco" - ATS-ASSL Nuoro	Asproni Rosanna; Casu Gavino; Fancello Tatiana; Fiamma Maura; Floris Anna Rita; Lo Maglio Iana; Mameli Giuseppe.; Monne Maria Itria; Palmas Angelo Domenico; Piras Giovanna; Sanna Filomena; Sulis Vincenzo; Toja Alessandro
EPI_ISL_1085013	Laboratorio Biologia Molecolare Sars Cov2 - UOC Laboratorio Analisi - Servizio Medicina di Laboratorio, Ospedale "San Francesco" - ATS-ASSL Nuoro	Laboratorio Biologia Molecolare Sars Cov2 - UOC Laboratorio Analisi - Servizio Medicina di Laboratorio, Ospedale "San Francesco" - ATS-ASSL Nuoro	; Asproni Rosanna; Fiamma Maura; Lo Maglio Iana; Mameli Giuseppe; Monne Maria Itria; Palmas Angelo Domenico; Piras Giovanna
EPI_ISL_1173202	Laboratorio Biologia Molecolare Sars Cov2 - UOC Laboratorio Analisi - Servizio Medicina di Laboratorio, Ospedale "San Francesco" - ATS-ASSL Nuoro	Laboratorio Specialistico Ematologia, UOC Ematologia, Ospedale "San Francesco" - ATS-ASSL Nuoro	Asproni Rosanna; Carta Franco; Fancello Patrizia; Fiamma Maura; Lo Maglio Iana; Malune Paolo; Mameli Giuseppe; Monne Maria Itria; Palmas Angelo Domenico; Pira Giovanna; Piras Giovanna; Rosu Valentina; Serra Silvia
EPI_ISL_458084	Laboratorio Biologia Molecolare Sars Cov2 - UOC Laboratorio Analisi - Servizio Medicina di Laboratorio, Ospedale "San Francesco" - ATS-ASSL Nuoro	Laboratorio specialistico UOC Ematologia - Ospedale "San Francesco" - ATS-ASSL Nuoro	Asproni Rosanna; Casu Gavino; Fancello Tatiana; Fiamma Maura; Floris Anna Rita; Lo Maglio Iana; Mameli Giuseppe.; Monne Maria Itria; Palmas Angelo Domenico; Piras Giovanna; Sanna Filomena; Sulis Vincenzo; Toja Alessandro
EPI_ISL_613706, EPI_ISL_613710, EPI_ISL_613953, EPI_ISL_613955, EPI_ISL_614396 to 614398, EPI_ISL_614889, EPI_ISL_637107 to 637109, EPI_ISL_965028, EPI_ISL_965031, EPI_ISL_965114, EPI_ISL_1034916, EPI_ISL_1063912, EPI_ISL_1082253, EPI_ISL_1104650, EPI_ISL_1180141 to 1180144, EPI_ISL_1180250 to 1180253, EPI_ISL_1191141 to 1191145, EPI_ISL_1191736 to 1191739, EPI_ISL_1219707 to 1219710, EPI_ISL_1229144 to 1229149, EPI_ISL_1311860 to 1311863			
see above	Laboratorio Biologia Molecolare Sars Cov2 - UOC Laboratorio Analisi - Servizio Medicina di Laboratorio, Ospedale "San Francesco" - ATS-ASSL Nuoro	Laboratorio specialistico UOC Ematologia - Ospedale "San Francesco" - ATS-ASSL Nuoro	; Angelo Domenico Palmas; Asproni Rosanna; Casu Gavino; Fancello Tatiana; Fiamma Maura; Floris Anna Rita; Giovanna Piras; Giuseppe Mameli; Iana Lo Maglio; Lo Maglio Iana; Malune Paolo; Mameli Giuseppe; Mameli Giuseppe.; Maria Itria Monne; Maura Fiamma; Monne Maria Itria; Palmas Angelo Domenico; Paolo Malune; Piras Giovanna; Rosanna Asproni; Sanna Filomena; Sulis Vincenzo; Toja Alessandro
EPI_ISL_613560	Laboratorio Biologia Molecolare Sars Cov2 - UOC Laboratorio Analisi - Servizio Medicina di Laboratorio, Ospedale "San Francesco" - ATS-ASSL Nuoro Via Mannironi 1, 08100 Nuoro	Laboratorio specialistico UOC Ematologia - Ospedale "San Francesco" - ATS-ASSL Nuoro Nuoro	Asproni Rosanna; Casu Gavino; Fancello Tatiana; Fiamma Maura; Floris Anna Rita; Lo Maglio Iana; Mameli Giuseppe; Monne Maria Itria; Palmas Angelo Domenico; Piras Giovanna; Sanna Filomena; Sulis Vincenzo; Toja Alessandro
EPI_ISL_547965	Laboratorio Biologia Molecolare SarsCov2 UOC LAboratorio Analisi SErvizio Medicina di Laboratorio Ospedale San Francesco ATS-ASSL Nuoro	Laboratorio Specialistico UOC Ematologia Ospedale San Francesco - ATS ASSL NUORO	Asproni Rosanna; Casu Gavino; Fancello Tatiana; Fiamma Maura; Floris Anna Rita; Lo Maglio Iana; Mameli Giuseppe.; Monne Maria Itria; Palmas Angelo Domenico; Piras Giovanna; Sanna Filomena; Sulis Vincenzo; Toja Alessandro
EPI_ISL_770018 to 770019	Laboratorio Cenahce	Inciensa, Instituto Costarricense de Investigación y Enseñanza en Nutrición y Salud	Adriana Godínez; Claudio Soto-Garita; Estela Cordero; Francisco Duarte; Hebleen Porras; Melany Calderón & Andrés Feoli-Grant
EPI_ISL_792474 to 792499	Laboratorio Central Mg. Luis Alfredo Pianciola	Hospital Regional Ushuaia - Centro Austral De Investigaciones Científicas - Universidad Nacional De Tierra Del Fuego on behalf of 'Proyecto Argentino Interinstitucional de genomica de SARS-CoV-2' (PAIS Consortium)	C; CF; Ceballos; F; Gallego; Gramundi; ID; M; MC; Mazzeo; Nardi; Pianciola, L.; Pinto; SG; Ziehm
EPI_ISL_964887, EPI_ISL_964891, EPI_ISL_964893 to 964896, EPI_ISL_964898 to 964900, EPI_ISL_964902 to 964912, EPI_ISL_964914, EPI_ISL_981053			
see above	Laboratorio Central Mg. Luis Alfredo Pianciola on behalf of 'Proyecto Argentino Interinstitucional de genomica de SARS-CoV-2' (PAIS Consortium)	Laboratorio Central Mg. Luis Alfredo Pianciola on behalf of 'Proyecto Argentino Interinstitucional de genomica de SARS-CoV-2' (PAIS Consortium)	C Pintos; C Ziehm; J Ousset; L Pianciola.; M Fernandez; M Mazzeo; M Nabaes
EPI_ISL_981030 to 981052, EPI_ISL_981054 to 981056	Laboratorio Central Sección Virología. Hospital Regional Artemides Zatti (Viedma, Rio Negro); Laboratorio MICROBIOM (General Roca, Río Negro); Hospital Zonal Dr. Ramón Carrillo (San Carlos De Bariloche, Río Negro)	Laboratorio Central Mg. Luis Alfredo Pianciola on behalf of 'Proyecto Argentino Interinstitucional de genomica de SARS-CoV-2' (PAIS Consortium)	Antonela De Fino; C Pintos; C Ziehm; Darío Fabián di Prátula; J Ousset; L Pianciola.; Liliana Fonseca; M Fernandez; M Mazzeo; M Nabaes; Marcela Nóbile; María Laura Álvarez; Patricia Valeria Blanco; Silvana Cecchi; Yesica Espasandin
EPI_ISL_1279263, EPI_ISL_1279268 to 1279269, EPI_ISL_1279274, EPI_ISL_1279277 to 1279278, EPI_ISL_1279283, EPI_ISL_1279286, EPI_ISL_1279288 to 1279293, EPI_ISL_1279296 to 1279297, EPI_ISL_1279299, EPI_ISL_1279303, EPI_ISL_1279383 to 1279439, EPI_ISL_1279441 to 1279461, EPI_ISL_1279537 to 1279603, EPI_ISL_1287768 to 1287770, EPI_ISL_1287774 to 1287777			
see above	Laboratorio Central de Epidemiología (LCE)	Instituto Nacional de Enfermedades Respiratorias (INER): Centro de Investigación en Enfermedades Infecciosas (CIENI)	Alejandro Sanchez-Flores; Alfredo Herrera-Estrella; Alicia Ocaña-Mondragón; Angel Gustavo Salas-Lais; Bernardo Martínez-Miguel; Blanca Taboada; Brenda Irasema Maldonado-Meza; Carla Ivón Herrera-Najera; Carlos F. Arias; Celia Boukadida; Clara Esperanza Santacruz-Tinoco; Concepción Grajales-Muñiz; Consorcio Mexicano de Vigilancia Genómica (CoViGen-Mex). Authors (in alphabetical order): Julio Elias Alvarado-Yaah; Fernando Fontove-Herrera; Francisco Pulido; Gloria Elena Espinoza-Ayala; Gloria María Molina-Salinas; Gloria Vazquez; Hector Esteban Paz-Juárez; Hector Montoya-Fuentes; Helen Haydee Fernanda Ramírez-Plascencia; Jorge Ivan Salinal-Nevarez; José Antonio Enciso-Moreno; José Esteban Muñoz-Medina; José de Jesús Nuñez-Contreras; Juan Bautista Chale-Dzul; Luis Alberto Ochoa-Carrera; Margarita Matías-Florentino; María Guadalupe Santiago-Mauricio; María Guadalupe de Jesús Mireles-Rivera; Nelly Sélem-Mojica; Pavel Isa; Ricardo Grande; Santiago Ávila-Ríos; Víctor Hugo Borja-Aburto
EPI_ISL_1288153 to 1288533	Laboratorio Central de Epidemiología (LCE)	Instituto de Biotecnología de la UNAM	Alejandro Sanchez-Flores; Alfredo Herrera-Estrella; Alicia Ocaña-Mondragón; Angel Gustavo Salas-Lais; Bernardo Martínez-Miguel; Blanca Taboada; Brenda Irasema Maldonado-Meza; Carla Ivón Herrera-Najera; Carlos F. Arias; Celia Boukadida; Clara Esperanza Santacruz-Tinoco; Concepción Grajales-Muñiz; Consorcio Mexicano de Vigilancia Genómica (CoViGen-Mex). Authors (in alphabetical order): Julio Elias Alvarado-Yaah; Fernando Fontove-Herrera; Francisco Pulido; Gloria Elena Espinoza-Ayala; Gloria María Molina-Salinas; Gloria Vazquez; Hector Esteban Paz-Juárez; Hector Montoya-Fuentes; Helen Haydee Fernanda Ramírez-Plascencia; Jorge Ivan Salinal-Nevarez; José Antonio Enciso-Moreno; José Esteban Muñoz-Medina; José de Jesús Nuñez-Contreras; Juan Bautista Chale-Dzul; Luis Alberto Ochoa-Carrera; Margarita Matías-Florentino; María Guadalupe Santiago-Mauricio; María Guadalupe de Jesús Mireles-Rivera; Nelly Sélem-Mojica; Pavel Isa; Ricardo Grande; Santiago Ávila-Ríos; Víctor Hugo Borja-Aburto
EPI_ISL_1301446, EPI_ISL_1301450, EPI_ISL_1301462 to 1301463, EPI_ISL_1301466 to 1301468, EPI_ISL_1301473, EPI_ISL_1301484, EPI_ISL_1301498, EPI_ISL_1301509, EPI_ISL_1301516, EPI_ISL_1301518, EPI_ISL_1301525, EPI_ISL_1301530, EPI_ISL_1301535 to 1301536, EPI_ISL_1301538, EPI_ISL_1301552 to 1301553, EPI_ISL_1301593 to 1301597, EPI_ISL_1301620 to 1301678, EPI_ISL_1301714 to 1301716, EPI_ISL_1302153, EPI_ISL_1302156 to 1302158, EPI_ISL_1302161, EPI_ISL_1302165, EPI_ISL_1302169, EPI_ISL_1302172 to 1302173, EPI_ISL_1302178, EPI_ISL_1302190, EPI_ISL_1302195 to 1302196, EPI_ISL_1302203 to 1302204, EPI_ISL_1302220 to 1302226, EPI_ISL_1302235 to 1302240, EPI_ISL_1302243 to 1302244, EPI_ISL_1302248, EPI_ISL_1302251 to 1302252, EPI_ISL_1302260 to 1302261, EPI_ISL_1302264, EPI_ISL_1302268, EPI_ISL_1302270, EPI_ISL_1302272, EPI_ISL_1302275, EPI_ISL_1302281 to 1302282, EPI_ISL_1302284 to 1302285, EPI_ISL_1302288, EPI_ISL_1302294, EPI_ISL_1302305, EPI_ISL_1302311, EPI_ISL_1302315, EPI_ISL_1302318, EPI_ISL_1302324, EPI_ISL_1302332, EPI_ISL_1302341, EPI_ISL_1302347 to 1302348, EPI_ISL_1302351 to 1302355, EPI_ISL_1302359, EPI_ISL_1302362, EPI_ISL_1302367, EPI_ISL_1302370, EPI_ISL_1302372, EPI_ISL_1302378 to 1302379, EPI_ISL_1302390, EPI_ISL_1302392 to 1302393, EPI_ISL_1302401			
see above	Laboratorio Central de Epidemiología IMSS	Instituto de Biotecnología de la UNAM	Alejandra Hernández-Terán; Alejandro Sanchez-Flores; Alma Rincón-Rubio; Andrea Santos Coy-Arechavaleta; Authors from IBT; Blanca Taboada; Celia Boukadida; Clara Esperanza Santacruz-Tinoco; Edgar Mendieta-Condado; Eduardo Becerril-Vargas; Fidencio Mejía-Nepomuceno; Francisco Pulido; Gisela Barrera-Badillo; Gloria Vazquez; Hector Esteban Paz-Juárez; IMSS; InDRE and INER (in alphabetical order): Carlos F. Arias; Irma Lopez-Martinez; Jerome Jean Verleyen; Joel Armando Vázquez-Pérez; Jorge Salas-Hernández; José Arturo Martínez-Orozco; José Ernesto Ramírez-González; José Esteban Muñoz-Medina; Larissa Fernandes-Matano; Lucia Hernandez-Rivas; Luis Alberto Ochoa-Carrera; Margarita Matías-Florentino; Mario Mújica-Sánchez; Natividad Cruz-Ortiz; Pavel Isa; Ricardo Grande; Santiago Ávila-Ríos; Tatiana Nunez-Garcia; Teresita Rojas-Mendoza

EPI_ISL_1181370 to 1181371, EPI_ISL_1181400 to 1181401, EPI_ISL_1181413 to 1181414	Laboratorio Central de Saude Publica do Estado Maranhao (LACEN-MA)	Laboratory of Respiratory Viruses and Measles, Oswaldo Cruz Institute, FIOCRUZ	Alice Sampaio Rocha; Ana Carolina Mendonca; Anna Carolina Paixao; Fernando Motta; Lidio Gonçalves Lima Neto; Luciana Appolinario; Marilda Siqueira on behalf of the Fiocruz COVID-19 Genomic Surveillance Network; Paola Resende; Renata Serrano Lopes
EPI_ISL_1181416, EPI_ISL_1181422	Laboratorio Central de Saude Publica do Estado da Paraiba (LACEN-PB)	Laboratory of Respiratory Viruses and Measles, Oswaldo Cruz Institute, FIOCRUZ	Alice Sampaio Rocha; Ana Carolina Mendonca; Anna Carolina Paixao; Dalane Loudal Florentino Teixeira; Fernando Motta; Joao Felipe Bezerra; Luciana Appolinario; Marilda Siqueira on behalf of the Fiocruz COVID-19 Genomic Surveillance Network; Paola Resende; Renata Serrano Lopes
EPI_ISL_1181399, EPI_ISL_1181426 to 1181428, EPI_ISL_1219137	Laboratorio Central de Saude Publica do Estado de Minas Gerais (LACEN-MG)	Laboratory of Respiratory Viruses and Measles, Oswaldo Cruz Institute, FIOCRUZ	Alice Sampaio Rocha; Ana Carolina Mendonca; Anna Carolina Paixao; Felipe Iani; Fernando Motta; Luciana Appolinario; Marilda Siqueira on behalf of the Fiocruz COVID-19 Genomic Surveillance Network; Paola Resende; Renata Serrano Lopes
EPI_ISL_1181403 to 1181404, EPI_ISL_1181412, EPI_ISL_1181453	Laboratorio Central de Saude Publica do Estado de Santa Catarina (LACEN-SC)	Laboratory of Respiratory Viruses and Measles, Oswaldo Cruz Institute, FIOCRUZ	Alice Sampaio Rocha; Ana Carolina Mendonca; Anna Carolina Paixao; Darcita Buerger Rovaris; Fernando Motta; Luciana Appolinario; Marilda Siqueira on behalf of the Fiocruz COVID-19 Genomic Surveillance Network; Paola Resende; Renata Serrano Lopes; Sandra Bianchini Fernandes
EPI_ISL_1181405 to 1181407, EPI_ISL_1181409, EPI_ISL_1181590, EPI_ISL_1181595	Laboratorio Central de Saude Publica do Estado de Sergipe (LACEN-SE)	Laboratory of Respiratory Viruses and Measles, Oswaldo Cruz Institute, FIOCRUZ	Alice Sampaio Rocha; Ana Carolina Mendonca; Anna Carolina Paixao; Cliomar Alves dos Santos; Fernando Motta; Luciana Appolinario; Marilda Siqueira on behalf of the Fiocruz COVID-19 Genomic Surveillance Network; Paola Resende; Renata Serrano Lopes
EPI_ISL_1181365, EPI_ISL_1181396, EPI_ISL_1181592, EPI_ISL_1219134 to 1219135	Laboratorio Central de Saude Publica do Estado do Alagoas (LACEN-AL)	Laboratory of Respiratory Viruses and Measles, Oswaldo Cruz Institute, FIOCRUZ	Alice Sampaio Rocha; Ana Carolina Mendonca; Anderson Brandao Leite; Anna Carolina Paixao; Fernando Motta; Luciana Appolinario; Marilda Siqueira on behalf of the Fiocruz COVID-19 Genomic Surveillance Network; Paola Resende; Renata Serrano Lopes
EPI_ISL_1181395, EPI_ISL_1181397, EPI_ISL_1181418, EPI_ISL_1181420 to 1181421, EPI_ISL_1181423, EPI_ISL_1181432, EPI_ISL_1181582 to 1181583, EPI_ISL_1181585			
see above	Laboratorio Central de Saude Publica do Estado do Espitito Santo (LACEN-ES)	Laboratory of Respiratory Viruses and Measles, Oswaldo Cruz Institute, FIOCRUZ	Alice Sampaio Rocha; Ana Carolina Mendonca; Anna Carolina Paixao; Fernando Motta; Luciana Appolinario; Marilda Siqueira on behalf of the Fiocruz COVID-19 Genomic Surveillance Network; Paola Resende; Renata Serrano Lopes; Rodrigo Ribeiro Rodrigues
EPI_ISL_1181390, EPI_ISL_1181392, EPI_ISL_1181394, EPI_ISL_1181474, EPI_ISL_1181498, EPI_ISL_1181517, EPI_ISL_1181526 to 1181528, EPI_ISL_1181530, EPI_ISL_1181533 to 1181534, EPI_ISL_1181536 to 1181545, EPI_ISL_1181551 to 1181559, EPI_ISL_1181563 to 1181566, EPI_ISL_1181568, EPI_ISL_1181570, EPI_ISL_1181576, EPI_ISL_1181580 to 1181581, EPI_ISL_1181584, EPI_ISL_1181605, EPI_ISL_1181609 to 1181610, EPI_ISL_1181614 to 1181619, EPI_ISL_1181623, EPI_ISL_1219132 to 1219133			
see above	Laboratorio Central de Saude Publica do Estado do Parana (LACEN-PR)	Laboratory of Respiratory Viruses and Measles, Oswaldo Cruz Institute, FIOCRUZ	Alice Sampaio Rocha; Ana Carolina Mendonca; Anna Carolina Paixao; Fernando Motta; Irina Nastassja Riediger; Luciana Appolinario; Maria do Carmo Debur; Marilda Siqueira on behalf of the Fiocruz COVID-19 Genomic Surveillance Network; Paola Resende; Renata Serrano Lopes
EPI_ISL_1181368, EPI_ISL_1181411, EPI_ISL_1181433 to 1181434, EPI_ISL_1181451, EPI_ISL_1181468, EPI_ISL_1181524, EPI_ISL_1181567, EPI_ISL_1181620			
see above	Laboratorio Central de Saude Publica do Estado do Rio Grande do Sul (LACEN-RS)	Laboratory of Respiratory Viruses and Measles, Oswaldo Cruz Institute, FIOCRUZ	Alice Sampaio Rocha; Ana Carolina Mendonca; Anna Carolina Paixao; Fernando Motta; Luciana Appolinario; Marilda Siqueira on behalf of the Fiocruz COVID-19 Genomic Surveillance Network; Paola Resende; Renata Serrano Lopes; Tatiana Schaffer Gregianini
EPI_ISL_1181353, EPI_ISL_1181372, EPI_ISL_1181425, EPI_ISL_1181431, EPI_ISL_1181600 to 1181603, EPI_ISL_1181606 to 1181607, EPI_ISL_1181611 to 1181613, EPI_ISL_1181624			
see above	Laboratorio Central de Saude Publica do Estado do Rio de Janeiro (LACEN-RJ)	Laboratory of Respiratory Viruses and Measles, Oswaldo Cruz Institute, FIOCRUZ	Alice Sampaio Rocha; Ana Carolina Mendonca; Andrea Cony Cavalcanti; Anna Carolina Paixao; Fernando Motta; Luciana Appolinario; Marilda Siqueira on behalf of the Fiocruz COVID-19 Genomic Surveillance Network; Paola Resende; Renata Serrano Lopes
EPI_ISL_792512 to 792521	Laboratorio Central de la Ciudad de Santa Fe	Grupo de Genómica y Bioinformática del Instituto de Investigación de la Cadena Láctea CONICET-INTA on behalf of 'Proyecto Argentino Interinstitucional de genomica de SARS-CoV-2' (PAIS Consortium)	AF; Amadio; C; Eberhardt; G; Irazoqui; JM; MF; Mugna; Ojeda; Pastor; Rompato; V
EPI_ISL_792526 to 792546	Laboratorio Central, Ministerio de Salud Córdoba	Instituto de Patologia Vegetal (CIAP-INTA) on behalf of 'Proyecto Argentino Interinstitucional de genomica de SARS-CoV-2' (PAIS Consortium)	Barbas, G.; Castro, G.; Debat, HJ.; FD; Fernández; MB; Pisano; Re; V
EPI_ISL_1220050	Laboratorio Clinico Clinica General del Norte	Instituto Nacional de Salud- Dirección de Investigación en Salud Pública	Carlos Franco-Muñoz; Diego A. Álvarez-Díaz; Diego Andrés Prada; Gerardo Santamaría; Hector Alejandro Ruiz-Moreno; Jhonnatan Reales-González; Julian Naizaque; Katherine Laiton-Donato; Magdalena Wiesner; Marcela Mercado-Reyes.; Maria T. Herrera-Sepúlveda; Martha Lucia Ospina Martínez; Sheryll Corchuelo
EPI_ISL_1220049	Laboratorio Clinico Clinica General del norte SAS	Instituto Nacional de Salud- Dirección de Investigación en Salud Pública	Carlos Franco-Muñoz; Diego A. Álvarez-Díaz; Diego Andrés Prada; Gerardo Santamaría; Hector Alejandro Ruiz-Moreno; Jhonnatan Reales-González; Julian Naizaque; Katherine Laiton-Donato; Magdalena Wiesner; Marcela Mercado-Reyes.; Maria T. Herrera-Sepúlveda; Martha Lucia Ospina Martínez; Sheryll Corchuelo
EPI_ISL_769987, EPI_ISL_769991	Laboratorio Clinico Labin	Inciensa, Instituto Costarricense de Investigación y Enseñanza en Nutrición y Salud	Adriana Godinez; Adriana Godínez; Claudio Soto-Garita; Estela Cordero; Francisco Duarte; Hebleen Porras; Melany Calderon & Pei Chan Ma; Melany Calderón & Mariel López
EPI_ISL_480321	Laboratorio Clínico San José	Charité Virology-University of Costa Rica	Andres Moreira-Soto; Eugenia Corrales-Aguilar; Hugo Núñez Navas; Ignacio Postigo-Hidalgo; Jan Felix Drexler
EPI_ISL_1092007	Laboratorio Continental	Instituto Nacional de Salud- Dirección de Investigación en Salud Pública	Carlos Franco-Muñoz; Diego A. Álvarez-Díaz; Diego Andrés Prada; Gerardo Santamaría; Hector Alejandro Ruiz-Moreno; Jhonnatan Reales-González; Julian Naizaque; Katherine Laiton-Donato; Magdalena Wiesner; Marcela Mercado-Reyes; Maria T. Herrera-Sepúlveda; Martha Lucia Ospina Martínez; Sheryll Corchuelo
EPI_ISL_750163 to 750164, EPI_ISL_751201	Laboratorio DILAVE/MGAP-INIA-UdelaR -Tacuarembó	Institut Pasteur de Montevideo	Ana Carolina Mendonça; Andres Lizasoain; Camila Simoes; Cecilia Alonso; Cecilia Salazar; Daiana Mir; Fernando Lopez-Tort; Fernando Motta; Gonzalo Bello; Ighor Arantes; Ignacio Ferrés; Jose Sotelo; Leticia Maya; Letícia Garay Martins; Luciana Appolinario; Lucia Spangenberg; Mailen Arleo; Mariana Brandes; Marilda Mendonça Siqueira; Marilda Tereza Mar da Rosa; Maria Jose Benitez-Galeano; Martín Graña; Matias Castells; Matias Victoria; Matías Salvo; Natalia Rego; Natalia Reyes; Pablo Smircich; Paola Cristina Resende; Rodney Colina; Tamara Fernandez-Calero; Tania Possi; Tatiana Schäffer Gregianini; Veronica Noya; Yasser Vega
EPI_ISL_1017702	Laboratorio Docente Asistencial-Universidad de Antioquia	Instituto Nacional de Salud- Dirección de Investigación en Salud Pública	Carlos Franco-Muñoz; Diego A. Álvarez-Díaz; Diego Andrés Prada; Gerardo Santamaría; Hector Alejandro Ruiz-Moreno; Jhonnatan Reales-González; Julian Naizaque; Katherine Laiton-Donato; Magdalena Wiesner; Marcela Mercado-Reyes; Maria T. Herrera-Sepúlveda; Martha Lucia Ospina Martínez; Sheryll Corchuelo
EPI_ISL_1060251 to 1060258, EPI_ISL_1060260	Laboratorio Echevarne S.A.	SeqCOVID-SPAIN consortium/IBV(CSIC)	Beatriz Fernández Forner and SeqCOVID-SPAIN consortium; Raúl Santamaría Merino
EPI_ISL_424667	Laboratorio Estatal de Salud Publica del Estado de México	Instituto de Diagnóstico y Referencia Epidemiológicos	Adnan Araiza Rodríguez; Alejandro Sánchez; Alfredo Ponce de León Garduño; Blanca Taboada; Carlos F. Arias.; Carolina González Torres; Celia Boukadida; Cesar Raúl González Bonilla; Concepción Grajales Muñiz; Edgar Mendieta Condado; Eduardo Becerril Vargas; Fabiola Garcés Ayala; Fernando Ledesma Barrientos; Francisco Javier Gaytán Cervantes; Francisco Pulido; Gisela Barrera Badillo; Gloria Vázquez; Guillermo M. Ruiz-Palacios; Irma López Martínez; Joel Armando Vázquez Pérez; José Arturo Martínez Orozco; José Ernesto Ramírez González; José Esteban Muñoz Medina; Lucia Hernández Rivas; Luis Alberto García Andrade; Mario Mújica Sánchez; Pavel Isa; Pilar Ramos Cervantes; Ricardo Grande; Santiago Avila Ríos; Victor Hugo Borja Aburto; Violeta Ibarra Gonzalez
EPI_ISL_424672	Laboratorio Estatal de Salud Publica del Estado de Puebla	Instituto de Diagnostico y Referencia Epidemiologicos	Adnan Araiza Rodríguez; Alejandro Sánchez; Alfredo Ponce de León Garduño; Blanca Taboada; Carlos F. Arias; Carolina González Torres; Celia Boukadida; Cesar Raúl González Bonilla; Concepción Grajales Muñiz; Edgar Mendieta Condado; Eduardo Becerril Vargas; Fabiola Garcés Ayala; Fernando Ledesma Barrientos; Francisco Javier Gaytán Cervantes; Francisco Pulido; Gisela Barrera Badillo; Gloria Vázquez; Guillermo M. Ruiz-Palacios; Irma López Martínez; Joel Armando Vázquez Pérez; José Arturo Martínez Orozco; José Ernesto Ramírez González; José Esteban Muñoz Medina; Lucia Hernández Rivas; Luis Alberto García Andrade; Mario Mújica Sánchez; Pavel Isa; Pilar Ramos Cervantes; Ricardo Grande; Santiago Avila Ríos; Victor Hugo Borja Aburto; Violeta Ibarra Gonzalez
EPI_ISL_424670	Laboratorio Estatal de Salud Publica del Estado de Queretaro	Instituto de Diagnóstico y Referencia Epidemiologicos	Adnan Araiza Rodríguez; Alejandro Sánchez; Alfredo Ponce de León Garduño; Blanca Taboada; Carlos F. Arias; Carolina González Torres; Celia Boukadida; Cesar Raúl González Bonilla; Concepción Grajales Muñiz; Edgar Mendieta Condado; Eduardo Becerril Vargas; Fabiola Garcés Ayala; Fernando Ledesma Barrientos; Francisco Javier Gaytán Cervantes; Francisco Pulido; Gisela Barrera Badillo; Gloria Vázquez; Guillermo M. Ruiz-Palacios;

			Irma López Martínez; Joel Armando Vázquez Pérez; José Arturo Martínez Orozco; José Ernesto Ramírez González; José Esteban Muñoz Medina; Lucia Hernández Rivas; Luis Alberto García Andrade; Mario Mújica Sánchez; Pavel Isa; Pilar Ramos Cervantes; Ricardo Grande; Santiago Avila Ríos; Victor Hugo Borja Aburto; Violeta Ibarra Gonzalez
EPI_ISL_779185 to 779198, EPI_ISL_979327 to 979352, EPI_ISL_1091241 to 1091246, EPI_ISL_1091249, EPI_ISL_1091253 to 1091257, EPI_ISL_1091262, EPI_ISL_1092458			
see above	Laboratorio Estatal de Salud Pública de Nuevo León	Laboratorio de Infectología Molecular, Departamento de Bioquímica y Medicina Molecular,Facultad de Medicina - Universidad Autónoma de Nuevo León	Ana M. Rivas-Estilla; Consuelo Treviño-Garza; Daniel Arellanos-Soto; Else del Carmen García-García; Gloria A. Jasso-de-la-Peña; Kame A. Galán-Huerta; Manuel E. de-la-O-Cavazos; María F. Herrera-Saldivar; Natalia Martínez-Acuña; Roberto Montes-de-Oca; Samuel Buentello-Wong; Sonia A. Lozano-Sepúlveda
EPI_ISL_794592	Laboratorio Estatal de Salud Pública de Tamaulipas	Instituto de diagnóstico y Referencia Epidemiologicos (INDRE)	Abril Rodriguez-Maldonado; Ana Maria Cortez-Calderon; Bernardita Reyes-Berrones; Celia Alpuche-Aranda; Claudia Wong-Arambula; Ernesto Ramirez-Gonzalez.; Fabiola Garces-Ayala; Gisela Barrera-Badillo; Gloria Molina-Gamboa; Hilda del Carmen-Selvera; Hugo Lopez Gatell-Ramirez; Irma Lopez-Martinez; Jose Luis Alomia-Zegarra; Lucia Hernandez-Rivas
EPI_ISL_735398	Laboratorio Fleury	Instituto Adolfo Lutz, Interdiciplinary Procedures Center, Strategic Laboratory	Claudia Regina Gonçalves; Claudio Tavares Sacchi; Erica Valessa Ramos Gomes; Karoline Rodrigues Campos
EPI_ISL_956301, EPI_ISL_956306	Laboratorio Gencore- Universidad de los Andes	Instituto Nacional de Salud- Dirección de Investigación en Salud Pública, Universidad de los Andes- Gencore	Carlos Franco-Muñoz; Diego A. Álvarez-Díaz; Diego Andrés Prada; Gerardo Santamaría; Hector Alejandro Ruiz-Moreno; Jhonnatan Reales-González; Julian Naizaque; Katherine Laiton-Donato; Magdalena Wiesner; Marcela Mercado-Reyes; Maria T. Herrera-Sepúlveda; Martha Lucia Ospina Martinez; Mauricio Pacheco-Montealegre; Sheryll Corchuelo
EPI_ISL_1195952, EPI_ISL_1217383	Laboratorio Genzano - ASL RM 10	INMI Lazzaro Spallanzani IRCCS	A Di Caro; B Bartolini; CEM Gruber; E Conti; E Giombini; F Messina; G Tramini; M Rueca; MR Capobianchi; O Butera
EPI_ISL_1222714	Laboratorio Genzano - ASL RM 11	INMI Lazzaro Spallanzani IRCCS	A Di Caro; B Bartolini; CEM Gruber; E Conti; E Giombini; F Messina; G Tramini; M Rueca; MR Capobianchi; O Butera
EPI_ISL_913446 to 913447, EPI_ISL_1063909, EPI_ISL_1091791 to 1091793, EPI_ISL_1091795, EPI_ISL_1169890, EPI_ISL_1195957, EPI_ISL_1217388, EPI_ISL_1222711, EPI_ISL_1229139, EPI_ISL_1295607			
see above	Laboratorio Genzano - ASL RM 6	INMI Lazzaro Spallanzani IRCCS	A Di Caro; Antonino Di Caro; B Bartolini; Barbara Bartolini; C.E.M Gruber; CEM Gruber; Cesare E.M. Gruber; Cesare EM Gruber; E Conti; E Giombini; Emanuela Conti; Emanuela Giombini; F Messina; F Santini; Francesco Messina; G Bonfiglio; G Tramini; Grazia Tramini; M Rueca; MR Capobianchi; Maria R Capobianchi; Maria R. Capobianchi; Martina Rueca; O Butera; Ornella Butera
EPI_ISL_1210498, EPI_ISL_1222712	Laboratorio Genzano - ASL RM 7	INMI Lazzaro Spallanzani IRCCS	A Di Caro; B Bartolini; CEM Gruber; E Conti; E Giombini; F Messina; G Tramini; M Rueca; MR Capobianchi; O Butera
EPI_ISL_1195958, EPI_ISL_1217380	Laboratorio Genzano - ASL RM 8	INMI Lazzaro Spallanzani IRCCS	A Di Caro; B Bartolini; CEM Gruber; E Conti; E Giombini; F Messina; G Tramini; M Rueca; MR Capobianchi; O Butera
EPI_ISL_1195951, EPI_ISL_1222713	Laboratorio Genzano - ASL RM 9	INMI Lazzaro Spallanzani IRCCS	A Di Caro; B Bartolini; CEM Gruber; E Conti; E Giombini; F Messina; G Tramini; M Rueca; MR Capobianchi; O Butera
EPI_ISL_1092006	Laboratorio IMAT	Instituto Nacional de Salud- Dirección de Investigación en Salud Pública	Carlos Franco-Muñoz; Diego A. Álvarez-Díaz; Diego Andrés Prada; Gerardo Santamaría; Hector Alejandro Ruiz-Moreno; Jhonnatan Reales-González; Julian Naizaque; Katherine Laiton-Donato; Magdalena Wiesner; Marcela Mercado-Reyes; Maria T. Herrera-Sepúlveda; Martha Lucia Ospina Martinez; Sheryll Corchuelo
EPI_ISL_480328	Laboratorio LABIN	Charité Virology-University of Costa Rica	Andres Moreira-Soto; Eugenia Corrales-Aguilar; Ignacio Postigo-Hidalgo; Ignacio Soto Pacheco; Jan Felix Drexler
EPI_ISL_1097016	Laboratorio Microbiologia e Virologia P.O. Cotugno A.O. dei Col	Laboratorio Microbiologia e Virologia P.O. Cotugno A.O. dei Colli	Anna Perfetti; Biancamaria Pierri; Claudia Tiberio; Luigi Atripaldi; Maria Concetta Cuomo; Pellegrino Cerino
EPI_ISL_969131, EPI_ISL_969227, EPI_ISL_969884, EPI_ISL_970647, EPI_ISL_974745, EPI_ISL_977598, EPI_ISL_977601, EPI_ISL_977603, EPI_ISL_977651, EPI_ISL_1095384, EPI_ISL_1095449, EPI_ISL_1096228 to 1096233, EPI_ISL_1096342, EPI_ISL_1096982, EPI_ISL_1096984, EPI_ISL_1096986, EPI_ISL_1096989, EPI_ISL_1096991, EPI_ISL_1096993 to 1096994, EPI_ISL_1096996, EPI_ISL_1096998, EPI_ISL_1097000, EPI_ISL_1097004 to 1097005, EPI_ISL_1097007 to 1097015, EPI_ISL_1097017, EPI_ISL_1097019 to 1097022, EPI_ISL_1118930, EPI_ISL_1118932, EPI_ISL_1118934, EPI_ISL_1119567			
see above	Laboratorio Microbiologia e Virologia P.O. Cotugno A.O. dei Colli	Laboratorio Microbiologia e Virologia P.O. Cotugno A.O. dei Colli	; Anna Perfetti; Biancamaria Pierri; Claudia Tiberio; Luigi Atripaldi; Maria Concetta Cuomo; Pellegrino Cerino
EPI_ISL_969297	Laboratorio Microbiologia e Virologia, P.O. Cotugno, A.O. dei Colli	Laboratorio Microbiologia e Virologia, P.O. Cotugno, A.O. dei Colli	Anna Perfetti; Claudia Tiberio; Luigi Atripaldi
EPI_ISL_861628, EPI_ISL_861678	Laboratorio Municipal de Guarulhos	Instituto Adolfo Lutz, Interdiciplinary Procedures Center, Strategic Laboratory	Claudia Regina Gonçalves; Claudio Tavares Sacchi; Erica Valessa Ramos Gomes; Karoline Rodrigues Campos
EPI_ISL_693246	Laboratorio Municipal de Rio Grande da Serra	Instituto Adolfo Lutz, Interdiciplinary Procedures Center, Strategic Laboratory	Claudia Regina Gonçalves; Claudio Tavares Sacchi; Erica Valessa Ramos Gomes; Karoline Rodrigues Campos
EPI_ISL_845628 to 845629	Laboratorio Médico Echavarria	Instituto Nacional de Salud - Dirección de Investigación en Salud Pública	Carlos Franco-Muñoz; Diego A. Álvarez-Díaz; Diego Andrés Prada; Gerardo Santamaría; Jonathan Reales; Julian Naizaque; Katherine Laiton-Donato; Magdalena Wiesner; Marcela Mercado-Reyes; Maria T. Herrera-Sepúlveda; Martha Lucia Ospina Martinez; Mauricio Pacheco-Montealegre; Paola Muñoz-Laiton; Sheryll Corchuelo
EPI_ISL_837551 to 837552, EPI_ISL_837579 to 837596	Laboratorio Nacional de Salud	Laboratory of Respiratory Viruses and Measles, Oswaldo Cruz Institute, FIOCRUZ	Ana Carolina Mendonca; Anna Carolina Paixao; Cesar Roberto Conde Pereira; Claudia Estrada; Fernando Motta; Luciana Appolinario; Marilda Siqueira on behalf of the Fiocruz COVID-19 Genomic Surveillance Network; Paola Resende
EPI_ISL_1091247 to 1091248, EPI_ISL_1091250 to 1091252, EPI_ISL_1091258 to 1091260, EPI_ISL_1091263 to 1091274	Laboratorio PGM	Laboratorio de Infectología Molecular, Departamento de Bioquímica y Medicina Molecular,Facultad de Medicina - Universidad Autónoma de Nuevo León	Ana M. Rivas-Estilla; Daniel Arellanos-Soto; Eduardo Garza-de-la-Peña; Gabriela Elizondo; Javier Ramos-Jimenez; Kame A. Galán-Huerta; María F. Herrera-Saldivar; Natalia Martínez-Acuña; Sonia A. Lozano-Sepúlveda
EPI_ISL_1253609	Laboratorio Unico Provinciale Azienda Ospedaliero-Universitaria di Ferrara	U.O. Microbiologia, Laboratorio Unico Centro Servizi - AUSL della Romagna	Giorgio Dirani; Michela Boni; Sara Ghisellini; Silvia Zannoli; Vittorio Sambri
EPI_ISL_1091261, EPI_ISL_1092361	Laboratorio de Apoyo a la Vigilancia e Investigación Epidemiológica - CIBO	Laboratorio de Infectología Molecular, Departamento de Bioquímica y Medicina Molecular,Facultad de Medicina - Universidad Autónoma de Nuevo León	Alma M. Perez-Rios; Ana M. Rivas-Estilla; Blanca M. Torres; Claudia L. Charles-Niño; Daniel Arellanos-Soto; Diego A. Gonzalez-Altamirano; Hector Montoya-Fuentes; Javier Ramos-Jimenez; Jose J. Figueroa-Delgadillo; Kame A. Galán-Huerta; María F. Herrera-Saldivar; Natalia Martínez-Acuña; Rafael Soto-Alvarez; Sonia A. Lozano-Sepúlveda
EPI_ISL_755301 to 755303	Laboratorio de Bioingeniería, Instituto de Ciencias de la Ingeniería, Universidad de O´Higgins	Center for Mathematical Modeling and Center for Genome Regulation. Santiago, Chile	Allende ML; Arriagada G; Bastias M; Bustos F; Castro E; G. Galvez; González M; J. Ortega; M; M. Latorre; Maass A; Meneses C.; Montecino; Orellana A; Sanhueza D; Travisany D
EPI_ISL_468753 to 468759	Laboratorio de Biologia Molecular, Facultad de Medicina, Universidad de Atacama	Center for Mathematical Modeling and Center for Genome Regulation. Santiago, Chile	Allende ML; C Echeverria; Gaete A; González M; Maass A; Palma R; Travisany D; Urra C; Varas M
EPI_ISL_1278277 to 1278280	Laboratorio de Biologia Molecular, Hospital San Pedro Claver	Molecular Genetics Laboratory, Instituto de Investigaciones Químicas, Universidad Mayor de San Andrés	Aneth Vasquez Michel; Carmen Delgado Barrera; Oscar M. Rollano-Peñaloza
EPI_ISL_1278281 to 1278284	Laboratorio de Biologia Molecular, SEDES-Potosi	Molecular Genetics Laboratory, Instituto de Investigaciones Químicas, Universidad Mayor de San Andrés	Aneth Vasquez Michel; Oscar M. Rollano-Peñaloza
EPI_ISL_457940 to 457973, EPI_ISL_480428 to 480429, EPI_ISL_480431 to 480438	Laboratorio de Biología Molecular Asociación Española Primera en Salud	Departments of Pathology and Medicine, New York University School of Medicine	Adriana Heguy; Christian Marier; Gael Westby; Gonzalo Manrique; Maria Noel Zubillaga; Maria Victoria Elizondo; Matthew T Maurano; Paul Zappile
EPI_ISL_842652	Laboratorio de Biología Molecular Hospital Pedro de Elizalde	Grupo de Genómica y Bioinformática del Instituto de Investigación de la Cadena Láctea CONICET-INTA on behalf of 'Proyecto Argentino Interinstitucional de genomica de SARS-CoV-2' (PAIS Consortium)	A; AF; Alegre; Alexay; Amadio; Aulicino; B; Bressan; C; Chamorro; Claps; D; Diaz; E; Eberhardt; F; FJ; G; Gondolessi; Goya; Gómez; Indart; Irazoqui; J; König; L; Lorenzo; Lusso; M; ME; MF; ML; MS; Marchetti; Martín; Montoto Piazza; Morandi; N; Nabaes Jodar; Natale; Osaba; P; Paez; Rocovich; Rosales; S; Sanchez; Sueiro; Torres; Valinotto; Viegas, M.; Wenk; Zamora
EPI_ISL_626549 to 626565	Laboratorio de Biología Molecular, Facultad de Medicina, Universidad de Atacama, Copiapo, Chile/ FONDAP CRG, Universidad Andrés Bello, Santiago, Chile	Center for Mathematical Modeling and Center for Genome Regulation. Santiago, Chile	Allende ML; Bastias M; Castro E; Echeverría C; González M; M; Maass A; Manríquez R; Meneses C.; Montecino; Orellana A; Sanhueza D; Travisany D

EPI_ISL_792354 to 792357	Laboratorio de Biología Molecular. Hospital Dr. Héctor Cura	Área de Secuenciación del Laboratorio de Virología del Hospital de Niños Dr. Ricardo Gutierrez on behalf of 'Proyecto Argentino Interinstitucional de genomica de SARS-CoV-2' (PAIS Consortium)	Ghiano; Goya; J; LE; Lusso; MB; MI; MS; N; Nabaes Jodar; Natale; R; S; Spina; Turrina; Valinotto; Viegas, M.; Zaffanella
EPI_ISL_1093145 to 1093147	Laboratorio de Diagnostico de Enfermedades Emergentes y Reemergentes	Instituto de Diagnostico y Referencia Epidemiologicos (INDRE)	Abril Rodriguez-Maldonado; Claudia Wong-Arambula; Ernesto Ramirez-Gonzalez.; Fabiola Garces-Ayala; Gisela Barrera-Badillo; Irma Lopez-Martinez; Lucia Hernandez-Rivas; Natali Vega-Magana; Natividad Cruz-Ortiz; Tatiana Nunez-Garcia
EPI_ISL_417034, EPI_ISL_792560, EPI_ISL_801386 to 801396, EPI_ISL_811148 to 811149, EPI_ISL_833131 to 833140, EPI_ISL_1034304 to 1034306, EPI_ISL_1068078 to 1068292, EPI_ISL_1114151, EPI_ISL_1261122 to 1261123			
see above	Laboratorio de Ecologia de Doencas Transmissiveis na Amazonia, Instituto Leonidas e Maria Deane - Fiocruz Amazonia	Laboratorio de Ecologia de Doencas Transmissiveis na Amazonia, Instituto Leonidas e Maria Deane - Fiocruz Amazonia	André Corado; Debora Duarte; Felipe Naveca; Felipe Naveca on behalf of the Fiocruz COVID-19 Genomic Surveillance Network; Fernanda Nascimento; George Silva; Karina Pessoa; Luciana Gonçalves; Maria Júlia Brandão; Matilde Mejía; Michele Jesus; Sérgio Luz; Valdinete Nascimento; Victor Souza; Ágatha Costa
EPI_ISL_1181378, EPI_ISL_1181452, EPI_ISL_1181471 to 1181473, EPI_ISL_1181478 to 1181480, EPI_ISL_1181487, EPI_ISL_1181492, EPI_ISL_1181516, EPI_ISL_1181525, EPI_ISL_1181532, EPI_ISL_1181535, EPI_ISL_1181546 to 1181550, EPI_ISL_1181569			
see above	Laboratorio de Imunologia de Transplantes de Goias LTDA (HLAGYN)	Laboratory of Respiratory Viruses and Measles, Oswaldo Cruz Institute, FIOCRUZ	Alessandro Leonardo Alvares Magalhaes; Alice Sampaio Rocha; Ana Carolina Mendonca; Anna Carolina Paixao; Erika Lopes Rocha Batista; Fernando Antonio Vinhal dos Santos; Fernando Motta; Luciana Appolinario; Marilda Siqueira on behalf of the Fiocruz COVID-19 Genomic Surveillance Network; Paola Resende; Renata Serrano Lopes
EPI_ISL_545955	Laboratorio de Infecciones Respiratorias Agudas. Centro Nacional de Salud Publica,Instituto Nacional de Salud	Laboratorio de Infecciones Respiratorias Agudas. Centro Nacional de Salud Publica,Instituto Nacional de Salud	Juscamayta, E.
EPI_ISL_591531 to 591534	Laboratorio de Infectologia y virologia molecular	Center for Mathematical Modeling and Center for Genome Regulation. Santiago, Chile	Allende ML; Ferres M.; Gaete A; González M; Maass A; Palma R; Travisany D; Urra C; Valiente F; Varas M
EPI_ISL_648380, EPI_ISL_648595, EPI_ISL_648597 to 648598, EPI_ISL_648600, EPI_ISL_648602 to 648603, EPI_ISL_648606	Laboratorio de Infectología Servicio de Infectología Hospital Universitario Dr. José Eleuterio González - Universidad Autónoma de Nuevo León	Laboratorio de Infectología Molecular Departamento de Bioquímica y Medicina Molecular Facultad de Medicina - Universidad Autónoma de Nuevo León	Adrian Camacho-Ortiz; Ana M. Rivas-Estilla; Consuelo Treviño-Garza; Daniel Arellanos-Soto; Eduardo Perez-Alba; Elvira Garza-González; Kame A. Galán-Huerta; Laura Nuzzolo-Shihadeh; Manuel E. de-la-O-Cavazos; María F. Herrera-Saldivar; Natalia Martínez-Acuña; Paola Bocanegra-Ibarias; Roberto Montes-de-Oca; Samantha M. Flores-Treviño; Sonia A. Lozano-Sepúlveda
EPI_ISL_648607 to 648609, EPI_ISL_779169 to 779184, EPI_ISL_961760 to 961800, EPI_ISL_965222	Laboratorio de Infectología, Servicio de Infectología, Hospital Universitario Dr. José Eleuterio González - Universidad Autónoma de Nuevo León	Laboratorio de Infectología Molecular, Departamento de Bioquímica y Medicina Molecular,Facultad de Medicina - Universidad Autónoma de Nuevo León	Adrian Camacho-Ortiz; Ana M. Rivas-Estilla; Consuelo Treviño-Garza; Daniel Arellanos-Soto; Eduardo Perez-Alba; Elvira Garza-González; Kame A. Galán-Huerta; Laura Nuzzolo-Shihadeh; Manuel E. de-la-O-Cavazos; María F. Herrera-Saldivar; Natalia Martínez-Acuña; Paola Bocanegra-Ibarias; Roberto Montes-de-Oca; Samantha M. Flores-Treviño; Sonia A. Lozano-Sepúlveda
EPI_ISL_792500 to 792511	Laboratorio de Inmunología del Hospital Perrando e Instituto de Medicina Regional de la UNNE	Grupo de Genómica y Bioinformática del Instituto de Investigación de la Cadena Láctea CONICET-INTA on behalf of 'Proyecto Argentino Interinstitucional de genomica de SARS-CoV-2' (PAIS Consortium)	A; AF; Amadio; Ayala; Cayré; Deluca; Eberhardt; Foussal; G; Giusiano; Gómez; H; Irazoqui; JM; L; Lescano; Lucero; M; MD; MF; MV; Marin; NA
EPI_ISL_792424 to 792442	Laboratorio de Inmunología del Hospital Perrando e Instituto de Medicina Regional de la UNNE	Instituto de Biotecnología, IABIMO (CONICET), Instituto de Virología, IVIT(CONICET), Instituto de Patobiología, IPVET(CONICET), CICVyA, INTA on behalf of 'Proyecto Argentino Interinstitucional de genomica de SARS-CoV-2' (PAIS Consortium)	A; AF; AJ; AV; Asurmendi; Ayala; Bengoa Luoni; Cacciabué; Cayré; D; Deluca; Distéfano; Farber; Fass; Foussal; G; GA; Giusiano; Gómez; H; König; L; LC; Lescano; Lozano Calderón; Lucero; M; MD; MG; MPD; MV; Marin; Muñoz Hidalgo; NA; NB; PA; Paniego; Pedroarias; Peralta; Puebla; Rivarola; S; VC; Vera; Viegas, M.; Zavallo
EPI_ISL_953402 to 953425	Laboratorio de Investigaciones de Baney	"Swiss Tropical and Public Health Institute"	"Carlos Cortes; Bonifacio Manguire Nlavo; Claudia Daubenberger; Diosdado Odjama Nseng Ada; Elizabeth Nyakarungu; Guillermo Garcia; Maximilian Mpina; Mitoha Ondo O Ayekaba; Philip Wonder Phiri"; Salome Hosch; Tobias Schindler
EPI_ISL_648303 to 648379, EPI_ISL_649155 to 649172	Laboratorio de Investigaciones de Baney	University Hospital Basel, Clinical Bacteriology	Adrian Egli; Alfredo Mari; Bonifacio Manguire Nlavo; Carlos Cortes; Claudia Daubenberger; Diosdado Odjama Nseng Ada; Elizabeth Nyakarungu; Guillermo Garcia; Helena Seth-Smith; Madlen Stange; Maximilian Mpina; Mitoha Ondo O Ayekaba; Philip Wonder Phiri; Salome Hosch; Tim Roloff; Tobias Schindler
EPI_ISL_1060214 to 1060242, EPI_ISL_1060245 to 1060246, EPI_ISL_1060248 to 1060250, EPI_ISL_1060259, EPI_ISL_1060261 to 1060285, EPI_ISL_1060293 to 1060297	Laboratorio de Microbiología, Hospital Marina Baixa, Villajoyosa	SeqCOVID-SPAIN consortium/IBV(CSIC)	Carmen Martínez Peinado and SeqCOVID-SPAIN consortium; Francisco José Arjona Zaragozí; Mónica Parra Grande
EPI_ISL_796127 to 796134, EPI_ISL_941220 to 941232, EPI_ISL_1060243 to 1060244, EPI_ISL_1060247, EPI_ISL_1060286 to 1060292	Laboratorio de Microbiología. Hospital General Universitario de Elda, Alicante	SeqCOVID-SPAIN consortium/IBV(CSIC)	Cristina Torregrosa Hetland; Eva Pastor Boix; Mª Isabel Gascón Ros; Paloma Cascales Ramos and SeqCOVID-SPAIN consortium
EPI_ISL_925173, EPI_ISL_1111068, EPI_ISL_1111070 to 1111073, EPI_ISL_1111385 to 1111445	Laboratorio de Referencia Nacional de Enteropatógenos. Instituto Nacional de Salud del Perú	Laboratorio de Referencia Nacional de Enteropatógenos. Instituto Nacional de Salud del Perú	; Fiorella Orellana Peralta; Iris Silva Molina; Junior Caro Castro; Ronnie Gavilan Chavez; Veronica Hurtado Vela; Willi Quino Sifuentes
EPI_ISL_517770, EPI_ISL_517959	Laboratorio de Referencia Nacional de Virus Respiratorio. Centro Nacional de Salud Publica. Instituto Nacional de Salud Peru	Laboratorio de Referencia Nacional de Biotecnología y Biología Molecular. Centro Nacional de Salud Publica. Instituto Nacional de Salud Peru.	Carlos Padilla Rojas; Henri Bailon Calderon; Johanna Balbuena Torres; Karolyn Vega Chozo; Marco Galarza Perez; Maribel Huaringa Nuñez; Nancy Rojas Serrano.; Omar Caceres Rey; Priscila Lope Pari
EPI_ISL_516626, EPI_ISL_516628, EPI_ISL_516646 to 516647, EPI_ISL_516653 to 516654, EPI_ISL_516685 to 516686, EPI_ISL_516721 to 516722, EPI_ISL_516987, EPI_ISL_517531, EPI_ISL_517686 to 517687, EPI_ISL_517713, EPI_ISL_517958, EPI_ISL_523810, EPI_ISL_523954, EPI_ISL_523979, EPI_ISL_523994, EPI_ISL_524471, EPI_ISL_524473, EPI_ISL_525206, EPI_ISL_527787, EPI_ISL_527789			
see above	Laboratorio de Referencia Nacional de Virus Respiratorio. Centro Nacional de Salud Publica. Instituto Nacional de Salud Peru.	Laboratorio de Referencia Nacional de Biotecnología y Biología Molecular. Centro Nacional de Salud Publica. Instituto Nacional de Salud Peru.	Carlos Padilla Rojas; Henri Bailon Calderon; Johanna Balbuena Torres; Karolyn Vega Chozo; Marco Galarza Perez; Maribel Huaringa Nuñez; Nancy Rojas Serrano.; Omar Caceres Rey; Priscila Lope Pari
EPI_ISL_489836 to 489839, EPI_ISL_489987 to 489990, EPI_ISL_490209, EPI_ISL_490315 to 490316, EPI_ISL_490975 to 490976, EPI_ISL_491427 to 491430, EPI_ISL_491433 to 491436, EPI_ISL_491458 to 491464, EPI_ISL_514226, EPI_ISL_514317, EPI_ISL_514338 to 514342, EPI_ISL_833038, EPI_ISL_943570, EPI_ISL_1092324 to 1092359, EPI_ISL_1093148 to 1093181, EPI_ISL_1137479 to 1137491, EPI_ISL_1138413 to 1138422			
see above	Laboratorio de Referencia Nacional de Virus Respiratorio. Instituto Nacional de Salud Perú	Laboratorio de Referencia Nacional de Biotecnología y Biología Molecular. Instituto Nacional de Salud Perú	Carlos Padilla Rojas; Henri Bailon Calderon; Johanna Balbuena Torres; Johanna Balbuena Torrez; Karolyn Vega Chozo; Karolyn Chozo Vega; Karolyn Vega Chozo; Luis Barcena; Marco Galarza Perez; Maribel Huaringa Nuñez; Nancy Rojas Serrano; Nancy Rojas Serrano.; Omar Caceres Rey; Priscila Lope Pari
EPI_ISL_491172	Laboratorio de Referencia Nacional de Virus Respiratorio. Instituto Nacional de Salud Perú	Laboratorio de Referencia Nacional de Biotecnología y Biología Molecular. Instituto Nacional de Salud Perú.	Carlos Padilla Rojas; Henri Bailon Calderon; Johanna Balbuena Torres; Karolyn Chozo Vega; Marco Galarza Perez; Maribel Huaringa Nuñez; Nancy Rojas Serrano; Omar Caceres Rey; Priscila Lope Pari
EPI_ISL_1111069, EPI_ISL_1111074 to 1111384, EPI_ISL_1111446 to 1111505	Laboratorio de Referencia Nacional de Virus Respiratorio. Instituto Nacional de Salud Perú	Laboratorio de Referencia Nacional de Enteropatógenos. Instituto Nacional de Salud del Perú	Fiorella Orellana Peralta; Iris Silva Molina; Junior Caro Castro; Ronnie Gavilan Chavez; Veronica Hurtado Vela; Willi Quino Sifuentes
EPI_ISL_487269	Laboratorio de Referencia Nacional de Virus Respiratorio. Instituto Nacional de Salud.	Laboratorio de Referencia Nacional de Biotecnología y Biología Molecular. Instituto Nacional de Salud.	Carlos Padilla Rojas; Henri Bailon Calderon; Johanna Balbuena Torrez; Karolyn Chozo Vega; Marco Galarza Perez; Maribel Huaringa Nuñez; Nancy Rojas Serrano; Omar Caceres Rey; Priscila Lope Pari
EPI_ISL_415787, EPI_ISL_514227	Laboratorio de Referencia Nacional de Virus Respiratorio. Instituto Nacional de Salud. Peru	Laboratorio de Referencia Nacional de Biotecnologia y Biologia Molecular.Instituto Nacional de Salud.Peru	Carlos Padilla Rojas; Hemri Bailon Calderon; Henri Bailon Calderon; Johanna Balbuena Torres; Johanna Balbuena Torrez; Karolyn Vega Chozo; Marco Galarza Perez; Maribel Huaringa Nuñez; Nancy Rojas Serrano; Omar Caceres Rey; Priscila Lope Pari
EPI_ISL_514264 to 514265	Laboratorio de Referencia Nacional de Virus Respiratorio. Instituto Nacional de Salud. Perú	Laboratorio de Referencia Nacional de Biotecnologia y Biologia Molecular.Instituto Nacional de Salud.Perú	Carlos Padilla Rojas; Henri Bailon Calderon; Johanna Balbuena Torrez; Karolyn Vega Chozo; Marco Galarza Perez; Maribel Huaringa Nuñez; Nancy Rojas Serrano; Omar Caceres Rey; Priscila Lope Pari
EPI_ISL_491431	Laboratorio de Referencia Nacional de Virus Respiratorio.	Laboratorio de Referencia Nacional de Biotecnología y	Carlos Padilla Rojas; Henri Bailon Calderon; Johanna Balbuena Torres; Karolyn Vega Chozo; Marco Galarza Perez; Maribel Huaringa Nuñez; Nancy

	Instituto Nacional de Salud. Perú	Biología Molecular. Instituto Nacional de Salud.Perú	Rojas Serrano.; Omar Caceres Rey; Priscila Lope Pari
EPI_ISL_491432	Laboratorio de Referencia Nacional de Virus Respiratorio. Instituto Nacional de Salud.Perú	Laboratorio de Referencia Nacional de Biotecnología y Biología Molecular. Instituto Nacional de Salud.Perú	Carlos Padilla Rojas; Henri Bailon Calderon; Johanna Balbuena Torres; Karolyn Vega Chozo; Marco Galarza Perez; Maribel Huaringa Nuñez; Nancy Rojas Serrano.; Omar Caceres Rey; Priscila Lope Pari
EPI_ISL_529065 to 529079, EPI_ISL_540923 to 540992, EPI_ISL_568510 to 568555, EPI_ISL_729862 to 729868, EPI_ISL_729872 to 729901, EPI_ISL_812443 to 812517, EPI_ISL_812712 to 812721			
see above	Laboratorio de Referencia Nacional de Virus Respiratorios, Instituto Nacional de Salud Peru	Laboratorio de Genómica Microbiana, Universidad Peruana Cayetano Heredia	Alejandra Dávila-Barclay; Brenda Ayzanoa; Camila Castillo-Vilcahuaman; Camila Castillo-Vilcahuamán; Guillermo Salvatierra; Janet Huancachoque; Luis González; Marco Galarza; Maribel Huaringa; Nancy Rojas; Oscar Escalante; Pablo Tsukayama; Pedro E. Romero; Pool Marcos; Priscila Lope
EPI_ISL_482468	Laboratorio de Referencia Nacional de Virus Respiratorios. Instituto Nacional de Salud Peru	Laboratorio de Referencia Nacional de Biotecnologia y Biologia Molecular. Instituto Nacional de Salud Peru	Carlos Padilla Rojas; Henri Bailon Calderon; Johanna Balbuena Torres; Karolyn Vega Chozo; Maribel Huaringa Nuñez; Nancy Rojas Serrano; Omar Caceres Rey; Priscila Lope Pari
EPI_ISL_906144 to 906145, EPI_ISL_906534, EPI_ISL_906557 to 906561	Laboratorio de Salud Publica de Amazonas	Instituto Nacional de Salud- Dirección de Investigación en Salud Pública, Universidad de los Andes- Applied genomics research group, Vicerrectoria de Investigación y Creación, Universidad de los Andes- Systems and Computing Engineering Department	Carlos Franco-Muñoz; Diego A. Álvarez-Díaz; Diego Andrés Prada; Gerardo Santamaría Jorge Duitama; Héctor Alejandro Ruiz-Moreno; Jhonnatan Reales-González; Jorge Ivan Diaz; Julian Naizaque; Katherine Laiton-Donato; Laura Natalia Gonzalez; Magdalena Wiesner; Marcela Mercado-Reyes; Maria T. Herrera-Sepúlveda; Martha Lucia Ospina Martinez; Mauricio Pacheco-Montealegre; Sheryll Corchuelo; Silvia Restrepo-Restrepo
EPI_ISL_1092008	Laboratorio de Salud Publica de Bogota	Instituto Nacional de Salud- Dirección de Investigación en Salud Pública	Carlos Franco-Muñoz; Diego A. Álvarez-Díaz; Diego Andrés Prada; Gerardo Santamaría; Hector Alejandro Ruiz-Moreno; Jhonnatan Reales-González; Julian Naizaque; Katherine Laiton-Donato; Magdalena Wiesner; Marcela Mercado-Reyes; Maria T. Herrera-Sepúlveda; Martha Lucia Ospina Martinez; Sheryll Corchuelo
EPI_ISL_1137615, EPI_ISL_1137619	Laboratorio de Salud Publica de Cauca	Instituto Nacional de Salud- Dirección de Investigación en Salud Pública	Carlos Franco-Muñoz; Diego A. Álvarez-Díaz; Diego Andrés Prada; Gerardo Santamaría; Hector Alejandro Ruiz-Moreno; Jhonnatan Reales-González; Julian Naizaque; Katherine Laiton-Donato; Magdalena Wiesner; Marcela Mercado-Reyes.; Maria T. Herrera-Sepúlveda; Martha Lucia Ospina Martínez; Sheryll Corchuelo
EPI_ISL_1092005	Laboratorio de Salud Publica de Cesar	Instituto Nacional de Salud- Dirección de Investigación en Salud Pública	Carlos Franco-Muñoz; Diego A. Álvarez-Díaz; Diego Andrés Prada; Gerardo Santamaría; Hector Alejandro Ruiz-Moreno; Jhonnatan Reales-González; Julian Naizaque; Katherine Laiton-Donato; Magdalena Wiesner; Marcela Mercado-Reyes; Maria T. Herrera-Sepúlveda; Martha Lucia Ospina Martinez; Sheryll Corchuelo
EPI_ISL_1235686	Laboratorio de Salud Pública (Bogota)	Gencore- Universidad de los Andes	Alejandro Gómez; David González; Johana Hernandez Gabriela Delgado; Luisa Sacristán; Marcela Guevara-Suarez; Silvia Restrepo
EPI_ISL_845635 to 845655	Laboratorio de Salud Pública - Secretaría Distrital de Salud	Instituto Nacional de Salud - Dirección de Investigación en Salud Pública	Carlos Franco-Muñoz; Diego A. Álvarez-Díaz; Diego Andrés Prada; Gerardo Santamaría; Jonathan Reales; Julian Naizaque; Katherine Laiton-Donato; Magdalena Wiesner; Marcela Mercado-Reyes; Maria T. Herrera-Sepúlveda; Martha Lucia Ospina Martinez; Mauricio Pacheco-Montealegre; Paola Muñoz-Laiton; Sheryll Corchuelo
EPI_ISL_1137478, EPI_ISL_1303378 to 1303380	Laboratorio de Salud Pública - Secretaría Distrital de Salud	Instituto Nacional de Salud- Dirección de Investigación en Salud Pública	Carlos Franco-Muñoz; Carmen Osorio; Diana Malo; Diego A. Álvarez-Díaz; Diego Andrés Prada; Gerardo Santamaría; Hector Alejandro Ruiz-Moreno; Jhonnatan Reales-González; Juan Camilo Martinez; Julian Naizaque; Katherine Laiton-Donato; Lisseth Pardo; Magdalena Wiesner; Marcela Mercado-Reyes; Marcela Mercado-Reyes.; Maria T. Herrera-Sepúlveda; Marta Lopez Blanco; Martha Lucia Ospina Martinez; Martha Lucia Ospina Martínez; Sergio Gomez; Sheryll Corchuelo; Ángela Alarcon Cruz
EPI_ISL_906548	Laboratorio de Salud Pública Fronterizo de Arauca	Instituto Nacional de Salud- Dirección de Investigación en Salud Pública, Universidad de los Andes- Applied genomics research group, Vicerrectoria de Investigación y Creación, Universidad de los Andes- Systems and Computing Engineering Department	Carlos Franco-Muñoz; Diego A. Álvarez-Díaz; Diego Andrés Prada; Gerardo Santamaría Jorge Duitama; Héctor Alejandro Ruiz-Moreno; Jhonnatan Reales-González; Jorge Ivan Diaz; Julian Naizaque; Katherine Laiton-Donato; Laura Natalia Gonzalez; Magdalena Wiesner; Marcela Mercado-Reyes; Maria T. Herrera-Sepúlveda; Martha Lucia Ospina Martinez; Mauricio Pacheco-Montealegre; Sheryll Corchuelo; Silvia Restrepo-Restrepo
EPI_ISL_1017703	Laboratorio de Salud Pública de Bogota	Instituto Nacional de Salud- Dirección de Investigación en Salud Pública	Carlos Franco-Muñoz; Diego A. Álvarez-Díaz; Diego Andrés Prada; Gerardo Santamaría; Hector Alejandro Ruiz-Moreno; Jhonnatan Reales-González; Julian Naizaque; Katherine Laiton-Donato; Magdalena Wiesner; Marcela Mercado-Reyes; Maria T. Herrera-Sepúlveda; Martha Lucia Ospina Martinez; Sheryll Corchuelo
EPI_ISL_1036756	Laboratorio de Salud Pública de Bogotá	Instituto Nacional de Salud- Direccion de Investigacion en Salud Publica	Carlos Franco-Muñoz; Diego A. Álvarez-Díaz; Diego Andrés Prada; Gerardo Santamaría; Hector Alejandro Ruiz-Moreno; Jhonnatan Reales-González; Julian Naizaque; Katherine Laiton-Donato; Magdalena Wiesner; Marcela Mercado-Reyes; Maria T. Herrera-Sepúlveda; Martha Lucia Ospina Martinez; Sheryll Corchuelo
EPI_ISL_1017701	Laboratorio de Salud Pública de Boyacá	Instituto Nacional de Salud- Dirección de Investigación en Salud Pública	Carlos Franco-Muñoz; Diego A. Álvarez-Díaz; Diego Andrés Prada; Gerardo Santamaría; Hector Alejandro Ruiz-Moreno; Jhonnatan Reales-González; Julian Naizaque; Katherine Laiton-Donato; Magdalena Wiesner; Marcela Mercado-Reyes; Maria T. Herrera-Sepúlveda; Martha Lucia Ospina Martinez; Sheryll Corchuelo
EPI_ISL_792358 to 792370	Laboratorio de Virologia - HIEAyC San Juan de Dios	Área de Secuenciación del Laboratorio de Virología del Hospital de Niños Dr. Ricardo Gutierrez on behalf of 'Proyecto Argentino Interinstitucional de genomica de SARS-CoV-2' (PAIS Consortium)	A; Colmeiro; Ercole; Ferioli; Gatelli; Goya; LE; Lusso; M; MI; MS; Nabaes Jodar; Natale; R; S; Valinotto; Viegas, M.
EPI_ISL_965029	Laboratorio de Virologia HUCA	Laboratorio de Virologia HUCA	Abreu F; Alvarez-Arguelles ME; Boga JA; Castelló C; Costales I; Coto E; Gómez de Oña J; Martín-Rodríguez G; Melón S; Perez-Martínez Z; Rojo S; Sandoval M
EPI_ISL_470615 to 470650, EPI_ISL_623106 to 623169, EPI_ISL_717791, EPI_ISL_717793, EPI_ISL_717801 to 717802, EPI_ISL_717805 to 717808, EPI_ISL_717810 to 717831, EPI_ISL_717837 to 717840, EPI_ISL_717842 to 717898, EPI_ISL_717920 to 717957, EPI_ISL_717959 to 717961			
see above	Laboratorio de Virologia Molecular / UFRJ	Bioinformatics Laboratory / LNCC	Alexandra Gerber; Alexandra L Gerber; Amilcar Tanuri; Ana Paula Guimarães; Ana Paula de C Guimarães; Ana Tereza R de Vasconcelos; Andréa Cony Cavalcanti; CADDE-group; Carolina M Voloch; Carolina Voloch; Claudia dos Santos Rodrigues; Covid19-UFRJ Workgroup; Cynthia C Cardoso; Diana Mariani; Ester Cerdeira Sabino; Filipe Romero; Ingra Morales Claro; Jaqueline Goes de Jesus; Laboratorio Hermes Pardini; Laboratorio Simile; Luiz G P de Almeida; Luiz Gonzaga Paula de Almeida; Luís Cristóvão Pôrto; Mariane Talon; Nuno Rodrigues Faria; Orlando C. Ferreira; Otavio Bustrolini; Otavio J. Brustolini; Renato S Aguiar; Renato Santana Aguiar e Ana Tereza Vasconcelos; Ronaldo S Francisco Jr; Ronaldo da Silva F Jr; Ronaldo da Silva Francisco Junior; Terezinha M P P Castiñeira; Terezinha M P P Castiñeiras; Terezinha Marta Pereira; working group UFMG; working group UFRJ; Átila Duque Rossi
EPI_ISL_964949	Laboratorio de Virologia del HUCA	Laboratorio de Virologia del HUCA	Abreu F; Alvarez-Arguelles ME; Boga JA; Castelló C; Costales I; Coto E; Gómez de Oña J; Martín-Rodriguez G; Melon S; Perez-Martinez Z; Rojo S; Sandoval M
EPI_ISL_906533, EPI_ISL_906538, EPI_ISL_906542, EPI_ISL_906549	Laboratorio de Virologia-Instituto Nacional de Salud	Instituto Nacional de Salud- Dirección de Investigación en Salud Pública, Universidad de los Andes- Applied genomics research group, Vicerrectoria de Investigación y Creación, Universidad de los Andes- Systems and Computing Engineering Department	Carlos Franco-Muñoz; Diego A. Álvarez-Díaz; Diego Andrés Prada; Gerardo Santamaría Jorge Duitama; Héctor Alejandro Ruiz-Moreno; Jhonnatan Reales-González; Jorge Ivan Diaz; Julian Naizaque; Katherine Laiton-Donato; Laura Natalia Gonzalez; Magdalena Wiesner; Marcela Mercado-Reyes; Maria T. Herrera-Sepúlveda; Martha Lucia Ospina Martinez; Mauricio Pacheco-Montealegre; Sheryll Corchuelo; Silvia Restrepo-Restrepo
EPI_ISL_974150	Laboratorio de Virología / Hospital Universitario Central de Asturias (HUCA)	Laboratorio de Virología / Hospital Universitario Central de Asturias (HUCA)	Abreu F; Alvarez-Arguelles ME; Boga JA; Castelló C; Costales I; Coto E; Gómez de Oña J; Martín-Rodríguez G; Melón S; Perez-Martínez Z; Rojo S; Sandoval M
EPI_ISL_965033, EPI_ISL_1020305, EPI_ISL_1033159, EPI_ISL_1033161 to 1033162, EPI_ISL_1034145 to 1034151, EPI_ISL_1034153, EPI_ISL_1034190 to 1034195, EPI_ISL_1034209 to 1034211, EPI_ISL_1034227 to 1034230, EPI_ISL_1034303, EPI_ISL_1098832, EPI_ISL_1098841 to 1098842, EPI_ISL_1098867 to 1098871, EPI_ISL_1098891 to 1098892, EPI_ISL_1098894, EPI_ISL_1098896, EPI_ISL_1098898, EPI_ISL_1103544 to 1103546, EPI_ISL_1103548 to 1103549, EPI_ISL_1103553 to 1103554, EPI_ISL_1123388, EPI_ISL_1125886, EPI_ISL_1138056, EPI_ISL_1138296, EPI_ISL_1138408 to 1138409, EPI_ISL_1242451, EPI_ISL_1310778 to 1310781, EPI_ISL_1310784 to 1310786			
see above	Laboratorio de Virología HUCA	Laboratorio de Virología HUCA	Abreu F; Alvarez-Arguelles ME; Boga JA; Castelló C; Costales I; Coto E; Gómez de Oña J; Martín-Rodríguez G; Melón S; Perez-Martínez Z; Rojo S; Sandoval M
EPI_ISL_992956	Laboratorio de Virología HUCA	Laboratorio de Virología Hospital Universitario Central de Asturias (HUCA)	Abreu F; Alvarez-Arguelles ME; Boga JA; Castelló C; Costales I; Coto E; Gómez de Oña J; Martín-Rodríguez G; Melón S; Perez-Martínez Z; Rojo S; Sandoval M

EPI_ISL_1123413 to 1123414, EPI_ISL_1125210, EPI_ISL_1125577	Laboratorio de Virología Hospital Universitario Central de Asturias (HUCA)	Laboratorio de Virología Hospital Universitario Central de Asturias (HUCA)	Abreu F; Alvarez-Arguelles ME; Boga JA; Castelló C; Costales I; Coto E; Gómez de Oña J; Martín-Rodríguez G; Melón S; Perez-Martínez Z; Rojo S; Sandoval M
EPI_ISL_792397 to 792401	Laboratorio de Virología del Hospital de Niños Dr. Ricardo Gutierrez	Biocódices SA. on behalf of 'Proyecto Argentino Interinstitucional de genomica de SARS-CoV-2' (PAIS Consortium)	Acevedo; Alexay; Alvarez Lopez; Berros JM; C; Dopazo H; E; Gravis; Jacques; ME; Mistchenko; O; S; Zubrzycki J
EPI_ISL_792522 to 792524	Laboratorio de Virología del Hospital de Niños Dr. Ricardo Gutierrez	Grupo de Genómica y Bioinformática del Instituto de Investigación de la Cadena Láctea CONICET-INTA on behalf of 'Proyecto Argentino Interinstitucional de genomica de SARS-CoV-2' (PAIS Consortium)	AF; AS; Acevedo; Alexay; Alvarez Lopez; Amadio; Aulicino; C; Eberhardt; G; Goya; Irazoqui; Jacques; König; M; ME; MF; MS; Mistchenko; Nabaes Jodar; O; P; S; Torres; Viegas, M.
EPI_ISL_430795, EPI_ISL_430799 to 430801, EPI_ISL_430803 to 430804, EPI_ISL_430807, EPI_ISL_430809 to 430818, EPI_ISL_792117 to 792290, EPI_ISL_793013			
see above	Laboratorio de Virología del Hospital de Niños Dr. Ricardo Gutierrez	Área de Secuenciación del Laboratorio de Virología del Hospital de Niños Dr. Ricardo Gutierrez on behalf of 'Proyecto Argentino Interinstitucional de genomica de SARS-CoV-2' (PAIS Consortium)	AS; Acevedo; Alexay; Alvarez Lopez; C; E; Gallino; Goya; Gravis; I; Jacques; LE; Lusso; ME; MI; MS; Mistchenko; Nabaes Jodar; Natale; O; Primost; S; Valinotto; Viegas, M.
EPI_ISL_671978	Laboratorio de Virología y Microbiología Molecular, Depto. de Microbiología, Facultad de Medicina, Universidad de El Salvador/INS-laboratorio de Ref. Ministerio de Salud	Laboratorio de Virología y Microbiología Molecular, Depto. de Microbiología, Facultad de Medicina, Universidad de El Salvador/INS-laboratorio de Ref. Ministerio de Salud	Rivera NR et al
EPI_ISL_672573	Laboratorio de Virología y Microbiología Molecular, Depto. de Microbiología, Facultad de Medicina, Universidad de El Salvador/INS-laboratorio de Ref. Ministerio de Salud. 1*: Dr. Noé Rigoberto Rivera, profesor del Departamento de Bioquímica e Investigador adjunto de la Sección de Virología y Microbiología Molecular;director de la Unidad de Investigaciones Científicas (UNICA), Facultad de Medicina Universidad de El Salvador,El Salvador C.A 1**: Dr. Carlos Alexander Ortega Pérez, Profesor del Departamento de Microbiología;investigador y Jefe de la Sección de Virología, Director Metodológico de la Unidad de Investigaciones Científicas(UNICA), Facultad de Medicina, Universidad de El Salvador, El Salvador C.A 2*: Dra. Xochitl Sandoval López:Directora e investigadora del Instituto Nacional de Salud (INS) El Salvador C.A 2** Dr. Hernandez Avila CarlosE Director de Gobernanza e investigador del Instituto Nacional de Salud (INS) El Salvador C.A	Laboratorio de Virología y Microbiología Molecular, Depto. de Microbiología, Facultad de Medicina, Universidad de El Salvador/INS-laboratorio de Ref. Ministerio de Salud	Rivera NR et al
EPI_ISL_672572	Laboratorio de Virología y Microbiología Molecular, Depto. de Microbiología, Facultad de Medicina, Universidad de El Salvador/INS-laboratorio de Ref. Ministerio de Salud. 1*: Dr. Noé Rigoberto Rivera, profesor del Departamento de Bioquímica e Investigador adjunto de la Sección de Virología y Microbiología Molecular; director de la Unidad de Investigaciones Científicas (UNICA), Facultad de Medicina Universidad de El Salvador, El Salvador C.A 1**: Dr. Carlos Alexander Ortega Pérez, Profesor del Departamento de Microbiología; investigador y Jefe de la Sección de Virología, Director Metodológico de la Unidad de Investigaciones Científicas (UNICA), Facultad de Medicina, Universidad de El Salvador, El Salvador C.A 2*: Dra. Xochitl Sandoval López: Directora e investigadora del Instituto Nacional de Salud (INS) El Salvador C.A 2** Dr. Hernandez Avila Carlos E Director de Gobernanza e investigador del Instituto Nacional de Salud (INS) El Salvador C.A	Laboratorio de Virología y Microbiología Molecular, Depto. de Microbiología, Facultad de Medicina, Universidad de El Salvador/INS-laboratorio de Ref. Ministerio de Salud.	Rivera NR et al
EPI_ISL_671974, EPI_ISL_672570	Laboratorio de Virología y Microbiología Molecular, Depto. de Microbiología, Facultad de Medicina, Universidad de El Salvador/INS-laboratorio de Ref. Ministerio de Salud	Laboratorio de Virología y Microbiología Molecular, Depto. de Microbiología, Facultad de Medicina, Universidad de El Salvador/INS-laboratorio de Ref. Ministerio de Salud	Carlos Hernández Ávila; Ortega Pérez CA et al; Ortega-Pérez C A; Rivera NR; Xochitl Sandoval López
EPI_ISL_672012	Laboratorio de Virología y Microbiología Molecular, Depto. de Microbiología, Facultad de Medicina, Universidad de El Salvador/INS-laboratorio de Ref. Ministerio de Salud. 1*: Dr. Noé Rigoberto Rivera, profesor del Departamento de Bioquímica e Investigador adjunto de la Sección de Virología y Microbiología Molecular; director de la Unidad de Investigaciones Científicas (UNICA), Facultad de Medicina Universidad de El Salvador, El Salvador C.A 1**: Dr. Carlos Alexander Ortega Pérez, Profesor del Departamento de Microbiología; investigador y Jefe de la Sección de Virología, Director Metodológico de la Unidad de Investigaciones Científicas (UNICA), Facultad de Medicina, Universidad de El Salvador, El Salvador C.A 2*: Dra. Xochitl Sandoval López: Directora e investigadora del Instituto Nacional de Salud (INS) El Salvador C.A 2** Dr. Hernandez Avila Carlos E Director de Gobernanza e investigador del Instituto Nacional de Salud (INS) El Salvador C.A	Laboratorio de Virología y Microbiología Molecular, Depto. de Microbiología, Facultad de Medicina, Universidad de El Salvador/INS-laboratorio de Ref. Ministerio de Salud.	Ortega Pérez CA et al
EPI_ISL_755304 to 755305	Laboratorio de Virología, Centro de Biotecnología Acuícola, Universidad de Santiato de Chile	Center for Mathematical Modeling and Center for Genome Regulation. Santiago, Chile	A. Sandino; Allende ML; Arriagada G; Bastias M; Bustos F; Castro E; González M; M; Maass A; Meneses C.; Montecino; Orellana A; Reyes-López F.; Sanhueza D; Travisany D
EPI_ISL_1220054	Laboratorio de biología molecular diagnóstico de COVID-19 U Caldas	Instituto Nacional de Salud- Dirección de Investigación en Salud Pública	Carlos Franco-Muñoz; Diego A. Álvarez-Díaz; Diego Andrés Prada; Gerardo Santamaría; Hector Alejandro Ruiz-Moreno; Jhonnatan Reales-González; Julian Naizaque; Katherine Laiton-Donato; Magdalena Wiesner; Marcela Mercado-Reyes.; Maria T. Herrera-Sepúlveda; Martha Lucia Ospina Martínez; Sheryll Corchuelo
EPI_ISL_792291 to 792298	Laboratorio de genética y biología molecular del Hospital de trauma y emergencia Dr Federico Abete	Área de Secuenciación del Laboratorio de Virología del Hospital de Niños Dr. Ricardo Gutierrez on behalf of 'Proyecto Argentino Interinstitucional de genomica de SARS-CoV-2' (PAIS Consortium)	Gallino; Goya; I; LE; Lusso; MI; MS; Nabaes Jodar; Natale; Primost; S; Valinotto; Viegas, M.
EPI_ISL_956299 to 956300	Laboratorio de salud publica Arauca	Instituto Nacional de Salud- Dirección de Investigación en	Carlos Franco-Muñoz; Diego A. Álvarez-Díaz; Diego Andrés Prada; Gerardo Santamaría; Hector Alejandro Ruiz-Moreno; Jhonnatan Reales-González;

		Salud Pública	Julian Naizaque; Katherine Laiton-Donato; Magdalena Wiesner; Marcela Mercado-Reyes; Maria T. Herrera-Sepúlveda; Martha Lucia Ospina Martinez; Mauricio Pacheco-Montealegre; Sheryll Corchuelo
EPI_ISL_906530	Laboratorio de salud publica de Bogota	Instituto Nacional de Salud- Dirección de Investigación en Salud Pública, Universidad de los Andes- Applied genomics research group, Vicerrectoria de Investigación y Creación, Universidad de los Andes- Systems and Computing Engineering Department	Carlos Franco-Muñoz; Diego A. Álvarez-Díaz; Diego Andrés Prada; Gerardo Santamaría Jorge Duitama; Héctor Alejandro Ruiz-Moreno; Jhonnatan Reales-González; Jorge Ivan Diaz; Julian Naizaque; Katherine Laiton-Donato; Laura Natalia Gonzalez; Magdalena Wiesner; Marcela Mercado-Reyes; Maria T. Herrera-Sepúlveda; Martha Lucia Ospina Martinez; Mauricio Pacheco-Montealegre; Sheryll Corchuelo; Silvia Restrepo-Restrepo
EPI_ISL_956298	Laboratorio de salud publica del Valle del Cauca	Instituto Nacional de Salud- Dirección de Investigación en Salud Pública	Carlos Franco-Muñoz; Diego A. Álvarez-Díaz; Diego Andrés Prada; Gerardo Santamaría; Hector Alejandro Ruiz-Moreno; Jhonnatan Reales-González; Julian Naizaque; Katherine Laiton-Donato; Magdalena Wiesner; Marcela Mercado-Reyes; Maria T. Herrera-Sepúlveda; Martha Lucia Ospina Martinez; Mauricio Pacheco-Montealegre; Sheryll Corchuelo
EPI_ISL_906531	Laboratorio de salud publica del Valle del Cauca	Instituto Nacional de Salud- Dirección de Investigación en Salud Pública, Universidad de los Andes- Applied genomics research group, Vicerrectoria de Investigación y Creación, Universidad de los Andes- Systems and Computing Engineering Department	Carlos Franco-Muñoz; Diego A. Álvarez-Díaz; Diego Andrés Prada; Gerardo Santamaría Jorge Duitama; Héctor Alejandro Ruiz-Moreno; Jhonnatan Reales-González; Jorge Ivan Diaz; Julian Naizaque; Katherine Laiton-Donato; Laura Natalia Gonzalez; Magdalena Wiesner; Marcela Mercado-Reyes; Maria T. Herrera-Sepúlveda; Martha Lucia Ospina Martinez; Mauricio Pacheco-Montealegre; Sheryll Corchuelo; Silvia Restrepo-Restrepo
EPI_ISL_792375 to 792385	Laboratorio de salud pública, Facultad de Ciencias Exactas, Universidad Nacional de La Plata	Área de Secuenciación del Laboratorio de Virología del Hospital de Niños Dr. Ricardo Gutierrez on behalf of 'Proyecto Argentino Interinstitucional de genomica de SARS-CoV-2' (PAIS Consortium)	A; Angeletti; Cordero; Goya; LE; Lusso; MI; MS; Nabaes Jodar; Nadalich; Natale; R; S; Toro; V; Valinotto; Viegas, M.
EPI_ISL_1091922 to 1091923	Laboratorio de salud público de Atlantico	Instituto Nacional de Salud- Dirección de Investigación en Salud Pública	Carlos Franco-Muñoz; Diego A. Álvarez-Díaz; Diego Andrés Prada; Gerardo Santamaría; Hector Alejandro Ruiz-Moreno; Jhonnatan Reales-González; Julian Naizaque; Katherine Laiton-Donato; Magdalena Wiesner; Marcela Mercado-Reyes; Maria T. Herrera-Sepúlveda; Martha Lucia Ospina Martinez; Sheryll Corchuelo
EPI_ISL_792299 to 792300	Laboratorio del Hospital Alemán	Área de Secuenciación del Laboratorio de Virología del Hospital de Niños Dr. Ricardo Gutierrez on behalf of 'Proyecto Argentino Interinstitucional de genomica de SARS-CoV-2' (PAIS Consortium)	Della Latta; E; García Allende; Goya; Ibañez; LE; Lusso; MI; MP; MS; N; Nabaes Jodar; Natale; S; Valinotto; Viegas, M.
EPI_ISL_792306 to 792319	Laboratorio del Hospital El Cruce Dr. Néstor C. Kirchner	Área de Secuenciación del Laboratorio de Virología del Hospital de Niños Dr. Ricardo Gutierrez on behalf of 'Proyecto Argentino Interinstitucional de genomica de SARS-CoV-2' (PAIS Consortium)	Goya; LE; Lusso; M; MI; MS; Nabaes Jodar; Natale; Rahhal; S; Valinotto; Viegas, M.; Zubieta
EPI_ISL_792371 to 792374	Laboratorio del Hospital Interzonal General de Agudos Eva Perón	Área de Secuenciación del Laboratorio de Virología del Hospital de Niños Dr. Ricardo Gutierrez on behalf of 'Proyecto Argentino Interinstitucional de genomica de SARS-CoV-2' (PAIS Consortium)	C; Carulla; Goya; Kairiyama; LE; Lusso; M; MI; MS; Nabaes Jodar; Natale; Pengue; Piñeyro C; S; Valinotto; Viegas, M.
EPI_ISL_792525	Laboratorio del Hospital Interzonal General de Agudos Evita	Grupo de Genómica y Bioinformática del Instituto de Investigación de la Cadena Láctea CONICET-INTA on behalf of 'Proyecto Argentino Interinstitucional de genomica de SARS-CoV-2' (PAIS Consortium)	AF; Alexay; Amadio; Aulicino; C; Desimone; E; Eberhardt; G; Goya; Grossi; I; Irazoqui; König; L; Luczac; M; MF; MS; Musto; Nabaes Jodar; O; P; S; Serrano; Torres; Viegas, M.
EPI_ISL_792333 to 792350	Laboratorio del Hospital Interzonal General de Agudos Evita	Área de Secuenciación del Laboratorio de Virología del Hospital de Niños Dr. Ricardo Gutierrez on behalf of 'Proyecto Argentino Interinstitucional de genomica de SARS-CoV-2' (PAIS Consortium)	A; Desimone; E; Goya; Grossi; I; L; LE; Luczac; Lusso; MI; MS; Musto; Nabaes Jodar; Natale; O; S; Serrano; Valinotto; Viegas, M.
EPI_ISL_792443 to 792448, EPI_ISL_792450 to 792473	Laboratorio del Hospital Regional Ushuaia Gdor. Ernesto Campos	Hospital Regional Ushuaia - Centro Austral De Investigaciones Científicas - Universidad Nacional De Tierra Del Fuego on behalf of 'Proyecto Argentino Interinstitucional de genomica de SARS-CoV-2' (PAIS Consortium)	Boutureira, MF.; CA; CB; CF; Castro; Ceballos; Cáceres; De Roccis; F; G; Gallego; Gramundi; ID; Nardi; SB; SG; Yulan
EPI_ISL_983326	Laboratorio di Genetica Medica Ospedale Belcolle	INMI Lazzaro Spallanzani IRCCS	A Di Caro; B Bartolini; C.E.M Gruber; E Giombini; F Messina; F Natoni; G Pessina; M Rueca; MR Capobianchi; O Butera
EPI_ISL_413489	Laboratorio di Microbiologia e Virologia, Università Vita-Salute San Raffaele, Milano	Laboratorio di Microbiologia e Virologia, Università Vita-Salute San Raffaele, Milano	C. Di Resta; E. Boeri; E. Criscuolo; G. Lo Raso; I. Negri; M. Castelli; M. Clementi; M. Sampaolo; N. Mancini & N. Clementi; R. Burioni; R. Ferrarese; R.A Diotti; V. Amato; V. Caputo
EPI_ISL_985027 to 985031	Laboratorio di Patologia Clinica, Ospedale San Paolo in Valloria, ASL 2 Liguria	U.O. Igiene, Ospedale Policlinico San Martino	Bruzzone Bianca; Caligiuri Patrizia; De Pace Vanessa; Domnich Alexander; Icardi Giancarlo; Lillo Flavia; Orsi Andrea; Ricucci Valentina
EPI_ISL_1020310, EPI_ISL_1020314, EPI_ISL_1020329, EPI_ISL_1020331, EPI_ISL_1020333 to 1020337, EPI_ISL_1020339, EPI_ISL_1081953, EPI_ISL_1176355, EPI_ISL_1241860			
see above	Laboratorio di Riferimento Regionale della Sicilia Occidentale per l'Emergenza COVID-19	Laboratorio di Riferimento Regionale della Sicilia Occidentale per l'Emergenza COVID-19	Carmelo Massimo Maida; Claudio Costantino; Daniela Di Naro; Fabio Tramuto; Francesco Vitale; Giorgio Graziano; Giulia Randazzo; Stefano Giaramida; Vincenzo Restivo; Walter Mazzucco
EPI_ISL_710503	Laboratorio specialistico UOC Ematologia - Ospedale "San Francesco" - ATS-ASSL Nuoro	Laboratorio specialistico UOC Ematologia - Ospedale "San Francesco" - ATS-ASSL Nuoro	Giovanna Piras
EPI_ISL_961466	Laboratorios Lister	Instituto de Diagnostico y Referencia Epidemiologicos (INDRE)	Abril Rodriguez-Maldonado; Claudia Wong-Arambula; Ernesto Ramirez-Gonzalez.; Fabiola Garces-Ayala; Gisela Barrera-Badillo; Irma Lopez-Martinez; Lucia Hernandez-Rivas; Natividad Cruz-Ortiz; Tatiana Nunez-Garcia
EPI_ISL_904125	Laboratorium Analityczne Szpital Specjalistyczny w Chorzowie	National Institute of Public Health - National Institute of Hygiene	Wokowicz Tomasz; Zacharczuk Katarzyna
EPI_ISL_1239556, EPI_ISL_1239600 to 1239605, EPI_ISL_1239680, EPI_ISL_1239749 to 1239755	Laboratorium Badawczo-Rozwojowe Vitagenum	1. National Institute of Public Health - National Institute of Hygiene; 2. Eurofins Genomics Europe Sequencing GmbH	ECDC COVID-19 WGS support team; Eurofins Genomics Europe Sequencing Team; Gierczyki Rafa; Sadkowska-Todys Magorzata; Wokowicz Tomasz; Zacharczuk Katarzyna
EPI_ISL_882960 to 882961	Laboratorium Diagnostyi Mikrobiologicznej z Pracowni Prtka Grulicy SPSzW im. Jana Boego w Lublinie	National Institute of Public Health - National Institute of Hygiene	Gawor Jan; Wokowicz Tomasz; Zacharczuk Katarzyna
EPI_ISL_1239606, EPI_ISL_1239756 to 1239757	Laboratorium Genetyczne Alab Plus Sp. z o.o.	1. National Institute of Public Health - National Institute of Hygiene; 2. Eurofins Genomics Europe Sequencing GmbH	ECDC COVID-19 WGS support team; Eurofins Genomics Europe Sequencing Team; Gierczyki Rafa; Sadkowska-Todys Magorzata; Wokowicz Tomasz; Zacharczuk Katarzyna
EPI_ISL_905762	Laboratorium Genloxa Sp. z o.o.	National Institute of Public Health - National Institute of Hygiene	Wokowicz Tomasz; Zacharczuk Katarzyna
EPI_ISL_511879	Laboratorium Kesehatan Provinsi Jawa Barat	Molecular Genetics Laboratory-Faculty of Medicine-Universitas Padjadjaran; School of Life Sciences and Technology & School of Pharmacy-Institut Teknologi Bandung; Laboratorium Kesehatan Provinsi Jawa Barat	Agung Eru Wibowo; Azzania Fibriani; Catur Riani; Cut Nur Cinthia Alamanda; Ema Rahmawati; Hammam Riza; Hesti Lina Wiraswati; Husna Nugrahapraja; Lia Faridah; Marselina Irasonia Tan; Rifky Waluyajati Rachman; Ryan Bayusantika Ristandi; Savira Ekawardhani; Soni Solistia Wirawan; Tarwadi; Yunia Sribudiani
EPI_ISL_528745	Laboratorium Kesehatan Provinsi Jawa Barat	School of Life Sciences and Technology & School of Pharmacy-Institut Teknologi Bandung; Molecular Genetics	Agung Eru Wibowo; Azzania Fibriani; Catur Riani; Cut Nur Cinthia Alamanda; Ema Rahmawati; Fensi Amalina; Hammam Riza; Hesti Lina Wiraswati; Husna Nugrahapraja; Irvan Faizal; Lia Faridah; Marselina Irasonia Tan; Mas Rizky A.A. Syamsunarno; Rifky Waluyajati Rachman; Ryan Bayusantika

		Laboratory-Faculty of Medicine-Universitas Padjadjaran; Laboratorium Kesehatan Provinsi Jawa Barat	Ristandi; Savira Ekawardhani; Sony Solistia Wirawan; Tarwadi; Tri Hanggono Achmad; Yunia Sribudiani
EPI_ISL_791977	Laboratorium Mikrobiologi Samarinda	National Institute of Health Research and Development	AA; HA; HD; Ikawati; KD; KNA; N; Nugraha; Pangesti; Pawestri; Puspa; Puspandari; Setiawaty; Soekarso; Subangkit; T; Tandirogang; V; Yadi
EPI_ISL_905752 to 905753	Laboratorium Mikrobiologiczne SYNEVO	National Institute of Public Health - National Institute of Hygiene	Wokowicz Tomasz; Zacharczuk Katarzyna
EPI_ISL_775589, EPI_ISL_831989	Laboratorium for klinisk mikrobiologi	The Public Health Agency of Sweden	Department of Microbiology; The Public Health Agency of Sweden
EPI_ISL_1239561, EPI_ISL_1239618 to 1239620, EPI_ISL_1239764	Laboratorum Epidemiologii WSSE w Szczecinie	1. National Institute of Public Health - National Institute of Hygiene; 2. Eurofins Genomics Europe Sequencing GmbH	ECDC COVID-19 WGS support team; Eurofins Genomics Europe Sequencing Team; Gierczyki Rafa; Sadkowska-Todys Magorzata; Wokowicz Tomasz; Zacharczuk Katarzyna
EPI_ISL_832182 to 832183, EPI_ISL_832188, EPI_ISL_832190 to 832195, EPI_ISL_832198, EPI_ISL_890895	Laboratory Analyses Med	National Reference Center for Viruses of Respiratory Infections, Institut Pasteur, Paris	Angela Brisebarre; Brieuc Gestin; Brieuc Lefaure; Camille Capel; Céline Menaut; Elise Liscoet; Etienne Simon-Lorière; Marion Barbet; Maud Vanpeene; Méline Bizard; Sylvie Behillil; Sylvie van der Werf; Vincent Enouf
EPI_ISL_1193260 to 1193373, EPI_ISL_1220894 to 1222442, EPI_ISL_1255086 to 1255101, EPI_ISL_1291198 to 1291306, EPI_ISL_1298247 to 1298429	Laboratory Corporation of America	Centers for Disease Control and Prevention Division of Viral Diseases, Pathogen Discovery	Amanda Douglas; Amanda Suchanek; Andrea Throop; Ayla Burns; Ben L. Rambo-Martin; Bobbi Croy; Brian Krueger; Brian Norvell; Christos Petropoulos; Clinton R. Paden; Craig Lukasik; Dakota Howard; Debbie Boles; Dhwani Batra; Duncan MacCannell; Eyad Almasri; Eyad Almasri Debbie Boles; Goran Stevovic; Howard Engler; Hrushikesh Deshmukh; Jake Humphrey; Jana Schroth; Joe Voshell; John Pruitt; Jonathan Meltzer; Jonathan Williams; Kimberly Wagner; Lax Iyer; Lyndon Tilson; Manoj Jain; Marcia Eisenberg; Mary Ann Cristobal; Mary Williamson; Michael Levandoski; Mike Sapeta; Mindy Nye; Minoo Agarwal; Mohan Kolli; Nuthawin Charoensri; Oren Cohen; Peter W. Cook; Prashant Gupta; Qian Zeng; Rama Ghatti; Scott Parker; Scott Ryan; Stanley Letovsky; Steven Ragan; Suresh Babu Selvaraju; Susan Countryman; Susan Hicks; Suxiang Tong; Suzanne Dale; Thomas Urban; Tim Kuphal; Tricia Zwiefelhofer; Vincent Drouillon
EPI_ISL_1020592 to 1022419, EPI_ISL_1025963 to 1026865, EPI_ISL_1027668 to 1029955, EPI_ISL_1030503 to 1032582, EPI_ISL_1036937 to 1038790, EPI_ISL_1081200 to 1081218, EPI_ISL_1081267 to 1081283, EPI_ISL_1159885 to 1160122, EPI_ISL_1160124, EPI_ISL_1160126, EPI_ISL_1160128, EPI_ISL_1160130, EPI_ISL_1160132, EPI_ISL_1160134, EPI_ISL_1160136, EPI_ISL_1160139, EPI_ISL_1160141, EPI_ISL_1160143, EPI_ISL_1160145, EPI_ISL_1160147, EPI_ISL_1160149, EPI_ISL_1160151, EPI_ISL_1160153 to 1160154, EPI_ISL_1160156, EPI_ISL_1160158, EPI_ISL_1160160, EPI_ISL_1160162, EPI_ISL_1160164, EPI_ISL_1160166, EPI_ISL_1160168, EPI_ISL_1160170, EPI_ISL_1160173, EPI_ISL_1160175, EPI_ISL_1160177, EPI_ISL_1160179, EPI_ISL_1160181, EPI_ISL_1160183, EPI_ISL_1160185, EPI_ISL_1160187, EPI_ISL_1160189, EPI_ISL_1160191, EPI_ISL_1160193, EPI_ISL_1160196 to 1160197, EPI_ISL_1160199, EPI_ISL_1160201, EPI_ISL_1160203, EPI_ISL_1160206, EPI_ISL_1160208, EPI_ISL_1160210, EPI_ISL_1160212, EPI_ISL_1160214, EPI_ISL_1160216, EPI_ISL_1160218, EPI_ISL_1160220 to 1161005, EPI_ISL_1161007 to 1161008, EPI_ISL_1161010, EPI_ISL_1161012, EPI_ISL_1161015, EPI_ISL_1161017, EPI_ISL_1161019, EPI_ISL_1161021, EPI_ISL_1161023, EPI_ISL_1161025, EPI_ISL_1161027, EPI_ISL_1161029, EPI_ISL_1161031, EPI_ISL_1161033, EPI_ISL_1161035, EPI_ISL_1161037, EPI_ISL_1161039, EPI_ISL_1161042, EPI_ISL_1161044, EPI_ISL_1161046, EPI_ISL_1161048, EPI_ISL_1161051, EPI_ISL_1161053, EPI_ISL_1161055, EPI_ISL_1161057, EPI_ISL_1161059, EPI_ISL_1161061, EPI_ISL_1161063, EPI_ISL_1161066, EPI_ISL_1161068, EPI_ISL_1161070, EPI_ISL_1161072, EPI_ISL_1161074, EPI_ISL_1161076, EPI_ISL_1161078 to 1161400, EPI_ISL_1161416 to 1162761, EPI_ISL_1162763 to 1163161, EPI_ISL_1163163 to 1163529, EPI_ISL_1255296 to 1255307, EPI_ISL_1255391 to 1255397			
see above	Laboratory Corporation of America	Respiratory Viruses Branch, Division of Viral Diseases, Centers for Disease Control and Prevention	Alexandre Bolze; Amanda Douglas; Amanda Suchanek; Andrea Throop; Ary Ascencio; Ayla Burns; Ben L. Rambo-Martin; Bobbi Croy; Brad Sickler; Brian Krueger; Brian Norvell; Charlotte Rivera-Garcia; Christine Tran; Christos Petropoulos; Clinton R. Paden; Craig Lukasik; Dakota Howard; David Becker; Dhwani Batra; Duncan MacCannell; Efren Sandoval; Eileen de Feo; Elizabeth Cirulli; Eric Allen; Eyad Almasri Debbie Boles; Geraint Levan; Goran Stevovic; Howard Engler; Hrushikesh Deshmukh; Jake Humphrey; James Lu; Jan Antico; Jana Schroth; Jason Nguyen; Jimmy Ramirez; Jingtao Liu; Joe Voshell; John Pruitt; Jonathan Meltzer; Jonathan Williams; Kelly Schiabor Barrett; Kim Gietzen; Kimberly Wagner; Lax Iyer; Lyndon Tilson; Magnus Isaksson; Manoj Jain; Marc Laurent; Marcia Eisenberg; Mary Ann Cristobal; Mary Williamson; Matthew Tolentino; Michael Levandoski; Mike Sapeta; Mindy Nye; Minoo Agarwal; Mohan Kolli; Nicole L. Washington; Nuthawin Charoensri; Oren Cohen; Peter W. Cook; Phil Febbo; Prashant Gupta; Qian Zeng; Rama Ghatti; Ryan Cho; Scott Parker; Scott Ryan; Shannon Wickline; Sherry Wang; Simon White; Stanley Letovsky; Steven Ragan; Summer Galloway; Suresh Babu Selvaraju; Susan Countryman; Susan Hicks; Suxiang Tong; Suzanne Dale; Thomas Urban; Tim Kuphal; Tricia Zwiefelhofer; Tyler Cassens; Vincent Drouillon; William Lee
EPI_ISL_476403 to 476405	Laboratory DASA	Instituto de Medicina Tropical da Univesidade de São Paulo	Camila Alves Maia da Silva; Darlan da Silva Candido; Erika Regina Manuli; Ester Sabino; Flavia Cristina da Silva Sales; Giula Magalhaes Ferreira; Jaqueline Goes de Jesus; Julien Theze; Mariana Severo Ramundo; Nuno Faria; Samples: José Eduardo Levi; Sequencing: Ingra Morales Claro; Thais de Moura Coletti
EPI_ISL_514425 to 514431, EPI_ISL_541649 to 541655, EPI_ISL_541657 to 541662, EPI_ISL_582530, EPI_ISL_644565 to 644567, EPI_ISL_904008	Laboratory Diagnostic, Veterinary Specialized Institute Kraljevo	Laboratory Diagnostic, Veterinary Specialized Institute Kraljevo	Afonso, C.; Banovic Djeri, B.; Jankovic, M.; Jovanovic, T.; Knezevic, A.; Petrovic, T.; Sekler, M.; Tesovic, B.; Vidanovic, D.; Volkening, J.
EPI_ISL_445086 to 445087, EPI_ISL_450415, EPI_ISL_482744, EPI_ISL_541656	Laboratory Diagnostic, Veterinary Specialized Institute Kraljevo	Laboratory Diagnostic, Veterinary Specialized Institute Kraljevo	Afonso, C.; Banovic Djeri, B.; C.L.; Debeljak; Debeljak, Z.; Dmitric, M.; J. and Alfonso; J. and Alfonso, C.; Jankovic, M.; Jovanovic, T.; Knezevic, A.; Matovic, K.; Petrovic, T.; Sekler, M.; Skadric, I.; Tesovic, B.; Tolic, A.; Vaskovic, N.; Vidanovic, D.; Volkening; Volkening, J.; Z. and Vaskovic, N.
EPI_ISL_754902 to 754913, EPI_ISL_1295168 to 1295170, EPI_ISL_1295175 to 1295176, EPI_ISL_1295181	Laboratory Diagnostics and Clinical Immunology of Developmental Age, Medical University of Warsaw	genXone SA, Research & Development Laboratory; The Faculty of Mathematics, Informatics and Mechanics of the University of Warsaw	Aleksandra Gidlewicz; Anna Brylak-Blaszkow; Anna Gambin; Grzegorz Nowicki; Jakub Grabowski; Karol Szeszko; Lukasz Krych; Maciej Sykulski; Michal Kaszuba; Micha Kaszuba; Monika Makowska-Woniak; Natalia Drweska-Matelska; Natalia Drwska-Matelska; Urszula Demkow; ukasz Krych
EPI_ISL_476220 to 476221, EPI_ISL_476230 to 476231, EPI_ISL_476233 to 476236, EPI_ISL_476406 to 476407, EPI_ISL_476426 to 476427	Laboratory Fleury	Instituto de Medicina Tropical da Univesidade de São Paulo	Camila Alves Maia da Silva; Darlan da Silva Candido; Erika Regina Manuli; Ester Sabino; Flavia Cristina da Silva Sales; Giula Magalhaes Ferreira; Jaqueline Goes de Jesus; Julien Theze; Mariana Severo Ramundo; Nuno Faria; Samples: Celso Granato; Sequencing: Ingra Morales Claro; Thais de Moura Coletti
EPI_ISL_415741 to 415743, EPI_ISL_417518 to 417525, EPI_ISL_424969 to 424975, EPI_ISL_424978, EPI_ISL_444274 to 444278, EPI_ISL_452178 to 452179, EPI_ISL_464092 to 464094, EPI_ISL_660543 to 660545, EPI_ISL_667807 to 667809, EPI_ISL_956317 to 956318, EPI_ISL_956320 to 956331			
see above	Laboratory Medicine	Department of Laboratory Medicine, Lin-Kou Chang Gung Memorial Hospital, Taoyuan, Taiwan	Cheng-Hsun Chiu; Cheng-Ta Yang; Chi-Hsien Huang; Chung-Guei Huang; Guang-Wu Chen; Kuang-Tso Le; Kuo-Chien Tsao; Kuo-Ming Lee; Mei-Jen Hsiao; Peng-Nien Huang; Po-Wei Huang; Shin-Ru Shih; Shu-Li Yang; Shu-Min Lin; Yi-Chun Liu; Yu-Nong Gong
EPI_ISL_411915	Laboratory Medicine	Department of Laboratory Medicine, Lin-Kou Chang Gung Memorial Hospital, Taoyuan, Taiwan.	Chung-Guei Huang; Kuo-Chien Tsao; Shin-Ru Shih; Shu-Li Yang; Yhu-Chering Huang; Yi-Chun Li; Yu-Nong Gong
EPI_ISL_529208 to 529212	Laboratory Medicine, University of Washington	University of Washington, Laboratory Medicine	Greninger, A.; Jerome, K.; Roychoudhury, P.
EPI_ISL_632286, EPI_ISL_738080	Laboratory Services Section, Texas Department of State Health Services	Laboratory Services Section, Texas Department of State Health Services	Bonser; Bonser, J.; J.D.; Koag, M.; Lee; Oh, B.; Pedrueza, M.; Pokharel, A.; R. and Kubin, G.; Rahman, M.; Tuladhar, R.; Wang, C.; Zhang, J.
EPI_ISL_622767	Laboratory Services Section, Texas Department of State Health Services-SARS-CoV-2 submission group	Laboratory Services Section, Texas Department of State Health Services-SARS-CoV-2 submission group	G. and Pedrueza, M.; Koag, M.; Kubin; Lee, R.; Oh, B.; Pokharel, A.; Rahman, M.; Tuladhar, R.; Wang, C.; Zhang, J.
EPI_ISL_1017690	Laboratory for COVID19 diagnostics, Clinical Centre of Serbia	Institute of microbiology and Immunology, Faculty of Medicine, University of Belgrade	Jankovic, M.; Jovanovic, T.; Knezevic, A.; Milicevic, O.; Sekler, M.; Tesovic, B.; Vidanovic, D.
EPI_ISL_933533, EPI_ISL_1138899, EPI_ISL_1138969, EPI_ISL_1138983, EPI_ISL_1209407	Laboratory for HIV and opportunistic infections diagnosis The Republican Research and Practical Center for Epidemiology and Microbiology (RRPCEM)	Laboratory for HIV and opportunistic infections diagnosis The Republican Research and Practical Center for Epidemiology and Microbiology (RRPCEM)	Analoly Krasko; Anatoly Krasko; Artur Akhremchuk; Elena Gasich; Kirill Bulda; Leonid Valentovich; Leonid Valentovich; Vladimir Gorbunov
EPI_ISL_445243, EPI_ISL_455468 to 455477, EPI_ISL_455479	Laboratory for Respiratory Viruses, Cantacuzino National Military-Medical Institute for Research and Development	Cantacuzino Institute	A.Cretu; L.Ustea; M.Lazar; T.Durfee; Tim Durfee
EPI_ISL_754238, EPI_ISL_861710 to 861713, EPI_ISL_888677 to 888678, EPI_ISL_910016, EPI_ISL_910029, EPI_ISL_910335 to 910339, EPI_ISL_940144, EPI_ISL_940554, EPI_ISL_981374 to 981375, EPI_ISL_981377, EPI_ISL_1224909, EPI_ISL_1224911 to 1224912, EPI_ISL_1228691 to 1228692, EPI_ISL_1228908 to 1228909, EPI_ISL_1295641, EPI_ISL_1295643 to 1295645			
see above	Laboratory for Respiratory Viruses, Cantacuzino National Military-Medical Institute for Research and Development	Cantacuzino Institute Virology	Catalina Pascu; Luiza Ustea; Mihaela Lazar; Mühlemann Barbara; Nicoleta Paraschiv; Nicoleta Parashiv; Tim Durfee
EPI_ISL_471416 to 471424	Laboratory for Respiratory Viruses, National Influenza Centre, Cantacuzino National Military-Medical Institute for Research and Development	Cantacuzino Institute	Luiza Ustea; Mihaela Lazar; Nicoleta Paraschiv; Tim Durfee

EPI_ISL_437689	Laboratory for Urgent Response to Biological Threats	Institut Pasteur CIBU / ERI	A. Kwasiborski; C. Balière; C. Batéjat; J. Vanhomwegen; JC. Manuguerra; V. Caro; V. Hourdel
EPI_ISL_437690	Laboratory for Urgent Response to Biological Threats	Institut Pasteur CIBU /ERI	A. Kwasiborski; C. Balière; C. Batéjat; H. Hourdel; J. Vanhomwegen; JC. Manuguerra; V. Caro
EPI_ISL_435045 to 435048	Laboratory of Applied Genetics	RSE "National Center for Biotechnology"	Alexandr Shevtsov; Asylulan Amirgazin; Ilyas Akhmetollayev; Ruslan Kalendar; Viktoriya Lutsay; Yerlan Ramanculov
EPI_ISL_572398	Laboratory of Biology and Identification of Arboviruses	Pathogenic Microorganisms Variability Laboratory	Alexander Gintsburg; Alexey Prilipov; Alexey Shchetinin; Alina Kozlova; Andrei Siniavin; Maria Nikiforova; Muhammad Saifullin; Victor Larichev; Vladimir Gushchin
EPI_ISL_1048805 to 1048813	Laboratory of Clinical Microbiology, Virology and Bioemergencies - Fatebenefratelli Sacco Hospital	Virology Laboratory, Scientific Department, Army Medical Center	Alberto Rizzo; Alessandro Mancon; Anella Monte; Anna Anselmo; Florigio Lista; Giovanni Faggioni; Maria Rita Gismondo; Riccardo De Sanctis; Silvia Fillo; Valeria Micheli; Vanessa Vera Fain
EPI_ISL_848070 to 848083	Laboratory of Clinical Research on Dermatozoonoses in Domestic Animals, Evandro Chagas National Institute of Infectious Diseases, Oswaldo Cruz Foundation (Fiocruz), Rio de Janeiro, RJ, Brazil.	Laboratory of Respiratory Viruses and Measles, Oswaldo Cruz Institute, FIOCRUZ	Alex Pauvolid-Corrêa; Alice Sampaio Barreto da Rocha; Anielle de Pina Costa; Artur Augusto Velho Mendes Junior; Ezequias Batista Martins; Fernando do Couto Motta; Guilherme Amaral Calvet; Isabella Campos Vargas de Moraes; Isabella Dib Ferreira Gremião; Lucas Oliveira Keidel; Lusiele Guaraldo; Maria Ogrzewalska; Marilda Mendonça Siqueira on behalf of the Fiocruz COVID-19 Genomic Surveillance Network; Michelle Fernanda Borges da Silva; PaolaCristina Resende; Patrícia Brasil; Renato Orsini Ornellas; Rodrigo Caldas Menezes; Sandro Antônio Pereira; Shanna Araujo dos Santos; Thiago C. Souza
EPI_ISL_1098895	Laboratory of Communicable Diseasea	Laboratory of Communicable Diseasea	Liidia Dotsenko
EPI_ISL_1138519 to 1138523, EPI_ISL_1138526 to 1138536, EPI_ISL_1138538 to 1138541, EPI_ISL_1138544 to 1138546, EPI_ISL_1138548 to 1138552, EPI_ISL_1138554 to 1138559, EPI_ISL_1138568 to 1138572, EPI_ISL_1190934 to 1190938, EPI_ISL_1190940 to 1190950, EPI_ISL_1190955, EPI_ISL_1259160 to 1259180			
see above	Laboratory of Communicable Diseases	1. Laboratory of Communicable Diseases (Estonia); 2. Eurofins Genomics Europe Sequencing GmbH	Liidia Dotsenko; Liidia Dotsenko et al.
EPI_ISL_1020323, EPI_ISL_1101253	Laboratory of Communicable Diseases	Laboratory of Communicable Diseases	Liidia Dotsenko
EPI_ISL_477163	Laboratory of Dr. John Lednicky	University of Florida	John A. Lednicky; John Glenn Morris, Jr.; Kuttichantran Subramaniam; Maha A. Elbadry; Thomas B. Waltzek
EPI_ISL_1281568 to 1281569, EPI_ISL_1281571, EPI_ISL_1281573	Laboratory of Extremely Dangerous Infections of Federal Budget Health Care Instute "Center of Hygiene and Epidemiology in Sakhalin region"	Group of Genomics and Postgenomic Technologies of Central Research Institute of Epidemiology	Akimkin VG; Arutiunian AE; Bulanenko VP; Byankina MA; Golubeva AG; Kaptelova VV; Kondrasheva LV; Korneenko EV; Saenko SS; Samoilov AE; Speranskaya AS; Tivanova EV; Valdokhina AV
EPI_ISL_735392 to 735395, EPI_ISL_760059, EPI_ISL_760246, EPI_ISL_1081837, EPI_ISL_1096335 to 1096341, EPI_ISL_1201427 to 1201436, EPI_ISL_1249449, EPI_ISL_1249452, EPI_ISL_1249455, EPI_ISL_1249457, EPI_ISL_1249460, EPI_ISL_1249463, EPI_ISL_1249467			
see above	Laboratory of Genetics and Personalized Medicine, Zan Mitrev Clinic	Laboratory of Genetics and Personalized Medicine, Zan Mitrev Clinic	2020; Kungilovski G. et al.; Kungulovski G. et al.; Kungulovski et al.; Kungulovski et al. 2020
EPI_ISL_435723	Laboratory of Genomics & Bioinformatics, Institute of Immunology and Experimental Therapy, Polish Academy of Sciences Oddzia Mikrobiologii Wojewódzkiej Stacji Sanitarno Epidemiologiczna	Laboratory of Genomics & Bioinformatics, Institute of Immunology and Experimental Therapy, Polish Academy of Sciences	Aleksandra Herud; Dariusz Martynowski; Dorota Kujawa; Grayna Zalewska; Joanna Sikorska; Krzysztof Jakub Pawlik; Oskar Karpiski and ukasz aczmaski; Paulina ebrowska
EPI_ISL_437625	Laboratory of Genomics & Bioinformatics, Institute of Immunology and Experimental Therapy, Polish Academy of Sciences Oddzial Mikrobiologii Wojewodzkiej Stacji Sanitarno-Epidemiologicznej.	Laboratory of Genomics & Bioinformatics, Institute of Immunology and Experimental Therapy, Polish Academy of Sciences	Aleksandra Herud; Dariusz Martynowski; Dorota Kujawa; Grazyna Zalewska; Joanna Sikorska; Krzysztof Jakub Pawlik; Oskar Karpinski and Lukasz Laczmanski; Paulina Zebrowska
EPI_ISL_856971	Laboratory of Histology-Embryology, Molecular Carcinogenesis Group Medical School National and Kapodistrian University of Athens	Prof. Gorgoulis Lab	Gorgoulis V.G.
EPI_ISL_1096992, EPI_ISL_1096999, EPI_ISL_1097001, EPI_ISL_1097003, EPI_ISL_1097006	Laboratory of Histology-Embryology, Molecular Carcinogenesis Group, Faculty of Medicine, National and Kapodistrian University of Athens	Laboratory of Histology-Embryology	Gorgoulis V.G.
EPI_ISL_536399	Laboratory of Immunovirology. Universidad de Antioquia	Instituto Nacional de Salud - Unidad de Secuenciación y Genómica	Carlos Franco-Muñoz; Diego Álvarez-Diaz and Marcela Mercado-Reyes; Francisco J. Díaz; Katherine Laiton-Donato; Lizdany Flórez; Wbeimar Aguilar-Jimenez
EPI_ISL_452327 to 452364	Laboratory of Infectious Diseases Center of Beijing Ditan Hospital	Laboratory of Infectious Diseases Center of Beijing Ditan Hospital	Chengjie Jie; Fengting Yu; Linghang Wang; Liting Yan; Siyuan Yang; Yunxia Tang
EPI_ISL_417445 to 417447, EPI_ISL_778860, EPI_ISL_779704, EPI_ISL_779707, EPI_ISL_779709, EPI_ISL_779712 to 779714, EPI_ISL_780375 to 780376, EPI_ISL_780378, EPI_ISL_780381, EPI_ISL_780411 to 780413, EPI_ISL_1085094, EPI_ISL_1085165, EPI_ISL_1085167, EPI_ISL_1085169 to 1085170, EPI_ISL_1085173 to 1085174, EPI_ISL_1085230			
see above	Laboratory of Infectious Diseases, Department of Biomedical and Clinical Sciences L. Sacco, University of Milan	Laboratory of Infectious Diseases, Department of Biomedical and Clinical Sciences L. Sacco, University of Milan	Agostino Riva; Alessia Lai; Annalisa Bergna; Arianna Gabrieli; Carla Della Ventura; Claudia Balotta; Dario Bernacchia; Gianguglielmo Zehender; Gianguglielmo Zehender on behalf of SARS-CoV-2 ITALIAN RESEARCH ENTERPRISE-(SCIRE) Collaborative Group; Gianguglielmo Zehender on behalf of SARS-CoV-2 ITALIAN RESEARCH ENTERPRISE-(SCIRE) Collaborative Group; Giuliano Rizzardini; Luca Meroni; Maciej Tarkowski; Massimo Galli; Spinello Antinori; Stefano Rusconi
EPI_ISL_717979	Laboratory of Microbiology and Infectious Diseases, Faculty of Veterinary Medicine, Aristotle University of Thessaloniki, University Campus, 541 24, Thessaloniki, Greece.	Laboratory of Biology, Department of Medicine, Democritus University of Thrace, Alexandroupolis, Greece	Bampali M.; Chaintoutis S.; Dimitriou M.; Dovas C.; Dovrolis N.; Karakasiliotis I.
EPI_ISL_869240	Laboratory of Microbiology, ASST Settelaghi, Varese, Italy	Laboratory of Microbiology, ASST Settelaghi, Varese, Italy	Azzi, L.; Baj, A.; Capuano, R.; Cassani, G.; Catanoso, G.; Colombo, A.; Donadini, A.; Focosi, D.; Genoni, A.; Maggi, F.; Novazzi, F.; P.G.; Pasciuta, R.; Prestia, M.; Rossi, A.; Spezia; Tamborini, A.; Zago, C.
EPI_ISL_418263 to 418265	Laboratory of Microbiology, Department of Medicine, National and Kapodistrian University of Athens, Greece	Laboratory of Biology, Department of Medicine, Democritus University of Thrace, Greece	Elisavet Gatzidou; Ioannis Karakasiliotis; Maria Bampali; Nikolaos Dovrolis; Nikolaos Spanakis; Stavroula Veletza
EPI_ISL_427043, EPI_ISL_434455 to 434486, EPI_ISL_437874 to 437911	Laboratory of Microbiology, Medical School, National and Kapodistrian University of Athens	Laboratory of Biology, Department of Medicine, Democritus University of Thrace	Bampali, M.; Dovrolis, N.; Froukala, E.; Gatzidou, E.; Kassela K.; N. and Karakasiliotis, I.; Spanakis; Stavropoulou, A.; Tsakris, A.; Veletza, S.
EPI_ISL_654016 to 654020	Laboratory of Microbiology, National Reference Lab, Charles Nicolle Hospital; 2-University of Tunis ElManar, Faculty of Medicine of Tunis, LR99ES09, Tunis, Tunisia	1-Clinical and Experimental Pharmacology Lab, LR16SP02, National Center of Pharmacovigilance, University of Tunis El Manar, Tunis, Tunisia. 2-Neurodegenerative diseases and psychiatric troubles, LR18SP03, Razi Hospital, University of Tunis El Manar, Tunis, Tunisia. 3- Ministry of Health, National Observatory of New and Emerging Diseases, 1006, Tunis, Tunisia	Alia Ben Kahla; Gaies Emna; Ilhem Boutiba-Ben Boubaker; Imen Kacem; Imen Mkada; Jalila Ben Khelil; Maher Kharrat; Mouna Ben Sassi; Mouna Safer; Nissaf Ben Alaya; Riadh Daghfous; Riadh Gouider.; Salma Abid; Sameh Trabelsi; Sana Ferjani; Soumaya Rammeh
EPI_ISL_796782, EPI_ISL_803119 to 803120, EPI_ISL_803430, EPI_ISL_803817, EPI_ISL_803851, EPI_ISL_811145	Laboratory of Microbiology, National Reference Lab, Charles Nicolle Hospital; 2-University of Tunis ElManar, Faculty of Medicine of Tunis, LR99ES09, Tunis, Tunisia	Clinical and Experimental Pharmacology Lab, LR16SP02, National Center of Pharmacovigilance, University of Tunis El Manar, Tunis, Tunisia. 2-Neurodegenerative diseases and psychiatric troubles, LR18SP03, Razi Hospital, University of Tunis El Manar, Tunis, Tunisia. 3- Ministry of Health, National Observatory of New and Emerging Diseases, 1006, Tunis, Tunisia	Ahmed Fakhfakh; Alia Ben Kahla; Alia BenKahla; Asma Ferjani; Awatef El Moussi; Gaies Emna; Guedi Ali Barreh; Habiba Ben Romdhane; Hanen El Jebari; Ilhem Boutiba-Ben Boubaker; Ines Mdini; Jalila Ben Khelil; Maher Kharrat; Mouna Ben Sassi; Mouna Safer; Nissaf Ben Alaya; Riadh Daghfous; Riadh Gouider.; Salma Abid; Sameh Trabelsi; Sana Ferjani; Sarra Chamman; Souissi Amira; Zaineb Hamzaoui
EPI_ISL_869166	Laboratory of Microbiology,ASST Settelaghi, Varese, Italy	Laboratory of Microbiology,ASST Settelaghi, Varese, Italy	Azzi, L.; Baj, A.; Capuano, R.; Cassani, G.; Catanoso, G.; Colombo, A.; Donadini, A.; Focosi, D.; Genoni, A.; Grossi, P.; L. and Bonelli, G.; Maffioli; Maggi, F.; Novazzi, F.; P.G.; Pasciuta, R.; Prestia, M.; Rossi, A.; Spezia; Tamborini, A.; Zago, C.

EPI_ISL_613563 to 613564, EPI_ISL_613707 to 613708	Laboratory of Molecular Biology, Blood Center of Ribeirão Preto	Laboratory of Molecular Biology, Blood Center of Ribeirão Preto, Faculty of Medicine of Ribeirão Preto, University of São Paulo	Aparecida Y Yamamoto; Diego Villa Clé; Dimas T Covas; Elaine V Santos; Evandra S Rodrigues; Glauco de Carvalho Pereira; Joilson Xavier; Luiz CJ Alcantara; Marta Giovanetti; Rodrigo T Calado; Simone Kashima; Svetoslav N Slavov; Talita Adelino; Vagner Fonseca
EPI_ISL_613709, EPI_ISL_613711, EPI_ISL_613951 to 613952, EPI_ISL_613954, EPI_ISL_613956, EPI_ISL_613961 to 613965, EPI_ISL_614011, EPI_ISL_614155 to 614156			
see above	Laboratory of Molecular Biology, Blood Center of Ribeirão Preto, Faculty of Medicine of Ribeirão Preto, University of São Paulo	Laboratory of Molecular Biology, Blood Center of Ribeirão Preto, Faculty of Medicine of Ribeirão Preto, University of São Paulo	Aparecida Y Yamamoto; Diego Villa Clé; Dimas T Covas; Elaine V Santos; Evandra S Rodrigues; Glauco de Carvalho Pereira; Joilson Xavier; Luiz CJ Alcantara; Marta Giovanetti; Rodrigo T Calado; Simone Kashima; Svetoslav N Slavov; Talita Adelino; Vagner Fonseca
EPI_ISL_428209, EPI_ISL_450746, EPI_ISL_451644 to 451647, EPI_ISL_906713, EPI_ISL_906720, EPI_ISL_906727, EPI_ISL_906730, EPI_ISL_906736, EPI_ISL_906740, EPI_ISL_906743, EPI_ISL_906755, EPI_ISL_906757 to 906759, EPI_ISL_930785			
see above	Laboratory of Molecular Biology, Diagnostyka sp. z o.o.	Laboratory of Recombinant Vaccines	Anna Piotrowska-Mietelska; Boguslaw Szewczyk; Izabela Szczygielska; Krystyna Bienkowska-Szewczyk; Lukasz Rabalski; Maciej Kosinski
EPI_ISL_852842, EPI_ISL_875959 to 876031, EPI_ISL_925033 to 925047, EPI_ISL_935033, EPI_ISL_935035, EPI_ISL_1048374, EPI_ISL_1181174 to 1181175, EPI_ISL_1250943 to 1250944			
see above	Laboratory of Molecular Biology, Diagnostyka sp. z o.o.	genXone SA, Research & Development Laboratory	Aleksandra Gidlewicz; Anna Brylak-Blaszkow; Grzegorz Nowicki; Jakub Grabowski; Karol Szeszko; Lukasz Krych; Maciej Sykulski; Michal Kaszuba; Micha Kaszuba; Monika Makowska-Woniak; Natalia Drweska-Matelska; Natalia Drwska-Matelska; ukasz Krych
EPI_ISL_425228 to 425229, EPI_ISL_426051, EPI_ISL_426356 to 426360	Laboratory of Molecular Genetics, 2nd Faculty of Medicine, Charles University in Prague, Prague, Czech Republic	Laboratory of Molecular Genetics, 2nd Faculty of Medicine, Charles University in Prague, Prague, Czech Republic	Katerina Polackova; Kateina Poláková; Lenka Kramna; Lenka Kramná; Ondrej Cinek; Ondej Cinek
EPI_ISL_625673 to 625682	Laboratory of Molecular Medicine, University of Magallanes	Centro Asistencial Docente y de Investigacion, Universidad de Magallanes	Diego Alvarez; Jacqueline Aldridge; Jorge Gonzalez; Marcelo Navarrete
EPI_ISL_428853, EPI_ISL_498559 to 498563	Laboratory of Molecular Virology International Center for Genetic Engineering and Biotechnology (ICGEB)	ARGO Open Lab Platform for Genome Sequencing	D'Agaro P; Dal Monego S; D'Agaro P; Licastro D; Marcello A; Rajasekharan S; Segat L
EPI_ISL_417419, EPI_ISL_417421, EPI_ISL_417423, EPI_ISL_428854	Laboratory of Molecular Virology International Center for Genetic Engineering and Biotechnology (ICGEB)	ARGO Open Lab Platform for Genome sequencing	D'Agaro P; Dal Monego S; Licastro D; Marcello A; Rajasekharan; Rajasekharan S; Segat L
EPI_ISL_417418	Laboratory of Molecular Virology International Center fro Genetic Engineering and Biotechnology (ICGEB)	ARGO Open Lab Platform for Genome sequencing	D'Agaro P; Dal Monego S; Licastro D; Marcello A; Rajasekharan S; Segat L
EPI_ISL_479616 to 479619, EPI_ISL_479790 to 479791, EPI_ISL_525495 to 525496	Laboratory of Molecular Virology of the International Centre for Genetic Engineering and Biotechnology (ICGEB)	ARGO Open Lab Platform for Genome Sequencing	Confalonieri M; Confalonieri M Marcello A; Confalonieri P; D; D'Agaro P; Dal Monego S; Licastro; Licastro D; Marcello A; Rajasekharan S; Salton F; Segat L
EPI_ISL_584048 to 584049, EPI_ISL_584051 to 584052	Laboratory of Molecular Virology, Department of Biomedical, Surgical and Dental Sciences University of Milano	Laboratory of Molecular Virology, Department of Biomedical, Surgical and Dental Sciences University of Milano	Anselmi; B. and Ferrante, P.; Basilico, N.; Bianchi, M.; Binda, S.; C. and Ferrante, P.; D'Alessandro, S.; Delbue, S.; Dolci, M.; E; Fattori, M.; Ferrante, P.; G. and Pariani; Galli; Galli, C.; Incorvaia; Modenese, A.; Parapini, S.; Pariani, E.; Primache, V.; Signorini, L.; Taramelli, D.
EPI_ISL_415658, EPI_ISL_415660 to 415661, EPI_ISL_801539 to 801860	Laboratory of Molecular Virology, Pontificia Universidad Católica de Chile	MSHS Pathogen Surveillance Program	Adolfo Garcia-Sastre; Adriana van De Guchte; Ajay Obla; Aldo Gaggero; Ana Maria Contreras; Ana S. Gonzalez-Reiche; Ana Silvia Gonzalez-Reiche; Bremy Alburquerque; Carlos Palma; Constanza Maldonado; Edward C. Holmes; Eileen Serrano; Erick Salinas; Fernando Valiente; Hala Alshammary; Harm van Bakel; Jayeeta Dutta; Jorge Levican; Juan Soto; Leonardo I. Almonacid; M. Belen Leyton; Manuel Ampuero; Marcela Ferres; Matthew Hernandez; Matthew M. Hernandez; Melissa Smith; Mitchell Sullivan; Pablo Vial; Rafael A. Medina; Rafael A. Medina.; Robert Sebra; Shwetha Hara Sridhar; Shwetha Sridhar Hara; Tamara Garcia; Tamara García-Salum; Viviana Simon; Ying-Chih Wang; Zenab Khan
EPI_ISL_1233875 to 1233876	Laboratory of Parasitic Diseases, Systems Genomics Section, National Institute of Allergy and Infectious Diseases, Nathional Institutes of Health	Laboratory of Parasitic Diseases, Systems Genomics Section, National Institute of Allergy and Infectious Diseases, Nathional Institutes of Health	Bailin, H.; Banakis, S.; Bell, M.; Chung, M.; Das, S.; Ghedin, E.; J.-h.; Kwan, J.; McCormick-Ell, J.; Mercado, R.; Palmore, T.; Roder, A.; Wang, W.; Youn
EPI_ISL_427294 to 427304, EPI_ISL_456071 to 456075, EPI_ISL_456079 to 456081, EPI_ISL_456084 to 456087, EPI_ISL_456089 to 456106, EPI_ISL_467344 to 467371, EPI_ISL_541347 to 541369, EPI_ISL_1181357 to 1181361, EPI_ISL_1181363, EPI_ISL_1181366 to 1181367, EPI_ISL_1181369, EPI_ISL_1181373 to 1181377, EPI_ISL_1181381 to 1181389, EPI_ISL_1181391, EPI_ISL_1181393, EPI_ISL_1181398, EPI_ISL_1181402, EPI_ISL_1181410, EPI_ISL_1181415, EPI_ISL_1181430, EPI_ISL_1181437 to 1181439, EPI_ISL_1181442, EPI_ISL_1181446 to 1181448, EPI_ISL_1181454 to 1181467, EPI_ISL_1181470, EPI_ISL_1181475 to 1181477, EPI_ISL_1181481 to 1181483, EPI_ISL_1181486, EPI_ISL_1181488 to 1181491, EPI_ISL_1181493 to 1181497, EPI_ISL_1181499 to 1181515, EPI_ISL_1181518 to 1181519, EPI_ISL_1181521, EPI_ISL_1181523, EPI_ISL_1181529, EPI_ISL_1181531, EPI_ISL_1181560 to 1181562, EPI_ISL_1181571 to 1181574, EPI_ISL_1181578 to 1181579, EPI_ISL_1181587, EPI_ISL_1181589, EPI_ISL_1181594, EPI_ISL_1181597, EPI_ISL_1181599, EPI_ISL_1181608			
see above	Laboratory of Respiratory Viruses and Measles, Oswaldo Cruz Institute, FIOCRUZ	Laboratory of Respiratory Viruses and Measles, Oswaldo Cruz Institute, FIOCRUZ	Alice Sampaio Rocha; Aline Mattos; Ana Carolina Mendonca; Ana Carolina Mendonça; Anna Carolina Paixao; Anna Carolina Paixão; Braulia Caetano; Cinthia Avila; Cristiana Garcia; Fernando Motta; Jonathan Lopes; Luciana Appolinario; Maria Ogrzewalska; Marilda Siqueira on behalf of the Fiocruz COVID-19 Genomic Surveillance Network; Milene Miranda; Paola Resende; Priscila Born; Renata Serrano Lopes; Roxana Loayza; Sunando Roy
EPI_ISL_939628 to 939629, EPI_ISL_939643 to 939644, EPI_ISL_939649 to 939650, EPI_ISL_940146, EPI_ISL_940157, EPI_ISL_940766, EPI_ISL_941163, EPI_ISL_944787, EPI_ISL_945547, EPI_ISL_946913, EPI_ISL_949176 to 949178, EPI_ISL_949180 to 949181, EPI_ISL_953426, EPI_ISL_954943, EPI_ISL_956413, EPI_ISL_956416, EPI_ISL_956420, EPI_ISL_959287, EPI_ISL_959290, EPI_ISL_959375 to 959376, EPI_ISL_959544, EPI_ISL_959546, EPI_ISL_962887, EPI_ISL_962889 to 962890, EPI_ISL_962893 to 962894, EPI_ISL_964881, EPI_ISL_964883, EPI_ISL_964950, EPI_ISL_965142, EPI_ISL_965145, EPI_ISL_965147			
see above	Laboratory of Virology and Molecular Diagnostics	Institute of Public Health of Republic of North Macedonia Laboratory of Virology and Molecular Diagnostics	Boshevska G.; Elizabeta Janchevska; G. Boshevska; Golubinka Bosehvska; Golubinka Boshevska; Kuzmanovska M.; M. Kuzmanovska; Maja Kuzmanovska; Maja Kuzmanovska. Golubinka Boshevska
EPI_ISL_962891, EPI_ISL_1198644	Laboratory of Virology and Molecular Diagnostics	Laboratory of Virology and Molecular Diagnostics, Institute of Public Health of Republic of North Macedonia	Boshevska G.; Boshevska Golubinka; Janchevska Elizabeta; Kuzmanovska M.; Kuzmanovska Maja
EPI_ISL_984682, EPI_ISL_1082298 to 1082319	Laboratory of Virology of Federal Budget Health Care Institution "Center of Hygiene and Epidemiology in Arkhangelsk region"	Group of Genomics and Postgenomic Technologies of Central Research Institute of Epidemiology	Akimkin VG; Bulanenko VP; Cherkashina AS; Golubeva AG; Kaptelova VV; Kondrasheva LV; Korneenko EV; Ponomareva YA; Saenko SS; Samoilov AE; Semyina LV; Shishko LA; Speranskaya AS; Tivanova EV; Valdokhina AV
EPI_ISL_984628 to 984658	Laboratory of Virology of Federal Budget Health Care Institution Center of Hygiene and Epidemiology in Arkhangelsk region	Group of Genomics and Postgenomic Technologies of Central Research Institute of Epidemiology	Akimkin VG; Bulanenko VP; Kaptelova VV; Kondrasheva LV; Korneenko EV; Ponomareva YA; Saenko SS; Samoilov AE; Semyina LV; Shishko LA; Speranskaya AS; Tivanova EV; Valdokhina AV
EPI_ISL_451298 to 451305	Laboratory of Virology, INMI Lazzaro Spallanzani IRCCS	Laboratory of Virology, INMI Lazzaro Spallanzani IRCCS	Antonino Di Caro; Barbara Bartolini; Cesare E.M. Gruber; Francesco Messina; Giuseppe Ippolito; Maria R. Capobianchi; Martina Rueca
EPI_ISL_1278274, EPI_ISL_1289956 to 1289960	Laboratory of Virology, Ribeirão Preto General Hospital, Ribeirão Preto Medical School, University of São Paulo	Laboratory of Oncology, Blood Center of Ribeirão Preto, Ribeirão Preto School of Medicine, University of São Paulo	A.L.P.; A.Y.; B.A.L.; BELLISSIMO-RODRIGUES, F.; CAMPOS; COLLI; FONSECA; L.M.; MR; SANTOS; YAMAMOTO
EPI_ISL_1165139 to 1165141, EPI_ISL_1180150, EPI_ISL_1180152 to 1180159, EPI_ISL_1191740 to 1191741, EPI_ISL_1191747, EPI_ISL_1191754 to 1191769, EPI_ISL_1191774 to 1191780			
see above	Laboratory of virology and molecular diagnostics	Laboratory of virology and molecular diagnostics	Boshevska Golubinka; Elizabeta Janchevska; Janchevska Elizabeta; Kuzmanovska Maja
EPI_ISL_454575	Laboratory of virology, National Center of Expertise	Laboratory of molecular-genetic research, National Center for Expertise, Kazakhstan National Center for Biotechnology, Kazakhstan	; Abdaliyev Askar; Akhmetollayev Ilyas; Amirgazin Asylulan; Aushakhmetova Zabira; Kalendar Ruslan; Lutsay Viktoriya; Rakhmetova Akbota; Ramankulov Yerlan; Shevtsov Alexandr
EPI_ISL_454584 to 454586, EPI_ISL_454589	Laboratory of virology, National Center of Expertise	Laboratory of molecular-genetic research, National Center for Expertise, Kazakhstan National Center for Biotechnology, Kazakhstan	; Abdaliyev Askar; Akhmetollayev Ilyas; Amirgazin Asylulan; Aushakhmetova Zabira; Kalendar Ruslan; Lutsay Viktoriya; Rakhmetova Akbota; Ramankulov Yerlan; Shevtsov Alexandr
EPI_ISL_454596 to 454597, EPI_ISL_454599 to 454600, EPI_ISL_454604	Laboratory of virology, National Center of Expertise	Laboratory of molecular-genetic research, National Center for Expertise, Kazakhstan National Center for Biotechnology, Kazakhstan	; Abdaliyev Askar; Akhmetollayev Ilyas; Amirgazin Asylulan; Aushakhmetova Zabira; Kalendar Ruslan; Lutsay Viktoriya; Rakhmetova Akbota; Ramankulov Yerlan; Shevtsov Alexandr
EPI_ISL_454576 to 454577, EPI_ISL_454579 to 454580, EPI_ISL_454582, EPI_ISL_454587, EPI_ISL_454590 to 454591, EPI_ISL_454593 to 454594, EPI_ISL_454598, EPI_ISL_454601, EPI_ISL_454603			
see above	Laboratory of virology, National Center of Expertise	Laboratory of molecular-genetic research, National Center of Expertise, Kazakhstan National Center for Biotechnology, Kazakhstan	; Abdaliyev Askar; Akhmetollayev Ilyas; Amirgazin Asylulan; Aushakhmetova Zabira; Kalendar Ruslan; Lutsay Viktoriya; Rakhmetova Akbota; Ramankulov Yerlan; Shevtsov Alexandr
EPI_ISL_576119	Laboratory, The Bio Arte Limited	Laboratory, The Bio Arte Limited	Biazzo, M.; Madeddu, S.; Pinzauti, D.; Santoro, F.
EPI_ISL_576117 to 576118, EPI_ISL_576120 to	Laboratory, The Bio Arte Limited	Laboratory, The Bio Arte Limited	Biazzo, M.; Madeddu, S.; Pinzauti, D.; Santoro, F.

576126			
EPI_ISL_1239111 to 1239113	Laboratório Central de Saúde Pública Noel Nutels	Coordenação Geral de Laboratórios de Saúde Pública (CGLAB)	; Vagner Fonseca et al
EPI_ISL_414045, EPI_ISL_456076 to 456077, EPI_ISL_456082 to 456083, EPI_ISL_456088	Laboratório Central de Saúde Pública Noel Nutels (LACEN-RJ)	Laboratory of Respiratory Viruses and Measles, Oswaldo Cruz Institute, FIOCRUZ	Aline Mattos; Alisson Fabri; Braulia Caetano; Cristiana Garcia; Fernando Motta; Joilson Xavier; Jonathan Lopes; Luciana Appolinario; Maria Nóbrega; Maria Ogrzewalska; Marilda Siqueira on behalf of the Fiocruz COVID-19 Genomic Surveillance Network; Marilda SiqueiraFiocruz COVID-19 Genomic Surveillance Network; Milene Miranda; Paola Resende; Sunando Roy
EPI_ISL_415105, EPI_ISL_427293	Laboratório Central de Saúde Pública Professor Gonçalo Moniz (LACEN-BA)	Laboratory of Respiratory Viruses and Measles, Oswaldo Cruz Institute, FIOCRUZ	Aline Mattos; Allison Fabri; Braulia Caetano; Cristiana Garcia; Fernando Motta; Joilson Xavier; Jonathan Lopes; Luciana Appolinario; Maria Nóbrega; Maria Ogrzewalska; Marilda Siqueira Fiocruz COVID-19 Genomic Surveillance Network; Marilda Siqueira on behalf of the Fiocruz COVID-19 Genomic Surveillance Network; Milene Miranda; Paola Resende; Priscila Born; Sunando Roy
EPI_ISL_1239114 to 1239123, EPI_ISL_1239125 to 1239136, EPI_ISL_1240639 to 1240641	Laboratório Central de Saúde Pública do Espírito Santo	Coordenação Geral de Laboratórios de Saúde Pública (CGLAB)	; Vagner Fonseca et al; Vagner Fonseca et al.
EPI_ISL_792561 to 792638	Laboratório Central de Saúde Pública do Estado da Paraíba (LACEN-PB)	Laboratory of Respiratory Viruses and Measles, Oswaldo Cruz Institute, FIOCRUZ	Ana Carolina Mendonca; Anna Carolina Paixao; Dalane Loudal Florentino Teixeira; Fernando Motta; João Felipe Bezerra; Luciana Appolinario; Marilda Siqueira on behalf of the Fiocruz COVID-19 Genomic Surveillance Network; Paola Resende; Romero Henrique Teixeira de Vasconcelos; Thiago Franco de Oliveira Carneiro
EPI_ISL_427292, EPI_ISL_792639 to 792644	Laboratório Central de Saúde Pública do Estado de Alagoas (LACEN-AL)	Laboratory of Respiratory Viruses and Measles, Oswaldo Cruz Institute, FIOCRUZ	Aline Mattos; Ana Carolina Mendonca; Anderson Brandao Leite; Anna Carolina Paixao; Braulia Caetano; Cristiana Garcia; Fernando Motta; Jonathan Lopes; Luciana Appolinario; Maria Ogrzewalska; Marilda Siqueira on behalf of the Fiocruz COVID-19 Genomic Surveillance Network; Milene Miranda; Paola Resende; Priscila Born; Sunando Roy
EPI_ISL_500460 to 500486, EPI_ISL_500865 to 500875, EPI_ISL_572334 to 572396, EPI_ISL_1239012 to 1239016	Laboratório Central de Saúde Pública do Estado de Pernambuco (LACEN-PE)	WallauLab, Aggeu Magalhaes Institute	Alexandre Freitas da Silva; Antonio Mauro Rezende; Armando de Menezes Neto; Bruna Santos Lima Figueiredo de Sá; Caroline Targino Alves da Silva; Claudio Eduardo Cavalcanti; Constância Flávia Junqueira Ayres; Cássia Docena; Derciliano Lopes da Cruz; Duschinka Ribeiro Duarte Guedes; Elisama Helvecio; Filipe Zimmer Dezordi; Gabriel Luz Wallau on behalf of the Fiocruz COVID-19 Genomic Surveillance Network; Gonzalo Bello; Kamila Gaudêncio da Silva Sales; Larissa Krokovsky; Laís Ceschini Machado; Luciane Caroline Albuquerque Bezerra; Luydson Richardson Silva Vasconcelos; Marcelo Henrique Santos Paiva; Marcelo Henrique dos Santos Paiva; Maria Almerice Lopes da Silva; Matheus Filgueira Bezerra; Michelle da Silva Barros; Paola Cristina Resende; Renata Pessôa Germano Mendes; Rodrigo Moraes Loyo Arcoverde; Severino Jefferson Ribeiro da Silva; Sinval Pinto Brandão Filho; Tiago Gräf; Wheverton Ricardo Correia do Nascimento
EPI_ISL_427305 to 427306, EPI_ISL_541370 to 541371	Laboratório Central de Saúde Pública do Estado de Santa Catarina (LACEN-SC)	Laboratory of Respiratory Viruses and Measles, Oswaldo Cruz Institute, FIOCRUZ	Aline Mattos; Ana Carolina Mendonça; Anna Carolina Paixão; Braulia Caetano; Cristiana Garcia; Fernando Motta; Jonathan Lopes; Luciana Appolinario; Maria Ogrzewalska; Marilda Siqueira on behalf of the Fiocruz COVID-19 Genomic Surveillance Network; Milene Miranda; Paola Resende; Priscila Born; Sandra Bianchini; Sunando Roy
EPI_ISL_541372 to 541396	Laboratório Central de Saúde Pública do Estado de Sergipe (LACEN-SE)	Laboratory of Respiratory Viruses and Measles, Oswaldo Cruz Institute, FIOCRUZ	Ana Carolina Mendonça; Anna Carolina Paixão; Clioma Santos; Fernando Motta; Jonathan Lopes; Luciana Appolinario; Marilda Siqueira on behalf of the Fiocruz COVID-19 Genomic Surveillance Network; Paola Resende
EPI_ISL_801397 to 801403	Laboratório Central de Saúde Pública do Estado do Amazonas (LACEN-AM)	Laboratorio de Ecologia de Doencas Transmissiveis na Amazonia, Instituto Leonidas e Maria Deane - Fiocruz Amazonia	André Corado; Debora Duarte; Felipe Naveca on behalf of the Fiocruz COVID-19 Genomic Surveillance Network; Fernanda Nascimento; George Silva; Luciana Gonçalves; Maria Júlia Brandão; Michele Jesus; Valdinete Nascimento; Victor Souza; Ágatha Costa
EPI_ISL_415128	Laboratório Central de Saúde Pública do Estado do Espírito Santo (LACEN-ES)	Laboratory of Respiratory Viruses and Measles, Oswaldo Cruz Institute, FIOCRUZ	Aline Mattos; Allison Fabri; Braulia Caetano; Cristiana Garcia; Fernando Motta; Joilson Xavier; Jonathan Lopes; Luciana Appolinario; Maria Nóbrega; Maria Ogrzewalska; Marilda Siqueira on behalf of the Fiocruz COVID-19 Genomic Surveillance Network; Milene Miranda; Paola Resende; Sunando Roy
EPI_ISL_541340 to 541346, EPI_ISL_792645 to 792654	Laboratório Central de Saúde Pública do Estado do Paraná (LACEN-PR)	Laboratory of Respiratory Viruses and Measles, Oswaldo Cruz Institute, FIOCRUZ	Ana Carolina Mendonca; Ana Carolina Mendonça; Anna Carolina Paixao; Anna Carolina Paixão; Fernando Motta; Irina Nastassja Riediger; Irina Riediger; Jonathan Lopes; Luciana Appolinario; Maria do Carmo Debur; Marilda Siqueira on behalf of the Fiocruz COVID-19 Genomic Surveillance Network; Paola Resende
EPI_ISL_729794 to 729861	Laboratório Central de Saúde Pública do Estado do Rio Grande do Sul (LACEN-RS)	Laboratory of Respiratory Viruses and Measles, Oswaldo Cruz Institute, FIOCRUZ	Ana Carolina Mendonça; Anna Carolina Paixão; Fernando Motta; Luciana Appolinario; Marilda Siqueira on behalf of the Fiocruz COVID-19 Genomic Surveillance Network; Marilda Tereza Mar da Rosa; Paola Resende; Tatiana Schaffer Gregianini
EPI_ISL_1182547, EPI_ISL_1182597, EPI_ISL_1182599, EPI_ISL_1182605 to 1182606, EPI_ISL_1182608, EPI_ISL_1182610, EPI_ISL_1182616, EPI_ISL_1182619 to 1182621, EPI_ISL_1182623			
see above	Laboratório Central de Saúde Pública do Rio Grande do Sul	Coordenação Geral de Laboratórios de Saúde Pública (CGLAB/DAEVS/SVS/MS)	Vagner Fonseca; et al.
EPI_ISL_1182542, EPI_ISL_1182546, EPI_ISL_1182562 to 1182563, EPI_ISL_1182565, EPI_ISL_1182567 to 1182568, EPI_ISL_1182571 to 1182572, EPI_ISL_1182575 to 1182576, EPI_ISL_1182580 to 1182584, EPI_ISL_1182588 to 1182589, EPI_ISL_1182592, EPI_ISL_1182594 to 1182596, EPI_ISL_1182603 to 1182604, EPI_ISL_1182613 to 1182614, EPI_ISL_1182617, EPI_ISL_1182622, EPI_ISL_1182624			
see above	Laboratório Central do Estado do Paraná	Coordenação Geral de Laboratórios de Saúde Pública (CGLAB/DAEVS/SVS/MS)	Vagner Fonseca; et al.
EPI_ISL_1182552 to 1182554, EPI_ISL_1182556 to 1182558	Laboratório Central do Estado do Rio de Janeiro	Coordenação Geral de Laboratórios de Saúde Pública (CGLAB/DAEVS/SVS/MS)	Vagner Fonseca; et al.
EPI_ISL_1213148 to 1213149, EPI_ISL_1213151, EPI_ISL_1213155, EPI_ISL_1213158, EPI_ISL_1213163, EPI_ISL_1213171, EPI_ISL_1213248 to 1213249, EPI_ISL_1213270, EPI_ISL_1213272, EPI_ISL_1213307, EPI_ISL_1213309 to 1213310, EPI_ISL_1213312, EPI_ISL_1213314, EPI_ISL_1213339 to 1213340, EPI_ISL_1213372, EPI_ISL_1213374, EPI_ISL_1213376, EPI_ISL_1213378 to 1213379, EPI_ISL_1213381 to 1213382, EPI_ISL_1213393, EPI_ISL_1213395, EPI_ISL_1213397, EPI_ISL_1213408, EPI_ISL_1213410, EPI_ISL_1213417 to 1213418, EPI_ISL_1213420, EPI_ISL_1213422, EPI_ISL_1213424 to 1213425			
see above	Laboratório HLA/UERJ	Bioinformatics Laboratory / LNCC	Alessandra P Lamarca; Alexandra L Gerber; Ana Paula Melo Mariano; Ana Paula de C Guimarães; Ana Tereza R Vasconcelos; Angela Maria Guimarães Santos; Bianca Mendes Maciel; Danielle Angst Secco; Eduardo Sérgio Soares Sousa; Eloiza Helena Campana; Francisco Paulo Freire Neto; George Rego Albuquerque; Kátia Castanho Scortecci; Lucymara Fassarella Agnez Lima; Luiz G P de Almeida; Luís Cristóvão Porto; Otavio J. Brustolini; Paulo Ricardo Nascimento; Ronaldo da Silva Francisco Jr; Sandra Rocha Gadelha; Selma Maria Bezerra Jeronimo; Vinícius Pietta Perez
EPI_ISL_417943	Laboratório Hermes Pardini	Bioinformatics Laboratory	Alexandra Gerber; Amilcar Tanuri; Ana Paula Guimarães; Cristiano Xavier Lima; Cássia Alves Gonçalves; Diana Mariani; Ester Cerdeira Sabino; Filipe Romero; Ingra Morales Claro; Isabela de Carvalho Leitão; Jaqueline Goes de Jesus; Laboratorio Hermes Pardini; Laboratorio Simile; Luciana Jesus Costa e André Felipe Santos; Luiz Gonzaga Paula de Almeida; Lídia Boullosa; Mariane Talon; Mauro Teixeira; Nuno Rodrigues Faria; Orlando Ferreira Jr.; Pinto Castiñeiras; Rafael de Mello Galliez; Renato Santana Aguiar e Ana Tereza Vasconcelos; Richard Araújo Maia; Rodrigo Brindeiro; Ronaldo da Silva Francisco Junior; Terezinha Marta Pereira; Érica Ramos dos Santos Nascimento
EPI_ISL_417928 to 417930, EPI_ISL_417934, EPI_ISL_417936, EPI_ISL_417940, EPI_ISL_417945, EPI_ISL_417949	Laboratório Hermes Pardini	Bioinformatics Laboratory - LNCC	Alexandra Gerber; Amilcar Tanuri; Ana Paula Guimarães; Cristiano Xavier Lima; Cássia Alves Gonçalves; Cássia Cristina Alves Gonçalves; Diana Mariani; Ester Cerdeira Sabino; Filipe Romero; Ingra Morales Claro; Isabela de Carvalho Leitão; Jaqueline Goes de Jesus; Laboratorio Hermes Pardini; Laboratorio Simile; Luciana Jesus Costa e André Felipe Santos; Luiz Gonzaga Paula de Almeida; Lídia Boullosa; Mariane Talon; Mauro Teixeira; Nuno Rodrigues Faria; Orlando Ferreira Jr.; Pinto Castiñeiras; Rafael de Mello Galliez; Renato Santana Aguiar e Ana Tereza Vasconcelos; Richard Araújo Maia; Rodrigo Brindeiro; Ronaldo da Silva Francisco Junior; Terezinha Marta Pereira; Érica Ramos dos Santos Nascimento
EPI_ISL_1133255 to 1133279	Laboratório Hermes Pardini	Laboratório de Biologia Integrativa, Instituto de Ciências Biológicas, Universidade Federal de Minas Gerais	Alessandro Clayton de Souza Ferreira; Aline Brito de Lima; Carolina Moreira Voloch; Daniel Costa Queiroz; Danielle Alves Gomes Zauli; Diego Menezes Bonfim; Filipe Romero Rebello Moreira; Frederico Scott Varella Malta; Joice do Prado Silva; Rafael Marques de Souza; Renan Pedra de Souza; Renato Santana Aguiar.; Rennan Garcias Moreira; Victor Cavalcanti Pardini; Victor Emmanuel Viana Geddes; Wagner Carlos Santos Magalhaes; Walyson Coelho Costa
EPI_ISL_693220, EPI_ISL_693223 to 693224, EPI_ISL_693243	Laboratório Municipal de Piracicaba	Instituto Adolfo Lutz, Interdiciplinary Procedures Center, Strategic Laboratory	Claudia Regina Gonçalves; Claudio Tavares Sacchi; Erica Valessa Ramos Gomes; Karoline Rodrigues Campos
EPI_ISL_1261691	Laboratório Paulo C. Azevedo	Evandro Chagas Institute	A.M.; Barbagelata; E.C.; E.M.A.; Ferreira; J.A.; Junior; K.C.; L.C.; L.S.; M.C.; P.S.; Pinheiro; Santos; Silva; Sousa; Sousa Junior; W.D.C.; da Silva
EPI_ISL_940608	Laboratório Sao Lucas	Instituto Adolfo Lutz, Interdiciplinary Procedures Center, Strategic Laboratory	Claudia Regina Gonçalves; Claudio Tavares Sacchi; Erica Valessa Ramos Gomes; Karoline Rodrigues Campos

EPI_ISL_417925 to 417926	Laboratório Simili	Bioinformatics Laboratory / LNCC	Alexandra Gerber; Amilcar Tanuri; Ana Paula Guimarães; Cristiano Xavier Lima; Cássia Cristina Alves Gonçalves; Diana Mariani; Ester Cerdeira Sabino; Filipe Romero; Francisco Junior; Ingra Morales Claro; Isabela de Carvalho Leitão; Jaqueline Goes de Jesus; Laboratorio Hermes Pardini; Laboratorio Simile; Luciana Jesus Costa e André Felipe Santos; Luiz Gonzaga Paula de Almeida; Lídia Boullosa; Mariane Talon; Mauro Teixeira; Nuno Rodrigues Faria; Orlando Ferreira Jr.; Pinto Castiñeiras; Rafael de Mello Galliez; Renato Santana Aguiar e Ana Tereza Vasconcelos; Richard Araújo Maia; Rodrigo Brindeiro; Ronaldo da Silva; Ronaldo da Silva Francisco Junior; Terezinha Marta Pereira; Érica Ramos dos Santos Nascimento
EPI_ISL_636737, EPI_ISL_636834 to 636838	Laboratório de Imunofarmacologia - Instituto Oswaldo Cruz	Laboratório de Imunofarmacologia - Instituto Oswaldo Cruz	A.D.; C.Q.; De Paula; F.B.; Ferreira; Fintelman-Rodrigues, N.; M.A. and Sacramento; Saraiva; Souza; T.M.
EPI_ISL_770551 to 770630, EPI_ISL_779155 to 779168	Laboratório de Microbiologia Molecular - Universidade FEEVALE	Bioinformatics Laboratory / LNCC	Alana Witt Hansen; Alessandra Pavan Lamarca da Silva; Alexandra L Gerber; Ana Karolina Eisen Antunes; Ana Luiza Ziulkoski; Ana Paula de C Guimarães; Ana Tereza R de Vasconcelos; Bruna Hermann; Fagner Henrique Heldt; Felipe Benites; Fernando Rosado Spilki; Juliana Schons; Juliane Deise Fleck; Karoline Schallenberger; Larissa Mallmann; Luiz G P de Almeida; Matheus Nunes Weber; Meriane Demoliner; Paula Rodrigues de Almeida; Ronaldo da Silva F Jr; Vyctoria Goes
EPI_ISL_831474, EPI_ISL_831645 to 831646, EPI_ISL_831660, EPI_ISL_831678, EPI_ISL_831681, EPI_ISL_831683, EPI_ISL_831685, EPI_ISL_831688 to 831689, EPI_ISL_831892, EPI_ISL_831898, EPI_ISL_831913, EPI_ISL_831938 to 831940, EPI_ISL_832009 to 832013			
see above	Laboratório de Microbiologia Molecular - Universidade FEEVALE	Universidade Federal de Ciências da Saúde de Porto Alegre	Amanda de Menezes Mayer; Carla Andretta Moreira Neves; Claudia Elizabeth Thompson; Fernando Rosado Spilki; Gabriel Dickin Caldana; Gabriela Bettella Cybis; Lívia Kmetzsch; Patrícia Aline Gröhs Ferrareze; Ricardo Ariel Zimerman; Vinícius Bonetti Franceschi
EPI_ISL_476152 to 476170, EPI_ISL_476337 to 476349, EPI_ISL_476387 to 476402, EPI_ISL_476408 to 476425	Laboratório de Patologia Clínica - UNICAMP	Laboratório de Estudos de Vírus Emergentes - UNICAMP	Angelica Schreiber; Camila Simeoni; Darlan da Silva Candido; Jaqueline Goes Jesus e William Marciel de Souza; José Luiz Proença-Modena; Julia Forato; Julien Theze; Luiz Gonzaga; Magnun Nueldo Nunes dos Santos; Marcilio Jorge Fumagalli; Mariene Ribeiro Amorim; Nuno Rodrigues Faria
EPI_ISL_1004233 to 1004242	Laboratório de Virologia - Instituto de Medicina Tropical - Universidade de São Paulo	Laboratório de Parasitologia Médica - Instituto de Medicina Tropical - Universidade de São Paulo	Alvina Clara Felix; Anderson de Paula; Brazil-UK Centre for Arbovirus Discovery Diagnosis Genomics and Epidemiology (CADDE) Genomic Network - Instituto de Medicina Tropical; Camila Malta Romano; Darlan Candido; Esmenia Coelho; Ester C. Sabino; Flavia Cristina Sales; Franciane Mendes; Giulia Magalhães Ferreira; Ingra Morales Claro; Jaqueline Goes de Jesus; Lucas A. Moyses Franco; Midiã Ferreira; Nuno Faria; Pamela dos Santos Andrade
EPI_ISL_541397 to 541399	Laboratório de Virologia Comparada e Ambiental - LVCA - IOC	Laboratory of Respiratory Viruses and Measles, Oswaldo Cruz Institute, FIOCRUZ	Ana Carolina Mendonça; Camille Ferreira Mannarino; Fernando Motta; Luciana Appolinario; Marilda Siqueira; Marize Pereira Miagostovich on behalf of the Fiocruz COVID-19 Genomic Surveillance Network; Paola Resende; Tatiana Prado; Tulio Machado Fumian
EPI_ISL_541400	Laboratório de Virologia Comparada e Ambiental- LVCA-IOC	Laboratory of Respiratory Viruses and Measles, Oswaldo Cruz Institute, FIOCRUZ	Ana Carolina Mendonça; Camille Ferreira Mannarino; Fernando Motta; Luciana Appolinario; Marilda Siqueira; Marize Pereira Miagostovich on behalf of the Fiocruz COVID-19 Genomic Surveillance Network; Paola Resende; Tatiana Prado; Tulio Machado Fumian
EPI_ISL_1144366 to 1144367, EPI_ISL_1144369, EPI_ISL_1144371, EPI_ISL_1144373 to 1144374, EPI_ISL_1146705 to 1146706, EPI_ISL_1146708 to 1146713, EPI_ISL_1146716, EPI_ISL_1147971 to 1147972, EPI_ISL_1147974, EPI_ISL_1148492, EPI_ISL_1148494 to 1148495, EPI_ISL_1148498, EPI_ISL_1148503, EPI_ISL_1152512, EPI_ISL_1152514, EPI_ISL_1152518, EPI_ISL_1152522 to 1152526, EPI_ISL_1152528, EPI_ISL_1155429, EPI_ISL_1155434 to 1155436, EPI_ISL_1155439 to 1155442, EPI_ISL_1155445, EPI_ISL_1155447 to 1155449, EPI_ISL_1155453 to 1155454, EPI_ISL_1155456, EPI_ISL_1155459 to 1155460, EPI_ISL_1155469, EPI_ISL_1155471, EPI_ISL_1216781 to 1216783, EPI_ISL_1216785 to 1216786, EPI_ISL_1216789, EPI_ISL_1216791, EPI_ISL_1216794 to 1216795, EPI_ISL_1216797, EPI_ISL_1216799, EPI_ISL_1216801, EPI_ISL_1216803 to 1216809, EPI_ISL_1216812, EPI_ISL_1216816, EPI_ISL_1216819, EPI_ISL_1216821, EPI_ISL_1216826, EPI_ISL_1216830 to 1216831, EPI_ISL_1216833, EPI_ISL_1216839, EPI_ISL_1216843, EPI_ISL_1216848, EPI_ISL_1216852, EPI_ISL_1216854, EPI_ISL_1216857, EPI_ISL_1216860, EPI_ISL_1216862, EPI_ISL_1216864 to 1216865, EPI_ISL_1216867, EPI_ISL_1216872, EPI_ISL_1216876, EPI_ISL_1216880, EPI_ISL_1216882, EPI_ISL_1216885, EPI_ISL_1216887 to 1216888, EPI_ISL_1216890 to 1216891, EPI_ISL_1216893, EPI_ISL_1216897, EPI_ISL_1216899, EPI_ISL_1216902, EPI_ISL_1216904, EPI_ISL_1216906, EPI_ISL_1216908, EPI_ISL_1216910, EPI_ISL_1216912 to 1216917, EPI_ISL_1216922, EPI_ISL_1216925 to 1216927, EPI_ISL_1216930, EPI_ISL_1216933, EPI_ISL_1216937 to 1216939, EPI_ISL_1216941, EPI_ISL_1216943, EPI_ISL_1216946 to 1216947, EPI_ISL_1216949 to 1216950, EPI_ISL_1216952 to 1216953, EPI_ISL_1216955, EPI_ISL_1216962 to 1216963, EPI_ISL_1216965 to 1216967, EPI_ISL_1216969, EPI_ISL_1216971 to 1216976, EPI_ISL_1216978 to 1216979, EPI_ISL_1216981 to 1216982, EPI_ISL_1216984, EPI_ISL_1216987 to 1216988, EPI_ISL_1216990, EPI_ISL_1216992, EPI_ISL_1216996 to 1217000, EPI_ISL_1217002 to 1217004, EPI_ISL_1280695, EPI_ISL_1284515, EPI_ISL_1284526, EPI_ISL_1284536, EPI_ISL_1284540, EPI_ISL_1284543, EPI_ISL_1284545, EPI_ISL_1284550 to 1284551			
see above	Labormedizin Darmstadt	Robert Koch Institute	
EPI_ISL_861856 to 861864, EPI_ISL_896069, EPI_ISL_896071, EPI_ISL_896073, EPI_ISL_896077, EPI_ISL_896079 to 896084, EPI_ISL_896087 to 896088, EPI_ISL_896090, EPI_ISL_896092, EPI_ISL_896096 to 896097, EPI_ISL_896099, EPI_ISL_896103 to 896104, EPI_ISL_896106, EPI_ISL_896108, EPI_ISL_896112, EPI_ISL_896115, EPI_ISL_959292 to 959293, EPI_ISL_1036058 to 1036139, EPI_ISL_1233628 to 1233693, EPI_ISL_1299885 to 1299938			
see above	Labormedizinisches Zentrum Dr Risch	University Hospital Basel, Clinical Bacteriology	Adrian Egli; Alfredo Mari; Hans Hirsch; Helena MB Seth-Smith; Julia Bielicki; Karoline Leuzinger; Lorenz Risch; Madlen Stange; Manuel Battegay; Martin Risch; Nadia Wohlwend; Tim Roloff
EPI_ISL_1268653, EPI_ISL_1268655, EPI_ISL_1268658, EPI_ISL_1268759, EPI_ISL_1268761, EPI_ISL_1268790, EPI_ISL_1268893			
see above	Laborzentrum Weser	Hannover Medical School, Institute of Virology	Lars Steinbrück
EPI_ISL_900513, EPI_ISL_900524	Labosud	CNR Virus des Infections Respiratoires - France SUD	Antonin Bal; Bruno Lina; Gregory Destras; Gwendolyne Burfin; Hadrien Règue; Laurence Josset; Martine Valette; Quentin Semanas
EPI_ISL_700466	Ladismith (Nissenville) Clinic wc LAF	NHLS/UCT	Arash Iranzadeh; Bruna Galvao; Carolyn Williamson; Deelan Doolabh; Diana Hardie; Innocent Mudau; Kruger Marais; Lynn Tyers; Marvin Hsiao; Stephen Korsman
EPI_ISL_745124, EPI_ISL_745152	Laetitia Bam CHC	National Health Laboratory Service (NHLS), Tygerberg	Bronwyn Kleinhans; Eduan Wilkindon; Gert van Zyl; Houriiyah Tegally; Kayla Delaney; Susan Engelbrecht; Tulio de Oliveira; Wolfgang Preiser
EPI_ISL_1069174	Laguna Medical Center, Inc	Research Institute for Tropical Medicine	Catalino Demetria; Daria Manalo; Edelwisa Mercado; Francisco Gerardo Polotan; Hannah Leah Morito; Inez Andrea Medado; John Leonard Chan; Joseph Hughes; Kirstyn Brunker; Ma Angelica Tujan; Othoniel Jan Onza
EPI_ISL_434669, EPI_ISL_475515	Lakargruppen	The Public Health Agency of Sweden	Anna Risberg; Anna-Malin Linde; Boris Klanger; Karin Tegmark-Wisell; Maria Lind Karlberg; Mattias Haukland; Mia Brytting; Olov Svartstrom; Oskar Karlsson Lindsjo; Reza Advani; Sandra Broddesson; Theresa Enkirch
EPI_ISL_660130 to 660136	Lancet	National Health Laboratory Service (NHLS), Tygerberg	Bronwyn Kleinhans; Davis M-A; Draper C; Eduan Wilkindon; Gert van Zyl; Houriiyah Tegally; Hsiao M; Kayla Delaney; Siegfried N; Susan Engelbrecht; Tulio de Oliveira; Williamson C; Wolfgang Preiser
EPI_ISL_622943, EPI_ISL_622946 to 622953, EPI_ISL_622955 to 622957, EPI_ISL_622959 to 622969, EPI_ISL_622978, EPI_ISL_622983, EPI_ISL_622985 to 622986, EPI_ISL_622988, EPI_ISL_622990, EPI_ISL_622994, EPI_ISL_623064, EPI_ISL_623068, EPI_ISL_623073			
see above	Lancet Laboratories	National Institute for Communicable Diseases of the National Health Laboratory Service	Allam M; Bhiman JN; Ismail A; Khumalo Z; Kwenda S; Mnyameni F; Mohale T; Mtshali P; Subramoney K
EPI_ISL_960835, EPI_ISL_960843, EPI_ISL_960846, EPI_ISL_960849, EPI_ISL_960851 to 960852, EPI_ISL_960884 to 960885, EPI_ISL_960887, EPI_ISL_960891 to 960892, EPI_ISL_960894 to 960898, EPI_ISL_960935 to 960940, EPI_ISL_1034795, EPI_ISL_1034797, EPI_ISL_1034800 to 1034803, EPI_ISL_1034805, EPI_ISL_1034812 to 1034814, EPI_ISL_1034817, EPI_ISL_1034821 to 1034826, EPI_ISL_1034830, EPI_ISL_1034835, EPI_ISL_1034848, EPI_ISL_1034852 to 1034853, EPI_ISL_1034855 to 1034865, EPI_ISL_1034880 to 1034892, EPI_ISL_1034894 to 1034896, EPI_ISL_1034902 to 1034906, EPI_ISL_1034911 to 1034915, EPI_ISL_1279615, EPI_ISL_1279617, EPI_ISL_1279620 to 1279621, EPI_ISL_1279623 to 1279624, EPI_ISL_1279627, EPI_ISL_1279630, EPI_ISL_1279638 to 1279646, EPI_ISL_1279649 to 1279650, EPI_ISL_1279653 to 1279659, EPI_ISL_1279665 to 1279670, EPI_ISL_1279678 to 1279684, EPI_ISL_1279691 to 1279697, EPI_ISL_1279704 to 1279705, EPI_ISL_1279709 to 1279712, EPI_ISL_1279719 to 1279731, EPI_ISL_1279733, EPI_ISL_1279738 to 1279744, EPI_ISL_1279748 to 1279753, EPI_ISL_1279757 to 1279765, EPI_ISL_1279769 to 1279770, EPI_ISL_1279774, EPI_ISL_1279786 to 1279790, EPI_ISL_1279793 to 1279799, EPI_ISL_1279801 to 1279804, EPI_ISL_1279809 to 1279812, EPI_ISL_1279816 to 1279818, EPI_ISL_1279820, EPI_ISL_1279822 to 1279827, EPI_ISL_1279829 to 1279830, EPI_ISL_1279842 to 1279843, EPI_ISL_1279849 to 1279850, EPI_ISL_1279857 to 1279864, EPI_ISL_1279874, EPI_ISL_1279880 to 1279890			
see above	Landesamt für Verbraucherschutz Sachsen Anhalt, Magdeburg	Institute of Medical Microbiology and Hospital Hygiene	Aljoscha Tersteegen; Prof. Dr. Achim Kaasch
EPI_ISL_1157143 to 1157154, EPI_ISL_1157156 to 1157166	Landesgesundheitsamt Baden-Württemberg	Robert Koch Institute	
EPI_ISL_1217620, EPI_ISL_1217623, EPI_ISL_1217630, EPI_ISL_1217641, EPI_ISL_1217643, EPI_ISL_1217648, EPI_ISL_1217658 to 1217659, EPI_ISL_1217661, EPI_ISL_1217668 to 1217669, EPI_ISL_1217672, EPI_ISL_1217675, EPI_ISL_1217679, EPI_ISL_1217692, EPI_ISL_1217694 to 1217695, EPI_ISL_1217703, EPI_ISL_1285060, EPI_ISL_1285069, EPI_ISL_1285079 to 1285080, EPI_ISL_1285093 to 1285094, EPI_ISL_1285096, EPI_ISL_1285110, EPI_ISL_1285115, EPI_ISL_1285117, EPI_ISL_1285120, EPI_ISL_1285127 to 1285130, EPI_ISL_1285134 to 1285135, EPI_ISL_1285143, EPI_ISL_1285146 to 1285147, EPI_ISL_1285149, EPI_ISL_1285152, EPI_ISL_1285154, EPI_ISL_1285156, EPI_ISL_1285158, EPI_ISL_1285163 to 1285165			
see above	Landesuntersuchungsanstalt für das Gesundheits- und Veterinärwesen (LUA) Sachsen	Robert Koch Institute	
EPI_ISL_982539, EPI_ISL_982585, EPI_ISL_982613, EPI_ISL_982627 to 982630, EPI_ISL_1038793 to 1038795, EPI_ISL_1091577, EPI_ISL_1091605, EPI_ISL_1091608 to 1091610, EPI_ISL_1091618, EPI_ISL_1091717 to 1091719, EPI_ISL_1261673 to 1261679			
see above	Landstuhl Regional Medical Center	US Air Force School of Aerospace Medicine	Amanda Javorina; Anthony Fries; Clarise Starr; Cole Anderson; Elizabeth Macias; Fritz Castillo; Jennifer Meyer; Sarah Purves; William Buggele; William Gruner
EPI_ISL_738317 to 738349, EPI_ISL_812258 to 812285	Landstuhl Regional Medical Center	United States Air Force School of Aerospace Medicine	Amanda Javorina; Anthony Fries; Clarise Starr; Cole Anderson; Elizabeth Macias; Fritz Castillo; Jennifer Meyer; Sarah Purves; William Gruner
EPI_ISL_745130, EPI_ISL_745155, EPI_ISL_745160	Langebaan Clinic wc LBC	National Health Laboratory Service (NHLS), Tygerberg	Bronwyn Kleinhans; Eduan Wilkindon; Gert van Zyl; Houriiyah Tegally; Kayla Delaney; Susan Engelbrecht; Tulio de Oliveira; Wolfgang Preiser
EPI_ISL_534237 to 534238, EPI_ISL_548255	Lanssjukhuset Kalmar	The Public Health Agency of Sweden	Anna Risberg; Anna-Malin Linde; Karin Tegmark-Wisell; Maria Lind Karlberg; Mattias Haukland; Mia Brytting; Olov Svartstrom; Oskar Karlsson Lindsjo;

			Petra Edquist; Reza Advani; Sandra Broddesson
EPI_ISL_407079	Lapland Central Hospital	Department of Virology, University of Helsinki and Helsinki University Hospital, Helsinki, Finland	Hannimari Kallio-Kokko; Olli Vapalahti; Suvi Kuivanen; Teemu Smura
EPI_ISL_961831, EPI_ISL_961852, EPI_ISL_961862 to 961863, EPI_ISL_961866, EPI_ISL_961869 to 961870, EPI_ISL_961876	Latvijas Infektologijas Centrs	Latvian Biomedical Research and Study Centre	Davids Fridmanis; Diana Dusacka; Ivars Silamikelis; Janis Pjalkovskis; Janis Klovins; Jurijs Perevoscikovs; Kaspars Megnis; Laila Silamikele; Lauma Freimane; Laura Ansone; Liga Birzniece; Monta Ustinova; Nikita Zrelovs; Reinis Zeltmatis; Uga Dumpis; Vita Rovite
EPI_ISL_437089 to 437096, EPI_ISL_486422 to 486426, EPI_ISL_486428, EPI_ISL_486430 to 486436, EPI_ISL_512647 to 512652, EPI_ISL_515185 to 515186, EPI_ISL_534204 to 534205, EPI_ISL_534209, EPI_ISL_639634, EPI_ISL_639639, EPI_ISL_770047, EPI_ISL_770060			
see above	Latvijas Infektoloijas centrs	Latvian Biomedical Research and Study Centre	Ivars Silamielis; Jeena Storoženko; Jnis Kloviš; Jnis Pjalkovskis; Kaspars Megnis; Monta Ustinova; Oksana Savicka; Tatjana Kolupajeva; Uga Dumpis; Vita Rovte; ikita Zrelovs
EPI_ISL_745095	Laverty Pathology	CIDM-PH, Westmead Hospital	CIDM-PH et al.
EPI_ISL_451489, EPI_ISL_451545, EPI_ISL_451572, EPI_ISL_451602, EPI_ISL_451609, EPI_ISL_451611, EPI_ISL_451631, EPI_ISL_451641, EPI_ISL_455041, EPI_ISL_455048, EPI_ISL_455063, EPI_ISL_498769 to 498772, EPI_ISL_513403 to 513408, EPI_ISL_526143, EPI_ISL_526202 to 526204, EPI_ISL_544957, EPI_ISL_544962, EPI_ISL_545040, EPI_ISL_591487 to 591491, EPI_ISL_593653, EPI_ISL_629007 to 629009, EPI_ISL_639731, EPI_ISL_639733, EPI_ISL_699644, EPI_ISL_740892 to 740893, EPI_ISL_767869, EPI_ISL_767878 to 767880, EPI_ISL_767893, EPI_ISL_767899, EPI_ISL_767903, EPI_ISL_767919, EPI_ISL_767938, EPI_ISL_792028 to 792030, EPI_ISL_792033, EPI_ISL_806720, EPI_ISL_872573, EPI_ISL_872576			
see above	Laverty Pathology	NSW Health Pathology - Institute of Clinical Pathology and Medical Research; Westmead Hospital; University of Sydney	CIDM-PH et al.
EPI_ISL_421507 to 421508	Le Château de Seine-Port	National Reference Center for Viruses of Respiratory Infections, Institut Pasteur, Paris	Angela Brisebarre; Etienne Simon-Lorière; Flora Donati; Marion Barbet; Maud Vanpeene; Mélanie Albert; Méline Bizard; Sylvie Behillil; Sylvie van der Werf; Vincent Enouf
EPI_ISL_498551 to 498552, EPI_ISL_498554, EPI_ISL_498556	Lebanese American University	Lebanese American University	Abdallah, J.; Abi Habib, W.; El Shesheny, R.; Goldstein; J. and Kayali, G.; Mokhbat, J.; R.J.; Webby
EPI_ISL_447588	Lednicky Lab	Lednicky lab	Alam; C.J.; Elbadry; Gibson; J.A.; J.C.; J.G. Jr. and Lednicky; M.A.; M.M.; Morris; Stephenson; Subramaniam, K.; T.B.; Waltzek
EPI_ISL_434636	Lednicky Laboratory, Emerging Pathogens Institute, University of Florida	Lednicky Laboratory at Emerging Pathogens Institute, University of Florida	Alam, M.; C.J.; Elbadry; Gibson; J.A.; J.C.; J.G. Jr. and Lednicky; M.A.; Morris; Stephenson; Subramaniam, K.; T.B.; Waltzek
EPI_ISL_434637	Lednicky Laboratory, Emerging Pathogens Institute, University of Florida.	Lednicky Laboratory, Emerging Pathogens Institute, University of Florida.	C.J.; Elbadry; Gibson; J.A.; J.C.; J.G. Jr. and Lednicky; M.A.; Morris; Stephenson; Subramaniam, K.; T.B.; Waltzek
EPI_ISL_569664 to 569688	Lee Lab	Lee Lab	Gina Faraci; Jane F. Emerson; Pamela M. Ward; Sung Yong Park; and Ha Youn Lee
EPI_ISL_538811, EPI_ISL_538818, EPI_ISL_538841, EPI_ISL_538851, EPI_ISL_538863, EPI_ISL_538866, EPI_ISL_538899, EPI_ISL_538917, EPI_ISL_538945, EPI_ISL_538987, EPI_ISL_538994, EPI_ISL_539006, EPI_ISL_539030, EPI_ISL_539070, EPI_ISL_539116 to 539117, EPI_ISL_539120, EPI_ISL_539143, EPI_ISL_539150, EPI_ISL_539152, EPI_ISL_539157, EPI_ISL_539185, EPI_ISL_539197, EPI_ISL_539213			
see above	Leeds Teaching Hospitals NHS Trust and Public Health England, National Infection Service (Leeds laboratory)	Wellcome Sanger Institute for the COVID-19 Genomics UK (COG-UK) Consortium	Antony Hale and Alex Alderton; Cordelia Langford; David K. Jackson; Dominic Kwiatkowski; Ewan Harrison; Holli Carden; Ian Johnston; John Sillitoe on behalf of the Wellcome Sanger Institute COVID-19 Surveillance Team; Katherine L. Harper; Louissa Macfarlane-Smith; Roberto Amato; Sonia Goncalves
EPI_ISL_538748 to 538810, EPI_ISL_538812 to 538817, EPI_ISL_538819 to 538840, EPI_ISL_538842 to 538850, EPI_ISL_538852 to 538862, EPI_ISL_538864 to 538865, EPI_ISL_538867 to 538898, EPI_ISL_538900 to 538916, EPI_ISL_538918 to 538944, EPI_ISL_538946 to 538986, EPI_ISL_538988 to 538993, EPI_ISL_538995 to 539005, EPI_ISL_539007 to 539029, EPI_ISL_539031 to 539069, EPI_ISL_539071 to 539115, EPI_ISL_539118 to 539119, EPI_ISL_539121 to 539142, EPI_ISL_539144 to 539149, EPI_ISL_539151, EPI_ISL_539153 to 539156, EPI_ISL_539158 to 539184, EPI_ISL_539186 to 539196, EPI_ISL_539198 to 539212, EPI_ISL_539214 to 539220			
see above	Leeds Teaching Hospitals NHS Trust and Public Health England, National Infection Service (Leeds laboratory)	Wellcome Sanger Institute for the COVID-19 Genomics UK (COG-UK) consortium	Antony Hale and Alex Alderton; Cordelia Langford; David K. Jackson; Dominic Kwiatkowski; Ewan Harrison; Holli Carden; Ian Johnston; John Sillitoe on behalf of the Wellcome Sanger Institute COVID-19 Surveillance Team; Katherine L. Harper; Louissa Macfarlane-Smith; Roberto Amato; Sonia Goncalves
EPI_ISL_1040702, EPI_ISL_1040709	Lentegeur Hospital wc LGH	NHLS/UCT	Arash Iranzadeh; Bruna Galvao; Carolyn Williamson; Deelan Doolabh; Diana Hardie; Innocent Mudau; Kruger Marais; Lynn Tyers; Marvin Hsiao; Stephen Korsman
EPI_ISL_1252723, EPI_ISL_1252805 to 1252808	Life Sciences Center, Vilnius University	Vilnius University Hospital Santaros Klinikos, Center of Laboratory Medicine	Daniel Naumovas; Dovile Ezerskyte; Gytis Dudas; Ingrida Olendraite; Laimonas Griskevicius; Ligita Raugaite; Mindaugas Stoskus; Monika Katenaite; Rimvydas Norvilas
EPI_ISL_511644	Life and Health Sciences Research Institute, University of Minho, Braga	Instituto Nacional de Saude (INSA)	Borges et al
EPI_ISL_551294, EPI_ISL_551327, EPI_ISL_551412, EPI_ISL_551453, EPI_ISL_551484, EPI_ISL_551496, EPI_ISL_551530, EPI_ISL_552492, EPI_ISL_552818, EPI_ISL_552841, EPI_ISL_552972 to 552973, EPI_ISL_553421, EPI_ISL_553568, EPI_ISL_553778, EPI_ISL_553784, EPI_ISL_553812, EPI_ISL_553846, EPI_ISL_553866, EPI_ISL_553946, EPI_ISL_554478, EPI_ISL_554484, EPI_ISL_554497, EPI_ISL_554537, EPI_ISL_554601, EPI_ISL_554667, EPI_ISL_554709, EPI_ISL_554763, EPI_ISL_554845, EPI_ISL_554868, EPI_ISL_554929, EPI_ISL_555019, EPI_ISL_555041, EPI_ISL_555127, EPI_ISL_555129, EPI_ISL_555148, EPI_ISL_555259, EPI_ISL_555303 to 555304, EPI_ISL_555391, EPI_ISL_555398, EPI_ISL_555432 to 555433, EPI_ISL_555502, EPI_ISL_555510 to 555511, EPI_ISL_555650 to 555651, EPI_ISL_555660, EPI_ISL_555794, EPI_ISL_555807, EPI_ISL_555815, EPI_ISL_555887, EPI_ISL_555947, EPI_ISL_555986, EPI_ISL_555990, EPI_ISL_556000, EPI_ISL_556141, EPI_ISL_556348, EPI_ISL_556350, EPI_ISL_556411, EPI_ISL_556428, EPI_ISL_556433, EPI_ISL_556435, EPI_ISL_556505, EPI_ISL_556510, EPI_ISL_557002, EPI_ISL_557043, EPI_ISL_557110, EPI_ISL_557142, EPI_ISL_557625, EPI_ISL_557711, EPI_ISL_557728, EPI_ISL_557730, EPI_ISL_557739, EPI_ISL_557760, EPI_ISL_557852, EPI_ISL_557896, EPI_ISL_557999, EPI_ISL_558024, EPI_ISL_558126, EPI_ISL_558185, EPI_ISL_558222, EPI_ISL_558311, EPI_ISL_558362, EPI_ISL_558387, EPI_ISL_558422, EPI_ISL_558432, EPI_ISL_558543, EPI_ISL_558605, EPI_ISL_558617, EPI_ISL_558796, EPI_ISL_558846, EPI_ISL_558872, EPI_ISL_558877, EPI_ISL_558888, EPI_ISL_558950, EPI_ISL_558994, EPI_ISL_559019, EPI_ISL_559042, EPI_ISL_559046, EPI_ISL_559120, EPI_ISL_559129, EPI_ISL_559213, EPI_ISL_559228, EPI_ISL_559261, EPI_ISL_559275, EPI_ISL_559337, EPI_ISL_559353, EPI_ISL_559447, EPI_ISL_559576, EPI_ISL_559591, EPI_ISL_559616, EPI_ISL_559644, EPI_ISL_567803, EPI_ISL_567811, EPI_ISL_575536, EPI_ISL_579951, EPI_ISL_579971, EPI_ISL_580062, EPI_ISL_580138, EPI_ISL_580187, EPI_ISL_580210, EPI_ISL_580285, EPI_ISL_580563, EPI_ISL_580686, EPI_ISL_580708, EPI_ISL_580780, EPI_ISL_580824, EPI_ISL_581423, EPI_ISL_586612, EPI_ISL_586617, EPI_ISL_586621, EPI_ISL_586709, EPI_ISL_586907, EPI_ISL_587085, EPI_ISL_587265, EPI_ISL_587977, EPI_ISL_588007, EPI_ISL_588026, EPI_ISL_589289, EPI_ISL_589302, EPI_ISL_589329, EPI_ISL_589342, EPI_ISL_589344, EPI_ISL_589376, EPI_ISL_597633, EPI_ISL_597984, EPI_ISL_598163, EPI_ISL_606948, EPI_ISL_606960, EPI_ISL_607189, EPI_ISL_607200, EPI_ISL_607894, EPI_ISL_608147, EPI_ISL_608216, EPI_ISL_608310, EPI_ISL_609209, EPI_ISL_610332, EPI_ISL_610502 to 610503, EPI_ISL_611093, EPI_ISL_623864, EPI_ISL_623904, EPI_ISL_623974, EPI_ISL_623983, EPI_ISL_624094, EPI_ISL_624244, EPI_ISL_624291, EPI_ISL_624350, EPI_ISL_624354, EPI_ISL_624909, EPI_ISL_625213, EPI_ISL_625240, EPI_ISL_630474, EPI_ISL_630533, EPI_ISL_630547, EPI_ISL_630780, EPI_ISL_630891, EPI_ISL_644095 to 644098, EPI_ISL_644100, EPI_ISL_644103 to 644106, EPI_ISL_644108 to 644111, EPI_ISL_644113, EPI_ISL_644118, EPI_ISL_645240, EPI_ISL_645266, EPI_ISL_645297, EPI_ISL_645303, EPI_ISL_645362, EPI_ISL_645368, EPI_ISL_645418, EPI_ISL_645452, EPI_ISL_645459, EPI_ISL_645464, EPI_ISL_645473, EPI_ISL_645499, EPI_ISL_645502 to 645503, EPI_ISL_645508, EPI_ISL_645530 to 645825, EPI_ISL_645827, EPI_ISL_645830 to 645831, EPI_ISL_645833 to 645835, EPI_ISL_645837, EPI_ISL_645839, EPI_ISL_645841 to 645849, EPI_ISL_645853 to 645865, EPI_ISL_645867, EPI_ISL_645870 to 645876, EPI_ISL_645878 to 645879, EPI_ISL_645881, EPI_ISL_645883 to 645884, EPI_ISL_645887 to 645895, EPI_ISL_645898, EPI_ISL_645900 to 645901, EPI_ISL_645903 to 645904, EPI_ISL_645906 to 645907, EPI_ISL_645909 to 645915, EPI_ISL_645919, EPI_ISL_645921 to 645922, EPI_ISL_645924, EPI_ISL_645928 to 645929, EPI_ISL_645931 to 645944, EPI_ISL_645946 to 645948, EPI_ISL_645950 to 645957, EPI_ISL_645960 to 645961, EPI_ISL_645963 to 645967, EPI_ISL_645969, EPI_ISL_645971 to 645978, EPI_ISL_645982, EPI_ISL_645984 to 645986, EPI_ISL_645988, EPI_ISL_645990 to 645991, EPI_ISL_645996 to 645997, EPI_ISL_645999 to 646008, EPI_ISL_646010 to 646013, EPI_ISL_646015 to 646016, EPI_ISL_646019 to 646021, EPI_ISL_646023 to 646026, EPI_ISL_646029, EPI_ISL_646033, EPI_ISL_646036 to 646038, EPI_ISL_646040, EPI_ISL_646042 to 646045, EPI_ISL_646048 to 646051, EPI_ISL_646053 to 646055, EPI_ISL_646057, EPI_ISL_646059 to 646066, EPI_ISL_646068 to 646069, EPI_ISL_646071 to 646075, EPI_ISL_646077 to 646082, EPI_ISL_646087 to 646088, EPI_ISL_646090, EPI_ISL_646092 to 646100, EPI_ISL_646103 to 646104, EPI_ISL_646106 to 646114, EPI_ISL_646116 to 646121, EPI_ISL_646123 to 646127, EPI_ISL_646130, EPI_ISL_646132 to 646134, EPI_ISL_646137 to 646138, EPI_ISL_646148, EPI_ISL_646150, EPI_ISL_646155 to 646157, EPI_ISL_646162, EPI_ISL_646166, EPI_ISL_646169 to 646170, EPI_ISL_646173, EPI_ISL_646180, EPI_ISL_646182, EPI_ISL_646185, EPI_ISL_646189, EPI_ISL_646191, EPI_ISL_646193, EPI_ISL_646198 to 646199, EPI_ISL_646204, EPI_ISL_646207 to 646208, EPI_ISL_646212, EPI_ISL_646215, EPI_ISL_646223 to 646224, EPI_ISL_646231, EPI_ISL_646235, EPI_ISL_646242, EPI_ISL_646245, EPI_ISL_646248 to 646249, EPI_ISL_646252, EPI_ISL_646261, EPI_ISL_646268 to 646269, EPI_ISL_646271, EPI_ISL_646276 to 646279, EPI_ISL_646283, EPI_ISL_646291, EPI_ISL_646297, EPI_ISL_646299, EPI_ISL_646303, EPI_ISL_646305, EPI_ISL_646307, EPI_ISL_646309, EPI_ISL_646313 to 646314, EPI_ISL_646318, EPI_ISL_646320, EPI_ISL_646323, EPI_ISL_646327, EPI_ISL_646334, EPI_ISL_646342 to 646343, EPI_ISL_646345 to 646347, EPI_ISL_646349 to 646350, EPI_ISL_646354, EPI_ISL_646356, EPI_ISL_646362 to 646363, EPI_ISL_646366, EPI_ISL_646369 to 646370, EPI_ISL_646373, EPI_ISL_646375 to 646377, EPI_ISL_646382, EPI_ISL_646385, EPI_ISL_646388, EPI_ISL_646397, EPI_ISL_646402, EPI_ISL_646412, EPI_ISL_646418 to 646420, EPI_ISL_646426, EPI_ISL_646428 to 646432, EPI_ISL_646434, EPI_ISL_646441 to 646443, EPI_ISL_646448, EPI_ISL_646454, EPI_ISL_646463, EPI_ISL_646466 to 646468, EPI_ISL_646476 to 646479, EPI_ISL_646485 to 646494, EPI_ISL_646497, EPI_ISL_646500, EPI_ISL_646502, EPI_ISL_646505 to 646506, EPI_ISL_646508 to 646516, EPI_ISL_646518, EPI_ISL_646520 to 646522, EPI_ISL_646525 to 646526, EPI_ISL_646528 to 646529, EPI_ISL_646532 to 646533, EPI_ISL_646535, EPI_ISL_646537 to 646538, EPI_ISL_646540 to 646546, EPI_ISL_646548 to 646549, EPI_ISL_646551, EPI_ISL_646555, EPI_ISL_646557 to 646561, EPI_ISL_646563 to 646564, EPI_ISL_646566 to 646569, EPI_ISL_646571 to 646573, EPI_ISL_646576 to 646577, EPI_ISL_646580 to 646581, EPI_ISL_646583, EPI_ISL_646585 to 646586, EPI_ISL_646591 to 646592, EPI_ISL_646594 to 646595, EPI_ISL_646597, EPI_ISL_646599 to 646601, EPI_ISL_646603, EPI_ISL_646605 to 646607, EPI_ISL_646610, EPI_ISL_646612 to 646614, EPI_ISL_646616, EPI_ISL_646618, EPI_ISL_646621, EPI_ISL_646623, EPI_ISL_646625 to 646626, EPI_ISL_646628, EPI_ISL_646631, EPI_ISL_646635, EPI_ISL_646638 to 646640, EPI_ISL_646642, EPI_ISL_646648, EPI_ISL_646651 to 646653, EPI_ISL_646655, EPI_ISL_646658, EPI_ISL_646660, EPI_ISL_646662, EPI_ISL_646664 to 646666, EPI_ISL_646668, EPI_ISL_646671, EPI_ISL_646673, EPI_ISL_646675 to 646676, EPI_ISL_646678, EPI_ISL_646683 to 646684, EPI_ISL_646686 to 646687, EPI_ISL_646689, EPI_ISL_646691 to 646693, EPI_ISL_646695 to 646698, EPI_ISL_646702, EPI_ISL_646706 to 646708, EPI_ISL_646710 to 646713, EPI_ISL_646715 to 646716, EPI_ISL_646718 to 646719, EPI_ISL_646721 to 646722, EPI_ISL_646724, EPI_ISL_646727, EPI_ISL_646731, EPI_ISL_646734, EPI_ISL_646736 to 646741, EPI_ISL_646743 to 646744, EPI_ISL_646746 to 646747, EPI_ISL_646749, EPI_ISL_646751 to 646753, EPI_ISL_646755 to 646758, EPI_ISL_646760 to 646763, EPI_ISL_646767 to 646771, EPI_ISL_646773, EPI_ISL_646776, EPI_ISL_646782, EPI_ISL_646784, EPI_ISL_646787, EPI_ISL_646789 to 646791, EPI_ISL_646793, EPI_ISL_646795 to 646797, EPI_ISL_646799 to 646809, EPI_ISL_646811 to 646817, EPI_ISL_646819 to 646824, EPI_ISL_646827 to 646836, EPI_ISL_646838 to 646839, EPI_ISL_646841 to 646849, EPI_ISL_646851 to 646852, EPI_ISL_646855 to 646867, EPI_ISL_646871, EPI_ISL_646873, EPI_ISL_646875 to 646876, EPI_ISL_646878 to 646879, EPI_ISL_646881 to 646884, EPI_ISL_646886 to 646890, EPI_ISL_646893 to 646901, EPI_ISL_646903 to 646904, EPI_ISL_646906 to 646911, EPI_ISL_646913 to 646914, EPI_ISL_646916 to 646920, EPI_ISL_646922 to 646928, EPI_ISL_646930 to 646935, EPI_ISL_646938, EPI_ISL_646940 to 646947, EPI_ISL_646949 to 646954, EPI_ISL_646956 to 646959, EPI_ISL_646961 to 646963, EPI_ISL_646965 to 646966, EPI_ISL_646968 to 646973, EPI_ISL_646978 to 646982, EPI_ISL_646984 to 646987, EPI_ISL_646989 to 646991, EPI_ISL_646993 to 646995, EPI_ISL_646997 to 647000, EPI_ISL_647002, EPI_ISL_647004 to 647005, EPI_ISL_647007 to 647013, EPI_ISL_647015 to 647022, EPI_ISL_647024 to 647030, EPI_ISL_647033 to 647034, EPI_ISL_647036 to 647037, EPI_ISL_647039 to 647044, EPI_ISL_647046 to 647049, EPI_ISL_647051 to 647052, EPI_ISL_647054 to 647058, EPI_ISL_647060, EPI_ISL_647062 to 647066, EPI_ISL_647068 to 647072, EPI_ISL_647074 to 647075, EPI_ISL_647077 to 647080, EPI_ISL_647082 to 647086, EPI_ISL_647088 to 647089, EPI_ISL_647091 to 647096, EPI_ISL_647098 to 647101, EPI_ISL_647103 to 647104, EPI_ISL_647106 to 647108, EPI_ISL_647111 to 647113, EPI_ISL_647116, EPI_ISL_647118 to 647120, EPI_ISL_647122, EPI_ISL_647468 to 647469, EPI_ISL_647473 to 647474, EPI_ISL_647477, EPI_ISL_647497, EPI_ISL_647499, EPI_ISL_647503, EPI_ISL_647506, EPI_ISL_647511, EPI_ISL_647513, EPI_ISL_647526, EPI_ISL_647528, EPI_ISL_647535 to 647536, EPI_ISL_647541, EPI_ISL_647546, EPI_ISL_647550, EPI_ISL_647553, EPI_ISL_647558, EPI_ISL_647560, EPI_ISL_647572 to 647573, EPI_ISL_647576, EPI_ISL_647579 to 647580, EPI_ISL_647584, EPI_ISL_647591, EPI_ISL_647594, EPI_ISL_647603, EPI_ISL_647606, EPI_ISL_647608, EPI_ISL_647612, EPI_ISL_647615, EPI_ISL_647618, EPI_ISL_647624, EPI_ISL_647630, EPI_ISL_647632, EPI_ISL_647634 to 647635, EPI_ISL_647638, EPI_ISL_647643, EPI_ISL_647650, EPI_ISL_647655, EPI_ISL_647659, EPI_ISL_647667 to 647668, EPI_ISL_647672, EPI_ISL_647677, EPI_ISL_647682 to 647683, EPI_ISL_647689, EPI_ISL_647693, EPI_ISL_647695, EPI_ISL_647698, EPI_ISL_647700, EPI_ISL_647704 to 647705, EPI_ISL_647708, EPI_ISL_647711, EPI_ISL_647726, EPI_ISL_647733, EPI_ISL_647741, EPI_ISL_647750, EPI_ISL_647752, EPI_ISL_647762, EPI_ISL_647771, EPI_ISL_647776, EPI_ISL_647780, EPI_ISL_647786 to 647787, EPI_ISL_647792, EPI_ISL_649193 to 649204, EPI_ISL_649206 to 649254, EPI_ISL_649256 to 649282, EPI_ISL_649284 to 649319, EPI_ISL_649321 to 649361, EPI_ISL_649363 to 649385, EPI_ISL_649387 to 649404, EPI_ISL_649406 to 649423, EPI_ISL_649425 to 649478, EPI_ISL_649480 to 649481, EPI_ISL_649483 to 649491, EPI_ISL_655273 to 655650, EPI_ISL_655652 to 655659, EPI_ISL_655661, EPI_ISL_655663 to 655671, EPI_ISL_655673 to			

655676, EPI_ISL_655678 to 655693, EPI_ISL_655696, EPI_ISL_655698, EPI_ISL_655700 to 655710, EPI_ISL_655712 to 655718, EPI_ISL_655720 to 655722, EPI_ISL_655724 to 655726, EPI_ISL_655728 to 655734, EPI_ISL_655736, EPI_ISL_655738, EPI_ISL_655740 to 655743, EPI_ISL_655746 to 655755, EPI_ISL_655757 to
655761, EPI_ISL_655763 to 655767, EPI_ISL_655771 to 655772, EPI_ISL_655774 to 655778, EPI_ISL_655781 to 655782, EPI_ISL_655784 to 655787, EPI_ISL_655789 to 655797, EPI_ISL_655799 to 655803, EPI_ISL_655805, EPI_ISL_655808, EPI_ISL_655810 to 655816, EPI_ISL_655818, EPI_ISL_655820 to 655821,
EPI_ISL_655823 to 655827, EPI_ISL_655829 to 655831, EPI_ISL_655833 to 655840, EPI_ISL_655842 to 655846, EPI_ISL_655848 to 655849, EPI_ISL_655851 to 655853, EPI_ISL_655855 to 655860, EPI_ISL_655862 to 655863, EPI_ISL_655866 to 655867, EPI_ISL_655869 to 655872, EPI_ISL_655875 to 655878,
EPI_ISL_655880 to 655882, EPI_ISL_655884 to 655890, EPI_ISL_655892 to 655906, EPI_ISL_655908 to 655910, EPI_ISL_655913 to 655917, EPI_ISL_655919, EPI_ISL_655921 to 655923, EPI_ISL_655925 to 655928, EPI_ISL_655930 to 655932, EPI_ISL_655934 to 655946, EPI_ISL_655949 to 655956, EPI_ISL_655958,
EPI_ISL_655960 to 655966, EPI_ISL_655968 to 655970, EPI_ISL_655972, EPI_ISL_655974, EPI_ISL_655976 to 656684, EPI_ISL_661956, EPI_ISL_661958, EPI_ISL_661961 to 661962, EPI_ISL_661964 to 661966, EPI_ISL_661968, EPI_ISL_661970, EPI_ISL_661974, EPI_ISL_661977 to 661978, EPI_ISL_661980,
EPI_ISL_661982 to 661983, EPI_ISL_661985 to 661986, EPI_ISL_661988 to 661992, EPI_ISL_661995, EPI_ISL_661998 to 662002, EPI_ISL_662004, EPI_ISL_662006, EPI_ISL_662008, EPI_ISL_662010, EPI_ISL_662013 to 662015, EPI_ISL_662017 to 662020, EPI_ISL_662022, EPI_ISL_662025, EPI_ISL_662029,
EPI_ISL_662031 to 662032, EPI_ISL_662034, EPI_ISL_662036, EPI_ISL_662038, EPI_ISL_662043, EPI_ISL_662045 to 662051, EPI_ISL_662053, EPI_ISL_662055 to 662058, EPI_ISL_662060 to 662063, EPI_ISL_662065, EPI_ISL_662067 to 662068, EPI_ISL_662070 to 662071, EPI_ISL_662074, EPI_ISL_662078 to 662081,
EPI_ISL_662086, EPI_ISL_662093, EPI_ISL_662097 to 662101, EPI_ISL_662105 to 662108, EPI_ISL_662114 to 662116, EPI_ISL_662118, EPI_ISL_662120, EPI_ISL_662122 to 662124, EPI_ISL_662129, EPI_ISL_662131 to 662133, EPI_ISL_662135, EPI_ISL_662137 to 662138, EPI_ISL_662140 to 662141, EPI_ISL_662143,
EPI_ISL_662145 to 662146, EPI_ISL_662148, EPI_ISL_662150 to 662151, EPI_ISL_662156, EPI_ISL_662159 to 662160, EPI_ISL_662162 to 662167, EPI_ISL_662169 to 662170, EPI_ISL_662173 to 662179, EPI_ISL_662181 to 662184, EPI_ISL_662186 to 662187, EPI_ISL_662191 to 662192, EPI_ISL_662194, EPI_ISL_662196,
EPI_ISL_662198, EPI_ISL_662200, EPI_ISL_662202 to 662203, EPI_ISL_662206 to 662209, EPI_ISL_662212, EPI_ISL_662214 to 662215, EPI_ISL_662217 to 662219, EPI_ISL_662223, EPI_ISL_662227 to 662229, EPI_ISL_662231, EPI_ISL_662233 to 662240, EPI_ISL_662244 to 662245, EPI_ISL_662247 to 662249,
EPI_ISL_662252, EPI_ISL_662254 to 662255, EPI_ISL_662258, EPI_ISL_662260, EPI_ISL_662263, EPI_ISL_662265 to 662267, EPI_ISL_662269 to 662270, EPI_ISL_662272 to 662276, EPI_ISL_662279, EPI_ISL_662281, EPI_ISL_662285 to 662286, EPI_ISL_662290, EPI_ISL_662292, EPI_ISL_662296, EPI_ISL_662300,
EPI_ISL_662305, EPI_ISL_662308 to 662309, EPI_ISL_662312, EPI_ISL_662320, EPI_ISL_662327, EPI_ISL_662334, EPI_ISL_662342 to 662344, EPI_ISL_662352, EPI_ISL_662354, EPI_ISL_662356, EPI_ISL_662358 to 662359, EPI_ISL_662365, EPI_ISL_662369, EPI_ISL_662373 to 662375, EPI_ISL_662381 to 662382,
EPI_ISL_662385, EPI_ISL_662389 to 662391, EPI_ISL_662393 to 662394, EPI_ISL_662403, EPI_ISL_662405, EPI_ISL_662410 to 662412, EPI_ISL_662415, EPI_ISL_662418 to 662419, EPI_ISL_662427, EPI_ISL_662429, EPI_ISL_662431, EPI_ISL_662433, EPI_ISL_662444, EPI_ISL_662446, EPI_ISL_662452,
EPI_ISL_662457, EPI_ISL_662462 to 662463, EPI_ISL_662468, EPI_ISL_662472, EPI_ISL_662474, EPI_ISL_662477 to 662478, EPI_ISL_662480, EPI_ISL_662486 to 662489, EPI_ISL_662492, EPI_ISL_662496, EPI_ISL_662499, EPI_ISL_662502, EPI_ISL_662507 to 662508, EPI_ISL_662514, EPI_ISL_662518,
EPI_ISL_662520, EPI_ISL_662526, EPI_ISL_662535, EPI_ISL_662549, EPI_ISL_662553, EPI_ISL_662558, EPI_ISL_662564, EPI_ISL_662575 to 662576, EPI_ISL_662578 to 662581, EPI_ISL_662583, EPI_ISL_662588, EPI_ISL_662593, EPI_ISL_662597, EPI_ISL_662604 to 662605, EPI_ISL_662609 to 662610,
EPI_ISL_662614, EPI_ISL_662616 to 662617, EPI_ISL_662621 to 662626, EPI_ISL_662628 to 662630, EPI_ISL_662647 to 662650, EPI_ISL_674159 to 674162, EPI_ISL_674164 to 674165, EPI_ISL_674168 to 674172, EPI_ISL_674174 to 674177, EPI_ISL_674179, EPI_ISL_674181 to 674182, EPI_ISL_674186, EPI_ISL_674188,
EPI_ISL_674190, EPI_ISL_674192 to 674195, EPI_ISL_674197, EPI_ISL_674199, EPI_ISL_674201, EPI_ISL_674203 to 674204, EPI_ISL_674206 to 674208, EPI_ISL_674211, EPI_ISL_674217, EPI_ISL_674222 to 674224, EPI_ISL_674226 to 674229, EPI_ISL_674231, EPI_ISL_674233 to 674234, EPI_ISL_674240 to 674243,
EPI_ISL_674245, EPI_ISL_674249, EPI_ISL_674253 to 674255, EPI_ISL_674260 to 674261, EPI_ISL_674264, EPI_ISL_674267 to 674268, EPI_ISL_674272, EPI_ISL_674276 to 674277, EPI_ISL_674281, EPI_ISL_674283 to 674287, EPI_ISL_674292, EPI_ISL_674294 to 674297, EPI_ISL_674299 to 674301, EPI_ISL_674305,
EPI_ISL_674308, EPI_ISL_674310 to 674311, EPI_ISL_674313, EPI_ISL_674317 to 674319, EPI_ISL_674322 to 674324, EPI_ISL_674328 to 674329, EPI_ISL_674332, EPI_ISL_674334, EPI_ISL_674337, EPI_ISL_674339 to 674341, EPI_ISL_674343, EPI_ISL_674345 to 674347, EPI_ISL_674351 to 674352, EPI_ISL_674354,
EPI_ISL_674357, EPI_ISL_674360 to 674362, EPI_ISL_674364, EPI_ISL_674366 to 674370, EPI_ISL_674372, EPI_ISL_674374 to 674376, EPI_ISL_674380, EPI_ISL_674383, EPI_ISL_674385, EPI_ISL_674389 to 674392, EPI_ISL_674394, EPI_ISL_674396 to 674397, EPI_ISL_674399 to 674400, EPI_ISL_674402,
EPI_ISL_674404, EPI_ISL_674406, EPI_ISL_674408 to 674409, EPI_ISL_674411 to 674413, EPI_ISL_674416, EPI_ISL_674418 to 674421, EPI_ISL_674424, EPI_ISL_674428 to 674429, EPI_ISL_674431 to 674432, EPI_ISL_674434 to 674436, EPI_ISL_674438 to 674439, EPI_ISL_674442, EPI_ISL_674445 to 674447,
EPI_ISL_674451 to 674452, EPI_ISL_674454 to 674456, EPI_ISL_674459, EPI_ISL_674462, EPI_ISL_674465 to 674467, EPI_ISL_674469 to 674472, EPI_ISL_674474, EPI_ISL_674476 to 674479, EPI_ISL_674484 to 674488, EPI_ISL_674490, EPI_ISL_674493, EPI_ISL_674495, EPI_ISL_675130, EPI_ISL_675133,
EPI_ISL_675135 to 675144, EPI_ISL_675146 to 675152, EPI_ISL_675154 to 675159, EPI_ISL_675161 to 675164, EPI_ISL_675166 to 675169, EPI_ISL_675171 to 675177, EPI_ISL_675180 to 675182, EPI_ISL_675184, EPI_ISL_675186 to 675187, EPI_ISL_675189 to 675209, EPI_ISL_675211, EPI_ISL_675213 to 675216,
EPI_ISL_675218, EPI_ISL_675220 to 675223, EPI_ISL_675225 to 675226, EPI_ISL_675229 to 675235, EPI_ISL_675237, EPI_ISL_675239 to 675240, EPI_ISL_675242, EPI_ISL_675245 to 675259, EPI_ISL_675261 to 675263, EPI_ISL_675265 to 675274, EPI_ISL_675278 to 675281, EPI_ISL_675283, EPI_ISL_675287 to 675291,
EPI_ISL_675293 to 675300, EPI_ISL_675302 to 675304, EPI_ISL_675310, EPI_ISL_675312, EPI_ISL_675314 to 675316, EPI_ISL_675321 to 675325, EPI_ISL_675327 to 675330, EPI_ISL_675333 to 675339, EPI_ISL_675341 to 675352, EPI_ISL_675354 to 675356, EPI_ISL_675358 to 675363, EPI_ISL_675365 to 675367,
EPI_ISL_675369 to 675370, EPI_ISL_675372 to 675385, EPI_ISL_675387 to 675388, EPI_ISL_675390 to 675392, EPI_ISL_675394 to 675396, EPI_ISL_675398, EPI_ISL_675400 to 675403, EPI_ISL_675405 to 675416, EPI_ISL_675418 to 675419, EPI_ISL_675421 to 675422, EPI_ISL_675425 to 675430, EPI_ISL_675433 to
675435, EPI_ISL_675437 to 675438, EPI_ISL_675440 to 675442, EPI_ISL_675445, EPI_ISL_675447 to 675461, EPI_ISL_675463 to 675466, EPI_ISL_675468 to 675471, EPI_ISL_675473 to 675474, EPI_ISL_675476 to 675479, EPI_ISL_675481 to 675484, EPI_ISL_675487 to 675489, EPI_ISL_675491 to 675494, EPI_ISL_675497
to 675498, EPI_ISL_675501 to 675502, EPI_ISL_675508 to 675512, EPI_ISL_675514 to 675517, EPI_ISL_675519 to 675527, EPI_ISL_675529 to 675532, EPI_ISL_675534, EPI_ISL_675536 to 675537, EPI_ISL_675539, EPI_ISL_675541, EPI_ISL_675543 to 675546, EPI_ISL_675548 to 675549, EPI_ISL_675551 to 675554,
EPI_ISL_675557, EPI_ISL_675559, EPI_ISL_675561 to 675563, EPI_ISL_675567 to 675568, EPI_ISL_675570 to 675571, EPI_ISL_675573 to 675575, EPI_ISL_675577, EPI_ISL_675579 to 675595, EPI_ISL_675597 to 675600, EPI_ISL_675603 to 675605, EPI_ISL_675607 to 675620, EPI_ISL_675622 to 675630, EPI_ISL_675632
to 675633, EPI_ISL_675635 to 675643, EPI_ISL_675645 to 675651, EPI_ISL_675653 to 675659, EPI_ISL_675662, EPI_ISL_675664 to 675666, EPI_ISL_675668 to 675675, EPI_ISL_675677 to 675681, EPI_ISL_675683 to 675689, EPI_ISL_675691 to 675692, EPI_ISL_675696 to 675697, EPI_ISL_675699 to 675703,
EPI_ISL_675705 to 675708, EPI_ISL_675710, EPI_ISL_675715 to 675716, EPI_ISL_675719 to 675721, EPI_ISL_675735, EPI_ISL_675739 to 675740, EPI_ISL_675746, EPI_ISL_675750 to 675751, EPI_ISL_675756 to 675757, EPI_ISL_675759 to 675760, EPI_ISL_675766, EPI_ISL_675770 to 675771, EPI_ISL_675774 to 675777,
EPI_ISL_675779, EPI_ISL_675785, EPI_ISL_675788, EPI_ISL_675794, EPI_ISL_675798 to 675800, EPI_ISL_675802 to 675803, EPI_ISL_675806, EPI_ISL_675810, EPI_ISL_675812 to 675813, EPI_ISL_675817 to 675818, EPI_ISL_675820, EPI_ISL_675822 to 675823, EPI_ISL_675825 to 675826, EPI_ISL_675829,
EPI_ISL_675833, EPI_ISL_675839, EPI_ISL_675847 to 675848, EPI_ISL_675852, EPI_ISL_675855, EPI_ISL_675868, EPI_ISL_675878, EPI_ISL_675880, EPI_ISL_675882, EPI_ISL_675890, EPI_ISL_675892, EPI_ISL_675894, EPI_ISL_675897, EPI_ISL_675901 to 675903, EPI_ISL_675908, EPI_ISL_675913, EPI_ISL_675916,
EPI_ISL_675920, EPI_ISL_675923 to 675925, EPI_ISL_675929 to 675930, EPI_ISL_675934, EPI_ISL_675937, EPI_ISL_675939, EPI_ISL_675942, EPI_ISL_675944, EPI_ISL_675952, EPI_ISL_675954, EPI_ISL_675962, EPI_ISL_675965, EPI_ISL_675968, EPI_ISL_675973 to 675974, EPI_ISL_675976, EPI_ISL_675978,
EPI_ISL_675980, EPI_ISL_675982, EPI_ISL_675986 to 675988, EPI_ISL_675993, EPI_ISL_676000, EPI_ISL_676002, EPI_ISL_676006, EPI_ISL_676010, EPI_ISL_681661 to 681662, EPI_ISL_681665, EPI_ISL_693332 to 693333, EPI_ISL_701673 to 701723, EPI_ISL_701725 to 701960, EPI_ISL_701962 to 702022,
EPI_ISL_702370 to 702384, EPI_ISL_702386 to 702410, EPI_ISL_702412 to 702437, EPI_ISL_702439 to 702463, EPI_ISL_702465 to 702468, EPI_ISL_702470, EPI_ISL_702472 to 702473, EPI_ISL_702475, EPI_ISL_702477, EPI_ISL_702479 to 702480, EPI_ISL_702482 to 702483, EPI_ISL_702485 to 702486, EPI_ISL_702488,
EPI_ISL_702490 to 702491, EPI_ISL_702493 to 702494, EPI_ISL_702496 to 702497, EPI_ISL_702499, EPI_ISL_702501 to 702503, EPI_ISL_702506, EPI_ISL_702508 to 702509, EPI_ISL_702511, EPI_ISL_702513 to 702514, EPI_ISL_702516 to 702517, EPI_ISL_702519, EPI_ISL_702521 to 702522, EPI_ISL_702524,
EPI_ISL_702526 to 702527, EPI_ISL_702529 to 702530, EPI_ISL_702532, EPI_ISL_702534 to 702535, EPI_ISL_702537 to 702538, EPI_ISL_702540, EPI_ISL_702542 to 702543, EPI_ISL_702545 to 702546, EPI_ISL_702548, EPI_ISL_702551 to 702552, EPI_ISL_702554 to 702555, EPI_ISL_702557, EPI_ISL_702559,
EPI_ISL_702563, EPI_ISL_702565, EPI_ISL_702567 to 702568, EPI_ISL_702570, EPI_ISL_702572 to 702573, EPI_ISL_702575 to 702576, EPI_ISL_702578, EPI_ISL_702580 to 702581, EPI_ISL_702583 to 702584, EPI_ISL_702586, EPI_ISL_702588 to 702589, EPI_ISL_702592 to 702593, EPI_ISL_702595 to 702596,
EPI_ISL_702598 to 702599, EPI_ISL_702601, EPI_ISL_702603 to 702604, EPI_ISL_702606, EPI_ISL_702608 to 702609, EPI_ISL_702611, EPI_ISL_702613 to 702614, EPI_ISL_702616 to 702617, EPI_ISL_702619, EPI_ISL_702621 to 702622, EPI_ISL_702624 to 702625, EPI_ISL_702627, EPI_ISL_702629 to 702630,
EPI_ISL_702632, EPI_ISL_702634 to 702635, EPI_ISL_702637 to 702638, EPI_ISL_702640 to 702641, EPI_ISL_702643, EPI_ISL_702645 to 702646, EPI_ISL_702648, EPI_ISL_702650 to 702651, EPI_ISL_702653 to 702654, EPI_ISL_702657 to 702658, EPI_ISL_702660, EPI_ISL_702662 to 702663, EPI_ISL_702665 to 702666,
EPI_ISL_702668, EPI_ISL_702670 to 702671, EPI_ISL_702673, EPI_ISL_702675 to 702676, EPI_ISL_702678, EPI_ISL_702680 to 702681, EPI_ISL_702683, EPI_ISL_702685 to 702686, EPI_ISL_702688 to 702689, EPI_ISL_702691, EPI_ISL_702693, EPI_ISL_702695 to 702696, EPI_ISL_702699, EPI_ISL_702701 to 702702,
EPI_ISL_702704, EPI_ISL_702706 to 702707, EPI_ISL_702709, EPI_ISL_702711 to 702712, EPI_ISL_702714 to 702715, EPI_ISL_702717, EPI_ISL_702719 to 702720, EPI_ISL_702722, EPI_ISL_702724 to 702725, EPI_ISL_702727 to 702728, EPI_ISL_702730, EPI_ISL_702732 to 702733, EPI_ISL_702735, EPI_ISL_702737 to
702738, EPI_ISL_702740 to 702741, EPI_ISL_702743, EPI_ISL_702745 to 702746, EPI_ISL_702748, EPI_ISL_702750, EPI_ISL_702752, EPI_ISL_702754 to 702755, EPI_ISL_702757, EPI_ISL_702759 to 702760, EPI_ISL_702762, EPI_ISL_702764 to 702765, EPI_ISL_702767 to 702768, EPI_ISL_702770, EPI_ISL_702772 to
702773, EPI_ISL_702775, EPI_ISL_702777 to 702778, EPI_ISL_702780 to 702781, EPI_ISL_702783, EPI_ISL_702785 to 702786, EPI_ISL_702788 to 702789, EPI_ISL_702791, EPI_ISL_702793, EPI_ISL_702796 to 702797, EPI_ISL_702799 to 702800, EPI_ISL_702802, EPI_ISL_702804 to 702805, EPI_ISL_702807 to 702808,
EPI_ISL_702810 to 702811, EPI_ISL_702813 to 702814, EPI_ISL_702816, EPI_ISL_702818 to 702819, EPI_ISL_702821 to 702822, EPI_ISL_702898 to 702899, EPI_ISL_702901, EPI_ISL_702903 to 702904, EPI_ISL_702906, EPI_ISL_702908 to 702909, EPI_ISL_702912, EPI_ISL_702914, EPI_ISL_702916, EPI_ISL_702918 to
702919, EPI_ISL_702921, EPI_ISL_702923 to 702924, EPI_ISL_702926 to 702927, EPI_ISL_702929, EPI_ISL_702931, EPI_ISL_702933 to 702934, EPI_ISL_702936, EPI_ISL_702938, EPI_ISL_702940, EPI_ISL_702942 to 702943, EPI_ISL_702945 to 702946, EPI_ISL_702948, EPI_ISL_702951 to 702952, EPI_ISL_702954 to
702955, EPI_ISL_702957 to 702958, EPI_ISL_702960, EPI_ISL_702962 to 702963, EPI_ISL_702965, EPI_ISL_702967 to 702968, EPI_ISL_702970, EPI_ISL_702972 to 702973, EPI_ISL_702975 to 702976, EPI_ISL_702978 to 702979, EPI_ISL_702981, EPI_ISL_702983 to 702984, EPI_ISL_702986, EPI_ISL_702988 to 702989,
EPI_ISL_702991, EPI_ISL_702993, EPI_ISL_702995, EPI_ISL_702997 to 702998, EPI_ISL_703000 to 703001, EPI_ISL_703003 to 703004, EPI_ISL_703006, EPI_ISL_703008 to 703009, EPI_ISL_703011 to 703012, EPI_ISL_703014, EPI_ISL_703016 to 703017, EPI_ISL_703019, EPI_ISL_703021 to 703022, EPI_ISL_703024 to
703025, EPI_ISL_703027 to 703028, EPI_ISL_703030 to 703031, EPI_ISL_703033, EPI_ISL_703035, EPI_ISL_703037, EPI_ISL_703039, EPI_ISL_703041 to 703042, EPI_ISL_703044 to 703045, EPI_ISL_703047, EPI_ISL_703049 to 703050, EPI_ISL_703052, EPI_ISL_703054 to 703055, EPI_ISL_703057 to 703058,
EPI_ISL_703060, EPI_ISL_703062 to 703063, EPI_ISL_703065, EPI_ISL_703067 to 703068, EPI_ISL_703070, EPI_ISL_703072 to 703073, EPI_ISL_703075, EPI_ISL_703078 to 703079, EPI_ISL_703081, EPI_ISL_703083 to 703084, EPI_ISL_703086 to 703087, EPI_ISL_703089, EPI_ISL_703091 to 703092, EPI_ISL_703094 to
703095, EPI_ISL_703097 to 703098, EPI_ISL_703100 to 703101, EPI_ISL_703103 to 703104, EPI_ISL_703106, EPI_ISL_703108 to 703109, EPI_ISL_703111 to 703112, EPI_ISL_703114, EPI_ISL_703116 to 703117, EPI_ISL_703119 to 703120, EPI_ISL_703122 to 703123, EPI_ISL_703125 to 703126, EPI_ISL_703128,
EPI_ISL_703130 to 703131, EPI_ISL_703134 to 703135, EPI_ISL_703137 to 703138, EPI_ISL_703140, EPI_ISL_703142 to 703143, EPI_ISL_703145 to 703146, EPI_ISL_703148 to 703149, EPI_ISL_703151, EPI_ISL_703153 to 703154, EPI_ISL_703156 to 703157, EPI_ISL_703159 to 703161, EPI_ISL_703163 to 703164,
EPI_ISL_703166, EPI_ISL_703168 to 703169, EPI_ISL_703171, EPI_ISL_703173, EPI_ISL_703175, EPI_ISL_703177 to 703178, EPI_ISL_703180 to 703181, EPI_ISL_703183, EPI_ISL_703185 to 703186, EPI_ISL_703188 to 703189, EPI_ISL_703191, EPI_ISL_703193 to 703194, EPI_ISL_703196 to 703197, EPI_ISL_703199,
EPI_ISL_703201 to 703202, EPI_ISL_703204 to 703205, EPI_ISL_703207, EPI_ISL_703209 to 703210, EPI_ISL_703212, EPI_ISL_703214 to 703215, EPI_ISL_703217 to 703218, EPI_ISL_703220 to 703221, EPI_ISL_703223 to 703224, EPI_ISL_703226, EPI_ISL_703228 to 703229, EPI_ISL_703231 to 703232, EPI_ISL_703234,
EPI_ISL_703236 to 703237, EPI_ISL_703239, EPI_ISL_703241 to 703242, EPI_ISL_703244 to 703245, EPI_ISL_703247 to 703248, EPI_ISL_703250, EPI_ISL_703252 to 703253, EPI_ISL_703255, EPI_ISL_703257 to 703258, EPI_ISL_703260, EPI_ISL_703265 to 703266, EPI_ISL_703268, EPI_ISL_703271, EPI_ISL_703274,
EPI_ISL_703276 to 703277, EPI_ISL_703282, EPI_ISL_703293 to 703294, EPI_ISL_703298, EPI_ISL_703301, EPI_ISL_703306 to 703307, EPI_ISL_703311 to 703312, EPI_ISL_703314, EPI_ISL_703317 to 703318, EPI_ISL_703322, EPI_ISL_703326, EPI_ISL_703328 to 703329, EPI_ISL_703333, EPI_ISL_703336,
EPI_ISL_703339, EPI_ISL_703341 to 703342, EPI_ISL_703344, EPI_ISL_703347, EPI_ISL_703357, EPI_ISL_703363, EPI_ISL_703365, EPI_ISL_703368, EPI_ISL_703373 to 703374, EPI_ISL_703376, EPI_ISL_703386, EPI_ISL_703388, EPI_ISL_703394, EPI_ISL_703396 to 703397, EPI_ISL_703402, EPI_ISL_703404 to
703405, EPI_ISL_703413, EPI_ISL_703416, EPI_ISL_703418, EPI_ISL_703420, EPI_ISL_703425, EPI_ISL_703433 to 703434, EPI_ISL_703436 to 703437, EPI_ISL_703441, EPI_ISL_703448, EPI_ISL_703450, EPI_ISL_703452, EPI_ISL_703455, EPI_ISL_703460 to 703461, EPI_ISL_703464, EPI_ISL_703467, EPI_ISL_703470,
EPI_ISL_703475, EPI_ISL_703477 to 703478, EPI_ISL_703480, EPI_ISL_703483, EPI_ISL_703485, EPI_ISL_703488, EPI_ISL_703490, EPI_ISL_703493 to 703494, EPI_ISL_703498, EPI_ISL_703503 to 703504, EPI_ISL_703507, EPI_ISL_703509, EPI_ISL_703518, EPI_ISL_703520, EPI_ISL_703523, EPI_ISL_703526,
EPI_ISL_703528, EPI_ISL_703531 to 703532, EPI_ISL_703536, EPI_ISL_703539, EPI_ISL_703543 to 703544, EPI_ISL_703547, EPI_ISL_703549, EPI_ISL_703552, EPI_ISL_703555, EPI_ISL_703558, EPI_ISL_703563, EPI_ISL_703565 to 703566, EPI_ISL_703568, EPI_ISL_703570 to 703571, EPI_ISL_703573 to 703574,
EPI_ISL_703576, EPI_ISL_703580 to 703581, EPI_ISL_703586, EPI_ISL_703588 to 703589, EPI_ISL_703591 to 703592, EPI_ISL_703594 to 703595, EPI_ISL_703597, EPI_ISL_703602 to 703603, EPI_ISL_703605, EPI_ISL_703613, EPI_ISL_703615, EPI_ISL_703618, EPI_ISL_703620, EPI_ISL_703623, EPI_ISL_703625,
EPI_ISL_703628, EPI_ISL_703630, EPI_ISL_703637 to 703638, EPI_ISL_703646, EPI_ISL_703648, EPI_ISL_703662, EPI_ISL_703664 to 703665, EPI_ISL_703667, EPI_ISL_703670, EPI_ISL_703675, EPI_ISL_703678, EPI_ISL_703680 to 703681, EPI_ISL_703683, EPI_ISL_703686, EPI_ISL_703688 to 703689,
EPI_ISL_703691 to 703692, EPI_ISL_703696, EPI_ISL_703699, EPI_ISL_703702, EPI_ISL_703705, EPI_ISL_703707, EPI_ISL_703777 to 703778, EPI_ISL_703780 to 703781, EPI_ISL_703783, EPI_ISL_703785, EPI_ISL_703788, EPI_ISL_703791, EPI_ISL_703793, EPI_ISL_703795 to 703796, EPI_ISL_703798,
EPI_ISL_703803 to 703804, EPI_ISL_703809, EPI_ISL_703812, EPI_ISL_703814, EPI_ISL_703817, EPI_ISL_708841 to 708842, EPI_ISL_708845 to 708846, EPI_ISL_708851 to 708854, EPI_ISL_708859 to 708861, EPI_ISL_708863 to 708868, EPI_ISL_708870, EPI_ISL_708872 to 708878, EPI_ISL_708880, EPI_ISL_708882,
EPI_ISL_708885 to 708888, EPI_ISL_708891 to 708892, EPI_ISL_708894 to 708895, EPI_ISL_708899, EPI_ISL_708902 to 708903, EPI_ISL_708907, EPI_ISL_708909, EPI_ISL_708911, EPI_ISL_708914 to 708915, EPI_ISL_708917 to 708919, EPI_ISL_708923 to 708924, EPI_ISL_708926 to 708927, EPI_ISL_708929,
EPI_ISL_708931 to 708932, EPI_ISL_708934 to 708940, EPI_ISL_708942 to 708943, EPI_ISL_708946 to 708947, EPI_ISL_708949 to 708950, EPI_ISL_708952, EPI_ISL_708954 to 708956, EPI_ISL_708959 to 708962, EPI_ISL_708965 to 708968, EPI_ISL_708970 to 708974, EPI_ISL_708976 to 708978, EPI_ISL_708980 to
708982, EPI_ISL_708987 to 708988, EPI_ISL_709295 to 709300, EPI_ISL_709302 to 709306, EPI_ISL_709308 to 709310, EPI_ISL_709313, EPI_ISL_709315 to 709319, EPI_ISL_709321 to 709323, EPI_ISL_709325 to 709326, EPI_ISL_709328 to 709331, EPI_ISL_709333, EPI_ISL_709335, EPI_ISL_709337 to 709340,
EPI_ISL_709342, EPI_ISL_709344, EPI_ISL_709346 to 709352, EPI_ISL_709355 to 709357, EPI_ISL_709361 to 709363, EPI_ISL_709368 to 709372, EPI_ISL_709374 to 709375, EPI_ISL_709377, EPI_ISL_709379, EPI_ISL_709381 to 709382, EPI_ISL_709385 to 709387, EPI_ISL_709389 to 709391, EPI_ISL_709393,
EPI_ISL_709397 to 709398, EPI_ISL_709400 to 709402, EPI_ISL_709405, EPI_ISL_709408, EPI_ISL_709410, EPI_ISL_709413 to 709416, EPI_ISL_709418, EPI_ISL_709420 to 709421, EPI_ISL_709425, EPI_ISL_709427 to 709428, EPI_ISL_709430 to 709435, EPI_ISL_709437, EPI_ISL_709442, EPI_ISL_709444 to 709445,
EPI_ISL_709447 to 709449, EPI_ISL_709451 to 709452, EPI_ISL_709455, EPI_ISL_709460 to 709461, EPI_ISL_709463, EPI_ISL_709467 to 709468, EPI_ISL_709470 to 709471, EPI_ISL_709473, EPI_ISL_709475 to 709480, EPI_ISL_709484 to 709485, EPI_ISL_709488 to 709489, EPI_ISL_709491 to 709492, EPI_ISL_709499,
EPI_ISL_709501, EPI_ISL_709504 to 709508, EPI_ISL_709512 to 709514, EPI_ISL_709516, EPI_ISL_709519 to 709521, EPI_ISL_709523, EPI_ISL_709526, EPI_ISL_709529, EPI_ISL_709531 to 709532, EPI_ISL_709534 to 709536, EPI_ISL_709538, EPI_ISL_709543 to 709548, EPI_ISL_709550 to 709554, EPI_ISL_709559 to
709560, EPI_ISL_709562 to 709564, EPI_ISL_709566, EPI_ISL_710614, EPI_ISL_710616 to 710618, EPI_ISL_710620, EPI_ISL_710625, EPI_ISL_710628, EPI_ISL_710631 to 710632, EPI_ISL_710636, EPI_ISL_710638, EPI_ISL_710645, EPI_ISL_710647 to 710648, EPI_ISL_710656, EPI_ISL_710661 to 710663,
EPI_ISL_710665, EPI_ISL_710671, EPI_ISL_710676, EPI_ISL_710682, EPI_ISL_710689, EPI_ISL_710693 to 710694, EPI_ISL_710698 to 710747, EPI_ISL_710749 to 710752, EPI_ISL_710837, EPI_ISL_710865 to 710866, EPI_ISL_710869 to 710871, EPI_ISL_710873 to 710876, EPI_ISL_710887 to 710902, EPI_ISL_710908 to
710994, EPI_ISL_711008 to 711013, EPI_ISL_711015 to 711022, EPI_ISL_711027 to 711037, EPI_ISL_711039 to 711047, EPI_ISL_718629, EPI_ISL_718631, EPI_ISL_718633, EPI_ISL_718635, EPI_ISL_718637, EPI_ISL_718639 to 718641, EPI_ISL_718643 to 718644, EPI_ISL_718647, EPI_ISL_718650, EPI_ISL_718653,
EPI_ISL_718657 to 718658, EPI_ISL_718662 to 718666, EPI_ISL_718670, EPI_ISL_718672, EPI_ISL_718674 to 718676, EPI_ISL_718678, EPI_ISL_718682, EPI_ISL_718686 to 718687, EPI_ISL_718691, EPI_ISL_718694 to 718695, EPI_ISL_718700, EPI_ISL_718705, EPI_ISL_718707, EPI_ISL_718710 to 718716,
EPI_ISL_718718 to 718719, EPI_ISL_718721, EPI_ISL_718723 to 718725, EPI_ISL_718728 to 718729, EPI_ISL_718735, EPI_ISL_718738 to 718740, EPI_ISL_718742, EPI_ISL_718744 to 718747, EPI_ISL_718750, EPI_ISL_718754 to 718755, EPI_ISL_718759, EPI_ISL_718761 to 718762, EPI_ISL_718764 to 718767,
EPI_ISL_718769, EPI_ISL_718772 to 718774, EPI_ISL_718777, EPI_ISL_718779 to 718783, EPI_ISL_718787, EPI_ISL_718789, EPI_ISL_718791, EPI_ISL_718795 to 718796, EPI_ISL_718798 to 718799, EPI_ISL_718801 to 718803, EPI_ISL_718806 to 718808, EPI_ISL_718811 to 718813, EPI_ISL_718819, EPI_ISL_718821,
EPI_ISL_718823, EPI_ISL_718827 to 718833, EPI_ISL_718837 to 718839, EPI_ISL_718841, EPI_ISL_718844, EPI_ISL_718847, EPI_ISL_718849 to 718850, EPI_ISL_718853 to 718854, EPI_ISL_718858 to 718859, EPI_ISL_718864 to 718866, EPI_ISL_718870 to 718873, EPI_ISL_718877 to 718878, EPI_ISL_718880 to 718881,
EPI_ISL_718885 to 718891, EPI_ISL_718894 to 718895, EPI_ISL_718901 to 718903, EPI_ISL_718907 to 718908, EPI_ISL_718911, EPI_ISL_718914 to 718915, EPI_ISL_718917, EPI_ISL_718919, EPI_ISL_718922 to 718923, EPI_ISL_718926, EPI_ISL_718929 to 718930, EPI_ISL_718932, EPI_ISL_718935 to 718936,
EPI_ISL_718939, EPI_ISL_718943, EPI_ISL_718945, EPI_ISL_718950, EPI_ISL_718952 to 718953, EPI_ISL_718955, EPI_ISL_718957, EPI_ISL_718959, EPI_ISL_718962 to 718964, EPI_ISL_718966 to 718967, EPI_ISL_718970, EPI_ISL_718974 to 718979, EPI_ISL_720683, EPI_ISL_720685 to 720686, EPI_ISL_720688,
EPI_ISL_720694 to 720695, EPI_ISL_720697, EPI_ISL_720702, EPI_ISL_720709, EPI_ISL_720711, EPI_ISL_720713, EPI_ISL_720715, EPI_ISL_720722, EPI_ISL_720734, EPI_ISL_720741, EPI_ISL_720745, EPI_ISL_720750, EPI_ISL_720756, EPI_ISL_720762, EPI_ISL_720764 to 720765, EPI_ISL_720770, EPI_ISL_720773,
EPI_ISL_720779 to 720780, EPI_ISL_720790, EPI_ISL_720796, EPI_ISL_720810, EPI_ISL_720814, EPI_ISL_720816, EPI_ISL_720823, EPI_ISL_720825, EPI_ISL_720827, EPI_ISL_720836, EPI_ISL_720842, EPI_ISL_720845 to 720846, EPI_ISL_720848 to 720849, EPI_ISL_720853, EPI_ISL_720857 to 720858,
EPI_ISL_720864, EPI_ISL_720869, EPI_ISL_720872, EPI_ISL_720875, EPI_ISL_720880, EPI_ISL_720885 to 720886, EPI_ISL_720891, EPI_ISL_720893 to 720894, EPI_ISL_720898, EPI_ISL_720900, EPI_ISL_720907, EPI_ISL_721185 to 721518, EPI_ISL_721520, EPI_ISL_721522 to 721523, EPI_ISL_721525,
EPI_ISL_730654 to 730655, EPI_ISL_730658, EPI_ISL_730660 to 730663, EPI_ISL_730665 to 730672, EPI_ISL_730674, EPI_ISL_730677, EPI_ISL_730679 to 730684, EPI_ISL_730686 to 730696, EPI_ISL_730698 to 730704, EPI_ISL_730706 to 730708, EPI_ISL_730711 to 730717, EPI_ISL_730719 to 730722, EPI_ISL_730724
to 730736, EPI_ISL_730738 to 730751, EPI_ISL_730753 to 730758, EPI_ISL_730760 to 730764, EPI_ISL_730766 to 730776, EPI_ISL_730779, EPI_ISL_730781 to 730784, EPI_ISL_730786 to 730788, EPI_ISL_730790 to 730792, EPI_ISL_730794, EPI_ISL_730797 to 730800, EPI_ISL_730807, EPI_ISL_730810 to 730812,
EPI_ISL_730814, EPI_ISL_730816 to 730823, EPI_ISL_730825 to 730827, EPI_ISL_730829 to 730830, EPI_ISL_730834 to 730839, EPI_ISL_730841 to 730845, EPI_ISL_730848, EPI_ISL_730851 to 730857, EPI_ISL_730859, EPI_ISL_730861, EPI_ISL_730863, EPI_ISL_730865 to 730866, EPI_ISL_730868, EPI_ISL_730870 to

730874, EPI_ISL_730878 to 730886, EPI_ISL_730888, EPI_ISL_730890 to 730893, EPI_ISL_730895 to 730897, EPI_ISL_730899 to 730907, EPI_ISL_730909, EPI_ISL_730912, EPI_ISL_730914 to 731250, EPI_ISL_731254, EPI_ISL_731260, EPI_ISL_731263, EPI_ISL_731265 to 731266, EPI_ISL_731268 to 731270,
EPI_ISL_731272, EPI_ISL_731274 to 731279, EPI_ISL_731285, EPI_ISL_731288 to 731289, EPI_ISL_731292, EPI_ISL_731294, EPI_ISL_731296, EPI_ISL_731298, EPI_ISL_731300 to 731304, EPI_ISL_731307, EPI_ISL_731311 to 731313, EPI_ISL_731315 to 731318, EPI_ISL_731323 to 731325, EPI_ISL_731328 to 731329,
EPI_ISL_731331, EPI_ISL_731335 to 731336, EPI_ISL_731339, EPI_ISL_731342 to 731344, EPI_ISL_731348, EPI_ISL_731350 to 731352, EPI_ISL_731356, EPI_ISL_731358 to 731360, EPI_ISL_731362 to 731366, EPI_ISL_731369 to 731371, EPI_ISL_731374 to 731375, EPI_ISL_731377, EPI_ISL_731381, EPI_ISL_731384 to
731385, EPI_ISL_731387 to 731389, EPI_ISL_731391, EPI_ISL_731394 to 731395, EPI_ISL_731399, EPI_ISL_731409 to 731410, EPI_ISL_731412 to 731413, EPI_ISL_731415 to 731416, EPI_ISL_731418 to 731419, EPI_ISL_731422 to 731424, EPI_ISL_731427 to 731428, EPI_ISL_731430, EPI_ISL_731434, EPI_ISL_731436,
EPI_ISL_731439 to 731443, EPI_ISL_731445 to 731446, EPI_ISL_731448, EPI_ISL_731451, EPI_ISL_731455, EPI_ISL_731457, EPI_ISL_731460 to 731465, EPI_ISL_731467 to 731468, EPI_ISL_731470, EPI_ISL_731473 to 731475, EPI_ISL_731478, EPI_ISL_731480 to 731481, EPI_ISL_731483, EPI_ISL_731487,
EPI_ISL_731489 to 731490, EPI_ISL_731492, EPI_ISL_731495, EPI_ISL_731498, EPI_ISL_731500, EPI_ISL_731502 to 731504, EPI_ISL_731506, EPI_ISL_731509 to 731512, EPI_ISL_731514 to 731516, EPI_ISL_731518, EPI_ISL_731520 to 731521, EPI_ISL_731524 to 731534, EPI_ISL_731538 to 731540, EPI_ISL_731542 to
731543, EPI_ISL_731545 to 731547, EPI_ISL_731549, EPI_ISL_731552 to 731890, EPI_ISL_733598, EPI_ISL_733603, EPI_ISL_733605, EPI_ISL_733609, EPI_ISL_733611, EPI_ISL_733613, EPI_ISL_733618, EPI_ISL_733621, EPI_ISL_733623, EPI_ISL_733625, EPI_ISL_733635, EPI_ISL_733638 to 733639, EPI_ISL_733641
to 733643, EPI_ISL_733647, EPI_ISL_733653 to 733654, EPI_ISL_733659, EPI_ISL_733682 to 733683, EPI_ISL_733685, EPI_ISL_733693, EPI_ISL_733697, EPI_ISL_733699, EPI_ISL_733701, EPI_ISL_733703, EPI_ISL_733706, EPI_ISL_733710, EPI_ISL_733712, EPI_ISL_733718, EPI_ISL_733725, EPI_ISL_733731,
EPI_ISL_733734, EPI_ISL_733737, EPI_ISL_733742, EPI_ISL_733754 to 733755, EPI_ISL_733759, EPI_ISL_733762, EPI_ISL_733766, EPI_ISL_733769, EPI_ISL_733777, EPI_ISL_733783, EPI_ISL_733785, EPI_ISL_733794 to 733795, EPI_ISL_733797, EPI_ISL_733803, EPI_ISL_733806 to 733808, EPI_ISL_733815,
EPI_ISL_733818 to 733819, EPI_ISL_733830, EPI_ISL_733833, EPI_ISL_733837, EPI_ISL_733843 to 733844, EPI_ISL_733848, EPI_ISL_733850, EPI_ISL_733859, EPI_ISL_733867, EPI_ISL_733877, EPI_ISL_733879 to 733880, EPI_ISL_733882, EPI_ISL_733885, EPI_ISL_733897, EPI_ISL_733901, EPI_ISL_733912 to
733913, EPI_ISL_735860, EPI_ISL_735862 to 735863, EPI_ISL_735865 to 735877, EPI_ISL_735879, EPI_ISL_735881 to 735885, EPI_ISL_735887 to 735893, EPI_ISL_735895 to 735908, EPI_ISL_735910 to 735919, EPI_ISL_735921 to 735926, EPI_ISL_735928 to 735936, EPI_ISL_735938, EPI_ISL_735940 to 735944,
EPI_ISL_735946 to 735948, EPI_ISL_735950 to 735951, EPI_ISL_735953 to 735955, EPI_ISL_735957 to 735960, EPI_ISL_735962 to 735964, EPI_ISL_735966 to 735967, EPI_ISL_735969 to 735971, EPI_ISL_735974 to 735977, EPI_ISL_735979 to 735981, EPI_ISL_735983 to 735986, EPI_ISL_735988 to 735992,
EPI_ISL_735994, EPI_ISL_735997 to 736000, EPI_ISL_736002, EPI_ISL_736004 to 736006, EPI_ISL_736008 to 736014, EPI_ISL_736016, EPI_ISL_736020 to 736023, EPI_ISL_736026, EPI_ISL_736028 to 736032, EPI_ISL_736034 to 736037, EPI_ISL_736039 to 736040, EPI_ISL_736042 to 736051, EPI_ISL_736053 to 736056,
EPI_ISL_736058, EPI_ISL_736060 to 736063, EPI_ISL_736065, EPI_ISL_736067 to 736071, EPI_ISL_736073 to 736089, EPI_ISL_736093 to 736095, EPI_ISL_736097 to 736098, EPI_ISL_736103, EPI_ISL_736105 to 736106, EPI_ISL_736108 to 736114, EPI_ISL_736116 to 736123, EPI_ISL_736125 to 736129, EPI_ISL_736133
to 736134, EPI_ISL_736136 to 736142, EPI_ISL_736144 to 736145, EPI_ISL_736147 to 736148, EPI_ISL_736150, EPI_ISL_736152 to 736158, EPI_ISL_736160 to 736161, EPI_ISL_736163 to 736166, EPI_ISL_736170 to 736174, EPI_ISL_736176 to 736181, EPI_ISL_736185 to 736189, EPI_ISL_736192 to 736200,
EPI_ISL_736481 to 736482, EPI_ISL_736484 to 736486, EPI_ISL_736493 to 736496, EPI_ISL_736498 to 736500, EPI_ISL_736505 to 736507, EPI_ISL_736509 to 736511, EPI_ISL_736513, EPI_ISL_736516 to 736518, EPI_ISL_736520 to 736522, EPI_ISL_736524, EPI_ISL_736526, EPI_ISL_736530 to 736531, EPI_ISL_736534
to 736535, EPI_ISL_736537, EPI_ISL_736539 to 736541, EPI_ISL_736543 to 736546, EPI_ISL_736548, EPI_ISL_736553 to 736555, EPI_ISL_736557, EPI_ISL_736559 to 736562, EPI_ISL_736564, EPI_ISL_736566, EPI_ISL_736568 to 736570, EPI_ISL_736572, EPI_ISL_736574 to 736577, EPI_ISL_736579, EPI_ISL_736581,
EPI_ISL_736583 to 736585, EPI_ISL_736587, EPI_ISL_736589, EPI_ISL_736591 to 736592, EPI_ISL_736595 to 736596, EPI_ISL_736599 to 736600, EPI_ISL_736603 to 736604, EPI_ISL_736606 to 736607, EPI_ISL_736611 to 736616, EPI_ISL_736619, EPI_ISL_736621, EPI_ISL_736623, EPI_ISL_736625 to 736626,
EPI_ISL_736629, EPI_ISL_736632, EPI_ISL_736634 to 736636, EPI_ISL_736638 to 736642, EPI_ISL_736644 to 736645, EPI_ISL_736647, EPI_ISL_736649, EPI_ISL_736651 to 736656, EPI_ISL_736658, EPI_ISL_736660 to 736667, EPI_ISL_736669 to 736671, EPI_ISL_736673 to 736674, EPI_ISL_736676, EPI_ISL_736679 to
736680, EPI_ISL_736684, EPI_ISL_736687 to 736688, EPI_ISL_736690, EPI_ISL_736692, EPI_ISL_736698, EPI_ISL_736701 to 736702, EPI_ISL_736704 to 736711, EPI_ISL_736714 to 736715, EPI_ISL_736717 to 736718, EPI_ISL_736720, EPI_ISL_736722, EPI_ISL_736726, EPI_ISL_736729 to 736730, EPI_ISL_736732,
EPI_ISL_736735, EPI_ISL_736739, EPI_ISL_736741 to 736742, EPI_ISL_736747 to 736748, EPI_ISL_736750 to 736751, EPI_ISL_736753, EPI_ISL_736755 to 736756, EPI_ISL_736758 to 736773, EPI_ISL_756378, EPI_ISL_756381 to 756383, EPI_ISL_756385, EPI_ISL_756387 to 756388, EPI_ISL_756390 to 756391,
EPI_ISL_756394, EPI_ISL_756396 to 756400, EPI_ISL_756402, EPI_ISL_756404 to 756408, EPI_ISL_756410 to 756412, EPI_ISL_756414, EPI_ISL_756421, EPI_ISL_756423, EPI_ISL_756425, EPI_ISL_756427 to 756428, EPI_ISL_756430, EPI_ISL_756433, EPI_ISL_756435 to 756437, EPI_ISL_756439 to 756441,
EPI_ISL_756443, EPI_ISL_756445, EPI_ISL_756447, EPI_ISL_756449 to 756452, EPI_ISL_756455, EPI_ISL_756457 to 756459, EPI_ISL_756461 to 756466, EPI_ISL_756468, EPI_ISL_756470 to 756473, EPI_ISL_756476 to 756477, EPI_ISL_756479, EPI_ISL_756482 to 756483, EPI_ISL_756488 to 756489, EPI_ISL_756493 to
756495, EPI_ISL_756503 to 756504, EPI_ISL_756509 to 756510, EPI_ISL_756514 to 756516, EPI_ISL_756520, EPI_ISL_756523 to 756524, EPI_ISL_756526 to 756527, EPI_ISL_756530 to 756533, EPI_ISL_756536 to 756539, EPI_ISL_756541, EPI_ISL_756544, EPI_ISL_756546 to 756548, EPI_ISL_756550 to 756552,
EPI_ISL_756555 to 756557, EPI_ISL_756560 to 756563, EPI_ISL_756565 to 756566, EPI_ISL_756569, EPI_ISL_756571, EPI_ISL_756573 to 756575, EPI_ISL_756578, EPI_ISL_756580 to 756581, EPI_ISL_756583, EPI_ISL_756587, EPI_ISL_756592 to 756593, EPI_ISL_756596, EPI_ISL_756598 to 756600, EPI_ISL_756602 to
756603, EPI_ISL_756609, EPI_ISL_756612, EPI_ISL_756616, EPI_ISL_756618 to 756620, EPI_ISL_756624, EPI_ISL_756626, EPI_ISL_756632, EPI_ISL_756634 to 756636, EPI_ISL_756638 to 756641, EPI_ISL_756644 to 756648, EPI_ISL_756650 to 756652, EPI_ISL_756654, EPI_ISL_756656 to 756658, EPI_ISL_756661 to
756664, EPI_ISL_756669 to 756670, EPI_ISL_756673, EPI_ISL_756681 to 756688, EPI_ISL_760249, EPI_ISL_760259, EPI_ISL_760261, EPI_ISL_760263, EPI_ISL_760266 to 760268, EPI_ISL_760273, EPI_ISL_760276, EPI_ISL_760278, EPI_ISL_760281, EPI_ISL_760284 to 760286, EPI_ISL_760288, EPI_ISL_760293,
EPI_ISL_760295 to 760296, EPI_ISL_760301, EPI_ISL_760305 to 760306, EPI_ISL_760311, EPI_ISL_760326, EPI_ISL_760333, EPI_ISL_760341, EPI_ISL_760344, EPI_ISL_760346, EPI_ISL_760350, EPI_ISL_760358 to 760360, EPI_ISL_760364, EPI_ISL_760369 to 760372, EPI_ISL_760381, EPI_ISL_760386 to 760387,
EPI_ISL_760389 to 760390, EPI_ISL_760392, EPI_ISL_760397, EPI_ISL_760405, EPI_ISL_760408, EPI_ISL_760415, EPI_ISL_760418 to 760419, EPI_ISL_760421, EPI_ISL_760424, EPI_ISL_760430, EPI_ISL_760432, EPI_ISL_760434, EPI_ISL_760436, EPI_ISL_760441 to 760442, EPI_ISL_760445, EPI_ISL_760448,
EPI_ISL_760450, EPI_ISL_760460 to 760461, EPI_ISL_760471, EPI_ISL_760474 to 760475, EPI_ISL_760485 to 760486, EPI_ISL_760491, EPI_ISL_760495, EPI_ISL_760498, EPI_ISL_760500 to 760501, EPI_ISL_760508 to 760509, EPI_ISL_760512, EPI_ISL_760516, EPI_ISL_760521, EPI_ISL_760524, EPI_ISL_760530 to
760531, EPI_ISL_760534 to 760535, EPI_ISL_760538, EPI_ISL_760543 to 760546, EPI_ISL_760549, EPI_ISL_760551 to 760552, EPI_ISL_760555, EPI_ISL_760557 to 760559, EPI_ISL_760561 to 760562, EPI_ISL_760564 to 760565, EPI_ISL_760567, EPI_ISL_760570, EPI_ISL_760572, EPI_ISL_760574, EPI_ISL_760582,
EPI_ISL_760586 to 760589, EPI_ISL_760591, EPI_ISL_760593, EPI_ISL_760595 to 760597, EPI_ISL_760599 to 760601, EPI_ISL_760603, EPI_ISL_760608 to 760609, EPI_ISL_760612 to 760613, EPI_ISL_760615, EPI_ISL_760617, EPI_ISL_760623 to 760625, EPI_ISL_760627 to 760628, EPI_ISL_760633, EPI_ISL_760637,
EPI_ISL_760639 to 760640, EPI_ISL_760644, EPI_ISL_760647, EPI_ISL_760649 to 760650, EPI_ISL_760652, EPI_ISL_760654, EPI_ISL_760656, EPI_ISL_760660 to 760661, EPI_ISL_760663 to 760667, EPI_ISL_760670, EPI_ISL_760677, EPI_ISL_760679 to 760680, EPI_ISL_760684 to 760685, EPI_ISL_760688,
EPI_ISL_760691, EPI_ISL_760693, EPI_ISL_760697, EPI_ISL_760699, EPI_ISL_760701 to 760702, EPI_ISL_760705, EPI_ISL_760708, EPI_ISL_760710 to 760711, EPI_ISL_760714 to 760715, EPI_ISL_760719, EPI_ISL_760721 to 760724, EPI_ISL_760726 to 760728, EPI_ISL_760733 to 760734, EPI_ISL_760736 to 760738,
EPI_ISL_760745, EPI_ISL_760748, EPI_ISL_760752 to 760753, EPI_ISL_760755 to 760756, EPI_ISL_760759, EPI_ISL_760761 to 760763, EPI_ISL_760769, EPI_ISL_760776, EPI_ISL_760780, EPI_ISL_760783 to 760786, EPI_ISL_760788, EPI_ISL_760790, EPI_ISL_760792, EPI_ISL_760794 to 760795, EPI_ISL_760799,
EPI_ISL_760802, EPI_ISL_760804, EPI_ISL_760806, EPI_ISL_760808, EPI_ISL_760812, EPI_ISL_760817, EPI_ISL_760825 to 760826, EPI_ISL_760832, EPI_ISL_760838 to 760839, EPI_ISL_760846, EPI_ISL_760852 to 760853, EPI_ISL_760857, EPI_ISL_760860 to 760862, EPI_ISL_760864 to 760865, EPI_ISL_760867 to
760868, EPI_ISL_760874, EPI_ISL_760878, EPI_ISL_760884, EPI_ISL_760887 to 760892, EPI_ISL_760897 to 760898, EPI_ISL_760900, EPI_ISL_760906, EPI_ISL_760908, EPI_ISL_760912, EPI_ISL_760915 to 760916, EPI_ISL_760918, EPI_ISL_760921, EPI_ISL_760924 to 760925, EPI_ISL_760928 to 760929,
EPI_ISL_760931, EPI_ISL_760933, EPI_ISL_760937 to 760938, EPI_ISL_760941 to 760942, EPI_ISL_760945, EPI_ISL_760948 to 760952, EPI_ISL_760960, EPI_ISL_760962, EPI_ISL_760967 to 760968, EPI_ISL_760971, EPI_ISL_760978 to 760979, EPI_ISL_760982, EPI_ISL_760989, EPI_ISL_760994, EPI_ISL_761000 to
761001, EPI_ISL_761008, EPI_ISL_761011, EPI_ISL_761013, EPI_ISL_761022, EPI_ISL_761025, EPI_ISL_761027, EPI_ISL_761029, EPI_ISL_761036, EPI_ISL_761040, EPI_ISL_761042, EPI_ISL_761046, EPI_ISL_761048 to 761049, EPI_ISL_761052, EPI_ISL_761054, EPI_ISL_761056, EPI_ISL_761060 to 761061,
EPI_ISL_761064 to 761066, EPI_ISL_761069, EPI_ISL_761080 to 761093, EPI_ISL_761096 to 761112, EPI_ISL_761115, EPI_ISL_761117, EPI_ISL_761119, EPI_ISL_761122 to 761124, EPI_ISL_761126 to 761127, EPI_ISL_761129 to 761130, EPI_ISL_761132, EPI_ISL_761135 to 761142, EPI_ISL_761144, EPI_ISL_761146 to
761153, EPI_ISL_761155 to 761156, EPI_ISL_761159 to 761161, EPI_ISL_761163 to 761167, EPI_ISL_761169 to 761171, EPI_ISL_761173 to 761175, EPI_ISL_761177 to 761186, EPI_ISL_761188 to 761190, EPI_ISL_761192, EPI_ISL_761195 to 761196, EPI_ISL_761198 to 761203, EPI_ISL_761205 to 761206,
EPI_ISL_761208, EPI_ISL_761210 to 761220, EPI_ISL_761222 to 761224, EPI_ISL_761226, EPI_ISL_761230 to 761238, EPI_ISL_761240, EPI_ISL_761242 to 761251, EPI_ISL_761253 to 761254, EPI_ISL_761256, EPI_ISL_761258, EPI_ISL_761260 to 761265, EPI_ISL_761267 to 761275, EPI_ISL_761277, EPI_ISL_761279,
EPI_ISL_761281 to 761282, EPI_ISL_761284 to 761286, EPI_ISL_761288, EPI_ISL_761290 to 761291, EPI_ISL_761294 to 761295, EPI_ISL_761297, EPI_ISL_761299 to 761301, EPI_ISL_761304 to 761306, EPI_ISL_761308 to 761312, EPI_ISL_761314 to 761325, EPI_ISL_761327 to 761330, EPI_ISL_761332 to 761337,
EPI_ISL_761340 to 761347, EPI_ISL_761349 to 761350, EPI_ISL_761352, EPI_ISL_761354, EPI_ISL_761356, EPI_ISL_761665 to 761920, EPI_ISL_762547, EPI_ISL_762554, EPI_ISL_762557 to 762558, EPI_ISL_762562, EPI_ISL_762564, EPI_ISL_762581, EPI_ISL_762588, EPI_ISL_762599, EPI_ISL_762605 to 762606,
EPI_ISL_762610, EPI_ISL_762617, EPI_ISL_762620, EPI_ISL_762625, EPI_ISL_762629, EPI_ISL_762634, EPI_ISL_762638, EPI_ISL_762642 to 762643, EPI_ISL_762647, EPI_ISL_762655, EPI_ISL_762659, EPI_ISL_762666, EPI_ISL_762671 to 762672, EPI_ISL_762674, EPI_ISL_762683, EPI_ISL_762686, EPI_ISL_762691 to
762693, EPI_ISL_762702 to 762703, EPI_ISL_762706 to 762707, EPI_ISL_762709 to 762710, EPI_ISL_762712, EPI_ISL_762714, EPI_ISL_762720, EPI_ISL_762722, EPI_ISL_762724, EPI_ISL_762730, EPI_ISL_762733, EPI_ISL_762735, EPI_ISL_762750, EPI_ISL_762754, EPI_ISL_762756, EPI_ISL_762763 to 762764,
EPI_ISL_762771, EPI_ISL_762777, EPI_ISL_762786, EPI_ISL_762789, EPI_ISL_762791, EPI_ISL_762793, EPI_ISL_762796, EPI_ISL_762798 to 762799, EPI_ISL_762812, EPI_ISL_762815, EPI_ISL_762819, EPI_ISL_762821, EPI_ISL_762823, EPI_ISL_762828, EPI_ISL_762830, EPI_ISL_762832, EPI_ISL_762835,
EPI_ISL_762839, EPI_ISL_762841, EPI_ISL_762843 to 762845, EPI_ISL_762847, EPI_ISL_762849, EPI_ISL_762862, EPI_ISL_762869, EPI_ISL_762878 to 762879, EPI_ISL_762883, EPI_ISL_762886 to 762887, EPI_ISL_762890, EPI_ISL_762894, EPI_ISL_762900 to 762902, EPI_ISL_762906, EPI_ISL_762910 to 762911,
EPI_ISL_762913, EPI_ISL_762916, EPI_ISL_762919, EPI_ISL_762921 to 762922, EPI_ISL_762926, EPI_ISL_762931 to 762933, EPI_ISL_762935, EPI_ISL_762938 to 762939, EPI_ISL_762947, EPI_ISL_762949, EPI_ISL_762951 to 762952, EPI_ISL_762955, EPI_ISL_762957, EPI_ISL_762964, EPI_ISL_762970 to 762971,
EPI_ISL_762973 to 762975, EPI_ISL_762977, EPI_ISL_762988 to 762989, EPI_ISL_767089, EPI_ISL_767091, EPI_ISL_767093 to 767095, EPI_ISL_767097, EPI_ISL_767102 to 767106, EPI_ISL_767116, EPI_ISL_767119 to 767133, EPI_ISL_767135 to 767137, EPI_ISL_767139 to 767238, EPI_ISL_767240 to 767333,
EPI_ISL_768841 to 768844, EPI_ISL_768898 to 768900, EPI_ISL_777011 to 777012, EPI_ISL_777014 to 777015, EPI_ISL_777017 to 777018, EPI_ISL_777020, EPI_ISL_777024 to 777028, EPI_ISL_777030, EPI_ISL_777032 to 777033, EPI_ISL_777035 to 777036, EPI_ISL_777041 to 777046, EPI_ISL_777048 to 777054,
EPI_ISL_777057, EPI_ISL_777060, EPI_ISL_777063, EPI_ISL_777067, EPI_ISL_777069 to 777072, EPI_ISL_777074 to 777079, EPI_ISL_777083, EPI_ISL_777086 to 777088, EPI_ISL_777090 to 777094, EPI_ISL_777096 to 777097, EPI_ISL_777103, EPI_ISL_777106 to 777107, EPI_ISL_777109 to 777111, EPI_ISL_777113 to
777114, EPI_ISL_777116, EPI_ISL_777119, EPI_ISL_777123, EPI_ISL_777125 to 777126, EPI_ISL_777128 to 777129, EPI_ISL_777132, EPI_ISL_777134 to 777139, EPI_ISL_777143 to 777144, EPI_ISL_777147, EPI_ISL_777150, EPI_ISL_777153, EPI_ISL_777155 to 777157, EPI_ISL_777159, EPI_ISL_777163,
EPI_ISL_777165 to 777172, EPI_ISL_777174 to 777175, EPI_ISL_777177 to 777179, EPI_ISL_777182 to 777183, EPI_ISL_777185 to 777186, EPI_ISL_777188, EPI_ISL_777190, EPI_ISL_777193 to 777195, EPI_ISL_777197 to 777199, EPI_ISL_777202 to 777204, EPI_ISL_777206, EPI_ISL_777210 to 777214, EPI_ISL_777216
to 777217, EPI_ISL_777220 to 777221, EPI_ISL_777226, EPI_ISL_777228, EPI_ISL_777231, EPI_ISL_777234, EPI_ISL_777237, EPI_ISL_777239 to 777240, EPI_ISL_777243 to 777244, EPI_ISL_777247 to 777249, EPI_ISL_777251, EPI_ISL_777254, EPI_ISL_777256 to 777260, EPI_ISL_777262 to 777267, EPI_ISL_777270 to
777274, EPI_ISL_777276 to 777282, EPI_ISL_777285 to 777288, EPI_ISL_777291 to 777292, EPI_ISL_777294, EPI_ISL_777296 to 777297, EPI_ISL_777299, EPI_ISL_777307 to 777309, EPI_ISL_777311 to 777312, EPI_ISL_777314 to 777315, EPI_ISL_777317 to 777319, EPI_ISL_777326 to 777327, EPI_ISL_777333,
EPI_ISL_777336, EPI_ISL_777338, EPI_ISL_777349, EPI_ISL_777375, EPI_ISL_777380, EPI_ISL_777386, EPI_ISL_777389, EPI_ISL_777394, EPI_ISL_777411 to 777412, EPI_ISL_777416, EPI_ISL_777421, EPI_ISL_777424 to 777425, EPI_ISL_777427, EPI_ISL_777430, EPI_ISL_777435, EPI_ISL_777437, EPI_ISL_777440,
EPI_ISL_777448, EPI_ISL_777451, EPI_ISL_777455, EPI_ISL_777458, EPI_ISL_777465, EPI_ISL_777467, EPI_ISL_777470, EPI_ISL_777477, EPI_ISL_777479, EPI_ISL_777481, EPI_ISL_777489, EPI_ISL_777491, EPI_ISL_777495, EPI_ISL_777502, EPI_ISL_777509, EPI_ISL_777515, EPI_ISL_777520, EPI_ISL_777522,
EPI_ISL_777524 to 777525, EPI_ISL_777529 to 777530, EPI_ISL_777532, EPI_ISL_777534 to 777535, EPI_ISL_777537 to 777539, EPI_ISL_777543, EPI_ISL_777548, EPI_ISL_777550, EPI_ISL_777552, EPI_ISL_777558, EPI_ISL_777561, EPI_ISL_777563, EPI_ISL_777567 to 777568, EPI_ISL_777572 to 777573,
EPI_ISL_777576, EPI_ISL_777580, EPI_ISL_777582, EPI_ISL_777584, EPI_ISL_777587, EPI_ISL_777589, EPI_ISL_777591 to 777593, EPI_ISL_777595, EPI_ISL_777597, EPI_ISL_777605, EPI_ISL_777621, EPI_ISL_777627, EPI_ISL_777629, EPI_ISL_777633 to 777634, EPI_ISL_777638 to 777639, EPI_ISL_777644,
EPI_ISL_777649 to 777651, EPI_ISL_777655, EPI_ISL_777659, EPI_ISL_777669, EPI_ISL_777672, EPI_ISL_778016, EPI_ISL_778018, EPI_ISL_778021, EPI_ISL_778029, EPI_ISL_778033, EPI_ISL_778035, EPI_ISL_778040, EPI_ISL_778043 to 778044, EPI_ISL_778047 to 778048, EPI_ISL_778050 to 778052,
EPI_ISL_778055, EPI_ISL_778059, EPI_ISL_778064 to 778065, EPI_ISL_778069 to 778070, EPI_ISL_778074, EPI_ISL_778083, EPI_ISL_778089, EPI_ISL_778101, EPI_ISL_778104, EPI_ISL_778108, EPI_ISL_778113, EPI_ISL_778116, EPI_ISL_778121, EPI_ISL_778128, EPI_ISL_778132, EPI_ISL_778140, EPI_ISL_778144,
EPI_ISL_778163 to 778166, EPI_ISL_778183, EPI_ISL_778187, EPI_ISL_778190 to 778191, EPI_ISL_778195 to 778196, EPI_ISL_778201, EPI_ISL_778207, EPI_ISL_778212, EPI_ISL_778225, EPI_ISL_778228, EPI_ISL_778230, EPI_ISL_778233 to 778234, EPI_ISL_778238 to 778240, EPI_ISL_778246, EPI_ISL_778249,
EPI_ISL_778256, EPI_ISL_778259, EPI_ISL_778261, EPI_ISL_778264, EPI_ISL_778266, EPI_ISL_778270, EPI_ISL_778272, EPI_ISL_778282, EPI_ISL_778287 to 778288, EPI_ISL_778290, EPI_ISL_778292, EPI_ISL_778295, EPI_ISL_778305, EPI_ISL_778309, EPI_ISL_778311, EPI_ISL_778315, EPI_ISL_778327 to 778328,
EPI_ISL_778331, EPI_ISL_778333, EPI_ISL_778335, EPI_ISL_778342, EPI_ISL_778346 to 778348, EPI_ISL_778353, EPI_ISL_778355, EPI_ISL_778357 to 778358, EPI_ISL_778360, EPI_ISL_778365, EPI_ISL_778368, EPI_ISL_778374, EPI_ISL_778380, EPI_ISL_778382, EPI_ISL_778385, EPI_ISL_778390, EPI_ISL_778392,
EPI_ISL_778396, EPI_ISL_778399, EPI_ISL_778401, EPI_ISL_778404, EPI_ISL_778408, EPI_ISL_778410, EPI_ISL_778412, EPI_ISL_778415 to 778417, EPI_ISL_778426, EPI_ISL_778430, EPI_ISL_778432, EPI_ISL_778444, EPI_ISL_778448, EPI_ISL_778454 to 778455, EPI_ISL_778471 to 778473, EPI_ISL_778478,
EPI_ISL_778484, EPI_ISL_778487 to 778488, EPI_ISL_778490 to 778491, EPI_ISL_778494, EPI_ISL_778500, EPI_ISL_778510, EPI_ISL_778512, EPI_ISL_778517, EPI_ISL_778519, EPI_ISL_778524, EPI_ISL_778528 to 778529, EPI_ISL_778531, EPI_ISL_778539, EPI_ISL_778543, EPI_ISL_778547, EPI_ISL_778556,
EPI_ISL_778558, EPI_ISL_778562, EPI_ISL_778564, EPI_ISL_778569, EPI_ISL_778571, EPI_ISL_778578, EPI_ISL_778584, EPI_ISL_778593, EPI_ISL_778598 to 778599, EPI_ISL_778601, EPI_ISL_778605, EPI_ISL_778607, EPI_ISL_778612, EPI_ISL_778614 to 778616, EPI_ISL_778618, EPI_ISL_778621, EPI_ISL_778623,
EPI_ISL_778627 to 778628, EPI_ISL_778630, EPI_ISL_778635, EPI_ISL_778638, EPI_ISL_781186, EPI_ISL_781194 to 781195, EPI_ISL_781201 to 781202, EPI_ISL_781204 to 781207, EPI_ISL_781209 to 781210, EPI_ISL_781214, EPI_ISL_781216, EPI_ISL_781219, EPI_ISL_781226, EPI_ISL_781230, EPI_ISL_781234 to
781235, EPI_ISL_781250, EPI_ISL_781256, EPI_ISL_781259, EPI_ISL_781262, EPI_ISL_781273, EPI_ISL_781280, EPI_ISL_781282, EPI_ISL_781288, EPI_ISL_781291 to 781292, EPI_ISL_781294 to 781295, EPI_ISL_781297, EPI_ISL_781301, EPI_ISL_781307, EPI_ISL_781313, EPI_ISL_781315, EPI_ISL_781318,
EPI_ISL_781323 to 781324, EPI_ISL_781330 to 781331, EPI_ISL_781336 to 781337, EPI_ISL_781348 to 781349, EPI_ISL_781352, EPI_ISL_781356, EPI_ISL_781366, EPI_ISL_781372, EPI_ISL_781374 to 781375, EPI_ISL_781378, EPI_ISL_781389, EPI_ISL_781400, EPI_ISL_781405 to 781406, EPI_ISL_781408,
EPI_ISL_781412, EPI_ISL_781424, EPI_ISL_781433, EPI_ISL_781435, EPI_ISL_781437, EPI_ISL_781443, EPI_ISL_781446 to 781447, EPI_ISL_781451, EPI_ISL_781456 to 781458, EPI_ISL_781463 to 781465, EPI_ISL_781469, EPI_ISL_781473, EPI_ISL_781475, EPI_ISL_781478, EPI_ISL_781495, EPI_ISL_781499 to
781500, EPI_ISL_781502, EPI_ISL_781505, EPI_ISL_781508, EPI_ISL_781510, EPI_ISL_781515 to 781516, EPI_ISL_781872, EPI_ISL_781874, EPI_ISL_781877, EPI_ISL_781879, EPI_ISL_781881, EPI_ISL_781883 to 781884, EPI_ISL_781887, EPI_ISL_781889, EPI_ISL_781892 to 781894, EPI_ISL_781896, EPI_ISL_781899
to 781902, EPI_ISL_781904 to 781906, EPI_ISL_781911 to 781912, EPI_ISL_781917, EPI_ISL_781923, EPI_ISL_781926 to 781927, EPI_ISL_781929, EPI_ISL_781931 to 781932, EPI_ISL_781934 to 781937, EPI_ISL_781939, EPI_ISL_781943, EPI_ISL_781945, EPI_ISL_781949, EPI_ISL_781951, EPI_ISL_781954,
EPI_ISL_781957 to 781959, EPI_ISL_781961 to 781966, EPI_ISL_781969 to 781970, EPI_ISL_781972, EPI_ISL_781974 to 781976, EPI_ISL_781981, EPI_ISL_781983, EPI_ISL_781985 to 781986, EPI_ISL_781989, EPI_ISL_781991, EPI_ISL_781993, EPI_ISL_781995 to 781996, EPI_ISL_782002, EPI_ISL_782005 to 782007,
EPI_ISL_782010 to 782013, EPI_ISL_782016, EPI_ISL_782019 to 782020, EPI_ISL_782022 to 782025, EPI_ISL_782029, EPI_ISL_782033 to 782034, EPI_ISL_782041, EPI_ISL_782043, EPI_ISL_782045, EPI_ISL_782047 to 782048, EPI_ISL_782050, EPI_ISL_782053, EPI_ISL_782057, EPI_ISL_782060, EPI_ISL_782063,
EPI_ISL_782068, EPI_ISL_782070 to 782072, EPI_ISL_782076, EPI_ISL_782078, EPI_ISL_782082 to 782083, EPI_ISL_782085 to 782086, EPI_ISL_782089 to 782091, EPI_ISL_782095 to 782097, EPI_ISL_782099 to 782100, EPI_ISL_782102, EPI_ISL_782104, EPI_ISL_782108 to 782111, EPI_ISL_782117, EPI_ISL_782119 to
782120, EPI_ISL_782122 to 782124, EPI_ISL_782126, EPI_ISL_782132, EPI_ISL_782135, EPI_ISL_782139 to 782142, EPI_ISL_782148 to 782150, EPI_ISL_782161, EPI_ISL_782165, EPI_ISL_782171, EPI_ISL_782184, EPI_ISL_782197, EPI_ISL_782201 to 782202, EPI_ISL_782207, EPI_ISL_782211 to 782212,
EPI_ISL_782220, EPI_ISL_782224, EPI_ISL_782226, EPI_ISL_782235, EPI_ISL_782242 to 782243, EPI_ISL_782245, EPI_ISL_782249 to 782250, EPI_ISL_782252, EPI_ISL_782256, EPI_ISL_782259, EPI_ISL_782264, EPI_ISL_782267 to 782268, EPI_ISL_782271, EPI_ISL_782274, EPI_ISL_782278, EPI_ISL_782282,
EPI_ISL_782287, EPI_ISL_782292, EPI_ISL_782307, EPI_ISL_782309, EPI_ISL_782313 to 782315, EPI_ISL_782317, EPI_ISL_782325 to 782326, EPI_ISL_782328, EPI_ISL_782335, EPI_ISL_782345, EPI_ISL_782352, EPI_ISL_782355, EPI_ISL_782358, EPI_ISL_782361 to 782362, EPI_ISL_782369, EPI_ISL_782371 to
782372, EPI_ISL_782377, EPI_ISL_782380, EPI_ISL_782388, EPI_ISL_782390, EPI_ISL_782393, EPI_ISL_782402, EPI_ISL_782409, EPI_ISL_782412, EPI_ISL_782420, EPI_ISL_782423, EPI_ISL_782425 to 782426, EPI_ISL_782435, EPI_ISL_782439, EPI_ISL_782451, EPI_ISL_782454, EPI_ISL_782461, EPI_ISL_782466,
EPI_ISL_782471, EPI_ISL_782474, EPI_ISL_782478, EPI_ISL_782737, EPI_ISL_782745, EPI_ISL_782747 to 782748, EPI_ISL_782758, EPI_ISL_782768, EPI_ISL_782771, EPI_ISL_782774, EPI_ISL_782783 to 782784, EPI_ISL_782787, EPI_ISL_782791 to 782792, EPI_ISL_782795, EPI_ISL_782801, EPI_ISL_782805 to
782806, EPI_ISL_782808, EPI_ISL_782813, EPI_ISL_782820, EPI_ISL_782825, EPI_ISL_782827, EPI_ISL_782832, EPI_ISL_782838, EPI_ISL_782841, EPI_ISL_782843 to 782844, EPI_ISL_782849, EPI_ISL_782855 to 782856, EPI_ISL_782869, EPI_ISL_782872 to 782873, EPI_ISL_782877 to 782878, EPI_ISL_782892 to
782894, EPI_ISL_782897, EPI_ISL_782899, EPI_ISL_782902 to 782905, EPI_ISL_782911, EPI_ISL_782918 to 782919, EPI_ISL_782924, EPI_ISL_782926, EPI_ISL_782931, EPI_ISL_782936, EPI_ISL_782945 to 782946, EPI_ISL_782956, EPI_ISL_782963 to 782964, EPI_ISL_782967, EPI_ISL_782969 to 782970,
EPI_ISL_782976, EPI_ISL_782988, EPI_ISL_782993 to 782994, EPI_ISL_782997, EPI_ISL_783000, EPI_ISL_783002, EPI_ISL_783007 to 783008, EPI_ISL_783014 to 783015, EPI_ISL_783026, EPI_ISL_783030, EPI_ISL_783032 to 783034, EPI_ISL_783043, EPI_ISL_783045 to 783047, EPI_ISL_783052, EPI_ISL_783056,
EPI_ISL_783068, EPI_ISL_796784 to 797117, EPI_ISL_797399, EPI_ISL_797402, EPI_ISL_797409, EPI_ISL_797411, EPI_ISL_797413, EPI_ISL_797416, EPI_ISL_797420 to 797421, EPI_ISL_797424, EPI_ISL_797428, EPI_ISL_797430, EPI_ISL_797435, EPI_ISL_797438, EPI_ISL_797441, EPI_ISL_797456, EPI_ISL_797461 to
797462, EPI_ISL_797468, EPI_ISL_797475, EPI_ISL_797480, EPI_ISL_797484, EPI_ISL_797489, EPI_ISL_797491, EPI_ISL_797499, EPI_ISL_797504, EPI_ISL_797509, EPI_ISL_797512, EPI_ISL_797533, EPI_ISL_797535, EPI_ISL_797540 to 797541, EPI_ISL_797543, EPI_ISL_797547, EPI_ISL_797549, EPI_ISL_797553,

EPI_ISL_797559 to 797560, EPI_ISL_797568, EPI_ISL_797575 to 797576, EPI_ISL_797586 to 797587, EPI_ISL_797599, EPI_ISL_797605 to 797606, EPI_ISL_797610, EPI_ISL_797613, EPI_ISL_797616, EPI_ISL_797619, EPI_ISL_797635, EPI_ISL_797637, EPI_ISL_797641 to 797642, EPI_ISL_797644, EPI_ISL_797654, EPI_ISL_797660, EPI_ISL_797663, EPI_ISL_797666, EPI_ISL_797673, EPI_ISL_797686, EPI_ISL_797699, EPI_ISL_797701, EPI_ISL_797709, EPI_ISL_797712, EPI_ISL_797714 to 797715, EPI_ISL_797717, EPI_ISL_797999, EPI_ISL_798002, EPI_ISL_798007, EPI_ISL_798009, EPI_ISL_798011, EPI_ISL_798013, EPI_ISL_798015, EPI_ISL_798025, EPI_ISL_798027, EPI_ISL_798029, EPI_ISL_798035, EPI_ISL_798054, EPI_ISL_798061 to 798062, EPI_ISL_798064, EPI_ISL_798066, EPI_ISL_798068, EPI_ISL_798073, EPI_ISL_798083 to 798084, EPI_ISL_798096, EPI_ISL_798101, EPI_ISL_798103, EPI_ISL_798106, EPI_ISL_798108, EPI_ISL_798119, EPI_ISL_798122 to 798125, EPI_ISL_798127, EPI_ISL_798131, EPI_ISL_798133 to 798134, EPI_ISL_798139, EPI_ISL_798152, EPI_ISL_798154, EPI_ISL_798169 to 798170, EPI_ISL_798173 to 798176, EPI_ISL_798181 to 798182, EPI_ISL_798202, EPI_ISL_798204, EPI_ISL_798208, EPI_ISL_798220 to 798222, EPI_ISL_798225, EPI_ISL_798229, EPI_ISL_798234 to 798236, EPI_ISL_798239, EPI_ISL_798241 to 798242, EPI_ISL_798245 to 798246, EPI_ISL_798248, EPI_ISL_798250, EPI_ISL_798255, EPI_ISL_798268, EPI_ISL_798274 to 798275, EPI_ISL_798277, EPI_ISL_798280, EPI_ISL_798282 to 798285, EPI_ISL_798292, EPI_ISL_798295 to 798296, EPI_ISL_798299, EPI_ISL_798305, EPI_ISL_798310, EPI_ISL_798314, EPI_ISL_798321, EPI_ISL_798323 to 798324, EPI_ISL_798328 to 798329, EPI_ISL_798334, EPI_ISL_798336, EPI_ISL_798341, EPI_ISL_798345 to 798346, EPI_ISL_798349, EPI_ISL_798356, EPI_ISL_798362, EPI_ISL_798368 to 798370, EPI_ISL_798378 to 798379, EPI_ISL_798384, EPI_ISL_798386, EPI_ISL_798388 to 798389, EPI_ISL_798396, EPI_ISL_798399, EPI_ISL_798401, EPI_ISL_798404 to 798405, EPI_ISL_798413, EPI_ISL_798416 to 798417, EPI_ISL_798431, EPI_ISL_798436, EPI_ISL_798444, EPI_ISL_798455, EPI_ISL_798465, EPI_ISL_798468, EPI_ISL_798475, EPI_ISL_798479, EPI_ISL_798483, EPI_ISL_798487, EPI_ISL_798492 to 798493, EPI_ISL_798500, EPI_ISL_798502, EPI_ISL_798508, EPI_ISL_798510, EPI_ISL_798513, EPI_ISL_798516 to 798517, EPI_ISL_798519, EPI_ISL_798524, EPI_ISL_798531, EPI_ISL_798534, EPI_ISL_798539, EPI_ISL_798541 to 798542, EPI_ISL_798544, EPI_ISL_798548, EPI_ISL_798550, EPI_ISL_798555, EPI_ISL_798561, EPI_ISL_798564, EPI_ISL_798579, EPI_ISL_798591, EPI_ISL_798593, EPI_ISL_798598, EPI_ISL_798601, EPI_ISL_798607, EPI_ISL_798609, EPI_ISL_798612, EPI_ISL_798615, EPI_ISL_798622, EPI_ISL_798631 to 798633, EPI_ISL_798635, EPI_ISL_798637, EPI_ISL_798639 to 798641, EPI_ISL_798643, EPI_ISL_798652, EPI_ISL_798659, EPI_ISL_798661, EPI_ISL_798665, EPI_ISL_798667 to 798669, EPI_ISL_798671, EPI_ISL_798676, EPI_ISL_798684, EPI_ISL_798688 to 798690, EPI_ISL_798692, EPI_ISL_798695, EPI_ISL_798704 to 798705, EPI_ISL_798709, EPI_ISL_798719, EPI_ISL_798722, EPI_ISL_798727, EPI_ISL_798729, EPI_ISL_798735, EPI_ISL_798742, EPI_ISL_798744, EPI_ISL_798749, EPI_ISL_798768, EPI_ISL_798771, EPI_ISL_798784, EPI_ISL_798791, EPI_ISL_798793, EPI_ISL_798798, EPI_ISL_798804, EPI_ISL_798811, EPI_ISL_798814, EPI_ISL_798819, EPI_ISL_798824, EPI_ISL_798826, EPI_ISL_798833, EPI_ISL_798845, EPI_ISL_798850, EPI_ISL_798852 to 798853, EPI_ISL_798869, EPI_ISL_798872, EPI_ISL_798878, EPI_ISL_798880, EPI_ISL_798884 to 798885, EPI_ISL_798888, EPI_ISL_798891, EPI_ISL_798898, EPI_ISL_798902, EPI_ISL_798905 to 798906, EPI_ISL_798914, EPI_ISL_798921, EPI_ISL_798924, EPI_ISL_798926, EPI_ISL_798928, EPI_ISL_798934 to 798935, EPI_ISL_798939, EPI_ISL_798941, EPI_ISL_798951 to 798952, EPI_ISL_798957, EPI_ISL_798962 to 798964, EPI_ISL_798968 to 798969, EPI_ISL_798984, EPI_ISL_798986, EPI_ISL_798988, EPI_ISL_798991, EPI_ISL_799000, EPI_ISL_799003 to 799005, EPI_ISL_799010, EPI_ISL_799021 to 799023, EPI_ISL_799025 to 799026, EPI_ISL_799029, EPI_ISL_799040, EPI_ISL_799045, EPI_ISL_799047, EPI_ISL_799053, EPI_ISL_799055, EPI_ISL_799057, EPI_ISL_799059, EPI_ISL_799069 to 799070, EPI_ISL_799079, EPI_ISL_799085 to 799086, EPI_ISL_799099, EPI_ISL_799103 to 799106, EPI_ISL_799111, EPI_ISL_799113 to 799114, EPI_ISL_799122, EPI_ISL_799124, EPI_ISL_799137 to 799139, EPI_ISL_799143, EPI_ISL_799145, EPI_ISL_799149, EPI_ISL_799154, EPI_ISL_799160 to 799161, EPI_ISL_799165 to 799166, EPI_ISL_799170 to 799171, EPI_ISL_799177 to 799179, EPI_ISL_799182, EPI_ISL_799187 to 799188, EPI_ISL_799191, EPI_ISL_799198, EPI_ISL_799200, EPI_ISL_799202, EPI_ISL_799212 to 799214, EPI_ISL_799219, EPI_ISL_799227 to 799229, EPI_ISL_799231, EPI_ISL_799233 to 799235, EPI_ISL_799237, EPI_ISL_799244, EPI_ISL_799248, EPI_ISL_799252, EPI_ISL_799257, EPI_ISL_799261, EPI_ISL_799265, EPI_ISL_799269, EPI_ISL_799271, EPI_ISL_799276, EPI_ISL_799278, EPI_ISL_799281, EPI_ISL_799637 to 799638, EPI_ISL_799644, EPI_ISL_799652, EPI_ISL_799655, EPI_ISL_799662, EPI_ISL_799665, EPI_ISL_799678, EPI_ISL_799683, EPI_ISL_799687, EPI_ISL_799691, EPI_ISL_799693, EPI_ISL_799702, EPI_ISL_799706, EPI_ISL_799711, EPI_ISL_799732, EPI_ISL_799734, EPI_ISL_799744, EPI_ISL_799747, EPI_ISL_799749 to 799751, EPI_ISL_799754, EPI_ISL_799760, EPI_ISL_799767, EPI_ISL_799784, EPI_ISL_799787, EPI_ISL_799796 to 799797, EPI_ISL_799799, EPI_ISL_799807, EPI_ISL_799809, EPI_ISL_799812, EPI_ISL_799814, EPI_ISL_799818, EPI_ISL_799821 to 799823, EPI_ISL_799831 to 799832, EPI_ISL_799840 to 799841, EPI_ISL_799844, EPI_ISL_799846, EPI_ISL_799859, EPI_ISL_799868, EPI_ISL_799877, EPI_ISL_799881, EPI_ISL_799891, EPI_ISL_799897 to 799899, EPI_ISL_799902, EPI_ISL_799908, EPI_ISL_799914, EPI_ISL_799920, EPI_ISL_799922, EPI_ISL_799924 to 799925, EPI_ISL_799930, EPI_ISL_799933, EPI_ISL_799938, EPI_ISL_799940 to 799943, EPI_ISL_799948, EPI_ISL_799956, EPI_ISL_799961, EPI_ISL_799964, EPI_ISL_799966 to 799967, EPI_ISL_799970, EPI_ISL_799975, EPI_ISL_799977 to 799979, EPI_ISL_799982 to 799984, EPI_ISL_799989, EPI_ISL_799991, EPI_ISL_799995, EPI_ISL_799998 to 799999, EPI_ISL_800002 to 800003, EPI_ISL_800009, EPI_ISL_800011, EPI_ISL_800013 to 800014, EPI_ISL_800016 to 800017, EPI_ISL_800019 to 800023, EPI_ISL_800025, EPI_ISL_800031, EPI_ISL_800033, EPI_ISL_800035 to 800036, EPI_ISL_800039 to 800044, EPI_ISL_800046, EPI_ISL_800049 to 800051, EPI_ISL_800053, EPI_ISL_800058 to 800063, EPI_ISL_800066 to 800067, EPI_ISL_800069, EPI_ISL_800073 to 800076, EPI_ISL_800080 to 800081, EPI_ISL_800087, EPI_ISL_800090 to 800091, EPI_ISL_800094, EPI_ISL_800097, EPI_ISL_800099, EPI_ISL_800101, EPI_ISL_800104 to 800105, EPI_ISL_800107 to 800110, EPI_ISL_800112, EPI_ISL_800118, EPI_ISL_800126 to 800130, EPI_ISL_800132, EPI_ISL_800134 to 800136, EPI_ISL_800140, EPI_ISL_800143 to 800146, EPI_ISL_800148, EPI_ISL_800150, EPI_ISL_800152, EPI_ISL_800154 to 800155, EPI_ISL_800159 to 800160, EPI_ISL_800162 to 800163, EPI_ISL_800165, EPI_ISL_800169 to 800171, EPI_ISL_800179, EPI_ISL_800181, EPI_ISL_800184 to 800185, EPI_ISL_800187, EPI_ISL_800193 to 800194, EPI_ISL_800197 to 800199, EPI_ISL_800202, EPI_ISL_800205 to 800209, EPI_ISL_800212 to 800213, EPI_ISL_800215 to 800216, EPI_ISL_800219, EPI_ISL_800222 to 800224, EPI_ISL_800226 to 800227, EPI_ISL_800229 to 800232, EPI_ISL_800239 to 800240, EPI_ISL_800242 to 800243, EPI_ISL_800246 to 800249, EPI_ISL_800262, EPI_ISL_800267, EPI_ISL_800271, EPI_ISL_800274, EPI_ISL_800280 to 800282, EPI_ISL_800286, EPI_ISL_800299, EPI_ISL_800301, EPI_ISL_800303, EPI_ISL_800308, EPI_ISL_800317, EPI_ISL_800338, EPI_ISL_800340, EPI_ISL_800342, EPI_ISL_800347 to 800348, EPI_ISL_800350 to 800351, EPI_ISL_800354 to 800355, EPI_ISL_800359 to 800360, EPI_ISL_800362, EPI_ISL_800365, EPI_ISL_800367, EPI_ISL_800370, EPI_ISL_800376, EPI_ISL_800384, EPI_ISL_800392, EPI_ISL_800395, EPI_ISL_800407, EPI_ISL_800420, EPI_ISL_800428, EPI_ISL_800441, EPI_ISL_800444, EPI_ISL_800446, EPI_ISL_800452 to 800454, EPI_ISL_800456 to 800457, EPI_ISL_800460, EPI_ISL_800468, EPI_ISL_800474, EPI_ISL_800481, EPI_ISL_800485, EPI_ISL_800494 to 800495, EPI_ISL_800501 to 800502, EPI_ISL_800506, EPI_ISL_800509, EPI_ISL_800516, EPI_ISL_800518, EPI_ISL_800524 to 800526, EPI_ISL_800528, EPI_ISL_800531, EPI_ISL_800542, EPI_ISL_800555, EPI_ISL_800558, EPI_ISL_800566, EPI_ISL_800575, EPI_ISL_800577 to 800580, EPI_ISL_820129 to 820130, EPI_ISL_820132 to 820133, EPI_ISL_820135, EPI_ISL_820137, EPI_ISL_820139 to 820140, EPI_ISL_820142 to 820143, EPI_ISL_820145, EPI_ISL_820148, EPI_ISL_820153, EPI_ISL_820155, EPI_ISL_820157 to 820158, EPI_ISL_820160, EPI_ISL_820162 to 820163, EPI_ISL_820165 to 820166, EPI_ISL_820168, EPI_ISL_820170, EPI_ISL_820173 to 820174, EPI_ISL_820179, EPI_ISL_820181, EPI_ISL_820185, EPI_ISL_820187, EPI_ISL_820189, EPI_ISL_820192, EPI_ISL_820194 to 820195, EPI_ISL_820198, EPI_ISL_820201, EPI_ISL_820203, EPI_ISL_820205, EPI_ISL_820207 to 820208, EPI_ISL_820210, EPI_ISL_820214, EPI_ISL_820217, EPI_ISL_820219, EPI_ISL_820222, EPI_ISL_820224, EPI_ISL_820228, EPI_ISL_820230, EPI_ISL_820232, EPI_ISL_820235, EPI_ISL_820237, EPI_ISL_820239 to 820240, EPI_ISL_820242, EPI_ISL_820245, EPI_ISL_820247 to 820248, EPI_ISL_820250, EPI_ISL_820252, EPI_ISL_820254 to 820255, EPI_ISL_820257 to 820258, EPI_ISL_820263, EPI_ISL_820265, EPI_ISL_820267, EPI_ISL_820269, EPI_ISL_820274 to 820275, EPI_ISL_820277 to 820278, EPI_ISL_820280 to 820281, EPI_ISL_820285 to 820286, EPI_ISL_820288, EPI_ISL_820290 to 820291, EPI_ISL_820293, EPI_ISL_820296, EPI_ISL_820298 to 820299, EPI_ISL_820301, EPI_ISL_820305, EPI_ISL_820307 to 820308, EPI_ISL_820310, EPI_ISL_820313, EPI_ISL_820315, EPI_ISL_820317, EPI_ISL_820322, EPI_ISL_820324 to 820325, EPI_ISL_820327, EPI_ISL_820329 to 820330, EPI_ISL_820332, EPI_ISL_820334, EPI_ISL_820336, EPI_ISL_820338 to 820339, EPI_ISL_820341 to 820342, EPI_ISL_820344, EPI_ISL_820346, EPI_ISL_820348, EPI_ISL_820351 to 820352, EPI_ISL_820354, EPI_ISL_820357, EPI_ISL_820359, EPI_ISL_820361 to 820362, EPI_ISL_820364, EPI_ISL_820366 to 820367, EPI_ISL_820369 to 820370, EPI_ISL_820372, EPI_ISL_820374 to 820375, EPI_ISL_820377, EPI_ISL_820379 to 820380, EPI_ISL_820382, EPI_ISL_820389 to 820390, EPI_ISL_820392 to 820393, EPI_ISL_820395, EPI_ISL_820398, EPI_ISL_820400 to 820401, EPI_ISL_820403, EPI_ISL_820405 to 820406, EPI_ISL_820408, EPI_ISL_820411, EPI_ISL_820413 to 820414, EPI_ISL_820416, EPI_ISL_820419, EPI_ISL_820422, EPI_ISL_820424 to 820425, EPI_ISL_820427, EPI_ISL_820429 to 820430, EPI_ISL_820432, EPI_ISL_820434 to 820435, EPI_ISL_820438, EPI_ISL_820440, EPI_ISL_820442 to 820443, EPI_ISL_820445, EPI_ISL_820447, EPI_ISL_820450, EPI_ISL_820452 to 820453, EPI_ISL_820455, EPI_ISL_820457 to 820458, EPI_ISL_820460, EPI_ISL_820463, EPI_ISL_820466, EPI_ISL_820469, EPI_ISL_820471, EPI_ISL_820473 to 820474, EPI_ISL_820476, EPI_ISL_820482, EPI_ISL_820486 to 820487, EPI_ISL_820491 to 820492, EPI_ISL_820494 to 820495, EPI_ISL_820497, EPI_ISL_820499 to 820500, EPI_ISL_820502 to 820503, EPI_ISL_820508, EPI_ISL_820515, EPI_ISL_820517, EPI_ISL_820520, EPI_ISL_820522 to 820523, EPI_ISL_820525, EPI_ISL_820527, EPI_ISL_820529 to 820530, EPI_ISL_820532, EPI_ISL_820534 to 820535, EPI_ISL_820537 to 820538, EPI_ISL_820540, EPI_ISL_820542, EPI_ISL_820544 to 820545, EPI_ISL_820548, EPI_ISL_820550 to 820551, EPI_ISL_820553, EPI_ISL_820555 to 820556, EPI_ISL_820559, EPI_ISL_820561 to 820562, EPI_ISL_820564, EPI_ISL_820566 to 820567, EPI_ISL_820569, EPI_ISL_820571 to 820572, EPI_ISL_820574, EPI_ISL_820576, EPI_ISL_820579, EPI_ISL_820581 to 820582, EPI_ISL_820584 to 820585, EPI_ISL_820587, EPI_ISL_820589, EPI_ISL_820592, EPI_ISL_820594 to 820595, EPI_ISL_820599 to 820600, EPI_ISL_820602, EPI_ISL_820604, EPI_ISL_820606, EPI_ISL_820608, EPI_ISL_820611 to 820612, EPI_ISL_820614 to 820616, EPI_ISL_820619, EPI_ISL_820621 to 820627, EPI_ISL_820629 to 820632, EPI_ISL_820635 to 820638, EPI_ISL_820640, EPI_ISL_820642 to 820644, EPI_ISL_820646, EPI_ISL_821002 to 821031, EPI_ISL_821033 to 821036, EPI_ISL_821038 to 821055, EPI_ISL_821057 to 821060, EPI_ISL_821063 to 821068, EPI_ISL_821070 to 821076, EPI_ISL_821078 to 821097, EPI_ISL_821099 to 821104, EPI_ISL_821106 to 821141, EPI_ISL_821143 to 821149, EPI_ISL_821151 to 821159, EPI_ISL_821161 to 821194, EPI_ISL_821196 to 821197, EPI_ISL_821199 to 821206, EPI_ISL_821208 to 821224, EPI_ISL_821226, EPI_ISL_821228 to 821240, EPI_ISL_821242 to 821244, EPI_ISL_821246 to 821248, EPI_ISL_821250 to 821256, EPI_ISL_821259 to 821265, EPI_ISL_821267 to 821268, EPI_ISL_821270 to 821273, EPI_ISL_821283, EPI_ISL_821285, EPI_ISL_821288, EPI_ISL_821292, EPI_ISL_821294, EPI_ISL_821298, EPI_ISL_821303, EPI_ISL_821308, EPI_ISL_821314, EPI_ISL_821318, EPI_ISL_821327, EPI_ISL_821329, EPI_ISL_821335, EPI_ISL_821347, EPI_ISL_821351, EPI_ISL_821366, EPI_ISL_821370 to 821372, EPI_ISL_821382, EPI_ISL_821384, EPI_ISL_821389 to 821390, EPI_ISL_821398, EPI_ISL_821401, EPI_ISL_821403, EPI_ISL_821412, EPI_ISL_821414 to 821415, EPI_ISL_821418 to 821419, EPI_ISL_821425 to 821426, EPI_ISL_821433, EPI_ISL_821436 to 821437, EPI_ISL_821440, EPI_ISL_821446, EPI_ISL_821451, EPI_ISL_821453, EPI_ISL_821456, EPI_ISL_821459, EPI_ISL_821465 to 821466, EPI_ISL_821469 to 821470, EPI_ISL_821476, EPI_ISL_821482 to 821483, EPI_ISL_821490, EPI_ISL_821493, EPI_ISL_821496 to 821497, EPI_ISL_821500, EPI_ISL_821502 to 821503, EPI_ISL_821511, EPI_ISL_821518, EPI_ISL_821525 to 821526, EPI_ISL_821528, EPI_ISL_821533 to 821534, EPI_ISL_821538, EPI_ISL_821548, EPI_ISL_821550 to 821553, EPI_ISL_821556, EPI_ISL_821558, EPI_ISL_821566, EPI_ISL_821584 to 821586, EPI_ISL_821599 to 821600, EPI_ISL_821606 to 821607, EPI_ISL_821614, EPI_ISL_821617, EPI_ISL_821626 to 821628, EPI_ISL_821971 to 821974, EPI_ISL_821976 to 821992, EPI_ISL_821994, EPI_ISL_821996 to 822000, EPI_ISL_822002 to 822003, EPI_ISL_822007 to 822010, EPI_ISL_822012 to 822021, EPI_ISL_822024 to 822028, EPI_ISL_822030 to 822032, EPI_ISL_822034, EPI_ISL_822036 to 822039, EPI_ISL_822042 to 822043, EPI_ISL_822045 to 822047, EPI_ISL_822049 to 822050, EPI_ISL_822052, EPI_ISL_822054 to 822058, EPI_ISL_822060 to 822062, EPI_ISL_822064 to 822066, EPI_ISL_822068, EPI_ISL_822070 to 822071, EPI_ISL_822073 to 822074, EPI_ISL_822076 to 822077, EPI_ISL_822079 to 822082, EPI_ISL_822084 to 822097, EPI_ISL_822100 to 822112, EPI_ISL_822114 to 822116, EPI_ISL_822118, EPI_ISL_822120 to 822121, EPI_ISL_822123 to 822126, EPI_ISL_822128 to 822129, EPI_ISL_822131 to 822139, EPI_ISL_822141 to 822147, EPI_ISL_822149 to 822152, EPI_ISL_822154 to 822155, EPI_ISL_822157, EPI_ISL_822160 to 822164, EPI_ISL_822166 to 822168, EPI_ISL_822171 to 822177, EPI_ISL_822179 to 822181, EPI_ISL_822183 to 822200, EPI_ISL_822202 to 822213, EPI_ISL_822215 to 822222, EPI_ISL_822224 to 822229, EPI_ISL_822231 to 822236, EPI_ISL_822238 to 822239, EPI_ISL_822241, EPI_ISL_822246, EPI_ISL_822248, EPI_ISL_822250 to 822252, EPI_ISL_822254 to 822255, EPI_ISL_822257 to 822259, EPI_ISL_822261 to 822263, EPI_ISL_822266, EPI_ISL_822269 to 822271, EPI_ISL_822273 to 822274, EPI_ISL_822276 to 822280, EPI_ISL_822283 to 822287, EPI_ISL_822289 to 822291, EPI_ISL_822294, EPI_ISL_822297 to 822298, EPI_ISL_822300, EPI_ISL_822303, EPI_ISL_822308, EPI_ISL_822317, EPI_ISL_826464 to 826465, EPI_ISL_826467 to 826472, EPI_ISL_826474, EPI_ISL_826476, EPI_ISL_826478, EPI_ISL_826480 to 826515, EPI_ISL_826517, EPI_ISL_826519, EPI_ISL_833933 to 834552, EPI_ISL_834798 to 835142, EPI_ISL_835426 to 835432, EPI_ISL_835434 to 835438, EPI_ISL_835440 to 835446, EPI_ISL_835449 to 835453, EPI_ISL_835455 to 835456, EPI_ISL_835458 to 835463, EPI_ISL_835466 to 835469, EPI_ISL_835471 to 835474, EPI_ISL_835476 to 835480, EPI_ISL_835482 to 835485, EPI_ISL_835489 to 835492, EPI_ISL_835494 to 835496, EPI_ISL_835498, EPI_ISL_835501, EPI_ISL_835504, EPI_ISL_835508 to 835514, EPI_ISL_835516 to 835517, EPI_ISL_835519 to 835526, EPI_ISL_835528 to 835529, EPI_ISL_835532, EPI_ISL_835534, EPI_ISL_835536 to 835538, EPI_ISL_835542 to 835546, EPI_ISL_835549 to 835552, EPI_ISL_835554, EPI_ISL_835556 to 835563, EPI_ISL_835565 to 835569, EPI_ISL_835571 to 835572, EPI_ISL_835575, EPI_ISL_835578, EPI_ISL_835580 to 835581, EPI_ISL_835583 to 835588, EPI_ISL_835591, EPI_ISL_835593 to 835596, EPI_ISL_835598 to 835599, EPI_ISL_835601 to 835602, EPI_ISL_835604 to 835609, EPI_ISL_835611 to 835613, EPI_ISL_835615, EPI_ISL_835617, EPI_ISL_835619 to 835621, EPI_ISL_835624 to 835630, EPI_ISL_835632 to 835642, EPI_ISL_835644 to 835645, EPI_ISL_835648 to 835650, EPI_ISL_835652 to 835658, EPI_ISL_835660 to 835663, EPI_ISL_835665 to 835670, EPI_ISL_835672, EPI_ISL_835674 to 835676, EPI_ISL_835679 to 835681, EPI_ISL_835684 to 835685, EPI_ISL_835689 to 835691, EPI_ISL_835693 to 835694, EPI_ISL_835696 to 835700, EPI_ISL_835702, EPI_ISL_835704 to 835706, EPI_ISL_835708 to 835709, EPI_ISL_836754, EPI_ISL_836765 to 836766, EPI_ISL_836769, EPI_ISL_836772, EPI_ISL_836777, EPI_ISL_836781, EPI_ISL_836788, EPI_ISL_836792, EPI_ISL_836795, EPI_ISL_836797, EPI_ISL_836802 to 836803, EPI_ISL_836806 to 836807, EPI_ISL_836811, EPI_ISL_836813 to 836814, EPI_ISL_836817, EPI_ISL_836819, EPI_ISL_836822, EPI_ISL_836836 to 836837, EPI_ISL_836840, EPI_ISL_836842 to 836843, EPI_ISL_836848, EPI_ISL_836851, EPI_ISL_836858 to 836859, EPI_ISL_836862 to 836863, EPI_ISL_836866 to 836867, EPI_ISL_836876 to 836878, EPI_ISL_836883, EPI_ISL_836887, EPI_ISL_836890 to 836891, EPI_ISL_836897, EPI_ISL_836903, EPI_ISL_836906, EPI_ISL_836909 to 836910, EPI_ISL_836913 to 836914, EPI_ISL_836920 to 836922, EPI_ISL_836926, EPI_ISL_836928 to 836929, EPI_ISL_836935 to 836936, EPI_ISL_836940, EPI_ISL_836946 to 836948, EPI_ISL_836955, EPI_ISL_836962, EPI_ISL_836975 to 836976, EPI_ISL_846200 to 846508, EPI_ISL_846510 to 846526, EPI_ISL_846536 to 846537, EPI_ISL_851673, EPI_ISL_851675, EPI_ISL_851677 to 851680, EPI_ISL_851682 to 851684, EPI_ISL_851688, EPI_ISL_851691 to 851692, EPI_ISL_851694 to 851700, EPI_ISL_851702 to 851713, EPI_ISL_851715 to 851719, EPI_ISL_851721 to 851724, EPI_ISL_851726, EPI_ISL_851728, EPI_ISL_851730 to 851733, EPI_ISL_851735 to 851737, EPI_ISL_851739 to 851742, EPI_ISL_851744 to 851746, EPI_ISL_851748, EPI_ISL_851750 to 851753, EPI_ISL_851755 to 851756, EPI_ISL_851758 to 851759, EPI_ISL_851761 to 851762, EPI_ISL_851764 to 851766, EPI_ISL_851768 to 851769, EPI_ISL_851771, EPI_ISL_851773, EPI_ISL_851775 to 851779, EPI_ISL_851782 to 851784, EPI_ISL_851786 to 851791, EPI_ISL_851793, EPI_ISL_851795, EPI_ISL_851800 to 851802, EPI_ISL_851804, EPI_ISL_851806, EPI_ISL_851808 to 851811, EPI_ISL_851813, EPI_ISL_851815 to 851828, EPI_ISL_851830 to 851836, EPI_ISL_851838 to 851839, EPI_ISL_851841 to 851845, EPI_ISL_851848 to 851850, EPI_ISL_851852 to 851855, EPI_ISL_851858, EPI_ISL_851860 to 851862, EPI_ISL_851867 to 851869, EPI_ISL_851872 to 851875, EPI_ISL_851878 to 851884, EPI_ISL_851886 to 851887, EPI_ISL_851889 to 851890, EPI_ISL_851893 to 851894, EPI_ISL_851896 to 851897, EPI_ISL_851899 to 851900, EPI_ISL_851902 to 851904, EPI_ISL_851907 to 851915, EPI_ISL_851917 to 851919, EPI_ISL_851921, EPI_ISL_851923 to 851928, EPI_ISL_851930 to 851933, EPI_ISL_851935, EPI_ISL_851937 to 851939, EPI_ISL_851941 to 851949, EPI_ISL_851951 to 851953, EPI_ISL_851964, EPI_ISL_851989, EPI_ISL_852025, EPI_ISL_852047, EPI_ISL_852074, EPI_ISL_852083, EPI_ISL_852091, EPI_ISL_852107, EPI_ISL_852183, EPI_ISL_852191, EPI_ISL_852214, EPI_ISL_852225, EPI_ISL_857874 to 857875, EPI_ISL_857877, EPI_ISL_857879 to 857880, EPI_ISL_857882 to 857884, EPI_ISL_857886, EPI_ISL_857890 to 857891, EPI_ISL_857960 to 858010, EPI_ISL_858064 to 858157, EPI_ISL_858207 to 858222, EPI_ISL_858269 to 858270, EPI_ISL_858287, EPI_ISL_858296, EPI_ISL_858306 to 858309, EPI_ISL_858311, EPI_ISL_858324 to 858326, EPI_ISL_858328 to 858329, EPI_ISL_858332, EPI_ISL_858334, EPI_ISL_858343, EPI_ISL_858354, EPI_ISL_858357, EPI_ISL_858368, EPI_ISL_858370, EPI_ISL_858375, EPI_ISL_858381 to 858382, EPI_ISL_858387 to 858389, EPI_ISL_858393, EPI_ISL_858397, EPI_ISL_858400, EPI_ISL_858403, EPI_ISL_858408 to 858409, EPI_ISL_858411, EPI_ISL_858413 to 858414, EPI_ISL_858416, EPI_ISL_858418, EPI_ISL_858422, EPI_ISL_858516, EPI_ISL_858521, EPI_ISL_858523, EPI_ISL_858531, EPI_ISL_858557, EPI_ISL_858563, EPI_ISL_858572 to 858582, EPI_ISL_858644 to 858645, EPI_ISL_858647 to 858649, EPI_ISL_858653 to 858655, EPI_ISL_858662 to 858663, EPI_ISL_858666 to 858667, EPI_ISL_858669 to 858710, EPI_ISL_858713 to 858714, EPI_ISL_858719 to 858720, EPI_ISL_858725, EPI_ISL_858732, EPI_ISL_858737, EPI_ISL_858739, EPI_ISL_858748, EPI_ISL_858752 to 858753, EPI_ISL_858779 to 858780, EPI_ISL_858784 to 858791, EPI_ISL_858809, EPI_ISL_858811 to 858814, EPI_ISL_858816 to 858829, EPI_ISL_858831 to 858841, EPI_ISL_858845 to 858858, EPI_ISL_858861 to 858862, EPI_ISL_858864, EPI_ISL_858866, EPI_ISL_858868 to 858873, EPI_ISL_858875 to 858899, EPI_ISL_858902, EPI_ISL_858905, EPI_ISL_858907, EPI_ISL_858909, EPI_ISL_858913 to 858915, EPI_ISL_858917 to 858918, EPI_ISL_858923 to 858926, EPI_ISL_858929, EPI_ISL_858931 to 858934, EPI_ISL_858938 to 858942, EPI_ISL_858944, EPI_ISL_858946 to 858952, EPI_ISL_858954 to 858957, EPI_ISL_858959, EPI_ISL_858961 to 858968, EPI_ISL_858971 to 858973, EPI_ISL_858976 to 858989, EPI_ISL_858991 to 858996, EPI_ISL_858998 to 859002, EPI_ISL_859004 to 859011, EPI_ISL_859013 to 859029, EPI_ISL_859035, EPI_ISL_859043 to 859044, EPI_ISL_859049, EPI_ISL_859051, EPI_ISL_859056, EPI_ISL_859061, EPI_ISL_859063, EPI_ISL_859066 to 859067, EPI_ISL_859070, EPI_ISL_859073, EPI_ISL_859075 to 859076, EPI_ISL_859080, EPI_ISL_859082, EPI_ISL_859085 to 859086, EPI_ISL_859089 to 859092, EPI_ISL_859095 to 859097, EPI_ISL_859099, EPI_ISL_859101, EPI_ISL_859103 to 859104, EPI_ISL_859107, EPI_ISL_859109 to 859111, EPI_ISL_859113, EPI_ISL_859115, EPI_ISL_859117, EPI_ISL_859121 to 859122, EPI_ISL_859124, EPI_ISL_859126, EPI_ISL_859128 to 859129, EPI_ISL_859132, EPI_ISL_859134 to 859136, EPI_ISL_859138, EPI_ISL_859141 to 859148, EPI_ISL_859154, EPI_ISL_859161, EPI_ISL_859165, EPI_ISL_859179, EPI_ISL_859181, EPI_ISL_859186, EPI_ISL_859194, EPI_ISL_859198, EPI_ISL_859201 to 859204, EPI_ISL_859206 to 859210, EPI_ISL_859212 to 859213, EPI_ISL_859215 to 859217, EPI_ISL_859220 to 859231, EPI_ISL_859233, EPI_ISL_859235, EPI_ISL_859237 to 859252, EPI_ISL_859257 to 859262, EPI_ISL_859264, EPI_ISL_859266 to 859267, EPI_ISL_859269, EPI_ISL_859271 to 859279, EPI_ISL_859281, EPI_ISL_859283 to 859287, EPI_ISL_859289 to 859293, EPI_ISL_859296, EPI_ISL_859298 to 859300, EPI_ISL_859302, EPI_ISL_859305, EPI_ISL_859307 to 859308, EPI_ISL_859310, EPI_ISL_859314 to 859315, EPI_ISL_859328 to 859421, EPI_ISL_859423 to 859434, EPI_ISL_859436 to 859455, EPI_ISL_859533 to 859539, EPI_ISL_862886 to 863244, EPI_ISL_863251, EPI_ISL_863254, EPI_ISL_863258, EPI_ISL_863268, EPI_ISL_863270 to 863272, EPI_ISL_863274 to 863275, EPI_ISL_863280 to 863281, EPI_ISL_863289 to 863290, EPI_ISL_863299, EPI_ISL_863302, EPI_ISL_863306, EPI_ISL_863310 to 863311, EPI_ISL_863318 to 863319, EPI_ISL_863326, EPI_ISL_863334, EPI_ISL_863338, EPI_ISL_863342 to 863343, EPI_ISL_863346, EPI_ISL_863356 to 863357, EPI_ISL_863360, EPI_ISL_863362 to 863364, EPI_ISL_863377, EPI_ISL_863379, EPI_ISL_863383, EPI_ISL_863387, EPI_ISL_863395 to 863397, EPI_ISL_863401, EPI_ISL_863403 to 863404, EPI_ISL_863406, EPI_ISL_863408, EPI_ISL_863411, EPI_ISL_863414, EPI_ISL_863416, EPI_ISL_863420 to 863421, EPI_ISL_863423 to 863424, EPI_ISL_863426, EPI_ISL_863432, EPI_ISL_863434, EPI_ISL_863437, EPI_ISL_863443, EPI_ISL_863446 to 863448, EPI_ISL_863454, EPI_ISL_863461, EPI_ISL_863467, EPI_ISL_863470, EPI_ISL_863477, EPI_ISL_863479, EPI_ISL_863484, EPI_ISL_863491, EPI_ISL_863503 to 863504, EPI_ISL_863506, EPI_ISL_863514 to 863516, EPI_ISL_863519 to 863520, EPI_ISL_863524, EPI_ISL_863527, EPI_ISL_863529, EPI_ISL_863869 to 863898, EPI_ISL_863900, EPI_ISL_863902 to 863911, EPI_ISL_863914 to 863920, EPI_ISL_863923 to 863955, EPI_ISL_863957 to 863968, EPI_ISL_863970 to 863995, EPI_ISL_863997 to 864004, EPI_ISL_864006 to 864008, EPI_ISL_864010 to 864025, EPI_ISL_864027 to 864035, EPI_ISL_864037 to 864042, EPI_ISL_864044 to 864097, EPI_ISL_864099 to 864123, EPI_ISL_864125 to 864132, EPI_ISL_864134 to 864164, EPI_ISL_864166 to 864168, EPI_ISL_864170 to 864172, EPI_ISL_864174, EPI_ISL_864177 to 864190, EPI_ISL_864192, EPI_ISL_864194 to 864202, EPI_ISL_864226, EPI_ISL_864267, EPI_ISL_864308, EPI_ISL_873961 to 874655, EPI_ISL_874657 to 874675, EPI_ISL_874677 to 874732, EPI_ISL_874734 to 874735, EPI_ISL_874738, EPI_ISL_874740 to 874752, EPI_ISL_874754 to 874774, EPI_ISL_874776 to 874797, EPI_ISL_874799 to 874805, EPI_ISL_874807, EPI_ISL_874809 to 874862, EPI_ISL_874864 to 874895, EPI_ISL_874897, EPI_ISL_874899 to 874904, EPI_ISL_874906 to 874921, EPI_ISL_874923 to 874934, EPI_ISL_874936 to 874942, EPI_ISL_874944 to 874949, EPI_ISL_874951 to 874964, EPI_ISL_874966, EPI_ISL_874968 to 874989, EPI_ISL_877770 to 878151, EPI_ISL_878153 to 878154, EPI_ISL_878156, EPI_ISL_878158, EPI_ISL_878160 to 878161, EPI_ISL_878163 to 878164, EPI_ISL_878166, EPI_ISL_878168 to 878169, EPI_ISL_878171, EPI_ISL_878173 to 878174, EPI_ISL_878176 to 878177, EPI_ISL_878179, EPI_ISL_878181 to 878182, EPI_ISL_878184 to 878185, EPI_ISL_878187 to 878188, EPI_ISL_878190, EPI_ISL_878192 to 878193, EPI_ISL_878195 to 878196, EPI_ISL_878198,

EPI_ISL_878200 to 878201, EPI_ISL_878203 to 878204, EPI_ISL_878206, EPI_ISL_878208, EPI_ISL_878210 to 878211, EPI_ISL_878213 to 878214, EPI_ISL_878216, EPI_ISL_878218 to 878220, EPI_ISL_878222, EPI_ISL_878224 to 878225, EPI_ISL_878227 to 878228, EPI_ISL_878230 to 878231, EPI_ISL_878233, EPI_ISL_878235 to 878236, EPI_ISL_878238, EPI_ISL_878240 to 878241, EPI_ISL_878243 to 878244, EPI_ISL_878246 to 878247, EPI_ISL_878249, EPI_ISL_878251 to 878252, EPI_ISL_878254 to 878255, EPI_ISL_878257, EPI_ISL_878259, EPI_ISL_878261 to 878262, EPI_ISL_878264 to 878265, EPI_ISL_878267, EPI_ISL_878269 to 878270, EPI_ISL_878272 to 878273, EPI_ISL_878275, EPI_ISL_878277 to 878278, EPI_ISL_878280, EPI_ISL_878282 to 878283, EPI_ISL_878285 to 878286, EPI_ISL_878288 to 878289, EPI_ISL_878291, EPI_ISL_878293 to 878294, EPI_ISL_878296 to 878297, EPI_ISL_878299, EPI_ISL_878301 to 878302, EPI_ISL_878304, EPI_ISL_878306 to 878307, EPI_ISL_878309 to 878310, EPI_ISL_878312 to 878313, EPI_ISL_878315, EPI_ISL_878317 to 878318, EPI_ISL_878320 to 878321, EPI_ISL_878323 to 878324, EPI_ISL_878326, EPI_ISL_878328 to 878329, EPI_ISL_878331 to 878332, EPI_ISL_878334 to 878335, EPI_ISL_878337, EPI_ISL_878339 to 878340, EPI_ISL_878409 to 878410, EPI_ISL_878412, EPI_ISL_878414 to 878415, EPI_ISL_878417, EPI_ISL_878419 to 878420, EPI_ISL_878422, EPI_ISL_878424 to 878425, EPI_ISL_878427 to 878428, EPI_ISL_878430, EPI_ISL_878432 to 878433, EPI_ISL_878435 to 878436, EPI_ISL_878438 to 878439, EPI_ISL_878441 to 878442, EPI_ISL_878444, EPI_ISL_878446 to 878447, EPI_ISL_878449, EPI_ISL_878451, EPI_ISL_878453 to 878454, EPI_ISL_878456, EPI_ISL_878458 to 878459, EPI_ISL_878461 to 878462, EPI_ISL_878464, EPI_ISL_878466 to 878467, EPI_ISL_878469, EPI_ISL_878471, EPI_ISL_878473, EPI_ISL_878475 to 878476, EPI_ISL_878478 to 878479, EPI_ISL_878481 to 878482, EPI_ISL_878484, EPI_ISL_878486 to 878487, EPI_ISL_878489 to 878490, EPI_ISL_878493 to 878494, EPI_ISL_878496 to 878497, EPI_ISL_878499, EPI_ISL_878501 to 878502, EPI_ISL_878504 to 878505, EPI_ISL_878507 to 878508, EPI_ISL_878510, EPI_ISL_878512 to 878513, EPI_ISL_878515, EPI_ISL_878517 to 878518, EPI_ISL_878520 to 878521, EPI_ISL_878523, EPI_ISL_878525, EPI_ISL_878527 to 878528, EPI_ISL_878530, EPI_ISL_878532 to 878533, EPI_ISL_878535 to 878536, EPI_ISL_878538, EPI_ISL_878540, EPI_ISL_878542 to 878543, EPI_ISL_878545 to 878546, EPI_ISL_878548, EPI_ISL_878550 to 878551, EPI_ISL_878553, EPI_ISL_878555 to 878556, EPI_ISL_878558 to 878559, EPI_ISL_878561 to 878562, EPI_ISL_878564, EPI_ISL_878566 to 878567, EPI_ISL_878569 to 878570, EPI_ISL_878572 to 878573, EPI_ISL_878575, EPI_ISL_878577 to 878578, EPI_ISL_878580, EPI_ISL_878582 to 878583, EPI_ISL_878585 to 878586, EPI_ISL_878588, EPI_ISL_878590 to 878591, EPI_ISL_878593 to 878594, EPI_ISL_878596, EPI_ISL_878598 to 878599, EPI_ISL_878601 to 878602, EPI_ISL_878604, EPI_ISL_878606 to 878607, EPI_ISL_878609 to 878610, EPI_ISL_878612, EPI_ISL_878614 to 878615, EPI_ISL_878617 to 878618, EPI_ISL_878620 to 878621, EPI_ISL_878623, EPI_ISL_878625 to 878626, EPI_ISL_878628, EPI_ISL_878630 to 878631, EPI_ISL_878633 to 878634, EPI_ISL_878636, EPI_ISL_878638 to 878639, EPI_ISL_878641 to 878642, EPI_ISL_878644, EPI_ISL_878646 to 878647, EPI_ISL_878649 to 878650, EPI_ISL_878652, EPI_ISL_878654 to 878655, EPI_ISL_878657, EPI_ISL_878659 to 878660, EPI_ISL_878662 to 878663, EPI_ISL_878665, EPI_ISL_878667 to 878668, EPI_ISL_878670, EPI_ISL_878672 to 878673, EPI_ISL_878675 to 878676, EPI_ISL_878678, EPI_ISL_878680 to 878681, EPI_ISL_878683, EPI_ISL_878685 to 878686, EPI_ISL_878688 to 878689, EPI_ISL_878691 to 878692, EPI_ISL_878694, EPI_ISL_878696, EPI_ISL_878698 to 878699, EPI_ISL_878701, EPI_ISL_878703 to 878704, EPI_ISL_878706, EPI_ISL_878708 to 878709, EPI_ISL_878711 to 878712, EPI_ISL_878714 to 878715, EPI_ISL_878717 to 878718, EPI_ISL_878720, EPI_ISL_878722 to 878723, EPI_ISL_878725, EPI_ISL_878727, EPI_ISL_878730, EPI_ISL_878741, EPI_ISL_878746, EPI_ISL_878754, EPI_ISL_878777, EPI_ISL_878785, EPI_ISL_878790, EPI_ISL_878804 to 878805, EPI_ISL_878808 to 878809, EPI_ISL_878812, EPI_ISL_878817, EPI_ISL_878819 to 878820, EPI_ISL_878822, EPI_ISL_878825, EPI_ISL_878835 to 878836, EPI_ISL_878840, EPI_ISL_878843 to 878844, EPI_ISL_878849, EPI_ISL_878853, EPI_ISL_878856, EPI_ISL_878859, EPI_ISL_878865 to 878866, EPI_ISL_878868, EPI_ISL_878875 to 878876, EPI_ISL_878878, EPI_ISL_878883, EPI_ISL_878886 to 878888, EPI_ISL_878891, EPI_ISL_878909, EPI_ISL_878913 to 878915, EPI_ISL_878917, EPI_ISL_878920, EPI_ISL_878925 to 878927, EPI_ISL_878929, EPI_ISL_878932, EPI_ISL_878936, EPI_ISL_878938, EPI_ISL_878946 to 878947, EPI_ISL_878955, EPI_ISL_878958 to 878959, EPI_ISL_878961, EPI_ISL_878965 to 878966, EPI_ISL_878970, EPI_ISL_878979, EPI_ISL_878981, EPI_ISL_878983, EPI_ISL_878985, EPI_ISL_878992 to 878994, EPI_ISL_878999 to 879000, EPI_ISL_879003, EPI_ISL_879007 to 879008, EPI_ISL_879015, EPI_ISL_879018, EPI_ISL_879026, EPI_ISL_879033, EPI_ISL_879040, EPI_ISL_879056, EPI_ISL_879061, EPI_ISL_879066, EPI_ISL_879070, EPI_ISL_879072 to 879073, EPI_ISL_879077, EPI_ISL_879079, EPI_ISL_879081, EPI_ISL_879083, EPI_ISL_879088, EPI_ISL_879091, EPI_ISL_879097 to 879101, EPI_ISL_879103, EPI_ISL_879108 to 879111, EPI_ISL_879113 to 879114, EPI_ISL_879116 to 879118, EPI_ISL_879120 to 879121, EPI_ISL_879124 to 879126, EPI_ISL_879128 to 879132, EPI_ISL_879134, EPI_ISL_879137 to 879138, EPI_ISL_879140, EPI_ISL_879144 to 879147, EPI_ISL_879149, EPI_ISL_879151 to 879152, EPI_ISL_879154, EPI_ISL_879157, EPI_ISL_879159 to 879162, EPI_ISL_879164, EPI_ISL_879167, EPI_ISL_879169, EPI_ISL_879171, EPI_ISL_879173, EPI_ISL_879181, EPI_ISL_879185, EPI_ISL_879187 to 879188, EPI_ISL_879191, EPI_ISL_879193 to 879195, EPI_ISL_879199, EPI_ISL_879201 to 879203, EPI_ISL_879206 to 879207, EPI_ISL_879212, EPI_ISL_879214, EPI_ISL_879218 to 879220, EPI_ISL_879224 to 879227, EPI_ISL_879229 to 879231, EPI_ISL_879234 to 879235, EPI_ISL_879237, EPI_ISL_879239, EPI_ISL_879241, EPI_ISL_879243 to 879244, EPI_ISL_879247 to 879250, EPI_ISL_879252 to 879254, EPI_ISL_879256, EPI_ISL_879258 to 879259, EPI_ISL_879262 to 879263, EPI_ISL_879265 to 879266, EPI_ISL_879268, EPI_ISL_879270 to 879271, EPI_ISL_879273 to 879274, EPI_ISL_879276 to 879279, EPI_ISL_879284, EPI_ISL_879286, EPI_ISL_879290, EPI_ISL_879296 to 879300, EPI_ISL_879302 to 879303, EPI_ISL_879305 to 879308, EPI_ISL_879313 to 879314, EPI_ISL_879316, EPI_ISL_879318, EPI_ISL_879322 to 879324, EPI_ISL_879329, EPI_ISL_879331, EPI_ISL_879333, EPI_ISL_879338 to 879348, EPI_ISL_879351, EPI_ISL_879354, EPI_ISL_879356 to 879363, EPI_ISL_879367, EPI_ISL_879371, EPI_ISL_879374 to 879375, EPI_ISL_879377, EPI_ISL_879382, EPI_ISL_879384, EPI_ISL_879386, EPI_ISL_879392 to 879393, EPI_ISL_879415 to 879418, EPI_ISL_879420, EPI_ISL_879427, EPI_ISL_879433 to 879434, EPI_ISL_879444, EPI_ISL_879446, EPI_ISL_879451 to 879452, EPI_ISL_879456, EPI_ISL_879460, EPI_ISL_879462, EPI_ISL_879466, EPI_ISL_879473, EPI_ISL_879475 to 879476, EPI_ISL_879480, EPI_ISL_879483, EPI_ISL_879486 to 879487, EPI_ISL_879493, EPI_ISL_879497, EPI_ISL_879501, EPI_ISL_879504 to 879505, EPI_ISL_879510, EPI_ISL_879515, EPI_ISL_879518 to 879520, EPI_ISL_879522, EPI_ISL_879524, EPI_ISL_879534 to 879535, EPI_ISL_879542, EPI_ISL_879546 to 879547, EPI_ISL_879550, EPI_ISL_879556, EPI_ISL_879563 to 879565, EPI_ISL_879578, EPI_ISL_879582, EPI_ISL_879589, EPI_ISL_879593, EPI_ISL_879596, EPI_ISL_879602 to 879604, EPI_ISL_879614, EPI_ISL_879616 to 879617, EPI_ISL_879619, EPI_ISL_879627, EPI_ISL_879629, EPI_ISL_879631, EPI_ISL_879641, EPI_ISL_879646 to 879647, EPI_ISL_879649, EPI_ISL_879665 to 879666, EPI_ISL_879672, EPI_ISL_879674, EPI_ISL_879682 to 879683, EPI_ISL_879687, EPI_ISL_879692 to 879693, EPI_ISL_879695, EPI_ISL_879700, EPI_ISL_879702, EPI_ISL_879714, EPI_ISL_879718 to 879719, EPI_ISL_879721, EPI_ISL_879726, EPI_ISL_879728, EPI_ISL_879736 to 879737, EPI_ISL_879740, EPI_ISL_879742, EPI_ISL_879744, EPI_ISL_879747 to 879748, EPI_ISL_879750 to 879753, EPI_ISL_879755 to 879762, EPI_ISL_879765 to 879767, EPI_ISL_879775, EPI_ISL_879778, EPI_ISL_879780, EPI_ISL_879783 to 879785, EPI_ISL_879787, EPI_ISL_879789, EPI_ISL_879791 to 879794, EPI_ISL_879800 to 879803, EPI_ISL_879808, EPI_ISL_879810 to 879811, EPI_ISL_879813 to 879814, EPI_ISL_879817, EPI_ISL_879819, EPI_ISL_879821 to 879822, EPI_ISL_879825 to 879827, EPI_ISL_879829, EPI_ISL_879831, EPI_ISL_879836 to 879838, EPI_ISL_879841 to 879844, EPI_ISL_879846, EPI_ISL_879849, EPI_ISL_879851, EPI_ISL_879854 to 879856, EPI_ISL_879858 to 879859, EPI_ISL_879863, EPI_ISL_879865 to 879866, EPI_ISL_879868 to 879873, EPI_ISL_879875, EPI_ISL_879878 to 879879, EPI_ISL_879882, EPI_ISL_879884 to 879888, EPI_ISL_879894 to 879895, EPI_ISL_879897 to 879898, EPI_ISL_879901, EPI_ISL_879903, EPI_ISL_879905 to 879907, EPI_ISL_879912 to 879916, EPI_ISL_879918 to 879919, EPI_ISL_879922 to 879923, EPI_ISL_879925, EPI_ISL_879927, EPI_ISL_879931, EPI_ISL_879933, EPI_ISL_879936, EPI_ISL_879938, EPI_ISL_879948 to 879949, EPI_ISL_879955, EPI_ISL_879957, EPI_ISL_879965 to 879966, EPI_ISL_879968, EPI_ISL_879970 to 879971, EPI_ISL_879974, EPI_ISL_879976, EPI_ISL_879978 to 879979, EPI_ISL_879981, EPI_ISL_879984, EPI_ISL_879986, EPI_ISL_879988, EPI_ISL_879991, EPI_ISL_879997, EPI_ISL_879999, EPI_ISL_880002, EPI_ISL_880007 to 880008, EPI_ISL_880010, EPI_ISL_880016, EPI_ISL_880018, EPI_ISL_880021, EPI_ISL_880023, EPI_ISL_880028, EPI_ISL_880030 to 880031, EPI_ISL_880036, EPI_ISL_880041, EPI_ISL_880043, EPI_ISL_880052, EPI_ISL_880055, EPI_ISL_880057, EPI_ISL_880062, EPI_ISL_880064, EPI_ISL_880067, EPI_ISL_880072, EPI_ISL_880083 to 880084, EPI_ISL_880086, EPI_ISL_880088 to 880089, EPI_ISL_880091, EPI_ISL_880093 to 880094, EPI_ISL_880096 to 880097, EPI_ISL_880099 to 880100, EPI_ISL_880102, EPI_ISL_880104 to 880105, EPI_ISL_880107 to 880108, EPI_ISL_880110, EPI_ISL_880112 to 880113, EPI_ISL_880115 to 880116, EPI_ISL_880118, EPI_ISL_880120 to 880121, EPI_ISL_880123 to 880124, EPI_ISL_880126 to 880127, EPI_ISL_880129, EPI_ISL_880131 to 880132, EPI_ISL_880134, EPI_ISL_880136 to 880137, EPI_ISL_880139, EPI_ISL_880210 to 880211, EPI_ISL_880213 to 880214, EPI_ISL_880216 to 880217, EPI_ISL_880219, EPI_ISL_880221 to 880222, EPI_ISL_880224, EPI_ISL_880226 to 880227, EPI_ISL_880229 to 880230, EPI_ISL_880232, EPI_ISL_880234 to 880235, EPI_ISL_880237 to 880238, EPI_ISL_880240, EPI_ISL_880242 to 880243, EPI_ISL_880245, EPI_ISL_880247 to 880248, EPI_ISL_880250, EPI_ISL_880252, EPI_ISL_880254 to 880255, EPI_ISL_880257 to 880258, EPI_ISL_880260, EPI_ISL_880262, EPI_ISL_880264 to 880265, EPI_ISL_880267, EPI_ISL_880269 to 880270, EPI_ISL_880272 to 880273, EPI_ISL_880275 to 880276, EPI_ISL_880278, EPI_ISL_880280 to 880281, EPI_ISL_880283, EPI_ISL_880285 to 880286, EPI_ISL_880288 to 880289, EPI_ISL_880291, EPI_ISL_880293, EPI_ISL_880295 to 880296, EPI_ISL_880298 to 880299, EPI_ISL_880301 to 880302, EPI_ISL_880304, EPI_ISL_880306 to 880307, EPI_ISL_880309, EPI_ISL_880311 to 880312, EPI_ISL_880314 to 880315, EPI_ISL_880317 to 880318, EPI_ISL_880320, EPI_ISL_880322 to 880323, EPI_ISL_880325, EPI_ISL_880327 to 880328, EPI_ISL_880330 to 880583, EPI_ISL_880936 to 881617, EPI_ISL_881623 to 882608, EPI_ISL_885155 to 886156, EPI_ISL_891278, EPI_ISL_891280, EPI_ISL_891285, EPI_ISL_891295, EPI_ISL_891299, EPI_ISL_891305, EPI_ISL_891311 to 891313, EPI_ISL_891317 to 891319, EPI_ISL_891323 to 891324, EPI_ISL_891326, EPI_ISL_891334, EPI_ISL_891337, EPI_ISL_891340, EPI_ISL_891342, EPI_ISL_891344, EPI_ISL_891349, EPI_ISL_891352, EPI_ISL_891357, EPI_ISL_891360, EPI_ISL_891364, EPI_ISL_891371, EPI_ISL_891374, EPI_ISL_891376 to 891378, EPI_ISL_891383 to 891386, EPI_ISL_891392, EPI_ISL_891400 to 891401, EPI_ISL_891403 to 891404, EPI_ISL_891411, EPI_ISL_891416 to 891417, EPI_ISL_891419, EPI_ISL_891421, EPI_ISL_891423, EPI_ISL_891438, EPI_ISL_891444, EPI_ISL_891448, EPI_ISL_891451, EPI_ISL_891454 to 891455, EPI_ISL_891458 to 891460, EPI_ISL_891463, EPI_ISL_891478, EPI_ISL_891484 to 891485, EPI_ISL_891489 to 891490, EPI_ISL_891496, EPI_ISL_891499, EPI_ISL_891503, EPI_ISL_891507, EPI_ISL_891511, EPI_ISL_891514, EPI_ISL_891520, EPI_ISL_891522, EPI_ISL_891525, EPI_ISL_891527 to 891529, EPI_ISL_891531, EPI_ISL_891536, EPI_ISL_891544 to 891545, EPI_ISL_891547 to 891548, EPI_ISL_891555, EPI_ISL_891560, EPI_ISL_891563 to 891564, EPI_ISL_891569, EPI_ISL_891572, EPI_ISL_891582, EPI_ISL_891586, EPI_ISL_891596, EPI_ISL_891599 to 891600, EPI_ISL_891602, EPI_ISL_891606, EPI_ISL_891615 to 891616, EPI_ISL_891623, EPI_ISL_891625, EPI_ISL_891629 to 891630, EPI_ISL_891633 to 891634, EPI_ISL_891640, EPI_ISL_891643, EPI_ISL_891647, EPI_ISL_891650, EPI_ISL_891659, EPI_ISL_891661 to 891662, EPI_ISL_891670, EPI_ISL_891672 to 891673, EPI_ISL_891677, EPI_ISL_891680 to 891681, EPI_ISL_891683, EPI_ISL_891687, EPI_ISL_891704, EPI_ISL_891710, EPI_ISL_891713 to 891714, EPI_ISL_891718 to 891719, EPI_ISL_891723 to 891724, EPI_ISL_891726, EPI_ISL_891729, EPI_ISL_891735 to 891736, EPI_ISL_891738, EPI_ISL_891742, EPI_ISL_891746, EPI_ISL_891751, EPI_ISL_891753, EPI_ISL_891757, EPI_ISL_891760, EPI_ISL_891762, EPI_ISL_891765 to 891766, EPI_ISL_891773 to 891777, EPI_ISL_891780, EPI_ISL_891786 to 891789, EPI_ISL_891794, EPI_ISL_891804, EPI_ISL_891807, EPI_ISL_891811, EPI_ISL_891813, EPI_ISL_891822, EPI_ISL_891824, EPI_ISL_891833, EPI_ISL_891838 to 891839, EPI_ISL_891843, EPI_ISL_891847 to 891848, EPI_ISL_891850, EPI_ISL_891856, EPI_ISL_891858, EPI_ISL_891860, EPI_ISL_891863 to 891864, EPI_ISL_891866 to 891867, EPI_ISL_891870, EPI_ISL_891878, EPI_ISL_891887, EPI_ISL_891895, EPI_ISL_891899 to 892200, EPI_ISL_892202 to 892209, EPI_ISL_892212 to 892213, EPI_ISL_892215 to 892216, EPI_ISL_892218 to 892223, EPI_ISL_892226, EPI_ISL_892230, EPI_ISL_892232 to 892233, EPI_ISL_892235, EPI_ISL_892237 to 892240, EPI_ISL_892242 to 892243, EPI_ISL_907371 to 907377, EPI_ISL_907380, EPI_ISL_907382 to 907383, EPI_ISL_907387 to 907392, EPI_ISL_907396, EPI_ISL_907399 to 907400, EPI_ISL_907403 to 907404, EPI_ISL_907406 to 907407, EPI_ISL_907409 to 907414, EPI_ISL_907416 to 907417, EPI_ISL_907422, EPI_ISL_907424, EPI_ISL_907427 to 907429, EPI_ISL_907432, EPI_ISL_907437 to 907438, EPI_ISL_907440 to 907441, EPI_ISL_907443 to 907444, EPI_ISL_907446 to 907450, EPI_ISL_907452, EPI_ISL_907457 to 907460, EPI_ISL_907462 to 907464, EPI_ISL_907466 to 907468, EPI_ISL_907470 to 907471, EPI_ISL_907477 to 907480, EPI_ISL_907482, EPI_ISL_907484 to 907485, EPI_ISL_907489, EPI_ISL_907491 to 907493, EPI_ISL_907496 to 907497, EPI_ISL_907500, EPI_ISL_907503, EPI_ISL_907505, EPI_ISL_907510 to 907512, EPI_ISL_907516 to 907519, EPI_ISL_907521 to 907523, EPI_ISL_907526, EPI_ISL_907528 to 907529, EPI_ISL_907532 to 907533, EPI_ISL_907536 to 907538, EPI_ISL_907542 to 907543, EPI_ISL_907546, EPI_ISL_907549, EPI_ISL_907551 to 907552, EPI_ISL_907557, EPI_ISL_907559, EPI_ISL_907561, EPI_ISL_907563, EPI_ISL_907567 to 907568, EPI_ISL_907571, EPI_ISL_907573, EPI_ISL_907576, EPI_ISL_907578 to 907580, EPI_ISL_907582 to 907584, EPI_ISL_907587, EPI_ISL_907589 to 907590, EPI_ISL_907594, EPI_ISL_907596, EPI_ISL_907598, EPI_ISL_907600 to 907601, EPI_ISL_907605 to 907607, EPI_ISL_907610 to 907612, EPI_ISL_907615 to 907616, EPI_ISL_907618, EPI_ISL_907621 to 907623, EPI_ISL_907626, EPI_ISL_907628, EPI_ISL_907630 to 907631, EPI_ISL_907636, EPI_ISL_907640 to 907642, EPI_ISL_907645, EPI_ISL_907647 to 907648, EPI_ISL_907651 to 907652, EPI_ISL_907654, EPI_ISL_907658 to 907659, EPI_ISL_907661, EPI_ISL_907664, EPI_ISL_907667, EPI_ISL_907672 to 907673, EPI_ISL_907675 to 907677, EPI_ISL_907680, EPI_ISL_907684, EPI_ISL_907686, EPI_ISL_907689 to 907690, EPI_ISL_907692 to 907697, EPI_ISL_907699 to 907701, EPI_ISL_907704, EPI_ISL_907709 to 907715, EPI_ISL_907717 to 907720, EPI_ISL_907724 to 907730, EPI_ISL_907732 to 907734, EPI_ISL_907737, EPI_ISL_907739 to 907744, EPI_ISL_907746, EPI_ISL_907748, EPI_ISL_907751 to 907754, EPI_ISL_907758 to 907759, EPI_ISL_907761 to 907762, EPI_ISL_907764, EPI_ISL_907766, EPI_ISL_907768 to 907770, EPI_ISL_907774 to 907775, EPI_ISL_907777, EPI_ISL_907779 to 907785, EPI_ISL_907790 to 907792, EPI_ISL_907798 to 907799, EPI_ISL_907803 to 907804, EPI_ISL_907806 to 907808, EPI_ISL_907810, EPI_ISL_907815 to 907816, EPI_ISL_907818 to 907819, EPI_ISL_907822 to 907823, EPI_ISL_907825, EPI_ISL_907827 to 907831, EPI_ISL_907836, EPI_ISL_907838 to 907846, EPI_ISL_907848 to 907850, EPI_ISL_907853, EPI_ISL_907856 to 907859, EPI_ISL_907861 to 907862, EPI_ISL_907864, EPI_ISL_907866, EPI_ISL_907869 to 907871, EPI_ISL_907873, EPI_ISL_907876, EPI_ISL_907880, EPI_ISL_907883 to 907888, EPI_ISL_907892, EPI_ISL_907896 to 907897, EPI_ISL_907900 to 907901, EPI_ISL_907904 to 907910, EPI_ISL_907913, EPI_ISL_907916 to 907920, EPI_ISL_907922, EPI_ISL_907924, EPI_ISL_907927 to 907930, EPI_ISL_907932 to 907933, EPI_ISL_907935, EPI_ISL_907937 to 907939, EPI_ISL_907941 to 907942, EPI_ISL_907947, EPI_ISL_907950 to 907952, EPI_ISL_907954 to 907955, EPI_ISL_907957 to 907959, EPI_ISL_907961 to 907966, EPI_ISL_907968, EPI_ISL_907971, EPI_ISL_907977 to 907978, EPI_ISL_907980, EPI_ISL_907983, EPI_ISL_907985 to 907986, EPI_ISL_907990 to 907993, EPI_ISL_907996 to 907997, EPI_ISL_907999 to 908001, EPI_ISL_908004 to 908006, EPI_ISL_908008, EPI_ISL_908012, EPI_ISL_908014 to 908015, EPI_ISL_908018 to 908020, EPI_ISL_908023 to 908024, EPI_ISL_908029, EPI_ISL_908031 to 908032, EPI_ISL_908268 to 908274, EPI_ISL_908276 to 908283, EPI_ISL_908285 to 908288, EPI_ISL_908292 to 908294, EPI_ISL_908296 to 908297, EPI_ISL_908299 to 908301, EPI_ISL_908304 to 908306, EPI_ISL_908309 to 908316, EPI_ISL_908318 to 908320, EPI_ISL_908322, EPI_ISL_908324 to 908325, EPI_ISL_908327 to 908328, EPI_ISL_908330 to 908333, EPI_ISL_908336, EPI_ISL_908339, EPI_ISL_908341 to 908347, EPI_ISL_908349 to 908352, EPI_ISL_908354 to 908356, EPI_ISL_908358 to 908359, EPI_ISL_908361 to 908364, EPI_ISL_908367 to 908379, EPI_ISL_908381 to 908382, EPI_ISL_908384 to 908386, EPI_ISL_908388 to 908392, EPI_ISL_908395 to 908402, EPI_ISL_908404, EPI_ISL_908406 to 908410, EPI_ISL_908413 to 908415, EPI_ISL_908417 to 908418, EPI_ISL_908420, EPI_ISL_908423 to 908428, EPI_ISL_908430, EPI_ISL_908432, EPI_ISL_908434 to 908441, EPI_ISL_908445 to 908446, EPI_ISL_908448 to 908450, EPI_ISL_908452 to 908454, EPI_ISL_908456 to 908461, EPI_ISL_908463 to 908469, EPI_ISL_908472 to 908480, EPI_ISL_908482 to 908485, EPI_ISL_908487, EPI_ISL_908490 to 908494, EPI_ISL_908497 to 908499, EPI_ISL_908501 to 908506, EPI_ISL_908510 to 908511, EPI_ISL_908513 to 908515, EPI_ISL_908517 to 908520, EPI_ISL_908523 to 908524, EPI_ISL_908526 to 908529, EPI_ISL_908531 to 908535, EPI_ISL_908537, EPI_ISL_908539 to 908546, EPI_ISL_908548 to 908550, EPI_ISL_908552 to 908556, EPI_ISL_908560 to 908563, EPI_ISL_908565 to 908567, EPI_ISL_908569 to 908572, EPI_ISL_908574 to 908581, EPI_ISL_908583 to 908588, EPI_ISL_908590, EPI_ISL_908592 to 908594, EPI_ISL_908596, EPI_ISL_909260, EPI_ISL_909270, EPI_ISL_909273, EPI_ISL_909275, EPI_ISL_909281, EPI_ISL_909285, EPI_ISL_909292, EPI_ISL_909294, EPI_ISL_909297, EPI_ISL_909302, EPI_ISL_909309, EPI_ISL_909311 to 909312, EPI_ISL_909314, EPI_ISL_909316, EPI_ISL_909318, EPI_ISL_909321 to 909322, EPI_ISL_909326, EPI_ISL_909328, EPI_ISL_909334, EPI_ISL_909339, EPI_ISL_909341, EPI_ISL_909343 to 909344, EPI_ISL_909358 to 909359, EPI_ISL_909364, EPI_ISL_909366 to 909368, EPI_ISL_909370, EPI_ISL_909372 to 909373, EPI_ISL_909380 to 909381, EPI_ISL_909385, EPI_ISL_909388 to 909389, EPI_ISL_909397, EPI_ISL_909401, EPI_ISL_909409, EPI_ISL_909414, EPI_ISL_909416 to 909417, EPI_ISL_909421, EPI_ISL_909424 to 909426, EPI_ISL_909440, EPI_ISL_909446 to 909447, EPI_ISL_909450, EPI_ISL_909453, EPI_ISL_909456 to 909457, EPI_ISL_909465, EPI_ISL_909467, EPI_ISL_909471, EPI_ISL_909476, EPI_ISL_909478, EPI_ISL_909482, EPI_ISL_909487 to 909488, EPI_ISL_909503 to 909504, EPI_ISL_909513, EPI_ISL_909518, EPI_ISL_909526, EPI_ISL_909531, EPI_ISL_909536, EPI_ISL_909539, EPI_ISL_909545 to 909546, EPI_ISL_909552 to 909553, EPI_ISL_909561, EPI_ISL_909564, EPI_ISL_909571 to 909573, EPI_ISL_909575, EPI_ISL_909577 to 909578, EPI_ISL_909580 to 909581, EPI_ISL_909586, EPI_ISL_909594 to 909651, EPI_ISL_912215 to 912218, EPI_ISL_912220 to 912223, EPI_ISL_912226, EPI_ISL_915440 to 916179, EPI_ISL_916184, EPI_ISL_916186, EPI_ISL_916195, EPI_ISL_916197 to 916199, EPI_ISL_916204, EPI_ISL_916206, EPI_ISL_916214 to 916215, EPI_ISL_916217 to 916221, EPI_ISL_916223 to 916224, EPI_ISL_916230, EPI_ISL_916235, EPI_ISL_916239 to 916240, EPI_ISL_916251, EPI_ISL_916253, EPI_ISL_916255, EPI_ISL_916257, EPI_ISL_916259, EPI_ISL_916265, EPI_ISL_916267, EPI_ISL_916272, EPI_ISL_916282, EPI_ISL_916286, EPI_ISL_916289, EPI_ISL_916293 to 916294, EPI_ISL_916298, EPI_ISL_916300, EPI_ISL_916302, EPI_ISL_916309, EPI_ISL_916311, EPI_ISL_916317, EPI_ISL_916319, EPI_ISL_916324, EPI_ISL_916326, EPI_ISL_916330, EPI_ISL_916337, EPI_ISL_916351, EPI_ISL_916353, EPI_ISL_916356, EPI_ISL_916359, EPI_ISL_916361, EPI_ISL_916385 to 916387, EPI_ISL_916389, EPI_ISL_916393, EPI_ISL_916395 to 916396, EPI_ISL_916398, EPI_ISL_916402, EPI_ISL_916404, EPI_ISL_916418, EPI_ISL_916422 to 916424, EPI_ISL_916426, EPI_ISL_916429, EPI_ISL_916448, EPI_ISL_916452, EPI_ISL_916465 to 916466, EPI_ISL_916468 to 916469, EPI_ISL_916471, EPI_ISL_916474, EPI_ISL_916482 to 916483, EPI_ISL_916488, EPI_ISL_916490, EPI_ISL_916498, EPI_ISL_916501, EPI_ISL_916622 to 916624, EPI_ISL_916627 to 916632, EPI_ISL_916634 to 916644, EPI_ISL_916646 to 916647, EPI_ISL_916650 to 916660, EPI_ISL_916663 to 916667, EPI_ISL_916669 to 916674, EPI_ISL_916676 to 916681, EPI_ISL_916683, EPI_ISL_916685 to 916687, EPI_ISL_916689 to 916693, EPI_ISL_916695, EPI_ISL_916697 to 916703, EPI_ISL_916706 to 916710, EPI_ISL_916712, EPI_ISL_916714 to 916715, EPI_ISL_916717 to 916727, EPI_ISL_916729, EPI_ISL_916731 to 916735, EPI_ISL_916737, EPI_ISL_916739 to 916757, EPI_ISL_916759 to 916760, EPI_ISL_916762 to 916770, EPI_ISL_916772 to 916781, EPI_ISL_916783, EPI_ISL_916785 to 916792, EPI_ISL_916794 to 916799, EPI_ISL_916801, EPI_ISL_916803 to 916808, EPI_ISL_916811, EPI_ISL_916813 to 916814, EPI_ISL_916816 to 916819, EPI_ISL_916822 to 916824, EPI_ISL_916826 to 916832, EPI_ISL_916835 to 916837, EPI_ISL_916839, EPI_ISL_916841, EPI_ISL_916843 to 916844, EPI_ISL_916846, EPI_ISL_916848 to 916849, EPI_ISL_916851 to 916854, EPI_ISL_916856 to 916868, EPI_ISL_916870 to 916878, EPI_ISL_916880 to 916881, EPI_ISL_916883 to 916886, EPI_ISL_916888 to 916890, EPI_ISL_916892 to 916893, EPI_ISL_916895, EPI_ISL_916897, EPI_ISL_916901 to 916904, EPI_ISL_916906, EPI_ISL_916910, EPI_ISL_916913 to 916915, EPI_ISL_916917, EPI_ISL_916919 to 916922, EPI_ISL_916924 to 916939, EPI_ISL_916941, EPI_ISL_916943 to 916944, EPI_ISL_916947, EPI_ISL_916950, EPI_ISL_916952, EPI_ISL_916955, EPI_ISL_916957 to 916960, EPI_ISL_916962 to 916966, EPI_ISL_916969, EPI_ISL_916971 to 916973, EPI_ISL_916975, EPI_ISL_916978, EPI_ISL_916980, EPI_ISL_916982 to 916983, EPI_ISL_916986, EPI_ISL_916988 to 916990, EPI_ISL_916992 to 916997, EPI_ISL_917000 to 917001, EPI_ISL_917003 to 917008, EPI_ISL_917010 to 917011, EPI_ISL_917015 to 917018, EPI_ISL_917020, EPI_ISL_917023, EPI_ISL_917027 to 917028, EPI_ISL_917030, EPI_ISL_917033 to 917035, EPI_ISL_917037, EPI_ISL_917039 to 917041, EPI_ISL_917043 to 917046, EPI_ISL_917048, EPI_ISL_917050 to 917052, EPI_ISL_917056 to 917062, EPI_ISL_917064 to 917066, EPI_ISL_917068 to 917069, EPI_ISL_917072 to 917073, EPI_ISL_917078, EPI_ISL_917080, EPI_ISL_917084 to 917085, EPI_ISL_917087, EPI_ISL_917089 to 917090, EPI_ISL_917092, EPI_ISL_917094 to 917095, EPI_ISL_917097 to 917098, EPI_ISL_917100 to 917103, EPI_ISL_917106, EPI_ISL_917108 to 917109, EPI_ISL_917111, EPI_ISL_917113, EPI_ISL_917115, EPI_ISL_917117 to 917119, EPI_ISL_917121 to 917124, EPI_ISL_917126 to 917127, EPI_ISL_917130 to 917132, EPI_ISL_917134 to 917138, EPI_ISL_917140, EPI_ISL_917142, EPI_ISL_917146 to 917147, EPI_ISL_917150, EPI_ISL_917152 to 917155, EPI_ISL_917157 to 917158, EPI_ISL_917162, EPI_ISL_917164 to 917165, EPI_ISL_917167 to 917843, EPI_ISL_918060 to 918061, EPI_ISL_918125, EPI_ISL_931586 to 932085, EPI_ISL_932087 to 932250, EPI_ISL_932254, EPI_ISL_932261, EPI_ISL_932264, EPI_ISL_932268, EPI_ISL_932273 to 932274, EPI_ISL_932284 to 932285, EPI_ISL_932287, EPI_ISL_932290, EPI_ISL_932292, EPI_ISL_932297, EPI_ISL_932300, EPI_ISL_932303, EPI_ISL_932310, EPI_ISL_932313, EPI_ISL_932325, EPI_ISL_932334, EPI_ISL_932345, EPI_ISL_932348, EPI_ISL_932357, EPI_ISL_932371, EPI_ISL_932376, EPI_ISL_932388, EPI_ISL_932393, EPI_ISL_932425, EPI_ISL_932433, EPI_ISL_932436, EPI_ISL_932440, EPI_ISL_932444, EPI_ISL_932453, EPI_ISL_932455, EPI_ISL_932457 to 932458, EPI_ISL_932463, EPI_ISL_932470, EPI_ISL_932473, EPI_ISL_932475, EPI_ISL_932479 to 932480, EPI_ISL_932486, EPI_ISL_932489, EPI_ISL_932502 to 932503, EPI_ISL_932511 to 932512, EPI_ISL_932515, EPI_ISL_932517, EPI_ISL_932522, EPI_ISL_932530, EPI_ISL_932532, EPI_ISL_932539, EPI_ISL_932543, EPI_ISL_932552, EPI_ISL_932567, EPI_ISL_932599, EPI_ISL_932607, EPI_ISL_932620, EPI_ISL_932640, EPI_ISL_932645, EPI_ISL_932647, EPI_ISL_932661, EPI_ISL_932668, EPI_ISL_932670, EPI_ISL_932673, EPI_ISL_932690, EPI_ISL_932696, EPI_ISL_932704, EPI_ISL_932709, EPI_ISL_932725, EPI_ISL_932734, EPI_ISL_932743, EPI_ISL_932749, EPI_ISL_932753,

EPI_ISL_932760, EPI_ISL_932768, EPI_ISL_932791, EPI_ISL_932800, EPI_ISL_932814, EPI_ISL_932817 to 932818, EPI_ISL_932834 to 932835, EPI_ISL_932840, EPI_ISL_932844, EPI_ISL_932862, EPI_ISL_932866 to 932868, EPI_ISL_932876, EPI_ISL_932878, EPI_ISL_932881 to 932883, EPI_ISL_932889 to 932891,
EPI_ISL_932893, EPI_ISL_932900 to 932902, EPI_ISL_932906 to 932907, EPI_ISL_932911 to 932912, EPI_ISL_932922 to 932924, EPI_ISL_932927, EPI_ISL_932935, EPI_ISL_932937 to 932938, EPI_ISL_932940, EPI_ISL_932942 to 932943, EPI_ISL_932947, EPI_ISL_932952, EPI_ISL_932956, EPI_ISL_932964,
EPI_ISL_932972 to 932973, EPI_ISL_932975 to 932976, EPI_ISL_932979, EPI_ISL_932981 to 932984, EPI_ISL_932988, EPI_ISL_932990, EPI_ISL_932993, EPI_ISL_933001, EPI_ISL_933003, EPI_ISL_933010, EPI_ISL_933013, EPI_ISL_933015, EPI_ISL_933017, EPI_ISL_933024 to 933026, EPI_ISL_933029,
EPI_ISL_933031, EPI_ISL_933041, EPI_ISL_933045, EPI_ISL_933049, EPI_ISL_933052, EPI_ISL_933059, EPI_ISL_933062, EPI_ISL_933064, EPI_ISL_933066, EPI_ISL_933083 to 933084, EPI_ISL_933086 to 933087, EPI_ISL_933089 to 933090, EPI_ISL_933092 to 933095, EPI_ISL_933097 to 933098, EPI_ISL_933102 to
933106, EPI_ISL_933111 to 933112, EPI_ISL_937545 to 937885, EPI_ISL_938935 to 938943, EPI_ISL_939604, EPI_ISL_939607 to 939608, EPI_ISL_945088, EPI_ISL_945099, EPI_ISL_945113, EPI_ISL_945117, EPI_ISL_945160, EPI_ISL_945201, EPI_ISL_945204, EPI_ISL_945235, EPI_ISL_945240, EPI_ISL_945252,
EPI_ISL_945282, EPI_ISL_945289, EPI_ISL_945306, EPI_ISL_945310, EPI_ISL_945323, EPI_ISL_945335, EPI_ISL_945343, EPI_ISL_945361, EPI_ISL_945374 to 945546, EPI_ISL_945548 to 945714, EPI_ISL_946421 to 946912, EPI_ISL_946914 to 947106, EPI_ISL_947406 to 947677, EPI_ISL_947679 to 948099,
EPI_ISL_948449 to 948786, EPI_ISL_956422 to 956426, EPI_ISL_956428 to 956429, EPI_ISL_956431 to 956434, EPI_ISL_956436 to 956448, EPI_ISL_956450 to 956451, EPI_ISL_956453 to 956454, EPI_ISL_956457 to 956462, EPI_ISL_956466, EPI_ISL_956468 to 956469, EPI_ISL_956472, EPI_ISL_956474 to 956481,
EPI_ISL_956483, EPI_ISL_956485, EPI_ISL_956487 to 956488, EPI_ISL_956490, EPI_ISL_956492 to 956494, EPI_ISL_956496 to 956497, EPI_ISL_956499 to 956500, EPI_ISL_956503 to 956504, EPI_ISL_956507 to 956511, EPI_ISL_956513 to 956514, EPI_ISL_956516 to 956521, EPI_ISL_956523 to 956525, EPI_ISL_956527
to 956528, EPI_ISL_956530 to 956536, EPI_ISL_956538, EPI_ISL_956540, EPI_ISL_956542, EPI_ISL_956544 to 956545, EPI_ISL_956547 to 956548, EPI_ISL_956550 to 956555, EPI_ISL_956557 to 956558, EPI_ISL_956560 to 956566, EPI_ISL_956568 to 956571, EPI_ISL_956573 to 956574, EPI_ISL_956576 to 956577,
EPI_ISL_956579 to 956597, EPI_ISL_956599, EPI_ISL_956601 to 956603, EPI_ISL_956606 to 956610, EPI_ISL_956614 to 956619, EPI_ISL_956623 to 956628, EPI_ISL_956631, EPI_ISL_956633 to 956645, EPI_ISL_956647, EPI_ISL_956650 to 956653, EPI_ISL_956655 to 956665, EPI_ISL_956667, EPI_ISL_956669 to 956674,
EPI_ISL_956676, EPI_ISL_956678 to 956680, EPI_ISL_956682 to 956684, EPI_ISL_956686, EPI_ISL_956688, EPI_ISL_956690 to 956701, EPI_ISL_956703 to 956704, EPI_ISL_956706 to 956708, EPI_ISL_956710 to 956715, EPI_ISL_956718 to 956721, EPI_ISL_956723 to 956724, EPI_ISL_956726, EPI_ISL_956728 to 956738,
EPI_ISL_956740 to 956748, EPI_ISL_956750 to 956751, EPI_ISL_956754, EPI_ISL_956756 to 956759, EPI_ISL_956761 to 956762, EPI_ISL_956764, EPI_ISL_958566, EPI_ISL_958917 to 959266, EPI_ISL_959268 to 959270, EPI_ISL_963061, EPI_ISL_963106, EPI_ISL_963120, EPI_ISL_963296, EPI_ISL_963321,
EPI_ISL_963651 to 963652, EPI_ISL_963654 to 963655, EPI_ISL_963657 to 963659, EPI_ISL_963662 to 963667, EPI_ISL_963669 to 963677, EPI_ISL_963679 to 963690, EPI_ISL_963692 to 963696, EPI_ISL_963698 to 963710, EPI_ISL_963712 to 963738, EPI_ISL_963740 to 963742, EPI_ISL_963744 to 963745,
EPI_ISL_963747 to 963751, EPI_ISL_963753 to 963756, EPI_ISL_963758 to 963776, EPI_ISL_963778 to 963787, EPI_ISL_963789 to 963790, EPI_ISL_963792, EPI_ISL_963794 to 963796, EPI_ISL_963798 to 963801, EPI_ISL_963803 to 963808, EPI_ISL_963810 to 963842, EPI_ISL_963844 to 963846, EPI_ISL_963849 to
963855, EPI_ISL_963857 to 963861, EPI_ISL_963863 to 963885, EPI_ISL_963887 to 963891, EPI_ISL_963893, EPI_ISL_963895 to 963898, EPI_ISL_963900 to 963904, EPI_ISL_963906 to 963916, EPI_ISL_963918 to 963927, EPI_ISL_963929 to 963932, EPI_ISL_963934 to 963942, EPI_ISL_963945, EPI_ISL_963947,
EPI_ISL_963949 to 963950, EPI_ISL_963952 to 963966, EPI_ISL_963968 to 963971, EPI_ISL_963973 to 963985, EPI_ISL_964007 to 964008, EPI_ISL_964016, EPI_ISL_964067, EPI_ISL_964087, EPI_ISL_964107, EPI_ISL_964114, EPI_ISL_964155, EPI_ISL_964183, EPI_ISL_964196, EPI_ISL_964212 to 964213,
EPI_ISL_964219, EPI_ISL_964232, EPI_ISL_964234, EPI_ISL_964236, EPI_ISL_964246, EPI_ISL_964269, EPI_ISL_964277, EPI_ISL_964283, EPI_ISL_964302, EPI_ISL_964646, EPI_ISL_964710, EPI_ISL_964726, EPI_ISL_964749, EPI_ISL_964759, EPI_ISL_964817, EPI_ISL_964837, EPI_ISL_964877, EPI_ISL_969454,
EPI_ISL_969486, EPI_ISL_969498, EPI_ISL_969521, EPI_ISL_969528, EPI_ISL_969532, EPI_ISL_969542, EPI_ISL_969546, EPI_ISL_969558, EPI_ISL_969624, EPI_ISL_969688, EPI_ISL_969701, EPI_ISL_969795, EPI_ISL_969833, EPI_ISL_969840, EPI_ISL_969861, EPI_ISL_969979, EPI_ISL_969988, EPI_ISL_970000,
EPI_ISL_970022, EPI_ISL_970032, EPI_ISL_970055, EPI_ISL_970101, EPI_ISL_970142, EPI_ISL_970150, EPI_ISL_970188, EPI_ISL_970225, EPI_ISL_970251, EPI_ISL_970314, EPI_ISL_970338, EPI_ISL_970400, EPI_ISL_970478, EPI_ISL_970490, EPI_ISL_970506, EPI_ISL_970508, EPI_ISL_970546, EPI_ISL_985612 to
985945, EPI_ISL_986824 to 987004, EPI_ISL_987006 to 987103, EPI_ISL_987105 to 987160, EPI_ISL_987327, EPI_ISL_987333, EPI_ISL_987336, EPI_ISL_987410, EPI_ISL_987448, EPI_ISL_987469, EPI_ISL_987524, EPI_ISL_987534, EPI_ISL_987584, EPI_ISL_987620, EPI_ISL_988686 to 988756, EPI_ISL_988758 to
988797, EPI_ISL_988799 to 988810, EPI_ISL_988812 to 989018, EPI_ISL_989020, EPI_ISL_989022 to 989028, EPI_ISL_989482 to 989483, EPI_ISL_989485, EPI_ISL_989487 to 989491, EPI_ISL_989494 to 989499, EPI_ISL_989501 to 989502, EPI_ISL_989504 to 989507, EPI_ISL_989522 to 989523, EPI_ISL_989529,
EPI_ISL_989703, EPI_ISL_989711, EPI_ISL_989742, EPI_ISL_989751 to 989752, EPI_ISL_989755, EPI_ISL_989763, EPI_ISL_989765, EPI_ISL_989768 to 989769, EPI_ISL_989780 to 989785, EPI_ISL_989787 to 989790, EPI_ISL_989792 to 989794, EPI_ISL_989856 to 989881, EPI_ISL_989998 to 990057, EPI_ISL_990141 to
990197, EPI_ISL_990199 to 991172, EPI_ISL_991174 to 991177, EPI_ISL_991179 to 991350, EPI_ISL_991565 to 991903, EPI_ISL_991905 to 992042, EPI_ISL_992044, EPI_ISL_992046, EPI_ISL_992048, EPI_ISL_992050, EPI_ISL_992053 to 992055, EPI_ISL_992057 to 992059, EPI_ISL_992061 to 992064, EPI_ISL_992066 to
992067, EPI_ISL_992069, EPI_ISL_992071, EPI_ISL_992073 to 992074, EPI_ISL_992076 to 992083, EPI_ISL_992085, EPI_ISL_992087, EPI_ISL_992089 to 992092, EPI_ISL_992097, EPI_ISL_992099 to 992101, EPI_ISL_992103, EPI_ISL_992105, EPI_ISL_992108 to 992109, EPI_ISL_992111, EPI_ISL_992116 to 992119,
EPI_ISL_992122, EPI_ISL_992125, EPI_ISL_992127 to 992128, EPI_ISL_992130 to 992131, EPI_ISL_992135, EPI_ISL_992138, EPI_ISL_992143 to 992144, EPI_ISL_992146 to 992147, EPI_ISL_992149, EPI_ISL_992151 to 992153, EPI_ISL_992155 to 992156, EPI_ISL_992159, EPI_ISL_992161, EPI_ISL_992164,
EPI_ISL_992166, EPI_ISL_992172 to 992173, EPI_ISL_992175, EPI_ISL_992177 to 992181, EPI_ISL_992183 to 992184, EPI_ISL_992187 to 992189, EPI_ISL_992196 to 992198, EPI_ISL_992204 to 992207, EPI_ISL_992209, EPI_ISL_992211, EPI_ISL_992214 to 992215, EPI_ISL_992218 to 992221, EPI_ISL_992225 to 992226,
EPI_ISL_992229, EPI_ISL_992234, EPI_ISL_992236 to 992237, EPI_ISL_992240 to 992241, EPI_ISL_992243, EPI_ISL_992246, EPI_ISL_992250, EPI_ISL_992252, EPI_ISL_992258, EPI_ISL_992260 to 992262, EPI_ISL_992265, EPI_ISL_992267, EPI_ISL_992269, EPI_ISL_992271 to 992273, EPI_ISL_992275 to 992276,
EPI_ISL_992279, EPI_ISL_992282 to 992283, EPI_ISL_992286 to 992289, EPI_ISL_992291 to 992292, EPI_ISL_992294 to 992295, EPI_ISL_992301 to 992306, EPI_ISL_992309 to 992312, EPI_ISL_992314 to 992315, EPI_ISL_992317 to 992318, EPI_ISL_992321 to 992323, EPI_ISL_992329 to 992332, EPI_ISL_992335 to
992338, EPI_ISL_992344 to 992345, EPI_ISL_992348 to 992955, EPI_ISL_992957 to 993055, EPI_ISL_993057 to 993082, EPI_ISL_993084, EPI_ISL_993096, EPI_ISL_993098, EPI_ISL_993100, EPI_ISL_993105 to 993106, EPI_ISL_993109 to 993111, EPI_ISL_993113 to 993114, EPI_ISL_993119, EPI_ISL_993127,
EPI_ISL_993133, EPI_ISL_993140, EPI_ISL_993146, EPI_ISL_993149, EPI_ISL_993152 to 993155, EPI_ISL_993157, EPI_ISL_993161 to 993162, EPI_ISL_993165, EPI_ISL_993168, EPI_ISL_993171, EPI_ISL_993176, EPI_ISL_993184, EPI_ISL_993187, EPI_ISL_993194, EPI_ISL_993198 to 993199, EPI_ISL_993202,
EPI_ISL_993205 to 993206, EPI_ISL_993208, EPI_ISL_993211, EPI_ISL_993213, EPI_ISL_993221, EPI_ISL_993225, EPI_ISL_993227, EPI_ISL_993239, EPI_ISL_993244 to 993245, EPI_ISL_993248, EPI_ISL_993255, EPI_ISL_993257 to 993258, EPI_ISL_993266, EPI_ISL_993282, EPI_ISL_993291 to 993292,
EPI_ISL_993297, EPI_ISL_993306, EPI_ISL_993309, EPI_ISL_993311, EPI_ISL_993313, EPI_ISL_993316 to 993317, EPI_ISL_993321, EPI_ISL_993325, EPI_ISL_993327, EPI_ISL_993334, EPI_ISL_993338, EPI_ISL_993341, EPI_ISL_993348, EPI_ISL_993353, EPI_ISL_993362, EPI_ISL_993364, EPI_ISL_993369 to 993370,
EPI_ISL_993377, EPI_ISL_993383, EPI_ISL_993385 to 993386, EPI_ISL_993390 to 993391, EPI_ISL_993393, EPI_ISL_993397 to 993412, EPI_ISL_994288 to 994289, EPI_ISL_994291 to 994293, EPI_ISL_994297 to 994298, EPI_ISL_994303 to 994304, EPI_ISL_994306 to 994309, EPI_ISL_994313 to 994314, EPI_ISL_994318,
EPI_ISL_994320 to 994325, EPI_ISL_994328 to 994330, EPI_ISL_994335, EPI_ISL_994338 to 994340, EPI_ISL_994342 to 994343, EPI_ISL_994345 to 994346, EPI_ISL_994348, EPI_ISL_994351 to 994352, EPI_ISL_994354 to 994355, EPI_ISL_994357 to 994358, EPI_ISL_994360 to 994361, EPI_ISL_994364, EPI_ISL_994368
to 994370, EPI_ISL_994375 to 994376, EPI_ISL_994379, EPI_ISL_994385 to 994389, EPI_ISL_994391 to 994394, EPI_ISL_994396, EPI_ISL_994399 to 994400, EPI_ISL_994402, EPI_ISL_994404 to 994405, EPI_ISL_994407, EPI_ISL_994411 to 994419, EPI_ISL_994422 to 994424, EPI_ISL_994426, EPI_ISL_994428,
EPI_ISL_994430, EPI_ISL_994433, EPI_ISL_994438, EPI_ISL_994442, EPI_ISL_994445 to 994446, EPI_ISL_994448 to 994449, EPI_ISL_994451, EPI_ISL_994453 to 994456, EPI_ISL_994460 to 994468, EPI_ISL_994471, EPI_ISL_994473 to 994476, EPI_ISL_994478 to 994479, EPI_ISL_994481 to 994485, EPI_ISL_994488 to
994489, EPI_ISL_994491 to 994496, EPI_ISL_994498 to 994500, EPI_ISL_994505 to 994507, EPI_ISL_994512 to 994515, EPI_ISL_994519 to 994520, EPI_ISL_994522, EPI_ISL_994525 to 994527, EPI_ISL_994529, EPI_ISL_994531 to 994532, EPI_ISL_994534 to 994535, EPI_ISL_994538 to 994541, EPI_ISL_994543,
EPI_ISL_994545, EPI_ISL_994547 to 994609, EPI_ISL_994611 to 994651, EPI_ISL_1006540 to 1006543, EPI_ISL_1006937 to 1006940, EPI_ISL_1007436 to 1007439, EPI_ISL_1007549 to 1007560, EPI_ISL_1007563 to 1007574, EPI_ISL_1007580 to 1007582, EPI_ISL_1011757 to 1012098, EPI_ISL_1012550 to 1012607,
EPI_ISL_1012672 to 1012694, EPI_ISL_1012713 to 1012807, EPI_ISL_1012809 to 1012861, EPI_ISL_1012863 to 1012864, EPI_ISL_1012866 to 1012872, EPI_ISL_1018329 to 1018688, EPI_ISL_1018950 to 1019285, EPI_ISL_1019482 to 1019503, EPI_ISL_1033537 to 1033565, EPI_ISL_1034141 to 1034142, EPI_ISL_1042834
to 1043164, EPI_ISL_1043569 to 1044224, EPI_ISL_1044386 to 1044701, EPI_ISL_1044703 to 1044724, EPI_ISL_1044865 to 1045197, EPI_ISL_1045970 to 1045979, EPI_ISL_1057684 to 1057763, EPI_ISL_1057857 to 1057876, EPI_ISL_1057979 to 1058018, EPI_ISL_1061697 to 1062058, EPI_ISL_1063016 to 1063045,
EPI_ISL_1071638 to 1071642, EPI_ISL_1071644 to 1071647, EPI_ISL_1071651, EPI_ISL_1071653 to 1071658, EPI_ISL_1071660 to 1071661, EPI_ISL_1071663 to 1071670, EPI_ISL_1071672 to 1071675, EPI_ISL_1071677 to 1071679, EPI_ISL_1071683 to 1071691, EPI_ISL_1071694, EPI_ISL_1071696 to 1071705,
EPI_ISL_1071707 to 1071712, EPI_ISL_1071714, EPI_ISL_1071716 to 1071726, EPI_ISL_1071729 to 1071730, EPI_ISL_1071732 to 1071736, EPI_ISL_1071739 to 1071740, EPI_ISL_1071742 to 1071744, EPI_ISL_1071746 to 1071749, EPI_ISL_1071751 to 1071763, EPI_ISL_1071766 to 1071773, EPI_ISL_1071775 to 1071776,
EPI_ISL_1071779 to 1071780, EPI_ISL_1071782, EPI_ISL_1071785 to 1071792, EPI_ISL_1071795 to 1071796, EPI_ISL_1071798 to 1071799, EPI_ISL_1071801 to 1071802, EPI_ISL_1071806 to 1071811, EPI_ISL_1071814 to 1071815, EPI_ISL_1071817 to 1071818, EPI_ISL_1071820 to 1071822, EPI_ISL_1071824,
EPI_ISL_1071826 to 1071827, EPI_ISL_1071829 to 1071836, EPI_ISL_1071839 to 1071840, EPI_ISL_1071842 to 1071845, EPI_ISL_1071847 to 1071848, EPI_ISL_1071850 to 1071857, EPI_ISL_1071859 to 1071866, EPI_ISL_1071870 to 1071871, EPI_ISL_1071876 to 1071877, EPI_ISL_1071879 to 1071880, EPI_ISL_1071882,
EPI_ISL_1071884 to 1071886, EPI_ISL_1071888 to 1071899, EPI_ISL_1071901 to 1071903, EPI_ISL_1071905 to 1071911, EPI_ISL_1071916 to 1071923, EPI_ISL_1071925 to 1071927, EPI_ISL_1071929 to 1071930, EPI_ISL_1071932, EPI_ISL_1071934 to 1071936, EPI_ISL_1071938, EPI_ISL_1071940 to 1071943,
EPI_ISL_1071945 to 1071946, EPI_ISL_1071948 to 1071950, EPI_ISL_1071952 to 1071956, EPI_ISL_1071958 to 1071961, EPI_ISL_1071963 to 1071970, EPI_ISL_1071972 to 1071977, EPI_ISL_1071979 to 1071982, EPI_ISL_1071985 to 1071986, EPI_ISL_1071989 to 1071991, EPI_ISL_1071993 to 1071994, EPI_ISL_1071996
to 1072019, EPI_ISL_1072021 to 1072342, EPI_ISL_1072603 to 1072605, EPI_ISL_1072607 to 1072608, EPI_ISL_1072610 to 1072612, EPI_ISL_1072614 to 1072615, EPI_ISL_1072619, EPI_ISL_1072621 to 1072625, EPI_ISL_1072628 to 1072634, EPI_ISL_1072636 to 1072637, EPI_ISL_1072639 to 1072640, EPI_ISL_1072642
to 1072655, EPI_ISL_1072657 to 1072671, EPI_ISL_1072673 to 1072677, EPI_ISL_1072679 to 1072684, EPI_ISL_1072686 to 1072689, EPI_ISL_1072695, EPI_ISL_1072697 to 1072700, EPI_ISL_1072702 to 1072704, EPI_ISL_1072706, EPI_ISL_1072711 to 1072712, EPI_ISL_1072714, EPI_ISL_1072716, EPI_ISL_1072718 to
1072720, EPI_ISL_1072722, EPI_ISL_1072724 to 1072726, EPI_ISL_1072728 to 1072732, EPI_ISL_1072734 to 1072738, EPI_ISL_1072741, EPI_ISL_1072743, EPI_ISL_1072746 to 1072749, EPI_ISL_1072752 to 1072753, EPI_ISL_1072755 to 1072777, EPI_ISL_1072779 to 1072781, EPI_ISL_1072783, EPI_ISL_1072785 to
1072791, EPI_ISL_1072793 to 1072794, EPI_ISL_1072796 to 1072799, EPI_ISL_1072801, EPI_ISL_1072803, EPI_ISL_1072805 to 1072811, EPI_ISL_1072813, EPI_ISL_1072815 to 1072816, EPI_ISL_1072819, EPI_ISL_1072821 to 1072823, EPI_ISL_1072825 to 1072827, EPI_ISL_1072829 to 1072833, EPI_ISL_1072835,
EPI_ISL_1072837 to 1072840, EPI_ISL_1072844 to 1072848, EPI_ISL_1072851, EPI_ISL_1072853 to 1072866, EPI_ISL_1072868 to 1072870, EPI_ISL_1072872 to 1072875, EPI_ISL_1072878 to 1072880, EPI_ISL_1072883 to 1072887, EPI_ISL_1072889 to 1072893, EPI_ISL_1072895, EPI_ISL_1072897 to 1072901,
EPI_ISL_1072903 to 1072906, EPI_ISL_1072908, EPI_ISL_1072910 to 1072911, EPI_ISL_1072913 to 1072920, EPI_ISL_1072922 to 1072932, EPI_ISL_1072936 to 1072942, EPI_ISL_1072945 to 1072946, EPI_ISL_1072948 to 1072949, EPI_ISL_1072953 to 1072958, EPI_ISL_1083130 to 1083589, EPI_ISL_1083591 to 1083816,
EPI_ISL_1084469, EPI_ISL_1084677 to 1084718, EPI_ISL_1098905 to 1098907, EPI_ISL_1098909, EPI_ISL_1098911 to 1098912, EPI_ISL_1098916 to 1098917, EPI_ISL_1098920, EPI_ISL_1098922, EPI_ISL_1098924, EPI_ISL_1098926 to 1098927, EPI_ISL_1098929 to 1098930, EPI_ISL_1098934, EPI_ISL_1098936 to
1098939, EPI_ISL_1098941, EPI_ISL_1098943, EPI_ISL_1098946 to 1098947, EPI_ISL_1098951 to 1098953, EPI_ISL_1098955, EPI_ISL_1098961 to 1098962, EPI_ISL_1098966 to 1098975, EPI_ISL_1098977 to 1098979, EPI_ISL_1098984, EPI_ISL_1098986, EPI_ISL_1098989 to 1098990, EPI_ISL_1098993,
EPI_ISL_1098996, EPI_ISL_1098998 to 1099000, EPI_ISL_1099003 to 1099004, EPI_ISL_1099007, EPI_ISL_1099012 to 1099013, EPI_ISL_1099015 to 1099018, EPI_ISL_1099020, EPI_ISL_1099023 to 1099027, EPI_ISL_1099030 to 1099031, EPI_ISL_1099033 to 1099034, EPI_ISL_1099039 to 1099040, EPI_ISL_1099042 to
1099043, EPI_ISL_1099045 to 1099046, EPI_ISL_1099051, EPI_ISL_1099053 to 1099056, EPI_ISL_1099059 to 1099063, EPI_ISL_1099065 to 1099066, EPI_ISL_1099068, EPI_ISL_1099070, EPI_ISL_1099072 to 1099073, EPI_ISL_1099076, EPI_ISL_1099078 to 1099083, EPI_ISL_1099087, EPI_ISL_1099089,
EPI_ISL_1099096 to 1099100, EPI_ISL_1099102, EPI_ISL_1099104 to 1099106, EPI_ISL_1099108, EPI_ISL_1099111 to 1099113, EPI_ISL_1099116 to 1099117, EPI_ISL_1099121, EPI_ISL_1099123 to 1099131, EPI_ISL_1099133, EPI_ISL_1099136 to 1099138, EPI_ISL_1099143, EPI_ISL_1099145 to 1099148,
EPI_ISL_1099150, EPI_ISL_1099153, EPI_ISL_1099157, EPI_ISL_1099159, EPI_ISL_1099162 to 1099163, EPI_ISL_1099165, EPI_ISL_1099167, EPI_ISL_1099170 to 1099172, EPI_ISL_1099174, EPI_ISL_1099180 to 1099182, EPI_ISL_1099184 to 1099185, EPI_ISL_1099187 to 1099188, EPI_ISL_1099190, EPI_ISL_1099193,
EPI_ISL_1099195 to 1099196, EPI_ISL_1099198 to 1099199, EPI_ISL_1099203 to 1099205, EPI_ISL_1099207 to 1099209, EPI_ISL_1099211, EPI_ISL_1099213, EPI_ISL_1099215 to 1099216, EPI_ISL_1099223, EPI_ISL_1099225, EPI_ISL_1099227, EPI_ISL_1099231, EPI_ISL_1100074 to 1100077, EPI_ISL_1100079 to
1100088, EPI_ISL_1100090 to 1100091, EPI_ISL_1100093 to 1100094, EPI_ISL_1100096 to 1100097, EPI_ISL_1100099, EPI_ISL_1100101 to 1100130, EPI_ISL_1100133 to 1100147, EPI_ISL_1100150 to 1100152, EPI_ISL_1100154 to 1100159, EPI_ISL_1100161 to 1100164, EPI_ISL_1100166 to 1100176, EPI_ISL_1100178 to
1100179, EPI_ISL_1100181 to 1100203, EPI_ISL_1100205 to 1100208, EPI_ISL_1100210 to 1100224, EPI_ISL_1100226 to 1100231, EPI_ISL_1100234 to 1100262, EPI_ISL_1100264 to 1100280, EPI_ISL_1100282 to 1100296, EPI_ISL_1100298 to 1100300, EPI_ISL_1100302 to 1100307, EPI_ISL_1100310 to 1100319,
EPI_ISL_1100321 to 1100322, EPI_ISL_1100324 to 1100330, EPI_ISL_1100332 to 1100339, EPI_ISL_1100341 to 1100354, EPI_ISL_1100358 to 1100360, EPI_ISL_1100362 to 1100384, EPI_ISL_1100386 to 1100394, EPI_ISL_1100396 to 1100407, EPI_ISL_1100409 to 1100414, EPI_ISL_1100417, EPI_ISL_1100419 to 1100420,
EPI_ISL_1100422 to 1100425, EPI_ISL_1100427 to 1100431, EPI_ISL_1100433, EPI_ISL_1100435 to 1100439, EPI_ISL_1100441 to 1100451, EPI_ISL_1100453 to 1100456, EPI_ISL_1100458 to 1100460, EPI_ISL_1100462 to 1100467, EPI_ISL_1100469 to 1100471, EPI_ISL_1100473, EPI_ISL_1100475 to 1100476,
EPI_ISL_1100480 to 1100481, EPI_ISL_1100486, EPI_ISL_1100488 to 1100508, EPI_ISL_1100510 to 1100511, EPI_ISL_1100513 to 1100521, EPI_ISL_1100523 to 1100526, EPI_ISL_1100528 to 1100530, EPI_ISL_1100532 to 1100535, EPI_ISL_1100537 to 1100540, EPI_ISL_1100542 to 1100546, EPI_ISL_1100549,
EPI_ISL_1100554 to 1100555, EPI_ISL_1100558 to 1100563, EPI_ISL_1100565 to 1100567, EPI_ISL_1100571 to 1100573, EPI_ISL_1100577 to 1100582, EPI_ISL_1100584 to 1100589, EPI_ISL_1100591 to 1100598, EPI_ISL_1100602 to 1100607, EPI_ISL_1100609 to 1100616, EPI_ISL_1100618 to 1100625, EPI_ISL_1100627,
EPI_ISL_1100629, EPI_ISL_1100632, EPI_ISL_1100634, EPI_ISL_1100637 to 1100647, EPI_ISL_1100649 to 1100654, EPI_ISL_1100656 to 1100659, EPI_ISL_1100662, EPI_ISL_1100665, EPI_ISL_1100668 to 1100672, EPI_ISL_1100674 to 1100675, EPI_ISL_1100678 to 1100680, EPI_ISL_1100682 to 1100683,
EPI_ISL_1100686, EPI_ISL_1100688, EPI_ISL_1100690 to 1100693, EPI_ISL_1100696 to 1100698, EPI_ISL_1100701 to 1100704, EPI_ISL_1100706 to 1100709, EPI_ISL_1100711 to 1100712, EPI_ISL_1100715 to 1100719, EPI_ISL_1100721 to 1100724, EPI_ISL_1100726 to 1100727, EPI_ISL_1100730 to 1100736,
EPI_ISL_1100739 to 1100740, EPI_ISL_1100742 to 1100746, EPI_ISL_1100748 to 1100754, EPI_ISL_1100991 to 1100992, EPI_ISL_1100999, EPI_ISL_1101001, EPI_ISL_1101007 to 1101010, EPI_ISL_1101018 to 1101020, EPI_ISL_1101027, EPI_ISL_1101029, EPI_ISL_1101031, EPI_ISL_1101033 to 1101034,
EPI_ISL_1101037 to 1101039, EPI_ISL_1101057, EPI_ISL_1101059 to 1101060, EPI_ISL_1101062, EPI_ISL_1101068, EPI_ISL_1101071, EPI_ISL_1101073 to 1101074, EPI_ISL_1101085, EPI_ISL_1101088, EPI_ISL_1101093, EPI_ISL_1101100, EPI_ISL_1101104 to 1101106, EPI_ISL_1101108, EPI_ISL_1101110 to 1101111,
EPI_ISL_1101114, EPI_ISL_1101116, EPI_ISL_1101118, EPI_ISL_1101130, EPI_ISL_1101141, EPI_ISL_1101147 to 1101148, EPI_ISL_1101152, EPI_ISL_1101171, EPI_ISL_1101176, EPI_ISL_1101178, EPI_ISL_1101180, EPI_ISL_1101187 to 1101188, EPI_ISL_1101192, EPI_ISL_1101195 to 1101196, EPI_ISL_1101201,
EPI_ISL_1101210, EPI_ISL_1101214, EPI_ISL_1101226 to 1101230, EPI_ISL_1101233, EPI_ISL_1101237, EPI_ISL_1101241 to 1101242, EPI_ISL_1101245, EPI_ISL_1101247, EPI_ISL_1101257, EPI_ISL_1101268 to 1101269, EPI_ISL_1101271 to 1101272, EPI_ISL_1101277, EPI_ISL_1101279 to 1101280, EPI_ISL_1101292
to 1101293, EPI_ISL_1101299 to 1101300, EPI_ISL_1101308 to 1101309, EPI_ISL_1101316 to 1101318, EPI_ISL_1101320, EPI_ISL_1101322 to 1101662, EPI_ISL_1103053, EPI_ISL_1114772, EPI_ISL_1114776, EPI_ISL_1114780, EPI_ISL_1114782, EPI_ISL_1114784, EPI_ISL_1114792, EPI_ISL_1114794 to 1114797,
EPI_ISL_1114799, EPI_ISL_1114810, EPI_ISL_1114814, EPI_ISL_1114819 to 1114820, EPI_ISL_1114831, EPI_ISL_1114835, EPI_ISL_1114840, EPI_ISL_1114844, EPI_ISL_1114846, EPI_ISL_1114852, EPI_ISL_1114855 to 1114856, EPI_ISL_1114858, EPI_ISL_1114860, EPI_ISL_1114862, EPI_ISL_1114866,
EPI_ISL_1114871 to 1114872, EPI_ISL_1114876, EPI_ISL_1114884, EPI_ISL_1114892, EPI_ISL_1114894, EPI_ISL_1114902 to 1114903, EPI_ISL_1114910 to 1114912, EPI_ISL_1114925, EPI_ISL_1114929, EPI_ISL_1114931 to 1114933, EPI_ISL_1114935 to 1114936, EPI_ISL_1114944, EPI_ISL_1114951, EPI_ISL_1114959,
EPI_ISL_1114962, EPI_ISL_1114968, EPI_ISL_1114973 to 1114974, EPI_ISL_1114976, EPI_ISL_1114978, EPI_ISL_1114980, EPI_ISL_1114982, EPI_ISL_1114987 to 1114988, EPI_ISL_1114990 to 1114991, EPI_ISL_1114994, EPI_ISL_1114997 to 1114999, EPI_ISL_1115005, EPI_ISL_1115017, EPI_ISL_1115020,
EPI_ISL_1115022, EPI_ISL_1115024, EPI_ISL_1115027 to 1115029, EPI_ISL_1115031, EPI_ISL_1115033, EPI_ISL_1115038, EPI_ISL_1115042, EPI_ISL_1115046, EPI_ISL_1115048 to 1115049, EPI_ISL_1115055, EPI_ISL_1115057, EPI_ISL_1115068, EPI_ISL_1115076, EPI_ISL_1115081, EPI_ISL_1115083,
EPI_ISL_1115087, EPI_ISL_1115089, EPI_ISL_1115091, EPI_ISL_1115095, EPI_ISL_1115098, EPI_ISL_1115685, EPI_ISL_1115688 to 1115693, EPI_ISL_1115695, EPI_ISL_1115697 to 1115702, EPI_ISL_1115704 to 1115708, EPI_ISL_1115710 to 1115716, EPI_ISL_1115798, EPI_ISL_1115800, EPI_ISL_1115802 to 1115824,
EPI_ISL_1116264 to 1116309, EPI_ISL_1122459 to 1122464, EPI_ISL_1122466 to 1122471, EPI_ISL_1122475 to 1122478, EPI_ISL_1122480 to 1122484, EPI_ISL_1122486, EPI_ISL_1122488, EPI_ISL_1122491 to 1122495, EPI_ISL_1122498 to 1122502, EPI_ISL_1122504 to 1122505, EPI_ISL_1122507 to 1122514,
EPI_ISL_1122517, EPI_ISL_1122519, EPI_ISL_1122522 to 1122532, EPI_ISL_1122534 to 1122539, EPI_ISL_1122543 to 1122547, EPI_ISL_1122549, EPI_ISL_1122551 to 1122552, EPI_ISL_1122555 to 1122579, EPI_ISL_1122581 to 1122582, EPI_ISL_1122584 to 1122605, EPI_ISL_1122607 to 1122610, EPI_ISL_1122612 to
1122617, EPI_ISL_1122619, EPI_ISL_1122622 to 1122624, EPI_ISL_1122628 to 1122631, EPI_ISL_1122633 to 1122637, EPI_ISL_1122639, EPI_ISL_1122641 to 1122643, EPI_ISL_1122645 to 1122652, EPI_ISL_1122657 to 1122664, EPI_ISL_1122666 to 1122667, EPI_ISL_1122671, EPI_ISL_1122673 to 1122676,
EPI_ISL_1122678, EPI_ISL_1122680, EPI_ISL_1122682, EPI_ISL_1122684, EPI_ISL_1122686 to 1122691, EPI_ISL_1122693 to 1122696, EPI_ISL_1122698 to 1122699, EPI_ISL_1122701 to 1122704, EPI_ISL_1122706 to 1122720, EPI_ISL_1122723 to 1122725, EPI_ISL_1122727 to 1122730, EPI_ISL_1122732 to 1122738,
EPI_ISL_1122740, EPI_ISL_1122743 to 1122744, EPI_ISL_1122746 to 1122760, EPI_ISL_1122762, EPI_ISL_1122765, EPI_ISL_1122767 to 1122770, EPI_ISL_1122772 to 1122774, EPI_ISL_1122776 to 1122783, EPI_ISL_1122785 to 1122790, EPI_ISL_1122793 to 1122799, EPI_ISL_1122802 to 1122804, EPI_ISL_1122806,
EPI_ISL_1174548 to 1174549, EPI_ISL_1174552 to 1174553, EPI_ISL_1174555, EPI_ISL_1174561, EPI_ISL_1174563 to 1174565, EPI_ISL_1174568, EPI_ISL_1174572 to 1174577, EPI_ISL_1174584 to 1174585, EPI_ISL_1174589 to 1174595, EPI_ISL_1174598 to 1174599, EPI_ISL_1174602, EPI_ISL_1174607,
EPI_ISL_1174611, EPI_ISL_1174614 to 1174615, EPI_ISL_1174617, EPI_ISL_1174620, EPI_ISL_1174625 to 1174626, EPI_ISL_1174628, EPI_ISL_1174631, EPI_ISL_1174634, EPI_ISL_1174637, EPI_ISL_1174639, EPI_ISL_1174641 to 1174642, EPI_ISL_1174644 to 1174646, EPI_ISL_1174648 to 1174651, EPI_ISL_1174654,
EPI_ISL_1174656 to 1174657, EPI_ISL_1174659 to 1174660, EPI_ISL_1174663, EPI_ISL_1174665 to 1174668, EPI_ISL_1174671 to 1174673, EPI_ISL_1174676 to 1174678, EPI_ISL_1174682 to 1174683, EPI_ISL_1174685 to 1174688, EPI_ISL_1174690 to 1174691, EPI_ISL_1174693 to 1174694, EPI_ISL_1174698 to 1174699,
EPI_ISL_1174701 to 1174702, EPI_ISL_1174712, EPI_ISL_1174716, EPI_ISL_1174718 to 1174719, EPI_ISL_1174728, EPI_ISL_1174730 to 1174731, EPI_ISL_1174735 to 1174738, EPI_ISL_1174740, EPI_ISL_1174742 to 1174744, EPI_ISL_1174748 to 1174751, EPI_ISL_1174760 to 1174764, EPI_ISL_1174766,
EPI_ISL_1174768, EPI_ISL_1174771 to 1174772, EPI_ISL_1174777, EPI_ISL_1174779, EPI_ISL_1174783, EPI_ISL_1174785, EPI_ISL_1174789, EPI_ISL_1174791, EPI_ISL_1174793 to 1174795, EPI_ISL_1174798 to 1174801, EPI_ISL_1174803 to 1174804, EPI_ISL_1174807 to 1174808, EPI_ISL_1174810 to 1174812,

EPI_ISL_1174814, EPI_ISL_1174816 to 1174820, EPI_ISL_1174825 to 1174830, EPI_ISL_1174833 to 1174837, EPI_ISL_1174842 to 1174845, EPI_ISL_1174848 to 1174852, EPI_ISL_1174854, EPI_ISL_1174857, EPI_ISL_1174860 to 1174861, EPI_ISL_1174863, EPI_ISL_1174865 to 1174866, EPI_ISL_1174869, EPI_ISL_1174878 to 1174879, EPI_ISL_1175418, EPI_ISL_1175420 to 1175424, EPI_ISL_1175426 to 1175428, EPI_ISL_1175430 to 1175433, EPI_ISL_1175436, EPI_ISL_1175444 to 1175449, EPI_ISL_1175451 to 1175453, EPI_ISL_1175460 to 1175464, EPI_ISL_1175466, EPI_ISL_1175469, EPI_ISL_1175472 to 1175475, EPI_ISL_1175479, EPI_ISL_1175481, EPI_ISL_1175483, EPI_ISL_1175485 to 1175486, EPI_ISL_1175489, EPI_ISL_1175492 to 1175493, EPI_ISL_1175500, EPI_ISL_1175506 to 1175508, EPI_ISL_1175511, EPI_ISL_1175513 to 1175514, EPI_ISL_1175521 to 1175527, EPI_ISL_1175531 to 1175532, EPI_ISL_1175534 to 1175535, EPI_ISL_1175538, EPI_ISL_1175543, EPI_ISL_1175545 to 1175546, EPI_ISL_1175548, EPI_ISL_1175553, EPI_ISL_1175562, EPI_ISL_1175565, EPI_ISL_1175567, EPI_ISL_1175569 to 1175570, EPI_ISL_1175573, EPI_ISL_1175575 to 1175579, EPI_ISL_1175581 to 1175582, EPI_ISL_1175584, EPI_ISL_1175588, EPI_ISL_1175591 to 1175592, EPI_ISL_1175596 to 1175597, EPI_ISL_1175599, EPI_ISL_1175603 to 1175604, EPI_ISL_1175607 to 1175608, EPI_ISL_1175615 to 1175621, EPI_ISL_1175623, EPI_ISL_1175625 to 1175626, EPI_ISL_1175628, EPI_ISL_1175630, EPI_ISL_1175632 to 1175633, EPI_ISL_1175635 to 1175637, EPI_ISL_1175639, EPI_ISL_1175642 to 1175646, EPI_ISL_1175649 to 1175650, EPI_ISL_1175653 to 1175655, EPI_ISL_1175658, EPI_ISL_1175660 to 1175661, EPI_ISL_1175664, EPI_ISL_1175666, EPI_ISL_1175668 to 1175669, EPI_ISL_1175671 to 1175672, EPI_ISL_1175674 to 1175678, EPI_ISL_1175681, EPI_ISL_1175683, EPI_ISL_1175687 to 1175688, EPI_ISL_1175690, EPI_ISL_1175695 to 1175696, EPI_ISL_1175699, EPI_ISL_1175702 to 1175707, EPI_ISL_1175713 to 1175714, EPI_ISL_1175716, EPI_ISL_1175723, EPI_ISL_1175726, EPI_ISL_1175729, EPI_ISL_1175733, EPI_ISL_1175735 to 1175737, EPI_ISL_1175739, EPI_ISL_1175741 to 1175742, EPI_ISL_1175744, EPI_ISL_1175746 to 1175748, EPI_ISL_1175750 to 1175751, EPI_ISL_1175753, EPI_ISL_1175756 to 1175759, EPI_ISL_1186252 to 1186264, EPI_ISL_1186266 to 1186272, EPI_ISL_1186274, EPI_ISL_1186276 to 1186283, EPI_ISL_1186285 to 1186295, EPI_ISL_1186297, EPI_ISL_1186300 to 1186303, EPI_ISL_1186306, EPI_ISL_1186309 to 1186315, EPI_ISL_1186319 to 1186325, EPI_ISL_1186328, EPI_ISL_1186330, EPI_ISL_1186332 to 1186334, EPI_ISL_1186336, EPI_ISL_1186340 to 1186343, EPI_ISL_1186345, EPI_ISL_1186347, EPI_ISL_1186355 to 1186356, EPI_ISL_1186358 to 1186359, EPI_ISL_1186361 to 1186362, EPI_ISL_1186364 to 1186367, EPI_ISL_1186369 to 1186374, EPI_ISL_1186376, EPI_ISL_1186378 to 1186380, EPI_ISL_1186382 to 1186387, EPI_ISL_1186389, EPI_ISL_1186397 to 1186398, EPI_ISL_1186401 to 1186406, EPI_ISL_1186408, EPI_ISL_1186412 to 1186414, EPI_ISL_1186416 to 1186417, EPI_ISL_1186419 to 1186424, EPI_ISL_1186426 to 1186430, EPI_ISL_1186432 to 1186435, EPI_ISL_1186437, EPI_ISL_1186439 to 1186442, EPI_ISL_1186444 to 1186452, EPI_ISL_1186454 to 1186455, EPI_ISL_1186457 to 1186460, EPI_ISL_1186462 to 1186463, EPI_ISL_1186466, EPI_ISL_1186469 to 1186470, EPI_ISL_1186472 to 1186473, EPI_ISL_1186477 to 1186479, EPI_ISL_1186481 to 1186488, EPI_ISL_1186491 to 1186504, EPI_ISL_1186506 to 1186507, EPI_ISL_1186510 to 1186511, EPI_ISL_1186513 to 1186515, EPI_ISL_1186517 to 1186519, EPI_ISL_1186521, EPI_ISL_1186523, EPI_ISL_1186526 to 1186528, EPI_ISL_1186530 to 1186532, EPI_ISL_1186534, EPI_ISL_1186536, EPI_ISL_1186543, EPI_ISL_1186545 to 1186547, EPI_ISL_1186549 to 1186551, EPI_ISL_1186553 to 1186554, EPI_ISL_1186556, EPI_ISL_1186558 to 1186559, EPI_ISL_1186562, EPI_ISL_1186564 to 1186565, EPI_ISL_1186568 to 1186573, EPI_ISL_1186575, EPI_ISL_1186578, EPI_ISL_1186582, EPI_ISL_1186584 to 1186589, EPI_ISL_1186591 to 1186592, EPI_ISL_1186594 to 1186600, EPI_ISL_1186608, EPI_ISL_1186613, EPI_ISL_1186615 to 1186617, EPI_ISL_1186619, EPI_ISL_1186621, EPI_ISL_1186624 to 1186625, EPI_ISL_1186630 to 1186632, EPI_ISL_1186637 to 1186642, EPI_ISL_1186644 to 1186646, EPI_ISL_1186648 to 1186651, EPI_ISL_1186655, EPI_ISL_1186657 to 1186660, EPI_ISL_1186662 to 1186665, EPI_ISL_1186667, EPI_ISL_1186670 to 1186671, EPI_ISL_1186676, EPI_ISL_1186678, EPI_ISL_1186680, EPI_ISL_1186683, EPI_ISL_1186688 to 1186693, EPI_ISL_1186696, EPI_ISL_1186698, EPI_ISL_1186700 to 1186702, EPI_ISL_1186705 to 1186708, EPI_ISL_1186710, EPI_ISL_1186712, EPI_ISL_1186715 to 1186718, EPI_ISL_1186720 to 1186721, EPI_ISL_1186723 to 1186725, EPI_ISL_1186727 to 1186728, EPI_ISL_1186731, EPI_ISL_1186734 to 1186736, EPI_ISL_1186739, EPI_ISL_1186742, EPI_ISL_1186744, EPI_ISL_1186746 to 1186748, EPI_ISL_1186751, EPI_ISL_1186757 to 1186758, EPI_ISL_1186760 to 1186762, EPI_ISL_1186765, EPI_ISL_1186767, EPI_ISL_1186770 to 1186771, EPI_ISL_1186774, EPI_ISL_1186777, EPI_ISL_1186782 to 1186784, EPI_ISL_1186786, EPI_ISL_1186788 to 1186789, EPI_ISL_1186791 to 1186792, EPI_ISL_1186795 to 1186798, EPI_ISL_1186800, EPI_ISL_1186802 to 1186805, EPI_ISL_1186811, EPI_ISL_1186814, EPI_ISL_1186816, EPI_ISL_1186819 to 1186822, EPI_ISL_1186826, EPI_ISL_1186828, EPI_ISL_1186830 to 1186833, EPI_ISL_1186836 to 1186838, EPI_ISL_1186841 to 1186842, EPI_ISL_1186844, EPI_ISL_1186846 to 1186852, EPI_ISL_1186854 to 1186855, EPI_ISL_1187255, EPI_ISL_1187257, EPI_ISL_1187267 to 1187270, EPI_ISL_1187274, EPI_ISL_1187287 to 1187288, EPI_ISL_1187291, EPI_ISL_1187294 to 1187295, EPI_ISL_1187298, EPI_ISL_1187304, EPI_ISL_1187307, EPI_ISL_1187311, EPI_ISL_1187313, EPI_ISL_1187323 to 1187324, EPI_ISL_1187326, EPI_ISL_1187331 to 1187332, EPI_ISL_1187342, EPI_ISL_1187344 to 1187345, EPI_ISL_1187349, EPI_ISL_1187351, EPI_ISL_1187354 to 1187356, EPI_ISL_1187358, EPI_ISL_1187361, EPI_ISL_1187366, EPI_ISL_1187370, EPI_ISL_1187377, EPI_ISL_1187379 to 1187380, EPI_ISL_1187388, EPI_ISL_1187391, EPI_ISL_1187393 to 1187394, EPI_ISL_1187396, EPI_ISL_1187398, EPI_ISL_1187400 to 1187401, EPI_ISL_1187404, EPI_ISL_1187409, EPI_ISL_1187416 to 1187417, EPI_ISL_1187423 to 1187424, EPI_ISL_1187431 to 1187432, EPI_ISL_1187443, EPI_ISL_1187446 to 1187447, EPI_ISL_1187451, EPI_ISL_1187453, EPI_ISL_1187456, EPI_ISL_1187460 to 1187463, EPI_ISL_1187469, EPI_ISL_1187480, EPI_ISL_1187483 to 1187484, EPI_ISL_1187489, EPI_ISL_1187492, EPI_ISL_1187494, EPI_ISL_1187496, EPI_ISL_1187498, EPI_ISL_1187500, EPI_ISL_1187505, EPI_ISL_1187507, EPI_ISL_1187510, EPI_ISL_1187515 to 1187517, EPI_ISL_1187524, EPI_ISL_1187528, EPI_ISL_1187530, EPI_ISL_1187532 to 1187533, EPI_ISL_1187535, EPI_ISL_1187754 to 1187755, EPI_ISL_1187757 to 1187762, EPI_ISL_1187764, EPI_ISL_1187766 to 1187767, EPI_ISL_1187770 to 1187772, EPI_ISL_1187775 to 1187776, EPI_ISL_1187778 to 1187780, EPI_ISL_1187782 to 1187784, EPI_ISL_1187787, EPI_ISL_1187789 to 1187793, EPI_ISL_1187796 to 1187798, EPI_ISL_1187801, EPI_ISL_1187805, EPI_ISL_1187807, EPI_ISL_1187809, EPI_ISL_1187812, EPI_ISL_1187817 to 1187819, EPI_ISL_1187824 to 1187825, EPI_ISL_1187828 to 1187830, EPI_ISL_1187832 to 1187834, EPI_ISL_1187836, EPI_ISL_1187846 to 1187847, EPI_ISL_1187851 to 1187852, EPI_ISL_1187854 to 1187855, EPI_ISL_1187857, EPI_ISL_1187859, EPI_ISL_1187866 to 1187867, EPI_ISL_1187869 to 1187872, EPI_ISL_1187874 to 1187875, EPI_ISL_1187878, EPI_ISL_1187881 to 1187882, EPI_ISL_1187885 to 1187889, EPI_ISL_1187891, EPI_ISL_1187893, EPI_ISL_1187895, EPI_ISL_1187899, EPI_ISL_1187901, EPI_ISL_1187904 to 1187905, EPI_ISL_1187908 to 1187909, EPI_ISL_1187912, EPI_ISL_1187915 to 1187918, EPI_ISL_1187930 to 1187931, EPI_ISL_1187934, EPI_ISL_1187936 to 1187939, EPI_ISL_1187944, EPI_ISL_1187947 to 1187950, EPI_ISL_1187953 to 1187954, EPI_ISL_1187956, EPI_ISL_1187958 to 1187961, EPI_ISL_1187963 to 1187964, EPI_ISL_1187966 to 1187971, EPI_ISL_1187973 to 1187974, EPI_ISL_1187980, EPI_ISL_1187982, EPI_ISL_1187986 to 1187987, EPI_ISL_1187989, EPI_ISL_1187992, EPI_ISL_1187996, EPI_ISL_1187998 to 1187999, EPI_ISL_1188002 to 1188005, EPI_ISL_1188007, EPI_ISL_1188009 to 1188011, EPI_ISL_1188018, EPI_ISL_1188646, EPI_ISL_1188649, EPI_ISL_1188651, EPI_ISL_1188653 to 1188656, EPI_ISL_1188659 to 1188660, EPI_ISL_1188662, EPI_ISL_1188664, EPI_ISL_1188666 to 1188668, EPI_ISL_1188670, EPI_ISL_1188672 to 1188674, EPI_ISL_1188676 to 1188677, EPI_ISL_1188683 to 1188686, EPI_ISL_1188688 to 1188689, EPI_ISL_1188691, EPI_ISL_1188693 to 1188694, EPI_ISL_1188697 to 1188699, EPI_ISL_1188701 to 1188704, EPI_ISL_1188706, EPI_ISL_1188708, EPI_ISL_1188710, EPI_ISL_1188713, EPI_ISL_1188715 to 1188717, EPI_ISL_1188719 to 1188720, EPI_ISL_1188723, EPI_ISL_1188725 to 1188726, EPI_ISL_1188728 to 1188729, EPI_ISL_1188731 to 1188734, EPI_ISL_1188738 to 1188745, EPI_ISL_1188748, EPI_ISL_1188752 to 1188754, EPI_ISL_1188756, EPI_ISL_1188758, EPI_ISL_1188761 to 1188766, EPI_ISL_1188769 to 1188770, EPI_ISL_1188772 to 1188774, EPI_ISL_1188777 to 1188778, EPI_ISL_1188780, EPI_ISL_1188783, EPI_ISL_1188786, EPI_ISL_1188788, EPI_ISL_1188790 to 1188794, EPI_ISL_1188796 to 1188798, EPI_ISL_1188802, EPI_ISL_1188804, EPI_ISL_1188806, EPI_ISL_1188809 to 1188811, EPI_ISL_1188814 to 1188815, EPI_ISL_1188817, EPI_ISL_1188820, EPI_ISL_1188823, EPI_ISL_1188825 to 1188826, EPI_ISL_1188828, EPI_ISL_1188833 to 1188834, EPI_ISL_1188842, EPI_ISL_1188844 to 1188845, EPI_ISL_1188848, EPI_ISL_1188852 to 1188859, EPI_ISL_1188862 to 1188870, EPI_ISL_1188877 to 1188885, EPI_ISL_1188888, EPI_ISL_1188890, EPI_ISL_1188894 to 1188903, EPI_ISL_1188907 to 1188908, EPI_ISL_1188912 to 1188915, EPI_ISL_1188917, EPI_ISL_1188919 to 1188920, EPI_ISL_1188922 to 1188924, EPI_ISL_1188928 to 1188930, EPI_ISL_1188933, EPI_ISL_1188935 to 1188936, EPI_ISL_1188938 to 1188939, EPI_ISL_1188941 to 1188943, EPI_ISL_1188947, EPI_ISL_1188951 to 1188952, EPI_ISL_1188955 to 1188957, EPI_ISL_1188959 to 1188962, EPI_ISL_1188964 to 1188966, EPI_ISL_1188968, EPI_ISL_1188972 to 1188975, EPI_ISL_1188977 to 1188982, EPI_ISL_1188984 to 1188987, EPI_ISL_1188990 to 1188996, EPI_ISL_1188999 to 1189001, EPI_ISL_1189003, EPI_ISL_1189005 to 1189012, EPI_ISL_1189017, EPI_ISL_1189019 to 1189023, EPI_ISL_1189025 to 1189026, EPI_ISL_1189028 to 1189031, EPI_ISL_1189033 to 1189037, EPI_ISL_1189039 to 1189040, EPI_ISL_1189042 to 1189049, EPI_ISL_1189051 to 1189061, EPI_ISL_1189063 to 1189064, EPI_ISL_1189068 to 1189071, EPI_ISL_1189076 to 1189080, EPI_ISL_1189082, EPI_ISL_1189084, EPI_ISL_1189088 to 1189090, EPI_ISL_1189092 to 1189094, EPI_ISL_1189096, EPI_ISL_1189098, EPI_ISL_1189101, EPI_ISL_1189103 to 1189108, EPI_ISL_1189111 to 1189113, EPI_ISL_1189115, EPI_ISL_1189117 to 1189126, EPI_ISL_1189128 to 1189130, EPI_ISL_1189132 to 1189134, EPI_ISL_1189136, EPI_ISL_1189138 to 1189139, EPI_ISL_1189142 to 1189144, EPI_ISL_1189152 to 1189156, EPI_ISL_1189158 to 1189162, EPI_ISL_1189165 to 1189169, EPI_ISL_1189171 to 1189177, EPI_ISL_1189179, EPI_ISL_1189181 to 1189192, EPI_ISL_1189194, EPI_ISL_1189196, EPI_ISL_1189198 to 1189204, EPI_ISL_1189206, EPI_ISL_1189208 to 1189216, EPI_ISL_1189219, EPI_ISL_1189221 to 1189228, EPI_ISL_1189231, EPI_ISL_1189233, EPI_ISL_1189235, EPI_ISL_1189237 to 1189238, EPI_ISL_1189242 to 1189249, EPI_ISL_1189252, EPI_ISL_1189254 to 1189257, EPI_ISL_1189259 to 1189264, EPI_ISL_1189267 to 1189272, EPI_ISL_1189275, EPI_ISL_1189277, EPI_ISL_1189281 to 1189285, EPI_ISL_1189287 to 1189294, EPI_ISL_1189296, EPI_ISL_1189298 to 1189305, EPI_ISL_1189307 to 1189312, EPI_ISL_1189314, EPI_ISL_1189316, EPI_ISL_1189318, EPI_ISL_1189320 to 1189328, EPI_ISL_1189403, EPI_ISL_1189507, EPI_ISL_1189509, EPI_ISL_1189513 to 1189514, EPI_ISL_1189516 to 1189518, EPI_ISL_1189523, EPI_ISL_1189527, EPI_ISL_1189530 to 1189534, EPI_ISL_1189536 to 1189538, EPI_ISL_1189541, EPI_ISL_1189543, EPI_ISL_1189545, EPI_ISL_1189547 to 1189549, EPI_ISL_1189551 to 1189552, EPI_ISL_1189556, EPI_ISL_1189558, EPI_ISL_1189561, EPI_ISL_1189567, EPI_ISL_1189572 to 1189573, EPI_ISL_1189575, EPI_ISL_1189577 to 1189579, EPI_ISL_1189583, EPI_ISL_1189586 to 1189588, EPI_ISL_1189590, EPI_ISL_1189594 to 1189595, EPI_ISL_1189597 to 1189598, EPI_ISL_1189600 to 1189602, EPI_ISL_1189604 to 1189611, EPI_ISL_1189613 to 1189615, EPI_ISL_1189617, EPI_ISL_1189620, EPI_ISL_1189622, EPI_ISL_1189625, EPI_ISL_1189628 to 1189630, EPI_ISL_1189635 to 1189638, EPI_ISL_1189643, EPI_ISL_1189645, EPI_ISL_1189647 to 1189648, EPI_ISL_1189650 to 1189651, EPI_ISL_1189653, EPI_ISL_1189655, EPI_ISL_1189657, EPI_ISL_1189659, EPI_ISL_1189662 to 1189663, EPI_ISL_1189667, EPI_ISL_1189671 to 1189673, EPI_ISL_1189675, EPI_ISL_1189677, EPI_ISL_1189679 to 1189680, EPI_ISL_1189683 to 1189684, EPI_ISL_1189687, EPI_ISL_1189691, EPI_ISL_1189693, EPI_ISL_1189695, EPI_ISL_1189698, EPI_ISL_1189700 to 1189702, EPI_ISL_1189707, EPI_ISL_1189709 to 1189713, EPI_ISL_1189715 to 1189717, EPI_ISL_1189725, EPI_ISL_1189728, EPI_ISL_1189730 to 1189732, EPI_ISL_1189734 to 1189737, EPI_ISL_1189739 to 1189741, EPI_ISL_1189744 to 1189746, EPI_ISL_1189748, EPI_ISL_1189755 to 1189760, EPI_ISL_1189763, EPI_ISL_1189768, EPI_ISL_1189770 to 1189771, EPI_ISL_1189774, EPI_ISL_1189777, EPI_ISL_1189779 to 1189787, EPI_ISL_1189789, EPI_ISL_1189795 to 1189802, EPI_ISL_1189805 to 1189806, EPI_ISL_1189808 to 1189810, EPI_ISL_1189813 to 1189815, EPI_ISL_1189818, EPI_ISL_1189821, EPI_ISL_1189824 to 1189825, EPI_ISL_1189827 to 1189828, EPI_ISL_1189832 to 1189833, EPI_ISL_1189835 to 1189838, EPI_ISL_1190389 to 1190395, EPI_ISL_1190552 to 1190559, EPI_ISL_1190725, EPI_ISL_1190728, EPI_ISL_1205707, EPI_ISL_1205709 to 1205712, EPI_ISL_1205714 to 1205715, EPI_ISL_1205719 to 1205721, EPI_ISL_1205723 to 1205728, EPI_ISL_1205731, EPI_ISL_1205733, EPI_ISL_1205736 to 1205738, EPI_ISL_1205742, EPI_ISL_1205745, EPI_ISL_1205747 to 1205748, EPI_ISL_1205751 to 1205754, EPI_ISL_1205757 to 1205768, EPI_ISL_1205770 to 1205771, EPI_ISL_1205775 to 1205777, EPI_ISL_1205782, EPI_ISL_1205785, EPI_ISL_1205789, EPI_ISL_1205791, EPI_ISL_1205794, EPI_ISL_1205796 to 1205802, EPI_ISL_1205804 to 1205805, EPI_ISL_1205808, EPI_ISL_1205812, EPI_ISL_1205814, EPI_ISL_1205819, EPI_ISL_1205822, EPI_ISL_1205824 to 1205826, EPI_ISL_1205828, EPI_ISL_1205830 to 1205832, EPI_ISL_1205834, EPI_ISL_1205838 to 1205840, EPI_ISL_1205842 to 1205844, EPI_ISL_1205846, EPI_ISL_1205848, EPI_ISL_1205850, EPI_ISL_1205854 to 1205862, EPI_ISL_1205865, EPI_ISL_1205868, EPI_ISL_1205873, EPI_ISL_1205875 to 1205876, EPI_ISL_1205878 to 1205879, EPI_ISL_1205881 to 1205882, EPI_ISL_1205886 to 1205890, EPI_ISL_1205892 to 1205893, EPI_ISL_1205895 to 1205898, EPI_ISL_1205900 to 1205902, EPI_ISL_1205905 to 1205907, EPI_ISL_1205910 to 1205911, EPI_ISL_1205913 to 1205914, EPI_ISL_1205916, EPI_ISL_1205918, EPI_ISL_1205920, EPI_ISL_1205922 to 1205924, EPI_ISL_1205927, EPI_ISL_1205932, EPI_ISL_1205934, EPI_ISL_1205938 to 1205941, EPI_ISL_1205944, EPI_ISL_1205948 to 1205949, EPI_ISL_1205951, EPI_ISL_1205953 to 1205958, EPI_ISL_1205961 to 1205963, EPI_ISL_1205965, EPI_ISL_1205967, EPI_ISL_1205969 to 1205970, EPI_ISL_1205978 to 1205979, EPI_ISL_1205982, EPI_ISL_1205987 to 1205988, EPI_ISL_1205990 to 1205992, EPI_ISL_1205996 to 1205997, EPI_ISL_1206000 to 1206003, EPI_ISL_1206005 to 1206006, EPI_ISL_1206008, EPI_ISL_1206011, EPI_ISL_1206021 to 1206028, EPI_ISL_1206030 to 1206032, EPI_ISL_1206034, EPI_ISL_1206037 to 1206038, EPI_ISL_1206502 to 1206503, EPI_ISL_1206505 to 1206506, EPI_ISL_1206508, EPI_ISL_1206511 to 1206512, EPI_ISL_1206515, EPI_ISL_1206519, EPI_ISL_1206522, EPI_ISL_1206524, EPI_ISL_1206527, EPI_ISL_1206529 to 1206530, EPI_ISL_1206532 to 1206533, EPI_ISL_1206535, EPI_ISL_1206538 to 1206539, EPI_ISL_1206541, EPI_ISL_1206544 to 1206545, EPI_ISL_1206548, EPI_ISL_1206551 to 1206552, EPI_ISL_1206555 to 1206556, EPI_ISL_1206558 to 1206564, EPI_ISL_1206569, EPI_ISL_1206571, EPI_ISL_1206573, EPI_ISL_1206575, EPI_ISL_1206577, EPI_ISL_1206582 to 1206583, EPI_ISL_1206586 to 1206587, EPI_ISL_1206589 to 1206590, EPI_ISL_1206593, EPI_ISL_1206597, EPI_ISL_1206610, EPI_ISL_1206614 to 1206615, EPI_ISL_1206619, EPI_ISL_1206621 to 1206623, EPI_ISL_1206625 to 1206626, EPI_ISL_1206629, EPI_ISL_1206632 to 1206636, EPI_ISL_1206638, EPI_ISL_1206642 to 1206644, EPI_ISL_1206646, EPI_ISL_1206648, EPI_ISL_1206650, EPI_ISL_1206654, EPI_ISL_1206656, EPI_ISL_1206658, EPI_ISL_1206665, EPI_ISL_1206669 to 1206670, EPI_ISL_1206673 to 1206676, EPI_ISL_1206679, EPI_ISL_1206681, EPI_ISL_1206684 to 1206687, EPI_ISL_1206691 to 1206692, EPI_ISL_1206694 to 1206696, EPI_ISL_1206698, EPI_ISL_1206701 to 1206703, EPI_ISL_1206705 to 1206706, EPI_ISL_1206709 to 1206710, EPI_ISL_1206713 to 1206714, EPI_ISL_1206716, EPI_ISL_1206721, EPI_ISL_1206723 to 1206724, EPI_ISL_1206732, EPI_ISL_1206734 to 1206736, EPI_ISL_1206739 to 1206741, EPI_ISL_1206743, EPI_ISL_1206746 to 1206747, EPI_ISL_1206750, EPI_ISL_1206752, EPI_ISL_1206758 to 1206762, EPI_ISL_1206767, EPI_ISL_1206771 to 1206773, EPI_ISL_1206775, EPI_ISL_1206777, EPI_ISL_1206782, EPI_ISL_1206785, EPI_ISL_1206795 to 1206797, EPI_ISL_1206800, EPI_ISL_1206802 to 1206803, EPI_ISL_1206806, EPI_ISL_1206808, EPI_ISL_1206811 to 1206817, EPI_ISL_1206820, EPI_ISL_1206822, EPI_ISL_1206824 to 1206825, EPI_ISL_1206827 to 1206829, EPI_ISL_1206836 to 1206837, EPI_ISL_1206840, EPI_ISL_1206842, EPI_ISL_1206844 to 1206846, EPI_ISL_1206849 to 1206856, EPI_ISL_1206858 to 1206859, EPI_ISL_1206861 to 1206864, EPI_ISL_1206866, EPI_ISL_1206868, EPI_ISL_1206870, EPI_ISL_1206872 to 1206874, EPI_ISL_1206876 to 1206877, EPI_ISL_1206879, EPI_ISL_1206881, EPI_ISL_1206883, EPI_ISL_1206886 to 1206887, EPI_ISL_1206892, EPI_ISL_1206894 to 1206896, EPI_ISL_1206898 to 1206902, EPI_ISL_1206904, EPI_ISL_1206907 to 1206913, EPI_ISL_1206915 to 1206917, EPI_ISL_1206919, EPI_ISL_1206921 to 1206922, EPI_ISL_1206925 to 1206927, EPI_ISL_1206929 to 1206930, EPI_ISL_1206932 to 1206935, EPI_ISL_1206937 to 1206940, EPI_ISL_1206947, EPI_ISL_1206951 to 1206953, EPI_ISL_1206955, EPI_ISL_1206958 to 1206965, EPI_ISL_1206967 to 1206970, EPI_ISL_1206972 to 1206973, EPI_ISL_1206976 to 1206977, EPI_ISL_1206979 to 1206983, EPI_ISL_1206987 to 1206988, EPI_ISL_1206991, EPI_ISL_1206993, EPI_ISL_1206995, EPI_ISL_1206997 to 1206998, EPI_ISL_1207000 to 1207003, EPI_ISL_1207008, EPI_ISL_1207010 to 1207013, EPI_ISL_1207015, EPI_ISL_1207020 to 1207021, EPI_ISL_1207023 to 1207024, EPI_ISL_1207028 to 1207029, EPI_ISL_1207031, EPI_ISL_1207034 to 1207035, EPI_ISL_1207038 to 1207040, EPI_ISL_1207043 to 1207044, EPI_ISL_1207046 to 1207048, EPI_ISL_1207050 to 1207051, EPI_ISL_1207053 to 1207055, EPI_ISL_1207057, EPI_ISL_1207060, EPI_ISL_1207062 to 1207065, EPI_ISL_1207067 to 1207070, EPI_ISL_1207072 to 1207075, EPI_ISL_1207077, EPI_ISL_1207079 to 1207080, EPI_ISL_1207082 to 1207085, EPI_ISL_1207087 to 1207088, EPI_ISL_1207090, EPI_ISL_1207092 to 1207095, EPI_ISL_1207097 to 1207098, EPI_ISL_1207100 to 1207104, EPI_ISL_1207106, EPI_ISL_1207108 to 1207109, EPI_ISL_1207111 to 1207112, EPI_ISL_1207114 to 1207122, EPI_ISL_1207125 to 1207126, EPI_ISL_1207128, EPI_ISL_1207131 to 1207132, EPI_ISL_1207135 to 1207137, EPI_ISL_1207139, EPI_ISL_1207144 to 1207149, EPI_ISL_1207153 to 1207154, EPI_ISL_1207158, EPI_ISL_1207161 to 1207162, EPI_ISL_1207168, EPI_ISL_1207170 to 1207171, EPI_ISL_1207178, EPI_ISL_1207182 to 1207183, EPI_ISL_1207187, EPI_ISL_1207189, EPI_ISL_1207199, EPI_ISL_1207204, EPI_ISL_1207206, EPI_ISL_1207210 to 1207212, EPI_ISL_1207214, EPI_ISL_1207217, EPI_ISL_1207219, EPI_ISL_1207228, EPI_ISL_1207233, EPI_ISL_1207239 to 1207242, EPI_ISL_1207244, EPI_ISL_1207252, EPI_ISL_1207255 to 1207257, EPI_ISL_1207260, EPI_ISL_1207263, EPI_ISL_1207269, EPI_ISL_1207271, EPI_ISL_1207275, EPI_ISL_1207278 to 1207279, EPI_ISL_1207284 to 1207286, EPI_ISL_1207290, EPI_ISL_1207295, EPI_ISL_1207297, EPI_ISL_1207299, EPI_ISL_1207305, EPI_ISL_1207308, EPI_ISL_1207311, EPI_ISL_1207314, EPI_ISL_1207319, EPI_ISL_1207330, EPI_ISL_1207332, EPI_ISL_1207349 to 1207352, EPI_ISL_1207354, EPI_ISL_1207360, EPI_ISL_1207363, EPI_ISL_1207370 to 1207371, EPI_ISL_1207373 to 1207374, EPI_ISL_1207380, EPI_ISL_1207383, EPI_ISL_1207386, EPI_ISL_1207389, EPI_ISL_1207393, EPI_ISL_1207395 to 1207396, EPI_ISL_1207399, EPI_ISL_1207401, EPI_ISL_1207417, EPI_ISL_1207419, EPI_ISL_1207423, EPI_ISL_1207426, EPI_ISL_1207428, EPI_ISL_1207434 to 1207435, EPI_ISL_1207438 to 1207439, EPI_ISL_1207441, EPI_ISL_1207444, EPI_ISL_1207453, EPI_ISL_1207456 to 1207457, EPI_ISL_1207465, EPI_ISL_1207468 to 1207469, EPI_ISL_1207473, EPI_ISL_1207475 to 1207480, EPI_ISL_1207483 to 1207485, EPI_ISL_1207487, EPI_ISL_1207489 to 1207490, EPI_ISL_1207492 to 1207493, EPI_ISL_1207495, EPI_ISL_1207498, EPI_ISL_1207500, EPI_ISL_1207503 to 1207505, EPI_ISL_1207507 to 1207508, EPI_ISL_1207512 to 1207513, EPI_ISL_1207516, EPI_ISL_1207518 to 1207519, EPI_ISL_1207523 to 1207525, EPI_ISL_1207528 to 1207531, EPI_ISL_1207534 to 1207537, EPI_ISL_1207540, EPI_ISL_1207547 to 1207548, EPI_ISL_1207550, EPI_ISL_1207552 to 1207553, EPI_ISL_1207555 to 1207556, EPI_ISL_1207558 to 1207564, EPI_ISL_1207566 to 1207569, EPI_ISL_1207571 to 1207574, EPI_ISL_1207576, EPI_ISL_1207578 to 1207579, EPI_ISL_1207582 to 1207583, EPI_ISL_1207585, EPI_ISL_1207588 to 1207590, EPI_ISL_1207601 to 1207603, EPI_ISL_1207605 to 1207606, EPI_ISL_1207609, EPI_ISL_1207611 to 1207613, EPI_ISL_1207615 to 1207616, EPI_ISL_1207619 to 1207621, EPI_ISL_1207626 to 1207629, EPI_ISL_1207636 to 1207637, EPI_ISL_1207640 to 1207642, EPI_ISL_1207645, EPI_ISL_1207647, EPI_ISL_1207650, EPI_ISL_1207652 to 1207653, EPI_ISL_1207656, EPI_ISL_1207658, EPI_ISL_1207660, EPI_ISL_1207664 to 1207666, EPI_ISL_1207668, EPI_ISL_1207670, EPI_ISL_1207673, EPI_ISL_1207675, EPI_ISL_1207677, EPI_ISL_1207679 to 1207683, EPI_ISL_1207685, EPI_ISL_1207687, EPI_ISL_1207689, EPI_ISL_1207691 to 1207692, EPI_ISL_1207695, EPI_ISL_1207699, EPI_ISL_1207702 to 1207703, EPI_ISL_1207707, EPI_ISL_1207712 to 1207714, EPI_ISL_1207716 to 1207718, EPI_ISL_1207720 to 1207721, EPI_ISL_1207723 to 1207724, EPI_ISL_1207726, EPI_ISL_1207731 to 1207732, EPI_ISL_1207735, EPI_ISL_1207739 to 1207742, EPI_ISL_1207744 to 1207748, EPI_ISL_1207750, EPI_ISL_1207752, EPI_ISL_1207755, EPI_ISL_1207760 to 1207763, EPI_ISL_1207765, EPI_ISL_1207768, EPI_ISL_1207770, EPI_ISL_1207772 to 1207773, EPI_ISL_1207776 to 1207777, EPI_ISL_1207779, EPI_ISL_1207781, EPI_ISL_1207784 to 1207786, EPI_ISL_1207788 to 1207791, EPI_ISL_1207801, EPI_ISL_1207804, EPI_ISL_1207806 to 1207808, EPI_ISL_1208077, EPI_ISL_1208090, EPI_ISL_1208110, EPI_ISL_1208262 to 1208264, EPI_ISL_1208356 to 1208357, EPI_ISL_1208359 to 1208361, EPI_ISL_1208363 to 1208366, EPI_ISL_1223658, EPI_ISL_1223660, EPI_ISL_1223666, EPI_ISL_1223670, EPI_ISL_1223672, EPI_ISL_1223674, EPI_ISL_1223680, EPI_ISL_1223682, EPI_ISL_1223686 to 1223687, EPI_ISL_1223689, EPI_ISL_1223695, EPI_ISL_1223699, EPI_ISL_1223704, EPI_ISL_1223706 to 1223708, EPI_ISL_1223717 to 1223719, EPI_ISL_1223725, EPI_ISL_1223732 to 1223734, EPI_ISL_1223737, EPI_ISL_1223740, EPI_ISL_1223742 to 1223743, EPI_ISL_1223746, EPI_ISL_1223750, EPI_ISL_1223761, EPI_ISL_1223765, EPI_ISL_1223767, EPI_ISL_1223769, EPI_ISL_1223777, EPI_ISL_1223780, EPI_ISL_1223792, EPI_ISL_1223794, EPI_ISL_1223796, EPI_ISL_1223799 to 1223800, EPI_ISL_1223802, EPI_ISL_1223811, EPI_ISL_1223817, EPI_ISL_1223820, EPI_ISL_1223822 to 1223823, EPI_ISL_1223828, EPI_ISL_1223830, EPI_ISL_1223833, EPI_ISL_1223835 to 1223836, EPI_ISL_1223839 to 1223841, EPI_ISL_1223843 to 1223845, EPI_ISL_1223847, EPI_ISL_1223858, EPI_ISL_1223862, EPI_ISL_1223866 to 1223867, EPI_ISL_1223869, EPI_ISL_1223874 to 1223875, EPI_ISL_1223879 to 1223880, EPI_ISL_1223883 to 1223884, EPI_ISL_1223890, EPI_ISL_1223892 to 1223893, EPI_ISL_1223897, EPI_ISL_1223900, EPI_ISL_1223905, EPI_ISL_1223909 to 1223910, EPI_ISL_1223915, EPI_ISL_1223920, EPI_ISL_1223923 to 1223924, EPI_ISL_1223927, EPI_ISL_1223929, EPI_ISL_1223931, EPI_ISL_1223935, EPI_ISL_1224707 to 1224745, EPI_ISL_1242828, EPI_ISL_1242831 to 1242832, EPI_ISL_1242834, EPI_ISL_1242839 to 1242840, EPI_ISL_1242845 to 1242846, EPI_ISL_1242848 to 1242850, EPI_ISL_1242854, EPI_ISL_1242857, EPI_ISL_1242859, EPI_ISL_1242861, EPI_ISL_1242866, EPI_ISL_1242868 to 1242870, EPI_ISL_1242873 to 1242879, EPI_ISL_1242882, EPI_ISL_1242891 to 1242892, EPI_ISL_1242895, EPI_ISL_1242899 to 1242901, EPI_ISL_1242903 to 1242904, EPI_ISL_1242911, EPI_ISL_1242913, EPI_ISL_1242915, EPI_ISL_1242917 to 1242919, EPI_ISL_1242921 to 1242923, EPI_ISL_1242933, EPI_ISL_1242936 to 1242937, EPI_ISL_1242940 to 1242942, EPI_ISL_1242944, EPI_ISL_1242949 to 1242950, EPI_ISL_1242953, EPI_ISL_1242962 to 1242963, EPI_ISL_1242965, EPI_ISL_1242967, EPI_ISL_1242972 to 1242973, EPI_ISL_1242975, EPI_ISL_1242978, EPI_ISL_1242984 to 1242985, EPI_ISL_1242988, EPI_ISL_1242990 to 1242994, EPI_ISL_1242997, EPI_ISL_1243004 to 1243005, EPI_ISL_1243007, EPI_ISL_1243015, EPI_ISL_1243017 to 1243018, EPI_ISL_1243021, EPI_ISL_1243027, EPI_ISL_1243029 to 1243030, EPI_ISL_1243032 to 1243033, EPI_ISL_1243035, EPI_ISL_1243040, EPI_ISL_1243043 to 1243044, EPI_ISL_1243047 to 1243048, EPI_ISL_1243053 to 1243055, EPI_ISL_1243060, EPI_ISL_1243065, EPI_ISL_1243068 to 1243070, EPI_ISL_1243075 to 1243078, EPI_ISL_1243082, EPI_ISL_1243084,

EPI_ISL_1243087, EPI_ISL_1243092 to 1243095, EPI_ISL_1243098, EPI_ISL_1243101 to 1243102, EPI_ISL_1243105, EPI_ISL_1243111, EPI_ISL_1243117, EPI_ISL_1243119, EPI_ISL_1243126 to 1243128, EPI_ISL_1243134, EPI_ISL_1243138, EPI_ISL_1243145 to 1243146, EPI_ISL_1243150, EPI_ISL_1243152, EPI_ISL_1243156 to 1243158, EPI_ISL_1243160, EPI_ISL_1243164, EPI_ISL_1243166 to 1243169, EPI_ISL_1243171, EPI_ISL_1243175, EPI_ISL_1243179, EPI_ISL_1243181, EPI_ISL_1243183 to 1243184, EPI_ISL_1243187, EPI_ISL_1243190, EPI_ISL_1243194, EPI_ISL_1243197 to 1243201, EPI_ISL_1243205 to 1243217, EPI_ISL_1243221 to 1243225, EPI_ISL_1243227, EPI_ISL_1243229, EPI_ISL_1243231 to 1243236, EPI_ISL_1243241, EPI_ISL_1243243, EPI_ISL_1243245 to 1243246, EPI_ISL_1243248, EPI_ISL_1243250 to 1243253, EPI_ISL_1243256 to 1243257, EPI_ISL_1243259, EPI_ISL_1243261, EPI_ISL_1243263, EPI_ISL_1243266 to 1243270, EPI_ISL_1243272, EPI_ISL_1243274, EPI_ISL_1243277 to 1243278, EPI_ISL_1243280, EPI_ISL_1243283 to 1243291, EPI_ISL_1243294 to 1243295, EPI_ISL_1243297 to 1243299, EPI_ISL_1243301 to 1243302, EPI_ISL_1243305, EPI_ISL_1243307, EPI_ISL_1243309, EPI_ISL_1243311 to 1243314, EPI_ISL_1243316, EPI_ISL_1243318 to 1243322, EPI_ISL_1243325 to 1243326, EPI_ISL_1243328, EPI_ISL_1243330 to 1243336, EPI_ISL_1243338, EPI_ISL_1243342 to 1243345, EPI_ISL_1243348 to 1243349, EPI_ISL_1243352, EPI_ISL_1243354 to 1243357, EPI_ISL_1243360, EPI_ISL_1243362, EPI_ISL_1243364 to 1243365, EPI_ISL_1243367 to 1243368, EPI_ISL_1243370 to 1243373, EPI_ISL_1243375 to 1243376, EPI_ISL_1243381 to 1243382, EPI_ISL_1243384, EPI_ISL_1243386, EPI_ISL_1243388 to 1243393, EPI_ISL_1243397 to 1243400, EPI_ISL_1243402, EPI_ISL_1243406, EPI_ISL_1243409 to 1243410, EPI_ISL_1243412 to 1243415, EPI_ISL_1243417 to 1243420, EPI_ISL_1243430 to 1243433, EPI_ISL_1243613, EPI_ISL_1243615, EPI_ISL_1243618 to 1243619, EPI_ISL_1243624, EPI_ISL_1243629 to 1243630, EPI_ISL_1243635 to 1243637, EPI_ISL_1243642, EPI_ISL_1243644 to 1243645, EPI_ISL_1243652, EPI_ISL_1243654, EPI_ISL_1243656 to 1243657, EPI_ISL_1243660 to 1243665, EPI_ISL_1243668, EPI_ISL_1243670, EPI_ISL_1243672 to 1243675, EPI_ISL_1243684, EPI_ISL_1243687 to 1243688, EPI_ISL_1243690, EPI_ISL_1243696, EPI_ISL_1243698 to 1243699, EPI_ISL_1243703, EPI_ISL_1243707 to 1243708, EPI_ISL_1243715, EPI_ISL_1243725 to 1243726, EPI_ISL_1243728 to 1243729, EPI_ISL_1243734 to 1243736, EPI_ISL_1243738, EPI_ISL_1243740, EPI_ISL_1243743 to 1243745, EPI_ISL_1243747 to 1243749, EPI_ISL_1243755, EPI_ISL_1243757, EPI_ISL_1243759 to 1243760, EPI_ISL_1243766, EPI_ISL_1243777, EPI_ISL_1243779, EPI_ISL_1243781 to 1243783, EPI_ISL_1243786 to 1243788, EPI_ISL_1243791 to 1243792, EPI_ISL_1243795, EPI_ISL_1243797, EPI_ISL_1243799, EPI_ISL_1243804, EPI_ISL_1243806 to 1243808, EPI_ISL_1243812, EPI_ISL_1243815 to 1243816, EPI_ISL_1243822, EPI_ISL_1243824 to 1243826, EPI_ISL_1243832, EPI_ISL_1243837, EPI_ISL_1243843, EPI_ISL_1243845, EPI_ISL_1243847, EPI_ISL_1243849, EPI_ISL_1243854, EPI_ISL_1243856, EPI_ISL_1243858 to 1243865, EPI_ISL_1243867, EPI_ISL_1243869, EPI_ISL_1243872, EPI_ISL_1243874 to 1243877, EPI_ISL_1243881 to 1243882, EPI_ISL_1243887, EPI_ISL_1243890 to 1243891, EPI_ISL_1243895, EPI_ISL_1243897 to 1243903, EPI_ISL_1243905, EPI_ISL_1243908 to 1243909, EPI_ISL_1243911, EPI_ISL_1243918, EPI_ISL_1243921 to 1243924, EPI_ISL_1243929, EPI_ISL_1243935 to 1243936, EPI_ISL_1243940, EPI_ISL_1243944 to 1243945, EPI_ISL_1243952, EPI_ISL_1243956 to 1243957, EPI_ISL_1243961 to 1243962, EPI_ISL_1243966, EPI_ISL_1243968, EPI_ISL_1243970, EPI_ISL_1243972 to 1243973, EPI_ISL_1243979, EPI_ISL_1243984, EPI_ISL_1243991 to 1243993, EPI_ISL_1243998, EPI_ISL_1244001 to 1244002, EPI_ISL_1244004 to 1244005, EPI_ISL_1244009, EPI_ISL_1244013 to 1244015, EPI_ISL_1244019, EPI_ISL_1244028, EPI_ISL_1244032, EPI_ISL_1244039, EPI_ISL_1244043, EPI_ISL_1244046 to 1244048, EPI_ISL_1244051, EPI_ISL_1244057, EPI_ISL_1244061, EPI_ISL_1244064 to 1244065, EPI_ISL_1244068 to 1244071, EPI_ISL_1244073 to 1244074, EPI_ISL_1244078 to 1244080, EPI_ISL_1244086, EPI_ISL_1244091 to 1244092, EPI_ISL_1244095, EPI_ISL_1244097, EPI_ISL_1244099 to 1244101, EPI_ISL_1244104, EPI_ISL_1244106, EPI_ISL_1244108, EPI_ISL_1244112, EPI_ISL_1244114, EPI_ISL_1244120, EPI_ISL_1244124 to 1244125, EPI_ISL_1244128, EPI_ISL_1244131, EPI_ISL_1244138, EPI_ISL_1244142, EPI_ISL_1244144, EPI_ISL_1244157, EPI_ISL_1244164 to 1244165, EPI_ISL_1244171 to 1244172, EPI_ISL_1244176, EPI_ISL_1244178 to 1244179, EPI_ISL_1244181, EPI_ISL_1244191, EPI_ISL_1244193, EPI_ISL_1244201, EPI_ISL_1244205, EPI_ISL_1244208, EPI_ISL_1244213 to 1244214, EPI_ISL_1244221 to 1244222, EPI_ISL_1244224 to 1244226, EPI_ISL_1244229 to 1244231, EPI_ISL_1244241 to 1244242, EPI_ISL_1244248, EPI_ISL_1244254, EPI_ISL_1244266, EPI_ISL_1244269 to 1244270, EPI_ISL_1244275, EPI_ISL_1244277, EPI_ISL_1244280, EPI_ISL_1244282, EPI_ISL_1244288, EPI_ISL_1244290 to 1244294, EPI_ISL_1244296 to 1244298, EPI_ISL_1244300, EPI_ISL_1244302, EPI_ISL_1244304 to 1244307, EPI_ISL_1244309 to 1244315, EPI_ISL_1244318 to 1244320, EPI_ISL_1244323 to 1244324, EPI_ISL_1244326, EPI_ISL_1244329 to 1244330, EPI_ISL_1244332 to 1244333, EPI_ISL_1244335 to 1244336, EPI_ISL_1244339, EPI_ISL_1244341 to 1244353, EPI_ISL_1244356 to 1244367, EPI_ISL_1244369 to 1244370, EPI_ISL_1244372 to 1244374, EPI_ISL_1244376 to 1244382, EPI_ISL_1244384, EPI_ISL_1244386 to 1244388, EPI_ISL_1244391 to 1244393, EPI_ISL_1244395 to 1244396, EPI_ISL_1244399 to 1244401, EPI_ISL_1244404 to 1244409, EPI_ISL_1244413 to 1244416, EPI_ISL_1244418 to 1244424, EPI_ISL_1244426 to 1244430, EPI_ISL_1244433 to 1244445, EPI_ISL_1244448 to 1244457, EPI_ISL_1244461 to 1244465, EPI_ISL_1244467 to 1244468, EPI_ISL_1244470 to 1244471, EPI_ISL_1244473 to 1244474, EPI_ISL_1244477 to 1244483, EPI_ISL_1244486 to 1244487, EPI_ISL_1244489 to 1244495, EPI_ISL_1244498 to 1244505, EPI_ISL_1244507 to 1244512, EPI_ISL_1244514 to 1244516, EPI_ISL_1244519 to 1244520, EPI_ISL_1244522 to 1244527, EPI_ISL_1244529 to 1244535, EPI_ISL_1244537 to 1244540, EPI_ISL_1244542 to 1244546, EPI_ISL_1244548 to 1244552, EPI_ISL_1244554 to 1244556, EPI_ISL_1244558 to 1244561, EPI_ISL_1244563 to 1244564, EPI_ISL_1244566 to 1244573, EPI_ISL_1244575, EPI_ISL_1244577 to 1244581, EPI_ISL_1244583 to 1244593, EPI_ISL_1244595, EPI_ISL_1244597 to 1244602, EPI_ISL_1244604, EPI_ISL_1244606 to 1244608, EPI_ISL_1244610 to 1244611, EPI_ISL_1245360, EPI_ISL_1245363, EPI_ISL_1245367, EPI_ISL_1245370 to 1245371, EPI_ISL_1245375, EPI_ISL_1245379 to 1245380, EPI_ISL_1245393, EPI_ISL_1245395, EPI_ISL_1245400 to 1245401, EPI_ISL_1245406, EPI_ISL_1245410 to 1245412, EPI_ISL_1245418 to 1245421, EPI_ISL_1245424, EPI_ISL_1245427, EPI_ISL_1245430, EPI_ISL_1245432 to 1245433, EPI_ISL_1245436, EPI_ISL_1245438 to 1245439, EPI_ISL_1245445, EPI_ISL_1245447, EPI_ISL_1245450 to 1245451, EPI_ISL_1245457, EPI_ISL_1245462, EPI_ISL_1245466, EPI_ISL_1245473 to 1245474, EPI_ISL_1245476, EPI_ISL_1245479 to 1245480, EPI_ISL_1245482 to 1245484, EPI_ISL_1245488, EPI_ISL_1245491 to 1245494, EPI_ISL_1245501, EPI_ISL_1245504, EPI_ISL_1245510, EPI_ISL_1245515, EPI_ISL_1245517 to 1245518, EPI_ISL_1245524, EPI_ISL_1245526 to 1245527, EPI_ISL_1245529, EPI_ISL_1245533, EPI_ISL_1245535, EPI_ISL_1245538, EPI_ISL_1245541, EPI_ISL_1245544, EPI_ISL_1245547 to 1245549, EPI_ISL_1245553 to 1245555, EPI_ISL_1245558, EPI_ISL_1245560, EPI_ISL_1245566 to 1245571, EPI_ISL_1245573, EPI_ISL_1245576 to 1245577, EPI_ISL_1245584, EPI_ISL_1245586, EPI_ISL_1245588 to 1245589, EPI_ISL_1245591, EPI_ISL_1245594 to 1245595, EPI_ISL_1245598 to 1245599, EPI_ISL_1245603, EPI_ISL_1245605 to 1245606, EPI_ISL_1245610, EPI_ISL_1245613 to 1245615, EPI_ISL_1245620 to 1245621, EPI_ISL_1245623 to 1245625, EPI_ISL_1245627 to 1245628, EPI_ISL_1245630 to 1245632, EPI_ISL_1245634 to 1245637, EPI_ISL_1245639, EPI_ISL_1245641, EPI_ISL_1245645 to 1245646, EPI_ISL_1245648, EPI_ISL_1245653, EPI_ISL_1245655, EPI_ISL_1245662, EPI_ISL_1245665 to 1245666, EPI_ISL_1245677, EPI_ISL_1245684 to 1245688, EPI_ISL_1245691, EPI_ISL_1245693 to 1245694, EPI_ISL_1245696 to 1245698, EPI_ISL_1245700, EPI_ISL_1245703, EPI_ISL_1245705, EPI_ISL_1245707 to 1245712, EPI_ISL_1245715 to 1245716, EPI_ISL_1245718, EPI_ISL_1245720, EPI_ISL_1245722 to 1245723, EPI_ISL_1245725 to 1245726, EPI_ISL_1245728 to 1245729, EPI_ISL_1245732, EPI_ISL_1245734 to 1245738, EPI_ISL_1245740 to 1245741, EPI_ISL_1245743 to 1245746, EPI_ISL_1245751, EPI_ISL_1245754, EPI_ISL_1245758 to 1245759, EPI_ISL_1245761, EPI_ISL_1245763 to 1245766, EPI_ISL_1245768 to 1245772, EPI_ISL_1245774 to 1245775, EPI_ISL_1245778 to 1245781, EPI_ISL_1245785, EPI_ISL_1245788, EPI_ISL_1245793 to 1245800, EPI_ISL_1245802 to 1245804, EPI_ISL_1245806 to 1245807, EPI_ISL_1245809 to 1245813, EPI_ISL_1245816 to 1245821, EPI_ISL_1245824 to 1245829, EPI_ISL_1245831 to 1245833, EPI_ISL_1245835 to 1245846, EPI_ISL_1245848 to 1245849, EPI_ISL_1245851, EPI_ISL_1245853 to 1245854, EPI_ISL_1245856 to 1245861, EPI_ISL_1245865 to 1245876, EPI_ISL_1245878, EPI_ISL_1245880, EPI_ISL_1245882, EPI_ISL_1245884 to 1245885, EPI_ISL_1245887, EPI_ISL_1245889, EPI_ISL_1245891 to 1245894, EPI_ISL_1245897 to 1245902, EPI_ISL_1245904 to 1245907, EPI_ISL_1245909, EPI_ISL_1245912 to 1245913, EPI_ISL_1245915, EPI_ISL_1245917 to 1245920, EPI_ISL_1245922 to 1245926, EPI_ISL_1245928 to 1245931, EPI_ISL_1245934, EPI_ISL_1245936 to 1245949, EPI_ISL_1245951 to 1245953, EPI_ISL_1245955, EPI_ISL_1245959, EPI_ISL_1245961, EPI_ISL_1245965 to 1245966, EPI_ISL_1245970 to 1245973, EPI_ISL_1245975 to 1245976, EPI_ISL_1245979 to 1245984, EPI_ISL_1245988 to 1245989, EPI_ISL_1245991, EPI_ISL_1245993, EPI_ISL_1245996, EPI_ISL_1246000 to 1246004, EPI_ISL_1246006 to 1246011, EPI_ISL_1246013, EPI_ISL_1246015 to 1246017, EPI_ISL_1246856 to 1246874, EPI_ISL_1246900, EPI_ISL_1255425, EPI_ISL_1255429 to 1255430, EPI_ISL_1255435 to 1255436, EPI_ISL_1255442 to 1255443, EPI_ISL_1255445, EPI_ISL_1255447, EPI_ISL_1255451, EPI_ISL_1255453 to 1255454, EPI_ISL_1255458, EPI_ISL_1255461, EPI_ISL_1255464, EPI_ISL_1255466, EPI_ISL_1255469 to 1255470, EPI_ISL_1255472 to 1255473, EPI_ISL_1255481, EPI_ISL_1255486, EPI_ISL_1255488 to 1255490, EPI_ISL_1255493 to 1255494, EPI_ISL_1255496, EPI_ISL_1255502 to 1255503, EPI_ISL_1255509, EPI_ISL_1255514 to 1255515, EPI_ISL_1255520, EPI_ISL_1255523 to 1255524, EPI_ISL_1255528, EPI_ISL_1255535, EPI_ISL_1255537 to 1255538, EPI_ISL_1255540, EPI_ISL_1255545 to 1255547, EPI_ISL_1255550, EPI_ISL_1255558, EPI_ISL_1255561, EPI_ISL_1255563, EPI_ISL_1255566 to 1255567, EPI_ISL_1255570, EPI_ISL_1255572 to 1255574, EPI_ISL_1255576, EPI_ISL_1255579 to 1255580, EPI_ISL_1255584 to 1255587, EPI_ISL_1255594, EPI_ISL_1255596, EPI_ISL_1255598 to 1255600, EPI_ISL_1255602 to 1255603, EPI_ISL_1255605, EPI_ISL_1255612, EPI_ISL_1255614, EPI_ISL_1255617, EPI_ISL_1255622, EPI_ISL_1255624 to 1255625, EPI_ISL_1255628 to 1255629, EPI_ISL_1255631 to 1255632, EPI_ISL_1255634 to 1255636, EPI_ISL_1255643, EPI_ISL_1255645 to 1255646, EPI_ISL_1255651, EPI_ISL_1255655 to 1255656, EPI_ISL_1255659, EPI_ISL_1255661 to 1255663, EPI_ISL_1255667, EPI_ISL_1255671, EPI_ISL_1255674, EPI_ISL_1255678, EPI_ISL_1255680, EPI_ISL_1255682, EPI_ISL_1255684 to 1255685, EPI_ISL_1255687, EPI_ISL_1255689, EPI_ISL_1255691 to 1255693, EPI_ISL_1255695 to 1255698, EPI_ISL_1255703 to 1255705, EPI_ISL_1255707 to 1255710, EPI_ISL_1255712, EPI_ISL_1255716, EPI_ISL_1255719 to 1255730, EPI_ISL_1255732 to 1255733, EPI_ISL_1255735 to 1255740, EPI_ISL_1255744, EPI_ISL_1255746, EPI_ISL_1255748, EPI_ISL_1255750 to 1255759, EPI_ISL_1255761 to 1255762, EPI_ISL_1255764 to 1255769, EPI_ISL_1255771 to 1255774, EPI_ISL_1255776, EPI_ISL_1255778 to 1255791, EPI_ISL_1255793, EPI_ISL_1255795, EPI_ISL_1255799 to 1255801, EPI_ISL_1255804 to 1255811, EPI_ISL_1255816 to 1255821, EPI_ISL_1255824 to 1255825, EPI_ISL_1255828 to 1255829, EPI_ISL_1255831 to 1255834, EPI_ISL_1255836 to 1255838, EPI_ISL_1255840 to 1255847, EPI_ISL_1255849 to 1255851, EPI_ISL_1255853, EPI_ISL_1255855 to 1255858, EPI_ISL_1255860, EPI_ISL_1255863, EPI_ISL_1255866 to 1255873, EPI_ISL_1255876 to 1255878, EPI_ISL_1255880 to 1255888, EPI_ISL_1255890, EPI_ISL_1255892 to 1255893, EPI_ISL_1255895 to 1255903, EPI_ISL_1255905 to 1255907, EPI_ISL_1255909 to 1255914, EPI_ISL_1255916 to 1255918, EPI_ISL_1255920, EPI_ISL_1255923 to 1255926, EPI_ISL_1255928 to 1255931, EPI_ISL_1255933 to 1255934, EPI_ISL_1255941 to 1255942, EPI_ISL_1255945, EPI_ISL_1255948, EPI_ISL_1255952 to 1255953, EPI_ISL_1255955, EPI_ISL_1255958 to 1255959, EPI_ISL_1255961 to 1255962, EPI_ISL_1255964 to 1255965, EPI_ISL_1255967 to 1255968, EPI_ISL_1255971, EPI_ISL_1255973 to 1255974, EPI_ISL_1255976, EPI_ISL_1255978, EPI_ISL_1255980 to 1255981, EPI_ISL_1255983 to 1255984, EPI_ISL_1255986 to 1255987, EPI_ISL_1255989 to 1255990, EPI_ISL_1255992 to 1255993, EPI_ISL_1255995 to 1255996, EPI_ISL_1255998, EPI_ISL_1256001, EPI_ISL_1256003 to 1256004, EPI_ISL_1256006 to 1256007, EPI_ISL_1256009 to 1256010, EPI_ISL_1256015, EPI_ISL_1256017 to 1256018, EPI_ISL_1256020, EPI_ISL_1256022 to 1256023, EPI_ISL_1256026, EPI_ISL_1256030 to 1256031, EPI_ISL_1256033 to 1256034, EPI_ISL_1256037, EPI_ISL_1256040, EPI_ISL_1256044, EPI_ISL_1256047, EPI_ISL_1256049 to 1256050, EPI_ISL_1256052, EPI_ISL_1256054 to 1256058, EPI_ISL_1256060 to 1256061, EPI_ISL_1256063 to 1256064, EPI_ISL_1256069 to 1256072, EPI_ISL_1256075 to 1256082, EPI_ISL_1256084 to 1256086, EPI_ISL_1256088 to 1256096, EPI_ISL_1256098, EPI_ISL_1256101 to 1256102, EPI_ISL_1256105 to 1256106, EPI_ISL_1256110 to 1256117, EPI_ISL_1256119 to 1256123, EPI_ISL_1256125 to 1256126, EPI_ISL_1256128 to 1256135, EPI_ISL_1256138 to 1256142, EPI_ISL_1256144 to 1256146, EPI_ISL_1256148 to 1256155, EPI_ISL_1256157 to 1256158, EPI_ISL_1256160 to 1256162, EPI_ISL_1256164 to 1256167, EPI_ISL_1256169 to 1256170, EPI_ISL_1256172 to 1256178, EPI_ISL_1256180 to 1256187, EPI_ISL_1256189 to 1256196, EPI_ISL_1256198 to 1256205, EPI_ISL_1256207 to 1256220, EPI_ISL_1256222 to 1256226, EPI_ISL_1256228 to 1256230, EPI_ISL_1256232, EPI_ISL_1256234 to 1256241, EPI_ISL_1256243 to 1256246, EPI_ISL_1256248 to 1256253, EPI_ISL_1256256 to 1256261, EPI_ISL_1256263, EPI_ISL_1256266 to 1256272, EPI_ISL_1256276 to 1256277, EPI_ISL_1256279 to 1256281, EPI_ISL_1256284, EPI_ISL_1256286 to 1256295, EPI_ISL_1256298 to 1256305, EPI_ISL_1256307 to 1256317, EPI_ISL_1256319 to 1256323, EPI_ISL_1256325 to 1256327, EPI_ISL_1256330, EPI_ISL_1256332 to 1256335, EPI_ISL_1256337 to 1256338, EPI_ISL_1256340 to 1256342, EPI_ISL_1256344, EPI_ISL_1256346, EPI_ISL_1256348 to 1256350, EPI_ISL_1256352, EPI_ISL_1256354 to 1256355, EPI_ISL_1256357 to 1256360, EPI_ISL_1256362 to 1256363, EPI_ISL_1256683 to 1256684, EPI_ISL_1256686, EPI_ISL_1256692 to 1256693, EPI_ISL_1256696, EPI_ISL_1256699, EPI_ISL_1256705 to 1256706, EPI_ISL_1256708, EPI_ISL_1256710 to 1256711, EPI_ISL_1256713, EPI_ISL_1256720, EPI_ISL_1256722, EPI_ISL_1256727 to 1256732, EPI_ISL_1256736 to 1256737, EPI_ISL_1256740, EPI_ISL_1256742 to 1256743, EPI_ISL_1256746, EPI_ISL_1256748, EPI_ISL_1256751, EPI_ISL_1256754, EPI_ISL_1256762 to 1256763, EPI_ISL_1256768 to 1256769, EPI_ISL_1256773 to 1256775, EPI_ISL_1256778, EPI_ISL_1256780 to 1256781, EPI_ISL_1256785, EPI_ISL_1256790 to 1256792, EPI_ISL_1256794, EPI_ISL_1256797, EPI_ISL_1256799 to 1256801, EPI_ISL_1256803, EPI_ISL_1256805 to 1256807, EPI_ISL_1256811 to 1256814, EPI_ISL_1256816, EPI_ISL_1256818, EPI_ISL_1256825, EPI_ISL_1256831 to 1256832, EPI_ISL_1256835 to 1256836, EPI_ISL_1256842, EPI_ISL_1256844 to 1256845, EPI_ISL_1256848 to 1256849, EPI_ISL_1256854 to 1256855, EPI_ISL_1256860 to 1256861, EPI_ISL_1256863 to 1256864, EPI_ISL_1256866, EPI_ISL_1256873 to 1256875, EPI_ISL_1256881 to 1256882, EPI_ISL_1256885, EPI_ISL_1256888, EPI_ISL_1256890, EPI_ISL_1256896 to 1256897, EPI_ISL_1256901 to 1256902, EPI_ISL_1256909, EPI_ISL_1256918, EPI_ISL_1256920 to 1256921, EPI_ISL_1256923, EPI_ISL_1256926 to 1256928, EPI_ISL_1256930, EPI_ISL_1256932, EPI_ISL_1256935 to 1256936, EPI_ISL_1256938, EPI_ISL_1256940, EPI_ISL_1264072, EPI_ISL_1264085, EPI_ISL_1264099, EPI_ISL_1264108, EPI_ISL_1264127, EPI_ISL_1264129, EPI_ISL_1264143, EPI_ISL_1264151, EPI_ISL_1264157, EPI_ISL_1264166, EPI_ISL_1264174, EPI_ISL_1264178, EPI_ISL_1264186, EPI_ISL_1264232 to 1264233, EPI_ISL_1264235, EPI_ISL_1264254, EPI_ISL_1264256, EPI_ISL_1264295, EPI_ISL_1264297, EPI_ISL_1264302, EPI_ISL_1264336, EPI_ISL_1264349, EPI_ISL_1264352, EPI_ISL_1264355, EPI_ISL_1264357, EPI_ISL_1264378, EPI_ISL_1264382, EPI_ISL_1264384, EPI_ISL_1264394, EPI_ISL_1264401, EPI_ISL_1264406, EPI_ISL_1264411, EPI_ISL_1264415, EPI_ISL_1264436 to 1264437, EPI_ISL_1264439, EPI_ISL_1264442, EPI_ISL_1264448, EPI_ISL_1264457, EPI_ISL_1264464, EPI_ISL_1264511 to 1264512, EPI_ISL_1264535 to 1264536, EPI_ISL_1264547, EPI_ISL_1264558, EPI_ISL_1264565, EPI_ISL_1264570, EPI_ISL_1264580, EPI_ISL_1264583, EPI_ISL_1264590, EPI_ISL_1264595, EPI_ISL_1264616, EPI_ISL_1264622, EPI_ISL_1264629, EPI_ISL_1264638, EPI_ISL_1273989, EPI_ISL_1273992, EPI_ISL_1273995 to 1273996, EPI_ISL_1273999 to 1274000, EPI_ISL_1274003, EPI_ISL_1274010 to 1274011, EPI_ISL_1274018, EPI_ISL_1274024 to 1274025, EPI_ISL_1274027, EPI_ISL_1274029, EPI_ISL_1274031, EPI_ISL_1274033 to 1274034, EPI_ISL_1274039, EPI_ISL_1274043 to 1274044, EPI_ISL_1274051 to 1274053, EPI_ISL_1274055, EPI_ISL_1274059, EPI_ISL_1274061, EPI_ISL_1274063 to 1274064, EPI_ISL_1274071, EPI_ISL_1274077, EPI_ISL_1274082, EPI_ISL_1274090, EPI_ISL_1274098, EPI_ISL_1274101 to 1274104, EPI_ISL_1274106, EPI_ISL_1274108 to 1274109, EPI_ISL_1274111, EPI_ISL_1274115, EPI_ISL_1274117 to 1274121, EPI_ISL_1274129, EPI_ISL_1274132, EPI_ISL_1274137, EPI_ISL_1274139, EPI_ISL_1274142, EPI_ISL_1274148 to 1274150, EPI_ISL_1274152 to 1274154, EPI_ISL_1274156, EPI_ISL_1274160, EPI_ISL_1274164, EPI_ISL_1274168, EPI_ISL_1274174, EPI_ISL_1274178, EPI_ISL_1274180, EPI_ISL_1274182, EPI_ISL_1274189, EPI_ISL_1274193, EPI_ISL_1274196, EPI_ISL_1274205 to 1274206, EPI_ISL_1274211, EPI_ISL_1274225 to 1274227, EPI_ISL_1274229 to 1274230, EPI_ISL_1274234 to 1274236, EPI_ISL_1274241, EPI_ISL_1274244, EPI_ISL_1274248, EPI_ISL_1274255, EPI_ISL_1274261, EPI_ISL_1274263, EPI_ISL_1274267 to 1274268, EPI_ISL_1274270, EPI_ISL_1274275, EPI_ISL_1274277 to 1274278, EPI_ISL_1274282, EPI_ISL_1274284 to 1274285, EPI_ISL_1274291, EPI_ISL_1274293 to 1274295, EPI_ISL_1274299, EPI_ISL_1276133, EPI_ISL_1276152, EPI_ISL_1276166, EPI_ISL_1276169, EPI_ISL_1276180, EPI_ISL_1276191, EPI_ISL_1276197, EPI_ISL_1276215, EPI_ISL_1276220, EPI_ISL_1276238, EPI_ISL_1276256 to 1276257, EPI_ISL_1276265, EPI_ISL_1276286, EPI_ISL_1276300, EPI_ISL_1276306, EPI_ISL_1276315, EPI_ISL_1276319, EPI_ISL_1276326, EPI_ISL_1276328, EPI_ISL_1276332, EPI_ISL_1276335, EPI_ISL_1276347 to 1276348, EPI_ISL_1276351, EPI_ISL_1276369, EPI_ISL_1276492, EPI_ISL_1276657, EPI_ISL_1276663 to 1276664, EPI_ISL_1276686, EPI_ISL_1276698, EPI_ISL_1276701, EPI_ISL_1276710, EPI_ISL_1276714, EPI_ISL_1276720, EPI_ISL_1276729, EPI_ISL_1276732, EPI_ISL_1276734, EPI_ISL_1276745, EPI_ISL_1276752, EPI_ISL_1276754, EPI_ISL_1276758, EPI_ISL_1276760 to 1276761, EPI_ISL_1276786, EPI_ISL_1276796, EPI_ISL_1276798, EPI_ISL_1276800, EPI_ISL_1276802, EPI_ISL_1276805 to 1276806, EPI_ISL_1276808, EPI_ISL_1276811, EPI_ISL_1276814, EPI_ISL_1276853 to 1276854, EPI_ISL_1276861, EPI_ISL_1276864, EPI_ISL_1276869, EPI_ISL_1276875 to 1276876, EPI_ISL_1276879, EPI_ISL_1276882, EPI_ISL_1276891, EPI_ISL_1277212, EPI_ISL_1277235, EPI_ISL_1277242, EPI_ISL_1277253, EPI_ISL_1277270, EPI_ISL_1277303, EPI_ISL_1277311, EPI_ISL_1277316, EPI_ISL_1277319 to 1277320, EPI_ISL_1277325, EPI_ISL_1277328 to 1277331, EPI_ISL_1277333 to 1277334, EPI_ISL_1277336 to 1277339, EPI_ISL_1277344, EPI_ISL_1277347, EPI_ISL_1277613, EPI_ISL_1277758, EPI_ISL_1277760, EPI_ISL_1277966 to 1277992, EPI_ISL_1277994 to 1278002

see above	Lighthouse Lab in Alderley Park	Wellcome Sanger Institute for the COVID-19 Genomics UK (COG-UK) Consortium	Cordelia Langford; David K. Jackson; Dominic Kwiatkowski; Ewan Harrison; Ian Johnston; Jacquelyn Wynn; Jeffrey Barrett; John Sillitoe on behalf of the Wellcome Sanger Institute COVID-19 Surveillance Team; John Sillitoe on behalf of the Wellcome Sanger Institute COVID-19 Surveillance Team (http://www.sanger.ac.uk/covid-team); Mairead Hyland; Roberto Amato; Sonia Goncalves; The Lighthouse Lab in Alderley Park and Alex Alderton

EPI_ISL_551285 to 551293, EPI_ISL_551295 to 551326, EPI_ISL_551328 to 551411, EPI_ISL_551413 to 551452, EPI_ISL_551454 to 551483, EPI_ISL_551485 to 551495, EPI_ISL_551497 to 551529, EPI_ISL_551531 to 551576, EPI_ISL_551735 to 551736, EPI_ISL_551740, EPI_ISL_551742 to 551743, EPI_ISL_551749, EPI_ISL_551751, EPI_ISL_551755 to 551756, EPI_ISL_551759 to 551764, EPI_ISL_551769, EPI_ISL_551772, EPI_ISL_551776, EPI_ISL_551779 to 551780, EPI_ISL_551784 to 551785, EPI_ISL_551789, EPI_ISL_551795 to 551796, EPI_ISL_551798 to 551799, EPI_ISL_551801, EPI_ISL_551803 to 551804, EPI_ISL_551806 to 551808, EPI_ISL_551810 to 551812, EPI_ISL_551815, EPI_ISL_551818 to 551819, EPI_ISL_551823 to 551827, EPI_ISL_551832, EPI_ISL_551834, EPI_ISL_552446, EPI_ISL_552449, EPI_ISL_552463 to 552464, EPI_ISL_552466, EPI_ISL_552468, EPI_ISL_552473, EPI_ISL_552476, EPI_ISL_552479, EPI_ISL_552481, EPI_ISL_552486, EPI_ISL_552488, EPI_ISL_552506 to 552508, EPI_ISL_552512, EPI_ISL_552515 to 552516, EPI_ISL_552523, EPI_ISL_552526 to 552527, EPI_ISL_552530, EPI_ISL_552533 to 552534, EPI_ISL_552539 to 552540, EPI_ISL_552547, EPI_ISL_552556, EPI_ISL_552562, EPI_ISL_552565 to 552566, EPI_ISL_552572, EPI_ISL_552574, EPI_ISL_552576 to 552577, EPI_ISL_552579 to 552580, EPI_ISL_552583, EPI_ISL_552587, EPI_ISL_552590 to 552591, EPI_ISL_552595 to 552596, EPI_ISL_552598, EPI_ISL_552607 to 552608, EPI_ISL_552614, EPI_ISL_552617 to 552618, EPI_ISL_552620, EPI_ISL_552622, EPI_ISL_552629, EPI_ISL_552639, EPI_ISL_552642, EPI_ISL_552644, EPI_ISL_552646, EPI_ISL_552655, EPI_ISL_552659, EPI_ISL_552665 to 552666, EPI_ISL_552669, EPI_ISL_552671, EPI_ISL_552679, EPI_ISL_552681, EPI_ISL_552686, EPI_ISL_552689, EPI_ISL_552699, EPI_ISL_552703, EPI_ISL_552706 to 552707, EPI_ISL_552712 to 552713, EPI_ISL_552717, EPI_ISL_552719 to 552724, EPI_ISL_552726, EPI_ISL_552728 to 552732, EPI_ISL_552735, EPI_ISL_552741 to 552742, EPI_ISL_552745 to 552746, EPI_ISL_552748 to 552750, EPI_ISL_552760 to 552761, EPI_ISL_552768, EPI_ISL_552770 to 552771, EPI_ISL_552773 to 552777, EPI_ISL_552779 to 552780, EPI_ISL_552782 to 552783, EPI_ISL_552785, EPI_ISL_552792 to 552794, EPI_ISL_552796 to 552798, EPI_ISL_552800 to 552801, EPI_ISL_552803 to 552808, EPI_ISL_552812 to 552813, EPI_ISL_552815 to 552817, EPI_ISL_552819, EPI_ISL_552821 to 552822, EPI_ISL_552824 to 552840, EPI_ISL_552843 to 552851, EPI_ISL_552853 to 552866, EPI_ISL_552868 to 552873, EPI_ISL_552876 to 552878, EPI_ISL_552881 to 552904, EPI_ISL_552906 to 552909, EPI_ISL_552911 to 552915, EPI_ISL_552917 to 552935, EPI_ISL_552937, EPI_ISL_552940 to 552946, EPI_ISL_552951 to 552952, EPI_ISL_552954 to 552960, EPI_ISL_552962, EPI_ISL_552964 to 552966, EPI_ISL_552968 to 552971, EPI_ISL_552974, EPI_ISL_552976 to 552978, EPI_ISL_552980 to 552986, EPI_ISL_552988 to 552989, EPI_ISL_552991 to 552994, EPI_ISL_552996, EPI_ISL_552998 to 553006, EPI_ISL_553269, EPI_ISL_553271 to 553274, EPI_ISL_553277 to 553294, EPI_ISL_553296 to 553322, EPI_ISL_553324 to 553420, EPI_ISL_553422 to 553567, EPI_ISL_553569 to 553731, EPI_ISL_553733 to 553777, EPI_ISL_553779 to 553783, EPI_ISL_553785 to 553811, EPI_ISL_553813 to 553845, EPI_ISL_553847 to 553865, EPI_ISL_553867 to 553870, EPI_ISL_553872 to 553878, EPI_ISL_553881 to 553884, EPI_ISL_553886 to 553888, EPI_ISL_553890 to 553892, EPI_ISL_553895, EPI_ISL_553899 to 553904, EPI_ISL_553906, EPI_ISL_553908 to 553928, EPI_ISL_553930 to 553933, EPI_ISL_553935 to 553938, EPI_ISL_553940 to 553945, EPI_ISL_553947 to 553951, EPI_ISL_553953, EPI_ISL_553958 to 553964, EPI_ISL_553966 to 553971, EPI_ISL_553973 to 553982, EPI_ISL_553984, EPI_ISL_553986, EPI_ISL_553988, EPI_ISL_553990 to 553992, EPI_ISL_553995 to 554002, EPI_ISL_554004, EPI_ISL_554006 to 554008, EPI_ISL_554010 to 554014, EPI_ISL_554016 to 554023, EPI_ISL_554027, EPI_ISL_554029 to 554041, EPI_ISL_554043 to 554047, EPI_ISL_554049 to 554053, EPI_ISL_554055 to 554056, EPI_ISL_554059 to 554064, EPI_ISL_554066 to 554079, EPI_ISL_554081 to 554096, EPI_ISL_554098, EPI_ISL_554102 to 554117, EPI_ISL_554120, EPI_ISL_554122, EPI_ISL_554126, EPI_ISL_554129 to 554131, EPI_ISL_554136,

EPI_ISL_554140, EPI_ISL_554149, EPI_ISL_554151, EPI_ISL_554155 to 554158, EPI_ISL_554164 to 554165, EPI_ISL_554170 to 554172, EPI_ISL_554174 to 554176, EPI_ISL_554183, EPI_ISL_554186 to 554190, EPI_ISL_554192 to 554194, EPI_ISL_554196 to 554199, EPI_ISL_554202 to 554203, EPI_ISL_554206 to 554207,
EPI_ISL_554209, EPI_ISL_554212, EPI_ISL_554215, EPI_ISL_554219, EPI_ISL_554222, EPI_ISL_554225, EPI_ISL_554227, EPI_ISL_554229 to 554230, EPI_ISL_554233, EPI_ISL_554235, EPI_ISL_554238, EPI_ISL_554241, EPI_ISL_554250, EPI_ISL_554252, EPI_ISL_554257, EPI_ISL_554260, EPI_ISL_554263,
EPI_ISL_554265 to 554266, EPI_ISL_554268, EPI_ISL_554270, EPI_ISL_554275, EPI_ISL_554278 to 554280, EPI_ISL_554282 to 554283, EPI_ISL_554288, EPI_ISL_554292, EPI_ISL_554296, EPI_ISL_554298 to 554299, EPI_ISL_554301, EPI_ISL_554305, EPI_ISL_554309, EPI_ISL_554318, EPI_ISL_554320 to 554477,
EPI_ISL_554479 to 554483, EPI_ISL_554485 to 554496, EPI_ISL_554498 to 554536, EPI_ISL_554538 to 554600, EPI_ISL_554602 to 554618, EPI_ISL_554620, EPI_ISL_554623 to 554626, EPI_ISL_554630 to 554632, EPI_ISL_554640, EPI_ISL_554642, EPI_ISL_554646, EPI_ISL_554651, EPI_ISL_554653 to 554654,
EPI_ISL_554656 to 554657, EPI_ISL_554661, EPI_ISL_554663 to 554664, EPI_ISL_554666, EPI_ISL_554677, EPI_ISL_554680 to 554681, EPI_ISL_554686, EPI_ISL_554688 to 554689, EPI_ISL_554692, EPI_ISL_554694, EPI_ISL_554696 to 554697, EPI_ISL_554703, EPI_ISL_554708, EPI_ISL_554710, EPI_ISL_554716,
EPI_ISL_554725 to 554727, EPI_ISL_554730, EPI_ISL_554732, EPI_ISL_554735, EPI_ISL_554741, EPI_ISL_554743, EPI_ISL_554754 to 554757, EPI_ISL_554759, EPI_ISL_554761, EPI_ISL_554767, EPI_ISL_554771 to 554772, EPI_ISL_554776 to 554780, EPI_ISL_554782, EPI_ISL_554792, EPI_ISL_554794 to 554795,
EPI_ISL_554797, EPI_ISL_554799, EPI_ISL_554802, EPI_ISL_554804, EPI_ISL_554807 to 554813, EPI_ISL_554815 to 554816, EPI_ISL_554820, EPI_ISL_554827, EPI_ISL_554830, EPI_ISL_554832 to 554834, EPI_ISL_554836 to 554837, EPI_ISL_554841, EPI_ISL_554846 to 554848, EPI_ISL_554850, EPI_ISL_554853,
EPI_ISL_554856, EPI_ISL_554861 to 554867, EPI_ISL_554869 to 554928, EPI_ISL_554930 to 555018, EPI_ISL_555020 to 555040, EPI_ISL_555042 to 555126, EPI_ISL_555128, EPI_ISL_555130 to 555147, EPI_ISL_555149 to 555258, EPI_ISL_555260 to 555302, EPI_ISL_555305 to 555390, EPI_ISL_555392 to 555397,
EPI_ISL_555399 to 555431, EPI_ISL_555434 to 555501, EPI_ISL_555503 to 555509, EPI_ISL_555512 to 555557, EPI_ISL_555559 to 555615, EPI_ISL_555617 to 555649, EPI_ISL_555652 to 555659, EPI_ISL_555661 to 555793, EPI_ISL_555795 to 555806, EPI_ISL_555808 to 555814, EPI_ISL_555816 to 555842,
EPI_ISL_555845 to 555863, EPI_ISL_555865 to 555867, EPI_ISL_555869 to 555874, EPI_ISL_555876 to 555886, EPI_ISL_555888 to 555909, EPI_ISL_555911 to 555938, EPI_ISL_555940 to 555944, EPI_ISL_555946, EPI_ISL_555949 to 555954, EPI_ISL_555956 to 555972, EPI_ISL_555974 to 555975, EPI_ISL_555977 to
555985, EPI_ISL_555987 to 555989, EPI_ISL_555991 to 555992, EPI_ISL_555994 to 555999, EPI_ISL_556001 to 556011, EPI_ISL_556013 to 556020, EPI_ISL_556023 to 556025, EPI_ISL_556027 to 556038, EPI_ISL_556040, EPI_ISL_556042 to 556046, EPI_ISL_556048 to 556057, EPI_ISL_556059 to 556062, EPI_ISL_556064
to 556065, EPI_ISL_556067 to 556068, EPI_ISL_556070 to 556074, EPI_ISL_556078, EPI_ISL_556080 to 556082, EPI_ISL_556084 to 556086, EPI_ISL_556088 to 556089, EPI_ISL_556096, EPI_ISL_556099 to 556101, EPI_ISL_556103, EPI_ISL_556105 to 556108, EPI_ISL_556110 to 556111, EPI_ISL_556113 to 556114,
EPI_ISL_556117 to 556122, EPI_ISL_556124 to 556125, EPI_ISL_556127 to 556132, EPI_ISL_556135 to 556137, EPI_ISL_556140, EPI_ISL_556142 to 556145, EPI_ISL_556147, EPI_ISL_556149, EPI_ISL_556151 to 556157, EPI_ISL_556159 to 556162, EPI_ISL_556167 to 556169, EPI_ISL_556171, EPI_ISL_556173 to 556175,
EPI_ISL_556177 to 556182, EPI_ISL_556184 to 556185, EPI_ISL_556188 to 556194, EPI_ISL_556196, EPI_ISL_556200, EPI_ISL_556202 to 556203, EPI_ISL_556205 to 556206, EPI_ISL_556209 to 556210, EPI_ISL_556212, EPI_ISL_556215 to 556217, EPI_ISL_556219 to 556222, EPI_ISL_556224 to 556231, EPI_ISL_556234,
EPI_ISL_556236, EPI_ISL_556238 to 556241, EPI_ISL_556243, EPI_ISL_556245 to 556247, EPI_ISL_556249 to 556250, EPI_ISL_556252, EPI_ISL_556254 to 556258, EPI_ISL_556260, EPI_ISL_556262, EPI_ISL_556264 to 556265, EPI_ISL_556268, EPI_ISL_556270 to 556274, EPI_ISL_556277 to 556281, EPI_ISL_556283 to
556284, EPI_ISL_556286, EPI_ISL_556288, EPI_ISL_556290, EPI_ISL_556292, EPI_ISL_556295 to 556297, EPI_ISL_556299, EPI_ISL_556301 to 556303, EPI_ISL_556306 to 556308, EPI_ISL_556310 to 556316, EPI_ISL_556319 to 556320, EPI_ISL_556322 to 556323, EPI_ISL_556325 to 556337, EPI_ISL_556339 to 556343,
EPI_ISL_556345 to 556347, EPI_ISL_556352 to 556356, EPI_ISL_556358 to 556410, EPI_ISL_556412 to 556424, EPI_ISL_556426 to 556427, EPI_ISL_556429 to 556432, EPI_ISL_556436 to 556445, EPI_ISL_556447 to 556458, EPI_ISL_556460 to 556470, EPI_ISL_556472 to 556481, EPI_ISL_556483 to 556487,
EPI_ISL_556489 to 556493, EPI_ISL_556495 to 556499, EPI_ISL_556501 to 556504, EPI_ISL_556506 to 556507, EPI_ISL_556511 to 556515, EPI_ISL_556576 to 556638, EPI_ISL_556985 to 557001, EPI_ISL_557003 to 557042, EPI_ISL_557044 to 557109, EPI_ISL_557111 to 557141, EPI_ISL_557143 to 557165,
EPI_ISL_557351, EPI_ISL_557354, EPI_ISL_557356, EPI_ISL_557359, EPI_ISL_557361, EPI_ISL_557363, EPI_ISL_557366, EPI_ISL_557371, EPI_ISL_557373 to 557374, EPI_ISL_557378, EPI_ISL_557380 to 557381, EPI_ISL_557383, EPI_ISL_557388, EPI_ISL_557390, EPI_ISL_557398 to 557399, EPI_ISL_557404 to
557406, EPI_ISL_557408, EPI_ISL_557411 to 557412, EPI_ISL_557414 to 557415, EPI_ISL_557417, EPI_ISL_557419, EPI_ISL_557423, EPI_ISL_557425, EPI_ISL_557428, EPI_ISL_557430, EPI_ISL_557433 to 557434, EPI_ISL_557436, EPI_ISL_557439 to 557440, EPI_ISL_557446 to 557447, EPI_ISL_557449,
EPI_ISL_557452 to 557454, EPI_ISL_557466, EPI_ISL_557468 to 557469, EPI_ISL_557471, EPI_ISL_557474, EPI_ISL_557476, EPI_ISL_557481 to 557482, EPI_ISL_557484 to 557485, EPI_ISL_557487 to 557488, EPI_ISL_557491, EPI_ISL_557493, EPI_ISL_557497, EPI_ISL_557499 to 557500, EPI_ISL_557507,
EPI_ISL_557511, EPI_ISL_557518, EPI_ISL_557522 to 557523, EPI_ISL_557526 to 557528, EPI_ISL_557530 to 557531, EPI_ISL_557533, EPI_ISL_557537, EPI_ISL_557539, EPI_ISL_557541, EPI_ISL_557543 to 557544, EPI_ISL_557551, EPI_ISL_557553 to 557624, EPI_ISL_557626 to 557700, EPI_ISL_557702 to 557710,
EPI_ISL_557712 to 557727, EPI_ISL_557729, EPI_ISL_557731 to 557738, EPI_ISL_557743 to 557744, EPI_ISL_557746 to 557747, EPI_ISL_557749, EPI_ISL_557751 to 557752, EPI_ISL_557754 to 557756, EPI_ISL_557759, EPI_ISL_557761 to 557763, EPI_ISL_557767, EPI_ISL_557769 to 557770, EPI_ISL_557772 to 557774,
EPI_ISL_557776 to 557777, EPI_ISL_557779 to 557780, EPI_ISL_557783, EPI_ISL_557785, EPI_ISL_557787 to 557788, EPI_ISL_557790, EPI_ISL_557792, EPI_ISL_557794, EPI_ISL_557797 to 557799, EPI_ISL_557801, EPI_ISL_557803 to 557806, EPI_ISL_557809 to 557810, EPI_ISL_557813 to 557815, EPI_ISL_557818 to
557820, EPI_ISL_557825 to 557826, EPI_ISL_557831, EPI_ISL_557833 to 557837, EPI_ISL_557839, EPI_ISL_557841 to 557842, EPI_ISL_557844, EPI_ISL_557846, EPI_ISL_557850 to 557851, EPI_ISL_557855 to 557857, EPI_ISL_557859 to 557864, EPI_ISL_557871 to 557875, EPI_ISL_557881 to 557883, EPI_ISL_557886 to
557888, EPI_ISL_557891 to 557892, EPI_ISL_557897, EPI_ISL_557899 to 557904, EPI_ISL_557906, EPI_ISL_557910, EPI_ISL_557912 to 557914, EPI_ISL_557916, EPI_ISL_557918, EPI_ISL_557920, EPI_ISL_557922 to 557924, EPI_ISL_557928 to 557932, EPI_ISL_557934 to 557936, EPI_ISL_557939, EPI_ISL_557943,
EPI_ISL_557945 to 557946, EPI_ISL_557950 to 557951, EPI_ISL_557955, EPI_ISL_557957, EPI_ISL_557960 to 557962, EPI_ISL_557966, EPI_ISL_557968 to 557969, EPI_ISL_557971, EPI_ISL_557973, EPI_ISL_557975, EPI_ISL_557978, EPI_ISL_557980 to 557983, EPI_ISL_557985 to 557988, EPI_ISL_557990 to 557998,
EPI_ISL_558000, EPI_ISL_558002 to 558020, EPI_ISL_558022 to 558023, EPI_ISL_558025, EPI_ISL_558027, EPI_ISL_558029, EPI_ISL_558031 to 558036, EPI_ISL_558038 to 558040, EPI_ISL_558042 to 558057, EPI_ISL_558059 to 558067, EPI_ISL_558069 to 558088, EPI_ISL_558090 to 558095, EPI_ISL_558097 to 558102,
EPI_ISL_558104 to 558105, EPI_ISL_558107 to 558125, EPI_ISL_558128 to 558154, EPI_ISL_558156 to 558184, EPI_ISL_558186 to 558194, EPI_ISL_558196 to 558221, EPI_ISL_558223 to 558233, EPI_ISL_558235 to 558236, EPI_ISL_558238, EPI_ISL_558240, EPI_ISL_558244, EPI_ISL_558256, EPI_ISL_558259 to 558260,
EPI_ISL_558263, EPI_ISL_558268 to 558270, EPI_ISL_558274, EPI_ISL_558276 to 558277, EPI_ISL_558288 to 558289, EPI_ISL_558291, EPI_ISL_558307, EPI_ISL_558312, EPI_ISL_558316, EPI_ISL_558321, EPI_ISL_558324, EPI_ISL_558329, EPI_ISL_558332, EPI_ISL_558335, EPI_ISL_558338, EPI_ISL_558343,
EPI_ISL_558345, EPI_ISL_558348, EPI_ISL_558350, EPI_ISL_558354 to 558355, EPI_ISL_558364, EPI_ISL_558368, EPI_ISL_558379, EPI_ISL_558389, EPI_ISL_558394 to 558396, EPI_ISL_558404 to 558421, EPI_ISL_558423 to 558431, EPI_ISL_558433 to 558542, EPI_ISL_558544 to 558604, EPI_ISL_558606 to 558616,
EPI_ISL_558618 to 558637, EPI_ISL_558639 to 558730, EPI_ISL_558732 to 558795, EPI_ISL_558797 to 558845, EPI_ISL_558847 to 558871, EPI_ISL_558873 to 558876, EPI_ISL_558878 to 558887, EPI_ISL_558889 to 558902, EPI_ISL_558904 to 558913, EPI_ISL_558915 to 558919, EPI_ISL_558922, EPI_ISL_558924,
EPI_ISL_558927, EPI_ISL_558929 to 558931, EPI_ISL_558933, EPI_ISL_558935 to 558936, EPI_ISL_558938 to 558948, EPI_ISL_558952 to 558958, EPI_ISL_558960 to 558964, EPI_ISL_558966 to 558969, EPI_ISL_558971 to 558972, EPI_ISL_558974, EPI_ISL_558976 to 558979, EPI_ISL_558982 to 558986, EPI_ISL_558988
to 558992, EPI_ISL_558995 to 558999, EPI_ISL_559001 to 559004, EPI_ISL_559006 to 559008, EPI_ISL_559010, EPI_ISL_559012 to 559016, EPI_ISL_559018, EPI_ISL_559020 to 559021, EPI_ISL_559023 to 559025, EPI_ISL_559027, EPI_ISL_559029 to 559041, EPI_ISL_559044 to 559045, EPI_ISL_559048, EPI_ISL_559050
to 559056, EPI_ISL_559058 to 559060, EPI_ISL_559062, EPI_ISL_559064 to 559070, EPI_ISL_559072 to 559079, EPI_ISL_559082, EPI_ISL_559085 to 559087, EPI_ISL_559089 to 559099, EPI_ISL_559101 to 559103, EPI_ISL_559105, EPI_ISL_559107 to 559116, EPI_ISL_559118, EPI_ISL_559121 to 559124, EPI_ISL_559127
to 559128, EPI_ISL_559130, EPI_ISL_559134 to 559135, EPI_ISL_559137 to 559146, EPI_ISL_559148, EPI_ISL_559150 to 559153, EPI_ISL_559155 to 559156, EPI_ISL_559159 to 559162, EPI_ISL_559165, EPI_ISL_559168 to 559170, EPI_ISL_559172 to 559173, EPI_ISL_559175 to 559180, EPI_ISL_559182 to 559186,
EPI_ISL_559188 to 559193, EPI_ISL_559196 to 559200, EPI_ISL_559202 to 559203, EPI_ISL_559205 to 559206, EPI_ISL_559208 to 559212, EPI_ISL_559214, EPI_ISL_559216 to 559217, EPI_ISL_559219 to 559222, EPI_ISL_559224 to 559227, EPI_ISL_559229 to 559231, EPI_ISL_559233 to 559234, EPI_ISL_559237 to
559240, EPI_ISL_559242 to 559243, EPI_ISL_559245, EPI_ISL_559248 to 559252, EPI_ISL_559255, EPI_ISL_559257 to 559258, EPI_ISL_559260, EPI_ISL_559262, EPI_ISL_559265 to 559274, EPI_ISL_559276, EPI_ISL_559279 to 559282, EPI_ISL_559285 to 559286, EPI_ISL_559288 to 559289, EPI_ISL_559291 to 559295,
EPI_ISL_559297 to 559299, EPI_ISL_559301 to 559311, EPI_ISL_559313, EPI_ISL_559315 to 559318, EPI_ISL_559321 to 559325, EPI_ISL_559327 to 559330, EPI_ISL_559332 to 559334, EPI_ISL_559336, EPI_ISL_559338 to 559351, EPI_ISL_559356 to 559367, EPI_ISL_559369 to 559446, EPI_ISL_559448 to 559575,
EPI_ISL_559577 to 559590, EPI_ISL_559592 to 559615, EPI_ISL_559617 to 559643, EPI_ISL_559645 to 559661, EPI_ISL_566110 to 566420, EPI_ISL_567533 to 567802, EPI_ISL_567804 to 567810, EPI_ISL_567812 to 568222, EPI_ISL_568467 to 568468, EPI_ISL_568474 to 568475, EPI_ISL_568477, EPI_ISL_575399 to
575535, EPI_ISL_575537 to 575760, EPI_ISL_575982 to 575983, EPI_ISL_579919 to 579950, EPI_ISL_579952 to 579970, EPI_ISL_579972 to 580061, EPI_ISL_580063 to 580137, EPI_ISL_580139 to 580186, EPI_ISL_580188 to 580209, EPI_ISL_580211 to 580281, EPI_ISL_580284, EPI_ISL_580288 to 580298, EPI_ISL_580302
to 580304, EPI_ISL_580306 to 580312, EPI_ISL_580314 to 580321, EPI_ISL_580323 to 580324, EPI_ISL_580326, EPI_ISL_580328 to 580331, EPI_ISL_580334 to 580337, EPI_ISL_580339, EPI_ISL_580342 to 580352, EPI_ISL_580355 to 580361, EPI_ISL_580363, EPI_ISL_580365 to 580366, EPI_ISL_580370, EPI_ISL_580372,
EPI_ISL_580374 to 580384, EPI_ISL_580386 to 580390, EPI_ISL_580392, EPI_ISL_580395, EPI_ISL_580397 to 580399, EPI_ISL_580401 to 580413, EPI_ISL_580415 to 580417, EPI_ISL_580419 to 580420, EPI_ISL_580422 to 580434, EPI_ISL_580436 to 580443, EPI_ISL_580445 to 580448, EPI_ISL_580450 to 580452,
EPI_ISL_580454 to 580458, EPI_ISL_580460 to 580464, EPI_ISL_580466, EPI_ISL_580468 to 580470, EPI_ISL_580472 to 580477, EPI_ISL_580482 to 580484, EPI_ISL_580487 to 580494, EPI_ISL_580496 to 580498, EPI_ISL_580500 to 580501, EPI_ISL_580503 to 580507, EPI_ISL_580510, EPI_ISL_580512 to 580514,
EPI_ISL_580516 to 580531, EPI_ISL_580534 to 580535, EPI_ISL_580537 to 580549, EPI_ISL_580551 to 580556, EPI_ISL_580558, EPI_ISL_580560 to 580562, EPI_ISL_580566, EPI_ISL_580568, EPI_ISL_580570 to 580572, EPI_ISL_580574 to 580575, EPI_ISL_580578, EPI_ISL_580580 to 580581, EPI_ISL_580583 to 580585,
EPI_ISL_580588 to 580592, EPI_ISL_580595 to 580602, EPI_ISL_580604 to 580606, EPI_ISL_580608 to 580609, EPI_ISL_580611 to 580617, EPI_ISL_580619 to 580620, EPI_ISL_580622 to 580624, EPI_ISL_580627 to 580630, EPI_ISL_580633, EPI_ISL_580635 to 580636, EPI_ISL_580640, EPI_ISL_580645, EPI_ISL_580648,
EPI_ISL_580650 to 580651, EPI_ISL_580655, EPI_ISL_580660, EPI_ISL_580663, EPI_ISL_580674 to 580675, EPI_ISL_580677 to 580682, EPI_ISL_580689 to 580692, EPI_ISL_580695 to 580697, EPI_ISL_580699, EPI_ISL_580706, EPI_ISL_580710, EPI_ISL_580720, EPI_ISL_580722 to 580723, EPI_ISL_580730,
EPI_ISL_580734, EPI_ISL_580737 to 580738, EPI_ISL_580740, EPI_ISL_580754, EPI_ISL_580763 to 580764, EPI_ISL_580769, EPI_ISL_580771, EPI_ISL_580784, EPI_ISL_580787 to 580789, EPI_ISL_580791, EPI_ISL_580821 to 580823, EPI_ISL_580825, EPI_ISL_580828, EPI_ISL_580833, EPI_ISL_580835,
EPI_ISL_580849, EPI_ISL_580851, EPI_ISL_580853, EPI_ISL_581367, EPI_ISL_581378 to 581380, EPI_ISL_581391, EPI_ISL_581404, EPI_ISL_581409, EPI_ISL_581411 to 581414, EPI_ISL_581416 to 581417, EPI_ISL_581424 to 581427, EPI_ISL_581429 to 581433, EPI_ISL_581435 to 581436, EPI_ISL_581442,
EPI_ISL_581444 to 581446, EPI_ISL_581448, EPI_ISL_586572 to 586611, EPI_ISL_586613 to 586616, EPI_ISL_586618 to 586620, EPI_ISL_586622 to 586708, EPI_ISL_586710 to 586906, EPI_ISL_586908 to 587084, EPI_ISL_587086 to 587264, EPI_ISL_587266 to 587302, EPI_ISL_587711 to 587976, EPI_ISL_587978 to
588006, EPI_ISL_588008 to 588025, EPI_ISL_588027 to 588059, EPI_ISL_589267 to 589288, EPI_ISL_589290 to 589296, EPI_ISL_589298 to 589301, EPI_ISL_589303, EPI_ISL_589305 to 589328, EPI_ISL_589330 to 589333, EPI_ISL_589336 to 589341, EPI_ISL_589343, EPI_ISL_589345 to 589354, EPI_ISL_589356 to
589375, EPI_ISL_589378 to 589406, EPI_ISL_589408 to 589416, EPI_ISL_589418 to 589422, EPI_ISL_589424 to 589429, EPI_ISL_589431 to 589439, EPI_ISL_589441 to 589492, EPI_ISL_589494 to 589518, EPI_ISL_589522 to 589525, EPI_ISL_589527 to 589566, EPI_ISL_589568 to 589571, EPI_ISL_589762,
EPI_ISL_589765, EPI_ISL_589778 to 589786, EPI_ISL_589802, EPI_ISL_590181, EPI_ISL_590186, EPI_ISL_590193 to 590194, EPI_ISL_590198, EPI_ISL_590200, EPI_ISL_590206, EPI_ISL_590210, EPI_ISL_590213 to 590215, EPI_ISL_590220, EPI_ISL_590224 to 590226, EPI_ISL_590228, EPI_ISL_590234 to 590235,
EPI_ISL_590239, EPI_ISL_590246, EPI_ISL_590250, EPI_ISL_590255, EPI_ISL_590257, EPI_ISL_590259 to 590261, EPI_ISL_590263, EPI_ISL_590265, EPI_ISL_590272, EPI_ISL_590274 to 590277, EPI_ISL_590282 to 590284, EPI_ISL_590286, EPI_ISL_590288, EPI_ISL_590295 to 590296, EPI_ISL_590300,
EPI_ISL_590306 to 590307, EPI_ISL_590309, EPI_ISL_590314, EPI_ISL_590319, EPI_ISL_590321, EPI_ISL_590327 to 590329, EPI_ISL_590335, EPI_ISL_590337, EPI_ISL_590346 to 590347, EPI_ISL_590351 to 590352, EPI_ISL_590355, EPI_ISL_590357, EPI_ISL_590359, EPI_ISL_590361, EPI_ISL_590364,
EPI_ISL_590366 to 590367, EPI_ISL_590369, EPI_ISL_590373, EPI_ISL_590379 to 590380, EPI_ISL_590383, EPI_ISL_590386, EPI_ISL_590388, EPI_ISL_590390, EPI_ISL_590392, EPI_ISL_590397 to 590399, EPI_ISL_590403, EPI_ISL_590405, EPI_ISL_590419, EPI_ISL_590422 to 590423, EPI_ISL_590438,
EPI_ISL_590441 to 590442, EPI_ISL_590446, EPI_ISL_590448, EPI_ISL_590452 to 590453, EPI_ISL_590462, EPI_ISL_597523 to 597632, EPI_ISL_597634 to 597983, EPI_ISL_597985 to 598162, EPI_ISL_598164 to 598248, EPI_ISL_598824 to 598830, EPI_ISL_600521, EPI_ISL_600523, EPI_ISL_600525 to 600526,
EPI_ISL_600528 to 600529, EPI_ISL_600531 to 600532, EPI_ISL_600534, EPI_ISL_600536 to 600537, EPI_ISL_602155, EPI_ISL_602158 to 602161, EPI_ISL_605932 to 606295, EPI_ISL_606924 to 606947, EPI_ISL_606949 to 606959, EPI_ISL_606961 to 607188, EPI_ISL_607190 to 607199, EPI_ISL_607201 to 607290,
EPI_ISL_607623 to 607893, EPI_ISL_607895 to 608146, EPI_ISL_608148 to 608215, EPI_ISL_608217 to 608309, EPI_ISL_608311 to 608346, EPI_ISL_608350 to 608352, EPI_ISL_608355 to 608356, EPI_ISL_608358, EPI_ISL_608361 to 608362, EPI_ISL_608364 to 608367, EPI_ISL_608369, EPI_ISL_608371 to 608372,
EPI_ISL_608374 to 608380, EPI_ISL_608383 to 608387, EPI_ISL_608390, EPI_ISL_608399, EPI_ISL_608401, EPI_ISL_608403, EPI_ISL_608405 to 608406, EPI_ISL_608409, EPI_ISL_608413 to 608415, EPI_ISL_608418 to 608422, EPI_ISL_608425, EPI_ISL_608431, EPI_ISL_608434 to 608435, EPI_ISL_608438,
EPI_ISL_608440 to 608441, EPI_ISL_608443 to 608445, EPI_ISL_608448, EPI_ISL_608452 to 608454, EPI_ISL_608457, EPI_ISL_608460 to 608462, EPI_ISL_608465, EPI_ISL_608468 to 608470, EPI_ISL_608473 to 608474, EPI_ISL_608479, EPI_ISL_608481, EPI_ISL_608483, EPI_ISL_608487 to 608489, EPI_ISL_608491,
EPI_ISL_608496, EPI_ISL_608499 to 608500, EPI_ISL_608502 to 608503, EPI_ISL_608505, EPI_ISL_608511 to 608513, EPI_ISL_608516 to 608520, EPI_ISL_608526, EPI_ISL_608528, EPI_ISL_608533 to 608534, EPI_ISL_608539, EPI_ISL_608541 to 608543, EPI_ISL_608548 to 608549, EPI_ISL_608551, EPI_ISL_608553 to
608556, EPI_ISL_608562 to 608564, EPI_ISL_608567, EPI_ISL_608569, EPI_ISL_608574 to 608575, EPI_ISL_608579 to 608584, EPI_ISL_608586 to 608587, EPI_ISL_608589 to 608595, EPI_ISL_608599 to 608601, EPI_ISL_608603, EPI_ISL_608605 to 608606, EPI_ISL_608609 to 608610, EPI_ISL_608614, EPI_ISL_608617,
EPI_ISL_608621 to 608623, EPI_ISL_608625, EPI_ISL_608630, EPI_ISL_608634 to 608635, EPI_ISL_608639 to 608640, EPI_ISL_608646 to 608649, EPI_ISL_608652, EPI_ISL_608655, EPI_ISL_608657 to 608659, EPI_ISL_608661 to 608663, EPI_ISL_608665, EPI_ISL_608667 to 608670, EPI_ISL_608672, EPI_ISL_608674 to
608677, EPI_ISL_608680, EPI_ISL_608684 to 608687, EPI_ISL_608690 to 608692, EPI_ISL_608694, EPI_ISL_608847, EPI_ISL_608849, EPI_ISL_608851, EPI_ISL_608853, EPI_ISL_608855 to 608856, EPI_ISL_608858, EPI_ISL_608860, EPI_ISL_608862 to 608866, EPI_ISL_608869, EPI_ISL_608872, EPI_ISL_608874,
EPI_ISL_608877 to 608878, EPI_ISL_608880, EPI_ISL_608883 to 608891, EPI_ISL_608895 to 608900, EPI_ISL_608903 to 608904, EPI_ISL_608909, EPI_ISL_608911, EPI_ISL_608913 to 608917, EPI_ISL_608919 to 608922, EPI_ISL_608925 to 608926, EPI_ISL_608928 to 608934, EPI_ISL_608938, EPI_ISL_608941 to 608942,
EPI_ISL_608944 to 608946, EPI_ISL_608948, EPI_ISL_608950, EPI_ISL_608952, EPI_ISL_608955 to 608957, EPI_ISL_608961 to 608964, EPI_ISL_608966, EPI_ISL_608970 to 608973, EPI_ISL_608976, EPI_ISL_608979 to 608981, EPI_ISL_608983 to 608985, EPI_ISL_608988 to 608989, EPI_ISL_608991 to 608995,
EPI_ISL_608998 to 609000, EPI_ISL_609002, EPI_ISL_609004, EPI_ISL_609007, EPI_ISL_609009 to 609013, EPI_ISL_609016, EPI_ISL_609019 to 609020, EPI_ISL_609022, EPI_ISL_609028, EPI_ISL_609031 to 609032, EPI_ISL_609034 to 609035, EPI_ISL_609039 to 609041, EPI_ISL_609043, EPI_ISL_609047,
EPI_ISL_609051 to 609053, EPI_ISL_609055 to 609057, EPI_ISL_609060 to 609061, EPI_ISL_609064 to 609065, EPI_ISL_609068 to 609070, EPI_ISL_609073 to 609075, EPI_ISL_609078 to 609080, EPI_ISL_609083, EPI_ISL_609085, EPI_ISL_609087, EPI_ISL_609089 to 609090, EPI_ISL_609092 to 609096, EPI_ISL_609098,
EPI_ISL_609102 to 609107, EPI_ISL_609109, EPI_ISL_609112, EPI_ISL_609120, EPI_ISL_609124 to 609125, EPI_ISL_609127, EPI_ISL_609130, EPI_ISL_609132 to 609135, EPI_ISL_609141, EPI_ISL_609144, EPI_ISL_609146 to 609148, EPI_ISL_609150, EPI_ISL_609152 to 609154, EPI_ISL_609157, EPI_ISL_609207 to
609208, EPI_ISL_609210 to 609218, EPI_ISL_610286 to 610331, EPI_ISL_610333 to 610501, EPI_ISL_610504 to 610628, EPI_ISL_610922 to 611092, EPI_ISL_611094 to 611286, EPI_ISL_623858 to 623862, EPI_ISL_623866 to 623867, EPI_ISL_623869 to 623871, EPI_ISL_623875, EPI_ISL_623877 to 623882,
EPI_ISL_623885, EPI_ISL_623887 to 623892, EPI_ISL_623894, EPI_ISL_623896 to 623902, EPI_ISL_623905 to 623906, EPI_ISL_623909 to 623910, EPI_ISL_623912 to 623915, EPI_ISL_623918 to 623920, EPI_ISL_623922 to 623923, EPI_ISL_623925 to 623929, EPI_ISL_623931 to 623932, EPI_ISL_623935 to 623941,
EPI_ISL_623943, EPI_ISL_623945 to 623948, EPI_ISL_623950 to 623953, EPI_ISL_623955, EPI_ISL_623957 to 623958, EPI_ISL_623960 to 623962, EPI_ISL_623964 to 623973, EPI_ISL_623978 to 623982, EPI_ISL_623984 to 623985, EPI_ISL_623987 to 623989, EPI_ISL_623992 to 623993, EPI_ISL_623996 to 624009,
EPI_ISL_624011, EPI_ISL_624013 to 624017, EPI_ISL_624020 to 624021, EPI_ISL_624023 to 624026, EPI_ISL_624030 to 624031, EPI_ISL_624033, EPI_ISL_624035 to 624042, EPI_ISL_624045 to 624048, EPI_ISL_624050 to 624066, EPI_ISL_624068 to 624072, EPI_ISL_624074 to 624076, EPI_ISL_624078, EPI_ISL_624080
to 624091, EPI_ISL_624093, EPI_ISL_624095 to 624104, EPI_ISL_624106 to 624111, EPI_ISL_624113, EPI_ISL_624115 to 624119, EPI_ISL_624121, EPI_ISL_624123 to 624127, EPI_ISL_624129 to 624131, EPI_ISL_624133 to 624139, EPI_ISL_624141 to 624144, EPI_ISL_624146, EPI_ISL_624149 to 624157, EPI_ISL_624159
to 624162, EPI_ISL_624166 to 624175, EPI_ISL_624177 to 624180, EPI_ISL_624182 to 624187, EPI_ISL_624190 to 624196, EPI_ISL_624198 to 624243, EPI_ISL_624245 to 624290, EPI_ISL_624292 to 624349, EPI_ISL_624351 to 624353, EPI_ISL_624355 to 624908, EPI_ISL_624910 to 625212, EPI_ISL_625214 to 625239,
EPI_ISL_625241 to 625280, EPI_ISL_625396 to 625404, EPI_ISL_625422 to 625423, EPI_ISL_625425, EPI_ISL_625427, EPI_ISL_625429, EPI_ISL_625431, EPI_ISL_625433 to 625436, EPI_ISL_630348 to 630473, EPI_ISL_630475 to 630532, EPI_ISL_630534 to 630546, EPI_ISL_630548 to 630701, EPI_ISL_630706 to 630711,
EPI_ISL_630713 to 630717, EPI_ISL_630720 to 630724, EPI_ISL_630726 to 630730, EPI_ISL_630732, EPI_ISL_630734 to 630740, EPI_ISL_630742 to 630744, EPI_ISL_630746 to 630748, EPI_ISL_630750 to 630760, EPI_ISL_630762 to 630763, EPI_ISL_630765 to 630774, EPI_ISL_630776 to 630777, EPI_ISL_630779,
EPI_ISL_630781, EPI_ISL_630783 to 630786, EPI_ISL_630788 to 630803, EPI_ISL_630805 to 630812, EPI_ISL_630814, EPI_ISL_630816 to 630823, EPI_ISL_630825 to 630826, EPI_ISL_630828 to 630832, EPI_ISL_630834 to 630837, EPI_ISL_630839 to 630849, EPI_ISL_630852, EPI_ISL_630854 to 630858, EPI_ISL_630860
to 630861, EPI_ISL_630863, EPI_ISL_630865 to 630869, EPI_ISL_630874, EPI_ISL_630877 to 630882, EPI_ISL_630884 to 630887, EPI_ISL_630889 to 630890, EPI_ISL_630892 to 630899, EPI_ISL_630901 to 630914, EPI_ISL_630916 to 630918, EPI_ISL_630920 to 630922, EPI_ISL_630924 to 630925, EPI_ISL_630927 to
630930, EPI_ISL_630932, EPI_ISL_630934 to 630940, EPI_ISL_630942 to 630947, EPI_ISL_630950, EPI_ISL_630952 to 630954, EPI_ISL_630956, EPI_ISL_630958, EPI_ISL_630960 to 630964, EPI_ISL_630966 to 630971, EPI_ISL_630975 to 630976, EPI_ISL_630982 to 630983, EPI_ISL_630985, EPI_ISL_630992,
EPI_ISL_630994, EPI_ISL_630999, EPI_ISL_631011 to 631012, EPI_ISL_631014, EPI_ISL_631018, EPI_ISL_631023, EPI_ISL_631027, EPI_ISL_631031, EPI_ISL_631033, EPI_ISL_631035, EPI_ISL_631041, EPI_ISL_631044, EPI_ISL_631046, EPI_ISL_631050, EPI_ISL_631056 to 631057, EPI_ISL_631059 to 631060,
EPI_ISL_631074, EPI_ISL_631081, EPI_ISL_631084, EPI_ISL_631089, EPI_ISL_631093, EPI_ISL_631096, EPI_ISL_631099, EPI_ISL_631107, EPI_ISL_631111, EPI_ISL_631113, EPI_ISL_631116, EPI_ISL_631127 to 631128, EPI_ISL_631131, EPI_ISL_631133, EPI_ISL_631150, EPI_ISL_631163, EPI_ISL_631165,
EPI_ISL_631173, EPI_ISL_631175, EPI_ISL_631184, EPI_ISL_631189 to 631190, EPI_ISL_631202, EPI_ISL_631212, EPI_ISL_631215, EPI_ISL_631217, EPI_ISL_631222, EPI_ISL_631228, EPI_ISL_631231, EPI_ISL_631241, EPI_ISL_631250, EPI_ISL_631265, EPI_ISL_631269, EPI_ISL_634754 to 634788

see above	Lighthouse Lab in Alderley Park	Wellcome Sanger Institute for the COVID-19 Genomics UK (COG-UK) consortium	Cordelia Langford; David K. Jackson; Dominic Kwiatkowski; Ewan Harrison; Ian Johnston; Jacquelyn Wynn; John Sillitoe on behalf of the Wellcome Sanger Institute COVID-19 Surveillance Team; John Sillitoe on behalf of the Wellcome Sanger Institute COVID-19 Surveillance Team (http://www.sanger.ac.uk/covid-team); Mairead Hyland; Roberto Amato; Sonia Goncalves; The Lighthouse Lab in Alderley Park and Alex Alderton

EPI_ISL_551850, EPI_ISL_556698, EPI_ISL_556770, EPI_ISL_556887, EPI_ISL_556940, EPI_ISL_566455, EPI_ISL_566474, EPI_ISL_580332, EPI_ISL_580594, EPI_ISL_596969, EPI_ISL_597074, EPI_ISL_597099, EPI_ISL_597189, EPI_ISL_608347, EPI_ISL_608455, EPI_ISL_608681, EPI_ISL_608907, EPI_ISL_609121,
EPI_ISL_609378, EPI_ISL_609393, EPI_ISL_609450, EPI_ISL_609472, EPI_ISL_609527, EPI_ISL_609534, EPI_ISL_609600, EPI_ISL_609746, EPI_ISL_610702, EPI_ISL_610779, EPI_ISL_610843, EPI_ISL_623477, EPI_ISL_623781, EPI_ISL_625289, EPI_ISL_625312, EPI_ISL_631159, EPI_ISL_633165, EPI_ISL_633221,
EPI_ISL_643315 to 643982, EPI_ISL_644115, EPI_ISL_644117, EPI_ISL_645219 to 645220, EPI_ISL_645222 to 645225, EPI_ISL_645227 to 645239, EPI_ISL_645242, EPI_ISL_645245 to 645262, EPI_ISL_645265, EPI_ISL_645267 to 645284, EPI_ISL_645286 to 645295, EPI_ISL_645298 to 645299, EPI_ISL_645301,
EPI_ISL_645304 to 645307, EPI_ISL_645309 to 645318, EPI_ISL_645320 to 645323, EPI_ISL_645325 to 645326, EPI_ISL_645328, EPI_ISL_645330 to 645344, EPI_ISL_645346 to 645353, EPI_ISL_645355 to 645359, EPI_ISL_645361, EPI_ISL_645363 to 645366, EPI_ISL_645370 to 645373, EPI_ISL_645376 to 645382,
EPI_ISL_645384 to 645390, EPI_ISL_645392, EPI_ISL_645394, EPI_ISL_645396 to 645398, EPI_ISL_645400 to 645404, EPI_ISL_645407 to 645417, EPI_ISL_645419 to 645420, EPI_ISL_645422 to 645423, EPI_ISL_645425 to 645437, EPI_ISL_645439 to 645440, EPI_ISL_645442 to 645451, EPI_ISL_645454 to 645458,
EPI_ISL_645460 to 645463, EPI_ISL_645465 to 645472, EPI_ISL_645474 to 645476, EPI_ISL_645478 to 645488, EPI_ISL_645490 to 645498, EPI_ISL_645500 to 645501, EPI_ISL_645504 to 645507, EPI_ISL_645509 to 645512, EPI_ISL_645514 to 645516, EPI_ISL_645519, EPI_ISL_645521 to 645529, EPI_ISL_645826,
EPI_ISL_645828 to 645829, EPI_ISL_645832, EPI_ISL_645836, EPI_ISL_645838, EPI_ISL_645840, EPI_ISL_645850 to 645852, EPI_ISL_645866, EPI_ISL_645868 to 645869, EPI_ISL_645877, EPI_ISL_645880, EPI_ISL_645882, EPI_ISL_645885 to 645886, EPI_ISL_645896 to 645897, EPI_ISL_645899, EPI_ISL_645902,
EPI_ISL_645905, EPI_ISL_645908, EPI_ISL_645916 to 645918, EPI_ISL_645920, EPI_ISL_645923, EPI_ISL_645925 to 645927, EPI_ISL_645930, EPI_ISL_645945, EPI_ISL_645949, EPI_ISL_645958 to 645959, EPI_ISL_645962, EPI_ISL_645968, EPI_ISL_645970, EPI_ISL_645979 to 645981, EPI_ISL_645983,
EPI_ISL_645987, EPI_ISL_645989, EPI_ISL_645992 to 645995, EPI_ISL_645998, EPI_ISL_646009, EPI_ISL_646014, EPI_ISL_646017 to 646018, EPI_ISL_646022, EPI_ISL_646027 to 646028, EPI_ISL_646030 to 646032, EPI_ISL_646034 to 646035, EPI_ISL_646039, EPI_ISL_646041, EPI_ISL_646046 to 646047,
EPI_ISL_646052, EPI_ISL_646056, EPI_ISL_646058, EPI_ISL_646067, EPI_ISL_646070, EPI_ISL_646076, EPI_ISL_646083 to 646086, EPI_ISL_646089, EPI_ISL_646091, EPI_ISL_646101 to 646102, EPI_ISL_646105, EPI_ISL_646115, EPI_ISL_646122, EPI_ISL_646128 to 646129, EPI_ISL_646131, EPI_ISL_646135 to
646136, EPI_ISL_646139, EPI_ISL_647123 to 647467, EPI_ISL_647470 to 647472, EPI_ISL_647475 to 647476, EPI_ISL_647478 to 647496, EPI_ISL_647498, EPI_ISL_647500 to 647502, EPI_ISL_647504 to 647505, EPI_ISL_647507 to 647510, EPI_ISL_647512, EPI_ISL_647514 to 647525, EPI_ISL_647527, EPI_ISL_647529 to
647534, EPI_ISL_647537 to 647540, EPI_ISL_647542 to 647545, EPI_ISL_647547 to 647549, EPI_ISL_647551 to 647552, EPI_ISL_647554 to 647557, EPI_ISL_647559, EPI_ISL_647561 to 647571, EPI_ISL_647574 to 647575, EPI_ISL_647577 to 647578, EPI_ISL_647581 to 647583, EPI_ISL_647585 to 647590, EPI_ISL_647592
to 647593, EPI_ISL_647595 to 647602, EPI_ISL_647604 to 647605, EPI_ISL_647607, EPI_ISL_647609 to 647611, EPI_ISL_647613 to 647614, EPI_ISL_647616 to 647617, EPI_ISL_647619 to 647623, EPI_ISL_647625 to 647629, EPI_ISL_647631, EPI_ISL_647633, EPI_ISL_647636 to 647637, EPI_ISL_647639 to 647642,
EPI_ISL_647644 to 647649, EPI_ISL_647651 to 647654, EPI_ISL_647656 to 647658, EPI_ISL_647660 to 647666, EPI_ISL_647669 to 647671, EPI_ISL_647673 to 647676, EPI_ISL_647678 to 647681, EPI_ISL_647684 to 647688, EPI_ISL_647690 to 647692, EPI_ISL_647694, EPI_ISL_647696 to 647697, EPI_ISL_647699,
EPI_ISL_647701 to 647703, EPI_ISL_647706 to 647707, EPI_ISL_647709 to 647710, EPI_ISL_647712 to 647725, EPI_ISL_647727 to 647732, EPI_ISL_647734 to 647740, EPI_ISL_647742 to 647749, EPI_ISL_647751, EPI_ISL_647753 to 647761, EPI_ISL_647763 to 647770, EPI_ISL_647772 to 647775, EPI_ISL_647777 to
647779, EPI_ISL_647781 to 647785, EPI_ISL_647788 to 647791, EPI_ISL_647793 to 647796, EPI_ISL_647900 to 647901, EPI_ISL_647906, EPI_ISL_647908 to 647909, EPI_ISL_647913, EPI_ISL_647916, EPI_ISL_649482, EPI_ISL_649492 to 649493, EPI_ISL_649625 to 649633, EPI_ISL_656686 to 656704, EPI_ISL_656706 to
656727, EPI_ISL_656729 to 656730, EPI_ISL_656734 to 656743, EPI_ISL_656747 to 656756, EPI_ISL_656758 to 656773, EPI_ISL_656775 to 656776, EPI_ISL_656780 to 656787, EPI_ISL_656790 to 656797, EPI_ISL_656800 to 656806, EPI_ISL_656809, EPI_ISL_656812 to 656823, EPI_ISL_656825 to 656826,
EPI_ISL_656828, EPI_ISL_656830 to 656833, EPI_ISL_656836 to 656837, EPI_ISL_656839, EPI_ISL_656842 to 656845, EPI_ISL_656847, EPI_ISL_656849 to 656859, EPI_ISL_656863 to 656864, EPI_ISL_656866, EPI_ISL_656868, EPI_ISL_656870, EPI_ISL_656872 to 656876, EPI_ISL_656878, EPI_ISL_656880 to 656886,
EPI_ISL_656888 to 656891, EPI_ISL_656894 to 656905, EPI_ISL_656907 to 656909, EPI_ISL_656911 to 656912, EPI_ISL_656914 to 656919, EPI_ISL_656921, EPI_ISL_656923, EPI_ISL_656925 to 656929, EPI_ISL_656931, EPI_ISL_656933 to 656934, EPI_ISL_656937, EPI_ISL_656939 to 656941, EPI_ISL_656943 to 656944,
EPI_ISL_656946, EPI_ISL_656948 to 656949, EPI_ISL_656951 to 656954, EPI_ISL_656956, EPI_ISL_656959 to 656960, EPI_ISL_656963 to 656964, EPI_ISL_656967 to 656968, EPI_ISL_656970 to 656972, EPI_ISL_656975, EPI_ISL_656977 to 656978, EPI_ISL_656981, EPI_ISL_656983 to 656986, EPI_ISL_656988 to 656990,
EPI_ISL_656992 to 656996, EPI_ISL_656998 to 657002, EPI_ISL_657005 to 657007, EPI_ISL_657009 to 657010, EPI_ISL_657012 to 657013, EPI_ISL_657015, EPI_ISL_657018 to 657020, EPI_ISL_657022, EPI_ISL_657024 to 657025, EPI_ISL_657356 to 657358, EPI_ISL_657362 to 657363, EPI_ISL_657366, EPI_ISL_657368,
EPI_ISL_657374, EPI_ISL_657376, EPI_ISL_657378, EPI_ISL_657380, EPI_ISL_657383, EPI_ISL_657387 to 657389, EPI_ISL_657394 to 657395, EPI_ISL_657397, EPI_ISL_657399, EPI_ISL_657402 to 657404, EPI_ISL_657407, EPI_ISL_657409, EPI_ISL_657412, EPI_ISL_657415, EPI_ISL_657421 to 657423,
EPI_ISL_657426 to 657427, EPI_ISL_657431 to 657437, EPI_ISL_657442, EPI_ISL_657444 to 657445, EPI_ISL_657447 to 657449, EPI_ISL_657454, EPI_ISL_657456, EPI_ISL_657461, EPI_ISL_657468 to 657471, EPI_ISL_657474 to 657478, EPI_ISL_657481 to 657482, EPI_ISL_657489, EPI_ISL_657492, EPI_ISL_657496,
EPI_ISL_657498 to 657499, EPI_ISL_657501 to 657502, EPI_ISL_657504, EPI_ISL_657506 to 657508, EPI_ISL_657511, EPI_ISL_657514, EPI_ISL_657516 to 657517, EPI_ISL_657520 to 657525, EPI_ISL_657528 to 657530, EPI_ISL_657534, EPI_ISL_657537 to 657541, EPI_ISL_657545 to 657546, EPI_ISL_657548 to 657549,
EPI_ISL_657551 to 657552, EPI_ISL_657559, EPI_ISL_657561 to 657562, EPI_ISL_657565 to 657566, EPI_ISL_657569, EPI_ISL_657572 to 657573, EPI_ISL_657576, EPI_ISL_657578, EPI_ISL_657580, EPI_ISL_657582, EPI_ISL_657586, EPI_ISL_657588, EPI_ISL_657590, EPI_ISL_657592, EPI_ISL_657595,
EPI_ISL_657597 to 657598, EPI_ISL_657600 to 657601, EPI_ISL_657603 to 657604, EPI_ISL_657612 to 657613, EPI_ISL_657617 to 657624, EPI_ISL_657626, EPI_ISL_657628, EPI_ISL_657630, EPI_ISL_657633 to 657634, EPI_ISL_657637 to 657638, EPI_ISL_657641, EPI_ISL_657643, EPI_ISL_657645 to 657646,
EPI_ISL_657649, EPI_ISL_657652 to 657656, EPI_ISL_657658 to 657660, EPI_ISL_657664, EPI_ISL_657668 to 657675, EPI_ISL_657677 to 657679, EPI_ISL_657684 to 657687, EPI_ISL_657689 to 657691, EPI_ISL_657693, EPI_ISL_657697 to 657699, EPI_ISL_657701, EPI_ISL_657704, EPI_ISL_657707 to 657708,
EPI_ISL_657713, EPI_ISL_657720 to 657721, EPI_ISL_657723 to 657726, EPI_ISL_657728, EPI_ISL_657731, EPI_ISL_657736 to 657737, EPI_ISL_657739, EPI_ISL_657744, EPI_ISL_657746 to 657747, EPI_ISL_657749 to 657750, EPI_ISL_657754, EPI_ISL_657756, EPI_ISL_657759 to 657760, EPI_ISL_657762 to 657767,
EPI_ISL_657769, EPI_ISL_657771, EPI_ISL_657780, EPI_ISL_657782, EPI_ISL_657786 to 657787, EPI_ISL_657790, EPI_ISL_657793, EPI_ISL_657795, EPI_ISL_657798 to 657799, EPI_ISL_657801 to 657807, EPI_ISL_657809 to 657810, EPI_ISL_657813, EPI_ISL_657815 to 657818, EPI_ISL_657824 to 657826,
EPI_ISL_657828, EPI_ISL_657831, EPI_ISL_657833 to 657834, EPI_ISL_657837 to 657839, EPI_ISL_657841, EPI_ISL_657845 to 657847, EPI_ISL_657849, EPI_ISL_657851 to 657853, EPI_ISL_657855, EPI_ISL_657857, EPI_ISL_657860, EPI_ISL_657863 to 657864, EPI_ISL_657866 to 657868, EPI_ISL_657871,
EPI_ISL_657873, EPI_ISL_657875 to 657877, EPI_ISL_657880 to 657885, EPI_ISL_657888, EPI_ISL_657890 to 657891, EPI_ISL_657893, EPI_ISL_657895 to 657897, EPI_ISL_657899 to 657900, EPI_ISL_657902 to 657904, EPI_ISL_657907 to 657909, EPI_ISL_657911 to 657912, EPI_ISL_657914, EPI_ISL_657917,
EPI_ISL_657920, EPI_ISL_657924, EPI_ISL_657926, EPI_ISL_657931, EPI_ISL_657933 to 657937, EPI_ISL_657939, EPI_ISL_657942 to 657943, EPI_ISL_657945 to 657947, EPI_ISL_657949, EPI_ISL_657951 to 657953, EPI_ISL_657957, EPI_ISL_657960, EPI_ISL_657964, EPI_ISL_657966 to 657968, EPI_ISL_657974,
EPI_ISL_657976, EPI_ISL_657978, EPI_ISL_657983, EPI_ISL_657986 to 657987, EPI_ISL_657989 to 657990, EPI_ISL_657992, EPI_ISL_657994, EPI_ISL_657998, EPI_ISL_658001, EPI_ISL_658007 to 658008, EPI_ISL_658010 to 658014, EPI_ISL_658019 to 658020, EPI_ISL_658024, EPI_ISL_658028 to 658034,
EPI_ISL_658036 to 658037, EPI_ISL_658041, EPI_ISL_658043, EPI_ISL_658045 to 658046, EPI_ISL_658048, EPI_ISL_658052, EPI_ISL_658054 to 658056, EPI_ISL_658063 to 658064, EPI_ISL_658069, EPI_ISL_658071 to 658078, EPI_ISL_658080, EPI_ISL_658083 to 658086, EPI_ISL_658089, EPI_ISL_658092 to 658093,
EPI_ISL_658095 to 658101, EPI_ISL_658104, EPI_ISL_658110, EPI_ISL_658116, EPI_ISL_658119 to 658120, EPI_ISL_658122, EPI_ISL_658124 to 658125, EPI_ISL_658128, EPI_ISL_658131, EPI_ISL_658133 to 658134, EPI_ISL_658138, EPI_ISL_658142, EPI_ISL_658144, EPI_ISL_658148, EPI_ISL_658153,
EPI_ISL_658156 to 658157, EPI_ISL_658159 to 658164, EPI_ISL_658166 to 658167, EPI_ISL_658171, EPI_ISL_658177 to 658179, EPI_ISL_658181, EPI_ISL_658186 to 658192, EPI_ISL_658194 to 658203, EPI_ISL_658206 to 658209, EPI_ISL_658212, EPI_ISL_658214 to 658215, EPI_ISL_658218, EPI_ISL_658220 to 658221,
EPI_ISL_658224, EPI_ISL_658226 to 658228, EPI_ISL_658231, EPI_ISL_658234, EPI_ISL_658236, EPI_ISL_658239, EPI_ISL_658242, EPI_ISL_658246, EPI_ISL_658252, EPI_ISL_658256, EPI_ISL_658258, EPI_ISL_658260, EPI_ISL_658263, EPI_ISL_658266, EPI_ISL_658270 to 658272, EPI_ISL_658274 to 658276,
EPI_ISL_658278 to 658279, EPI_ISL_658285 to 658287, EPI_ISL_658292, EPI_ISL_658299, EPI_ISL_658304 to 658305, EPI_ISL_658307, EPI_ISL_658310 to 658314, EPI_ISL_658316 to 658317, EPI_ISL_658320 to 658321, EPI_ISL_658323, EPI_ISL_658326 to 658327, EPI_ISL_658330 to 658332, EPI_ISL_658334 to 658335,
EPI_ISL_658337, EPI_ISL_658340 to 658341, EPI_ISL_658343, EPI_ISL_658345, EPI_ISL_658348, EPI_ISL_658351 to 658352, EPI_ISL_658355 to 658356, EPI_ISL_658360 to 658364, EPI_ISL_658366 to 658367, EPI_ISL_658370 to 658371, EPI_ISL_658376, EPI_ISL_658381 to 658383, EPI_ISL_658387 to 658388,
EPI_ISL_658390, EPI_ISL_658394, EPI_ISL_658396 to 658398, EPI_ISL_658402, EPI_ISL_658744 to 658745, EPI_ISL_658750, EPI_ISL_658752 to 658753, EPI_ISL_658756, EPI_ISL_658759 to 658760, EPI_ISL_658763 to 658764, EPI_ISL_658767 to 658769, EPI_ISL_658775, EPI_ISL_658781, EPI_ISL_658787 to 658788,
EPI_ISL_658795, EPI_ISL_658800, EPI_ISL_658807, EPI_ISL_658809, EPI_ISL_658816, EPI_ISL_658829, EPI_ISL_658833, EPI_ISL_658836 to 658838, EPI_ISL_658846, EPI_ISL_658852, EPI_ISL_658864, EPI_ISL_658866, EPI_ISL_658877, EPI_ISL_658881, EPI_ISL_658885, EPI_ISL_658900, EPI_ISL_658905,
EPI_ISL_658909, EPI_ISL_658912 to 658914, EPI_ISL_658916, EPI_ISL_658919, EPI_ISL_658921, EPI_ISL_658923, EPI_ISL_658925, EPI_ISL_658930 to 658931, EPI_ISL_658933, EPI_ISL_658936, EPI_ISL_658948, EPI_ISL_658954, EPI_ISL_658959, EPI_ISL_658963, EPI_ISL_658970 to 658971, EPI_ISL_658976,
EPI_ISL_658979, EPI_ISL_658995 to 658996, EPI_ISL_659000, EPI_ISL_659005, EPI_ISL_659007, EPI_ISL_659011, EPI_ISL_659017 to 659019, EPI_ISL_659025, EPI_ISL_659028, EPI_ISL_659040, EPI_ISL_659045, EPI_ISL_659050, EPI_ISL_659053 to 659054, EPI_ISL_659070 to 659073, EPI_ISL_659076 to 659077,
EPI_ISL_659079 to 659080, EPI_ISL_659082 to 659084, EPI_ISL_659090 to 659091, EPI_ISL_659093 to 659094, EPI_ISL_659097 to 659743, EPI_ISL_661638, EPI_ISL_661640 to 661641, EPI_ISL_661647, EPI_ISL_661651 to 661654, EPI_ISL_661656, EPI_ISL_661664, EPI_ISL_661675, EPI_ISL_661679 to 661680,
EPI_ISL_661684, EPI_ISL_661687, EPI_ISL_661691, EPI_ISL_661693, EPI_ISL_661695 to 661697, EPI_ISL_661702, EPI_ISL_661710 to 661713, EPI_ISL_661717, EPI_ISL_661721, EPI_ISL_661726, EPI_ISL_661729, EPI_ISL_661732, EPI_ISL_661734, EPI_ISL_661741 to 661743, EPI_ISL_661747, EPI_ISL_661749,
EPI_ISL_661751 to 661752, EPI_ISL_661759, EPI_ISL_661761, EPI_ISL_661775, EPI_ISL_661778 to 661779, EPI_ISL_661781, EPI_ISL_661783, EPI_ISL_661785, EPI_ISL_661789, EPI_ISL_661791, EPI_ISL_661797, EPI_ISL_661799, EPI_ISL_661801, EPI_ISL_661805, EPI_ISL_661811, EPI_ISL_661815, EPI_ISL_661817,
EPI_ISL_661830, EPI_ISL_661833 to 661834, EPI_ISL_661836, EPI_ISL_661844 to 661845, EPI_ISL_661853 to 661854, EPI_ISL_661856, EPI_ISL_661863, EPI_ISL_661869, EPI_ISL_661873 to 661874, EPI_ISL_661876, EPI_ISL_661881, EPI_ISL_661883, EPI_ISL_661886 to 661887, EPI_ISL_661895, EPI_ISL_661897,
EPI_ISL_661900, EPI_ISL_661902 to 661903, EPI_ISL_661917 to 661918, EPI_ISL_661925 to 661926, EPI_ISL_661928, EPI_ISL_661930, EPI_ISL_661941, EPI_ISL_661944, EPI_ISL_661947, EPI_ISL_661951, EPI_ISL_661953 to 661954, EPI_ISL_662631 to 662632, EPI_ISL_662635, EPI_ISL_662651 to 662653,
EPI_ISL_667811 to 667813, EPI_ISL_667815, EPI_ISL_667817 to 667821, EPI_ISL_667823, EPI_ISL_667826 to 667827, EPI_ISL_667829 to 667830, EPI_ISL_667833 to 667835, EPI_ISL_667837, EPI_ISL_667839 to 667842, EPI_ISL_667845 to 667846, EPI_ISL_667848, EPI_ISL_667852 to 667854, EPI_ISL_667856,
EPI_ISL_667858, EPI_ISL_667861 to 667862, EPI_ISL_667865, EPI_ISL_667869 to 667871, EPI_ISL_667874 to 667875, EPI_ISL_667877 to 667878, EPI_ISL_667880, EPI_ISL_667882, EPI_ISL_667884, EPI_ISL_667886, EPI_ISL_667890, EPI_ISL_667892, EPI_ISL_667894, EPI_ISL_667896 to 667897, EPI_ISL_667899,
EPI_ISL_667901 to 667903, EPI_ISL_667905 to 667906, EPI_ISL_667908 to 667911, EPI_ISL_667913 to 667914, EPI_ISL_667916, EPI_ISL_667921, EPI_ISL_667923, EPI_ISL_667926, EPI_ISL_667932 to 667937, EPI_ISL_667942, EPI_ISL_667944 to 667946, EPI_ISL_667949, EPI_ISL_667951, EPI_ISL_667953 to 667956,
EPI_ISL_667961, EPI_ISL_667964 to 667965, EPI_ISL_667968, EPI_ISL_667971, EPI_ISL_667974, EPI_ISL_667979, EPI_ISL_667981, EPI_ISL_667984 to 667985, EPI_ISL_667988, EPI_ISL_667991, EPI_ISL_667993 to 667995, EPI_ISL_667999, EPI_ISL_668002 to 668003, EPI_ISL_668007 to 668009, EPI_ISL_668011 to
668012, EPI_ISL_668014 to 668019, EPI_ISL_668023, EPI_ISL_668025 to 668027, EPI_ISL_668037, EPI_ISL_668039 to 668043, EPI_ISL_668046 to 668048, EPI_ISL_668051, EPI_ISL_668058 to 668059, EPI_ISL_668061, EPI_ISL_668065, EPI_ISL_668068 to 668072, EPI_ISL_668076 to 668077, EPI_ISL_668079 to 668081,
EPI_ISL_668085 to 668092, EPI_ISL_668097, EPI_ISL_668099 to 668101, EPI_ISL_668103 to 668104, EPI_ISL_668108 to 668110, EPI_ISL_668112, EPI_ISL_668115, EPI_ISL_668122 to 668123, EPI_ISL_668127 to 668128, EPI_ISL_668133, EPI_ISL_668136 to 668138, EPI_ISL_668140 to 668141, EPI_ISL_668148 to 668149,
EPI_ISL_668153 to 668155, EPI_ISL_668163, EPI_ISL_668166, EPI_ISL_668169, EPI_ISL_668171 to 668172, EPI_ISL_668180, EPI_ISL_668188, EPI_ISL_668190, EPI_ISL_668192, EPI_ISL_668194, EPI_ISL_668200 to 668201, EPI_ISL_668211 to 668212, EPI_ISL_668219, EPI_ISL_668222, EPI_ISL_668227,
EPI_ISL_668237, EPI_ISL_668239, EPI_ISL_668251 to 668252, EPI_ISL_668254 to 668255, EPI_ISL_668261 to 668262, EPI_ISL_668265, EPI_ISL_668267, EPI_ISL_668275, EPI_ISL_668280 to 668281, EPI_ISL_668283, EPI_ISL_668289, EPI_ISL_668294, EPI_ISL_668296 to 668299, EPI_ISL_668313, EPI_ISL_668315 to
668317, EPI_ISL_668319, EPI_ISL_668322, EPI_ISL_668329, EPI_ISL_668336, EPI_ISL_668341 to 668342, EPI_ISL_668345, EPI_ISL_668353, EPI_ISL_668355, EPI_ISL_668365, EPI_ISL_668368 to 668369, EPI_ISL_668373 to 668374, EPI_ISL_668376, EPI_ISL_668379, EPI_ISL_668382, EPI_ISL_668385, EPI_ISL_668388
to 668389, EPI_ISL_672912 to 673565, EPI_ISL_673567 to 673576, EPI_ISL_673578 to 673591, EPI_ISL_673593, EPI_ISL_673595 to 673598, EPI_ISL_673600, EPI_ISL_673602, EPI_ISL_673604 to 673615, EPI_ISL_673617 to 673620, EPI_ISL_673622 to 673632, EPI_ISL_673635 to 673641, EPI_ISL_673643 to 673644,
EPI_ISL_673647 to 673651, EPI_ISL_673653 to 673654, EPI_ISL_673656, EPI_ISL_673658, EPI_ISL_673660 to 673662, EPI_ISL_673664 to 673672, EPI_ISL_673674 to 673675, EPI_ISL_673677 to 673685, EPI_ISL_673687, EPI_ISL_673689 to 673690, EPI_ISL_673692 to 673695, EPI_ISL_673697 to 673701, EPI_ISL_673703
to 673707, EPI_ISL_673710 to 673714, EPI_ISL_673716 to 673721, EPI_ISL_673723 to 673725, EPI_ISL_673727 to 673728, EPI_ISL_673731 to 673732, EPI_ISL_673734 to 673737, EPI_ISL_673739 to 673744, EPI_ISL_673747 to 673749, EPI_ISL_673751 to 673759, EPI_ISL_673761 to 673763, EPI_ISL_673766,
EPI_ISL_673768 to 673769, EPI_ISL_673771 to 673777, EPI_ISL_673779 to 673784, EPI_ISL_673786, EPI_ISL_673788 to 673789, EPI_ISL_673791 to 673795, EPI_ISL_673797, EPI_ISL_673799 to 673800, EPI_ISL_673802, EPI_ISL_673804 to 673811, EPI_ISL_673815 to 673820, EPI_ISL_673823 to 673826, EPI_ISL_673830
to 673831, EPI_ISL_673836 to 673839, EPI_ISL_673841 to 673847, EPI_ISL_673849 to 673855, EPI_ISL_673858, EPI_ISL_673860 to 673864, EPI_ISL_673866, EPI_ISL_673868 to 673870, EPI_ISL_674826, EPI_ISL_674828, EPI_ISL_674834, EPI_ISL_674837 to 674838, EPI_ISL_674840, EPI_ISL_674845 to 674847,
EPI_ISL_674849 to 674851, EPI_ISL_674859, EPI_ISL_674862, EPI_ISL_674866 to 674868, EPI_ISL_674871, EPI_ISL_674873, EPI_ISL_674876, EPI_ISL_674883, EPI_ISL_674893, EPI_ISL_674896, EPI_ISL_674899, EPI_ISL_674906, EPI_ISL_674908 to 674910, EPI_ISL_674913, EPI_ISL_674917, EPI_ISL_674919 to
674920, EPI_ISL_674922 to 674924, EPI_ISL_674926, EPI_ISL_674933, EPI_ISL_674936, EPI_ISL_674939 to 674940, EPI_ISL_674944, EPI_ISL_674946, EPI_ISL_674949 to 674953, EPI_ISL_674957, EPI_ISL_674959, EPI_ISL_674962, EPI_ISL_674972 to 674973, EPI_ISL_674977, EPI_ISL_674983, EPI_ISL_674988,
EPI_ISL_674992, EPI_ISL_674995, EPI_ISL_674998, EPI_ISL_675006, EPI_ISL_675008 to 675009, EPI_ISL_675012, EPI_ISL_675018, EPI_ISL_675024, EPI_ISL_675026 to 675027, EPI_ISL_675033, EPI_ISL_675038 to 675040, EPI_ISL_675042, EPI_ISL_675046, EPI_ISL_675053, EPI_ISL_675056, EPI_ISL_675064,
EPI_ISL_675071, EPI_ISL_675075, EPI_ISL_675080, EPI_ISL_675082, EPI_ISL_675087, EPI_ISL_675094, EPI_ISL_675104, EPI_ISL_675107, EPI_ISL_675110, EPI_ISL_675117, EPI_ISL_675120, EPI_ISL_675126, EPI_ISL_676383, EPI_ISL_681656 to 681659, EPI_ISL_681671 to 681672, EPI_ISL_702023 to 702096,
EPI_ISL_702098 to 702175, EPI_ISL_702177 to 702243, EPI_ISL_702245 to 702369, EPI_ISL_704688 to 704689, EPI_ISL_704691, EPI_ISL_704693 to 704694, EPI_ISL_704696 to 704697, EPI_ISL_704699 to 704700, EPI_ISL_704702, EPI_ISL_704704 to 704705, EPI_ISL_704707 to 704708, EPI_ISL_704710, EPI_ISL_704712
to 704713, EPI_ISL_704715, EPI_ISL_704717 to 704718, EPI_ISL_704720 to 704721, EPI_ISL_704723 to 704724, EPI_ISL_704726 to 704727, EPI_ISL_704729 to 704730, EPI_ISL_704732 to 704733, EPI_ISL_704735, EPI_ISL_704737 to 704738, EPI_ISL_704740, EPI_ISL_704742 to 704743, EPI_ISL_704745, EPI_ISL_704747
to 704748, EPI_ISL_704750 to 704751, EPI_ISL_704753, EPI_ISL_704755 to 704756, EPI_ISL_704758, EPI_ISL_704760 to 704761, EPI_ISL_704763 to 704764, EPI_ISL_704766 to 704767, EPI_ISL_704769, EPI_ISL_704771, EPI_ISL_704773 to 704774, EPI_ISL_704776, EPI_ISL_704778 to 704779, EPI_ISL_704781 to 704782,
EPI_ISL_704784 to 704785, EPI_ISL_704787, EPI_ISL_704789 to 704790, EPI_ISL_704792 to 704793, EPI_ISL_704795 to 704796, EPI_ISL_704798, EPI_ISL_704800 to 704801, EPI_ISL_704803, EPI_ISL_704805 to 704806, EPI_ISL_704808, EPI_ISL_704810 to 704811, EPI_ISL_704813 to 704814, EPI_ISL_704816,
EPI_ISL_704818 to 704819, EPI_ISL_704821 to 704822, EPI_ISL_704824 to 704825, EPI_ISL_704827, EPI_ISL_704829 to 704830, EPI_ISL_704832 to 704833, EPI_ISL_704835, EPI_ISL_704837 to 704838, EPI_ISL_704840 to 704841, EPI_ISL_704843 to 704844, EPI_ISL_704846, EPI_ISL_704848 to 704849, EPI_ISL_704851
to 704852, EPI_ISL_704854, EPI_ISL_704856 to 704857, EPI_ISL_704859 to 704860, EPI_ISL_704862 to 704863, EPI_ISL_704865, EPI_ISL_704867 to 704868, EPI_ISL_704870 to 704871, EPI_ISL_704873, EPI_ISL_704875 to 704876, EPI_ISL_704878, EPI_ISL_704880 to 704881, EPI_ISL_704883 to 704884, EPI_ISL_704886,
EPI_ISL_704888 to 704889, EPI_ISL_704891, EPI_ISL_704893 to 704894, EPI_ISL_704896, EPI_ISL_704898 to 704899, EPI_ISL_704901 to 704902, EPI_ISL_704904, EPI_ISL_704906 to 704907, EPI_ISL_704909 to 704910, EPI_ISL_704912 to 704913, EPI_ISL_704915, EPI_ISL_704917, EPI_ISL_704919 to 704920,
EPI_ISL_704922, EPI_ISL_704924 to 704925, EPI_ISL_704927 to 704928, EPI_ISL_704930, EPI_ISL_704932 to 704933, EPI_ISL_704935 to 704936, EPI_ISL_704938, EPI_ISL_704940 to 704941, EPI_ISL_704943 to 704944, EPI_ISL_704946, EPI_ISL_704948 to 704949, EPI_ISL_704951, EPI_ISL_704953 to 704954,
EPI_ISL_704956, EPI_ISL_704958 to 704959, EPI_ISL_704961 to 704962, EPI_ISL_704964, EPI_ISL_704966 to 704967, EPI_ISL_704969, EPI_ISL_704971 to 704972, EPI_ISL_704974 to 704975, EPI_ISL_704977, EPI_ISL_704979 to 704980, EPI_ISL_704982, EPI_ISL_704984 to 704985, EPI_ISL_704987 to 704988,
EPI_ISL_704990, EPI_ISL_704992 to 704993, EPI_ISL_704995 to 704996, EPI_ISL_704998, EPI_ISL_705000 to 705001, EPI_ISL_705003 to 705004, EPI_ISL_705006, EPI_ISL_705008 to 705009, EPI_ISL_705011 to 705012, EPI_ISL_705014, EPI_ISL_705016 to 705017, EPI_ISL_705019 to 705020, EPI_ISL_705022 to 705023,
EPI_ISL_705025, EPI_ISL_705027 to 705029, EPI_ISL_705031, EPI_ISL_705033 to 705034, EPI_ISL_705036 to 705037, EPI_ISL_705039, EPI_ISL_705041 to 705042, EPI_ISL_705044 to 705045, EPI_ISL_705047, EPI_ISL_705049 to 705050, EPI_ISL_705052 to 705053, EPI_ISL_705055, EPI_ISL_705057 to 705058,
EPI_ISL_705060 to 705061, EPI_ISL_705063, EPI_ISL_705065 to 705066, EPI_ISL_705068 to 705069, EPI_ISL_705071, EPI_ISL_705073 to 705074, EPI_ISL_705076 to 705077, EPI_ISL_705079 to 705080, EPI_ISL_705082, EPI_ISL_705084 to 705085, EPI_ISL_705087, EPI_ISL_705089 to 705090, EPI_ISL_705092 to 705093,
EPI_ISL_705095, EPI_ISL_705097 to 705098, EPI_ISL_705100 to 705101, EPI_ISL_705103 to 705104, EPI_ISL_705106 to 705107, EPI_ISL_705109, EPI_ISL_705111 to 705112, EPI_ISL_705114, EPI_ISL_705116 to 705117, EPI_ISL_705119 to 705120, EPI_ISL_705122 to 705123, EPI_ISL_705125, EPI_ISL_705127 to 705128,
EPI_ISL_705130 to 705131, EPI_ISL_705133, EPI_ISL_705135 to 705136, EPI_ISL_705138 to 705139, EPI_ISL_705141 to 705142, EPI_ISL_705144, EPI_ISL_705146 to 705147, EPI_ISL_705149, EPI_ISL_705151 to 705152, EPI_ISL_705154 to 705155, EPI_ISL_705157, EPI_ISL_705159 to 705160, EPI_ISL_705162,
EPI_ISL_705164 to 705165, EPI_ISL_705167, EPI_ISL_705169 to 705170, EPI_ISL_705172 to 705173, EPI_ISL_705175, EPI_ISL_705177 to 705178, EPI_ISL_705180 to 705181, EPI_ISL_705183, EPI_ISL_705185 to 705186, EPI_ISL_705188 to 705189, EPI_ISL_708989 to 708990, EPI_ISL_708992 to 708997, EPI_ISL_708999
to 709003, EPI_ISL_709006 to 709008, EPI_ISL_709010 to 709012, EPI_ISL_709014 to 709021, EPI_ISL_709023 to 709030, EPI_ISL_709032 to 709035, EPI_ISL_709037 to 709039, EPI_ISL_709041 to 709043, EPI_ISL_709045 to 709047, EPI_ISL_709049, EPI_ISL_709051 to 709055, EPI_ISL_709057, EPI_ISL_709059,
EPI_ISL_709061 to 709064, EPI_ISL_709066 to 709076, EPI_ISL_709080 to 709081, EPI_ISL_709083 to 709090, EPI_ISL_709092 to 709097, EPI_ISL_709099 to 709103, EPI_ISL_709105 to 709106, EPI_ISL_709111 to 709118, EPI_ISL_709120 to 709125, EPI_ISL_709127 to 709130, EPI_ISL_709134, EPI_ISL_709136 to
709138, EPI_ISL_709143, EPI_ISL_709145 to 709147, EPI_ISL_709149, EPI_ISL_709152 to 709158, EPI_ISL_709162 to 709169, EPI_ISL_709172 to 709173, EPI_ISL_709175 to 709181, EPI_ISL_709184 to 709188, EPI_ISL_709192 to 709198, EPI_ISL_709200, EPI_ISL_709203 to 709214, EPI_ISL_709216 to 709230,
EPI_ISL_709232 to 709233, EPI_ISL_709237 to 709238, EPI_ISL_709240 to 709241, EPI_ISL_709244, EPI_ISL_709246 to 709247, EPI_ISL_709249 to 709263, EPI_ISL_709265 to 709273, EPI_ISL_709275 to 709276, EPI_ISL_709278 to 709281, EPI_ISL_709284, EPI_ISL_709286 to 709287, EPI_ISL_709289, EPI_ISL_709293
to 709294, EPI_ISL_709804 to 709805, EPI_ISL_709807 to 709808, EPI_ISL_709810, EPI_ISL_709813 to 709814, EPI_ISL_709817, EPI_ISL_709821 to 709822, EPI_ISL_709825, EPI_ISL_709827, EPI_ISL_709829, EPI_ISL_709836 to 709837, EPI_ISL_709842 to 709844, EPI_ISL_709846, EPI_ISL_709850, EPI_ISL_709854,
EPI_ISL_709864 to 709865, EPI_ISL_709867, EPI_ISL_709869 to 709870, EPI_ISL_709873, EPI_ISL_709876 to 709878, EPI_ISL_709880, EPI_ISL_710821 to 710829, EPI_ISL_710835 to 710836, EPI_ISL_710878, EPI_ISL_711023 to 711026, EPI_ISL_711038, EPI_ISL_718318 to 718628, EPI_ISL_718630, EPI_ISL_718632,

EPI_ISL_718634, EPI_ISL_718636, EPI_ISL_718638, EPI_ISL_718642, EPI_ISL_718645 to 718646, EPI_ISL_718648 to 718649, EPI_ISL_718651 to 718652, EPI_ISL_718654 to 718656, EPI_ISL_718659 to 718661, EPI_ISL_718667 to 718669, EPI_ISL_718671, EPI_ISL_718673, EPI_ISL_718677, EPI_ISL_718679 to 718681, EPI_ISL_718683 to 718685, EPI_ISL_718688 to 718690, EPI_ISL_718692 to 718693, EPI_ISL_718696 to 718699, EPI_ISL_718701 to 718704, EPI_ISL_718706, EPI_ISL_718708 to 718709, EPI_ISL_718717, EPI_ISL_718720, EPI_ISL_718722, EPI_ISL_718726 to 718727, EPI_ISL_718730 to 718734, EPI_ISL_718736 to 718737, EPI_ISL_718741, EPI_ISL_718743, EPI_ISL_718748 to 718749, EPI_ISL_718751 to 718753, EPI_ISL_718756 to 718758, EPI_ISL_718760, EPI_ISL_718763, EPI_ISL_718768, EPI_ISL_718770 to 718771, EPI_ISL_718775 to 718776, EPI_ISL_718778, EPI_ISL_718784 to 718786, EPI_ISL_718788, EPI_ISL_718790, EPI_ISL_718792 to 718794, EPI_ISL_718797, EPI_ISL_718800, EPI_ISL_718804 to 718805, EPI_ISL_718809 to 718810, EPI_ISL_718814 to 718818, EPI_ISL_718820, EPI_ISL_718822, EPI_ISL_718824 to 718826, EPI_ISL_718834 to 718836, EPI_ISL_718840, EPI_ISL_718842 to 718843, EPI_ISL_718845 to 718846, EPI_ISL_718848, EPI_ISL_718851 to 718852, EPI_ISL_718855 to 718857, EPI_ISL_718860 to 718863, EPI_ISL_718867 to 718869, EPI_ISL_718874 to 718876, EPI_ISL_718879, EPI_ISL_718882 to 718884, EPI_ISL_718892 to 718893, EPI_ISL_718896 to 718900, EPI_ISL_718904 to 718906, EPI_ISL_718909 to 718910, EPI_ISL_718912 to 718913, EPI_ISL_718916, EPI_ISL_718918, EPI_ISL_718920 to 718921, EPI_ISL_718924 to 718925, EPI_ISL_718927 to 718928, EPI_ISL_718931, EPI_ISL_718933 to 718934, EPI_ISL_718937 to 718938, EPI_ISL_718940 to 718942, EPI_ISL_718944, EPI_ISL_718946 to 718949, EPI_ISL_718951, EPI_ISL_718954, EPI_ISL_718956, EPI_ISL_718958, EPI_ISL_718960 to 718961, EPI_ISL_718965, EPI_ISL_718968 to 718969, EPI_ISL_718971 to 718973, EPI_ISL_718980 to 719604, EPI_ISL_728299, EPI_ISL_733596 to 733597, EPI_ISL_733599, EPI_ISL_733601 to 733602, EPI_ISL_733604, EPI_ISL_733606, EPI_ISL_733608, EPI_ISL_733610, EPI_ISL_733612, EPI_ISL_733614 to 733615, EPI_ISL_733619 to 733620, EPI_ISL_733622, EPI_ISL_733624, EPI_ISL_733626 to 733627, EPI_ISL_733629, EPI_ISL_733631 to 733634, EPI_ISL_733637, EPI_ISL_733640, EPI_ISL_733646, EPI_ISL_733649 to 733651, EPI_ISL_733655 to 733656, EPI_ISL_733660 to 733663, EPI_ISL_733665, EPI_ISL_733667 to 733668, EPI_ISL_733670 to 733672, EPI_ISL_733674 to 733677, EPI_ISL_733680 to 733681, EPI_ISL_733684, EPI_ISL_733688 to 733691, EPI_ISL_733694 to 733696, EPI_ISL_733698, EPI_ISL_733700, EPI_ISL_733702, EPI_ISL_733704 to 733705, EPI_ISL_733708 to 733709, EPI_ISL_733714 to 733717, EPI_ISL_733719 to 733724, EPI_ISL_733726 to 733730, EPI_ISL_733732 to 733733, EPI_ISL_733736, EPI_ISL_733738, EPI_ISL_733740 to 733741, EPI_ISL_733744 to 733745, EPI_ISL_733750 to 733753, EPI_ISL_733758, EPI_ISL_733763, EPI_ISL_733765, EPI_ISL_733767, EPI_ISL_733770, EPI_ISL_733772 to 733775, EPI_ISL_733778 to 733779, EPI_ISL_733782, EPI_ISL_733784, EPI_ISL_733786 to 733788, EPI_ISL_733790 to 733793, EPI_ISL_733799 to 733800, EPI_ISL_733802, EPI_ISL_733804 to 733805, EPI_ISL_733809 to 733810, EPI_ISL_733812, EPI_ISL_733814, EPI_ISL_733816 to 733817, EPI_ISL_733821 to 733824, EPI_ISL_733827, EPI_ISL_733831, EPI_ISL_733834 to 733835, EPI_ISL_733839 to 733841, EPI_ISL_733846 to 733847, EPI_ISL_733849, EPI_ISL_733852 to 733854, EPI_ISL_733856 to 733857, EPI_ISL_733860, EPI_ISL_733862, EPI_ISL_733866, EPI_ISL_733868 to 733871, EPI_ISL_733874, EPI_ISL_733876, EPI_ISL_733878, EPI_ISL_733881, EPI_ISL_733883, EPI_ISL_733886, EPI_ISL_733888, EPI_ISL_733892, EPI_ISL_733895 to 733896, EPI_ISL_733898 to 733899, EPI_ISL_733906 to 733911, EPI_ISL_733915, EPI_ISL_756379 to 756380, EPI_ISL_756384, EPI_ISL_756386, EPI_ISL_756389, EPI_ISL_756392 to 756393, EPI_ISL_756395, EPI_ISL_756403, EPI_ISL_756409, EPI_ISL_756413, EPI_ISL_756415 to 756420, EPI_ISL_756422, EPI_ISL_756424, EPI_ISL_756426, EPI_ISL_756429, EPI_ISL_756431 to 756432, EPI_ISL_756434, EPI_ISL_756438, EPI_ISL_756442, EPI_ISL_756444, EPI_ISL_756446, EPI_ISL_756448, EPI_ISL_756453 to 756454, EPI_ISL_756456, EPI_ISL_756460, EPI_ISL_756467, EPI_ISL_756469, EPI_ISL_756474 to 756475, EPI_ISL_756478, EPI_ISL_756480 to 756481, EPI_ISL_756484 to 756487, EPI_ISL_756490 to 756492, EPI_ISL_756496 to 756502, EPI_ISL_756505 to 756508, EPI_ISL_756511 to 756513, EPI_ISL_756517 to 756519, EPI_ISL_756521 to 756522, EPI_ISL_756525, EPI_ISL_756528 to 756529, EPI_ISL_756534 to 756535, EPI_ISL_756540, EPI_ISL_756542 to 756543, EPI_ISL_756545, EPI_ISL_756549, EPI_ISL_756553 to 756554, EPI_ISL_756558 to 756559, EPI_ISL_756564, EPI_ISL_756567 to 756568, EPI_ISL_756570, EPI_ISL_756572, EPI_ISL_756576 to 756577, EPI_ISL_756579, EPI_ISL_756582, EPI_ISL_756584 to 756586, EPI_ISL_756588 to 756591, EPI_ISL_756594 to 756595, EPI_ISL_756597, EPI_ISL_756601, EPI_ISL_756604 to 756608, EPI_ISL_756610 to 756611, EPI_ISL_756613 to 756615, EPI_ISL_756617, EPI_ISL_756621 to 756623, EPI_ISL_756625, EPI_ISL_756627 to 756631, EPI_ISL_756633, EPI_ISL_756637, EPI_ISL_756642 to 756643, EPI_ISL_756649, EPI_ISL_756653, EPI_ISL_756655, EPI_ISL_756659 to 756660, EPI_ISL_756665 to 756668, EPI_ISL_756671 to 756672, EPI_ISL_756674 to 756680, EPI_ISL_757119, EPI_ISL_760813, EPI_ISL_760822, EPI_ISL_760833, EPI_ISL_760858 to 760859, EPI_ISL_760883, EPI_ISL_760894, EPI_ISL_760903, EPI_ISL_760920, EPI_ISL_760922, EPI_ISL_760932, EPI_ISL_760936, EPI_ISL_760939, EPI_ISL_760969, EPI_ISL_760976, EPI_ISL_760988, EPI_ISL_761017, EPI_ISL_761021, EPI_ISL_761057, EPI_ISL_761074 to 761075, EPI_ISL_761922, EPI_ISL_761927, EPI_ISL_761929, EPI_ISL_761935, EPI_ISL_761937, EPI_ISL_761939, EPI_ISL_761946, EPI_ISL_761952 to 761953, EPI_ISL_761955 to 761956, EPI_ISL_761961, EPI_ISL_761967 to 761968, EPI_ISL_761970, EPI_ISL_761979, EPI_ISL_761981, EPI_ISL_761994 to 761995, EPI_ISL_761997, EPI_ISL_761999, EPI_ISL_762007 to 762009, EPI_ISL_762014 to 762016, EPI_ISL_762018 to 762019, EPI_ISL_762023 to 762030, EPI_ISL_762036 to 762038, EPI_ISL_762041 to 762043, EPI_ISL_762045, EPI_ISL_762050, EPI_ISL_762057, EPI_ISL_762059 to 762061, EPI_ISL_762063 to 762067, EPI_ISL_762069, EPI_ISL_762072 to 762075, EPI_ISL_762078, EPI_ISL_762085, EPI_ISL_762087, EPI_ISL_762089 to 762091, EPI_ISL_762096 to 762098, EPI_ISL_762103, EPI_ISL_762109, EPI_ISL_762112 to 762114, EPI_ISL_762117 to 762118, EPI_ISL_762122, EPI_ISL_762124 to 762126, EPI_ISL_762131, EPI_ISL_762133, EPI_ISL_762135 to 762140, EPI_ISL_762142, EPI_ISL_762146 to 762148, EPI_ISL_762152 to 762153, EPI_ISL_762155, EPI_ISL_762163, EPI_ISL_762166, EPI_ISL_762173, EPI_ISL_762175, EPI_ISL_762178, EPI_ISL_762181, EPI_ISL_762183, EPI_ISL_762186 to 762187, EPI_ISL_762189, EPI_ISL_762192 to 762193, EPI_ISL_762195, EPI_ISL_762546, EPI_ISL_762549 to 762553, EPI_ISL_762559, EPI_ISL_762563, EPI_ISL_762565 to 762566, EPI_ISL_762568 to 762572, EPI_ISL_762575, EPI_ISL_762577 to 762580, EPI_ISL_762582, EPI_ISL_762584 to 762585, EPI_ISL_762587, EPI_ISL_762590 to 762596, EPI_ISL_762598, EPI_ISL_762600 to 762601, EPI_ISL_762603, EPI_ISL_762607 to 762608, EPI_ISL_762611 to 762613, EPI_ISL_762615 to 762616, EPI_ISL_762619, EPI_ISL_762621, EPI_ISL_762623 to 762624, EPI_ISL_762626 to 762628, EPI_ISL_762632, EPI_ISL_762635 to 762637, EPI_ISL_762640 to 762641, EPI_ISL_762645, EPI_ISL_762649 to 762651, EPI_ISL_762658, EPI_ISL_762660, EPI_ISL_762664 to 762665, EPI_ISL_762667 to 762668, EPI_ISL_762670, EPI_ISL_762673, EPI_ISL_762675, EPI_ISL_762677, EPI_ISL_762679 to 762682, EPI_ISL_762684 to 762685, EPI_ISL_762687 to 762689, EPI_ISL_762695 to 762697, EPI_ISL_762699 to 762700, EPI_ISL_762705, EPI_ISL_762713, EPI_ISL_762715 to 762719, EPI_ISL_762721, EPI_ISL_762723, EPI_ISL_762725, EPI_ISL_762727, EPI_ISL_762729, EPI_ISL_762736 to 762738, EPI_ISL_762740 to 762742, EPI_ISL_762745, EPI_ISL_762747 to 762748, EPI_ISL_762751 to 762753, EPI_ISL_762755, EPI_ISL_762757, EPI_ISL_762762, EPI_ISL_762765, EPI_ISL_762767 to 762769, EPI_ISL_762772, EPI_ISL_762774, EPI_ISL_762778, EPI_ISL_762780 to 762784, EPI_ISL_762787 to 762788, EPI_ISL_762790, EPI_ISL_762792, EPI_ISL_762795, EPI_ISL_762797, EPI_ISL_762800, EPI_ISL_762803 to 762808, EPI_ISL_762810 to 762811, EPI_ISL_762817 to 762818, EPI_ISL_762820, EPI_ISL_762822, EPI_ISL_762825, EPI_ISL_762827, EPI_ISL_762829, EPI_ISL_762831, EPI_ISL_762833 to 762834, EPI_ISL_762836, EPI_ISL_762838, EPI_ISL_762840, EPI_ISL_762842, EPI_ISL_762848, EPI_ISL_762852, EPI_ISL_762855, EPI_ISL_762857 to 762858, EPI_ISL_767115, EPI_ISL_767138, EPI_ISL_768845, EPI_ISL_768901 to 769222, EPI_ISL_777133, EPI_ISL_777173, EPI_ISL_777181, EPI_ISL_777201, EPI_ISL_777218, EPI_ISL_777275, EPI_ISL_777289, EPI_ISL_777293, EPI_ISL_777320, EPI_ISL_777323, EPI_ISL_777328 to 777332, EPI_ISL_777334, EPI_ISL_777339, EPI_ISL_777342 to 777345, EPI_ISL_777347 to 777348, EPI_ISL_777350 to 777352, EPI_ISL_777354 to 777355, EPI_ISL_777357, EPI_ISL_777359 to 777360, EPI_ISL_777362, EPI_ISL_777365 to 777368, EPI_ISL_777370 to 777371, EPI_ISL_777374, EPI_ISL_777378 to 777379, EPI_ISL_777381, EPI_ISL_777384, EPI_ISL_777387 to 777388, EPI_ISL_777390 to 777391, EPI_ISL_777396, EPI_ISL_777398, EPI_ISL_777400 to 777401, EPI_ISL_777403 to 777406, EPI_ISL_777408 to 777409, EPI_ISL_777413 to 777415, EPI_ISL_777418 to 777419, EPI_ISL_777422, EPI_ISL_777426, EPI_ISL_777428 to 777429, EPI_ISL_777431, EPI_ISL_777433 to 777434, EPI_ISL_777439, EPI_ISL_777441 to 777442, EPI_ISL_777445 to 777446, EPI_ISL_777450, EPI_ISL_777452 to 777454, EPI_ISL_777456 to 777457, EPI_ISL_777460, EPI_ISL_777463 to 777464, EPI_ISL_777468 to 777469, EPI_ISL_777472 to 777473, EPI_ISL_777476, EPI_ISL_777478, EPI_ISL_777480, EPI_ISL_777482 to 777483, EPI_ISL_777485 to 777486, EPI_ISL_777488, EPI_ISL_777492, EPI_ISL_777494, EPI_ISL_777499 to 777501, EPI_ISL_777503, EPI_ISL_777507 to 777508, EPI_ISL_777511 to 777513, EPI_ISL_777516 to 777517, EPI_ISL_777519, EPI_ISL_777521, EPI_ISL_777527 to 777528, EPI_ISL_777536, EPI_ISL_777540 to 777542, EPI_ISL_777544 to 777547, EPI_ISL_777549, EPI_ISL_777551, EPI_ISL_777553, EPI_ISL_777555 to 777556, EPI_ISL_777559, EPI_ISL_777564 to 777566, EPI_ISL_777570 to 777571, EPI_ISL_777575, EPI_ISL_777577 to 777578, EPI_ISL_777581, EPI_ISL_777585 to 777586, EPI_ISL_777588, EPI_ISL_777590, EPI_ISL_777594, EPI_ISL_777596, EPI_ISL_777598 to 777599, EPI_ISL_777602 to 777604, EPI_ISL_777606 to 777609, EPI_ISL_777611, EPI_ISL_777613 to 777620, EPI_ISL_777624 to 777626, EPI_ISL_777631 to 777632, EPI_ISL_777635 to 777637, EPI_ISL_777640, EPI_ISL_777642 to 777643, EPI_ISL_777645, EPI_ISL_777647 to 777648, EPI_ISL_777652, EPI_ISL_777656, EPI_ISL_777661, EPI_ISL_777664 to 777666, EPI_ISL_777671, EPI_ISL_777673 to 777901, EPI_ISL_777903 to 778002, EPI_ISL_778005 to 778006, EPI_ISL_778010 to 778013, EPI_ISL_778015, EPI_ISL_778017, EPI_ISL_778019 to 778020, EPI_ISL_778022 to 778023, EPI_ISL_778025, EPI_ISL_778027 to 778028, EPI_ISL_778030, EPI_ISL_778032, EPI_ISL_778037 to 778039, EPI_ISL_778041, EPI_ISL_778045, EPI_ISL_778049, EPI_ISL_778054, EPI_ISL_778056 to 778057, EPI_ISL_778061, EPI_ISL_778063, EPI_ISL_778066 to 778067, EPI_ISL_778071 to 778072, EPI_ISL_778075, EPI_ISL_778078 to 778081, EPI_ISL_778084 to 778087, EPI_ISL_778091, EPI_ISL_778094 to 778097, EPI_ISL_778099 to 778100, EPI_ISL_778103, EPI_ISL_778105, EPI_ISL_778109 to 778110, EPI_ISL_778112, EPI_ISL_778114 to 778115, EPI_ISL_778118, EPI_ISL_778120, EPI_ISL_778123 to 778126, EPI_ISL_778130, EPI_ISL_778134 to 778135, EPI_ISL_778137 to 778139, EPI_ISL_778141 to 778143, EPI_ISL_778146 to 778148, EPI_ISL_778150, EPI_ISL_778152, EPI_ISL_778157 to 778158, EPI_ISL_778160 to 778162, EPI_ISL_778169 to 778175, EPI_ISL_778177 to 778182, EPI_ISL_778185 to 778186, EPI_ISL_778188 to 778189, EPI_ISL_778193 to 778194, EPI_ISL_778197 to 778200, EPI_ISL_778204 to 778206, EPI_ISL_778208 to 778211, EPI_ISL_778213 to 778214, EPI_ISL_778217 to 778218, EPI_ISL_778220 to 778224, EPI_ISL_778226 to 778227, EPI_ISL_778231 to 778232, EPI_ISL_778235, EPI_ISL_778237, EPI_ISL_778241 to 778243, EPI_ISL_778245, EPI_ISL_778251 to 778255, EPI_ISL_778257, EPI_ISL_778262, EPI_ISL_778265, EPI_ISL_778267 to 778268, EPI_ISL_778271, EPI_ISL_778273, EPI_ISL_778275, EPI_ISL_778277 to 778278, EPI_ISL_778280, EPI_ISL_778284 to 778285, EPI_ISL_778291, EPI_ISL_778293 to 778294, EPI_ISL_778296, EPI_ISL_781181, EPI_ISL_781183, EPI_ISL_781185, EPI_ISL_781187 to 781190, EPI_ISL_781192, EPI_ISL_781199, EPI_ISL_781220 to 781221, EPI_ISL_781223 to 781224, EPI_ISL_781248 to 781249, EPI_ISL_781251, EPI_ISL_781258, EPI_ISL_781260 to 781261, EPI_ISL_781263, EPI_ISL_781269, EPI_ISL_781279, EPI_ISL_781281, EPI_ISL_781287, EPI_ISL_781299, EPI_ISL_781304, EPI_ISL_781306, EPI_ISL_781308, EPI_ISL_781319, EPI_ISL_781325, EPI_ISL_781327, EPI_ISL_781332 to 781333, EPI_ISL_781340 to 781342, EPI_ISL_781345, EPI_ISL_781354 to 781355, EPI_ISL_781358 to 781360, EPI_ISL_781364, EPI_ISL_781367 to 781368, EPI_ISL_781371, EPI_ISL_781376 to 781377, EPI_ISL_781379, EPI_ISL_781385, EPI_ISL_781387, EPI_ISL_781392, EPI_ISL_781396, EPI_ISL_781401 to 781403, EPI_ISL_781407, EPI_ISL_781409, EPI_ISL_781413, EPI_ISL_781426, EPI_ISL_781431 to 781432, EPI_ISL_781434, EPI_ISL_781436, EPI_ISL_781438, EPI_ISL_781440 to 781442, EPI_ISL_781448, EPI_ISL_781466, EPI_ISL_781471 to 781472, EPI_ISL_781480 to 781481, EPI_ISL_781483, EPI_ISL_781488 to 781490, EPI_ISL_781494, EPI_ISL_781497 to 781498, EPI_ISL_781506, EPI_ISL_781512 to 781514, EPI_ISL_781517 to 781521, EPI_ISL_781523 to 781526, EPI_ISL_781528, EPI_ISL_781530 to 781535, EPI_ISL_781538, EPI_ISL_781540, EPI_ISL_781542 to 781543, EPI_ISL_781545 to 781546, EPI_ISL_781549 to 781553, EPI_ISL_781557, EPI_ISL_781559 to 781566, EPI_ISL_781568 to 781575, EPI_ISL_781577, EPI_ISL_781580 to 781581, EPI_ISL_781583 to 781596, EPI_ISL_781600 to 781604, EPI_ISL_781606 to 781608, EPI_ISL_781610, EPI_ISL_781612 to 781614, EPI_ISL_781616, EPI_ISL_781620, EPI_ISL_781622, EPI_ISL_781625 to 781626, EPI_ISL_781628 to 781631, EPI_ISL_781633 to 781635, EPI_ISL_781637 to 781648, EPI_ISL_781650, EPI_ISL_781653 to 781654, EPI_ISL_781656 to 781662, EPI_ISL_781664, EPI_ISL_781666 to 781668, EPI_ISL_781670 to 781673, EPI_ISL_781675 to 781676, EPI_ISL_781678 to 781679, EPI_ISL_781682 to 781683, EPI_ISL_781685, EPI_ISL_781687 to 781694, EPI_ISL_781696 to 781698, EPI_ISL_781700, EPI_ISL_781702 to 781719, EPI_ISL_781722 to 781723, EPI_ISL_781726 to 781729, EPI_ISL_781731, EPI_ISL_781733 to 781734, EPI_ISL_781736 to 781741, EPI_ISL_781744 to 781745, EPI_ISL_781748, EPI_ISL_781750 to 781752, EPI_ISL_781755 to 781762, EPI_ISL_781764 to 781766, EPI_ISL_781768 to 781769, EPI_ISL_781771 to 781772, EPI_ISL_781774 to 781776, EPI_ISL_781778 to 781782, EPI_ISL_781786 to 781787, EPI_ISL_781790 to 781794, EPI_ISL_781796 to 781797, EPI_ISL_781799 to 781800, EPI_ISL_781802 to 781806, EPI_ISL_781808 to 781831, EPI_ISL_781833, EPI_ISL_781835 to 781836, EPI_ISL_781838, EPI_ISL_781842 to 781850, EPI_ISL_781852 to 781853, EPI_ISL_781855 to 781857, EPI_ISL_781859 to 781861, EPI_ISL_781863, EPI_ISL_781865 to 781868, EPI_ISL_782739, EPI_ISL_782746, EPI_ISL_782757, EPI_ISL_782759, EPI_ISL_782761, EPI_ISL_782763 to 782764, EPI_ISL_782769, EPI_ISL_782776 to 782777, EPI_ISL_782780, EPI_ISL_782788, EPI_ISL_782796 to 782797, EPI_ISL_782800, EPI_ISL_782803, EPI_ISL_782809 to 782810, EPI_ISL_782814, EPI_ISL_782816 to 782818, EPI_ISL_782822, EPI_ISL_782830, EPI_ISL_782834, EPI_ISL_782840, EPI_ISL_782842, EPI_ISL_782846, EPI_ISL_782850, EPI_ISL_782853 to 782854, EPI_ISL_782857, EPI_ISL_782860, EPI_ISL_782862, EPI_ISL_782866 to 782868, EPI_ISL_782880, EPI_ISL_782882 to 782883, EPI_ISL_782885 to 782887, EPI_ISL_782889 to 782890, EPI_ISL_782907 to 782910, EPI_ISL_782912 to 782913, EPI_ISL_782920, EPI_ISL_782923, EPI_ISL_782928 to 782930, EPI_ISL_782932, EPI_ISL_782937, EPI_ISL_782941 to 782942, EPI_ISL_782947, EPI_ISL_782954, EPI_ISL_782959 to 782960, EPI_ISL_782966, EPI_ISL_782971, EPI_ISL_782974, EPI_ISL_782979, EPI_ISL_782983 to 782985, EPI_ISL_782987, EPI_ISL_782991, EPI_ISL_782996, EPI_ISL_782998, EPI_ISL_783012, EPI_ISL_783019, EPI_ISL_783023, EPI_ISL_783028, EPI_ISL_783031, EPI_ISL_783036 to 783038, EPI_ISL_783042, EPI_ISL_783044, EPI_ISL_783051, EPI_ISL_783055, EPI_ISL_783057, EPI_ISL_783066, EPI_ISL_783367 to 783535, EPI_ISL_797397 to 797398, EPI_ISL_797400 to 797401, EPI_ISL_797403 to 797404, EPI_ISL_797406 to 797407, EPI_ISL_797410, EPI_ISL_797412, EPI_ISL_797414 to 797415, EPI_ISL_797417 to 797419, EPI_ISL_797422 to 797423, EPI_ISL_797425 to 797427, EPI_ISL_797429, EPI_ISL_797431, EPI_ISL_797433 to 797434, EPI_ISL_797436 to 797437, EPI_ISL_797439 to 797440, EPI_ISL_797442 to 797444, EPI_ISL_797446 to 797450, EPI_ISL_797452 to 797455, EPI_ISL_797457 to 797458, EPI_ISL_797460, EPI_ISL_797463 to 797467, EPI_ISL_797469 to 797474, EPI_ISL_797476 to 797479, EPI_ISL_797481 to 797483, EPI_ISL_797485 to 797488, EPI_ISL_797490, EPI_ISL_797492 to 797498, EPI_ISL_797501 to 797503, EPI_ISL_797505 to 797508, EPI_ISL_797510 to 797511, EPI_ISL_797513, EPI_ISL_797515 to 797530, EPI_ISL_797532, EPI_ISL_797534, EPI_ISL_797536 to 797539, EPI_ISL_797542, EPI_ISL_797544 to 797546, EPI_ISL_797548, EPI_ISL_797550 to 797552, EPI_ISL_797555 to 797558, EPI_ISL_797561 to 797562, EPI_ISL_797565 to 797567, EPI_ISL_797569 to 797574, EPI_ISL_797577 to 797585, EPI_ISL_797588 to 797598, EPI_ISL_797600 to 797604, EPI_ISL_797607 to 797609, EPI_ISL_797611 to 797612, EPI_ISL_797614, EPI_ISL_797617 to 797618, EPI_ISL_797621 to 797626, EPI_ISL_797628 to 797631, EPI_ISL_797633 to 797634, EPI_ISL_797636, EPI_ISL_797638 to 797640, EPI_ISL_797643, EPI_ISL_797645 to 797653, EPI_ISL_797655 to 797659, EPI_ISL_797661 to 797662, EPI_ISL_797664 to 797665, EPI_ISL_797667 to 797672, EPI_ISL_797674 to 797676, EPI_ISL_797679 to 797684, EPI_ISL_797687 to 797698, EPI_ISL_797700, EPI_ISL_797702 to 797703, EPI_ISL_797705 to 797708, EPI_ISL_797710 to 797711, EPI_ISL_797718, EPI_ISL_798000, EPI_ISL_798003, EPI_ISL_798018, EPI_ISL_798032, EPI_ISL_798038, EPI_ISL_798040, EPI_ISL_798046, EPI_ISL_798048, EPI_ISL_798053, EPI_ISL_798055, EPI_ISL_798058, EPI_ISL_798060, EPI_ISL_798069, EPI_ISL_798072, EPI_ISL_798076 to 798079, EPI_ISL_798086, EPI_ISL_798088, EPI_ISL_798093 to 798095, EPI_ISL_798097, EPI_ISL_798105, EPI_ISL_798110 to 798113, EPI_ISL_798115, EPI_ISL_798117, EPI_ISL_798121, EPI_ISL_798126, EPI_ISL_798128, EPI_ISL_798130, EPI_ISL_798132, EPI_ISL_798137, EPI_ISL_798140, EPI_ISL_798143, EPI_ISL_798145, EPI_ISL_798148 to 798149, EPI_ISL_798151, EPI_ISL_798153, EPI_ISL_798160 to 798161, EPI_ISL_798166 to 798168, EPI_ISL_798183, EPI_ISL_798185 to 798186, EPI_ISL_798193, EPI_ISL_798197, EPI_ISL_798199, EPI_ISL_798201, EPI_ISL_798203, EPI_ISL_798211, EPI_ISL_798213 to 798214, EPI_ISL_798216 to 798218, EPI_ISL_798224, EPI_ISL_798230, EPI_ISL_798243 to 798244, EPI_ISL_798251 to 798254, EPI_ISL_798256, EPI_ISL_798258, EPI_ISL_798266, EPI_ISL_798270, EPI_ISL_798272 to 798273, EPI_ISL_798281, EPI_ISL_798288 to 798289, EPI_ISL_798294, EPI_ISL_798298, EPI_ISL_798300, EPI_ISL_798307, EPI_ISL_798311 to 798312, EPI_ISL_798315 to 798316, EPI_ISL_798319, EPI_ISL_798322, EPI_ISL_798326 to 798327, EPI_ISL_798664, EPI_ISL_798673 to 798674, EPI_ISL_798677 to 798679, EPI_ISL_798681 to 798683, EPI_ISL_798685 to 798687, EPI_ISL_798694, EPI_ISL_798697 to 798700, EPI_ISL_798703, EPI_ISL_798706, EPI_ISL_798708, EPI_ISL_798711, EPI_ISL_798713 to 798715, EPI_ISL_798717 to 798718, EPI_ISL_798723, EPI_ISL_798726, EPI_ISL_798730 to 798734, EPI_ISL_798736 to 798737, EPI_ISL_798743, EPI_ISL_798745 to 798748, EPI_ISL_798751, EPI_ISL_798753 to 798754, EPI_ISL_798756 to 798763, EPI_ISL_798765 to 798766, EPI_ISL_798772 to 798773, EPI_ISL_798775, EPI_ISL_798778, EPI_ISL_798780, EPI_ISL_798782 to 798783, EPI_ISL_798785 to 798790, EPI_ISL_798792, EPI_ISL_798794 to 798797, EPI_ISL_798799 to 798800, EPI_ISL_798802, EPI_ISL_798805 to 798807, EPI_ISL_798810, EPI_ISL_798813, EPI_ISL_798815 to 798816, EPI_ISL_798818, EPI_ISL_798820, EPI_ISL_798825, EPI_ISL_798827 to 798829, EPI_ISL_798831, EPI_ISL_798834 to 798836, EPI_ISL_798839 to 798843, EPI_ISL_798846 to 798849, EPI_ISL_798854, EPI_ISL_798857 to 798862, EPI_ISL_798865 to 798868, EPI_ISL_798871, EPI_ISL_798873, EPI_ISL_798877 to 798881, EPI_ISL_798883, EPI_ISL_798886 to 798887, EPI_ISL_798890, EPI_ISL_798893 to 798897, EPI_ISL_798899 to 798901, EPI_ISL_798903 to 798904, EPI_ISL_798907 to 798909, EPI_ISL_798912 to 798913, EPI_ISL_798916 to 798918, EPI_ISL_798920, EPI_ISL_798923, EPI_ISL_798927, EPI_ISL_798929, EPI_ISL_798931 to 798932, EPI_ISL_798937 to 798938, EPI_ISL_798940, EPI_ISL_798943 to 798947, EPI_ISL_798949, EPI_ISL_798953 to 798954, EPI_ISL_798956, EPI_ISL_798958 to 798960, EPI_ISL_798965, EPI_ISL_798971, EPI_ISL_798974, EPI_ISL_798976, EPI_ISL_798978, EPI_ISL_798980, EPI_ISL_798982, EPI_ISL_798985, EPI_ISL_798989 to 798990, EPI_ISL_798992 to 798993, EPI_ISL_798997 to 798999, EPI_ISL_799001 to 799002, EPI_ISL_799007 to 799008, EPI_ISL_799013, EPI_ISL_799017 to 799018, EPI_ISL_799020, EPI_ISL_799031 to 799032, EPI_ISL_799035 to 799037, EPI_ISL_799039, EPI_ISL_799048, EPI_ISL_799052, EPI_ISL_799058, EPI_ISL_799061, EPI_ISL_799074, EPI_ISL_799076, EPI_ISL_799078, EPI_ISL_799083, EPI_ISL_799087 to 799088, EPI_ISL_799091 to 799095, EPI_ISL_799097, EPI_ISL_799109 to 799110, EPI_ISL_799116 to 799117, EPI_ISL_799119, EPI_ISL_799121, EPI_ISL_799128, EPI_ISL_799133, EPI_ISL_799135 to 799136, EPI_ISL_799141 to 799142, EPI_ISL_799146, EPI_ISL_799148, EPI_ISL_799152 to 799153, EPI_ISL_799159, EPI_ISL_799168, EPI_ISL_799173 to 799174, EPI_ISL_799181, EPI_ISL_799184 to 799186, EPI_ISL_799195, EPI_ISL_799199, EPI_ISL_799206, EPI_ISL_799208, EPI_ISL_799210 to 799211, EPI_ISL_799217, EPI_ISL_799220, EPI_ISL_799223, EPI_ISL_799225 to 799226, EPI_ISL_799238 to 799239, EPI_ISL_799241 to 799242, EPI_ISL_799247, EPI_ISL_799251, EPI_ISL_799253 to 799254, EPI_ISL_799259, EPI_ISL_799266, EPI_ISL_799284, EPI_ISL_799291 to 799293, EPI_ISL_799295, EPI_ISL_799935 to 799936, EPI_ISL_799939, EPI_ISL_799944, EPI_ISL_799947, EPI_ISL_799950, EPI_ISL_799952 to 799953, EPI_ISL_799955, EPI_ISL_799957 to 799960, EPI_ISL_799962 to 799963, EPI_ISL_799971 to 799972, EPI_ISL_799974, EPI_ISL_799988, EPI_ISL_799993 to 799994, EPI_ISL_799996, EPI_ISL_800004, EPI_ISL_800006 to 800008, EPI_ISL_800012, EPI_ISL_800018, EPI_ISL_800024, EPI_ISL_800026, EPI_ISL_800028, EPI_ISL_800037, EPI_ISL_800045, EPI_ISL_800047, EPI_ISL_800054 to 800055, EPI_ISL_800065, EPI_ISL_800072, EPI_ISL_800077, EPI_ISL_800082 to 800083, EPI_ISL_800085, EPI_ISL_800088 to 800089, EPI_ISL_800092 to 800093, EPI_ISL_800095 to 800096, EPI_ISL_800098, EPI_ISL_800100, EPI_ISL_800102 to 800103, EPI_ISL_800114 to 800116, EPI_ISL_800119, EPI_ISL_800121, EPI_ISL_800123, EPI_ISL_800125, EPI_ISL_800133, EPI_ISL_800137, EPI_ISL_800139, EPI_ISL_800149, EPI_ISL_800153, EPI_ISL_800156 to 800157, EPI_ISL_800166 to 800168, EPI_ISL_800172 to 800173, EPI_ISL_800177 to 800178, EPI_ISL_800182, EPI_ISL_800192, EPI_ISL_800195 to 800196, EPI_ISL_800203, EPI_ISL_800211, EPI_ISL_800214, EPI_ISL_800218, EPI_ISL_800220, EPI_ISL_800225, EPI_ISL_800228, EPI_ISL_800233, EPI_ISL_800238, EPI_ISL_800244, EPI_ISL_800250, EPI_ISL_800252, EPI_ISL_800254 to 800261, EPI_ISL_800263 to 800266, EPI_ISL_800268 to 800270, EPI_ISL_800272 to 800273, EPI_ISL_800275 to 800279, EPI_ISL_800283 to 800285, EPI_ISL_800287 to 800298, EPI_ISL_800300, EPI_ISL_800302, EPI_ISL_800304 to 800307, EPI_ISL_800309 to 800316, EPI_ISL_800318 to 800337, EPI_ISL_800339, EPI_ISL_800341, EPI_ISL_800343 to 800346, EPI_ISL_800349, EPI_ISL_800352 to 800353, EPI_ISL_800356 to 800358, EPI_ISL_800361, EPI_ISL_800363 to 800364, EPI_ISL_800366, EPI_ISL_800368 to 800369, EPI_ISL_800371 to 800375, EPI_ISL_800377 to 800383, EPI_ISL_800385 to 800391, EPI_ISL_800393 to 800394, EPI_ISL_800396 to 800406, EPI_ISL_800408 to 800419, EPI_ISL_800421 to 800427, EPI_ISL_800429 to 800440, EPI_ISL_800442 to 800443, EPI_ISL_800445, EPI_ISL_800447 to 800451, EPI_ISL_800455, EPI_ISL_800458 to 800459, EPI_ISL_800461 to 800467, EPI_ISL_800469 to 800473, EPI_ISL_800475 to 800480, EPI_ISL_800482 to 800484, EPI_ISL_800486 to 800493, EPI_ISL_800496 to 800500, EPI_ISL_800503 to 800505, EPI_ISL_800507 to 800508, EPI_ISL_800510 to 800515, EPI_ISL_800517, EPI_ISL_800519 to 800523, EPI_ISL_800527, EPI_ISL_800529 to 800530, EPI_ISL_800532 to 800541, EPI_ISL_800543 to 800554, EPI_ISL_800556 to 800557, EPI_ISL_800559 to 800565, EPI_ISL_800567 to 800574, EPI_ISL_800586 to 800587, EPI_ISL_800589 to 800590, EPI_ISL_800592, EPI_ISL_800597, EPI_ISL_800600, EPI_ISL_800606, EPI_ISL_800609 to 800610, EPI_ISL_800615, EPI_ISL_800622, EPI_ISL_800626, EPI_ISL_800637, EPI_ISL_800641, EPI_ISL_800645, EPI_ISL_800647 to 800649, EPI_ISL_800658, EPI_ISL_800663, EPI_ISL_800665, EPI_ISL_800669, EPI_ISL_800672, EPI_ISL_800679 to 800680, EPI_ISL_800682 to 800683, EPI_ISL_800686 to 800687, EPI_ISL_800690, EPI_ISL_800702, EPI_ISL_800707 to 800709, EPI_ISL_800712, EPI_ISL_800714, EPI_ISL_800716 to 800717, EPI_ISL_800720 to 800721, EPI_ISL_800724, EPI_ISL_800730, EPI_ISL_800732 to 800734, EPI_ISL_800737, EPI_ISL_800744, EPI_ISL_800746, EPI_ISL_800755, EPI_ISL_800761, EPI_ISL_800763, EPI_ISL_800768, EPI_ISL_800770 to 800771, EPI_ISL_800776,

EPI_ISL_800784, EPI_ISL_800791 to 800793, EPI_ISL_800795, EPI_ISL_800798, EPI_ISL_800802 to 800803, EPI_ISL_800806, EPI_ISL_800808, EPI_ISL_800810, EPI_ISL_800816 to 800817, EPI_ISL_800819, EPI_ISL_800828, EPI_ISL_800832, EPI_ISL_800839, EPI_ISL_800842, EPI_ISL_800853 to 800855, EPI_ISL_800859, EPI_ISL_800865, EPI_ISL_800870, EPI_ISL_800872 to 800873, EPI_ISL_800876, EPI_ISL_800883, EPI_ISL_800885 to 801178, EPI_ISL_811457 to 811468, EPI_ISL_811470 to 811471, EPI_ISL_811473 to 811477, EPI_ISL_811479 to 811481, EPI_ISL_811483, EPI_ISL_811485 to 811486, EPI_ISL_811488 to 811489, EPI_ISL_811491 to 811493, EPI_ISL_811495 to 811497, EPI_ISL_811499, EPI_ISL_811501, EPI_ISL_811503 to 811510, EPI_ISL_811512 to 811515, EPI_ISL_811517 to 811518, EPI_ISL_811520 to 811523, EPI_ISL_811526 to 811529, EPI_ISL_811531 to 811537, EPI_ISL_811539 to 811540, EPI_ISL_811542 to 811544, EPI_ISL_811546 to 811547, EPI_ISL_811550 to 811555, EPI_ISL_811557 to 811558, EPI_ISL_811560 to 811562, EPI_ISL_811564, EPI_ISL_811566 to 811568, EPI_ISL_811570 to 811572, EPI_ISL_811574 to 811579, EPI_ISL_811581 to 811583, EPI_ISL_811585 to 811590, EPI_ISL_811592 to 811593, EPI_ISL_811595 to 811602, EPI_ISL_811604 to 811605, EPI_ISL_811607 to 811609, EPI_ISL_811612 to 811616, EPI_ISL_811619, EPI_ISL_811623 to 811624, EPI_ISL_811628 to 811633, EPI_ISL_811635 to 811643, EPI_ISL_811646 to 811649, EPI_ISL_811652 to 811656, EPI_ISL_811658 to 811659, EPI_ISL_811662 to 811663, EPI_ISL_811665, EPI_ISL_811667 to 811668, EPI_ISL_811674 to 811676, EPI_ISL_811679 to 811681, EPI_ISL_811683 to 811687, EPI_ISL_811689 to 811695, EPI_ISL_811697, EPI_ISL_811699 to 811700, EPI_ISL_811704 to 811707, EPI_ISL_811709 to 811719, EPI_ISL_811721 to 811725, EPI_ISL_811728 to 811735, EPI_ISL_811737 to 811739, EPI_ISL_811742 to 811749, EPI_ISL_811751 to 811752, EPI_ISL_811755, EPI_ISL_811757 to 811758, EPI_ISL_811760 to 811761, EPI_ISL_811763 to 811766, EPI_ISL_811768 to 811775, EPI_ISL_811777 to 811784, EPI_ISL_811786, EPI_ISL_811788 to 811801, EPI_ISL_811803 to 811806, EPI_ISL_821276, EPI_ISL_821278 to 821279, EPI_ISL_821284, EPI_ISL_821286, EPI_ISL_821289 to 821290, EPI_ISL_821295, EPI_ISL_821309 to 821311, EPI_ISL_821316, EPI_ISL_821320, EPI_ISL_821333, EPI_ISL_821338 to 821339, EPI_ISL_821342 to 821343, EPI_ISL_821348, EPI_ISL_821354, EPI_ISL_821360, EPI_ISL_821363, EPI_ISL_821365, EPI_ISL_821367 to 821368, EPI_ISL_821374 to 821376, EPI_ISL_821386, EPI_ISL_821392 to 821394, EPI_ISL_821399, EPI_ISL_821404, EPI_ISL_821408, EPI_ISL_821411, EPI_ISL_821416, EPI_ISL_821421, EPI_ISL_821427, EPI_ISL_821435, EPI_ISL_821439, EPI_ISL_821441 to 821442, EPI_ISL_821444, EPI_ISL_821447, EPI_ISL_821454, EPI_ISL_821463, EPI_ISL_821473, EPI_ISL_821479, EPI_ISL_821484 to 821485, EPI_ISL_821488, EPI_ISL_821491 to 821492, EPI_ISL_821506, EPI_ISL_821510, EPI_ISL_821513, EPI_ISL_821516, EPI_ISL_821519, EPI_ISL_821523, EPI_ISL_821529, EPI_ISL_821544, EPI_ISL_821546 to 821547, EPI_ISL_821549, EPI_ISL_821555, EPI_ISL_821563 to 821565, EPI_ISL_821568, EPI_ISL_821570, EPI_ISL_821576 to 821577, EPI_ISL_821579, EPI_ISL_821583, EPI_ISL_821588, EPI_ISL_821590, EPI_ISL_821592 to 821593, EPI_ISL_821596, EPI_ISL_821602, EPI_ISL_821604 to 821605, EPI_ISL_821608, EPI_ISL_821613, EPI_ISL_821616, EPI_ISL_821618, EPI_ISL_821620, EPI_ISL_821622 to 821624, EPI_ISL_821661 to 821970, EPI_ISL_822299, EPI_ISL_822301, EPI_ISL_822310 to 822316, EPI_ISL_822322, EPI_ISL_826473, EPI_ISL_826475, EPI_ISL_826518, EPI_ISL_833585, EPI_ISL_833587 to 833589, EPI_ISL_833591 to 833592, EPI_ISL_833594 to 833595, EPI_ISL_833598, EPI_ISL_833600 to 833601, EPI_ISL_833603, EPI_ISL_833605, EPI_ISL_833608 to 833610, EPI_ISL_833613, EPI_ISL_833616, EPI_ISL_833622 to 833628, EPI_ISL_833630 to 833631, EPI_ISL_833633, EPI_ISL_833635 to 833642, EPI_ISL_833644 to 833646, EPI_ISL_833649 to 833651, EPI_ISL_833653 to 833655, EPI_ISL_833657, EPI_ISL_833659 to 833666, EPI_ISL_833668 to 833675, EPI_ISL_833677, EPI_ISL_833679 to 833684, EPI_ISL_833686 to 833692, EPI_ISL_833694 to 833699, EPI_ISL_833701 to 833711, EPI_ISL_833713 to 833715, EPI_ISL_833717 to 833718, EPI_ISL_833720, EPI_ISL_833722 to 833724, EPI_ISL_833727 to 833733, EPI_ISL_833737, EPI_ISL_833739 to 833742, EPI_ISL_833744 to 833754, EPI_ISL_833757 to 833758, EPI_ISL_833760 to 833765, EPI_ISL_833769 to 833778, EPI_ISL_833781 to 833788, EPI_ISL_833790, EPI_ISL_833792 to 833794, EPI_ISL_833798 to 833800, EPI_ISL_833802, EPI_ISL_833805 to 833808, EPI_ISL_833810 to 833813, EPI_ISL_833815 to 833816, EPI_ISL_833819 to 833820, EPI_ISL_833822, EPI_ISL_833824 to 833829, EPI_ISL_833831 to 833833, EPI_ISL_833836 to 833840, EPI_ISL_833842 to 833844, EPI_ISL_833847, EPI_ISL_833849 to 833855, EPI_ISL_833857 to 833860, EPI_ISL_833862 to 833864, EPI_ISL_833866 to 833867, EPI_ISL_833869 to 833871, EPI_ISL_833873 to 833878, EPI_ISL_833880, EPI_ISL_833882 to 833883, EPI_ISL_833885 to 833888, EPI_ISL_833891 to 833893, EPI_ISL_833896 to 833900, EPI_ISL_833902 to 833907, EPI_ISL_833909 to 833915, EPI_ISL_833917 to 833921, EPI_ISL_833923, EPI_ISL_833925 to 833929, EPI_ISL_833932, EPI_ISL_836421 to 836747, EPI_ISL_845893 to 846199, EPI_ISL_846509, EPI_ISL_851062 to 851069, EPI_ISL_851071 to 851076, EPI_ISL_851078 to 851080, EPI_ISL_851083 to 851084, EPI_ISL_851086 to 851093, EPI_ISL_851096 to 851100, EPI_ISL_851103, EPI_ISL_851105 to 851106, EPI_ISL_851108 to 851115, EPI_ISL_851117 to 851118, EPI_ISL_851120 to 851123, EPI_ISL_851127 to 851129, EPI_ISL_851131 to 851133, EPI_ISL_851136, EPI_ISL_851138 to 851141, EPI_ISL_851143 to 851145, EPI_ISL_851149 to 851153, EPI_ISL_851155 to 851166, EPI_ISL_851168 to 851177, EPI_ISL_851179, EPI_ISL_851181, EPI_ISL_851183 to 851186, EPI_ISL_851188 to 851189, EPI_ISL_851191 to 851195, EPI_ISL_851197 to 851205, EPI_ISL_851208 to 851214, EPI_ISL_851216, EPI_ISL_851219 to 851224, EPI_ISL_851226 to 851228, EPI_ISL_851230 to 851235, EPI_ISL_851238 to 851240, EPI_ISL_851242 to 851245, EPI_ISL_851247 to 851251, EPI_ISL_851254 to 851256, EPI_ISL_851260 to 851263, EPI_ISL_851265 to 851269, EPI_ISL_851271 to 851275, EPI_ISL_851277 to 851284, EPI_ISL_851286 to 851295, EPI_ISL_851297 to 851300, EPI_ISL_851302 to 851304, EPI_ISL_851308 to 851309, EPI_ISL_851311 to 851324, EPI_ISL_851326, EPI_ISL_851328, EPI_ISL_851330, EPI_ISL_851332 to 851334, EPI_ISL_851336 to 851340, EPI_ISL_851342 to 851345, EPI_ISL_851347 to 851350, EPI_ISL_851352 to 851357, EPI_ISL_851359 to 851364, EPI_ISL_851366, EPI_ISL_851368 to 851370, EPI_ISL_851372 to 851373, EPI_ISL_851375 to 851379, EPI_ISL_851381, EPI_ISL_857542 to 857869, EPI_ISL_859554 to 859560, EPI_ISL_873282 to 873283, EPI_ISL_873286, EPI_ISL_873291 to 873292, EPI_ISL_873295, EPI_ISL_873297, EPI_ISL_873302, EPI_ISL_873304, EPI_ISL_873306, EPI_ISL_873310, EPI_ISL_873313, EPI_ISL_873316, EPI_ISL_873318, EPI_ISL_873320, EPI_ISL_873324 to 873326, EPI_ISL_873334, EPI_ISL_873337, EPI_ISL_873339, EPI_ISL_873341 to 873343, EPI_ISL_873347 to 873348, EPI_ISL_873350, EPI_ISL_873357, EPI_ISL_873359, EPI_ISL_873362, EPI_ISL_873364 to 873365, EPI_ISL_873368 to 873371, EPI_ISL_873374, EPI_ISL_873381, EPI_ISL_873388, EPI_ISL_873399, EPI_ISL_873412, EPI_ISL_873416, EPI_ISL_873418, EPI_ISL_873422, EPI_ISL_873426, EPI_ISL_873428, EPI_ISL_873431 to 873433, EPI_ISL_873443, EPI_ISL_873451, EPI_ISL_873454, EPI_ISL_873457, EPI_ISL_873459, EPI_ISL_873476, EPI_ISL_873481 to 873482, EPI_ISL_873489, EPI_ISL_873492, EPI_ISL_873494 to 873495, EPI_ISL_873497, EPI_ISL_873499 to 873501, EPI_ISL_873510, EPI_ISL_873513, EPI_ISL_873518, EPI_ISL_873532, EPI_ISL_873537, EPI_ISL_873552, EPI_ISL_873561, EPI_ISL_873566, EPI_ISL_873571, EPI_ISL_873573 to 873574, EPI_ISL_873576, EPI_ISL_873605, EPI_ISL_873611, EPI_ISL_873613 to 873614, EPI_ISL_873621, EPI_ISL_873624 to 873625, EPI_ISL_873629 to 873630, EPI_ISL_873632 to 873636, EPI_ISL_873638 to 873640, EPI_ISL_873642 to 873644, EPI_ISL_873646 to 873651, EPI_ISL_873653, EPI_ISL_873655, EPI_ISL_873658 to 873659, EPI_ISL_873661 to 873668, EPI_ISL_873670 to 873671, EPI_ISL_873673 to 873678, EPI_ISL_873680 to 873683, EPI_ISL_873685 to 873703, EPI_ISL_873705 to 873715, EPI_ISL_873717 to 873723, EPI_ISL_873727 to 873728, EPI_ISL_873731 to 873733, EPI_ISL_873735 to 873740, EPI_ISL_873742, EPI_ISL_873745 to 873747, EPI_ISL_873750 to 873754, EPI_ISL_873756 to 873759, EPI_ISL_873761 to 873763, EPI_ISL_873765 to 873768, EPI_ISL_873770 to 873782, EPI_ISL_873784 to 873785, EPI_ISL_873787 to 873793, EPI_ISL_873795 to 873796, EPI_ISL_873798 to 873805, EPI_ISL_873807 to 873808, EPI_ISL_873810 to 873811, EPI_ISL_873813 to 873816, EPI_ISL_873818 to 873821, EPI_ISL_873823 to 873826, EPI_ISL_873828, EPI_ISL_873830 to 873832, EPI_ISL_873835 to 873839, EPI_ISL_873841, EPI_ISL_873843 to 873846, EPI_ISL_873849, EPI_ISL_873851 to 873852, EPI_ISL_873854 to 873856, EPI_ISL_873858, EPI_ISL_873861, EPI_ISL_873863 to 873864, EPI_ISL_873866 to 873869, EPI_ISL_873871 to 873872, EPI_ISL_873874, EPI_ISL_873876 to 873879, EPI_ISL_873882 to 873883, EPI_ISL_873885, EPI_ISL_873888 to 873899, EPI_ISL_873901 to 873903, EPI_ISL_873906 to 873909, EPI_ISL_873911 to 873912, EPI_ISL_873914 to 873915, EPI_ISL_873917 to 873920, EPI_ISL_873922, EPI_ISL_873927, EPI_ISL_873929 to 873933, EPI_ISL_873936, EPI_ISL_873938 to 873941, EPI_ISL_873944 to 873946, EPI_ISL_873949, EPI_ISL_873951 to 873953, EPI_ISL_873955, EPI_ISL_873957 to 873959, EPI_ISL_878728, EPI_ISL_878731, EPI_ISL_878733, EPI_ISL_878735, EPI_ISL_878737 to 878738, EPI_ISL_878740, EPI_ISL_878743, EPI_ISL_878745, EPI_ISL_878748 to 878749, EPI_ISL_878753, EPI_ISL_878758, EPI_ISL_878761 to 878762, EPI_ISL_878764, EPI_ISL_878766 to 878767, EPI_ISL_878770, EPI_ISL_878774, EPI_ISL_878783, EPI_ISL_878787 to 878788, EPI_ISL_878795 to 878796, EPI_ISL_878799, EPI_ISL_878801 to 878803, EPI_ISL_878810 to 878811, EPI_ISL_878813, EPI_ISL_878815 to 878816, EPI_ISL_878818, EPI_ISL_878821, EPI_ISL_878823, EPI_ISL_878826 to 878832, EPI_ISL_878834, EPI_ISL_878837 to 878839, EPI_ISL_878841, EPI_ISL_878847, EPI_ISL_878850, EPI_ISL_878855, EPI_ISL_878857, EPI_ISL_878862 to 878864, EPI_ISL_878867, EPI_ISL_878869 to 878873, EPI_ISL_878877, EPI_ISL_878881, EPI_ISL_878885, EPI_ISL_878889 to 878890, EPI_ISL_878893 to 878895, EPI_ISL_878897 to 878901, EPI_ISL_878903 to 878905, EPI_ISL_878907 to 878908, EPI_ISL_878910 to 878912, EPI_ISL_878916, EPI_ISL_878921 to 878924, EPI_ISL_878928, EPI_ISL_878930 to 878931, EPI_ISL_878933, EPI_ISL_878935, EPI_ISL_878937, EPI_ISL_878939 to 878940, EPI_ISL_878948 to 878950, EPI_ISL_878953 to 878954, EPI_ISL_878956 to 878957, EPI_ISL_878963 to 878964, EPI_ISL_878968 to 878969, EPI_ISL_878973 to 878978, EPI_ISL_878980, EPI_ISL_878984, EPI_ISL_878986 to 878989, EPI_ISL_878991, EPI_ISL_878995 to 878998, EPI_ISL_879002, EPI_ISL_879004 to 879006, EPI_ISL_879010 to 879012, EPI_ISL_879017, EPI_ISL_879020, EPI_ISL_879022, EPI_ISL_879024, EPI_ISL_879027, EPI_ISL_879029 to 879031, EPI_ISL_879035 to 879036, EPI_ISL_879039, EPI_ISL_879041, EPI_ISL_879045 to 879046, EPI_ISL_879048, EPI_ISL_879051, EPI_ISL_879053 to 879054, EPI_ISL_879058 to 879060, EPI_ISL_879062, EPI_ISL_879067 to 879069, EPI_ISL_879071, EPI_ISL_879074, EPI_ISL_879076, EPI_ISL_879078, EPI_ISL_879080, EPI_ISL_879082, EPI_ISL_879084 to 879085, EPI_ISL_879090, EPI_ISL_879095, EPI_ISL_879102, EPI_ISL_879104 to 879107, EPI_ISL_879112, EPI_ISL_879115, EPI_ISL_879119, EPI_ISL_879122 to 879123, EPI_ISL_879127, EPI_ISL_879133, EPI_ISL_879135 to 879136, EPI_ISL_879139, EPI_ISL_879141 to 879143, EPI_ISL_879148, EPI_ISL_879150, EPI_ISL_879153, EPI_ISL_879155 to 879156, EPI_ISL_879158, EPI_ISL_879163, EPI_ISL_879165 to 879166, EPI_ISL_879168, EPI_ISL_879170, EPI_ISL_879172, EPI_ISL_879174 to 879180, EPI_ISL_879182 to 879184, EPI_ISL_879186, EPI_ISL_879189 to 879190, EPI_ISL_879192, EPI_ISL_879196 to 879198, EPI_ISL_879200, EPI_ISL_879204 to 879205, EPI_ISL_879208 to 879211, EPI_ISL_879213, EPI_ISL_879215 to 879217, EPI_ISL_879221 to 879223, EPI_ISL_879228, EPI_ISL_879232 to 879233, EPI_ISL_879236, EPI_ISL_879238, EPI_ISL_879240, EPI_ISL_879242, EPI_ISL_879245 to 879246, EPI_ISL_879251, EPI_ISL_879255, EPI_ISL_879257, EPI_ISL_879260 to 879261, EPI_ISL_879264, EPI_ISL_879267, EPI_ISL_879269, EPI_ISL_879272, EPI_ISL_879275, EPI_ISL_879280 to 879283, EPI_ISL_879285, EPI_ISL_879287 to 879289, EPI_ISL_879291 to 879295, EPI_ISL_879301, EPI_ISL_879304, EPI_ISL_879309 to 879312, EPI_ISL_879315, EPI_ISL_879317, EPI_ISL_879319 to 879321, EPI_ISL_879325 to 879328, EPI_ISL_879330, EPI_ISL_879332, EPI_ISL_879334 to 879337, EPI_ISL_879349 to 879350, EPI_ISL_879352 to 879353, EPI_ISL_879355, EPI_ISL_879364 to 879366, EPI_ISL_879368 to 879370, EPI_ISL_879372 to 879373, EPI_ISL_879376, EPI_ISL_879381, EPI_ISL_879383, EPI_ISL_879385, EPI_ISL_879387, EPI_ISL_879389 to 879391, EPI_ISL_879394, EPI_ISL_879398, EPI_ISL_879400 to 879403, EPI_ISL_879405 to 879408, EPI_ISL_879410 to 879411, EPI_ISL_879414, EPI_ISL_879419, EPI_ISL_879421 to 879423, EPI_ISL_879428, EPI_ISL_879430 to 879432, EPI_ISL_879435 to 879441, EPI_ISL_879443, EPI_ISL_879445, EPI_ISL_879447, EPI_ISL_879453 to 879454, EPI_ISL_879463, EPI_ISL_879465, EPI_ISL_879468 to 879470, EPI_ISL_879472, EPI_ISL_879474, EPI_ISL_879478, EPI_ISL_879481 to 879482, EPI_ISL_879484 to 879485, EPI_ISL_879488 to 879491, EPI_ISL_879494, EPI_ISL_879496, EPI_ISL_879498, EPI_ISL_879500, EPI_ISL_879503, EPI_ISL_879506 to 879509, EPI_ISL_879511 to 879513, EPI_ISL_879516 to 879517, EPI_ISL_879521, EPI_ISL_879523, EPI_ISL_879525, EPI_ISL_879527 to 879531, EPI_ISL_879533, EPI_ISL_879536 to 879537, EPI_ISL_879541, EPI_ISL_879543 to 879545, EPI_ISL_879551, EPI_ISL_879555, EPI_ISL_879557 to 879559, EPI_ISL_879562, EPI_ISL_879567 to 879570, EPI_ISL_879573 to 879574, EPI_ISL_879577, EPI_ISL_879579 to 879580, EPI_ISL_879583 to 879584, EPI_ISL_879587 to 879588, EPI_ISL_879590, EPI_ISL_879595, EPI_ISL_879598 to 879599, EPI_ISL_879605 to 879607, EPI_ISL_879609 to 879610, EPI_ISL_879612, EPI_ISL_879615, EPI_ISL_879620, EPI_ISL_879622, EPI_ISL_879624 to 879626, EPI_ISL_879630, EPI_ISL_879632 to 879640, EPI_ISL_879644 to 879645, EPI_ISL_879648, EPI_ISL_879650 to 879652, EPI_ISL_879657 to 879662, EPI_ISL_879667 to 879671, EPI_ISL_879673, EPI_ISL_879676 to 879677, EPI_ISL_879680, EPI_ISL_879684 to 879685, EPI_ISL_879688, EPI_ISL_879690 to 879691, EPI_ISL_879694, EPI_ISL_879696 to 879699, EPI_ISL_879701, EPI_ISL_879704 to 879706, EPI_ISL_879708 to 879709, EPI_ISL_879716, EPI_ISL_879720, EPI_ISL_879722 to 879725, EPI_ISL_879727, EPI_ISL_879729 to 879735, EPI_ISL_879738 to 879739, EPI_ISL_879741, EPI_ISL_879743, EPI_ISL_879745 to 879746, EPI_ISL_879749, EPI_ISL_879754, EPI_ISL_879763 to 879764, EPI_ISL_879768 to 879774, EPI_ISL_879776 to 879777, EPI_ISL_879779, EPI_ISL_879781 to 879782, EPI_ISL_879786, EPI_ISL_879788, EPI_ISL_879790, EPI_ISL_879795 to 879799, EPI_ISL_879804 to 879807, EPI_ISL_879809, EPI_ISL_879812, EPI_ISL_879815 to 879816, EPI_ISL_879818, EPI_ISL_879820, EPI_ISL_879823 to 879824, EPI_ISL_879828, EPI_ISL_879830, EPI_ISL_879832 to 879835, EPI_ISL_879839 to 879840, EPI_ISL_879845, EPI_ISL_879847 to 879848, EPI_ISL_879850, EPI_ISL_879852 to 879853, EPI_ISL_879857, EPI_ISL_879860 to 879862, EPI_ISL_879864, EPI_ISL_879867, EPI_ISL_879874, EPI_ISL_879876 to 879877, EPI_ISL_879880 to 879881, EPI_ISL_879883, EPI_ISL_879889 to 879893, EPI_ISL_879896, EPI_ISL_879899 to 879900, EPI_ISL_879902, EPI_ISL_879904, EPI_ISL_879908 to 879911, EPI_ISL_879917, EPI_ISL_879920 to 879921, EPI_ISL_879928, EPI_ISL_879930, EPI_ISL_879935, EPI_ISL_879940 to 879941, EPI_ISL_879943 to 879944, EPI_ISL_879946, EPI_ISL_879951 to 879952, EPI_ISL_879954, EPI_ISL_879958, EPI_ISL_879960, EPI_ISL_879962 to 879963, EPI_ISL_879973, EPI_ISL_879983, EPI_ISL_879989, EPI_ISL_879992, EPI_ISL_879994, EPI_ISL_879996, EPI_ISL_880001, EPI_ISL_880004 to 880005, EPI_ISL_880012 to 880013, EPI_ISL_880015, EPI_ISL_880020, EPI_ISL_880025 to 880026, EPI_ISL_880033, EPI_ISL_880035, EPI_ISL_880038 to 880039, EPI_ISL_880044, EPI_ISL_880046 to 880047, EPI_ISL_880049, EPI_ISL_880051, EPI_ISL_880054, EPI_ISL_880059 to 880060, EPI_ISL_880065, EPI_ISL_880068, EPI_ISL_880070, EPI_ISL_880073, EPI_ISL_880075 to 880076, EPI_ISL_880078, EPI_ISL_880080 to 880081, EPI_ISL_881619 to 881620, EPI_ISL_881622, EPI_ISL_891273 to 891277, EPI_ISL_891282, EPI_ISL_891284, EPI_ISL_891287, EPI_ISL_891289 to 891294, EPI_ISL_891296, EPI_ISL_891300 to 891303, EPI_ISL_891306 to 891310, EPI_ISL_891314, EPI_ISL_891316, EPI_ISL_891321 to 891322, EPI_ISL_891325, EPI_ISL_891328 to 891329, EPI_ISL_891331 to 891333, EPI_ISL_891336, EPI_ISL_891339, EPI_ISL_891341, EPI_ISL_891343, EPI_ISL_891346 to 891348, EPI_ISL_891350 to 891351, EPI_ISL_891354 to 891356, EPI_ISL_891359, EPI_ISL_891362 to 891363, EPI_ISL_891366 to 891370, EPI_ISL_891373, EPI_ISL_891379, EPI_ISL_891381, EPI_ISL_891387 to 891390, EPI_ISL_891394 to 891395, EPI_ISL_891397 to 891399, EPI_ISL_891406 to 891409, EPI_ISL_891412 to 891414, EPI_ISL_891418, EPI_ISL_891422, EPI_ISL_891425, EPI_ISL_891427 to 891429, EPI_ISL_891431 to 891434, EPI_ISL_891436 to 891437, EPI_ISL_891439, EPI_ISL_891441 to 891443, EPI_ISL_891447, EPI_ISL_891449 to 891450, EPI_ISL_891452, EPI_ISL_891457, EPI_ISL_891461, EPI_ISL_891466 to 891468, EPI_ISL_891470 to 891472, EPI_ISL_891474 to 891477, EPI_ISL_891480 to 891483, EPI_ISL_891486, EPI_ISL_891488, EPI_ISL_891492 to 891494, EPI_ISL_891498, EPI_ISL_891500 to 891501, EPI_ISL_891504, EPI_ISL_891506, EPI_ISL_891510, EPI_ISL_891513, EPI_ISL_891515, EPI_ISL_891517 to 891519, EPI_ISL_891521, EPI_ISL_891523 to 891524, EPI_ISL_891526, EPI_ISL_891530, EPI_ISL_891533 to 891535, EPI_ISL_891537, EPI_ISL_891540 to 891541, EPI_ISL_891543, EPI_ISL_891546, EPI_ISL_891550 to 891554, EPI_ISL_891556 to 891558, EPI_ISL_891561, EPI_ISL_891565 to 891567, EPI_ISL_891570, EPI_ISL_891573, EPI_ISL_891575 to 891581, EPI_ISL_891583 to 891584, EPI_ISL_891589 to 891595, EPI_ISL_891597 to 891598, EPI_ISL_891601, EPI_ISL_891607 to 891608, EPI_ISL_891614, EPI_ISL_891617, EPI_ISL_891619 to 891621, EPI_ISL_891627, EPI_ISL_891631 to 891632, EPI_ISL_891635, EPI_ISL_891637 to 891638, EPI_ISL_891641 to 891642, EPI_ISL_891644 to 891646, EPI_ISL_891648 to 891649, EPI_ISL_891651 to 891655, EPI_ISL_891657, EPI_ISL_891660, EPI_ISL_891663, EPI_ISL_891665 to 891667, EPI_ISL_891669, EPI_ISL_891671, EPI_ISL_891674 to 891676, EPI_ISL_891678 to 891679, EPI_ISL_891682, EPI_ISL_891684, EPI_ISL_891686, EPI_ISL_891688, EPI_ISL_891690 to 891693, EPI_ISL_891695 to 891699, EPI_ISL_891701 to 891703, EPI_ISL_891706 to 891709, EPI_ISL_891712, EPI_ISL_891715 to 891716, EPI_ISL_891720, EPI_ISL_891728, EPI_ISL_891730 to 891734, EPI_ISL_891737, EPI_ISL_891739, EPI_ISL_891743 to 891745, EPI_ISL_891748, EPI_ISL_891750, EPI_ISL_891752, EPI_ISL_891754 to 891756, EPI_ISL_891758 to 891759, EPI_ISL_891763 to 891764, EPI_ISL_891767 to 891768, EPI_ISL_891770, EPI_ISL_891778 to 891779, EPI_ISL_891781 to 891785, EPI_ISL_891792, EPI_ISL_891795 to 891801, EPI_ISL_891803, EPI_ISL_891805 to 891806, EPI_ISL_891808, EPI_ISL_891810, EPI_ISL_891812, EPI_ISL_891814 to 891818, EPI_ISL_891820, EPI_ISL_891825, EPI_ISL_891827, EPI_ISL_891829 to 891832, EPI_ISL_891835, EPI_ISL_891837, EPI_ISL_891840 to 891842, EPI_ISL_891844, EPI_ISL_891849, EPI_ISL_891854, EPI_ISL_891859, EPI_ISL_891862, EPI_ISL_891868, EPI_ISL_891871 to 891872, EPI_ISL_891874, EPI_ISL_891876 to 891877, EPI_ISL_891879 to 891880, EPI_ISL_891882 to 891883, EPI_ISL_891886, EPI_ISL_891888 to 891894, EPI_ISL_891896 to 891898, EPI_ISL_892225, EPI_ISL_907119, EPI_ISL_907133, EPI_ISL_907136, EPI_ISL_907139, EPI_ISL_907144, EPI_ISL_907147 to 907148, EPI_ISL_907152, EPI_ISL_907155, EPI_ISL_907160, EPI_ISL_907163 to 907164, EPI_ISL_907167 to 907168, EPI_ISL_907173, EPI_ISL_907179, EPI_ISL_907182, EPI_ISL_907186 to 907187, EPI_ISL_907190, EPI_ISL_907192 to 907195, EPI_ISL_907200, EPI_ISL_907206, EPI_ISL_907209, EPI_ISL_907212, EPI_ISL_907214, EPI_ISL_907219, EPI_ISL_907221 to 907225, EPI_ISL_907238, EPI_ISL_907241, EPI_ISL_907250, EPI_ISL_907254, EPI_ISL_907256, EPI_ISL_907260, EPI_ISL_907263 to 907264, EPI_ISL_907267, EPI_ISL_907269, EPI_ISL_907273, EPI_ISL_907279, EPI_ISL_907281, EPI_ISL_907284, EPI_ISL_907287 to 907288, EPI_ISL_907292, EPI_ISL_907300, EPI_ISL_907303 to 907304, EPI_ISL_907308, EPI_ISL_907315, EPI_ISL_907318, EPI_ISL_907321 to 907323, EPI_ISL_907325, EPI_ISL_907328, EPI_ISL_907336, EPI_ISL_907341, EPI_ISL_907347 to 907348, EPI_ISL_907350 to 907353, EPI_ISL_907355, EPI_ISL_907359, EPI_ISL_907362 to 907364, EPI_ISL_907367, EPI_ISL_907369, EPI_ISL_908035, EPI_ISL_908037 to 908050, EPI_ISL_908052 to 908053, EPI_ISL_908055, EPI_ISL_908057 to 908058, EPI_ISL_908062 to 908063, EPI_ISL_908065 to 908069, EPI_ISL_908071, EPI_ISL_908076 to 908080, EPI_ISL_908082 to 908083, EPI_ISL_908088, EPI_ISL_908090 to 908091, EPI_ISL_908093, EPI_ISL_908095, EPI_ISL_908098 to 908103, EPI_ISL_908105 to 908112, EPI_ISL_908114 to 908115, EPI_ISL_908117 to 908118, EPI_ISL_908121, EPI_ISL_908123 to 908125, EPI_ISL_908127 to 908130, EPI_ISL_908132 to 908138, EPI_ISL_908140 to 908150, EPI_ISL_908153 to 908167, EPI_ISL_908169 to 908170, EPI_ISL_908172 to 908173, EPI_ISL_908175 to 908176, EPI_ISL_908178, EPI_ISL_908180, EPI_ISL_908182, EPI_ISL_908184 to 908186, EPI_ISL_908188 to 908193, EPI_ISL_908195 to 908199, EPI_ISL_908201 to 908205, EPI_ISL_908207, EPI_ISL_908209 to 908213, EPI_ISL_908215 to 908217, EPI_ISL_908219 to 908228, EPI_ISL_908230, EPI_ISL_908232 to 908236, EPI_ISL_908239 to 908240, EPI_ISL_908244 to 908251, EPI_ISL_908253, EPI_ISL_908255, EPI_ISL_908257 to 908259, EPI_ISL_908264, EPI_ISL_908267, EPI_ISL_908597 to 908931, EPI_ISL_908933, EPI_ISL_908935, EPI_ISL_908942, EPI_ISL_908946, EPI_ISL_908949, EPI_ISL_908951 to 908952, EPI_ISL_908955, EPI_ISL_908957, EPI_ISL_908959, EPI_ISL_908961, EPI_ISL_908963, EPI_ISL_908967 to 908968, EPI_ISL_908977 to 908979, EPI_ISL_908983, EPI_ISL_908985, EPI_ISL_908990 to 908992, EPI_ISL_908998 to 908999, EPI_ISL_909006 to 909007, EPI_ISL_909009, EPI_ISL_909011, EPI_ISL_909015, EPI_ISL_909019, EPI_ISL_909035 to 909036, EPI_ISL_909040, EPI_ISL_909046 to 909050, EPI_ISL_909052, EPI_ISL_909060, EPI_ISL_909066 to 909067, EPI_ISL_909071 to 909072, EPI_ISL_909075, EPI_ISL_909079 to 909081, EPI_ISL_909088, EPI_ISL_909093, EPI_ISL_909095 to 909097, EPI_ISL_909102 to 909103, EPI_ISL_909105, EPI_ISL_909109, EPI_ISL_909119, EPI_ISL_909122, EPI_ISL_909128, EPI_ISL_909144 to 909145, EPI_ISL_909148, EPI_ISL_909155 to 909157, EPI_ISL_909173, EPI_ISL_909178, EPI_ISL_909180, EPI_ISL_909182, EPI_ISL_909187, EPI_ISL_909189, EPI_ISL_909192 to 909193, EPI_ISL_909202 to 909204, EPI_ISL_909217, EPI_ISL_909220, EPI_ISL_909223, EPI_ISL_909230, EPI_ISL_909234, EPI_ISL_909238, EPI_ISL_909240, EPI_ISL_909243, EPI_ISL_909251, EPI_ISL_909254, EPI_ISL_912214, EPI_ISL_916180 to 916183, EPI_ISL_916185, EPI_ISL_916187 to 916194, EPI_ISL_916196, EPI_ISL_916200 to 916203, EPI_ISL_916205, EPI_ISL_916207 to 916213, EPI_ISL_916216, EPI_ISL_916222, EPI_ISL_916225 to 916229, EPI_ISL_916231 to 916234, EPI_ISL_916236 to 916238, EPI_ISL_916241 to 916250, EPI_ISL_916252, EPI_ISL_916254, EPI_ISL_916256, EPI_ISL_916258, EPI_ISL_916260 to 916264, EPI_ISL_916266, EPI_ISL_916268 to 916271, EPI_ISL_916273 to 916281, EPI_ISL_916283 to 916285, EPI_ISL_916287 to 916288, EPI_ISL_916290 to 916292, EPI_ISL_916295 to 916297, EPI_ISL_916299, EPI_ISL_916301, EPI_ISL_916303 to 916308, EPI_ISL_916310, EPI_ISL_916312 to 916316, EPI_ISL_916318, EPI_ISL_916320 to 916323, EPI_ISL_916325, EPI_ISL_916327 to 916329, EPI_ISL_916331 to 916336, EPI_ISL_916338 to 916350, EPI_ISL_916352, EPI_ISL_916354 to 916355, EPI_ISL_916357 to 916358, EPI_ISL_916360, EPI_ISL_916362 to 916384, EPI_ISL_916388, EPI_ISL_916390 to 916392, EPI_ISL_916394, EPI_ISL_916397, EPI_ISL_916399 to 916401, EPI_ISL_916403, EPI_ISL_916405 to 916417, EPI_ISL_916419 to 916421, EPI_ISL_916425, EPI_ISL_916427 to 916428, EPI_ISL_916430 to 916447, EPI_ISL_916449 to 916451, EPI_ISL_916453 to 916464, EPI_ISL_916467, EPI_ISL_916470, EPI_ISL_916472 to 916473, EPI_ISL_916475 to 916481, EPI_ISL_916484 to 916487, EPI_ISL_916489, EPI_ISL_916491 to 916497, EPI_ISL_916499 to 916500, EPI_ISL_916502, EPI_ISL_932576, EPI_ISL_932581, EPI_ISL_932596, EPI_ISL_932598, EPI_ISL_932603, EPI_ISL_932611, EPI_ISL_932613 to 932614, EPI_ISL_932619, EPI_ISL_932621, EPI_ISL_932644, EPI_ISL_932646, EPI_ISL_932648, EPI_ISL_932651 to 932652, EPI_ISL_932655, EPI_ISL_932664, EPI_ISL_932666, EPI_ISL_932669, EPI_ISL_932671, EPI_ISL_932674, EPI_ISL_932677 to 932678, EPI_ISL_932680, EPI_ISL_932686 to 932687, EPI_ISL_932689, EPI_ISL_932691, EPI_ISL_932693, EPI_ISL_932703, EPI_ISL_932706, EPI_ISL_932710, EPI_ISL_932712, EPI_ISL_932715, EPI_ISL_932718, EPI_ISL_932721 to 932722, EPI_ISL_932728, EPI_ISL_932737, EPI_ISL_932739, EPI_ISL_932742, EPI_ISL_932754 to 932755, EPI_ISL_932758, EPI_ISL_932770 to 932771, EPI_ISL_932776, EPI_ISL_932778, EPI_ISL_932782, EPI_ISL_932790, EPI_ISL_932794, EPI_ISL_932796, EPI_ISL_932805, EPI_ISL_932808, EPI_ISL_932812, EPI_ISL_932816, EPI_ISL_932820, EPI_ISL_932822, EPI_ISL_932827, EPI_ISL_932829 to 932830, EPI_ISL_932836, EPI_ISL_932838 to 932839, EPI_ISL_932842 to 932843, EPI_ISL_933113 to 933139, EPI_ISL_933141 to 933424, EPI_ISL_938234 to 938578, EPI_ISL_945049, EPI_ISL_945051, EPI_ISL_945054, EPI_ISL_945056, EPI_ISL_945061,

EPI_ISL_945063, EPI_ISL_945067 to 945068, EPI_ISL_945070, EPI_ISL_945072, EPI_ISL_945076, EPI_ISL_945083 to 945085, EPI_ISL_945089, EPI_ISL_945095, EPI_ISL_945100, EPI_ISL_945102 to 945103, EPI_ISL_945106 to 945107, EPI_ISL_945109, EPI_ISL_945114 to 945116, EPI_ISL_945122, EPI_ISL_945125,
EPI_ISL_945130 to 945131, EPI_ISL_945139, EPI_ISL_945142 to 945143, EPI_ISL_945146 to 945148, EPI_ISL_945152, EPI_ISL_945155, EPI_ISL_945157, EPI_ISL_945163, EPI_ISL_945166, EPI_ISL_945169 to 945170, EPI_ISL_945176, EPI_ISL_945180 to 945181, EPI_ISL_945186, EPI_ISL_945188, EPI_ISL_945190 to
945192, EPI_ISL_945197, EPI_ISL_945199, EPI_ISL_945202 to 945203, EPI_ISL_945207 to 945208, EPI_ISL_945212, EPI_ISL_945217, EPI_ISL_945221 to 945222, EPI_ISL_945226, EPI_ISL_945232, EPI_ISL_945236 to 945238, EPI_ISL_945241, EPI_ISL_945243 to 945244, EPI_ISL_945247 to 945250, EPI_ISL_945254,
EPI_ISL_945257, EPI_ISL_945261, EPI_ISL_945268, EPI_ISL_945271, EPI_ISL_945275, EPI_ISL_945279, EPI_ISL_945285, EPI_ISL_945288, EPI_ISL_945297, EPI_ISL_945304 to 945305, EPI_ISL_945309, EPI_ISL_945311, EPI_ISL_945319 to 945321, EPI_ISL_945324, EPI_ISL_945332, EPI_ISL_945334, EPI_ISL_945337,
EPI_ISL_945341, EPI_ISL_945345 to 945347, EPI_ISL_945350, EPI_ISL_945356, EPI_ISL_945363 to 945367, EPI_ISL_948101 to 948102, EPI_ISL_948104, EPI_ISL_948107, EPI_ISL_948110, EPI_ISL_948114, EPI_ISL_948116 to 948118, EPI_ISL_948120, EPI_ISL_948122 to 948125, EPI_ISL_948128, EPI_ISL_948131,
EPI_ISL_948133 to 948134, EPI_ISL_948136 to 948137, EPI_ISL_948139, EPI_ISL_948145, EPI_ISL_948147, EPI_ISL_948149 to 948151, EPI_ISL_948153, EPI_ISL_948155, EPI_ISL_948159 to 948160, EPI_ISL_948163 to 948166, EPI_ISL_948172, EPI_ISL_948174, EPI_ISL_948176 to 948178, EPI_ISL_948180 to 948181,
EPI_ISL_948183, EPI_ISL_948185 to 948189, EPI_ISL_948191 to 948194, EPI_ISL_948197 to 948201, EPI_ISL_948203 to 948205, EPI_ISL_948207 to 948216, EPI_ISL_948219 to 948221, EPI_ISL_948223 to 948224, EPI_ISL_948227 to 948228, EPI_ISL_948230 to 948231, EPI_ISL_948233 to 948235, EPI_ISL_948237,
EPI_ISL_948239 to 948241, EPI_ISL_948244, EPI_ISL_948247, EPI_ISL_948249 to 948251, EPI_ISL_948253, EPI_ISL_948255, EPI_ISL_948257 to 948259, EPI_ISL_948261, EPI_ISL_948264 to 948266, EPI_ISL_948269, EPI_ISL_948274, EPI_ISL_948277 to 948284, EPI_ISL_948286, EPI_ISL_948288 to 948289,
EPI_ISL_948292, EPI_ISL_948294, EPI_ISL_948296, EPI_ISL_948298, EPI_ISL_948300 to 948302, EPI_ISL_948305 to 948306, EPI_ISL_948308, EPI_ISL_948310 to 948314, EPI_ISL_948316, EPI_ISL_948321 to 948323, EPI_ISL_948326, EPI_ISL_948328, EPI_ISL_948330 to 948332, EPI_ISL_948337 to 948338,
EPI_ISL_948340 to 948342, EPI_ISL_948344 to 948346, EPI_ISL_948350, EPI_ISL_948355, EPI_ISL_948359, EPI_ISL_948361, EPI_ISL_948364, EPI_ISL_948366, EPI_ISL_948368, EPI_ISL_948372, EPI_ISL_948374, EPI_ISL_948376, EPI_ISL_948378, EPI_ISL_948380 to 948383, EPI_ISL_948388, EPI_ISL_948391 to
948393, EPI_ISL_948395 to 948397, EPI_ISL_948402, EPI_ISL_948407 to 948408, EPI_ISL_948412 to 948414, EPI_ISL_948420 to 948424, EPI_ISL_948431, EPI_ISL_948433 to 948435, EPI_ISL_948437, EPI_ISL_948441 to 948442, EPI_ISL_948445, EPI_ISL_957784, EPI_ISL_959267, EPI_ISL_971425, EPI_ISL_980235 to
980550, EPI_ISL_985411 to 985432, EPI_ISL_985434 to 985611, EPI_ISL_986439 to 986823, EPI_ISL_987161 to 987281, EPI_ISL_987283, EPI_ISL_987287 to 987293, EPI_ISL_987295 to 987296, EPI_ISL_987299, EPI_ISL_987301 to 987304, EPI_ISL_987306 to 987307, EPI_ISL_987309 to 987310, EPI_ISL_987312 to
987313, EPI_ISL_987315, EPI_ISL_987318 to 987319, EPI_ISL_987321 to 987322, EPI_ISL_987325 to 987326, EPI_ISL_987328, EPI_ISL_987332, EPI_ISL_987339 to 987340, EPI_ISL_987344, EPI_ISL_987348, EPI_ISL_987357, EPI_ISL_987361, EPI_ISL_987365, EPI_ISL_987367 to 987369, EPI_ISL_987372,
EPI_ISL_987378 to 987384, EPI_ISL_987387, EPI_ISL_987389, EPI_ISL_987392 to 987393, EPI_ISL_987397 to 987399, EPI_ISL_987401 to 987402, EPI_ISL_987406 to 987409, EPI_ISL_987411 to 987419, EPI_ISL_987421, EPI_ISL_987424 to 987430, EPI_ISL_987432, EPI_ISL_987435, EPI_ISL_987441, EPI_ISL_987443,
EPI_ISL_987445 to 987446, EPI_ISL_987449, EPI_ISL_987451 to 987453, EPI_ISL_987455 to 987456, EPI_ISL_987458, EPI_ISL_987462, EPI_ISL_987464 to 987465, EPI_ISL_987467, EPI_ISL_987472, EPI_ISL_987474 to 987476, EPI_ISL_987478 to 987480, EPI_ISL_987482, EPI_ISL_987484, EPI_ISL_987488 to 987490,
EPI_ISL_987493, EPI_ISL_987498 to 987499, EPI_ISL_987505 to 987506, EPI_ISL_987508 to 987510, EPI_ISL_987513 to 987514, EPI_ISL_987518 to 987521, EPI_ISL_987523, EPI_ISL_987525 to 987527, EPI_ISL_987530, EPI_ISL_987540 to 987542, EPI_ISL_987545 to 987547, EPI_ISL_987553 to 987554, EPI_ISL_987557,
EPI_ISL_987559 to 987562, EPI_ISL_987566 to 987567, EPI_ISL_987573, EPI_ISL_987575, EPI_ISL_987577 to 987579, EPI_ISL_987581, EPI_ISL_987583, EPI_ISL_987587, EPI_ISL_987589, EPI_ISL_987591, EPI_ISL_987594 to 987599, EPI_ISL_987602 to 987607, EPI_ISL_987609 to 987613, EPI_ISL_987615,
EPI_ISL_987617, EPI_ISL_987622 to 987625, EPI_ISL_987627 to 987628, EPI_ISL_987631, EPI_ISL_987633, EPI_ISL_987637, EPI_ISL_987640, EPI_ISL_987683, EPI_ISL_987698, EPI_ISL_987704, EPI_ISL_987713, EPI_ISL_987745, EPI_ISL_987757, EPI_ISL_987767, EPI_ISL_987775, EPI_ISL_987816, EPI_ISL_987822,
EPI_ISL_987827, EPI_ISL_987862, EPI_ISL_987878, EPI_ISL_987888, EPI_ISL_987893, EPI_ISL_987920, EPI_ISL_987975, EPI_ISL_988011 to 988204, EPI_ISL_988208, EPI_ISL_988212, EPI_ISL_988214, EPI_ISL_988216, EPI_ISL_988219 to 988220, EPI_ISL_988226, EPI_ISL_988228, EPI_ISL_988230 to 988233,
EPI_ISL_988237, EPI_ISL_988240, EPI_ISL_988245 to 988247, EPI_ISL_988251 to 988253, EPI_ISL_988255, EPI_ISL_988258 to 988260, EPI_ISL_988264 to 988269, EPI_ISL_988272 to 988275, EPI_ISL_988277 to 988278, EPI_ISL_988280 to 988283, EPI_ISL_988285, EPI_ISL_988288, EPI_ISL_988291 to 988295,
EPI_ISL_988298, EPI_ISL_988300 to 988304, EPI_ISL_988311, EPI_ISL_988315 to 988316, EPI_ISL_988318 to 988320, EPI_ISL_988323 to 988324, EPI_ISL_988326 to 988327, EPI_ISL_988330, EPI_ISL_988333, EPI_ISL_988336, EPI_ISL_988338 to 988344, EPI_ISL_988346, EPI_ISL_988349 to 988350, EPI_ISL_988353,
EPI_ISL_988355 to 988356, EPI_ISL_988359, EPI_ISL_988361, EPI_ISL_988366 to 988367, EPI_ISL_988369, EPI_ISL_988372 to 988374, EPI_ISL_988376, EPI_ISL_988379, EPI_ISL_988381, EPI_ISL_988386 to 988388, EPI_ISL_988390, EPI_ISL_988392 to 988393, EPI_ISL_988396 to 988397, EPI_ISL_988401 to 988402,
EPI_ISL_988406, EPI_ISL_988408 to 988416, EPI_ISL_988419 to 988422, EPI_ISL_988424 to 988425, EPI_ISL_988429, EPI_ISL_988431, EPI_ISL_988433, EPI_ISL_988435, EPI_ISL_988437 to 988438, EPI_ISL_988441 to 988444, EPI_ISL_988447, EPI_ISL_988450 to 988451, EPI_ISL_988454 to 988458, EPI_ISL_988462,
EPI_ISL_988464 to 988465, EPI_ISL_988467, EPI_ISL_988470 to 988471, EPI_ISL_988473, EPI_ISL_988475 to 988479, EPI_ISL_988481, EPI_ISL_989363 to 989443, EPI_ISL_993415, EPI_ISL_993418, EPI_ISL_993420, EPI_ISL_993424, EPI_ISL_993430 to 993431, EPI_ISL_993435, EPI_ISL_993437, EPI_ISL_993439 to
993440, EPI_ISL_993448 to 993449, EPI_ISL_993455, EPI_ISL_993457, EPI_ISL_993465, EPI_ISL_993469, EPI_ISL_993477, EPI_ISL_993487, EPI_ISL_993496, EPI_ISL_993504, EPI_ISL_993507, EPI_ISL_993519, EPI_ISL_993522 to 993523, EPI_ISL_993532, EPI_ISL_993538, EPI_ISL_993542 to 993543, EPI_ISL_993546,
EPI_ISL_993549 to 993550, EPI_ISL_993560, EPI_ISL_993562, EPI_ISL_993571, EPI_ISL_993581 to 993582, EPI_ISL_993584, EPI_ISL_993589, EPI_ISL_993593, EPI_ISL_993595, EPI_ISL_993598 to 993599, EPI_ISL_993616, EPI_ISL_993619 to 993621, EPI_ISL_993634, EPI_ISL_993638 to 993641, EPI_ISL_993654,
EPI_ISL_993656, EPI_ISL_993661, EPI_ISL_993682 to 993684, EPI_ISL_993690, EPI_ISL_993697 to 993698, EPI_ISL_993704, EPI_ISL_993706, EPI_ISL_993718, EPI_ISL_993720, EPI_ISL_993722, EPI_ISL_993736, EPI_ISL_993738 to 993744, EPI_ISL_993746 to 993747, EPI_ISL_993750, EPI_ISL_993754,
EPI_ISL_993756 to 993758, EPI_ISL_993761 to 993762, EPI_ISL_993766, EPI_ISL_993770 to 993771, EPI_ISL_993774, EPI_ISL_993776, EPI_ISL_993778 to 993779, EPI_ISL_993782, EPI_ISL_993784, EPI_ISL_993791 to 993794, EPI_ISL_993797 to 993798, EPI_ISL_993802 to 993804, EPI_ISL_993807 to 993811,
EPI_ISL_993813 to 993814, EPI_ISL_993816 to 993817, EPI_ISL_993819, EPI_ISL_993821, EPI_ISL_993823 to 993824, EPI_ISL_993827, EPI_ISL_993830, EPI_ISL_993832 to 993833, EPI_ISL_993836 to 993838, EPI_ISL_993843, EPI_ISL_993847, EPI_ISL_993849 to 993850, EPI_ISL_993853 to 993856, EPI_ISL_993859,
EPI_ISL_993863 to 993869, EPI_ISL_993871 to 993873, EPI_ISL_993878 to 993879, EPI_ISL_993881, EPI_ISL_993887, EPI_ISL_993890, EPI_ISL_993892 to 993895, EPI_ISL_993900, EPI_ISL_993903, EPI_ISL_993906 to 993907, EPI_ISL_993909, EPI_ISL_993911 to 993912, EPI_ISL_993915, EPI_ISL_993920 to 993922,
EPI_ISL_993926 to 993927, EPI_ISL_993937, EPI_ISL_993942, EPI_ISL_993945 to 993946, EPI_ISL_993949, EPI_ISL_993954, EPI_ISL_993956 to 993957, EPI_ISL_993960 to 993961, EPI_ISL_993963, EPI_ISL_993966, EPI_ISL_993968 to 993969, EPI_ISL_993972 to 993973, EPI_ISL_993975 to 993976, EPI_ISL_993979,
EPI_ISL_993982 to 993984, EPI_ISL_993987, EPI_ISL_993992, EPI_ISL_993994, EPI_ISL_993996 to 993997, EPI_ISL_994000 to 994001, EPI_ISL_994003 to 994004, EPI_ISL_994006 to 994008, EPI_ISL_994010 to 994011, EPI_ISL_994014 to 994015, EPI_ISL_994018 to 994020, EPI_ISL_994022, EPI_ISL_994024 to 994025,
EPI_ISL_994028, EPI_ISL_994031 to 994033, EPI_ISL_994040, EPI_ISL_994042, EPI_ISL_994044 to 994046, EPI_ISL_994048, EPI_ISL_994050, EPI_ISL_994052 to 994053, EPI_ISL_994055, EPI_ISL_994058 to 994061, EPI_ISL_994064, EPI_ISL_994066, EPI_ISL_994287, EPI_ISL_994290, EPI_ISL_994294 to 994296,
EPI_ISL_994299 to 994300, EPI_ISL_994310, EPI_ISL_994315, EPI_ISL_994319, EPI_ISL_994327, EPI_ISL_994331, EPI_ISL_994334, EPI_ISL_994341, EPI_ISL_994349 to 994350, EPI_ISL_994356, EPI_ISL_994359, EPI_ISL_994362 to 994363, EPI_ISL_994372 to 994374, EPI_ISL_994382 to 994383, EPI_ISL_994390,
EPI_ISL_994395, EPI_ISL_994397, EPI_ISL_994401, EPI_ISL_994403, EPI_ISL_994408 to 994410, EPI_ISL_994420 to 994421, EPI_ISL_994425, EPI_ISL_994427, EPI_ISL_994429, EPI_ISL_994431 to 994432, EPI_ISL_994434 to 994436, EPI_ISL_994440 to 994441, EPI_ISL_994444, EPI_ISL_994447, EPI_ISL_994452,
EPI_ISL_994457 to 994459, EPI_ISL_994470, EPI_ISL_994472, EPI_ISL_994477, EPI_ISL_994480, EPI_ISL_994497, EPI_ISL_994501, EPI_ISL_994504, EPI_ISL_994508, EPI_ISL_994511, EPI_ISL_994516 to 994517, EPI_ISL_994521, EPI_ISL_994524, EPI_ISL_994530, EPI_ISL_994536, EPI_ISL_994544, EPI_ISL_994610,
EPI_ISL_994653, EPI_ISL_1005700 to 1005717, EPI_ISL_1005719 to 1005726, EPI_ISL_1005728 to 1005730, EPI_ISL_1005732 to 1005740, EPI_ISL_1005744 to 1005745, EPI_ISL_1005747 to 1005752, EPI_ISL_1005754 to 1005759, EPI_ISL_1005761 to 1005764, EPI_ISL_1005766 to 1005772, EPI_ISL_1005775 to 1005777,
EPI_ISL_1005779 to 1005781, EPI_ISL_1005783 to 1005784, EPI_ISL_1005786 to 1005798, EPI_ISL_1005800 to 1005803, EPI_ISL_1005805 to 1005806, EPI_ISL_1005808, EPI_ISL_1005810 to 1005815, EPI_ISL_1005818 to 1005830, EPI_ISL_1005832 to 1005835, EPI_ISL_1005837, EPI_ISL_1005839 to 1005840,
EPI_ISL_1005842, EPI_ISL_1005844 to 1005856, EPI_ISL_1005858 to 1005861, EPI_ISL_1005863 to 1005872, EPI_ISL_1005875 to 1005882, EPI_ISL_1005884, EPI_ISL_1005886 to 1005888, EPI_ISL_1005890 to 1005891, EPI_ISL_1005893 to 1005894, EPI_ISL_1005896 to 1005900, EPI_ISL_1005904 to 1005908,
EPI_ISL_1005910, EPI_ISL_1005913, EPI_ISL_1005915 to 1005921, EPI_ISL_1005923 to 1005926, EPI_ISL_1005928 to 1005934, EPI_ISL_1005936 to 1005938, EPI_ISL_1005941 to 1005950, EPI_ISL_1005953 to 1005956, EPI_ISL_1005959 to 1005960, EPI_ISL_1006186 to 1006340, EPI_ISL_1006548 to 1006549,
EPI_ISL_1006552, EPI_ISL_1006554, EPI_ISL_1006557 to 1006558, EPI_ISL_1006561 to 1006564, EPI_ISL_1006567, EPI_ISL_1006569, EPI_ISL_1006571 to 1006572, EPI_ISL_1006575, EPI_ISL_1006577 to 1006578, EPI_ISL_1006581, EPI_ISL_1006585, EPI_ISL_1006587 to 1006588, EPI_ISL_1006592 to 1006593,
EPI_ISL_1006595 to 1006600, EPI_ISL_1006603, EPI_ISL_1006608, EPI_ISL_1006610 to 1006612, EPI_ISL_1006615, EPI_ISL_1006618 to 1006619, EPI_ISL_1006622 to 1006623, EPI_ISL_1006625, EPI_ISL_1006627 to 1006629, EPI_ISL_1006631, EPI_ISL_1006633, EPI_ISL_1006635, EPI_ISL_1006638, EPI_ISL_1006640
to 1006641, EPI_ISL_1006643 to 1006644, EPI_ISL_1006647, EPI_ISL_1006652 to 1006655, EPI_ISL_1006864 to 1006936, EPI_ISL_1007181 to 1007433, EPI_ISL_1007445 to 1007525, EPI_ISL_1007528 to 1007548, EPI_ISL_1007583, EPI_ISL_1012099 to 1012402, EPI_ISL_1012407, EPI_ISL_1012409, EPI_ISL_1012414,
EPI_ISL_1012416, EPI_ISL_1012428, EPI_ISL_1012431, EPI_ISL_1012438, EPI_ISL_1012449 to 1012450, EPI_ISL_1012458, EPI_ISL_1012482, EPI_ISL_1012484, EPI_ISL_1012493, EPI_ISL_1012498 to 1012499, EPI_ISL_1012514, EPI_ISL_1012521, EPI_ISL_1012531, EPI_ISL_1012541, EPI_ISL_1012549,
EPI_ISL_1012609 to 1012668, EPI_ISL_1018689 to 1018765, EPI_ISL_1018767 to 1018949, EPI_ISL_1019286 to 1019408, EPI_ISL_1019504 to 1019506, EPI_ISL_1033163 to 1033288, EPI_ISL_1033290 to 1033536, EPI_ISL_1033601, EPI_ISL_1033610, EPI_ISL_1033616, EPI_ISL_1033620, EPI_ISL_1033644,
EPI_ISL_1033660, EPI_ISL_1033669 to 1033670, EPI_ISL_1033673, EPI_ISL_1033699, EPI_ISL_1033701, EPI_ISL_1033704, EPI_ISL_1033755, EPI_ISL_1033764, EPI_ISL_1033766 to 1033767, EPI_ISL_1033772 to 1033773, EPI_ISL_1033780, EPI_ISL_1033791, EPI_ISL_1033793, EPI_ISL_1033797, EPI_ISL_1033802,
EPI_ISL_1033806 to 1033807, EPI_ISL_1033823, EPI_ISL_1033836, EPI_ISL_1033845, EPI_ISL_1033850, EPI_ISL_1033853, EPI_ISL_1033855, EPI_ISL_1033862, EPI_ISL_1033868, EPI_ISL_1033880, EPI_ISL_1033884, EPI_ISL_1033889, EPI_ISL_1033893, EPI_ISL_1033895, EPI_ISL_1033898, EPI_ISL_1033904,
EPI_ISL_1033909, EPI_ISL_1034143, EPI_ISL_1042003, EPI_ISL_1042008, EPI_ISL_1042011, EPI_ISL_1042014, EPI_ISL_1042016 to 1042017, EPI_ISL_1042019 to 1042020, EPI_ISL_1042023 to 1042024, EPI_ISL_1042028, EPI_ISL_1042030 to 1042031, EPI_ISL_1042033, EPI_ISL_1042035 to 1042037, EPI_ISL_1042040,
EPI_ISL_1042042 to 1042043, EPI_ISL_1042045 to 1042051, EPI_ISL_1042053, EPI_ISL_1042057, EPI_ISL_1042059, EPI_ISL_1042062, EPI_ISL_1042064, EPI_ISL_1042066 to 1042067, EPI_ISL_1042069, EPI_ISL_1042071, EPI_ISL_1042073 to 1042077, EPI_ISL_1042080 to 1042082, EPI_ISL_1042084 to 1042085,
EPI_ISL_1042091 to 1042092, EPI_ISL_1042094, EPI_ISL_1042096 to 1042098, EPI_ISL_1042100 to 1042101, EPI_ISL_1042103, EPI_ISL_1042108 to 1042109, EPI_ISL_1042112 to 1042113, EPI_ISL_1042115, EPI_ISL_1042117, EPI_ISL_1042120, EPI_ISL_1042122 to 1042123, EPI_ISL_1042130, EPI_ISL_1042134 to
1042135, EPI_ISL_1042140, EPI_ISL_1042143, EPI_ISL_1042149 to 1042150, EPI_ISL_1042152 to 1042153, EPI_ISL_1042157 to 1042158, EPI_ISL_1042160, EPI_ISL_1042162 to 1042165, EPI_ISL_1042168, EPI_ISL_1042172, EPI_ISL_1042175 to 1042177, EPI_ISL_1042179, EPI_ISL_1042181, EPI_ISL_1042184,
EPI_ISL_1042187, EPI_ISL_1042189 to 1042191, EPI_ISL_1042194, EPI_ISL_1042196, EPI_ISL_1042202, EPI_ISL_1042208, EPI_ISL_1042213, EPI_ISL_1042222 to 1042223, EPI_ISL_1042226, EPI_ISL_1042228, EPI_ISL_1042231 to 1042233, EPI_ISL_1042238, EPI_ISL_1042240 to 1042243, EPI_ISL_1042245,
EPI_ISL_1042248 to 1042250, EPI_ISL_1042252 to 1042253, EPI_ISL_1042259, EPI_ISL_1042266, EPI_ISL_1042269, EPI_ISL_1042271 to 1042272, EPI_ISL_1042274, EPI_ISL_1042279 to 1042280, EPI_ISL_1042282, EPI_ISL_1042284, EPI_ISL_1042286, EPI_ISL_1042288 to 1042289, EPI_ISL_1042291, EPI_ISL_1042293,
EPI_ISL_1042295, EPI_ISL_1042297 to 1042300, EPI_ISL_1042306 to 1042308, EPI_ISL_1042312, EPI_ISL_1042314 to 1042315, EPI_ISL_1042317, EPI_ISL_1042320, EPI_ISL_1042325 to 1042326, EPI_ISL_1042329, EPI_ISL_1042333 to 1042335, EPI_ISL_1042342, EPI_ISL_1042344 to 1042345, EPI_ISL_1042350,
EPI_ISL_1042356, EPI_ISL_1044225 to 1044385, EPI_ISL_1044725 to 1044864, EPI_ISL_1045682 to 1045704, EPI_ISL_1045706, EPI_ISL_1045708, EPI_ISL_1045727 to 1045728, EPI_ISL_1045730, EPI_ISL_1045733, EPI_ISL_1045747, EPI_ISL_1045751, EPI_ISL_1045755, EPI_ISL_1045757, EPI_ISL_1045761 to 1045762,
EPI_ISL_1045768, EPI_ISL_1045773, EPI_ISL_1045777 to 1045778, EPI_ISL_1045788, EPI_ISL_1045809, EPI_ISL_1045812, EPI_ISL_1045815, EPI_ISL_1045819 to 1045820, EPI_ISL_1045822, EPI_ISL_1045831, EPI_ISL_1045842, EPI_ISL_1045844 to 1045846, EPI_ISL_1045848, EPI_ISL_1045850, EPI_ISL_1045853 to
1045854, EPI_ISL_1045870, EPI_ISL_1045872, EPI_ISL_1045876 to 1045878, EPI_ISL_1045880 to 1045882, EPI_ISL_1045884, EPI_ISL_1045887, EPI_ISL_1045890 to 1045894, EPI_ISL_1045896 to 1045899, EPI_ISL_1045901, EPI_ISL_1045903 to 1045904, EPI_ISL_1045907 to 1045908, EPI_ISL_1045912,
EPI_ISL_1045914, EPI_ISL_1045917 to 1045918, EPI_ISL_1045920, EPI_ISL_1045924, EPI_ISL_1045926 to 1045927, EPI_ISL_1045931 to 1045933, EPI_ISL_1045937, EPI_ISL_1045939, EPI_ISL_1045943, EPI_ISL_1045945, EPI_ISL_1045947 to 1045949, EPI_ISL_1045955 to 1045956, EPI_ISL_1045958, EPI_ISL_1045962,
EPI_ISL_1045965, EPI_ISL_1045967, EPI_ISL_1046336, EPI_ISL_1057818 to 1057856, EPI_ISL_1057879, EPI_ISL_1057881, EPI_ISL_1057888, EPI_ISL_1057893, EPI_ISL_1057899, EPI_ISL_1057902, EPI_ISL_1057904, EPI_ISL_1057907, EPI_ISL_1057914, EPI_ISL_1057917 to 1057918, EPI_ISL_1057922,
EPI_ISL_1057924, EPI_ISL_1057931 to 1057932, EPI_ISL_1057934, EPI_ISL_1057936, EPI_ISL_1057939, EPI_ISL_1057942, EPI_ISL_1057944 to 1057946, EPI_ISL_1057949, EPI_ISL_1057951, EPI_ISL_1057956 to 1057958, EPI_ISL_1057960 to 1057961, EPI_ISL_1057965 to 1057966, EPI_ISL_1057972, EPI_ISL_1057974,
EPI_ISL_1057976 to 1057978, EPI_ISL_1061435 to 1061696, EPI_ISL_1062412, EPI_ISL_1062416, EPI_ISL_1062419, EPI_ISL_1062429, EPI_ISL_1062435, EPI_ISL_1062438, EPI_ISL_1062442 to 1062443, EPI_ISL_1062452 to 1062453, EPI_ISL_1062462 to 1062463, EPI_ISL_1062467 to 1062468, EPI_ISL_1062476,
EPI_ISL_1062482 to 1062483, EPI_ISL_1062485, EPI_ISL_1062491, EPI_ISL_1062500, EPI_ISL_1062507, EPI_ISL_1062519 to 1062520, EPI_ISL_1062528, EPI_ISL_1062532, EPI_ISL_1062536 to 1062537, EPI_ISL_1062547, EPI_ISL_1062550, EPI_ISL_1062553, EPI_ISL_1062556 to 1062557, EPI_ISL_1062561,
EPI_ISL_1062565, EPI_ISL_1062574, EPI_ISL_1062578, EPI_ISL_1062586, EPI_ISL_1062592, EPI_ISL_1062597, EPI_ISL_1062602, EPI_ISL_1062607 to 1062609, EPI_ISL_1062611, EPI_ISL_1062615, EPI_ISL_1062618, EPI_ISL_1062622 to 1062623, EPI_ISL_1062627, EPI_ISL_1062629, EPI_ISL_1062642,
EPI_ISL_1062648 to 1062649, EPI_ISL_1062657 to 1062658, EPI_ISL_1062662, EPI_ISL_1062664 to 1062665, EPI_ISL_1062672, EPI_ISL_1062677, EPI_ISL_1062683, EPI_ISL_1062690, EPI_ISL_1062698 to 1062699, EPI_ISL_1062705, EPI_ISL_1062708, EPI_ISL_1062725, EPI_ISL_1062730, EPI_ISL_1062748,
EPI_ISL_1062763, EPI_ISL_1062765, EPI_ISL_1062767, EPI_ISL_1063396, EPI_ISL_1069993 to 1069994, EPI_ISL_1069996 to 1069997, EPI_ISL_1069999 to 1070000, EPI_ISL_1070002 to 1070004, EPI_ISL_1070007, EPI_ISL_1070009, EPI_ISL_1070011 to 1070018, EPI_ISL_1070021, EPI_ISL_1070023 to 1070036,
EPI_ISL_1070039 to 1070041, EPI_ISL_1070043 to 1070044, EPI_ISL_1070046, EPI_ISL_1070049, EPI_ISL_1070051 to 1070052, EPI_ISL_1070054 to 1070057, EPI_ISL_1070059 to 1070060, EPI_ISL_1070062 to 1070063, EPI_ISL_1070065 to 1070070, EPI_ISL_1070072, EPI_ISL_1070074 to 1070081, EPI_ISL_1070086 to
1070089, EPI_ISL_1070091 to 1070093, EPI_ISL_1070096 to 1070099, EPI_ISL_1070101 to 1070105, EPI_ISL_1070108, EPI_ISL_1070110 to 1070112, EPI_ISL_1070114, EPI_ISL_1070116 to 1070117, EPI_ISL_1070119 to 1070120, EPI_ISL_1070123, EPI_ISL_1070126 to 1070128, EPI_ISL_1070130, EPI_ISL_1070133,
EPI_ISL_1070138, EPI_ISL_1070141 to 1070144, EPI_ISL_1070148, EPI_ISL_1070151, EPI_ISL_1070158, EPI_ISL_1070160, EPI_ISL_1070162, EPI_ISL_1070166, EPI_ISL_1070170, EPI_ISL_1070176 to 1070177, EPI_ISL_1070179, EPI_ISL_1070181, EPI_ISL_1070185 to 1070186, EPI_ISL_1070191, EPI_ISL_1070193 to
1070197, EPI_ISL_1070199, EPI_ISL_1070208, EPI_ISL_1070211, EPI_ISL_1070214, EPI_ISL_1070218, EPI_ISL_1070221, EPI_ISL_1070223, EPI_ISL_1070225, EPI_ISL_1070227, EPI_ISL_1070232, EPI_ISL_1070237 to 1070239, EPI_ISL_1070244, EPI_ISL_1070246 to 1070247, EPI_ISL_1070250 to 1070254,
EPI_ISL_1070257, EPI_ISL_1070259, EPI_ISL_1070268, EPI_ISL_1070271, EPI_ISL_1070277 to 1070278, EPI_ISL_1070280 to 1070283, EPI_ISL_1070285 to 1070286, EPI_ISL_1070288, EPI_ISL_1070290, EPI_ISL_1070293, EPI_ISL_1070296, EPI_ISL_1070302 to 1070304, EPI_ISL_1070309 to 1070315, EPI_ISL_1070321
to 1070323, EPI_ISL_1070328, EPI_ISL_1070333 to 1070334, EPI_ISL_1070337 to 1070338, EPI_ISL_1070340 to 1070341, EPI_ISL_1070348, EPI_ISL_1070350, EPI_ISL_1070352 to 1070353, EPI_ISL_1070355, EPI_ISL_1070357, EPI_ISL_1070359, EPI_ISL_1070361, EPI_ISL_1070366, EPI_ISL_1070369, EPI_ISL_1070371,
EPI_ISL_1070376, EPI_ISL_1070385 to 1070386, EPI_ISL_1070390 to 1070391, EPI_ISL_1070399, EPI_ISL_1070403, EPI_ISL_1070405, EPI_ISL_1070413, EPI_ISL_1070420 to 1070421, EPI_ISL_1070424, EPI_ISL_1070426, EPI_ISL_1070428, EPI_ISL_1070430, EPI_ISL_1070435 to 1070436, EPI_ISL_1070440 to 1070441,
EPI_ISL_1070451, EPI_ISL_1070454 to 1070456, EPI_ISL_1070460, EPI_ISL_1070464, EPI_ISL_1070466 to 1070467, EPI_ISL_1070472, EPI_ISL_1070474, EPI_ISL_1070480, EPI_ISL_1070484, EPI_ISL_1070487, EPI_ISL_1070489 to 1070490, EPI_ISL_1070493 to 1070494, EPI_ISL_1070498 to 1070501, EPI_ISL_1070503
to 1070504, EPI_ISL_1070507 to 1070508, EPI_ISL_1070510, EPI_ISL_1070516, EPI_ISL_1070520, EPI_ISL_1070525, EPI_ISL_1070527, EPI_ISL_1070531 to 1070532, EPI_ISL_1070540, EPI_ISL_1070544 to 1070545, EPI_ISL_1070547, EPI_ISL_1070549, EPI_ISL_1070552, EPI_ISL_1070555, EPI_ISL_1070558,
EPI_ISL_1070561, EPI_ISL_1070567, EPI_ISL_1070570, EPI_ISL_1070574, EPI_ISL_1070577 to 1070581, EPI_ISL_1070591, EPI_ISL_1070594, EPI_ISL_1070597 to 1070598, EPI_ISL_1070601, EPI_ISL_1070612, EPI_ISL_1070616 to 1070617, EPI_ISL_1070619, EPI_ISL_1070621 to 1070622, EPI_ISL_1070625,
EPI_ISL_1070628 to 1070725, EPI_ISL_1071372 to 1071373, EPI_ISL_1071379, EPI_ISL_1071388, EPI_ISL_1071393 to 1071394, EPI_ISL_1071396 to 1071399, EPI_ISL_1071402, EPI_ISL_1071404, EPI_ISL_1071407, EPI_ISL_1071410 to 1071412, EPI_ISL_1071414, EPI_ISL_1071419, EPI_ISL_1071431 to 1071433,
EPI_ISL_1071435, EPI_ISL_1071438 to 1071439, EPI_ISL_1071441, EPI_ISL_1071444 to 1071445, EPI_ISL_1071450, EPI_ISL_1071452, EPI_ISL_1071463, EPI_ISL_1071466, EPI_ISL_1071469, EPI_ISL_1071471, EPI_ISL_1071473, EPI_ISL_1071482, EPI_ISL_1071484, EPI_ISL_1071486, EPI_ISL_1071499,
EPI_ISL_1071501, EPI_ISL_1071503 to 1071504, EPI_ISL_1071506, EPI_ISL_1071510, EPI_ISL_1071512, EPI_ISL_1071514 to 1071515, EPI_ISL_1071520, EPI_ISL_1071524 to 1071525, EPI_ISL_1071527, EPI_ISL_1071535 to 1071538, EPI_ISL_1071543, EPI_ISL_1071555, EPI_ISL_1071565, EPI_ISL_1071567,
EPI_ISL_1071571, EPI_ISL_1071582, EPI_ISL_1071584, EPI_ISL_1071587, EPI_ISL_1071600 to 1071603, EPI_ISL_1071612 to 1071613, EPI_ISL_1071620, EPI_ISL_1071625, EPI_ISL_1071628, EPI_ISL_1082531, EPI_ISL_1082538, EPI_ISL_1082551, EPI_ISL_1082559, EPI_ISL_1082567, EPI_ISL_1082571,
EPI_ISL_1082573 to 1082574, EPI_ISL_1082583, EPI_ISL_1082593, EPI_ISL_1082601, EPI_ISL_1082604, EPI_ISL_1082606, EPI_ISL_1082609, EPI_ISL_1082614, EPI_ISL_1082617, EPI_ISL_1082619, EPI_ISL_1082627, EPI_ISL_1082641, EPI_ISL_1082663, EPI_ISL_1082665, EPI_ISL_1082674, EPI_ISL_1082679,
EPI_ISL_1082687, EPI_ISL_1082692 to 1082693, EPI_ISL_1082695, EPI_ISL_1082704, EPI_ISL_1082710, EPI_ISL_1082714, EPI_ISL_1082723 to 1082724, EPI_ISL_1082726, EPI_ISL_1082729 to 1082730, EPI_ISL_1082756, EPI_ISL_1082764, EPI_ISL_1082767, EPI_ISL_1082772 to 1082774, EPI_ISL_1082776,
EPI_ISL_1082780, EPI_ISL_1082783 to 1082784, EPI_ISL_1082792 to 1082793, EPI_ISL_1082804, EPI_ISL_1082809, EPI_ISL_1082815, EPI_ISL_1082821, EPI_ISL_1082823, EPI_ISL_1082833, EPI_ISL_1082835 to 1082836, EPI_ISL_1082838 to 1082839, EPI_ISL_1082844 to 1082847, EPI_ISL_1082852 to 1082853,
EPI_ISL_1082856 to 1082857, EPI_ISL_1082859, EPI_ISL_1082862, EPI_ISL_1082865, EPI_ISL_1082867, EPI_ISL_1082870, EPI_ISL_1082873, EPI_ISL_1082876, EPI_ISL_1082880, EPI_ISL_1082890, EPI_ISL_1082894, EPI_ISL_1082896, EPI_ISL_1082905, EPI_ISL_1082908, EPI_ISL_1082911, EPI_ISL_1082919,
EPI_ISL_1082924, EPI_ISL_1082928 to 1082932, EPI_ISL_1082935, EPI_ISL_1082937 to 1082938, EPI_ISL_1082941, EPI_ISL_1082948, EPI_ISL_1082956, EPI_ISL_1082962, EPI_ISL_1082971, EPI_ISL_1082974 to 1082977, EPI_ISL_1082986, EPI_ISL_1082990, EPI_ISL_1082994, EPI_ISL_1082998 to 1082999,
EPI_ISL_1083010 to 1083014, EPI_ISL_1083016 to 1083017, EPI_ISL_1083024 to 1083027, EPI_ISL_1083033 to 1083035, EPI_ISL_1083038 to 1083040, EPI_ISL_1083044 to 1083045, EPI_ISL_1083054, EPI_ISL_1083056, EPI_ISL_1083060 to 1083061, EPI_ISL_1083064 to 1083065, EPI_ISL_1083067, EPI_ISL_1083077,
EPI_ISL_1083085, EPI_ISL_1083087, EPI_ISL_1083089, EPI_ISL_1083094, EPI_ISL_1083101, EPI_ISL_1083105, EPI_ISL_1083112, EPI_ISL_1083114 to 1083115, EPI_ISL_1083124, EPI_ISL_1083126, EPI_ISL_1083128 to 1083129, EPI_ISL_1084474, EPI_ISL_1084477, EPI_ISL_1084479, EPI_ISL_1084483,
EPI_ISL_1084486, EPI_ISL_1084489 to 1084490, EPI_ISL_1084494, EPI_ISL_1084497 to 1084499, EPI_ISL_1084501, EPI_ISL_1084512 to 1084514, EPI_ISL_1084516, EPI_ISL_1084518, EPI_ISL_1084522 to 1084523, EPI_ISL_1084528, EPI_ISL_1084530 to 1084532, EPI_ISL_1084540, EPI_ISL_1084544 to 1084545,

EPI_ISL_1084547, EPI_ISL_1084550, EPI_ISL_1084559 to 1084560, EPI_ISL_1084564, EPI_ISL_1084572 to 1084574, EPI_ISL_1084576, EPI_ISL_1084583, EPI_ISL_1084586, EPI_ISL_1084590, EPI_ISL_1084593 to 1084595, EPI_ISL_1084598, EPI_ISL_1084600, EPI_ISL_1099242, EPI_ISL_1099246, EPI_ISL_1099249,
EPI_ISL_1099256, EPI_ISL_1099258 to 1099259, EPI_ISL_1099263, EPI_ISL_1099268, EPI_ISL_1099271, EPI_ISL_1099279, EPI_ISL_1099287, EPI_ISL_1099301, EPI_ISL_1099304, EPI_ISL_1099306 to 1099307, EPI_ISL_1099314, EPI_ISL_1099318, EPI_ISL_1099321, EPI_ISL_1099335, EPI_ISL_1099340,
EPI_ISL_1099350, EPI_ISL_1099353, EPI_ISL_1099355, EPI_ISL_1099363 to 1099364, EPI_ISL_1099371, EPI_ISL_1099374, EPI_ISL_1099377 to 1099378, EPI_ISL_1099380 to 1099381, EPI_ISL_1099387, EPI_ISL_1099389, EPI_ISL_1099397, EPI_ISL_1099411 to 1099412, EPI_ISL_1099415, EPI_ISL_1099422,
EPI_ISL_1099424, EPI_ISL_1099427 to 1099428, EPI_ISL_1099430, EPI_ISL_1099435, EPI_ISL_1099442, EPI_ISL_1099453, EPI_ISL_1099460, EPI_ISL_1099465, EPI_ISL_1099468 to 1099469, EPI_ISL_1099472 to 1099473, EPI_ISL_1099475, EPI_ISL_1099488, EPI_ISL_1099495, EPI_ISL_1099744, EPI_ISL_1099746 to
1099748, EPI_ISL_1099751, EPI_ISL_1099755, EPI_ISL_1099758, EPI_ISL_1099762 to 1099766, EPI_ISL_1099771, EPI_ISL_1099774 to 1099775, EPI_ISL_1099777 to 1099778, EPI_ISL_1099780, EPI_ISL_1099782, EPI_ISL_1099785 to 1099786, EPI_ISL_1099788 to 1099790, EPI_ISL_1099792, EPI_ISL_1099794,
EPI_ISL_1099796 to 1099797, EPI_ISL_1099800 to 1099801, EPI_ISL_1099816, EPI_ISL_1099818 to 1099819, EPI_ISL_1099821, EPI_ISL_1099824 to 1099826, EPI_ISL_1099828, EPI_ISL_1099830 to 1099831, EPI_ISL_1099833, EPI_ISL_1099835, EPI_ISL_1099838, EPI_ISL_1099841 to 1099842, EPI_ISL_1099845 to
1099846, EPI_ISL_1099848, EPI_ISL_1099850, EPI_ISL_1099852, EPI_ISL_1099857 to 1099858, EPI_ISL_1099868, EPI_ISL_1099876, EPI_ISL_1099878, EPI_ISL_1099880, EPI_ISL_1099887, EPI_ISL_1099895, EPI_ISL_1099899 to 1099901, EPI_ISL_1099903 to 1099905, EPI_ISL_1099908 to 1099909, EPI_ISL_1099912,
EPI_ISL_1099914, EPI_ISL_1099917, EPI_ISL_1099919 to 1099920, EPI_ISL_1099922, EPI_ISL_1099925, EPI_ISL_1099927 to 1099928, EPI_ISL_1099931 to 1099932, EPI_ISL_1099934 to 1099936, EPI_ISL_1099941, EPI_ISL_1099945, EPI_ISL_1099951, EPI_ISL_1099954 to 1099957, EPI_ISL_1099959 to 1099960,
EPI_ISL_1099962, EPI_ISL_1099965, EPI_ISL_1099967, EPI_ISL_1099972, EPI_ISL_1099978, EPI_ISL_1099981, EPI_ISL_1099983 to 1099985, EPI_ISL_1099990, EPI_ISL_1099993, EPI_ISL_1099995 to 1099997, EPI_ISL_1100002, EPI_ISL_1100006 to 1100007, EPI_ISL_1100010, EPI_ISL_1100013 to 1100017,
EPI_ISL_1100020 to 1100021, EPI_ISL_1100023, EPI_ISL_1100025, EPI_ISL_1100045, EPI_ISL_1100047, EPI_ISL_1100050 to 1100052, EPI_ISL_1100055 to 1100057, EPI_ISL_1100060 to 1100061, EPI_ISL_1100063, EPI_ISL_1100065, EPI_ISL_1100067, EPI_ISL_1100755 to 1100758, EPI_ISL_1100760, EPI_ISL_1100767,
EPI_ISL_1100774, EPI_ISL_1100779, EPI_ISL_1100782, EPI_ISL_1100786 to 1100788, EPI_ISL_1100795 to 1100796, EPI_ISL_1100804, EPI_ISL_1100808 to 1100809, EPI_ISL_1100818 to 1100819, EPI_ISL_1100821 to 1100823, EPI_ISL_1100831, EPI_ISL_1100835, EPI_ISL_1100839, EPI_ISL_1100856, EPI_ISL_1100859,
EPI_ISL_1100862, EPI_ISL_1100864, EPI_ISL_1100866, EPI_ISL_1100870, EPI_ISL_1100874, EPI_ISL_1100876 to 1100878, EPI_ISL_1100882, EPI_ISL_1100884, EPI_ISL_1100891, EPI_ISL_1100895, EPI_ISL_1100898, EPI_ISL_1100901, EPI_ISL_1100903, EPI_ISL_1100905 to 1100906, EPI_ISL_1100911 to 1100912,
EPI_ISL_1100915 to 1100916, EPI_ISL_1100918 to 1100920, EPI_ISL_1100924 to 1100925, EPI_ISL_1100929, EPI_ISL_1100934, EPI_ISL_1100938, EPI_ISL_1100947, EPI_ISL_1100950 to 1100951, EPI_ISL_1100953, EPI_ISL_1100955, EPI_ISL_1100957 to 1100959, EPI_ISL_1100961, EPI_ISL_1100963, EPI_ISL_1100966,
EPI_ISL_1100969 to 1100971, EPI_ISL_1100973 to 1100975, EPI_ISL_1100985, EPI_ISL_1100988, EPI_ISL_1101663 to 1101665, EPI_ISL_1101669, EPI_ISL_1101671 to 1101673, EPI_ISL_1101675, EPI_ISL_1101678 to 1101681, EPI_ISL_1101685 to 1101688, EPI_ISL_1101690, EPI_ISL_1101693 to 1101694,
EPI_ISL_1101696 to 1101700, EPI_ISL_1101702 to 1101706, EPI_ISL_1101709 to 1101710, EPI_ISL_1101713 to 1101717, EPI_ISL_1101719 to 1101721, EPI_ISL_1101723 to 1101724, EPI_ISL_1101728 to 1101730, EPI_ISL_1101733, EPI_ISL_1101737 to 1101738, EPI_ISL_1101742 to 1101747, EPI_ISL_1101749,
EPI_ISL_1101751, EPI_ISL_1101756 to 1101758, EPI_ISL_1101761, EPI_ISL_1101763 to 1101765, EPI_ISL_1101769, EPI_ISL_1101772 to 1101773, EPI_ISL_1101776, EPI_ISL_1101778 to 1101779, EPI_ISL_1101781, EPI_ISL_1101783 to 1101785, EPI_ISL_1101788 to 1101793, EPI_ISL_1101795 to 1101798,
EPI_ISL_1101801, EPI_ISL_1101803 to 1101804, EPI_ISL_1101807, EPI_ISL_1101810 to 1101813, EPI_ISL_1101815, EPI_ISL_1101817 to 1101818, EPI_ISL_1101820 to 1101822, EPI_ISL_1101825 to 1101829, EPI_ISL_1101832 to 1101835, EPI_ISL_1101837, EPI_ISL_1101839 to 1101843, EPI_ISL_1101846 to 1101847,
EPI_ISL_1101849 to 1101850, EPI_ISL_1101852 to 1101858, EPI_ISL_1101860, EPI_ISL_1101862, EPI_ISL_1101864, EPI_ISL_1101866 to 1101870, EPI_ISL_1101872, EPI_ISL_1101874 to 1101878, EPI_ISL_1101881 to 1101883, EPI_ISL_1101887, EPI_ISL_1101890 to 1101891, EPI_ISL_1101894, EPI_ISL_1101899,
EPI_ISL_1101902, EPI_ISL_1101904, EPI_ISL_1101906 to 1101908, EPI_ISL_1101910 to 1101913, EPI_ISL_1101915, EPI_ISL_1101917 to 1101919, EPI_ISL_1101921, EPI_ISL_1101925 to 1101932, EPI_ISL_1101934 to 1101937, EPI_ISL_1101939 to 1101940, EPI_ISL_1101942, EPI_ISL_1101944 to 1101946,
EPI_ISL_1101948, EPI_ISL_1101950 to 1101953, EPI_ISL_1101955, EPI_ISL_1101957, EPI_ISL_1101959 to 1101960, EPI_ISL_1101962, EPI_ISL_1101964, EPI_ISL_1101966, EPI_ISL_1102718 to 1102719, EPI_ISL_1102721, EPI_ISL_1102723, EPI_ISL_1102727, EPI_ISL_1102730 to 1102731, EPI_ISL_1102734 to 1102735,
EPI_ISL_1102738 to 1102740, EPI_ISL_1102743, EPI_ISL_1102745 to 1102747, EPI_ISL_1102749 to 1102751, EPI_ISL_1102753 to 1102755, EPI_ISL_1102757 to 1102758, EPI_ISL_1102760 to 1102761, EPI_ISL_1102763, EPI_ISL_1102765 to 1102767, EPI_ISL_1102769 to 1102771, EPI_ISL_1102773 to 1102774,
EPI_ISL_1102778 to 1102779, EPI_ISL_1102784 to 1102785, EPI_ISL_1102788 to 1102792, EPI_ISL_1102794, EPI_ISL_1102796, EPI_ISL_1102799, EPI_ISL_1102802 to 1102803, EPI_ISL_1102805 to 1102810, EPI_ISL_1102812, EPI_ISL_1102814, EPI_ISL_1102816 to 1102817, EPI_ISL_1102824 to 1102826,
EPI_ISL_1102828 to 1102829, EPI_ISL_1102831, EPI_ISL_1102833 to 1102836, EPI_ISL_1102838 to 1102839, EPI_ISL_1102841 to 1102843, EPI_ISL_1102845 to 1102849, EPI_ISL_1102854, EPI_ISL_1102857 to 1102858, EPI_ISL_1102863 to 1102867, EPI_ISL_1102869, EPI_ISL_1102871, EPI_ISL_1102873,
EPI_ISL_1102877 to 1102879, EPI_ISL_1102883 to 1102884, EPI_ISL_1102886 to 1102887, EPI_ISL_1102897, EPI_ISL_1102899, EPI_ISL_1102901, EPI_ISL_1102905 to 1102906, EPI_ISL_1102908, EPI_ISL_1102913, EPI_ISL_1102916, EPI_ISL_1102919, EPI_ISL_1102921 to 1102924, EPI_ISL_1102927 to 1102931,
EPI_ISL_1102933 to 1102935, EPI_ISL_1102938, EPI_ISL_1102941, EPI_ISL_1102943 to 1102945, EPI_ISL_1102947 to 1102956, EPI_ISL_1102959 to 1102960, EPI_ISL_1102962 to 1102964, EPI_ISL_1102967, EPI_ISL_1102972, EPI_ISL_1102980 to 1102983, EPI_ISL_1102985, EPI_ISL_1102988 to 1102990,
EPI_ISL_1102992 to 1102994, EPI_ISL_1102996, EPI_ISL_1102998 to 1103000, EPI_ISL_1103002 to 1103003, EPI_ISL_1103007 to 1103015, EPI_ISL_1103017 to 1103021, EPI_ISL_1103025, EPI_ISL_1103028, EPI_ISL_1103030, EPI_ISL_1103035, EPI_ISL_1103039, EPI_ISL_1103044 to 1103046, EPI_ISL_1103048,
EPI_ISL_1103050 to 1103052, EPI_ISL_1115402, EPI_ISL_1115404, EPI_ISL_1115406, EPI_ISL_1115408 to 1115409, EPI_ISL_1115411, EPI_ISL_1115423, EPI_ISL_1115425, EPI_ISL_1115430, EPI_ISL_1115438, EPI_ISL_1115440, EPI_ISL_1115442 to 1115443, EPI_ISL_1115445 to 1115446, EPI_ISL_1115450,
EPI_ISL_1115453, EPI_ISL_1115455 to 1115456, EPI_ISL_1115460, EPI_ISL_1115464, EPI_ISL_1115466, EPI_ISL_1115470, EPI_ISL_1115474, EPI_ISL_1115476, EPI_ISL_1115479, EPI_ISL_1115484, EPI_ISL_1115486 to 1115487, EPI_ISL_1115491, EPI_ISL_1115496 to 1115499, EPI_ISL_1115503 to 1115509,
EPI_ISL_1115513 to 1115514, EPI_ISL_1115519, EPI_ISL_1115523, EPI_ISL_1115525, EPI_ISL_1115529, EPI_ISL_1115536 to 1115538, EPI_ISL_1115541, EPI_ISL_1115544, EPI_ISL_1115549, EPI_ISL_1115553, EPI_ISL_1115556 to 1115557, EPI_ISL_1115559, EPI_ISL_1115563, EPI_ISL_1115565, EPI_ISL_1115568,
EPI_ISL_1115574, EPI_ISL_1115579, EPI_ISL_1115586 to 1115588, EPI_ISL_1115593 to 1115594, EPI_ISL_1115600 to 1115601, EPI_ISL_1115604, EPI_ISL_1115606, EPI_ISL_1115609, EPI_ISL_1115611 to 1115612, EPI_ISL_1115614 to 1115616, EPI_ISL_1115622, EPI_ISL_1115624, EPI_ISL_1115626, EPI_ISL_1115629,
EPI_ISL_1115634, EPI_ISL_1115638, EPI_ISL_1115640, EPI_ISL_1115642, EPI_ISL_1115656 to 1115657, EPI_ISL_1115662, EPI_ISL_1115664, EPI_ISL_1115668 to 1115669, EPI_ISL_1115671 to 1115672, EPI_ISL_1116310, EPI_ISL_1174033, EPI_ISL_1174035, EPI_ISL_1174037, EPI_ISL_1174039, EPI_ISL_1174044 to
1174046, EPI_ISL_1174048, EPI_ISL_1174054, EPI_ISL_1174061, EPI_ISL_1174069, EPI_ISL_1174078, EPI_ISL_1174087, EPI_ISL_1174094 to 1174095, EPI_ISL_1174098 to 1174100, EPI_ISL_1174106 to 1174107, EPI_ISL_1174115, EPI_ISL_1174128 to 1174129, EPI_ISL_1174135, EPI_ISL_1174139, EPI_ISL_1174145 to
1174147, EPI_ISL_1174149 to 1174150, EPI_ISL_1174152, EPI_ISL_1174159, EPI_ISL_1174163, EPI_ISL_1174165, EPI_ISL_1174171, EPI_ISL_1174181, EPI_ISL_1174185, EPI_ISL_1174187 to 1174188, EPI_ISL_1174194, EPI_ISL_1174196 to 1174197, EPI_ISL_1174199 to 1174200, EPI_ISL_1174206, EPI_ISL_1174221,
EPI_ISL_1174226 to 1174227, EPI_ISL_1174230, EPI_ISL_1174234, EPI_ISL_1174238, EPI_ISL_1174245, EPI_ISL_1174248, EPI_ISL_1174250, EPI_ISL_1174253, EPI_ISL_1174256, EPI_ISL_1174263, EPI_ISL_1174269, EPI_ISL_1174272, EPI_ISL_1174275, EPI_ISL_1174278, EPI_ISL_1174281, EPI_ISL_1174285,
EPI_ISL_1174288 to 1174289, EPI_ISL_1174294, EPI_ISL_1174301 to 1174302, EPI_ISL_1174307, EPI_ISL_1174311, EPI_ISL_1174321, EPI_ISL_1174328, EPI_ISL_1174333, EPI_ISL_1174335, EPI_ISL_1174343 to 1174344, EPI_ISL_1174358, EPI_ISL_1174360, EPI_ISL_1174378 to 1174379, EPI_ISL_1174382,
EPI_ISL_1174387, EPI_ISL_1174389, EPI_ISL_1174395, EPI_ISL_1174397, EPI_ISL_1174400 to 1174401, EPI_ISL_1174413, EPI_ISL_1174425, EPI_ISL_1174431, EPI_ISL_1174443, EPI_ISL_1174445, EPI_ISL_1174452, EPI_ISL_1174459, EPI_ISL_1174462 to 1174464, EPI_ISL_1174467, EPI_ISL_1174472,
EPI_ISL_1174474, EPI_ISL_1174494, EPI_ISL_1174507, EPI_ISL_1174509, EPI_ISL_1174524, EPI_ISL_1174529, EPI_ISL_1174539, EPI_ISL_1174544, EPI_ISL_1174547, EPI_ISL_1174890, EPI_ISL_1174895, EPI_ISL_1174899, EPI_ISL_1174910, EPI_ISL_1174919, EPI_ISL_1174925, EPI_ISL_1174937, EPI_ISL_1174949,
EPI_ISL_1174954 to 1174955, EPI_ISL_1174968 to 1174969, EPI_ISL_1174978, EPI_ISL_1174992, EPI_ISL_1175003, EPI_ISL_1175013, EPI_ISL_1175020, EPI_ISL_1175044, EPI_ISL_1175058, EPI_ISL_1175093, EPI_ISL_1175101, EPI_ISL_1175106, EPI_ISL_1175114, EPI_ISL_1175136, EPI_ISL_1175139,
EPI_ISL_1175141, EPI_ISL_1175143 to 1175144, EPI_ISL_1175147 to 1175149, EPI_ISL_1175153, EPI_ISL_1175157 to 1175158, EPI_ISL_1175160, EPI_ISL_1175162, EPI_ISL_1175168, EPI_ISL_1175172, EPI_ISL_1175174, EPI_ISL_1175177, EPI_ISL_1175180, EPI_ISL_1175182 to 1175183, EPI_ISL_1175185,
EPI_ISL_1175187, EPI_ISL_1175190 to 1175191, EPI_ISL_1175196 to 1175199, EPI_ISL_1175201, EPI_ISL_1175204 to 1175205, EPI_ISL_1175213, EPI_ISL_1175216, EPI_ISL_1175223, EPI_ISL_1175229, EPI_ISL_1175232, EPI_ISL_1175237 to 1175238, EPI_ISL_1175240 to 1175241, EPI_ISL_1175243 to 1175244,
EPI_ISL_1175246, EPI_ISL_1175248, EPI_ISL_1175253 to 1175254, EPI_ISL_1175256 to 1175257, EPI_ISL_1175260, EPI_ISL_1175262, EPI_ISL_1175265, EPI_ISL_1175267 to 1175269, EPI_ISL_1175273 to 1175275, EPI_ISL_1175279 to 1175281, EPI_ISL_1175283, EPI_ISL_1175286, EPI_ISL_1175288, EPI_ISL_1175292
to 1175293, EPI_ISL_1175295 to 1175298, EPI_ISL_1175301, EPI_ISL_1175303, EPI_ISL_1175306, EPI_ISL_1175308, EPI_ISL_1175314 to 1175315, EPI_ISL_1175317 to 1175320, EPI_ISL_1175323 to 1175331, EPI_ISL_1175334, EPI_ISL_1175336, EPI_ISL_1175342, EPI_ISL_1175345, EPI_ISL_1175347, EPI_ISL_1175352,
EPI_ISL_1175356, EPI_ISL_1175360 to 1175363, EPI_ISL_1175368, EPI_ISL_1175370 to 1175371, EPI_ISL_1175373 to 1175374, EPI_ISL_1175376, EPI_ISL_1175378, EPI_ISL_1175380, EPI_ISL_1175382, EPI_ISL_1175384, EPI_ISL_1175386 to 1175388, EPI_ISL_1175390 to 1175391, EPI_ISL_1175393, EPI_ISL_1175397,
EPI_ISL_1175400, EPI_ISL_1175403 to 1175404, EPI_ISL_1175406, EPI_ISL_1175408 to 1175409, EPI_ISL_1175411 to 1175415, EPI_ISL_1186871, EPI_ISL_1186923, EPI_ISL_1186926, EPI_ISL_1187008, EPI_ISL_1187022, EPI_ISL_1187029, EPI_ISL_1187053 to 1187057, EPI_ISL_1187059, EPI_ISL_1187061,
EPI_ISL_1187063 to 1187064, EPI_ISL_1187066 to 1187069, EPI_ISL_1187071 to 1187083, EPI_ISL_1187085 to 1187090, EPI_ISL_1187092 to 1187093, EPI_ISL_1187095 to 1187107, EPI_ISL_1187110 to 1187111, EPI_ISL_1187113 to 1187128, EPI_ISL_1187130 to 1187151, EPI_ISL_1187155 to 1187158, EPI_ISL_1187160
to 1187163, EPI_ISL_1187165 to 1187168, EPI_ISL_1187170 to 1187172, EPI_ISL_1187174, EPI_ISL_1187176 to 1187179, EPI_ISL_1187181, EPI_ISL_1187183 to 1187184, EPI_ISL_1187186 to 1187199, EPI_ISL_1187201 to 1187204, EPI_ISL_1187206 to 1187208, EPI_ISL_1187210 to 1187215, EPI_ISL_1187217 to
1187221, EPI_ISL_1187224 to 1187240, EPI_ISL_1187242, EPI_ISL_1187244 to 1187247, EPI_ISL_1187250 to 1187254, EPI_ISL_1189840, EPI_ISL_1189854, EPI_ISL_1189860, EPI_ISL_1189873, EPI_ISL_1189875, EPI_ISL_1189879, EPI_ISL_1189888, EPI_ISL_1189893, EPI_ISL_1189899, EPI_ISL_1189902,
EPI_ISL_1189911, EPI_ISL_1189913, EPI_ISL_1189918, EPI_ISL_1189920, EPI_ISL_1189924, EPI_ISL_1189942, EPI_ISL_1189952, EPI_ISL_1189960, EPI_ISL_1189979, EPI_ISL_1189982, EPI_ISL_1189993, EPI_ISL_1189999, EPI_ISL_1190002, EPI_ISL_1190007, EPI_ISL_1190016, EPI_ISL_1190036, EPI_ISL_1190059 to
1190060, EPI_ISL_1190062, EPI_ISL_1190074, EPI_ISL_1190099, EPI_ISL_1190101, EPI_ISL_1190107, EPI_ISL_1190111, EPI_ISL_1190302, EPI_ISL_1190506, EPI_ISL_1190527, EPI_ISL_1190541, EPI_ISL_1190726, EPI_ISL_1206040 to 1206046, EPI_ISL_1206048 to 1206050, EPI_ISL_1206052 to 1206053,
EPI_ISL_1206055 to 1206057, EPI_ISL_1206059 to 1206064, EPI_ISL_1206067 to 1206072, EPI_ISL_1206074, EPI_ISL_1206077 to 1206080, EPI_ISL_1206082 to 1206091, EPI_ISL_1206094 to 1206103, EPI_ISL_1206105 to 1206112, EPI_ISL_1206114, EPI_ISL_1206116, EPI_ISL_1206118 to 1206123, EPI_ISL_1206125 to
1206131, EPI_ISL_1206133 to 1206135, EPI_ISL_1206137 to 1206144, EPI_ISL_1206146 to 1206149, EPI_ISL_1206152 to 1206155, EPI_ISL_1206158 to 1206165, EPI_ISL_1206168, EPI_ISL_1206170, EPI_ISL_1206172 to 1206173, EPI_ISL_1206175 to 1206176, EPI_ISL_1206178 to 1206183, EPI_ISL_1206185 to 1206187,
EPI_ISL_1206190 to 1206194, EPI_ISL_1206196, EPI_ISL_1206198 to 1206201, EPI_ISL_1206203, EPI_ISL_1206206 to 1206207, EPI_ISL_1206211 to 1206219, EPI_ISL_1206221 to 1206224, EPI_ISL_1206226 to 1206233, EPI_ISL_1206235 to 1206237, EPI_ISL_1206242, EPI_ISL_1206244 to 1206248, EPI_ISL_1206250 to
1206252, EPI_ISL_1206255 to 1206259, EPI_ISL_1206262 to 1206263, EPI_ISL_1206265 to 1206266, EPI_ISL_1206268 to 1206277, EPI_ISL_1206279 to 1206281, EPI_ISL_1206283 to 1206284, EPI_ISL_1206291 to 1206313, EPI_ISL_1206315 to 1206341, EPI_ISL_1206343 to 1206347, EPI_ISL_1206349 to 1206390,
EPI_ISL_1206392 to 1206479, EPI_ISL_1206481 to 1206501, EPI_ISL_1208368 to 1208369, EPI_ISL_1223159 to 1223161, EPI_ISL_1223163 to 1223168, EPI_ISL_1223170 to 1223200, EPI_ISL_1223202 to 1223233, EPI_ISL_1223235 to 1223254, EPI_ISL_1223256 to 1223260, EPI_ISL_1223262 to 1223328, EPI_ISL_1223331,
EPI_ISL_1223333 to 1223334, EPI_ISL_1223336, EPI_ISL_1223339 to 1223340, EPI_ISL_1223342 to 1223343, EPI_ISL_1223345 to 1223346, EPI_ISL_1223348, EPI_ISL_1223350 to 1223351, EPI_ISL_1223353 to 1223354, EPI_ISL_1223356 to 1223357, EPI_ISL_1223359 to 1223360, EPI_ISL_1223362 to 1223363,
EPI_ISL_1223365 to 1223366, EPI_ISL_1223368 to 1223369, EPI_ISL_1223371 to 1223372, EPI_ISL_1223374 to 1223375, EPI_ISL_1223377, EPI_ISL_1223380, EPI_ISL_1223382 to 1223383, EPI_ISL_1223385 to 1223386, EPI_ISL_1223388 to 1223389, EPI_ISL_1223391 to 1223392, EPI_ISL_1223394 to 1223395,
EPI_ISL_1223397 to 1223398, EPI_ISL_1223400 to 1223401, EPI_ISL_1223403 to 1223404, EPI_ISL_1223406 to 1223407, EPI_ISL_1223411 to 1223436, EPI_ISL_1224500 to 1224706, EPI_ISL_1242048, EPI_ISL_1242050 to 1242072, EPI_ISL_1242074 to 1242093, EPI_ISL_1242095 to 1242114, EPI_ISL_1242117 to 1242130,
EPI_ISL_1242132 to 1242158, EPI_ISL_1242160 to 1242165, EPI_ISL_1242167 to 1242200, EPI_ISL_1242202 to 1242228, EPI_ISL_1242230 to 1242236, EPI_ISL_1242238 to 1242265, EPI_ISL_1242267 to 1242274, EPI_ISL_1242276 to 1242280, EPI_ISL_1242572, EPI_ISL_1242577, EPI_ISL_1242581, EPI_ISL_1242585,
EPI_ISL_1242619, EPI_ISL_1242632, EPI_ISL_1242656, EPI_ISL_1242675, EPI_ISL_1242689, EPI_ISL_1242703, EPI_ISL_1242707, EPI_ISL_1242713, EPI_ISL_1242719, EPI_ISL_1242744, EPI_ISL_1242754, EPI_ISL_1242758, EPI_ISL_1242761, EPI_ISL_1242770, EPI_ISL_1242785 to 1242786, EPI_ISL_1242801,
EPI_ISL_1242812, EPI_ISL_1244852 to 1244870, EPI_ISL_1244873 to 1244874, EPI_ISL_1244876, EPI_ISL_1244878 to 1244879, EPI_ISL_1244881, EPI_ISL_1244883 to 1244904, EPI_ISL_1244906 to 1244912, EPI_ISL_1244914, EPI_ISL_1244916 to 1244919, EPI_ISL_1244921, EPI_ISL_1244923 to 1244931,
EPI_ISL_1244933 to 1244935, EPI_ISL_1244937 to 1244938, EPI_ISL_1244940, EPI_ISL_1244942 to 1244948, EPI_ISL_1244950 to 1244978, EPI_ISL_1244980 to 1245001, EPI_ISL_1245003, EPI_ISL_1245005 to 1245006, EPI_ISL_1246026, EPI_ISL_1246070, EPI_ISL_1246083, EPI_ISL_1246096, EPI_ISL_1246103,
EPI_ISL_1246106, EPI_ISL_1246125, EPI_ISL_1246128, EPI_ISL_1246163, EPI_ISL_1246176, EPI_ISL_1246202 to 1246287, EPI_ISL_1256366 to 1256427, EPI_ISL_1256429 to 1256465, EPI_ISL_1256943 to 1257062, EPI_ISL_1257639, EPI_ISL_1257643, EPI_ISL_1257646, EPI_ISL_1263853 to 1264064, EPI_ISL_1275560
to 1275872, EPI_ISL_1275874 to 1275878, EPI_ISL_1275881 to 1275901, EPI_ISL_1275903 to 1275968, EPI_ISL_1275970 to 1275980, EPI_ISL_1275982 to 1276007, EPI_ISL_1276009 to 1276047, EPI_ISL_1276049 to 1276050, EPI_ISL_1276052 to 1276055, EPI_ISL_1276057 to 1276063, EPI_ISL_1276065 to 1276083,
EPI_ISL_1276085 to 1276102, EPI_ISL_1276104 to 1276112, EPI_ISL_1276892 to 1277204, EPI_ISL_1277865 to 1277866, EPI_ISL_1277993, EPI_ISL_1294557, EPI_ISL_1294618, EPI_ISL_1294620 to 1294623

see above	Lighthouse Lab in Cambridge	Wellcome Sanger Institute for the COVID-19 Genomics UK (COG-UK) Consortium	Cordelia Langford; David K. Jackson; Dominic Kwiatkowski; Ewan Harrison; Ian Johnston; Jeffrey Barrett; John Sillitoe on behalf of the Wellcome Sanger Institute COVID-19 Surveillance Team; John Sillitoe on behalf of the Wellcome Sanger Institute COVID-19 Surveillance Team (http://www.sanger.ac.uk/covid-team); Rob Howes; Roberto Amato; Sonia Goncalves; The Lighthouse Lab in Cambridge and Alex Alderton

EPI_ISL_551835 to 551849, EPI_ISL_551851 to 551921, EPI_ISL_551925, EPI_ISL_551932, EPI_ISL_551951, EPI_ISL_551953, EPI_ISL_551967, EPI_ISL_551980, EPI_ISL_551983, EPI_ISL_551990, EPI_ISL_551994, EPI_ISL_552001, EPI_ISL_552005, EPI_ISL_552008 to 552010, EPI_ISL_552017, EPI_ISL_552051,
EPI_ISL_552065, EPI_ISL_552073, EPI_ISL_552091, EPI_ISL_552094, EPI_ISL_552098, EPI_ISL_552105, EPI_ISL_552124, EPI_ISL_552151, EPI_ISL_552166, EPI_ISL_552170, EPI_ISL_552173, EPI_ISL_552180, EPI_ISL_552185, EPI_ISL_555843 to 555844, EPI_ISL_555864, EPI_ISL_555868, EPI_ISL_555875,
EPI_ISL_555910, EPI_ISL_555939, EPI_ISL_555945, EPI_ISL_555948, EPI_ISL_555955, EPI_ISL_555973, EPI_ISL_555976, EPI_ISL_555993, EPI_ISL_556012, EPI_ISL_556021 to 556022, EPI_ISL_556026, EPI_ISL_556039, EPI_ISL_556041, EPI_ISL_556047, EPI_ISL_556058, EPI_ISL_556063, EPI_ISL_556066,
EPI_ISL_556069, EPI_ISL_556305, EPI_ISL_556309, EPI_ISL_556317 to 556318, EPI_ISL_556321, EPI_ISL_556324, EPI_ISL_556338, EPI_ISL_556344, EPI_ISL_556349, EPI_ISL_556351, EPI_ISL_556357, EPI_ISL_556425, EPI_ISL_556434, EPI_ISL_556446, EPI_ISL_556459, EPI_ISL_556471, EPI_ISL_556482,
EPI_ISL_556488, EPI_ISL_556494, EPI_ISL_556500, EPI_ISL_556508 to 556509, EPI_ISL_556516 to 556575, EPI_ISL_556639 to 556697, EPI_ISL_556699 to 556769, EPI_ISL_556771 to 556886, EPI_ISL_556888 to 556939, EPI_ISL_556941 to 556984, EPI_ISL_566423, EPI_ISL_566428 to 566429, EPI_ISL_566444 to 566445,
EPI_ISL_566453, EPI_ISL_566456 to 566457, EPI_ISL_566467, EPI_ISL_566473, EPI_ISL_566492 to 566493, EPI_ISL_566495, EPI_ISL_566500, EPI_ISL_566508, EPI_ISL_566518 to 566519, EPI_ISL_566524, EPI_ISL_566528, EPI_ISL_566534, EPI_ISL_566537, EPI_ISL_566543 to 566544, EPI_ISL_566567,
EPI_ISL_566576, EPI_ISL_566586, EPI_ISL_566589, EPI_ISL_566610 to 566611, EPI_ISL_566625, EPI_ISL_566631, EPI_ISL_566638, EPI_ISL_566659, EPI_ISL_566661 to 566662, EPI_ISL_566666, EPI_ISL_566672, EPI_ISL_566674, EPI_ISL_568385, EPI_ISL_568436, EPI_ISL_568453 to 568454, EPI_ISL_568458,
EPI_ISL_568461, EPI_ISL_568472 to 568473, EPI_ISL_580282 to 580283, EPI_ISL_580286 to 580287, EPI_ISL_580299 to 580301, EPI_ISL_580305, EPI_ISL_580313, EPI_ISL_580322, EPI_ISL_580325, EPI_ISL_580327, EPI_ISL_580333, EPI_ISL_580338, EPI_ISL_580340 to 580341, EPI_ISL_580353 to 580354,
EPI_ISL_580362, EPI_ISL_580364, EPI_ISL_580367 to 580369, EPI_ISL_580371, EPI_ISL_580373, EPI_ISL_580385, EPI_ISL_580391, EPI_ISL_580393 to 580394, EPI_ISL_580396, EPI_ISL_580400, EPI_ISL_580414, EPI_ISL_580418, EPI_ISL_580421, EPI_ISL_580435, EPI_ISL_580444, EPI_ISL_580449, EPI_ISL_580453,
EPI_ISL_580459, EPI_ISL_580465, EPI_ISL_580467, EPI_ISL_580471, EPI_ISL_580478 to 580481, EPI_ISL_580485 to 580486, EPI_ISL_580495, EPI_ISL_580499, EPI_ISL_580502, EPI_ISL_580508 to 580509, EPI_ISL_580511, EPI_ISL_580515, EPI_ISL_580532 to 580533, EPI_ISL_580536, EPI_ISL_580550,
EPI_ISL_580557, EPI_ISL_580559, EPI_ISL_580564 to 580565, EPI_ISL_580567, EPI_ISL_580569, EPI_ISL_580573, EPI_ISL_580576 to 580577, EPI_ISL_580579, EPI_ISL_580582, EPI_ISL_580586 to 580587, EPI_ISL_580593, EPI_ISL_580603, EPI_ISL_580607, EPI_ISL_580610, EPI_ISL_580618, EPI_ISL_580621,
EPI_ISL_580625 to 580626, EPI_ISL_581394, EPI_ISL_581396, EPI_ISL_590173, EPI_ISL_596928 to 596968, EPI_ISL_596970 to 597073, EPI_ISL_597075 to 597098, EPI_ISL_597100 to 597188, EPI_ISL_597190 to 597233, EPI_ISL_597237 to 597239, EPI_ISL_597241, EPI_ISL_597243, EPI_ISL_597246 to 597248,
EPI_ISL_597250 to 597251, EPI_ISL_597253 to 597259, EPI_ISL_597262, EPI_ISL_597264 to 597266, EPI_ISL_597269 to 597271, EPI_ISL_597273 to 597274, EPI_ISL_597279, EPI_ISL_597282 to 597287, EPI_ISL_597289, EPI_ISL_597293 to 597295, EPI_ISL_597297, EPI_ISL_597300 to 597301, EPI_ISL_597304 to 597310,
EPI_ISL_597316 to 597317, EPI_ISL_597319 to 597321, EPI_ISL_597323 to 597325, EPI_ISL_597327, EPI_ISL_597331, EPI_ISL_597335, EPI_ISL_597337, EPI_ISL_597340 to 597346, EPI_ISL_597349 to 597350, EPI_ISL_597354, EPI_ISL_597358, EPI_ISL_597361, EPI_ISL_597365 to 597368, EPI_ISL_597371 to 597375,
EPI_ISL_597377 to 597378, EPI_ISL_597380 to 597381, EPI_ISL_597388, EPI_ISL_597390, EPI_ISL_597392, EPI_ISL_597396 to 597399, EPI_ISL_597401 to 597403, EPI_ISL_597405, EPI_ISL_597412, EPI_ISL_597414, EPI_ISL_597418, EPI_ISL_597424 to 597428, EPI_ISL_597432, EPI_ISL_597435, EPI_ISL_597437 to
597438, EPI_ISL_597441, EPI_ISL_597445 to 597446, EPI_ISL_597449 to 597451, EPI_ISL_597453, EPI_ISL_597458 to 597462, EPI_ISL_597464, EPI_ISL_597466 to 597470, EPI_ISL_597472 to 597473, EPI_ISL_597476 to 597480, EPI_ISL_597483, EPI_ISL_597487 to 597490, EPI_ISL_597494 to 597495, EPI_ISL_597497,
EPI_ISL_597502, EPI_ISL_597504 to 597505, EPI_ISL_597507 to 597508, EPI_ISL_597510, EPI_ISL_597512, EPI_ISL_597517, EPI_ISL_597519 to 597520, EPI_ISL_601493, EPI_ISL_601495, EPI_ISL_601499, EPI_ISL_601501, EPI_ISL_601506, EPI_ISL_601515, EPI_ISL_601522, EPI_ISL_601530, EPI_ISL_601541,
EPI_ISL_601547, EPI_ISL_601549 to 601550, EPI_ISL_601555, EPI_ISL_601567, EPI_ISL_601569, EPI_ISL_601571, EPI_ISL_601578 to 601580, EPI_ISL_601585, EPI_ISL_601588 to 601591, EPI_ISL_601594, EPI_ISL_601604, EPI_ISL_601606, EPI_ISL_601611, EPI_ISL_601615, EPI_ISL_601617 to 601619,
[truncated: 2,024,175 more chars]
